# Rediscovering Diazaborines: Synthesis and Bioactivity Profiling of Boron-Containing FabI Inhibitors against Gram-Negative Bacteria

**DOI:** 10.1021/acs.jmedchem.5c01766

**Published:** 2026-02-07

**Authors:** Polina Ilina, Vladimir Iashin, Cristina D. Cruz, Juho Heininen, Iiro Järvi, Inna Pönniö, Sami Heikkinen, Pauli Johan Wrigstedt, Leo Ghemtio, Karina Moslova, Henri Xhaard, Paula Kiuru, Jesús Perea-Buceta, Päivi Tammela

**Affiliations:** † Drug Research Program, Faculty of Pharmacy, 3835 University of Helsinki, P.O. Box 56, Helsinki FI-00014, Finland; ‡ Department of Chemistry, Faculty of Science, University of Helsinki, P.O. Box 55, FI-00014, Helsinki, Finland

## Abstract

In this study, we investigated the potential of diazaborine compounds for antibacterial drug development. Most promising diazaborines demonstrated activity against several Gram-negative pathogens including *Escherichia coli*, *Klebsiella pneumoniae*, *Acinetobacter baumannii*, and *Salmonella enterica* ser. Typhimurium. For a subset of diazaborines, we showed inhibitory activity against isolated FabI (enoyl-acyl carrier protein reductase) enzyme aligning with antimicrobial activity, suggesting a mechanism of action via the FabI enzyme and providing early information on structure–activity relationships. Optimized diazaborine scaffold **11** features an amino group in a meta-relative position to the sulfonamide group and exhibited the most favorable bioactivity profile, showing MIC of 6.25 μM against *E. coli*, low cytotoxicity, and high stability in human plasma. Furthermore, diazaborine **11** had synergistic effect with colistin (FICI 0.25) and preliminary data show that it may rescue *Galleria mellonella* larvae from lethal *E. coli* infection at the therapeutic dose of 1.13 and 2.81 mg/kg, demonstrating efficacy similar to ciprofloxacin.

## Graphical abstract

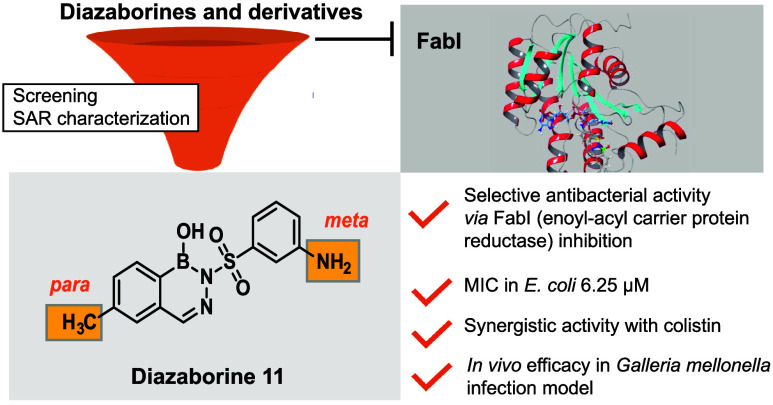

## Introduction

Diazaborines, a group of synthetic boron-containing heterocyclic compounds, were first reported in the 1960s.^1^ Soon after, their promising selective activity against Gram-negative bacteria was reported in several publications.^2–4^ That significant interest culminated in the article by Grassberger (Sandoz) in 1984, where the authors reported a new synthetic pathway accessing the 2,3,1-benzodiazaborine series and investigated their structure–activity relationship in a panel of clinically relevant Gram-negative species, including e.g. *Escherichia coli*, *Klebsiella pneumoniae,* and *Neisseria gonorrhoeae*.^5^ Despite the encouraging results, the investigation of the pharmaceutical use of these compounds was suddenly halted; and none of the reported compounds or their derivatives reached the antibiotic drug market, presumably due to concerns on intrinsic cytotoxicity of boron-based functionalities prevalent at the time.^6^ A large body of scientific evidence accumulated by the beginning of 21st century proving this claim a misconception, resulting in a burst of interest to boron in pharmaceutical drug discovery, and FDA approval of five boron-containing drugs.^7^

In recent years, members of the diazaborine family have shown potential in a number of biomedical applications. In particular, Dukes et al. designed fluorescent diazaborine mimics of endogenous estrogens.^8^ Antonio et al. reported use of diazaborines as reactive oxygen species (ROS)-responsive linkers for creating antibody–cytotoxic drug conjugates against cancer.^9^ Pertschy et al. described the diazaborine compound inhibiting growth of yeast by interfering with translation.^10^ In other recent studies, diazaborines have been suggested as novel inhibitors of human neutrophil elastase, a potential therapeutic target in several inflammatory diseases. Notably, the authors reported no appreciable in vitro toxicity and good stability in biologically relevant conditions.^11^

Given the growing concern about the spread of antimicrobial resistance, there is an urgent need for new antibacterial drugs, especially those that are selective for certain groups of bacteria or act via novel mechanisms. In this context, compounds targeting the fatty acid biosynthesis pathway are particularly promising as this pathway is organized differently in most bacteria than in mammals, allowing for selective inhibition.^12^ Only two antimicrobials with this mechanism of action are currently commercially available. Triclosan, a broad-spectrum antimicrobial agent, is mainly used as an antiseptic in consumer products and surgeries, while isoniazid is a narrow-spectrum drug for the treatment of tuberculosis. Both agents are in restricted use due to side effects.^12^ The intracellular target responsible for the antibacterial activity of diazaborines has been identified as enoyl-acyl carrier protein reductase FabI, an enzyme involved in the biosynthesis of fatty acids, which are an essential component of cell walls.^13^ Not all bacteria rely solely on this enzyme since some bacterial pathogens are able to scavenge fatty acids from the host.^14^ Still, FabI is believed to be a promising target for narrow-spectrum antibiotics against several crucially important pathogens, including e.g. *E. coli*, *Acinetobacter baumannii,* and *Staphylococcus aureus*.^15^ Narrow-activity spectrum of antimicrobial action can also be considered an advantage. FabI inhibition is believed to have minimal impact on the gut microbiota as most microorganisms in the microbiome utilize other enoyl reductase isozymes.^16^

Our study aimed to expand the existing knowledge of the potential of diazaborine compounds for antimicrobial drug development. With modern methods of organoboron chemistry and compound characterization in hand, we synthesized a series of novel derivatives of phenyl and thiophene diazaborines including selected charged derivatives and amino acid conjugates to more closely explore the substitution patterns and optimize the diazaborine scaffold and studied their antibacterial and cytotoxic properties. We tested a subset of these diazaborines for inhibition of the isolated Fabl enzyme to confirm the mechanism of action, as well as to obtain early information on structure–activity relationships. To more extensively characterize the most potent compounds of the series, we assessed their stability, time-kill kinetics, resistance-related properties, and synergistic activity. In addition, we attempted to enhance the diazaborine antimicrobial activity by conjugation with amino acids and phosphonium salts.

## Results and Discussion

### Antibacterial Activity and Structure–Activity Relationships

The antibacterial activity of 59 synthesized diazaborine derivatives (chemical structures shown in Figure 1) was evaluated against *Enterococcus faecalis*, *E. coli*, *Pseudomonas aeruginosa*, and *S. aureus* first at 50 μM by following the Clinical & Laboratory Standards Institute (CLSI) guidelines for antimicrobial susceptibility testing. The study included iterative rounds of compound synthesis and antibacterial testing, where the results guided the design of new derivatives with potentially improved properties. The results of the antibacterial assays are presented as a percentage of bacterial growth inhibition in Table S1.

**Figure 1:**
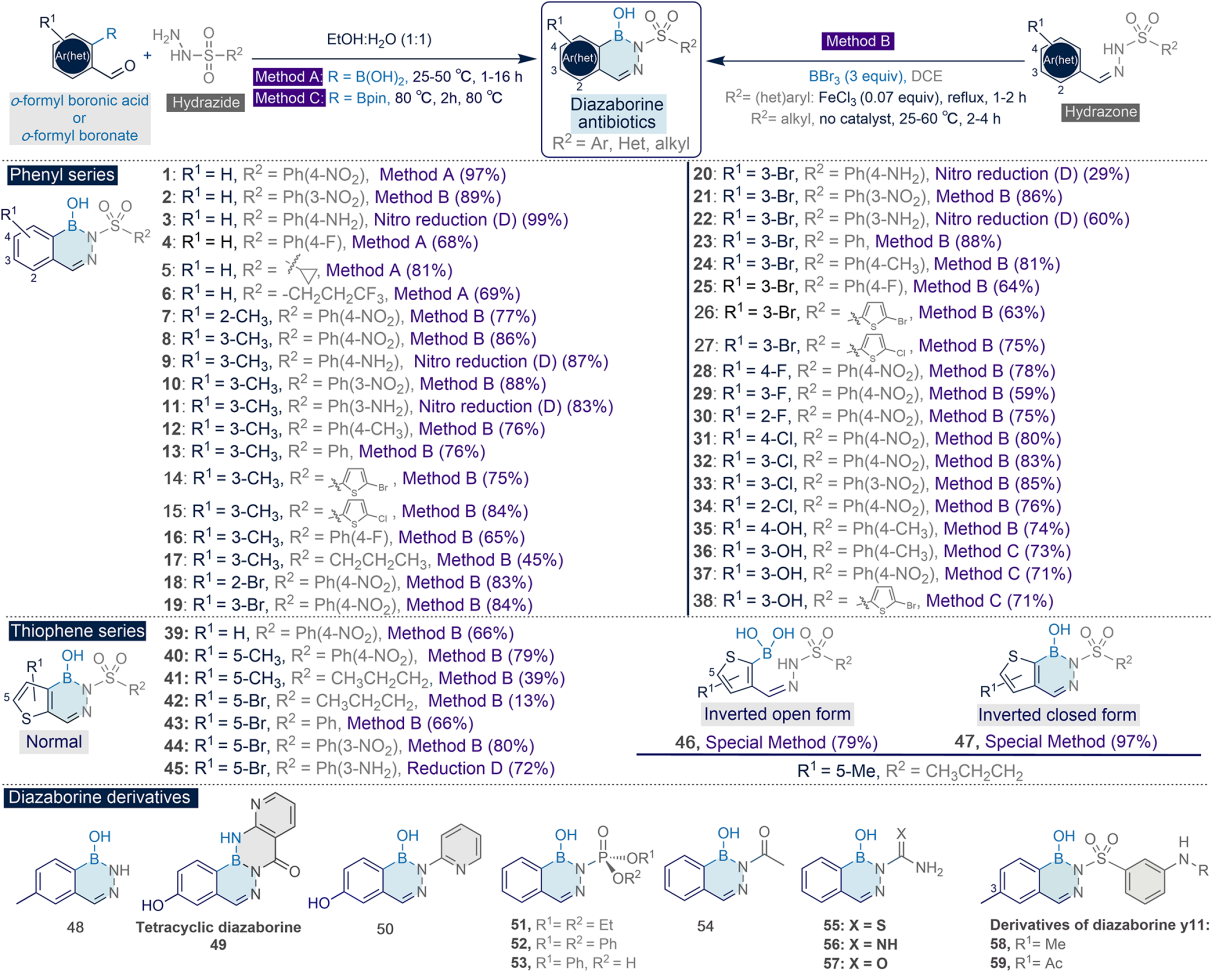
Structures of the diazaborine series evaluated in this study.

Diazaborine derivatives were most effective in inhibiting the growth of *E. coli*. Specifically, 65% (26 of 38) of the phenyl-series compounds and 78% (7 of 9) of the thiophene series showed growth inhibition above 50%. About a half of those active compounds (17 of 33) achieved ≥90% *E. coli* growth inhibition. Overall, diazaborines did not display significant inhibition of *P. aeruginosa* and *E. faecalis* (i.e., inhibitions were below 50%). Similar mostly negative results were observed for *S. aureus*, except for tetracyclic diazaborine derivative **49** and six compounds belonging to the thiophene-series (**39**, **40**, **42**, **45**), for which inhibition rates were ≥50%.

For compounds leading to ≥50% bacterial growth inhibition in the initial screening, we evaluated the antimicrobial potency by determining their minimum inhibitory concentration (MIC) values (Table 1 and S1). MIC values in *E. coli* varied from 6.25 μM to >125 μM (highest concentration tested). The most potent compounds were diazaborines **11** and **22** from the phenyl series and diazaborine **47** from the thiophene-series (MIC value of 6.25 μM, Table 1). For *S. aureus,* MIC could only be achieved for diazaborine **49** (25 μM, Table S1). A possible reason underlying the activity of this compound is the fact that it does not contain the B-OH functional group that is able to interact with the diol residues in the peptidoglycan of Gram-positive bacteria membranes. Masking the group may allow the diazaborine scaffold to circumvent those interactions on the membrane and permeate into the bacterial cell.

**Table 1:** Detailed Biological Activity Characterization of Diazaborine Compounds Displaying ≥50% Growth Inhibition against *Escherichia coli* ATCC25922 in the Screening Assay at 50 μM Concentration (Complete Screening Data in Table S1)­^c^ ^,^ ^e^

	antibacterial activity								cytotoxicity	
	MIC^a^ (μM) or (maximum inhibition growth, % ± SD)^a^								IC_50_ ^b^ (μM)	
diazaborine	*E. coli* 25922	*E. coli*CB9615	*E. coli*CFT073	*E. coli*UMN026	*A. baumannii*19606	*K. aerogenes* 13048	*K. pneumoniae* 700603	*S. enterica*ser. Typhimurium 19585	HepG2 cells	Hs27 cells
Phenyl Series										
2	50	ND	ND	ND	ND	ND	ND	ND	ND	ND
3	75	ND	ND	ND	ND	ND	ND	ND	ND	ND
8	25	ND	ND	ND	ND	ND	ND	ND	ND	ND
9	12.5	12.5	12.5	25	50	25	>50 (30.2 ± 1.8)	12.5	95 ± 1	>250
10	25	ND	ND	ND	ND	ND	ND	ND	ND	ND
11	6.25	12.5	6.25	12.5	6.25	12.5	75	6.25	106 ± 13	240 ± 9
12	50	ND	ND	ND	ND	ND	ND	ND	ND	ND
13	12.5	6.25	6.25	12.5^d^	12.5	12.5	>50 (38.2 ± 3.4)	6.25	63 ± 9	170 ± 9
14	75	ND	ND	ND	ND	ND	ND	ND	ND	ND
15	50	ND	ND	ND	ND	ND	ND	ND	ND	ND
16	50	ND	ND	ND	ND	ND	ND	ND	ND	ND
17	25	ND	ND	ND	ND	ND	ND	ND	ND	ND
18	>125	ND	ND	ND	ND	ND	ND	ND	ND	ND
19	25	ND	ND	ND	ND	ND	ND	ND	ND	ND
20	12.5	25	12.5	25	25	25	>50 (31.9 ± 3.5)	12.5	58 ± 10	220 ± 46
21	50	ND	ND	ND	ND	ND	ND	ND	ND	ND
22	6.25	6.25	6.25	12.5	12.5	12.5	>50 (57.4 ± 3.5)	6.25	55 ± 5	116 ± 2
23	25	ND	ND	ND	ND	ND	ND	ND	ND	ND
24	75	ND	ND	ND	ND	ND	ND	ND	ND	ND
30	>125	ND	ND	ND	ND	ND	ND	ND	ND	ND
32	25	ND	ND	ND	ND	ND	ND	ND	ND	ND
33	75	ND	ND	ND	ND	ND	ND	ND	ND	ND
34	>125	ND	ND	ND	ND	ND	ND	ND	ND	ND
38	>125	ND	ND	ND	ND	ND	ND	ND	ND	ND
Thiophene Series										
41	12.5	12.5	12.5	25	12.5	12.5	50	6.25	222 ± 23	>250
42	25–100^d^	ND	ND	ND	ND	ND	ND	ND	ND	ND
43	25	50	25	50	25	25	>50 (54.8 ± 2.1)	12.5	87 ± 4	202 ± 10
44	12.5–100^d^	ND	ND	ND	ND	ND	ND	ND	ND	ND
45	50	ND	ND	ND	ND	ND	ND	ND	ND	ND
46	25	12.5	25	50	25	25	>50 (49.3 ± 6.7)	12.5	>250	>250
47	6.25	6.25	6.25	6.25	6.25	6.25	25	3.13	>250	>250
Diazaborine Derivatives										
58	25	ND	ND	ND	ND	ND	ND	ND	ND	ND
59	50	ND	ND	ND	ND	ND	ND	ND	ND	ND
Positive Controls										
triclosan	0.39	ND	0.39	0.39	0.78	0.78	3.125	0.39	18 ± 2	21 ± 2
ciprofloxacin	0.05	0.06	0.12	0.02	3.6	0.12	1.5	0.05	ND	ND
camptothecin	ND	ND	ND	ND	ND	ND	ND	ND	<2	<2

a MIC (μM) was determined using the broth microdilution method, as outlined by CLSI.^17^ Three independent experiments in triplicate were performed. A positive control ciprofloxacin was used on each plate.

b Half maximal inhibitory concentrations (μM) were determined using ATP-based cell viability assay after 72 h incubation in the presence of the compound. A positive control camptothecin (2 μM) was used on each plate.

c ND = not determined.

d These compounds showed high variation between repeats, presumably due to degradation, and were excluded from further studies.

e For most active compounds (shaded), activity range and cytotoxicity assessment studies are also shown.

Previous studies have identified the enoyl-acyl carrier protein reductase (FabI) as the molecular target of diazaborines.^13,18,19^ Diazaborine compounds are thought to mimic the enzyme’s natural enoyl substrate. There is direct crystallographic evidence from the *E. coli* FabI bound to thiocarbamoylated benzodiazaborine inhibitors (PDB codes 5CG1 and 5CG2, resolution 2.07–2.2 Å) of the formation of a covalent bond between the inhibitor’s boron atom and the ribose 2́ hydroxyl of the enzyme-bound cofactor NAD^+^.^13,19^ The diazaborine series presented here is likely to also adopt a covalent binding mode. The mode of binding involves a disordered loop that folds to cover the inhibitor site (amino acids Leu195 to Met206 in *E. coli* ^13,18^ ) which makes the prediction of binding modes via molecular docking challenging.

To confirm the mechanism of action (FabI inhibition) as well as to obtain information on structure–activity relationships, we tested a subset of 11 diazaborines (**10**, **11**, **13**, **17**, **18**, **22**, **30**, **34**, **41**, **43**, **46**) with diverse level of antibacterial activity for inhibition of isolated *E. coli* FabI enzyme (Table S2). Triclosan was used as a positive control. As a result, diazaborines **11**, **13**, and **22** from the phenyl series were among the most potent against FabI, with inhibition in the range 73.2–80.2% at 2 μM. For **11** and **13**, we calculated preliminary *K*_i_s of 0.32 and 0.34 μM, respectively (Table S2 and Figure S1). The control triclosan showed a FabI inhibition of 87.8% and a preliminary *K*_i_ of 0.041 μM. Compounds **11** and **22** differ from compound **13** by the presence of an amine at the R^2^ benzenesulfonyl side chain instead of an unsubstituted phenyl (Figure 1), showing that an amine substitution is not required for activity at FabI in *E. coli*. This can be explained structurally since, in the crystal structures of *E. coli* FabI, the majority of contacts between the NAD^+^-inhibitor complexes and the protein are made by NAD^+^, with few direct contacts between the inhibitor and the FabI protein. Diazaborines **10** and **17** were also active, with about 50% inhibition at 2 μM. These have different substitutions at R^2^ compared to **11**, Ph­(3-NO_2_) and propyl. For diazaborines **41**, **43**, and **46**, activity was less than 30%, and **18**, **30**, and **34** showed only residual inhibition (∼10% or less).

The target-based action at FabI in *E. coli* can help us understand better the bacterial growth inhibition; however, different strains of bacteria expressing different enzymes, as well as the fact that other proteins might be involved, can make this process less than straightforward. Overall, the FabI target-based inhibition values follow the MIC in *E. coli*, with diazaborines the most active against FabI having also numerically lower MIC (Table S2). Conversely, the diazaborines **18**, **30**, and **34** do not have notable inhibitory activity. An exception is diazaborine **41**, which is not a strong inhibitor of FabI, however its MIC was 12.5 μM. At R^1^ (Figure 1), substituting the diazaborine phenyl ring with a 3-methyl group, as in compounds **11** and **13**, yields the most favorable results both in *E. coli* ATCC 25922 and FabI inhibition assay (Tables 1 and S2). 3-Bromo substitution at R^1^ is also tolerated as in compounds **20** and **22**. In contrast, derivatives that lack substitution or possess 3-hydroxyl or halogen groups at the 2-position of the phenyl ring of R^1^ generally exhibit low activity in *E. coli* ATCC 25922 strain. This is in line with the observation by Levy et al. that substitutes at 3-position should be small and hydrophobic.^18^ Regioisomers **41** and **47** from the thiophene series exhibit good activity. At R^2^, compounds with a 3-aminophenyl, such as **11** and **22**, are the most active in terms of MIC against *E. coli*. This is in agreement with the activity of 4-aminophenyl-containing compounds **9** and **20**, reported earlier by Grassberberger et al. (Table S3).^5^ The synthesis route intermediates with a nitro at R^2^ (**10**, **19**, and **21**) show moderate MICs against the *E. coli* ATCC 25922 strain (Table 1). Earlier studies have shown that the corresponding R^2^ sulfonyl chain is important in stabilization of the tetrahedral boron-NAD^+^ adduct, and electron-withdrawing substituents in the benzenesulfonyl ring might reduce the sulfonyl’s stabilization ability.^18,19^

All 59 diazaborines were also screened for antibacterial activity against *P. aeruginosa* ATCC 27853, *S. aureus* ATCC 29213, and *E. faecalis* ATCC 29212 at 50 μM (Table S1), but no antibacterial activity was detected; only residual activity against *S. aureus* for the thiophene series (**39**, **40**, **42**–**45**) and MIC of 25 μM for diazaborine **49** were measured. The lack of activity in *P. aeruginosa* and *E. faecalis* could be expected as these species encode other enoyl-acyl carrier protein reductase isozymes.^15^ However, *S. aureus* solely relies on FabI, and therefore, resistance of this species to diazaborines was surprising. Other previously described FabI inhibitors are highly active against *S. aureus* (reviewed in a study by Rana et al.^15^). For example, reported MIC for fabimycin against *S. aureus* is 250–500 times lower than in *E. coli*.^15^ Many other FabI inhibitors, such as triclosan, triclosan derivatives, and imidazole derivatives, also show activity against both species.^20^ To confirm the lack of activity, we performed additional screening against five Staphylococci strains. We tested compounds which demonstrated over 50% inhibition in the initial screening at single 50 μM concentration, as well as compounds that showed the highest activity against *E. coli*. With the exception of diazaborine **49**, none of the compounds was able to fully inhibit bacterial growth (Table S4). One possible explanation for the lack of activity observed in our study could be interspecies differences in the FabI enzyme structure. Indeed, sequence alignment shows moderate to poor conservation of amino acid residues present at the substrate-binding pocket between *E. coli* and *S. aureus*.^21,22^

In conclusion, among the 30 novel synthesized diazaborines from phenyl and thiophene series, 3-aminophenylsulfonyl compounds **11** and **22** displayed most promising antibacterial activity against Gram-negative *E. coli*.

### Antibacterial Activity of Diazaborine Conjugates

Optimized diazaborine scaffold (diazaborine **11**) showed MIC of 6.25 μM against *E. coli*. In attempt to further improve antibacterial activity and possibly expand activity spectrum, we synthesized a set of charged diazaborines and diazaborines conjugated to other chemical moieties (structures shown in Figure S2) and tested them against the primary 4-species panel. Inspired by the work of Hergenrother^23,24^ who showed that small compounds with ionizable nitrogen atoms have an increased likelihood to accumulate in *E. coli* and other bacteria, we first evaluated the effect of ionizing the diazaborine scaffold. However, ionization at either the boron warhead or a peripheric amine function did not promote any activity change (Table S5).

The other strategy evaluated was the conjugation of diazaborines to alkyl triphenylphosphonium cations and amino acids. Lipophilicity of triphenylphosphonium cations is well-known to allow them to easily permeate through bilipid cell membranes,^25^ potentially enabling diazaborines to permeate through the membrane of a broader microbial spectrum, thus amplifying their antimicrobial profile. However, diazaborine **11** conjugated to the phosphonium moiety by either a noncleavable amide or cleavable carbamate linker (conjugates **67** and **68**) lost activity against *E. coli* (Table S5). Interestingly, the compound linked by the cleavable carbamate linker showed activity in *S. aureus* (MIC 25 μM), suggesting that the phosphonium moiety helps diazaborines to permeate through the membrane of this Gram-positive species. The carbamate linker is cleaved more rapidly than amide bonds at pH 4 inside bacteria. While carbamate bonds undergo acid-mediated hydrolysis, amide bonds are primarily cleaved through enzymatic hydrolysis or oxidation. Interestingly, conjugation of these compounds to either l- or d-forms of leucin and tryptophan resulted in further increase of activity against Gram-positive species *S. aureus* and *E. faecalis* (conjugates **71**, **72**, **73**, **74**, **77**, **78**, **79**, **80**, Table S5). From this series, the most potent compounds were diazaborine **11**-phosphonium conjugates with either l-or d-tryptophan moieties, compounds **77** and **78**, respectively (MIC in *S. aureus* 6.25 μM and about 80% growth inhibition in *E. faecalis* at 50 μM). Furthermore, the diazaborines conjugated with l- or d-tryptophan without phosphonium (conjugates **69** and **70**) exhibited activity similar to that of their parent compound, indicating no effect of amino acid alone. Despite these interesting findings that may warrant future research, conjugation failed to enhance activity against Gram-negative bacteria, which was our primary focus. Therefore, in further studies, we returned to the initial set of diazaborine derivatives.

### Antibacterial Activity Spectrum

Based on their antibacterial activity against the *E. coli* reference strain, we selected a set of the most potent compounds from each class (i.e., MIC values of 6.25–12.5 μM), in total 9 compounds, in Table 1. For this set, we performed more detailed antibacterial activity profiling with a panel of clinical *E. coli* isolates (enteropathogenic and uropathogenic strains) and other clinically relevant Gram-negative species, including members of the ESKAPEE panel, such as *A. baumannii*, *K. pneumoniae*, and *K. aerogenes* (previously known as Enterobacter aerogenes). All compounds were equally active against all three pathogenic *E. coli* strains, including uropathogenic strain UMN026 characterized by multiple antibiotic resistances.^26^ The MIC values (Table 1) for these strains were within 2-fold difference from each other and representative ATCC strain used for the initial screening, which is within method variation. MIC values similar to those in *E. coli* were obtained for *A. baumannii*, *K. aerogenes*, and *Salmonella enterica* ser. Typhimurium, whereas activity against *K. pneumoniae* was substantially lower (Table 1). For comparison, fabimycin, recently reported FabI inhibitor, shows activity against *E. coli* and *A. baumannii* comparable to our most potent diazaborines (2.5–10 μM MIC values),^20^ whereas its activity in *K. pneumoniae* strains, including a set of clinical isolates, is somewhat better (MIC 10 μM).^20^

Compounds **9**, **12**, **17**, **19**, **20**, **24**, **30**, **35**, **41**, **43**, and **47** evaluated in this study were originally reported by Grassberger and co-authors.^5^ Despite some differences in the experimental setup (e.g., different bacterial strains and culture medium), overall the antibacterial activity data obtained in *E. coli* corroborate well (for side-by-side comparison, see Table S3). MIC values are similar (within 2-fold difference) to each other, with one exception of diazaborine **43** which showed about 3 times higher MIC in our study. Moreover, our study showed low activity of the compounds against *K. pneumoniae*, whereas in the work of Grassberger, the same compounds showed in this species activity similar to that of *E. coli* (Table S3). The diazaborine **47**, shown to be the most potent in Grassberger work,^5^ was also among the most active ones in our study.

### Cytotoxicity Screening and Dose–Response Experiments

To evaluate the diazaborine series in terms of their toxicity to mammalian cells, we studied their cytotoxicity in human hepatocarcinoma cell line HepG2, which is widely used in the toxicological assessment of drug candidates.^27^ Additionally, we evaluated their cytotoxicity in the noncancerous, immortalized human skin fibroblast cell line Hs27. For cytotoxicity screening, we utilized an ATP-based cell viability assay. The initial testing was performed at 250 μM concentration, i.e., at concentration exceeding MIC of our most active compounds about 40 times. It must be noted that in these experiments, we used extended incubation times of 72 h, whereas 24 or 48 h are commonly used. Higher cytotoxicity levels after this prolonged incubation allowed us to observe clearer differences and compare compounds of this study to fabimycin.^20^ Overall, cytotoxicity profiles were similar in the two cell lines, although, in general, the compounds were more toxic to HepG2 cells than to Hs27 cells (Table S1).

We studied in more detail the cytotoxicity of the 9 selected compounds (shaded in gray in Table 1). Using the same experimental conditions as those in the initial cytotoxicity experiments, we determined IC_50_ values in the two cell lines (Table 1). This parameter represents concentration that causes a 50% reduction in cell viability or growth and is typically used to compare the cytotoxicity of compounds. In line with the initial screening data, IC_50_ values obtained in HepG2 cells were always lower than those in Hs27 cells. Inverted thiophenes **46** and **47** showed the lowest cytotoxicity as their IC_50_ values were above the tested concentration range in both cell lines (i.e., >250 μM). Among the phenyl series compounds, cytotoxicity was the lowest for diazaborine **11** (IC_50_ in HepG2 cells 106 ± 13 μM), followed by diazaborine **9** (95 ± 1 μM), **13** (95 ± 1 μM), **20** (58 ± 10 μM), and **22** (55 ± 5 μM). Thiophene series diazaborine **41** (IC_50_ 222 ± 23 μM) was significantly less cytotoxic than the second compound from the same series **43** (IC_50_ 87 ± 4 μM).

For comparison, IC_50_ of 75 μM for the FabI inhibitor fabimycin has been reported in the HepG2 cell line at experimental conditions similar to those used in our study.^20^ Triclosan was reported to have significant in vitro cytotoxicity with IC_50_ values of 70 ± 10 μM, 20 ± 10 μM, and 60 ± 20 μM after 48 h of incubation with HepG2, MCF-10A, and MCF-7/1B1 cells, respectively.^28^ The MIC value of this approved antibacterial against *E. coli* is about 0.3 μg/mL (1 μM).^22^

Our study indicates that in vitro cytotoxicity varies largely between diazaborines (Table S1). Cytotoxic effects are rather related to the molecular structure and not to the presence of boron atom per se. Indeed, Antonio et al. demonstrated serine protease inhibiting diazaborines to have IC_50_ > 100 μM after 48 h incubation with HEK293T cells.^11^ Bandyopadhyay et al. studied the diazaborine conjugate of semicarbazide with the synthetic amino acid and showed no significant cytotoxic effect after 24 h incubation with HEK293T cells at 50 μM concentration.^29^

### Compound Selection for Follow-Up Studies

Initially we selected three diazaborines: **11**, **41**, and **47** as the most promising representatives of the phenyl-, thiophene-, and inverted thiophene series, respectively (Table 1). Additionally, we selected diazaborine **13** from the phenyl series due to its promising combination of relatively high antibacterial activity and structural simplicity. At a later stage, **47** was excluded from further evaluation due to stability concerns (see below).

### Stability Studies

We investigated the stability of four selected diazaborines (**11**, **13**, **41**, and **47**) in dimethyl sulfoxide (DMSO) to exclude the possibility that our results were affected by the presence of degradation products. In addition, we included diazaborines **46** (open form of **47**) and **61** (salt form of **11**). We also included diazaborine **42** to investigate whether variable MIC values (Table 1) could be explained by its relative instability under physiological conditions. In these experiments, we used NMR spectroscopy to evaluate the stability of DMSO stock solutions of these compounds during 5 consecutive freeze–thaw cycles (1 cycle per day) to mimic the typical storage conditions of stock solutions during biological experiments. During 5 cycles, decomposition of phenyl-series diazaborines **11** and **13**, as well as thiophene **41** was negligible (below 5%) (Figure S3). Opened form of the inverted thiophene **46** followed the same trend. However, closed form **47** was less stable, showing decomposition of about 10% after 2 cycles and 40% after 5 cycles. Comparative analysis of the ^1^H NMR spectra revealed that the main decomposition product was its opened form **46**, the compound which also shows low cytotoxicity and antibacterial activity, even though it is about 4 times less potent (Table 1). In contrast, the 5-bromothiophene derivative **42** showed significant (33%) degradation already after one freeze–thaw cycle. This observation is in accordance with the inconsistent results of our biological screening (Table 1).

We then investigated the stability of selected diazaborines at 37 °C in water to mimic conditions in our biological assays (Figure 2A) and human plasma as an indicator of *in vivo* blood stability (Figure 2B). As these compounds are barely soluble in water, we used their water-soluble sodium salts to avoid using DMSO due to potential interference with plasma stability assays. Such substitution did not alter this stability study since the biological performance of the salts was identical with the original compounds. In particular, chloride- sodium- and potassium salts of diazaborine **11** had the same MIC values as the parental compound (Table S5). Although the method of quantification is semiquantitative, some trends can be found. For all the salts, decrease of about 20% from the initial concentration was observed (Figure 2A). Furthermore, whereas the concentration of phenyl diazaborines **13** and **11** varied significantly over time, the change of concentration of **41** over time was negligible. However, unlike those of the aforementioned compounds, the concentration of the salt of inverted thiophene **47** gradually decomposed over time. Additionally, the behavior of all these compounds was studied by measuring their ^11^B NMR spectra and after 72 h (aliquot maintained in plasma at 37 °C). While salts of diazaborines **11**, **13**, and **41** showed the characteristic single peak of the initial salts at ca. +6.5 ppm, diazaborine **47** gave a complex set of signals. This observation further proves the instability of the inverted thiophene **47** in human plasma and the relative stability of phenyl and normal thiophene salts, opening gates toward their potential use as water-soluble antibacterial drugs.

**Figure 2:**
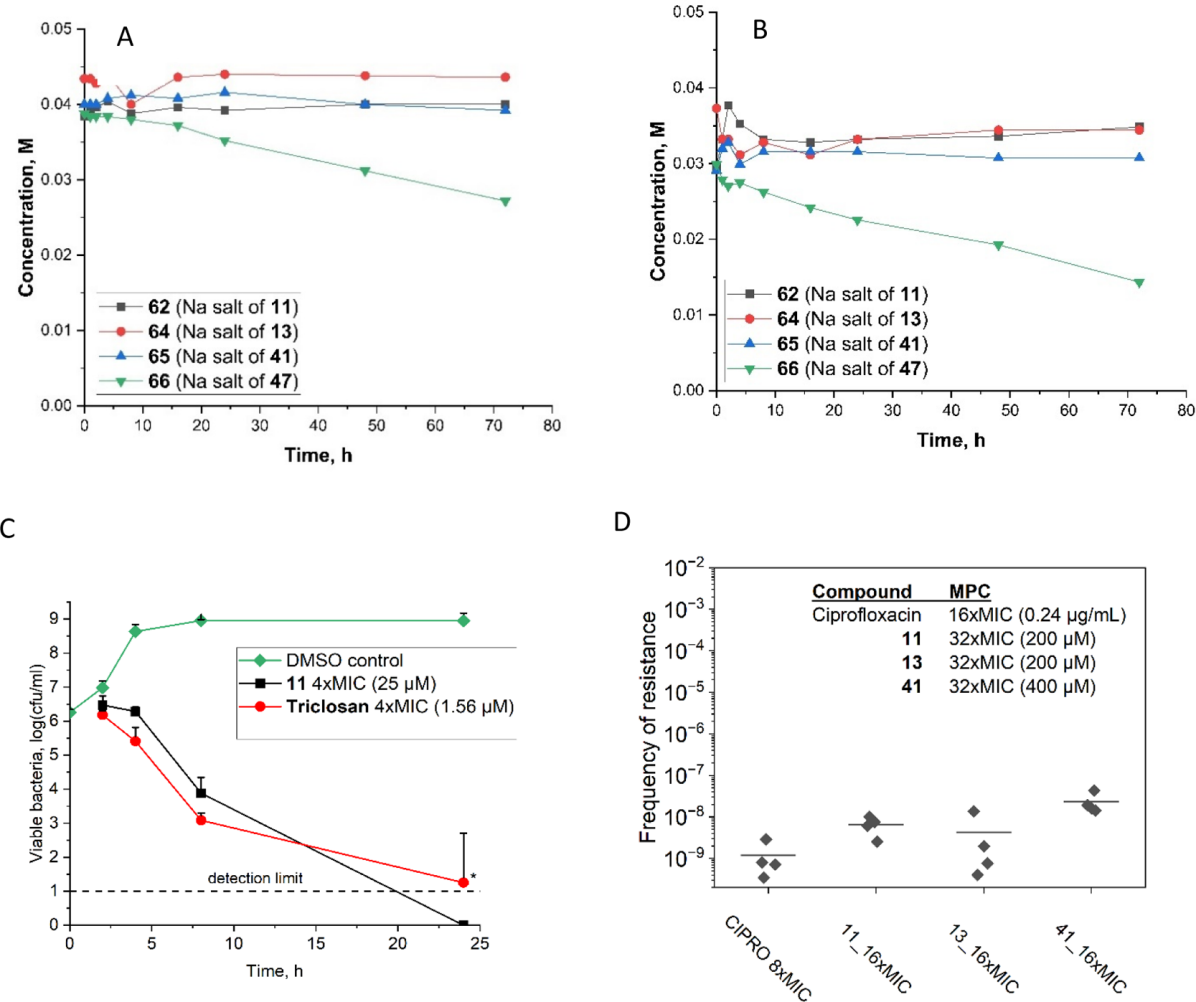
Stability and detailed antibacterial activity characterization of most active diazaborines. (A) Stability in water. (B) Stability in human plasma. (C) Time-kill kinetics against *E. coli* ATCC25922 at 4 × MIC concentration. The data points representing mean value of 3 independent experiments ± SD * indicate resistance development after 24 h, which was observed in at least one of triplicate wells in each repeat for triclosan control. The resistant wells were excluded from the analysis. In diazaborine-treated wells, no resistance was observed. (D) Resistance development to diazaborines and ciprofloxacin (Cipro). Mutant prevention concentrations (MPCs) were expressed as MIC-fold and in μM. Spontaneous resistance frequencies were determined for a half-MPC concentration. Data points represent independent experiments, and the mean value is shown as line.

### Synergistic Activity of Diazaborines

Given the lengthy and often unsuccessful process of drug discovery, repurposing existing antibiotics in combination therapy has emerged as an effective approach.^30^ Predicting antibiotic synergy remains challenging due to the complexity of bacterial responses, and empirical testing still plays an essential role. Polymyxins such as polymyxin B and colistin are the last resort drugs for carbapenem-resistant Gram-negative pathogens. Increasing resistance to polymyxins among bacteria via easily transferred plasmid has already been reported in many countries.^31,32^ In this study, we assessed the efficacy of colistin-based combinations with diazaborines **11**, **13**, **41**, and **47**. Checkerboard assays revealed a clear dose-dependent synergistic activity of compounds with colistin. As shown in Table 2, the MIC of colistin could be decreased from 1 to 0.25 μg/mL, in most cases when combined with 1.25 μM of compounds. Exceptionally diazaborine **41** required higher amounts (i.e., 10 μM) to decrease colistin MIC to 0.0625 μg/mL.

**Table 2:** Results of Checkerboard Assays of Colistin in Combination with Diazaborines **11**, **13**, **41**, and **47** against *E. coli* ATCC25922^b^ ^,^ ^c^ ^,^ ^e^

colistin MICs (μg/mL) at different compound concentrations (μM)									
compound concentration (μM)									
	0	0.32	0.62	1.25	2.50	5.01	10	20	FICI^d^
**11**	1	1	0.5	0.25^a^	0.5	0.125^a^	MIC^*c*^		0.25
**13**	1	1	0.5	0.25^a^	0.5	0.25^a^	MIC^*c*^		0.25
**41**	1	1	1	1	1	0.5	0.0625^a^	MIC^*c*^	0.56
**47**	1	1	0.5	0.25^a^	0.5	0.125^a^	MIC^*c*^		0.38

a Combinations in which colistin/compounds yielded a synergistic activity, 4-fold MIC reduction of colistin, in comparison to MIC in the absence of compounds.

b The lower concentration of compounds needed for synergy to colistin is shaded in gray.

c MIC indicates new MIC achieved for compounds alone when performing the checkerboard assay.

d FICIs are interpreted as synergy (FICI is ≤0.5), additive (0.5 < FICI ≤ 1), indifference (1 < FICI ≤ 4), and antagonism (FICI is >4). Experiments were performed three times with one replicate per combination each.

e Fractional inhibitory concentration index (FICI) below 0.5 indicates synergy.

Diazaborine **11** was also tested for synergistic effects with other biologically active compounds, including antibacterial drugs metronidazole, sulfadiazine, meropenem, amoxicillin, and ciprofloxacin, as well as metformin (diabetes treatment), citric acid (food preservative, insecticide), and choline chloride (food additive). However, no synergy was detected in those experiments (Table S6).

### Membrane Integrity Assay

To further characterize four selected diazaborines (**11**, **13**, **41**, and **47**) for possible cytotoxic effects to mammalian cells, we tested their effect on the cell membrane using LDH release assay, indicative for membrane damage, at the extended concentration range (Figure S4). Diazaborines **41** and **47** did not significantly affect the integrity of mammalian cell membrane after 24 h incubation at the concentration up to 750 μM. Diazaborine **11** showed minor LDH leakage at the concentration of 250 μM which increased to about 20% at 750 μM concentration. Diazaborine **13** caused most LDH leakage, showing 16% cytotoxicity at the concentration of 250 μM. The results show the same trend as IC_50_ values obtained with ATP-based cell viability assay (Table 1): cytotoxicity decreases in a row diazaborine **13** > **11** > **41** ≥ **47**. Overall, even for diazaborines **11** and **13** detectable membrane damage was observed only at concentrations exceeding their MIC in *E. coli* by about 40 times.

### Time-Kill Kinetics

To assess the dynamic interactions between antimicrobial agents and bacteria, we investigated time-kill kinetics of diazaborine **11**, and triclosan as a control with the same target against *E. coli*. Overall, the compounds showed slow kinetics, typical of antibiotics with intracellular targets (Figure 2C). After 8 h, viable bacterial numbers decreased about 2 logs for diazaborine **11** and 3 logs for control compound triclosan. After 24 h, no viable bacteria remained in diazaborine **11**-treated wells. This experiment was performed 3 times in triplicate wells. After 24 h incubation, at least one triclosan-treated well demonstrated regrowth indicating development of resistant bacteria. In contrast, all diazaborine-treated wells remained clear.

### Resistance Development

Resistance-related parameters like MPC and spontaneous frequency of resistance help to determine the dose of the new compound in preclinical in vivo studies.^33^ MPC represents a minimum concentration capable of preventing the proliferation of single-step resistant mutants. The difference between MIC and MPC defines the mutant selection window, and lower MPC/MIC ratio values indicate greater ability to prevent mutant formation. For all tested compounds (diazaborines **11**, **13**, and **41**), the MPC/MIC ratio was 32, which is 2 times higher than MPC/MIC ratio for control antibiotic ciprofloxacin (Figure 2D). It must be noted that the MPC/MIC ratio is both antibiotic- and bacterial strain-dependent, complicating comparisons between different studies. The MPC/MIC values reported in the literature for ciprofloxacin in the same quality control strain are between 11 and 16 ^34–36^ which is close to our value, given intrinsic variation of the methodology. Similar ratios were reported for other fluoroquinolones e.g., pradofloxacin, enrofloxacin, and marbofloxacin.^34^ For fabimycin, a compound which is believed to have the same intracellular target as diazaborines, the MPC/MIC ratio of 32 has been reported,^20^ similar to our compounds.

Diazaborines are subject to resistance arising from the acquisition of single-point mutations in the *fabI* gene encoding for their target enoyl-acyl carrier protein reductase FabI. We measured the spontaneous frequency of resistance to diazaborines in *E. coli* at the half MPC compound concentration (Figure 2D). Spontaneous mutations arose in vitro at low frequencies 2 × 10^–8^ to 9 × 10^–9^ which was comparable to reference fluoroquinolone antibiotic ciprofloxacin (1 × 10^–9^).

### *In Vivo* Evaluation

The use of mammalian models is essential for the evaluation of drug safety and efficacy. The larvae of the great wax moth *Galleria mellonella* have shown to be an excellent alternative, combined with low cost, easy maintenance, and inoculation, and the ability to generate results within 24–72 h. Moreover, their use does not require any legal or ethical permits. The larvae are responsive at 37 °C, which allows for temperature-dependent virulence factors of human pathogens to be active.^37^ For this study, we initially determined the infective dose of *E. coli* ATCC25922 based on 50% lethality within 24 h (LD_50_). This was set to be 3 × 10^5^ cfu/mL. The results presented in this study are derived from experiments conducted during the winter and early spring months, when colder temperatures during transportation were faced, which may have influenced larval physiology. Previous work by Mowlds and Kavanagh^38^ demonstrated that exposure of *G. mellonella* larvae to 4 °C prior to infection increased larval survival followed infection, potentially through induction of antimicrobial peptides and increased hemocyte density, thus justifying not achieving the expect mortality rate within 24 h (LD_50_, Figure 3), which was based on previous determinations. Importantly, survival in infected groups was significantly lower than that of our PBS control group, and all statistical comparisons were performed relative to the infected group, thus taking into account possibly lower mortality. Moreover, each experimental condition comprised 36 *G. mellonella* larvae, a group size selected to provide sufficient replication, thereby strengthening the statistical analysis and the conclusions drawn from the *in vivo* assessment.

**Figure 3:**
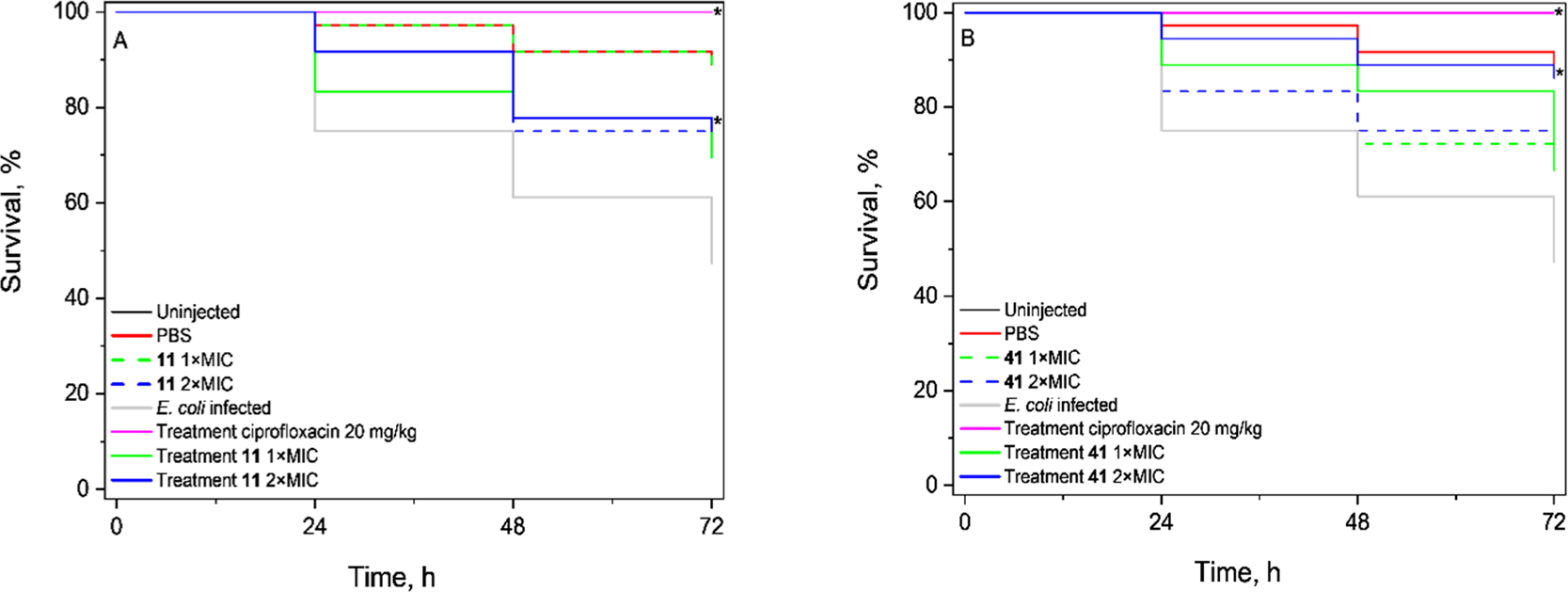
Effect of treatment on the survival of *G. mellonella* larvae infected with *E. coli* ATCC25922 (*n* = 36) with (A) diazaborine **11** and (B) diazaborine **41**, up to 72 h postinfection. Treatments were administered at 1 × MIC and 2 × MIC, 1 h after inoculation. Ciprofloxacin (20 mg/kg) was used as positive control. *Asterisks indicate statistically only significant treatment in comparison to infected, nontreated group (*p* < 0.05). Experiments were performed three times with *n* = 12 per group.

Diazaborines **11** and **41** were tested in *G. mellonella* larvae for their ability to rescue *E. coli* infection and evaluate any toxic effects. Diazaborine **13** was excluded from this final stage due to less favorable cytotoxicity properties in cell culture (Table 1 and Figure S4). As a treatment control, ciprofloxacin was used at a concentration of 20 mg/kg. The results are summarized in Figure 3 and S5. Ciprofloxacin performed as expected against *E. coli* by clearing the infection in 100% of the larvae (Figure 3). In our experiments, the PBS-injected group had an average survival rate of ≥80%. Some larvae were older than optimal at the time of use (due to shipment time frame), which may have contributed to the slightly lower baseline survival rates observed in the assays (observed as well in the survival rate of the noninjected group). Later stage of *G. mellonella* life cycle is known to affect survival and immune function.^39^ This biological factor, combined with the mechanical stress of injection, likely contributed to the observed decrease in the optimal survival rate (i.e., ≥90%) in our study, which has also been observed by others.^40,41^

Diazaborine **11** treated the infection caused by *E. coli* at a therapeutic dose of 1.13 mg/kg, which corresponds to a concentration of 2 × MIC, being significantly different to nontreated, infected group (*p* < 0.05). Survival rates were on average 75%. The group of larvae treated with a lower concentration of diazaborine **11**, i.e., 0.563 mg/kg, corresponding to 1 × MIC, did not significantly differ from the nontreated, infected group (*p* = 0.06226), albeit having a survival rate of 70% (Figure 3A). At a higher dosage, i.e., 2.81 mg/kg, diazaborine **11** was equally effective as the reference antibiotic ciprofloxacin (*p* > 0.05). No significant toxic effect was observed up to 72 h with the highest concentration tested, i.e., 5.63 mg/kg (10 × MIC), when compared to the PBS-only injected group (Figures 3A and S5A).

Diazaborine **41** at 0.915 mg/kg did not treat the infection, rescuing on average 72.5% of the infected larvae (*p* = 0.06683, Figure 3B). However, diazaborine **41** effectively rescued *Galleria* from *E. coli* infection at therapeutic dose of 1.83 mg/kg (*p* < 0.05) with a survival rate of 87.5% at 72 h postinfection. Unfortunately, at this same concentration, it was significantly toxic, i.e., survival rates of noninfected groups with diazaborine **41**-injected and PBS-injected controls were 70% vs 87.5%, respectively (*p* = 0.06687, Figure 3B). It is known that bacteria-induced *Galleria* has the immune system activated by the infection process,^42^ thus we speculate that the activation of this mechanism of defense could have offered an advantage toward the toxicity of diazaborine **41**, thus still showing significant survival rate for the infected group.

Still diazaborine **41** at the highest dosages tested (4.57 mg/kg and 9.15 mg/kg) did not achieve similar results as diazaborine **11** and ciprofloxacin (*p* < 0.05) with significantly lower survival rates for infected groups (67.5% and 70%, respectively, Figure S5B).

### Early Computational ADME Analysis

Predicted ADME properties of the synthesized compounds were evaluated using computational approaches (Figures S6 and S7 and Table S7). Specifically, QikProp software^43^ and the SwissADME online tool^44^ were used to predict key pharmacokinetic parameters, such as blood–brain barrier penetration, cytochrome P450 enzyme inhibition, and other relevant ADME descriptors (Table S7). To assess the potential oral availability and pharmacological properties of the compounds, Lipinski’s rule of five was employed as a guideline.^13,45^ All compounds fell within the recommended range (MW < 500 Da), with the majority exhibiting molecular weights between 300 and 400 g/mol. The distribution of calculated octanol–water partition coefficient (log *P*) values indicated a peak above 0 units, suggesting a favorable balance between hydrophilicity and lipophilicity. The frequency distribution analysis showed a maximum frequency for hydrogen bond donors (HBD) at 2 units, and for hydrogen bond acceptors (HBA) at 5 units, well within the recommended limits.^18^ Gastrointestinal absorption and blood–brain barrier permeability, crucial determinants of a drug’s bioavailability, were evaluated based on log *P* and topological polar surface area (PSA) descriptors. This evaluation used the BOILED-Egg model^46^ via the SwissADME tool (Figure S7). All compounds exhibited favorable gastrointestinal absorption and blood–brain barrier permeability. For instance, diazaborines **11** and **47** were identified as nonsubstrates for P-glycoprotein (P-gp), suggesting that their bioavailability may not be compromised by this efflux transporter.^19^ Through SwissADME, we assessed the potential of the synthesized compounds to inhibit key CYP450 isozymes involved in drug metabolism including CYP1A2, CYP2C9, CYP2C19, CYP2D6, and CYP3A4. Diazaborines **11** and **47** exhibited no inhibition across all five subfamilies analyzed. However, 19% of the compounds demonstrated inhibitory activity toward a single CYP subfamily (Table S7). These findings suggest good potential for oral bioavailability and desirable pharmacokinetic profiles, providing a solid foundation for further development and optimization of these compounds as drug candidates.

## Conclusions

Although boron plays a crucial role in the enzymatic systems of higher plants and animals, the application of boron-containing compounds in medicine is currently less advanced than that of other elements. This study presents scientific evidence of potential of diazaborine compounds as antimicrobial agents against Gram-negative pathogens, including e.g. *E. coli*, *S. enterica* ser. Typhimurium, *A. baumannii,* and *K. pneumoniae*. Cytotoxicity profiling suggests that the toxic effects are not inherent to the diazaborine family but are dependent on the specific compound structure. Performance and stability of optimized diazaborine scaffold **11** are comparable to those of the recently reported prospective antibacterial compound fabimycin, which targets the same enoyl reductase FabI. The observed synergy with the last-resort antibiotic colistin opens possibilities for the use of diazaborines in combination treatments.

## Experimental Section

### Compound Synthesis

Generally, we followed the procedures described below. Further details on the synthesis of individual compounds as well as NMR spectra (Figures S8–S561) are described in the Supporting Information.

Unless stated otherwise, all the synthetic manipulations were conducted under an argon atmosphere either in a glovebox or using conventional Schlenk techniques on a dual-manifold gas-inlet/vacuum line. All glassware was flame-dried before use. HPLC-quality grade reaction solvents were dried by conventional methods, thoroughly degassed with three freeze–pump–thaw cycles, and stored over flame-activated 3 Å and 4 Å molecular sieves on the glovebox. The solvents required to conduct the rest of the synthetic operations were purchased at HPLC-quality grade and used as received. Deuterated solvents were purchased at the highest purity level and used without further purification. Quantitative flash column chromatography was carried out using silica gel (Merck Silica Gel 60 Å, 230 × 400 mesh), or deactivated silica gel (packed into the column with the solvent system of choice containing 5% in Et_3_N and subsequently washed twice with the same solvent system free of Et_3_N). The eluent is quoted in volume ratios (v1/v2). All nuclear magnetic resonance (NMR) experiments (^1^H, ^13^C, ^19^F, ^11^B) were performed on Bruker AVANCE NEO spectrometers operating with the frequency, deuterated solvent, and temperature indicated in parentheses. All chemical shift values (δ) are reported in parts per million (ppm) downfield in relation to tetramethylsilane using the residual undeuterated solvent signal as the secondary internal standard (CHCl_3_ in CDCl_3_; δ_H_ = 7.26 ppm and δ_C_ = 77.16 ppm), (CHDCl_2_ in CD_2_Cl_2_; δ_H_ = 5.32 ppm and δ_C_ = 53.8 ppm), and (C_6_HD_5_ in C_6_D_6_; δ_H_ = 7.16 ppm and δ_C_ = 128.1 ppm). The resonances on the ^19^F NMR spectra are reported in parts per million (ppm) downfield of CFCl_3_, using hexafluorobenzene (C_6_F_6_ at −164.9 ppm) as an internal standard. Unless otherwise noted, all coupling constants (*J*) are quoted to the nearest 0.1 Hz with the involved nuclei subscripted, and the resonances are noted as follows: ^1^H: δ chemical shift in ppm (number of protons, multiplicity, *J* value(s), assignment). ^13^C: δ chemical shift in ppm (multiplicity [if applicable], *J* value(s) [if applicable], assignment). ^19^F: δ chemical shift in ppm (number of fluorines, multiplicity, *J* value(s), assignment). Splitting patterns are denoted as s (singlet), d (doublet), t (triplet), and q (quartet) in ^1^H NMR or quaternary in ^13^C NMR, p (pentet), s (sextet), m (multiplet), dd (doublet of doublets), ddd (doublet of doublet of doublets), dt (doublet of triplets), td (triplet of doublets), br (broad resonance), and app (apparent). Unless otherwise stated, both of the resonances on the ^13^C and ^19^F NMR spectra were proton-decoupled. High-resolution electrospray-ionization mass spectra (ESI-MS) were recorded on a Bruker microTOF mass spectrometer operated in a positive- or negative-ion mode, using a 0.05 M solution of sodium formate as a calibrant. Stock solutions (400–1000 ppm) were prepared in methanol (99.9% VWR Chemicals, France) or acetonitrile (99.9% Honeywell, Riedel-de Haën) and were diluted (3 ppm) with either 0.05% FA/MeOH solution 70/30 (v/v %) or acetonitrile prior to the measurement. Only the prevalent ion peak (HCOO^–^, H^+^, or Na^+^ adduct) is given for each compound. Elemental analysis was performed with an automatic elemental analyzer vario MICRO cube (HANAU Elementar Analysensysteme GmbH, Germany). The sample was weighted for three repetitions about 1800 mg into tin boats. The packing material was handled with a tweezers. Boats were closed gas-tightly. Foiled samples were compressed to remove air. Before the sample analysis, the instrument was stabilized.

### General Method A for the Syntheses of Diazaborines

Unless stated otherwise, an EtOH/H_2_O solution (1:1, 0.2 M) of the corresponding hydrazide (1 equiv) was slowly added to an EtOH/H_2_O solution (1:1, 0.2 M) of the corresponding *o*-formylphenylboronic acid (1 equiv), and the resulting reaction mixture was vigorously stirred for 1–3 h at room temperature (RT) under argon. Then, a precipitate was formed in the solution, which was subsequently filtered rinsing with water. The resulting solid was dried under high vacuum to afford the corresponding diazaborine with a purity amenable for biological evaluation (≥95%).

### General Method B for the Syntheses of Diazaborines

Unless stated otherwise, the corresponding hydrazone (1 equiv) and anhydrous iron­(III) chloride (0.07 equiv) were dissolved in anhydrous 1,2-dichloroethane (final concentration 0.05 M) in a three-necked round-bottom flask equipped with the septum, reflux condenser, and magnetic stirrer under argon. Then, boron tribromide (3.06 equiv) was slowly added through the septum, which was replaced by a glass stopper before refluxing the solution for 1–2 h at 70–80 °C. Then, the reaction was worked up via the following two procedures:

Workup 1: the reaction mixture was cooled to RT and slowly quenched with water upon stirring. Caution: vigorous evolution of gas occurs at the beginning of the quenching. Then, the organic phase was washed with three portions of water, which were discarded. The organic phase was then extracted three times with a 1 M aqueous solution of NaOH. The aqueous layers were combined and slowly acidified with a 1 M HCl solution until pH ∼ 2. The resulting water solution was extracted three times with dichloromethane. These organic fractions were combined, washed with brine, dried over Na_2_SO_4_, filtered, evaporated, and dried under high vacuum to afford the corresponding diazaborine with a purity amenable for biological evaluation (≥95%).

Workup 2: the reaction mixture was cooled to RT and slowly quenched with water upon stirring. Caution: vigorous evolution of gas occurs at the beginning of the quenching. Then, the organic phase was washed with three portions of water which were discarded. The remaining organic phase was extracted three times with a 1 M aqueous solution of NaOH. The aqueous phases were combined and slowly acidified with a 1 M HCl solution until pH ∼ 2. The resulting water solution was extracted three times with ethyl acetate. The resulting combined organic extracts were washed with brine, dried over Na_2_SO_4_, filtered, and evaporated. The remaining solid residue was suspended in 30 mL of diethyl ether, shaken, filtered, and washed additionally with 15 mL of ether, and finally dried under high vacuum to afford the corresponding diazaborine with a purity amenable for biological evaluation (≥95%).

### General Method C for the Syntheses of Diazaborines

Unless stated otherwise, the Bpin derivative of the corresponding *o*-formylphenylboronic acid (1 equiv) was dissolved in a mixture of CH_3_OH/H_2_O (1:1, 0.1 M) and argon was bubbled through the solution before adding the corresponding hydrazide (1 equiv) and stirring the resulting solution for 2 h at 80 °C. Then, a precipitate was formed in the solution, which was subsequently filtered rinsing with water. The resulting solid was dried under high vacuum to afford the corresponding diazaborine with a purity amenable for biological evaluation (≥95%).

### General Method D for the Synthesis of Diazaborines by the Reduction of Aromatic Nitro Groups

Unless stated otherwise, the corresponding diazaborine bearing an aromatic nitro group and ammonium chloride (15 equiv) were suspended in a H_2_O/MeOH mixture (1:1, 0.1 M). Then, iron turnings (10 equiv) were added portionwise to the solution, which was subsequently stirred at 70 °C for 2 h. Then, the mixture was cooled, and the liquid phase was transferred to a separating funnel where it was extracted with CHCl_3_. The resulting organic layer was washed several times with a 0.5 M aqueous solution of HCl until the coloration of the water phase disappeared. Subsequently, the resulting organic phase was successively washed with water until the water layer reached neutral pH. Then, the organic layer later was washed with brine, dried over Na_2_SO_4_, evaporated, and dried under high vacuum to afford the corresponding diazaborine with a purity amenable for biological evaluation (≥95%).

### Compound Purity Assessment

The purity of the compounds is ≥95%, which was quantitatively determined by ^1^H or ^19^F NMR spectroscopy using relaxation delay values “*d*_1_” five times the typical *T*_1_ values for the slowest relaxing signals in medium-sized molecules that is *d*_1_ = 30 s (for ^1^H NMR quantification) and *d*_1_ = 30 s (for ^19^F NMR quantification). The NMR spectra of **a** are shown in Figures S8–S561. In addition, the purity of most promising compounds (**11**, **13**, and **41**) was assessed by HPLC. The HPLC analysis was performed on a Waters Acquity ultrahigh-performance liquid chromatography (UHPLC) system equipped with a Waters Acquity photodiode array (PDA) detector. Samples (20 μM) were analyzed using a 10 μL full-loop injection onto an Omega Luna analytical column (Polar C18, 1.6 μm, 100 Å, 50 × 2.1 mm) maintained at 30 °C. The mobile phase consisted of 15 mM phosphate buffer in water (A) and acetonitrile (B). Separation was achieved using a 5 min linear gradient from 10% B to 80% B at a flow rate of 0.5 mL/min. PDA detection was carried out over the 205–370 nm range with a spectral resolution of 1.2 nm and a sampling rate of 20 Hz. Chromatographic data were acquired and processed using Waters Empower software with ApexTrack peak integration with the following wavelengths: 259 nm for compounds **11** and **13**, and 285 nm for compound **41**. The chromatograms are shown in Figures S649–S651. Compound **11**, **13**, and **41** purities were 96.8, 96.4, and 96.8%, respectively.

### Antibacterial Activity Assays

Initial compound screening was performed at 50 μM against four clinical bacterial strains: *E. faecalis* ATCC29212, *E. coli* ATCC25922, *P. aeruginosa* ATCC27853, and *S. aureus* ATCC29213 and growth inhibition was determined. For the most potent diazaborine derivatives, similar assays were performed against other human pathogenic Gram-negative bacterial strains: *A. baumannii* ATCC19606, *Klebsiella aerogenes* ATCC13048, *K. pneumoniae* ATCC700603, *S. enterica* serovar Typhimurium ATCC19585, enteropathogenic *E. coli* CB9615 (clonal strain of enterohemorrhagic *E. coli*), and uropathogenic *E. coli* CFT073 (DSM 103538) and UMN026 (ATCC BAA1161). Strains were obtained from Microbiologics Inc. (St. Cloud, Minnesota, USA) or the Leibniz Institute DSMZ German Collection of Microorganisms and Cell Culture. For compounds displaying ≥50% growth inhibition against *E. coli* (*n* = 38) and *S. aureus* (*n* = 7), MICs were determined by dose–response assays. For initial MIC assays (i.e., *E. coli* and *S. aureus*), the range tested was 125, 100, 75, 50, 25, 12.5, 6.25, and 3.13 μM. For additional bacterial strains, the range tested was 75, 50, 25, 12.5, 6.25, 3.13, 1.56, and 0.78 μM. Triclosan control (Merck) was tested as 2-fold serial dilutions at 50–0.2, 25–0.1, or 6.25–0.04 μM depending on the strain. Antimicrobial assays were performed by the broth microdilution method in the 96-well plate (Nunc, Thermo Fisher Scientific) format according to the CLSI guidelines.^17^ All bacterial strains were stored at −80 °C, and working cultures were maintained on Mueller–Hinton Agar (MHA, Lab M Limited, Lancashire, UK) plates and stored at 2–8 °C. Fresh bacteria were routinely grown onto MHA plates at 37 °C for 16–20 h prior to each assay. Briefly, few colonies were taken from MHA overnight culture, inoculated into 0.9% saline solution, and vortexed to ensure that the bacterial suspension was homogeneous. Bacterial suspensions were analyzed using a DEN-1 densitometer (BioSan) and adjusted to 1 × 10^6^ colony forming units (cfu/mL) by diluting with cation-adjusted Mueller–Hinton broth (CAMHB, BD). *E. faecalis* experiments were performed in brain heart infusion broth (Sigma-Aldrich). Compound stocks were prepared in DMSO (VWR). In each well, 100 μL of bacterial suspension was added into 100 μL of compound solution diluted into assay media. Plates were incubated for 24 h at 37 °C. Absorbance values measured at 620 nm were used for evaluating the antimicrobial effects of test compounds by comparing to untreated controls and expressed as percentage inhibition of growth. Wells with media only were used as background controls. Ciprofloxacin (ICN Biomedicals) was used as a positive control on every assay plate at MIC (previously determined in our laboratory) for each bacterium, i.e., *E. coli* (0.05 μM), *S. aureus* (1.5 μM), *P. aeruginosa* (3 μM), *E. faecalis* (3 μM), *A. baumannii* (3.6 μM), *K. aerogenes* (0.12 μM), *K. pneumoniae* (1.5 μM), *S. enterica* serovar Typhimurium (0.05 μM), enteropathogenic *E. coli* CB9615 (0.06 μM), and uropathogenic *E. coli* CFT073 (0.12 μM) and UMN026 (0.02 μM). To account for solvent effects, a diluent control (DMSO) was included in all of the assay plates. Screening experiments were performed once in triplicate, while MIC determinations were performed three times in triplicate, unless stated otherwise.

### Freeze–Thaw Stability Assay

In the typical experiment, 0.047 mmol diazaborine of choice and 0.047 mM 1,3,5-trimethoxybenzene were dissolved in 0.7 mL of DMSO-*d*_6_ and the ^1^H NMR spectrum (number of transients 8, relaxation delay 20) was recorded at 37 °C. The NMR tube was put into −20 °C for 23 h and defrozen for 1 h at RT, and the ^1^H NMR spectrum was recorded again with the same parameters. After that the NMR tube was again put into −20 °C. Cycle was repeated for 5 days. Degree of decomposition was measured by calculating the concentration from NMR quantification against 1,3,5-trimethoxybenzene as the internal standard (Figures S563–S597).

### Plasma Stability Assay

For plasma stability assessment, 1.633 mM diazaborine **62**, **64**, **65**, or **66** was dissolved in 4 mL of D_2_O, added to 36 mL of human blood plasma, and stirred for 72 h at 37 °C in a closed 100 mL round-bottom flask at 100 rpm. Aliquots were taken after 0, 1, 2, 4, 8, 16, 24, 48, and 72 h. For the measurement, 1.5 mL of suspension was taken by the measuring pipet, evaporated in the rotavapor with heating below 37 °C, and additionally dried in vacuo for 1 h to remove residual water. Solid rests were redissolved in 1 mL of the equimolar stock solution of acetamide in D_2_O, and ^1^H NMR spectrum was measured (number of transients 8, relaxation delay 20 s). Baseline for each single spectrum was corrected. The concentration in each point was calculated from integrations of characteristic peaks of diazaborine against acetamide in ^1^H NMR spectra (Figures S599–S648). Additionally, for the initial point and 72 h point, ^11^B NMR spectra (number of transients 512, relaxation delay 1) were measured and compared (Figures S603, S608, S613, S618).

### Checkerboard Assays

MICs of colistin and selected compounds were determined again during the checkerboard assays with the antimicrobial activity of the combinations. Some compounds had a shift in MIC, when compared to previously determined in the chapter Antibacterial Activity Assays. This is probably due to small differences in methodologies between assays. Checkerboard assays were carried out using MHB and 96-well microtiter plates. Each well was inoculated with 100 μL of a suspension of 5 × 10^5^ cfu/mL of *E. coli* ATCC25922. Compounds and colistin serial dilutions were distributed in deep-well plates (Abgene, Thermo Fisher Scientific) and inoculated into plates (i.e., 1.6 μL). For compound dispensing, an automated liquid handler Biomek i7 (Beckman Coulter) was used. Absorbance results at 612 nm were measured before and after incubation at 37 °C for 24 h. Data were obtained in three independent experiments with one replicate per combination. DMSO 100% was added proportionally into wells as control when one or no compound was added to ensure similar concentrations in all the wells. To evaluate the synergistic, additive, indifference, or antagonistic effect of the combinations, the fractional inhibitory concentration index (FICI) was calculated according to CLSI (2015)^17^ as follows: FICI = MIC_AB_/MIC_A_ + MIC_BA_/MIC_B_ where MIC_AB_ = MIC of A in the presence of drug B; MIC_A_ = MIC of A alone; MIC_BA_ = MIC of B in the presence of drug A; and MIC_B_ = MIC of B alone.

### Cytotoxicity Assessment

The HepG2 human hepatocarcinoma cell line (ECACC85011430, obtained from ECACC) was maintained in high glucose DMEM with Glutamax and 10% (v/v) fetal bovine serum (FBS). Hs27 human normal fibroblast cell line (ATCC CRL-1634, kindly provided by Dr. Carmen Escobedo-Lucea from the University of Helsinki) was maintained in MEM supplemented with Glutamax, nonessential amino acids, and 10% FBS. All cell culture reagents were obtained from Gibco. Both cell lines were cultivated at 37 °C and 5% CO_2_. One day prior to the experiment, the cells were seeded to white frame and clear bottom 96-well plates (PerkinElmer) at the density of 10,000 cells/well for HepG2 cell lines and 5000 cells/well for Hs27 cell lines. The cells were grown at 37 °C, 5% CO_2_ until they reached 50–70% confluency. Stock solutions of test compounds and a positive control (camptothecin, Sigma-Aldrich) were prepared in DMSO and diluted into assay medium (growth medium with 5% FBS) to the final concentration. For dose–response experiments, 2-fold serial dilutions of compounds and triclosan were prepared in DMSO to achieve the final DMSO concentration of 0.5% in all samples. To account for solvent effects, a diluent control (DMSO) was included in all assay plates. The culture medium was removed from the plate, and compounds were added, 200 μL/well. After 24 or 72 h incubation, the amount of ATP, which is directly proportional to the number of viable cells present in culture, was quantified using the CellTiter-Glo Luminescent Cell Viability kit (Promega), according to manufacturer’s instructions. Origin Graphing and Analysis, version 9.55 (OriginLab) was used for determining IC_50_ values. In LDH release experiments, HepG2 cells were incubated with 100 μL of compounds for 24 h. Then, 50 μL of medium was collected and analyzed with CyQUANT LDH cytotoxicity assay kit (Invitrogen) according to manufacturer’s instructions.

### Time-to-Kill Assay

Compound dilutions were prepared at two times final concentration in liquid culture medium and dispensed 500 μL per well onto a deep-well microtiter plate (Nunc 96 DeepWell). Then, a suspension with bacterial concentration of 10^7^ cfu/mL was prepared, 500 μL was added per well, and the mixture was mixed with the compound dilutions. For the colony count assay, 30 μL samples were collected at each time point onto an empty clear 96-well microtiter plate and serially diluted 1:10 in PBS. Total of 4–6 dilutions were prepared, and 10 μL of each dilution was dispensed onto gridded square Lysogeny Broth Agar (LBA, Hispanlab) plates in triplicate. Plates were incubated overnight at 37 °C, and colonies were counted from dilutions which resulted in 5–25 colonies.

### Resistance Study

MPC was determined as the lowest antibiotic concentration that prevents the growth of the least susceptible first-step resistant mutant among a large bacterial population of 10^10^ cells. A colony from a fresh overnight culture grown on LBA plate was used to inoculate 100 mL of MHB and incubated at 37 °C with shaking for 24 h. On the day of the assay, the culture was pelleted by centrifugation at 20 °C, 3000 rpm for 10 min, and the cells were resuspended in 5 mL of fresh 0.9% saline and used for the assay. MHA plates were prepared with compound concentrations ranging from 1 × MIC to 64 × MIC, increasing by a factor of 2. Plates containing DMSO with the same concentration as in the highest compound plate (1% or less, depending on the compound) were used as controls. To measure MPC, 100 μL of the bacterial suspension was spread on agar plates. After incubation at 37 °C for 48 h, the plates were examined for the presence of colonies. Seven 10-fold dilutions of the initial suspension were prepared and plated to evaluate the bacterial concentration in the original suspension. Frequency of spontaneous mutation was determined as the number of colonies grown on the plate with compound divided by the number of colonies grown on the compound-free plate (taking into account the dilution of the original suspension).

### *In Vitro* FabI Enzyme Inhibition Assay

Inhibition of *E. coli* FabI was evaluated using a continuous spectrophotometric NADH consumption assay in the 384-well format (20 μL final volume). Recombinant *E. coli* FabI enzyme, a kind gift from Prof. Deborah T. Hung (Broad Institute of MIT and Harvard, USA), was assayed in reaction buffer consisting of 50 mM HEPES (pH 7.4), 150 mM NaCl, 1 mM EDTA, 0.01 mg/mL BSA, and 0.01% Tween-20. Final assay conditions were 25 nM FabI, 50 μM NAD^+^ (Sigma-Aldrich), 330 μM crotonyl-CoA (Creative Enzymes), 380 μM NADH (Roche Diagnostics), and ≤0.02% DMSO. For *K*_i_ determination, compounds (including diazaborines **11** and **13**, and triclosan as control) were tested in nine-point 2-fold serial dilutions. Each concentration was assayed in triplicate wells with two independent biological replicates. Compounds and controls were dispensed into low-absorbance flat-bottom 384-well plates, and 50 μM NAD^+^ and FabI was added and incubated at room temperature for 20 min. Then, the reaction was initiated by adding a 5× crotonyl-CoA substrate/NADH mix to achieve the final assay concentrations. Control wells lacking an enzyme or substrate were included on each plate. Plates were immediately transferred to a Varioskan LUX microplate reader and shaken briefly (3 s), and NADH absorbance was monitored kinetically at 340 nm at 25 °C. Initial reaction rates were determined from the linear portion of the progress curves. Percentage activity was calculated relative to DMSO-only controls, with background correction using no enzyme and no-substrate wells. Inhibition constants (*K*_i_) were obtained by fitting percentage activity versus inhibitor concentration to Morrison’s quadratic equation using GraphPad Prism 10.1.2. In a separate experiment, a small panel of selected diazaborines was evaluated at a single concentration of 2 μM and tested in duplicate wells with two independent biological replicates.

### *In Vivo* Studies Using the *G. mellonella* Larvae Model

We evaluated the potential of selected compounds to rescue *G. mellonella* larvae from *E. coli* ATCC25922 infection as well as the toxicity of those compounds at their treatment concentrations. Batches of *G. mellonella* larvae (ReptileManiacs Ltd., Nokia, Finland) in their final instar stage were obtained weekly, stored in the dark at RT, and used within 5 days from shipment. Larvae were sourced by the supplier from overseas (Netherlands). This fact limits our control over age, rearing conditions and genetic background of the batches acquired. Larvae weights varied from 300 to 400 mg. Any larvae with excessive melanization was discarded. Larvae were grouped (*n* = 12) in Petri dishes, and groups were weighed before each assay. Groups were adjusted to have similar average weights. Prior to injections, groups of larvae were kept at 20 °C in the dark. Bacterial infection of *G. mellonella* was performed as follows. Colonies from an overnight *E. coli* ATCC25922 culture grown in MHA plates were used to prepare the bacteria inoculum at the final concentration of 3 × 10^7^ cfu/mL in PBS. The infective inoculum was previously determined in our laboratory based on the median lethal dose (LD_50_) at 24 h postinfection. The LD_50_ was calculated based on a nonlinear sigmoidal dose–response curve using OriginPro software (OriginPro, Version 2023b). Bacterial colony counts on MHA plates were used to confirm the inoculum size.

For all the injections, a glass syringe (Hamilton 701N, needle size 26 s, cone tip) was used to inject 10 μL aliquots of the inoculum/treatment into the hemocoel of each larva via the last pair of proleg. The bacterial injection was performed in the left proleg. After injection, larvae were incubated in Petri dishes in the dark at 37 °C for 1 h. All larvae were confirmed to be alive 1 h postinfection. For the treatment, the tested diazaborines **11** and **41** were injected at the following concentrations: 1 × MIC and 2 × MIC and for toxicity studies at 1 × MIC, 2 × MIC, 5 × MIC, and 10 × MIC via the last right proleg. Compound stocks were prepared in DMSO and further diluted in PBS. As a positive treatment control, ciprofloxacin at 20 mg/kg in PBS was used. This dose was previously determined in our laboratory to rescue ≥90% of *E. coli* 25922 infection after 24 h. In all experiments, there were two negative control groups; one group that did not receive any injection, while the other group was injected with PBS supplemented with DMSO at the highest concentration used (i.e., 25%). The safety of DMSO in *G. mellonella* has been validated in prior literature, where concentrations up to 30% have been shown to cause no significant toxicity or mortality in larvae.^47^ This enabled us to assess any impact related to physical trauma and/or DMSO toxicity. In addition, one group of larvae, which was infected with *E. coli* ATCC25922, received only PBS as treatment. To evaluate the toxicity of compounds, noninfected group of larvae (which received the initial left proleg injection of PBS only), was injected with the same compound concentrations used for the infection treatment. All groups were injected twice. Typically, there were no deaths in the uninjected control group and there was never more than one death within 24 h and maximum three deaths at end of assay (i.e., 72 h), per PBS-double injected group, in each experiment. Larvae were inspected every 24 h, up to 72 h postinfection, and were considered dead if they did not move in response to touch.

For statistical testing, data from triplicate experiments were pooled, resulting in ***n*** = 36. The pooled survival data were plotted using the Kaplan–Meier method, and differences in survival were calculated using the log-rank test, with a ***p*** value ≤0.05 indicating statistical significance. In all comparisons, the negative control used was the uninfected PBS-injected group, rather than the unmanipulated group. Treatments were evaluated in comparison to the group infected with *E. coli*, which did not receive any treatment. All statistical analyses were performed (OriginPro, Version 2023b). To estimate the mg/kg dosage of compounds, average individual larvae weigh was assumed as 350 mg.

### Computational ADME Prediction

The ADME properties of the synthesized compounds were predicted using both QikProp software^43^ and SwissADME web tool.^44^ Various descriptors, including the octanol/water partition coefficient (log *P*), PSA, and aqueous solubility (log *S*), were calculated to assess the pharmacokinetic profiles of the compounds. The SwissADME platform was used to generate the BOILED-Egg plot,^46^ which provides a visual representation of the compounds’ gastrointestinal absorption and brain penetration capabilities. Additionally, Lipinski’s rule of five^45^ was applied to evaluate the compounds’ drug-likeness.

## Supplementary Material







## Notes

Materials and methods, detailed synthesis description, and characterization of chemical products including HPLC traces and NMR spectra (PDF)
ADME prediction results (XLSX)
Molecular strings (CSV)

## Abbreviations

ADME: absorption, distribution, metabolism, and excretion
cfu: colony-forming unit
DMSO: dimethyl sulfoxide
FICI: fractional inhibitory concentration index
MPC: mutant prevention concentration
MIC: minimum inhibitory concentration
MHA: Mueller–Hinton agar
MHB: Mueller–Hinton broth
SAR: structure–activity relationship
