# Supplementary material for: Rediscovering Diazaborines: Synthesis and Bioactivity Profiling of Boron-Containing FabI Inhibitors against Gram-Negative Bacteria
Source: J Med Chem. 2026 Feb 7;69(4):3796–810. doi: 10.1021/acs.jmedchem.5c01766 (PMC12951463; doi:10.1021/acs.jmedchem.5c01766)
Supplement: Supplementary file 1 [file jm5c01766_si_001.pdf]

Supporting Information

Rediscovering Diazaborines: Synthesis and Bioactivity Profiling of Boron-Containing FabI Inhibitors against Gram-Negative Bacteria

Polina Ilina[1,#], Vladimir Iashin[2,#], Cristina D. Cruz[1], Juho Heininen[1], Iiro Järvi[1], Inna Pönniö[1], Sami Heikkinen[2], Pauli Johan Wrigstedt[2], Leo Ghemtio[1], Karina Moslova[2], Henri Xhaard[1], Paula Kiuru[1], Jesús Perea-Buceta[2], Päivi Tammela[1,*]

1 Drug Research Program, Faculty of Pharmacy, University of Helsinki, P.O. Box 56, FI-00014, Helsinki, Finland

2 Department of Chemistry, Faculty of Science, University of Helsinki, P.O. Box 55, FI-00014, Helsinki, Finland

[#] Equal contribution; [*]Corresponding author

Corresponding author email: paivi.tammela@helsinki.fi

TABLE OF CONTENTS

Biological evaluation and stability 3

Table S1. Bioactivity screening for 59 diazaborine compounds shown in Figure 1. 3

Table S2. Inhibition of *E. coli* FabI by selected diazaborine compounds 4

Figure S1. Biochemical inhibition of FabI from *E. coli*. 5

Table S3. Comparison of antibacterial activity of diazaborine compounds from this study to the same compounds in the study of Grassberger et al., 1984. 5

Table S4. Antibacterial activity screening of selected compounds against a panel of *Staphylococci* strains. 6

Figure S2. Charged diazaborines and conjugates. 7

Table S5. Antibacterial activity screening for charged diazaborines and conjugates shown in Figure S2. 8

Figure S3. Stability of selected diazaborines in DMSO and freeze-thawing tolerance. 8

Table S6. Testing of diazaborine 11 for synergistic activity against *E. coli* ATCC25922. Experimental procedure described in the Materials and Methods. 9

Figure S4. Mechanisms of cytotoxicity of diazaborines 11, 13, 41 and 47. 9

Figure S5. Survival of infected *Galleria mellonella* larvae after diazaborine treatment 10

Figure S6. Physicochemical property distributions of active vs. non-selected compounds. 11

Figure S7. Comprehensive BOILED-egg diagram analysis: contrasting global dataset with selected active compounds for gastrointestinal absorption and blood- brain barrier permeation potential. 12

Detailed synthesis procedures of diazaborine compounds 13
Detailed synthesis procedures of diazaborine precursors 41
NMR spectra (Figures S8-S648) 58
Diazaborines 58
Diazaborine derivatives 183
Diazaborine salts 185
Conjugates 207
Conjugates Diazaborine-Phosphonium salt 207
Conjugates Diazaborine-Aminoacid 215
Conjugates Diazaborine-Aminoacid-Phosphonium salt 221
Stability studies 305
Freezing-defreezing studies 305
Stability studies in human plasma at 37 °C 326
HPLC analysis of lead compounds (Figures S649-S651) 347

Biological evaluation and stability

Table S1. Bioactivity screening for 59 diazaborine compounds shown in Figure 1.
Antibacterial activity screening in *E. coli* ATCC25922, *P. aeruginosa* ATCC27853, *S. aureus* ATCC29213, *E. faecalis* ATCC29212 at 50 µM. For compounds demonstrating over 50% growth inhibition (shown in bold), minimum inhibitory concentration (MIC) was determined in dose-response experiments (three independent experiments). Cytotoxicity was determined by ATP-based cell viability assay after 72 h of incubation of cells at 250 µM. The cytotoxicity values represent median value of 3 independent experiments (performed in a single well) ± SD. Experimental procedures are described in the Materials and Methods.

**Diazaborine**	**Antibacterial activity at 50 µM, % of growth inhibition (MIC, µM)**				**Cytotoxicity at 250 µM, %**	
	E. coli	*P. aeruginosa*	*S. aureus*	*E. faecalis*	*HepG2 cells*	*Hs27 cells*
**Phenyl series**						
1	37.9	-1.0	13.6	-7.4	61.9±4.7	22.0±11.8
**2**	**69.5 (50)**	1.5	12.0	-4.4	95.8±1.3	57.8±8.4
**3**	**54.5 (75)**	8.9	8.4	0.0	57.7±5.9	34.7±10.4
4	25.1	-5.5	3.3	-12.9	94.7±1.4	49.9±10.7
5	24.7	4.2	17.2	4.5	8.9±7.1	18.3±9.1
6	28.2	7.2	12.4	6.3	0.8±3.1	12.3±10.1
7	36.7	1.4	8.3	-4.7	60.2±5.2	28.2±9.8
**8**	**99.9 (25)**	4.5	11.1	-7.6	61.6±10.2	27.8±14.5
**9**	**103.0 (12.5)**	1.1	0.8	-5.8	59.8±5.9	32.5±11.9
**10**	**99.0 (25)**	-2.0	-1.7	-18.6	98.6±6.5	83.2±8.2
**11**	**99.5 (6.25)**	4.2	-9.1	-12.8	91.8±5.2	49.3±6.8
**12**	**97.5 (50)**	1.1	-5.0	-18.6	60.6±5.3	34.8±10.2
**13**	**101.0 (12.5)**	-1.7	-3.3	-10.5	97.8±0.8	94.2±0.9
**14**	**61.8 (75)**	-1.7	3.3	-18.6	97.1±1.2	86.4±4.3
**15**	**98.0 (50)**	0.8	10.7	-8.1	97.9±0.7	100.0±0.4
**16**	**101.0 (50)**	-1.3	8.2	3.5	96.0±1.0	56.5±8.7
**17**	**100.0 (25)**	6.6	9.3	1.0	47.0±4.1	19.7±6.3
**18**	**58.9 (>125)**	-1.4	13.9	-6.0	72.3±4.1	51.8±5.3
**19**	**95.2 (25)**	-1.6	10.4	-5.4	87.5±3.9	49.9±6.4
**29**	**98.5 (12.5)**	-4.8	5.0	-16.3	88.3±3.5	58.0±5.1
**21**	**94.0 (50)**	-2.8	6.6	-1.2	98.6±1.1	97.5±0.5
**22**	**99.0 (12.5)**	-6.8	14.0	-18.6	99.8±0.7	98.4±0.3
**23**	**99.5 (25)**	-2.0	13.2	-7.0	99.9±0.6	99.0±0.2
**24**	**79.4 (75)**	-4.0	9.1	-5.8	95.4±1.6	56.1±6.6
25	26.7	-4.4	8.2	-3.5	98.3±0.2	96.7±0.3
26	39.2	-1.4	7.4	10.5	98.0±0.4	97.2±0.5
27	35.2	-4.5	8.3	-1.2	97.1±0.9	95.9±1.1
28	18.2	-2.0	7.3	-7.0	37.6±4.5	25.4±8.2
29	39.3	-0.2	11.2	-7.2	60.8±5.9	23.0±5.4
**30**	**59.3 (>125)**	-3.5	6.1	-4.9	65.1±4.0	22.4±8.6
31	43.3	-6.7	22.7	-2.1	70.6±2.6	36.2±8.8
**32**	**98.5 (25)**	-0.3	7.4	-15.1	83.8±1.4	45.2±12.1
**33**	**87.7 (75)**	-4.7	5.7	-17.6	98.2±0.5	96.7±0.4
**34**	**65.2**	0.8	12.8	-1.5	75.5±5.0	42.1±8.4
35	-1.3	-0.3	8.6	0.9	51.2±3.4	9.6±5.2
36	-2.6	-0.4	10.6	-5.6	58.3±5.2	21.6±8.5
37	24.6	-6.3	24.6	-30.6	54.6±6.4	22.9±7.2
**38**	**64.5 (>125)**	2.7	5.8	-6.5	98.7±2.1	96.0±5.4
**Thiophene series**						
**39**	23.5	-0.5	**66.4 (> 125)**	-0.5	51.2±16.6	19.7±6.9
**40**	36.9	-3.5	**57.7 (> 125)**	-1.3	62.9±5.1	15.0±5.5
**41**	**100.0 (12.5)**	9.3	22.8	2.3	51.1±3.3	21.1±6.7
**42**	**100.0 (25-100)***	9.5	**54.3 (>125)**	7.1	48.8±12.8	15.8±8.3
**43**	**100.0 (25)**	6.3	**62.0 (>125)**	9.7	93.1±2.4	64.2±8.2
**44**	**100.0 (12.5-100)***	6.9	**61.2 (>125)**	20.9	95.3±6.7	56.8±14.8
**45**	**100.0 (50)**	10.4	**55.9 (>125)**	9.4	91.9±8.1	34.4±11.4
**46**	**100.0 (25)**	7.4	1.1	5.1	5.4±6.8	9.7±2.7
**47**	**100.0 (6.25)**	7.4	3.4	2.9	11.4±25.0	10.6±7.7
**Diazaborine derivatives**						

48	4.5	4.5	-0.3	1.3	52.2±4.1	14.3±7.7
50	17.6	14.5	4.9	7.0	98.1±0.2	96.3±0.3
**49**	12.2	5.2	**84.8 (25)**	35.6	44.1±10.2	29.9±8.4
51	7.0	7.6	21.0	7.7	1.3±8.1	8.5±2.3
52	4.1	4.2	22.1	3.1	44.1±10.2	79.8±1.9
53	3.1	6.5	12.3	5.3	11.4±2.1	19.4±3.8
54	1.9	-0.2	23.9	1.6	32.6±4.8	1.3±8.36
55	28.9	-3.2	14.8	0.7	41.5±17.4	12.7±11.7
5	2.7	-1.2	6.2	2.9	26.0±12.0	7.8±10.45
57	33.8	2.1	11.9	0.7	15.7±22.0	2.2±12.4
**58**	**100.0 (25)**	4.2	11.4	4.6	96.8±2.9	87.0±13.7
**59**	**100.0 (50)**	8.3	12.1	3.9	93.8±2.8	55.1±5.3

*Compounds were excluded from further studies due to inconsistent results.

Table S2. Inhibition of *E. coli* FabI by selected diazaborine compounds.
Compounds are listed in order of increasing minimum inhibitory concentrations (MICs) against *Escherichia coli* ATCC 25922. FabI inhibition was assessed at 2 µM inhibitor concentration, and inhibition constants (Ki) were determined for selected compounds and for the known FabI inhibitor triclosan as a control. Ki values are reported as the mean of the two biological replicates (see **Figure S1** for Morrison-fitted FabI inhibition curves). Data obtained in individual biological replicates (Experiments) are reported in brackets. Experimental procedures are described in the Materials and Methods.

Compound	MIC in *E.coli* ATCC25922 (µM)	FabI inhibition at 2µM Mean, % (Experiment 1, Experiment 2)	Ki Mean, µM (Experiment 1, Experiment 2)
**Diazaborine 11**	6.25	80.3 (81.1; 79.6)	0.32 (0.33, 0.31)
**Diazaborine 22**	6.25	75.2 (67.0; 83.4)	ND
**Diazaborine 41**	12.5	27.0 (32.3; 21.8)	ND
**Diazaborine 13**	12.5	73.2 (75.9; 70.5)	0.34 (0.35, 0.33)
**Diazaborine 10**	25	50.5 (41.4; 59.5)	ND
**Diazaborine 17**	25	45.2 (48.5; 42.0)	ND
**Diazaborine 43**	25	17.9 (27.3; 8.4)	ND
**Diazaborine 46**	25	15.6 (25.5; 5.7)	ND
**Diazaborine 18**	>125	7.0 (17.0; -2.9)	ND
**Diazaborine 30**	>125	10.2 (17.1; 3.3)	ND
**Diazaborine 34**	>125	9.2 (21.2; -2.7)	ND
**Triclosan**	0.4	87.8 (84.8; 90.7)	0.041 (0.049, 0.032)

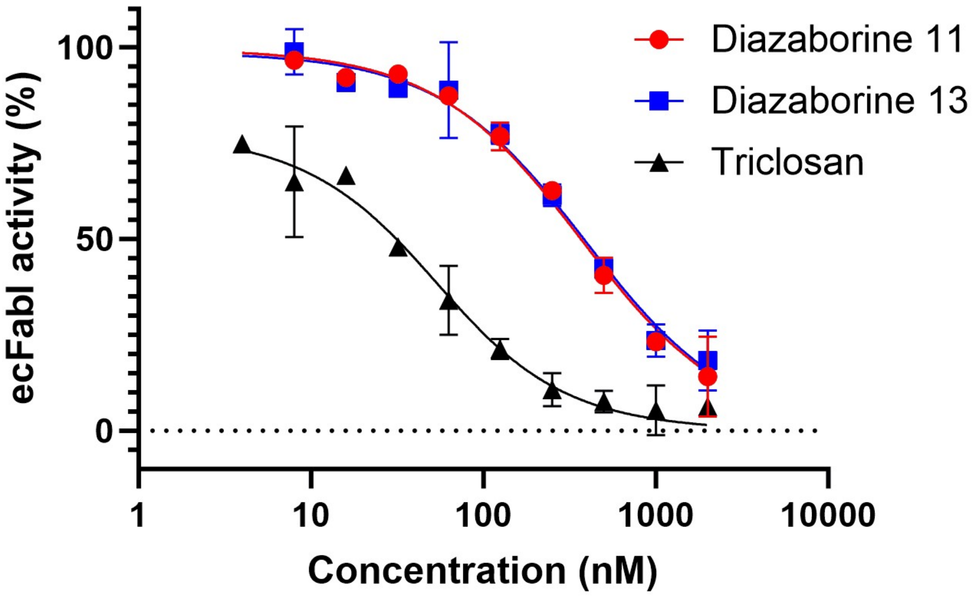

Figure S1. Biochemical inhibition of FabI from *E. coli*.
Inhibition constant Ki **(Table S2)** was calculated by fitting the data to Morrison's Quadratic model. Each data point represents the average of two biological replicates (performed in triplicate wells each) ± SD. Detailed experimental procedure described in the Materials and Methods.

Table S3. Comparison of antibacterial activity of diazaborine compounds from this study to the same compounds in the study of Grassberger et al., 1984.

	**MIC in *E. coli***		**MIC in *K. aerogenes****		**MIC in *S. typhimurium***		**MIC in *K. pneumoniae***	
**Diazaborine**	**ATCC 25922 µM**	**Δ120 µM (µg/ml)****	**ATCC 13048 µM**	**Δ220 µM (µg/ml)****	**ATCC 19585 µM**	**Δ119 µM (µg/ml)****	**ATCC 700603 µM**	**Δ217 µM (µg/ml)****
**9**	12.5	19.8 (6.25)	25	79.2 (25)	12.5	9.9 (3.12)	>50	4.9 (1.56)
**12**	50	39.8 (12.5)						
**17**	25	23.5 (6.25)						
**19**	25	15.2 (6.25)						
**20**	12.5	26.3 (10)	25	65.8 (25)	12.5	8.2 (3.12)	>50	(1.56)
**24**	75	66 (25)						
**30**	>125	>143 (>50)						
**35**	>50	>158 (>50)						
**41**	12.5	6 (1.56)	12.5	12 (3.12)	6.25	3 (0.78)	>50	1.5 (0.39)
**43**	25	8.41 (3.12)	25	33.7 (12.5)	12.5	2.1 (0.78)	>50	2.1 (0.78)
**47**	6.25	4.5 (1.25)	6.25	5.6 (1.56)	3.13	0.68 (0.19)	25	1.1 (0.31)

*(previously known as *E. aerogenes*)
**Reported by Grassberger et al. (1984). Concentrations were given as µg/ml in the original publication.

Reference: Grassberger, M. A.; Turnowsky, F.; Hildebrandt, J. Preparation and antibacterial activities of new 1,2,3-diazaborine derivatives and analogues. J Med Chem 1984, 27 (8), 947-953. DOI: 10.1021/jm00374a003

Table S4. Antibacterial activity screening of selected compounds against a panel of *Staphylococci* strains. Single screening experiment was performed at 50 µM concentration in triplicate wells generally following the procedure described in the Materials and Methods (Antibacterial activity screening).

	Antibacterial activity at 50 µM (inhibition growth, % ±SD)				
**Diazaborine**	***S. aureus* ATCC25691**	***S. aureus* ATCC28763**	***S. aureus* ATCC43300**	***S. epidermidis* ATCC12228**	***S. epidermidis* ATCC35984**
**11**	17.5±4.3	10.3±2.7	23.2±1.7	4.1±10.2	25.1±2.6
**13**	18.4±3.1	11.6±3.1	20.1±3.7	6.5±13.5	31.2±14.2
**39**	57.0±0.8	61.5±2.7	70.3±12.0	47.8±5.8	-17.1±8.0
**40**	49.3±5.0	51.6±0.9	50.9±2.2	32.6±3.8	-20.0±4.3
**41**	53.8±5.6	31.1±8.1	44.9±10.4	11.2±8.2	-14.5±2.3
**43**	54.3±8.6	41.2±2.8	54.1±14.9	14.8±11.9	-9.5±3.5
**45**	54.0±6.7	30.5±2.7	47.6±27.0	11.8±5.7	-15.1±4.9
**46**	4.8±1.7	6.7±1.5	4.7±6.0	0.4±11.4	12.2±12.1
**47**	-1.8±2.5	4.6±4.0	2.1±3.7	-11.2±11.6	5.1±3.3
**49**	78.4±6.5	46.0±7.2	96.8±1.2	101.9±2.5	96.8±0.4

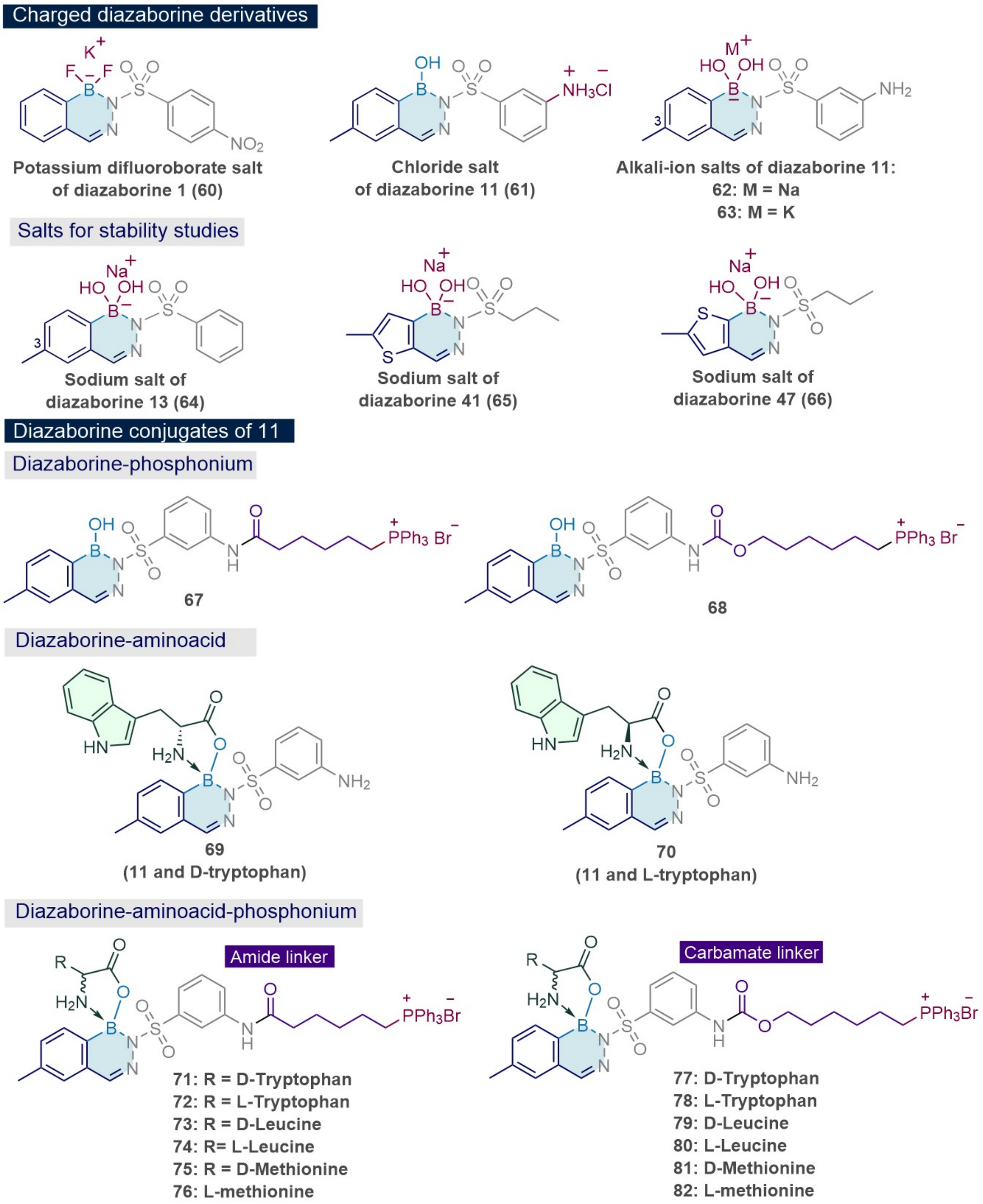

Figure S2. Charged diazaborines and conjugates.

Table S5. Antibacterial activity screening for charged diazaborines and conjugates shown in **Figure S2**. Screening performed in *E. coli* ATCC25922, *P. aeruginosa* ATCC27853, *S. aureus* ATCC29213, *E. faecalis* ATCC29212. Experimental procedure described in the Materials and Methods.

Compound	Description	MIC[a] (µM) or (maximum growth inhibition, % ± SD)			
		E. coli	*P. aeruginosa*	*S. aureus*	*E. faecalis*
**Reference diazaborines**					
**1**		>50 (37.9±2.3)	> 50 (-1.0±6.2)	>50 (13.6±10.7)	>50 (-7.4±1.0)
**11**		6.25	> 50 (4.2±2.2)	>50 (-9.1±8.9)	>50 (-5.8±2.0)
**Charged diazaborine derivatives**					
**60**	potass difluorb salt of **1**	>50 (34.7±1.9)	>50 (-1.8±7.6)	>50 (18.5±3.7)	>50 (-3.1±4.2)
**61**	chloride salt of **11**	6.25	>50 (13.1±3.8)	>50 (20.4±3.0)	>50 (2.06±4.0)
**62**	sodium salt of **11**	6.25	>50 (13.5±3.3)	>50 (8.3±6.0)	>50 (-1.7±4.7)
**63**	potassium salt of **11**	6.25	>50 (-7.7±7.7)	>50 (38.8±3.7)	>50 (3.1±5.0)
**Diazaborine conjugates of 11**					
**67**	phosphonium, amide linker	>50 (-15.5±8.0)	>50 (6.7±0.9)	>50 (3.9±7.0)	>50 (-6.3±0.0)
**68**	phosphonium, carb linker	>50 (-2.4±8.5)	>50 (16.1±4.5)	25	>50 (24.5±2.9)
**69**	D-trp	12.5	>50 (17.4±6.3)	>50 (14.0±4.9)	>50 (1.1±3.5)
**70**	L-trp	6.25	>50 (11.3±5.1)	>50 (7.7±7.2)	>50 (-0.1±1.4)
**71**	D-trp, phosph, amide linker	>50 (-1.0±7.1)	> 50 (18.7±7.8)	25	>50 (33.3±15.4)
**72**	L-trp, phosph, amide linker	>50 (-7.0±2.1)	> 50 (24.5±7.8)	25	>50 (32.0±11.8)
**73**	D-leu, phosph, amide linker	>50 (-21.1±2.3)	> 50 (-20.0±12.6)	50	>50 (4.4±4.7)
**74**	L-leu, phosph, amide linker	>50 (-22.4±1.9)	>50 (17.0±3.8)	50	>50 (14.3±2.9)
**75**	D-met, phosph, amide linker	>50 (-18.7±9.4)	>50 (8.6±3.0)	>50 (7.8±2.7)	>50 (-7.3±4.8)
**76**	L-met, phosph, amide linker	>50 (-11.4±11.5)	>50 (11.6±3.7)	>50 (31.3±7.5)	>50 (-1.0±14.8)
**77**	D-trp, phosph, carb linker	> 50 (-5.0±1.5)	>50 (-3.4±4.8)	6.25	>50 (78.2±4.8)
**78**	L-trp, phosph, carb linker	> 50 (-5.1±3.1)	>50 (24.7±7.6)	6.25	> 50 (83.7±6.9)
**79**	D-leu, phosph, carb linker	> 50 (-4.8±6.1)	>50 (24.1±4.6)	12.5	> 50 (56.3±17.1)
**80**	L-leu, phosph, carb linker	> 50 (-10.5±10.0)	>50 (32.1±2.7)	12.5	> 50 (56.1±18.4)
**81**	D-met, phosph, carb linker	> 50 (-2.9±8.5)	>50 (20.7±9.3)	25	> 50 (21.4±2.0)
**82**	L-met, phosph, carb linker	> 50 (-0.8±7.8)	>50 (-8.5±1.6)	25	> 50 (31.3±4.9)

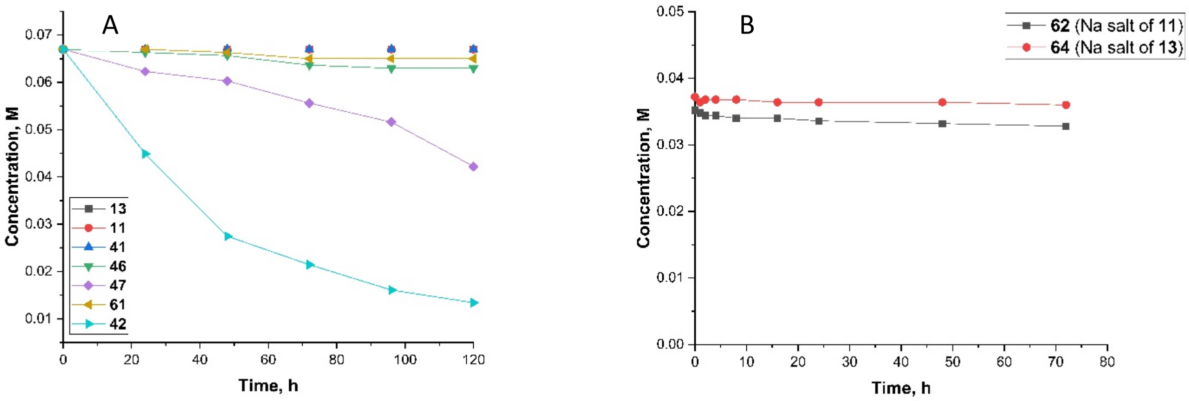

Figure S3. Stability of selected diazaborines in DMSO and freeze-thawing tolerance.
(A) DMSO diazaborine solutions were subjected to repeated freeze-thawing (1 cycle per day) over the period of 5 days. **(B)** Decomposition rate of sodium salts of diazaborines **11** and **13** (Figure 1) in DMSO at 37°C. Degree of decomposition was measured by calculating the concentration from NMR quantification as described in the Materials and Methods.

Table S6. Testing of diazaborine **11** for synergistic activity against *E. coli* ATCC25922. Experimental procedure described in the Materials and Methods.

Compound	Class/Application	MIC (µM)	Concentration range tested (µM)	FICI*	Antibacterial effect
**Metronidazole**	Nitroimidazole derivative/antibacterial	>250	62.5 - 1000	>1	Indifference
**Sulfadiazine**	Sulfonamide/antibacterial	125	7.81 - 125	0.75	Additive
**Meropenem**	Carbapenem/antibacterial	0.20	0.05 - 25	0.75	Additive
**Amoxicillin**	Aminopenicillin/antibacterial	25 - 50	0.39 - 25	1	Indifference
**Ciprofloxacin**	Fluoroquinolone/antibacterial	0.05	0.01 - 0.78	0.83	Additive
**Metformin**	Biguanide/diabetes treatment	>1250	15.63 - 250	1	Indifference
**Citric acid**	Tricarboxylic acid/food preservative, insecticide	>1250	250 – 1250	> 0.75	Indifference
**Choline chloride**	Quaternary ammonium salt/food additive	>1250	250 – 1250	> 1.5	Indifference

*FICI was calculated for assays performed with the highest concentration.

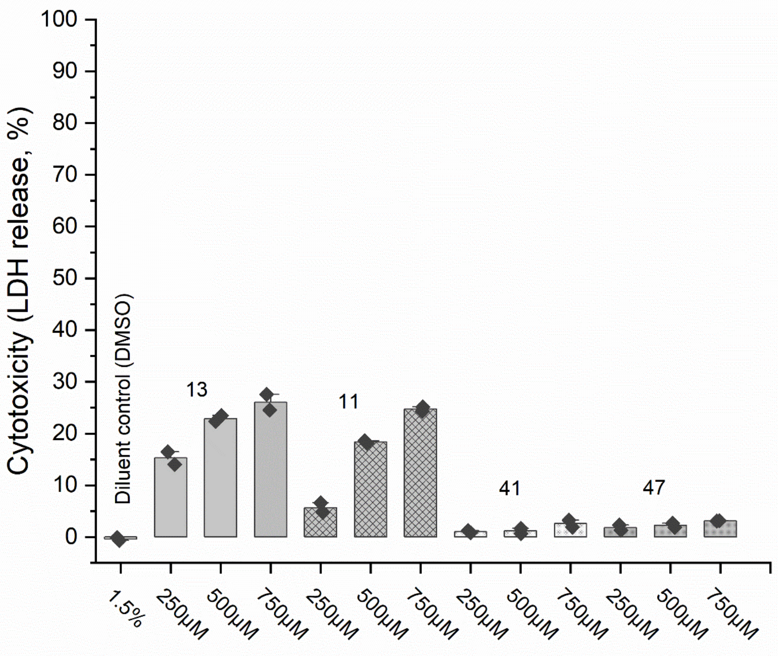

Figure S4. Mechanisms of cytotoxicity of diazaborines **11**, **13**, **41** and **47**.
Membrane-damaging activity of diazaborines in HepG2 hepatocarcinoma cell line after 24 hours incubation with the compounds measured by LDH release cytotoxicity assay. The percentages are relative to LDH release after lysis buffer (detergent) treatment (100% release). The bars represent median value of 2 independent experiments (performed in triplicate wells) ± SD. Experimental procedure described in Materials and Methods.

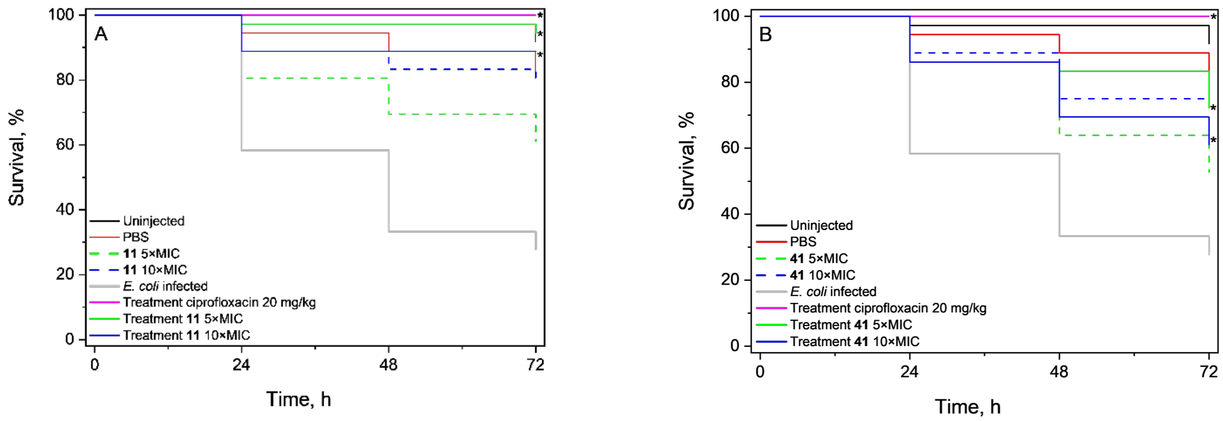

Figure S5. Survival of infected *Galleria mellonella* larvae after diazaborine treatment.

The larvae were infected with *E. coli* ATCC25922 (n=36) with (**A**) diazaborine **11** and (**B**) diazaborine **41**, up to 72 hours post-infection. Treatments were administered at 5×MIC and 10×MIC, 1 hour after inoculation. Ciprofloxacin (20 mg/kg) was used as positive control. *Asterisks indicate statistically significant treatments in comparison to infected, non-treated group ($p<0.05$). Experiments were performed three times with n=12 per group. Experimental procedure described in the Materials and Methods.

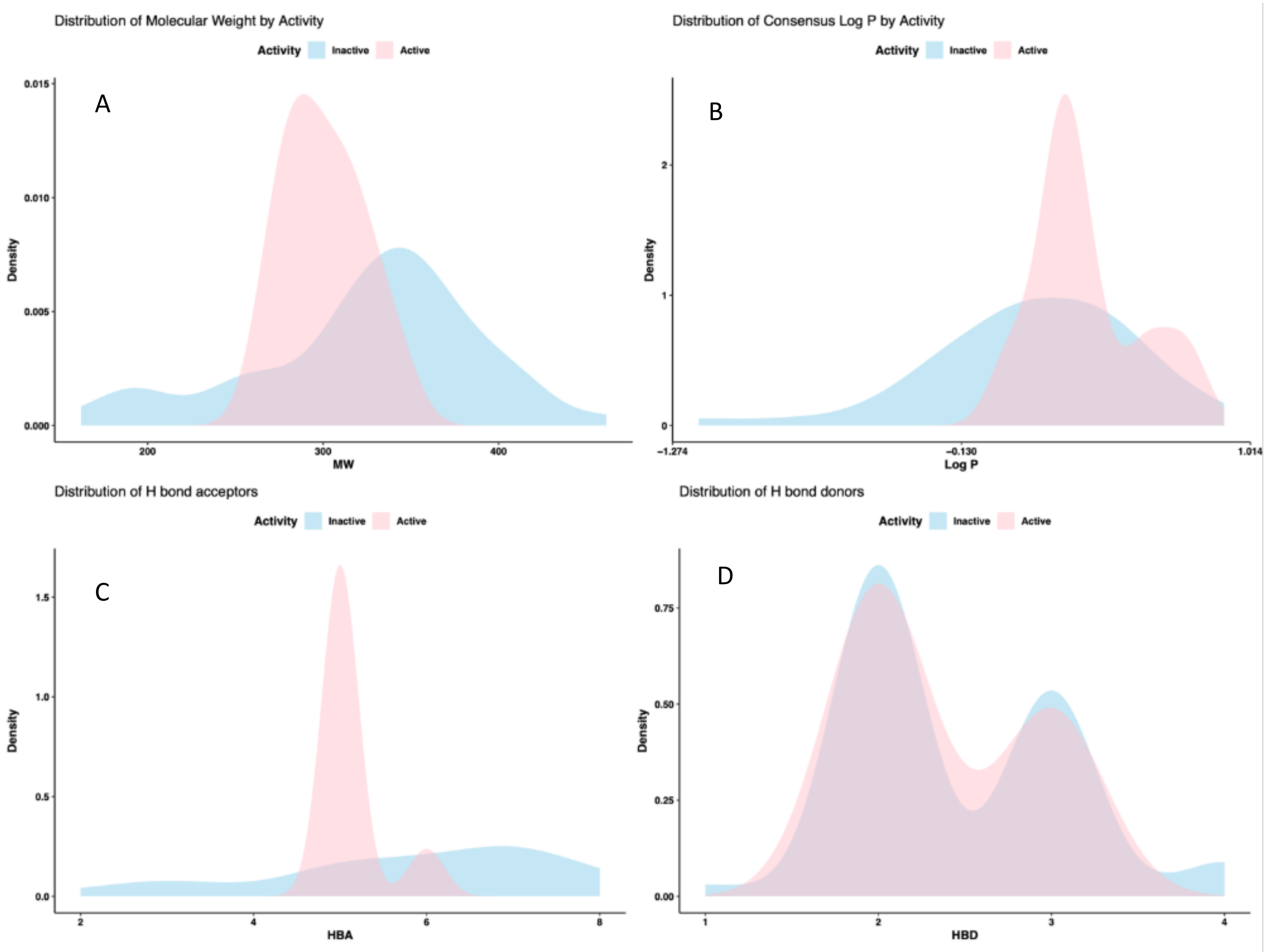

Figure S6. Physicochemical property distributions of active vs. non-selected compounds. Comparative density distributions of **(A)** molecular weight, **(B)** Log P, **(C)** H-Bond acceptors (HBA), and **(D)** H-Bond donors (HBD). Probability curves contrasting selected active compounds (diazaborine **11**, **13**, **41**, **42**, **46**, **47**, **61**) versus non-selected compounds.

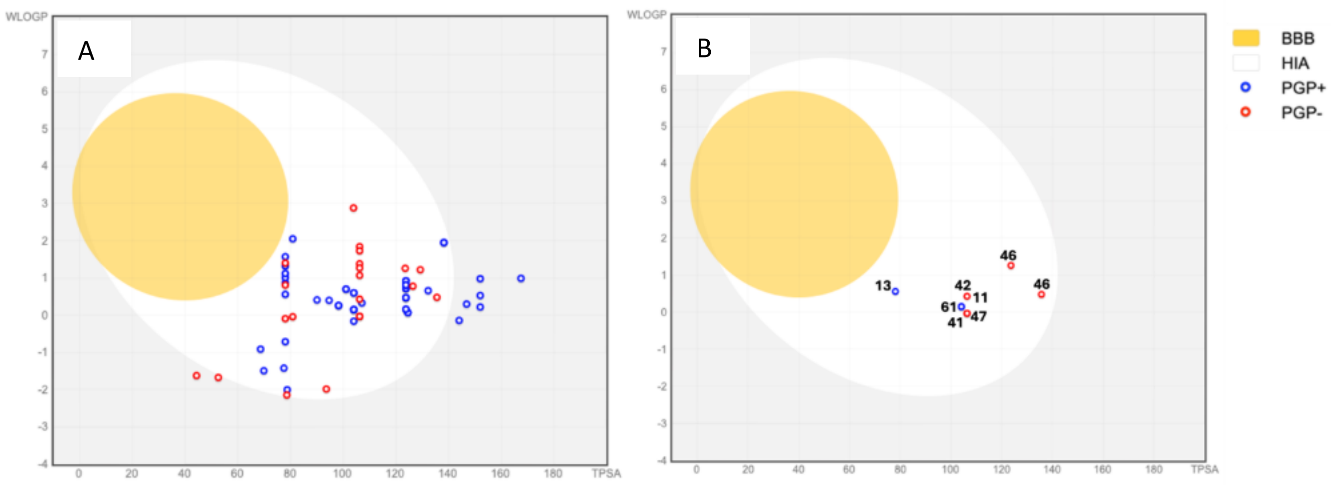

Figure S7. Comprehensive BOILED-egg diagram analysis: contrasting global dataset with selected active compounds for gastrointestinal absorption and blood- brain barrier permeation potential.
(A) full dataset overview. **(B)** focused examination of selected active diazaborines **11**, **13**, **41**, **42**, **46**, **47**, and **61** -a visual guide to oral bioavailability and central nervous system penetration likelihood. The BOILED-Egg model evaluates passive gastrointestinal absorption (HIA) and brain penetration (BBB) based on WLOGP versus TPSA values. Compounds in the white region show high HIA probability, while those in the yellow region (yolk) indicate high BBB penetration potential. Markers are colored blue for P-gp substrates (PGP+) and red for non-substrates (PGP).

Detailed synthesis procedures of diazaborine compounds

Unless stated otherwise, all the synthetic manipulations were conducted under an argon atmosphere either in a glovebox or using conventional Schlenk techniques on a dual-manifold gas-inlet/vacuum line. All glassware was flame-dried prior to use. HPLC-quality grade reaction solvents were dried by conventional methods, thoroughly degassed with three freeze-pump-thaw cycles and stored over flame-activated 3Å and 4Å molecular sieves on the glovebox. The solvents required to conduct the rest of synthetic operations were purchased at HPLC-quality grade and used as received. Deuterated solvents were purchased at the highest purity level and used without further purification. Quantitative flash column chromatography was carried out using silica gel (Merck Silica Gel 60 Å, 230 X 400 mesh), or deactivated silica gel (packed into the column with the solvent system of choice containing 5% in Et_3N, and subsequently washed twice with the same solvent system free of Et_3N). The eluent is quoted in volume ratios (v1:v2). All nuclear magnetic resonance (NMR) experiments (^1H, ^{13}C, ^{19}F, ^{11}B) were performed on Bruker Avance NEO spectrometers operating with the frequency, deuterated solvent and at the temperature indicated in parentheses. All chemical shifts values (δ) are reported in parts per million (ppm) downfield in relation to tetramethylsilane using the residual undeuterated solvent signal as secondary internal standard ($CHCl_3$ in $CDCl_3$; δ_H = 7.26 ppm and δ_C = 77.16 ppm), ($CHDCl_2$ in CD_2Cl_2; δ_H = 5.32 ppm and δ_C = 53.8 ppm), (C_6HD_5 in C_6D_6; δ_H = 7.16 ppm and δ_C = 128.1 ppm).[S4] The resonances on the ^{19}F-NMR spectra are reported in parts per million (ppm) downfield of $CFCl_3$, using hexafluorobenzene (C_6F_6 at –164.9 ppm) as internal standard. Unless otherwise noted, all coupling constants (J) are quoted to the nearest 0.1 Hz with the involved nuclei subscripted, and the resonances are noted as follows: ^1H: δ chemical shift in ppm (number of protons, multiplicity, J value(s), assignment). ^{13}C: δ chemical shift in ppm (multiplicity [if applicable], J value(s) [if applicable], assignment). ^{19}F: δ chemical shift in ppm (number of fluorines, multiplicity, J value(s), assignment). Splitting patterns are denoted as s (singlet), d (doublet), t (triplet), q (quartet) in ^1H-NMR or quaternary in ^{13}C-NMR, p (pentet), s (sext), m (multiplet), dd (doublet of doublets), ddd (doublet of doublet of doublets), dt (doublet of triplets), td (triplet of doublets), br (broad resonance), app (apparent). Unless otherwise stated, both the resonances on the ^{13}C and ^{19}F-NMR spectra were proton-decoupled. High-resolution electrospray-ionization mass spectra (ESI-MS) were recorded on a Bruker microTOF mass spectrometer operated in a positive or negative ion mode, using a 0.05 M solution of sodium formiate as a calibrant. Stock solutions (400-1000 ppm) were prepared in methanol (99.9% VWR Chemicals, France) or acetonitrile (99.9% Honeywell, Riedel-de Haën) and were diluted (3 ppm) with either 0,05% FA/MeOH solution 70/30 (v/v%) or acetonitrile prior to the measurement. Only the prevalent ion peak ($HCOO^-$, H^+ or Na^+ adduct) is given for each compound. –Elemental Analysis was performed with an Automatic elemental analyzer vario MICRO cube (HANAU Elementar Analysensysteme GmbH, Germany). The sample was weighted for three repetitions about 1,800 mg into tin boats. The packing material was handled with tweezers. Boats were closed gas-tightly. Foiled samples were compressed to remove air. Before the sample analysis, the instrument was stabilized. The purity of the compounds is ≥95%, which was quantitatively determined by ^1H or ^{19}F NMR spectroscopy using relaxation delay values "d1" five times the typical T1 values for the slowest relaxing signals in medium-sized molecules according to Claridge,[S1] that is d1=30s (for ^1H NMR quantification), and d1=30s (for ^{19}F NMR quantification).

General Method A for the syntheses of diazaborines: Unless stated otherwise, a $EtOH:H_2O$ solution (1:1, 0.2M) of the corresponding hydrazide (1 equiv) was slowly added to an $EtOH:H_2O$ solution (1:1, 0.2M) of the

corresponding o-formylphenylboronic acid (1 equiv), and the resulting reaction mixture was vigorously stirred for 1-3 h at room temperature under argon. Then, a precipitate was formed in the solution, which was subsequently filtered rinsing with water. The resulting solid was dried under high vacuum to afford the corresponding diazaborine with a purity amenable for biological evaluation (≥95%).

General Method B for the syntheses of diazaborines: Unless stated otherwise, the corresponding hydrazone (1 equiv) and anhydrous iron (III) chloride (0.07 equiv) were dissolved in anhydrous 1,2-dichloroethane (final concentration 0.05 M) in a three-neck round-bottom flask equipped with septum, reflux condenser and magnetic stirrer under argon. Then, boron tribromide (3.06 equiv) was slowly added through the septum which was replaced by a glass stopper before refluxing the solution for 1-2 h at 70-80 °C. Then the reaction was worked up *via* the following two procedures:

Work-up 1: The reaction mixture was cooled down to room temperature and slowly quenched with water upon stirring. ***Caution***: ***vigorous evolution of gas occurs at the beginning of the quenching***. Then, the organic phase was washed with three portions of water which were discarded. The organic phase was then extracted three times with a 1M aqueous solution of NaOH. The aqueous layers were combined and slowly acidified with a 1M HCl solution until pH~2. The resulting water solution was extracted three times with dichloromethane. These organic fractions were combined, washed with brine, dried over Na_2SO_4, filtered, evaporated and dried under high vacuum to afford the corresponding diazaborine with a purity amenable for biological evaluation (≥95%).

Work-up 2: The reaction mixture was cooled down to room temperature and slowly quenched with water upon stirring. ***Caution***: ***vigorous evolution of gas occurs at the beginning of the quenching***. Then, the organic phase was washed with three portions of water which were discarded. The remaining organic phase was extracted three times with a 1M aqueous solution of NaOH. The aqueous phases were combined and slowly acidified with a 1M HCl solution until pH~2. The resulting water solution was extracted three times with ethyl acetate. The resulting combined organic extracts were washed with brine, dried over Na_2SO_4, filtered and evaporated. The remaining solid residue was suspended in 30 ml of diethyl ether, shaken, filtered and washed additionally with 15 ml of ether, and finally dried under high vacuum to afford the corresponding diazaborine with a purity amenable for biological evaluation (≥95%).

General Method C for the syntheses of diazaborines: Unless stated otherwise, the Bpin derivative of the corresponding o-formylphenylboronic acid (1 equiv) was dissolved in a mixture of CH_3OH:H_2O (1:1, 0.1 M) and argon was bubbled through the solution, before adding the corresponding hydrazide (1 equiv) and stirring the resulting solution for 2h at 80 °C. Then, a precipitate was formed in the solution, which was subsequently filtered rinsing with water. The resulting solid was dried under high vacuum to afford the corresponding diazaborine with a purity amenable for biological evaluation (≥95%).

General Method D for the synthesis of diazaborines by reduction of aromatic nitro groups: Unless stated otherwise, the corresponding diazaborine bearing an aromatic nitro group and ammonium chloride (15 equiv) were suspended in a H_2O:MeOH mixture (1:1, 0.1 M). Then, iron turnings (10 equiv) were added portionwise to the solution, which was subsequently stirred at 70 C for 2 h. Then, the mixture was cooled down and the liquid phase was transferred to a separating funnel where it was extracted with $CHCl_3$.The resulting organic layer was washed several times with a 0.5 M aqueous solution of HCl until the coloration of the water phase disappeared. Subsequently, the resulting organic phase was successively washed with water until the water layer reached neutral pH. Then, the organic later was washed with brine, dried over Na_2SO_4, evaporated and dried under high vacuum to afford the corresponding diazaborine with a purity amenable for biological evaluation (≥95%).

2-((4-Nitrophenyl)sulfonyl)benzo[*d*][1,2,3]diazaborinin-1(2*H*)-ol (diazaborine 1) was obtained according to the general method A by reacting 2-formylphenylboronic acid (5.0 g, 33 mmol, 1 equiv) and hydrazide **S4** (7.1 g, 33 mmol, 1 equiv) in an EtOH:H_2O solution (1:1, 240 mL) for 90 min at room temperature. Obtained 10.51 g (97% yield) of the title compound an off-white solid. **^{1}H NMR (600 MHz, $CDCl_3$)** δ 8.40-8.37 (2H, m, 2x Ar***H***), 8.27-8.24 (2H, m, 2x Ar***H***), 8.23 (1H, ddt, *J*=7.5, 1.5, 0.7 Hz, Ar***H***), 8.03 (1H, d, *J*=0.7 Hz,

H*C=N), 7.76 (1H, td, *J*=7.5, 1.3 Hz, ArH***), 7.69 (1H, td, *J*=7.7, 1.3 Hz, Ar***H***), 7.59 (1H, dt, *J*=7.7, 1.0 Hz, Ar***H***), 7.27 (1H, s, B-O***H***); **^{13}C NMR (151 MHz, $CDCl_3$, 298 K)** δ 151.0 (Ar***C***-NO_2), 144.8 (***C***=N-N), 143.4 (Ar***C***), 134.0 (Ar***C***), 133.4 (Ar***C***H), 132.5 (Ar***C***H), 131.4 (Ar***C***H), 130.0 (2x Ar***C***H), 128.1 (Ar***C***H), 124.4 (2x Ar***C***H). *Note*: *The resonance corresponding to the quaternary carbon directly bonded to the B-OH group was not observed due to signal broadening caused by the fast relaxation induced by the quadrupolar boron nucleus as well as possible residual J-coupling to boron*; **^{11}B NMR (128 MHz, $CDCl_3$)** δ 28.6; **HRMS (ESI)**: m/z calcd. for $C_{13}H_{10}BN_3O_5SNa$ [M+Na]$^+$: 354.0329, found 354.0324, error 0.769 ppm.

2-((3-Nitrophenyl)sulfonyl)benzo[*d*][1,2,3]diazaborinin-1(2*H*)-ol (diazaborine 2) was synthesized according to general method B and work-up 1 by refluxing for 1 h hydrazone **S15** (1.0 g, 3.3 mmol, 1 equiv), boron tribromide (2.5 g, 10.0 mmol, 3.06 equiv), and anhydrous iron (III) chloride (39 mg, 0.24 mmol, 0.07 equiv) in 70 ml of DCE. Obtained 964 mg (89% yield) of the title compound as a white solid. **^{1}H NMR (600 MHz, $CDCl_3$, 298 K)** δ 8.90 (1H, t, *J*=2.0 Hz, Ar***H***), 8.49 (1H, ddd, *J*=8.2, 2.3, 1.1 Hz, Ar***H***), 8.39 (1H, ddd, *J*=7.9, 1.8, 1.0 Hz, Ar***H***), 8.24 (1H, app. d, *J* =7.4 Hz, Ar***H***), 8.04 (1H, s, ***H***C=N), 7.78 (1H, t, *J*=8.1 Hz, Ar***H***), 7.75 (1H, td, *J*=7.5, 1.6 Hz, Ar***H***), 7.69 (1H, td, *J*=7.4, 1.2 Hz, Ar***H***), 7.59 (1H, dd, *J*=7.7, 1.0 Hz, Ar***H***) , 7.27 (1H, s, B-O***H***); **^{13}C NMR (126 MHz, $CDCl_3$)** δ 148.4 (Ar***C***-NO_2), 144.8 (***C***=N-N), 139.9 (Ar***C***), 134.1 (Ar***C***H), 134.0 (Ar***C***), 133.3 (Ar***C***H), 132.5 (Ar***C***H), 131.4 (Ar***C***H), 130.6 (Ar***C***H), 128.5 (Ar***C***H), 128.0 (Ar***C***H), 124.0 (Ar***C***H). *Note*: *The resonance corresponding to the quaternary carbon directly bonded to the B-OH group was not observed due to signal broadening caused by the fast relaxation induced by the quadrupolar boron nucleus as well as possible residual J-coupling to boron*; **^{11}B NMR (160 MHz, $CDCl_3$)** δ 28.6; **HRMS (ESI)**: m/z calcd. for $C_{13}H_{10}BN_3O_5SNa$ [M+Na]$^+$: 354.0329, found 354.0326, error 0.123 ppm.

2-((4-Aminophenyl)sulfonyl)benzo[*d*][1,2,3]diazaborinin-1(2*H*)-ol (diazaborine 3). An acetic acid solution (60 ml) of diazaborine **1** (1.0 g, 3.02 mmol, 1 equiv) was heated up to 80 °C, then iron turnings (1.81 g, 32.4 mmol, 10.7 equiv) were added portionwise and the mixture was stirred for 30 min. Subsequently, the reaction mixture was cooled down to room temperature, the inorganic solids were filtered off and the filtrate was evaporated. The remaining solid was redissolved in dichloromethane and the solution was filtered through a plug of silica rinsing with ethyl acetate until not product was detected on TLC. The final solution was evaporated to afford the title compound (906 mg, 99% yield) as a yellowish solid. **^{1}H NMR (500 MHz, DMSO-*d*$_6$, 298 K)** δ 8.50 (1H, s, B-O***H***), 8.19-8.13 (2H, m, Ar***H*** and ***H***C=N), 7.82-7.72 (2H, m, 2x Ar***H***), 7.69 (1H, td, *J*=7.2, 1.5 Hz, Ar***H***), 7.66-7.59 (2H, m, 2x Ar***H***), 6.66-6.59 (2H, m, 2x Ar***H***), 6.24 (2H, br. s, ArN***H***$_2$); **^{13}C NMR (126 MHz, DMSO-*d*$_6$, 298 K)** δ 154.0 (Ar***C***-NH_2), 141.9 (***C***=N-N), 134.1 (Ar***C***), 132.7 (Ar***C***H), 131.6 (Ar***C***H), 130.5 (Ar***C***H), 130.1 (2x (Ar***C***H)), 129.8 (br., Ar***C***-B(OH)), 127.8 (Ar***C***H), 122.3 (Ar***C***), 112.5 (2x Ar***C***H); **^{11}B NMR (160 MHz, DMSO-*d*$_6$, 298 K)** δ 28.9 (br.); **HRMS (ESI)**: m/z calcd. for $C_{13}H_{13}BN_3O_3S$ [M+H]$^+$: 302.0768, found 302.0762, error 1.019 ppm.

2-((4-Fluorophenyl)sulfonyl)benzo[*d*][1,2,3]diazaborinin-1(2*H*)-ol (diazaborine 4) was prepared according to the general method A by reacting 2-formylphenylboronic acid (300 mg, 1.96 mmol, 1 equiv) and 4-fluorophenylsulfonyl hydrazide **S5** (373 mg, 1.96 mmol, 1 equiv) in an EtOH:H_2O solution (1:1, 20 mL) for 2 h at room temperature. Obtained 407 mg (68% yield) of the title compound as a white solid. **^{1}H NMR (500 MHz, $CDCl_3$, 298 K)** δ 8.22 (1H, app. d, *J*=7.5 Hz, Ar***H***), 8.11-8.04 (2H, m, 2x Ar***H***), 8.03 (1H, s, ***H***C=N), 7.73 (1H, td, *J*=7.5, 1.4 Hz, Ar***H***), 7.67 (1H, td, *J*=7.5 Hz, 1.2 Hz, Ar***H***), 7.58 (1H, app. d, *J*=7.7 Hz, Ar***H***), 7.35 (1H, s, B-O***H***), 7.25-7.18 (2H, m, 2x Ar***H***); **^{13}C NMR (126 MHz, $CDCl_3$, 298 K)** δ 166.1 (Ar***C***-F, d, *J*=257.1 Hz), 144.1 (***C***=N-N), 134.2 (Ar***C***), 134.0 (Ar***C***, d, *J*=3.3 Hz), 133.1 (Ar***C***H), 132.3 (Ar***C***H), 131.5 (2x Ar***C***H, d, *J*=9.7 Hz), 131.1 (Ar***C***H), 129.8 (br., Ar***C***-B(OH)), 127.9 (Ar***C***H), 116.6 (2x Ar***C***H, d, *J*=22.8 Hz); **^{19}F NMR (377 MHz, $CDCl_3$, 298 K)** δ -102.6 (tt, *J* = 8.1, 5.0 Hz); **^{11}B NMR (160 MHz, $CDCl_3$, 298 K)** δ 28.5; **HRMS (ESI)**: m/z calcd. for $C_{13}H_9BFN_2O_3S$ [M-H]$^-$: 303.0417, found 303.0415, error 0.424 ppm.

2-(Cyclopropylsulfonyl)benzo[*d*][1,2,3]diazaborinin-1(2*H*)-ol (diazaborine 5) was synthesized according to the general method A by reacting 2-formylphenylboronic acid (150 mg, 0.98 mmol, 1 equiv) and crude cyclopropylsulfonyl hydrazide **S10** (purity 80%, 167 mg, 0.98 mmol, 1 equiv) in an EtOH:H_2O solution (1:1, 10 mL) during 16 h at room temperature. Obtained 200 mg (81% yield) of the title compound as a white solid. **^{1}H NMR (500 MHz, $CDCl_3$, 298 K)** δ 8.21 (1H, d, *J*=7.7 Hz, Ar***H***), 8.15 (1H, s, ***H***C=N), 7.77 (1H, td, *J*=7.5,

1.4 Hz, Ar***H***), 7.72-7.63 (2H, m, Ar***H***), 7.09 (1H, s, B-O***H***), 2.98 (1H, tt, *J*=8.0, 4.8 Hz, C***H***), 1.48-1.41 (2H, m, C***H₂***), 1.19-1.11 (2H, m, C***H₂***); **^{13}C NMR (126 MHz, $CDCl_3$, 298 K)** δ 143.8 (***C***=N-N), 134.4 (Ar***C***), 133.0 (Ar***C***H), 132.3 (Ar***C***H), 131.0 (Ar***C***H), 127.8 (Ar***C***H), 30.6 (***C***H), 6.6 (2x ***C***H_2). *Note*: *The resonance corresponding to the quaternary carbon directly bonded to the B-OH group was not observed due to signal broadening caused by the fast relaxation induced by the quadrupolar boron nucleus as well as possible residual J-coupling to boron*; **^{11}B (160 MHz, $CDCl_3$, 298 K)** δ 28.3; **HRMS (ESI)**: m/z calcd. for $C_{12}H_{16}BN_2O_4S$ $[M+OC_2H_5]^-$: 295.0930, found 295.0929, error 0.043 ppm.

2-((3,3,3-Trifluoropropyl)sulfonyl)benzo[*d*][1,2,3]diazaborinin-1(2*H*)-ol (diazaborine 6) was prepared according to the general method A by reacting 2-formylphenylboronic acid (127 mg, 0.83 mmol, 1 equiv) and 2,2,2-trifluoropropylsulfonyl hydrazide **S7** (199 mg, 0.83 mmol, 1 equiv) in an EtOH:H_2O solution (1:1, 10 mL) for 16 h at 50 °C. Obtained 175 mg (69% yield) of the title compound as a white solid. **^{1}H NMR (500 MHz, $CDCl_3$, 298 K)** δ 8.22 (1H, d, *J*=7.5 Hz, Ar***H***), 8.16 (1H, s, ***H***C=N), 7.80 (1H, td, *J*=7.5, 1.4 Hz, Ar***H***), 7.72 (1H, td, *J*=7.5, 1.2 Hz, Ar***H***), 7.68 (1H, d, *J*=7.7 Hz, Ar***H***), 7.05 (1H, s, B-O***H***), 3.78-3.70 (2H, m, CF_3C***H₂***-CH_2), 2.73-2.60 (2H, m, CF_3CH_2-C***H₂***); **^{13}C NMR (126 MHz, $CDCl_3$, 298 K)** δ 144.8 (***C***=N-N), 134.1 (Ar***C***), 133.4 (ArC***H***), 132.5 (Ar***C***H), 131.5 (Ar***C***H), 129.8 (br., Ar***C***-B(OH)), 128.2 (Ar***C***H), 125.3 (***C***F_3-CH_2, q, *J*=276.6 Hz), 45.1 (CF_3CH_2***C***H_2, q, *J*=3.2 Hz), 28.9 (CF_3***C***H_2CH_2., q, *J*=31.6 Hz); **^{19}F NMR (470 MHz, $CDCl_3$, 298 K)** δ – 65.9 (t, *J*=9.9 Hz); **^{11}B NMR (160 MHz, $CDCl_3$, 298 K)** δ 28.5; **HRMS (ESI)**: m/z calcd. for $C_{12}H_{15}BF_3N_2O_4S$ $[M+OC_2H_5]^-$: 351.0804, found 351.0806, error 0.912 ppm.

5-Methyl-2-((4-nitrophenyl)sulfonyl)benzo[*d*][1,2,3]diazaborinin-1(2*H*)-ol (diazaborine 7) was was synthesized according to the general method B and work-up 2 by refluxing for 2 h hydrazone **S16** (1.5 g, 4.70 mmol, 1 equiv), boron tribromide (3.6 g, 14.4 mmol, 3.06 equiv), and anhydrous iron (III) chloride (56 mg, 0.34 mmol, 0.07 equiv) in 100 ml of DCE. Obtained 1.24 g (77% yield) of the title compound as a yellowish solid. **^{1}H NMR (600 MHz, DMSO-*d₆*, 300 K)** δ 9.06 (1H, br. s, B-O***H***), 8.46-8.39 (2H, m, 2x Ar***H***), 8.32 (1H, s, ***H***C=N), 8.29-8.23 (2H, m, 2x Ar***H***), 8.04 (1H, m, Ar***H***), 7.58-7.52 (2H, m, 2x Ar***H***), 2.54 (3H, s, ArC***H₃***); **^{13}C NMR(151 MHz, DMSO-*d₆*, 300 K)** δ 150.2 (Ar***C***-NO_2), 144.3 (Ar***C***), 140.0 (***C***=N-N), 136.0 (Ar***C***-CH_3), 134.0 (Ar***C***H), 131.7 (Ar***C***), 130.8 (br., Ar***C***-B(OH)), 130.5 (Ar***C***H), 129.6 (Ar***C***H), 129.4 (2x Ar***C***H), 124.5 (2x Ar***C***H), 18.0 (ArC-***C***H_3); **^{11}B NMR (128 MHz, DMSO-*d₆*, 298 K)** δ 25.1; **HRMS (ESI)**: m/z calcd. for $C_{14}H_{13}BN_3O_6S$ $[M+OH]^-$: 362.0626, found 362.0623, error 0.199 ppm.

6-Methyl-2-((4-nitrophenyl)sulfonyl)benzo[*d*][1,2,3]diazaborinin-1(2*H*)-ol (diazaborine 8) was obtained according to the general method B work-up 1 by refluxing hydrazone **S17** (1.0 g, 3.13 mmol, 1 equiv), boron tribromide (2.4 g, 9.58 mmol, 3.06 equiv), and anhydrous iron (III) chloride (37 mg, 0.23 mmol, 0.07 equiv) in 65 ml of DCE for 30 min at 70 °C. Obtained 0.93 g (86% yield) of the title compound as a yellowish solid. **^{1}H NMR (500 MHz, $CDCl_3$, 298 K)** δ 8.41-8.35 (2H, m, 2x Ar***H***), 8.27-8.22 (2H, m, 2x Ar***H***), 8.11 (1H, d, *J*=7.7 Hz, Ar***H***), 7.97 (1H, s, ***H***C=N), 7.51 (1H, ddd, *J*=7.7, 1.6, 0.7 Hz, Ar***H***), 7.39 (1H, app. dt, *J*=1.5, 0.7 Hz, Ar***H***), 7.21 (1H, s, B-(O***H***)), 2.49 (3H, s, ArC***H₃***); **^{13}C NMR (126 MHz, $CDCl_3$, 298 K)** δ 150.9 (Ar***C***-NO_2), 144.9 (***C***=N-N), 144.1 (Ar***C***-CH_3), 143.5 (Ar***C***), 134.3 (Ar***C***), 132.7 (Ar***C***H), 132.4 (Ar***C***H), 130.0 (2x Ar***C***H), 128.2 (Ar***C***H), 124.4 (2x Ar***C***H), 22.0 (ArC-***C***H_3). *Note*: *The resonance corresponding to the quaternary carbon directly bonded to the B-OH group was not observed due to signal broadening caused by the fast relaxation induced by the quadrupolar boron nucleus as well as possible residual J-coupling to boron*; **^{11}B (160 MHz, $CDCl_3$, 298 K)** δ 29.0; **HRMS (ESI)**: m/z calcd. for $C_{14}H_{12}BN_3O_5SNa$ $[M+Na]^+$: 368.0486, found 368.0484, error 0.268 ppm.

2-((4-Aminophenyl)sulfonyl)-6-methylbenzo[*d*][1,2,3]diazaborinin-1(2*H*)-ol (diazaborine 9) was prepared adapting the general method D as follows: The diazaborine **8** (500 mg, 1.45 mol, 1 equiv) and ammonium chloride (1.16 g, 21.7 mmol, 15 equiv) were suspended in a H_2O:MeOH mixture (1:1, 20 ml). Then, iron turnings (809 mg, 14.5 mmol, 10 equiv) were added portionwise to the solution, which was subsequently stirred for 2 h at 70 C. Then, the mixture was cooled down and the liquid phase was transferred to a separating funnel where it was extracted with $CHCl_3$.The resulting organic layer was washed with brine, dried over Na_2SO_4, filtered, evaporated, redissolved in dichloromethane, and plugged through silica using ethyl acetate as eluent. Obtained 0.40 g (87% yield) of the title compound as a yellow solid. **^{1}H NMR (500 MHz,**

$CDCl_3$, 298 K) δ 8.09 (1H, d, *J*=7.7 Hz, Ar***H***), 7.96 (1H, s, ***H***C=N), 7.82-7.76 (2H, m, 2x Ar***H***), 7.47 (1H, app. d, *J*=7.8 Hz, Ar***H***), 7.42 (1H, s, B-(O***H***)), 7.36 (1H, s, Ar***H***), 6.69-6.64 (2H, m, 2x Ar***H***), 4.21 (2H, s, ArN$\boldsymbol{H_2}$), 2.48 (3H, s, ArC$\boldsymbol{H_3}$); **^{13}C NMR (126 MHz, $CDCl_3$, 298 K)** δ 151.7 (Ar***C***-NH_2), 143.5 (***C***=N-N), 143.3 (Ar***C***-CH_3), 134.7 (Ar***C***), 132.2 (Ar***C***H), 132.1 (Ar***C***H), 130.8 (2x Ar***C***H), 127.8 (Ar***C***H), 125.9 (Ar***C***), 114.0 (2x Ar***C***H), 22.0 (ArC-***C***H_3). *Note*: *The resonance corresponding to the quaternary carbon directly bonded to the B-OH group was not observed due to signal broadening caused by the fast relaxation induced by the quadrupolar boron nucleus as well as possible residual J-coupling to boron*; **^{11}B NMR (160 MHz, $CDCl_3$, 298 K)** δ 28.6; **HRMS (ESI)**: m/z calcd. for $C_{14}H_{14}BN_3O_3SNa$ $[M+Na]^+$: 338.0741, found 338.0734, error 1.412 ppm.

6-Methyl-2-((3-nitrophenyl)sulfonyl)benzo[*d*][1,2,3]diazaborinin-1(2*H*)-ol (diazaborine 10) was obtained through the general method B work-up 2 by refluxing for 2 h at 70 °C hydrazone **S18** (3.0 g, 9.39 mmol, 1 equiv), boron tribromide (7.2 g, 28.7 mmol, 3.06 equiv), and anhydrous iron (III) chloride (112 mg, 0.69 mmol, 0.07 equiv) in 200 ml of DCE. Obtained 2.87 g (88% yield) of the title compound as a yellowish solid. **^{1}H NMR (500 MHz, $CDCl_3$, 298 K)** δ 8.88 (1H, t, *J*=2.0 Hz, Ar***H***), 8.48 (1H, ddd, *J*=8.3, 2.2, 1.1 Hz, Ar***H***), 8.38 (1H, dt, *J*=7.9, 1.4 Hz, Ar***H***), 8.10 (1H, d, *J*=7.7 Hz, Ar***H***), 7.98 (1H, s, ***H***C=N), 7.78 (1H, t, *J*=8.1 Hz, Ar***H***), 7.50 (1H, dd, *J*=7.7, 1.6 Hz, Ar***H***), 7.38 (1H, s, Ar***H***), 7.20 (1H, s, B-(O***H***)), 2.48 (3H, s, ArC$\boldsymbol{H_3}$); **^{13}C NMR (126 MHz, $CDCl_3$, 298 K)** δ 148.3 (Ar***C***-NO_2), 144.8 (***C***=N-N), 144.0 (Ar***C***-CH_3), 140.0 (Ar***C***), 134.3 (Ar***C***), 134.1 (Ar***C***H), 132.7 (Ar***C***H), 132.4 (Ar***C***H), 130.6 (Ar***C***H), 126.8 (br., Ar***C***-B(OH)), 128.5 (Ar***C***H), 128.2 (Ar***C***H), 123.9 (Ar***C***H), 22.0 (ArC-***C***H_3); **^{11}B NMR (160 MHz, $CDCl_3$, 298 K)** δ 28.7; **HRMS (ESI)**: m/z calcd. for $C_{28}H_{24}B_2N_6O_{10}S_2Na$ $[2M+Na]^+$: 713.1080, found 713.1084, error 0.890 ppm.

2-((3-Aminophenyl)sulfonyl)-6-methylbenzo[*d*][1,2,3]diazaborinin-1(2*H*)-ol (diazaborine 11) was synthesized according to the general method D by refluxing diazaborine **10** (4.99 g, 14.4 mmol, 1 equiv), ammonium chloride (11.6 g, 217 mmol, 15 equiv) and iron turnings (8.07 g, 144 mmol, 10 equiv) in a H_2O:MeOH mixture (1:1, 105 ml) for 2 h at 70 C. Obtained 3.76 g (83% yield) of the title compound as a yellowish solid. **^{1}H NMR (600 MHz, $CDCl_3$, 300 K)** δ 8.10 (1H, d, *J*=7.7 Hz, Ar***H***), 7.98 (1H, s, ***H***C=N), 7.48 (1H, d, *J*=8.1 Hz, Ar***H***), 7.38-7.34 (3H, m, 2x Ar***H*** and B-O***H***), 7.30-7.27 (2H, m, 2x Ar***H***), 6.86 (1H, ddd, *J*=8.0, 2.4, 1.0 Hz, Ar***H***), 3.94 (2H, br. s, ArN$\boldsymbol{H_2}$), 2.48 (3H, s, ArC$\boldsymbol{H_3}$); **^{13}C NMR (151 MHz, $CDCl_3$, 300 K)** δ 147.2 (Ar***C***-NH_2), 144.0 (***C***=N-N), 143.6 (Ar***C***-CH_3), 138.9 (Ar***C***), 134.6 (Ar***C***), 132.3 (Ar***C***H), 132.3 (Ar***C***H), 130.2 (Ar***C***H), 128.0 (Ar***C***H), 120.2 (Ar***C***H), 117.8 (Ar***C***H), 113.9 (Ar***C***H), 22.0 (ArC-***C***H_3). *Note*: *The resonance corresponding to the quaternary carbon directly bonded to the B-OH group was not observed due to signal broadening caused by the fast relaxation induced by the quadrupolar boron nucleus as well as possible residual J-coupling to boron*; **^{11}B NMR (160 MHz, $CDCl_3$, 298 K)** δ 28.4; **HRMS (ESI)**: m/z calcd. for $C_{28}H_{28}B_2N_6O_6S_2Na$ $[2M+Na]^+$: 653.1600, found 653.1585, error 0.821 ppm.

6-Methyl-2-tosylbenzo[*d*][1,2,3]diazaborinin-1(2*H*)-ol (diazaborine 12) was prepared *via* the general method B work-up 2 by refluxing for 2 h at 70 °C hydrazone **S20** (3.0 g, 10.4 mmol, 1 equiv), boron tribromide (8.0 g, 31.8 mmol, 3.06 equiv), and anhydrous iron (III) chloride (124 mg, 0.77 mmol, 0.07 equiv) in 220 ml of DCE. Obtained 2.50 g (76% yield) of the title compound as a white solid. **^{1}H NMR (600 MHz, $CDCl_3$, 298 K)** δ 8.09 (1H, d, *J*=7.7 Hz, Ar***H***), 7.96 (1H, s, ***H***C=N), 7.93-7.90 (2H, m, 2x Ar***H***), 7.47 (1H, ddt, *J*=7.7, 1.6, 0.8 Hz, Ar***H***), 7.38 (1H, s, B-O***H***), 7.36 (1H, br. s, Ar***H***), 7.34-7.31 (2H, m, 2x Ar***H***), 2.47 (3H, s, ArC$\boldsymbol{H_3}$), 2.42 (3H, s, ArC$\boldsymbol{H_3}$); **^{13}C NMR (151 MHz, $CDCl_3$, 298 K)** δ 145.2 (Ar***C***-CH_3), 143.9 (***C***=N-N), 143.5 (Ar***C***-CH_3), 135.1 (Ar***C***), 134.5 (Ar***C***), 132.3 (2x Ar***C***H), 129.8 (2x Ar***C***H), 128.5 (2x Ar***C***H), 127.9 (Ar***C***H), 127.1 (br., Ar***C***-B(OH)), 22.0 (ArC-***C***H_3), 21.8 (ArC-***C***H_3); **^{11}B NMR (160 MHz, $CDCl_3$, 298 K)** δ 28.6; **HRMS (ESI)**: m/z calcd. for $C_{30}H_{30}B_2N_4O_6S_2Na$ $[2M+Na]^+$: 651.1696, found 651.1677, error 1.316 ppm.

6-Methyl-2-(phenylsulfonyl)benzo[*d*][1,2,3]diazaborinin-1(2*H*)-ol (diazaborine 13) was prepared according to the general method B work-up 2 by refluxing for 2 h at 70 °C hydrazone **S19** (10.0 g, 36.4 mmol, 1 equiv), boron tribromide (27.9 g, 111 mmol, 3.06 equiv), and anhydrous iron (III) chloride (435 mg, 2.68 mmol, 0.07 equiv) in 780 ml of DCE. Obtained 8.33 g (76% yield) of the title compound as a white solid. **^{1}H NMR (500 MHz, $CDCl_3$, 298 K)** δ 8.10 (1H, d, *J*=7.7 Hz, Ar***H***), 8.06-8.01 (2H, m, 2x Ar***H***), 7.97 (1H, s, ***H***C=N), 7.62 (1H, m, Ar***H***), 7.57-7.51 (2H, m, 2x Ar***H***), 7.48 (1H, m, Ar***H***), 7.36 (2H, m, Ar***H*** and B-O***H***),

2.48 (3H, s, ArC***H*₃**); **^{13}C NMR (126 MHz, $CDCl_3$, 298 K)** δ 144.1 (***C***=N-N), 143.6 (Ar***C***-CH_3), 138.1 (Ar***C***), 134.5 (Ar***C***), 134.1 (Ar***C***H), 132.3 (Ar***C***H), 132.3 (Ar***C***H), 129.2 (2x Ar***C***H), 128.4 (2x Ar***C***H), 128.0 (Ar***C***H), 127.0 (br., Ar***C***-B(OH)), 22.0 (ArC-***C***H_3); **^{11}B NMR (160 MHz, $CDCl_3$, 298 K)** δ 28.6; **HRMS (ESI)**: m/z calcd. for $C_{28}H_{26}B_2N_4O_6S_2Na$ $[2M+Na]^+$: 623.1372, found 623.1370, error 0.405 ppm.

2-((5-Bromothiophen-2-yl)sulfonyl)-6-methylbenzo[*d*][1,2,3]diazaborinin-1(2*H*)-ol (diazaborine 14) was obtained *via* the general method B work-up 2 by refluxing for 2 h at 70 °C hydrazone **S22** (2.0 g, 5.57 mmol, 1 equiv), boron tribromide (4.27 g, 17.0 mmol, 3.06 equiv), and anhydrous iron (III) chloride (66 mg, 0.41 mmol, 0.07 equiv) in 120 ml of DCE. Obtained 1.60 g (75% yield) of the title compound as a yellow solid. **^{1}H NMR (600 MHz, $CDCl_3$, 298 K)** δ 8.09 (1H, d, *J*=7.7 Hz, Ar***H***), 8.05 (1H, s, ***H***C=N), 7.60 (1H, d, *J*=4.1 Hz, Het***H***), 7.50 (1H, dt, *J*=7.7, 1.1 Hz, Ar***H***), 7.41 (1H, s, Ar***H***), 7.09 (1H, s, B-O***H***), 7.09 (1H, d, *J*=4.1 Hz, Het***H***), 2.50 (3H, s, ArC***H*₃**); **^{13}C NMR (151 MHz, $CDCl_3$, 298 K)** δ 144.4 (***C***=N-N), 143.8 (Ar***C***-CH_3), 138.6 (Het***C***), 134.7 (Het***C***H), 134.5 (Ar***C***), 132.5 (Ar***C***H), 132.4 (Ar***C***H), 130.4 (Het***C***H), 128.2 (Ar***C***H), 123.1 (Het***C***-Br), 22.0 (ArC-***C***H_3). *Note*: *The resonance corresponding to the quaternary carbon directly bonded to the B-OH group was not observed due to signal broadening caused by the fast relaxation induced by the quadrupolar boron nucleus as well as possible residual J-coupling to boron*; **^{11}B NMR (160 MHz, $CDCl_3$, 298 K)** δ 28.4; **HRMS (ESI)**: m/z calcd. for $C_{12}H_{10}BBrN_2O_3S_2Na$ $[M+Na]^+$: 406.9301, found 406.9306, error 1.074 ppm.

2-((5-Chlorothiophen-2-yl)sulfonyl)-6-methylbenzo[*d*][1,2,3]diazaborinin-1(2*H*)-ol (diazaborine 15) was obtained *via* the general method B work-up 2 by refluxing for 2 h at 70 °C hydrazone **S23** (1.5 g, 4.77 mmol, 1 equiv), boron tribromide (3.65 g, 14.6 mmol, 3.06 equiv), and anhydrous iron (III) chloride (57 mg, 0.35 mmol, 0.07 equiv) in 100 ml of DCE. Obtained 1.37 g (84% yield) of the title compound as a yellow solid. **^{1}H NMR (600 MHz, $CDCl_3$, 298 K)** δ 8.09 (1H, d, *J*=7.7 Hz, Ar***H***), 8.05 (1H, d, *J*=0.7 Hz, ***H***C=N), 7.64 (1H, d, *J*=4.1 Hz, Het***H***), 7.50 (1H, ddd, *J*=7.7, 1.6 Hz, 0.8 Hz, Ar***H***), 7.41 (1H, dt, *J*=1.6, 0.8 Hz, Ar***H***), 7.10 (1H, s, B-O***H***), 6.95 (1H, d, *J*=4.1 Hz, Het***H***), 2.50 (3H, s, ArC***H*₃**); **^{13}C NMR (151 MHz, $CDCl_3$, 298 K)** δ 144.4 (***C***=N-N), 143.8 (Ar***C***-CH_3), 140.3 (Het***C***), 135.7 (Het***C***-Cl), 134.5 (Ar***C***), 134.1 (Het***C***H), 132.5 (Ar***C***H), 132.4 (Ar***C***H), 128.2 (Ar***C***H), 126.8 (Het***C***H), 22.0 (ArC-***C***H_3). *Note*: *The resonance corresponding to the quaternary carbon directly bonded to the B-OH group was not observed due to signal broadening caused by the fast relaxation induced by the quadrupolar boron nucleus as well as possible residual J-coupling to boron*; **^{11}B NMR (160 MHz**, $CDCl_3$, 298 K) δ 28.3; **HRMS (ESI)**: m/z calcd. for $C_{12}H_{10}BClN_2O_3S_2Na$ $[M+Na]^+$: 362.9807, found 362.9808, error 0.289 ppm.

2-((4-Fluorophenyl)sulfonyl)-6-methylbenzo[*d*][1,2,3]diazaborinin-1(2*H*)-ol (diazaborine 16) was obtained *via* the general method B work-up 2 by refluxing for 2 h at 70 °C hydrazone **S21** (0.56 g, 1.57 mmol, 1 equiv), boron tribromide (1.2 g, 4.8 mmol, 3.06 equiv), and anhydrous iron (III) chloride (36 mg, 0.220 mmol, 0.14 equiv) in 35 ml of DCE. Obtained 327 mg (65% yield) of the title compound as a white solid. **^{1}H NMR (500 MHz, $CDCl_3$, 298 K)** δ 8.13-8.03 (3H, m, 3x Ar***H***), 7.97 (1H, s, ***H***C=N), 7.49 (1H, d, *J*=7.7 Hz, Ar***H***), 7.38 (1H, s, Ar***H***), 7.30 (1H, s, B-O***H***), 7.24-7.17 (2H, m, 2x Ar***H***), 2.48 (3H, s, ArC***H*₃**); **^{13}C NMR (126 MHz, $CDCl_3$, 298 K)** δ 166.0 (Ar***C***-F, d, *J*=257.2 Hz), 144.2 (***C***=N-N), 143.8 (Ar***C***-CH_3), 134.5 (Ar***C***), 134.1 (Ar***C***, d, *J*=3.3 Hz), 132.4 (Ar***C***H), 132.3 (Ar***C***H), 131.4 (2x Ar***C***H, d, *J*=9.8 Hz), 128.8 (Ar***C***H), 116.6 (2x Ar***C***H, d, *J*=22.9 Hz), 22.0 (ArC-***C***H_3). *Note*: *The resonance corresponding to the quaternary carbon directly bonded to the B-OH group was not observed due to signal broadening caused by the fast relaxation induced by the quadrupolar boron nucleus as well as possible residual J-coupling to boron*; **^{19}F NMR (377 MHz, $CDCl_3$, 298 K)** δ -102.7 (tt, *J*=8.3, 4.9 Hz); **^{11}B NMR (160 MHz, $CDCl_3$, 298 K)** δ 28.5; **HRMS (ESI)**: m/z calcd. for $C_{14}H_{11}BFN_2O_3S$ $[M-H]^-$: 317.0576, found 317.0576, error 1.063 ppm.

6-Methyl-2-(propylsulfonyl)benzo[*d*][1,2,3]diazaborinin-1(2*H*)-ol (diazaborine 17). To a DCE solution (15 ml, 0.05M) of hydrazone **S24** (200 mg, 0.83 mmol, 1 equiv) was added dropwise boron tribromide (638 mg, 2.55 mmol, 3.07 equiv), and the reaction mixture was allowed to stir for 45 min at room temperature when it was gently quenched with 20 ml of water. Then, 50 ml of DCM were added to the resulting solution which was washed with water (2x). The remaining organic phase was extracted three times with a 1M aqueous solution of NaOH. The aqueous phases were combined and slowly acidified with a 1M HCl solution until

pH~2. The resulting water solution was extracted with ethyl acetate (3x). The resulting combined organic extracts were washed with brine, dried over Na_2SO_4, filtered and evaporated. The remaining solid residue was recrystallized overnight from ca. 70 ml of a pentane-toluene 1:1 mixture at -20 °C to afford the title compound (100.0 mg, 45 % yield) as a white solid. **^{1}H NMR (500 MHz, $CDCl_3$, 298 K)** δ 8.09 (1H, d, *J*=7.8 Hz, Ar***H***), 8.08 (1H, s, ***H***C=N), 7.51 (1H, dd, *J*=7.8, 1.6 Hz, Ar***H***), 7.45 (1H, s, Ar***H***), 7.15 (1H, s, B-O***H***), 3.50-3.41 (2H, m, SO_2-C***H₂***), 2.51 (3H, s, ArC***H₃***), 1.88-1.75 (2H, m, SO_2CH_2-C***H₂***), 1.05 (3H, t, *J*=7.5 Hz, $SO_2CH_2CH_2$-C***H₃***); **^{13}C NMR (126 MHz, $CDCl_3$, 298 K)** δ 144.1 (***C***=N-N), 143.7 (ArC-***C***H₃), 134.5 (Ar***C***), 132.4 (Ar***C***H), 132.3 (Ar***C***H), 128.1 (Ar***C***H), 126.9 (br., Ar***C***-B(OH)), 53.3 (SO_2-***C***H₂), 22.0 (ArC-***C***H₃), 17.0 (SO_2CH_2-***C***H₂), 12.8 ($SO_2CH_2CH_2$-***C***H₃); **^{11}B NMR (160 MHz, $CDCl_3$, 298 K)** δ 28.6; **HRMS (ESI)**: m/z calcd. for $C_{13}H_{20}BN_2O_4S$ $[M+C_2H_5O]^-$: 311.1243, found 311.1242, error 0.078 ppm.

5-Bromo-2-((4-nitrophenyl)sulfonyl)benzo[*d*][1,2,3]diazaborinin-1(2*H*)-ol (diazaborine 18) was obtained according to the general method B work-up 1 by refluxing for 2 h at 70 °C hydrazone **S25** (1.2 g, 3.12 mmol, 1 equiv), boron tribromide (2.4 g, 9.55 mmol, 3.06 equiv), and anhydrous iron (III) chloride (37 mg, 0.23 mmol, 0.07 equiv) in 65 ml of DCE. Obtained 1.07 g (83% yield) of the title compound as a yellowish solid. **^{1}H NMR (500 MHz, $CDCl_3$)** δ 8.48 (1H, s, ***H***C=N), 8.42-8.37 (2H, m, 2x Ar***H***), 8.29-8.23 (2H, m, 2x Ar***H***), 8.17 (1H, d, *J*=7.5 Hz, Ar***H***), 7.92 (1H, dd, *J*=7.9, 1.2 Hz, Ar***H***), 7.52 (1H, app. t, *J*=7.7 Hz, Ar***H***), 7.25 (1H, br. s, B-O***H***); **^{13}C NMR (126 MHz, $CDCl_3$)** δ 151.1 (Ar***C***-NO₂), 143.0 (Ar***C***), 142.9 (***C***=N-N), 137.5 (Ar***C***H), 132.4 (Ar***C***H), 132.2 (Ar***C***), 131.7 (Ar***C***H), 130.2 (2x Ar***C***H), 124.5 (2x Ar***C***H), 123.4 (Ar***C***-Br). *Note*: *The resonance corresponding to the quaternary carbon directly bonded to the B-OH group was not observed due to signal broadening caused by the fast relaxation induced by the quadrupolar boron nucleus as well as possible residual J-coupling to boron*; **^{11}B NMR (160 MHz, $CDCl_3$, 298 K))** δ 28.1; **HRMS (ESI)**: m/z calcd. for $C_{26}H_{18}B_2Br_2N_6O_{10}S_2Na$ $[2M+Na]^+$: 840.8984, found 840.8987, error 1.905 ppm.

6-Bromo-2-((4-nitrophenyl)sulfonyl)benzo[*d*][1,2,3]diazaborinin-1(2*H*)-ol (diazaborine 19) was prepared according to the general method B work-up 1 by refluxing for 2 h at 70 °C hydrazone **S26** (1.0 g, 2.60 mmol, 1 equiv), boron tribromide (2.0 g, 7.96 mmol, 3.06 equiv), and anhydrous iron (III) chloride (31 mg, 0.19 mmol, 0.07 equiv) in 60 ml of DCE. Obtained 0.9 g (84% yield) of the title compound as a yellowish solid. **^{1}H NMR (600 MHz, $CDCl_3$)** δ 8.42-8.37 (2H, m, 2x Ar***H***), 8.27-8.22 (2H, m, 2x Ar***H***), 8.08 (1H, d, *J*=8.1 Hz, Ar***H***), 7.95 (1H, s, ***H***C=N), 7.80 (1H, ddd, *J*=8.1, 1.8, 0.8 Hz, Ar***H***), 7.75 (1H, m, Ar***H***), 7.25 (1H, br. s, B-O***H***); **^{13}C NMR (151 MHz, $CDCl_3$)** δ 151.1 (Ar***C***-NO₂), 143.2 (***C***=N-N), 143.1 (Ar***C***), 135.4 (Ar***C***), 134.6 (Ar***C***H), 134.1 (Ar***C***H), 130.7 (Ar***C***H), 130.1 (2x Ar***C***H), 128.3 (Ar***C***-Br), 124.5 (2x Ar***C***H). *Note*: *The resonance corresponding to the quaternary carbon directly bonded to the B-OH group was not observed due to signal broadening caused by the fast relaxation induced by the quadrupolar boron nucleus as well as possible residual J-coupling to boron*; **^{11}B NMR (128 MHz, $CDCl_3$)** δ 28.8; **HRMS (ESI)**: m/z calcd. for $C_{13}H_9BBrN_3O_5SNa$ $[M+Na]^+$: 431.9432, found 431.9431, error 0.055 ppm.

2-((4-Aminophenyl)sulfonyl)-6-bromobenzo[*d*][1,2,3]diazaborinin-1(2*H*)-ol (diazaborine 20) was synthesized according to the general method D by refluxing diazaborine **19** (500 mg, 1.22 mmol, 1 equiv), ammonium chloride (978 mg, 18 mmol, 15 equiv) and iron turnings (681 g, 12 mmol, 10 equiv) in a H_2O:MeOH mixture (1:1, 20 ml) for 2 h at 70 C. Obtained 136 mg (29% yield) of the title compound as a yellowish solid. **^{1}H NMR (600 MHz, DMSO-*d₆*, 300K)** δ 8.61 (1H, br. s, B-O***H***), 8.13 (1H, s, ***H***C=N), 8.08 (1H, d, *J*=8.1 Hz, Ar***H***), 8.03 (1H, d, *J*=1.9 Hz, Ar***H***), 7.86 (1H, dd, *J*=8.1, 1.9 Hz, Ar***H***), 7.65-7.58 (2H, m, 2x Ar***H***), 6.64-6.57 (2H, m, 2x Ar***H***), 6.22 (2H, br. s, ArN***H₂***); **^{13}C NMR (151 MHz, DMSO-*d₆*, 298K)** δ 154.0 (Ar***C***-NH₂), 140.2 (***C***=N-N), 135.7 (Ar***C***), 133.8 (Ar***C***H), 133.2 (Ar***C***H), 130.1 (3x Ar***C***H), 128.5 (br., Ar***C***-B(OH)), 126.2 (Ar***C***), 122.3 (Ar***C***-Br), 112.5 (2x Ar***C***H); **^{11}B NMR (128 MHz, DMSO-*d₆*)** δ 26.2; **HRMS (ESI)**: m/z calcd. for $C_{13}H_{11}BBrN_3O_3SNa$ $[M+Na]^+$: 401.9689, found 401.9692, error 0.255 ppm.

6-Bromo-2-((3-nitrophenyl)sulfonyl)benzo[*d*][1,2,3]diazaborinin-1(2*H*)-ol (diazaborine 21) was prepared according to the general method B work-up 2 by refluxing for 2 h at 70 °C hydrazone **S27** (3.0 g, 7.81 mmol, 1 equiv), boron tribromide (5.98 g, 23.9 mmol, 3.06 equiv), and anhydrous iron (III) chloride (93 mg, 0.57 mmol, 0.07 equiv) in 165 ml of DCE. Obtained 2.76 g (86% yield) of the title compound as a yellowish solid. **^{1}H NMR (600 MHz, $CDCl_3$, 300K)** δ 8.89 (1H, t, *J*=2.0 Hz, Ar***H***), 8.51 (1H, ddd, *J*=8.3, 2.2, 1.1 Hz, Ar***H***),

8.39 (1H, ddd, *J*=7.9, 1.8, 1.1 Hz, Ar***H***), 8.09 (1H, d, *J*=8.1 Hz, Ar***H***), 7.96 (1H, d, *J*=0.7 Hz, ***H***C=N), 7.82-7.77 (2H, m, 2x Ar***H***), 7.75 (1H, d, *J*=1.8 Hz, Ar***H***), 7.25 (1H, s, B-O***H***); **^{13}C NMR (151 MHz, $CDCl_3$, 298K)** δ 148.4 (Ar***C***-NO_2), 143.2 (***C***=N-N), 139.6 (Ar***C***), 135.4 (Ar***C***), 134.6 (Ar***C***H), 134.2 (Ar***C***H), 134.2 (Ar***C***H), 130.7 (Ar***C***H), 130.6 (Ar***C***H), 128.7 (Ar***C***H), 128.3 (Ar***C***-Br), 124.1 (Ar***C***H). *Note*: *The resonance corresponding to the quaternary carbon directly bonded to the B-OH group was not observed due to signal broadening caused by the fast relaxation induced by the quadrupolar boron nucleus as well as possible residual J-coupling to boron*; **^{11}B NMR (128 MHz, $CDCl_3$, 298K)** δ 28.3; **HRMS (ESI)**: m/z calcd. for $C_{13}H_{10}BBrN_3O_5S$ $[M+H]^+$: 409.9615, found 409.9613, error 0.303 ppm.

2-((3-Aminophenyl)sulfonyl)-6-bromobenzo[*d*][1,2,3]diazaborinin-1(2*H*)-ol (diazaborine 22) was synthesized *via* the general method D by refluxing diazaborine **21** (500 mg, 1.22 mmol, 1 equiv), ammonium chloride (978 mg, 18 mmol, 15 equiv) and iron turnings (681 g, 12 mmol, 10 equiv) in a H_2O:MeOH mixture (1:1, 20 ml) for 2 h at 70 C. Then, the mixture was cooled down and the liquid phase was transferred to a separating funnel where it was extracted three times with $CHCl_3$. The combined organic layer was washed with brine, dried over Na_2SO_4, evaporated, redissolved in dichloromethane and plugged through silica using ethyl acetate as an eluent to afford the title compound (277 mg, 60% yield) as a yellow solid. **^{1}H NMR (500 MHz, $CDCl_3$, 298K)** δ 8.07 (1H, d, *J*=8.1 Hz, Ar***H***), 7.96 (1H, s, ***H***C=N), 7.77 (1H, dd, *J*=8.1, 1.8 Hz, Ar***H***), 7.74 (1H, d, *J*=1.8 Hz, Ar***H***), 7.40 (1H, s, B-O***H***), 7.36 (1H, ddd, *J*=7.9, 1.8, 1.0 Hz, Ar***H***), 7.31-7.26 (2H, m, 2x Ar***H***), 6.88 (1H, ddd, *J*=8.0, 2.4, 1.0 Hz, Ar***H***), 3.94 (2H, br. s, ArN***H₂***); **^{13}C NMR (151 MHz, $CDCl_3$, 298K)** δ 147.3 (Ar***C***-NH_2), 142.3 (***C***=N-N), 138.5 (Ar***C***), 135.8 (Ar***C***), 134.1 (Ar***C***H), 134.0 (Ar***C***H), 130.4 (Ar***C***H), 130.3 (Ar***C***H), 127.9 (Ar***C***-Br), 120.3 (Ar***C***H), 117.9 (Ar***C***H), 113.9 (Ar***C***H). *Note*: *The resonance corresponding to the quaternary carbon directly bonded to the B-OH group was not observed due to signal broadening caused by the fast relaxation induced by the quadrupolar boron nucleus as well as possible residual J-coupling to boron*; **^{11}B NMR (160 MHz, $CDCl_3$, 298K)** δ 28.4; **HRMS (ESI)**: m/z calcd. for $C_{13}H_{12}BBrN_3O_3S$ $[M+H]^+$: 379.9873, found 379.987, error 0.070 ppm.

6-Bromo-2-(phenylsulfonyl)benzo[*d*][1,2,3]diazaborinin-1(2*H*)-ol (diazaborine 23) was prepared according to the general method B work-up 2 by refluxing for 2 h at 70 °C hydrazone **S28** (2.0 g, 5.90 mmol, 1 equiv), boron tribromide (4.52 g, 18.0 mmol, 3.06 equiv), and anhydrous iron (III) chloride (70 mg, 0.43 mmol, 0.07 equiv) in 125 ml of DCE. Obtained 1.90 g (88% yield) of the title compound as a yellowish solid. **^{1}H NMR (600 MHz, $CDCl_3$, 298K)** δ 8.07 (1H, d, *J*=8.1 Hz, Ar***H***), 8.06-8.03 (2H, m, 2x Ar***H***), 7.94 (1H, s, ***H***C=N), 7.77 (1H, dd, *J*=8.1, 1.7 Hz, Ar***H***), 7.73 (1H, d, *J*=1.8 Hz, Ar***H***), 7.65 (1H, m, Ar***H***), 7.58-7.53 (2H, m, 2x Ar***H***), 7.40 (1H, br. s, B-O***H***); **^{13}C NMR (151 MHz, $CDCl_3$, 298K)** δ 142.4 (***C***=N-N), 137.7 (Ar***C***), 135.7 (Ar***C***), 134.3 (Ar***C***H), 134.2 (Ar***C***H), 134.0 (Ar***C***H), 130.4 (Ar***C***H), 129.3 (2x Ar***C***H), 128.5 (2x Ar***C***H), 127.9 (Ar***C***-Br). *Note*: *The resonance corresponding to the quaternary carbon directly bonded to the B-OH group was not observed due to signal broadening caused by the fast relaxation induced by the quadrupolar boron nucleus as well as possible residual J-coupling to boron*; **^{11}B NMR (160 MHz, $CDCl_3$, 298K)** δ 28.5; **HRMS (ESI):** m/z calcd. for $C_{13}H_9BBrN_2O_3S$ $[M-H]^-$: 362.9618, found 362.9614, error 0.567 ppm.

6-Bromo-2-tosylbenzo[*d*][1,2,3]diazaborinin-1(2*H*)-ol (diazaborine 24) was prepared according to the general method B work-up 2 by refluxing for 2 h at 70 °C hydrazone **S29** (2.5 g, 7.08 mmol, 1 equiv), boron tribromide (5.42 g, 21.6 mmol, 3.06 equiv), and anhydrous iron (III) chloride (84 mg, 0.52 mmol, 0.07 equiv) in 150 ml of DCE. Obtained 2.17 g (81% yield) of the title compound as a white solid. **^{1}H NMR (500 MHz, $CDCl_3$, 298K)** δ 8.06 (1H, d, *J*=8.1 Hz, Ar***H***), 7.93 (1H, s, ***H***C=N) 7.93-7.88 (2H, m, Ar***H***), 7.76 (1H, dd, *J*= 8.1, 1.8 Hz, Ar***H***), 7.71 (1H, d, *J*=1.8 Hz, Ar***H***), 7.41 (1H, s, B-O***H***), 7.37-7.31 (2H, m, 2x Ar***H***), 2.43 (3H, s, ArC***H₃***); **^{13}C NMR (126 MHz, $CDCl_3$, 298K)** δ 145.5 (Ar***C***-CH_3), 142.3 (***C***=N-N), 135.7 (Ar***C***), 134.7 (Ar***C***), 134.1 (Ar***C***H), 134.0 (Ar***C***H), 130.3 (Ar***C***H), 129.9 (2x Ar***C***H), 128.6 (2x Ar***C***H), 127.8 (Ar***C***-Br), 21.8 (ArC-***C***H₃). *Note*: *The resonance corresponding to the quaternary carbon directly bonded to the B-OH group was not observed due to signal broadening caused by the fast relaxation induced by the quadrupolar boron nucleus as well as possible residual J-coupling to boron*; **^{11}B NMR (160 MHz, $CDCl_3$, 298K)** δ 28.4; **HRMS (ESI)**: m/z calcd. for $C_{14}H_{13}BBrN_2O_4S$ $[M+OH]^-$: 394.9881, found 394.9882, error 1.018 ppm.

6-Bromo-2-((4-fluorophenyl)sulfonyl)benzo[*d*][1,2,3]diazaborinin-1(2*H*)-ol (diazaborine 25) was obtained following the general method B work-up 1 by refluxing for 2 h at 70 °C hydrazone **S30** (440 mg, 1.23 mmol, 1 equiv), boron tribromide (0.94 g, 3.77 mmol, 3.06 equiv), and anhydrous iron (III) chloride (28 mg, 0.172 mmol, 0.14 equiv) in 25 ml of DCE. Obtained 301 mg (64% yield) of the title compound as an off-white solid. **^{1}H NMR (500 MHz, $CDCl_3$, 298K)** δ 8.12-8.01 (3H, m, 3x Ar***H***), 7.95 (1H, s, ***H***C=N), 7.78 (1H, dd, *J*=8.1, 1.8 Hz, Ar***H***), 7.74 (1H, d, *J*=1.9 Hz, Ar***H***), 7.34 (1H, s, B-O***H***), 7.26-7.19 (2H, m, 2x Ar***H***); **^{13}C NMR (126 MHz, $CDCl_3$, 298K)** δ 166.2 (Ar***C***-F, d, *J*=257.4 Hz), 142.6 (***C***=N-N), 135.6 (Ar***C***), 134.3 (Ar***C***H), 134.0 (Ar***C***H), 133.7 (Ar***C*, d,** *J*=3.2 Hz), 131.6 (2x Ar***C***H, d, *J*=9.7 Hz), 130.5 (Ar***C***H), 128.0 (Ar***C***-Br), 116.7 (2x Ar***C***H, d, *J*=22.7 Hz). *Note*: *The resonance corresponding to the quaternary carbon directly bonded to the B-OH group was not observed due to signal broadening caused by the fast relaxation induced by the quadrupolar boron nucleus as well as possible residual J-coupling to boron*; **^{19}F NMR (470 MHz, $CDCl_3$, 298K)** δ -102.2 (tt, *J*=8.7, 4.6 Hz); **^{11}B NMR (160 MHz, $CDCl_3$, 298K)** δ 28.3; **HRMS (ESI)**: m/z calcd. for $C_{13}H_8BBrFN_2O_3S$ $[M–H]^-$: 380.9524, found 380.9526, error 1.142 ppm.

6-Bromo-2-((5-bromothiophen-2-yl)sulfonyl)benzo[*d*][1,2,3]diazaborinin-1(2*H*)-ol (diazaborine 26) was prepared according to the general method B work-up 2 by refluxing for 2 h at 70 °C hydrazone **S31** (1.8 g, 4.24 mmol, 1 equiv), boron tribromide (3.25 g, 13.0 mmol, 3.06 equiv), and anhydrous iron (III) chloride (51 mg, 0.31 mmol, 0.07 equiv) in 90 ml of DCE. Obtained 1.20 g (63% yield) of the title compound as an off-white solid. **^{1}H NMR (600 MHz, $CDCl_3$, 298K)** δ 8.06 (1H, d, *J*=8.0 Hz, Ar***H***), 8.03 (1H, s, ***H***C=N), 7.80-7.75 (2H, m, 2x Ar***H***), 7.61 (1H, d, *J*=4.1 Hz, Het***H***), 7.15 (1H, s, B-O***H***), 7.11 (1H, d, *J*=4.1 Hz, Het***H***); **^{13}C NMR (151 MHz, $CDCl_3$, 298K)** δ 142.7 (***C***=N-N), 138.1 (Het***C***), 135.6 (Ar***C***), 135.1 (Het***C***H), 134.4 (Ar***C***H), 134.1 (Ar***C***H), 130.6 (Ar***C***H), 130.5 (Het***C***H), 128.1 (Ar***C***-Br), 123.5 (Het***C***-Br). *Note*: *The resonance corresponding to the quaternary carbon directly bonded to the B-OH group was not observed due to signal broadening caused by the fast relaxation induced by the quadrupolar boron nucleus as well as possible residual J-coupling to boron*; **^{11}B NMR (128 MHz, $CDCl_3$, 298K)** δ 28.1; **HRMS (ESI)**: m/z calcd. for $C_{11}H_8BBr_2N_2O_4S_2$ $[M+OH]^-$: 464.8393, found 464.8386, error 1.026 ppm.

6-Bromo-2-((5-chlorothiophen-2-yl)sulfonyl)benzo[*d*][1,2,3]diazaborinin-1(2*H*)-ol (diazaborine 27) was prepared according to the general method B work-up 2 by refluxing for 2 h at 70 °C hydrazone **S32** (1.5 g, 3.95 mmol, 1 equiv), boron tribromide (3.03 g, 12.1 mmol, 3.06 equiv), and anhydrous iron (III) chloride (47 mg, 0.29 mmol, 0.07 equiv) in 90 ml of DCE. Obtained 1.20 g (75% yield) of the title compound as an off-white solid. **^{1}H NMR (600 MHz, $CDCl_3$, 298K)** δ 8.07 (1H, dt, *J*=7.9, 0.6 Hz, Ar***H***), 8.04 (1H, d, *J*=0.7 Hz, ***H***C=N), 7.79 (1H, dd, *J*=7.9 , 1.8 Hz, Ar***H***), 7.78 (1H, dd, *J*=1.8 Hz, 0.6 Hz, Ar***H***), 7.65 (1H, d, *J*=4.1 Hz, Het***H***), 7.14 (1H, s, B-O***H***), 6.97 (1H, d, *J*=4.1 Hz, Het***H***); **^{13}C NMR (151 MHz, $CDCl_3$, 298K)** δ 142.7 (***C***=N-N), 140.8 (Het***C***), 135.6 (Het***C***-Cl), 135.2 (Ar***C***), 134.5 (Het***C***H), 134.4 (Ar***C***H), 134.1 (Ar***C***H), 130.6 (Ar***C***H), 128.1 (Ar***C***-Br), 126.9 (Het***C***H). *Note*: *The resonance corresponding to the quaternary carbon directly bonded to the B-OH group was not observed due to signal broadening caused by the fast relaxation induced by the quadrupolar boron nucleus as well as possible residual J-coupling to boron*; **^{11}B NMR (160 MHz, $CDCl_3$, 298K)** δ 28.1; **HRMS (ESI)**: m/z calcd. for $C_{11}H_8BBrClN_2O_4S_2$ $[M+OH]^-$: 420.8898, found 420.8899, error 0.714 ppm.

7-Fluoro-2-((4-nitrophenyl)sulfonyl)benzo[*d*][1,2,3]diazaborinin-1(2*H*)-ol (diazaborine 28) was prepared according to the general method B work-up 1 by refluxing for 1 h at 70 °C hydrazone **S33** (1.0 g, 3.09 mmol, 1 equiv), boron tribromide (2.37 g, 9.46 mmol, 3.06 equiv), and anhydrous iron (III) chloride (37 mg, 0.23 mmol, 0.07 equiv) in 65 ml of DCE. Obtained 843 mg (78% yield) of the title compound as a yellowish solid. **^{1}H NMR (500 MHz, Acetone-*d_6*, 298K)** δ 8.51-8.47 (2H, m, 2x Ar***H***), 8.42-8.36 (2H, m, 2x Ar***H***), 8.21 (1H, s, ***H***C=N), 7.93 (1H, dd, *J*=8.7, 5.0 Hz, Ar***H***), 7.83 (1H, dd, *J*=8.6, 2.7 Hz, Ar***H***), 7.62 (1H, td, *J*=8.8, 2.7 Hz, Ar***H***). *Note: The resonance corresponding to the B-OH group was not detected*; **^{13}C NMR (126 MHz, Acetone-*d_6*, 298K)** δ 164.8 (Ar***C***-F, d, *J*=253 Hz), 152.0 (Ar***C***-NO_2), 144.3 (Ar***C***), 143.9 (***C***=N-N), 132.5 (Ar***C***H, d, *J*=8.6 Hz), 132.0 (Ar***C***, d, *J*=2.5 Hz), 131.0 (2x Ar***C***H), 125.3 (2x Ar***C***H), 121.9 (Ar***C***H, d, *J*=23.2 Hz), 117.9 (Ar***C***H, d, *J*=20.4 Hz). *Note*: *The resonance corresponding to the quaternary carbon directly bonded to the B-OH group was not observed due to signal broadening caused by the fast relaxation induced*

by the quadrupolar boron nucleus as well as possible residual J-coupling to boron; **^{19}F NMR (377 MHz, Acetone-*d$_6$*, 298K)** δ -108.4 (td, *J*=8.7, 5.1 Hz); **^{11}B NMR (128 MHz, Acetone-*d$_6$*, 298K)** δ 27.9; **HRMS (ESI):** m/z calcd. for $C_{13}H_{10}BFN_3O_5S$ $[M+H]^+$: 350.0415, found 350.0418, error 1.528 ppm.

6-Fluoro-2-((4-nitrophenyl)sulfonyl)benzo[*d*][1,2,3]diazaborinin-1(2*H*)-ol (diazaborine 29) was prepared according to the general method B work-up 1 by refluxing for 1 h at 70 °C hydrazone **S34** (1.0 g, 3.09 mmol, 1 equiv), boron tribromide (2.37 g, 9.46 mmol, 3.06 equiv), and anhydrous iron (III) chloride (64 mg, 0.40 mmol, 0.13 equiv) in 65 ml of DCE. Obtained 1.82 g (59% yield) of the title compound as a yellowish solid. **^{1}H NMR (500 MHz, Acetone-*d$_6$*, 298K)** δ 8.52-8.46 (2H, m, 2x Ar***H***), 8.43-8.36 (2H, m, 2x Ar***H***), 8.26 (1H, dd, *J*=8.4, 5.9 Hz, Ar***H***), 8.21 (1H, s, ***H***C=N), 7.70 (1H, s, B-O***H***), 7.58 (1H, dd, *J*=9.2, 2.4 Hz, Ar***H***), 7.53 (1H, ddd, *J*=8.9, 8.8, 2.5 Hz, Ar***H***); **^{13}C NMR (126 MHz, Acetone-*d$_6$*, 298K)** δ 166.5 (Ar***C***-F, d, *J*=251.4 Hz), 152.0 (Ar***C***-NO_2), 144.3 (Ar***C***), 143.9 (***C***=N-N), 137.5 (Ar***C***, d, *J*=8.8 Hz), 135.9 (Ar***C***H, d, *J*=9.0 Hz), 131.0 (2x Ar***C***H), 125.3 (2x Ar***C***H), 119.9 (Ar***C***H, d, *J*=21.8 Hz), 114.7 (Ar***C***H, d, *J*=21.4 Hz). *Note*: *The resonance corresponding to the quaternary carbon directly bonded to the B-OH group was not observed due to signal broadening caused by the fast relaxation induced by the quadrupolar boron nucleus as well as possible residual J-coupling to boron*; **^{19}F NMR (377 MHz, Acetone-*d$_6$*, 298K)** δ -106.7 (m); **^{11}B NMR (128 MHz, Acetone-*d$_6$*, 298K)** δ 27.9; **HRMS (ESI):** m/z calcd. for $C_{13}H_9BFN_3O_5SNa$ $[M+Na]^+$: 372.0235, found 372.0235, error 0.775 ppm.

5-Fluoro-2-((4-nitrophenyl)sulfonyl)benzo[*d*][1,2,3]diazaborinin-1(2*H*)-ol (diazaborine 30) was obtained through the general method B work-up 1 by refluxing for 2 h at 70 °C hydrazone **S35** (1.0 g, 3.09 mmol, 1 equiv), boron tribromide (2.37 g, 9.46 mmol, 3.06 equiv), and anhydrous iron (III) chloride (37 mg, 0.23 mmol, 0.07 equiv) in 65 ml of DCE. Obtained 810 mg (75% yield) of the title compound as a yellowish solid. **^{1}H NMR (500 MHz, $CDCl_3$, 298K)** δ 8.42-8.36 (2H, m, 2x Ar***H***), 8.34 (1H, s, ***H***C=N), 8.29-8.20 (2H, m, 2x Ar***H***), 7.99 (1H, d, *J*=7.5 Hz, Ar***H***), 7.66 (1H, td, *J*=7.8, 4.9 Hz, Ar***H***), 7.40 (1H, ddd, *J*=9.5, 8.2, 1.1 Hz, Ar***H***), 7.31 (1H, br. s, B-O***H***); **^{13}C NMR (126 MHz, $CDCl_3$, 298K)** δ 159.6 (Ar***C***-F, d, *J*=258.2 Hz), 151.0 (Ar***C***-NO_2), 143.1 (Ar***C***), 137.5 (***C***=N-N, d, *J*=6.3 Hz), 133.1 (Ar***C***H, d, *J*=7.5 Hz), 130.1 (2x Ar***C***H), 128.1 (Ar***C***H, d, *J*=4.1 Hz), 124.4 (2x Ar***C***H), 122.3 (Ar***C***, d, *J*=10.5 Hz), 119.3 (Ar***C***H, d, *J*=19.7 Hz). *Note*: *The resonance corresponding to the quaternary carbon directly bonded to the B-OH group was not observed due to signal broadening caused by the fast relaxation induced by the quadrupolar boron nucleus as well as possible residual J-coupling to boron*; **^{19}F NMR (470 MHz, $CDCl_3$, 298K)** δ -121.4 (dd, *J*=9.7, 5.1 Hz); **^{11}B NMR (160 MHz, $CDCl_3$, 298K)** δ 28.1; **HRMS (ESI)**: m/z calcd. for $C_{13}H_9BFN_3O_5SNa$ $[M+Na]^+$: 372.0235, found 372.0231, error 0.775 ppm.

7-Chloro-2-((4-nitrophenyl)sulfonyl)benzo[*d*][1,2,3]diazaborinin-1(2*H*)-ol (diazaborine 31) was obtained according to the general method B work-up 1 by refluxing for 2 h at 70 °C hydrazone **S36** (0.7 g, 2.05 mmol, 1 equiv), boron tribromide (1.57 g, 6.27 mmol, 3.06 equiv), and anhydrous iron (III) chloride (25 mg, 0.15 mmol, 0.13 equiv) in 45 ml of DCE. Obtained 0.6 g (80% yield) of the title compound as a yellowish solid. **^{1}H NMR (500 MHz, Acetone-*d$_6$*-DMSO-*d$_6$* 2:1, 298 K)** δ 8.48-8.41 (2H, m, 2x Ar*H*), 8.35-8.30 (2H, m, 2x Ar***H***), 8.14 (1H, s, Ar***H***), 8.04 (1H, s, ***H***C=N), 7.71 (2H, br. s, 2x Ar***H***). *The resonance corresponding to the B-OH group was not detected*; **^{13}C NMR (126 MHz, Acetone-*d$_6$*-DMSO-*d$_6$* 2:1, 298 K)** δ 150.8 (Ar***C***-NO_2), 145.5 (Ar***C***), 141.9 (***C***=N-N), 136.3 (Ar***C***-Cl), 132.4 (Ar***C***), 132.0 (Ar***C***H), 131.6 (Ar***C***H), 130.1 (3x Ar***C***H), 124.7 (2x Ar***C***H). *Note*: *The resonance corresponding to the quaternary carbon directly bonded to the B-OH group was not observed due to signal broadening caused by the fast relaxation induced by the quadrupolar boron nucleus as well as possible residual J-coupling to boron*; **^{11}B NMR (160 MHz, Acetone-*d$_6$*-DMSO-*d$_6$* 2:1, 298 K)** δ 21.9. **HRMS (ESI)**: m/z calcd. for $C_{13}H_9BClN_3O_5SNa$ $[M+Na]^+$: 387.9939, found 387.9942, error 1.286 ppm.

6-Chloro-2-((4-nitrophenyl)sulfonyl)benzo[*d*][1,2,3]diazaborinin-1(2*H*)-ol (diazaborine 32) was prepared according to the general method B work-up 2 by refluxing for 2 h at 70 °C hydrazone **S37** (1.5 g, 4.41 mmol, 1 equiv), boron tribromide (3.38 g, 13.5 mmol, 3.06 equiv), and anhydrous iron (III) chloride (53 mg, 0.32 mmol, 0.07 equiv) in 100 ml of DCE. Obtained 1.34 g (83% yield) of the title compound as a yellowish solid. **^{1}H NMR (500 MHz, $CDCl_3$, 298 K)** δ 8.43-8.37 (2H, m, 2x Ar***H***), 8.29-8.22 (2H, m, 2x Ar***H***), 8.16 (1H, d,

J=8.1 Hz, Ar***H***), 7.96 (1H, s, ***H***C=N), 7.65 (1H, dd, *J*= 8.1, 1.9 Hz, Ar***H***), 7.58 (1H, d, *J*=1.9 Hz, Ar***H***), 7.25 (1H, s, B-O***H***); **^{13}C NMR (126 MHz, $CDCl_3$, 298 K)** δ 151.1 (Ar***C***-NO_2), 143.4 (***C***=N-N), 143.1 (Ar***C***), 139.9 (Ar***C***-Cl), 135.3 (Ar***C***), 134.2 (Ar***C***H), 131.8 (Ar***C***H), 130.1 (2x Ar***C***H), 127.6 (Ar***C***H), 124.5 (2x Ar***C***H). *Note*: *The resonance corresponding to the quaternary carbon directly bonded to the B-OH group was not observed due to signal broadening caused by the fast relaxation induced by the quadrupolar boron nucleus as well as possible residual J-coupling to boron*; **^{11}B NMR (160 MHz, $CDCl_3$, 298 K)** δ 28.3; **HRMS (ESI):** m/z calcd. for $C_{13}H_{10}BClN_3O_6S$ $[M+OH]^-$: 382.0080, found 382.0075, error 0.563 ppm.

6-Chloro-2-((3-nitrophenyl)sulfonyl)benzo[*d*][1,2,3]diazaborinin-1(2*H*)-ol (diazaborine 33) was prepared according to the general method B work-up 1 by refluxing for 2 h at 70 °C hydrazone **S39** (1.2 g, 3.53 mmol, 1 equiv), boron tribromide (2.71 g, 10.8 mmol, 3.06 equiv), and anhydrous iron (III) chloride (42 mg, 0.26 mmol, 0.07 equiv) in 75 ml of DCE. Obtained 1.10 g (85% yield) of the title compound as a yellowish solid. **^{1}H NMR (600 MHz, DMSO:$CDCl_3$ 1:1.2, 298 K)** δ 8.83 (1H, m, Ar***H***), 8.46 (1H, br. s, B-O***H***), 8.42 (1H, app. d, *J*=8.1 Hz, Ar***H***), 8.35 (1H, app. d, *J*= 7.9 Hz, Ar***H***), 8.12 (1H, m, Ar***H***), 7.95 (1H, d, *J*=2.4 Hz, ***H***C=N), 7.77 (1H, td, *J*=8.1, 2.4 Hz, Ar***H***), 7.57 (1H, m, Ar***H***), 7.52 (1H, m, Ar***H***); **^{13}C NMR (151 MHz, DMSO:$CDCl_3$ 1:1.2, 298 K)** δ 147.5 (Ar***C***-NO_2), 141.8 (***C***=N-N), 139.6 (Ar***C***), 138.3 (Ar***C***-Cl), 134.8 (Ar***C***), 133.5 (Ar***C***H), 133.5 (Ar***C***H), 130.5 (Ar***C***H), 130.2 (Ar***C***H), 127.7 (Ar***C***H), 126.8 (Ar***C***H), 123.0 (Ar***C***H). *Note*: *The resonance corresponding to the quaternary carbon directly bonded to the B-OH group was not observed due to signal broadening caused by the fast relaxation induced by the quadrupolar boron nucleus as well as possible residual J-coupling to boron*; **^{11}B NMR (128 MHz, DMSO:$CDCl_3$ 1:1.2, 298 K)** δ 31.7; **HRMS (ESI)**: m/z calcd. for $C_{13}H_8BClN_3O_5S$ $[M–H]^-$: 363.9974, found 363.9968, error 0.953 ppm.

5-Chloro-2-((4-nitrophenyl)sulfonyl)benzo[*d*][1,2,3]diazaborinin-1(2*H*)-ol (diazaborine 34) was prepared according to the general method B work-up 1 by refluxing for 2 h at 70 °C hydrazone **S38** (1.1 g, 3.24 mmol, 1 equiv), boron tribromide (2.48 g, 9.90 mmol, 3.06 equiv), and anhydrous iron (III) chloride (39 mg, 0.24 mmol, 0.07 equiv) in 70 ml of DCE. Obtained 0.90 g (76% yield) of the title compound as a yellowish solid. **^{1}H NMR (400 MHz, $CDCl_3$, 298 K)** δ 8.51 (1H, s, ***H***C=N), 8.44-8.36 (2H, m, 2x Ar***H***), 8.30-8.22 (2H, m, 2x Ar***H***), 8.13 (1H, d, *J*=7.5 Hz, Ar***H***), 7.73 (1H, dd, *J*=7.9, 1.2 Hz, Ar***H***), 7.60 (1H, app. t., *J*=7.7 Hz, Ar***H***), 7.27 (1H, br. s, B-O***H***); **^{13}C NMR (101 MHz, $CDCl_3$, 298 K)** δ 151.1 (Ar***C***-NO_2), 143.0 (Ar***C***), 140.5 (***C***=N-N), 134.1 (Ar***C***H), 133.5 (Ar***C***-Cl), 132.1 (Ar***C***H), 131.0 (Ar***C***H), 130.8 (Ar***C***), 130.2 (2x Ar***C***H), 124.5 (2x Ar***C***H). *Note*: *The resonance corresponding to the quaternary carbon directly bonded to the B-OH group was not observed due to signal broadening caused by the fast relaxation induced by the quadrupolar boron nucleus as well as possible residual J-coupling to boron*; **^{11}B NMR (128 MHz, $CDCl_3$, 298 K)** δ 27.7; **HRMS (ESI)**: m/z calcd. for $C_{13}H_9BClN_3O_5SNa$ $[M+Na]^+$: 387.9939, found 387.9947, error 2.658 ppm.

2-Tosylbenzo[*d*][1,2,3]diazaborinine-1,7(2*H*)-diol (diazaborine 35) was obtained according to the general method B work-up 1 by refluxing for 2 h at 70 °C hydrazone **S40** (1.0 g, 3.44 mmol, 1 equiv), boron tribromide (3.45 g, 13.8 mmol, 4 equiv), and anhydrous iron (III) chloride (41 mg, 0.25 mmol, 0.07 equiv) in 75 ml of DCE. Obtained 0.89 g (74% yield) of the title compound as a white solid. **^{1}H NMR (500 MHz, $CDCl_3$, 298 K)** δ 7.93-7.89 (3H, m, ***H***C=N and 2x Ar***H***), 7.55 (1H, d, *J*=2.6 Hz, Ar***H***), 7.46 (1H, d, *J*=8.4 Hz, Ar***H***), 7.41 (1H, s, B-O***H***), 7.35-7.30 (2H, m, 2x Ar***H***), 7.20 (1H, dd, *J*=8.4, 2.6 Hz, Ar***H***), 5.91 (1H, br. s, ArO***H***), 2.42 (3H, s, ArC***H₃***); **^{13}C NMR (126 MHz, $CDCl_3$, 298 K)** δ 158.0 (Ar***C***-OH), 145.3 (Ar***C***-CH_3), 143.3 (***C***=N-N), 135.0 (Ar***C***), 130.3 (Ar***C***H), 129.9 (2x Ar***C***H), 128.5 (2x Ar***C***H), 128.2 (Ar***C***), 121.3 (Ar***C***H), 117.1 (Ar***C***H), 21.8 (ArC-***C***H_3). *Note*: *The resonance corresponding to the quaternary carbon directly bonded to the B-OH group was not observed due to signal broadening caused by the fast relaxation induced by the quadrupolar boron nucleus as well as possible residual J-coupling to boron*; **^{11}B NMR (160 MHz, $CDCl_3$, 298 K)** δ 28.3; **HRMS (ESI)**: m/z calcd. for $C_{14}H_{14}BN_2O_4S$ $[M+H]^+$: 317.0765, found 317.0761, error 0.209 ppm.

2-Tosylbenzo[*d*][1,2,3]diazaborinine-1,6(2*H*)-diol (diazaborine 36) was synthesized according to general method C by reacting 5-hydroxy-2-(4,4,5,5-tetramethyl-1,3,2-dioxaborolan-2-yl)benzaldehyde (600 mg, 2.42 mmol, 1 equiv) and 4-methylbenzenesulfonohydrazide **S2** (450 mg, 2.42 mmol, 1 equiv) in an EtOH:H_2O solution (1:1, 18 mL) for 2 h at 80 °C. Obtained 556 mg (73% yield) of the title compound as a white solid. **^{1}H NMR (500 MHz, DMSO-*d₆*, 298 K)** δ 10.41 (1H, s, ArO***H***), 8.73 (1H, br. s, B-O***H***), 8.06 (1H, s, ***H***C=N),

8.04 (1H, s, Ar***H***), 7.90-7.84 (2H, m, 2x Ar***H***), 7.44-7.38 (2H, m, 2x Ar***H***), 7.12 (1H, dd, $^{3}J_{H\text{-}H}$ = 8.2 Hz, $^{4}J_{H\text{-}H}$ = 2.3 Hz, Ar***H***), 7.05 (1H, d, $^{4}J_{H\text{-}H}$ = 2.3 Hz, Ar***H***), 2.36 (3H, s, ArC***H₃***); **^{13}C NMR (126 MHz, DMSO-*d₆*, 298 K)** δ 161.4 (Ar***C***-OH), 144.4 (Ar***C***-CH_3), 142.4 (***C***=N-N), 136.3 (Ar***C***), 136.0 (Ar***C***), 134.1 (Ar***C***H), 129.7 (2x Ar***C***H), 127.7 (2x Ar***C***H), 120.3 (br., Ar***C***-B(OH)), 119.5 (Ar***C***H), 112.7 (Ar***C***H), 21.1 (ArC-***C***H_3); **^{11}B NMR (128 MHz, $CDCl_3$, 298 K)** δ 28.2; **HRMS (ESI)**: m/z calcd. for $C_{28}H_{26}B_2N_4O_8S_2Na$ $[2M+Na]^+$: 655.1280, found 655.1272, error 0.245 ppm.

2-((4-Nitrophenyl)sulfonyl)benzo[*d*][1,2,3]diazaborinine-1,6(2*H*)-diol (diazaborine 37) was synthesized according to general method C by reacting 5-hydroxy-2-(4,4,5,5-tetramethyl-1,3,2-dioxaborolan-2-yl)benzaldehyde (200 mg, 0.81 mmol, 1 equiv) and 4-nitrobenzenesulfonohydrazide **x4** (175 mg, 0.81 mmol, 1 equiv) in an EtOH:H_2O solution (1:1, 6 mL) for 2 h at 80 °C. Obtained 198 mg (71% yield) of the title compound as a white solid. **^{1}H NMR (600 MHz, Acetone-*d₆*, 298 K)** δ 9.34 (1H, s, ArO***H***), 8.52-8.45 (2H, m, 2x Ar***H***), 8.41-8.33 (2H, m, 2x Ar***H***), 8.08 (1H, s, ***H***C=N), 8.06 (1H, m, Ar***H***), 7.47 (1H, s, B-O***H***), 7.24 (1H, dd, *J*=8.2, 2.3 Hz, Ar***H***), 7.17 (1H, d, *J*=2.3 Hz, Ar***H***); **^{13}C NMR (126 MHz, DMSO-*d₆*, 298 K)** δ 161.4 (Ar***C***-OH), 150.2 (Ar***C***-NO_2), 144.3 (Ar***C***), 143.0 (***C***=N-N), 136.2 (Ar***C***), 134.2 (Ar***C***H), 129.2 (2x Ar***C***H), 124.6 (2x Ar***C***H), 120.2 (br., Ar***C***-B(OH)), 119.5 (Ar***C***H), 112.9 (Ar***C***H); **^{11}B NMR (160 MHz, Acetone-*d₆*, 298 K)** δ 28.2; **HRMS (ESI)**: m/z calcd. for $C_{13}H_9BN_3O_6S$ $[M–H]^-$: 346.0313, found 346.0311, error 0.092 ppm.

2-((5-Bromothiophen-2-yl)sulfonyl)benzo[*d*][1,2,3]diazaborinine-1,6(2*H*)-diol (diazaborine 38) was synthesized according to general method C by reacting 5-hydroxy-2-(4,4,5,5-tetramethyl-1,3,2-dioxaborolan-2-yl)benzaldehyde (200 mg, 0.81 mmol, 1 equiv) and 5-Bromothiophene-2-sulfonohydrazide **S8** (207 mg, 0.81 mmol, 1 equiv) in an EtOH:H_2O solution (1:1, 6 mL) for 2 h at 80 °C. Obtained 221 mg (71% yield) of the title compound as an off-white solid. **^{1}H NMR (600 MHz, DMSO-*d₆*, 300 K)** δ 10.43 (1H, s, ArO***H***), 9.16 (1H, br. s, B-O***H***), 8.13 (1H, s, ***H***C=N), 8.08 (1H, d, *J*=8.3 Hz, Ar***H***), 7.69 (1H, d, *J*=4.1 Hz, Het***H***), 7.38 (1H, d, *J*=4.1 Hz, Het***H***), 7.12 (1H, dd, *J*=8.3, 2.3 Hz, Ar***H***), 7.08 (1H, d, *J*=2.3 Hz, Ar***H***); **^{13}C NMR (151 MHz, DMSO-*d₆*, 300 K)** δ 161.4 (Ar***C***-OH), 142.8 (***C***=N-N), 139.7 (Het***C***), 136.2 (Ar***C***), 134.6 (Het***C***H), 134.2 (Ar***C***H), 131.2 (Het***C***H), 121.2 (Het***C***-Br), 120.2 (br., Ar***C***-B(OH)), 119.5 (Ar***C***H), 113.0 (Ar***C***H); **^{11}B NMR (160 MHz, $CDCl_3$, 298 K)** δ 28.0; **HRMS (ESI)**: m/z calcd. for $C_{11}H_9BBrN_2O_4S_2$ $[M+H]^+$: 386.9277, found 386.9276, error 0.304 ppm.

2-((4-Nitrophenyl)sulfonyl)thieno[3,2-*d*][1,2,3]diazaborinin-1(2*H*)-ol (diazaborine 39) was obtained according to the general method B work-up 1 by refluxing for 2 h at 70 °C hydrazone **S42** (1.1 g, 3.53 mmol, 1 equiv), boron tribromide (2.71 g, 10.8 mmol, 3.06 equiv), and anhydrous iron (III) chloride (42 mg, 0.26 mmol, 0.07 equiv) in 75 ml of DCE. Obtained 0.79 g (66% yield) of the title compound as a yellow solid. **^{1}H NMR (600 MHz, $CDCl_3$, 298 K)** δ 8.42-8.34 (2H, m, 2x Ar***H***), 8.29-8.21 (2H, m, 2x Ar***H***), 8.14 (1H, s, ***H***C=N), 7.69 (1H, d, *J*=4.9 Hz, Het***H***), 7.64 (1H, d, *J*=4.9 Hz, Het***H***), 7.20 (1H, br. s, B-O***H***); **^{13}C NMR (101 MHz, $CDCl_3$, 298 K)** δ 151.0 (Ar***C***-NO_2), 144.8 (Het***C***), 143.4 (Ar***C***), 137.4 (***C***=N-N), 131.6 (Het***C***H), 130.2 (Het***C***H), 130.0 (2x Ar***C***H), 124.4 (2x Ar***C***H). *Note*: *The resonance corresponding to the quaternary carbon directly bonded to the B-OH group was not observed due to signal broadening caused by the fast relaxation induced by the quadrupolar boron nucleus as well as possible residual J-coupling to boron*; **^{11}B NMR (128 MHz, $CDCl_3$, 298 K)** δ 27.4; **HRMS (ESI)**: m/z calcd. for $C_{11}H_9BN_3O_5S_2$ $[M+H]^+$: 338.0073, found 338.0070, error 0.325 ppm.

6-Methyl-2-((4-nitrophenyl)sulfonyl)thieno[3,2-*d*][1,2,3]diazaborinin-1(2*H*)-ol (diazaborine 40) was obtained according to the general method B work-up 1 by refluxing for 2 h at 70 °C hydrazone **S43** (1.1 g, 3.38 mmol, 1 equiv), boron tribromide (2.59 g, 10.3 mmol, 3.06 equiv), and anhydrous iron (III) chloride (40 mg, 0.25 mmol, 0.07 equiv) in 70 ml of DCE. Obtained 0.94 g (79% yield) of the title compound as a yellow solid. **^{1}H NMR (500 MHz, $CDCl_3$, 298 K)** δ 8.42-8.32 (2H, m, 2x Ar***H***), 8.27-8.17 (2H, m, 2x Ar***H***), 8.01 (1H, s, ***H***C=N), 7.27 (1H, br. s, Het***H***), 7.13 (1H, br. s, B-O***H***), 2.62 (3H, s, HetC***H₃***); **^{13}C NMR (126 MHz, $CDCl_3$, 298 K)** δ 150.9 (Ar***C***-NO_2), 147.6 (Het***C***-CH_3), 143.6 (Het***C***), 143.5 (Ar***C***), 137.3 (***C***=N-N), 130.0 (2x Ar***C***H), 128.4 (Het***C***H), 124.4 (2x Ar***C***H), 15.7 (HetC-***C***H_3). *Note*: *The resonance corresponding to the quaternary carbon directly bonded to the B-OH group was not observed due to signal broadening caused by*

the fast relaxation induced by the quadrupolar boron nucleus as well as possible residual J-coupling to boron; **^{11}B NMR (128 MHz, $CDCl_3$, 298 K)** δ 26.9; **HRMS (ESI)**: m/z calcd. for $C_{12}H_{11}BN_3O_5S_2$ $[M+H]^+$: 352.0230, found 352.0228, error 0.176 ppm.

6-Methyl-2-(propylsulfonyl)thieno[3,2-*d*][1,2,3]diazaborinin-1(2*H*)-ol (diazaborine 41) To an anhydrous DCM solution (75 ml, 0.06M) of hydrazone **S44 (**1.2 g, 4.87 mmol, 1 equiv) in a three-neck round-bottom flask equipped with septum, reflux condenser and magnetic stirrer under argon was added dropwise boron tribromide (3.66 g, 14.6 mmol, 3.0 equiv). Then, the resulting reaction mixture was allowed to stir for 2.5 h at room temperature. Subsequently, the reaction mixture was gently quenched with 100 ml of water, transferred to a separating funnel, and 100 ml of dichloromethane were added. The layers were shaken, separated, and the organic phase was additionally washed twice with water. The resulting organic layer was extracted with a 0.5 M aqueous solution of NaOH (3x 150 ml), and the remaining water extracts were combined and transferred to a beaker and gently acidified with an 1M aqueous solution of HCl until pH~2. The resulting suspension was extracted with ethyl acetate (3x150 ml), then the combined organic extracts were washed with brine, dried over Na_2SO_4, and concentrated. The resulting oil was suspended in 125 ml of pentane with vigorous stirring for 15 min and then the layers were separated. This last extraction was repeated several times until no product was observed by TLC in the oily residue. The resulting pentane layers were combined, evaporated and dried on vacuo to afford the title compound (0.52 g, 39% yield) as a yellow solid. **^{1}H NMR (500 MHz, $CDCl_3$, 298 K)** δ 8.12 (1H, s, ***H***C=N), 7.26 (1H, s, Het***H***), 6.97 (1H, br. s, B-O***H***), 3.50-3.41 (2H, m, SO_2-C***H₂***), 2.63 (3H, d, *J*=1.1 Hz, HetC***H₃***), 1.87-1.74 (2H, m, SO_2CH_2-C***H₂***), 1.04 (3H, t, *J*=7.5 Hz, $SO_2CH_2CH_2$-C***H₃***); **^{13}C NMR (126 MHz, $CDCl_3$, 298 K)** δ 147.1 (Het***C***-CH_3), 143.7 (Het***C***), 140.1 (br., Ar***C***-B(OH)), 136.5 (***C***=N-N), 128.3 (Het***C***H), 53.7 (SO_2-***C***H_2), 17.0 (SO_2CH_2-***C***H_2), 15.7 (HetC-***C***H_3), 12.8 ($SO_2CH_2CH_2$-***C***H_3); **^{11}B NMR (161 MHz, $CDCl_3$, 298 K)** δ 27.2; **HRMS (ESI)**: m/z calcd. for $C_{11}H_{18}BN_2O_4S_2$ $[M+OC_2H_5]^-$: 317.0807, found 317.0809, error 0.817 ppm.

6-Bromo-2-(propylsulfonyl)thieno[3,2-*d*][1,2,3]diazaborinin-1(2*H*)-ol (diazaborine 42) To an anhydrous DCE solution (25 ml, 0.04M) of hydrazone **S45 (**0.5 g, 1.60 mmol, 1 equiv) in a three-neck round-bottom flask equipped with septum, reflux condenser and magnetic stirrer under argon was added dropwise boron tribromide (1.2 g, 4.82 mmol, 3.0 equiv). Then, the septum was replaced by a glass stopper and the resulting solution was stirred at 60 °C for 2 h. Subsequently, the reaction mixture was gently quenched with 50 ml of water, transferred to a separating funnel, and 50 ml of dichloromethane were added. The layers were shaken, separated, and the organic phase was additionally washed twice with water. The resulting organic layer was extracted with a 0.5 M aqueous solution of NaOH (3x 100 ml). The remaining water extracts were combined, transferred to a beaker, and gently acidified with an 1M aqueous solution of HCl until pH~2. The resulting suspension was extracted with ethyl acetate (3x100 ml), then the combined organic extracts were washed with brine, dried over Na_2SO_4, and concentrated. The remaining solid was suspended in 40 ml of diethyl ether with vigorous stirring for 15 min and then 150 ml of pentane were slowly added. A precipitate was formed in the solution which was filtered-off and discarded. The resulting ether-pentane filtrate was evaporated, and the resulting solid residue was stirred for 30 min in 125 ml of pentane. This last extraction was repeated several times until no product was observed by TLC in the oily residue. The resulting pentane layers were combined, evaporated, and dried on vacuo to afford the title compound (68 mg, 13% yield) as a yellow solid. **^{1}H NMR (600 MHz, CD_2Cl_2, 300 K)** δ 8.13 (1H, s, ***H***C=N), 7.57 (1H, s, Het***H***), 7.08 (1H, s, B-O***H***), 3.47-3.43 (2H, m, SO_2-C***H₂***), 1.83-1.75 (2H, m, SO_2CH_2-C***H₂***), 1.03 (3H, t, *J*=7.5 Hz, $SO_2CH_2CH_2$-C***H₃***); **^{13}C NMR (151 MHz, CD_2Cl_2, 300 K)** δ 146.4 (Het***C***), 135.6 (***C***=N-N), 132.9 (Het***C***H), 120.5 (Het***C***-Br), 54.0 (SO_2-***C***H_2), 17.3 (SO_2CH_2-***C***H_2), 12.8 ($SO_2CH_2CH_2$-***C***H_3). *Note*: *The resonance corresponding to the quaternary carbon directly bonded to the B-OH group was not observed due to signal broadening caused by the fast relaxation induced by the quadrupolar boron nucleus as well as possible residual J-coupling to boron*; **^{11}B NMR (128 MHz, CD_2Cl_2, 298 K)** δ 26.5; **HRMS (ESI)**: m/z calcd. for $C_{10}H_{15}BBrN_2O_4S_2$ $[M+OC_2H_5]^-$: 380.9756, found -380.9757, error 0.491 ppm.

6-Bromo-2-(phenylsulfonyl)thieno[3,2-*d*][1,2,3]diazaborinin-1(2*H*)-ol (diazaborine 43) was prepared according to the general method B work-up 2 by refluxing for 2 h at 70 °C hydrazone **S46** (0.5 g, 1.45 mmol,

1 equiv), boron tribromide (1.11 g, 4.43 mmol, 3.06 equiv), and anhydrous iron (III) chloride (17 mg, 0.11 mmol, 0.07 equiv) in 30 ml of DCE. Obtained 356 mg (66% yield) of the title compound as a white solid. **^{1}H NMR (500 MHz, $CDCl_3$, 298 K)** δ 8.06-8.01 (2H, m, 2x Ar***H***), 7.98 (1H, s, ***H***C=N), 7.65 (1H, m, Ar***H***), 7.59-7.51 (3H, m, Het***H*** and 2x Ar***H***), 7.29 (1H, s, B-O***H***); **^{13}C NMR (126 MHz, $CDCl_3$, 298 K)** δ 145.9 (Het***C***), 137.8 (Ar***C***), 135.2 (***C***=N-N), 134.4 (Ar***C***H), 132.8 (Het***C***H), 129.3 (2x Ar***C***H), 128.5 (2x Ar***C***H), 120.1 (Het***C***-Br). *Note*: *The resonance corresponding to the quaternary carbon directly bonded to the B-OH group was not observed due to signal broadening caused by the fast relaxation induced by the quadrupolar boron nucleus as well as possible residual J-coupling to boron;* **^{11}B NMR (160 MHz, $CDCl_3$, 298 K)** δ 26.5; **HRMS (ESI)**: m/z calcd. for $C_{11}H_7BBrN_2O_3S_2$ $[M–H]^-$: 368.9181, found 368.9181, error 0.361 ppm.

6-Bromo-2-((3-nitrophenyl)sulfonyl)thieno[3,2-*d*][1,2,3]diazaborinin-1(2*H*)-ol (diazaborine 44) was prepared according to the general method B work-up 2 by refluxing for 2 h at 70 °C hydrazone **S47** (5 g, 12.81 mmol, 1 equiv), boron tribromide (9.63 g, 38 mmol, 3 equiv), and anhydrous iron (III) chloride (145 mg, 0.90 mmol, 0.07 equiv) in 260 ml of DCE. After proceeding with work up 2 and the ethyl acetate extraction. The resulting crude solid was purified by vigorous stirring in diethyl ether for 4 h, then the precipitate was filtered to afford the title compound (4.28 g, 80%) as a yellow solid. **^{1}H NMR (500 MHz, $CDCl_3$:DMSO-*d_6* 1.2:1, 298 K)** δ 9.22 (1H, br. s, B-O***H***), 8.82 (1H, m, Ar***H***), 8.42 (1H, app. d., *J* = 8.3 Hz, Ar***H***), 8.34 (1H, app. d, *J* = 7.9 Hz, Ar***H***), 8.05 (1H, s, ***H***C=N), 7.77 (1H, m, Ar***H***), 7.64 (1H, s, Het***H***); **^{13}C NMR (126 MHz, $CDCl_3$:DMSO-*d_6* 1.2:1, 298 K)** δ 147.4 (Ar***C***-NO_2), 145.1 (Het***C***), 140.2 (Ar***C***), 139.8 (br., Ar***C***-B(OH)), 134.1 (***C***=N-N), 133.4 (Ar***C***H), 132.7 (Het***C***H), 130.3 (Ar***C***H), 127.6 (Ar***C***H), 122.8 (Ar***C***H), 118.7 (Het***C***-Br); **^{11}B NMR (160 MHz, $CDCl_3$:DMSO-*d_6* 1.2:1, 298 K)** δ 25.4; **HRMS (ESI)**: m/z calcd. for $C_{13}H_{12}BBrN_3O_6S_2$ $[M+OC_2H_5]^-$: 459.9450, found 459.9447, error 0.625 ppm.

2-((3-Aminophenyl)sulfonyl)-6-bromothieno[3,2-*d*][1,2,3]diazaborinin-1(2*H*)-ol (diazaborine 45). was synthesized according to the general method D by refluxing diazaborine **S44** (1.50 g, 3.6 mmol, 1 equiv), ammonium chloride (2.9 g, 54 mmol, 15 equiv) and iron turnings (2.01 g, 36 mmol, 10 equiv) in a H_2O:MeOH mixture (1:1, 50 ml) for 2 h at 70 C. After the standard work up, the crude product was purified by stirring for 4 h in diethyl ether and filtration from the solution. Obtained 1.06 g (72% yield) of the title compound as a yellowish solid. **^{1}H NMR (500 MHz, $CDCl_3$:DMSO-*d_6* 2:1, 298 K)** δ 7.97 (1H, s, ***H***C=N), 7.79 (1H, br. s, B-O***H***), 7.52 (1H, m, Het***H***), 7.19 (1H, m, Ar***H***), 7.16-7.11 (2H, m, 2x Ar***H***), 6.80 (1H, m, Ar***H***), 4.69 (2H, br. s, ArN***H_2***); **^{13}C NMR (126 MHz, $CDCl_3$:DMSO-*d_6* 2:1, 298 K)** δ 147.9 (Ar***C***-NH_2), 145.3 (Het***C***), 139.5 (br., Ar***C***-B(OH)), 137.9 (Ar***C***), 133.9 (***C***=N-N), 132.1 (Het***C***H), 129.1 (Ar***C***H), 119.2 (Ar***C***H), 118.8 (Het***C***-Br), 115.5 (Ar***C***H), 112.5 (Ar***C***H); **^{11}B NMR (160 MHz, $CDCl_3$:DMSO-*d_6* 2:1, 298 K)** δ 25.4; **HRMS (ESI)**: m/z calcd. for $C_{13}H_{14}BBrN_3O_4S_2$ $[M+OC_2H_5]^-$: 429.9708, found 429.9710, error 0.466 ppm.

(5-Methyl-3-((2-(propylsulfonyl)hydrazineylidene)methyl)thiophen-2-yl)boronic acid (diazaborine 46). A chloroform solution (20 ml, 0.07M) of the prepared thiophene boronic acid **S57** (250 mg, 1.32 mmol, 1 equiv) and propane-1-sulfonohydrazide **S6** (192 mg, 1.32 mmol, 1 equiv) were stirred for 4 h at room temperature, and a white precipitate was formed which was then filtered and dried in high vacuum to afford the title compound (302 mg, 79% yield) as a white solid. **^{1}H NMR (500 MHz, CD_3CN:DMSO-*d_6* 10:1, 298 K)** δ 10.47 (1H, s, HC=N-N***H***), 8.19 (1H, s, ***H***C=N-NH), 7.91 (2H, br. s, 2x B-O***H***), 7.09 (1H, q, *J*=1.0 Hz, Het***H***), 3.18-3.08 (2H, m, SO_2-C***H_2***), 2.46 (3H, d, *J*=1.3 Hz, HetC***H_3***), 1.81-1.70 (2H, m, SO_2CH_2-C***H_2***), 1.00 (3H, t, *J*=7.5 Hz, $SO_2CH_2CH_2$-C***H_3***); **^{13}C NMR (126 MHz, CD_3CN:DMSO-*d_6* 10:1, 298 K)** δ 146.6 (Het***C***-CH_3), 145.7 (***C***=N-N), 142.7 (Het***C***), 130.1 (Het***C***H), 53.6 (SO_2-***C***H_2), 17.8 (SO_2CH_2-***C***H_2), 15.2 (HetC-***C***H_3), 13.1 ($SO_2CH_2CH_2$-***C***H_3). *Note*: *The resonance corresponding to the quaternary carbon directly bonded to the B-OH group was not observed due to signal broadening caused by the fast relaxation induced by the quadrupolar boron nucleus as well as possible residual J-coupling to boron*; **^{11}B NMR (160 MHz, DMSO-*d_6* , 298 K)** δ 27.8; **HRMS (ESI)**: m/z calcd. for $C_9H_{14}BN_2O_4S_2$ $[M–H]^-$: 289.0494, found 289.0494, error 0.004 ppm.

6-Methyl-2-(propylsulfonyl)thieno[2,3-*d*][1,2,3]diazaborinin-1(2*H*)-ol (diazaborine 47). The open form of the inverted thiophene diazaborine **46** (150 mg, 0.517 mmol, 1 equiv) was suspended in 50 ml of acetonitrile and evaporated in a rotavapor at 38 °C. This operation was repeated four times. The oily residue was dried in

high vacuum to afford the title compound (136 mg, 97% yield) as a pale pink solid. **^{1}H NMR (500 MHz, $CDCl_3$, 298 K)** δ 8.11 (1H, s, ***H***C=N), 7.06 (1H, q, *J*=1.1 Hz, Het***H***), 7.00 (1H, s, B-O***H***), 3.50-3.40 (2H, m, SO_2-C***H₂***), 2.64 (3H, d, *J*=1.1 Hz, HetC***H₃***), 1.87-1.75 (2H, m, SO_2CH_2-C***H₂***), 1.05 (3H, t, *J*=7.5 Hz, $SO_2CH_2CH_2$-C***H₃***); **^{13}C NMR (126 MHz, $CDCl_3$, 298 K)** δ 151.9 (Het***C***-CH_3), 144.2 (Het***C***), 138.0 (***C***=N-N), 135.7 (br., Ar***C***-B(OH)), 123.8 (Het***C***H), 53.7 (SO_2-***C***H_2), 17.0 (SO_2CH_2-***C***H_2), 15.9 (HetC-***C***H_3), 12.8 ($SO_2CH_2CH_2$-***C***H_3); **^{11}B NMR (128 MHz, $CDCl_3$, 298 K)** δ 27.3; **HRMS (ESI)**: m/z calcd. for $C_{11}H_{18}BN_2O_4S_2$ $[M+OC_2H_5]^-$: 317.0809, found 317.0809, error 0.628 ppm.

Diazaborine derivatives

6-Methylbenzo[*d*][1,2,3]diazaborinin-1(2*H*)-ol (**diazaborine 48**) was synthesized according to general procedure method B and work-up 1 by refluxing for 2 h at 100 °C hydrazone **S24** (9.0 g, 37.5 mmol, 1 equiv), boron tribromide (28.2 g, 113 mmol, 3.0 equiv), and anhydrous iron (III) chloride (424 mg, 2.61 mmol, 0.07 equiv) in 750 ml of DCE. After that, the reaction was cooled down to room temperature and slowly quenched with 50 ml of water upon stirring. ***Caution***: ***in the beginning of the quenching, a vigorous evolution of gas occurs***. The resulting organic phase was extracted with water (3x 200 ml), and the combined water extracts were basified with an aqueous solution of sodium bicarbonate which led to the formation of a white precipitate in the solution. Then, the target compound was extracted with chloroform, the combined organic extracts were dried over Na_2SO_4, and evaporated. The remaining solid residue was partitioned between 100 ml of water and 400 ml of ethyl acetate, and the layers were shaken and separated. The resulting organic phase was washed twice with water and brine, dried over Na_2SO_4, and evaporated to afford the title compound (4.5 g, 75% yield) as a white solid. **^{1}H NMR (500 MHz, CD_3OD, 298 K)** δ 7.94 (1H, s, ***H***C=N), 7.91 (1H, d, *J*=7.7 Hz, Ar***H***), 7.47 (1H, s, Ar***H***), 7.40 (1H, dd, *J*=7.8, 1.7 Hz, Ar***H***), 2.46 (3H, s, ArC***H₃***). *Note: The resonance corresponding to the B-OH group was not detected*; **^{13}C NMR (126 MHz, CD_3OD, 298 K)** δ 142.5 (Ar***C***-CH_3), 141.7 (***C***=N-N), 137.1 (Ar***C***), 131.6 (Ar***C***H), 131.5 (Ar***C***H), 129.4 (br., Ar***C***-B(OH)), 127.9 (Ar***C***H), 21.8 (ArC-***C***H_3); **^{11}B NMR (160 MHz, CD_3OD, 298 K)** δ 27.4; **HRMS (ESI)**: m/z calcd. for $C_8H_{10}BN_2O$ $[M+H]^+$: 161.0881, found 161.0881, error 0.359 ppm.

Tetracyclic diazaborine (**diazaborine 49**) was prepared according to the general method C by reacting 5-hydroxy-2-(4,4,5,5-tetramethyl-1,3,2-dioxaborolan-2-yl)benzaldehyde **S52** (200 mg, 0.81 mmol, 1 equiv) and 2-aminonicotinic acid hydrazide (123 mg, 0.81 mmol, 1 equiv) for 2 h at 80 °C. Obtained 154 mg (72% yield) of the title compound as a white solid. **^{1}H NMR (500 MHz, DMSO-*d₆*, 298 K)** δ 10.52-10.39 (2H, m, ArO***H*** and N***H***), 8.67 (1H, dd, *J*=4.7, 1.9 Hz, Ar***H***), 8.61 (1H, d, *J*=8.3 Hz, Ar***H***), 8.56 (1H, dd, *J*=7.8, 1.9 Hz, Ar***H***), 8.50 (1H, s, ***H***C=N), 7.27-7.24 (2H, m, 2x Ar***H***), 7.22 (1H, dd, *J*=8.3, 2.3 Hz, Ar***H***); **^{13}C NMR (126 MHz, DMSO-*d₆*, 298 K)** δ 163.1 (***C***=O), 161.3 (Ar***C***), 154.2 (Ar***C***-OH), 153.8 (Ar***C***H), 146.1 (***C***=N-N), 138.5 (Ar***C***H), 135.0 (Ar***C***), 134.4 (Ar***C***H), 119.6 (Ar***C***H), 119.1 (br., Ar***C***-B(OH)), 117.5 (Ar***C***H), 113.2 (Ar***C***H), 112.4 (Ar***C***); **^{11}B NMR (160 MHz, DMSO-*d₆*, 298 K)** δ 26.8; **HRMS (ESI):** m/z calcd. for $C_{13}H_{10}BN_4O_2$ $[M+H]^+$: 265.0891, found 265.0885, error 2.309 ppm.

2-(Pyridin-2-yl)benzo[*d*][1,2,3]diazaborinine-1,6(2*H*)-diol (diazaborine 50) was prepared according to the general method C by reacting 5-hydroxy-2-(4,4,5,5-tetramethyl-1,3,2-dioxaborolan-2-yl)benzaldehyde **S52** (200 mg, 0.81 mmol, 1 equiv) and 2-hydrazinylpyridine in a EtOH:H_2O solution (1:1, 6 ml) for 2 h at 80 °C. Obtained 136 mg (71% yield) of the title compound as a white solid. **^{1}H NMR (400 MHz, DMSO-*d₆*, 298 K)** δ 9.58 (1H, s, ArO***H***), 7.84 (1H, s, ***H***C=N), 7.83 (1H, m, Ar***H***), 7.70 (1H, ddd, *J*=8.9, 6.9, 1.8 Hz, Ar***H***), 7.33 (1H, d, *J*=8.0 Hz, Ar***H***), 6.97 (1H, d, *J*=2.4 Hz, Ar***H***), 6.90-6.84 (2H, m, 2x Ar***H***), 6.64 (1H, td, *J*=6.7, 1.3 Hz, Ar***H***). *Note: The resonance corresponding to the B-OH group was not detected*; **^{13}C NMR (101 MHz, DMSO-*d₆*, 298 K)** δ 157.4 (Ar***C***-OH), 156.2 (Ar***C***-N), 142.9 (***C***=N-N), 141.4 (Ar***C***H), 140.5 (Ar***C***H), 133.6 (Ar***C***), 131.8 (Ar***C***H), 130.8 (br., Ar***C***-B(OH)), 118.5 (Ar***C***H), 113.3 (Ar***C***H), 112.5 (Ar***C***H), 111.4 (Ar***C***H); **^{11}B NMR (128 MHz, DMSO-*d₆*, 298 K)** δ 1.6; **HRMS (ESI)**: m/z calcd. for $C_{12}H_{11}BN_3O_2$ $[M+H]^+$: 240.0941, found 240.0941, error 1.054 ppm.

Diethyl (1-hydroxybenzo[*d*][1,2,3]diazaborinin-2(1*H*)-yl)phosphonate (**diazaborine 51**) A EtOH:H_2O solution (1:1, 5 ml) of 2-formylphenylboronic acid (147 mg, 0.96 mmol, 1 equiv) was mixed with a EtOH:H_2O

solution (1:1, 5 ml) of phosphohydrazide **S12** (180 mg, 0.96 mmol, 1 equiv). The resulting mixture was stirred for 16 h at room temperature, then partitioned between water and ethyl acetate, and the layers were shaken and separated. The water layer was subsequently extracted with ethyl acetate (3x), and the combined organic layers were washed with brine, dried over Na_2SO_4, concentrated and dried in high vacuum to afford the title compound (256 mg, 94% yield) as a colourless oil. **^{1}H NMR (500 MHz, CDCl$_3$, 298 K)** δ 8.25-8.17 (2H, m, B-O***H*** and Ar***H***), 8.16 (1H, s, ***H***C=N), 7.73 (1H, td, *J*=7.5, 1.4 Hz, Ar***H***), 7.65 (1H, td, *J*=7.4, 1.2 Hz, Ar***H***), 7.61 (1H, d, *J*=7.7 Hz, Ar***H***), 4.33-4.23 (2H, m, POC***H$_2$***), 4.21-4.11 (2H, m, POC***H$_2$***), 1.38-1.34 (6H, m, 2x POCH$_2$C***H$_3$***); **^{13}C NMR (126 MHz, CDCl$_3$, 298 K)** δ 145.0 (***C***=N-N, d, *J*=14.4 Hz), 134.5 (Ar***C***, d, *J*=2.4 Hz), 132.5 (Ar***C***H), 132.0 (Ar***C***H, d, *J*=1.4 Hz), 130.7 (Ar***C***H), 129.7 (br., Ar***C***-B(OH)), 127.6 (Ar***C***H), 64.5 (PO-***C***H$_2$, d, *J*=5.7 Hz), 16.2 (POCH$_2$-***C***H$_3$, d, *J*=6.9 Hz); **^{31}P{^{1}H} NMR (162 MHz, CDCl$_3$, 298 K)** 4.9 (s); **^{11}B NMR (128 MHz, CDCl$_3$, 298 K)** δ 30.2; **HRMS (ESI)**: m/z calcd. for $C_{13}H_{21}BN_2O_5P$ [M+OC$_2$H$_5$]$^-$: 327.1287, found 327.1289, error 0.822 ppm.

Diphenyl (1-hydroxybenzo[*d*][1,2,3]diazaborinin-2(1*H*)-yl)phosphonate (diazaborine 52). A EtOH:H_2O solution (1:1, 100 ml) of 2-formylphenylboronic acid (3 g, 19.6 mmol, 1 equiv) was mixed with a EtOH:H_2O solution (1:1, 100 ml) of phosphohydrazide **S11** (5.2 g, 19.6 mmol, 1 equiv). The resulting mixture was stirred for 16 h at room temperature, and the precipitate formed in the solution was filtered, washed with water, and dried in high vacuum to afford the title compound (7.16 g, 97% yield) as a white solid. **^{1}H NMR (500 MHz, CDCl$_3$, 298 K)** δ 8.25 (1H, s, ***H***C=N), 8.14 (1H, dd, *J*=7.4, 1.3 Hz, Ar***H***), 7.84 (1H, br. s, B-O***H***), 7.73 (1H, td, *J*=7.6, 1.4 Hz, Ar***H***), 7.66-7.59 (2H, m, 2x Ar***H***), 7.35-7.27 (8H, m, 8x Ar***H***), 7.20-7.14 (2H, m, 2x Ar***H***); **^{13}C NMR (126 MHz, CDCl$_3$, 298 K)** δ 150.0 (2x Ar***C***, d, *J*=6.5 Hz), 145.7 (***C***=N-N, d, *J*= 15.3 Hz), 134.4 (Ar***C***, d, *J*=2.8 Hz), 132.7 (Ar***C***H), 132.1 (Ar***C***H, d, *J*=1.9 Hz), 131.0 (Ar***C***H), 130.0 (4x Ar***C***H, d, *J*=1.2 Hz), 127.8 (Ar***C***H), 125.9 (2x Ar***C***H, d, *J*=2.2 Hz), 120.7 (4x Ar***C***H, d, *J*=4.5 Hz). *Note*: *The resonance corresponding to the quaternary carbon directly bonded to the B-OH group was not observed due to signal broadening caused by the fast relaxation induced by the quadrupolar boron nucleus as well as possible residual J-coupling to boron*; **^{31}P{^{1}H} NMR (202 MHz, CDCl$_3$, 298 K)** δ -3.6; **^{11}B NMR (128 MHz, CDCl$_3$, 298 K)** δ 29.9; **HRMS (ESI)**: m/z calcd. for $C_{21}H_{21}BN_2O_5P$ [M+OC$_2$H$_5$]$^-$: 423.1287, found 423.1287, error 0.028 ppm.

Phenyl hydrogen (1-hydroxybenzo[*d*][1,2,3]diazaborinin-2(1*H*)-yl)phosphonate (diazaborine 53). Diazaborine **52** (200 mg, 0.53 mmol, 1 equiv) was dissolved in 25 ml of a 1 M NaOH aqueous solution, and the resulting reaction mixture was stirred at room temperature for 4 h. Then, the solution was acidified with aqueous HCl until reaching pH~1-2, and extracted with ethyl acetate (2x). The combined organic layers were dried over Na_2SO_4, and evaporated. The remaining crude solid was suspended in 10 ml of ether, and the solution was stirred for 2 h,the precipitate was then filtered and dried in high vacuum to afford the title compound (50 mg, 31% yield) as a white solid. *For the sake of clarity, only the signals of the major stereoisomer are reported:* **^{1}H NMR (500 MHz, DMSO-*d$_6$*:D$_2$O 1:1, 298 K)** δ 8.16 (1H, s, ***H***C=N), 7.88 (1H, d, *J*=7.5 Hz, Ar***H***), 7.73-7.65 (2H, m, 2x Ar***H***), 7.58 (1H, app. t, *J*=7.0 Hz, Ar***H***), 7.16-7.08 (2H, m, 2x Ar***H***), 6.95 (1H, m, Ar***H***), 6.93-6.88 (2H, m, 2x Ar***H***); *Note: B-OH and P-OH signals were not observed due to their fast exchange rate in protic media*; **^{13}C NMR (126 MHz, DMSO-*d$_6$*:D$_2$O 1:1, 298 K)** δ 153.0 (Ar***C***, d, *J*=7.2 Hz), 143.9 (***C***=N-N, d, *J*=13.3 Hz), 135.6 (Ar***C***), 133.8 (Ar***C***H), 132.2 (Ar***C***H), 131.9 (Ar***C***H), 130.7 (2x Ar***C***H), 130.0 (br., Ar***C***-B(OH)), 129.1 (Ar***C***H), 125.4 (Ar***C***H), 121.8 (2x Ar***C***H, d, *J*=3.9 Hz); **^{31}P{^{1}H} NMR (202 MHz, DMF-*d$_7$*, 298 K)** δ -0.9 (s); **^{11}B NMR (160 MHz, DMF-*d$_7$*, 298 K)** δ 29.6; **HRMS (ESI)**: m/z calcd. for $C_{13}H_{11}BN_2O_4P$ [M–H]$^-$: 301.0556, found 301.0554, error 0.238 ppm.

1-(1-Hydroxybenzo[*d*][1,2,3]diazaborinin-2(1*H*)-yl)ethan-1-one (diazaborine 54) was obtained according to the general method A by reacting 2-formylphenylboronic acid (300 mg, 1.96 mmol, 1 equiv) and acetylhydrazide (113 mg, 1.53 mmol, 0.8 equiv) in an EtOH:H_2O solution (1:1, 20 mL) for 90 min at room temperature. Obtained 179 mg (49% yield) of the title compound as a white solid. *Note: The B-OH signal was not observed due to fast exchange rin protic media*; **^{1}H NMR (400 MHz, DMSO-*d$_6$*, 298 K)** δ 8.14 (1H, s, ***H***C=N), 7.63 (1H, m, Ar***H***), 7.59 (1H, m, Ar***H***), 7.54-7.44 (2H, m, 2x Ar***H***), 2.39 (3H, s, C***H$_3$***); **^{13}C NMR (126 MHz, DMSO-*d$_6$*, 298 K)** δ 177.3 (***C***=O), 151.3 (***C***=N-N), 140.1 (br., Ar***C***-B(OH)), 131.3 (Ar***C***H), 130.3

(Ar***C***H), 130.3 (Ar***C***), 128.5 (Ar***C***H), 128.4 (Ar***C***H), 18.7 (***C***H_3); **^{11}B NMR (128 MHz, DMSO-*d$_6$*, 298 K)** δ 0.9; **HRMS (ESI)**: m/z calcd. for $C_9H_{10}BN_2O_2$ $[M+H]^+$: 189.0832, found 189.0833, error 1.772 ppm.

1-Hydroxybenzo[*d*][1,2,3]diazaborinine-2(1*H*)-carbothioamide (diazaborine 55) was prepared according to the general method A by reacting 2-formylphenylboronic acid (200 mg, 1.31 mmol, 1 equiv) and thiosemicarbazide (119 mg, 1.31 mmol, 1 equiv) in an EtOH:H_2O solution (1:1, 15 mL) for 19 h at room temperature. Obtained 141 mg (53% yield) of the title compound as a white solid. **^{1}H NMR (400 MHz, $CDCl_3$, 298 K)** δ 10.45 (1H, s, B-O***H***), 8.93 (1H, br. s, N***H***H), 8.28 (1H, d, *J*=7.4 Hz, Ar***H***), 7.97 (1H, s, ***H***C=N), 7.74 (1H, td, *J*=7.4, 1.5 Hz, Ar***H***), 7.68 (1H, td, *J*=7.3, 1.3 Hz, Ar***H***), 7.63 (1H, d, *J*=7.5 Hz, Ar***H***), 6.80 (1H, br. s, NH***H***); **^{13}C NMR (101 MHz, $CDCl_3$, 298 K)** δ 187.3 (***C***=S), 141.8 (***C***=N-N), 133.9 (Ar***C***), 132.9 (Ar***C***H), 132.7 (Ar***C***H), 131.2 (Ar***C***H), 127.8 (Ar***C***H). *Note*: *The resonance corresponding to the quaternary carbon directly bonded to the B-OH group was not observed due to signal broadening caused by the fast relaxation induced by the quadrupolar boron nucleus as well as possible residual J-coupling to boron*; **^{11}B NMR (128 MHz, $CDCl_3$, 298 K)** δ 30.3; **HRMS (ESI)**: m/z calcd. for $C_8H_9BN_3OS$ $[M+H]^+$: 206.0555, found 206.0554, error 0.161 ppm.

1-Hydroxybenzo[*d*][1,2,3]diazaborinine-2(1*H*)-carboximidamide (diazaborine 56). was prepared according to the general method A by reacting 2-formylphenylboronic acid (900 mg, 5.88 mmol, 1 equiv), aminoguanidine hydrochloride (650 mg, 5.88 mmol, 1 equiv) and triethylamine (0.82 ml, 5.88 mmol, 1 equiv) in an EtOH:H_2O solution (1:1, 60 mL) for 19 h at room temperature. Then, the precipitate formed in the solution was filtered rinsing with water and dried in high vacuum to afford the title compound (828 mg, 75% yield) as a white solid. **^{1}H NMR (500 MHz, DMSO-*d$_6$*, 298 K)** δ 7.56-7.48 (2H, m, ***H***C=N and Ar***H***), 7.37-7.26 (3H, m, 3x Ar***H***), 6.34 (2H, br. s, N***H$_2$***), 5.81 (1H, s, N***H***); **^{13}C NMR (126 MHz, DMSO-*d$_6$*, 298 K)** δ 156.4 (***C***(=NH)NH_2), 144.7 (br., Ar***C***-B(OH)), 140.7 (***C***=N-N), 131.6 (Ar***C***), 130.9 (Ar***C***H), 129.0 (Ar***C***H), 126.6 (Ar***C***H), 126.2 (Ar***C***H); **^{11}B NMR (160 MHz, DMSO-*d$_6$*, 298 K)** δ -3.2; **HRMS (ESI)**: m/z calcd. for $C_8H_{10}BN_4O$ $[M+H]^+$: 189.0942, found 189.0946, error 1.953 ppm.

1-Hydroxybenzo[*d*][1,2,3]diazaborinine-2(1*H*)-carboxamide (**diazaborine 57**) was prepared according to the general method A by reacting 2-formylphenylboronic acid (200 mg, 1.31 mmol, 1 equiv) and semicarbazidium hydrochloride (146 mg, 1.31 mmol, 1 equiv) in an EtOH:H_2O solution (1:1, 15 mL) for 2 h at room temperature. Obtained 203 mg (82% yield) of the title compound as a white solid. **^{1}H NMR (400 MHz, DMSO-*d$_6$*, 298 K)** δ 7.89-7.71 (3H, s, ***H***C=N and N***H$_2$***), 7.63 (1H, app.d, *J*=6.9 Hz, Ar***H***), 7.51-7.37 (3H, m, 3x Ar***H***); **^{13}C NMR (126 MHz, DMSO-*d$_6$*, 298 K)** δ 160.7 (C(=O)NH_2), 145.5 (***C***=N-N), 141.0 (br., Ar***C***-B(OH)), 131.2 (Ar***C***), 130.2 (Ar***C***H), 130.1 (Ar***C***H), 127.7 (Ar***C***H), 127.3 (Ar***C***H); **^{11}B NMR (160 MHz, DMSO-*d$_6$*, 298 K)** δ 2.5; **HRMS (ESI)**: m/z calcd. for $C_8H_8BN_3O_2Na$ $[M+Na]^+$: 212.0603, found 212.0605, error 1.550 ppm.

N*-Methyl derivative of diazaborine 11 (58)**. To a MeOH solution (16 ml, 0.15 M) of **diazaborine 11** (0.6 g, 1.90 mmol, 1 equiv) and paraformaldehyde (0.183 g, 3.81 mmol, 2 equiv) was added dropwise a 5.4 M solution of sodium methoxide in methanol (2.5 ml, 0.720 g, 13.3 mmol, 7 equiv) and the solution was allowed to stir for 4 h at room temperature. Then, $NaBH_4$ (0.14 g, 3.81 mmol, 2 equiv) was added, and the resulting reaction mixture was stirred at 50 °C for 4 h. Subsequently, the mixture was cooled down to room temperature and partitioned between water and ethyl acetate. The layers were shaken and separated, and the organic layer was washed with a saturated aqueous solution of ammonium chloride, brine, dried over Na_2SO_4, filtered and evaporated. Purification by flash column chromatography (SiO_2, EtOAc/hexanes; 1:3) provided the title compound (0.21 g, 34% yield) as a white solid. **^{1}H NMR (600 MHz, DMSO-*d$_6$*, 298 K)** δ 8.91 (1H, s, B-OH***), 8.16 (1H, s, ***H***C=N), 8.15 (1H, app. d, *J*= 7.9 Hz, Ar***H***), 7.60 (1H, s, Ar***H***), 7.57 (1H, dd, *J*=7.7, 1.6 Hz, Ar***H***), 7.34 (1H, t, *J*=7.9 Hz, Ar***H***), 7.18-7.11 (2H, m, 2x Ar***H***), 6.83 (1H, m, Ar***H***), 6.32 (1H, br. q, N***H***), 2.73 (3H, d, *J*=4.7 Hz, NH-C***H$_3$***), 2.47 (3H, s, ArC***H$_3$***); **^{13}C NMR (151 MHz, DMSO-*d$_6$*, 298 K)** δ 150.2 (Ar***C***-NHCH_3), 142.9 (Ar***C***-CH_3), 142.3 (***C***=N-N), 139.3 (Ar***C***), 134.3 (Ar***C***), 131.9 (Ar***C***H), 131.7 (Ar***C***H), 129.7 (Ar***C***H), 127.8 (Ar***C***H), 126.8 (br., Ar***C***-B(OH)), 116.0 (Ar***C***H), 113.8 (Ar***C***H), 109.8 (Ar***C***H), 29.4 (ArCNH-***C***H_3), 21.3 (ArC-***C***H_3); **^{11}B NMR (160 MHz, DMSO-*d$_6$*, 298 K)** δ 28.4; **HRMS (ESI)**: m/z calcd. for $C_{30}H_{32}B_2N_6O_6S_2Na$ $[2M+Na]^+$: 681.1906, found 681.1903, error 1.634 ppm.

N*-Acetyl derivative of diazaborine 11 (59)**. To an anhydrous DCM solution (10 ml, 0.15 M) of **diazaborine 11** (0.5 g, 1.59 mmol, 1 equiv) at 0 °C was added pyridine (0.3 g, 3.81 mmol, 2.4 equiv) and acetic anhydride (0.37 g, 3.65 mmol, 2.3 equiv). The resulting reaction mixture was stirred at room temperature for 5 h when it was quenched by adding water and extracted with DCM (3x). The resulting organic layer was washed twice with an HCl aqueous solution (0.5M), brine, dried over Na_2SO_4, filtered, evaporated and dried on vacuo to afford the title compound (0.48 g, 85% yield) as a white solid. **^{1}H NMR (400 MHz, DMSO-*d₆*, 298 K)** δ 10.30 (1H, s, NH***-$COCH_3$), 9.07 (1H, s, B-O***H***), 8.28 (1H, app. t, *J*=2.0 Hz, Ar***H***), 8.13 (1H, m, Ar***H***), 8.12 (1H, s, ***H***C=N), 7.84 (1H, ddd, *J*=8.2, 2.2, 1.1 Hz, Ar***H***), 7.64 (1H, ddd, *J*=7.9, 1.9, 1.1 Hz, Ar***H***), 7.59-7.49 (3H, m, 3x Ar***H***), 2.43 (3H, s, ArC$\boldsymbol{H_3}$), 2.05 (3H, s, NHCO-C$\boldsymbol{H_3}$); **^{13}C NMR (101 MHz, DMSO-*d₆*, 298 K)** δ 168.8 (***C***=O), 142.9 (Ar***C***-CH_3), 142.5 (***C***=N-N), 139.9 (Ar***C***-$NHCOCH_3$), 139.3 (Ar***C***), 134.3 (Ar***C***), 132.0 (Ar***C***H), 131.8 (Ar***C***H), 129.8 (Ar***C***H), 127.9 (Ar***C***H), 126.8 (br., Ar***C***-B(OH)), 123.5 (Ar***C***H), 121.8 (Ar***C***H), 117.5 (Ar***C***H), 24.0 (ArCNHCO-***C***H_3), 21.3 (ArC-***C***H_3); **^{11}B NMR (160 MHz, DMSO-*d₆*, 298 K)** δ 27.9; **HRMS (ESI)**: m/z calcd. for $C_{32}H_{32}B_2N_6O_8S_2Na$ $[2M+Na]^+$: 737.1805, found 737.1799, error 0.324 ppm.

Diazaborine salts

Potassium difluoroborate salt of diazaborine 1 (diazaborine 60). To a MeOH: H_2O solution (1:1, 45 ml, 0.05 M) of diazaborine **1** (700 mg, 2.11 mmol, 1 equiv) was added KHF_2 (495 mg, 6.34 mmol, 3 equiv) at 0 °C, and the resulting mixture was stirred at room temperature for 2 h. Then, the solution was concentrated *in vacuo*, the remaining crude solid was extracted with acetone (3x) and the remaining inorganics were filtered off. The acetone filtrate was then evaporated, and the resulting solid was stirred in 40 ml of dichloromethane for 1 h at room temperature, and then filtered to afford the title compound (752 mg, 91% yield) as a yellow solid. **^{1}H NMR (400 MHz, Acetone-*d₆*, 298 K)** δ 8.32-8.28 (2H, m, 2x Ar***H***), 8.27-8.20 (2H, m, 2x Ar***H***), 7.63 (1H, d, *J*=7.2 Hz, Ar***H***), 7.36 (1H, s, ***H***C=N), 7.30 (1H, td, *J*=7.2, 1.5 Hz, Ar***H***), 7.25 (1H, td, *J*=7.4, 1.6 Hz, Ar***H***), 7.19 (1H, d, *J*=7.5 Hz, Ar***H***); **^{13}C NMR (126 MHz, Acetone-*d₆*, 298 K)** δ 150.3 (Ar***C***-NO_2), 149.3 (Ar***C***), 140.8 (***C***=N-N), 132.3 (Ar***C***, t, *J*=3.7 Hz), 131.2 (Ar***C***H), 130.1 (2x Ar***C***H), 129.7 (Ar***C***H), 127.7 (Ar***C***H), 126.3 (Ar***C***H), 124.2 (2x Ar***C***H). *Note*: *The resonance corresponding to the quaternary carbon directly bonded to the B-OH group was not observed due to signal broadening caused by the fast relaxation induced by the quadrupolar boron nucleus as well as possible residual J-coupling to boron*; **^{19}F NMR (377 MHz, Acetone-*d₆*, 298 K)** δ -131.3 (m); **^{11}B NMR (128 MHz, Acetone-*d₆*, 298 K)** δ 3.0; HRMS (ESI): m/z calcd. for $C_{13}H_{10}BF_2KN_3O_4S$ $[M+H]^+$: 392.0087, found 392.0078, error 1.723 ppm.

Chloride salt of diazaborine 11 (61). To a dioxane solution (20 ml, 0.05M) of **diazaborine 11** (0.3 g, 0.95 mmol, 1 equiv) was added a 4M dioxane solution of HCl (0.48 ml, 1.90 mmol, 2 equiv) and the reaction mixture was stirred for 2 h at room temperature under argon. Then, a precipitate was formed which was filtered from the solution and dried in vacuo to afford the title compound (0.28 g, 84% yield) as an off-white solid. **^{1}H NMR (500 MHz, DMSO-*d₆*, 298 K)** δ 8.92 (4H, br. s, N$\boldsymbol{H_3}^+$ and B-O***H***), 8.15 (1H, d, *J*=7.7 Hz, Ar***H***), 8.12 (1H, s, ***H***C=N), 7.59-7.51 (4H, m, 4x Ar***H***), 7.48 (1H, t, *J*=7.9 Hz, Ar***H***), 7.26 (1H, d, *J*=7.8 Hz, Ar***H***), 2.43 (3H, s, ArC$\boldsymbol{H_3}$); **^{13}C NMR (101 MHz, DMSO-*d₆*, 298 K)** δ 142.9 (Ar***C***-CH_3), 142.5 (***C***=N-N), 139.6 (Ar***C***), 134.3 (Ar***C***), 132.0 (Ar***C***H), 131.8 (Ar***C***H), 130.1 (Ar***C***H), 127.9 (Ar***C***H), 126.9 (Ar***C***-$NH_3^+Cl^-$), 122.2 (Ar***C***H), 118.9 (Ar***C***H), 115.6 (Ar***C***H), 21.3 (ArC-***C***H_3). *Note*: *The resonance corresponding to the quaternary carbon directly bonded to the B-OH group was not observed due to signal broadening caused by the fast relaxation induced by the quadrupolar boron nucleus as well as possible residual J-coupling to boron*; **^{11}B NMR (160 MHz, DMSO-*d₆*, 298 K)** δ 28.3; **HRMS (ESI)**: m/z calcd. for $C_{14}H_{15}BN_3O_3S$ $[M]^+$: 316.0922, found 316.0924, error 0.579 ppm.

Sodium salt of diazaborine 11 (diazaborine 62). **Diazaborine 11** (2.5 g, 7.93 mmol, 1 equiv) was suspended in a NaOH aqueous solution (0.32 g, 7.93 mmol NaOH in 25 ml of water). The resulting solution was stirred for 2 h at room temperature. Then, the solid was fully dissolved in solution and the water layer was washed with DCM (3x 50 ml), the aqueous layer was separated, evaporated, and dried on vacuo to afford the title compound (2.77 g, 98% yield) as a white solid. **^{1}H NMR (600 MHz, DMSO-*d₆*, 298 K)** δ 7.45 (1H, d, *J*=7.5 Hz, Ar***H***), 7.17 (1H, t, *J*=2.0 Hz, Ar***H***), 7.11 (1H, m, Ar***H***), 7.09 (1H, s, ***H***C=N), 7.06-6.99 (2H, m, 2x Ar***H***), 6.85 (1H, s, Ar***H***), 6.63 (1H, dd, *J*=7.9, 1.3 Hz, Ar***H***), 5.24 (2H, s, ArN$\boldsymbol{H_2}$), 2.37 (2H, s, 2x B-O***H***), 2.25 (3H,

s, Ar*C**H*$_3$); **^{13}C NMR (151 MHz, DMSO-*d*$_6$, 298 K)** δ 148.3 (Ar***C***-NH$_2$), 146.5 (br., Ar***C***-B(OH)$_2$), 143.8 (Ar***C***), 137.4 (***C***=N-N), 133.9 (Ar***C***-CH$_3$), 131.3 (Ar***C***H), 131.0 (Ar***C***), 128.8 (Ar***C***H), 128.4 (Ar***C***H), 124.6 (Ar***C***H), 116.0 (Ar***C***H), 114.8 (Ar***C***H), 112.9 (Ar***C***H), 20.8 (ArC-***C***H$_3$); **^{11}B NMR (128 MHz, DMSO-*d*$_6$, 298 K)** δ 2.2; **HRMS (ESI)**: m/z calcd. for anion $[C_{16}H_{19}BN_3O_4S]^-$ $[M–2OH+2OCH_3]^-$: 360.1196, found 360.1192, error 0.770 ppm.

Potassium salt of diazaborine 11 (diazaborine 63). **Diazaborine 11** (0.2 g, 0.63 mmol, 1 equiv) was suspended in a KOH aqueous solution (36 mg, 0.63 mmol KOH in 10 ml of water). The resulting solution was stirred for 2 h at room temperature. Then, the solid was fully dissolved in solution and the water layer was washed with DCM (3x 50 ml), the aqueous layer was separated, evaporated, and dried on vacuo to afford the title compound (218 mg, 93% yield) as a white solid. **^{1}H NMR (600 MHz, DMSO-*d*$_6$, 298 K)** δ 7.44 (1H, d, *J*=7.5 Hz, Ar***H***), 7.17 (1H, t, *J*=2.0 Hz, Ar***H***), 7.11 (1H, dt, *J*=7.7, 1.3 Hz, Ar***H***), 7.09 (1H, s, ***H***C=N), 7.03 (1H, t, *J*=7.8 Hz, Ar***H***), 7.00 (1H, dd, *J*=7.5, 1.7 Hz, Ar***H***), 6.83 (1H, s, Ar***H***), 6.62 (1H, ddd, *J*=7.9, 2.4, 1.1 Hz, Ar***H***), 5.24 (2H, s, ArN***H***$_2$), 2.24 (3H, s, ArC***H***$_3$), 2.02 (2H, br. s, 2x B-O***H***); **^{13}C NMR (151 MHz, DMSO-*d*$_6$, 298 K)** δ 148.3 (Ar***C***-NH$_2$), 146.7 (br., Ar***C***-B(OH)), 143.9 (Ar***C***), 137.6 (***C***=N-N), 133.6 (Ar***C***-CH$_3$), 131.1 (Ar***C***H), 131.0 (Ar***C***), 128.7 (Ar***C***H), 128.3 (Ar***C***H), 124.6 (Ar***C***H), 116.0 (Ar***C***H), 114.8 (Ar***C***H), 112.8 (Ar***C***H), 20.8 (ArC-***C***H$_3$); **^{11}B NMR (128 MHz, DMSO-*d*$_6$, 298 K)** δ 2.7; **HRMS (ESI)**: m/z calcd. for anion $[C_{14}H_{15}BN_3O_4S]^-$ $[M]^-$: 332.0884, found 332.0885, error 0.918 ppm.

Sodium salt of diazaborine 13 (diazaborine 64). **Diazaborine 13** (0.3 g, 1.0 mmol, 1 equiv) was suspended in a NaOH aqueous solution (40 mg, 1.0 mmol NaOH in 7 ml of water). The resulting solution was stirred for 2 h at room temperature. Then, the solid was fully dissolved in solution and the water layer was washed with DCM (3x 50 ml), the aqueous layer was separated, evaporated, and dried on vacuo to afford the title compound (328 mg, 97% yield) as a white solid. **^{1}H NMR (400 MHz, D$_2$O, 298 K)** δ 8.03-7.96 (2H, m, 2x Ar***H***), 7.52-7.39 (4H, m, 4x Ar***H***), 7.11 (1H, s, ***H***C=N), 7.02 (1H, dd, *J*=7.6, 1.7 Hz, Ar***H***), 6.84 (1H, s, Ar***H***), 2.45 (2H, s, 2x B-O***H***), 2.24 (3H, s, ArC***H***$_3$); **^{13}C NMR (101 MHz, D$_2$O, 298 K)** δ 146.5 (br., Ar***C***-B(OH)), 143.3 (Ar***C***), 138.0 (***C***=N-N), 133.9 (Ar***C***-CH$_3$), 131.3 (Ar***C***H), 130.9 (Ar***C***), 130.9 (Ar***C***H), 128.9 (Ar***C***H), 128.0 (2x Ar***C***H), 127.6 (2x Ar***C***H), 124.8 (Ar***C***H), 20.8 (ArC-***C***H$_3$); **^{11}B NMR (128 MHz, D$_2$O, 298 K)** δ 2.8; **HRMS (ESI)**: m/z calcd. for anion $[C_{14}H_{14}BN_2O_4S]^-$ $[M]^-$: 317.0773, found 317.0771, error 0.479 ppm.

Sodium salt of diazaborine 41 (diazaborine 65). **Diazaborine 41** (0.2 g, 0.73 mmol, 1 equiv) was suspended in a NaOH aqueous solution (29 mg, 0.73 mmol of NaOH in 5 ml of water). The resulting solution was stirred for 2 h at room temperature. Then, the solid was fully dissolved in solution and the water layer was washed with DCM (3x 50 ml), the aqueous layer was separated, evaporated, and dried on vacuo to afford the title compound (220 mg, 96% yield) as a brownish solid. **^{1}H NMR (400 MHz, D$_2$O, 298 K)** δ 7.43 (1H, s, ***H***C=N), 6.94 (1H, m, Het***H***), 3.31-3.21 (2H, m, SO$_2$-C***H***$_2$), 2.53 (3H, d, *J*=1.1 Hz, HetC***H***$_3$), 1.89-1.73 (2H, m, SO$_2$CH$_2$-C***H***$_2$), 1.02 (3H, t, *J*=7.5 Hz, SO$_2$CH$_2$CH$_2$-C***H***$_3$); **^{13}C NMR (101 MHz, D$_2$O, 298 K)** δ 152.9 (br., Ar***C***-B(OH)), 144.4 (Het***C***-CH$_3$), 133.9 (***C***=N-N), 131.2 (Het***C***), 128.8 (Het***C***H), 54.0 (SO$_2$-***C***H$_2$), 16.5 (SO$_2$CH$_2$-***C***H$_2$), 14.4 (HetC-***C***H$_3$), 12.3 (SO$_2$CH$_2$CH$_2$-***C***H$_3$); **^{11}B NMR (128 MHz, D$_2$O, 298 K)** δ 2.2; **HRMS (ESI)**: m/z calcd. for anion $[C_9H_{14}BN_2O_4S_2]^-$ $[M]^-$: 289.0494, found 289.0492, error 0.479 ppm.

Sodium salt of diazaborine 47 (diazaborine 66). **Diazaborine 47** (1.5 g, 5.54 mmol, 1 equiv) was suspended in a NaOH aqueous solution (221 mg, 5.54 mmol of NaOH in 40 ml of water). The resulting solution was stirred for 2 h at room temperature. Then, the solid was fully dissolved in solution and the water layer was washed with DCM (3x 50 ml), the aqueous layer was separated, evaporated, and dried on vacuo to afford the title compound (1.69 g, 98% yield) as a white solid. **^{1}H NMR (600 MHz, D$_2$O, 298 K)** δ 7.42 (1H, s, ***H***C=N-NH), 6.84 (1H, s, Het***H***), 3.30-3.23 (2H, m, SO$_2$-C***H***$_2$), 2.49 (3H, s, HetC***H***$_3$), 1.87-1.76 (2H, m, SO$_2$CH$_2$-C***H***$_2$), 1.02 (3H, t, *J*=7.5 Hz, SO$_2$CH$_2$CH$_2$-C***H***$_3$); **^{13}C NMR (101 MHz, D$_2$O, 298 K)** δ 150.2 (br., Ar***C***-B(OH)), 142.3 (Het***C***-CH$_3$), 135.6 (***C***=N-N), 134.1 (Het***C***), 123.0 (Het***C***H), 54.0 (SO$_2$-***C***H$_2$), 16.5 (SO$_2$CH$_2$-***C***H$_2$), 14.1 (HetC-***C***H$_3$), 12.3 (SO$_2$CH$_2$CH$_2$-***C***H$_3$); **^{11}B NMR (128 MHz, D$_2$O, 298 K)** δ 2.1; **HRMS (ESI):** m/z calcd. for anion $[C_9H_{14}BN_2O_4S_2]^-$ $[M]^-$: 289.0483, found 289.0488, error 0.697 ppm.

Conjugates of diazaborine 11 and phosphonium salts

Conjugate 67. To a DCM solution (27 ml) of **diazaborine 11** (1.6 g, 5.08 mmol, 1 equiv) was added EDC (2.43 g, 12.7 mmol, 2.5 equiv), DMAP (0.62 g, 5.08 mmol, 1 equiv) and (6-carboxyhexyl)triphenylphosphonium bromide **S59** (2.55 g, 5.58 mmol, 1.1 equiv), and the resulting reaction mixture was stirred for 48 h at room temperature. Then, an aqueous HBr solution (0.5 M, 20 ml) was added to quench the mixture, which was additionally stirred for 5 min. Subsequently, the solution was extracted with DCM (3x), and the resulting organic layer was dried over Na_2SO_4, filtered and evaporated. The remaining solid was redissolved in DCM and the product was precipitated by slowly adding a diethyl-ether-hexanes solution (1:3), filtered rinsing with diethyl-ether-hexanes solution (1:3), and dried in high vacuum to afford the title compound (3.42 g, 89%) as a white solid. **^{1}H NMR (500 MHz, $CDCl_3$, 298 K)** δ 10.63 (1H, s, N***H***), 8.83 (1H, t, *J*=2.0 Hz, Ar***H***), 8.04 (1H, app. d, *J*=8.2 Hz, Ar***H***), 8.00 (1H, d, *J*=7.7 Hz, Ar***H***), 7.89 (1H, s, ***H***C=N-NH), 7.76-7.67 (9H, m, 9x Ar***H***), 7.66-7.59 (7H, m, 7x Ar***H***), 7.39 (1H, dd, *J*=7.7, 1.0 Hz, Ar***H***), 7.35-7.26 (3H, m, B-O***H*** and 2x Ar***H***), 3.52-3.39 (2H, m, C***H₂***), 2.54 (2H, app. t, *J*=7.3 Hz, C***H₂***), 2.42 (3H, s, ArC***H₃***), 1.85-1.76 (2H, m, C***H₂***), 1.76-1.58 (4H, m, 2x C***H₂***); **^{13}C NMR (126 MHz, $CDCl_3$, 298 K)** δ 173.1 (NH***C***=O), 143.7 (***C***=N-N), 143.3 (Ar***C***-CH₃), 140.6 (Ar***C***), 137.8 (Ar***C***), 135.3 (3x Ar***C***H, d, *J*=3.0 Hz), 134.5 (Ar***C***), 133.6 (6x Ar***C***H, d, *J*=10.0 Hz), 132.2 (Ar***C***H), 132.0 (Ar***C***H), 130.6 (6x Ar***C***H, d, *J*=12.5 Hz), 129.0 (Ar***C***H), 127.8 (Ar***C***H), 127.1 (br., Ar***C***-B(OH)), 125.5 (Ar***C***H), 122.8 (Ar***C***H), 118.8 (Ar***C***H), 118.0 (3x Ar***C***, d, *J*=86.0 Hz), 36.9 (***C***H₂), 29.9 (***C***H₂, d, *J*=15.7 Hz), 24.4 (***C***H₂), 22.8 (***C***H₂, d, *J*=50.9 Hz), 21.9 (ArC-***C***H₃), 21.6 (***C***H₂, d, *J*=4.2 Hz); **^{11}B NMR (160 MHz, $CDCl_3$, 298 K)** δ 29.9; **^{31}P{^{1}H} (202 MHz, $CDCl_3$, 298 K)** δ 24.2; **HRMS (ESI)**: m/z calcd. for cation $[C_{38}H_{38}BN_3O_4PS]^+$ $[M]^+$: 674.2410, found 674.2414, error 0.841 ppm.

Diazaborine conjugate 68. To a dry 1,4-dioxane solution (40 ml) of **diazaborine 11** (0.8 g, 2.54 mmol, 1 equiv) was added triphosgene (1.88 g, 6.35 mmol, 2.5 equiv), and the resulting reaction mixture was refluxed for 3 h. Then, the reaction was allowed to cool down to room temperature, and the solvent was evaporated (***CAUTION****: This operation is recommended to be carried out under high vacuum with a trap directly connected to the reaction vessel due to the toxicity of triphosgene*). The remaining solid was redissolved in dry acetonitrile (40 ml), the phosphonium alcohol bromide **S58** (1.12 g, 2.54 mmol, 1 equiv), and the resulting solution was stirred at room temperature for 48 h. Then, the solvent was evaporated in the trap, and the remaining crude solid was suspended in 30 ml of ethyl acetate and stirred for 4 h with occasional scratching of the walls of the flask to induce precipitation. The formed precipitated was filtered from the solution, dried, and evaporated three times with $CDCl_3$ to remove the traces of ethyl acetate to afford the title compound (1.50 g, 68% yield) as an orange solid with ca. 90% purity. **^{1}H NMR (500 MHz, $CDCl_3$, 298 K)** δ 9.65 (1H, br. s, N***H***), 8.55 (1H, s, Ar***H***), 8.07-7.96 (2H, m, 2x Ar***H***), 7.91 (1H, s, ***H***C=N-NH), 7.82-7.69 (9H, m, 9x Ar***H***), 7.69-7.59 (6H, m, 6x Ar***H***), 7.57 (1H, app. d, *J*=7.7 Hz, Ar***H***), 7.40 (1H, app. d, *J*=7.7 Hz, Ar***H***), 7.33-7.27 (2H, m, 2x Ar***H***), 4.13-4.02 (2H, m, C***H₂***), 3.76-3.66 (2H, m, C***H₂***), 2.42 (3H, s, ArC***H₃***), 1.76-1.65 (2H, m, C***H₂***), 1.65-1.48 (6H, m, 3x C***H₂***). *Note: The resonance corresponding to the B-OH group was not detected.* **^{13}C (101 MHz, $CDCl_3$, 298 K)** δ 154.2 (NH***C***(=O)OR), 143.7 (***C***=N-N), 143.3 (Ar***C***-CH₃), 140.7 (Ar***C***), 137.9 (Ar***C***), 135.1 (3x Ar***C***H, d, *J*=2.9 Hz), 134.5 (Ar***C***), 133.7 (6x Ar***C***H, d, *J*=9.9 Hz), 132.2 (Ar***C***H), 132.0 (Ar***C***H), 130.5 (6x Ar***C***H, d, *J*=12.6 Hz), 129.2 (Ar***C***H), 127.8 (Ar***C***H), 127.0 (br., Ar***C***-B(OH)), 124.7 (Ar***C***H), 121.9 (Ar***C***H), 118.3 (3x Ar***C***, d, *J*=86.0 Hz), 117.4 (Ar***C***H), 65.0 (***C***H₂), 29.9 (***C***H₂, d, *J*=16.4 Hz), 28.1 (***C***H₂), 26.0 (***C***H₂), 22.6 (***C***H₂, d, *J*=50.1 Hz), 22.5 (***C***H₂, d, *J*=4.4 Hz), 21.9 (ArC-***C***H₃); **^{11}B (128 MHz, $CDCl_3$, 298 K)** δ 27.5; **^{31}P{^{1}H} (202 MHz, $CDCl_3$, 298 K)** δ 24.4; **HRMS (ESI)**: m/z calcd. for cation $[C_{39}H_{40}BN_3O_5PS]^+$ $[M]^+$: 704.2516, found 704.2512, error 0.272 ppm.

Conjugates of ***diazaborine 11*** *and tryptophan*

Diazaborine conjugate 69 (diazaborine 11 and *D*-Tryptophan). A solution of **Diazaborine 11** (2.0 g, 6.35 mmol, 1 equiv) and *D*-Tryptophan (2.59 g, 12.69 mmol, 2 equiv) in dry acetonitrile (120 ml) was refluxed for 48 h. Then, it was allowed cool down to room temperature and the unreacted D-tryptophan was filtered off. The filtrate solution was evaporated and the remaining crude solid was redissolved in 50 ml of CH_2Cl_2. Then, diethyl ether (150 ml) was added, and the resulting suspension was stirred overnight. A precipitate formed which was filtered from the solution, and dried on vacuo to afford the title compound (1.32 g, 42% yield) as

a white solid. **^{1}H NMR (600 MHz, DMSO-d_6, 298 K)** δ 11.07 (1H, d, *J*=1.9 Hz, N***H*** *indole*), 7.88 (1H, app. dd, *J*=13.3, 8.5 Hz, CH-N***H***H), 7.62 (1H, d, *J*=7.9 Hz, Ar***H***), 7.47 (1H, s, ***H***C=N-NH), 7.45 (1H, d, *J*=8.1 Hz, Ar***H***), 7.42 (1H, d, *J*=2.5 Hz, C***H***=C *indole*), 7.17-7.08 (3H, m, 3x Ar***H***), 7.07 (1H, t, *J*=2.1 Hz, Ar***H***), 7.06-6.99 (2H, m, 2x Ar***H***), 6.96 (1H, ddd, *J*=8.0, 2.3, 1.0 Hz, Ar***H***), 6.70 (1H, ddd, *J*=8.0, 2.3, 1.0 Hz, Ar***H***), 6.42 (1H, d, *J*=7.6 Hz, Ar***H***), 6.05 (1H, app. dd, *J*=13.7, 6.2 Hz, CH-N***H***H), 5.47 (2H, s, ArN$\boldsymbol{H_2}$), 4.35 (1H, m, CH_2-C***H***-NH_2), 3.45-3.35 (2H, m, C$\boldsymbol{H_2}$-CH-NH_2), 2.28 (3H, s, ArC$\boldsymbol{H_3}$); **^{13}C NMR (151 MHz, DMSO-d_6, 298 K)** δ 174.0 (***C***OO), 148.8 (Ar***C***-NH_2), 141.2 (Ar***C***), 139.3 (***C***=N-N), 137.7 (Ar***C***-CH_3), 136.4 (Ar***C***), 133.3 (br., Ar***C***-B(OH)), 131.0 (Ar***C***H), 130.5 (Ar***C***H), 130.4 (Ar***C***), 129.1 (Ar***C***H), 126.9 (Ar***C***), 126.0 (Ar***C***H), 125.1 (***C***H=C *indole*), 121.4 (Ar***C***H), 118.8 (Ar***C***H), 118.3 (Ar***C***H), 117.2 (Ar***C***H), 113.9 (Ar***C***H), 112.4 (Ar***C***H), 111.5 (Ar***C***H), 108.3 (CH=***C*** *indole*), 55.7 (CH_2-***C***H-NH_2), 26.3 (***C***H_2-CH-NH_2), 20.8 (ArC-***C***H_3); **^{11}B NMR (128 MHz, DMSO-d_6, 298 K)** δ 2.2; **HRMS (ESI)**: m/z calcd. for $C_{25}H_{23}BN_5O_4S$ [M–H]$^-$: 500.1574, found 500.1573, error 0.684 ppm.

Diazaborine conjugate 70 (diazaborine 11 and *L*-Tryptophan).The title conjugate was prepared *via* the same procedure used to synthesize **conjugate 69** by using **diazaborine 11** (1.96 g, 6.23 mmol, 1 equiv) and *L*-tryptophan (2.55 g, 12.46 mmol, 2 equiv). Obtained 2.11 g (67% yield) as a white solid. **^{1}H NMR (500 MHz, DMSO-d_6, 298 K)** δ 11.07 (1H, d, *J*=2.5 Hz, N***H*** *indole*), 7.88 (1H, app. dd, *J*=13.4, 8.6 Hz, CH-N***H***H), 7.62 (1H, d, *J*=7.9 Hz, Ar***H***), 7.47 (1H, s, ***H***C=N-NH), 7.45 (1H, d, *J*=8.1 Hz, Ar***H***), 7.42 (1H, d, *J*=2.4 Hz, C***H***=C *indole*), 7.16-7-08 (3H, m, 3x Ar***H***), 7.07 (1H, t, *J*=2.0 Hz, Ar***H***), 7.06-7.00 (2H, m, 2x Ar***H***), 6.96 (1H, d, *J*=8.2 Hz, Ar***H***), 6.70 (1H, ddd, *J*=8.0, 2.4, 1.0 Hz, Ar***H***), 6.42 (1H, d, *J*=7.6 Hz, Ar***H***), 6.05 (1H, app. dd, *J*=13.7, 6.2 Hz, CH-N***H***H), 5.47 (2H, br. s, ArN$\boldsymbol{H_2}$), 4.35 (1H, m, CH_2-C***H***-NH_2), 3.46-3.35 (2H, m, C$\boldsymbol{H_2}$-CH-NH_2), 2.28 (3H, s, ArC$\boldsymbol{H_3}$); **^{13}C NMR (126 MHz, DMSO-d_6, 298 K)** δ 174.0 (***C***OO), 148.8 (Ar***C***-NH_2), 141.2 (Ar***C***), 139.3 (***C***=N-N), 137.7 (Ar***C***-CH_3), 136.4 (Ar***C***), 133.3 (br., Ar***C***-B(OH)), 130.9 (Ar***C***H), 130.5 (Ar***C***H), 130.4 (Ar***C***), 129.1 (Ar***C***H), 126.9 (Ar***C***), 126.0 (Ar***C***H), 125.1 (***C***H=C *indole*), 121.4 (Ar***C***H), 118.8 (Ar***C***H), 118.3 (Ar***C***H), 117.2 (Ar***C***H), 113.9 (Ar***C***H), 112.4 (Ar***C***H), 111.5 (Ar***C***H), 108.3 (CH=***C*** *indole*), 55.7 (CH_2-***C***H-NH_2), 26.3 (***C***H_2-CH-NH_2), 20.8 (ArC-***C***H_3); **^{11}B NMR (160 MHz, DMSO-d_6, 298 K)** δ 2.6; **HRMS (ESI)**: m/z calcd. for $C_{25}H_{25}BN_5O_4S$ [M+H]$^+$: 502.1716, found 502.1715, error 0.106 ppm.

Conjugates of diazaborine 11 and aminoacids with phosphonium salts connected by an amide linker

Conjugate 71 (diazaborine 11-amide linker-phosphonium salt and *D*-Tryptophan). A dry EtOH solution (8 ml) of **conjugate 67** (102 mg, 0.14 mmol, 1 equiv) and *D*-Tryptophan (55 mg, 0.27 mmol, 2 equiv) was refluxed under argon for 24 h. After cooling down the reaction mixture to room temperature, the ethanol was evaporated, and the remaining solid was suspended in DCM. The resulting turbid solution was filtered rinsing with DCM to remove the unreacted aminoacid. The filtrate was evaporated and dried under vacuo to afford the title compound (94 mg, 74% yield) as a diastereomeric mixture (dr 6:1). Off-white solid. *For clarity only the signals corresponding to the major diastereomer are reported*: **^{1}H NMR (600 MHz, DMSO-d_6, 298 K)** δ 11.07 (1H, d, *J*=2.4 Hz), 10.16 (1H, br s), 8.10 (1H, app. t, *J*=2.0 Hz), 7.94 (1H, dd, *J*=13.6, 8.6 Hz), 7.91-7.85 (3H, m), 7.82-7.72 (13H, m), 7.60 (1H, app. dd, *J*=8.0, 1.1 Hz), 7.49-7.46 (2H, m), 7.45-7.42 (2H, m), 7.40 (1H, m), 7.12 (1H, m), 7.09 (1H, m), 7.03 (2H, m), 6.43 (1H, d, *J*=7.6 Hz), 6.12 (1H, dd, *J*=13.6, 6.2 Hz), 4.34 (1H, m), 3.63-3.52 (2H, m), 3.43-3.34 (2H, m), 2.33-2.24 (5H, m), 1.67-1.43 (6H, m); **^{13}C NMR (151 MHz, DMSO-d_6, 298 K)** δ 174.0, 171.4, 141.1, 139.9, 139.3, 137.7, 136.4, 134.8 (3, d, *J*=3.0 Hz), 133.6 (6, d, *J*=10.3 Hz), 131.0, 130.7, 130.2 (6, d, *J*=12.2 Hz), 130.2, 129.2, 126.8, 126.2, 125.1, 122.5, 121.4, 121.3, 118.7, 118.5 (3, d, *J*=85.6 Hz), 118.3, 118.1, 111.5, 108.3, 55.6, 35.9, 29.5 (d, *J*=16.6 Hz), 26.3, 24.2, 21.7 (d, *J*=4.4 Hz), 20.7, 20.2 (d, *J*=49.9 Hz). *Note*: *The resonance corresponding to the quaternary carbon directly bonded to the B-OH group was not observed due to signal broadening caused by the fast relaxation induced by the quadrupolar boron nucleus as well as possible residual J-coupling to boron*. **^{11}B (128 MHz, DMSO-d_6, 298 K)** δ 1.7; **^{31}P{^{1}H} NMR (243 MHz, DMSO-d_6, 298 K)** δ 24.0; **HRMS (ESI)**: m/z calcd. for cation [$C_{49}H_{48}BN_5O_5PS$]$^+$ [M]$^+$: 860.3204, found 860.3204, error 0.286 ppm.

Conjugate 72 (diazaborine 11-amide linker-phosphonium salt and *L*-Tryptophan). A dry MeCN solution (8 ml) of **conjugate 67** (200 mg, 0.27 mmol, 1 equiv) and *L*-Tryptophan (111 mg, 0.53 mmol, 2 equiv) was refluxed under argon for 48 h. After cooling down the reaction mixture to room temperature, the turbid solution

was filtered to remove the unreacted aminoacid. The filtrate was evaporated and the remaining solid was redissolved in 5 ml of DCM and ca. 20 ml of Et_2O were added to induce precipitation of the product. The resulting solution was stirred for 4 h, and the precipitate was filtered from the solution and dried in vacuo to afford the title compound (172 mg, 69% yield) as a diastereomeric mixture (dr: *ca.* 2.2 to 1). Brown solid. The major and minor diastereomers are indicated as *a* and *b*, respectively. **Diastereomer *A*: ^{1}H NMR (500 MHz, DMSO-*d₆*, 298 K)** δ 11.10 (1H, d, *J*=2.5 Hz), 10.20 (1H, s), 8.12 (1H, t, *J*=2.0 Hz), 7.96 (1H, dd, *J*=13.6, 8.8 Hz), 7.92-7.85 (3H, m), 7.83-7.71 (13H, m), 7.59 (1H, m), 7.50-7.40 (5H, m), 7.16-7.08 (2H, m), 7.06-7.00 (2H, m), 6.43 (1H, d, *J*=7.6 Hz), 6.13 (1H, dd, *J*=13.5, 6.2 Hz), 4.34 (1H, m), 3.65-3.52 (2H, m), 3.44-3.36 (2H, m), 2.37-2.20 (5H, m), 1.69-1.42 (6H, m); **^{13}C NMR (126 MHz, DMSO-*d₆*, 298 K)** δ 173.9, 171.3, 141.1, 139.8, 139.3, 137.7, 136.4, 134.8 (3, d, *J*=3.0 Hz), 133.5 (6, d, *J*=10.0 Hz), 131.0, 130.6, 130.2 (6, d, *J*=12.4 Hz), 130.2, 129.2, 126.8, 126.1, 125.1, 122.5, 121.3, 121.3, 118.7, 118.5 (3, d, *J*=85.5 Hz), 118.3, 118.1, 111.5, 108.3, 55.6, 35.9, 29.4 (d, *J*=16.8 Hz), 26.3, 24.1, 21.7 (d, *J*=4.5 Hz), 20.7, 20.2 (d, *J*=49.9 Hz). *Note*: *The resonance corresponding to the quaternary carbon directly bonded to the B-OH group was not observed due to signal broadening caused by the fast relaxation induced by the quadrupolar boron nucleus as well as possible residual J-coupling to boron*. **Diastereomer *B*: ^{1}H NMR (500 MHz, DMSO-*d₆*, 298 K)** δ 10.95 (1H, d, *J*=2.5 Hz), 10.23 (1H, s), 8.19 (1H, t, *J*=2.0 Hz), 7.92-7.85 (4H, m), 7.83-7.73 (13H, m), 7.62-7.56 (2H, m), 7.51 (1H, s), 7.50-7.40 (3H, m), 7.38 (1H, d, *J*=8.1 Hz), 7.23 (1H, d, *J*=7.5 Hz), 7.16-7.08 (2H, m), 7.03 (1H, m), 6.58 (1H, dd, *J*=13.2, 7.5 Hz), 4.40 (1H, m), 3.65-3.52 (3H, m), 3.38 (1H, m), 2.37-2.20 (5H, m), 1.69-1.42 (6H, m); ^{13}C (126 MHz, DMSO-*d₆*, 298 K) δ 172.9, 171.4, 141.0, 140.1, 139.2, 137.8, 136.2, 134.8 (3, d, *J*=3.0 Hz), 133.5 (6, d, *J*=10.0 Hz), 131.6, 130.7, 130.2 (6, d, *J*=12.4 Hz), 130.0, 129.2, 127.0, 126.3, 124.2, 122.6, 121.5, 121.0, 118.5 (3, d, *J*=85.5 Hz), 118.4, 118.1, 118.0, 111.3, 109.7, 56.0, 35.9, 29.4 (d, *J*=16.8 Hz), 26.4, 24.1, 21.7 (d, *J*=4.5 Hz), 20.8, 20.2 (d, *J*=49.9 Hz). *Note*: *The resonance corresponding to the quaternary carbon directly bonded to the B-OH group was not observed due to signal broadening caused by the fast relaxation induced by the quadrupolar boron nucleus as well as possible residual J-coupling to boron*. **Both diastereomers: ^{11}B NMR (128 MHz, DMSO-*d₆*, 298 K)** δ 1.7; **^{31}P {^{1}H} NMR (162 MHz, DMSO-*d₆*, 298 K)** δ 24.0; **HRMS (ESI)**: m/z calcd. for cation $[C_{49}H_{48}BN_5O_5PS]^+$ $[M]^+$:860.3204, found 860.3200, error 0.102 ppm.

Conjugate 73 (diazaborine 11-amide linker-phosphonium salt and *D*-Leucine). The title conjugate was prepared according to the same procedure followed for the synthesis of **conjugate 72** by using **conjugate 67** (200 mg, 0.26 mmol, 1 equiv) and *D*-Leucine (71 mg, 0.53 mmol, 2 equiv). After filtering off the excess of aminoacid and precipitating the product in a DCM:Et_2O solution, it was evaporated three times with $CDCl_3$ to remove the traces of diethyl ether. Obtained 164 mg (71% yield, dr: *ca.* 1.7 to 1) as a brown solid. The major and minor diastereomers are indicated as *a* and *b*, respectively (when it was possible to distinguish their signals). **Diastereomer *A*: ^{1}H NMR (500 MHz, DMSO-*d₆*, 298 K)** δ 10.19 (1H, s), 8.15 (1H, s), 7.91-7.85 (3H, m), 7.83-7.72 (13H, m), 7.68 (1H, m), 7.54 (1H, d, *J*=8.0 Hz), 7.52 (1H, m), 7.46-7.41 (2H, m), 7.30 (1H, m), 7.15 (1H, s), 6.49 (1H, dd, *J*=13.1, 7.7 Hz), 4.04 (1H, m), 3.67-3.52 (2H, m), 2.36-2.25 (5H, m), 1.96-1.83 (2H, m), 1.77 (1H, m), 1.66-1.45 (6H, m), 1.01-0.89 (6H, m); **^{13}C (101 MHz, DMSO-*d₆*, 298 K)** δ 173.6, 171.4, 141.1, 140.0, 139.3, 137.8, 134.9 (3, d, *J*=3.1 Hz), 133.6 (6, d, *J*=10.0 Hz), 131.6, 130.8, 130.2 (6, d, *J*=12.5 Hz), 130.0, 129.1, 126.3, 122.5, 121.5, 118.5 (3, d, *J*=85.6 Hz), 118.3, 53.8, 39.7, 35.9, 29.5 (d, *J*=16.9 Hz), 24.2, 24.2, 23.2, 21.7 (d, *J*=4.4 Hz), 21.1, 20.8, 20.2 (d, *J*=50.0 Hz). *Note*: *The resonance corresponding to the quaternary carbon directly bonded to the B-OH group was not observed due to signal broadening caused by the fast relaxation induced by the quadrupolar boron nucleus as well as possible residual J-coupling to boron*. **Diastereomer *B*: ^{1}H NMR (500 MHz, DMSO-*d₆*, 298 K)** δ 10.19 (1H, s), 8.15 (1H, s), 7.91-7.85 (3H, m), 7.83-7.72 (13H, m), 7.68 (1H, m), 7.52 (1H, m), 7.49 (1H, d, *J*=8.1 Hz), 7.44 (1H, m), 7.30 (1H, m), 7.23 (1H, d, *J*=7.6 Hz), 7.17 (1H, s), 6.80 (1H, dd, *J*=13.7, 5.3 Hz), 3.94 (1H, m), 3.67-3.52 (2H, m), 2.36-2.25 (5H, m), 1.89 (1H, m), 1.82-1.69 (2H, m), 1.66-1.45 (6H, m), 1.01-0.89 (6H, m); **^{13}C NMR (101 MHz, DMSO-*d₆*, 298 K)** δ 174.6, 171.4, 141.1, 140.0, 139.2, 137.9, 134.9 (3, d, *J*=3.1 Hz), 133.6 (6, d, *J*=10.0 Hz), 131.6, 130.9, 130.2 (6, d, *J*=12.5 Hz), 130.0, 129.2, 126.3, 122.5, 121.3, 118.5 (3, d, *J*=85.6 Hz), 118.2, 53.4, 40.1, 35.9, 29.5 (d, *J*=16.9 Hz), 24.2, 24.2, 22.9, 21.7 (d, *J*=4.4 Hz), 20.8, 20.8, 20.2 (d, *J*=50.0 Hz). *Note*: *The resonance corresponding to the quaternary carbon directly bonded to the B-OH group was not observed due*

to signal broadening caused by the fast relaxation induced by the quadrupolar boron nucleus as well as possible residual J-coupling to boron. **Both diastereomers: ^{11}B NMR (160 MHz, DMSO-*d₆*, 298 K)** δ 2.0; **^{31}P{^{1}H} NMR (202 MHz, DMSO-*d₆*, 298 K)** δ 24.0; **HRMS (ESI)**: m/z calcd. for cation $[C_{44}H_{49}BN_4O_5PS]^+$ $[M]^+$: 787.3251, found 787.3252, error 0.422ppm.

Conjugate 74 (diazaborine 11-amide linker-phosphonium salt and *L*-Leucine). The title conjugate was prepared according to the same procedure followed for the synthesis of **conjugate 72** by using **conjugate 67** (200 mg, 0.26 mmol, 1 equiv) and *L*-Leucine (71 mg, 0.53 mmol, 2 equiv). After filtering off the excess of aminoacid and precipitating the product in a DCM:Et_2O solution, it was evaporated three times with $CDCl_3$ to remove the traces of diethyl ether. Obtained 161 mg (70% yield, dr: 1.8 to 1) as a brown solid. The major and minor diastereomers are indicated as *a* and *b*, respectively (when it was possible to distinguish their signals). **Diastereomer *A*: ^{1}H NMR (500 MHz, DMSO-*d₆*, 298 K)** δ 10.20 (1H, s, NH-CO), 8.15 (1H, s), 7.91-7.84 (3H, m), 7.84-7.72 (13H, m), 7.68 (1H, m), 7.54 (1H, d, *J*=7.9 Hz), 7.52 (1H, m), 7.47-7.41 (2H, m), 7.30 (1H, m), 7.15 (1H, s), 6.49 (1H, dd, *J*=13.2, 7.7 Hz), 4.04 (1H, m), 3.66-3.53 (2H, m), 2.36-2.26 (5H, m), 1.97-1.83 (2H, m), 1.77 (1H, m), 1.67-1.46 (6H, m), 1.01-0.91 (6H, m); **^{13}C NMR (151 MHz, DMSO-*d₆*, 298 K)** δ 174.6, 171.4, 141.1, 140.0, 139.2, 137.8, 134.8 (3, d, *J*=3.3 Hz), 133.6 (6, d, *J*=10.3 Hz), 131.6, 130.8, 130.2 (6, d, *J*=12.4 Hz), 130.0, 129.1, 126.3, 122.5, 121.5, 118.5 (3, d, *J*=85.6 Hz), 118.3, 53.8, 39.8, 35.9, 29.4 (d, *J*=17.0 Hz), 24.2, 24.2, 23.2, 21.7 (d, *J*=4.4 Hz), 21.1, 20.8, 20.2 (d, *J*=50.2 Hz). *Note*: *The resonance corresponding to the quaternary carbon directly bonded to the B-OH group was not observed due to signal broadening caused by the fast relaxation induced by the quadrupolar boron nucleus as well as possible residual J-coupling to boron.* **Diastereomer *B*: ^{1}H NMR (500 MHz, DMSO-*d₆*, 298 K)** δ 10.20 (1H, s, NH-CO), 8.15 (1H, s), 7.91-7.84 (3H, m), 7.84-7.72 (13H, m), 7.68 (1H, m), 7.52 (1H, m), 7.49 (1H, d, *J*=7.7 Hz), 7.44 (1H, m), 7.30 (1H, m), 7.23 (1H, d, *J*=7.7 Hz), 7.17 (1H, s), 6.80 (1H, dd, *J*=13.5, 5.5 Hz), 3.94 (1H, m), 3.66-3.53 (2H, m), 2.36-2.26 (5H, m), 1.88 (1H, m), 1.82-1.69 (2H, m), 1.67-1.46 (6H, m), 1.01-0-91 (6H, m); **^{13}C NMR (151 MHz, DMSO-*d₆*, 298 K)** δ 173.6, 171.4, 141.1, 139.9, 139.3, 137.9, 134.8 (3, d, *J*=3.3 Hz), 133.6 (6, d, *J*=10.3 Hz), 131,6, 130.9, 130.2 (6, d, *J*=12.4 Hz), 130.0, 129.2, 126.2, 122.5, 121.3, 118.5 (3, d, *J*=85.6 Hz), 118.3, 53.4, 40.5, 35.9, 29.4 (d, *J*=17.0 Hz), 24.2, 24.2, 22.9, 21.7 (d, *J*=4.4 Hz), 20.8, 20.8, 20.2 (d, *J*=50.2 Hz). *Note*: *The resonance corresponding to the quaternary carbon directly bonded to the B-OH group was not observed due to signal broadening caused by the fast relaxation induced by the quadrupolar boron nucleus as well as possible residual J-coupling to boron*. **Both diastereomers: ^{11}B NMR (160 MHz, DMSO-*d₆*, 298 K)** δ 2.0; **^{31}P{^{1}H} NMR (202 MHz, DMSO-*d₆*, 298 K)** δ 24.0; **HRMS (ESI)**: m/z calcd. for cation $[C_{44}H_{49}BN_4O_5PS]^+$ $[M]^+$: 787.3251, found 787.3252, error 0.422 ppm.

Conjugate 75 (diazaborine 11-amide linker-phosphonium salt and *D*-Methionine). The title conjugate was prepared according to the same procedure followed for the synthesis of **conjugate 71** by using **conjugate 67** (104 mg, 0.14 mmol, 1 equiv) and *D*-Methionine (41 mg, 0.28 mmol, 2 equiv). Obtained 97 mg (79% yield, dr: 1.8 to 1) as an off-white solid. The major and minor diastereomers are indicated as *a* and *b*, respectively (when it was possible to distinguish their signals). **Diastereomer A: ^{1}H NMR (600 MHz, DMSO-*d₆*, 298 K)** δ 10.20 (1H, s), 8.16 (1H, app. t, *J*=2.0 Hz), 7.90-7.86 (3H, m), 7.82-7.72 (14H, m), 7.55 (1H, ddd, *J*=7.9, 1.8, 1.1 Hz), 7.52 (1H, m), 7.44 (1H, m), 7.37 (1H, d, *J*=7.6 Hz), 7.30 (1H, m), 7.16 (1H, s), 6.69 (1H, dd, *J*=13.1, 7.8 Hz), 4.16 (1H, m), 3.64-3.55 (2H, m), 2.79 (1H, m), 2.65 (1H, m), 2.34-2.29 (5H, m), 2.27 (1H, m), 2.14 (1H, m), 2.09 (3H, s), 1.66-1.47 (6H, m); **^{13}C NMR (151 MHz, DMSO-*d₆*, 298 K)** δ 172.9, 171.4, 141.0, 140.0, 139.2, 138.0, 134.9 (3, d, *J*=2.9 Hz), 133.6 (6, *J*=10.0 Hz), 131.3, 130.9, 130.2 (6, d, *J*=12.5 Hz), 130.0, 129.2, 126.4, 122.6, 121.5, 118.5 (3, d, *J*=85.6 Hz), 118.3, 54.5, 35.9, 30.0, 30.0, 29.5 (d, *J*=16.9 Hz), 24.2, 21.7 (d, *J*=4.4 Hz), 20.8, 20.2 (d, *J*=50.1 Hz), 14.3. *Note*: *The resonance corresponding to the quaternary carbon directly bonded to the B-OH group was not observed due to signal broadening caused by the fast relaxation induced by the quadrupolar boron nucleus as well as possible residual J-coupling to boron*. **Diastereomer *B*: ^{1}H NMR (600 MHz, DMSO-*d₆*, 298 K)** δ 10.21 (1H, s, b), 8.15 (1H, app. t, *J*=2.1 Hz, b), 7.90-7.86 (3H, m), 7.82-7.72 (14H, m), 7.52 (1H, m), 7.49 (1H, dt, *J*=8.0, 1.4 Hz, b), 7.44 (1H, m), 7.31 (1H, m, b), 7.26 (1H, d, *J*=7.6 Hz, b), 7.17 (1H, s, b), 6.92 (1H, dd, *J*=13.5, 5.6 Hz, b), , 4.16 (1H, m), 3.64-3.55 (2H, m), 2.79 (1H, m), 2.65 (1H, m), 2.34-2.29 (5H, m), 2.22 (1H, m, b), 2.09 (3H, s), 2.03 (1H, m, b), 1.66-1.47 (6H, m); **^{13}C NMR (151 MHz, DMSO-*d₆*, 298 K)** δ 174.1 b, 171.4, 141.0, 139.9, 139.3, 138.0, 134.9 (3,

d, *J*=2.9 Hz), 133.6 (6, *J*=10.0 Hz), 131.6, 130.9, 130.2 (6, d, *J*=12.5 Hz), 130.0, 129.3, 126.3, 122.6, 121.3, 118.5 (3, d, *J*=85.6 Hz), 118.1, 53.8, 35.9, 30.4, 29.9, 29.5 (d, *J*=16.9 Hz), 24.2, 21.7 (d, *J*=4.4 Hz), 20.8, 20.2 (d, *J*=50.1 Hz), 14.2; *Note*: *The resonance corresponding to the quaternary carbon directly bonded to the B-OH group was not observed due to signal broadening caused by the fast relaxation induced by the quadrupolar boron nucleus as well as possible residual J-coupling to boron.* **Both diastereomers: ^{11}B NMR (128 MHz, DMSO-*d*$_6$, 298K)** δ 2.6; **^{31}P{^{1}H} NMR (243 MHz, DMSO-*d*$_6$, 298K)** δ 24.0; **HRMS (ESI):** m/z calcd. for cation $[C_{43}H_{47}BN_4O_5PS_2]^+$ $[M]^+$: calcd. 805.2815, found 805.2810, error 0.373 ppm.

Conjugate 76 (diazaborine 11-amide linker-phosphonium salt and *L*-Methionine). The title conjugate was prepared according to the same procedure followed for the synthesis of **conjugate 71** by using **conjugate 67** (104 mg, 0.14 mmol, 1 equiv) and *L*-Methionine (41 mg, 0.28 mmol, 2 equiv). Obtained 110 mg (90% yield, dr: 1.8 to 1) as an off-white solid. The major and minor diastereomers are indicated as *a* and *b*, respectively (when it was possible to distinguish their signals). **Diastereomer A: ^{1}H NMR (600 MHz, DMSO-*d*$_6$, 298 K)** δ 10.17 (s, 1H), 8.15 (1H, app.t, *J*=2.0 Hz), 7.91-7.86 (3H, m), 7.82-7.73 (14H, m), 7.55 (1H, ddd, *J*=8.0, 1.8, 1.0 Hz), 7.52 (1H, m), 7.44 (1H, m), 7.37 (1H, d, *J*=7.6 Hz), 7.28 (1H, m), 7.16 (1H, s), 6.69 (1H, dd, *J*=13.1, 7.8 Hz), 4.16 (1H, m), 3.66-3.50 (2H, m), 2.79 (1H, m), 2.63 (1H, m), 2.34-2.28 (5H, m), 2.26 (1H, m), 2.14 (1H, m), 2.09 (3H, s), 1.66-1.42 (6H, m); **^{13}C NMR (151 MHz, DMSO-*d*$_6$, 298 K)** δ 172.9, 171.4, 141.0, 140.0, 139.2, 138.0, 134.9 (3, d, *J*=2.9 Hz), 133.6 (6, d, *J*=10.0 Hz), 131.3, 130.9, 130.2 (6, d, *J*=12.5 Hz), 130.0, 129.2, 126.4, 122.6, 121.5, 118.5 (3, d, *J*=85.6 Hz), 118.3, 54.5, 35.9, 30.0, 30.0, 29.5 (d, *J*=16.9 Hz), 24.2, 21.7 (d, *J*=4.4 Hz), 20.8, 20.2 (d, *J*=50.1 Hz), 14.3. *Note*: *The resonance corresponding to the quaternary carbon directly bonded to the B-OH group was not observed due to signal broadening caused by the fast relaxation induced by the quadrupolar boron nucleus as well as possible residual J-coupling to boron.* **Diastereomer *B*: ^{1}H NMR (600 MHz, DMSO-*d*$_6$, 298 K)** δ 10.17 (s, 1H), 8.13 (1H, app.t, *J*=2.0 Hz), 7.91-7.86 (3H, m), 7.82-7.73 (14H, m), 7.52 (1H, m), 7.48 (1H, dt, *J*=8.0, 1.4 Hz), 7.44 (1H, m), 7.31 (1H, m), 7.25 (1H, d, *J*=7.6 Hz), 7.17 (1H, s), 6.91 (1H, dd, *J*=13.4, 5.3 Hz), 4.16 (1H, m), 3.66-3.50 (2H, m), 2.79 (1H, m), 2.63 (1H, m), 2.34-2.28 (5H, m), 2.21 (1H, m), 2.09 (3H, s), 2.05 (1H, m), 1.66-1.42 (6H, m); **^{13}C NMR (151 MHz, DMSO-*d*$_6$, 298 K)** δ 174.1 b, 171.4, 141.0 b, 139.9 b, 139.3 b, 138.0 b, 134.9 (3, d, *J*=2.9 Hz), 133.6 (6, d, *J*=10.0 Hz), 131.6 b, 130.9 b, 130.2 (6, d, *J*=12.5 Hz), 130.0, 129.3 b, 126.3 b, 122.6 b, 121.3 b, 118.5 (3, d, *J*=85.6 Hz), 118.1 b, 53.8 b, 35.9, 30.4 b, 29.9 b, 29.5 (d, *J*=16.9 Hz), 24.2, 21.7 (d, *J*=4.4 Hz), 20.8 b, 20.2 (d, *J*=50.1 Hz), 14.3 b. *Note*: *The resonance corresponding to the quaternary carbon directly bonded to the B-OH group was not observed due to signal broadening caused by the fast relaxation induced by the quadrupolar boron nucleus as well as possible residual J-coupling to boron*. **Both diastereomers: ^{11}B NMR (128 MHz, DMSO-*d*$_6$, 298 K)** δ 2.7; **^{31}P{^{1}H} NMR (243 MHz, DMSO-*d*$_6$, 298 K)** δ 24.0; **HRMS (ESI)**: m/z calcd. for cation $[C_{43}H_{47}BN_4O_5PS_2]^+$ $[M]^+$: calcd. 805.2815, found 805.2818, error 0.608 ppm.

Conjugates of diazaborine 11 and aminoacids with phosphonium salts connected by a carbamate linker

Conjugate 77 (diazaborine 11-carbamate linker-phosphonium salt and *D*-Tryptophan). A dry MeCN solution (15 ml) of **conjugate 68** (200 mg, 0.26 mmol, 1 equiv) and *D*-Tryptophan (104 mg, 0.52 mmol, 2 equiv) was refluxed under argon for 36 h. After cooling down the reaction mixture to room temperature, the turbid solution was filtered to remove the unreacted aminoacid. The filtrate was evaporated and the remaining solid was redissolved in 10 ml of MeCN and ca. 30 ml of Et_2O were added to induce precipitation of the product. The resulting solution was further stirred overnight, then the precipitate was filtered from the solution, the traces of diethyl ether were removed by azeotropic distillation with $CDCl_3$, and the remaining solid was dried in vacuo to afford the title compound (70 mg, 28% yield) as a diastereomeric mixture (dr: 3 to 1). Brown solid. The major and minor diastereomers are indicated as *a* and *b*, respectively (when it was possible to distinguish their signals). **Diastereomer A: ^{1}H NMR (500 MHz, DMSO-*d*$_6$, 298 K)** δ 11.09 (1H, s), 9.87 (1H, s), 8.04 (1H, s), 8.00-7.85 (4H, m), 7.83-7.70 (12H, m), 7.63-7.56 (2H, m), 7.49-7.35 (5H, m), 7.15-7.07 (2H, m), 7.07-7.00 (2H, m), 6.43 (1H, d, *J*=7.7 Hz), 6.12 (1H, dd, *J*=14.4, 6.3 Hz), 4.33 (1H, m), 4.13-3.97 (2H, m), 3.68-3.48 (2H, m), 3.44-3.35 (2H, m), 2.27 (3H, s), 1.63-1.44 (6H, m), 1.42-1.31 (2H, m); **^{13}C NMR (126 MHz, DMSO-*d*$_6$, 298 K)** δ 173.9 a, 153.5 a, 141.3 a, 139.8 a, 139.3 a, 137.7 a, 136.4 a, , 134.9 (3, d, *J*=3.1

Hz), 133.6 (6, d, *J*=10.1 Hz), 131.0 a, 130.6 a, 130.2 (6, d, *J*=12.4 Hz), 130.2 a, 129.2 (ab), 126.8 a, 126.1 a, 125.0 a, 121.7, 121.3 ab, 120.7 a, 118.7 a, 118.5 (3, d, *J*=85.6 Hz), 118.3 ab, 117.3 a, 111.5 a, 108.3 a, 64.1, 55.6 a, 29.4 (d, *J*=16.6 Hz), 28.1, 26.3 a, 24.4, 21.7 (d, *J*=4.4 Hz), 20.7 a, 20.1 (d, *J*=50.0 Hz). *Note*: *The resonance corresponding to the quaternary carbon directly bonded to the B-OH group was not observed due to signal broadening caused by the fast relaxation induced by the quadrupolar boron nucleus as well as possible residual J-coupling to boron*. **Diastereomer B: ^{1}H NMR (500 MHz, DMSO-*d*$_6$, 298 K)** δ 10.94 (1H, s, b), 9.90 (1H, s, b), 8.11 (1H, s, b), 8.00-7.85 (4H, m), 7.83-7.70 (12H, m), 7.63-7.56 (2H, m), 7.54 (1H, d, *J*=7.6 Hz, b), 7.51 (1H, s, b), 7.49-7.35 (3H, m), 7.23 (1H, m, b), 7.15-7.07 (2H, m), 7.03 (1H, m), 6.55 (1H, dd, *J*=13.5, 6.4 Hz), 4.41 (1H, m, b), 4.13-3.97 (2H, m), 3.68-3.48 (3H, m), 3.38 (1H, m), 2.30 (3H, s), 1.63-1.44 (6H, m), 1.42-1.31 (2H, m); **^{13}C NMR (126 MHz, DMSO-*d*$_6$, 298 K)** δ 172.9 b, 153.5 b, 141.2 b, 140.1 b, 139.3 b, 137.8 b, 136.2 b, 134.9 (3, d, *J*=3.1 Hz), 133.6 (6, d, *J*=10.1 Hz), 131.6 b, 130.7 b, 130.2 (6, d, *J*=12.4 Hz), 130.0 b, 129.2 (ab), 127.0 b, 126.2 b, 124.2 b, 121.7 b, 121.0 b, 120.9 b, 118.5 (3, d, *J*=85.6 Hz), 118.4 b, 118.0 b, 117.4 b, 111.3 b, 109.7 b, 64.1, 56.0 b, 29.4 (d, *J*=16.6 Hz), 28.1, 26.4 b, 24.4, 21.7 (d, *J*=4.4 Hz), 20.8 b, 20.1 (d, *J*=50.0 Hz). **Both diastereomers: ^{11}B NMR (128 MHz, DMSO-*d*$_6$, 298 K)** δ 2.7; **^{31}P{^{1}H} NMR (162 MHz, DMSO-*d*$_6$, 298 K)** δ 24.1; **HRMS (ESI)**: m/z calcd. for cation $[C_{50}H_{50}BN_5O_6PS]^+$ $[M]^+$: 890.3309, found 890.3309, error 0.221 ppm.

Conjugate 78 (diazaborine 11-carbamate linker-phosphonium salt and *L*-Tryptophan). The title conjugate was prepared according to the same procedure followed for the synthesis of **conjugate 77** by refluxing for 48 h a MeCN solution (15 ml) of **conjugate 68** (200 mg, 0.26 mmol, 1 equiv) and *L*-Tryptophan (104 mg, 0.52 mmol, 2 equiv). Obtained 118 mg (48% yield, dr: 2.0 to 1) as a brownish solid. The major and minor diastereomers are indicated as *a* and *b*, respectively (when it was possible to distinguish their signals). **Diastereomer A: ^{1}H NMR (600 MHz, DMSO-*d*$_6$, 298 K)** δ 11.09 (1H, s), 9.87 (1H, s), 8.05 (1H, s), 7.95 (1H, dd, *J*=13.5, 8.8 Hz), 7.91-7.83 (3H, m), 7.84-7.72 (12H, m), 7.64-7.56 (2H, m), 7.47 (1H, s), 7.46-7.43 (2H, m), 7.42-7.39 (2H, m), 7.15-7.08 (2H, m), 7.05-6.99 (2H, m), 6.43 (1H, d, *J*=7.6 Hz), 6.11 (1H, dd, *J*=13.4, 6.4 Hz), 4.34 (1H, m), 4.11-4.01 (2H, m), 3.64-3.49 (2H, m), 3.43-3.35 (2H, m), 2.27 (3H, s), 1.63-1.43 (6H, m), 1.42-1.32 (2H, m); **^{13}C NMR (126 MHz, DMSO-*d*$_6$, 298 K)** δ 173.9, 153.5, 141.2, 139.8, 139.3, 137.7, 136.4, 134.9 (3, d, *J*=3.2 Hz), 133.6 (6, d, *J*=10.0 Hz), 131.0, 130.6, 130.2 (6, d, *J*=12.4 Hz), 130.2, 129.2, 126.8, 126.1, 125.0, 121.7, 121.3, 120.7, 118.7, 118.5 (3, d, *J*=85.5 Hz), 118.3, 117.3, 111.5, 108.3, 64.1, 55.6, 29.4 (d, *J*=16.9 Hz), 28.1, 26.3, 24.4, 21.7 (d, *J*=4.5 Hz), 20.7, 20.1 (d, *J*=49.8 Hz). *Note*: *The resonance corresponding to the quaternary carbon directly bonded to the B-OH group was not observed due to signal broadening caused by the fast relaxation induced by the quadrupolar boron nucleus as well as possible residual J-coupling to boron*. **Diastereomer B: ^{1}H NMR (600 MHz, DMSO-*d*$_6$, 298 K)** δ 10.95 (1H, s), 9.89 (1H, s), 8.12 (1H, s), 7.91-7.83 (4H, m), 7.84-7.72 (12H, m), 7.64-7.56 (2H, m), 7.54 (1H, d, *J*=7.9 Hz), 7.51 (1H, s), 7.47 (1H, s), 7.44 (1H, m), 7.41 (1H, m), 7.38 (1H, d, *J*=8.2 Hz), 7.23 (1H, dd, *J*=7.8, 1.7 Hz), 7.15-7.08 (2H, m), 7.03 (1H, m), 6.54 (1H, dd, *J*=12.9, 7.7 Hz), 4.40 (1H, m), 4.11-4.01 (2H, m), 3.64-3.49 (3H, m), 3.38 (1H, m), 2.30 (3H, s, b), 1.63-1.43 (6H, m), 1.42-1.32 (2H, m); **^{13}C NMR (126 MHz, DMSO-*d*$_6$, 298 K)** δ 172.9, 153.5, 141.2, 140.1, 139.3, 137.8, 136.2, 134.9 (3, d, *J*=3.2 Hz), 133.6 (6, d, *J*=10.0 Hz), 131.6, 130.7, 130.2 (6, d, *J*=12.4 Hz), 130.0, 129.2, 127.0, 126.3, 124.2, 121.8, 121.0, 120.9, 118.5 (3, d, *J*=85.5 Hz), 118.4, 117.9, 117.4, 111.3, 109.7, 64.1, 56.0, 29.4 (d, *J*=16.9 Hz), 28.1, 26.4, 24.4, 21.7 (d, *J*=4.5 Hz), 20.8, 20.1 (d, *J*=49.8 Hz). *Note*: *The resonance corresponding to the quaternary carbon directly bonded to the B-OH group was not observed due to signal broadening caused by the fast relaxation induced by the quadrupolar boron nucleus as well as possible residual J-coupling to boron*. **Both diastereomers: ^{11}B NMR (160 MHz, DMSO-*d*$_6$, 298 K)** δ 2.7; **^{31}P{^{1}H} NMR (202 MHz, DMSO-*d*$_6$, 298 K)** δ 24.1; **HRMS (ESI)**: m/z calcd. for cation $[C_{50}H_{50}BN_5O_6PS]^+$ $[M]^+$: 890.3309, found 890.3308, error 0.070 ppm.

Conjugate 79 (diazaborine 11-carbamate linker-phosphonium salt and *D*-Leucine). The title conjugate was prepared according to the same procedure followed for the synthesis of **conjugate 77** by refluxing for 48 h a MeCN solution (15 ml) of **conjugate 68** (200 mg, 0.26 mmol, 1 equiv) and *D*-Leucine (67 mg, 0.52 mmol, 2 equiv). Obtained 173 mg (76% yield, dr: 7.0 to 1.0) as a yellowish solid. The major and minor diastereomers are indicated as *a* and *b*, respectively (when it was possible to distinguish their signals). **Diastereomer A: ^{1}H NMR (500 MHz, DMSO-*d*$_6$, 298 K)** δ 9.87 (1H, s), 8.07 (1H, s), 7.92-7.86 (3H, m), 7.84-7.73 (12H, m), 7.68

(1H, m), 7.59 (1H, d, *J*=8.0 Hz), 7.53-7.48 (2H, m), 7.47-7.38 (2H, m), 7.29 (1H, m), 7.16 (1H, m), 6.48 (1H, dd, *J*=13.2, 7.8 Hz), 4.10-4.00 (3H, m), 3.64-3.50 (2H, m), 2.32 (3H, s), 1.96-1.70 (3H, m), 1.62-1.44 (6H, m), 1.41-1.33 (2H, m), 1.00-0.86 (6H, m); **^{13}C NMR (151 MHz, DMSO-*d*$_6$, 298 K)** δ 173.5, 153.5, 141.2, 140.0, 139.2, 137.8, 134.8 (3, d, *J*=3.2 Hz), 133.5 (6, *J*=10.0 Hz), 131.6, 130.8, 130.2 (6, *J*=12.4 Hz), 130.0, 129.1, 126.3, 121.8, 120.9, 118.5 (3, d, *J*=85.6 Hz), 117.4, 64.1, 53.8, 40.0, 29.3 (d, *J*=16.8 Hz), 28.1, 24.4, 24.2, 23.2, 21.7 (d, *J*=4.2 Hz), 21.1, 20.8, 20.2 (d, *J*=49.8 Hz). *Note*: *The resonance corresponding to the quaternary carbon directly bonded to the B-OH group was not observed due to signal broadening caused by the fast relaxation induced by the quadrupolar boron nucleus as well as possible residual J-coupling to boron*. **Diastereomer B: ^{1}H NMR (500 MHz, DMSO-*d*$_6$, 298 K)** δ 9.87 (1H, s), 8.07 (1H, s), 7.92-7.86 (3H, m), 7.84-7.73 (12H, m), 7.68 (1H, m), 7.59 (1H, d, *J*=8.0 Hz), 7.51 (1H, m), 7.47-7.38 (2H, m), 7.29 (1H, m), 7.24 (1H, d, *J*=7.6 Hz), 7.16 (1H, m), 6.79 (1H, dd, *J*=14.1, 5.5 Hz), 4.10-4.00 (2H, m), 3.94 (1H, m), 3.64-3.50 (2H, m), 2.32 (3H, s), 1.96-1.70 (3H, m), 1.62-1.44 (6H, m), 1.41-1.33 (2H, m), 1.00-0.86 (6H, m); **^{13}C NMR (151 MHz, DMSO-*d*$_6$, 298 K)** δ 174.5, 153.5, 141.2, 139.9, 139.3, 137.8, 134.8 (3, d, *J*=3.2 Hz), 133.5 (6, *J*=10.0 Hz), 131.6, 130.8, 130.2 (6, *J*=12.4 Hz), 130.0, 129.2, 126.2, 121.8, 120.7, 118.5 (3, d, *J*=85.6 Hz), 117.3, 64.1, 53.4, 40.5, 29.3 (d, *J*=16.8 Hz), 28.1, 24.4, 24.2, 22.8, 21.7 (d, *J*=4.2 Hz), 20.8, 20.8, 20.2 (d, *J*=49.8 Hz). *Note*: *The resonance corresponding to the quaternary carbon directly bonded to the B-OH group was not observed due to signal broadening caused by the fast relaxation induced by the quadrupolar boron nucleus as well as possible residual J-coupling to boron*. **Both diastereomers: ^{11}B NMR (128 MHz, DMSO-*d*$_6$, 298 K)** δ 2.8; **^{31}P{^{1}H} NMR (162 MHz, DMSO-*d*$_6$, 298 K)** δ 24.1; **HRMS (ESI)**: m/z calcd. for cation $[C_{45}H_{51}BN_4O_6PS]^+$ $[M]^+$: 817.3356, found 817.3356, error 0.125 ppm.

Conjugate 80 (diazaborine 11-carbamate linker-phosphonium salt and *L*-Leucine). The title conjugate was prepared according to the same procedure followed for the synthesis of **conjugate 77** by refluxing for 48 h a MeCN solution (15 ml) of **conjugate 68** (200 mg, 0.26 mmol, 1 equiv) and *L*-Leucine (67 mg, 0.52 mmol, 2 equiv). Obtained 58 mg (25% yield, dr: 2.0 to 1) as a yellowish solid. The major and minor diastereomers are indicated as *a* and *b*, respectively (when it was possible to distinguish their signals). **Diastereomer A: ^{1}H NMR (400 MHz, DMSO-*d*$_6$, 298 K)** δ 9.88 (1H, s), 8.08 (1H, s), 7.92-7.86 (3H, m), 7.85-7.72 (12H, m), 7.68 (1H, m), 7.60 (1H, m), 7.54-7.48 (2H, m), 7.48-7.38 (2H, m), 7.29 (1H, m), 7.16 (1H, m), 6.48 (1H, dd, *J*=13.4, 7.8 Hz), 4.10-4.00 (3H, m), 3.65-3.50 (2H, m), 2.32 (3H, s), 1.99-1.68 (3H, m), 1.60-1.44 (6H, m), 1.42-1.31 (2H, m), 1.02-0.85 (6H, m); **^{13}C NMR (151 MHz, DMSO-*d*$_6$, 298 K)** δ 173.6, 153.5, 141.3, 140.0, 139.2, 137.8, 134.9 (3, d, *J*=3.1 Hz), 133.6 (6, d, *J*=10.1 Hz), 131.6, 130.8, 130.2 (6, d, *J*=12.5 Hz), 130.0, 129.2, 126.3, 121.8, 120.9, 118.6 (3, d, *J*=85.6 Hz), 117.4, 64.2, 53.8, 39.6, 29.4 (d, *J*=16.8 Hz), 28.1, 24.4, 24.2, 23.2, 21.7 (d, *J*=4.2 Hz), 21.1, 20.8, 20.1 (d, *J*=49.8 Hz). *Note*: *The resonance corresponding to the quaternary carbon directly bonded to the B-OH group was not observed due to signal broadening caused by the fast relaxation induced by the quadrupolar boron nucleus as well as possible residual J-coupling to boron*. **Diastereomer B: ^{1}H NMR (400 MHz, DMSO-*d*$_6$, 298 K)** δ 9.88 (1H, s), 8.08 (1H, s), 7.92-7.86 (3H, m), 7.85-7.72 (12H, m), 7.68 (1H, m), 7.60 (1H, m), 7.51 (1H, m), 7.48-7.38 (2H, m), 7.29 (1H, m), 7.24 (1H, d, *J*=7.6 Hz, b), 7.16 (1H, m), 6.80 (1H, dd, *J*=13.9, 5.4 Hz), 4.10-4.00 (2H, m), 3.94 (1H, m), 3.65-3.50 (2H, m), 2.32 (3H, s), 1.99-1.68 (3H, m), 1.60-1.44 (6H, m), 1.42-1.31 (2H, m), 1.02-0.85 (6H, m); **^{13}C NMR (151 MHz, DMSO-*d*$_6$, 298 K)** δ 174.5, 153.5, 141.2, 139.9, 139.3, 137.9, 134.9 (3, d, *J*=3.1 Hz), 133.6 (6, d, *J*=10.1 Hz), 131.6, 130.9, 130.2 (6, d, *J*=12.5 Hz), 130.0, 129.3, 126.3, 121.8, 120.7, 118.6 (3, d, *J*=85.6 Hz), 117.3, 64.2, 53.4, 40.0, 29.4 (d, *J*=16.8 Hz), 28.1, 24.4, 24.2, 22.9, 21.7 (d, *J*=4.2 Hz), 21.1, 20.8, 20.1 (d, *J*=49.8 Hz); *Note*: *The resonance corresponding to the quaternary carbon directly bonded to the B-OH group was not observed due to signal broadening caused by the fast relaxation induced by the quadrupolar boron nucleus as well as possible residual J-coupling to boron*. **Both diastereomers: ^{11}B NMR (160 MHz, DMSO-*d*$_6$, 298 K)** δ 2.7; **^{31}P{^{1}H} NMR (202 MHz, DMSO-*d*$_6$, 298 K)** δ 24.1; **HRMS (ESI)**: m/z calcd. for cation $[C_{45}H_{51}BN_4O_6PS]^+$ $[M]^+$: 817.3356, found 817.3356, error 0.223 ppm.

Conjugate 81 (diazaborine 11-carbamate linker-phosphonium salt and *D*-Methionine). The title conjugate was prepared according to the same procedure followed for the synthesis of **conjugate 77** by refluxing for 48 h a MeCN solution (15 ml) of **conjugate 68** (200 mg, 0.26 mmol, 1 equiv) and *D*-Methionine (76 mg, 0.52 mmol, 2 equiv). Obtained 65 mg (28% yield, *ca.* dr: 2.0 to 1) as a yellowish solid. The major and

minor diastereomers are indicated as *a* and *b*, respectively (when it was possible to distinguish their signals). **Diastereomer A: ^{1}H NMR (600 MHz, DMSO-*d*$_6$, 298 K)** δ 9.88 (1H, s), 8.08 (1H, s), 7.93-7.86 (3H, m), 7.84-7.68 (13H, m), 7.58 (1H, m), 7.54-7.48 (2H, m), 7.43 (1H, m), 7.37 (1H, d, *J*=7.6 Hz), 7.30 (1H, m), 7.16 (1H, s), 6.69 (1H, m), 4.15 (1H, m), 4.06 (2H, t, *J*=6.7 Hz), 3.63-3.50 (2H, m), 2.79 (1H, m), 2.63 (1H, m), 2.37-2.20 (4H, m), 2.18-2.01 (4H, m), 1.64-1.45 (6H, m), 1.42-1.32 (2H, m); **^{13}C NMR (151 MHz, DMSO-*d*$_6$, 298 K)** δ 172.9, 153.5, 141.1, 140.0, 139.3, 138.0, 134.9 (3, d, *J*=3.1 Hz), 133.6 (6, d, *J*=10.3 Hz), 131.3, 130.9, 130.2 (6, d, *J*=12.2 Hz), 130.0, 129.2, 126.4, 121.8 (br), 120.9, 118.5 (3, d, *J*=85.9 Hz), 117.5 (br), 64.2, 54.4, 30.0, 29.9, 29.4 (d, *J*=16.8 Hz), 28.1, 24.4, 21.7 (d, *J*=3.8 Hz), 20.8, 20.1 (d, *J*=50.2 Hz), 14.3; *Note*: *The resonance corresponding to the quaternary carbon directly bonded to the B-OH group was not observed due to signal broadening caused by the fast relaxation induced by the quadrupolar boron nucleus as well as possible residual J-coupling to boron*. **Diastereomer B**: **^{1}H NMR (600 MHz, DMSO-*d*$_6$, 298 K)** δ 9.88 (1H, s), 8.07 (1H, s), 7.93-7.86 (3H, m), 7.84-7.68 (13H, m), 7.58 (1H, m), 7.51 (1H, m), 7.46-7.39 (2H, m), 7.30 (1H, m), 7.26 (1H, d, *J*=7.7 Hz), 7.16 (1H, s), 6.91 (1H, m), 4.15 (1H, m), 4.06 (2H, t, *J*=6.7 Hz), 3.63-3.50 (2H, m), 2.79 (1H, m), 2.63 (1H, m), 2.37-2.20 (4H, m), 2.18-2.01 (4H, m), 1.64-1.45 (6H, m), 1.42-1.32 (2H, m); **^{13}C NMR (151 MHz, DMSO-*d*$_6$, 298 K)** δ 174.1, 153.5, 141.1, 140.0, 139.4, 138.0, 134.9 (3, d, *J*=3.1 Hz), 133.6 (6, d, *J*=10.3 Hz), 131.3, 130.9, 130.2 (6, d, *J*=12.2 Hz), 130.0, 129.3, 126.3, 121.8, 120.7, 118.5 (3, d, *J*=85.9 Hz), 117.5 (br), 64.2, 54.4, 30.0, 29.9, 29.4 (d, *J*=16.8 Hz), 28.1, 24.4, 21.7 (d, *J*=3.8 Hz), 20.8, 20.1 (d, *J*=50.2 Hz), 14.2; *Note*: *The resonance corresponding to the quaternary carbon directly bonded to the B-OH group was not observed due to signal broadening caused by the fast relaxation induced by the quadrupolar boron nucleus as well as possible residual J-coupling to boron*. **Both diastereomers: ^{11}B NMR (160 MHz, DMSO-*d*$_6$, 298 K)** δ 2.2; **^{31}P{^{1}H} NMR (243 MHz, DMSO-*d*$_6$, 298 K)** δ 24.0; **HRMS (ESI)**: m/z calcd for cation $[C_{44}H_{49}BN_4O_6PS_2]^+$ $[M]^+$: 835.2921, found 835.2925, error 0.738 ppm.

Conjugate 82 (diazaborine 11-carbamate linker-phosphonium salt and *L*-Methionine,). The title conjugate was prepared according to the same procedure followed for the synthesis of **conjugate 77** by refluxing for 48 h a MeCN solution (15 ml) of **conjugate 68** (200 mg, 0.26 mmol, 1 equiv) and *L*-Methionine (76 mg, 0.52 mmol, 2 equiv). Obtained 25 mg (11% yield, *ca.* dr: 2.0 to 1) as a yellowish solid. The major and minor diastereomers are indicated as *a* and *b*, respectively (when it was possible to distinguish their signals). **Diastereomer A: ^{1}H NMR (500 MHz, DMSO-*d*$_6$, 298 K)** δ 9.87 (1H, s), 8.08 (1H, s), 7.92-7.85 (3H, m), 7.84-7.66 (13H, m), 7.59 (1H, d, *J*=7.9 Hz), 7.54-7.48 (2H, m), 7.43 (1H, m), 7.37 (1H, d, *J*=7.6 Hz), 7.28 (1H, m), 7.16 (1H, s), 6.68 (1H, dd, *J*=13.1, 7.8 Hz), 4.15 (1H, m), 4.06 (2H, t, *J*=6.7 Hz), 3.65-3.49 (2H, m), 2.79 (1H, m), 2.63 (1H, m), 2.38-2.21 (4H, m), 2.20-1.99 (4H, m), 1.63-1.44 (6H, m), 1.42-1.31 (2H, m); **^{13}C NMR (151 MHz, DMSO-*d*$_6$, 298 K)** δ 172.9, 153.5, 141.2, 140.0, 139.3, 138.0, 134.9 (3, d, *J*=3.0 Hz), 133.6 (6, d, *J*=10.2 Hz), 131.3, 130.9, 130.2 (6, d, *J*=12.2 Hz), 130.0, 129.2, 126.4, 121.8 (br), 120.9, 118.5 (3, d, *J*=85.8 Hz), 117.4 (br), 64.2, 54.5, 30.0, 29.9, 29.4 (d, *J*=16.9 Hz), 28.1, 24.4, 21.7 (d, *J*=4.0 Hz), 20.8, 20.1 (d, *J*=50.3 Hz), 14.3. *Note*: *The resonance corresponding to the quaternary carbon directly bonded to the B-OH group was not observed due to signal broadening caused by the fast relaxation induced by the quadrupolar boron nucleus as well as possible residual J-coupling to boron*. **Diastereomer B**: **^{1}H NMR (500 MHz, DMSO-*d*$_6$, 298 K)** δ 9.87 (1H, s), 8.07 (1H, s), 7.92-7.85 (3H, m), 7.84-7.66 (13H, m), 7.59 (1H, d, *J*=7.9 Hz), 7.51 (1H, m), 7.47-7.40 (2H, m), 7.33-7.23 (2H, m), 7.16 (1H, s), 6.90 (1H, dd, *J*=13.1, 5.1 Hz), 4.15 (1H, m), 4.06 (2H, t, *J*=6.7 Hz), 3.65-3.49 (2H, m), 2.79 (1H, m), 2.63 (1H, m), 2.38-2.21 (4H, m), 2.20-1.99 (4H, m), 1.63-1.44 (6H, m), 1.42-1.31 (2H, m); **^{13}C NMR (151 MHz, DMSO-*d*$_6$, 298 K)** δ 174.1, 153.5, 141.2, 140.0, 139.4, 138.0, 134.9 (3, d, *J*=3.0 Hz), 133.6 (6, d, *J*=10.2 Hz), 131.3, 130.9, 130.2 (6, d, *J*=12.2 Hz), 130.0, 129.3, 126.3, 121.8 (br), 120.7, 118.5 (3, d, *J*=85.8 Hz), 117.4 (br), 64.2, 54.5, 30.0, 29.9, 29.4 (d, *J*=16.9 Hz), 28.1, 24.4, 21.7 (d, *J*=4.0 Hz), 20.8, 20.1 (d, *J*=50.3 Hz), 14.3. **Both diastereomers: ^{11}B NMR (160 MHz, DMSO-*d*$_6$, 298 K)** δ 2.3; **^{31}P{^{1}H} NMR (243 MHz, DMSO-*d*$_6$, 298 K)** δ 24.0; **HRMS (ESI)**: m/z calcd for cation $[C_{44}H_{49}BN_4O_6PS_2]^+$ $[M]^+$: 835.2921, found 835.2917, error 0.228 ppm.

References

[S1] Amin, N.; Claridge, T. Quantitative NMR Spectroscopy. University of Oxford Lectures, **2015**, https://nmr.chem.ox.ac.uk/files/quantitativenmrpdf

Detailed synthesis procedures of diazaborine precursors

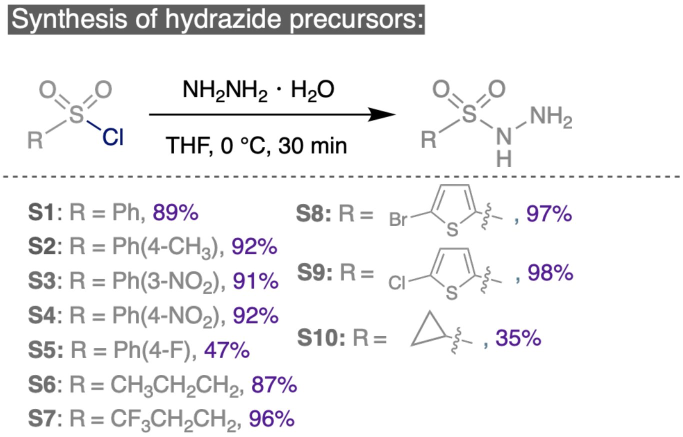

Syntheses of intermediate hydrazides S1-S10 (General Method E). Unless stated otherwise, hydrazine hydrate (2.5 equiv) was added dropwise to a dry THF solution (0.2M) of the corresponding sulfonyl chloride (1 equiv) at 0 °C under Ar. The resulting reaction mixture was stirred for 30-120 min, and then the mixture was partitioned between ethyl acetate and brine. The organic phase was separated, and the aqueous phase was back-extracted two times with ethyl acetate. The combined organic extracts were dried over Na_2SO_4 and dried under high vacuum to afford the corresponding hydrazide intermediate, which was used in the next step without further purification.

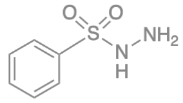

Benzenesulfonohydrazide (S1). A water solution (900 mL) of hydrazine monohydrate (111.1 g, 2.2 mol, 10 equiv) was cooled down to 5 °C, and benzenesulfonyl chloride (40.0 g, 0.22 mol, 1 equiv) was added dropwise *via* dropping funnel. After completion of the addition, the reaction mixture was further stirred at 5 °C for 30 minutes. Then, the reaction mixture was extracted with ethyl acetate (2x), and the combined organic extracts were washed with brine. The resulting organic phase was dried over Na_2SO_4 and concentrated in *vacuo* to furnish the title compound (33.7 g, 89% yield) as a white solid, which was used without further purification in the next step. **^{1}H NMR (400 MHz, DMSO-d$_6$, 298 K)** δ 8.40 (1H, br. s, SO_2-N***H***-NH_2), 7.88-7.76 (2H, m, 2x Ar***H***), 7.70-7.53 (3H, m 3x Ar***H***), 4.12 (2H, br. s, SO_2-NH-N***H$_2$***); **^{13}C NMR (101 MHz, DMSO-d$_6$, 298 K)** δ 138.2 (Ar***C***), 132.6 (Ar***C***), 129.0 (2x Ar***C***H), 127.6 (2x Ar***C***H). The spectral data matches the reported one.[S1]

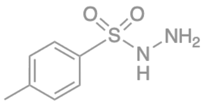

4-Methylbenzenesulfonohydrazide (S2). The title compound was prepared as a white solid (9.1 g, 92% yield) through the general method E by reacting *p*-tosyl chloride (10.1 g, 53 mmol, 1 equiv) and hydrazine hydrate (6.0 g, 120 mmol, 2.3 equiv) for 30 min, and it was used in the next step without further purification. The spectral data is fully consisting with the reported one.[S2]

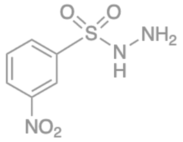

3-Nitrobenzenesulfonohydrazide (S3). The title compound was synthesized following the general method E as a yellowish solid (35.8 g, 91% yield) by reacting 3-nitrobenzenesulfonyl chloride (40 g, 0.18 mol, 1 equiv) and hydrazine hydrate (22.6 g, 0.45 mol, 2.5 equiv). **^{1}H NMR (400 MHz, DMSO-d$_6$, 298 K)** δ 8.73 (1H, br. s, SO_2-N***H***NH_2), 8.53 (1H, app. t, J=2.0 Hz, Ar***H***), 8.50 (1H, ddd, J=8.2, 2.3, 1.0 Hz, Ar***H***), 8.21 (1H, ddd, J=7.8, 1.7, 1.0 Hz, Ar***H***), 7.91 (1H, t, J=8.0 Hz, Ar***H***), 4.34 (2H, br. s, SO_2NH-N***H$_2$***); **^{13}C NMR (101 MHz, DMSO-d$_6$, 298 K)** δ 147.8 (Ar***C***-NO_2), 140.0 (Ar***C***), 133.8 (Ar***C***H), 131.0 (Ar***C***H), 127.2 (Ar***C***H), 122.5 (Ar***C***H). The spectral data agrees with the literature.[S3]

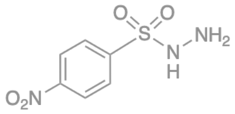

4-Nitrobenzenesulfonohydrazide (S4). A water solution (700 mL) of hydrazine monohydrate (88.5 g, 1.75 mol, 10 equiv) was cooled down to 5 °C, and 4-nitrobenzenesulfonyl chloride (40.0 g, 0.18 mol, 1 equiv) was added dropwise *via* dropping funnel. After completion of the addition, the reaction mixture was further

stirred at 5 °C for 30 minutes. Then, filtration followed by high vacuum drying directly yielded the title compound from the reaction mixture as an off-white solid (35.0 g, 92% yield), which was used in the next step without further purification. **^{1}H NMR (400 MHz, DMSO-*d$_6$*, 298 K)** δ 8.78 (1H, br. s, SO_2-N***H***NH_2), 8.47-8.37 (2H, m, 2x Ar***H***), 8.09-8.01 (2H, m, 2x Ar***H***), 4.45 (2H, br. s, SO_2NH-N***H$_2$***); **^{13}C NMR (101 MHz, DMSO-*d$_6$*, 298 K)** δ 149.7 (Ar***C***-NO_2), 144.2 (Ar***C***), 129.3 (2x Ar***C***H), 124.2 (2x Ar***C***H). The spectroscopic data of the compound is consisting with the literature.[S3]

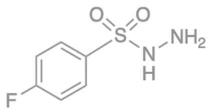

4-Fluorobenzenesulfonohydrazide (S5). The title compound was prepared through the general method E as an off-white solid (1.0 g, 47% yield) by reacting 3-fluorobenzenesulfonyl chloride (2.0 g, 10.1 mmol, 1 equiv) and hydrazine hydrate (1.3 g, 25 mol, 2.5 equiv) for 1 h. After the work-up, the crude solid product was dissolved in 25 mL of ethanol and recrystallized at –20 °C overnight, which was used in the next step without further purification. *Note: An analogous literature procedure proceeds in up to 70% yield.[S4] We attribute our low yield due to the impurities of the commercial sulfonyl chloride used as starting material.* **^{1}H NMR (400 MHz, DMSO-*d$_6$*, 298 K)** δ 8.41 (1H, br. s, SO_2N***H***-NH_2), 7.90-7.81 (2H, m, 2x Ar***H***), 7.48-7.39 (2H, m, 2x Ar***H***), 4.15 (2H, br. s, SO_2NH-N***H$_2$***); **^{13}C NMR (101 MHz, DMSO-*d$_6$*, 298 K)** δ 164.3 (Ar***C***-F, d, *J*=250.6 Hz), 134.6 (Ar***C***, d, *J*=3.0 Hz), 130.6 (2x Ar***C***H, d, *J*=9.7 Hz), 116.1 (2x Ar***C***H, d, *J*=22.6 Hz); **^{19}F NMR (377 MHz, DMSO-*d$_6$*, 298 K)** δ -107.0 (m). The spectroscopic data of the compound is consisting with the literature.[S4]

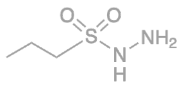

Propane-1-sulfonohydrazide (S6). The title compound was obtained through the general method E as a viscous oil (16.8 g, 87% yield) by reacting *n*-propylsulfonyl chloride (20 g, 0.14 mol, 1 equiv) and hydrazine hydrate (17.6 g, 0.35 mol, 2.5 equiv) at 0°C for 30 min, and it was used in the next step without further purification. **^{1}H NMR (400 MHz, DMSO-d$_6$, 298 K)** δ 7.77 (1H, br. s, SO_2-N***H***-NH_2), 4.30 (2H, br. s, SO_2-NH-N***H$_2$***), 3.06-2.97 (2H, m, C***H$_2$***-SO_2), 1.71-1.55 (2H, m, C***H$_2$***-CH_2SO_2), 0.97 (3H, app. t, *J*=7.4 Hz, C***H$_3$***-$CH_2CH_2SO_2$); **^{13}C NMR (101 MHz, DMSO-*d$_6$*, 298 K)** δ 48.6 (SO_2-***C***H_2), 16.6 (SO_2CH_2-***C***H_2), 12.8 ($SO_2CH_2CH_2$-***C***H_3).The spectral data is in accordance with the literature.[S5]

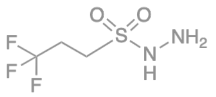

3,3,3-trifluoropropane-1-sulfonohydrazide (S7). The title compound was prepared through the general method E (0.47 g, 96% yield) by reacting 3,3,3-trifluoropropane-1-sulfonyl chloride (0.5 g, 2.54 mmol, 1 equiv) and hydrazine hydrate (0.32 g, 6.4 mmol, 2.5 equiv) at room temperature for 2 h, and it was used in the next step without further purification. **^{1}H NMR (400 MHz, DMSO-*d$_6$*, 298 K)** δ 8.14 (1H, br. s SO_2-N***H***-NH_2), 4.52 (2H, br. s, SO_2-NH-N***H$_2$***), 3.37-3.25 (2H, m, C***H$_2$***-SO_2), 2.73-2.57 (2H, m, C***H$_2$***-CH_2SO_2); **^{13}C NMR (126 MHz, DMSO-*d$_6$*, 298 K)** δ 126.8 (F_3***C***, q, *J*=276.4 Hz), 40.7 (SO_2-***C***H_2-, q, *J*=3.0 Hz), 28.4 (SO_2-CH_2-***C***H_2-CF_3, q, *J*=29.9 Hz); **^{19}F NMR (377 MHz, DMSO-*d$_6$*, 298 K)** δ -64.5 (t, *J*=11.0 Hz).

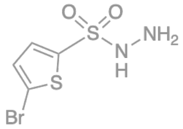

5-Bromothiophene-2-sulfonohydrazide (S8). The title compound was prepared through the general method E (1.90 g, 97% yield) by reacting 5-bromothiophene-2-sulfonyl chloride (2.0 g, 7.65 mmol, 1 equiv) and hydrazine hydrate (0.96 g, 19.1 mmol, 2.5 equiv) at room temperature for 1 h, and it was used in the next step without further purification. **^{1}H NMR (400 MHz, DMSO-*d$_6$*, 298 K)** δ 8.63 (1H, br. s, SO_2-N***H***-NH_2), 7.41 (1H, d, *J*=4.0 Hz, Het***H***), 7.36 (1H, d, *J*=4.0 Hz, Het***H***), 4.38 (2H, br. s, SO_2-NH-N***H$_2$***); **^{13}C NMR (126 MHz, DMSO-*d$_6$*, 298 K)** δ 139.6 (Het***C***), 133.1 (Het***C***H), 131.1 (Het***C***H), 118.7 (Het***C***-Br).

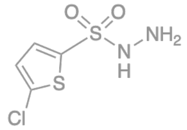

5-Chlorothiophene-2-sulfonohydrazide (S9). The title compound was prepared *via* the general method E as a yellowish solid (3.74 g, 98% yield) by reacting 5-chlorothiophene-2-sulfonyl chloride (4.0 g, 18 mmol, 1 equiv) and hydrazine hydrate (4.1 g, 45 mmol, 2.5 equiv) at room temperature for 1 h, and it was used in the next step without further purification.

^{1}H NMR (400 MHz, DMSO-*d$_6$*, 298 K) δ 7.50 (1H, d, *J*=4.0 Hz, Het***H***), 6.99 (1H, d, *J*=4.0 Hz, Het***H***), 5.97 (1H, br. s, SO_2-N***H***-NH_2), 3.75 (2H, br. s, SO_2-NH-N***H$_2$***); **^{13}C NMR (126 MHz, DMSO-*d$_6$*, 298 K)** δ 139.1 (Het***C***), 134.6 (Het***C***), 133.8 (Het***C***H), 127.3 (Het***C***H).

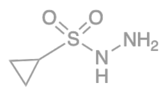

Cyclopropane-1-sulfonyl hydrazide (S10). The title compound was synthesized according to the general method E as a white solid (2.1 g, 35% yield) by reacting cyclopropane-1-sulfonyl chloride (5.0 g, 35.6 mmol, 1 equiv) and hydrazine hydrate (5.3 g, 106 mmol, 3.0 equiv) at room temperature for 2 h. Purification of the crude was carried out by flash column chromatography purification (SiO_2, DCM-MeOH, 20 :1). **^{1}H NMR (400 MHz, DMSO-*d₆*, 298 K)** δ 7.81 (1H, br. s, SO_2-N***H***-NH_2), 4.31 (2H, br. s, SO_2-NH-N***H₂***), 2.62 (1H, m, C***H***), 1.07-0.86 (4H, m, 2x C***H₂***); **^{13}C NMR (126 MHz, DMSO-*d₆*, 298 K)** δ 26.4 (***C***H), 4.9 (2x ***C***H_2). The spectral data is in accordance with the literature.[S6]

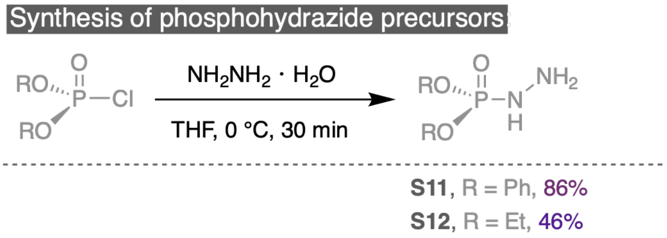

Phosphohydrazidic acid diphenyl ester (S11). Hydrazine hydrate (0.75 g, 14.9 mmol, 2 equiv) was added dropwise to a EtOH solution (150 mL) of diphenylchlorophosphate (2.0 g, 7.5 mmol, 1 equiv) at -15 °C, and the reaction mixture was allowed to stir for 2 h at -15 °C. Then, the mixture was partitioned between ethyl acetate and water, and the aqueous phase was extracted (2x) with ethyl acetate. The combined organic extracts were washed with brine and evaporated to afford the title compound (1.69 g, 86% yield) as a white solid, which was used without further purification in the next step. **^{1}H NMR (400 MHz, DMSO-*d₆*, 298 K)** δ 7.43-7.35 (4H, m, 4x Ar***H***), 7.30-7.24 (4H, m, 4x Ar***H***), 7.23-7.17 (2H, m, 2x Ar***H***), 6.88 (1H, app. d, *J*=38.4 Hz, N***H***-NH_2), 4.01 (2H, app. d, *J*=8.5 Hz, NH-N***H₂***); **^{13}C NMR (126 MHz, DMSO-*d₆*, 298 K)** δ 150.6 (2x Ar***C***, d, *J*=6.4 Hz), 129.7 (4x Ar***C***H), 124.7 (2x Ar***C***H), 120.5 (4x Ar***C***H); **^{31}P NMR (202 MHz, DMSO-*d₆*, 298 K)** δ 0.89 (dt, *J*=38.3, 8.6 Hz). This spectroscopic data agrees with the literature.[S7]

Phosphohydrazidic acid diethyl ester (S12). The title compound was obtained as a colourless oil (0.37 g, 46% yield) by reacting diethylphosphochloridate (0.84 g, 4.84 mmol, 1 equiv) with hydrazine hydrate (0.49 g, 9.7 mmol, 2 equiv), and used without further purification. **^{1}H NMR (400 MHz, DMSO-*d₆*, 298 K)** δ 6.02 (1H, app. d, *J*=31.3 Hz, N***H***-NH_2), 3.92 (4H, app. dq, *J*=7.8, 7.1 Hz, 2x C***H₂***), 3.75(2H, br. s, NH-N***H₂***), 1.21 (6H, app. td, *J*=7.1, 0.7 Hz, 2x C***H₃***); **^{31}P{^{1}H} NMR (162 MHz, DMSO-*d₆*, 298 K)** δ 9.5 (s). This spectroscopic data agrees with the literature.[S8]

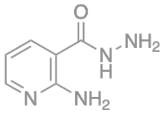

2-Aminonicotinohydrazide (S13). Hydrazine hydrate (3.0 ml, 62.4 mmol, 10 equiv) was added to a EtOH solution (25 mL) of ethyl-2-aminonicotinate (1.09 g, 6.24 mmol, 1 equiv). The suspension was stirred at 90°C for 15 h. Then, the mixture was cooled down to room temperature and the formed precipitate was filtered. The resulting filtrate layer was kept at -20 °C for 5 h, and a second precipitate arose which was filtered. Both precipitate crops were combined and dried to afford the title compound (0.62 g, 65% yield) as a white solid which was used in the next step without further purification. **^{1}H NMR (400 MHz, DMSO-*d₆*, 298 K)** δ 9.66 (1H, br. s, N***H***-NH_2), 8.05 (1H, dd, *J*=4.8, 1.8 Hz, Ar***H***), 7.80 (1H, dd, *J*=7.7, 1.9 Hz, Ar***H***), 6.98 (2H, br. s, ArN***H₂***), 6.54 (1H, dd, *J*=7.7, 4.8 Hz, Ar***H***), 4.43 (2H, s, br., NH-N***H₂***); **^{13}C NMR (101 MHz, DMSO-*d₆*, 298 K)** δ 167.1 (Ar***C***=O), 158.6 (Ar***C***-NH_2), 151.1 (Ar***C***H), 135.9 (Ar***C***H), 111.3 (Ar***C***H), 108.6 (Ar***C***).

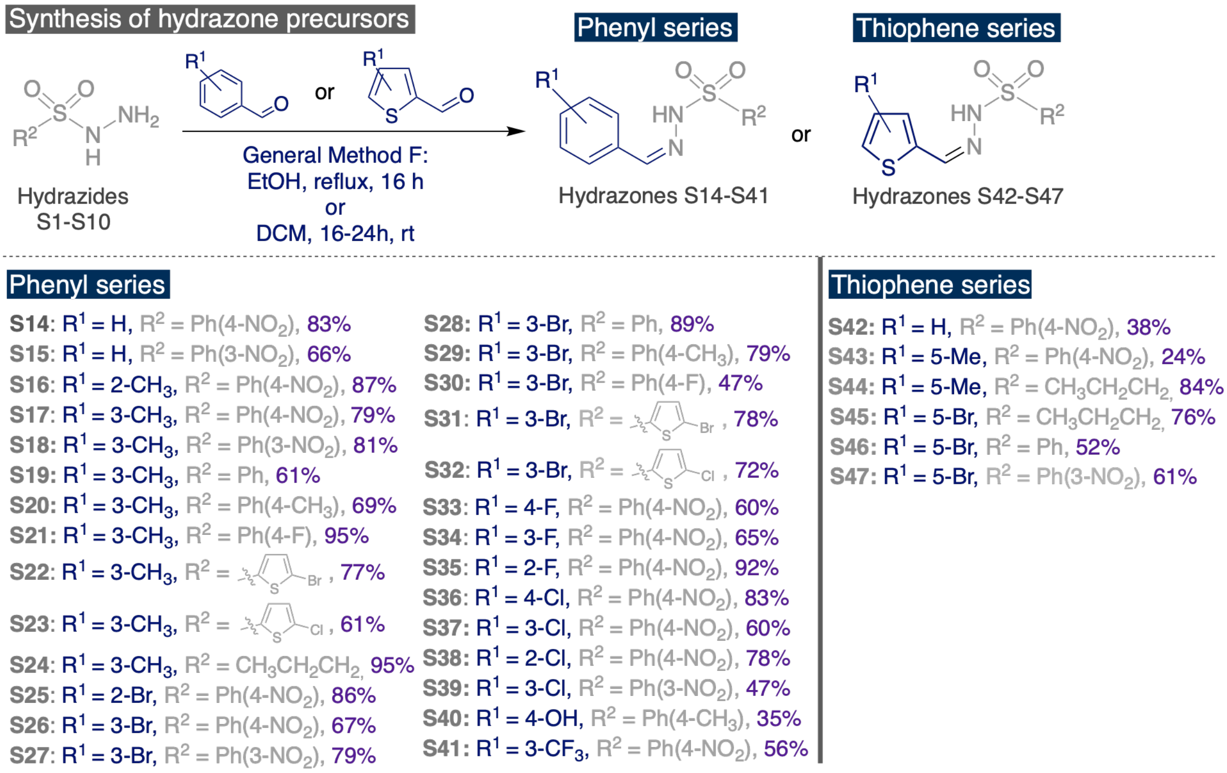

General Method F for the synthesis of intermediate hydrazones: The corresponding hydrazide **S1-10** (5.65 mmol, 1 equiv) was dissolved in 14 ml of ethanol and argon was bubbled through the solution. Then, the corresponding aldehyde (5.65 mmol, 1 equiv) was added and the mixture was refluxed for 16 h. Subsequently, the mixture was allowed to cool down to room temperature and the resulting hydrazone was isolated as it is described. Unless, otherwise noted, the crude product was used without further purification in the next step.

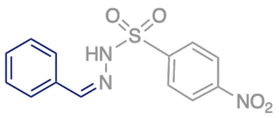

N'*-Benzylidene-4-nitrobenzenesulfonohydrazide S14** was prepared according to the general method F by reacting 4-nitrophenylsulfonohydrazide **S4** (16.0 g, 74.0 mmol, 1 equiv) and benzaldehyde (7.82 g, 74.0 mmol, 1 equiv) in 75 ml of ethanol for 4 h at 80 °C. After allowing the reaction mixture to cool down to room temperature, a precipitate arose, which was filtered rinsing with cold ethanol and dried to afford 18.6 g of **S14** as a white solid (83% yield). **^{1}H NMR (400 MHz, DMSO-*d_6*, 298 K)** δ 11.86 (1H, br. s, C=N-NH***), 8.47-8.38 (2H, m, 2x Ar***H***), 8.18-8.09 (2H, m, 2x Ar***H***), 7.97 (1H, s, ***H***C=N-NH), 7.63-7.53 (2H, m, 2x Ar***H***), 7.43-7.34 (3H, m, 3x Ar***H***); **^{13}C NMR (101 MHz, DMSO-*d_6*, 298 K)** δ 150.0 (Ar***C***-NO_2), 148.3 (***C***=N-NH), 144.2 (Ar***C***), 133.3 (Ar***C***), 130.4 (Ar***C***H), 128.8 (2x Ar***C***H), 128.8 (2x Ar***C***H), 126.9 (2x Ar***C***H), 124.6 (2x Ar***C***H).

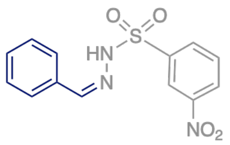

N'*-Benzylidene-3-nitrobenzenesulfonohydrazide S15** was synthesized according to the general method F by reacting 3-nitrophenylsulfonohydrazide **S3** (1.23 g, 5.65 mmol, 1 equiv) and benzaldehyde (0.6 g, 5.65 mmol, 1 equiv) in 14 ml of ethanol for 16 h at 80 °C. Then, the reaction mixture was cooled down to -20 °C for 3 h. The resulting precipitate was quickly filtered and rinsed with cold ethanol and dried under high vacuum to afford 1.14 g of **S15** as an off-white solid (66% yield). **^{1}H NMR (400 MHz, DMSO-*d_6*, 298 K)** δ 11.80 (1H, br. s, C=N-NH***), 8.59 (1H, t, *J*=2.0 Hz, Ar***H***), 8.49 (1H, ddd, *J*=8.3, 2.3, 1.0 Hz, Ar***H***), 8.32 (1H, ddd, *J*=7.9, 1.7, 1.0 Hz, Ar***H***), 7.97 (1H, s, ***H***C=N-NH), 7.93 (1H, t, *J*=7.1 Hz, Ar***H***), 7.61-7.54 (2H, m, 2x Ar***H***), 7.43-7.34 (3H, m, 3x Ar***H***); **^{13}C NMR (101 MHz, DMSO-*d_6*, 298 K)** δ 148.6 (***C***=N-NH), 147.9 (Ar***C***-NO_2), 140.3 (Ar***C***), 133.3 (Ar***C***), 133.3 (Ar***C***H), 131.4 (Ar***C***H), 130.4 (Ar***C***H), 128.8 (2x Ar***C***H), 127.8 (Ar***C***H), 126.9 (2x Ar***C***H), 121.9 (Ar***C***H).

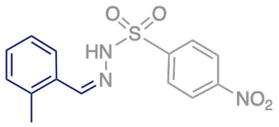

N*'-(2-Methylbenzylidene)-4-nitrobenzenesulfonohydrazide S16** was obtained according to the general method F by reacting 4-nitrobenzenesulfonohydrazide **S4** (1.81 g, 8.32 mmol, 1 equiv) and 2-methylbenzaldehyde (1.0 g, 8.32 mmol, 1 equiv) in 20 ml of ethanol for 16 h at 80 °C. After cooling down the reaction mixture, the product precipitated by gently scratching the flask walls with an iron spatula. Then, **S16** was obtained as an off-white solid (2.3 g, 87% yield) after filtration and high vacuum drying. **^{1}H NMR (400 MHz, DMSO-d_6, 298 K)** δ 11.82 (1H, br. s, C=N-NH***), 8.48-8.40 (2H, m, 2x Ar***H***), 8.20 (1H, s, ***H***C=N-NH), 8.17-8.10 (2H, m, 2x Ar***H***), 7.59 (1H, m, Ar***H***), 7.29 (1H, m, Ar***H***), 7.25-7.17 (2H, m, 2x Ar***H***), 2.33 (3H, s, ArC***H$_3$***); **^{13}C (101 MHz, DMSO-d_6, 298 K)** δ 150.0 (Ar***C***-NO$_2$), 147.3 (***C***=N-NH), 144.3 (Ar***C***), 136.8 (Ar***C***-CH$_3$), 131.3 (Ar***C***), 130.9 (Ar***C***H), 130.0 (Ar***C***H), 128.8 (2x Ar***C***H), 126.4 (Ar***C***H), 126.2 (Ar***C***H), 124.6 (2x Ar***C***H), 19.2 (ArC-***C***H$_3$).

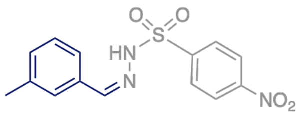

N*'-(3-Methylbenzylidene)-4-nitrobenzenesulfonohydrazide S17** was prepared according to the general method F by reacting 4-nitrophenylsulfonohydrazide **S4** (18.1 g, 83.2 mmol, 1 equiv) and 3-methylbenzaldehyde (10 g, 83.2 mmol, 1 equiv) in 205 ml of ethanol for 16 h at 80 °C. Then, the reaction mixture was allowed to cool down and concentrated to approximately 50 ml. Subsequently precipitation occurred upon cooling down to room temperature. Then, filtration rinsing twice with 15 mL of ethanol followed by high vacuum drying afforded the first crop of the title compound (17.4 g, 66% yield). A second crop of pure product (3.4g) was obtained by recrystallization overnight at -20 °C from the initial liquor solution, followed by filtration rinsing with cold ethanol. In total, 20.9 g (79% yield) of **S17** were isolated as a white solid. **^{1}H NMR (400 MHz, DMSO-d_6, 298 K)** δ 11.84 (1H, br. s, C=N-NH***), 8.46-8.39 (2H, m, 2x Ar***H***), 8.18-8.09 (2H, m, 2x Ar***H***), 7.93 (1H, s, ***H***C=N-NH), 7.37 (2H, m, 2x Ar***H***), 7.27 (1H, app. t, *J*=7.5 Hz, Ar***H***), 7.21 (1H, m, Ar***H***), 2.30 (3H, s, ArC***H$_3$***); **^{13}C NMR (101 MHz, DMSO-d_6, 298 K)** δ 149.9 (Ar***C***-NO$_2$), 148.5 (***C***=N-NH), 144.3 (Ar***C***), 138.1 (Ar***C***-CH$_3$), 133.3 (Ar***C***), 131.1 (Ar***C***H), 128.8 (2x Ar***C***H), 128.7 (Ar***C***H), 127.3 (Ar***C***H), 124.6 (2x Ar***C***H), 124.3 (Ar***C***H), 20.8 (ArC-***C***H$_3$). The spectroscopic data of this compound is consisting with the literature.[S9]

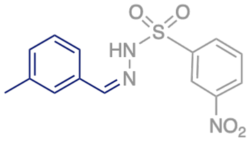

N*'-(3-Methylbenzylidene)-3-nitrobenzenesulfonohydrazide S18** was obtained according to the general method F by reacting 3-nitrobenzenesulfonohydrazide **S3** (3.62g, 16.7 mmol, 1 equiv) and 3-methylbenzaldehyde (2.0 g, 16.7 mmol, 1 equiv) in 40 ml of ethanol for 16 h at 80 °C. After cooling down to room temperature, a precipitate formed from the reaction mixture which was filtered, washed with 10 ml of ethanol and dried on high vacuum. The title compound **S18** was isolated as a yellowish solid (4.32 g, 81% yield). **^{1}H NMR (400 MHz, DMSO-d_6, 298 K)** δ 11.77 (1H, s, C=N-NH***), 8.61 (1H, app. t, *J*=2.0 Hz, Ar***H***), 8.49 (1H, ddd, *J*=8.3, 2.3, 1.1 Hz, Ar***H***), 8.31 (1H, ddd, *J*=7.9, 1.8, 1.0 Hz, Ar***H***), 7.96-7.90 (2H, m, ***H***C=N-NH and Ar***H***), 7.40 (1H, s, Ar***H***), 7.36 (1H, m, Ar***H***), 7.27 (1H, app. t, *J*=7.5 Hz, Ar***H***), 7.21 (1H, m, Ar***H***), 2.30 (3H, s, ArC***H$_3$***); **^{13}C NMR (101 MHz, DMSO-d_6, 298 K)** δ 148.7 (***C***=N-NH), 147.8 (Ar***C***-NO$_2$), 140.3 (Ar***C***), 138.1 (Ar***C***-CH$_3$), 133.3 (Ar***C***), 133.2 (Ar***C***H), 131.4 (Ar***C***H), 131.1 (Ar***C***H), 128.7 (Ar***C***H), 127.7 (Ar***C***H), 127.2 (Ar***C***H), 124.3 (Ar***C***H), 122.0 (Ar***C***H), 20.8 (ArC-***C***H$_3$).

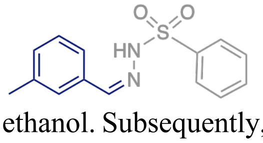

N*'-(3-Methylbenzylidene)benzenesulfonohydrazide S19** was prepared following the general method F by refluxing overnight benzenesulfonohydrazide **S1** (25.0 g, 145 mmol, 1 equiv) and 3-methylbenzaldehyde (17.4 g, 145 mmol, 1 equiv) in 145 ml of ethanol. Subsequently, extensive precipitation from the mixture arose upon cooling down to room temperature. Filtration rinsing with ethanol afforded the first crop of the title compound. A second crop was obtained from the initial liquor solution by recrystallization at 0 °C for 2 h, which was filtered rinsing with cold ethanol and dried on high vacuum. In total, 24.4 g (61% yield) of **S19** were isolated as a white solid. **^{1}H NMR (400 MHz, DMSO-d_6, 298 K)** δ 11.50 (1H, s, C=N-NH***), 7.93-7.83 (3H, m, ***H***C=N-NH and 2x Ar***H***), 7.70-7.56 (3H, m, 3x Ar***H***), 7.35 (1H, s, Ar***H***), 7.34-7.31 (1H, m, Ar***H***), 7.27 (1H, app. t, *J*=7.5 Hz, Ar***H***), 7.19 (1H, m, Ar***H***), 2.29 (3H, s, ArC***H$_3$***); **^{13}C NMR (101 MHz, DMSO-d_6, 298 K)** δ 147.3 (***C***=N-NH), 139.1 (Ar***C***), 138.0 (Ar***C***-CH$_3$), 133.6 (Ar***C***), 133.0 (Ar***C***H), 130.8 (Ar***C***H), 129.2 (2x Ar***C***H), 128.7 (Ar***C***H), 127.2 (Ar***C***H), 127.1 (2x Ar***C***H), 124.0 (Ar***C***H), 20.8 (ArC-***C***H$_3$).

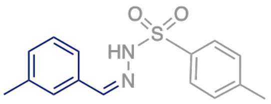

4-Methyl-*N'*-(3-methylbenzylidene)benzenesulfonohydrazide S20 was prepared according to the general method F by refluxing overnight 4-methylbenzenesulfonohydrazide **S2** (3.1 g, 16.6 mmol, 1 equiv) and 3-methylbenzaldehyde (2.0 g, 16.6 mmol, 1 equiv) in 40 ml of ethanol. After cooling down to room temperature, the ethanol was evaporated, and the remaining oil was suspended in 50 ml of diethyl ether and shaken. Then, a precipitate arose which was filtered and dried on vacuum to afford 2.3 g of **S20** as first crop. Subsequently, the filtrate was kept at -20 °C overnight and a second crop arose which was filtered and dried. In total, 3.3 g (69% yield) of **S20** were isolated as a white solid. **^{1}H NMR (400 MHz, DMSO-d$_6$, 298 K)** δ 11.42 (1H, br. s, C=N-N***H***), 7.88 (1H, s, ***H***C=N-NH), 7.80-7.72 (2H, m, 2x Ar***H***), 7.43-7.37 (2H, m, 2x Ar***H***), 7.37-7.31 (2H, m, 2x Ar***H***), 7.26 (1H, app. t, J=7.5 Hz, Ar***H***), 7.19 (1H, m, Ar***H***), 2.35 (3H, s, ArC***H*$_3$**), 2.29 (3H, s, ArC***H*$_3$**); **^{13}C NMR (101 MHz, DMSO-d$_6$, 298 K)** δ 147.1 (***C***=N-NH), 143.4 (Ar***C***), 138.0 (Ar***C***-CH$_3$), 136.2 (Ar***C***-CH$_3$), 133.6 (Ar***C***), 130.8 (Ar***C***H), 129.6 (2x Ar***C***H), 128.7 (Ar***C***H), 127.2 (2x Ar***C***H), 127.1 (Ar***C***H), 124.0 (Ar***C***H), 21.0 (Ar***C***H$_3$), 20.8 (ArC-***C***H$_3$).

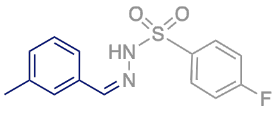

4-Fluoro-*N'*-(3-methylbenzylidene)benzenesulfonohydrazide S21 was prepared following the general method F by reacting 4-fluorobenzenesulfonohydrazide **S5** (400 mg, 2.10 mmol, 1 equiv) and 3-methylbenzaldehyde (253 mg, 2.10 mmol, 1 equiv) in 5 ml of ethanol for 16 h at 70 °C. Then, the reaction mixture was allowed to cool down to room temperature, the solvent was evaporated, and the remaining solid was dried on vacuum to afford 584 mg (95% yield) of **S21** with 95 % purity as an off-white solid. Out attempts to crystallize **S21** were not successful, and the compound was used as such in the next step. **^{1}H NMR (400 MHz, DMSO-*d$_6$*, 298 K)** δ 11.51 (1H, s, C=N-N***H***), 7.97-7.91 (2H, m, 2x Ar***H***), 7.89 (1H, s, ***H***C=N-NH), 7.50-7.41 (2H, m, 2x Ar***H***), 7.39-7.32 (2H, m, 2x Ar***H***), 7.28 (1H, app. t, *J*=7.5 Hz, Ar***H***), 7.21 (1H, app. d, *J*=7.5 Hz, Ar***H***), 2.30 (3H, s, ArC***H*$_3$**); **^{19}F{^{1}H} NMR (377 MHz, DMSO-*d$_6$*, 298 K)** δ -105.8 (s).

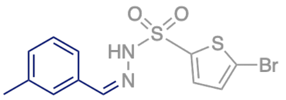

5-Bromo-*N'*-(3-methylbenzylidene)thiophene-2-sulfonohydrazide S22 was obtained *via* the general method F by refluxing overnight 5-bromothiophene-2-sulfonohydrazide **S8** (2.14 g, 8.32 mmol, 1 equiv) and 3-methylbenzaldehyde (1.0 g, 8.32 mmol, 1 equiv) in 20 ml of ethanol. The mixture was cooled down to 0 °C, filtered, washed with ethanol, and dried on high vacuum to afford the title compound (2.29 g, 77% yield) as an off-white solid. **^{1}H NMR (400 MHz, DMSO-*d$_6$*, 298 K)** δ 11.74 (1H, br. s, C=N-N***H***), 7.94 (1H, s, ***H***C=N-NH), 7.53 (1H, d, *J*= 4.0 Hz, Het***H***), 7.47-7.39 (2H, m, 2x Ar***H***), 7.36 (1H, d, *J*=4.0 Hz, Het***H***), 7.31 (1H, app.t, *J*=7.5 Hz, Ar***H***), 7.24 (1H, app. d, *J*=7.5 Hz, Ar***H***), 2.32 (3H, s, ArC***H*$_3$**); **^{13}C (101 MHz, DMSO-*d$_6$*, 298 K)** δ 148.9 (***C***=N-NH), 140.5 (Het***C***), 138.6 (Ar***C***-CH$_3$), 133.9 (Ar***C***), 133.8 (Het***C***H), 131.8 (Het***C***H), 131.6 (Ar***C***H), 129.3 (Ar***C***H), 127.9 (Ar***C***H), 124.7 (Ar***C***H), 120.2 (Het***C***-Br), 21.3 (ArC-***C***H$_3$).

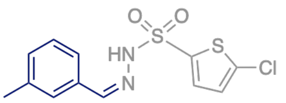

5-Chloro-*N'*-(3-methylbenzylidene)thiophene-2-sulfonohydrazide S23 was prepared *via* the general method F by refluxing overnight 5-chlorothiophene-2-sulfonohydrazide **S9** (1.77 g, 8.32 mmol, 1 equiv) and 3-methylbenzaldehyde (1.0 g, 8.32 mmol, 1 equiv) in 20 ml of ethanol. The reaction mixture was cooled down to 0 °C, filtered, washed with 5 ml of ethanol, and dried to afford the title compound (1.61 g, 61% yield) as an off-white solid. **^{1}H NMR (400 MHz, DMSO-*d$_6$*, 298 K)** δ 11.75 (1H, s, C=N-N***H***), 7.94 (1H, s, ***H***C=N-NH), 7.59 (1H, d, *J*=4.1 Hz, Het***H***), 7.47-7.39 (2H, m, 2x Ar***H***), 7.32 (1H, app. t, *J*=7.5 Hz, Ar***H***), 7.28 (1H, d, *J*=4.1 Hz, Het***H***), 7.25 (1H, app. d, *J*=7.5 Hz, Ar***H***), 2.32 (3H, s, ArC***H*$_3$**); **^{13}C NMR (101 MHz, DMSO-*d$_6$*, 298 K)** δ 148.5 (***C***=N-NH), 138.1 (Ar***C***-CH$_3$), 137.3 (Het***C***), 135.8 (Het***C***-Cl), 133.4 (Ar***C***), 132.6 (Het***C***H), 131.1 (Ar***C***H), 128.8 (Ar***C***H), 128.0 (Ar***C***H), 127.4 (Het***C***H), 124.2 (Ar***C***H), 20.9 (ArC-***C***H$_3$).

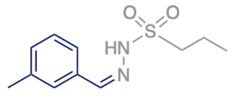

***N'*-(3-Methylbenzylidene)propane-1-sulfonohydrazide S24** was yielded *via* the general method F by refluxing overnight propane-1-sulfonohydrazide **S6** (10.0 g, 72.4 mmol, 1 equiv) and 3-methylbenzaldehyde (8.7 g, 72.4 mmol, 1 equiv) in 72 ml of ethanol. The reaction mixture was allowed to cool down to room temperature and concentrated in *vacuo*. The remaining solid was suspended in 125 mL of hexanes, and the solution was stirred for 30 min. The resulting precipitate was filtered, washed with hexanes, and dried to afford the title compound (16.5 g, 95% yield) as a

white solid. **^{1}H NMR (500 MHz, DMSO-*d*$_6$, 298 K)** δ 11.14 (1H, s, C=N-N***H***), 7.96 (1H, s, ***H***C=N-NH), 7.47 (1H, s, Ar***H***), 7.42 (1H, app. d, *J*=7.6 Hz, Ar***H***), 7.31 (1H, t, *J*=7.6 Hz, Ar***H***), 7.22 (1H, app. d, *J*=7.5 Hz, Ar***H***), 3.22-3.13 (2H, m, SO_2-C***H$_2$***), 2.33 (3H, s, ArC***H$_3$***), 1.76-1.64 (2H, m, SO_2CH_2-C***H$_2$***), 0.98 (3H, app. t, *J*=7.5 Hz, $SO_2CH_2CH_2$-C***H$_3$***); **^{13}C NMR (126 MHz, DMSO-*d*$_6$, 298 K)** δ 146.3 (***C***=N-NH), 138.0 (Ar***C***-CH_3), 133.8 (Ar***C***), 130.7 (Ar***C***H), 128.7 (Ar***C***H), 127.0 (Ar***C***H), 124.2 (Ar***C***H), 51.9 (SO_2-***C***H_2), 20.8 (ArC-***C***H_3), 16.6 (SO_2CH_2-***C***H_2), 12.6 ($SO_2CH_2CH_2$-***C***H_3).

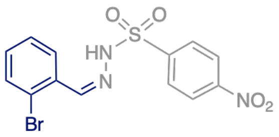

N*'-(2-Bromobenzylidene)-4-nitrobenzenesulfonohydrazide S25** was obtained according to the general method F by refluxing overnight 4-nitrobenzenesulfonohydrazide **S4** (1.53 g, 7.03 mmol, 1 equiv) and 2-bromobenzaldehyde (1.3 g, 7.03 mmol, 1 equiv) in 17 ml of ethanol. The reaction mixture was allowed to cool down at room temperature. Then, the resulting precipitate was filtered rinsing with ethanol and dried on high vacuum to afford the title compound (2.33 g, 86% yield) as a yellowish solid. **^{1}H NMR (400 MHz, DMSO-*d*$_6$, 298 K)** δ 12.18 (1H, br. s, C=N-NH***), 8.47-8.40 (2H, m, 2x Ar***H***), 8.26 (1H, s, ***H***C=N-NH), 8.18-8.10 (2H, m, 2x Ar***H***), 7.76 (1H, dd, *J*=7.8, 1.8 Hz, Ar***H***), 7.64 (1H, dd, *J*=8.0, 1.2 Hz, Ar***H***), 7.41 (1H, m, Ar***H***), 7.34 (1H, ddd, *J*=8.0, 7.4, 1.8 Hz, Ar***H***); **^{13}C NMR (101 MHz, DMSO-*d*$_6$, 298 K)** δ 150.0 (Ar***C***-NO_2), 146.2 (***C***=N-NH), 144.1 (Ar***C***), 133.2 (Ar***C***H), 132.1 (Ar***C***H), 132.0 (Ar***C***), 128.8 (2x Ar***C***H), 128.2 (Ar***C***H), 127.1 (Ar***C***H), 124.7 (2x Ar***C***H), 123.3 (Ar***C***-Br). The spectroscopic data of this compound is consisting with the literature.[S9]

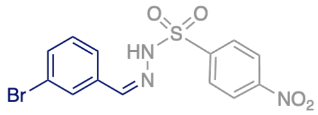

N*'-(3-Bromobenzylidene)-4-nitrobenzenesulfonohydrazide S26** was prepared through the general method F by refluxing overnight 4-nitrobenzenesulfonohydrazide **S4** (4.36 g, 20.1 mmol, 1 equiv) and 3-bromobenzaldehyde (3.71 g, 20.1 mmol, 1 equiv) in 49 ml of ethanol. After cooling down the reaction mixture, the ethanol was evaporated, and the remaining solid was suspended in 50 ml of diethyl ether. The resulting precipitate was filtered, washed with 30 ml of diethyl ether and dried on high vacuum. The resulting ether phase was transferred to a 250 ml flask and the rest of the product precipitated by gentle scratching with a spatula, which was filtered, washed with 15 ml of ether and dried in high vacuum. Both solids were combined to furnish the title compound (5.12 g, 67 % yield) as a yellowish solid. **^{1}H NMR (400 MHz, DMSO-*d*$_6$, 298 K)** δ 12.05 (1H, s, C=N-NH***), 8.47-8.39 (2H, m, 2x Ar***H***), 8.18-8.09 (2H, m, 2x Ar***H***), 7.95 (1H, s, ***H***C=N-NH), 7.76 (1H, t, *J*=1.8 Hz, Ar***H***), 7.64-7.55 (2H, m, 2x Ar***H***), 7.36 (1H, t, *J*=7.9 Hz, Ar***H***); **^{13}C NMR (101 MHz, DMSO-*d*$_6$, 298 K)** δ 150.5 (Ar***C***-NO_2), 147.1 (***C***=N-NH), 144.6 (Ar***C***), 136.2 (Ar***C***), 133.4 (Ar***C***H), 131.5 (Ar***C***H), 129.8 (Ar***C***H), 129.3 (2x Ar***C***H), 126.3 (Ar***C***H), 125.2 (2x Ar***C***H), 122.6 (Ar***C***-Br).

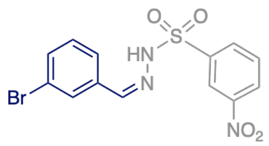

N*'-(3-Bromobenzylidene)-3-nitrobenzenesulfonohydrazide S27** was synthesized according to the general method F by refluxing overnight 3-nitrobenzenesulfonohydrazide **S3** (2.4 g, 10.8 mmol, 1 equiv) and 3-bromobenzaldehyde (2.0 g, 7.03 mmol, 1 equiv) in 27 ml of ethanol. After cooling down to room temperature the reaction mixture, the ethanol was concentrated up to 40 ml of ethanol. Then the formed precipitate was filtered rinsing with ethanol and dried on high vacuum to afford the title compound (3.3 g, 79% yield) as a yellowish solid. **^{1}H NMR (400 MHz, DMSO-*d*$_6$, 298 K)** δ 11.99 (1H, s, C=N-NH***), 8.59 (1H, t, *J*=2.0 Hz, Ar***H***), 8.50 (1H, ddd, *J*=8.3, 2.4, 1.0 Hz, Ar***H***), 8.32 (1H, m, Ar***H***), 7.99-7.88 (2H, m, ***H***C=N-NH and Ar***H***), 7.76 (1H, t, *J*=1.8 Hz, Ar***H***), 7.64-7.52 (2H, m, 2x Ar***H***), 7.35 (1H, t, *J*=7.9 Hz, Ar***H***); **^{13}C NMR (101 MHz, DMSO-*d*$_6$, 298 K)** δ 147.9 (Ar***C***-NO_2), 146.8 (***C***=N-NH), 140.2 (Ar***C***), 135.7 (Ar***C***), 133.2 (Ar***C***H), 132.9 (Ar***C***H), 131.4 (Ar***C***H), 131.0 (Ar***C***H), 129.2 (Ar***C***H), 127.8 (Ar***C***H), 125.9 (Ar***C***H), 122.1 (Ar***C***-Br), 121.9 (Ar***C***H).

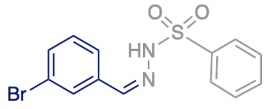

***N*'-(3-Bromobenzylidene)benzenesulfonohydrazide S28** was synthesized according to the general method F by refluxing overnight benzenesulfonohydrazide **S1** (1.4 g, 8.1 mmol, 1 equiv) and 3-bromobenzaldehyde (1.5 g, 8.1 mmol, 1 equiv) in 20 ml of ethanol. After cooling down to room temperature, the ethanol was evaporated, and the remaining

solid was suspended in 20 ml of diethyl ether. The precipitate was formed by gently scratching the walls of the flask, and then filtered, washed with 10 ml of diethyl ether and dried on high vacuum to furnish the title compound (2.45 g, 89% yield) as a yellowish solid. **^{1}H NMR (400 MHz, DMSO-*d$_6$*, 298 K)** δ 11.72 (1H, s, C=N-N***H***), 7.94-7.83 (3H, m, ***H***C=N-NH and 2x Ar***H***), 7.73 (1H, t, *J*=1.8 Hz, Ar***H***), 7.70-7.59 (3H, m, 3x Ar***H***), 7.59-7.52 (2H, m, 2x Ar***H***), 7.34 (1H, t, *J*=7.9 Hz, Ar***H***); **^{13}C NMR (101 MHz, DMSO-*d$_6$*, 298 K)** δ 145.4 (***C***=N-NH), 138.9 (Ar***C***), 136.0 (Ar***C***), 133.1 (Ar***C***H), 132.6 (Ar***C***H), 131.0 (Ar***C***H), 129.3 (2x Ar***C***H), 129.1 (Ar***C***H), 127.1 (2x Ar***C***H), 125.7 (Ar***C***H), 122.1 (Ar***C***-Br).

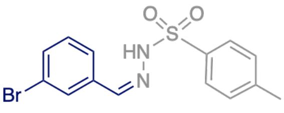

N'*-(3-Bromobenzylidene)-4-methylbenzenesulfonohydrazide S29** was prepared according to the general method F by refluxing overnight *p*-toluenesulfonohydrazide **S2** (2.7 g, 14.5 mmol, 1 equiv) and 3-bromobenzaldehyde (2.7 g, 14.5 mmol, 1 equiv) in 35 ml of ethanol. After cooling down the reaction mixture, the ethanol was concentrated *in vacuo*, then the remaining solid was suspended in 50 ml of diethyl ether and the resulting solution was kept at -20 °C overnight. A precipitate was formed, which was filtered rinsing with cold diethyl ether, and dried on high vacuum to afford 2.76 g of the title compound. The resulting ether phase was concentrated to 20 ml of diethyl ether and put to crystallize overnight at -20 °C. The formed precipitate was filtered, washed with diethyl ether and dried in high vacuum. Both solids were combined to furnish the title compound (4.04 g, 79% yield) as a white solid. **^{1}H NMR (400 MHz, DMSO-*d$_6$*, 298 K)** δ 11.63 (1H, s, C=N-NH***), 7.89 (1H, s, ***H***C=N-NH), 7.79-7.74 (2H, m, 2x Ar***H***), 7.72 (1H, t, *J*=1.8 Hz, Ar***H***), 7.60-7.52 (2H, m, 2x Ar***H***), 7.45-7.37 (2H, m, 2x Ar***H***), 7.34 (1H, t, *J*=7.9 Hz, Ar***H***), 2.35 (3H, s, ArC***H$_3$***); **^{13}C NMR (101 MHz, DMSO-*d$_6$*, 298 K)** δ 145.2 (***C***=N-NH), 143.6 (Ar***C***-CH$_3$), 136.1 (2x Ar***C***), 132.6 (Ar***C***H), 130.9 (Ar***C***H), 129.7 (2x Ar***C***H), 129.0 (Ar***C***H), 127.2 (2x Ar***C***H), 125.7 (Ar***C***H), 122.1 (Ar***C***-Br), 21.0 (ArC-***C***H$_3$). The spectroscopic data of this compound is consisting with the literature.[S10]

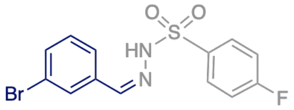

N'*-(3-Bromobenzylidene)-4-fluorobenzenesulfonohydrazide S30** was prepared according to the general method F by refluxing overnight 4-fluorobenzenesulfonohydrazide **S5** (0.5 g, 2.63 mmol, 1 equiv) and 3-bromobenzaldehyde (0.49 g, 2.63 mmol, 1 equiv) in 6 ml of ethanol. After cooling down the reaction mixture to room temperature, the ethanol was concentrated in *vacuo* and the remaining solid was suspended in 10 ml of diethyl ether. A precipitate was formed by gently scratching the walls of the flask, which was filtered rinsing with 5 ml of diethyl ether and dried to furnish the title compound (0.44 g, 47% yield) as a white solid. **^{1}H NMR (500 MHz, DMSO-*d$_6$*, 298 K)** δ 11.73 (1H, s, C=N-NH***), 7.99-7.92 (2H, m, 2x Ar***H***), 7.91 (1H, s, ***H***C=N-NH), 7.74 (1H, t, *J*=1.8 Hz, Ar***H***), 7.62-7.54 (2H, m, 2x Ar***H***), 7.46 (2H, app. t, *J*=8.8 Hz, 2x Ar***H***), 7.35 (1H, t, *J*=7.9 Hz, Ar***H***); **^{13}C NMR (126 MHz, DMSO-*d$_6$*, 298 K)** δ 164.5 (Ar***C***-F, d, *J*=251.9 Hz), 145.8 (***C***=N-NH), 136.0 (Ar***C***), 135.2 (Ar***C***, d, *J*=3.1 Hz), 132.7 (Ar***C***H), 131.0 (Ar***C***H), 130.3 (2x Ar***C***H, d, *J*=9.7 Hz), 129.2 (Ar***C***H), 125.7 (Ar***C***H), 122.1 (Ar***C***-Br), 116.5 (2x Ar***C***H, d, *J*=22.8 Hz); **^{19}F NMR (470 MHz, DMSO-*d$_6$*, 298 K)** δ -105.6 (tt, *J*=8.9, 5.2 Hz).

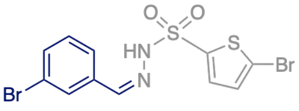

5-Bromo-*N'*-(3-bromobenzylidene)thiophene-2-sulfonohydrazide S31 was synthesized according to the general method F by refluxing overnight 5-bromothiophene-2-sulfonohydrazide **S8** (1.67 g, 6.49 mmol, 1 equiv) and 3-bromobenzaldehyde (1.2 g, 6.49 mmol, 1 equiv) in 16 ml of ethanol. After cooling down the reaction mixture, it was put to crystallize overnight at -20 °C. Then, the formed precipitate was filtered rinsing with ethanol and dried to afford 1.56 g of the title compound. The remaining ethanol filtrate was concentrated to ca. 10 ml and the crystallization at -20 °C was repeated to provide additional 0.57 g of **S31**. In total, 2.13 g (78% yield) of the title compound were isolated as an off-white solid. **^{1}H NMR (400 MHz, DMSO-*d$_6$*, 298 K)** δ 11.95 (1H, s, C=N-N***H***), 7.96 (1H, s, ***H***C=N-NH), 7.81 (1H, s, Ar***H***), 7.70-7.58 (2H, m, 2x Ar***H***), 7.55 (1H, app. d, *J*=4.1 Hz, Het***H***), 7.45-7.31 (2H, m, Het***H*** and Ar***H***); **^{13}C NMR (101 MHz, DMSO-*d$_6$*, 298 K)** δ 146.6 (***C***=N-NH), 139.8 (Het***C***), 135.8 (Ar***C***), 133.5 (Het***C***H), 132.9 (Ar***C***H), 131.4 (Het***C***H), 131.0 (Ar***C***H), 129.4 (Ar***C***H), 125.8 (Ar***C***H), 122.1 (Ar***C***-Br), 119.9 (Het***C***-Br).

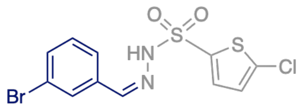

N'*-(3-Bromobenzylidene)-5-chlorothiophene-2-sulfonohydrazide S32** was prepared according to the general method F by refluxing overnight 5-chlorothiophene-2-sulfonohydrazide **S9** (1.38 g, 6.49 mmol, 1 equiv) and 3-bromobenzaldehyde (1.2 g, 6.49 mmol, 1 equiv) in 16 ml of ethanol. After cooling down the solution to room temperature, the ethanol was evaporated. The remaining solid was suspended in 20 ml of diethyl ether and led to crystallize at overnight -20 °C. The resulting precipitate was filtered rinsing with cold diethyl ether, and dried in high vacuum to afford 0.99 g of title compound. The remaining ether filtrate was concentrated to ca. 5 ml and the crystallization was repeated overnight at -20 °C to provide additional 0.79 g of **S32**. Both solids were combined to furnish the title compound (1.78 g, 72% yield) as an off-white solid. **^{1}H NMR (400 MHz, DMSO-*d*$_6$, 298 K)** δ 11.97 (1H, s, C=N-NH***), 7.96 (1H, s, ***H***C=N-NH), 7.81 (1H, s, Ar***H***), 7.65 (1H, m, Ar***H***), 7.63-7.57 (2H, m, Ar***H*** and Het***H***), 7.39 (1H, t, *J*=7.9 Hz, Ar***H***), 7.28 (1H, app. d, *J*=4.1 Hz, Het***H***); **^{13}C NMR (101 MHz, DMSO-*d*$_6$, 298 K)** δ 146.6 (***C***=N-NH), 137.1 (Het***C***), 136.0 (Ar***C***), 135.8 (Het***C***-Cl), 132.9 (Ar***C***H), 132.8 (Het***C***H), 131.0 (Ar***C***H), 129.4 (Ar***C***H), 128.0 (Het***C***H), 125.9 (Ar***C***H), 122.1 (Ar***C***-Br).

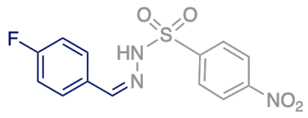

N'*-(4-Fluorobenzylidene)-4-nitrobenzenesulfonohydrazide S33** was prepared by suspending 1.75 g (8.06 mmol, 1 equiv) of 4-nitrobenzenesulfonohydrazide **S4** and 9.7 g (80.6 mmol. 10 equiv) of anhydrous $MgSO_4$ in 116 ml of anhydrous dichloromethane. Subsequently, 1.0 g (0.86 ml, 8.06 mmol, 1 equiv) of 4-fluorobenzaldehyde were added and the reaction mixture was stirred for 3 h at room temperature. Then, the inorganics were filtered off from the solution rinsing with 60 ml of DCM. The combined DCM filtrate was evaporated to afford 1.56 g (60% yield) of title compound as a yellowish solid, which was used in the next step without further purification. **^{1}H NMR (400 MHz, DMSO-*d*$_6$, 298 K)** δ 11.88 (1H, s, C=N-NH***), 8.47-8.39 (2H, m, 2x Ar***H***), 8.17-8.10 (2H, m, 2x Ar***H***), 7.97 (1H, s, ***H***C=N-NH), 7.69-7.60 (2H, m, 2x Ar***H***), 7.28-7.18 (2H, m, 2x Ar***H***); **^{13}C NMR (101 MHz, DMSO-*d*$_6$, 298 K)** δ 163.2 (Ar***C***-F, d, *J*=248.2 Hz), 150.0 (Ar***C***-NO_2), 147.2 (***C***=N-NH), 144.2 (Ar***C***), 130.0 (Ar***C***, d, *J*=3.0 Hz), 129.2 (2x Ar***C***H, d, *J*=8.7 Hz), 128.8 (2x Ar***C***H), 124.6 (2x Ar***C***H), 115.9 (2x Ar***C***H, d, *J*=22.1 Hz); **^{19}F (377 MHz, DMSO-*d*$_6$, 298 K)** δ -110.0 (tt, *J*=8.5, 5.6 Hz).

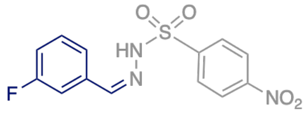

N'*-(3-Fluorobenzylidene)-4-nitrobenzenesulfonohydrazide S34** was synthesized by suspending 3.5 g (16.1 mmol, 1 equiv) of 4-nitrobenzenesulfonohydrazide **S4** and 19.3 g (162 mmol, 10 equiv) of anhydrous $MgSO_4$ in 70 ml of anhydrous dichloromethane. Subsequently, 2.0 g (1.7 ml, 16.1 mmol, 1 equiv) of 3-fluorobenzaldehyde were added and the reaction mixture was stirred for 15 h at room temperature. Then, the inorganics were filtered off from the remaining solution rinsing with 60 ml of CH_2Cl_2. The combined DCM filtrate was evaporated to afford 3.41 g (65% yield) of the title compound as a yellowish solid, which was used in the next step without further purification. **^{1}H NMR (400 MHz, DMSO-*d*$_6$, 298 K)** δ 12.03 (1H, s, C=N-NH***), 8.47-8.39 (2H, m, 2x Ar***H***), 8.19-8.11 (2H, m, 2x Ar***H***), 7.97 (1H, s, ***H***C=N-NH), 7.48-7.36 (3H, m, 3x Ar***H***), 7.23 (1H, m, Ar***H***); **^{13}C NMR (101 MHz, DMSO-*d*$_6$, 298 K)** δ 162.3 (Ar***C***-F, d, *J*=244.1 Hz), 150.0 (Ar***C***-NO_2), 146.9 (***C***=N-NH, d, *J*=3.0 Hz), 144.1 (Ar***C***), 135.8 (Ar***C***, d, *J*=8.0 Hz), 130.9 (Ar***C***H, d, *J*=8.3 Hz), 128.9 (2x Ar***C***H), 124.6 (2x Ar***C***H), 123.3 (Ar***C***H, d, *J*=2.8 Hz), 117.1 (Ar***C***H, d, *J*=21.4 Hz), 113.0 (Ar***C***H, d, *J*=22.6 Hz); **^{19}F NMR (377 MHz, DMSO-*d*$_6$, 298 K)** δ -112.6 (m).

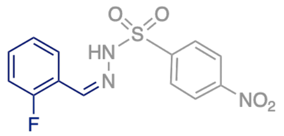

N'*-(2-Fluorobenzylidene)-4-nitrobenzenesulfonohydrazide S35** was prepared by suspending 2.3 g (10.5 mmol, 1 equiv) of 4-nitrobenzenesulfonohydrazide **S4** and 12.6 g (105 mmol, 10 equiv) of anhydrous $MgSO_4$ in 45 ml of anhydrous dichloromethane. Subsequently, 1.3 g (1.1 ml, 10.5 mmol, 1 equiv) of 2-fluorobenzaldehyde were added and the reaction mixture was stirred for 15 h at room temperature. Then, the inorganics were filtered off rinsing with 50 ml of CH_2Cl_2. The combined DCM phase was evaporated to afford 3.33 g (92% yield) of the title compound as a yellowish solid with a purity of ca. 93%, which was used in the next step without further purification. **^{1}H NMR (400 MHz, DMSO-*d*$_6$, 298 K)** δ 12.09 (1H, s, C=N-NH***), 8.46-8.39 (2H, m, 2x Ar***H***), 8.17-8.10 (3H, m, ***H***C=N-NH and 2x Ar***H***), 7.72 (1H, app. td, *J*=7.6, 1.7 Hz, Ar***H***),

7.45 (1H, m, Ar***H***), 7.28-7.17 (2H, m, 2x Ar***H***); **^{13}C NMR (101 MHz, DMSO-*d$_6$*, 298 K)** δ 160.5 (Ar***C***-F, d, *J*=250.6 Hz), 150.0 (Ar***C***-NO_2), 144.1 (Ar***C***), 141.0 (***C***=N-NH, d, *J*=4.6 Hz), 132.4 (Ar***C***H, d, *J*=8.4 Hz), 128.8 (2x Ar***C***H), 126.3 (Ar***C***H, d, *J*=2.5 Hz), 124.9 (Ar***C***H, d, *J*=3.3 Hz), 124.7 (2x Ar***C***H), 120.9 (Ar***C***, d, *J*=9.9 Hz), 116.0 (Ar***C***H, d, *J*=20.7 Hz); **^{19}F NMR (377 MHz, DMSO-*d$_6$*, 298 K)** δ -120.6 (m).

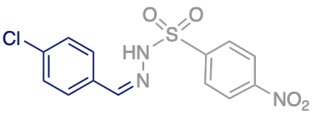

N'*-(4-Chlorobenzylidene)-4-nitrobenzenesulfonohydrazide S36** was synthesized according to the general method F by refluxing overnight 4-nitrobenzenesulfonohydrazide **S4** (1.47 g, 6.76 mmol, 1 equiv) and 4-chlorobenzaldehyde (1.0 g, 6.76 mmol, equiv) in 7 ml of ethanol. After cooling down the reaction mixture to room temperature, a precipitate was formed which was filtered rinsing with 15 ml of ethanol and dried to afford 1.9 g (83% yield) of the title compound as a yellowish solid. **^{1}H NMR (400 MHz, DMSO-*d$_6$*, 298 K)** δ 11.97 (1H, s, C=N-NH***), 8.46-8.39 (2H, m, 2x Ar***H***), 8.17-8.10 (2H, m, 2x Ar***H***), 7.97 (1H, s, ***H***C=N-NH), 7.64-7.57 (2H, m, 2x Ar***H***), 7.48-7.41 (2H, m, 2x Ar***H***); **^{13}C NMR (101 MHz, DMSO-*d$_6$*, 298 K)** δ 150.0 (Ar***C***-NO_2), 147.0 (***C***=N-NH), 144.2 (Ar***C***), 134.8 (Ar***C***-Cl), 132.3 (Ar***C***), 128.9 (2x Ar***C***H), 128.8 (2x Ar***C***H), 128.6 (2x Ar***C***H), 124.6 (2x Ar***C***H).

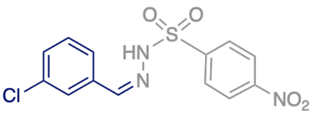

N'*-(3-Chlorobenzylidene)-4-nitrobenzenesulfonohydrazide S37** was obtained according to the general method F by refluxing overnight 4-nitrobenzenesulfonohydrazide **S4** (1.85 g, 8.5 mmol, 1 equiv) and 3-chlorobenzaldehyde (1.2 g, 8.5 mmol, 1equiv) in 20 ml of ethanol. After cooling down, the solution was concentrated in *vacuo* to ca. 10 mL, then a precipitate was formed by gently scratching the walls of the flask, which was filtered rinsing with ethanol, and dried on high vacuum to furnish the title compound (0.83 g). The remaining ethanol filtrate was put to crystallize overnight at -20 °C to afford additional 0.92 g of **S37**. Both solids were combined to furnish in total 1.75 g (72% yield) of the title compound as an off-white solid. **^{1}H NMR (400 MHz, DMSO-*d$_6$*, 298 K)** δ 12.05 (1H, s, C=N-NH***), 8.47-8.39 (2H, m, 2x Ar***H***), 8.18-8.10 (2H, m, 2x Ar***H***), 7.96 (1H, s, ***H***C=N-NH), 7.63 (1H, t, *J*=1.8 Hz, Ar***H***), 7.56 (1H, dt, *J*=7.3, 1.6 Hz, Ar***H***), 7.49-7.38 (2H, m, 2x Ar***H***); **^{13}C NMR (101 MHz, DMSO-*d$_6$*, 298 K)** δ 150.0 (Ar***C***-NO_2), 146.7 (***C***=N-NH), 144.1 (Ar***C***), 135.5 (Ar***C***-Cl), 133.6 (Ar***C***), 130.7 (Ar***C***H), 130.0 (Ar***C***H), 128.8 (2x Ar***C***H), 126.4 (Ar***C***H), 125.5 (Ar***C***H), 124.7 (2x Ar***C***H).

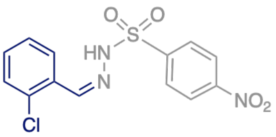

N'*-(2-Chlorobenzylidene)-4-nitrobenzenesulfonohydrazide S38** was prepared according to the general method F by refluxing overnight 4-nitrobenzenesulfonohydrazide **S4** (2.01 g, 9.25 mmol, 1 equiv) and 2-chlorobenzaldehyde (1.3 g, 9.25 mmol, 1 equiv) in 23 ml of ethanol. After cooling down the solution at room temperature, a precipitate was formed which was filtered rinsing with ethanol, and dried on high vacuum to furnish the title compound (2.44 g, 78% yield) as a white solid. **^{1}H NMR (400 MHz, DMSO-*d$_6$*, 298 K)** δ 12.18 (1H, br. s, C=N-NH***), 8.46-8.38 (2H, m, 2x Ar***H***), 8.28 (1H, s, ***H***C=N-NH), 8.17-8.09 (2H, m, 2x Ar***H***), 7.78 (1H, dd, *J*=7.7, 1.8 Hz, Ar***H***), 7.47 (1H, m, Ar***H***), 7.41 (1H, ddd, *J*=8.0, 7.6, 2.0 Hz, Ar***H***), 7.36 (1H, m, Ar***H***); **^{13}C NMR (101 MHz, DMSO-*d$_6$*, 298 K)** δ 150.0 (Ar***C***-NO_2), 144.1 (Ar***C***), 143.9 (***C***=N-NH), 133.0 (Ar***C***-Cl), 131.8 (Ar***C***H), 130.5 (Ar***C***), 129.9 (Ar***C***H), 128.8 (2x Ar***C***H), 127.7 (Ar***C***H), 126.7 (Ar***C***H), 124.7 (2x Ar***C***H). The spectroscopic data of this compound is consisting with the literature.[S9]

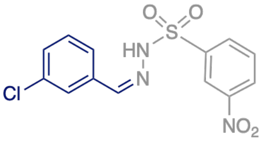

N'*-(3-Chlorobenzylidene)-3-nitrobenzenesulfonohydrazide S39** was prepared *via* the general method F by refluxing overnight 3-nitrobenzenesulfonohydrazide **S3** (1.85 g, 8.54 mmol, 1 equiv) and 3-chlorobenzaldehyde (1.2 g, 8.54 mmol, 1 equiv) in 21 ml of ethanol. After cooling down the reaction mixture at room temperature, a precipitate was formed by gently scratching the walls of the flask which was filtered rinsing with ethanol, and dried on high vacuum to afford the title compound (1.37 g, 47% yield) as an off-white solid. **^{1}H NMR (400 MHz, DMSO-*d$_6$*, 298 K)** δ 11.99 (1H, s, C=N-NH***), 8.59 (1H, t, *J*=2.0 Hz, Ar***H***), 8.50 (1H, app. d, *J*=8.3 Hz, Ar***H***), 8.32 (1H, app. d, *J*=8.0 Hz, Ar***H***), 7.95 (1H, s, ***H***C=N-NH), 7.94 (1H, m, Ar***H***), 7.62 (1H, t, *J*=1.8 Hz, Ar***H***), 7.55 (1H, dt, *J*=7.2, 1.6 Hz, Ar***H***), 7.49-7.37 (2H, m, 2x Ar***H***); **^{13}C NMR**

(101 MHz, DMSO-d_6, 298 K) δ 147.9 (Ar***C***-NO$_2$), 146.9 (***C***=N-NH), 140.2 (Ar***C***), 135.5 (Ar***C***-Cl), 133.6 (Ar***C***), 133.2 (Ar***C***H), 131.4 (Ar***C***H), 130.7 (Ar***C***H), 130.0 (Ar***C***H), 127.8 (Ar***C***H), 126.3 (Ar***C***H), 125.5 (Ar***C***H), 121.9 (Ar***C***H).

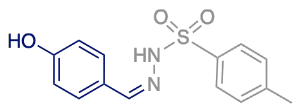

N*'-(4-Hydroxybenzylidene)-4-methylbenzenesulfonohydrazide S40** was synthesized according to the general method F by refluxing overnight *p*-toluenesulfonohydrazide **S2** (3.05 g, 16.4 mmol, 1 equiv) and 4-hydroxybenzaldehyde (2.0 g, 16.4 mmol, 1 equiv) in 20 ml of ethanol. After cooling down the reaction mixture, the ethanol was concentrated *in vacuo*, and the remaining solid was crystallized overnight at -20 °C from 30 ml of DCM. The formed precipitate was filtered, and dried on high vacuum to afford the title compound (1.78 g, 35% yield) as a yellowish solid. with a purity of ca. 93%, which was used in the next step without further purification. **^{1}H NMR (600 MHz, DMSO-d_6, 298 K)** δ 11.12 (1H, s, C=N-NH***), 9.88 (1H, s, ArO***H***), 7.80 (1H, s, ***H***C=N-NH), 7.74 (2H, app. d, *J*=8.2 Hz, 2x Ar***H***), 7.38 (4H, app. t, *J*=8.8 Hz, 4x Ar***H***), 6.76 (2H, app. d, *J*=8.5 Hz, 2x Ar***H***), 2.35 (3H, s, ArC***H$_3$***); **^{13}C NMR (151 MHz, DMSO-d_6, 298 K)** δ 159.4 (Ar***C***-OH), 147.5 (***C***=N-NH), 143.3 (Ar***C***-CH$_3$), 136.3 (Ar***C***), 129.6 (2x Ar***C***H), 128.5 (2x Ar***C***H), 127.2 (2x Ar***C***H), 124.7 (Ar***C***), 115.6 (2x Ar***C***H), 21.0 (ArC-***C***H$_3$).

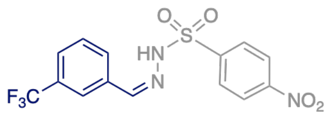

N*'-(3-(Trifluoromethyl)benzylidene)-4-nitrobenzenesulfonohydrazide S41** was prepared by suspending 4-nitrobenzenesulfonohydrazide **S4** (1.62 g, 7.5 mmol, 1 equiv) and anhydrous MgSO$_4$ (9 g, 75 mmol, 10 equiv) in 60 ml of anhydrous dichloromethane. Subsequently, 3-trifluoromethylbenzaldehyde (1.3 g, 7.5 mmol, 1 equiv) was added and the reaction mixture was allowed to stir at room temperature for 24 h. Then, the inorganics were filtered off and washed with additional 60 ml of CH$_2$Cl$_2$. The combined DCM filtrate was evaporated and the resulting solid was suspended in 10 mL of ethanol. A precipitate formed which was filtered rinsing with ethanol, and dried on high vacuum to provide 1.56 g (56% yield) of the title compound as a yellowish solid, which was used in the next step without further purification. **^{1}H NMR (400 MHz, DMSO-d_6, 298 K)** δ 12.15 (1H, s, C=N-NH***), 8.46-8.39 (2H, m, 2x Ar***H***), 8.19-8.12 (2H, m, 2x Ar***H***), 8.08 (1H, s, ***H***C=N-NH), 7.94-7.85 (2H, m, 2x Ar***H***), 7.73 (1H, m, Ar***H***), 7.62 (1H, app. t, *J*=7.7 Hz, Ar***H***); **^{13}C NMR (101 MHz, DMSO-d_6, 298 K)** δ 150.0 (Ar***C***-NO$_2$), 146.6 (***C***=N-NH), 144.2 (Ar***C***), 134.5 (Ar***C***), 130.3 (Ar***C***H), 130.0 (Ar***C***H), 129.6 (Ar***C***-CF$_3$, q, *J*=31.9 Hz), 128.8 (2x Ar***C***H), 126.6 (Ar***C***H, q, *J*=3.7 Hz), 124.7 (2x Ar***C***H), 123.9 (ArC-***C***F$_3$, q, *J*=272.5 Hz), 123.6 (Ar***C***H, q, *J*=3.9 Hz); **^{19}F{^{1}H} NMR (377 MHz, DMSO-d_6, 298 K)** δ -61.3 (s).

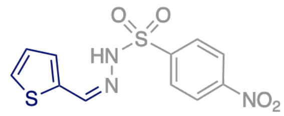

N*'-(Thiophen-2-ylmethylene)-4-nitrobenzenesulfonohydrazide S42** was synthesized according to the general method F by refluxing overnight 4-nitrobenzenesulfonohydrazide **S4** (1.94 g, 8.92 mmol, 1 equiv) and thiophene-2-carbaldehyde (1.0 g, 8.92 mmol, 1 equiv) in 22 ml of ethanol. After cooling down the reaction mixture, it was put to crystallize at -20 °C for 3 h. Then, the formed precipitate was filtered and dried on high vacuum to afford 0.87 g of the title compound. The recrystallization was repeated, by keeping the remaining ethanol filtrate at -20 °C overnight, to obtain additional 0.18 g of **S42**. In total, 1.05 g (38% yield) of the title compound was isolated as a yellow solid. **^{1}H NMR (400 MHz, DMSO-d_6, 298 K)** δ 11.80 (1H, s, C=N-NH***), 8.44 (2H, app. d, *J*=8.3 Hz, 2x Ar***H***), 8.14 (1H, s, ***H***C=N-NH), 8.09 (2H, app. d, *J*=8.3 Hz, 2x Ar***H***), 7.61 (1H, d, *J*=5.0 Hz, Het***H***), 7.39 (1H, d, *J*=3.7 Hz, Het***H***), 7.07 (1H, app. t, *J*=4.4 Hz, Het***H***); **^{13}C NMR (101 MHz, DMSO-d_6, 298 K)** δ 150.0 (Ar***C***-NO$_2$), 144.2 (Ar***C***), 143.5 (***C***=N-NH), 137.9 (Het***C***), 131.3 (Het***C***H), 129.1 (Het***C***H), 128.8 (2x Ar***C***H), 127.9 (Het***C***H), 124.6 (2x Ar***C***H).

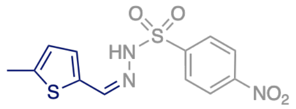

***N*'-((5-Methylthiophen-2-yl)methylene)-4-nitrobenzenesulfonohydrazide S43** was obtained according to the general method F by refluxing overnight 4-nitrobenzenesulfonohydrazide **x4** (2.3 g, 10.6 mmol, 1 equiv) and 5-methylthiophene-2-carbaldehyde (1.3 g, 10.6 mmol, 1 equiv) in 25 ml of ethanol. After cooling down the reaction mixture, the solution was concentrated up to ca. 10 ml of ethanol. Then, the formed precipitate was filtered and dried on high vacuum to afford the title compound (0.83 g, 24% yield) as

a yellow solid. **^{1}H NMR (400 MHz, DMSO-*d_6*, 298 K)** δ 11.67 (1H, s, C=N-N***H***), 8.47-8.39 (2H, m, 2x Ar***H***), 8.11-8.04 (2H, m, 2x Ar***H***), 8.03 (1H, s, ***H***C=N-NH), 7.18 (1H, d, *J*=3.5 Hz, Het***H***), 6.77 (1H, dd, *J*=3.5, 1.3 Hz, Het***H***), 2.42 (3H, s, HetC***H_3***); **^{13}C NMR (101 MHz, DMSO-*d_6*, 298 K)** δ 150.0 (Ar***C***-NO_2), 144.2 (Ar***C***), 143.7 (***C***=N-NH), 143.2 (Het***C***-CH_3), 135.6 (Het***C***), 131.7 (Het***C***H), 128.8 (2x Ar***C***H), 126.3 (Het***C***H), 124.6 (2x Ar***C***H), 15.2 (HetC-***C***H_3).

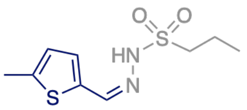

N'*-((5-Methylthiophen-2-yl)methylene)propane-1-sulfonohydrazide S44** was obtained following the general method F by refluxing overnight propane-1-sulfonohydrazide **S6** (3.0 g, 21.7 mmol, 1 equiv) and 5-methylthiophene-2-carbaldehyde (2.74 g, 21.7 mmol, 1 equiv) in 22 ml of ethanol. After cooling down the reaction mixture, it was put to crystallize at -20 °C for 3 h. Precipitation was induced by gently scratching the walls of the flask and the suspension was kept at -20 °C for an additional period of 1 h. Then, the precipitate was filtered rinsing with cold ethanol and dried on vacuum to obtain the title compound (4.47 g, 84% yield) as a white solid, which was used in the next step without further purification. **^{1}H NMR (500 MHz, DMSO-*d_6*, 298 K)** δ 10.98 (1H, s, C=N-NH***), 8.06 (1H, s, ***H***C=N-NH), 7.19 (1H, d, *J*=3.5 Hz, Het***H***), 6.80 (1H, dd, *J*=3.5, 0.9 Hz, Het***H***), 3.16-3.06 (2H, m, SO_2-C***H_2***), 2.44 (3H, s, HetC***H_3***), 1.74-1.62 (2H, m, *J*=7.5 Hz, SO_2CH_2-C***H_2***), 0.97 (3H, t, *J*=7.4 Hz, $SO_2CH_2CH_2$-C***H_3***); **^{13}C NMR (126 MHz, DMSO-*d_6*, 298 K)** δ 142.5 (Het***C***-CH_3), 141.8 (***C***=N-NH), 136.1 (Het***C***), 130.9 (Het***C***H), 126.2 (Het***C***H), 51.8 (SO_2-***C***H_2), 16.5 (SO_2CH_2-***C***H_2), 15.2 (HetC-***C***H_3), 12.6 ($SO_2CH_2CH_2$-***C***H_3).

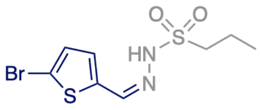

N'*-((5-Bromothiophen-2-yl)methylene)propane-1-sulfonohydrazide S45** was synthesized according to the general method F by refluxing overnight propane-1-sulfonohydrazide **S6** (3.0 g, 21.7 mmol, 1 equiv) and 5-bromothiophene-2-carbaldehyde (4.15 g, 21.7 mmol, 1 equiv) of in 22 ml of ethanol. After cooling down the reaction mixture at room temperature, a dark-red solid was filtered off from the solution which was washed with 10 ml of ethanol. Then, the resulting ethanol filtrate was put to crystallize overnight at -20 °C. As a result, a precipitate arose which was filtered and dried in high vacuum to afford the title compound (5.14 g, 76% yield) as a yellow solid. **^{1}H NMR (500 MHz, DMSO-*d_6*, 298 K)** δ 11.22 (1H, s, C=N-NH***), 8.08 (1H, s, ***H***C=N-NH), 7.25 (1H, d, *J*=3.9 Hz, Het***H***), 7.23 (1H, d, *J*=3.9 Hz, Het***H***), 3.17-3.08 (2H, m, SO_2-C***H_2***), 1.74-1.63 (2H, m, *J*=7.5 Hz, SO_2CH_2-C***H_2***), 0.97 (3H, t, *J*=7.5 Hz, $SO_2CH_2CH_2$-C***H_3***); **^{13}C NMR NMR (126 MHz, DMSO-*d_6*, 298 K)** δ 140.5 (***C***=N-NH), 140.3 (Het***C***), 131.2 (Het***C***H), 131.0 (Het***C***H), 114.4 (Het***C***-Br), 52.0 (SO_2-***C***H_2), 16.5 (SO_2CH_2-***C***H_2), 12.6 ($SO_2CH_2CH_2$-***C***H_3).

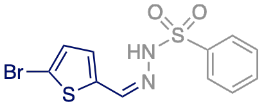

N'*-((5-Bromothiophen-2-yl)methylene)benzenesulfonohydrazide S46** was obtained *via* the general method F by refluxing overnight benzenesulfonohydrazide **S1** (1.2 g, 6.97 mmol, 1 equiv) and 5-bromothiophene-2-carbaldehyde (1.33 g, 6.97 mmol, 1 equiv) in 7 ml of ethanol. After cooling down the reaction mixture at room temperature, the black solid formed was filtered rinsing with 10 ml of ethanol. The resulting ethanol filtrate was evaporated, and the remaining solid residue was redissolved in 10 ml of ethanol. A precipitate was formed by gently scratching the walls of the flask, which was filtered and dried in high vacuum to afford the title compound (1.24 g, 52% yield) as a yellow solid. **^{1}H NMR (400 MHz, DMSO-*d_6*, 298 K)** δ 11.55 (1H, s, C=N-NH***), 8.00 (1H, s, ***H***C=N-NH), 7.87-7.79 (2H, m, 2x Ar***H***), 7.71-7.56 (3H, m, 3x Ar***H***), 7.24-7.14 (2H, m, 2x Het***H***); **^{13}C NMR (101 MHz, DMSO-*d_6*, 298 K)** δ 141.5 (***C***=N-NH), 140.1 (Het***C***), 138.7 (Ar***C***), 133.2 (Ar***C***H), 131.4 (Het***C***H), 131.2 (Het***C***H), 129.3 (2x Ar***C***H), 127.1 (2x Ar***C***H), 114.6 (Het***C***-Br).

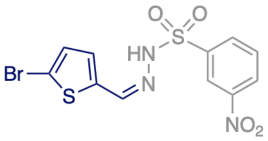

***N'*-((5-Bromothiophen-2-yl)methylene)-3-nitrobenzenesulfonohydrazide S47** was obtained *via* the general method F by refluxing overnight 3-nitrobenzenesulfonohydrazide **S4** (4.86 g, 22.4 mmol, 1 equiv) and 5-bromothiophene-2-carbaldehyde (4.28 g, 22.4 mmol, 1 equiv) in 22 ml of ethanol. After cooling down the reaction mixture at room temperature, the black solid formed was filtered-off rinsing with 10 ml of ethanol. The ethanol filtrate was poured on a flask which was gently scratched to induce precipitation and the solution was put to recrystallize for 1 h at -20 °C. The resulting

precipitate was filtered and dried in high vacuum to afford the title compound (5.3 g, 61% yield) as a yellow solid. **^{1}H NMR (500 MHz, DMSO-*d$_6$*, 298 K)** δ 11.83 (1H, br. s, C=N-N***H***), 8.54 (1H, t, *J*=2.0 Hz, Ar***H***), 8.51 (1H, ddd, *J*=8.2, 2.4, 1.1 Hz, Ar***H***), 8.25 (1H, dt, *J*=4.9, 1,4 Hz, Ar***H***), 8.05 (1H, s, ***H***C=N-NH), 7.95 (1H, t, *J*=8.0 Hz, Ar***H***), 7.24 (1H ,d, *J*=3.9 Hz, Het***H***), 7.20 (1H, d, *J*=3.9 Hz, Het***H***); **^{13}C NMR (126 MHz, DMSO-*d$_6$*, 298 K)** δ 147.8 (Ar***C***-NO_2), 142.8 (***C***=N-NH), 140.0 (Ar***C***), 139.7 (Het***C***), 133.2 (Ar***C***H), 131.9 (Ar***C***H), 131.4 (Het***C***H), 131.3 (Het***C***H), 127.8 (Ar***C***H), 121.9 (Ar***C***H), 115.1 (Het***C***-Br).

Other hydrazones

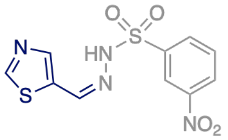

N'*-(Thiazol-5-ylmethylene)-3-nitrobenzenesulfonohydrazide S48** was prepared according to the general method F by refluxing overnight 3-nitrophenylsulfonyl hydrazide **S3** (1.4 g, 6.45 mmol, 1 equiv) and thiazole-5-carboxaldehyde (729 mg, 6.45 mmol, 1 equiv) in 15 ml of ethanol. After cooling down the reaction mixture to room temperature, crystals were formed, which were filtered rinsing with 5 ml of ethanol and dried to afford 1.32 g (66 %) of the title compound as an off-white solid. **^{1}H NMR (500 MHz, DMSO-*d$_6$*, 298 K)** δ 11.98 (1H, br. s, C=N-NH***), 9.11 (1H, s, Het***H***), 8.56 (1H, app. t, *J*=2.0 Hz, Ar***H***), 8.50 (1H, ddd, *J*=8.3, 2.2, 0.9 Hz, Ar***H***), 8.26 (1H, dt, *J*=8.0, 1.4 Hz, Ar***H***), 8.23 (1H, s, Het***H***), 8.18 (1H, s, ***H***C=N-NH), 7.93 (1H, t, *J*=8.1 Hz, Ar***H***); **^{13}C NMR (126 MHz, DMSO-*d$_6$*, 298 K)** δ 156.4 (Het***C***H), 147.9 (Ar***C***-NO_2), 146.1 (Het***C***H), 141.3 (***C***=N-NH), 140.1 (Ar***C***), 133.7 (Het***C***), 133.1 (Ar***C***H), 131.4 (Ar***C***H), 127.9 (Ar***C***H), 122.0 (Ar***C***H).

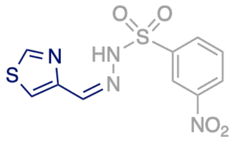

N'*-(Thiazol-4-ylmethylene)-3-nitrobenzenesulfonohydrazide S49** was synthesized following the general method F by refluxing overnight 3-nitrophenylsulfonyl hydrazide **S3** (1.4 g, 6.45 mmol, 1 equiv) and thiazole-4-carboxaldehyde (729 mg, 6.45 mmol, 1 equiv) in 15 ml of ethanol. After cooling down the reaction mixture to room temperature, crystals were formed which were filtered rinsing with 5 ml of ethanol and dried on high vacuum to afford the title compound (1.59 g, 79% yield) as a yellowish solid. **^{1}H NMR (400 MHz, DMSO-*d$_6$*, 298 K)** δ 11.93 (1H, br. s, C=N-NH***), 9.11 (1H, d, *J*=2.0 Hz, Het***H***), 8.58 (1H, app. t, *J*=2.0 Hz, Ar***H***), 8.49 (1H, ddd, *J*=8.3, 2.4, 1.0 Hz, Ar***H***), 8.30 (1H, dt, *J*=7.9, 1.3 Hz, Ar***H***), 8.10 (1H, s, ***H***C=N-NH), 8.05 (1H, d, *J*=2.0 Hz, Het***H***), 7.93 (1H, t, *J*=8.1 Hz, Ar***H***); **^{13}C NMR (101 MHz, DMSO-*d$_6$*, 298 K)** δ 155.3 (Het***C***H), 150.4 (Het***C***), 147.9 (Ar***C***-NO_2), 143.0 (***C***=N-NH), 140.4 (Ar***C***), 133.2 (Ar***C***H), 131.4 (Ar***C***H), 127.8 (Ar***C***H), 121.9 (Ar***C***H), 120.8 (Het***C***H).

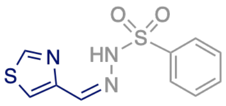

N'*-(Thiazol-4-ylmethylene)benzenesulfonohydrazide S50** was obtained according to the general method F by refluxing overnight benzenesulfohydrazide **S1** (1.1 g, 6.4 mmol, 1 equiv) and thiazole-4-carboxaldehyde (723 mg, 6.4 mmol, 1 equiv) in 15 ml of ethanol. After cooling down, the solution was evaporated, and the remaining solid was suspended in ca. 50 ml of hexane. The resulting precipitate was filtered and dried in high vacuum to afford the title compound (632 mg, 37 % yield) as an off-white solid. **^{1}H NMR (400 MHz, DMSO-*d$_6$*, 298 K)** δ 12.45 (1H, s, C=N-NH***), 9.41 (1H, s, Het***H***), 8.31 (1H, s, ***H***C=N-NH), 7.95-7.79 (2H, m, Het***H*** and Ar***H***), 7.74-7.50 (4H, m, 4x Ar***H***); **^{13}C NMR (101 MHz, DMSO-*d$_6$*, 298 K)** δ 156.3 (Het***C***H), 148.5 (Het***C***), 138.6 (Ar***C***), 135.1 (***C***=N-NH), 133.3 (Ar***C***H), 129.4 (2x Ar***C***H), 127.2 (2x Ar***C***H), 126.9 (Het***C***H).

Syntheses of *o*-formylboronic acids and other precursors for diazaborine formation

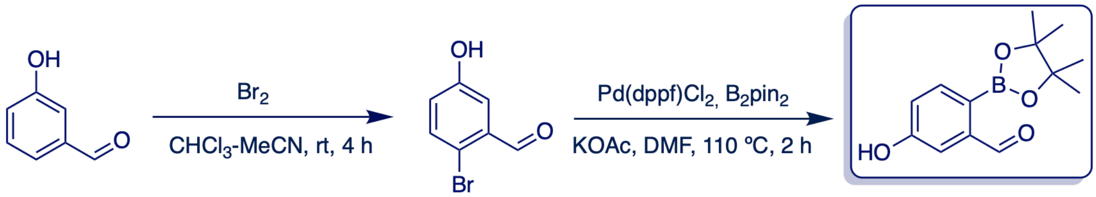

S51, 47% **S52**, 39%

2-Bromo-5-hydroxybenzaldehyde S51 was prepared by adapting an existing procedure,[S11] as follows: 3-hydroxybenzaldehyde (50.0 g, 0.41 mol, 1 equiv) was added to a chloroform-acetonitrile solution (10:1, 2.1 L) in a three-neck flask equipped with a dropping funnel. Then, a chloroform solution (205 ml) of bromine (68.7 g, 0.43 mol, 1.05 equiv) was added dropwise over a period of 30 min. The reaction mixture was further stirred for 3 h, until quenching with a $Na_2S_2O_3$ saturated aqueous solution. The layers were separated, and the aqueous layer was extracted with dichloromethane (3x 500 ml). The combined organic layers were washed with brine, dried over Na_2SO_4, and concentrated in vacuo up to ca. 250 mL of DCM. Then, recrystallization overnight at - 20 °C afforded the title compound (38.5 g, 47% yield) as an off-white solid which was used without further purification in the next step. **^{1}H NMR (500 MHz, CDCl$_3$, 298 K)** δ 10.29 (1H, s, ArC***H***O), 7.52 (1H, d, *J*=8.6 Hz, Ar***H***), 7.44 (1H, d, *J*=3.2 Hz, Ar***H***), 7.02 (1H, dd, *J*=8.6, 3.1 Hz, Ar***H***), 5.83 (1H, br s, ArO***H***); **^{13}C NMR (101 MHz, CDCl$_3$, 298 K)** δ 192.4 (Ar-***C***HO), 155.8 (Ar***C***-OH), 135.1 (Ar***C***H), 134.2 (Ar***C***), 123.6 (Ar***C***H), 117.9 (Ar***C***-Br), 115.9 (Ar***C***H). This spectroscopic data agrees with the literature.[S11]

5-Hydroxy-2-(4,4,5,5-tetramethyl-1,3,2-dioxaborolan-2-yl)benzaldehyde S52: To an anhydrous DMF solution (160 ml) of 2-bromo-5-hydroxybenzaldehyde **S51** (8 g, 40 mmol, 1 equiv) under argon was added potassium acetate (11.71 g, 120 mmol, 3 equiv), Pd(dppf)Cl_2 (0.96 g, 1.3 mmol, 0.03 equiv), and bis(pinacolato)diboron (11.1 g, 44 mmol, 1.1 equiv). Then, the resulting reaction mixture was allowed to stir at 110 °C for 2 h, when it was quenched by adding ethyl acetate and water. The layers were shaken, the organic layer was separated, dried over Na_2SO_4, and evaporated. Purification by flash column chromatography (SiO_2, EtOAc/hexanes; 1:3) yielded the title compound (3.82 g, 39% yield) as a yellow solid. **^{1}H NMR (400 MHz, CDCl$_3$, 298 K)** δ 10.65 (1H, s, ArC***H***O), 7.86 (1H, d, *J*=8.2 Hz, Ar***H***), 7.49 (1H, m, Ar***H***), 7.09 (1H, dd, *J*=8.2, 2.6 Hz, Ar***H***), 5.66 (1H, br, ArO***H***), 1.37 (12H, s, 4x C***H$_3$***); **^{13}C NMR (101 MHz, CDCl$_3$, 298 K)** δ 195.3 (Ar-***C***HO), 158.5 (Ar***C***-OH), 143.7 (Ar***C***), 138.6 (Ar***C***H), 120.5 (Ar***C***H), 113.3 (Ar***C***H), 84.4 (2x ***C***(CH_3)$_2$), 25.0 (4x C(***C***H_3)$_2$). *Note*: *the resonance corresponding of the quaternary ipso carbon directly bound to the B-OH group was not observed due to the signal broadening caused by the fast relaxation induced by* the *quadrupolar boron nucleus as well as the possible residual J-coupling to boron*; **^{11}B NMR (128 MHz, CDCl$_3$, 298 K)** δ 30.9. The spectrocopic data agree with the literature. [S12]

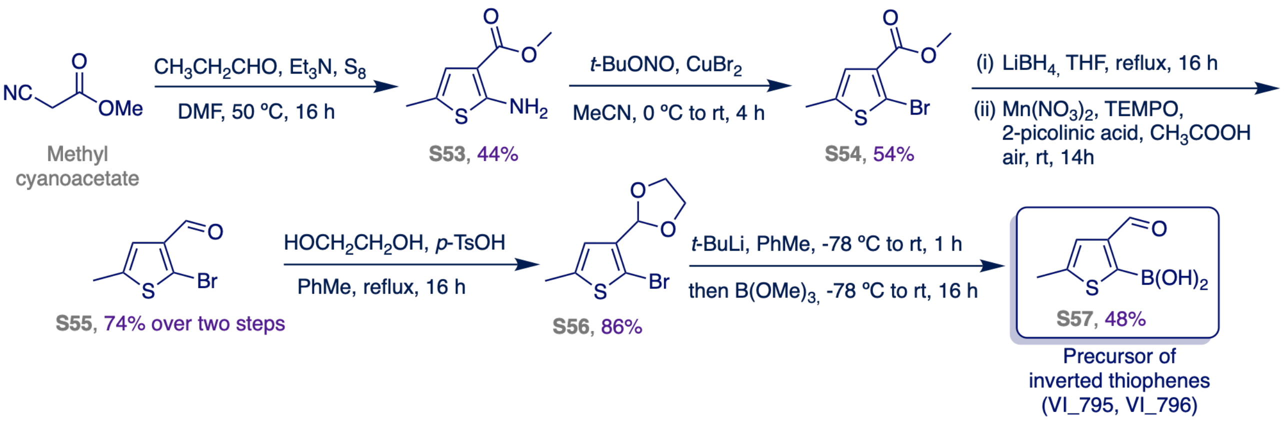

Methyl 2-amino-5-methylthiophene-3-carboxylate S53. To an anhydrous DMF solution (160 ml) of elemental sulfur (15g, 0.47 mol, 1 equiv) was sequentially added methyl cyanoacetate (46.4 g, 0.47 mol, 1 equiv), triethylamine (28.4 g, 0.28 mol, 0.6 equiv) and propionaldehyde (27.2 g, 0.47 mol, 1 equiv) under nitrogen. The reaction mixture was allowed to stir at 50 °C for 16 h. After cooling down the mixture to room temperature, 100 ml of water was added, and the mixture was thoroughly extracted with ethyl acetate. The combined organic extracts were washed with brine, dried over Na_2SO_4, filtered and evaporated. Then, the crude was suspended in *ca.* 300 ml of diethyl ether, and a precipitate was formed which was filtered washing twice with 40 mL diethyl ether and dried in high vacuum to afford the title compound (35.5 g, 44% yield) as an off-white solid . **^{1}H NMR (500 MHz, CDCl$_3$, 298 K)** δ 6.59 (1H, q, *J*=1.3 Hz, Het***H***), 5.77 (2H, br. s, HetN***H$_2$***), 3.78 (3H, s, OC***H$_3$***), 2.25 (1H, d, *J*=1.3 Hz, HetC***H$_3$***); **^{13}C NMR (126 MHz, CDCl$_3$, 298 K)** δ 165.8

(**C**O_2CH_3), 161.7 (Het**C**-NH_2), 122.6 (Het**C**H), 121.1 (Het**C**-CH_3), 106.3 (Het**C**), 51.0 (-O**C**H_3), 15.0 (HetC-**C**H_3).

***tert*-Butyl nitrite** was synthesized according to a known procedure,[S13] as follows: In a 500 ml flask fitted with a thermometer, a stirrer and a dropping funnel, 74 g of *tert*-butyl alcohol were added to a solution of 76 g of sodium nitrite in 440 ml of water. The mixture was cooled to 0 °C, and 90 ml of concentrated hydrochloric acid were added dropwise *via* the dropping funnel, so that the solution temperature did not rise above 5 °C. Once all the hydrochloric acid was added, the solution was transferred to a separatory funnel and washed with 400 ml of water. The aqueous layer was discarded, then the organic phase was washed with a 5% aqueous sodium bicarbonate solution (3x 50 ml) and water (3x50 ml). Subsequently, the organic layer was dried with calcium chloride and purified by distillation (bp: 62-64 °C). Obtained 56.5 g (55% yield) as a colourless liquid.

Methyl 2-bromo-5-methylthiophene-3-carboxylate S54: To a MeCN solution (315 ml) of copper (II) bromide (36.5 g, 0.16 mol, 1.4 equiv) at 0 °C was added dropwise *tert*-butyl nitrite (13.8 g, 0.13 mol, 1.15 equiv). The solution was stirred for 15 min, and then **S53** (20g, 0.11 mol, 1 equiv) was added portionwise. The resulting reaction mixture was allowed to warm up at room temperature and stirred for a further period of 4 h. Then, the solution was partitioned between aqueous HCl (2M, 150 ml) and ethyl acetate (150 ml). The layers were shaken and separated, and the water layer was further extracted with ethyl acetate (2x, 150 ml). The combined organic layers were dried over Na_2SO_4, filtered and evaporated. Subsequently, the crude product was purified with flash-column chromatography (SiO_2, EtOAc/Hex 1:3) to afford the title compound (14.9 g, 54% yield) as a yellow syrup. **^{1}H NMR (400 MHz, DMSO-*d$_6$*, 298 K)** δ 7.05 (1H, q, *J*=1.2 Hz, Het***H***), 3.78 (3H, s, OC***H$_3$***), 2.39 (3H, d, *J*=1.3 Hz, HetC***H$_3$***); **^{13}C NMR (101 MHz, DMSO-*d$_6$*, 298 K)** δ 161.5 (**C**O_2CH_3), 140.7 (Het**C**-CH_3), 130.2 (Het**C**), 126.8 (Het**C**H), 116.1 (Het**C**-Br), 51.8 (-O**C**H_3), 14.9 (HetC-**C**H_3).

2-Bromo-5-methylthiophene-3-carbaldehyde S55: To a dry THF solution (360 ml) of ester **S54** (14 g, 59 mmol, 1 equiv) was added slowly at room temperature a 2M THF solution of lithium borohydride (59.6 ml, 119 mmol, 2 equiv). The reaction mixture was gently refluxed overnight, then it was allowed to cool down to room temperature and partitioned between ethyl acetate and water. The layers were shaken and separated, and the organic layer was further washed with water (3x). Then, all the aqueous layers were combined and additionally extracted with ethyl acetate (3x). Subsequently, all the organic layers were combined, washed with brine, dried over Na_2SO_4, filtered and evaporated. The resulting crude was subjected to purification by flash-column chromatography (SiO_2, EtOAc/Hex 1:3) to afford (2-Bromo-5-methylthiophen-3-yl)methanol (11.1 g, 90% yield) as a white solid. **^{1}H NMR (400 MHz, $CDCl_3$, 298 K)** δ 6.75 (1H, q, *J*=1.2 Hz, Het***H***), 5.17 (1H, t, *J*=5.7 Hz, CH_2O***H***), 4.30 (2H, d, *J*=5.7 Hz, C***H$_2$***OH), 2.38 (3H, d, *J*=1.2 Hz, HetC***H$_3$***); **^{13}C NMR (101 MHz, $CDCl_3$, 298 K)** δ 142.1 (Het**C**-CH_3), 139.6 (Het**C**), 126.6 (Het**C**H), 104.1 (Het**C**-Br), 57.8 (**C**H_2OH), 15.1 (HetC-**C**H_3).

The title compound was prepared *via* oxidation of (2-Bromo-5-methylthiophen-3-yl)methanol by adapting the protocol reported by Lagerblom et al.,[S14] as follows: Manganese (II) nitrate tetrahydrate (940 mg, 3.76 mmol, 0.07 equiv), 2-picolinic acid (0.73 g, 5.9 mmol, 0.11 equiv), TEMPO (0.59 g, 3.76 mmol, 0.07 equiv) and sodium nitrate (1.82 g, 21.5 mmol, 0.4 equiv) were dissolved in 460 ml of glacial acetic acid and stirred for 5 minutes in opened-air conditions. Then, (2-Bromo-5-methylthiophen-3-yl)methanol (11.1 g, 53 mmol, 1 equiv) was added and the solution was stirred at room temperature till the reaction was completed (ca. 14 h). Then, diethyl ether (0.5 L) was added to the solution which was washed with water (3x). The aqueous layers were combined and extracted with diethyl ether. All the organic extracts were combined, washed with brine, dried over Na_2SO_4, filtered, evaporated, and dried in high vacuum. In total, 9.15 g (83% yield, 74% yield over two steps) of the title compound were afforded as a yellow solid and used directly in the next step without further purification. **^{1}H NMR (400 MHz, $CDCl_3$, 298 K)** δ 9.83 (1H, s, C***H***O), 7.01 (1H, q, *J*=1.2 Hz, Het***H***), 2.43 (3H, d, *J*=1.2 Hz, HetC***H$_3$***); **^{13}C NMR (126 MHz, $CDCl_3$, 298 K)** δ 184.8 (**C**HO), 141.5 (Het**C**-CH_3), 138.8 (Het**C**), 123.7 (Het**C**H), 122.9 (Het**C**-Br), 15.6 (HetC-**C**H_3); **HRMS (ESI)**: m/z calcd. for C_6H_5BrOS $[M+H]^+$: 204.9317, found 204.9313, error 1.859 ppm.

2-(2-Bromo-5-methylthiophen-3-yl)-1,3-dioxolane S56: To a toluene solution (120 ml) of aldehyde **S55** (6.58 g, 32.1 mmol, 1 equiv) were added ethylene glycol (2.27 g, 36.6 mmol, 1.15 equiv) and *p*-toluenesulfonic

acid monohydrate (8.3 mg, 0.04 mmol, 0.001 equiv). The resulting reaction mixture was refluxed overnight connected to a Dean-Stark apparatus for azeotropic water removal. Then, the solution was allowed to cool down to room temperature and poured into 100 ml of a 10% NaOH aqueous solution. The layers were shaken and separated, and the aqueous layer was additionally extracted with toluene. Then, the combined organic layers were washed with an aqueous ammonium chloride solution, followed by washing with brine, dried over Na_2SO_4, filtered, and dried in high vacuum to afford the title compound (7.07 g, 86% yield) as a yellow syrup which was used directly in the next step without further purification. **^{1}H NMR (600 MHz, $CDCl_3$, 298 K)** δ 6.70 (1H, q, *J*=1.2 Hz, Het***H***), 5.79 (1H, s, C***H***(OCH_2CH_2O)), 4.16-4.09 (2H, m, CH(OC$\boldsymbol{H_2}$$CH_2O$)), 4.04-3.98 (2H, m, CH($OCH_2C$$\boldsymbol{H_2}$O)), 2.40 (3H, d, *J*=1.2 Hz, HetC$\boldsymbol{H_3}$); **^{13}C NMR (126 MHz, $CDCl_3$, 298 K)** δ 140.8 (Het***C***-CH_3), 137.6 (Het***C***), 124.1 (Het***C***H), 109.4 (Het***C***-Br), 99.8 (***C***H(OCH_2CH_2O)), 65.5 (CH(O***C***H_2***C***H_2O)), 15.7 (HetC-***C***H_3); HRMS (ESI): m/z calcd. for $C_8H_{10}BrO_2S$ $[M+H]^+$: 248.9579, found 248.9581, error 0.697 ppm.

(3-Formyl-5-methylthiophen-2-yl)boronic acid S57: Acetal **S56 (**2.03 g, 8.15 mmol, 1 equiv) was dissolved in 41 ml of dry toluene and cooled down to -78 °C. Then, a *tert*-butyl lithium 1.9 M solution in pentane (8.7 ml, 16.6 mmol, 2.05 equiv) was added dropwise. After completion of the addition, the resulting reaction mixture was slowly warmed up to room temperature in 30 min and stirred for an additional hour. The solution was again cooled down to -78 °C, then trimethylborate (2.54 g, 2.7 ml, 24.5 mmol, 3 equiv) was added dropwise. The resulting reaction mixture was allowed to warm up at room temperature overnight. Then, the reaction mixture was quenched with water, stirred for 5 minutes, added a 0.5M solution of HCl, and further stirred for 10 min. The resulting solution was extracted with ethyl acetate (3x 100 ml). The combined organic layers were washed with brine, dried over Na_2SO_4, filtered and evaporated. Subsequently, the remaining solid crude was redissolved in a mixture of 10 ml of methanol and 25 ml of water. Then, the solution was evaporated at 40 °C in order to hydrolyze the corresponding methyl ester impurities of the boronic acid formed. The resulting solid was dried in high vacuum, suspended in 100 ml of toluene, stirred for 1 h, filtered and dried in vacuo to get the title compound (0.74 g, 48% yield) with ca. 90% purity as a yellow solid. **^{1}H NMR (400 MHz, $CDCl_3$, 298 K)** δ 9.80 (1H, s, C***H***O), 7.40 (2H, br. s, B(O$\boldsymbol{H}$)$_2$), 7.34 (1H, q, *J*=1.1 Hz, Het***H***), 2.57 (3H, d, *J*=1.1 Hz, HetC$\boldsymbol{H_3}$); **^{13}C NMR(101 MHz, $CDCl_3$, 298 K)** δ 189.0 (***C***HO), 147.2 (Het***C***-CH_3), 145.7 (Het***C***), 132.3 (Het***C***H), 15.2 (HetC-***C***H_3). *Note*: *the resonance corresponding of the quaternary carbon directly bound to the B-OH group was not observed due to the signal broadening caused by the fast relaxation induced by the quadrupolar boron nucleus as well as the possible residual J-coupling to boron*; **^{11}B NMR (128 MHz, $CDCl_3$, 298 K)** δ 26.08; **HRMS (ESI):** m/z calcd. for $C_6H_7BO_3S$ $[3M-3H_2O+OCH_3]^-$: 487.0503, found 487.0498, error 0.359 ppm.

Syntheses of the precursors of phosphonium precursors

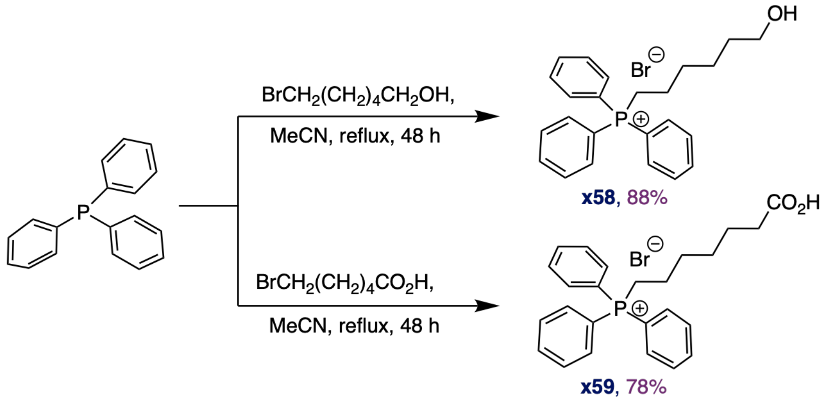

(6-Hydroxyhexyl)triphenylphosphonium bromide S58: 6-bromohexan-1-ol (3.7 g, 20.4 mmol, 1 equiv) and triphenylphosphine (5.9 g, 22.5 mmol, 1.1 equiv) were refluxed in 25 ml of dry acetonitrile for 48 h. After cooling down the reaction to room temperature, acetone was added to the reaction mixture, and the suspension

was vigorously stirred for 15 minutes, the precipitate formed was filtered rinsing twice with acetone, and dried in high vacuum to obtain the title compound (8.0 g, 88% yield) as a white solid. The spectroscopic data of the compound fully matched the reported one.[S16]

(6-Carboxyhexyl)triphenylphosphonium bromide S59: 6-bromohexanoic acid (4.1 g, 21 mmol, 1 equiv) and triphenylphosphine (6.1 g, 23 mmol, 1.1 equiv) were refluxed in 12 ml of dry acetonitrile for 48 h. After cooling down to room temperature, the reaction mixture was poured into a CH_2Cl_2:Acetone solution (1:1) with vigorous stirring for 15 min. The precipitate formed was filtered rinsing twice with acetone, and dried in high vacuum to obtain the title compound (7.5 g, 86%) as a white solid. **^{1}H NMR (400 MHz, DMSO-*d*$_6$, 298 K)** δ 12.01 (1H, br. s, $CO_2\boldsymbol{H}$), 7.95-7.87 (3H, m, 3x Ar***H***), 7.85-7.72 (12H, m, 12x Ar***H***), 3.65-3.51 (2H, m, $C\boldsymbol{H_2}$), 2.17 (2H, app. t, *J*=6.6 Hz, $C\boldsymbol{H_2}$), 1.60-1.40 (6H, m, 3x $C\boldsymbol{H_2}$); **^{13}C NMR (101 MHz, DMSO-*d*$_6$, 298 K)** δ 174.3 ($\boldsymbol{C}O_2H$), 134.9 (3x Ar***C***H, d, *J*=3.1 Hz), 133.6 (6x Ar***C***H, d, *J*=9.8 Hz), 130.2 (6x Ar***C***H, d, *J*=12.4 Hz), 118.5 (3x Ar***C***, d, *J*=85.5 Hz), 33.3 ($\boldsymbol{C}H_2$), 29.3 ($\boldsymbol{C}H_2$, d, *J*=17.1 Hz), 23.6 ($\boldsymbol{C}H_2$), 21.6 ($\boldsymbol{C}H_2$, d, *J*=4.4 Hz), 20.1 ($\boldsymbol{C}H_2$, d, *J*=50.1 Hz); **^{31}P{^{1}H} NMR (162 MHz, DMSO-*d*$_6$, 298 K)** δ 24.0.

References

[S1] Khan, K. M. et al., *Lett. Org. Chem.* **2015**, *12*, 637–644.

[S2] Tathe, A. G.; Patil, N. T. *Org. Lett.* **2022**, *24*, 4459–4463.

[S3] Tripathi, C. B.; Mukherjee, S. *Org. Lett.* **2014**, *16*, 3368–3371.

[S4] Kim, H.; Lee, S. *Eur. J. Org. Chem.* **2019**, 6951–6955.

[S5] Lv, Y.; Lai, J.; Pu, W.; Wang, J.; Han, W.; Wang, A.; Zhang, M.; Wang, X. *J. Org. Chem.* **2023**, *88*, 2034–2045.

[S6] Zhou, Z.; Liu, Q.; Huang, Z.; Zhao, Y. *Org. Lett.* **2022**, *24*, 4433–4437.

[S7] Asadi, L.; Gholivand, K.; Zare, K. Phosphorhydrazides as Urease and Acetylcholinesterase Inhibitors: Biological Evaluation and QSAR Study. *J. Iran Chem. Soc.* **2016**, *13*, 1213–1223.

[S8] Kraicheva, I., Hydrazones of phosphorohydrazidic acid diethyl ester. *Phosphorus, Sulfur Silicon Relat. Elem.* **2001**, *173*, 243–252. https://doi.org/10.1080/10426500108045273.

[S9] Cao, Z.; Yan, S.; Zhao, C.; Sun, X.; Tian, L.; Meng, X. *Tetrahedron* **2019**, *75*, 130534.

[S10] Xie, Z.; Song, Y.; Xu, L.; Guo, Y.; Zhang, M.; Li, L.; Chen, K.; Liu, X. *ChemistryOpen* **2018**, *7*, 977–983.

[S11] Kaiser, F., Schwink, L., Velder, J. and Schmalz, H.-G., *J. Org. Chem.* **2002**, *67*, 9248-9256.

[S12] Rej, S.; Chatani, N. *J. Am. Chem. Soc.* **2021**, *143*, 2920–2929.

[S13] Noyes, W. A. *Org. Synth.* **1936**, *16*, 7.

[S14] Lagerblom, K. et al. *ChemCatChem* **2018**, *10*, 2908–2914.

[S15] Cruz, C.D.; Wrigstedt, P.; Moslova, K.; Iashin, V.; Mäkkylä, H.; Ghemtio, L.; Heikkinen, S.; Tammela, P.; Perea-Buceta, J.E. *Eur. J. Med. Chem.* **2021**, *211*, 113002.

[S16] Goujon, A. et al., *J. Am. Chem. Soc.* **2019**, *141*, 8, 3380–3384.

NMR spectra (Figures S8-S648)

Diazaborines

Diazaborine 1

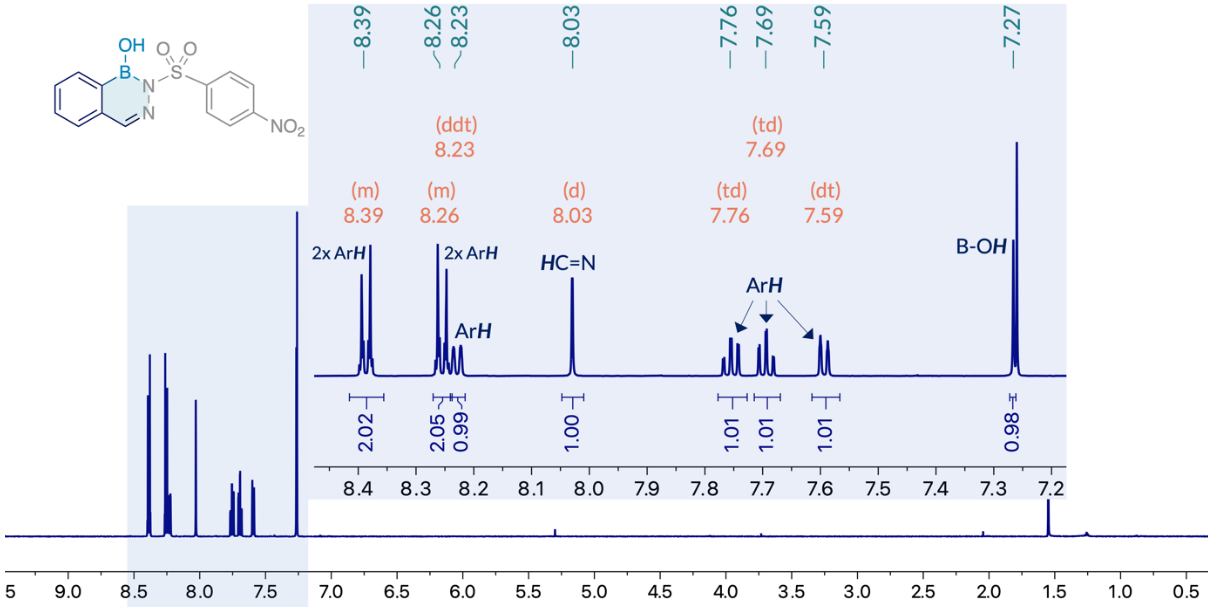

Figure S8. Diazaborine 1: ^{1}H NMR (600 MHz, $CDCl_3$, 298 K)

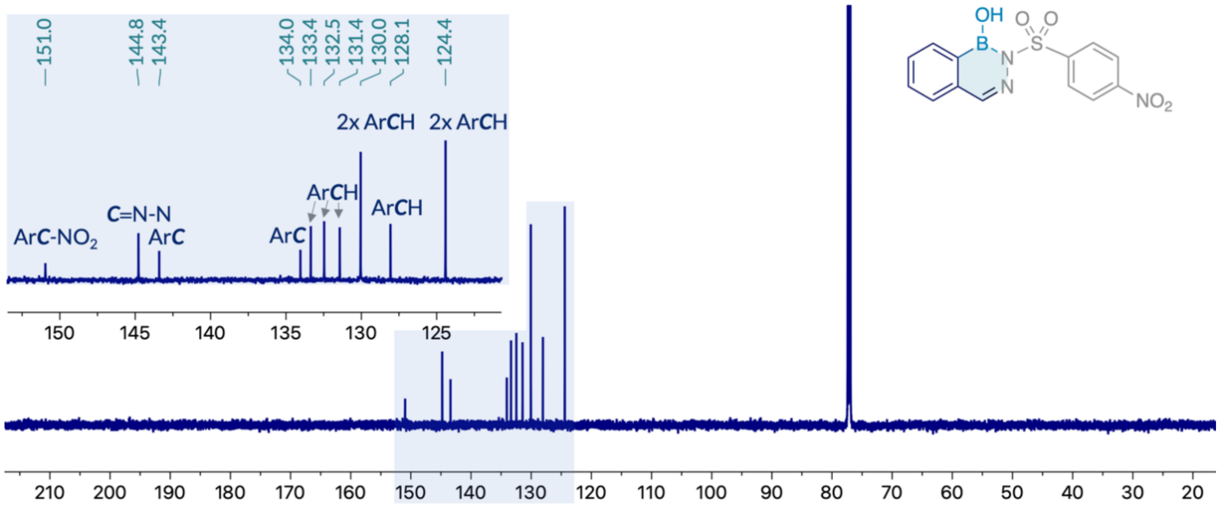

Figure S9. Diazaborine 1: ^{13}C NMR (151 MHz, $CDCl_3$, 298 K)

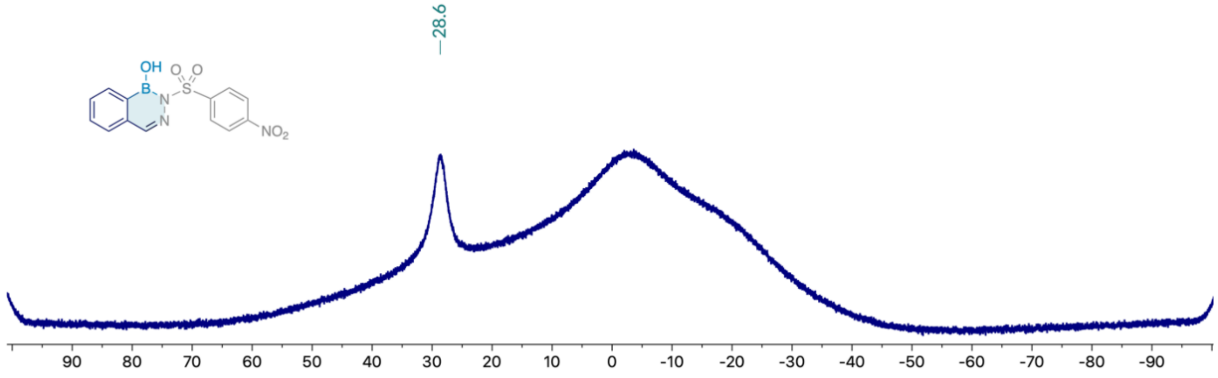

Figure S10. Diazaborine 1: ^{11}B NMR (128 MHz, $CDCl_3$, 298 K)

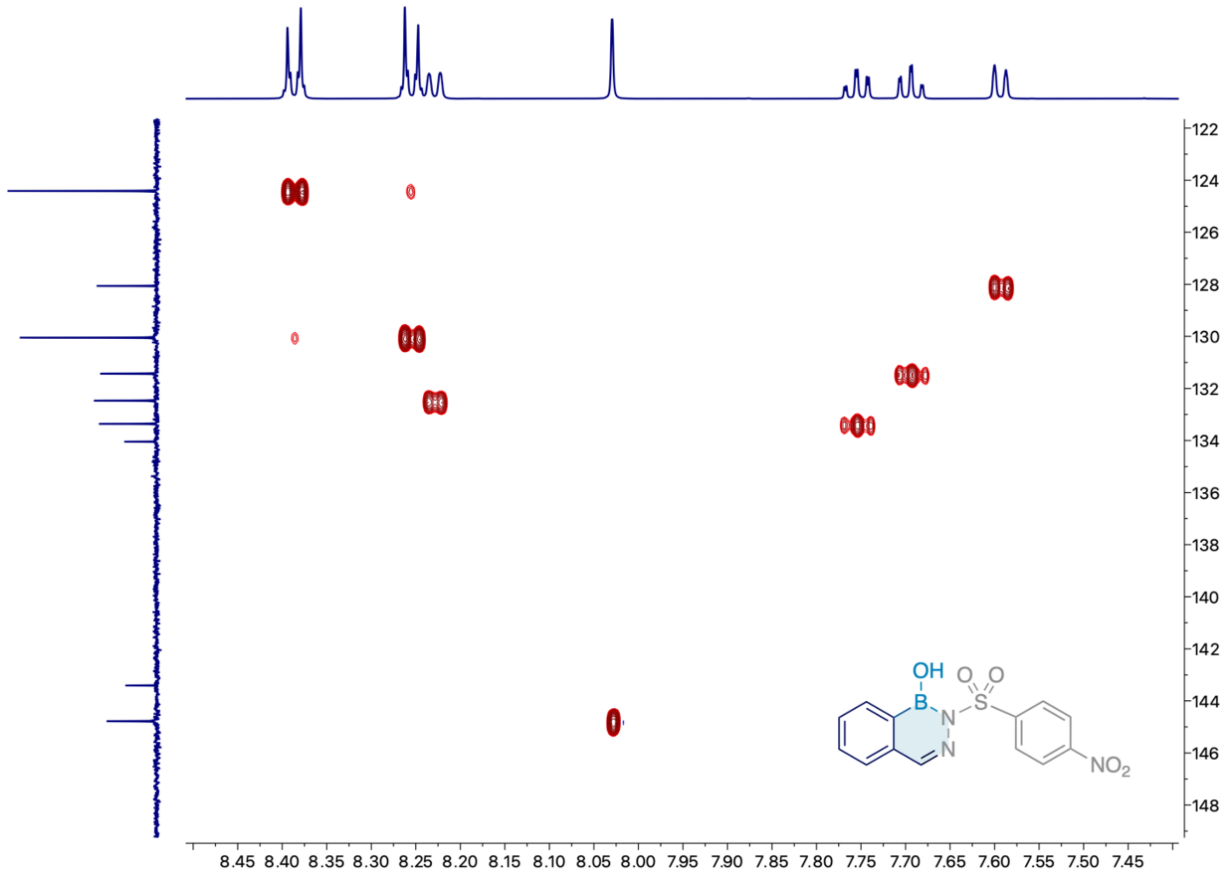

Figure S11. Diazaborine 1:^{1}H-^{13}C gHSQC NMR ($CDCl_3$, 298 K)

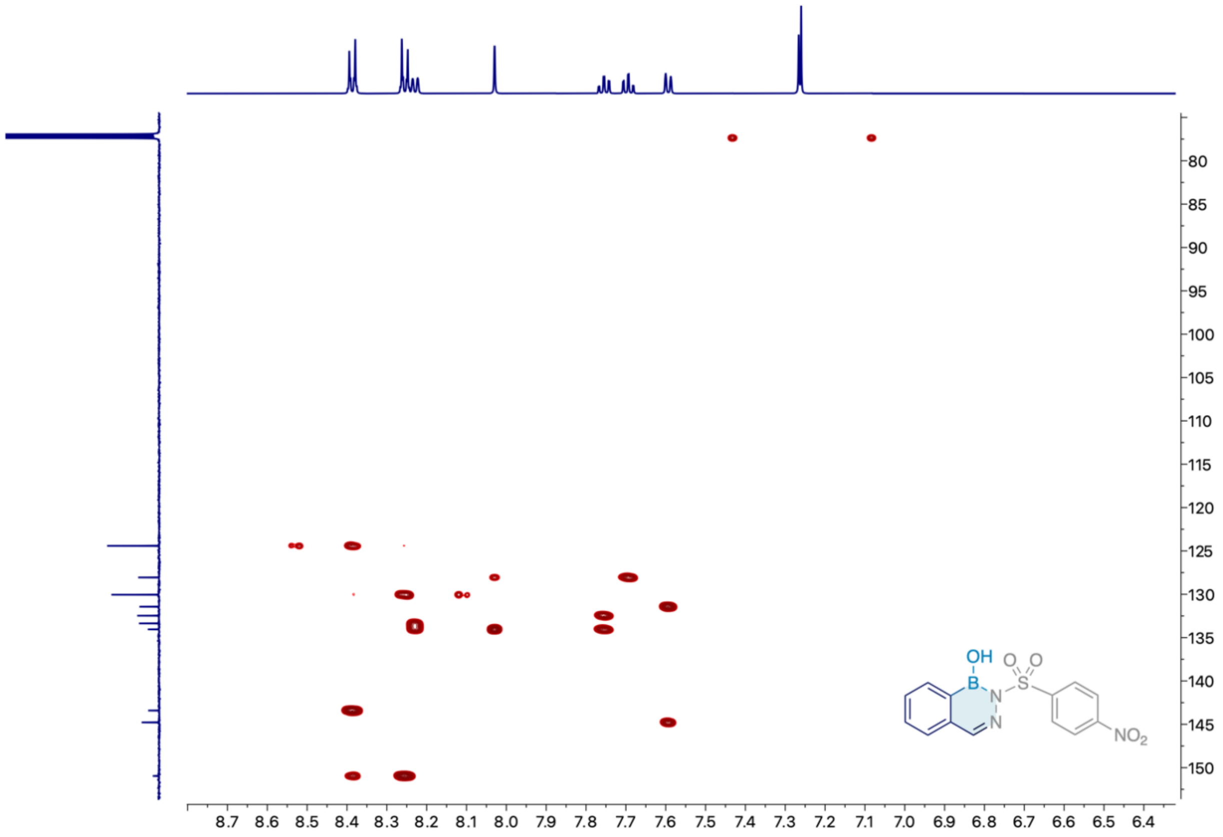

Figure S12. Diazaborine 1: ^{1}H-^{13}C gHMBC NMR ($CDCl_3$, 298 K)

Diazaborine 2

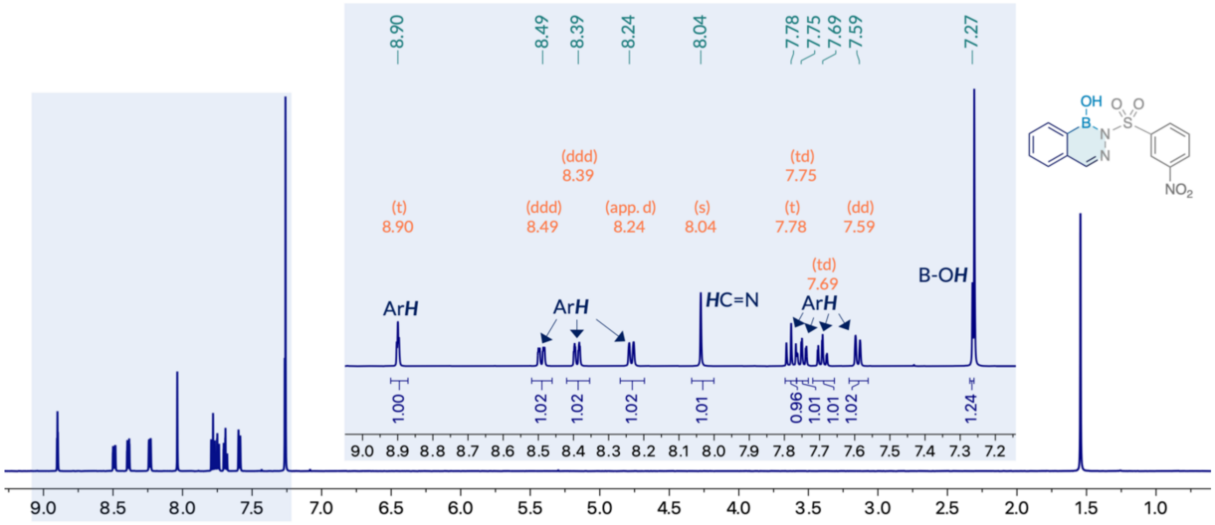

Figure S13. Diazaborine 2: ^{1}H NMR (600 MHz, $CDCl_3$, 298 K)

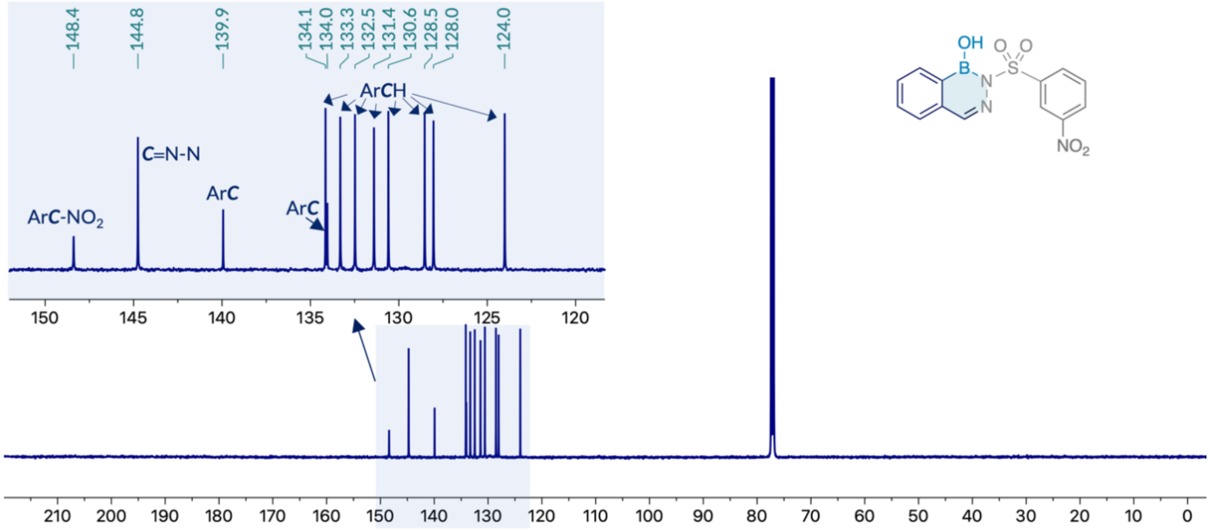

Figure S14. Diazaborine 2: ^{13}C NMR (126 MHz, $CDCl_3$, 298 K)

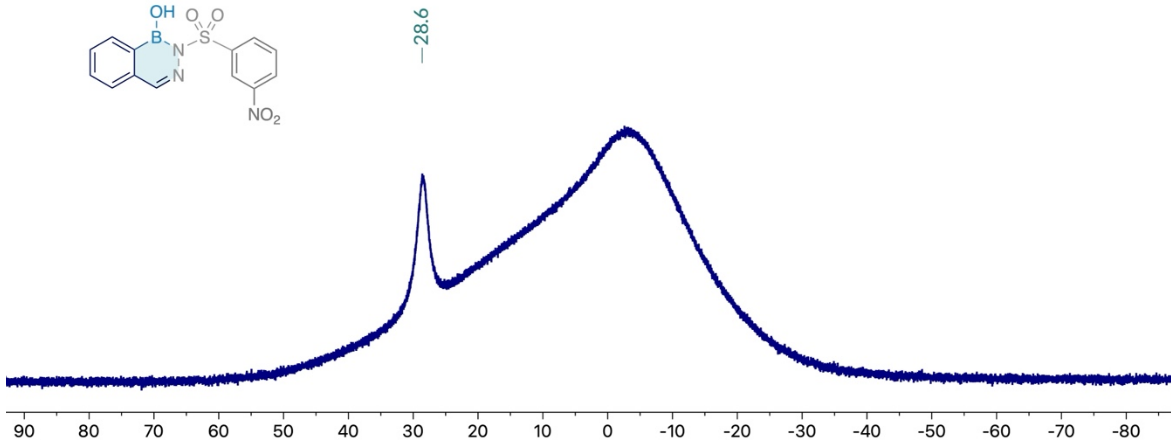

Figure S15. Diazaborine 2: ^{11}B NMR (160 MHz, $CDCl_3$, 298 K)

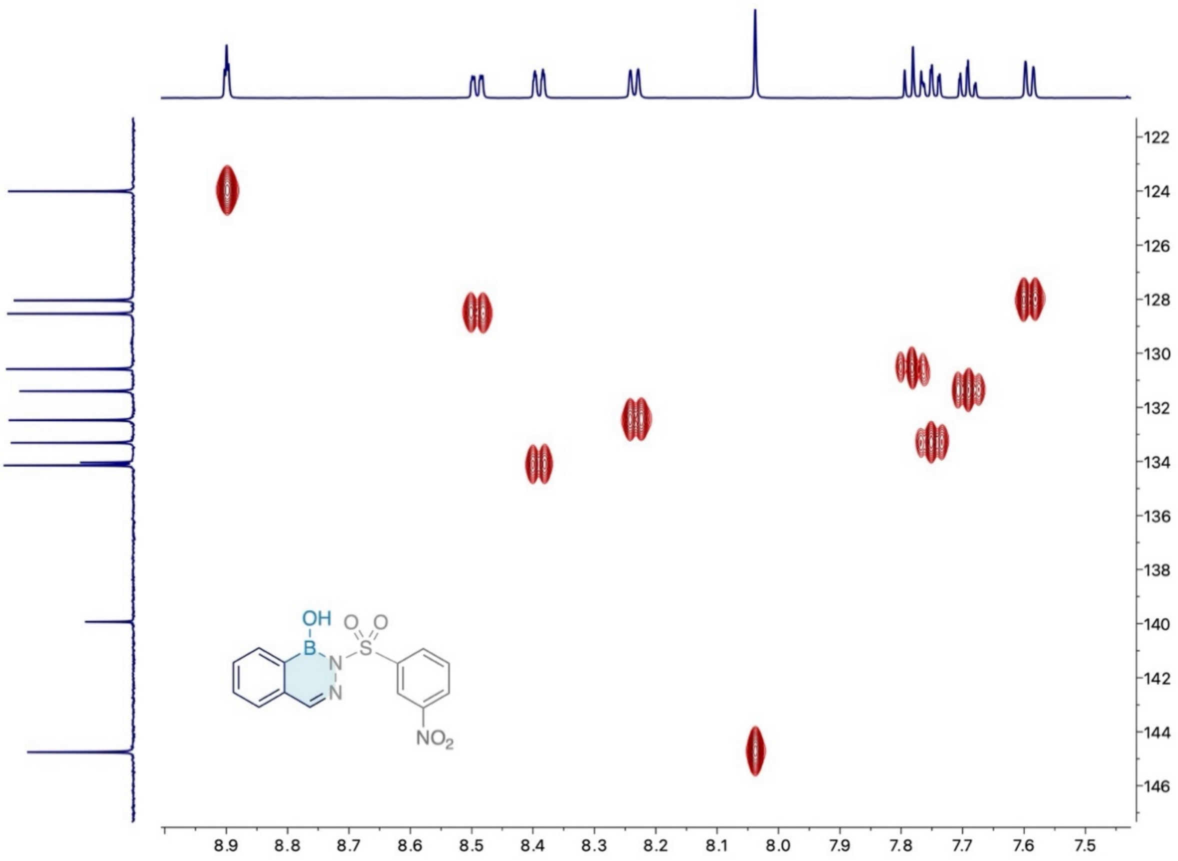

Figure S16. Diazaborine 2:^{1}H-^{13}C gHSQC NMR ($CDCl_3$, 298 K)

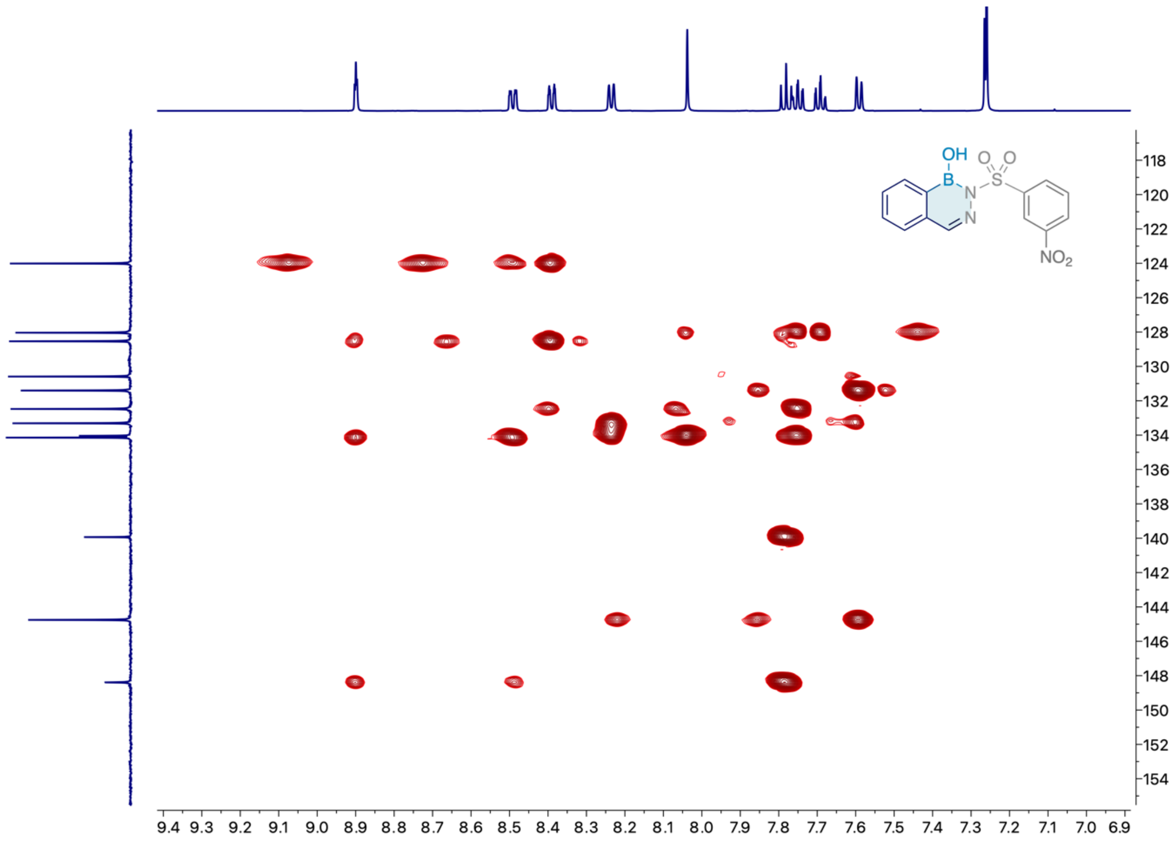

Figure S17. Diazaborine 2:^{1}H-^{13}C gHMBC NMR ($CDCl_3$, 298 K)

Diazaborine 3

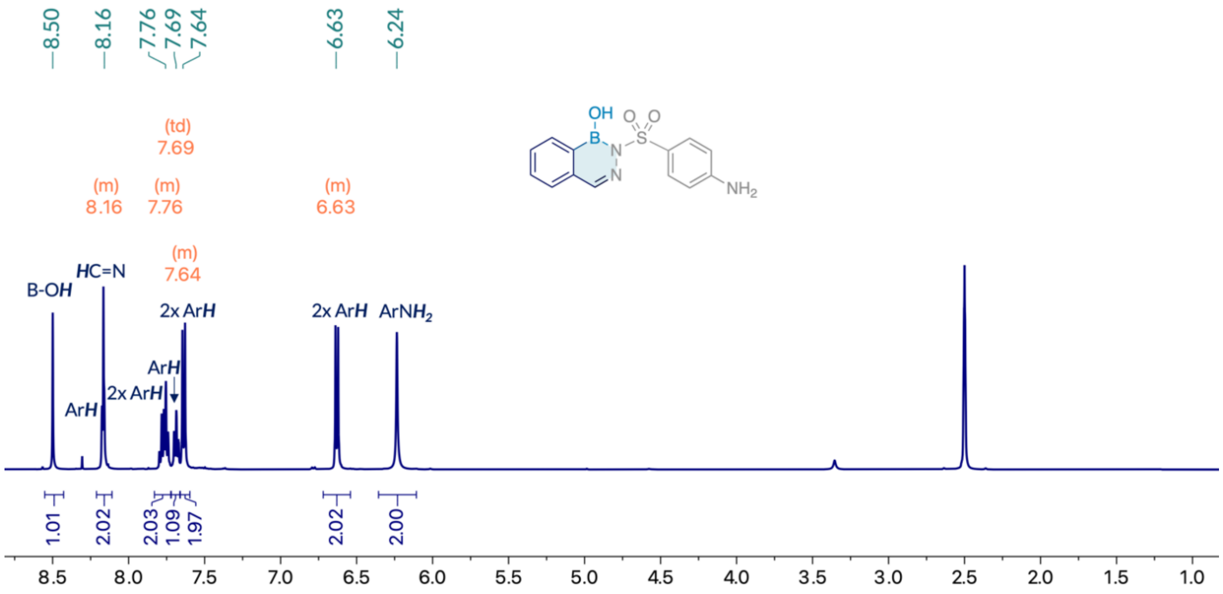

Figure S18. Diazaborine 3: ^{1}H NMR (500 MHz, DMSO-d_6, 298 K)

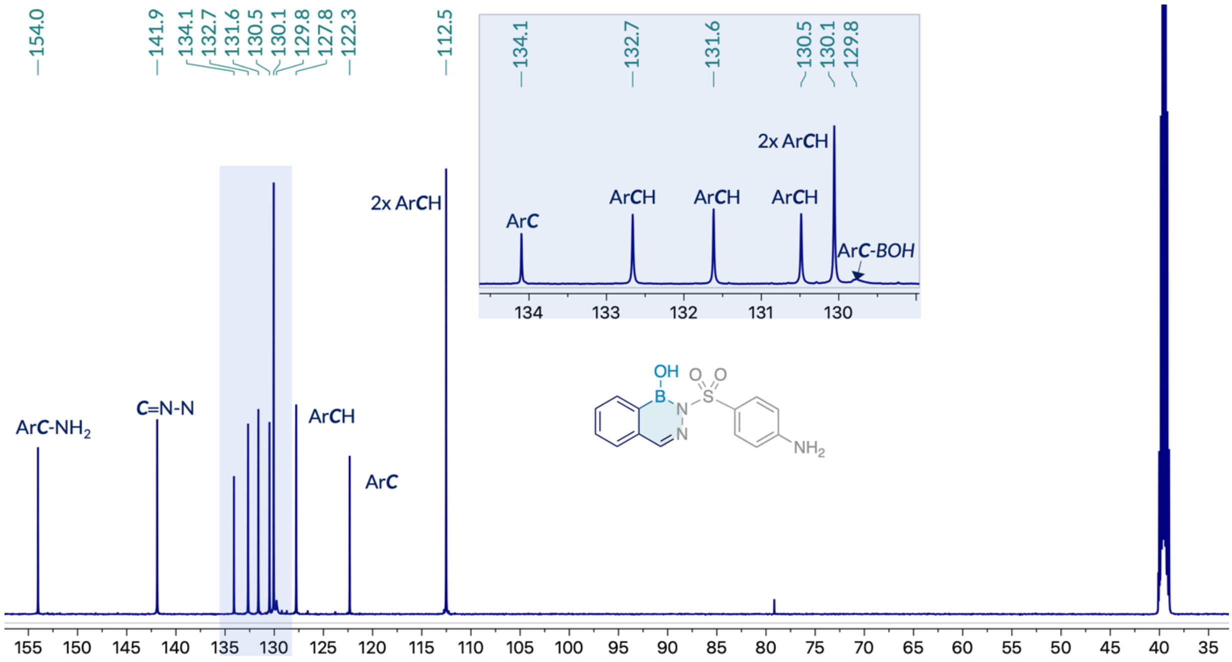

Figure S19. Diazaborine 3: ^{13}C NMR (126 MHz, DMSO-d_6, 298 K)

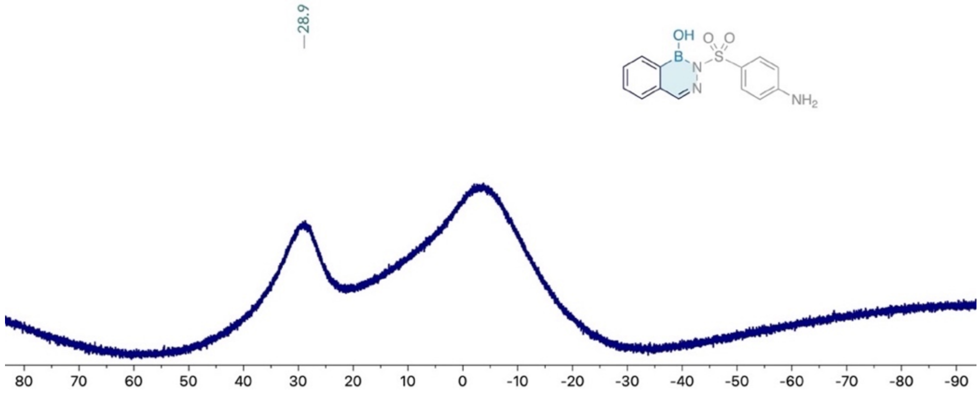

Figure S20. Diazaborine 3: ^{11}B NMR (160 MHz, DMSO-d_6, 298 K)

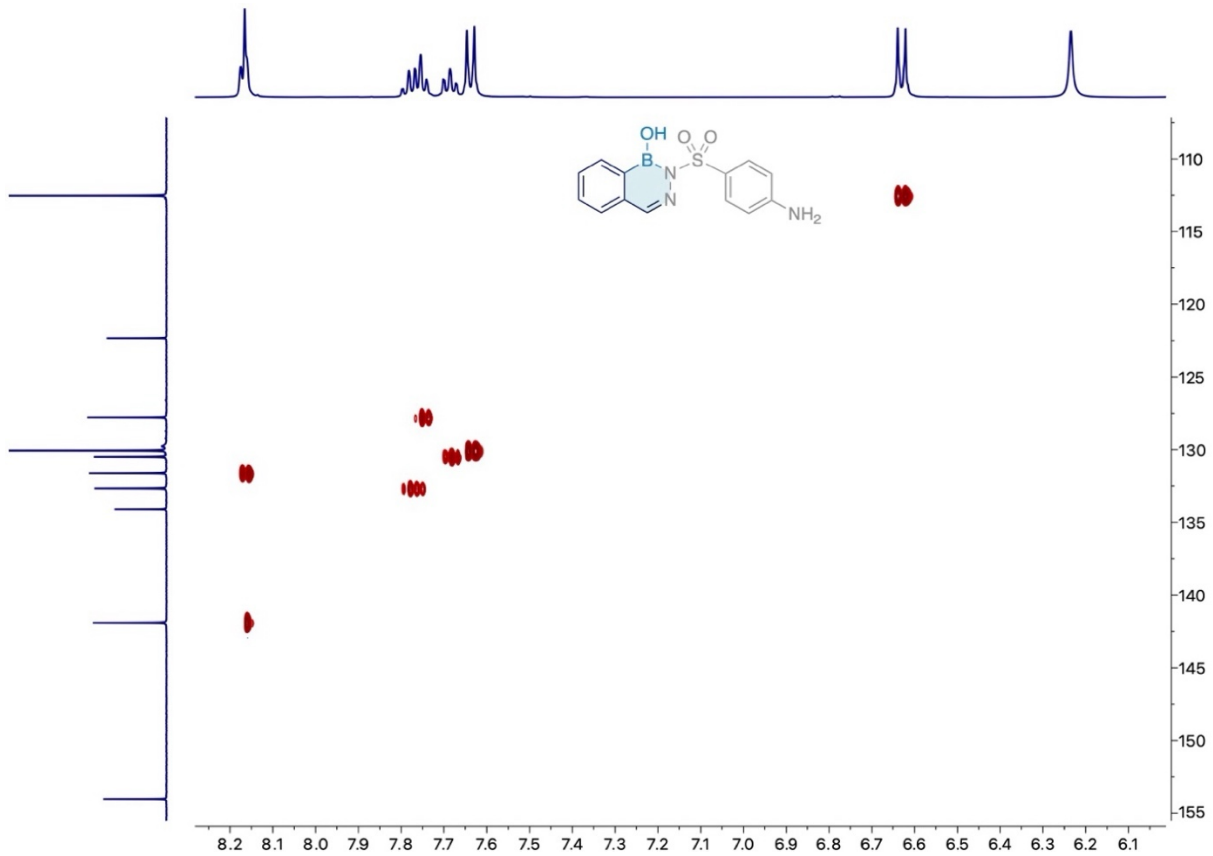

Figure S21. Diazaborine 3:^{1}H-^{13}C gHSQC NMR (DMSO-d_6, 298 K)

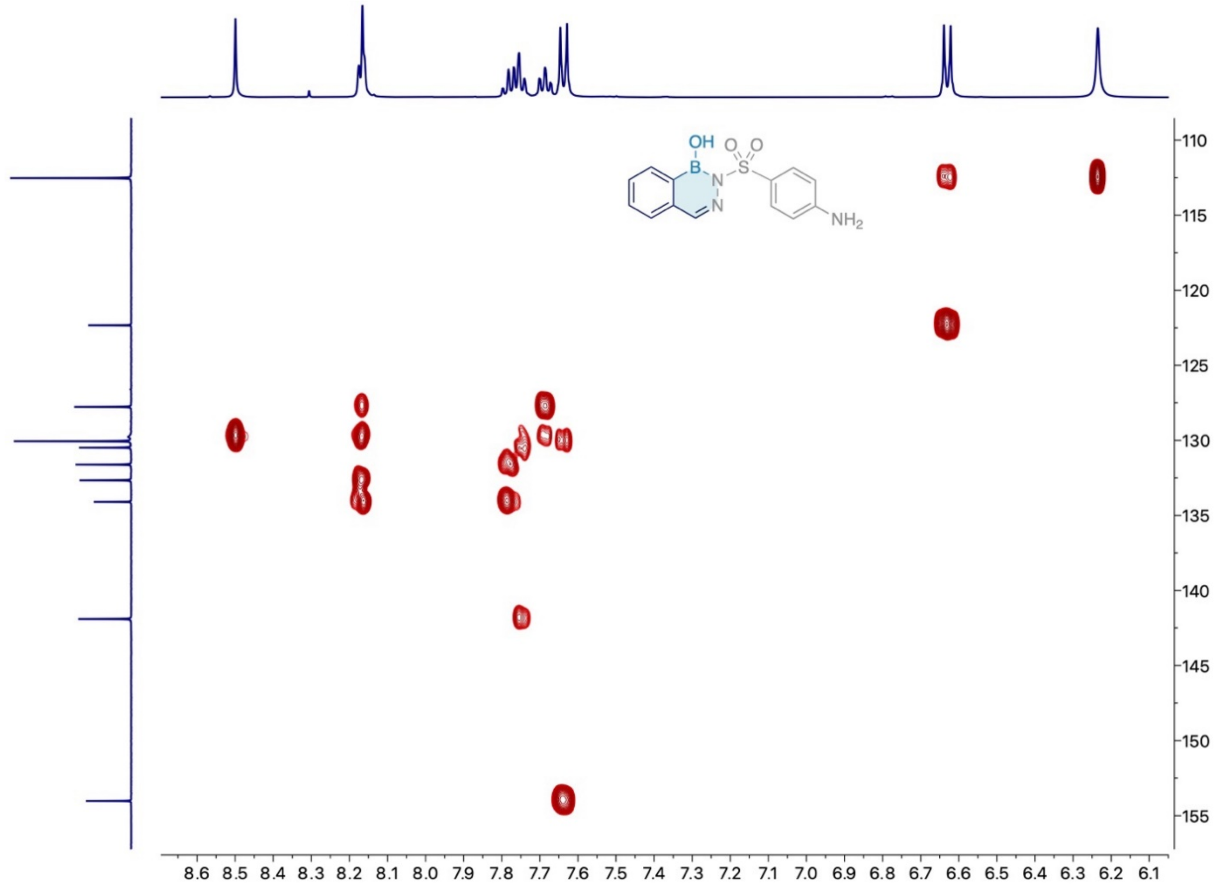

Figure S22. Diazaborine 3:^{1}H-^{13}C gHMBC NMR (DMSO-d_6, 298 K)

Diazaborine 4

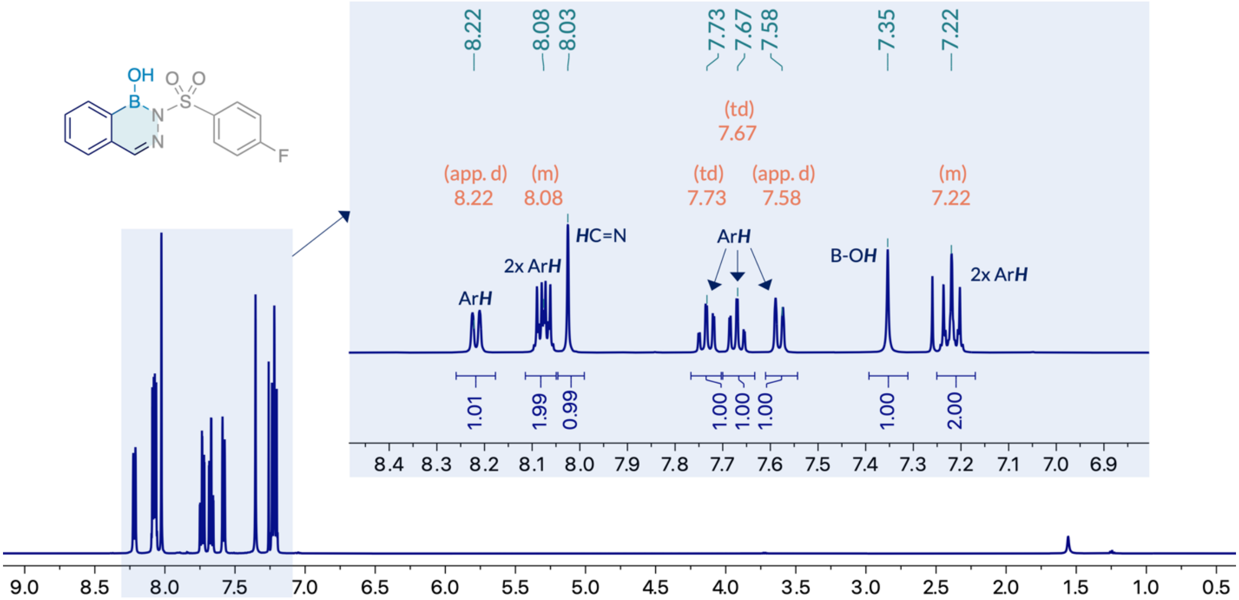

Figure S23. Diazaborine 4:^{1}H NMR (500 MHz, $CDCl_3$, 298 K)

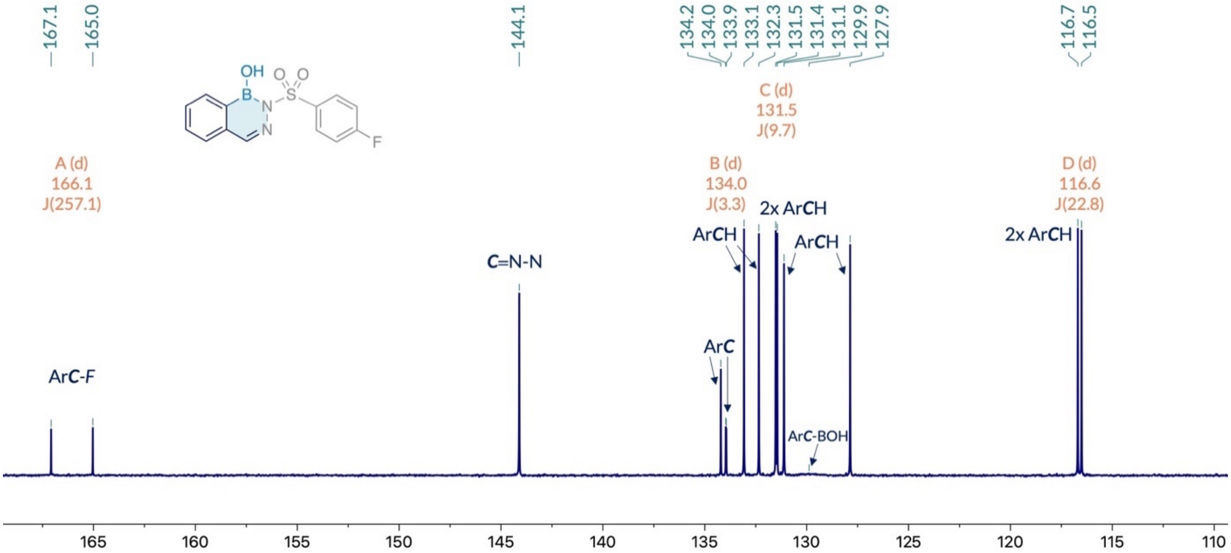

Figure S24. Diazaborine 4:^{13}C NMR (126 MHz, $CDCl_3$, 298 K)

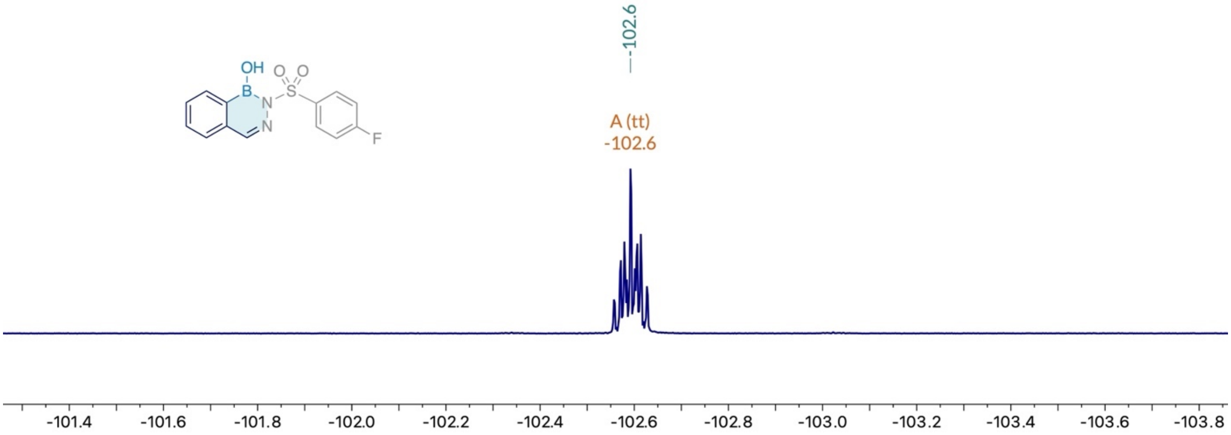

Figure S25. Diazaborine 4:^{19}F NMR (377 MHz, $CDCl_3$, 298 K)

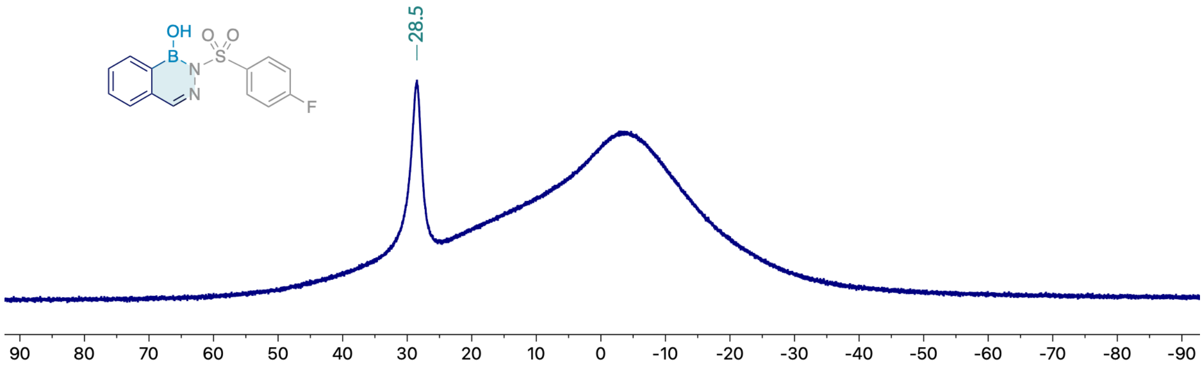

Figure S26. Diazaborine 4: ^{11}B NMR (160 MHz, $CDCl_3$, 298 K)

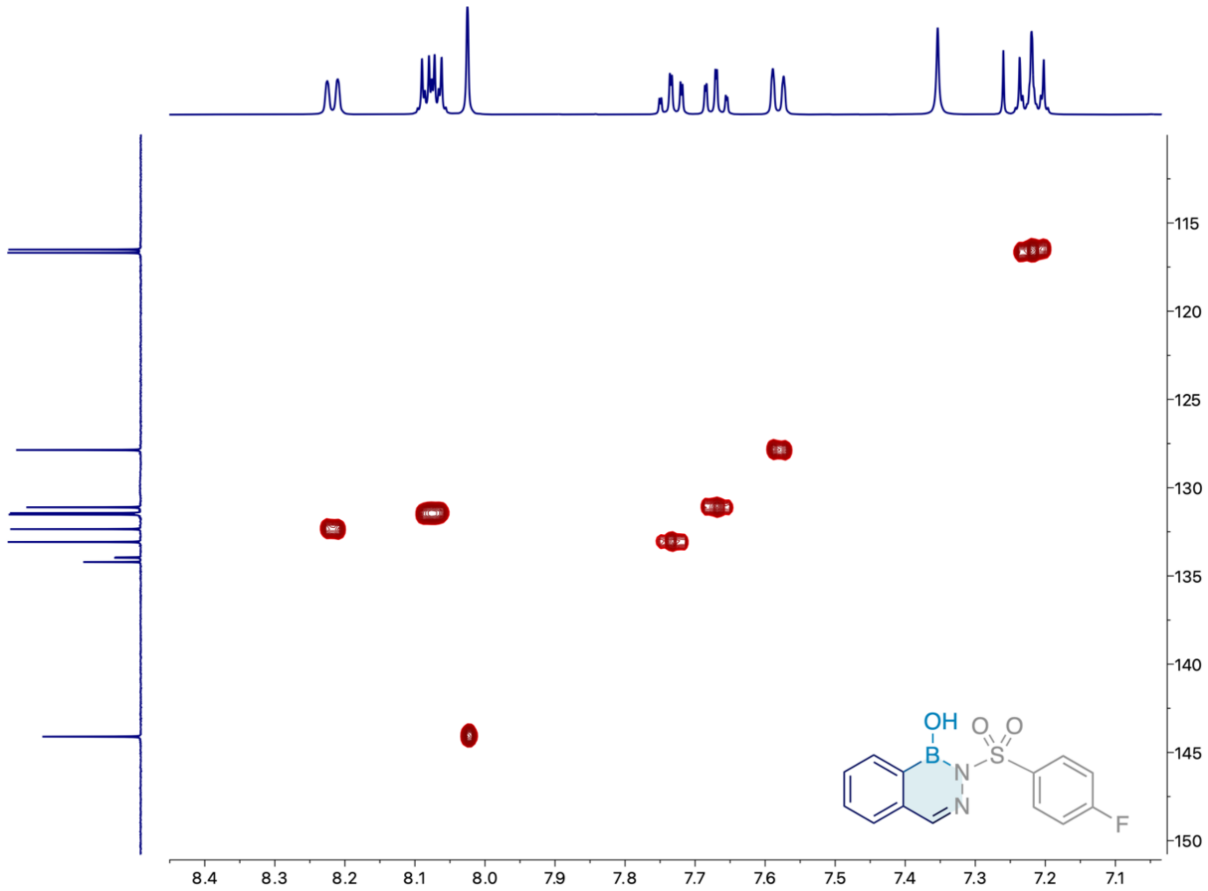

Figure S27. Diazaborine 4: ^{1}H-^{13}C gHSQC NMR ($CDCl_3$, 298 K)

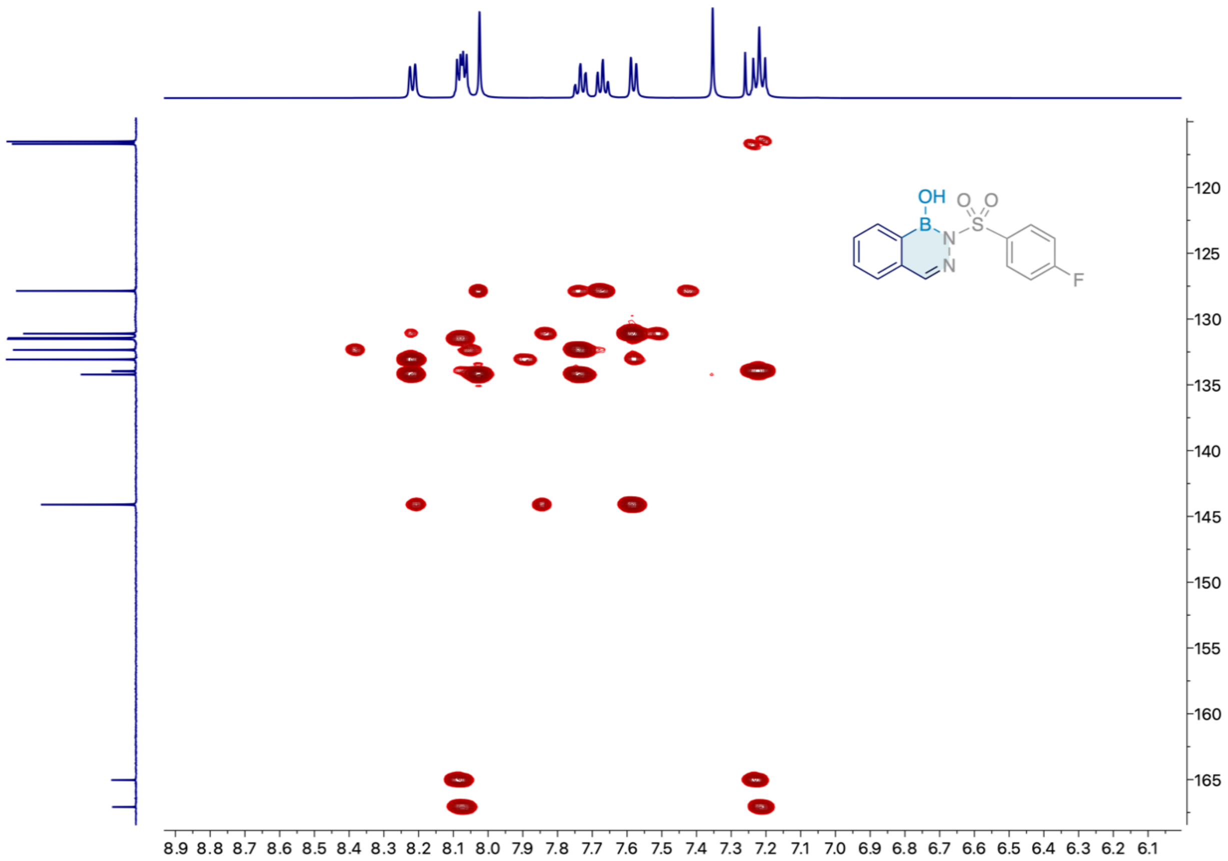

Figure S28. Diazaborine 4:^{1}H-^{13}C gHMBC NMR ($CDCl_3$, 298 K)

Diazaborine 5

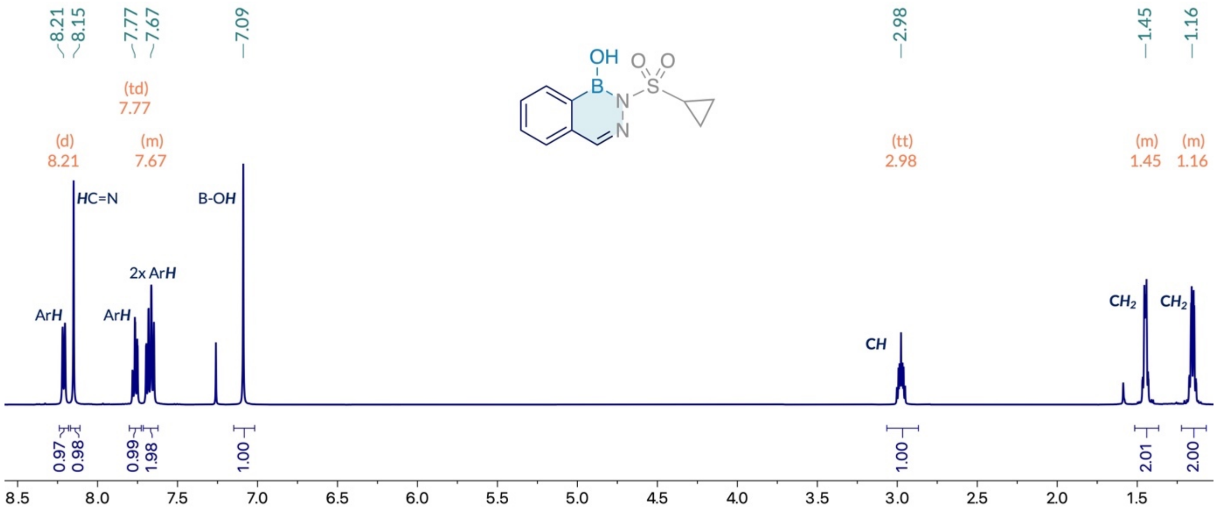

Figure S29. Diazaborine 5:^{1}H NMR (500 MHz, $CDCl_3$, 298 K)

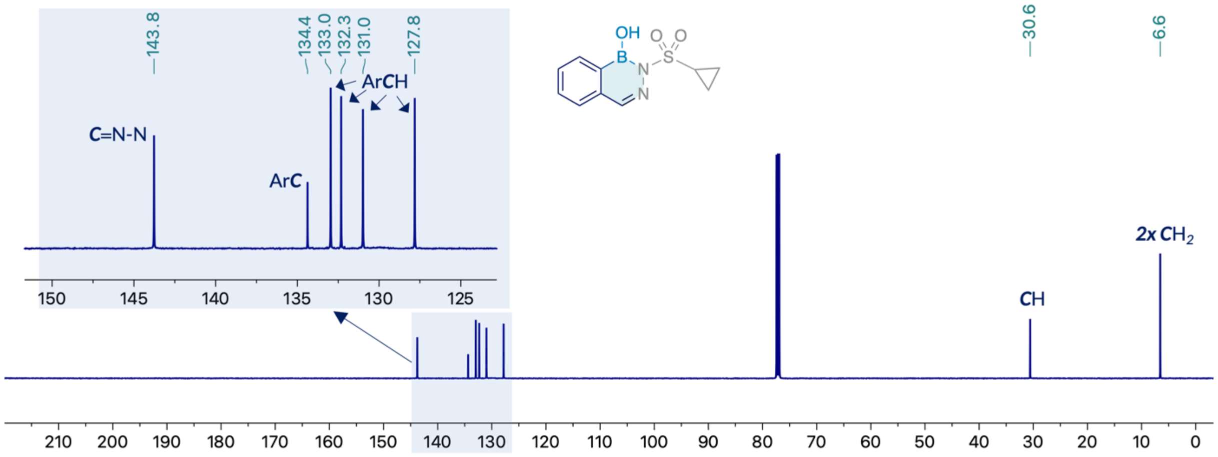

Figure S30. Diazaborine 5:^{1}H NMR (126 MHz, $CDCl_3$, 298 K)

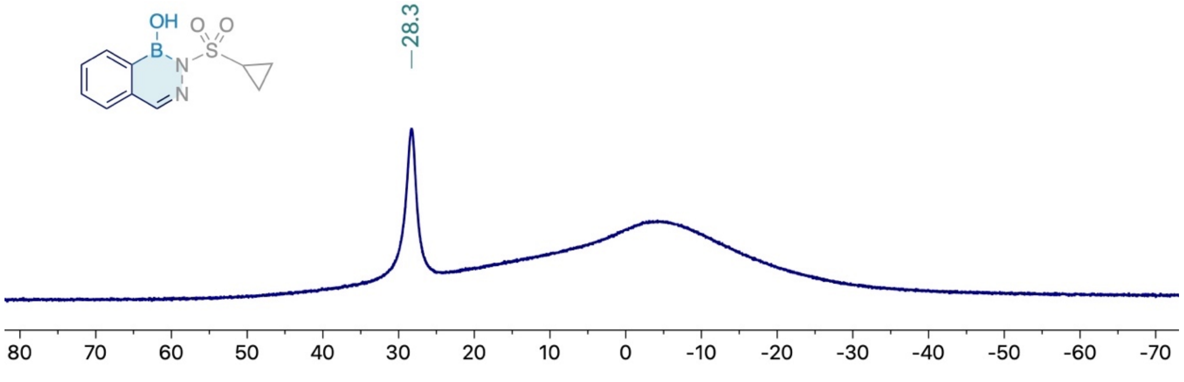

Figure S31. Diazaborine 5:^{11}B NMR (160 MHz, $CDCl_3$, 298 K)

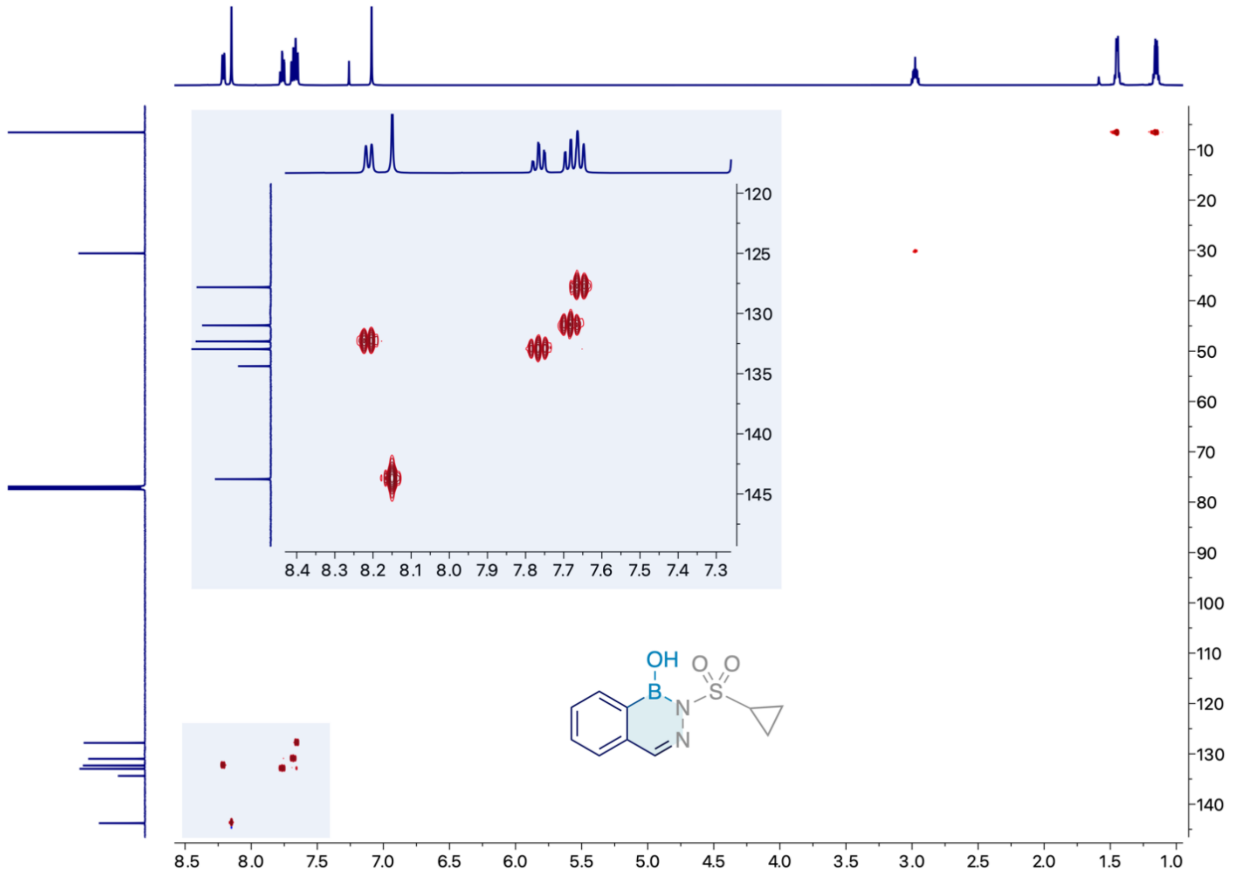

Figure S32. Diazaborine 5:^{1}H-^{13}C gHSQC NMR ($CDCl_3$, 298 K)

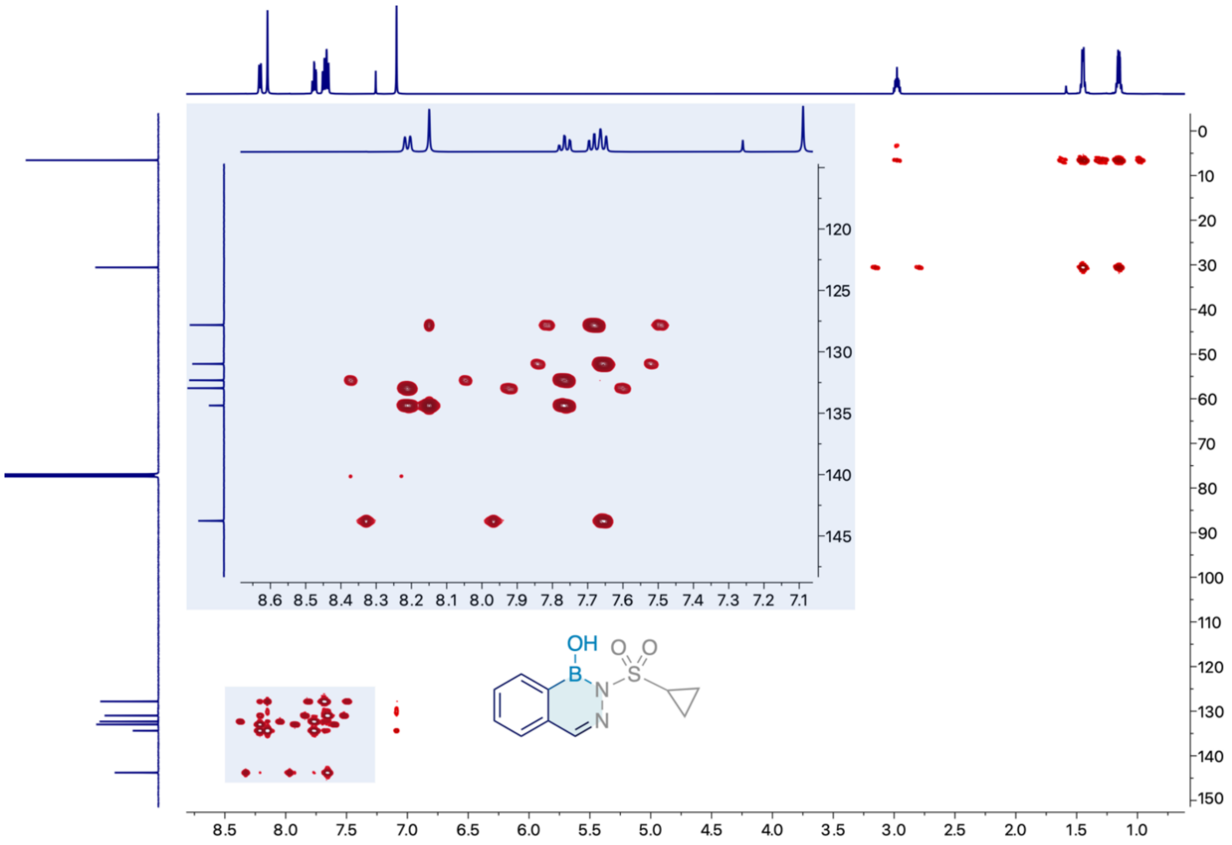

Figure S33. Diazaborine 5:^{1}H-^{13}C gHMBC NMR ($CDCl_3$, 298 K)

Diazaborine 6

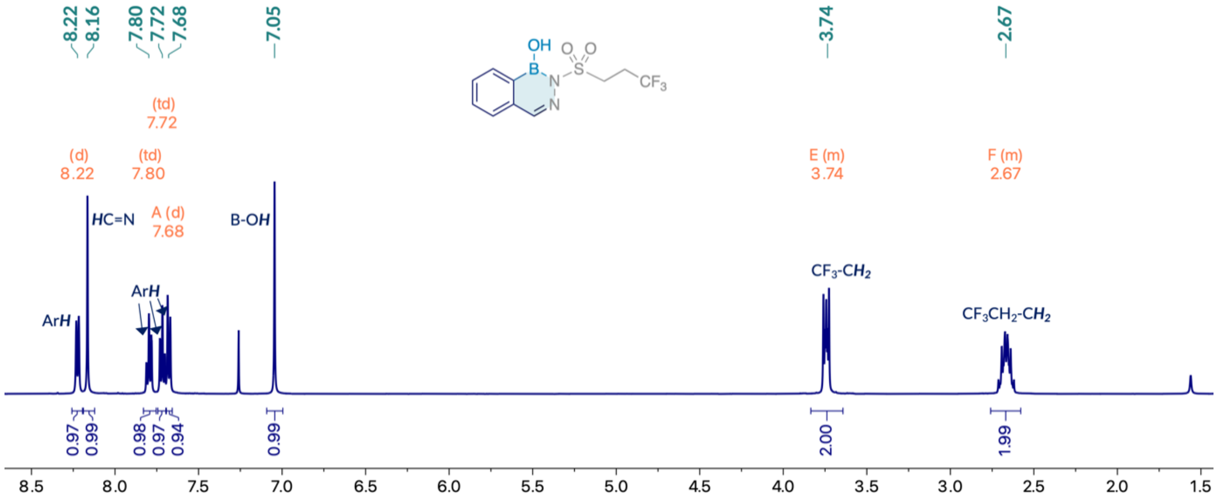

Figure S34. Diazaborine 6:^{1}H NMR (500 MHz, $CDCl_3$, 298 K)

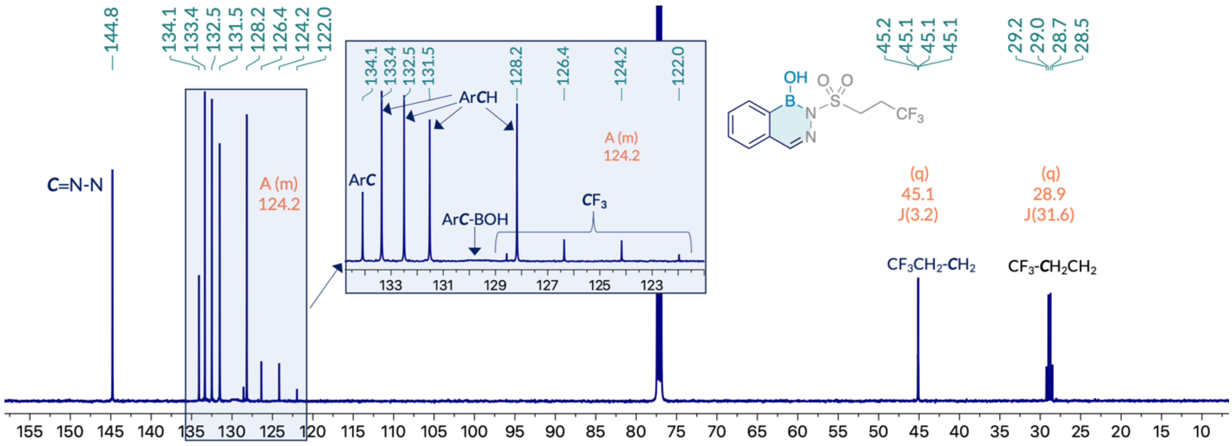

Figure S35. Diazaborine 6:^{13}C NMR (126 MHz, $CDCl_3$, 298 K)

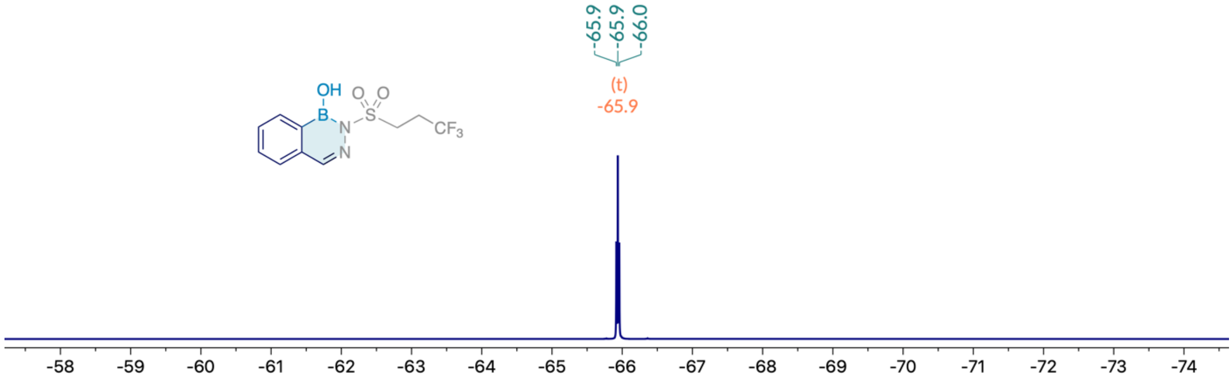

Figure S36. Diazaborine 6:^{19}F NMR (470 MHz, $CDCl_3$, 298 K)

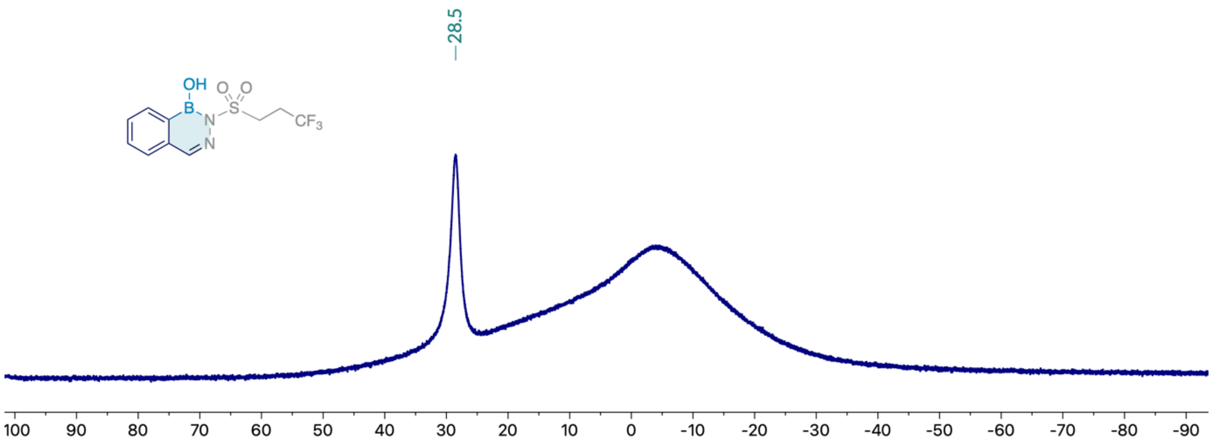

Figure S37. Diazaborine 6:^{11}B NMR (160 MHz, $CDCl_3$, 298 K)

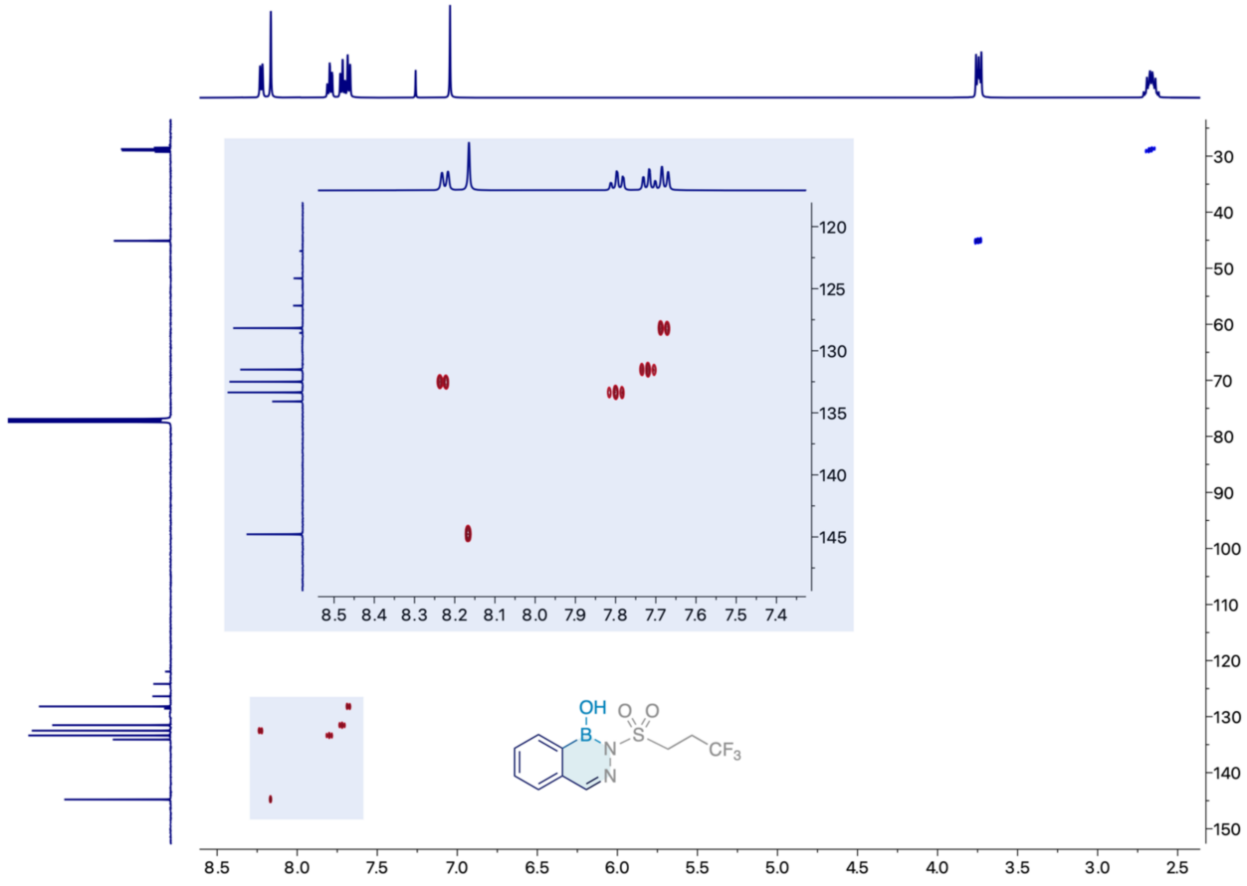

Figure S38. Diazaborine 6:^{1}H-^{13}C gHSQC NMR ($CDCl_3$, 298 K)

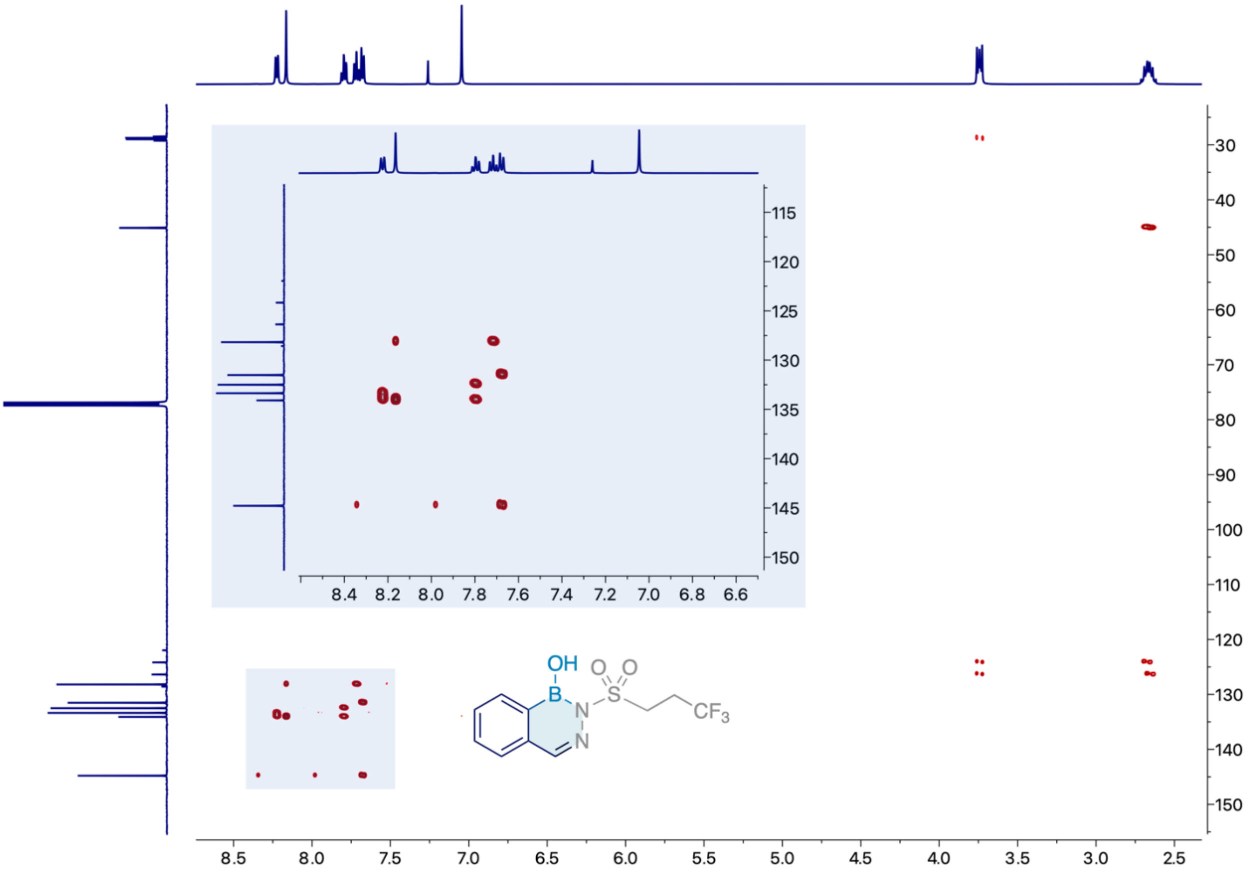

Figure S39. Diazaborine 6:^{1}H-^{13}C gHMBC NMR ($CDCl_3$, 298 K)

Diazaborine 7

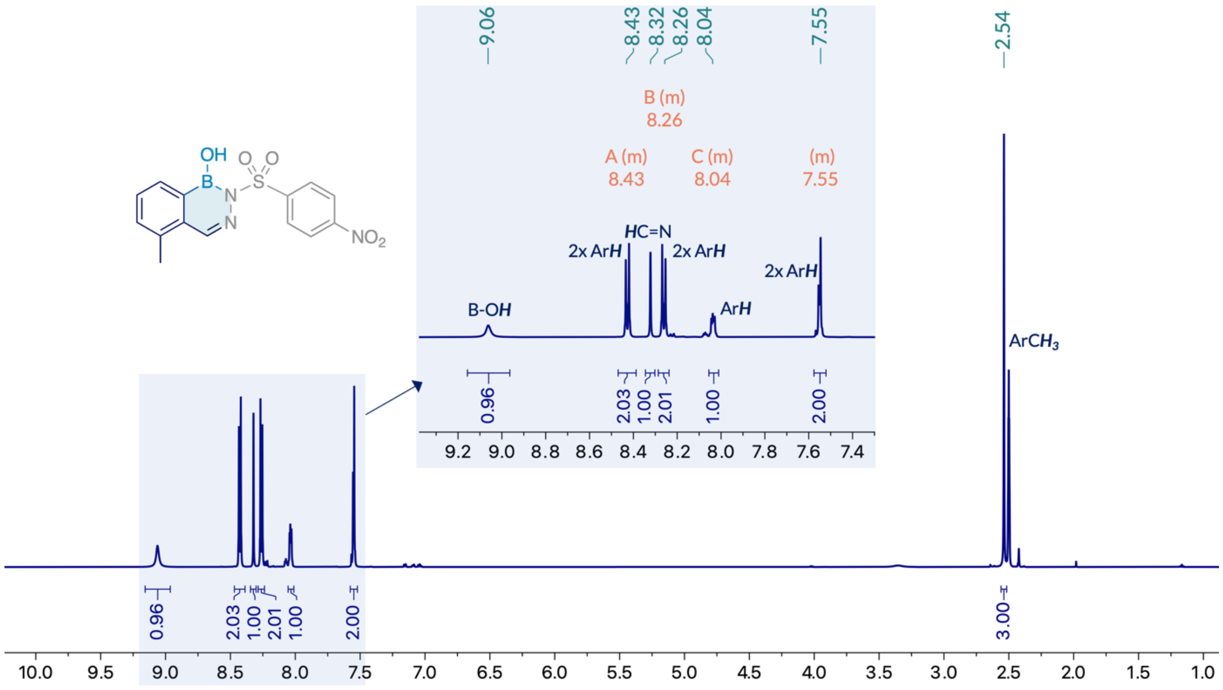

Figure S40. Diazaborine 7:^{1}H NMR (600 MHz, DMSO-d_6, 298 K)

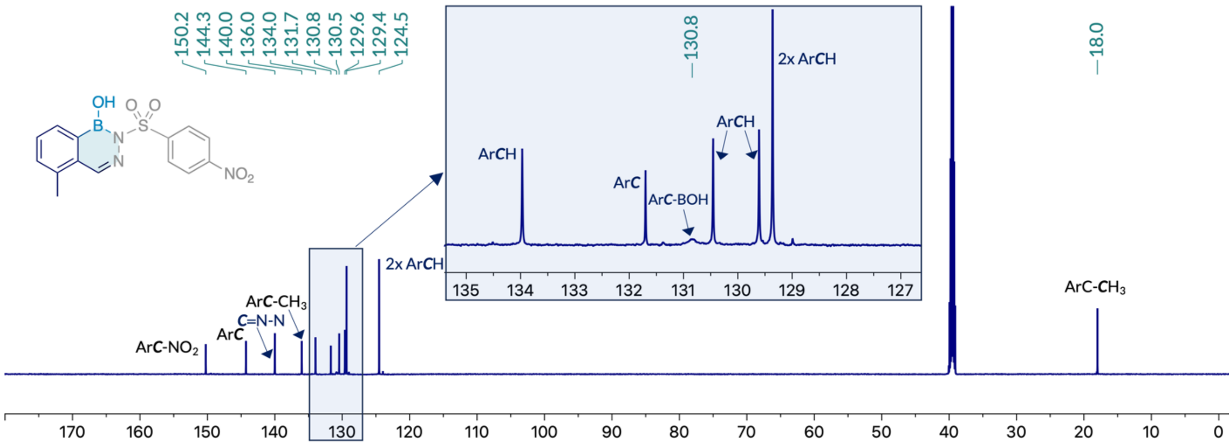

Figure S41. Diazaborine 7:^{13}C NMR (151 MHz, DMSO-d_6, 298 K)

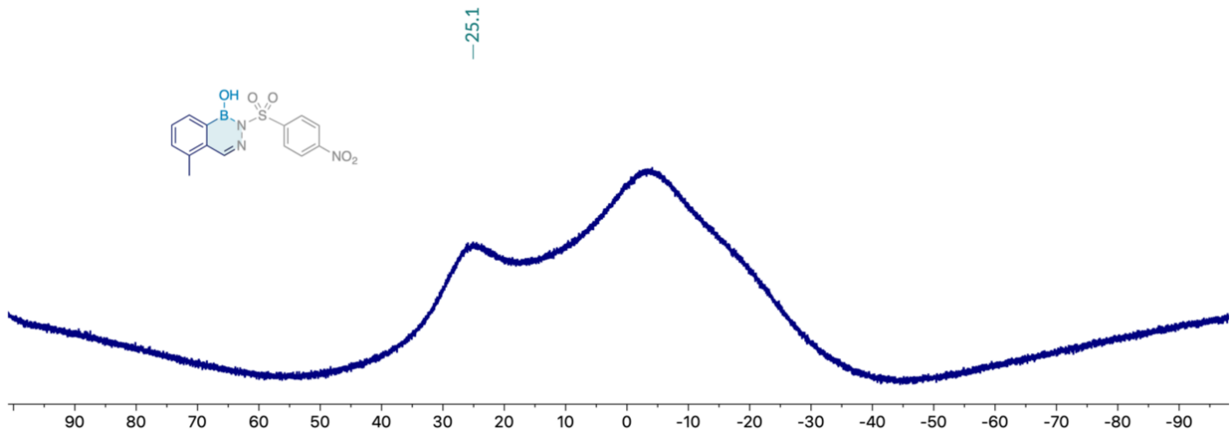

Figure S42. Diazaborine 7:^{11}B NMR (128 MHz, DMSO-d_6, 298 K)

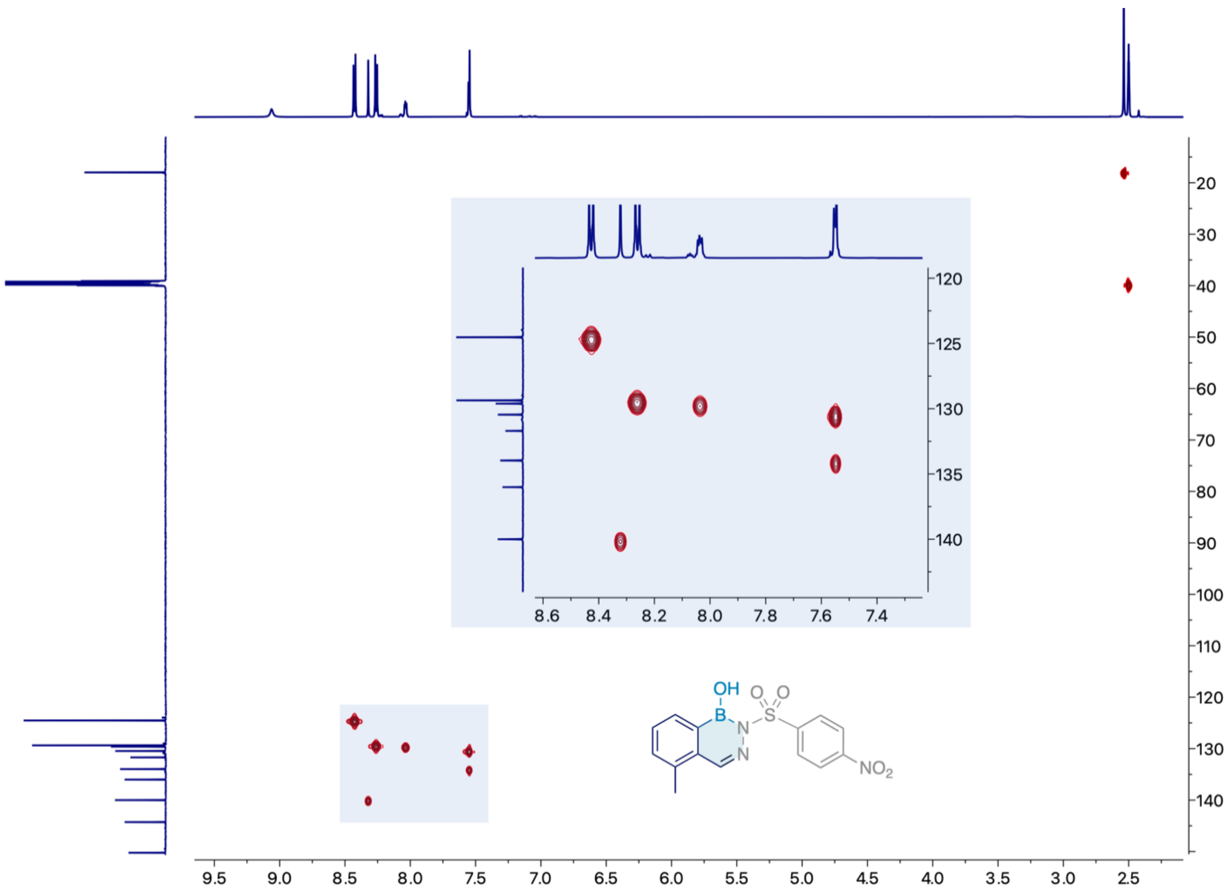

Figure S43. Diazaborine 7:^{1}H-^{13}C gHSQC NMR (DMSO-d_6, 298 K)

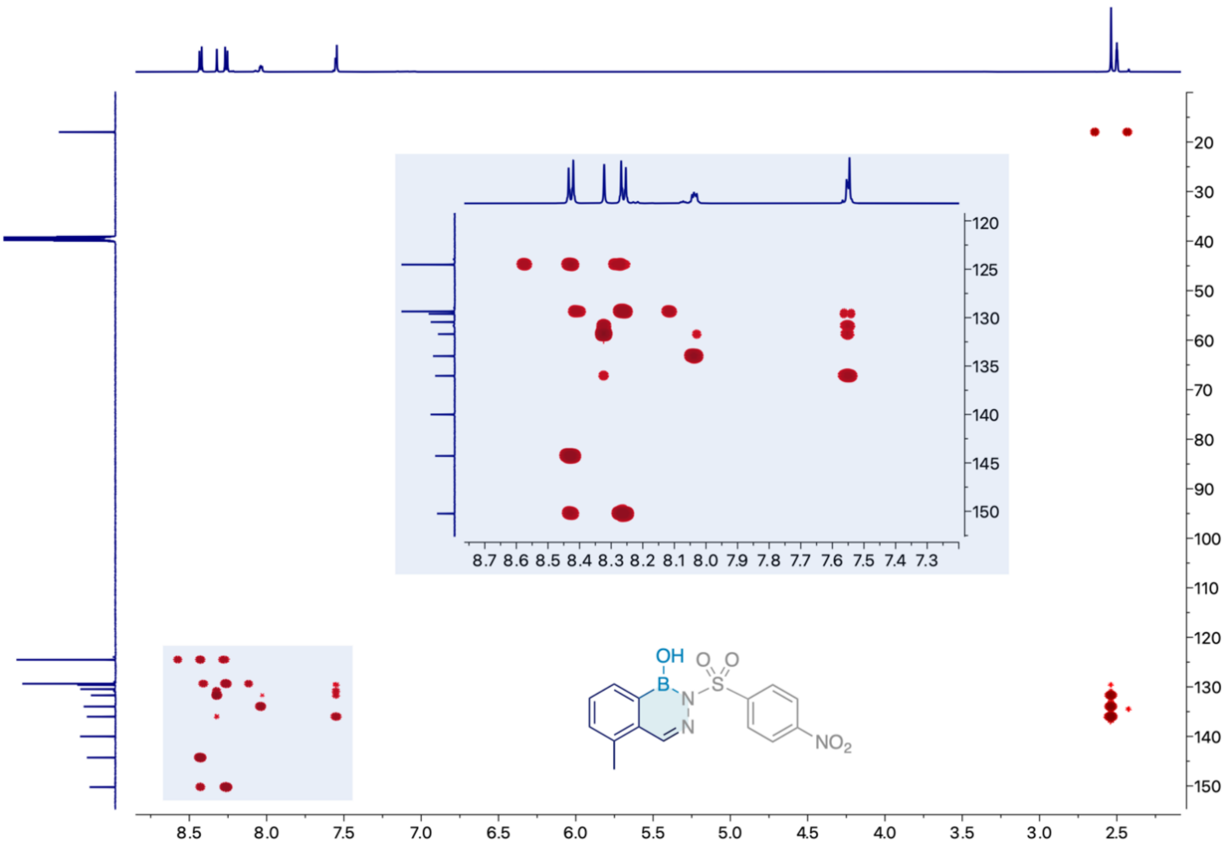

Figure S44. Diazaborine 7:^{1}H-^{13}C gHMBC NMR (DMSO-d_6, 298 K)

Diazaborine 8

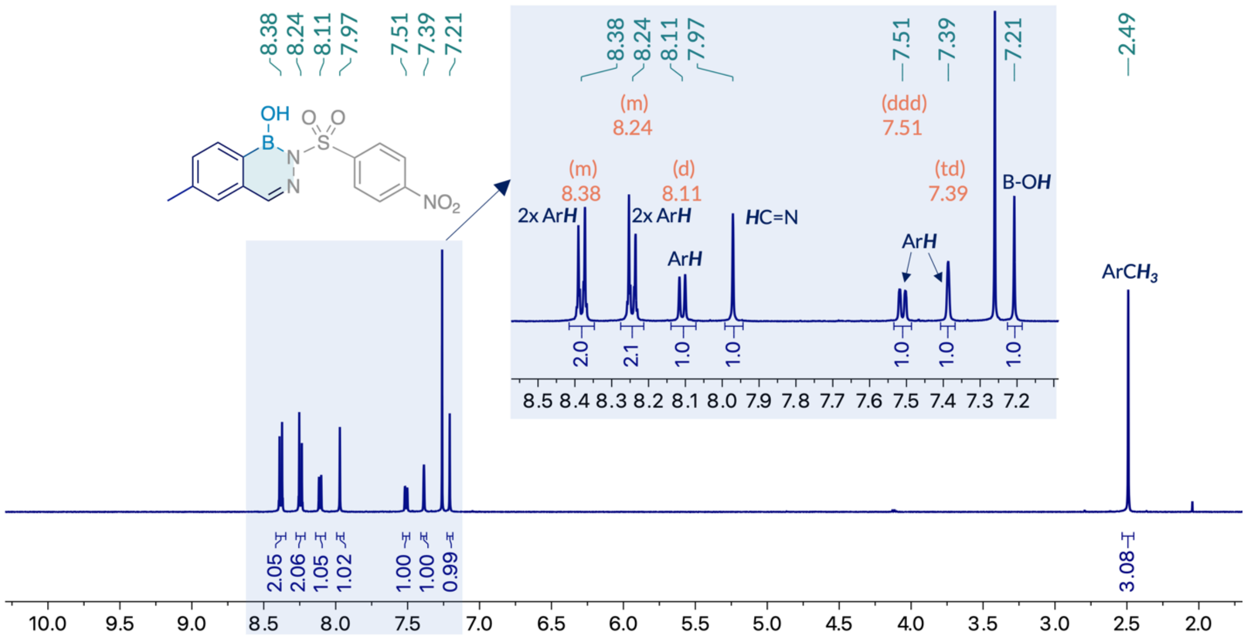

Figure S45. Diazaborine 8: ^{1}H NMR (500 MHz, $CDCl_3$, 298 K)

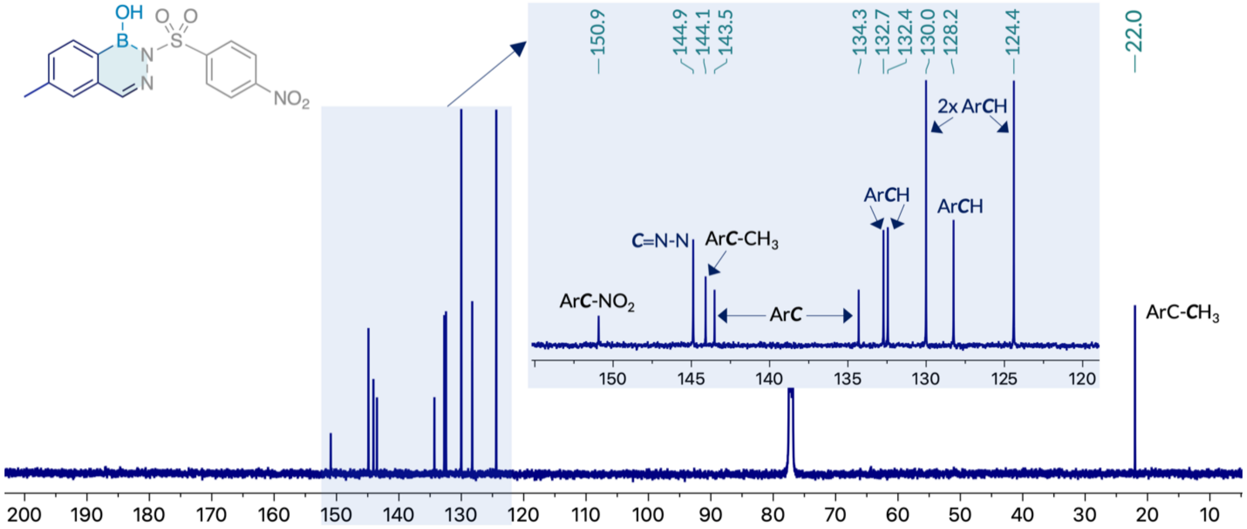

Figure S46. Diazaborine 8: ^{13}C NMR (126 MHz, $CDCl_3$, 298 K)

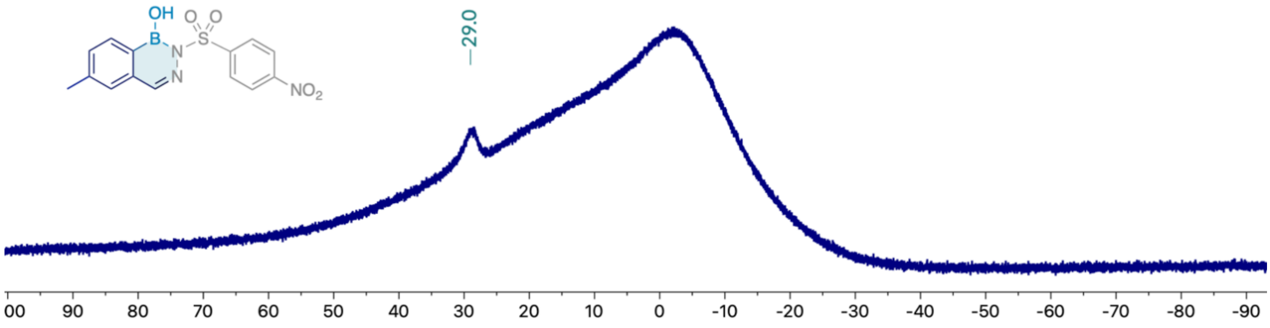

Figure S47. Diazaborine 8: ^{11}B NMR (160 MHz, $CDCl_3$, 298 K)

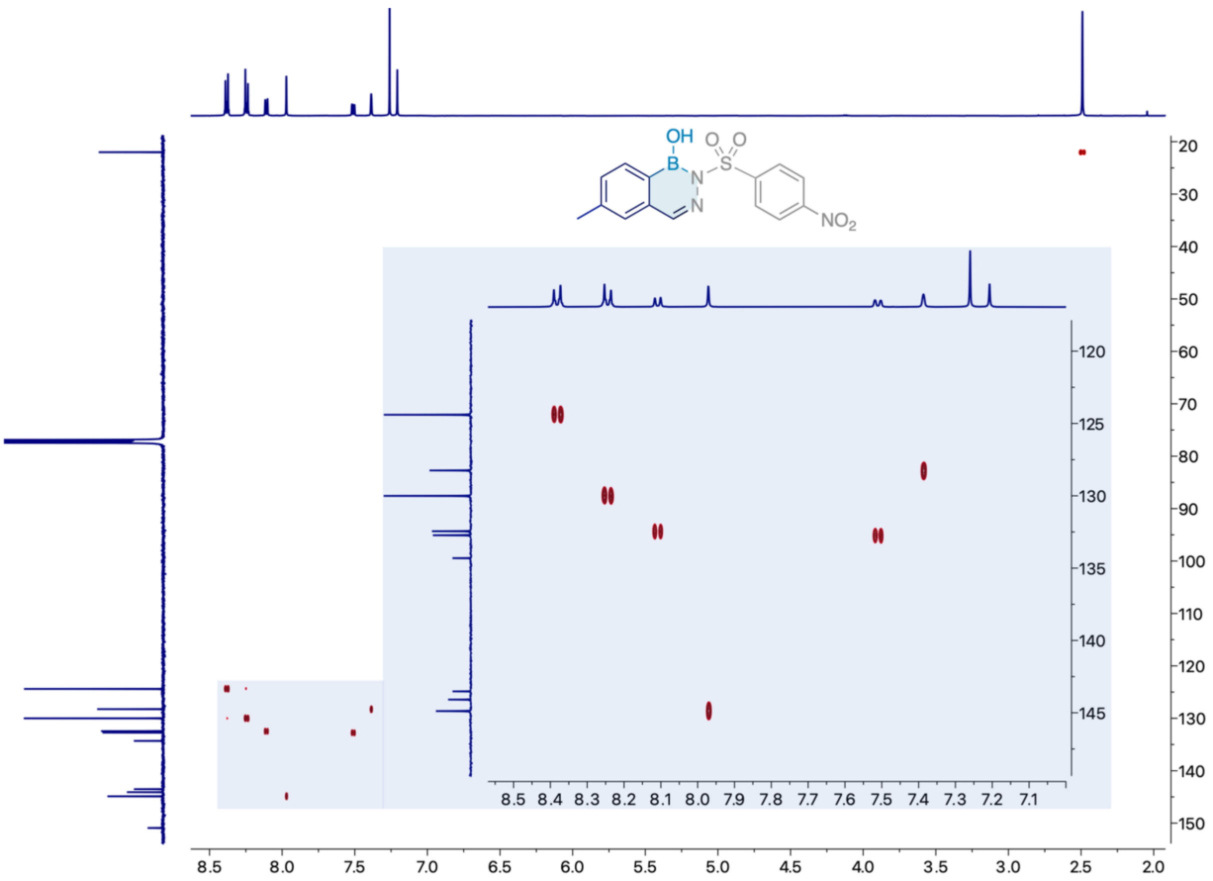

Figure S48. Diazaborine 8:^{1}H-^{13}C gHSQC NMR ($CDCl_3$, 298 K)

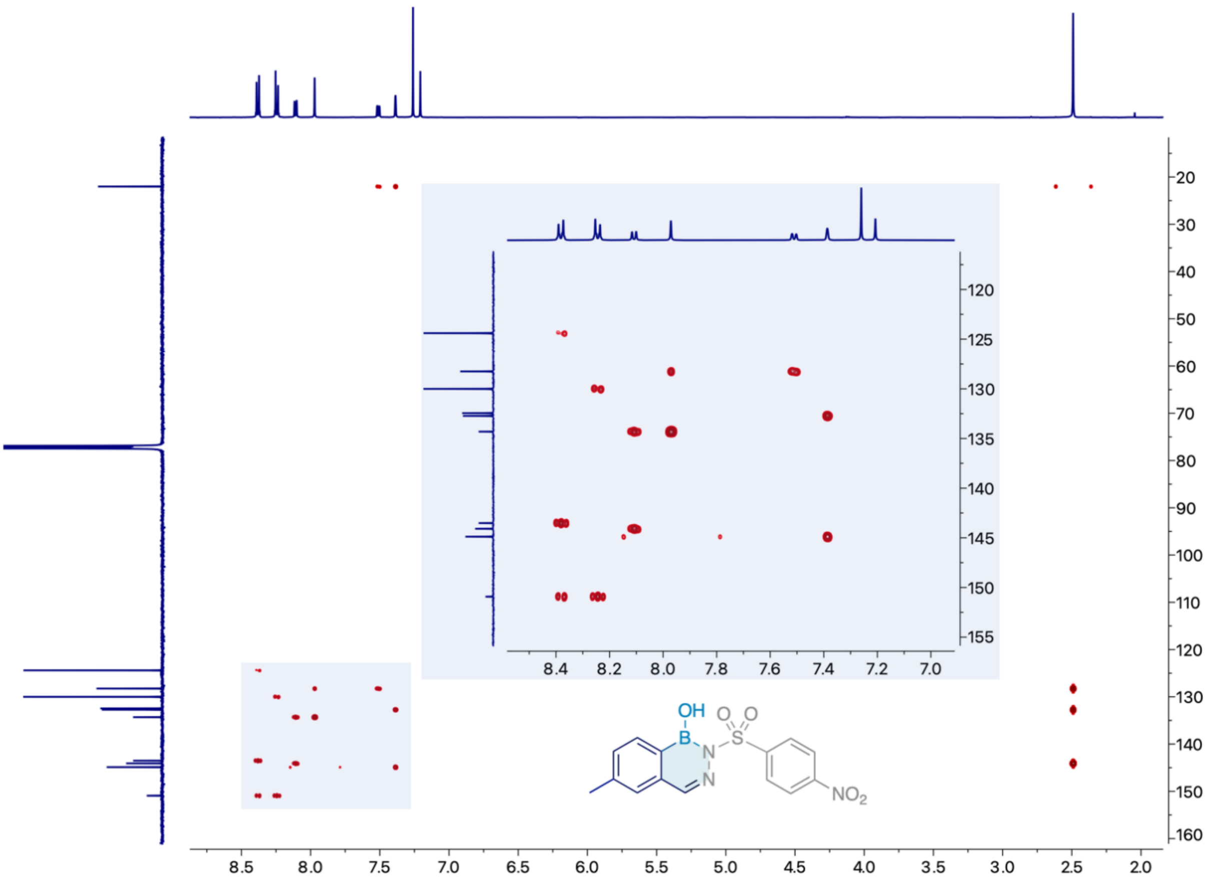

Figure S49. Diazaborine 8:^{1}H-^{13}C gHMBC NMR ($CDCl_3$, 298 K)

Diazaborine 9

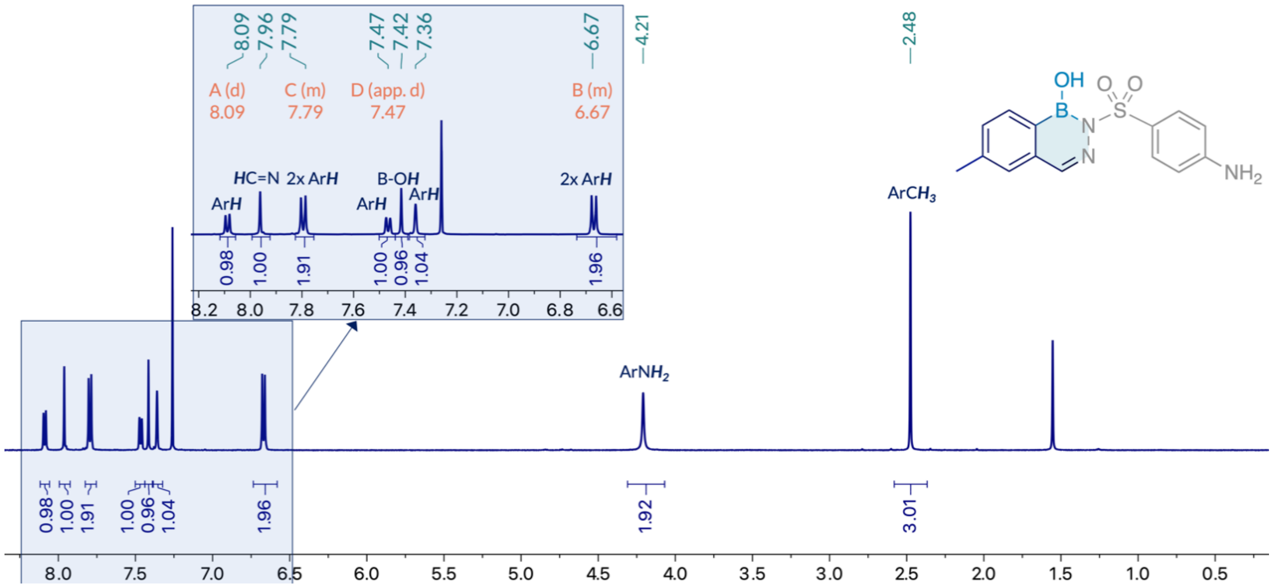

Figure S50. Diazaborine 9:^{1}H NMR (500 MHz, $CDCl_3$, 298 K)

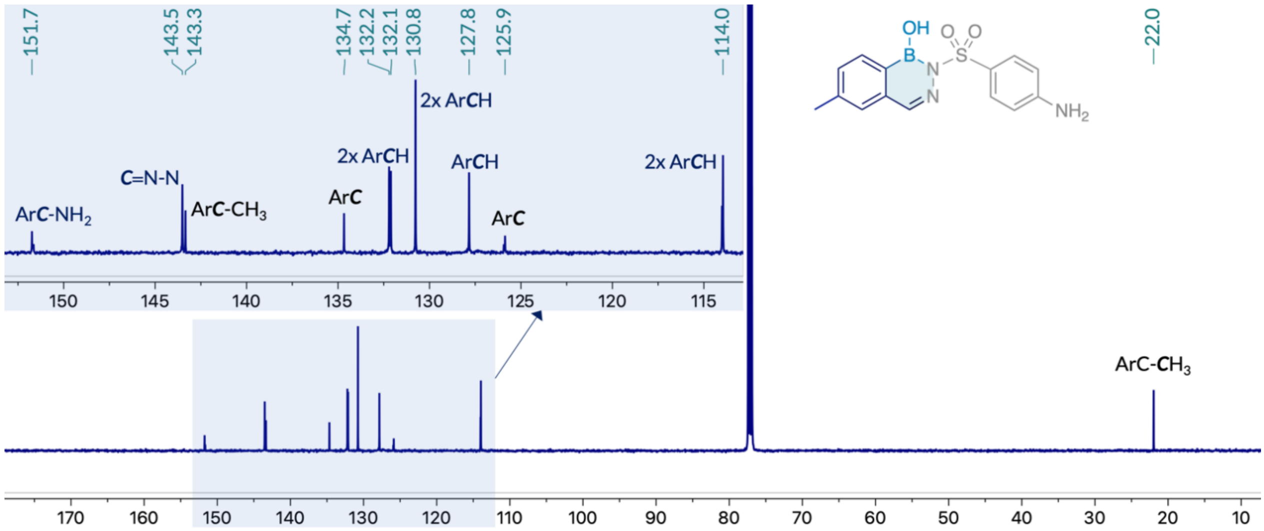

Figure S51. Diazaborine 9:^{13}C NMR (126 MHz, $CDCl_3$, 298 K)

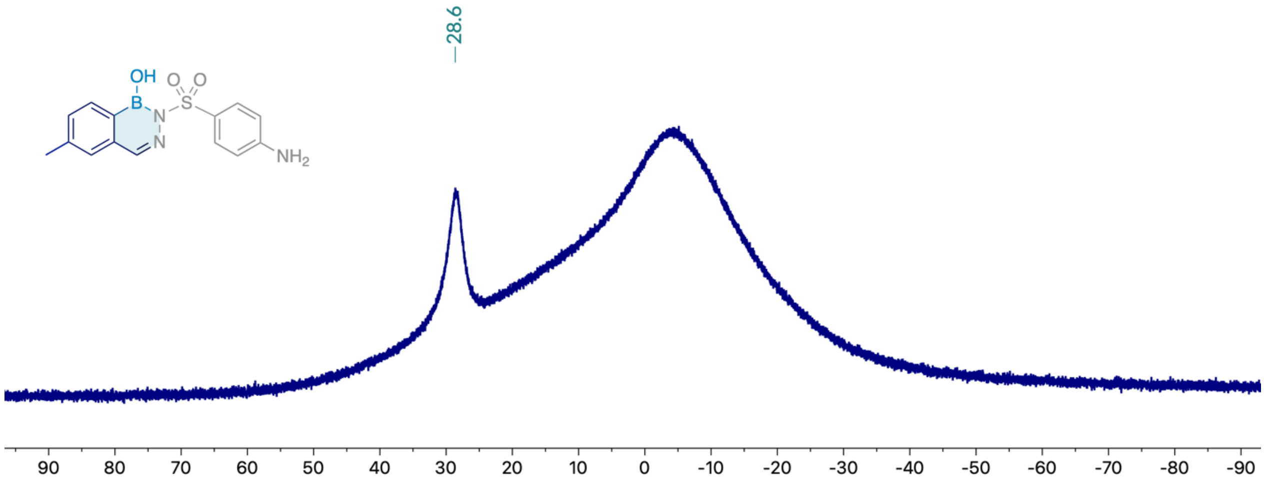

Figure S52. Diazaborine 9:^{11}B NMR (160 MHz, $CDCl_3$, 298 K)

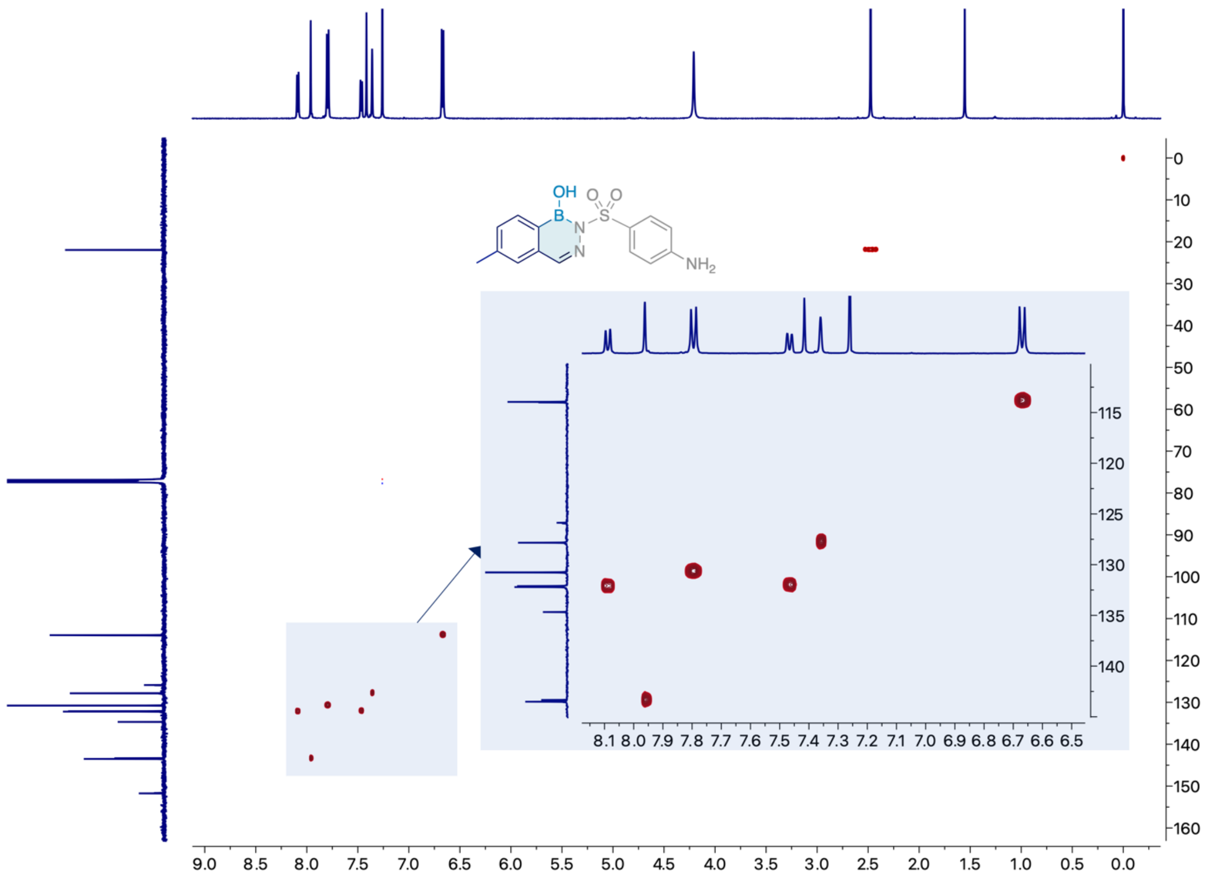

Figure S53. Diazaborine 9:^{1}H-^{13}C gHSQC NMR ($CDCl_3$, 298 K)

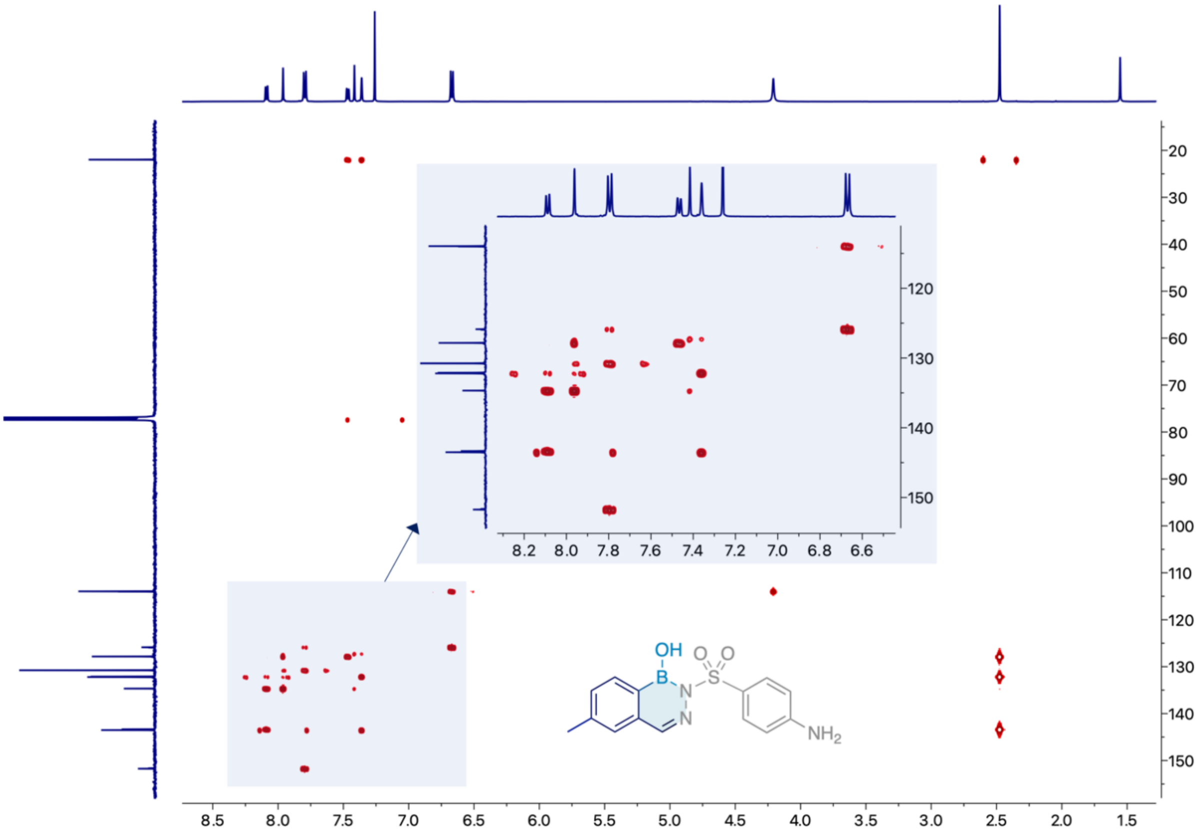

Figure S54. Diazaborine 9:^{1}H-^{13}C gHMBC NMR ($CDCl_3$, 298 K)

Diazaborine 10

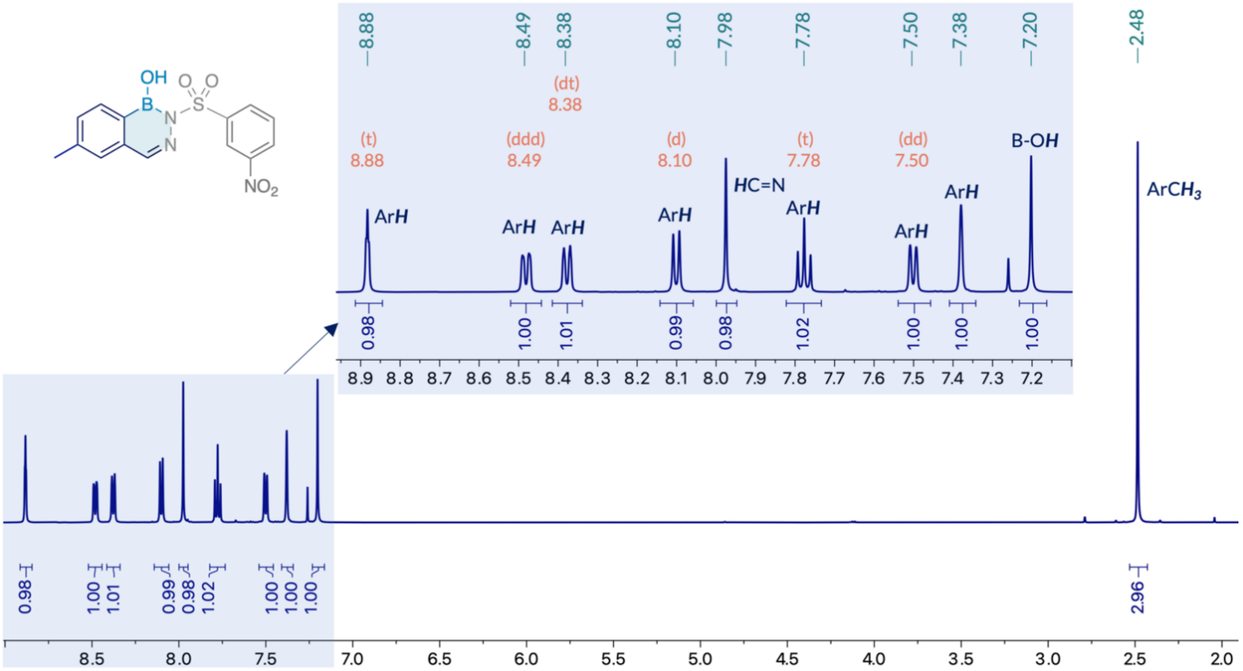

Figure S55. Diazaborine 10: ^{1}H NMR (500 MHz, $CDCl_3$, 298 K)

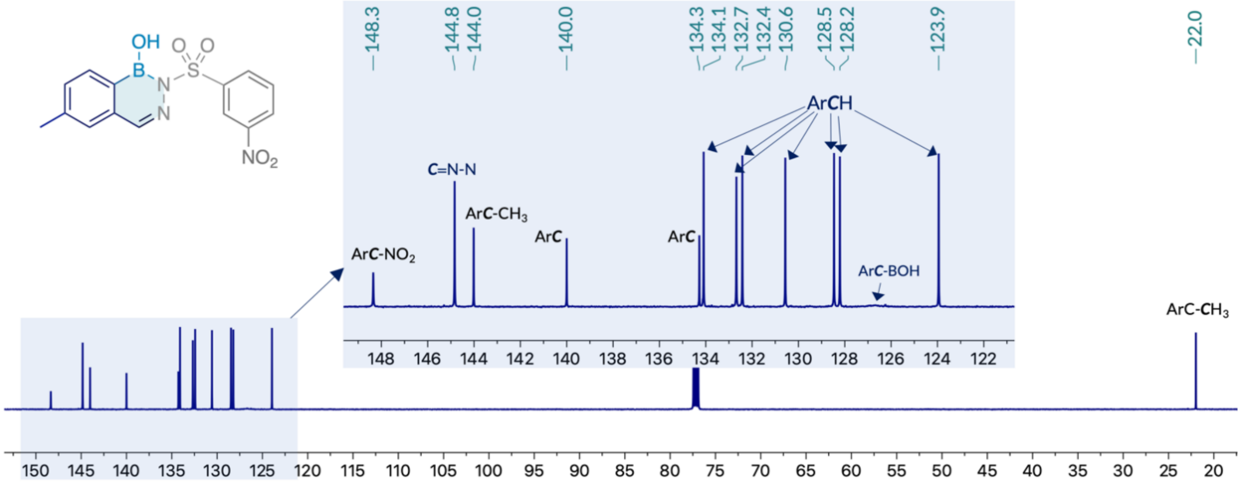

Figure S56. Diazaborine 10: ^{13}C NMR (126 MHz, $CDCl_3$, 298 K)

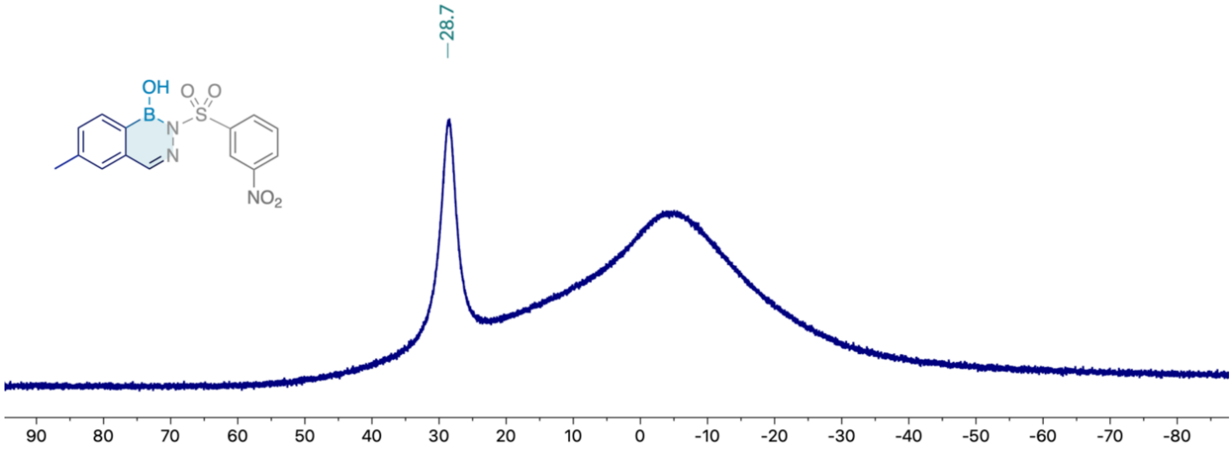

Figure S57. Diazaborine 10: ^{11}B NMR (160 MHz, $CDCl_3$, 298 K)

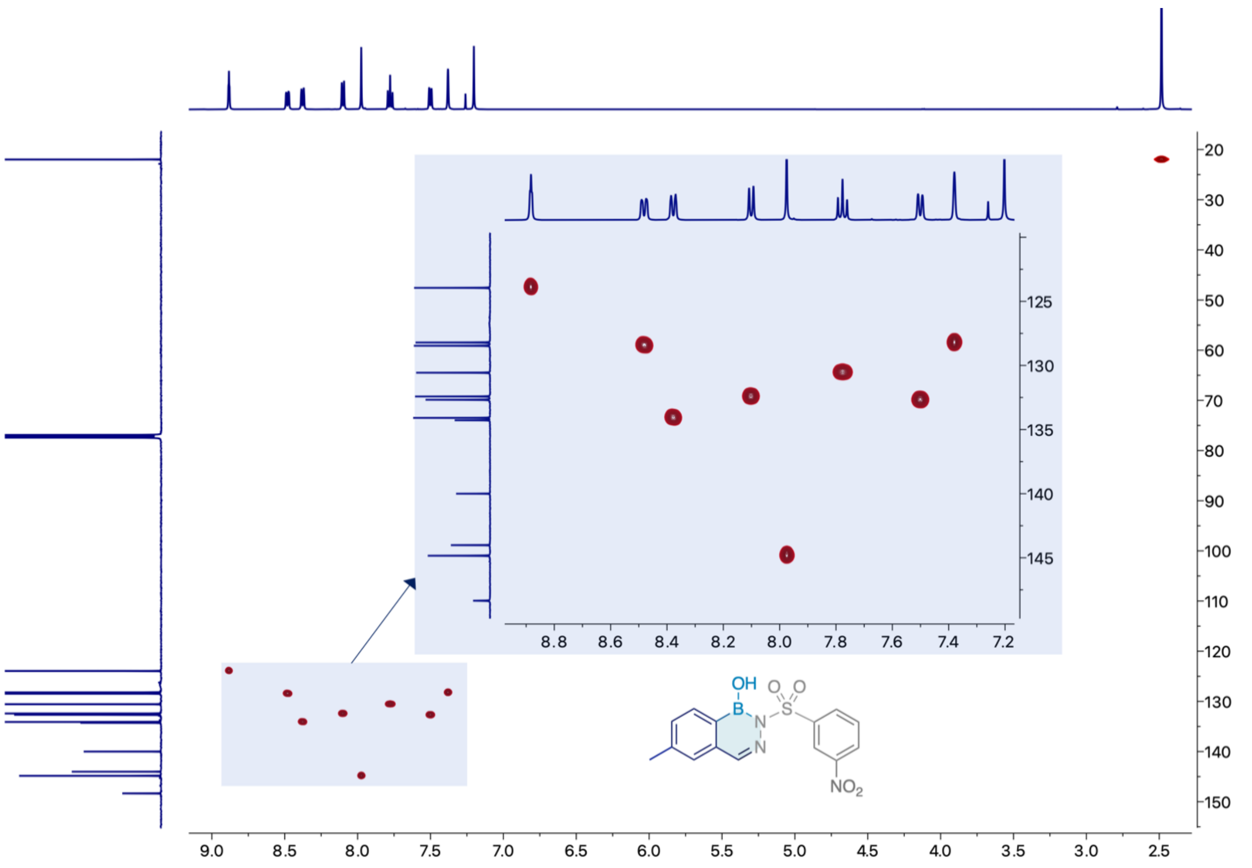

Figure S58. Diazaborine 10:^{1}H-^{13}C gHSQC NMR ($CDCl_3$, 298 K)

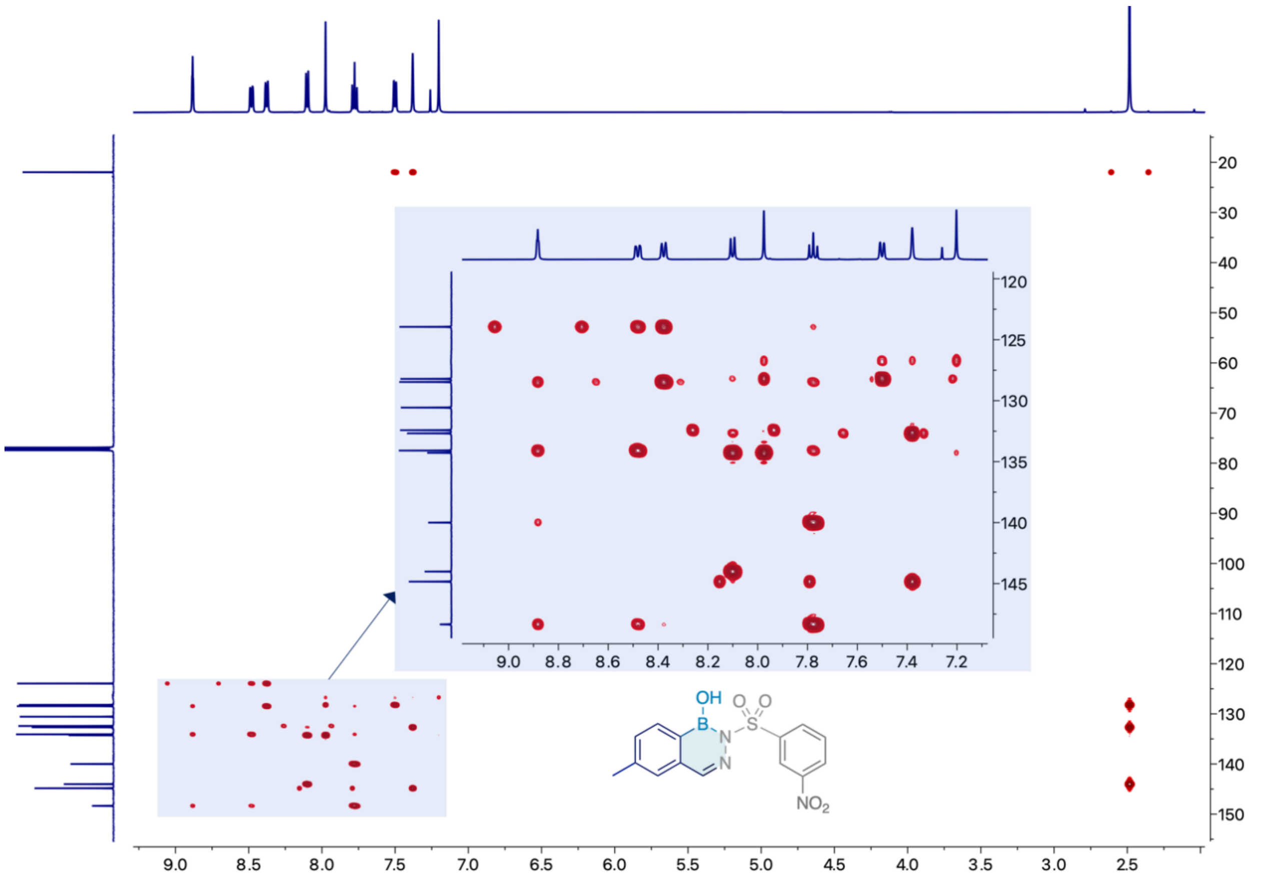

Figure S59. Diazaborine 10:^{1}H-^{13}C gHMBC NMR ($CDCl_3$, 298 K)

Diazaborine 11

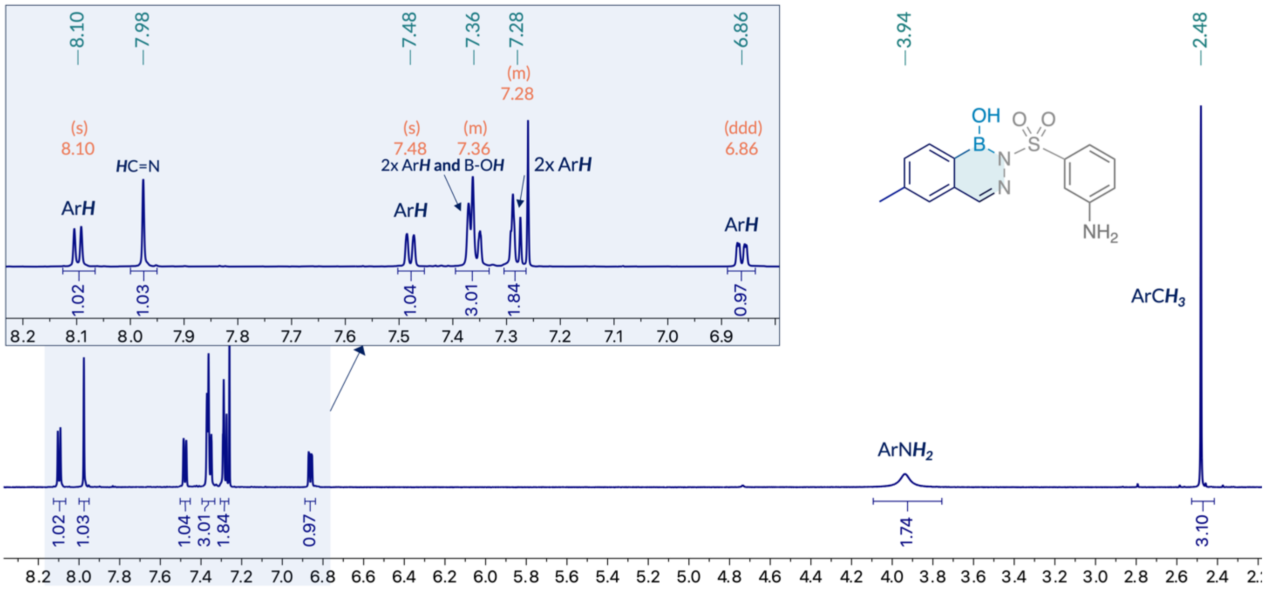

Figure S60. Diazaborine 11: ^{1}H NMR (600 MHz, $CDCl_3$, 298 K)

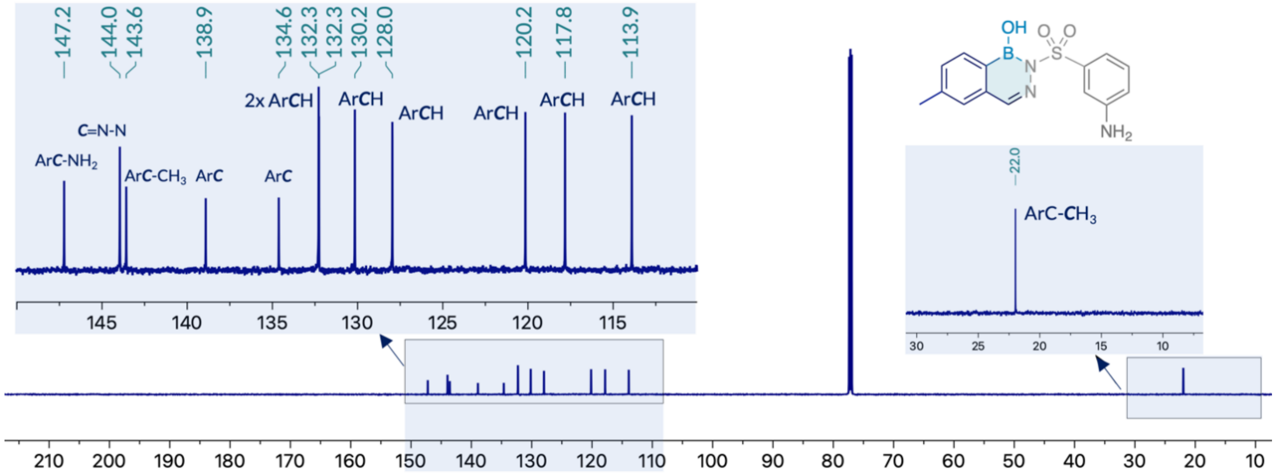

Figure S61. Diazaborine 11: ^{13}C NMR (151 MHz, $CDCl_3$, 298 K)

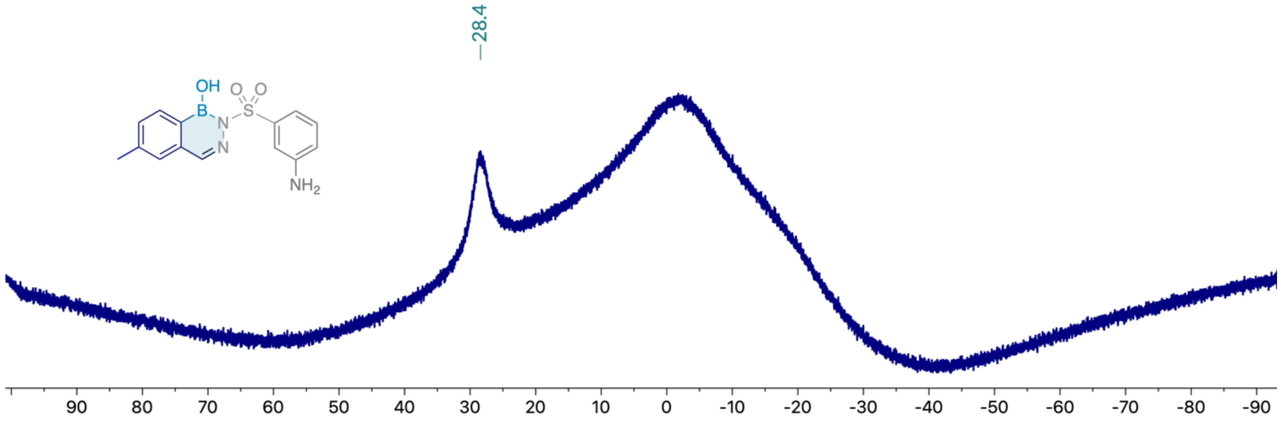

Figure S62. Diazaborine 11: ^{11}B NMR (128 MHz, $CDCl_3$, 298 K)

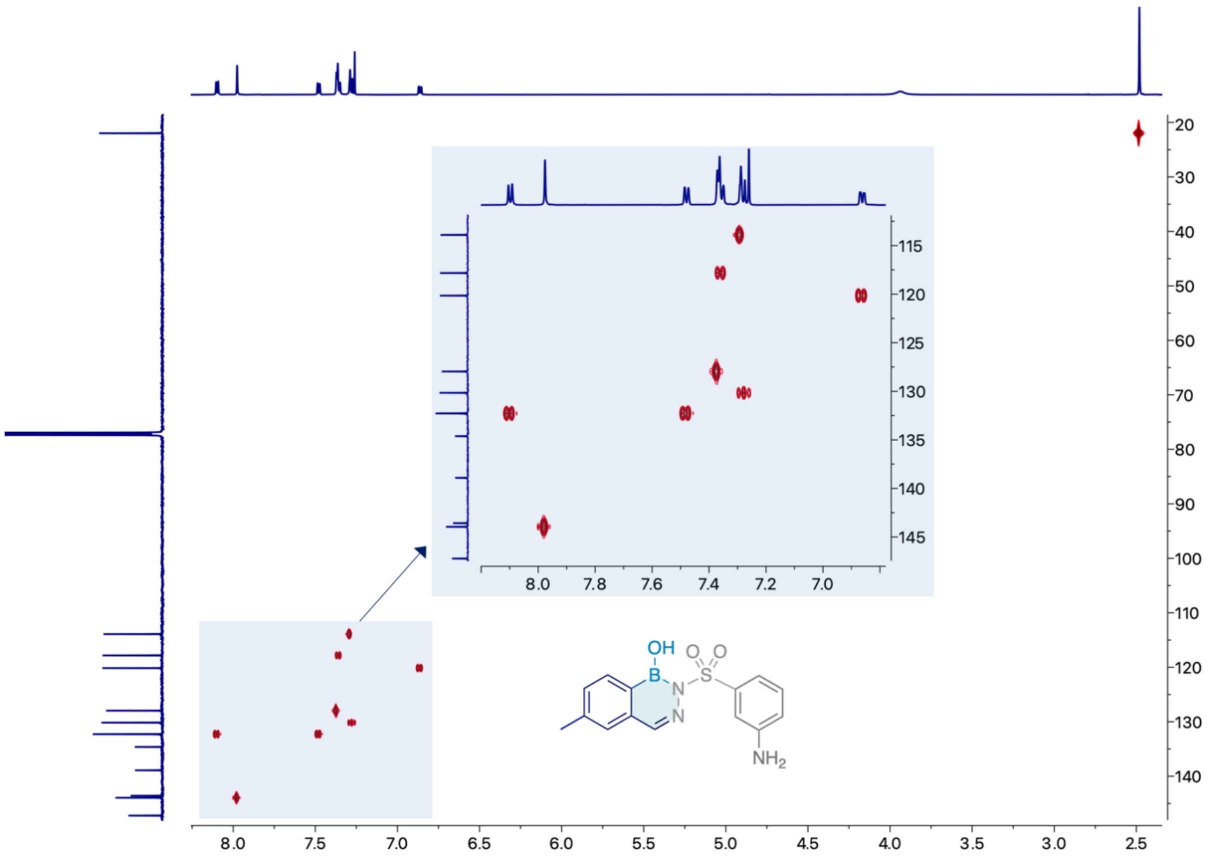

Figure S63. Diazaborine 11:^{1}H-^{13}C gHSQC NMR ($CDCl_3$, 298 K)

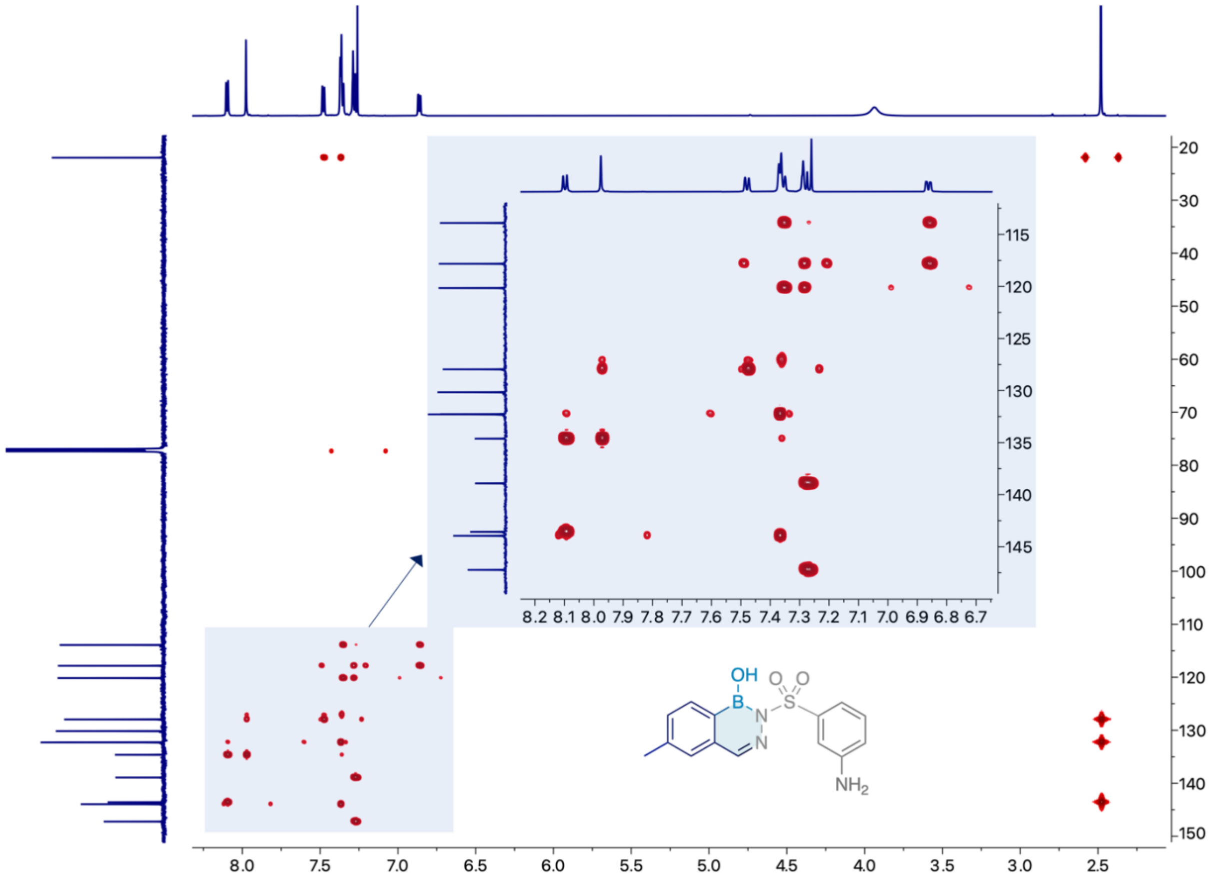

Figure S64. Diazaborine 11:^{1}H-^{13}C gHMBC NMR ($CDCl_3$, 298 K)

Diazaborine 12

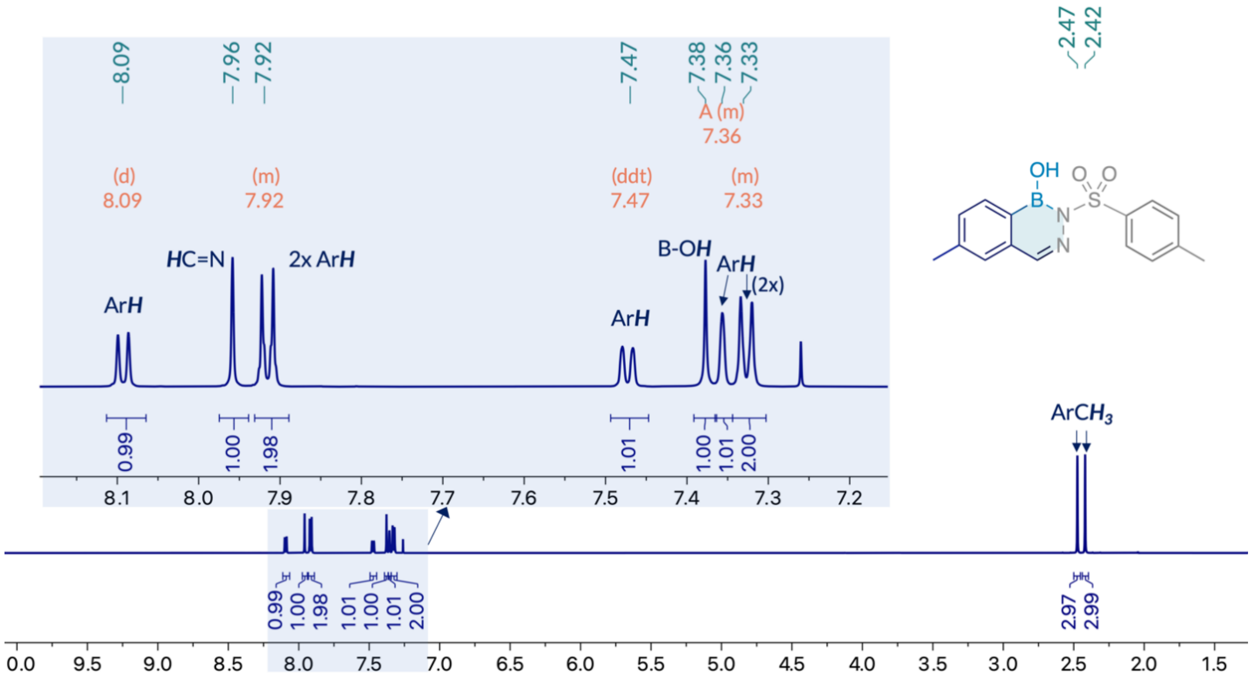

Figure S65. Diazaborine 12: ^{1}H NMR (600 MHz, $CDCl_3$, 298 K)

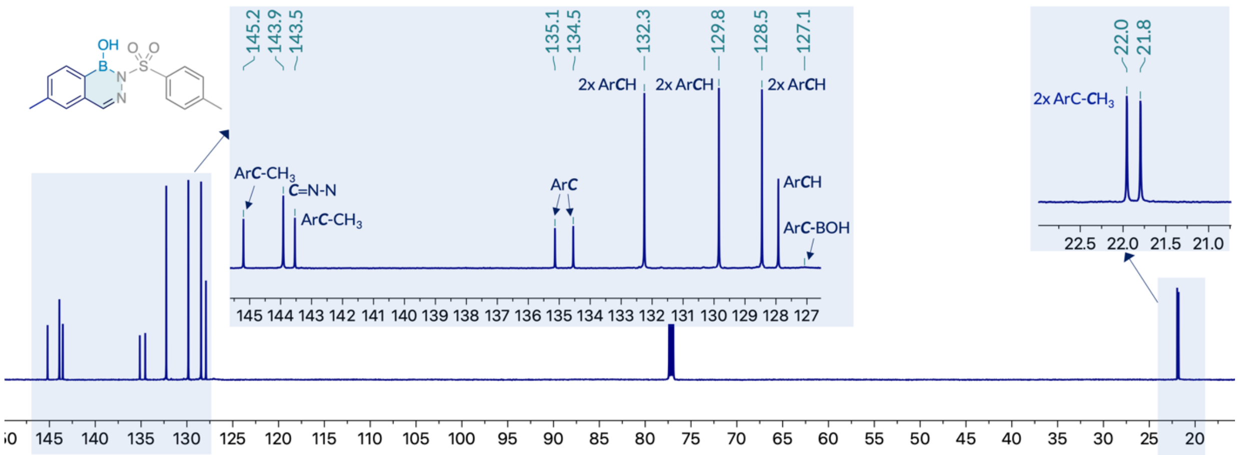

Figure S66. Diazaborine 12: ^{13}C NMR (151 MHz, $CDCl_3$, 298 K)

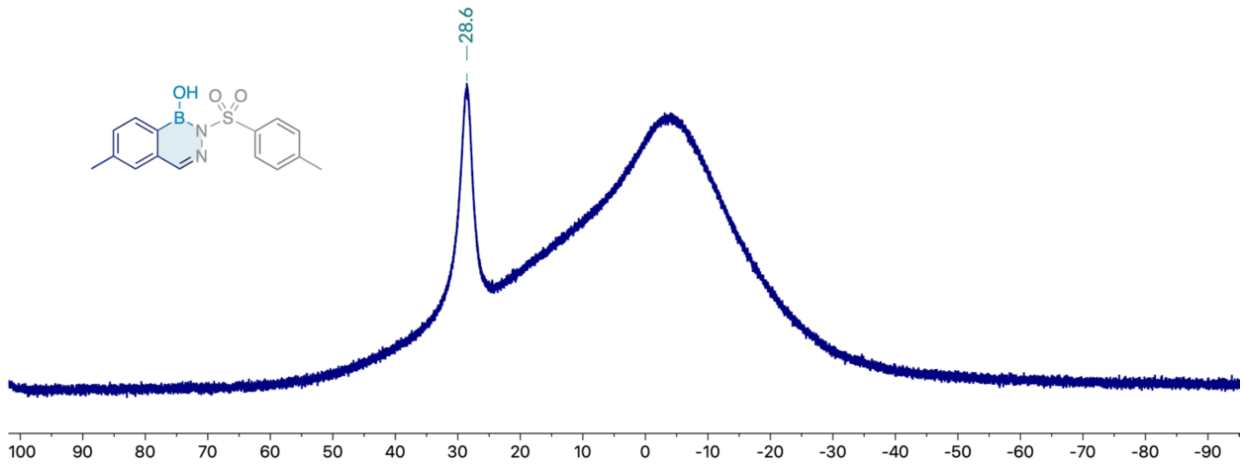

Figure S67. Diazaborine 12: ^{11}B NMR (160 MHz, $CDCl_3$, 298 K)

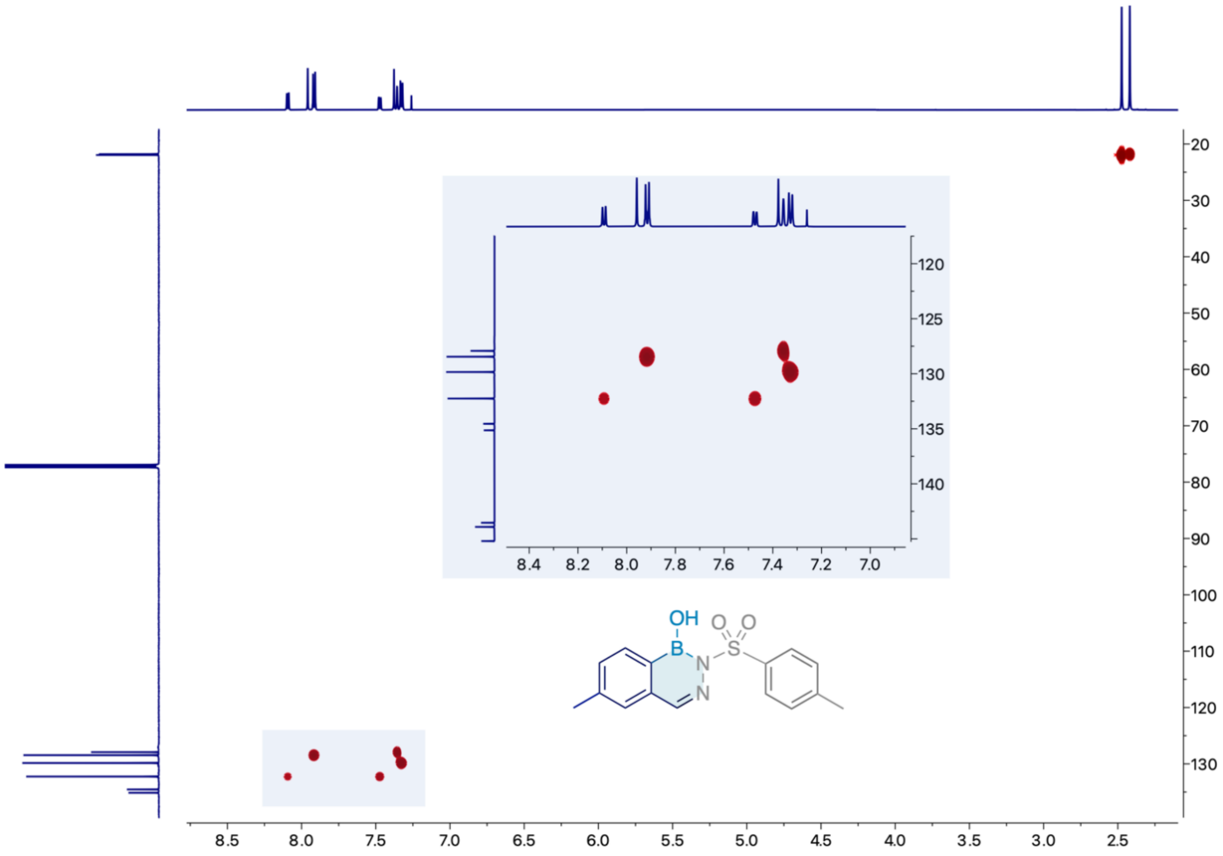

Figure S68. Diazaborine 12:^{1}H-^{13}C gHSQC NMR ($CDCl_3$, 298 K)

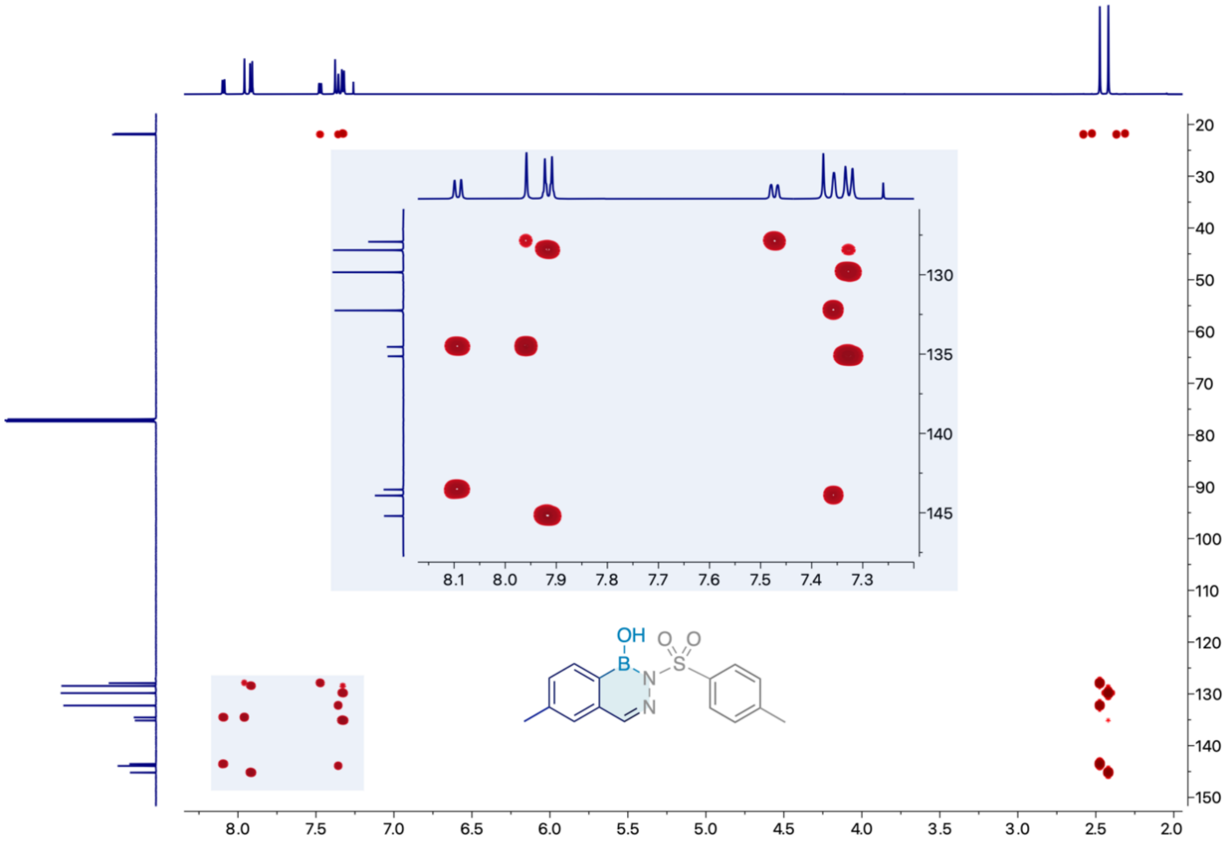

Figure S69. Diazaborine 12:^{1}H-^{13}C gHMBC NMR ($CDCl_3$, 298 K)

Diazaborine 13

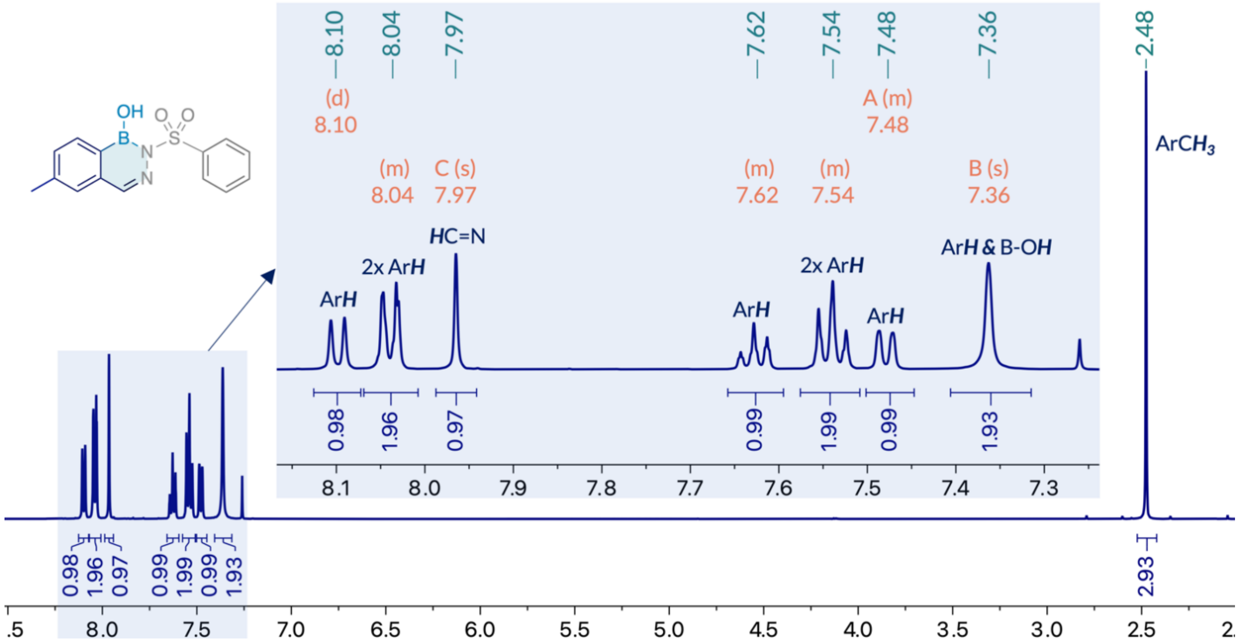

Figure S70. Diazaborine 13:^{1}H NMR (500 MHz, $CDCl_3$, 298 K)

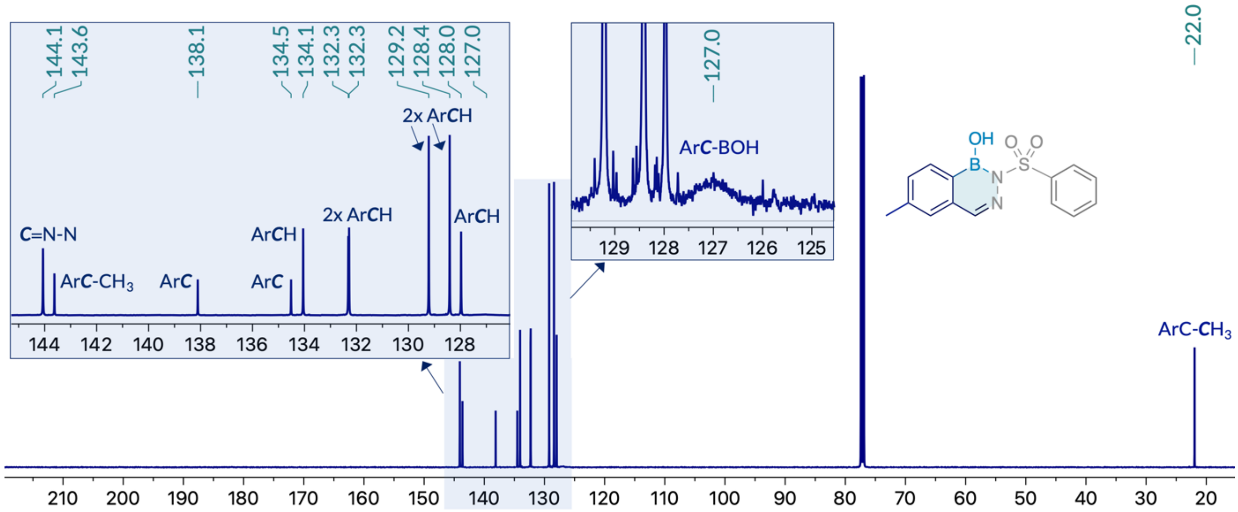

Figure S71. Diazaborine 13:^{13}C NMR (126 MHz, $CDCl_3$, 298 K)

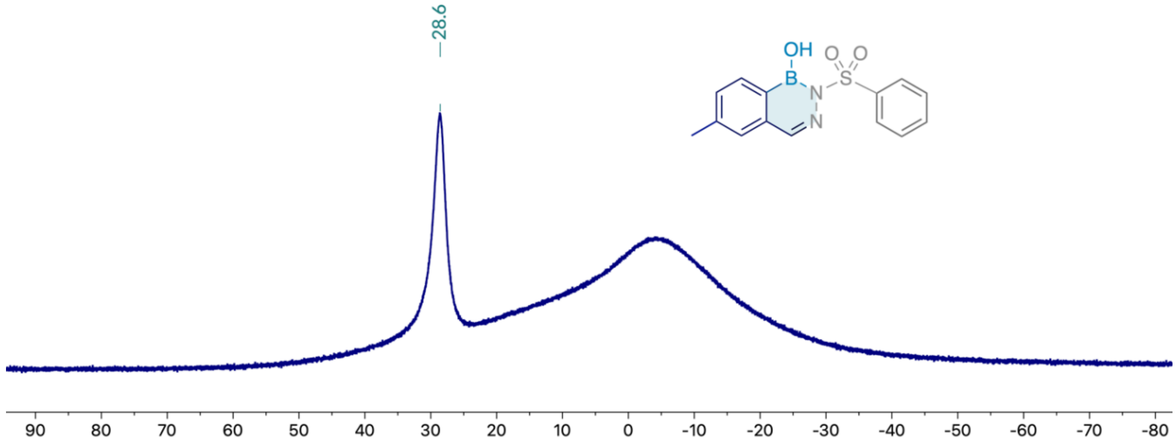

Figure S72. Diazaborine 13:^{11}B NMR (160 MHz, $CDCl_3$, 298 K)

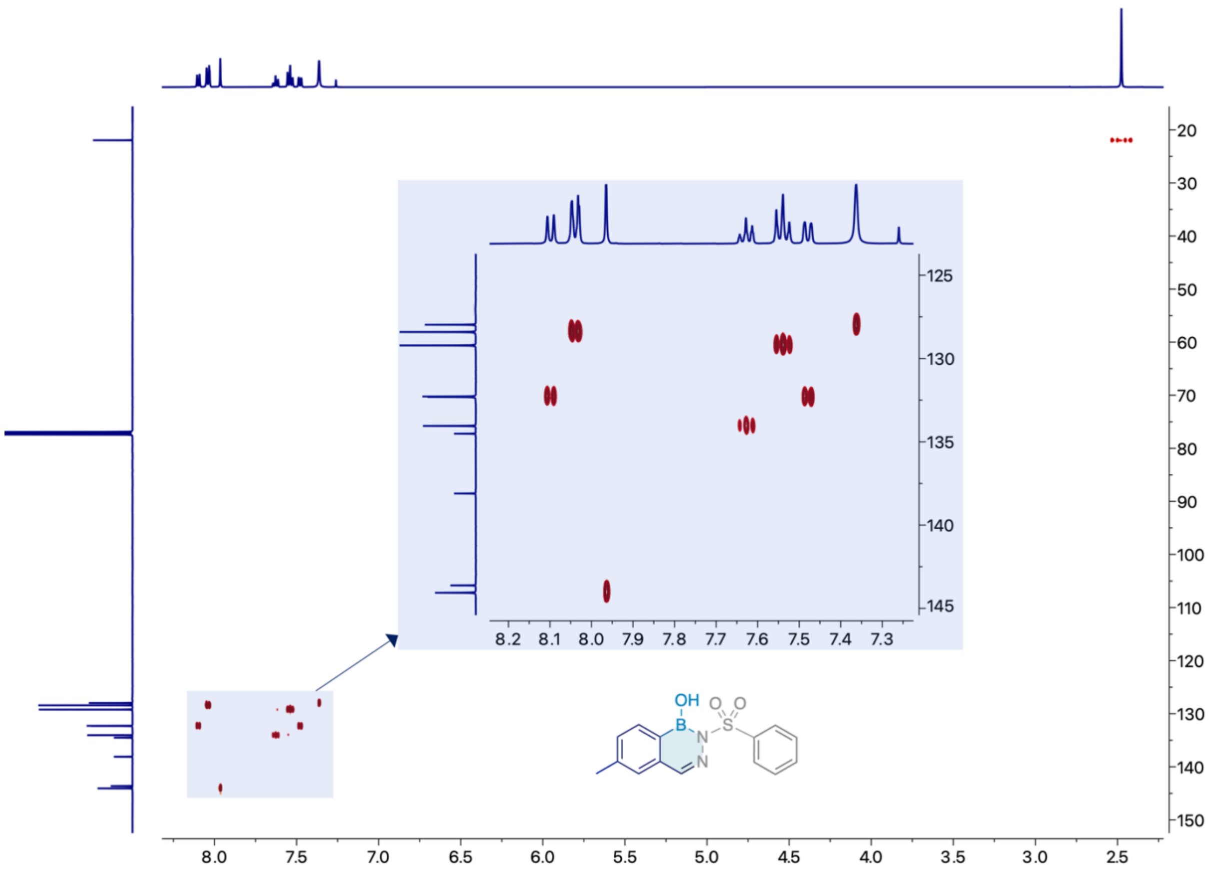

Figure S73. Diazaborine 13:^{1}H-^{13}C gHSQC NMR ($CDCl_3$, 298 K)

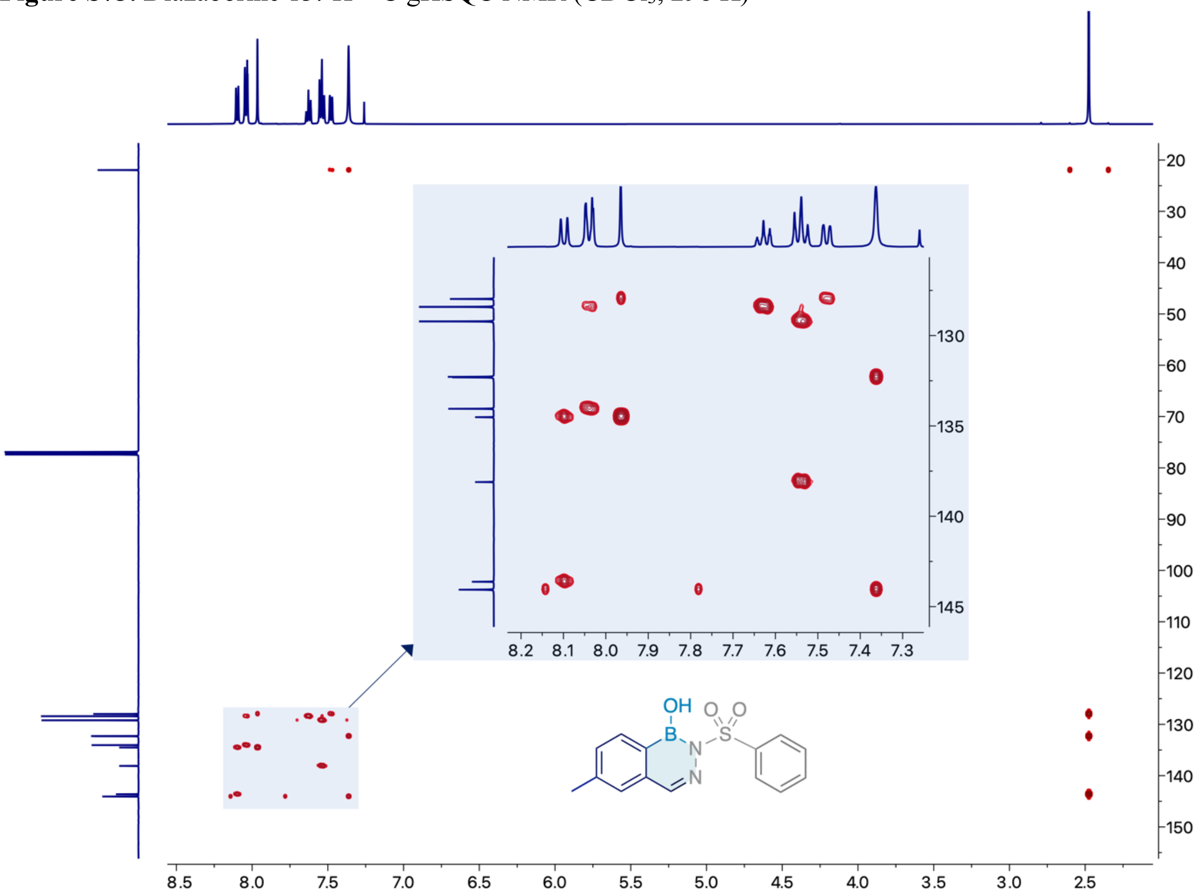

Figure S74. Diazaborine 13:^{1}H-^{13}C gHMBC NMR ($CDCl_3$, 298 K)

Diazaborine 14

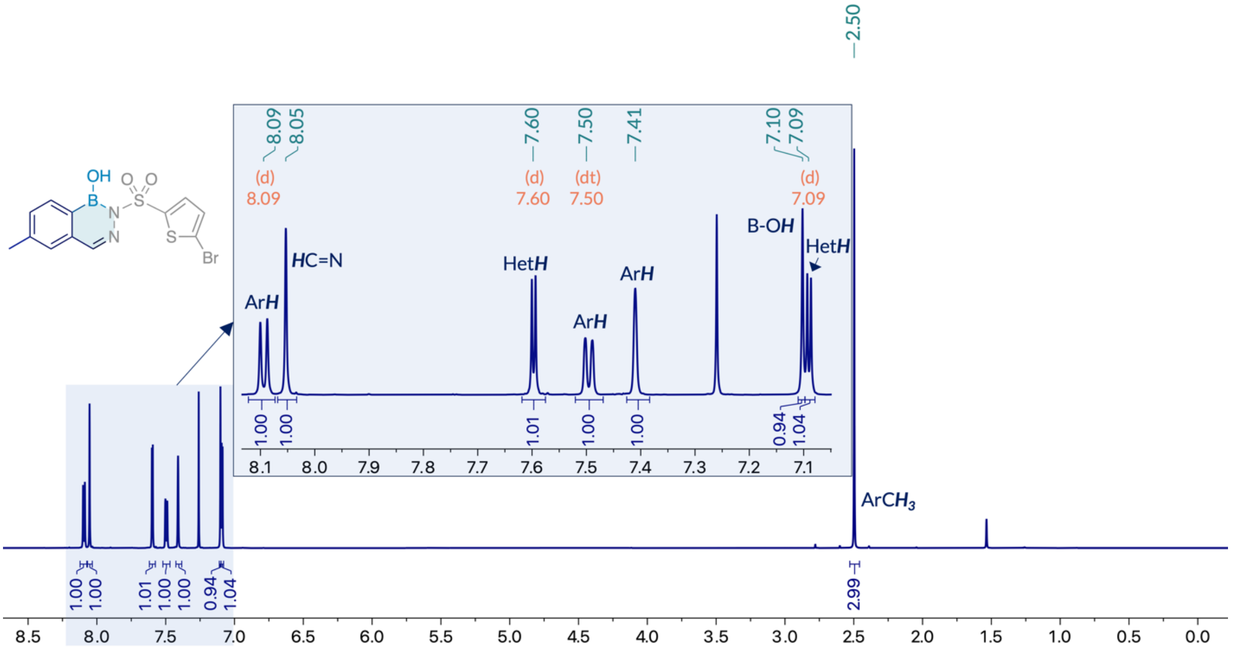

Figure S75. Diazaborine 14:^{1}H NMR (600 MHz, $CDCl_3$, 298 K)

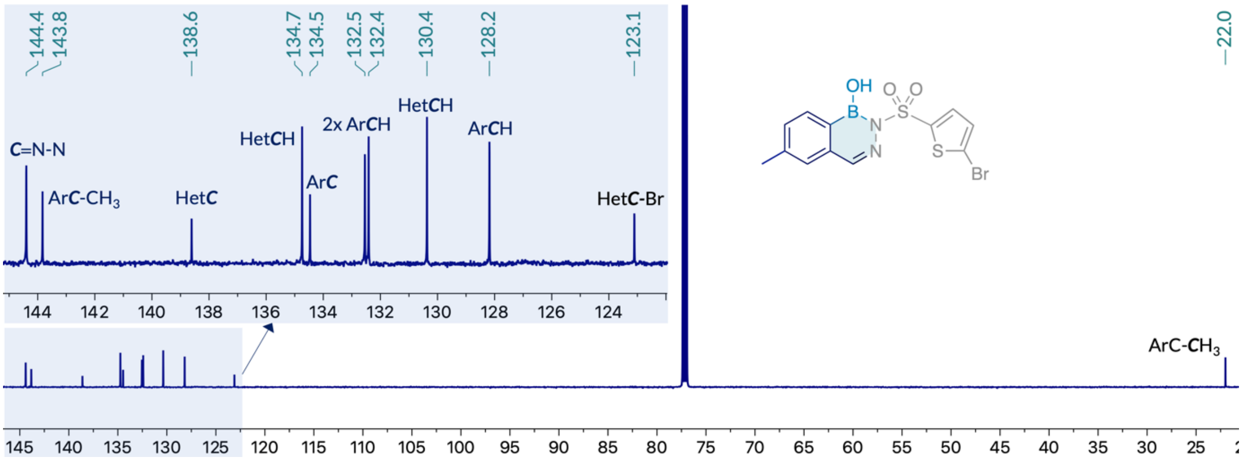

Figure S76. Diazaborine 14:^{13}C NMR (151 MHz, $CDCl_3$, 298 K)

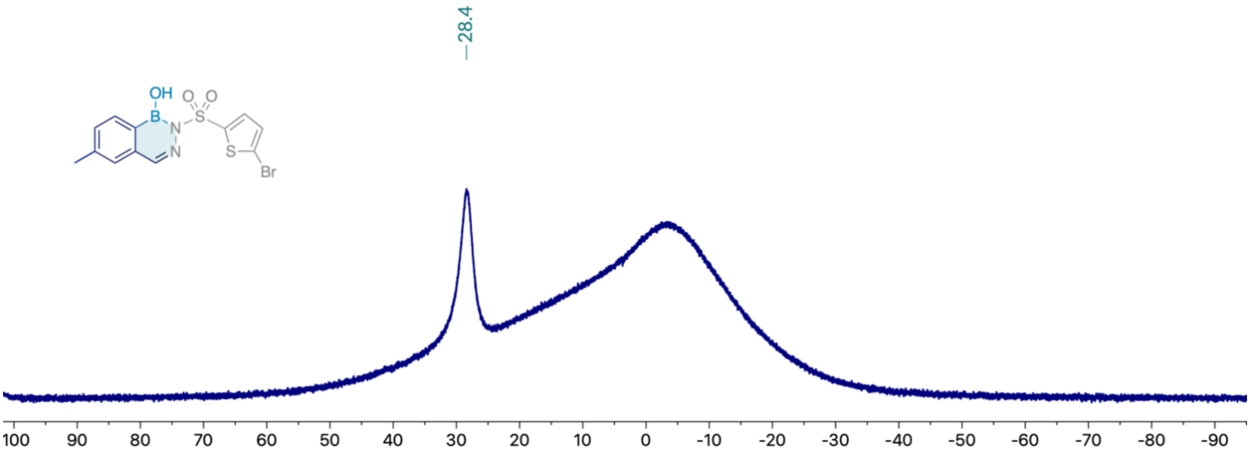

Figure S77. Diazaborine 14:^{11}B NMR (160 MHz, $CDCl_3$, 298 K)

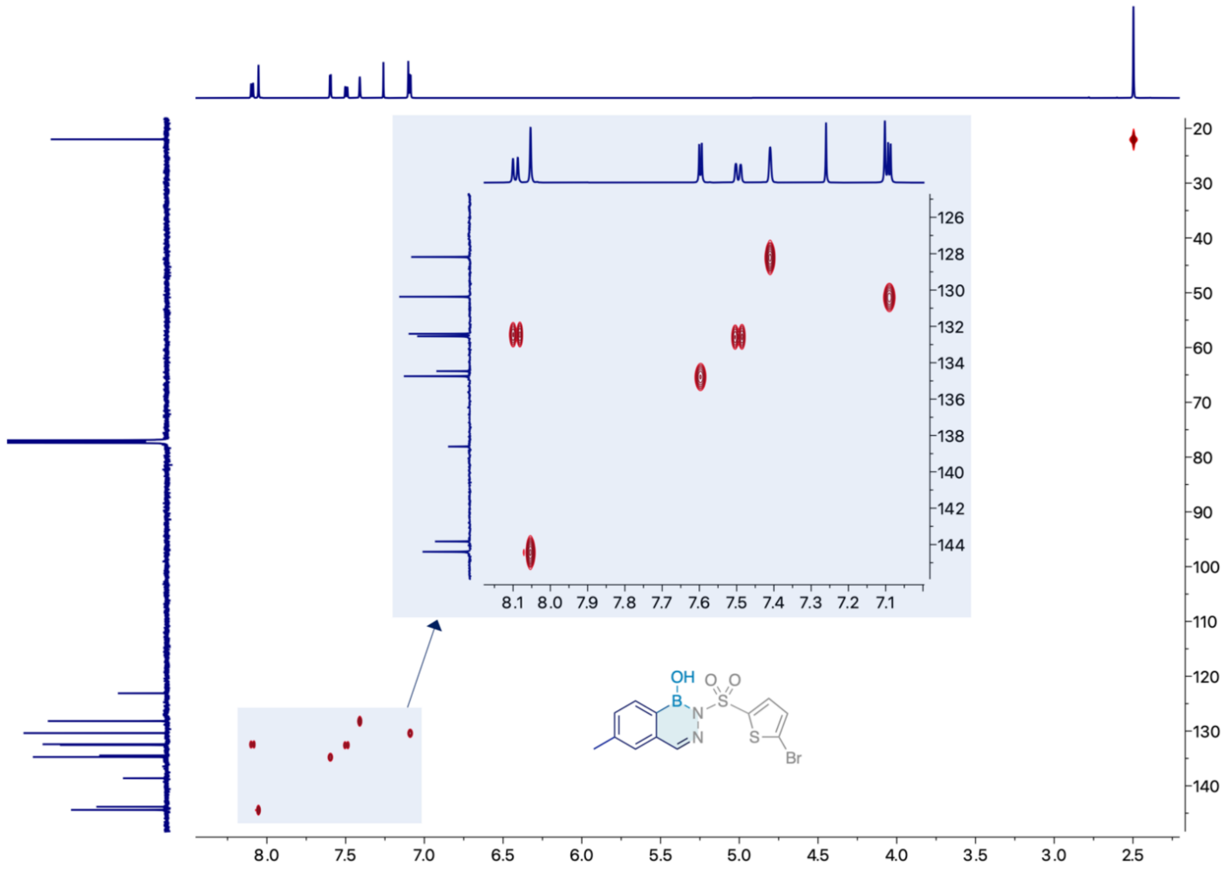

Figure S78. Diazaborine 14: ^{1}H-^{13}C gHSQC NMR ($CDCl_3$, 298 K)

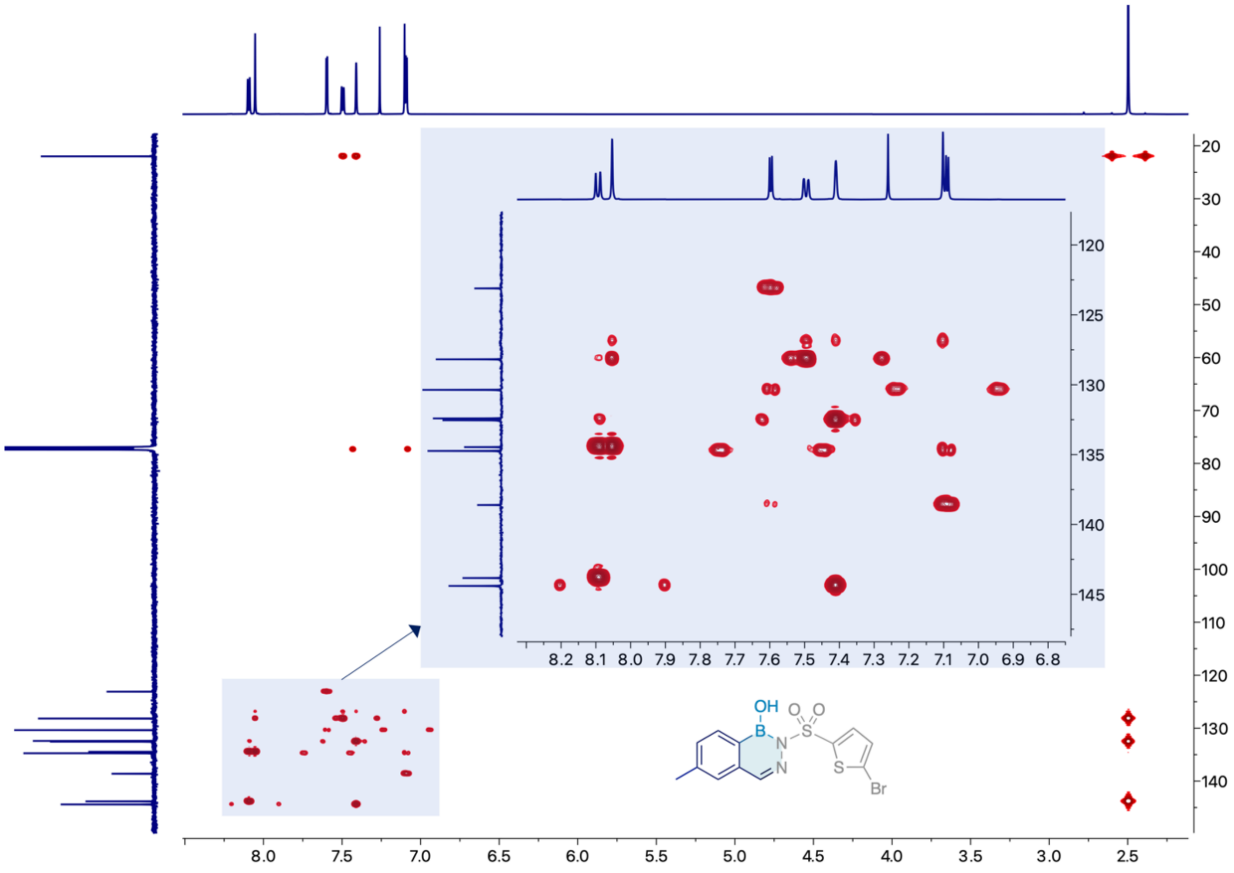

Figure S79. Diazaborine 14: ^{1}H-^{13}C gHMBC NMR ($CDCl_3$, 298 K)

Diazaborine 15

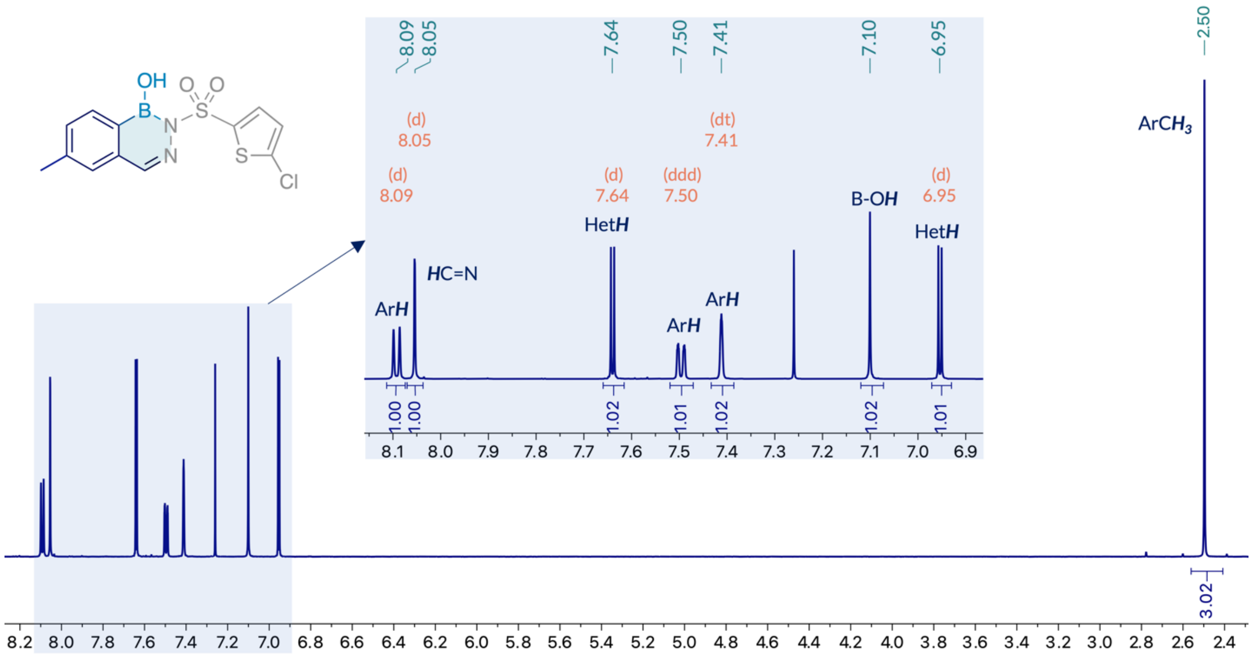

Figure S80. Diazaborine 15: ^{1}H NMR (600 MHz, $CDCl_3$, 298 K)

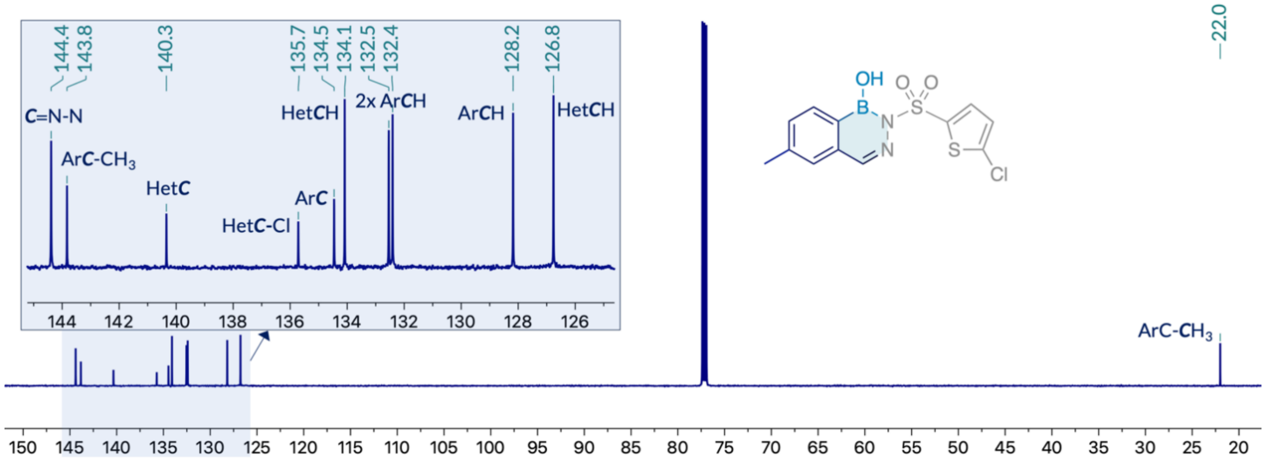

Figure S81. Diazaborine 15: ^{13}C NMR (151 MHz, $CDCl_3$, 298 K)

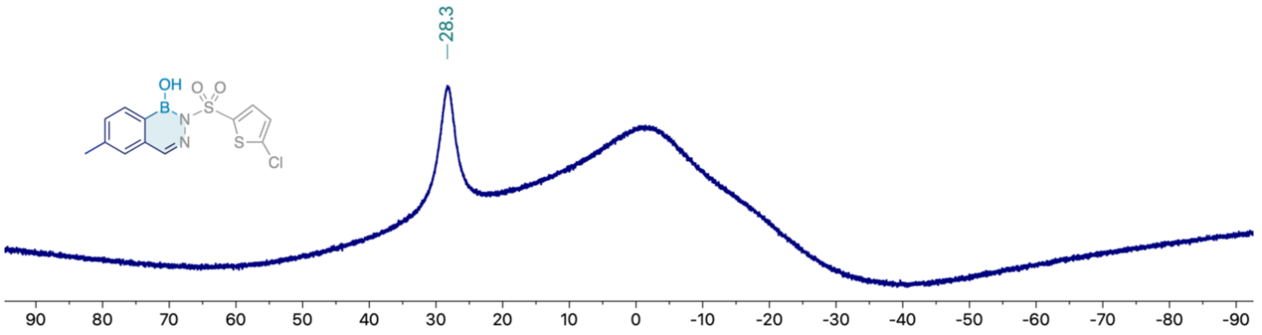

Figure S82. Diazaborine 15: ^{11}B NMR (160 MHz, $CDCl_3$, 298 K)

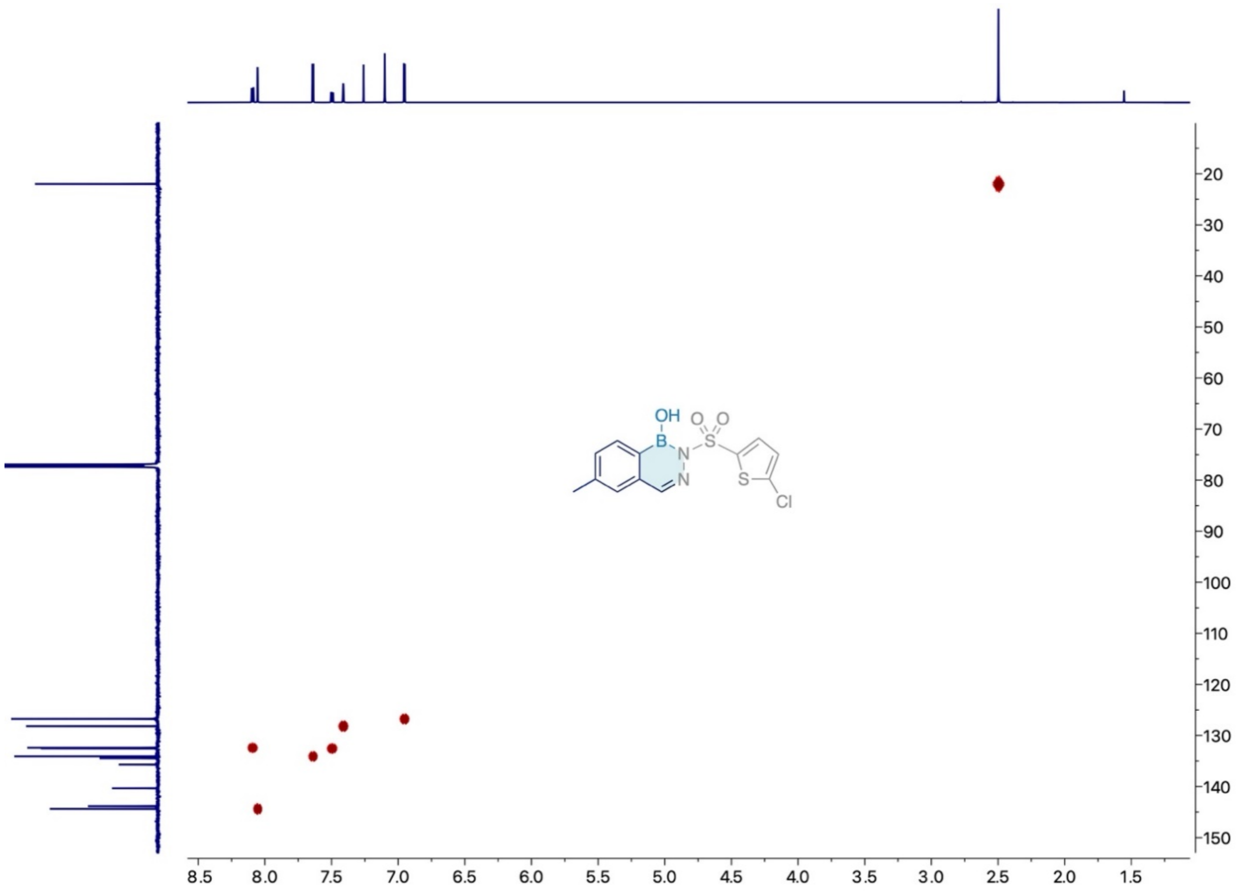

Figure S83. Diazaborine 15:^{1}H-^{13}C gHSQC NMR ($CDCl_3$, 298 K)

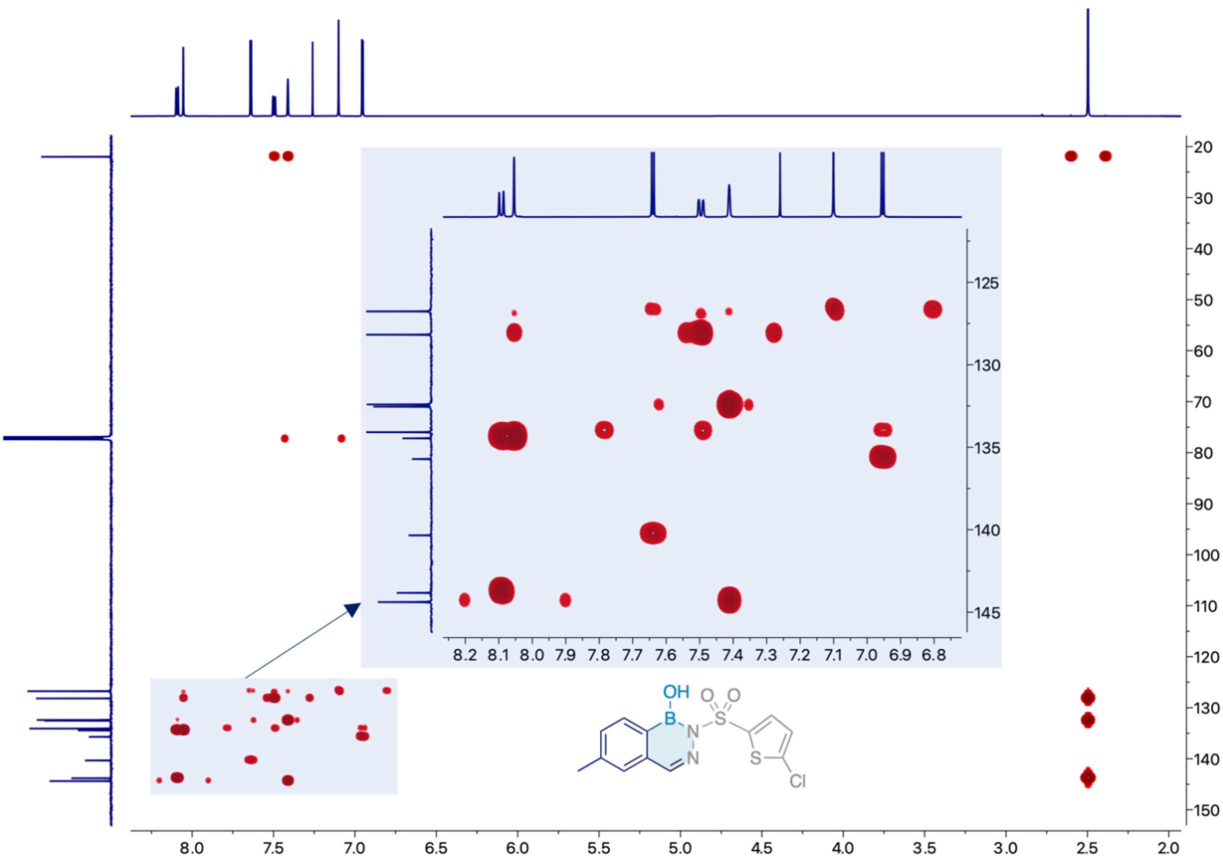

Figure S84. Diazaborine 15:^{1}H-^{13}C gHMBC NMR ($CDCl_3$, 298 K)

Diazaborine 16

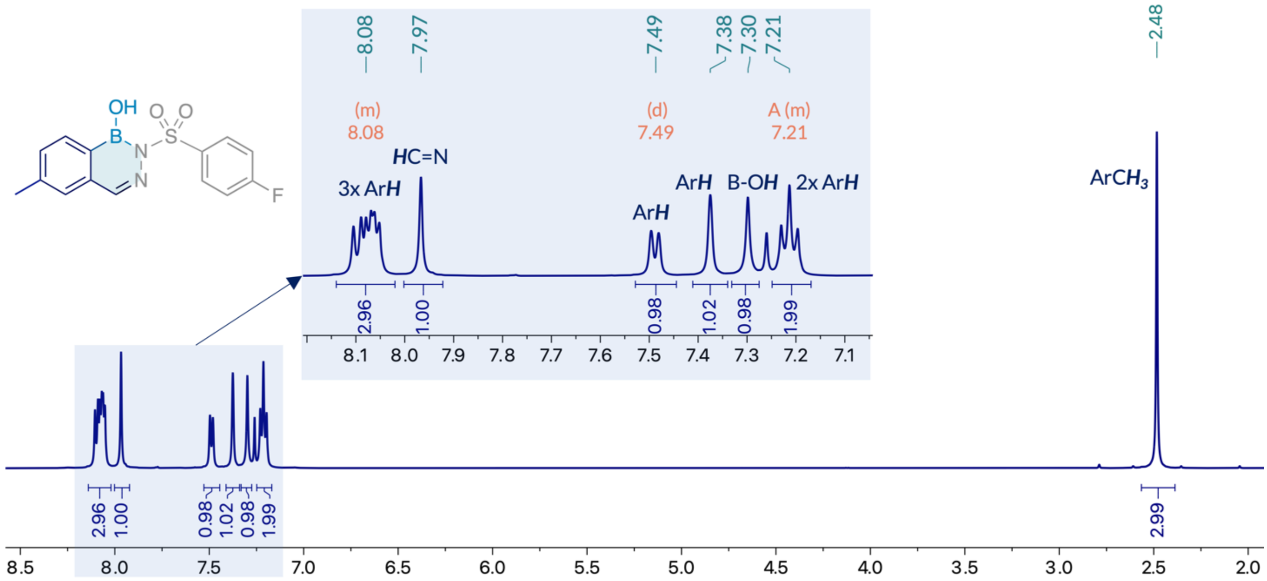

Figure S85. Diazaborine 16:^{1}H NMR (500 MHz, $CDCl_3$, 298 K)

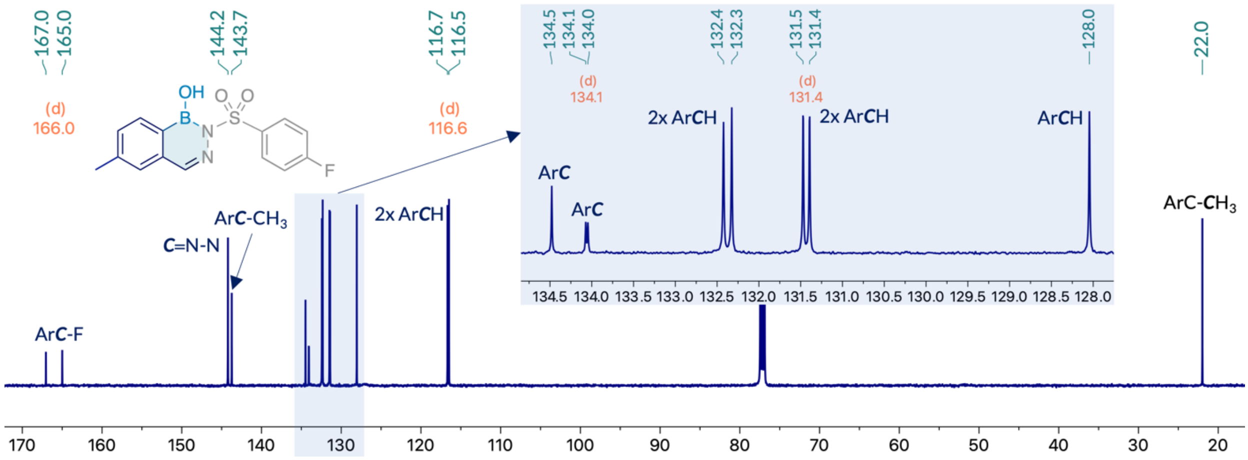

Figure S86. Diazaborine 16:^{13}C NMR (126 MHz, $CDCl_3$, 298 K)

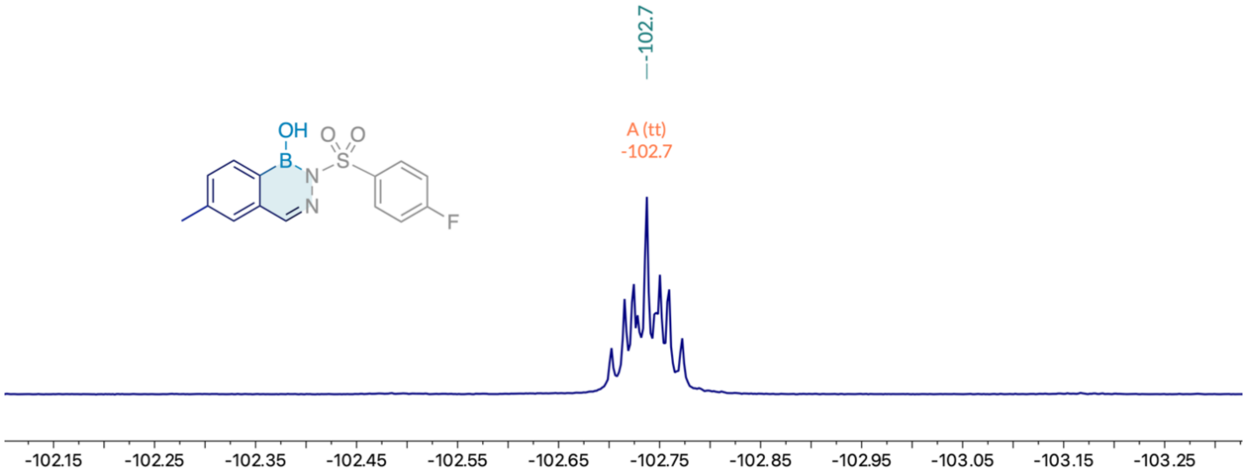

Figure S87. Diazaborine 16:^{19}F NMR (377 MHz, $CDCl_3$, 298 K)

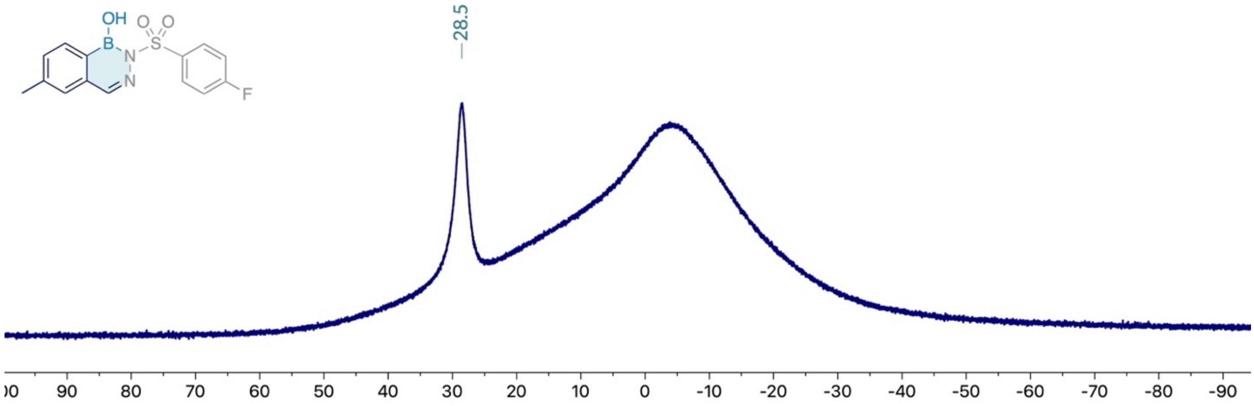

Figure S88. Diazaborine 16:^{11}B NMR (160 MHz, $CDCl_3$, 298 K)

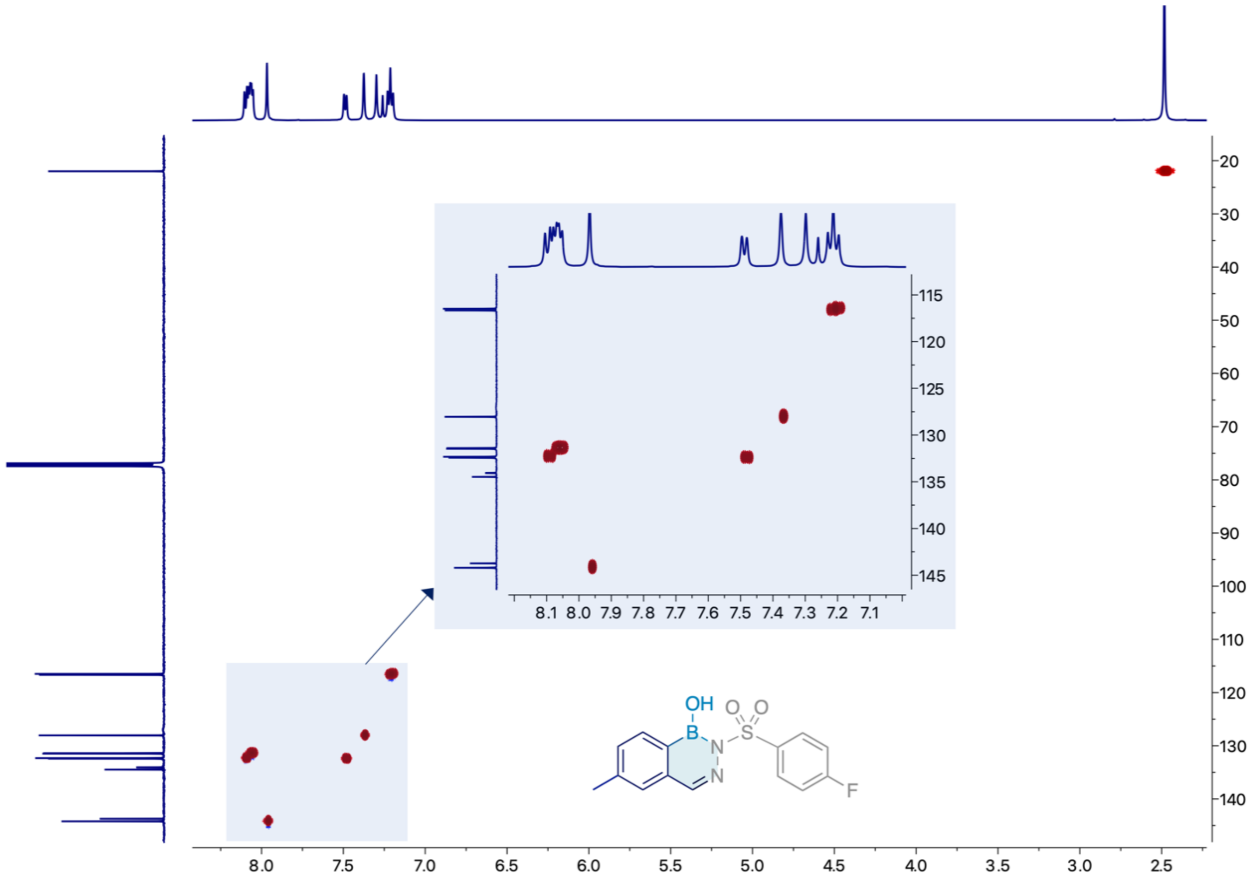

Figure S89. Diazaborine 16:^{1}H-^{13}C gHSQC NMR ($CDCl_3$, 298 K)

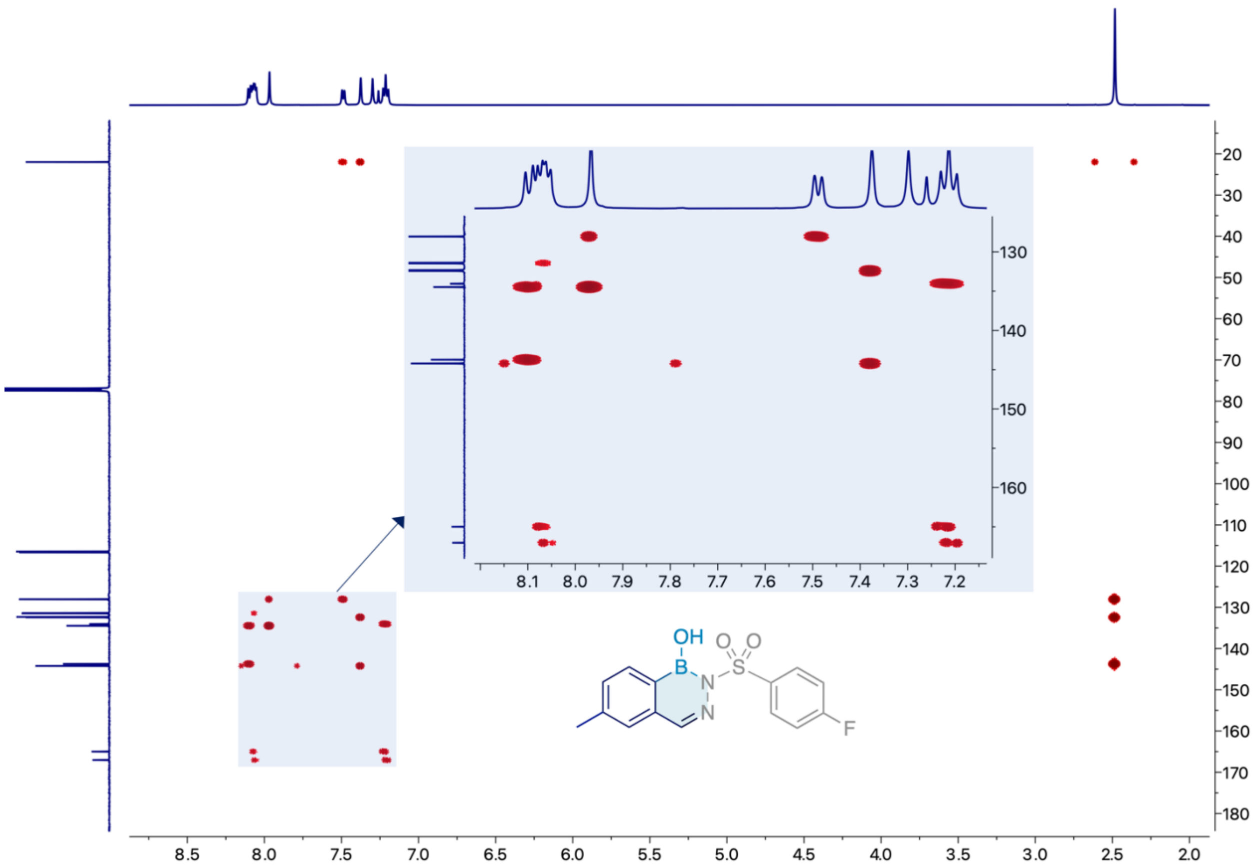

Figure S90. Diazaborine 16:¹H-¹³C gHMBC NMR ($CDCl_3$, 298 K)

Diazaborine 17

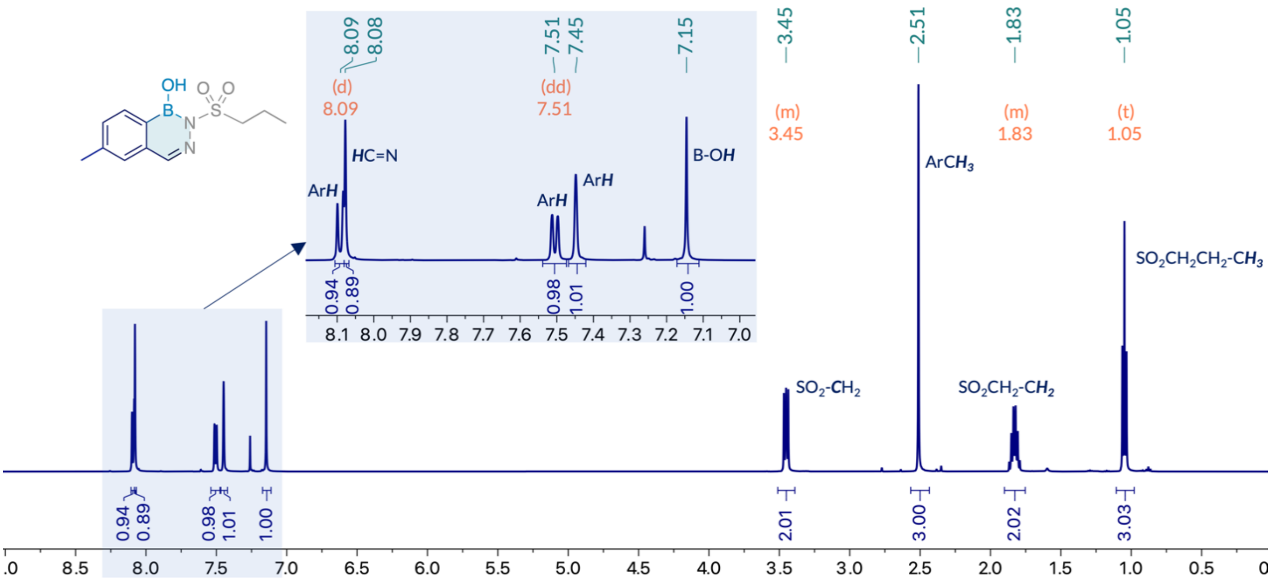

Figure S91. Diazaborine 17 ^{1}H NMR (500 MHz, $CDCl_3$, 298 K)

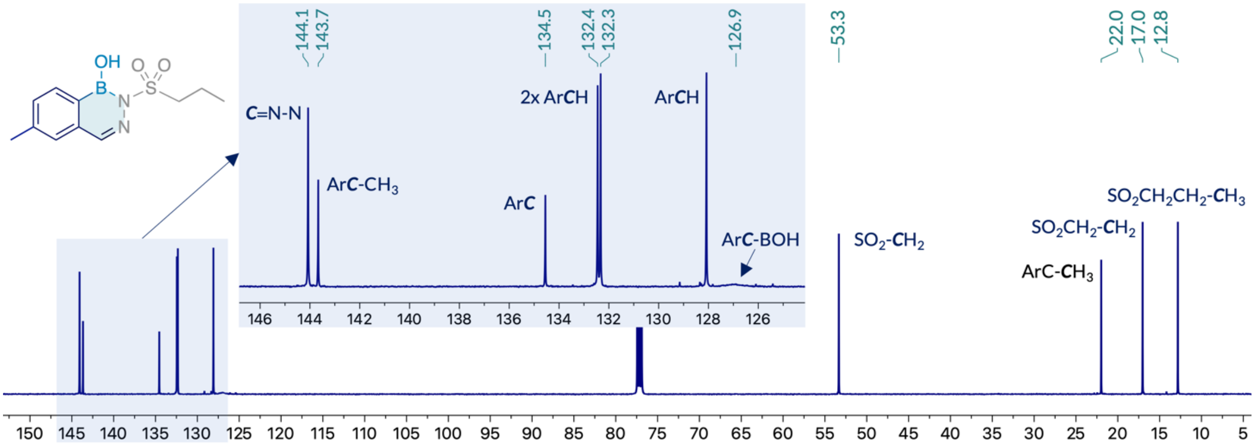

Figure S92. Diazaborine 17:^{13}C NMR (126 MHz, $CDCl_3$, 298 K)

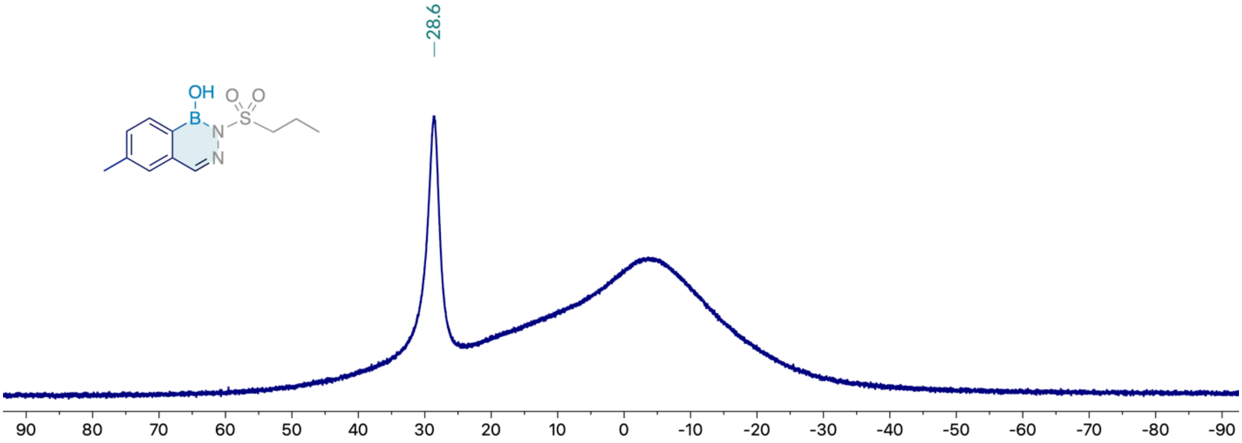

Figure S93. Diazaborine 17:^{11}B NMR (160 MHz, $CDCl_3$, 298 K)

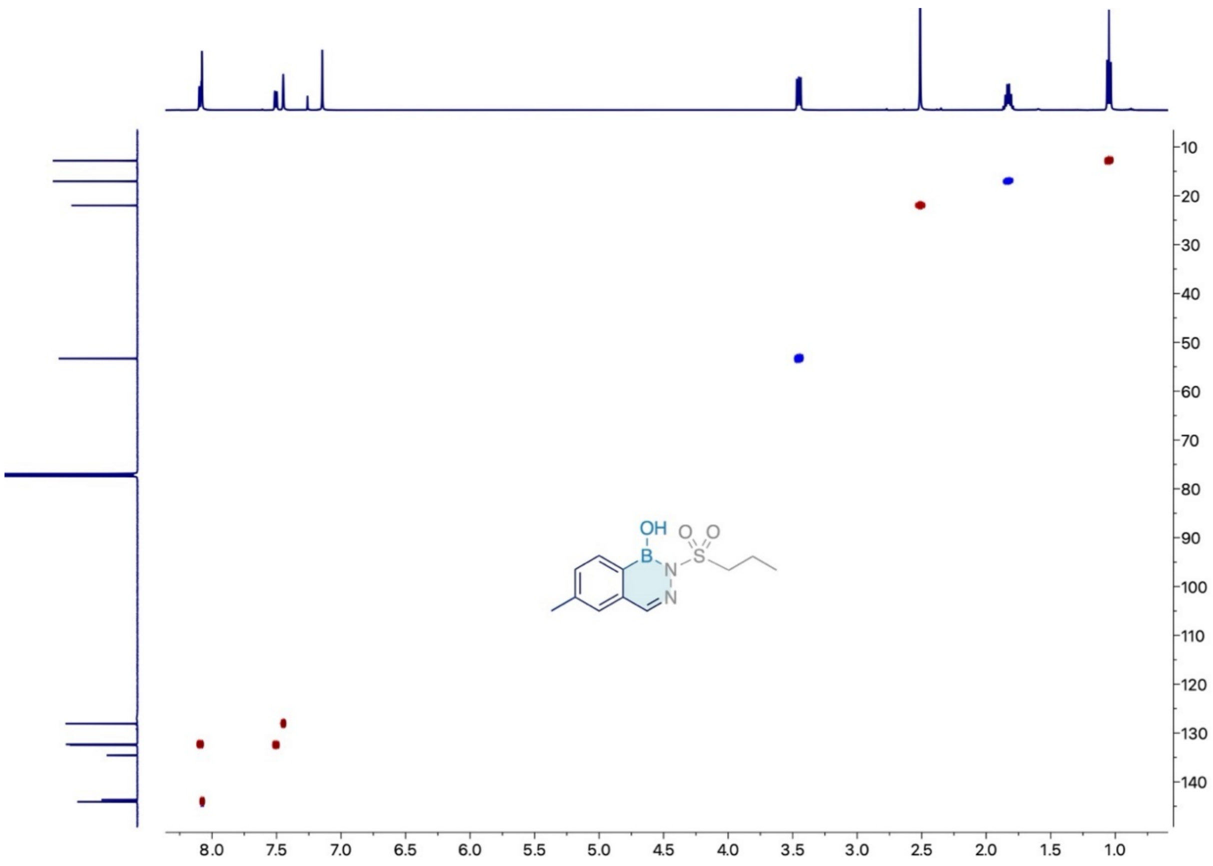

Figure S94. Diazaborine 17: ^{1}H-^{13}C gHSQC NMR ($CDCl_3$, 298 K)

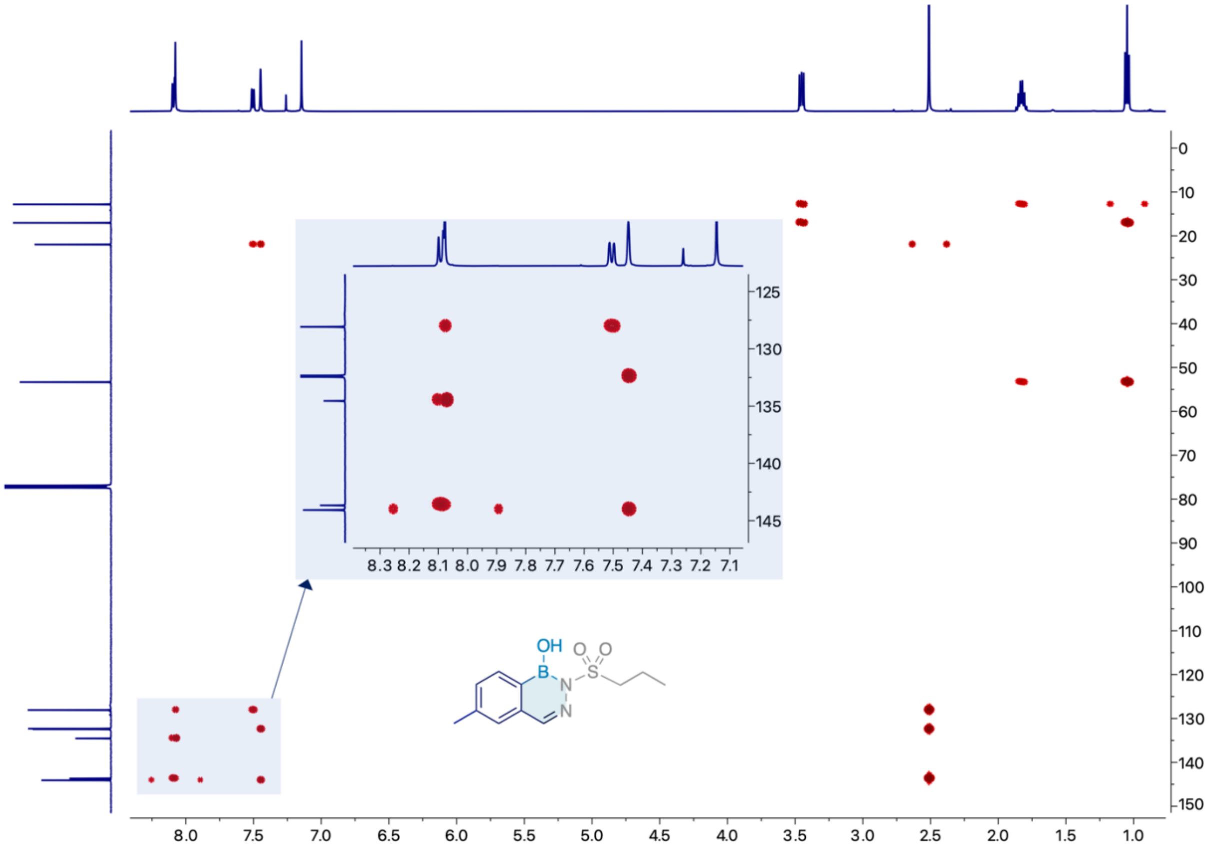

Figure S95. Diazaborine 17: ^{1}H-^{13}C gHMBC NMR ($CDCl_3$, 298 K)

Diazaborine 18

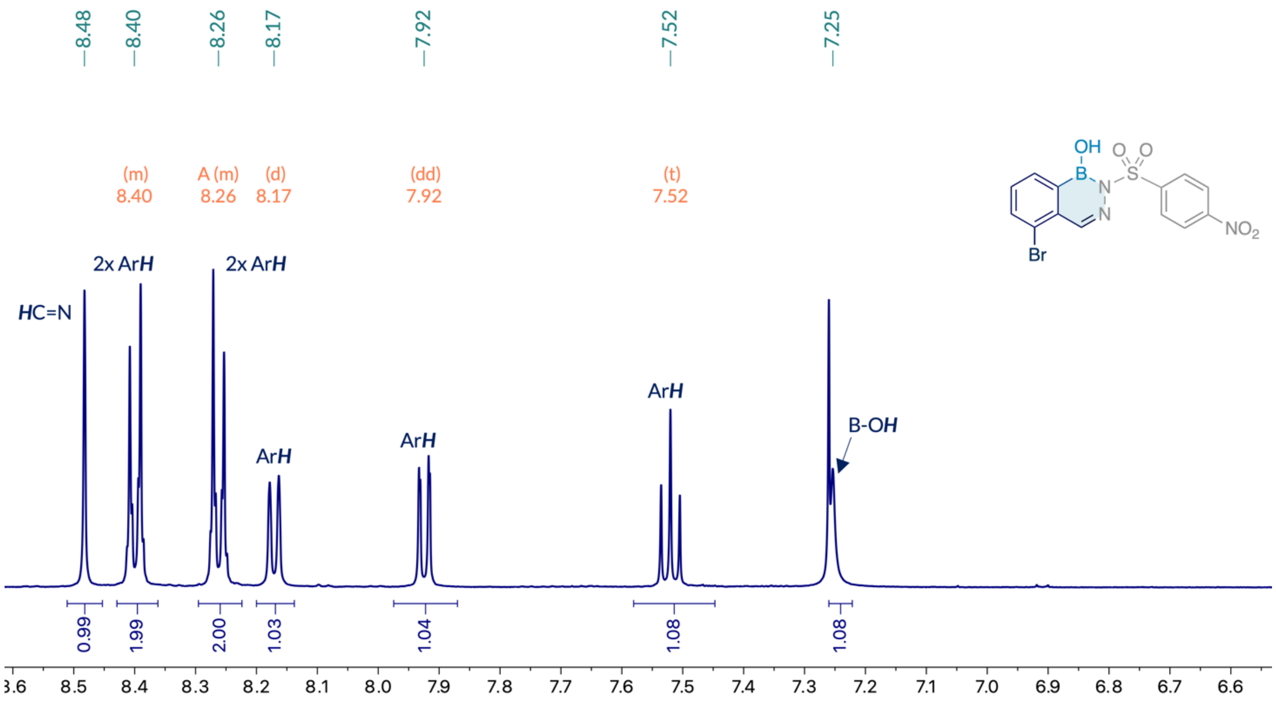

Figure S96. Diazaborine 18:^{1}H NMR (500 MHz, $CDCl_3$, 298 K)

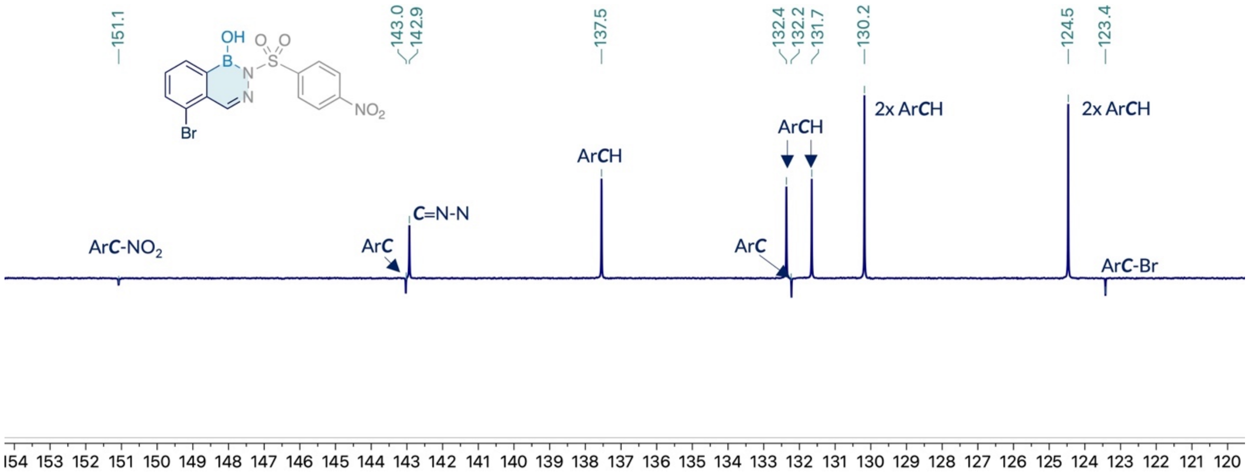

Figure S97. Diazaborine 18:^{13}C-APT NMR (126 MHz, $CDCl_3$, 298 K)

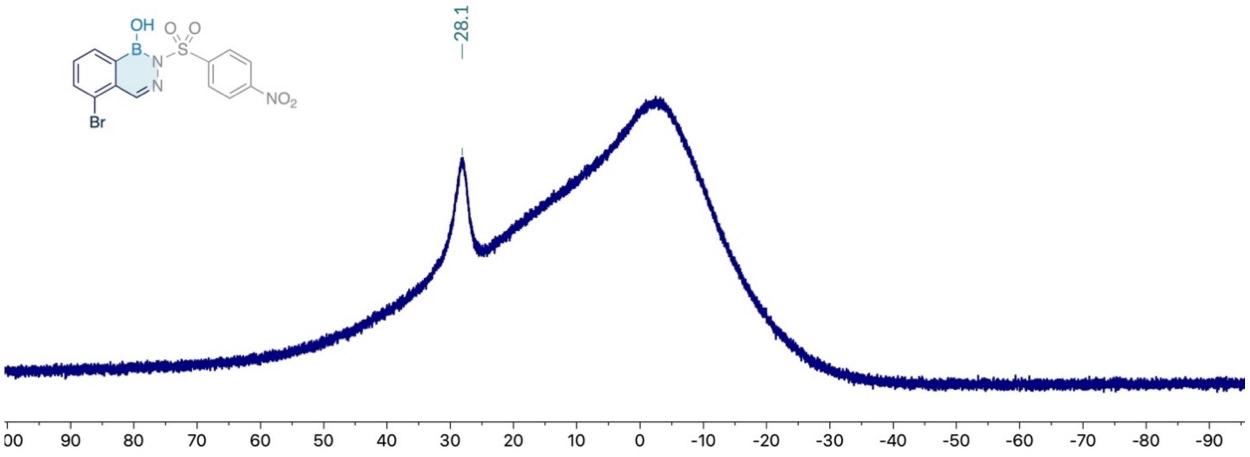

Figure S98. Diazaborine 18:^{11}B NMR (160 MHz, $CDCl_3$, 298 K)

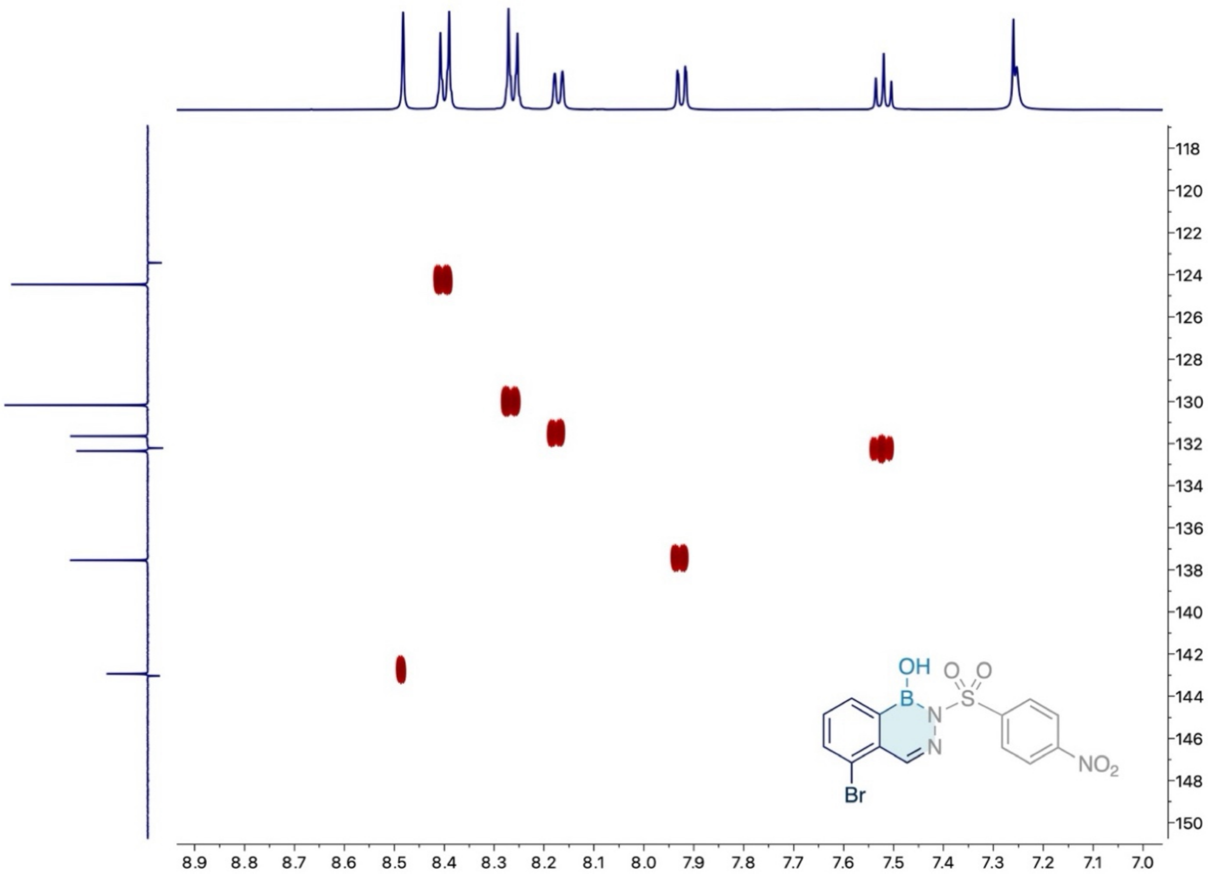

Figure S99. Diazaborine 18: ^{1}H-^{13}C gHSQC NMR ($CDCl_3$, 298 K)

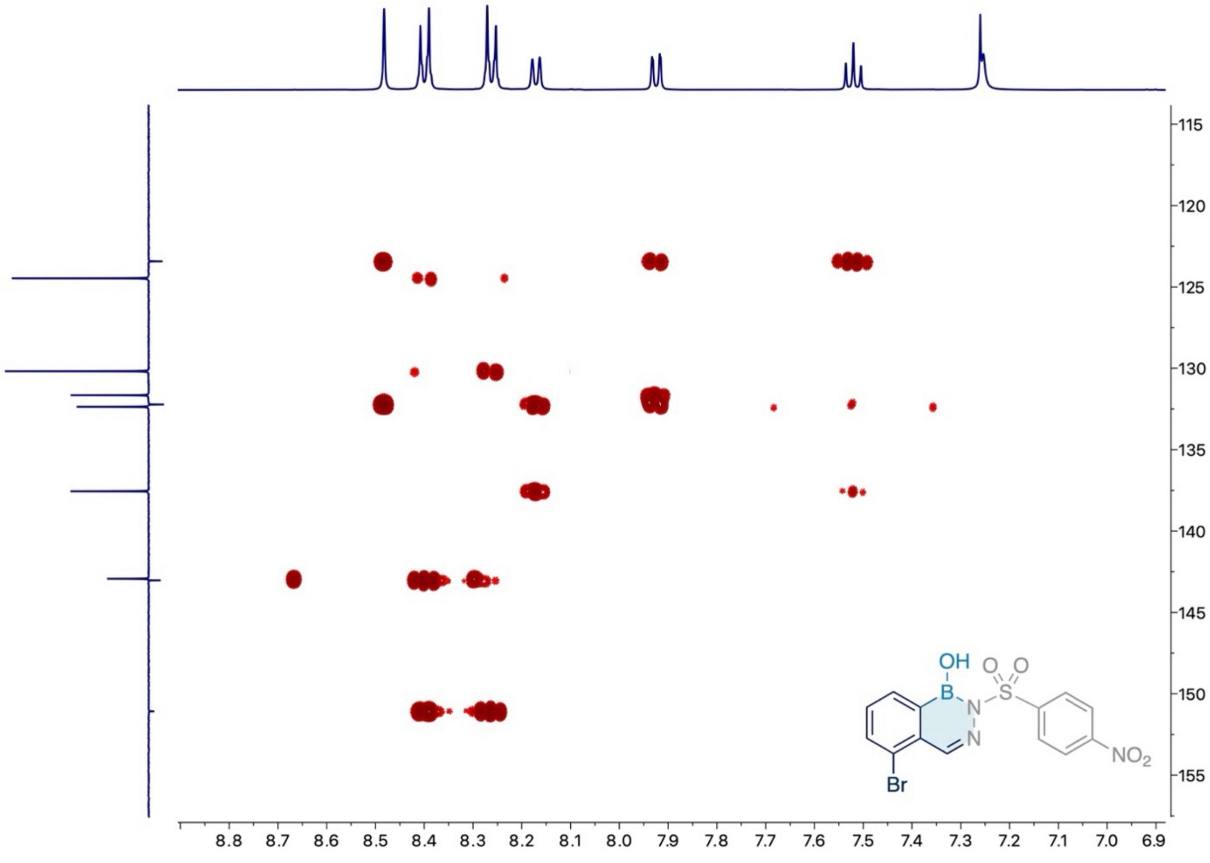

Figure S100. Diazaborine 18: ^{1}H-^{13}C gHMBC NMR ($CDCl_3$, 298 K)

Diazaborine 19

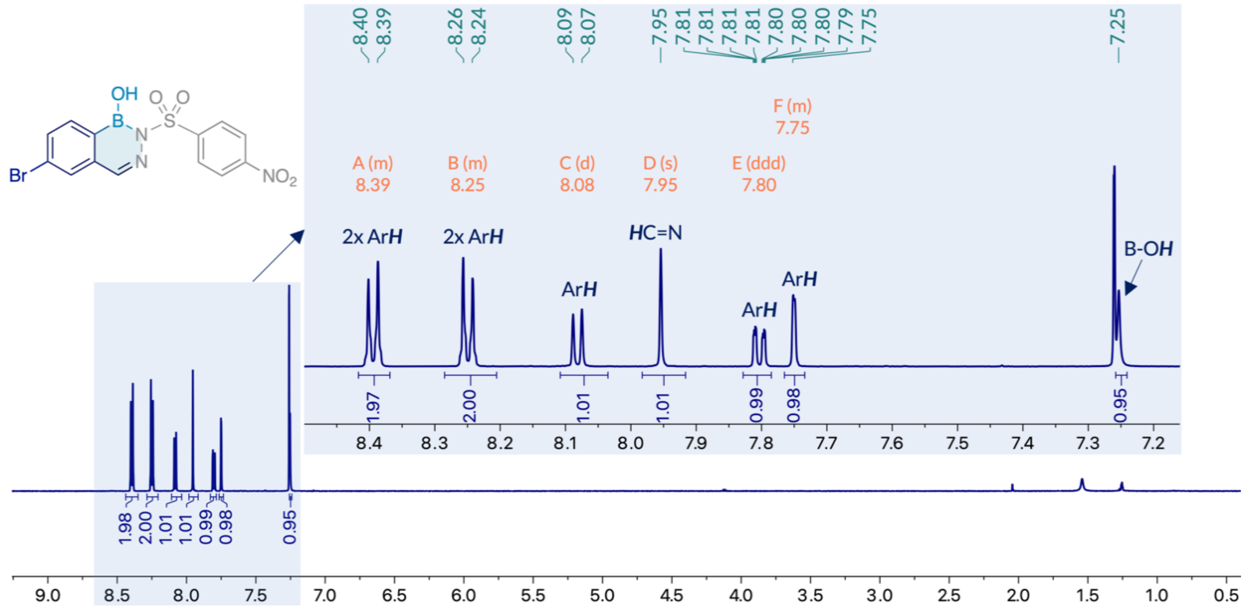

Figure S101. Diazaborine 19:^{1}H NMR (600 MHz, $CDCl_3$, 298 K)

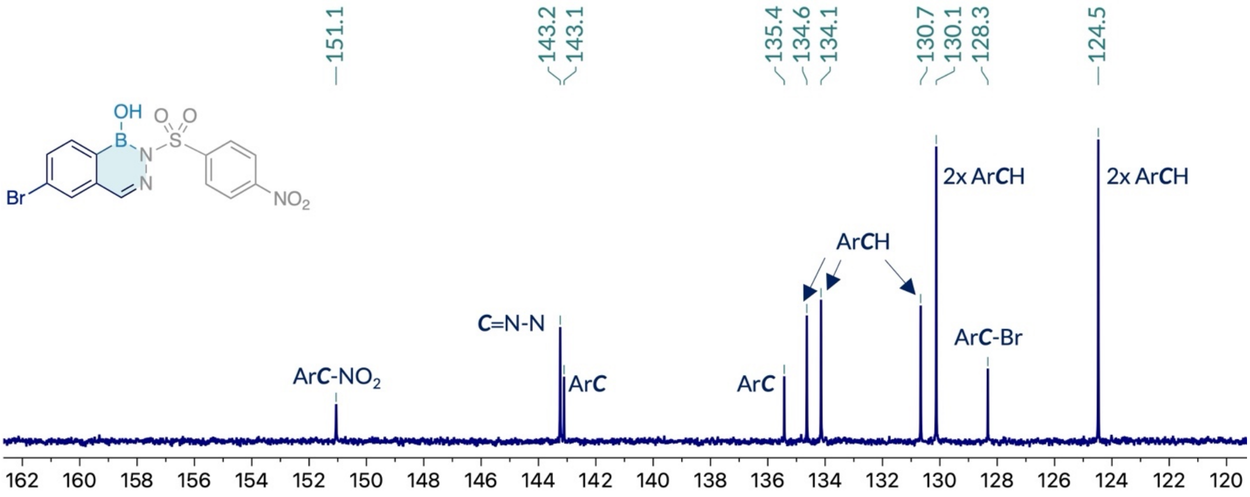

Figure S102. Diazaborine 19:^{13}C NMR (151 MHz, $CDCl_3$, 298 K)

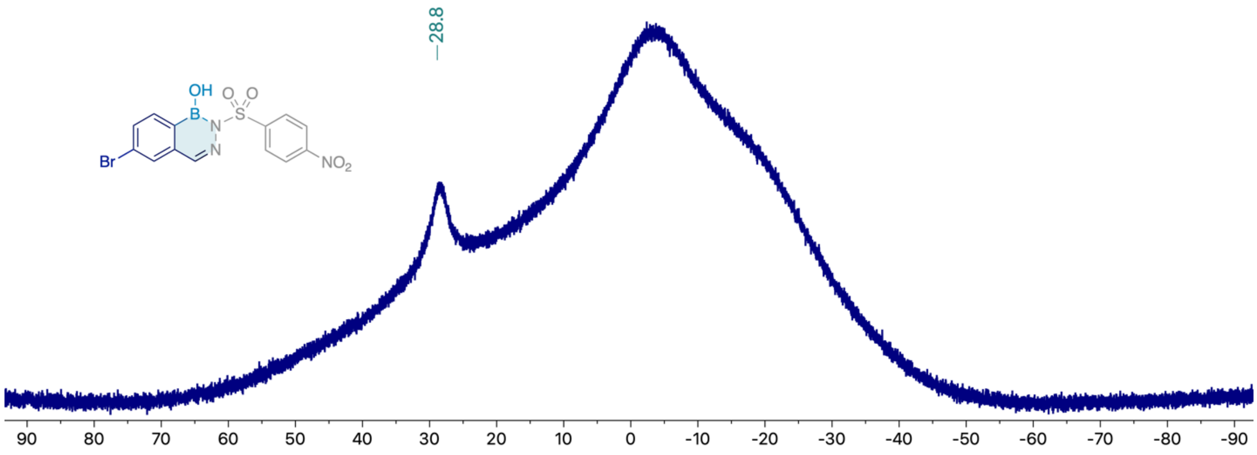

Figure S103. Diazaborine 19:^{11}B NMR (128 MHz, $CDCl_3$, 298 K)

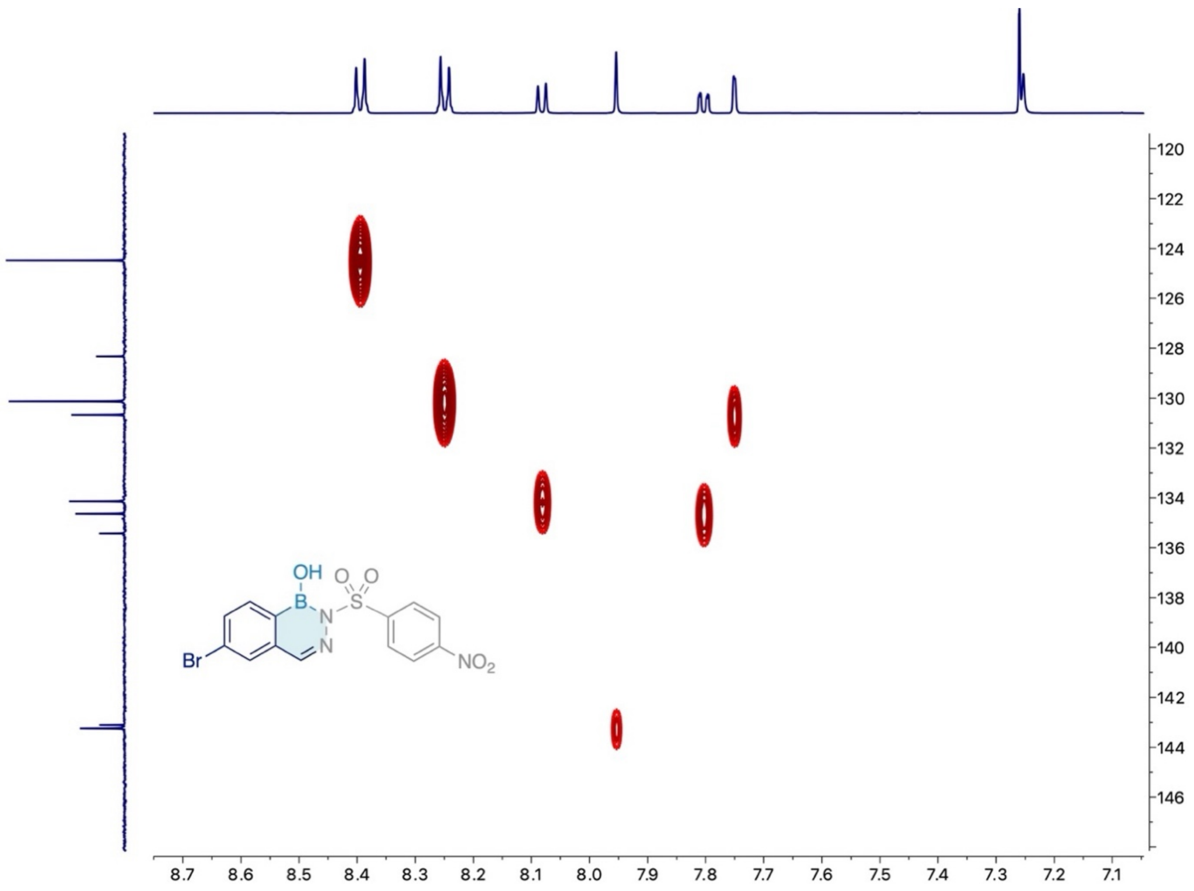

Figure S104. Diazaborine 19:^{1}H-^{13}C gHSQC NMR ($CDCl_3$, 298 K)

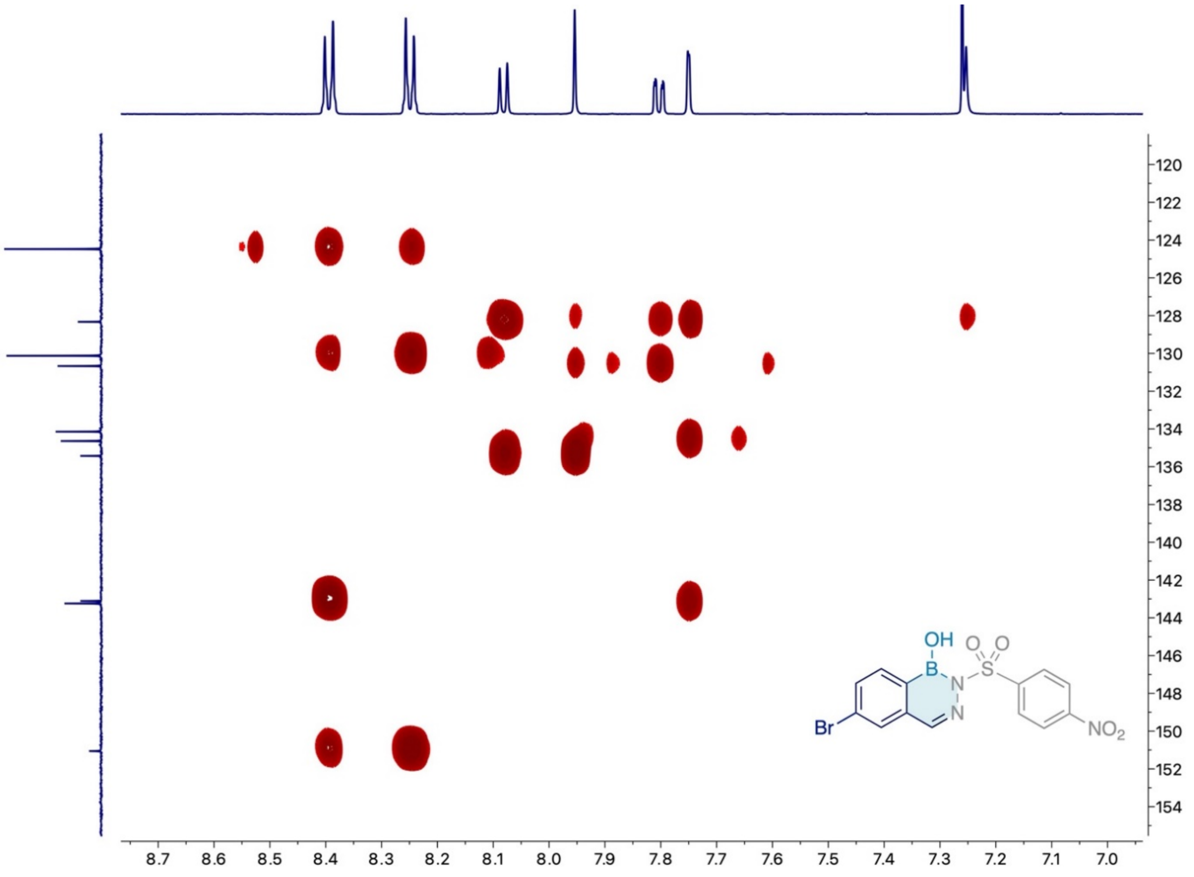

Figure S105. Diazaborine 19:^{1}H-^{13}C gHMBC NMR ($CDCl_3$, 298 K)

Diazaborine 20

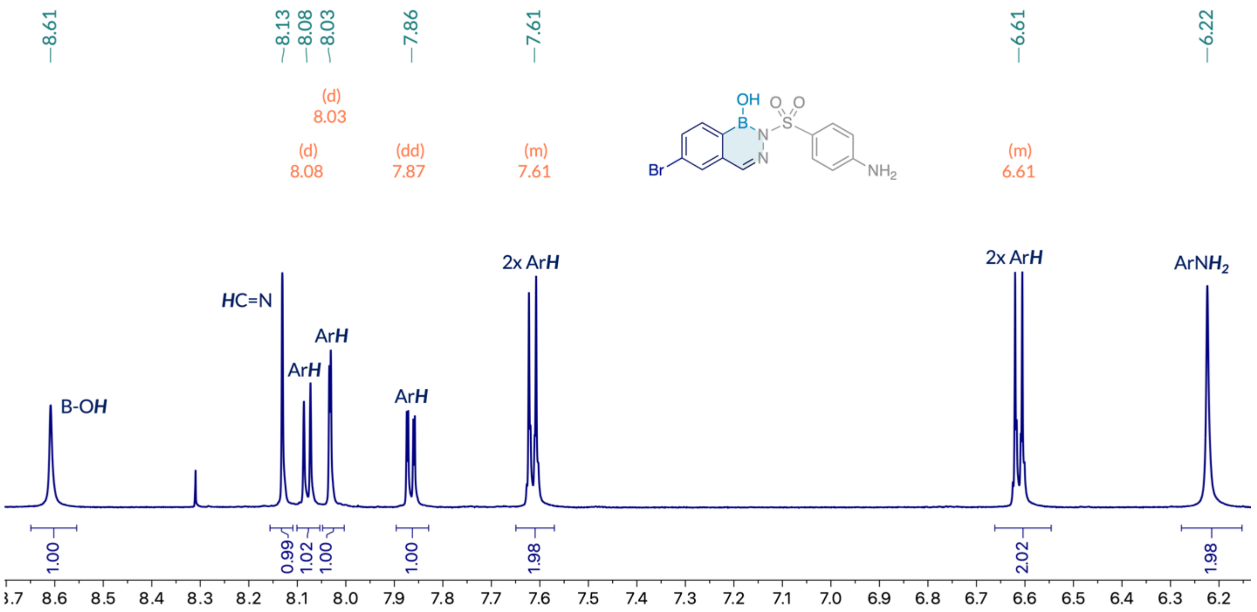

Figure S106. Diazaborine 20:^{1}H NMR (600 MHz, DMSO-d_6, 298 K)

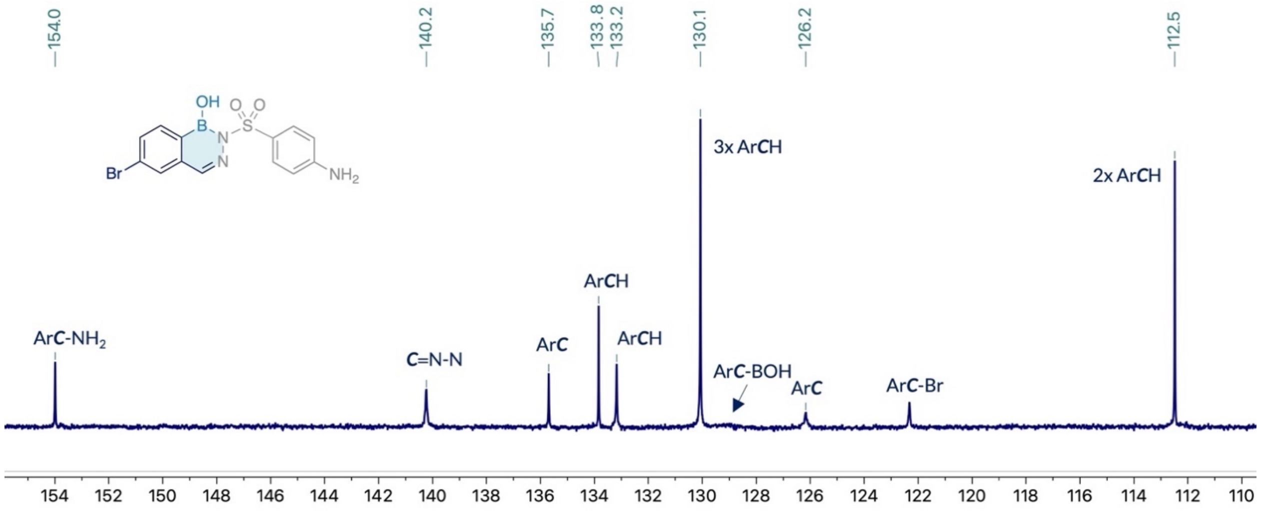

Figure S107. Diazaborine 20:^{13}C NMR (151 MHz, DMSO-d_6, 298 K)

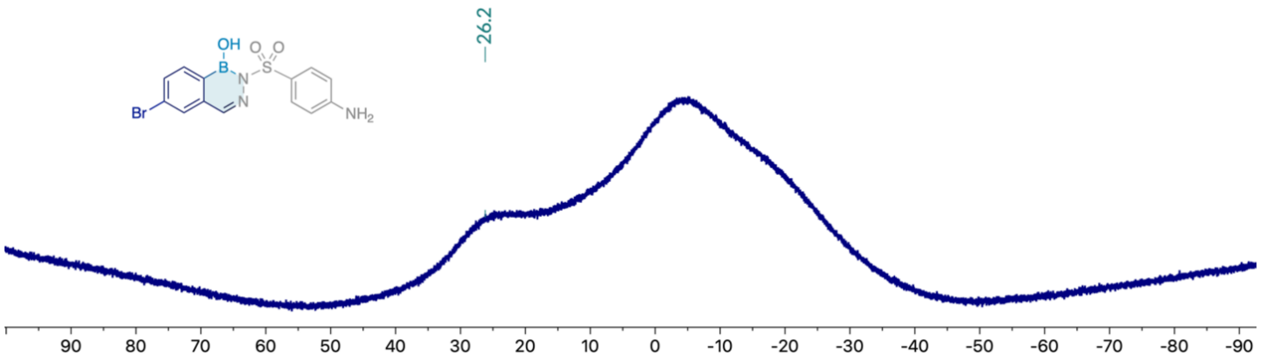

Figure S108. Diazaborine 20:^{11}B NMR (128 MHz, DMSO-d_6, 298 K)

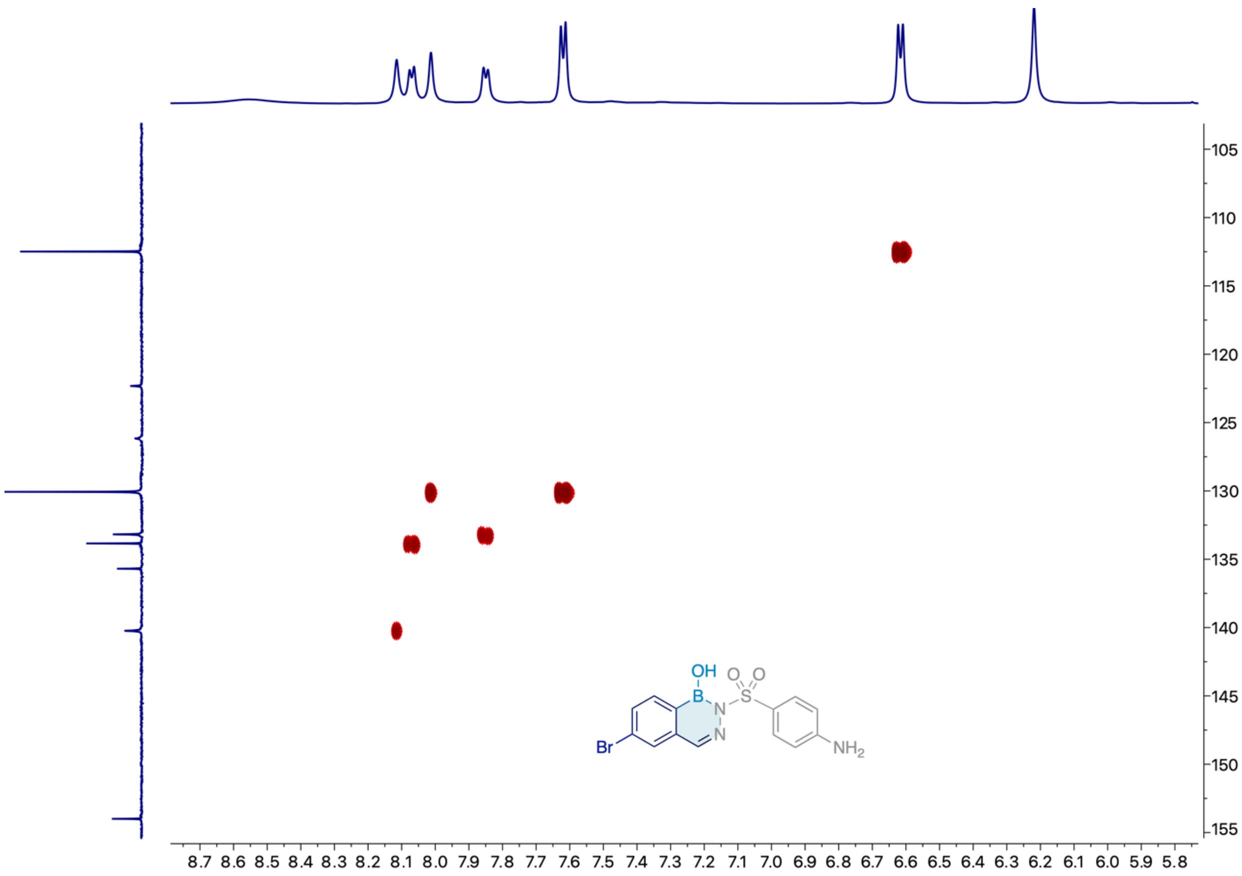

Figure S109. Diazaborine 20:^{1}H-^{13}C gHSQC NMR (DMSO-d_6, 298 K)

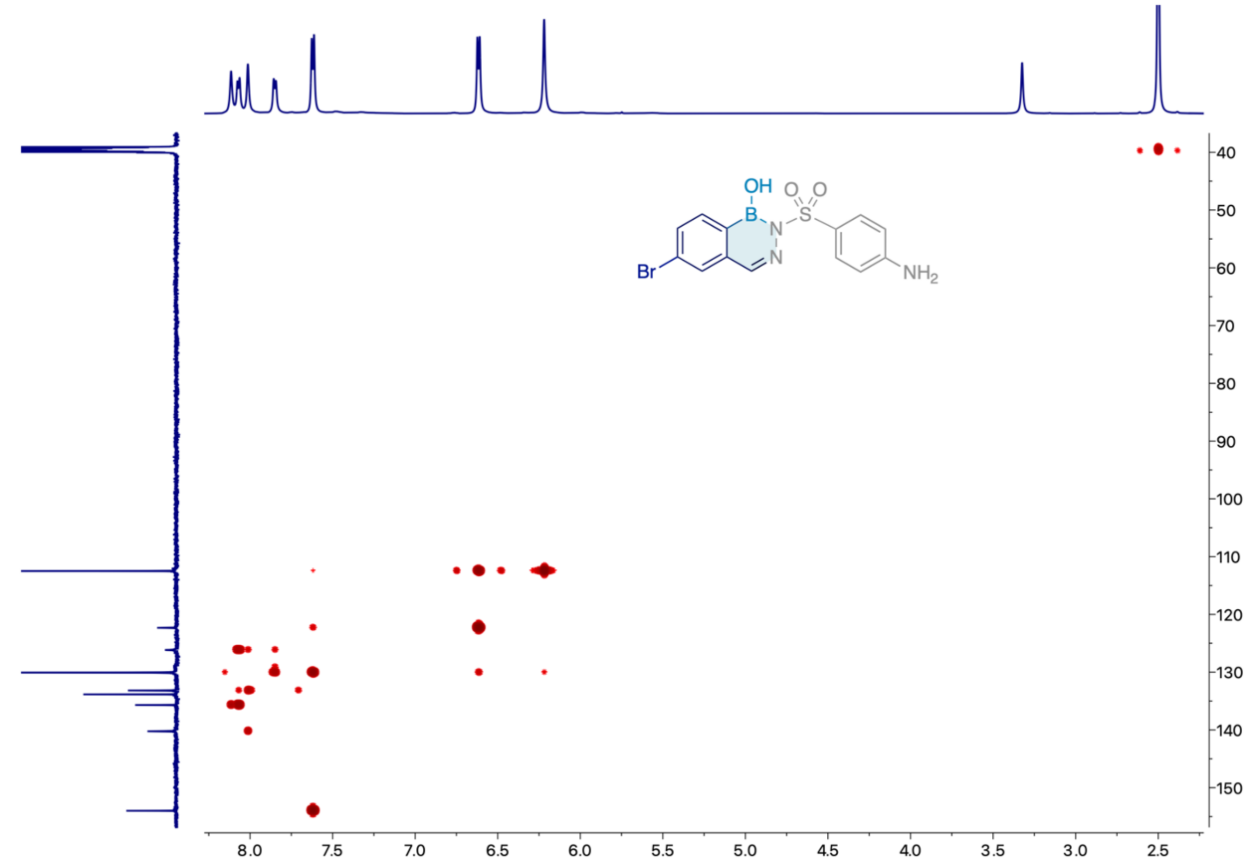

Figure S110. Diazaborine 20:^{1}H-^{13}C gHMBC NMR (DMSO-d_6, 298 K)

Diazaborine 21

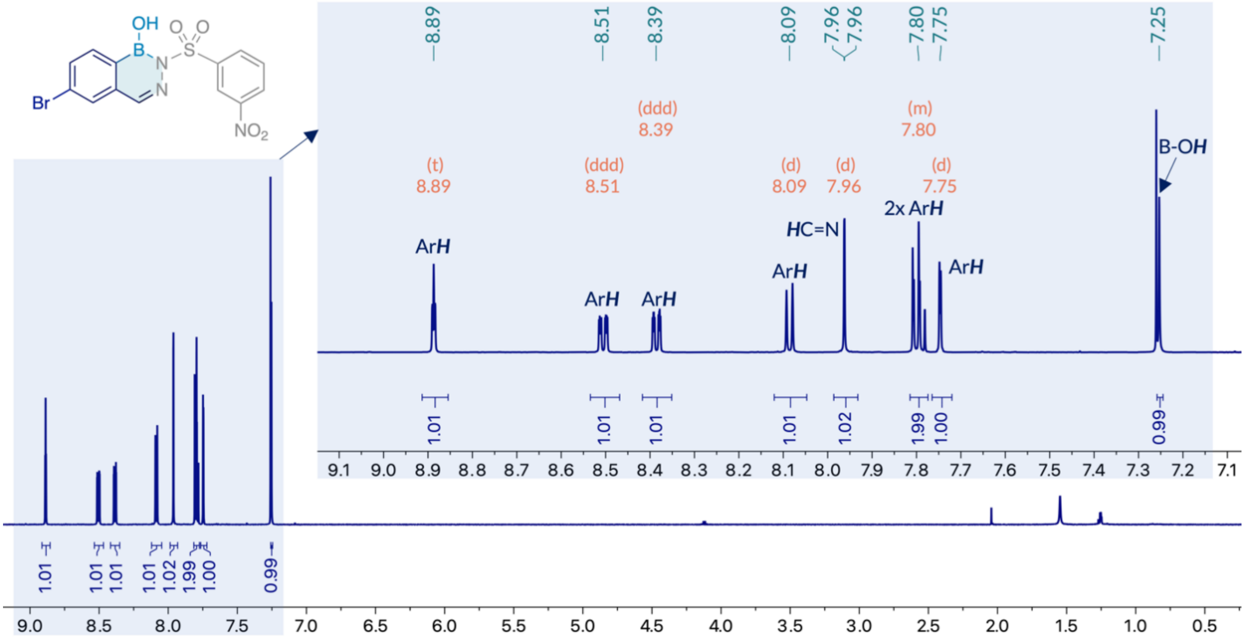

Figure S111. Diazaborine 21:^{1}H NMR (600 MHz, $CDCl_3$, 298 K)

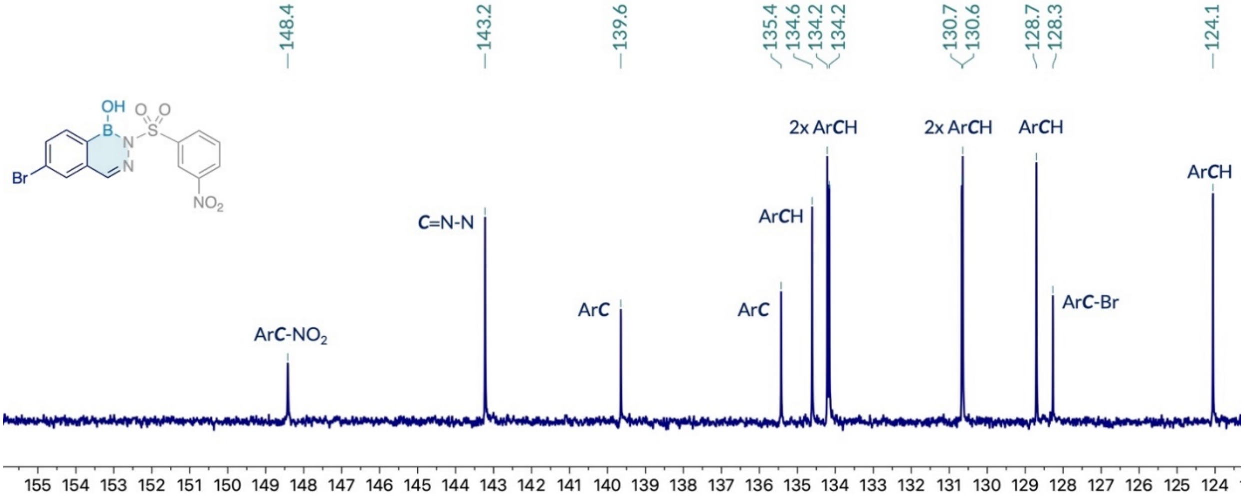

Figure S112. Diazaborine 21:^{13}C NMR (151 MHz, $CDCl_3$, 298 K)

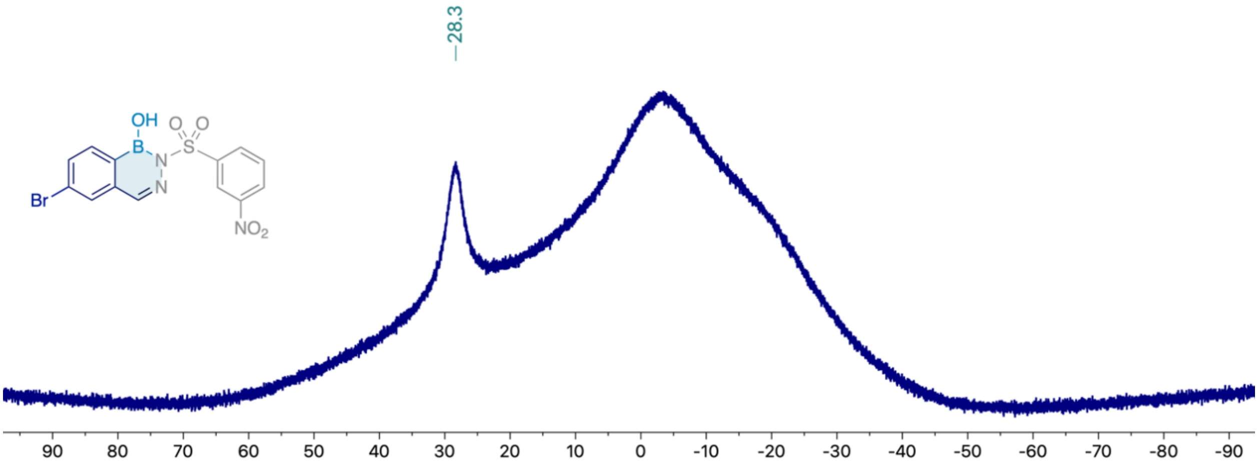

Figure S113. Diazaborine 21:^{11}B NMR (128 MHz, $CDCl_3$, 298 K)

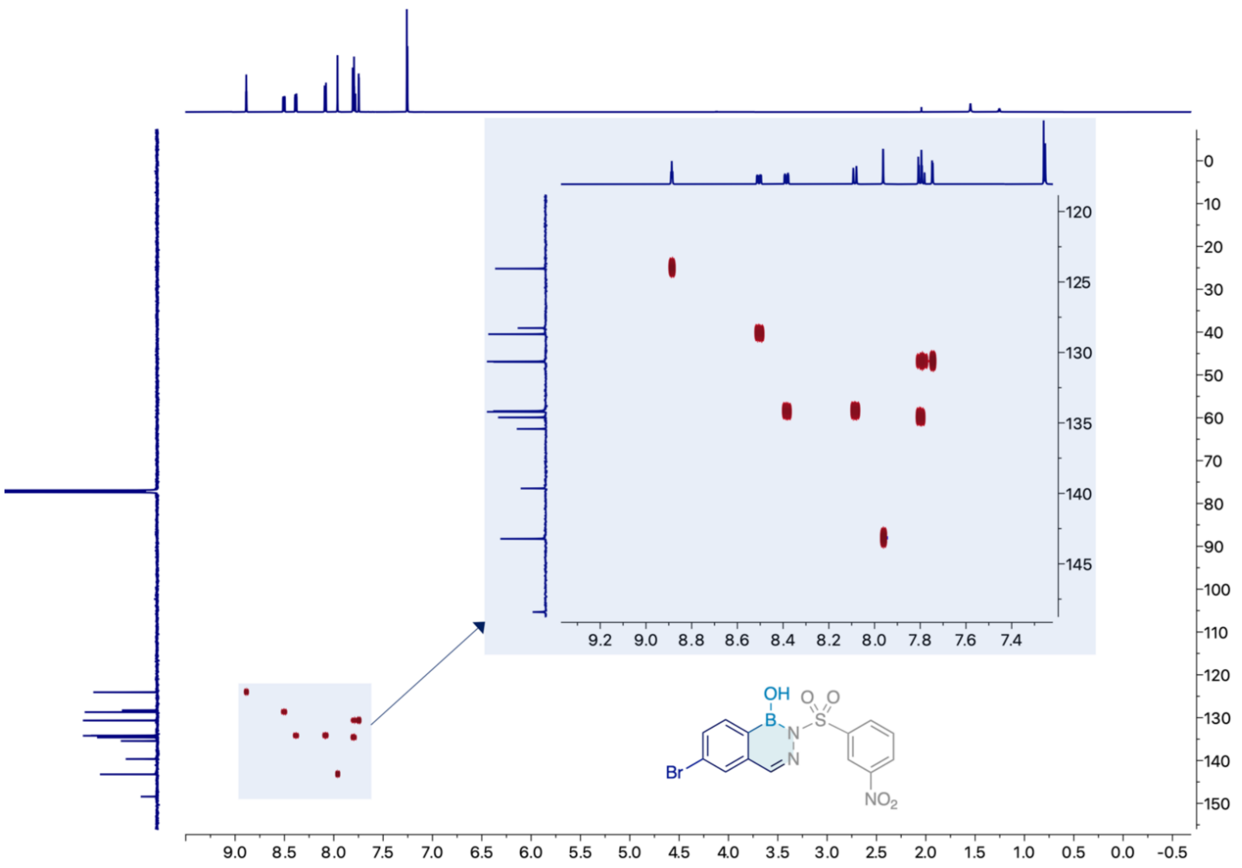

Figure S114. Diazaborine 21:^{1}H-^{13}C gHSQC NMR ($CDCl_3$, 298 K)

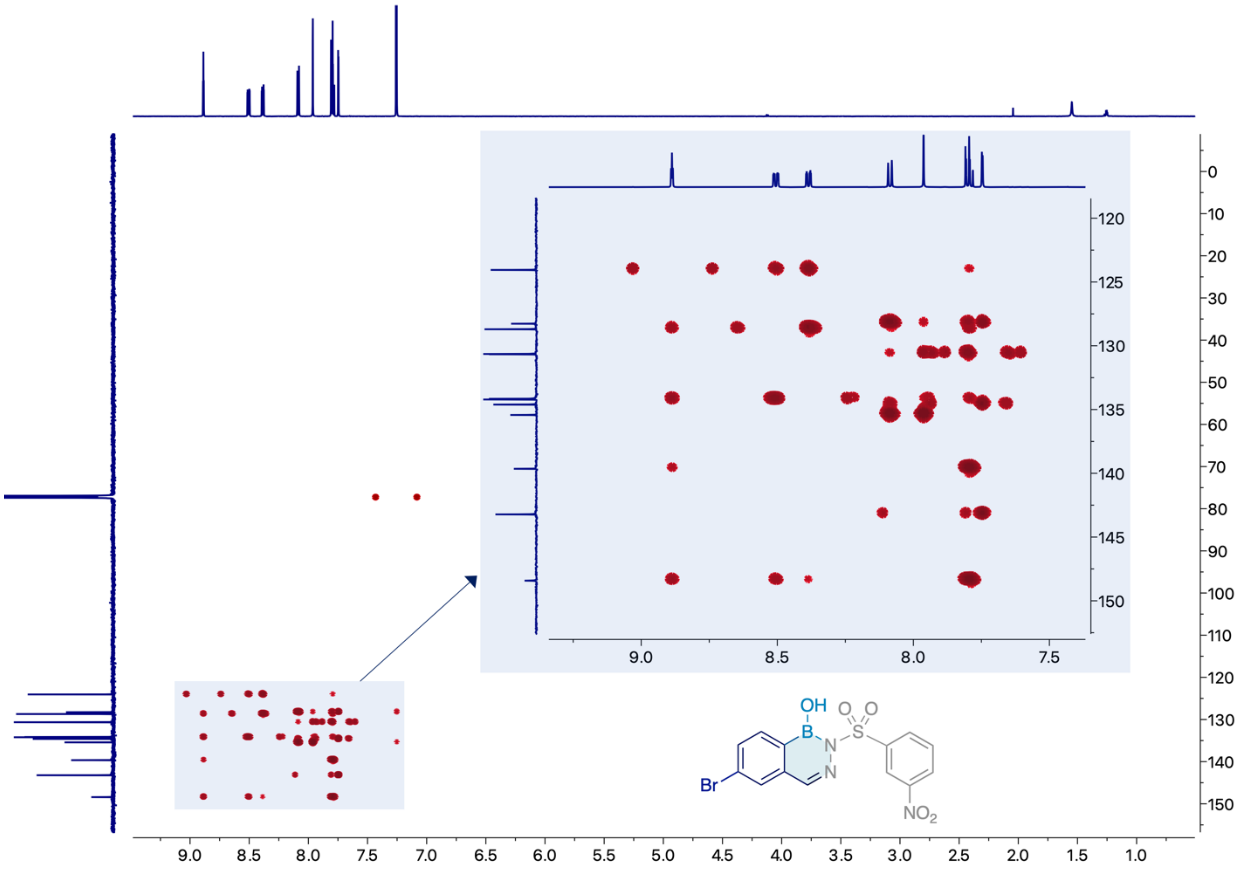

Figure S115. Diazaborine 21:^{1}H-^{13}C gHMBC NMR ($CDCl_3$, 298 K)

Diazaborine 22

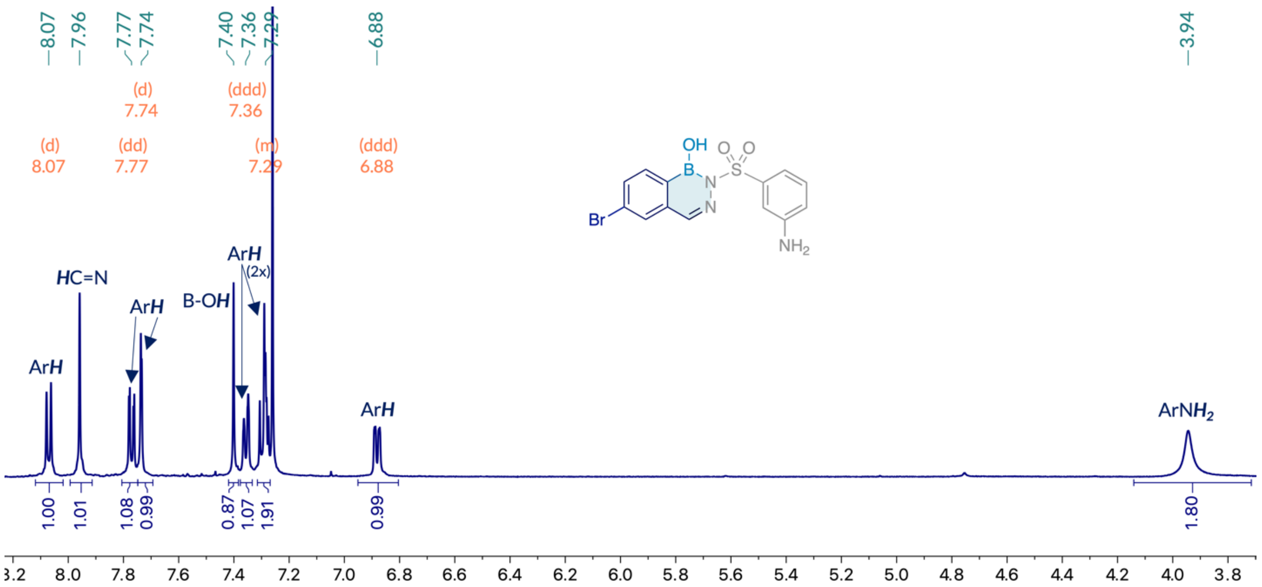

Figure S116. Diazaborine 22:^{1}H NMR (500 MHz, $CDCl_3$, 298 K)

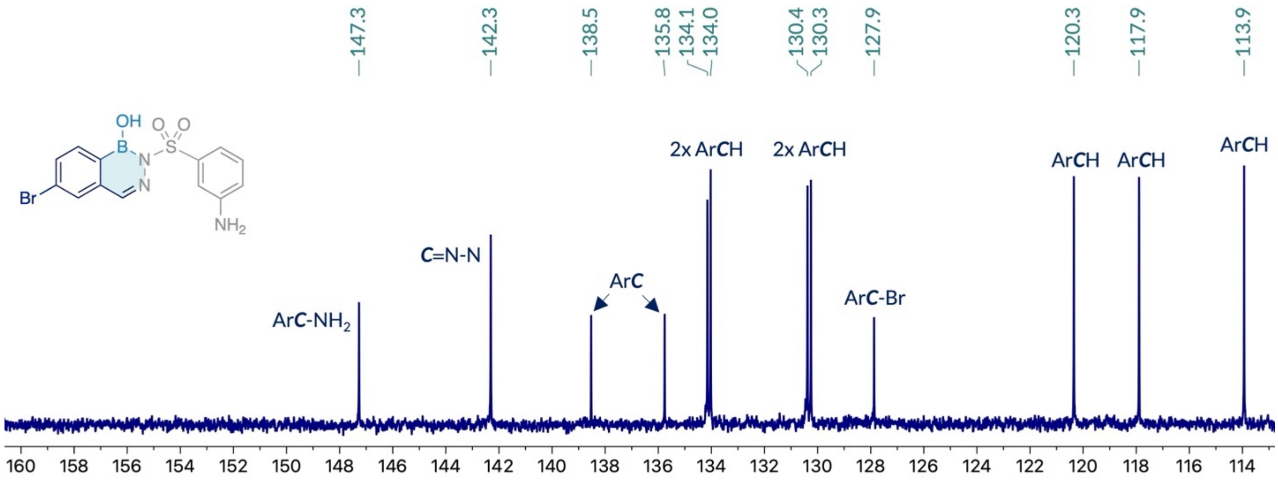

Figure S117. Diazaborine 22:^{13}C NMR (126 MHz, $CDCl_3$, 298 K)

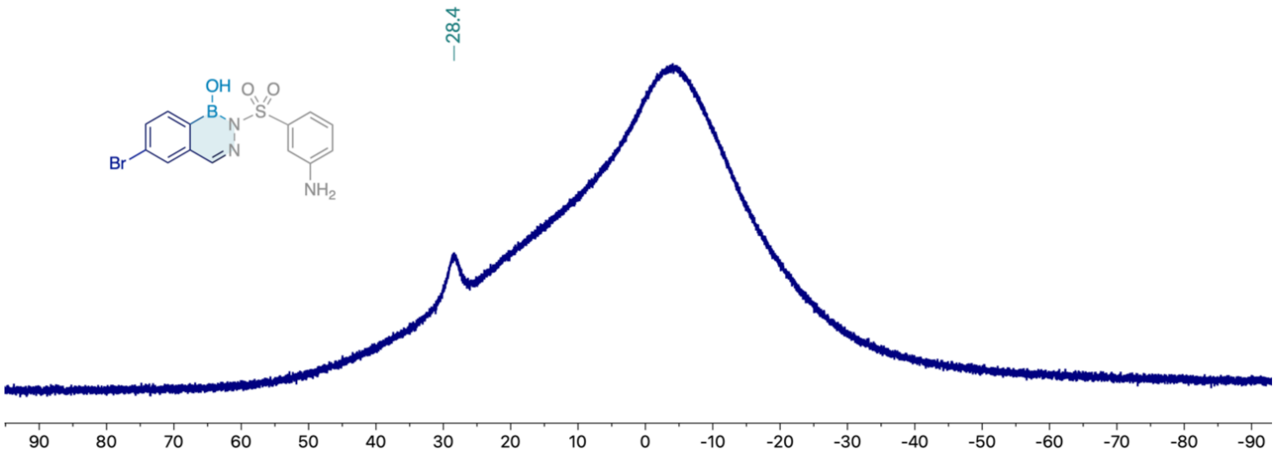

Figure S118. Diazaborine 22:^{11}B NMR (160 MHz, $CDCl_3$, 298 K)

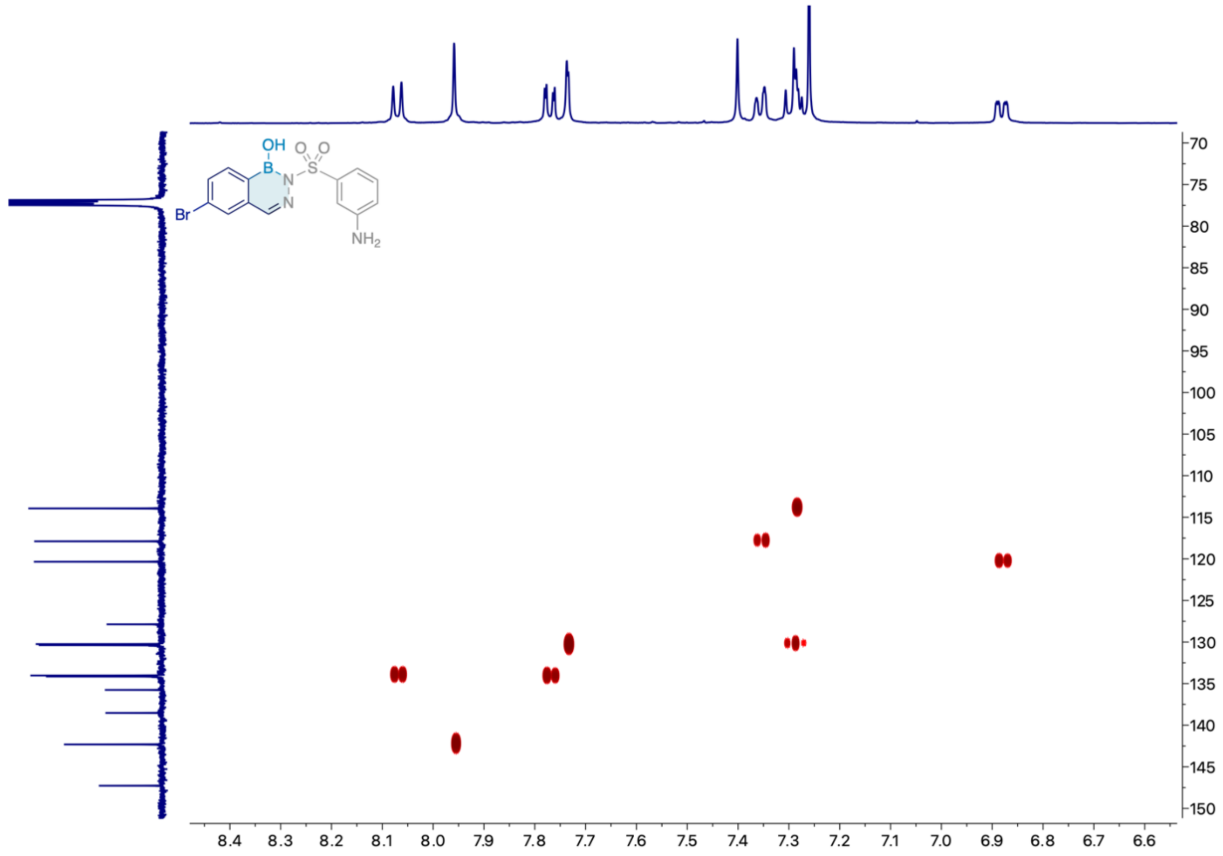

Figure S119. Diazaborine 22:^{1}H-^{13}C gHSQC NMR ($CDCl_3$, 298 K)

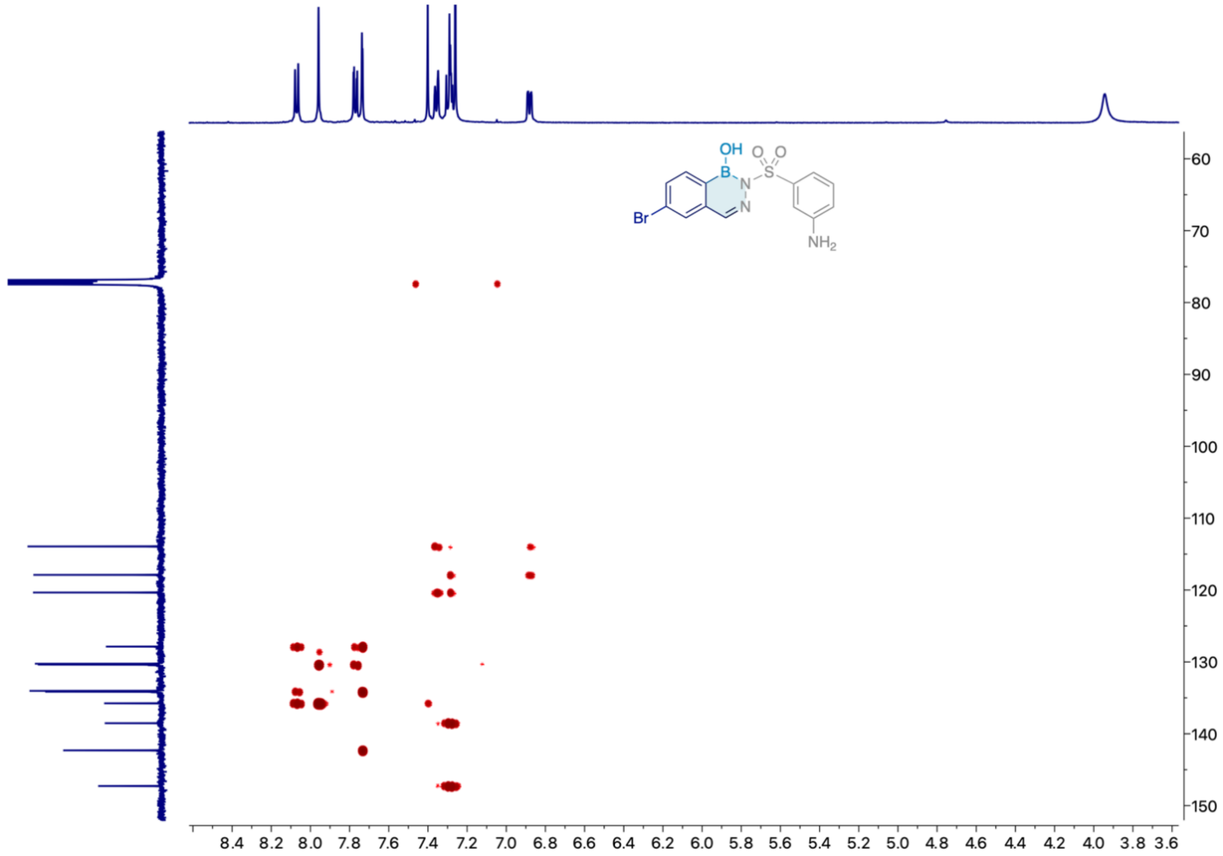

Figure S120. Diazaborine 22:^{1}H-^{13}C gHMBC NMR ($CDCl_3$, 298 K)

Diazaborine 23

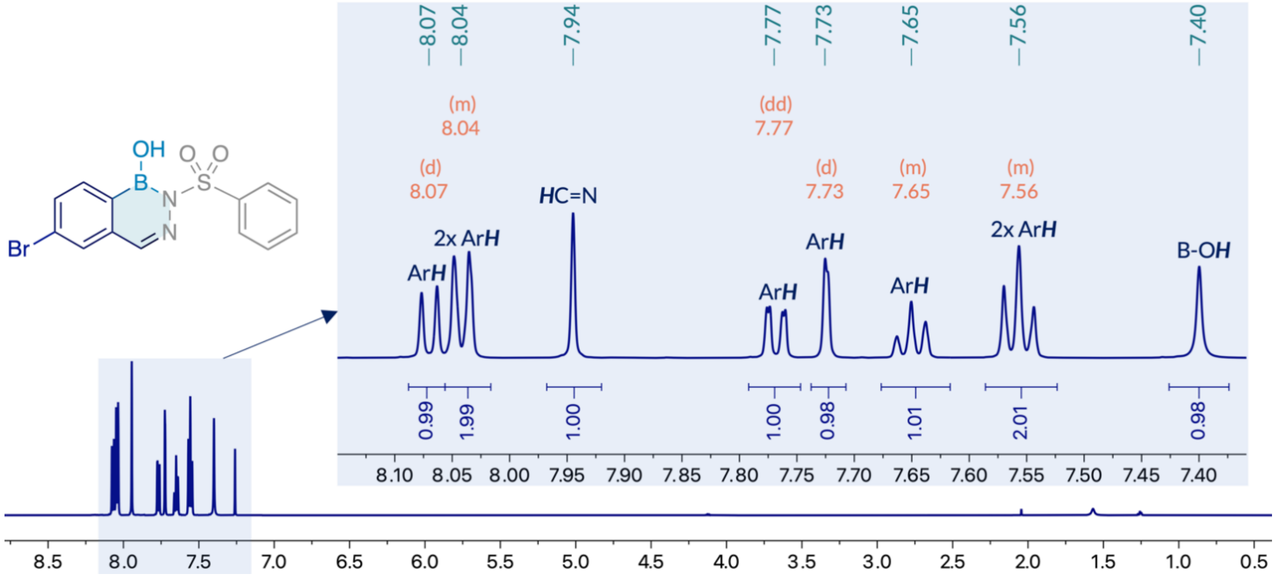

Figure S121. Diazaborine 23: ^{1}H NMR (600 MHz, $CDCl_3$, 298 K)

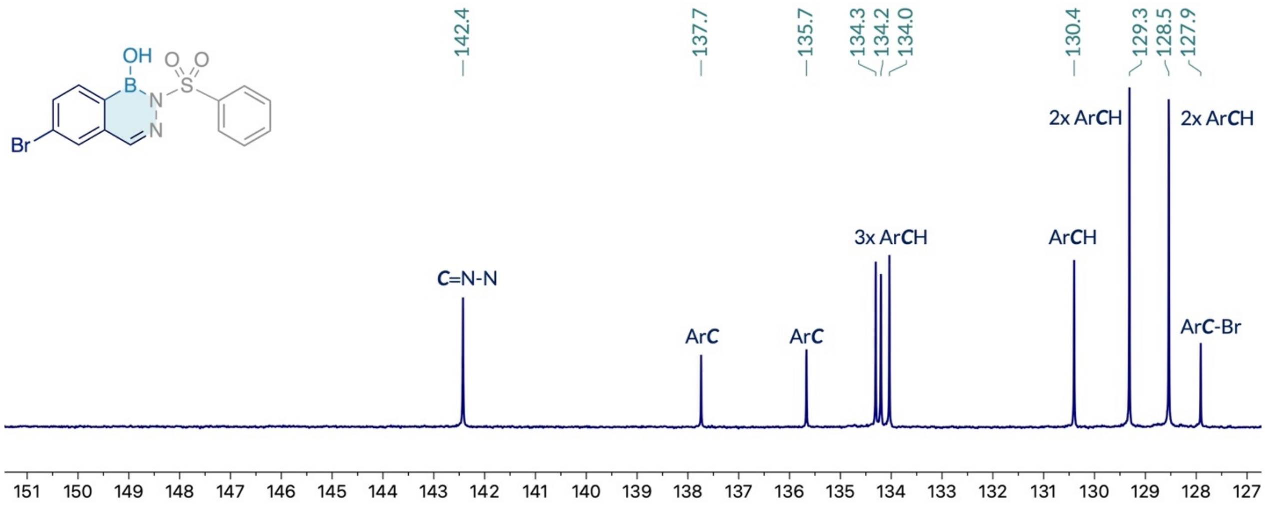

Figure S122. Diazaborine 23: ^{13}C NMR (151 MHz, $CDCl_3$, 298 K)

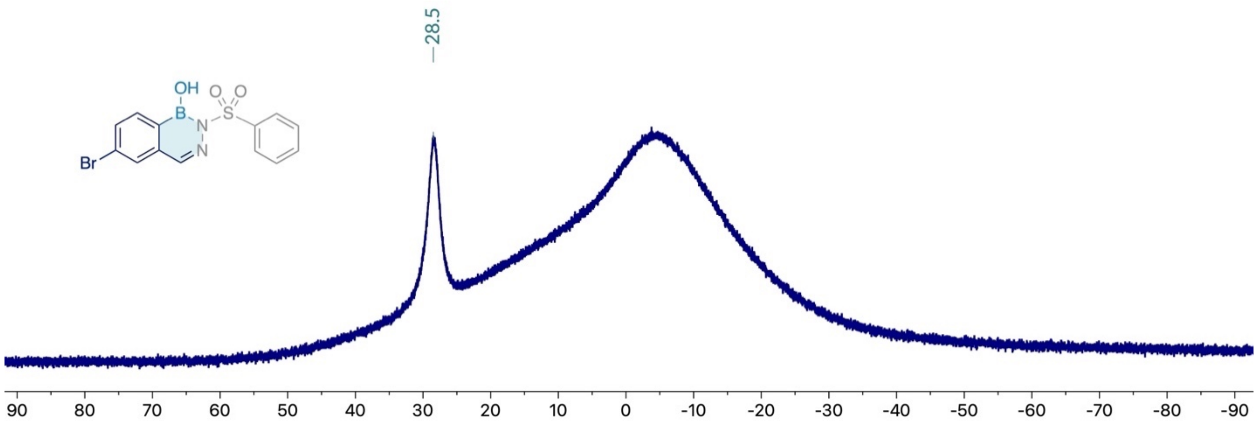

Figure S123. Diazaborine 23: ^{11}B NMR (160 MHz, $CDCl_3$, 298 K)

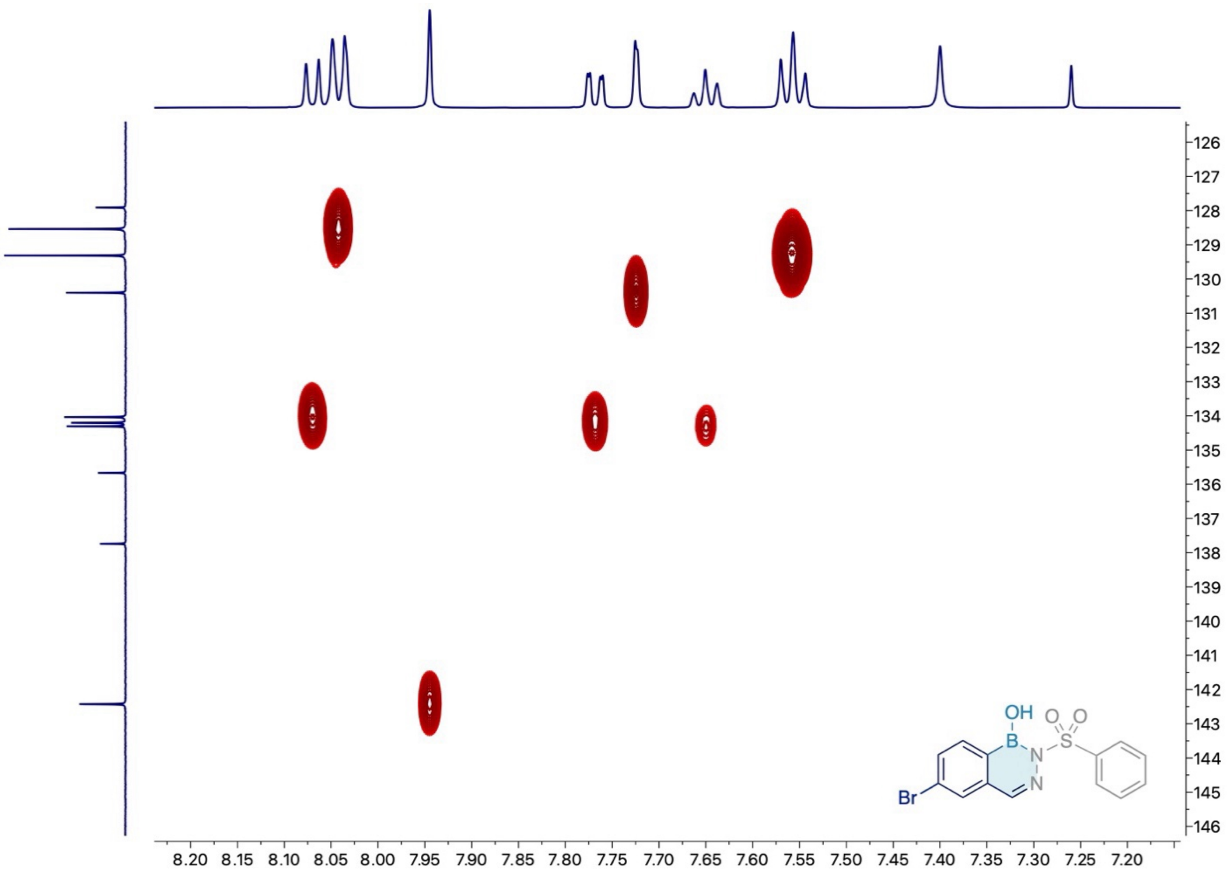

Figure S124. Diazaborine 23:^{1}H-^{13}C gHSQC NMR ($CDCl_3$, 298 K)

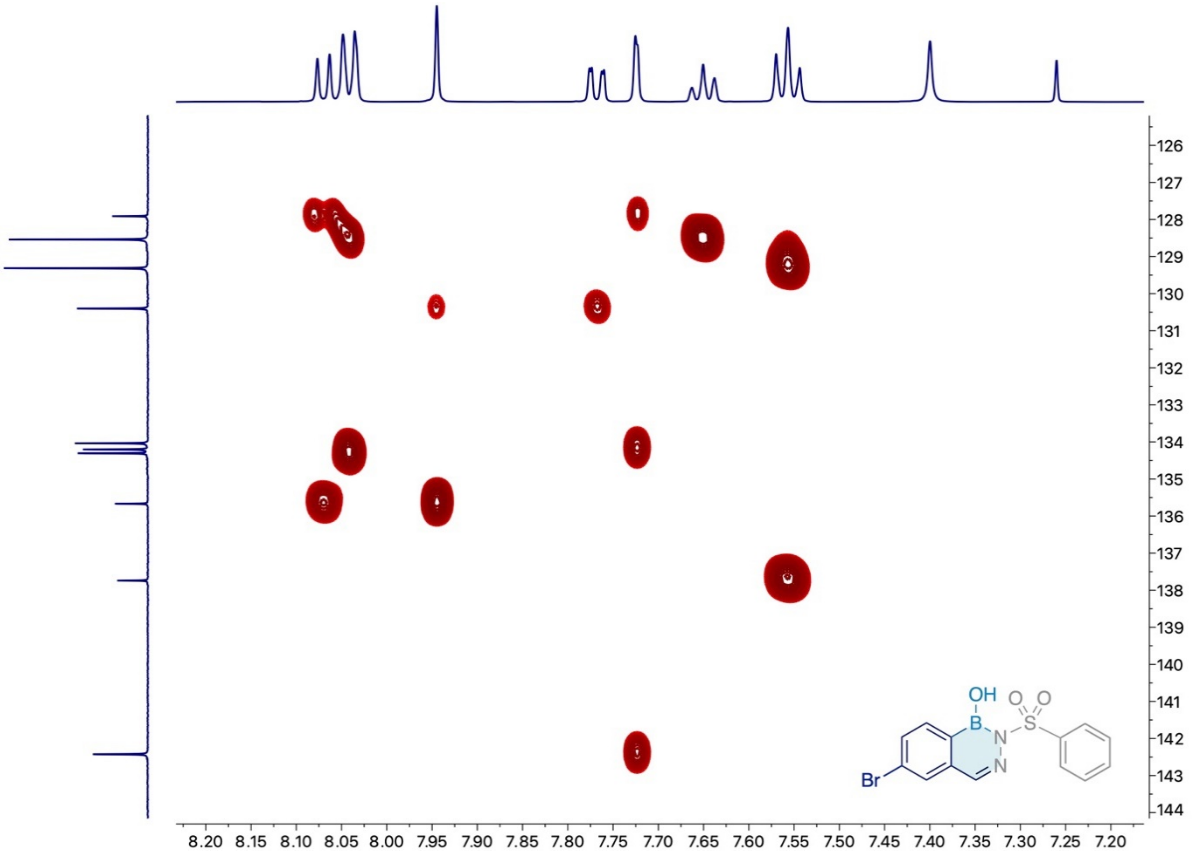

Figure S125. Diazaborine 23:^{1}H-^{13}C gHMBC NMR ($CDCl_3$, 298 K)

Diazaborine 24

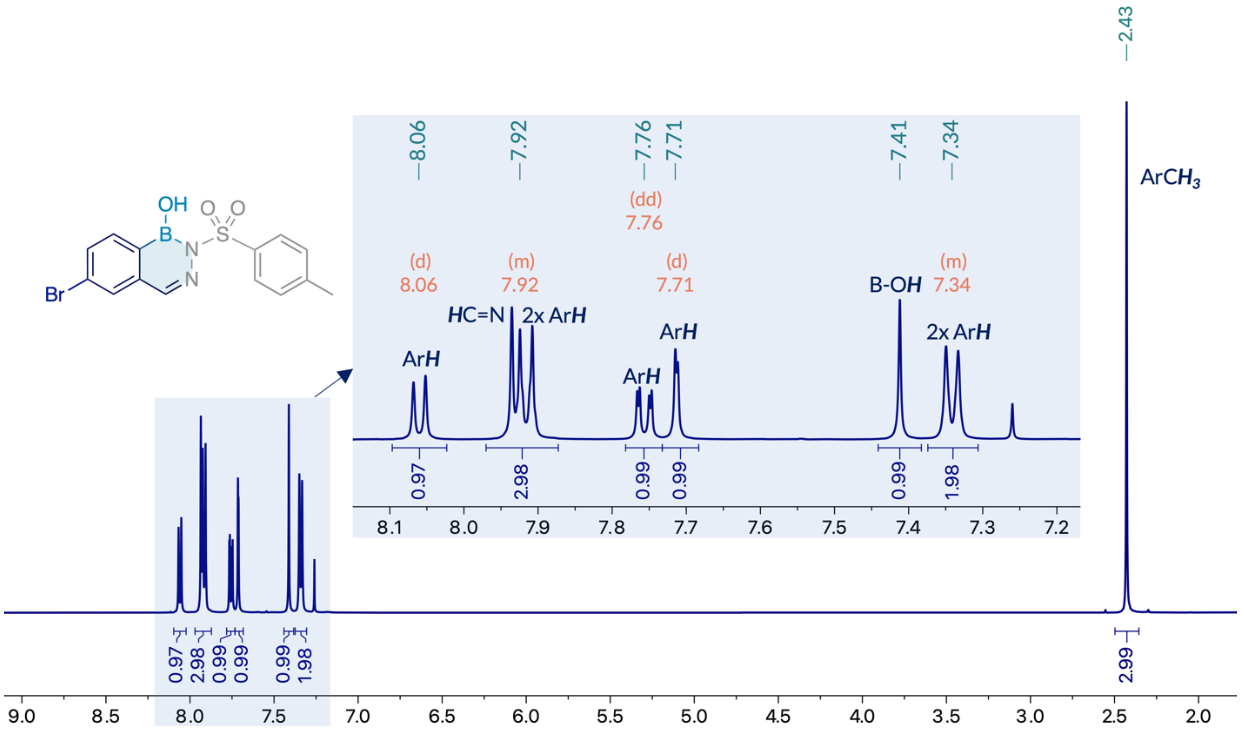

Figure S126. Diazaborine 24:^{1}H NMR (500 MHz, $CDCl_3$, 298 K)

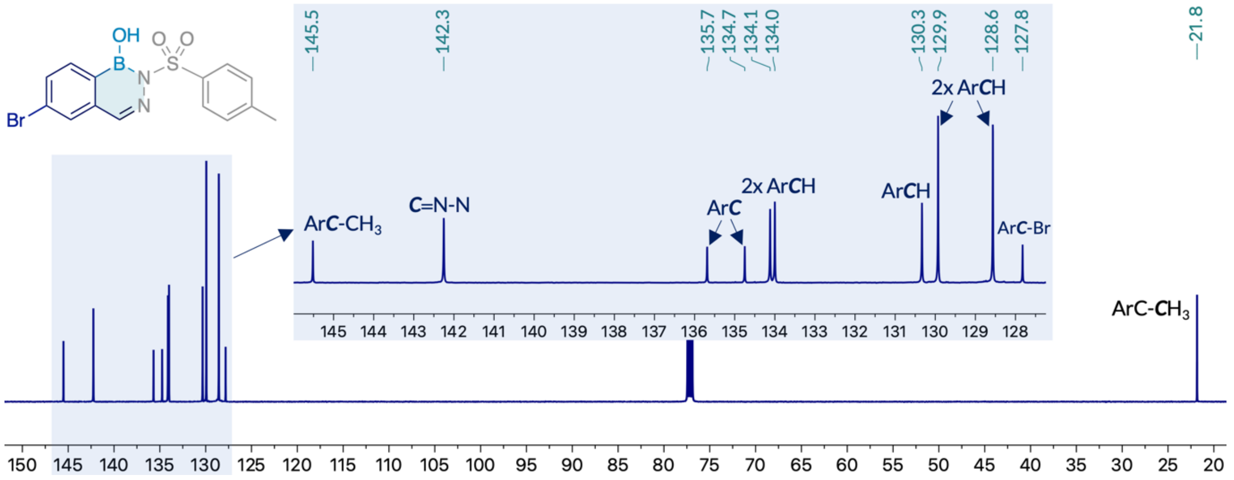

Figure S127. Diazaborine 24:^{13}C NMR (126 MHz, $CDCl_3$, 298 K)

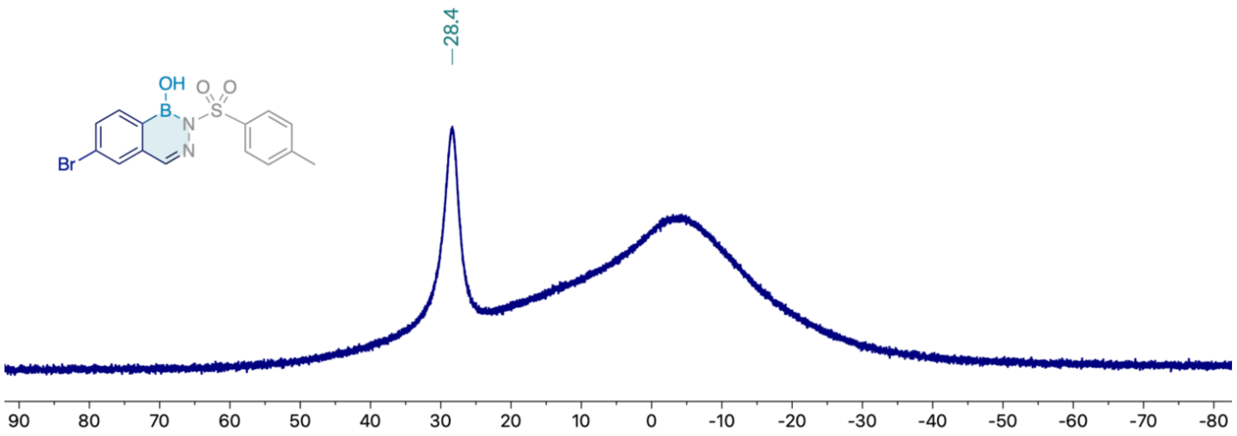

Figure S128. Diazaborine 24:^{11}B NMR (160 MHz, $CDCl_3$, 298 K)

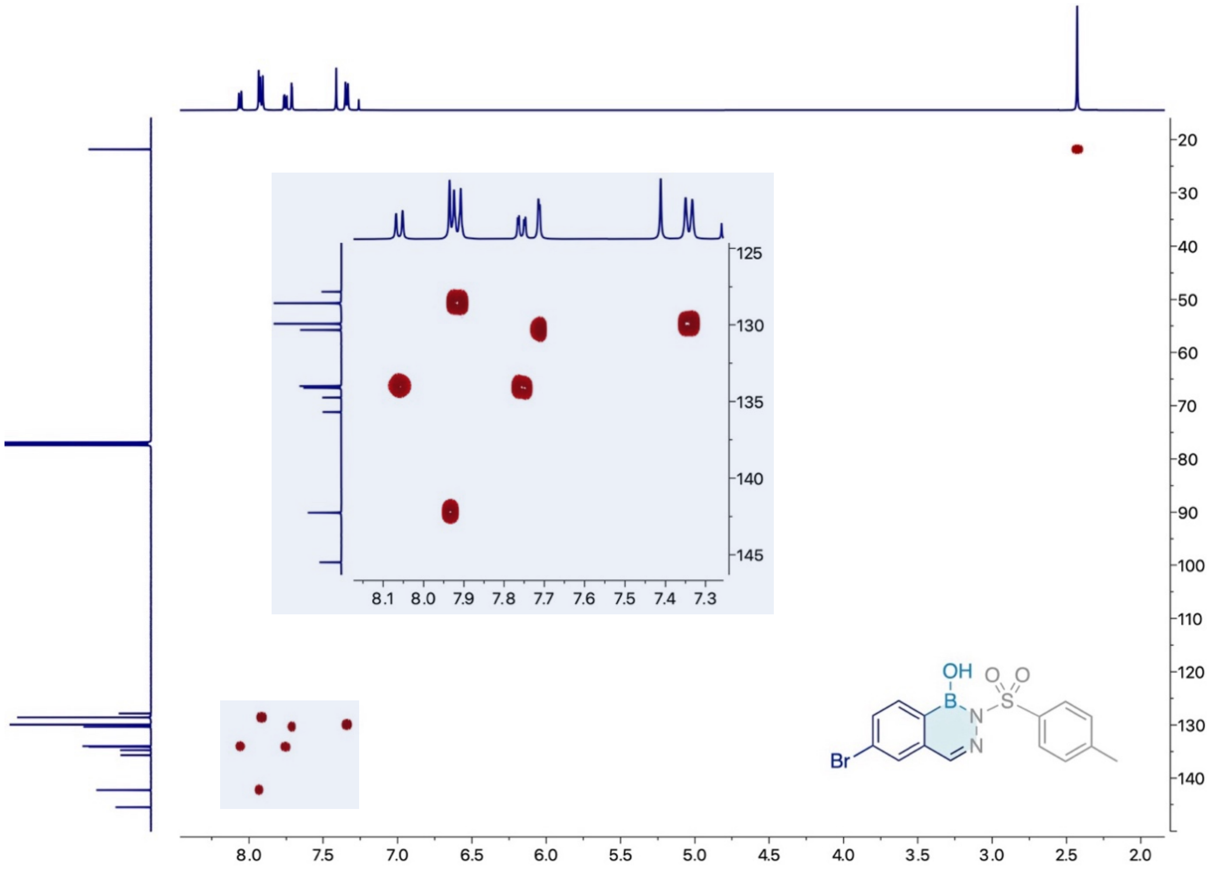

Figure S129. Diazaborine 24: ^{1}H-^{13}C gHSQC NMR ($CDCl_3$, 298 K)

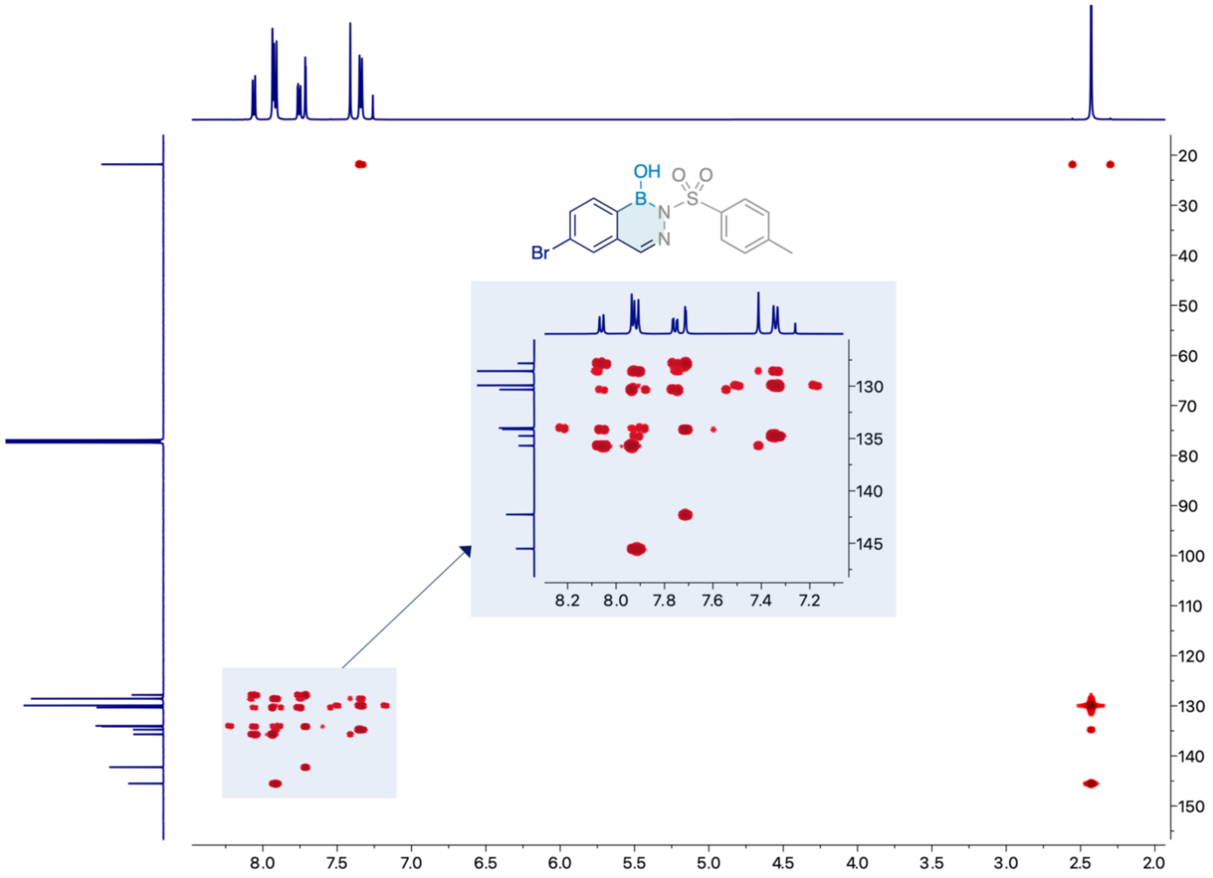

Figure S130. Diazaborine 24: ^{1}H-^{13}C gHMBC NMR ($CDCl_3$, 298 K)

Diazaborine 25

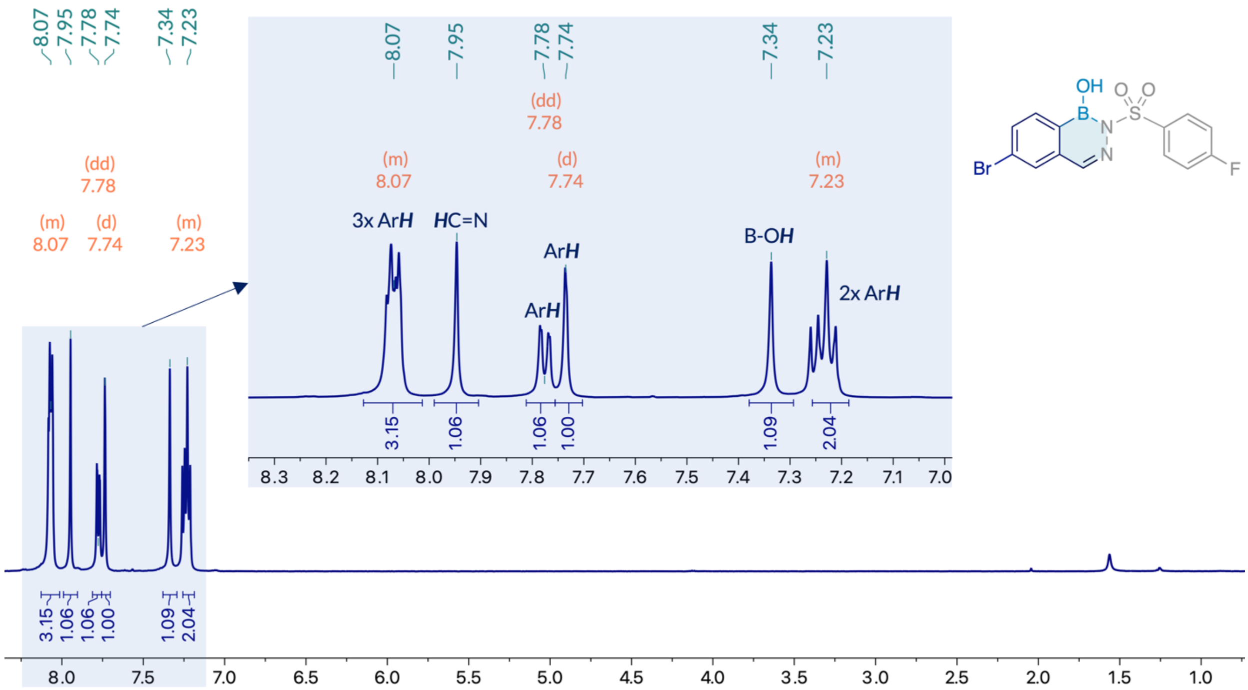

Figure S131. Diazaborine 25:^{1}H NMR (500 MHz, $CDCl_3$, 298 K)

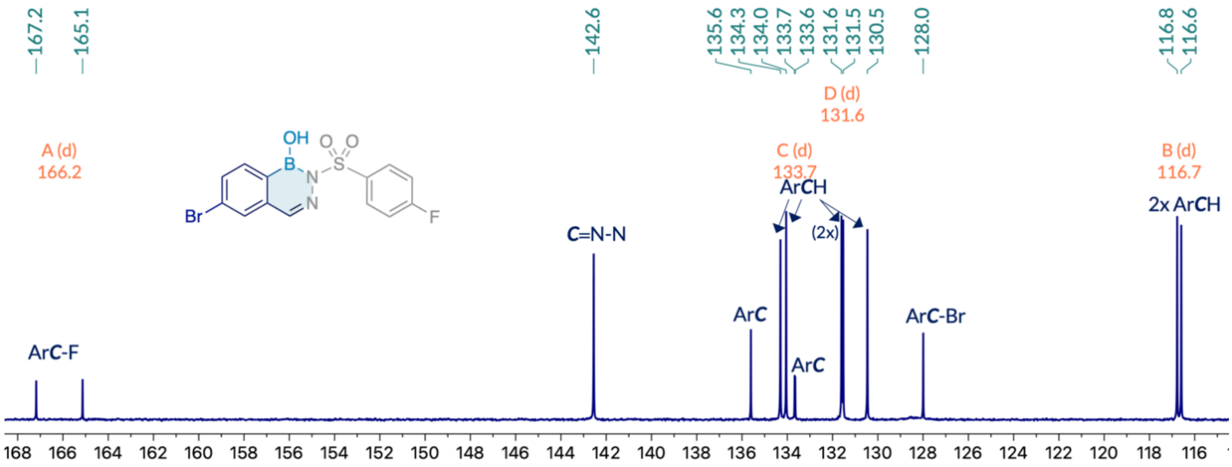

Figure S132. Diazaborine 25:^{13}C NMR (126 MHz, $CDCl_3$, 298 K)

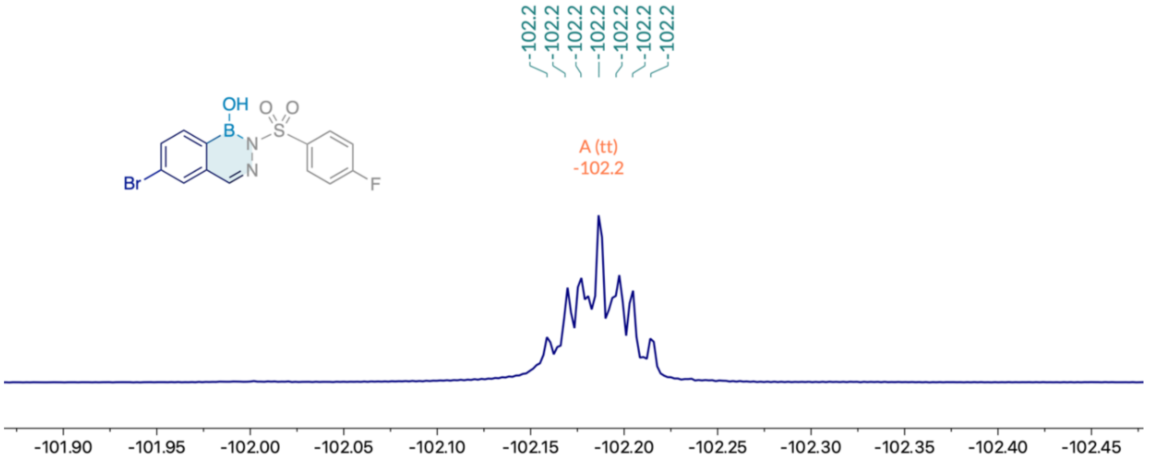

Figure S133. Diazaborine 25:^{19}F NMR (470 MHz, $CDCl_3$, 298 K)

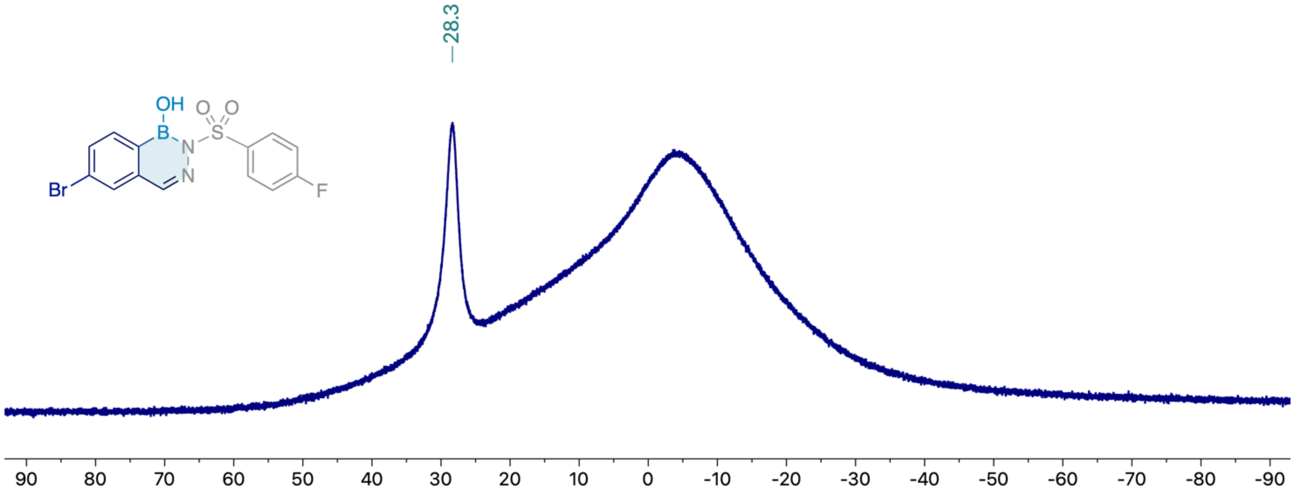

Figure S134. Diazaborine 25:^{11}B NMR (160 MHz, $CDCl_3$, 298 K)

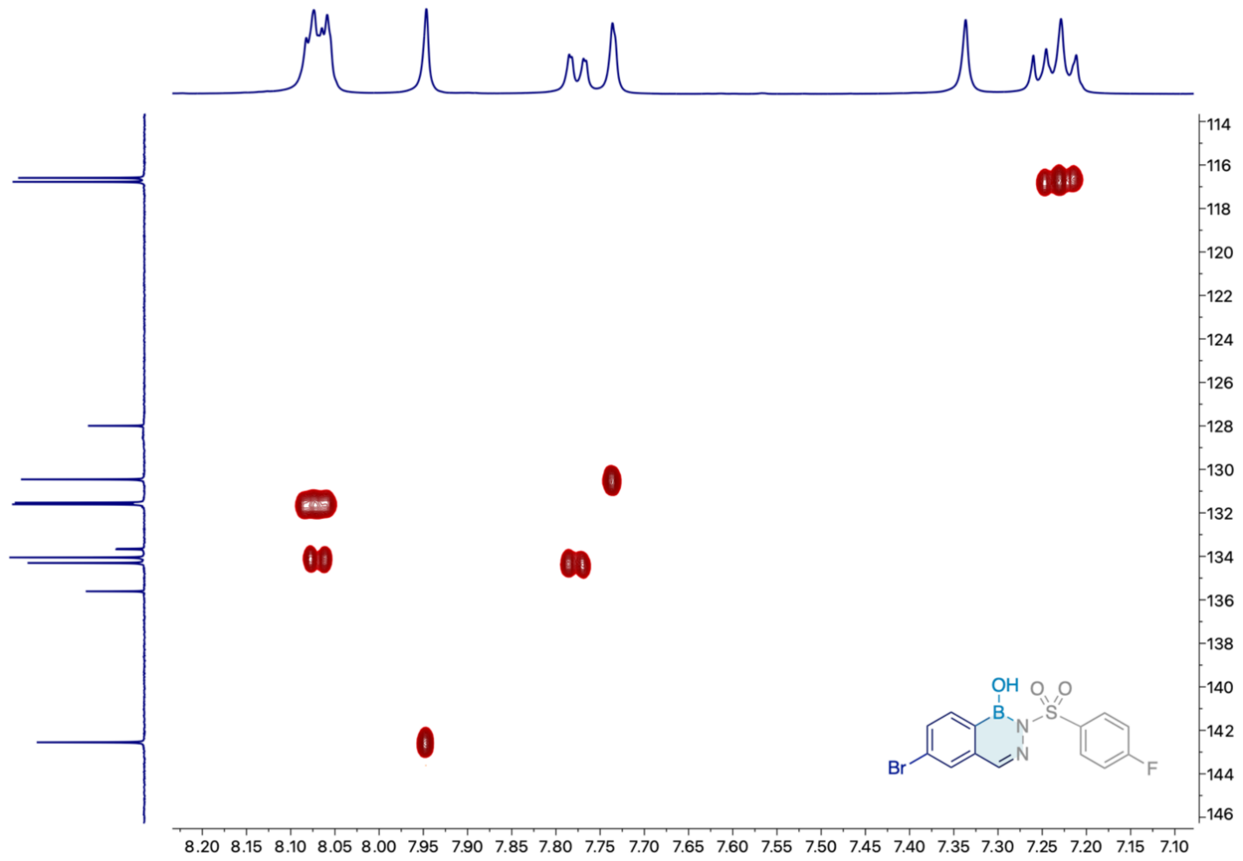

Figure S135. Diazaborine 25:^{1}H-^{13}C gHSQC NMR ($CDCl_3$, 298 K)

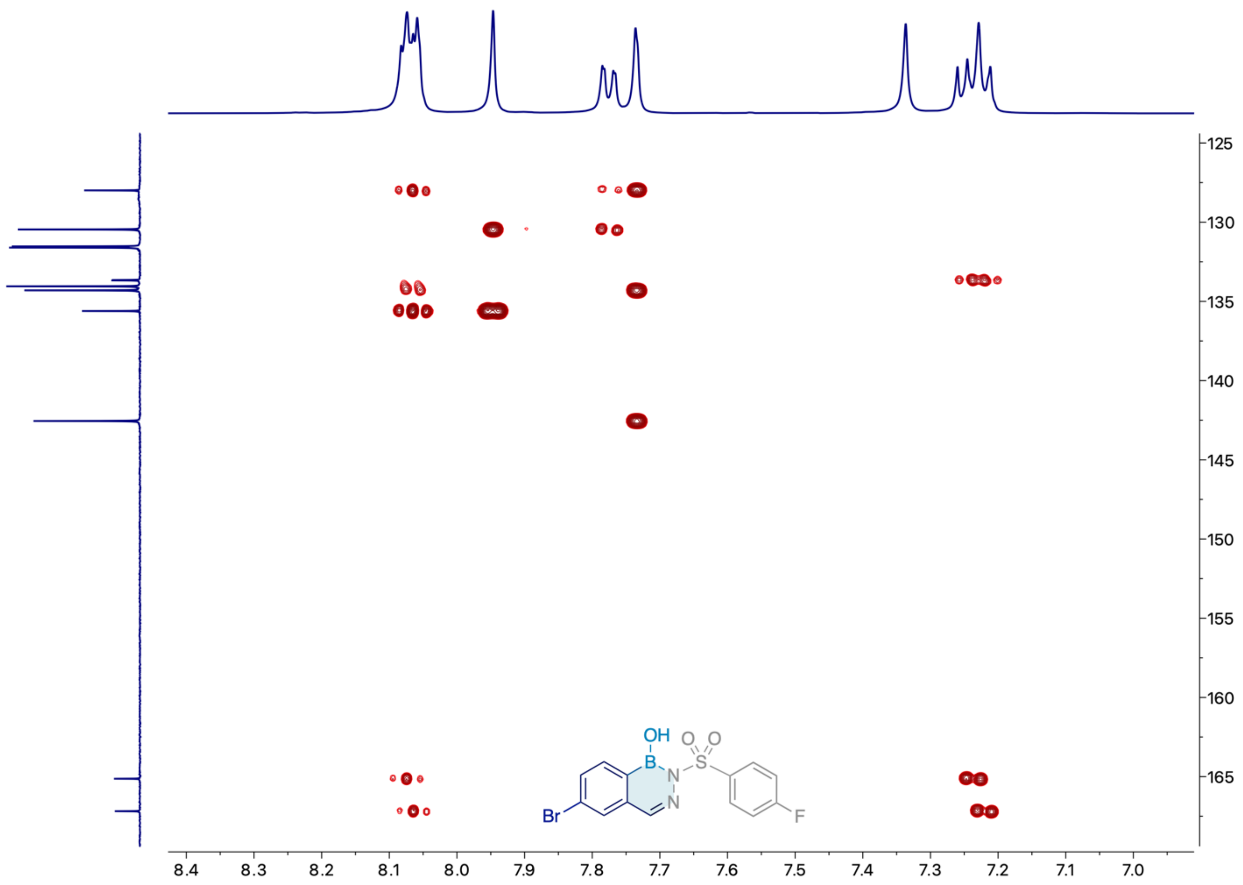

Figure S136. Diazaborine 25: ^{1}H-^{13}C gHMBC NMR ($CDCl_3$, 298 K)

Diazaborine 26

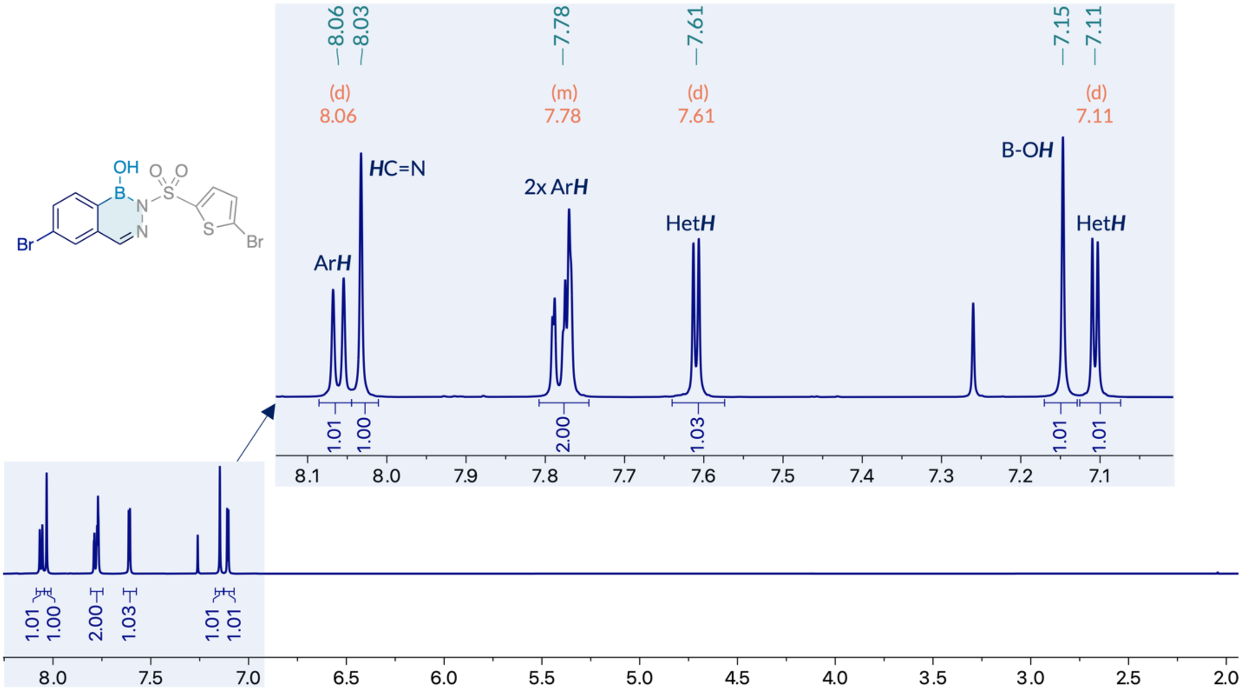

Figure S137. Diazaborine 26: ^{1}H NMR (600 MHz, $CDCl_3$, 298 K)

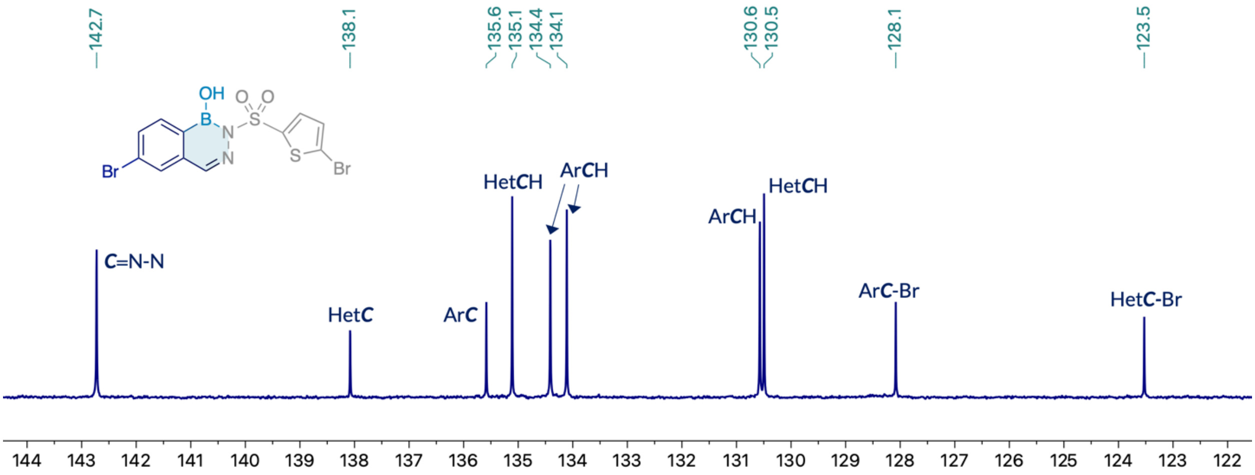

Figure S138. Diazaborine 26: ^{13}C NMR (151 MHz, $CDCl_3$, 298 K)

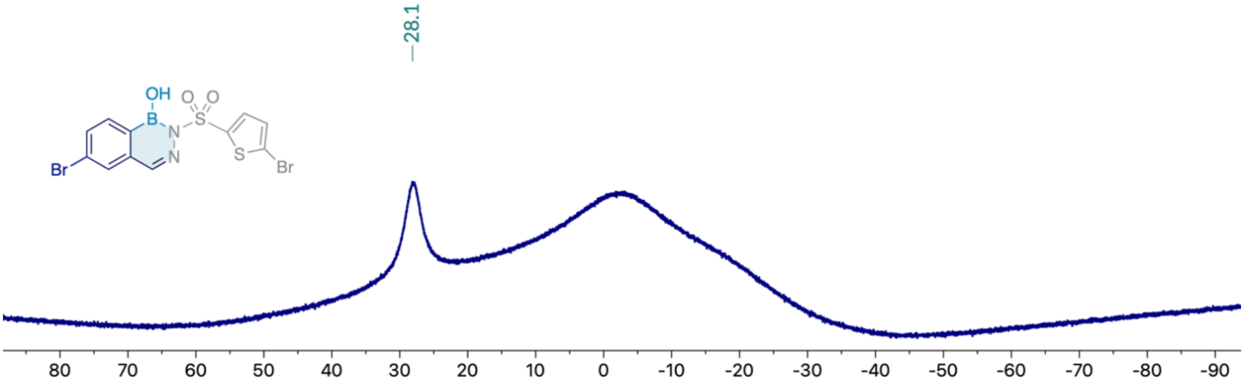

Figure S139. Diazaborine 26: ^{11}B NMR (160 MHz, $CDCl_3$, 298 K)

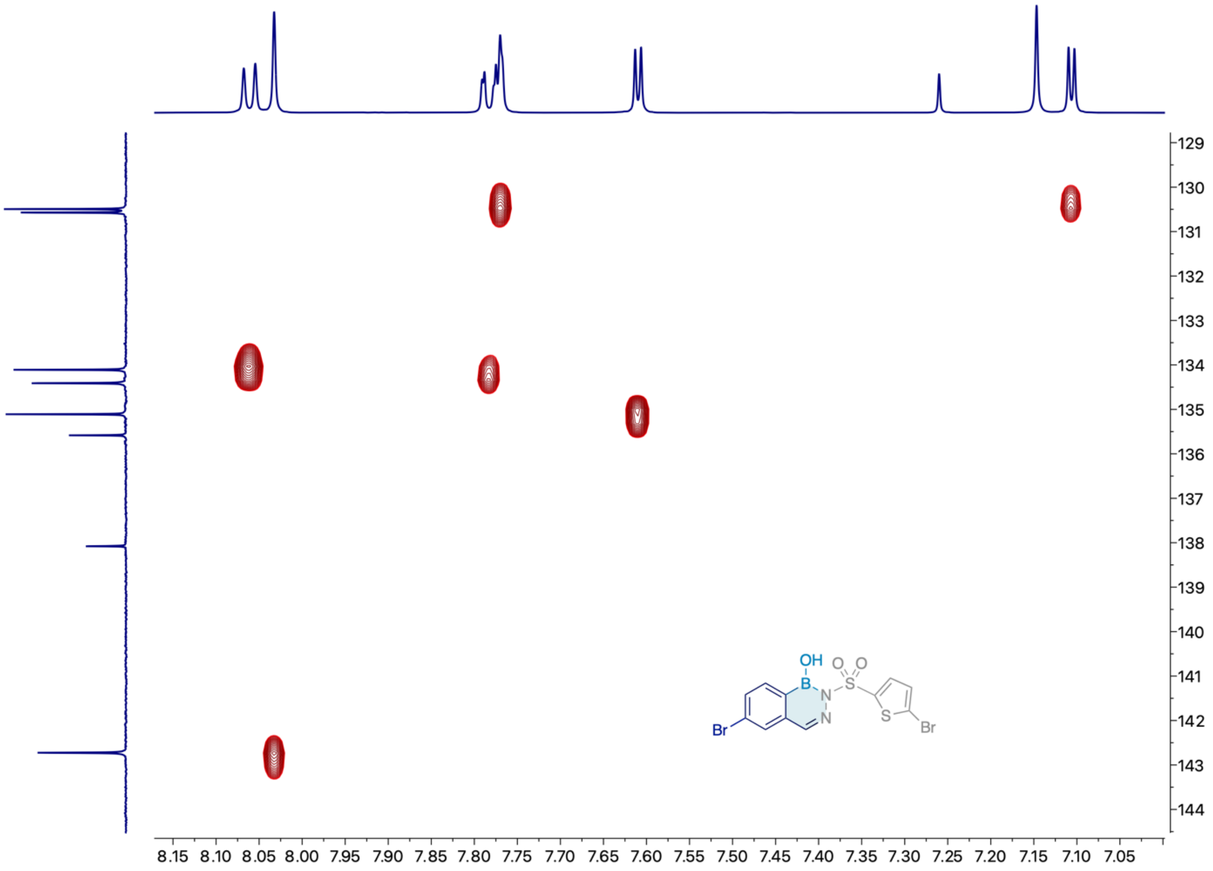

Figure S140. Diazaborine 26:^{1}H-^{13}C gHSQC NMR ($CDCl_3$, 298 K)

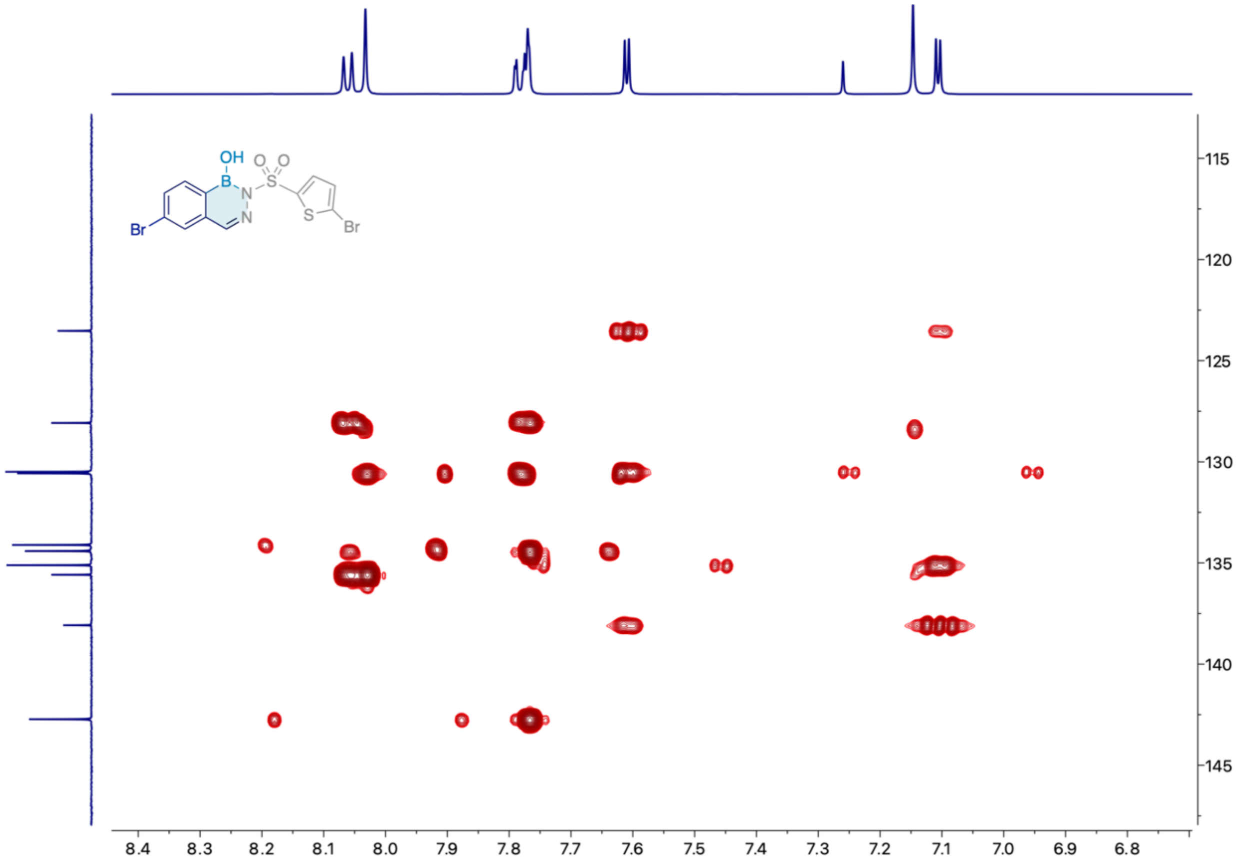

Figure S141. Diazaborine 26:^{1}H-^{13}C gHMBC NMR ($CDCl_3$, 298 K)

Diazaborine 27

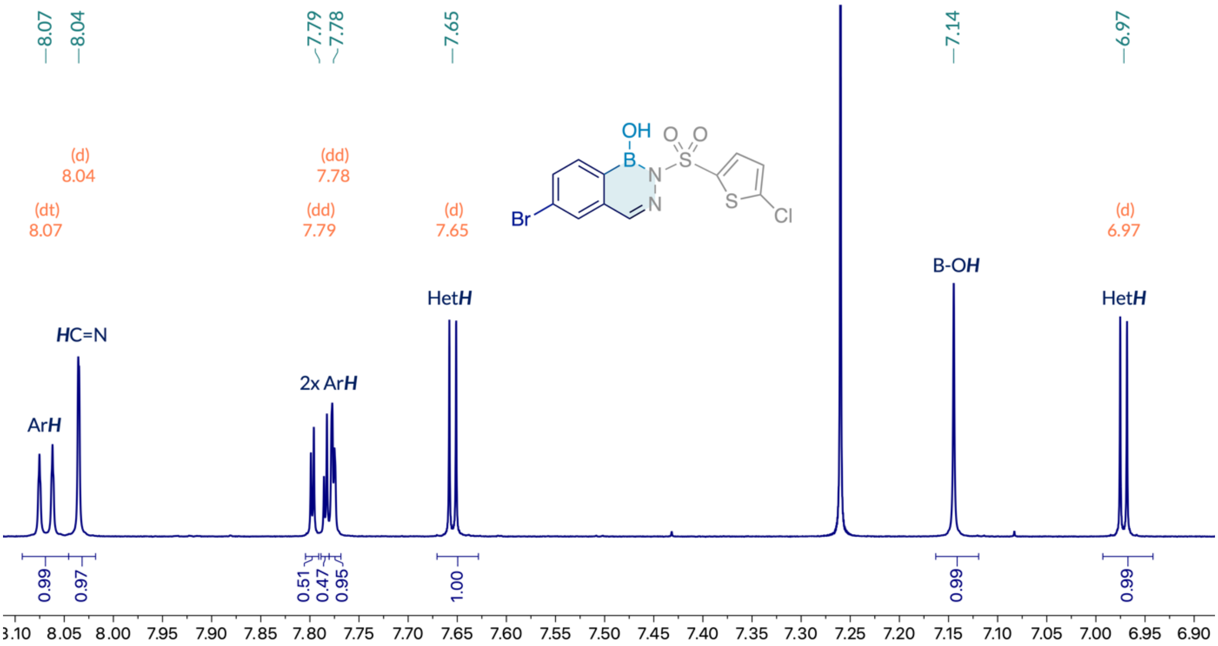

Figure S142. Diazaborine 27: ^{1}H NMR (600 MHz, $CDCl_3$, 298 K)

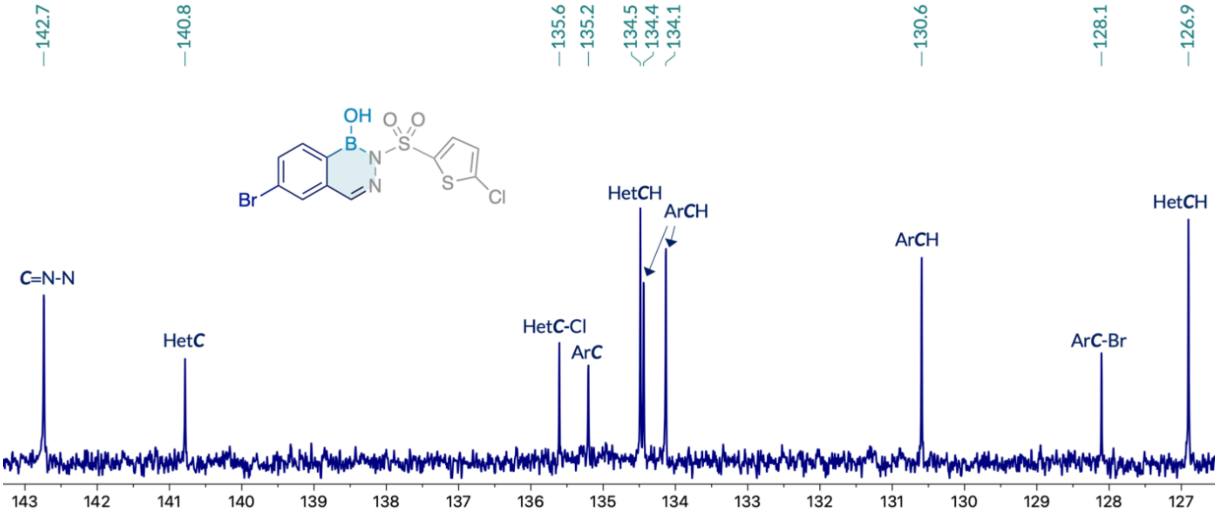

Figure S143. Diazaborine 27: ^{13}C NMR (151 MHz, $CDCl_3$, 298 K)

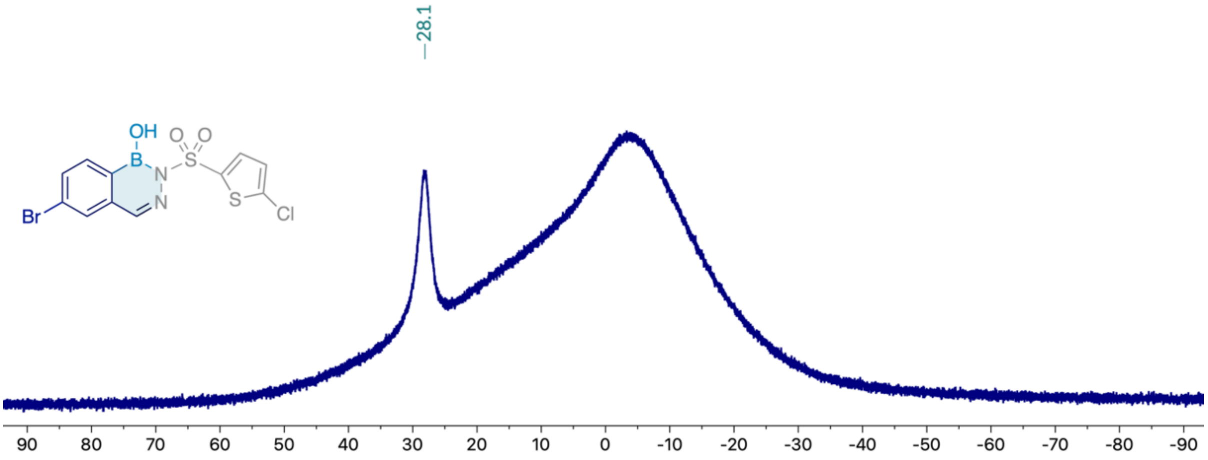

Figure S144. Diazaborine 27: ^{11}B NMR (160 MHz, $CDCl_3$, 298 K)

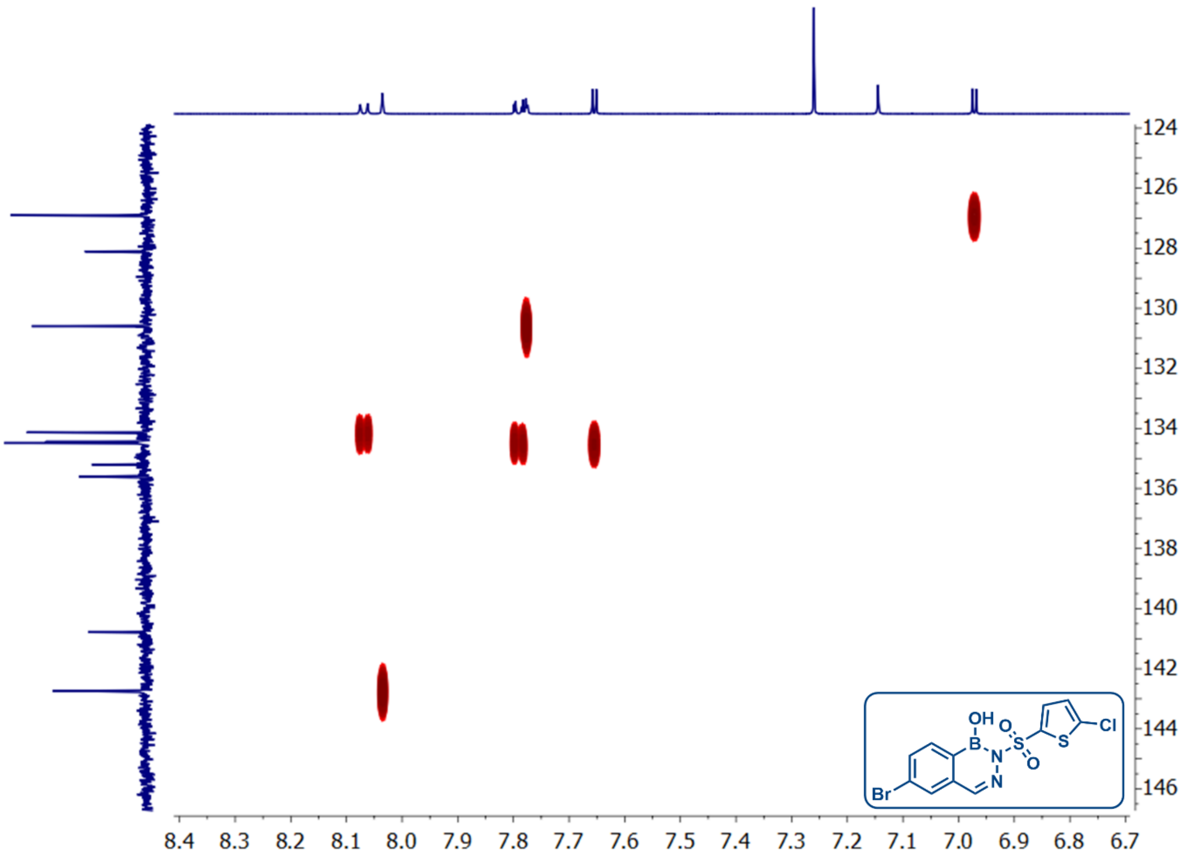

Figure S145. Diazaborine 27:^{1}H-^{13}C gHSQC NMR ($CDCl_3$, 298 K)

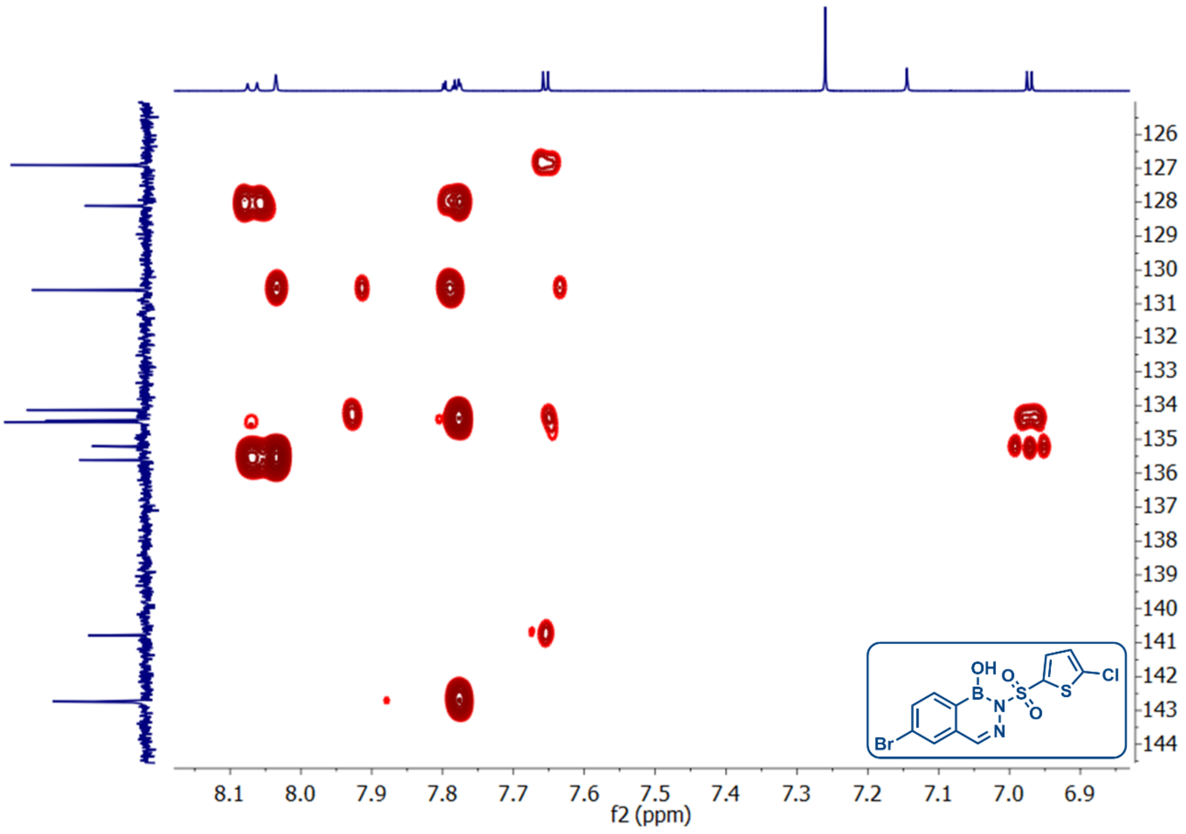

Figure S146. Diazaborine 27:^{1}H-^{13}C gHMBC NMR ($CDCl_3$, 298 K)

Diazaborine 28

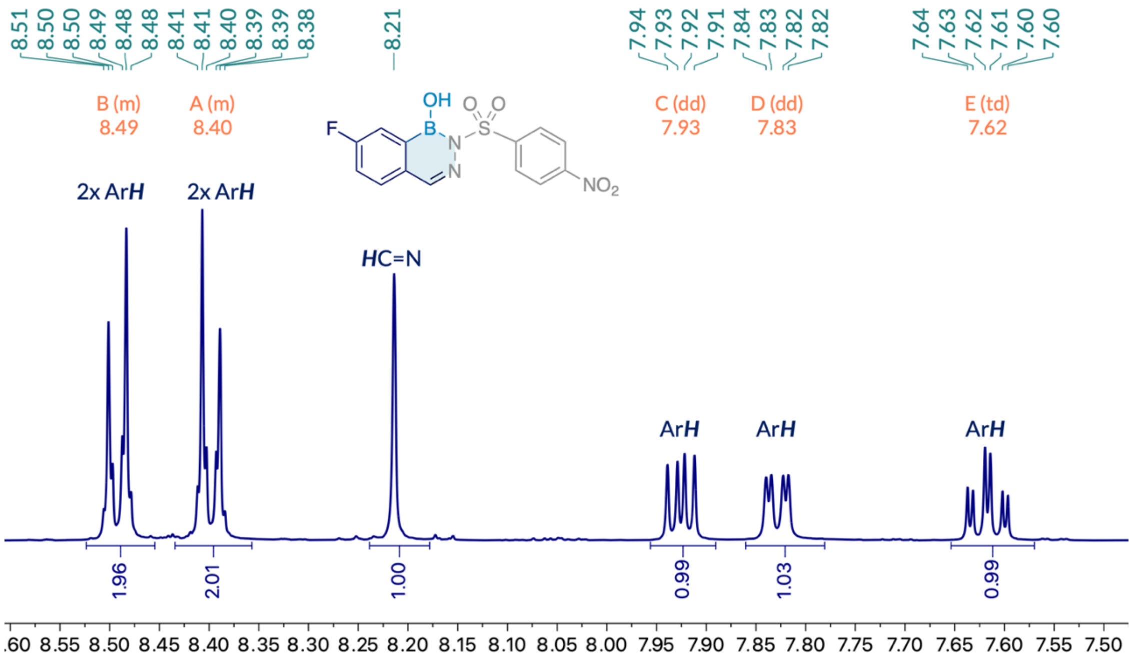

Figure S147. Diazaborine 28:^{1}H NMR (600 MHz, Acetone-d_6, 298 K)

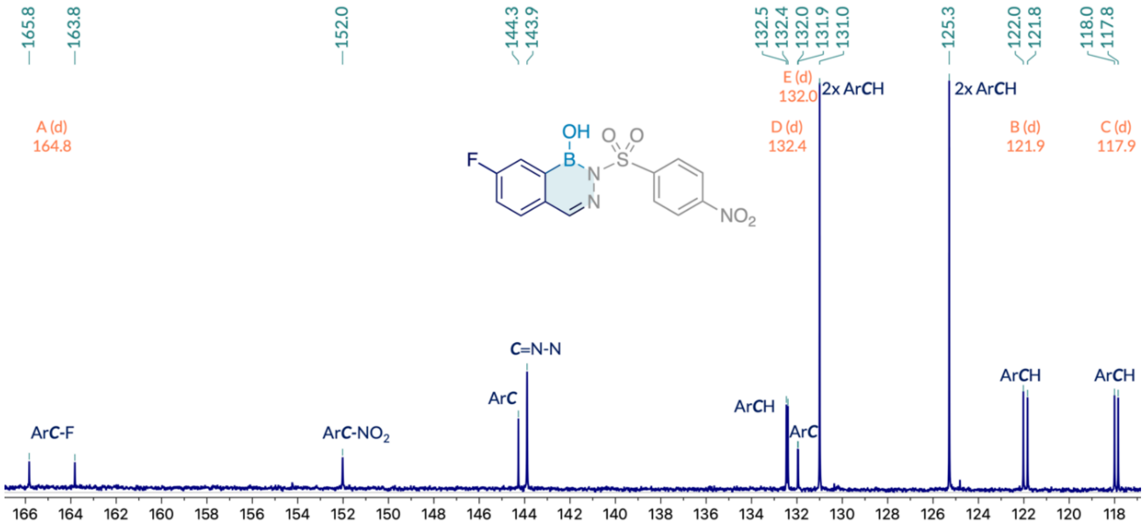

Figure S148. Diazaborine 28:^{13}C NMR (126 MHz, Acetone-d_6, 298 K)

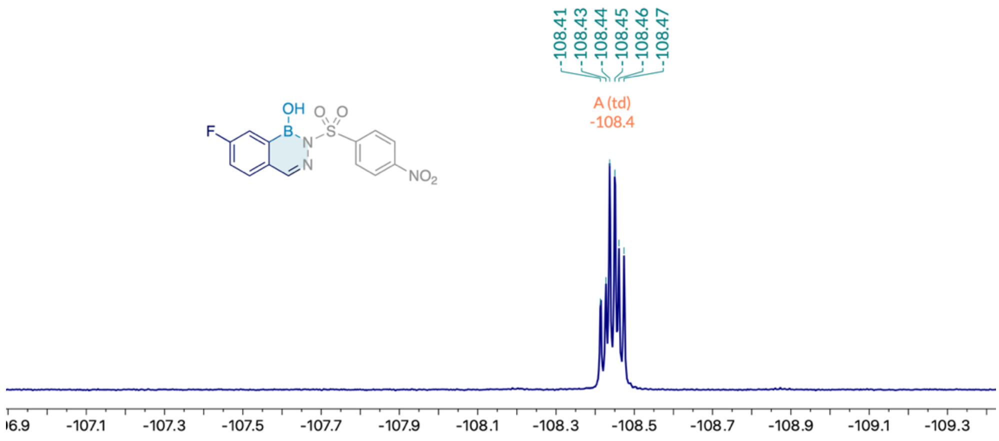

Figure S149. Diazaborine 28:^{19}F NMR (377 MHz, Acetone-d_6, 298 K)

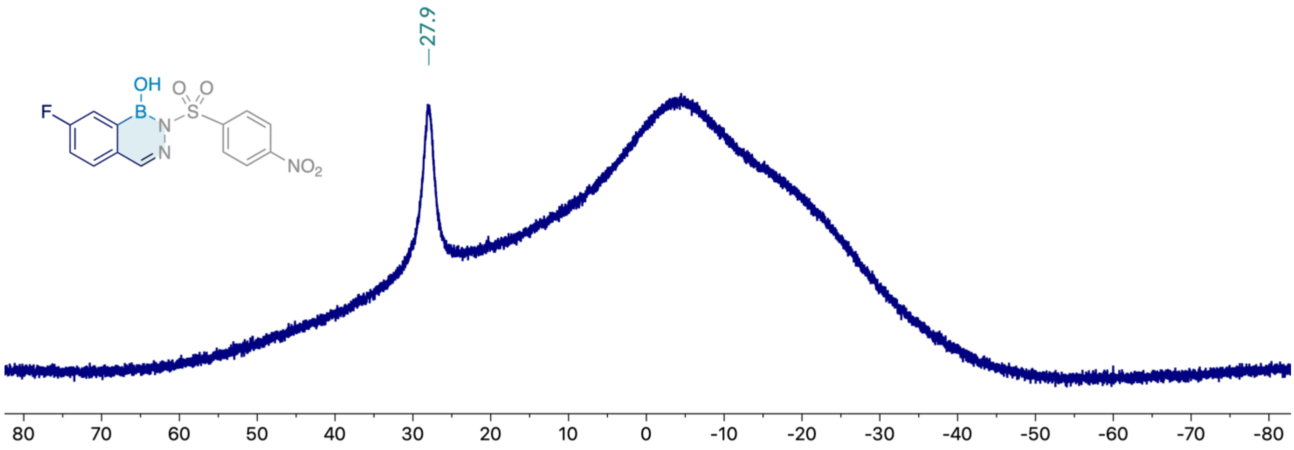

Figure S150. Diazaborine 28:^{11}B NMR (128 MHz, Acetone-d_6, 298 K)

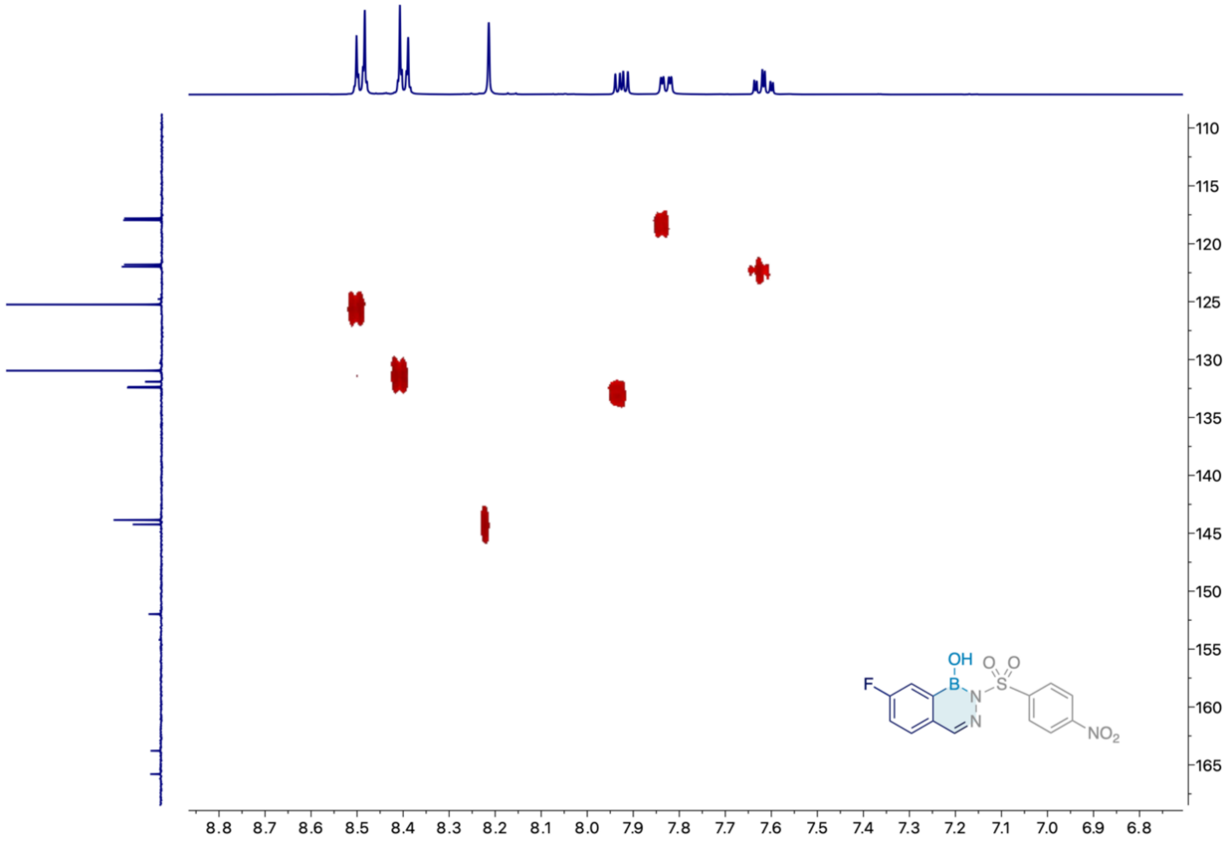

Figure S151. Diazaborine 28:^{1}H-^{13}C gHSQC NMR (Acetone-d_6, 298 K)

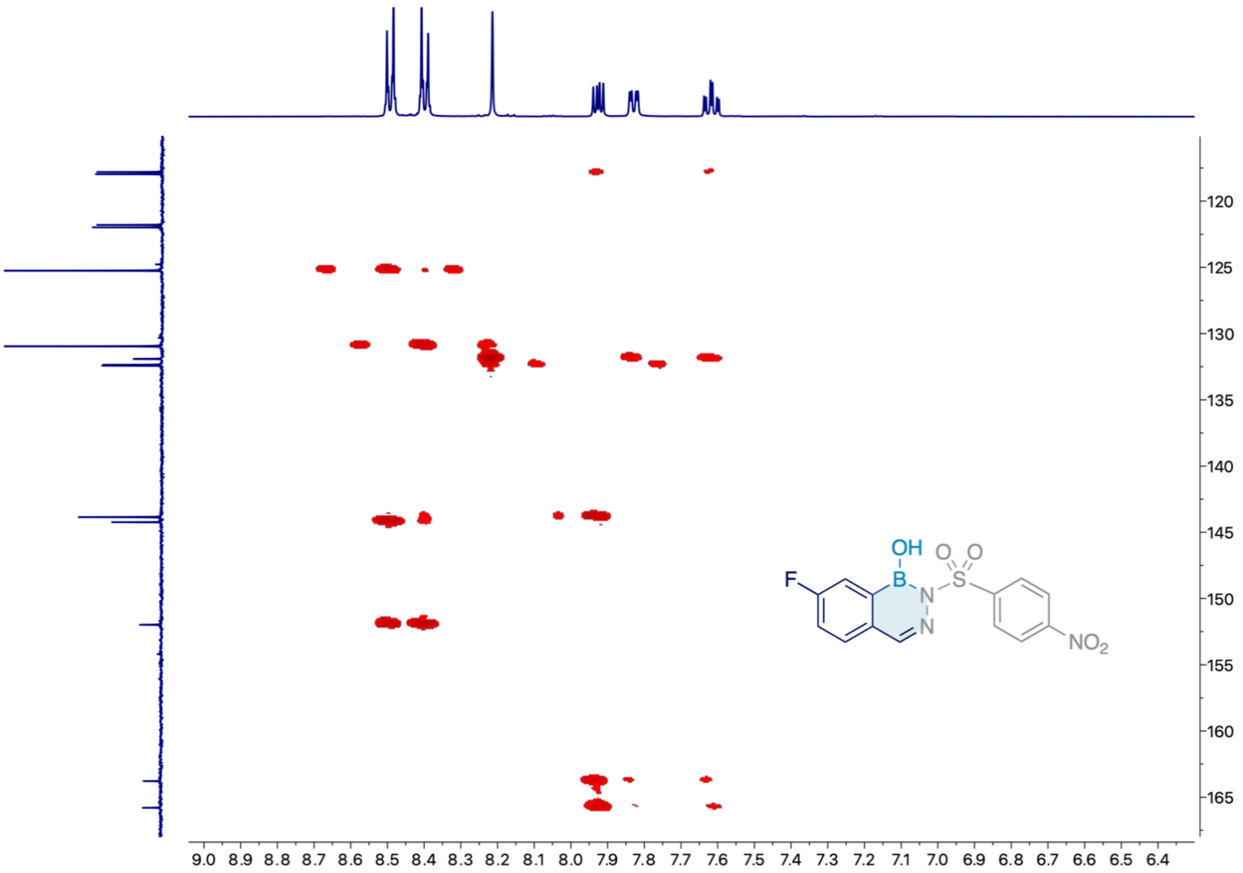

Figure S152. Diazaborine 28:^{1}H-^{13}C gHMBC NMR (Acetone-d_6, 298 K)

Diazaborine 29

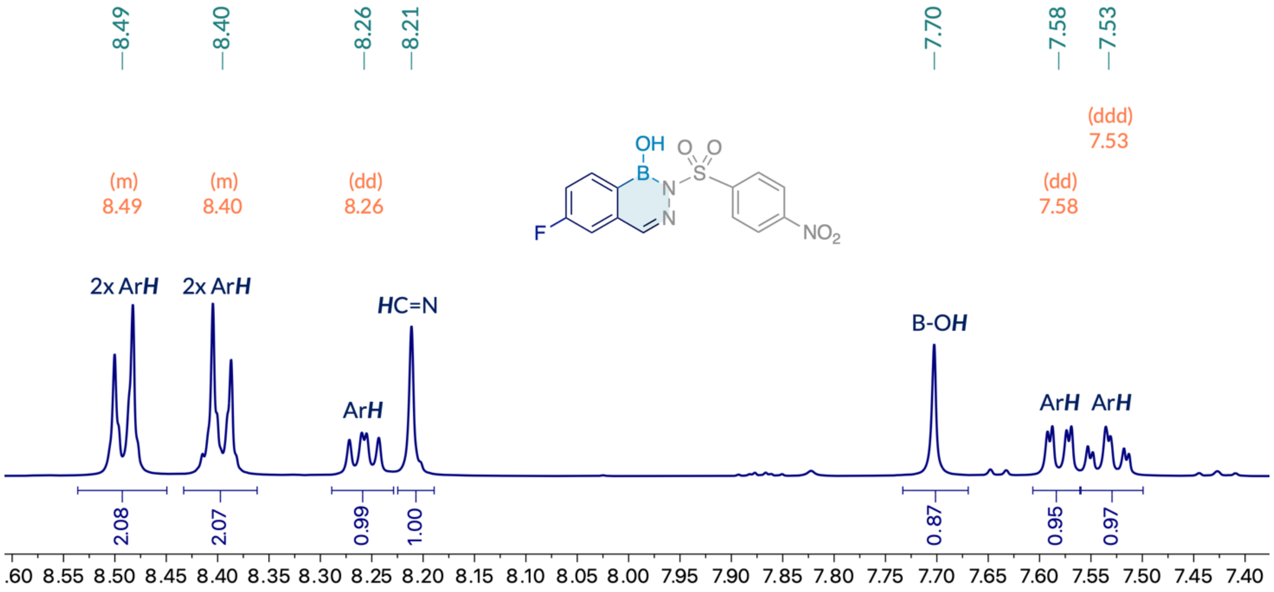

Figure S153. Diazaborine 29:^{1}H NMR (500 MHz, Acetone-d_6, 298 K)

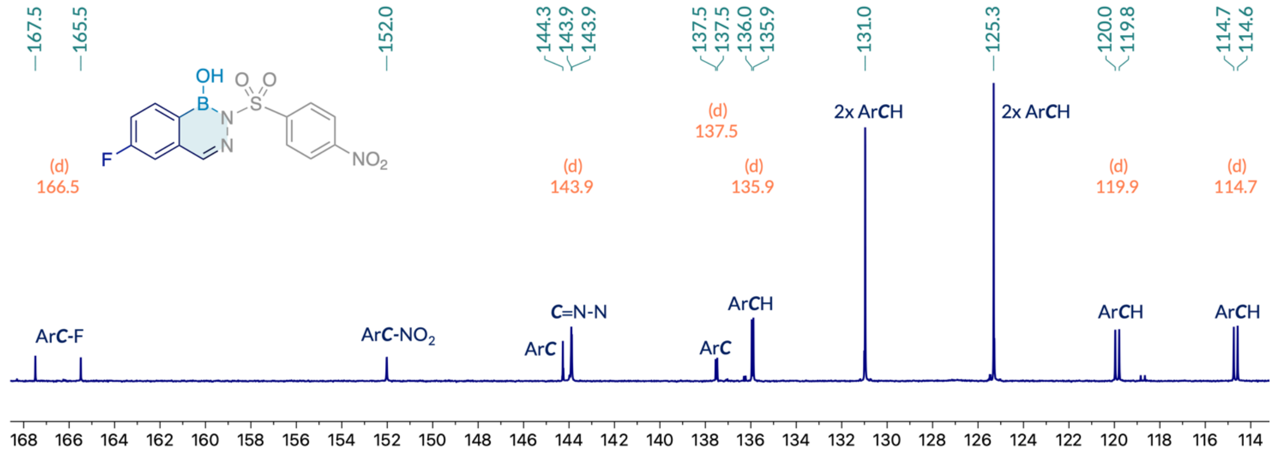

Figure S154. Diazaborine 29:^{13}C NMR (126 MHz, Acetone-d_6, 298 K)

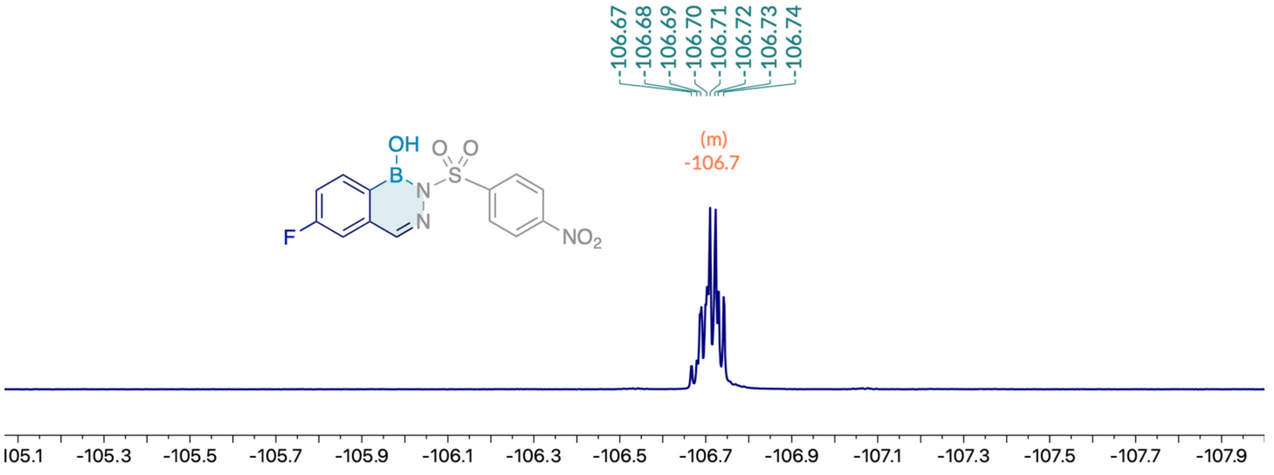

Figure S155. Diazaborine 29:^{19}F NMR (377 MHz, Acetone-d_6, 298 K)

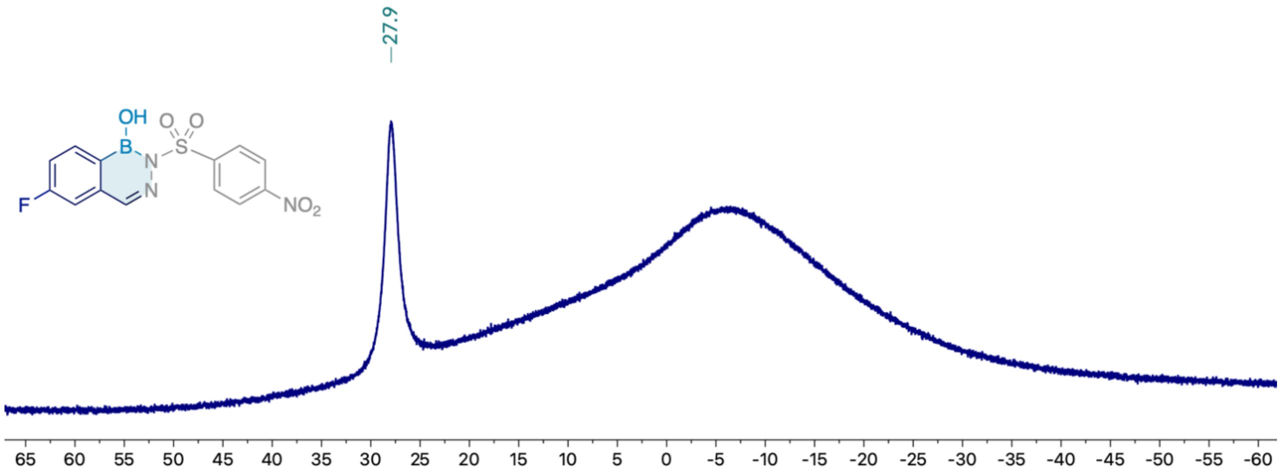

Figure S156. Diazaborine 29:^{11}B NMR (128 MHz, Acetone-d_6, 298 K)

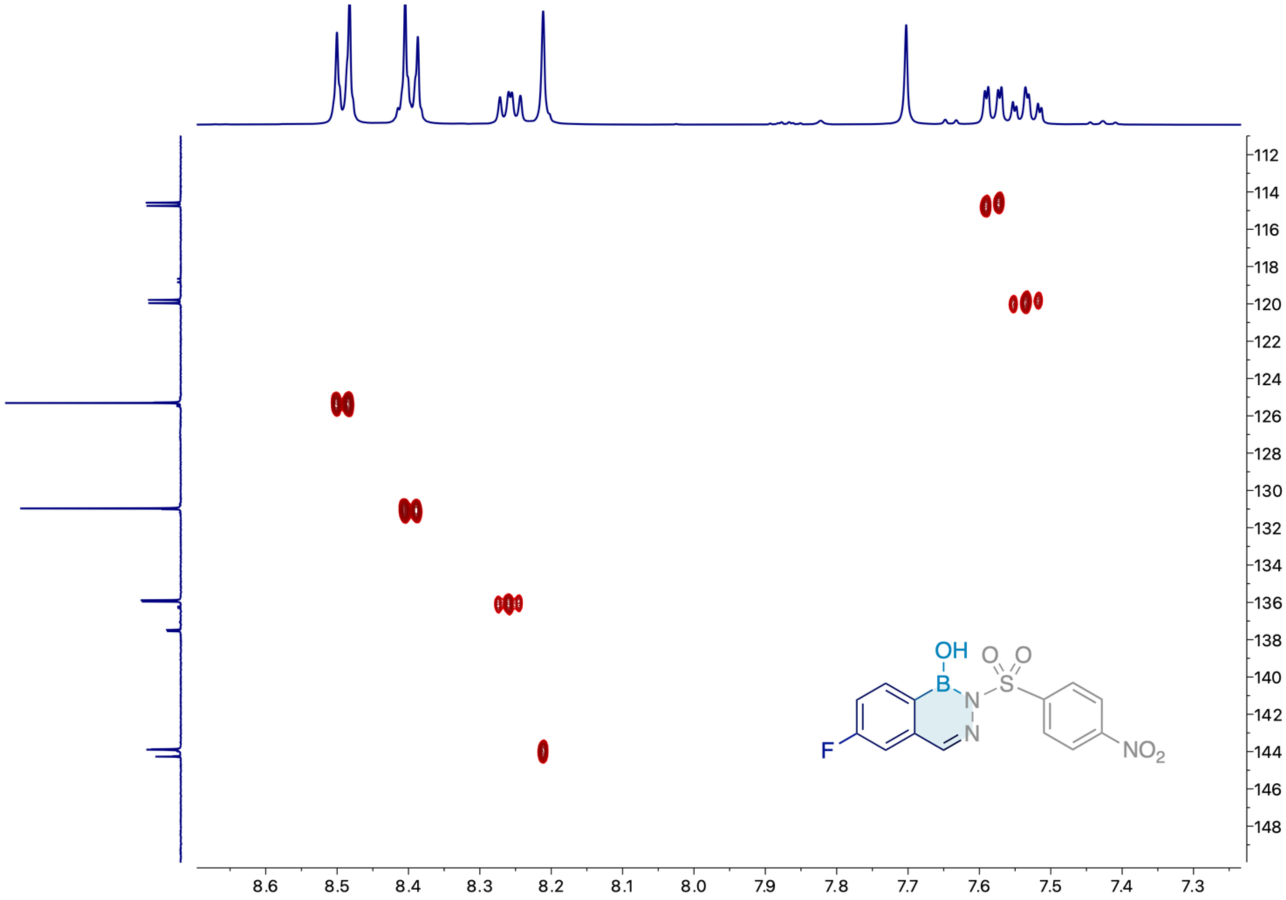

Figure S157. Diazaborine 29:^{1}H-^{13}C gHSQC NMR (Acetone-d_6, 298 K)

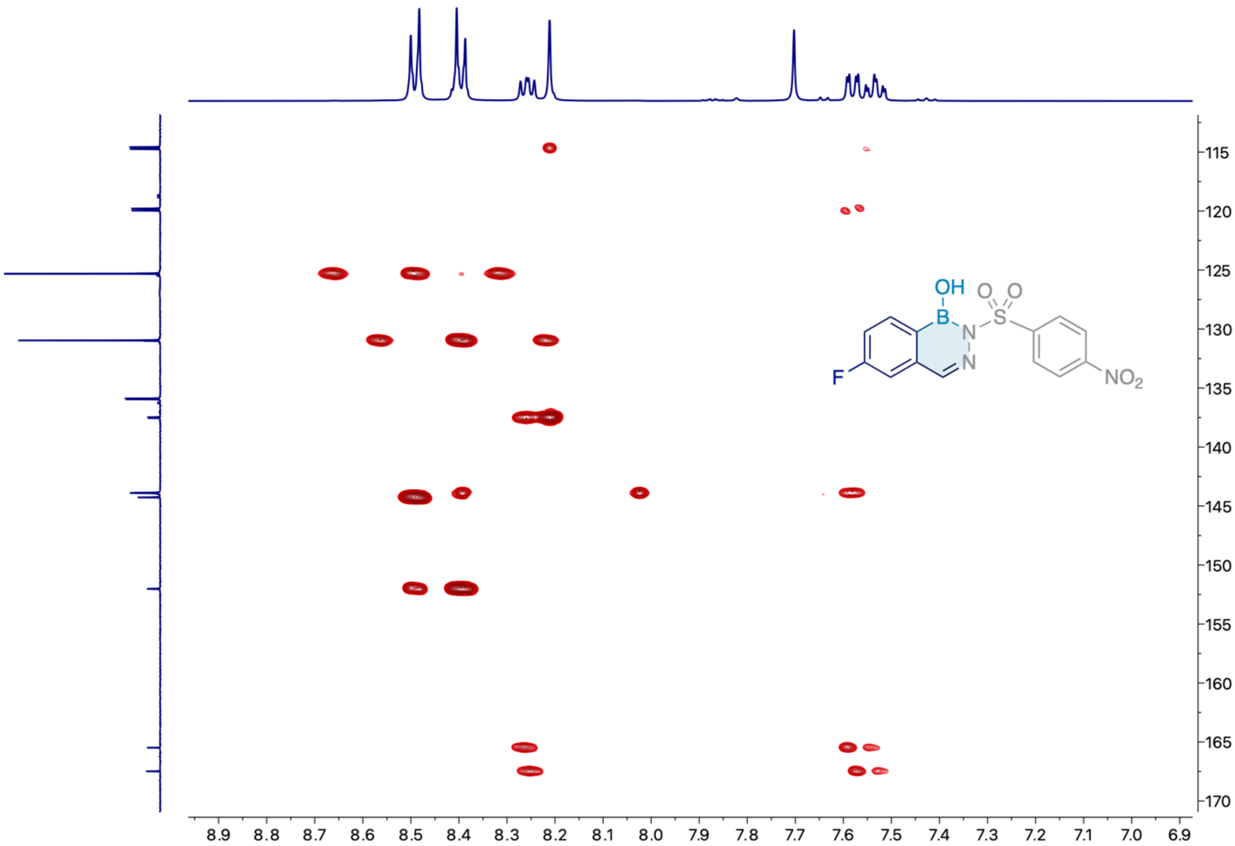

Figure S158. Diazaborine 29:^{1}H-^{13}C gHMBC NMR (Acetone-d_6, 298 K)

Diazaborine 30

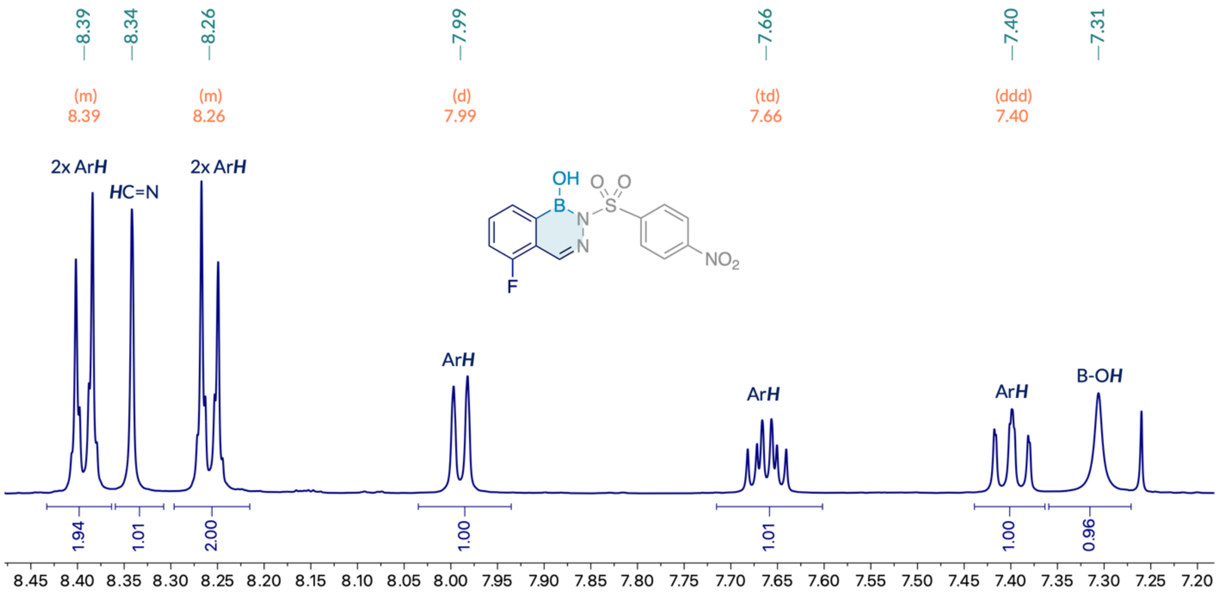

Figure S159. Diazaborine 30: ^{1}H NMR (500 MHz, $CDCl_3$, 298 K)

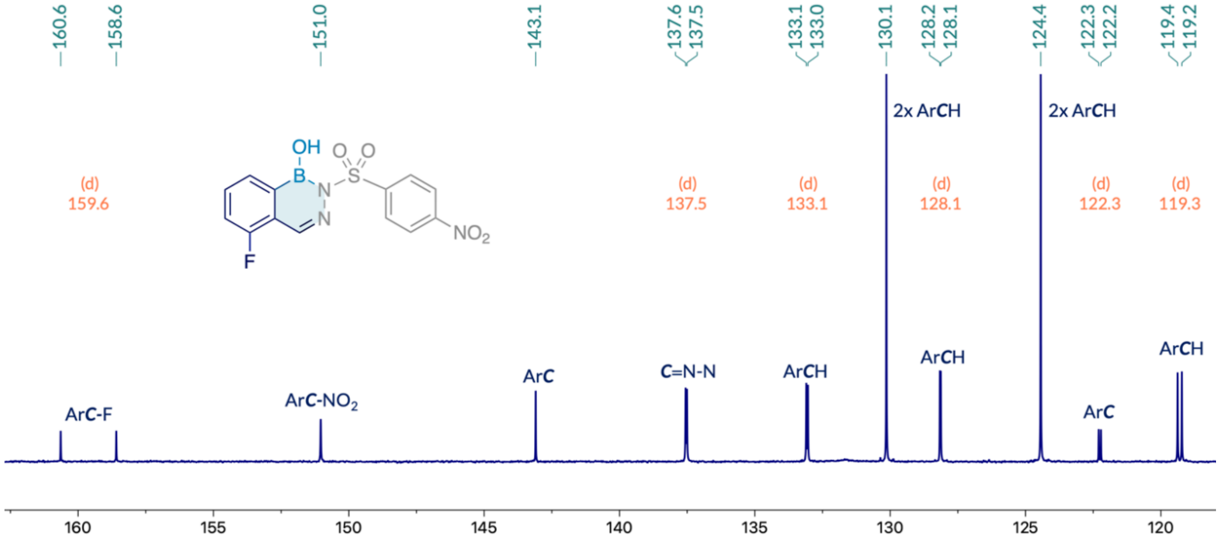

Figure S160. Diazaborine 30: ^{13}C NMR (126 MHz, $CDCl_3$, 298 K)

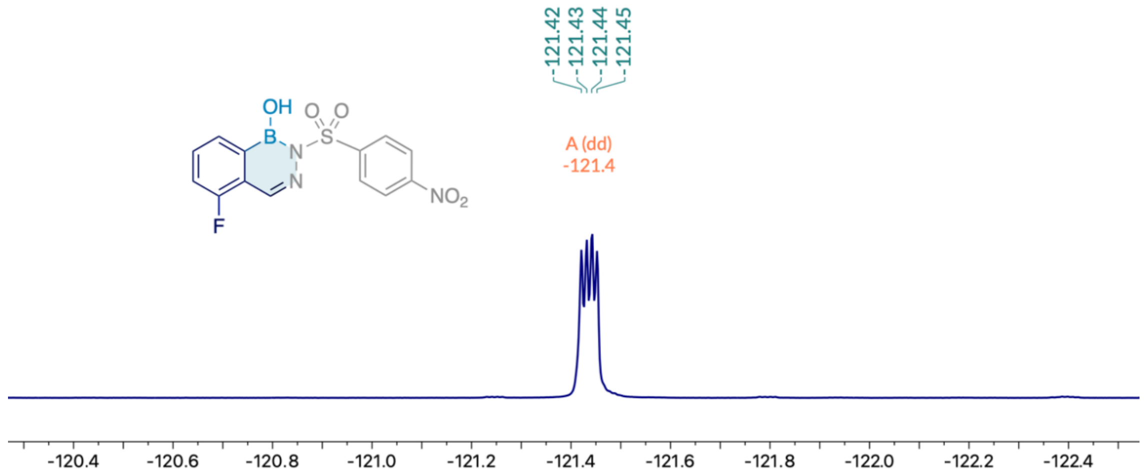

Figure S161. Diazaborine 30: ^{19}F NMR (470 MHz, $CDCl_3$, 298 K)

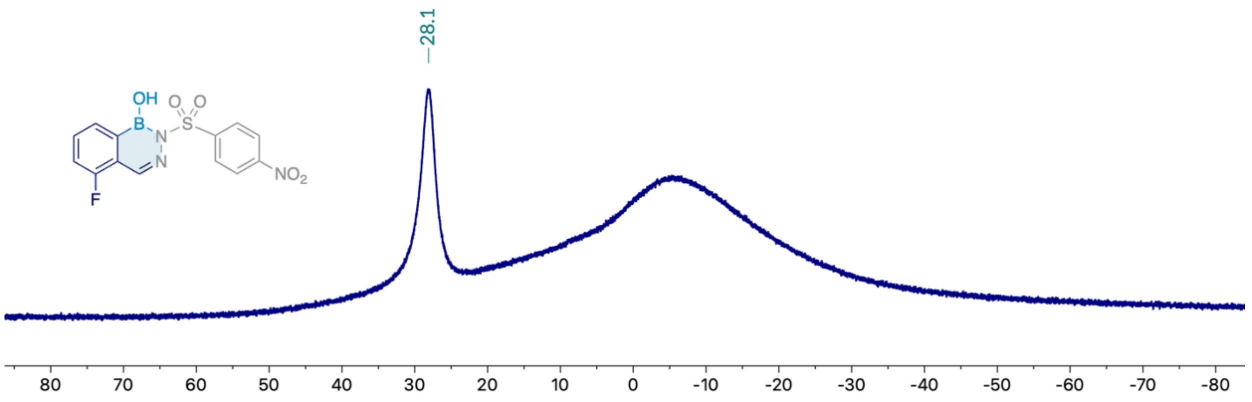

Figure S162. Diazaborine 30:^{11}B NMR (160 MHz, $CDCl_3$, 298 K)

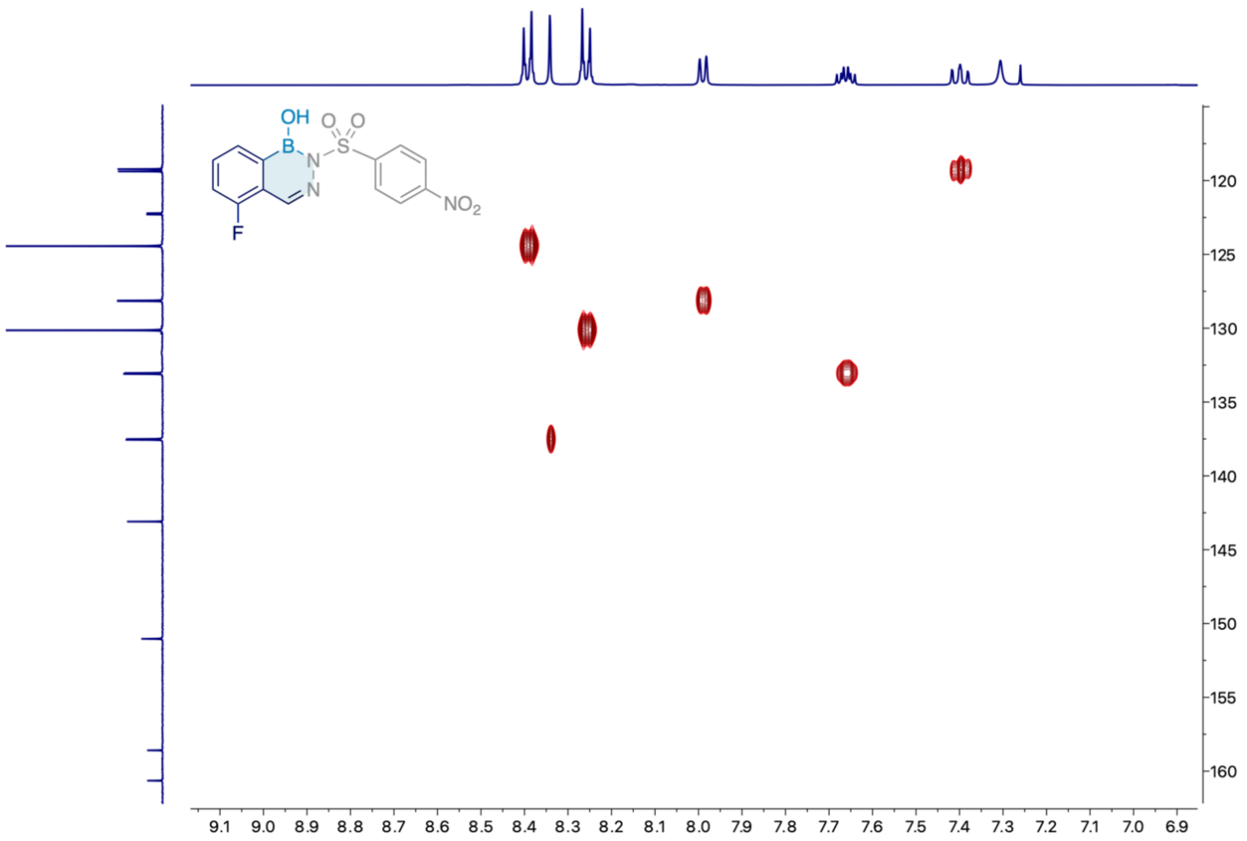

Figure S163. Diazaborine 30:^{1}H-^{13}C gHSQC NMR ($CDCl_3$, 298 K)

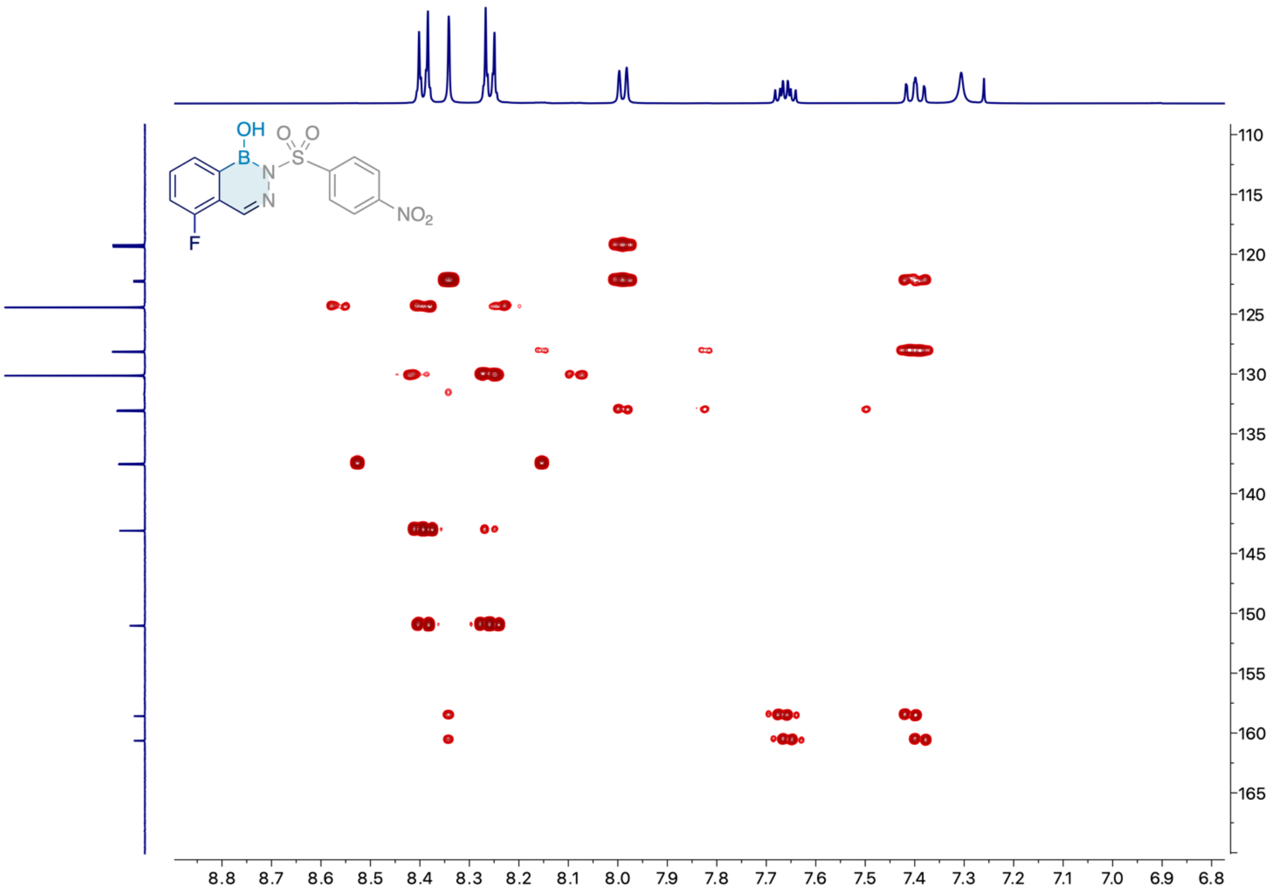

Figure S164. Diazaborine 30:^{1}H-^{13}C gHMBC NMR ($CDCl_3$, 298 K)

Diazaborine 31

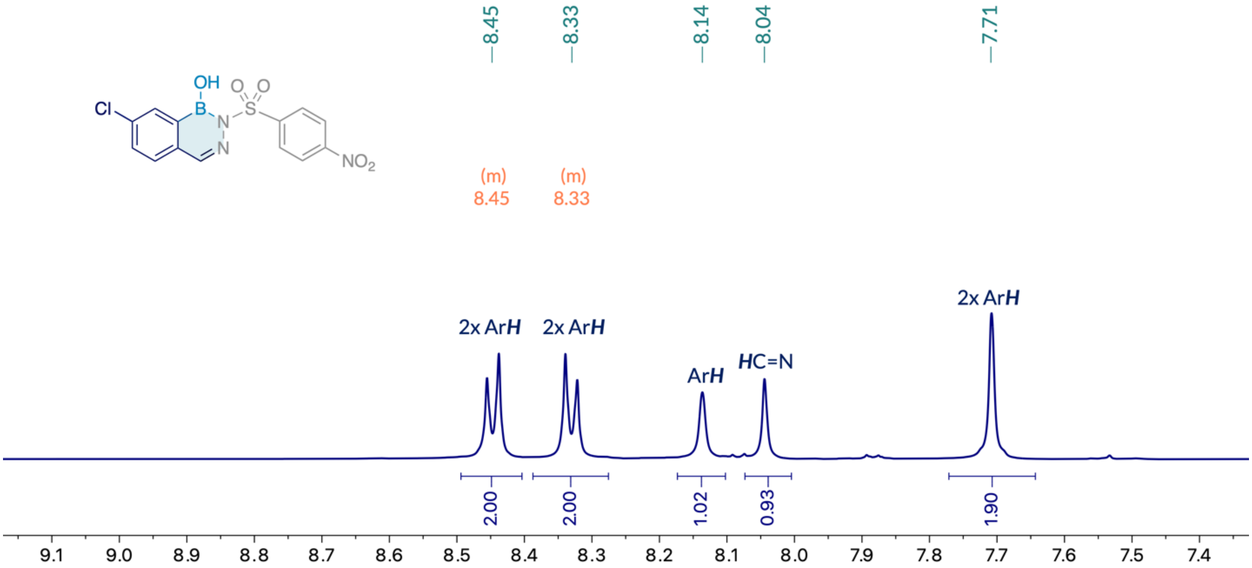

Figure S165. Diazaborine 31:^{1}H NMR (500 MHz, Acetone-d_6-DMSO-d_6 2:1, 298 K)

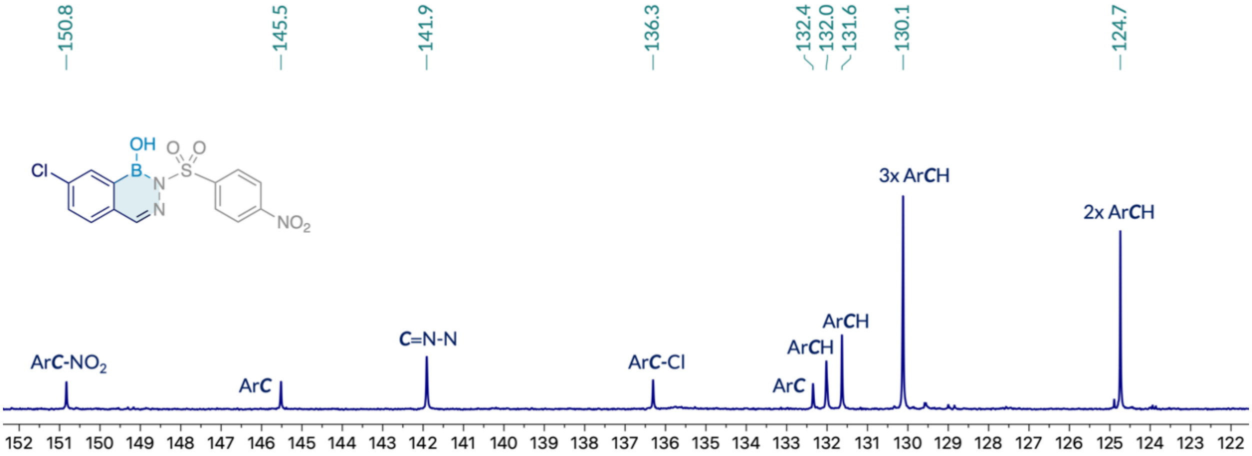

Figure S166. Diazaborine 31:^{13}C NMR (126 MHz, Acetone-d_6-DMSO-d_6 2:1, 298 K)

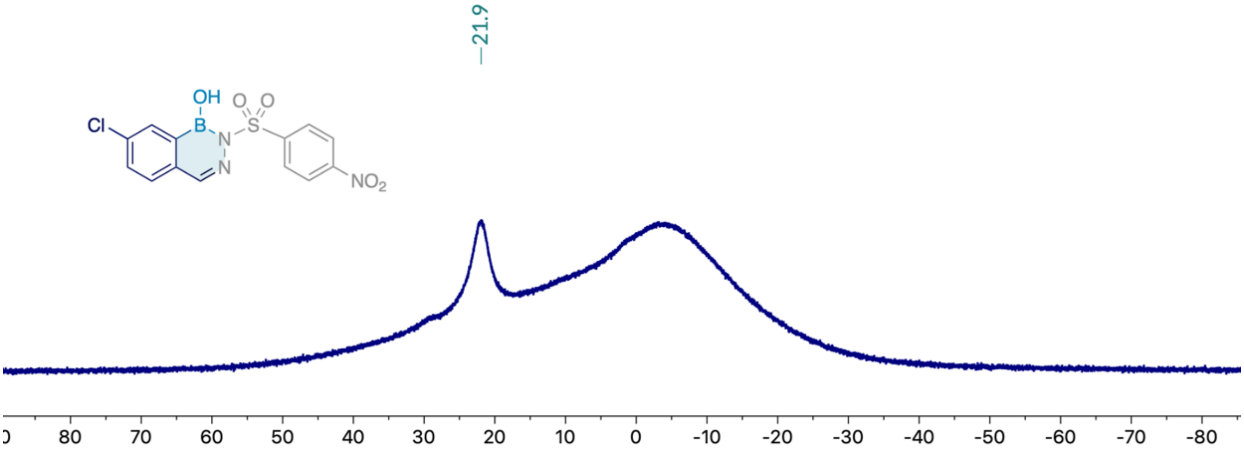

Figure S167. Diazaborine 31:^{11}B NMR (160 MHz, Acetone-d_6-DMSO-d_6 2:1, 298 K)

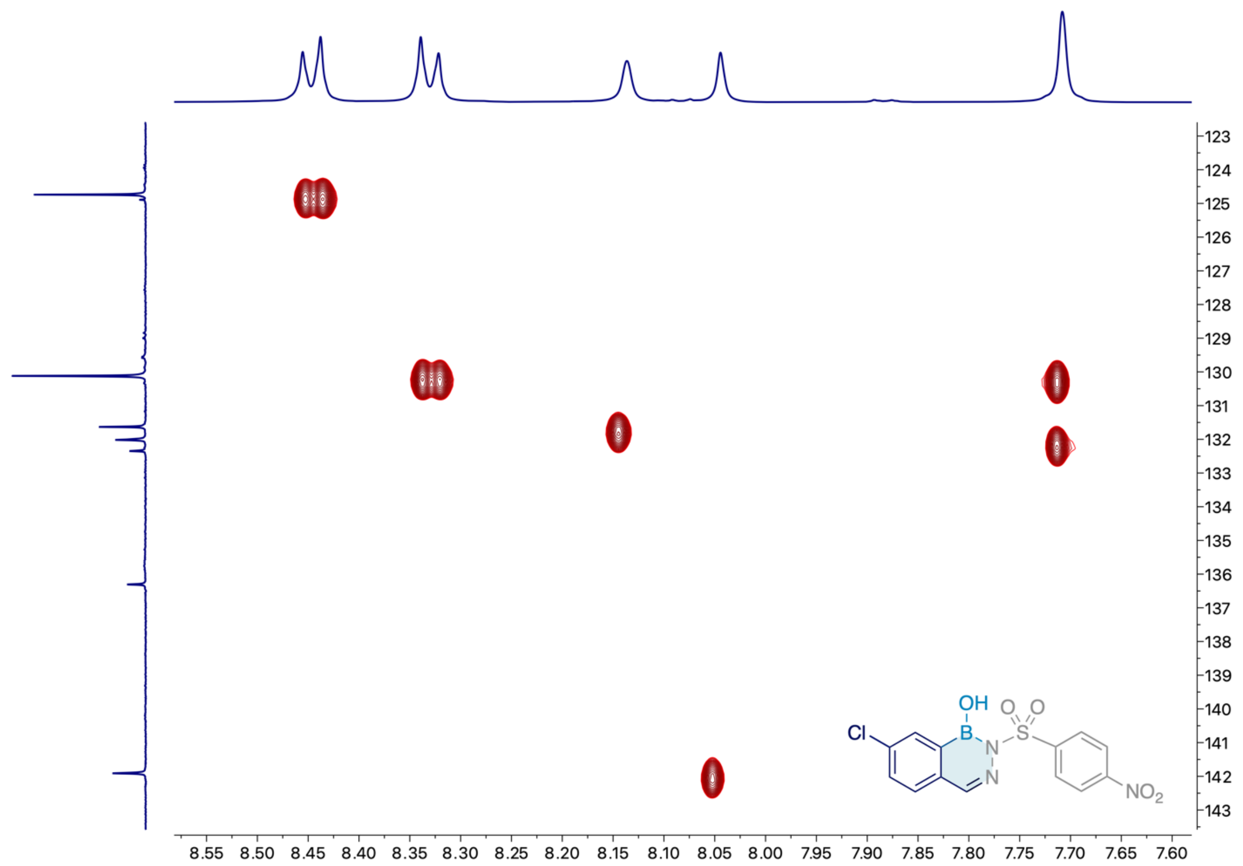

Figure S168. Diazaborine 31:^{1}H-^{13}C gHSQC NMR (Acetone-d_6-DMSO-d_6 2:1, 298 K)

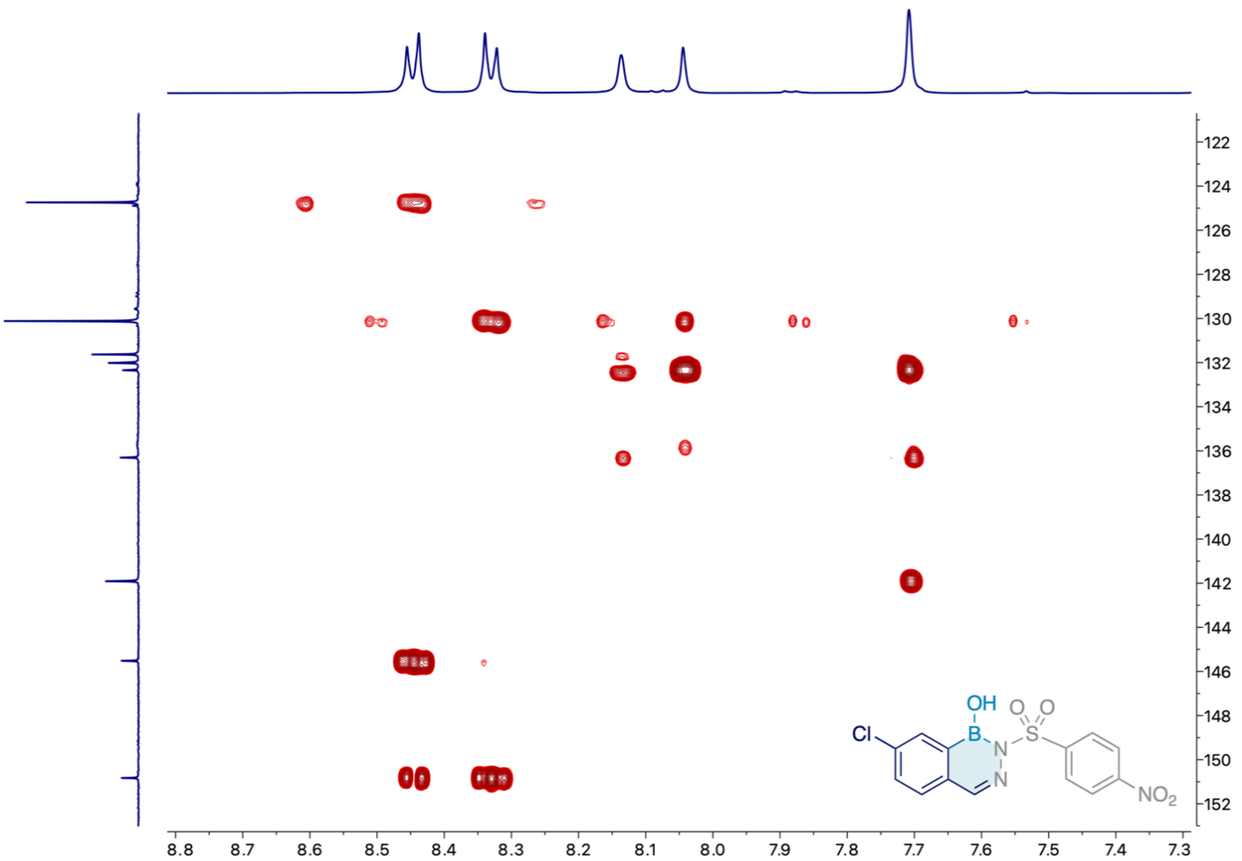

Figure S169. Diazaborine 31:^{1}H-^{13}C gHMBC NMR (Acetone-d_6-DMSO-d_6 2:1, 298 K)

Diazaborine 32

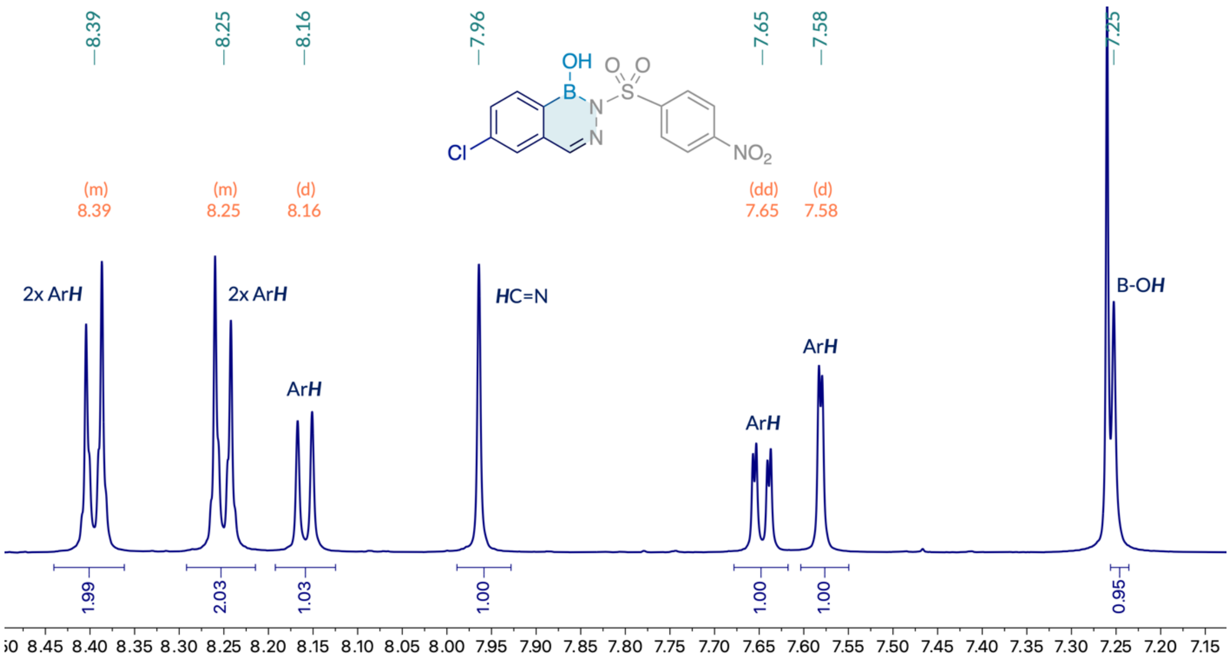

Figure S170. Diazaborine 32: ^{1}H NMR (500 MHz, $CDCl_3$, 298 K)

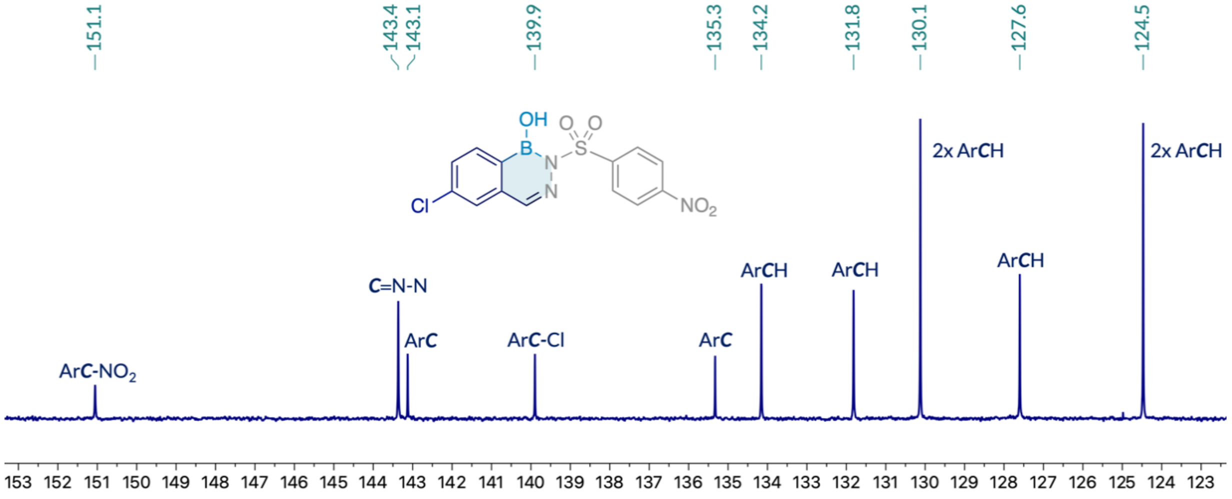

Figure S171. Diazaborine 32: ^{13}C NMR (126 MHz, $CDCl_3$, 298 K)

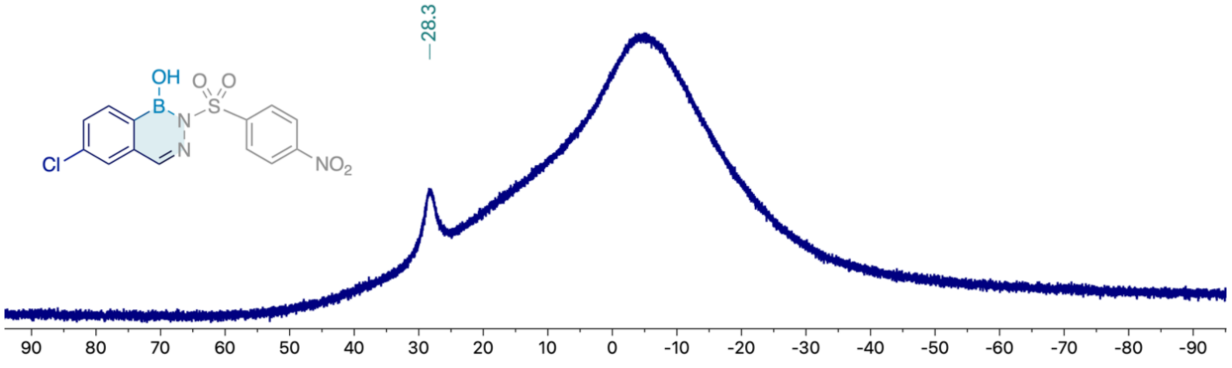

Figure S172. Diazaborine 32: ^{11}B NMR (160 MHz, $CDCl_3$, 298 K)

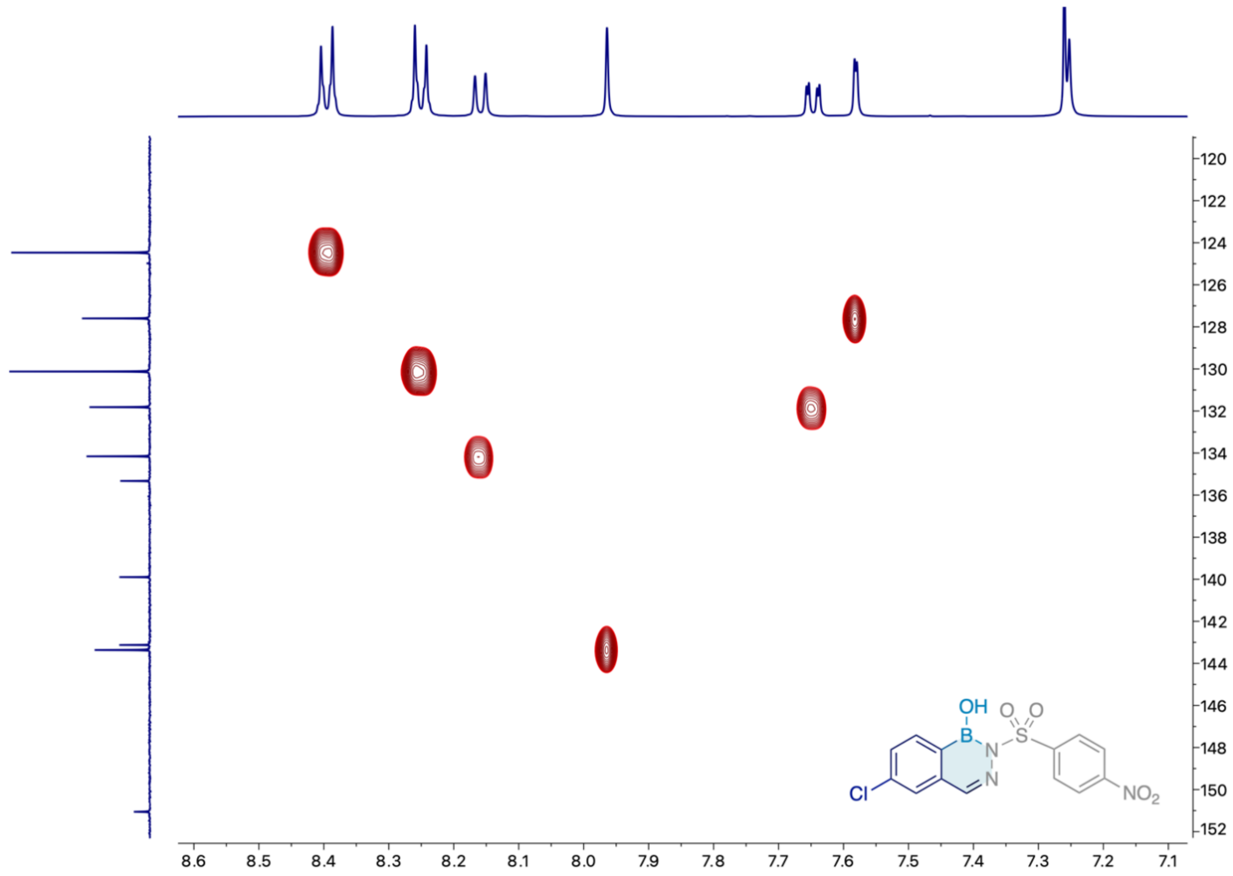

Figure S173. Diazaborine 32:^{1}H-^{13}C gHSQC NMR ($CDCl_3$, 298 K)

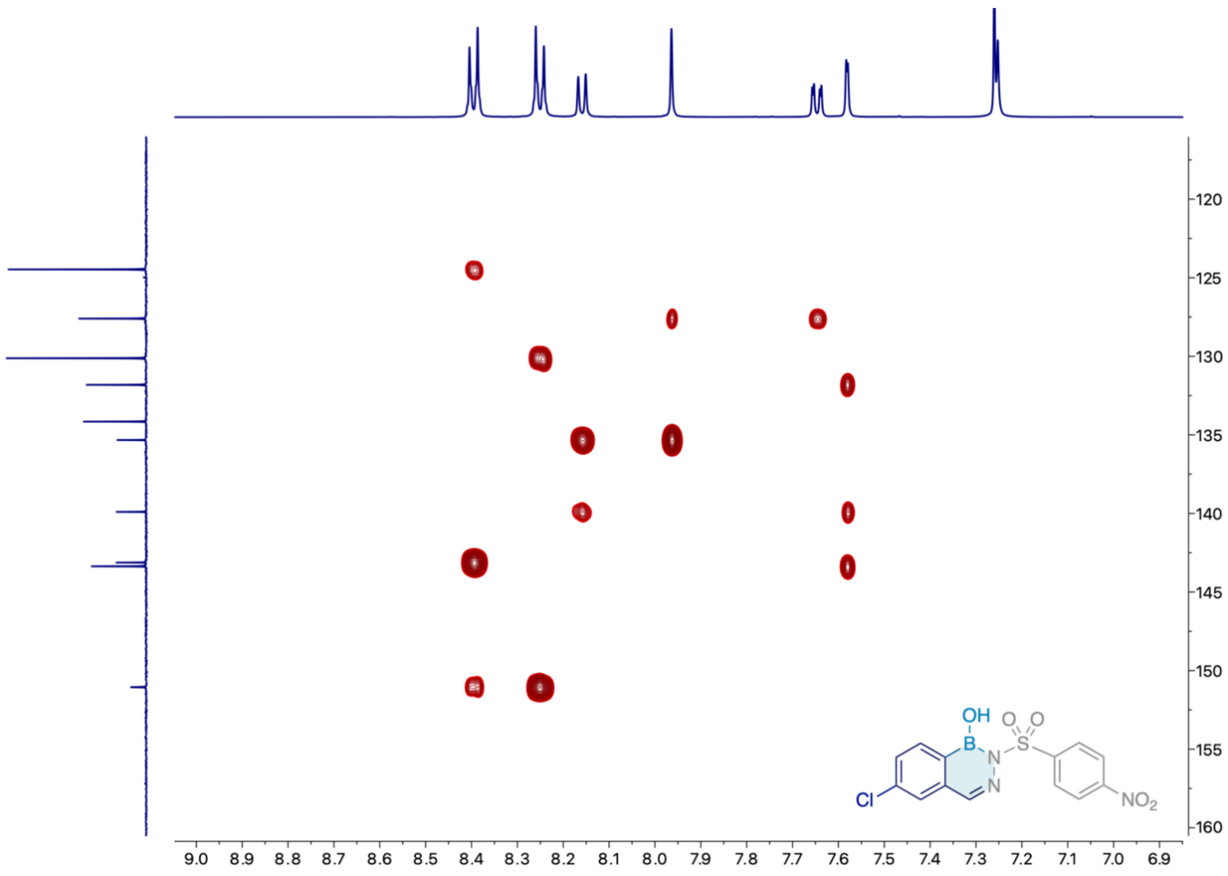

Figure S174. Diazaborine 32:^{1}H-^{13}C gHMBC NMR ($CDCl_3$, 298 K)

Diazaborine 33

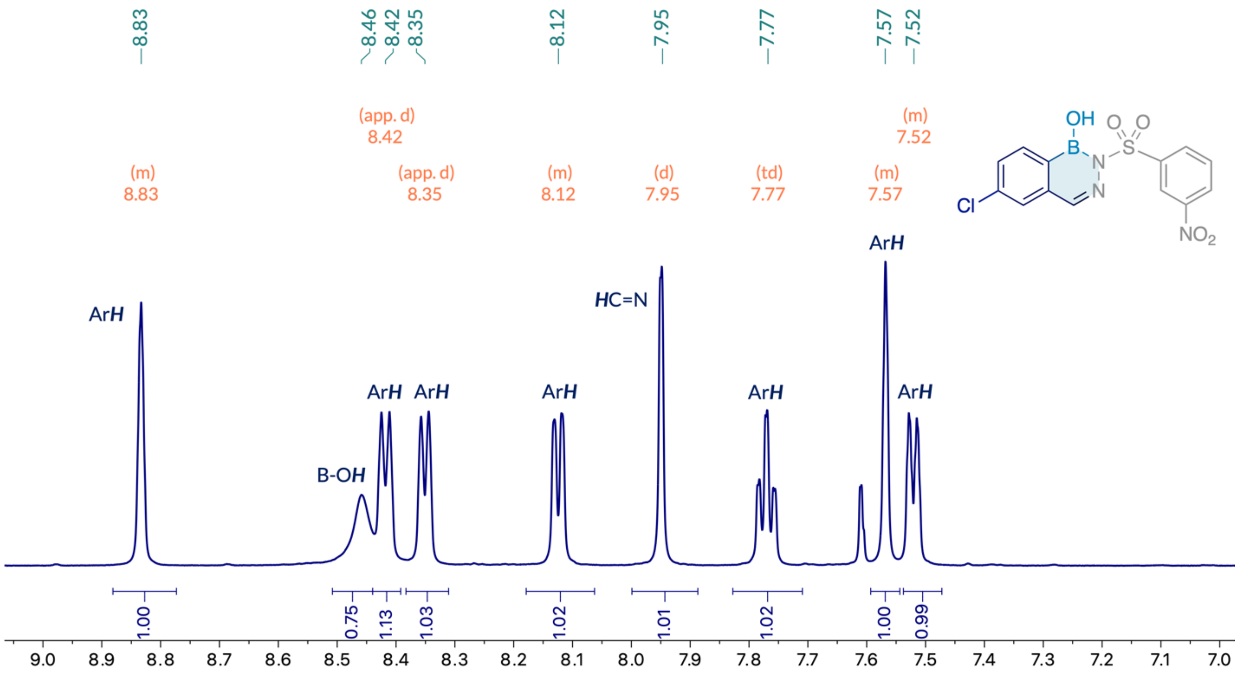

Figure S175. Diazaborine 33:^{1}H NMR (600 MHz, DMSO-d_6:$CDCl_3$ 1:1.2, 298 K)

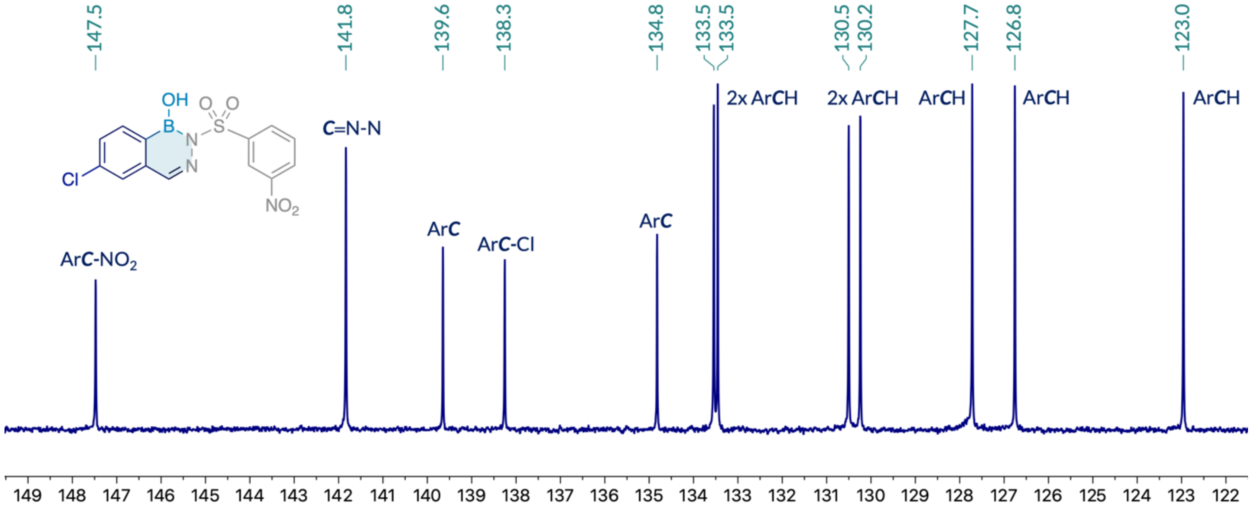

Figure S176. Diazaborine 33:^{13}C NMR (151 MHz, DMSO-d_6:$CDCl_3$ 1:1.2, 298 K)

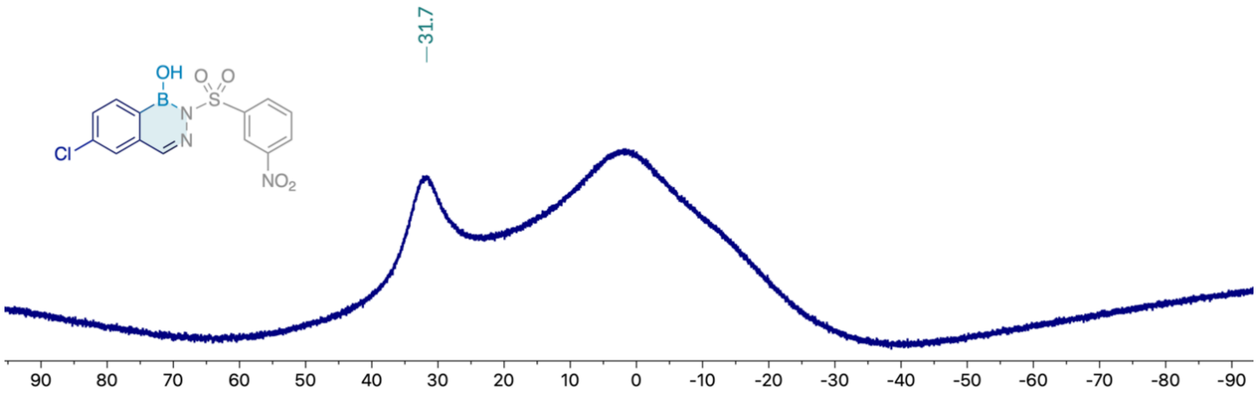

Figure S177. Diazaborine 33:^{11}B NMR (128 MHz, DMSO-d_6:$CDCl_3$ 1:1.2, 298 K)

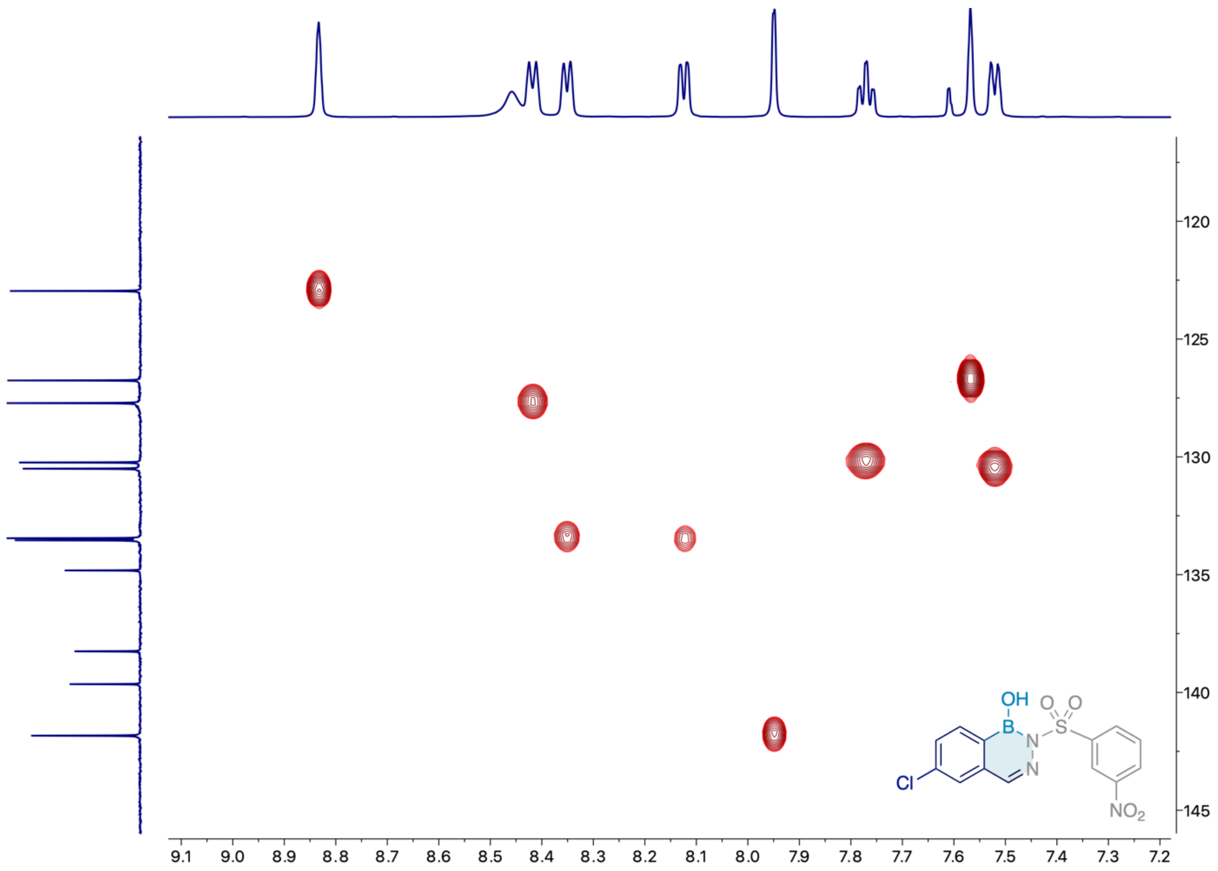

Figure S178. Diazaborine 33:^{1}H-^{13}C gHSQC NMR (DMSO-d_6:$CDCl_3$ 1:1.2, 298 K)

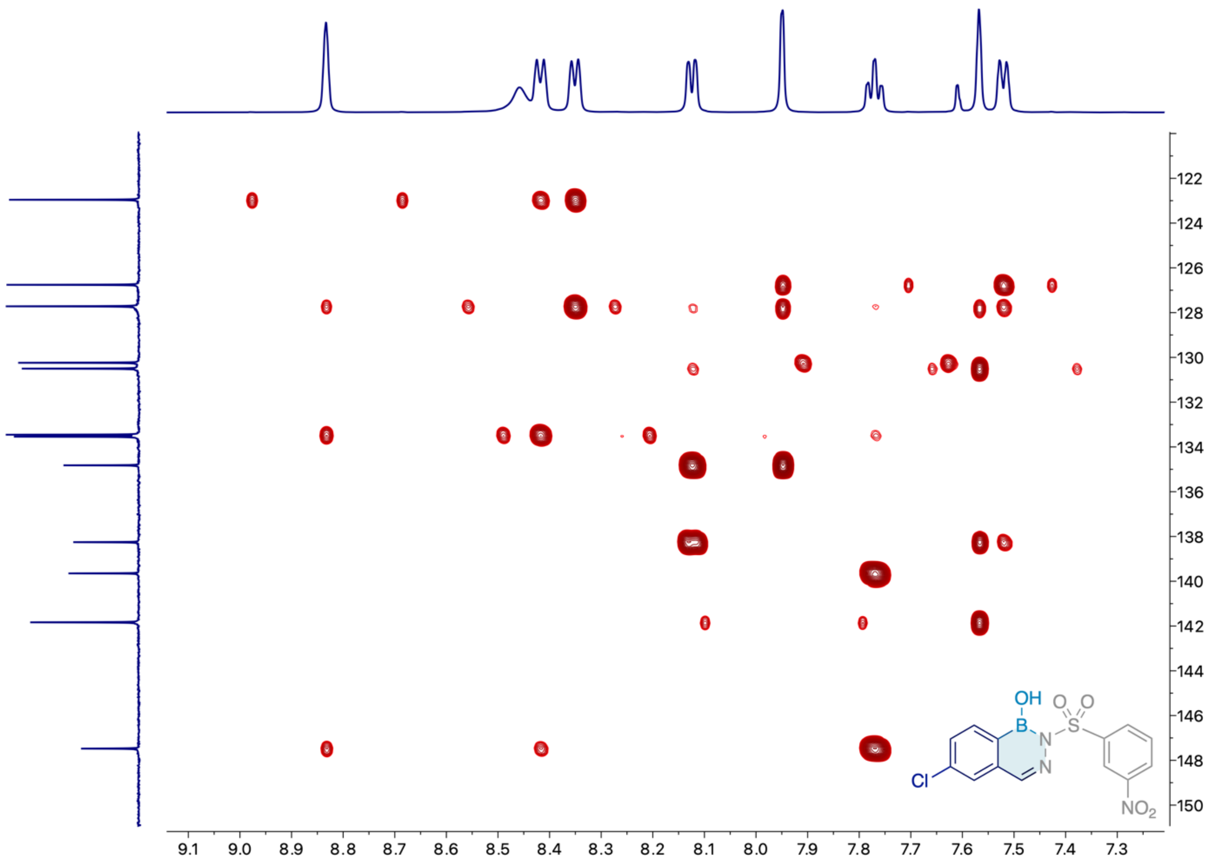

Figure S179. Diazaborine 33:^{1}H-^{13}C gHMBC NMR (DMSO-d_6:$CDCl_3$ 1:1.2, 298 K)

Diazaborine 34

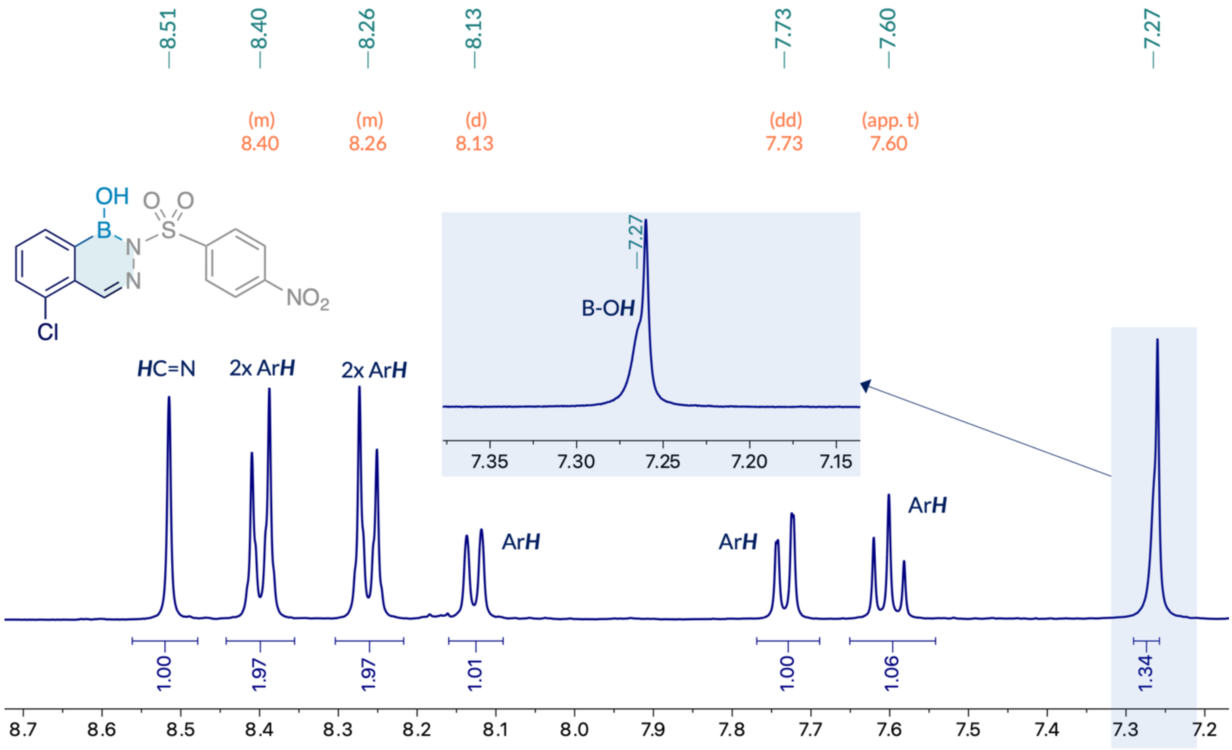

Figure S180. Diazaborine 34: ^{1}H NMR (400 MHz, $CDCl_3$, 298 K)

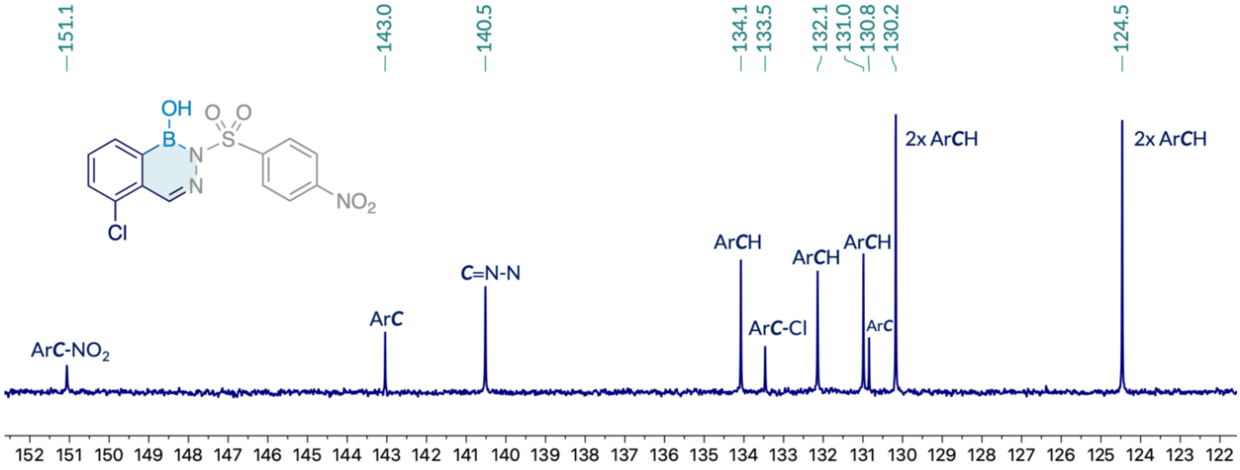

Figure S181. Diazaborine 34: ^{13}C NMR (101 MHz, $CDCl_3$, 298 K)

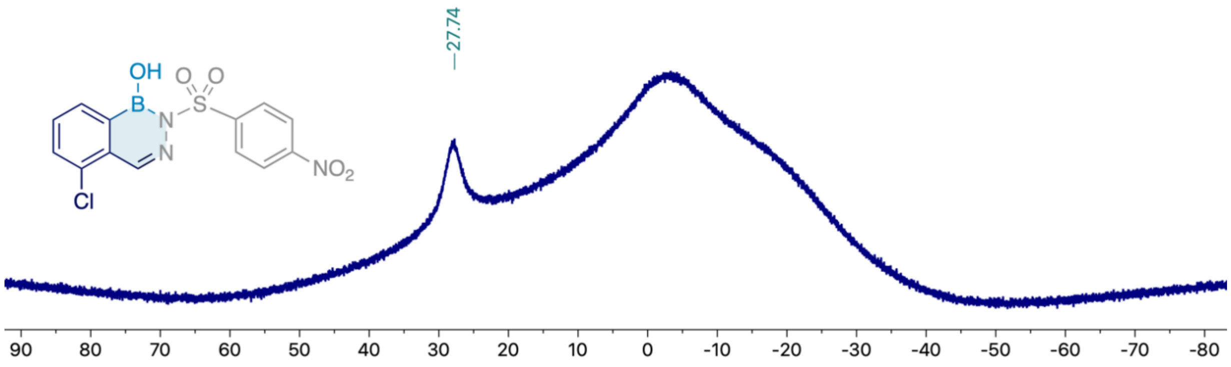

Figure S182. Diazaborine 34: ^{11}B NMR (128 MHz, $CDCl_3$, 298 K)

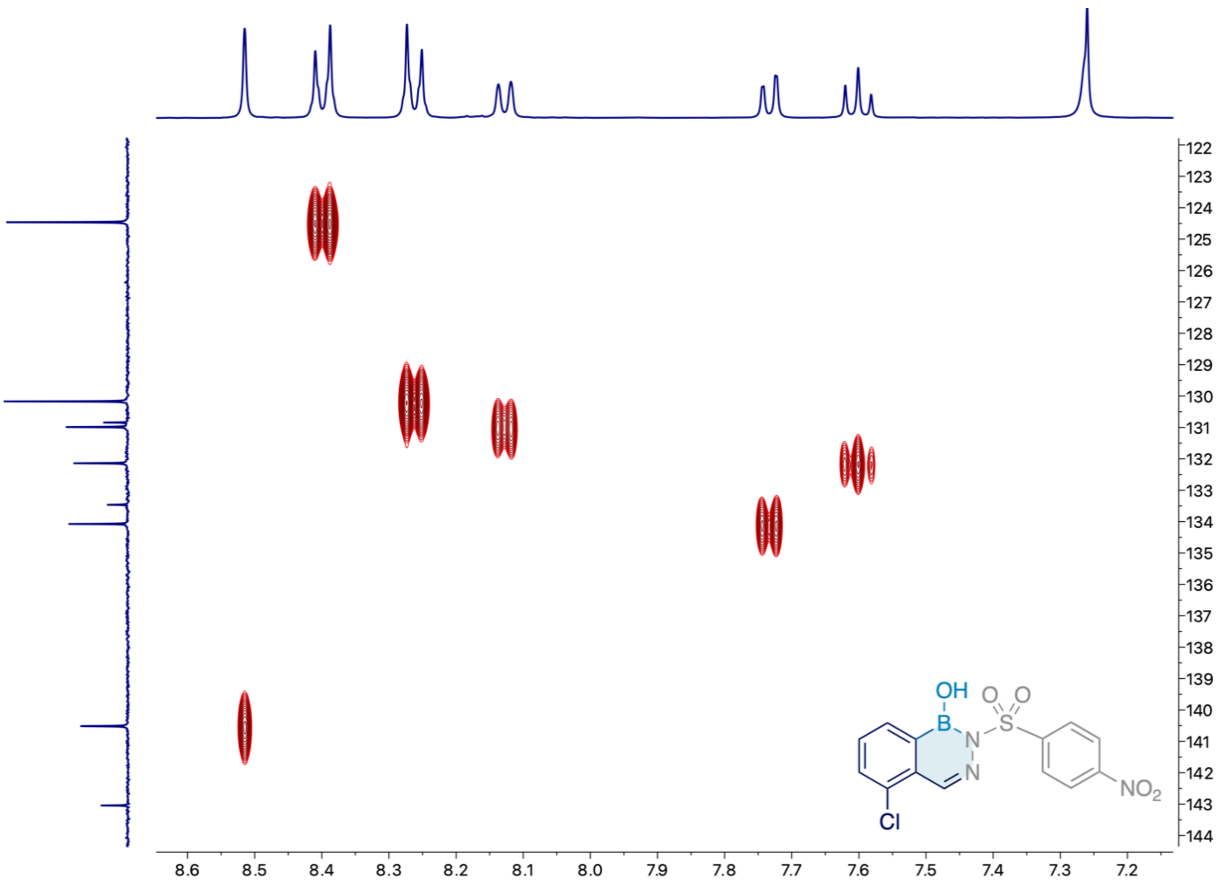

Figure S183. Diazaborine 34:^{1}H-^{13}C gHSQC NMR ($CDCl_3$, 298 K)

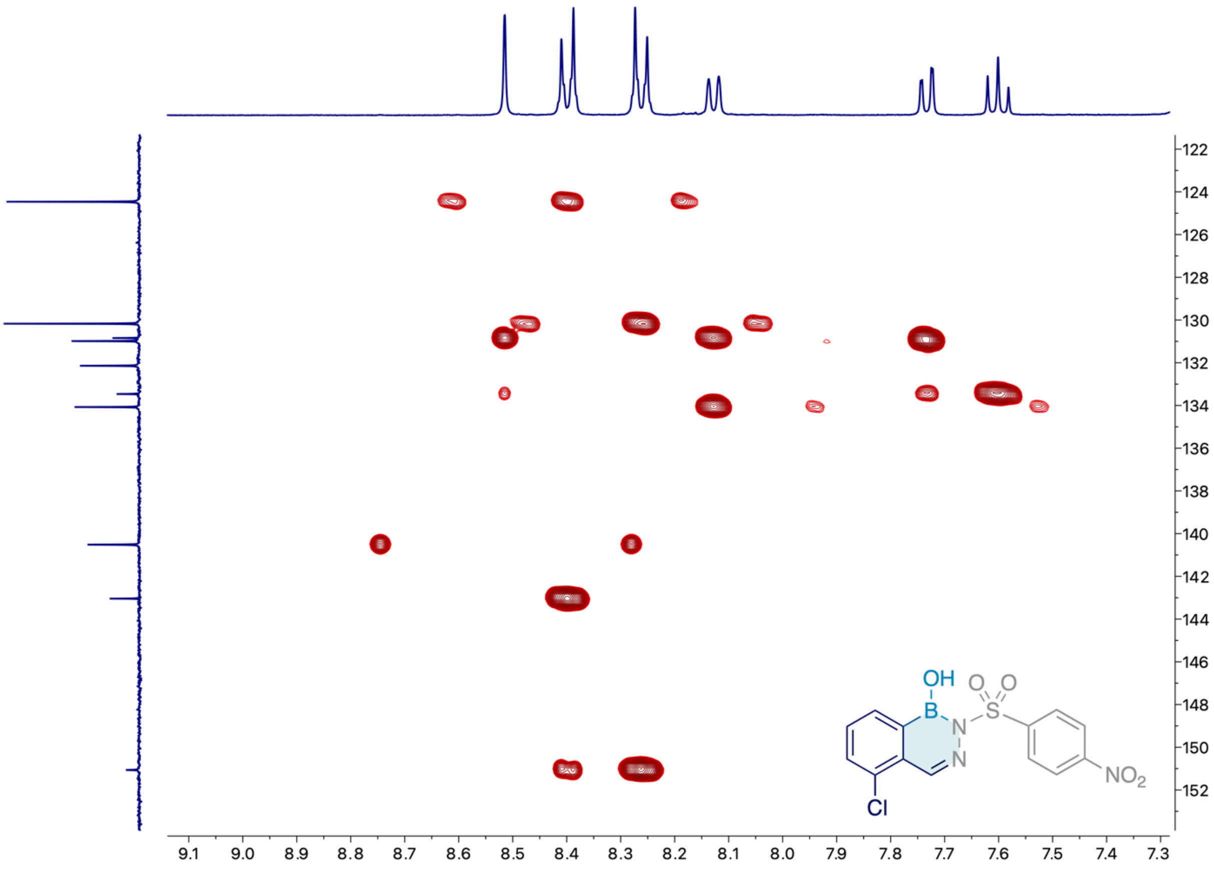

Figure S184. Diazaborine 34:^{1}H-^{13}C gHMBC NMR ($CDCl_3$, 298 K)

Diazaborine 35

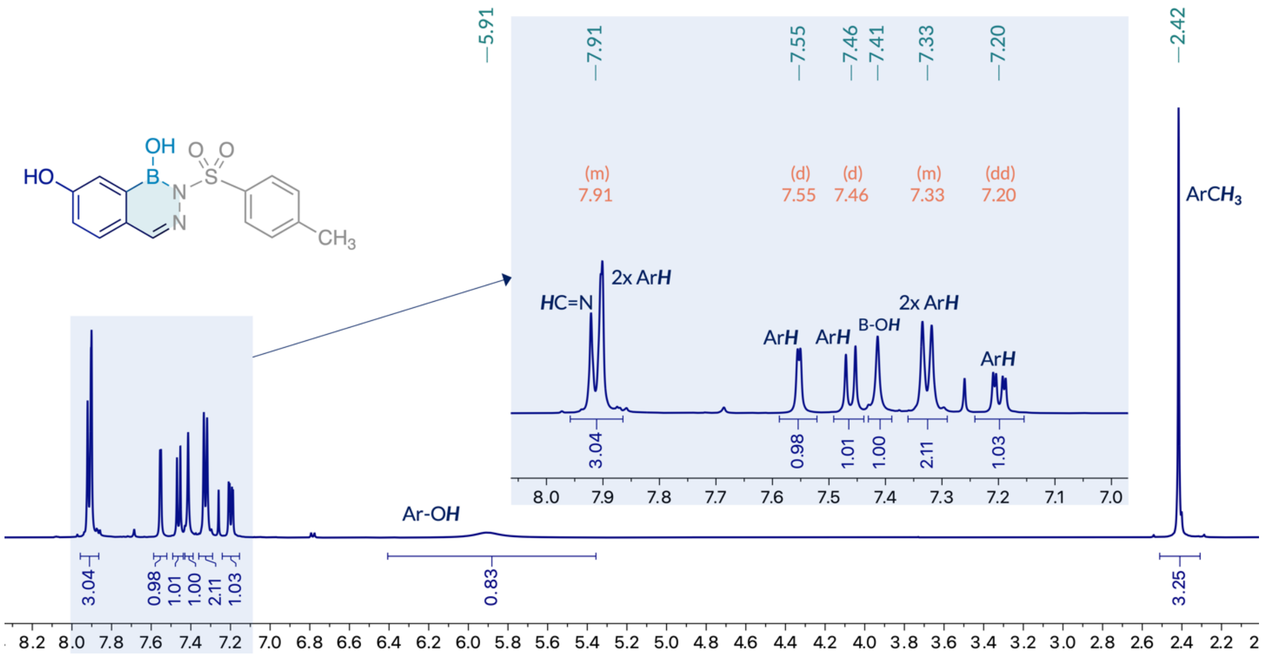

Figure S185. Diazaborine 35:^{1}H NMR (500 MHz, $CDCl_3$, 298 K)

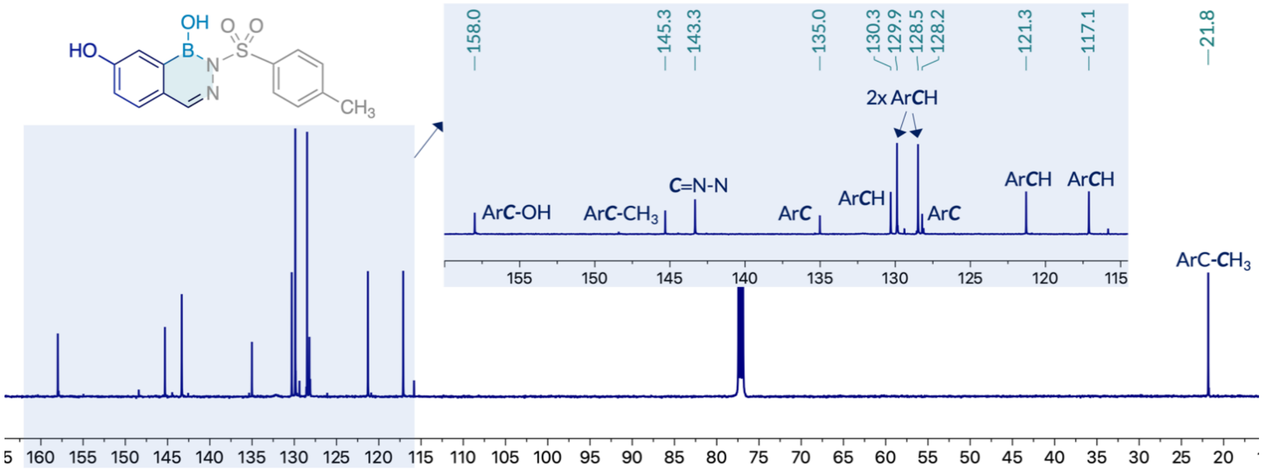

Figure S186. Diazaborine 35:^{13}C NMR (126 MHz, $CDCl_3$, 298 K)

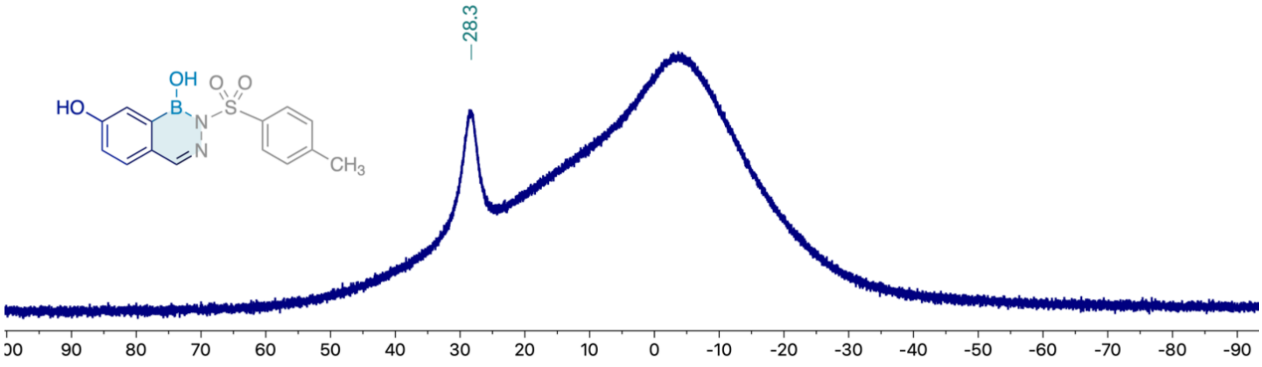

Figure S187. Diazaborine 35:^{11}B NMR (160 MHz, $CDCl_3$, 298 K)

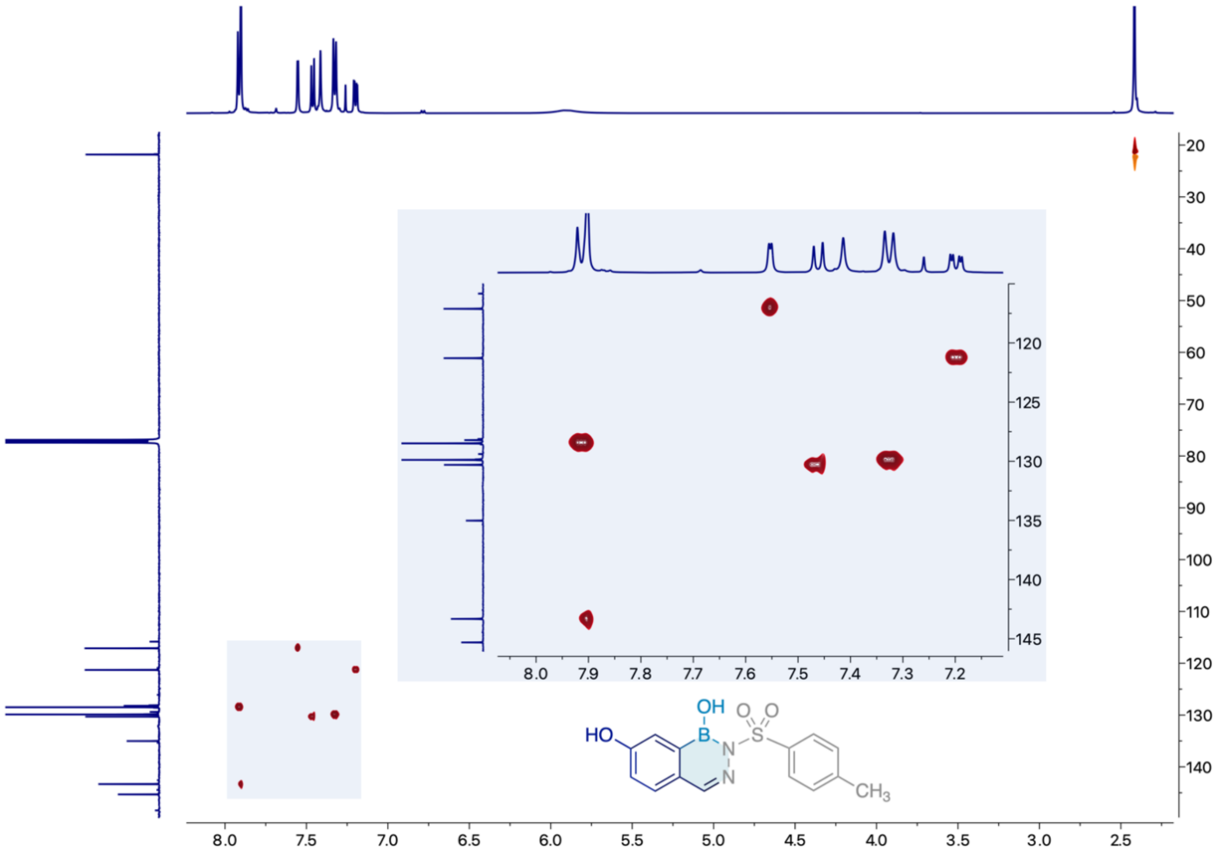

Figure S188. Diazaborine 35:^{1}H-^{13}C gHSQC NMR ($CDCl_3$, 298 K)

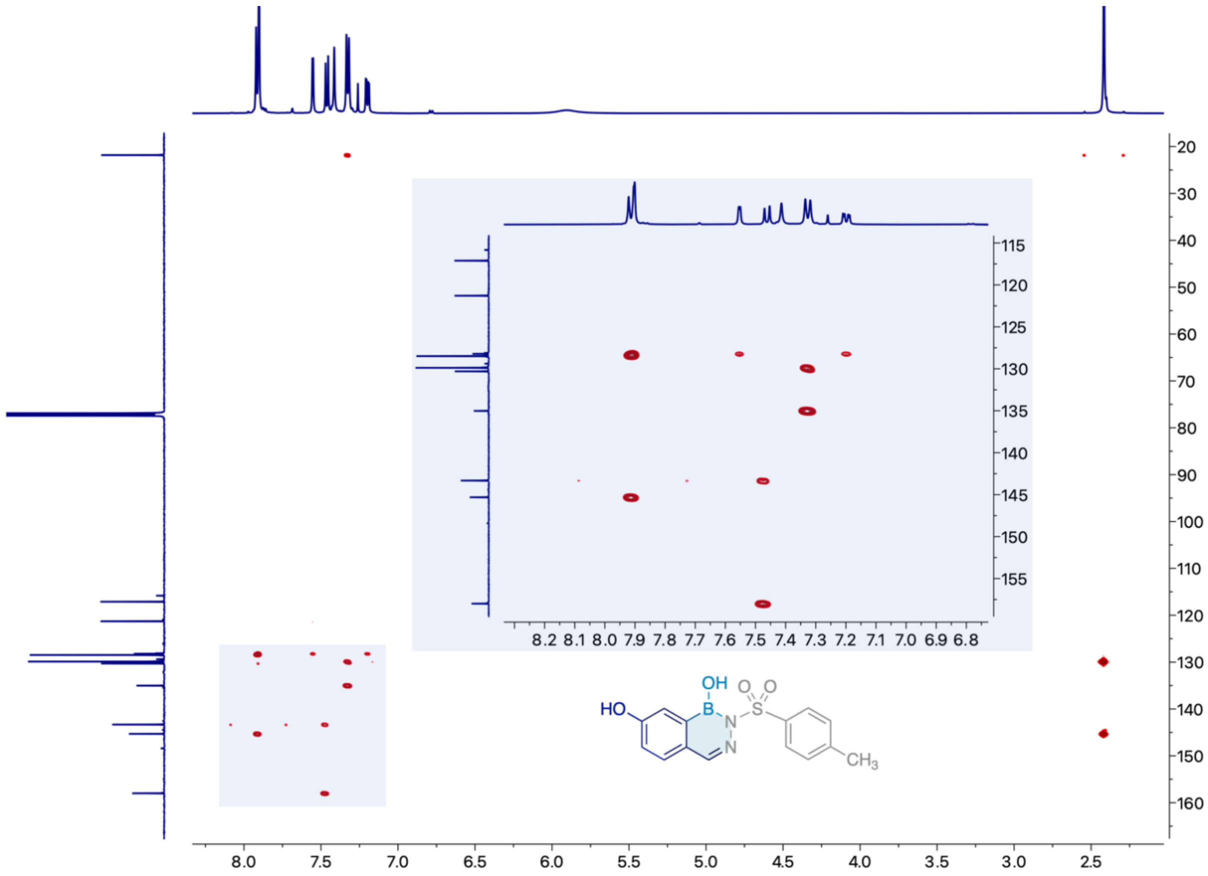

Figure S189. Diazaborine 35:^{1}H-^{13}C gHMBC NMR ($CDCl_3$, 298 K)

Diazaborine 36

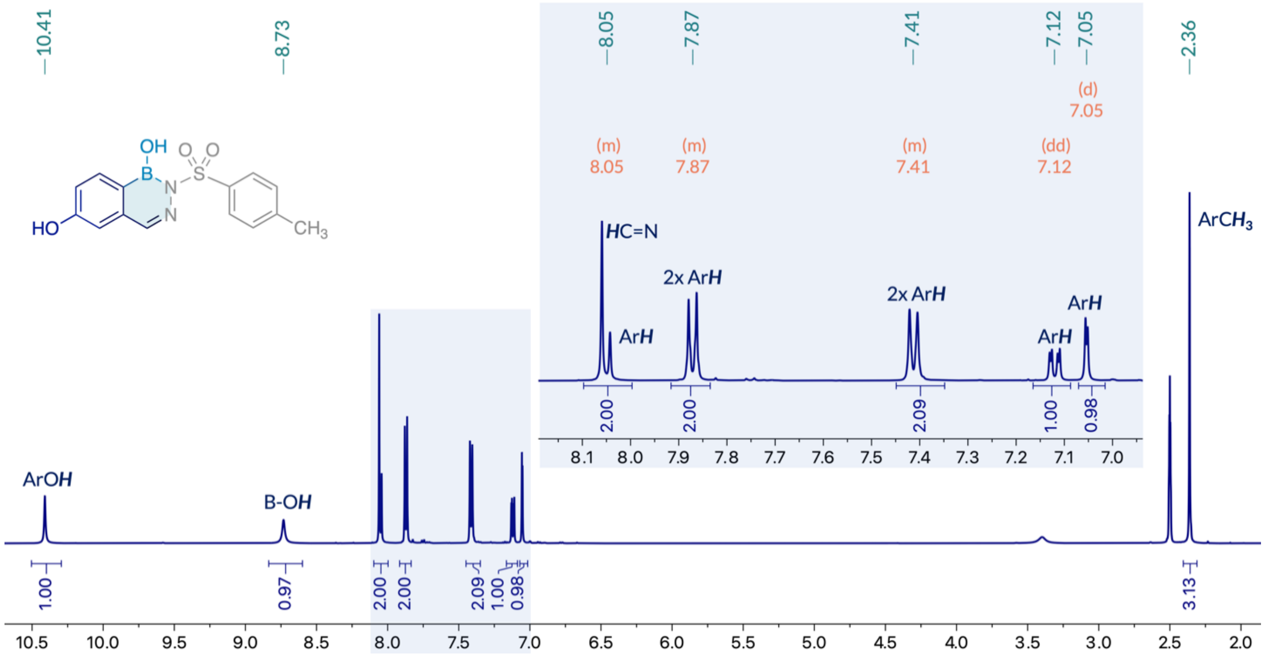

Figure S190. Diazaborine 36: ^{1}H NMR (500 MHz, DMSO-d_6, 298 K)

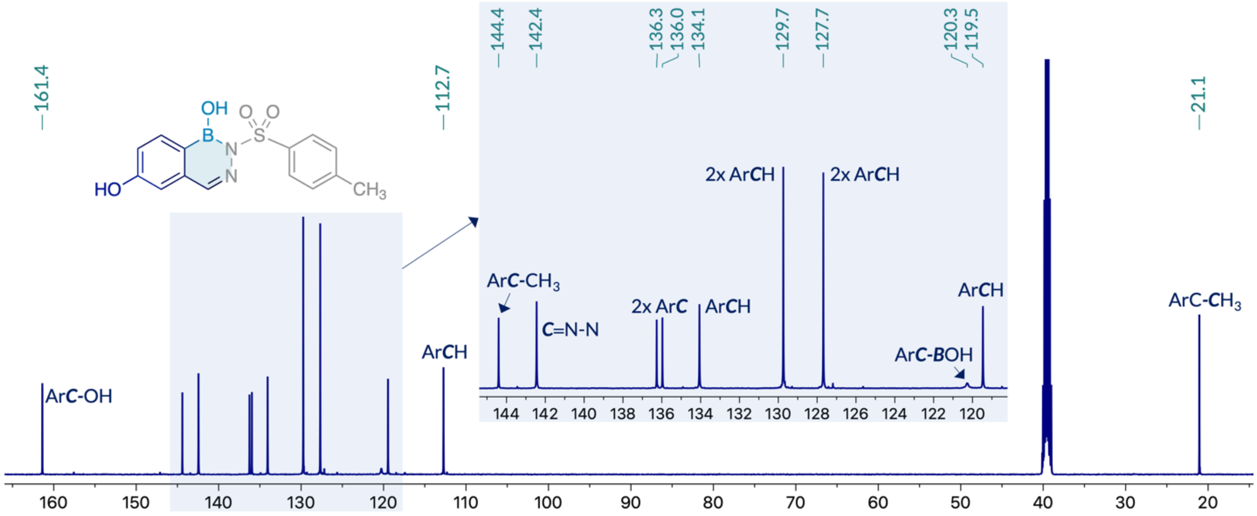

Figure S191. Diazaborine 36: ^{13}C NMR (126 MHz, DMSO-d_6, 298 K)

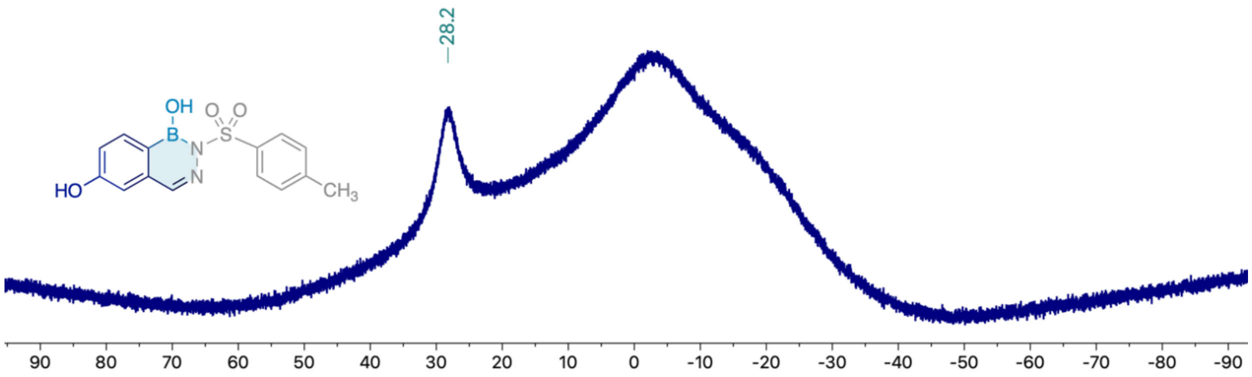

Figure S192. Diazaborine 36: ^{11}B NMR (128 MHz, $CDCl_3$, 298 K)

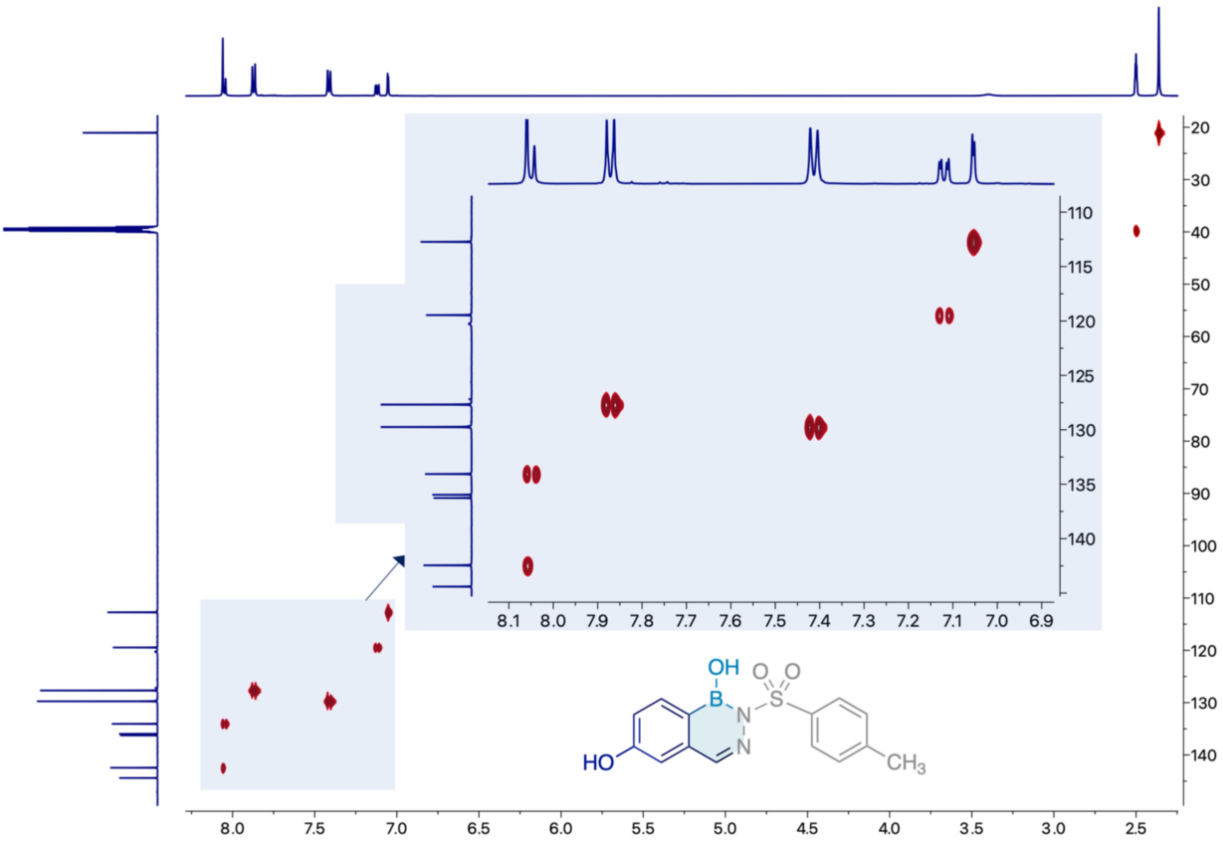

Figure S193. Diazaborine 36:^{1}H-^{13}C gHSQC NMR (DMSO-d_6, 298 K)

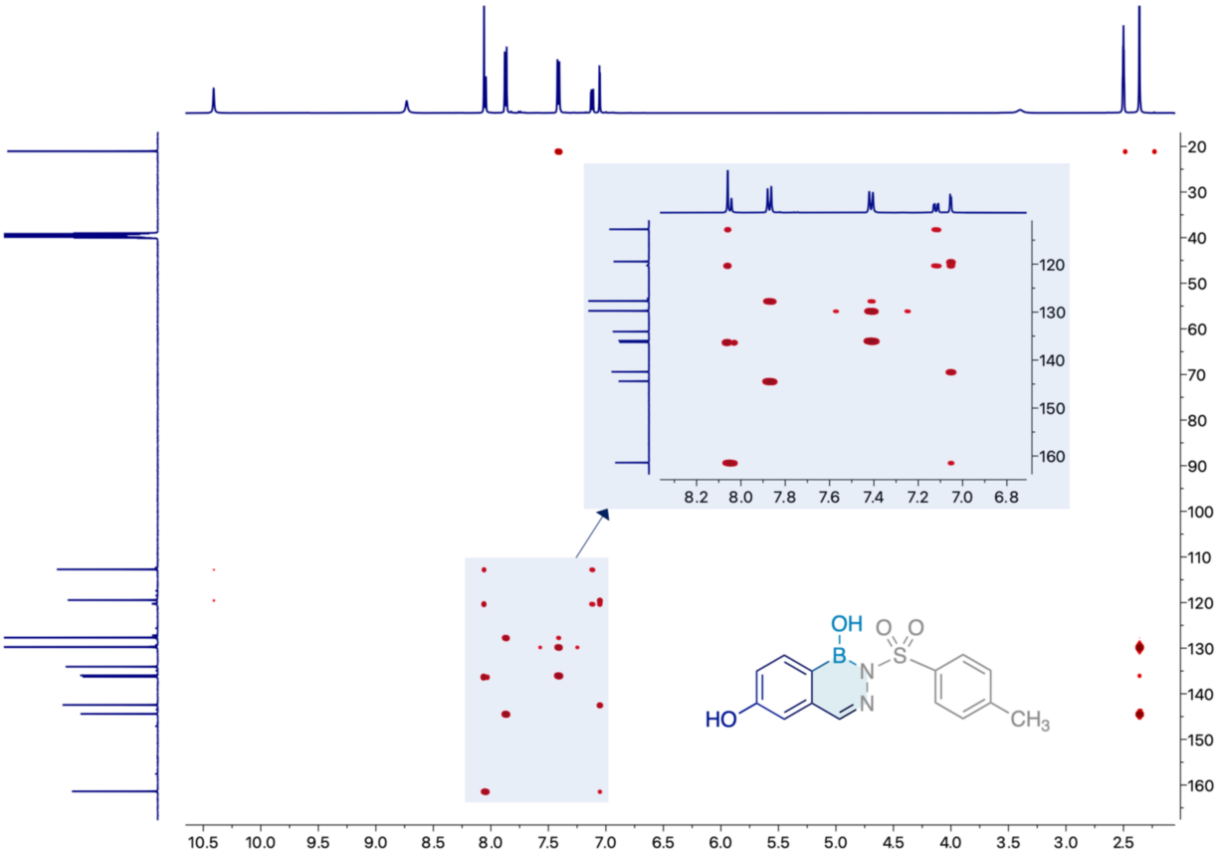

Figure S194. Diazaborine 36:^{1}H-^{13}C gHMBC NMR (DMSO-d_6, 298 K)

Diazaborine 37

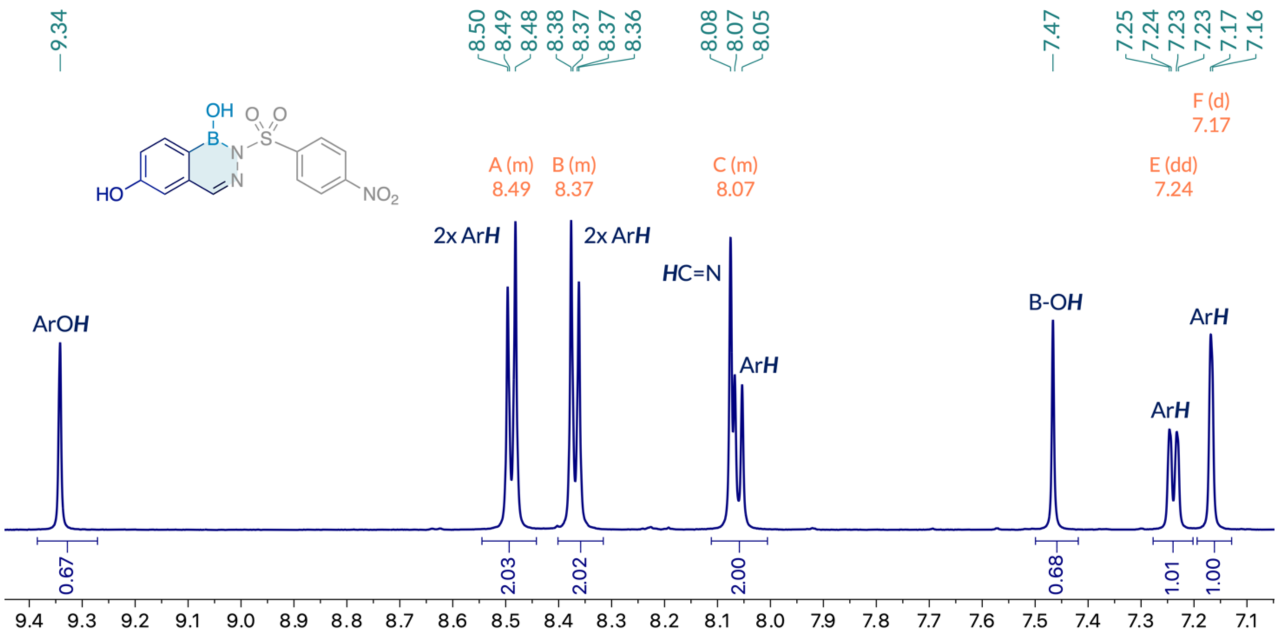

Figure S195. Diazaborine 37:^{1}H NMR (600 MHz, Acetone-d_6, 298 K)

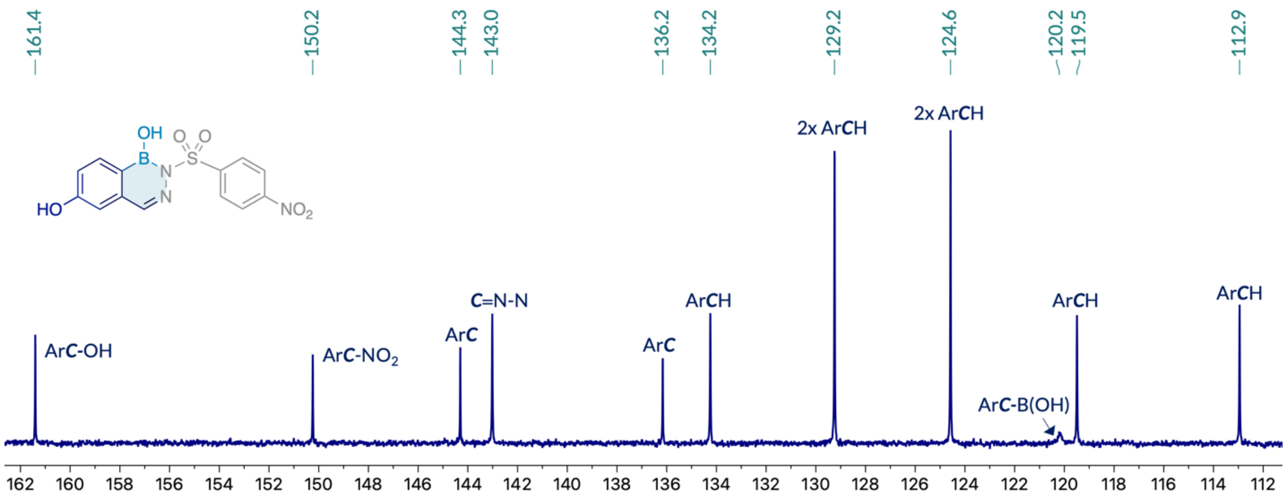

Figure S196. Diazaborine 37:^{13}C NMR (126 MHz, DMSO-d_6, 298 K)

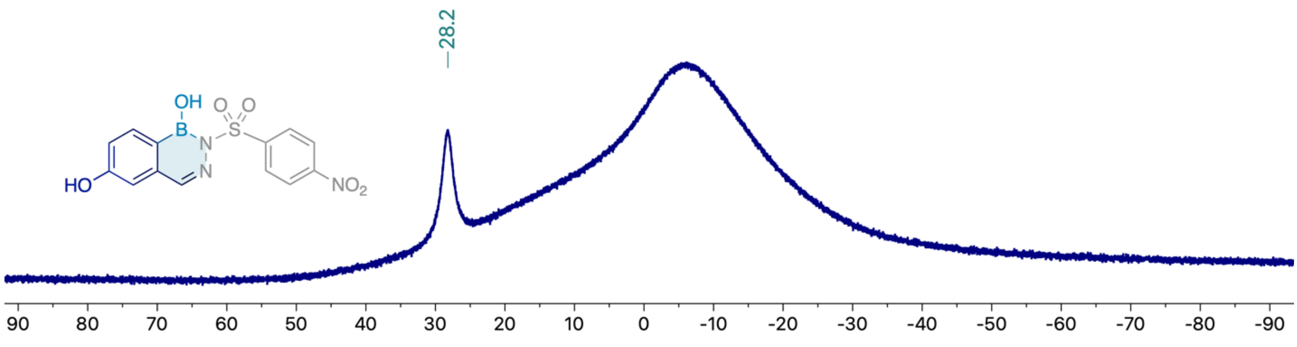

Figure S197. Diazaborine 37:^{11}B NMR (160 MHz, Acetone-d_6, 298 K)

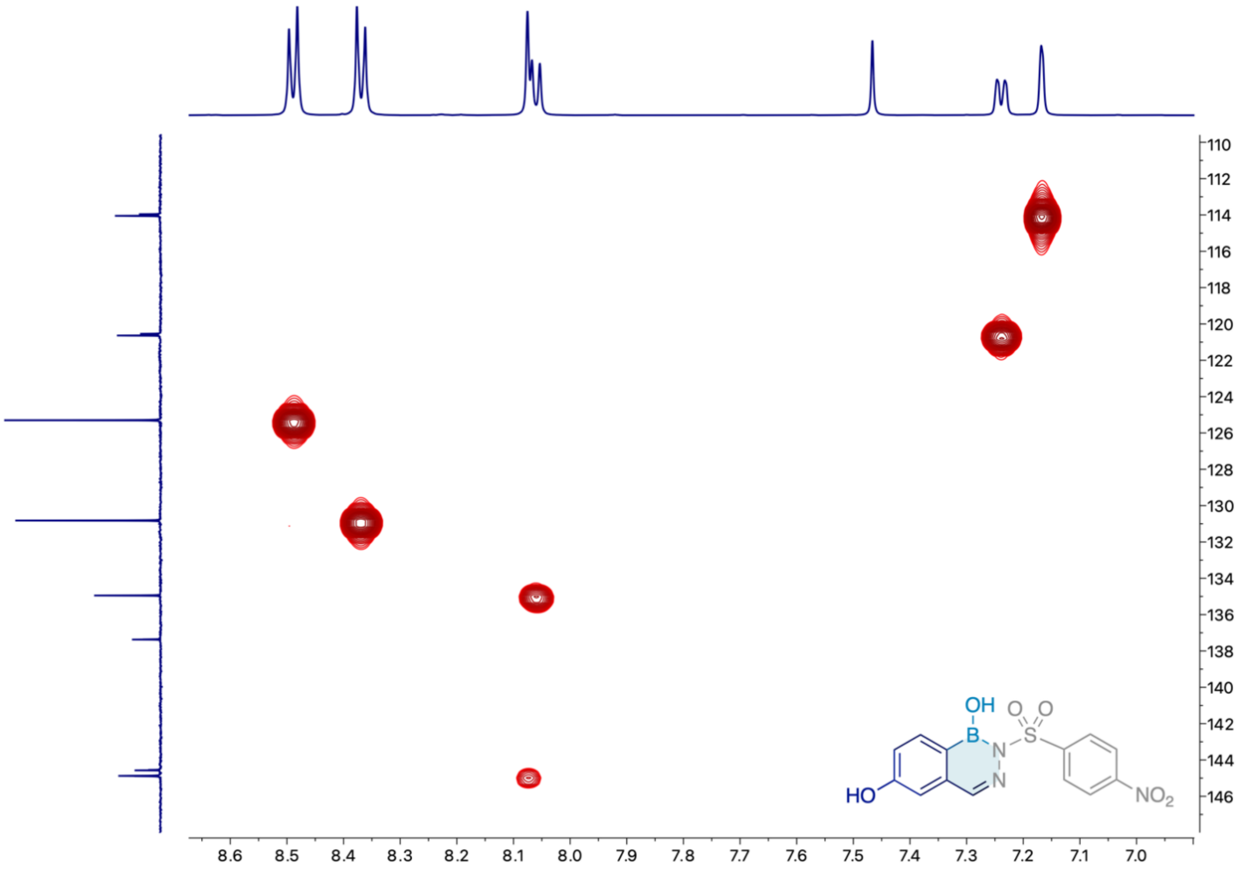

Figure S198. Diazaborine 37: ^{1}H-^{13}C gHSQC NMR (DMSO-d_6, 298 K)

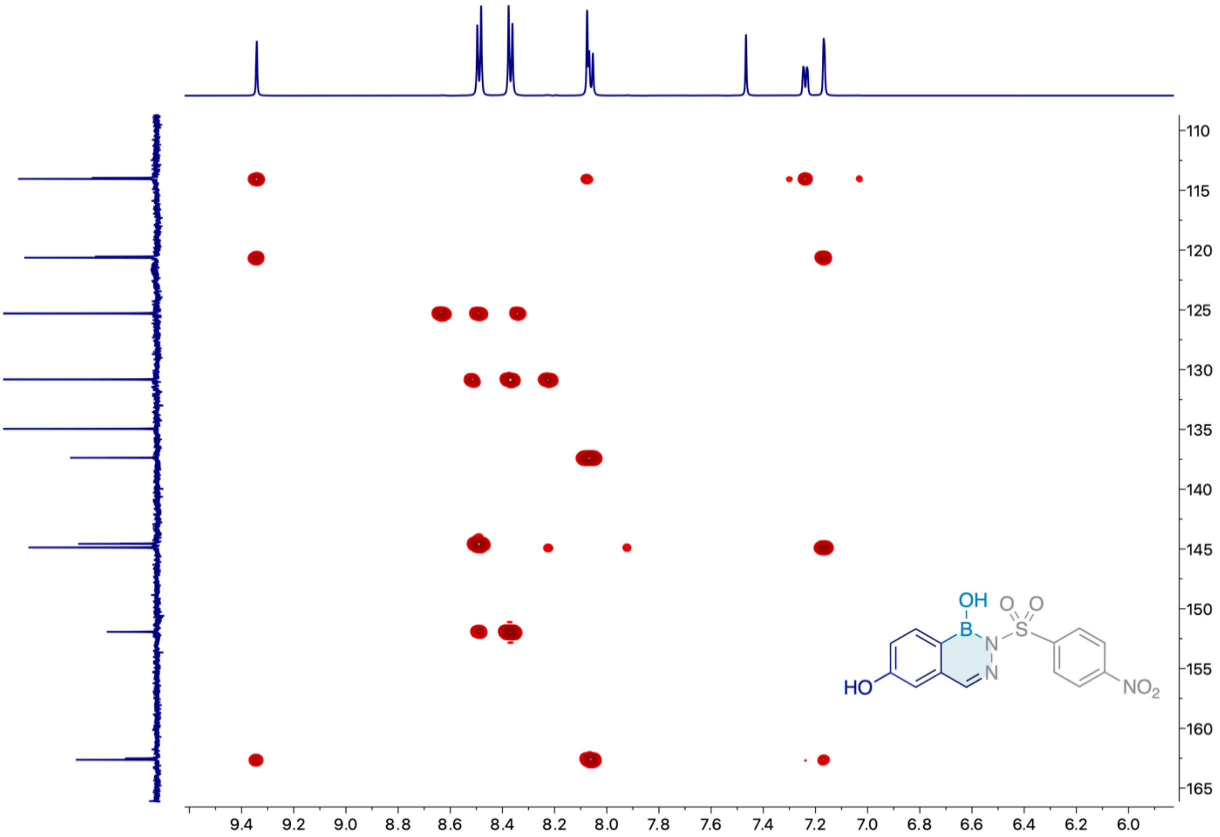

Figure S199. Diazaborine 37: ^{1}H-^{13}C gHMBC NMR (DMSO-d_6, 298 K)

Diazaborine 38

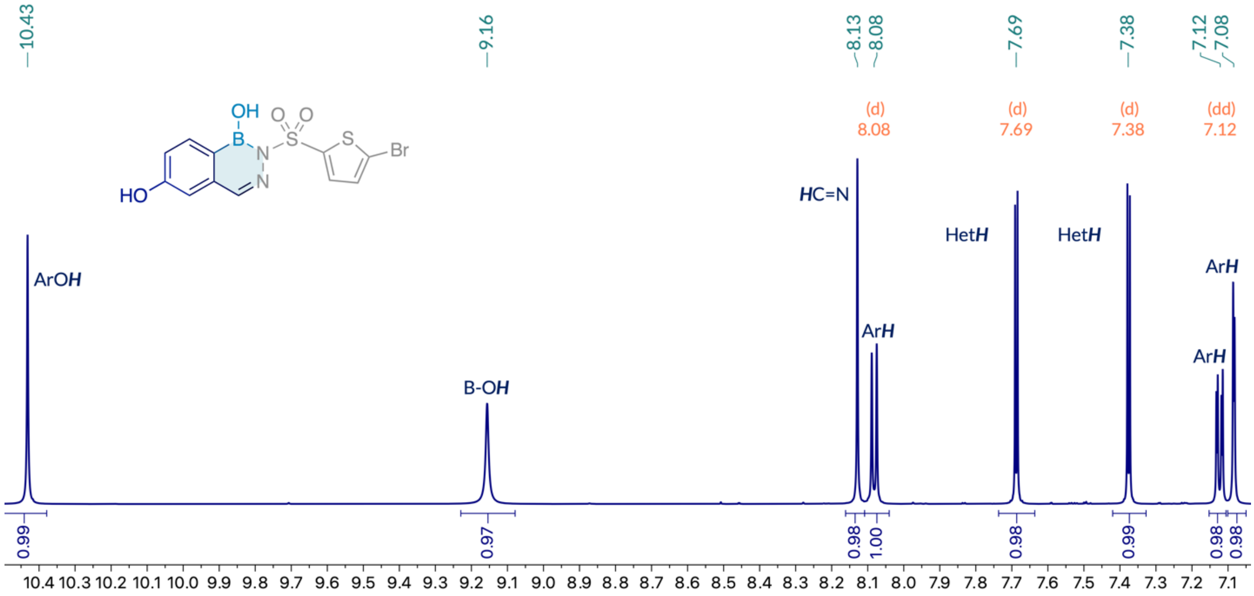

Figure S200. Diazaborine 38:^{1}H NMR (600 MHz, DMSO-d_6, 298 K)

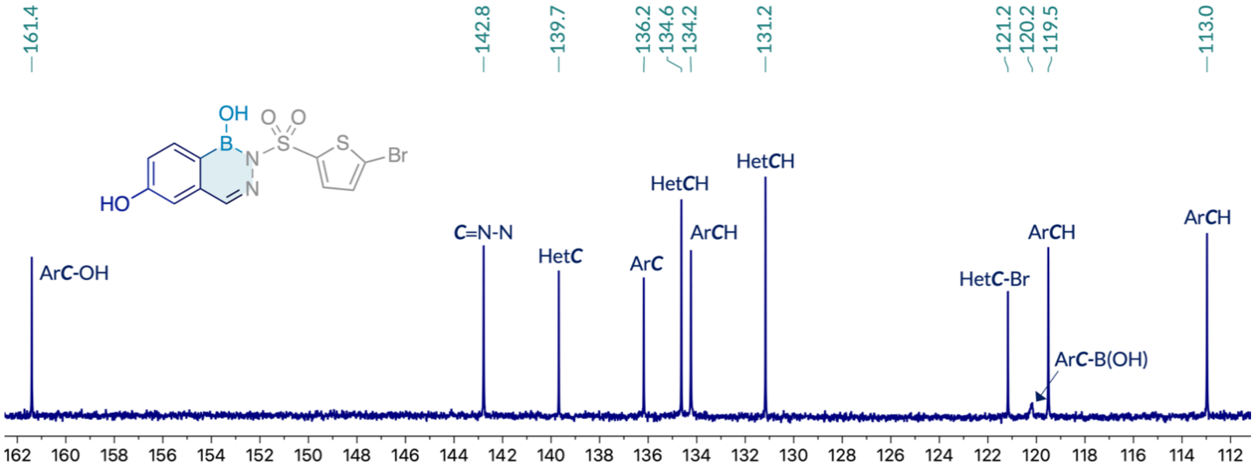

Figure S201. Diazaborine 38:^{13}C NMR (151 MHz, DMSO-d_6, 298 K)

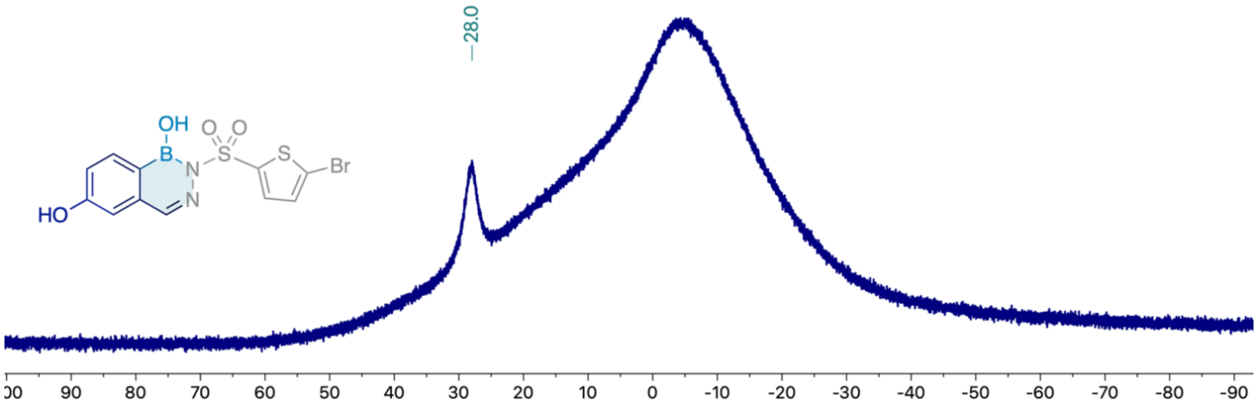

Figure S202. Diazaborine 38:^{11}B NMR (160 MHz, DMSO-d_6, 298 K)

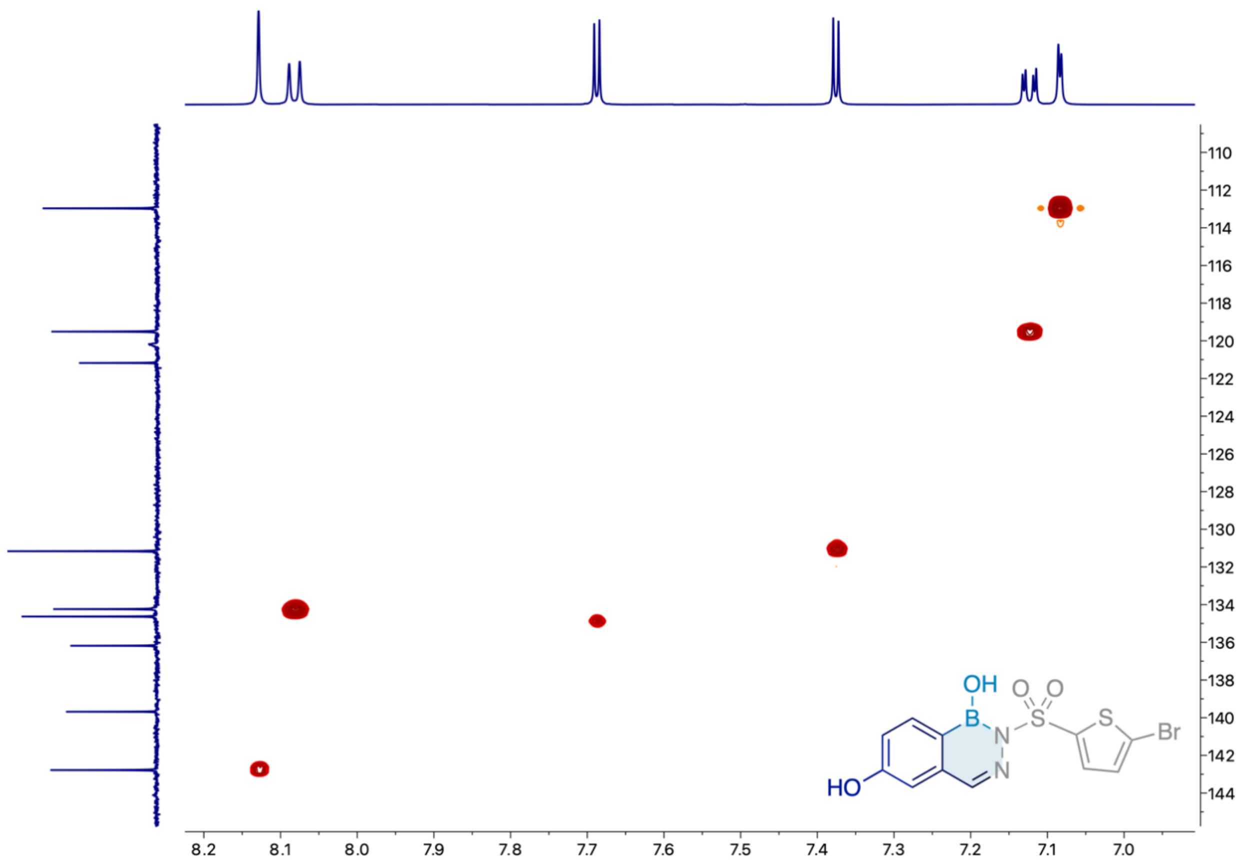

Figure S203. Diazaborine 38: ^{1}H-^{13}C gHSQC NMR (DMSO-d_6, 298 K)

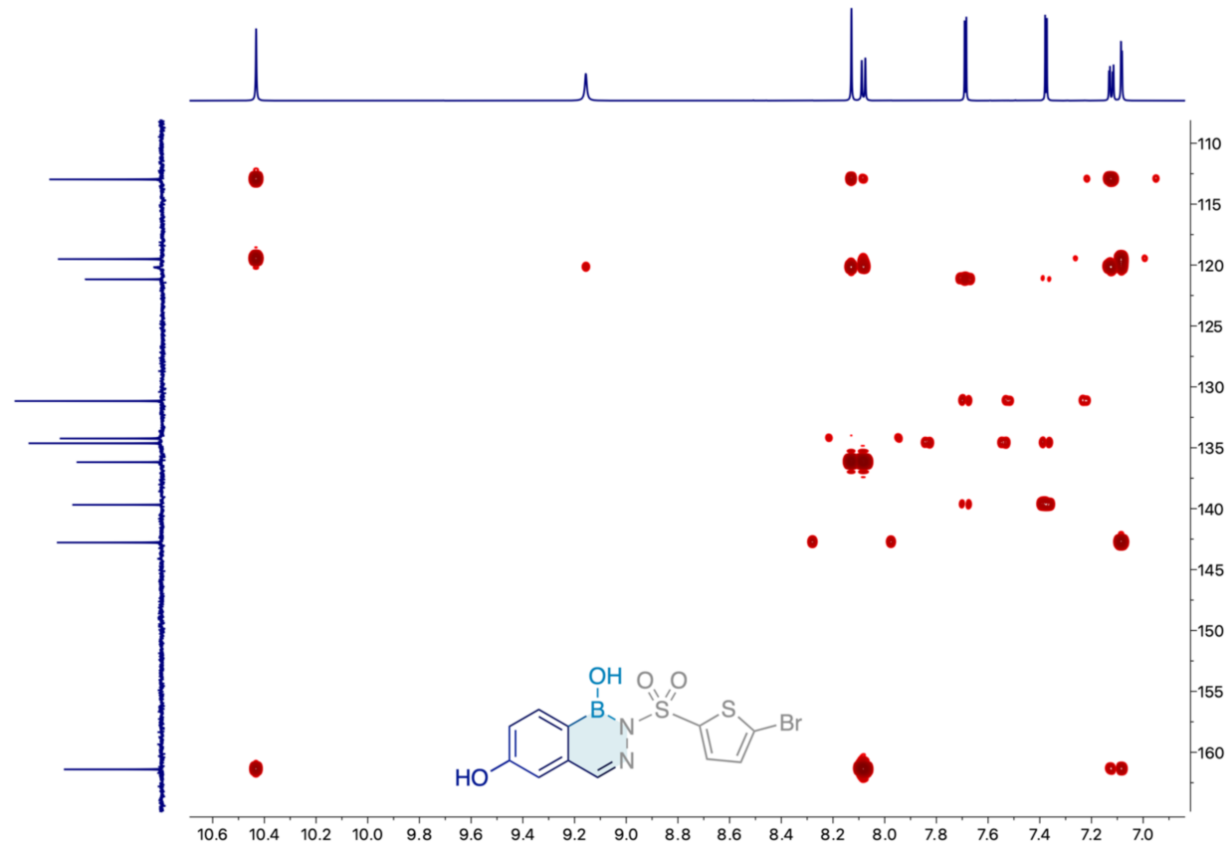

Figure S204. Diazaborine 38: ^{1}H-^{13}C gHMBC NMR (DMSO-d_6, 298 K)

Diazaborine 39

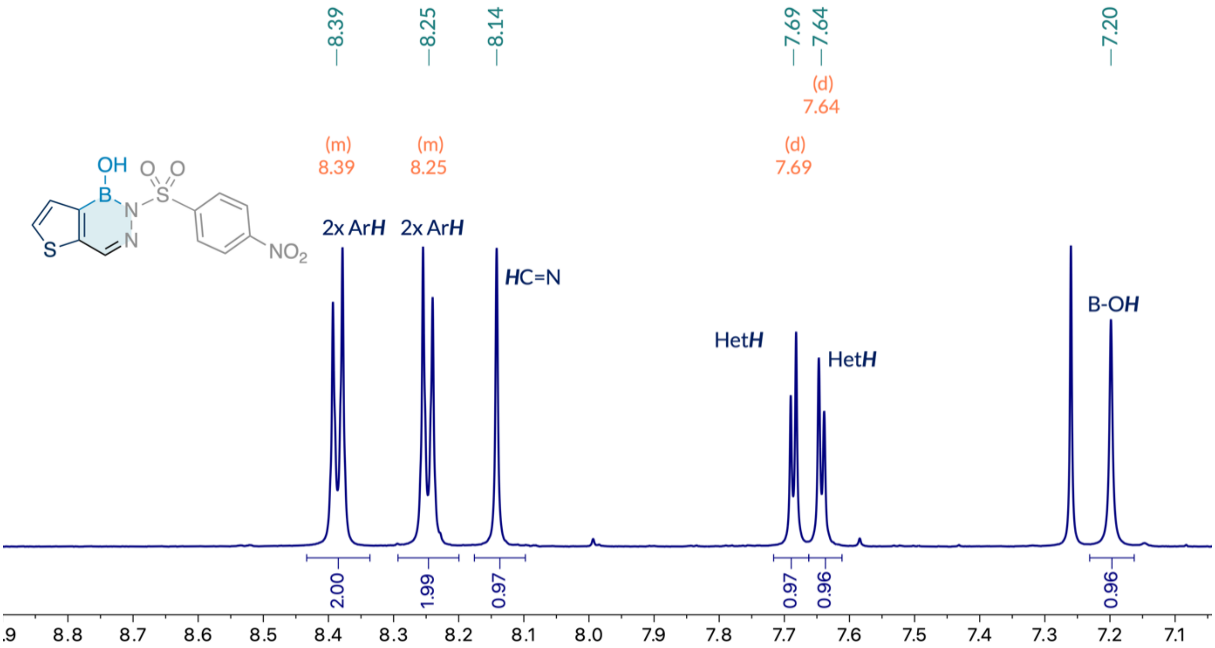

Figure S205. Diazaborine 39:^{1}H NMR (600 MHz, $CDCl_3$, 298 K)

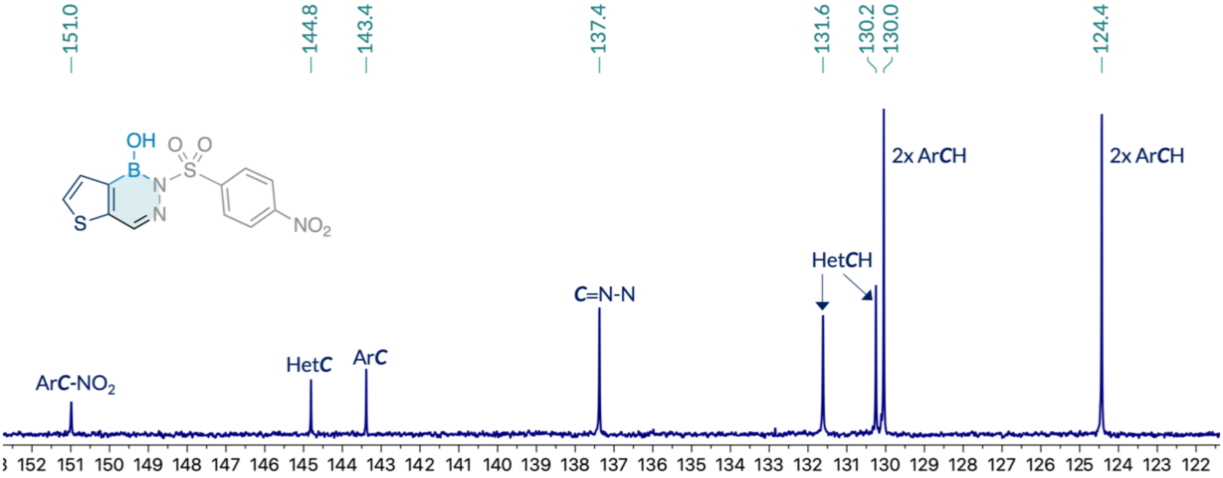

Figure S206. Diazaborine 39:^{13}C NMR (101 MHz, $CDCl_3$, 298 K)

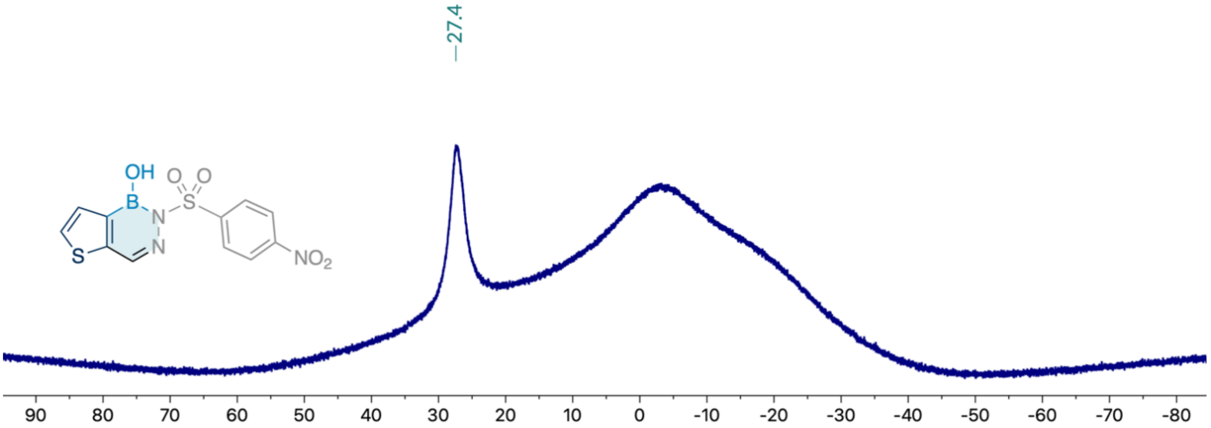

Figure S207. Diazaborine 39:^{11}B NMR (128 MHz, $CDCl_3$, 298 K)

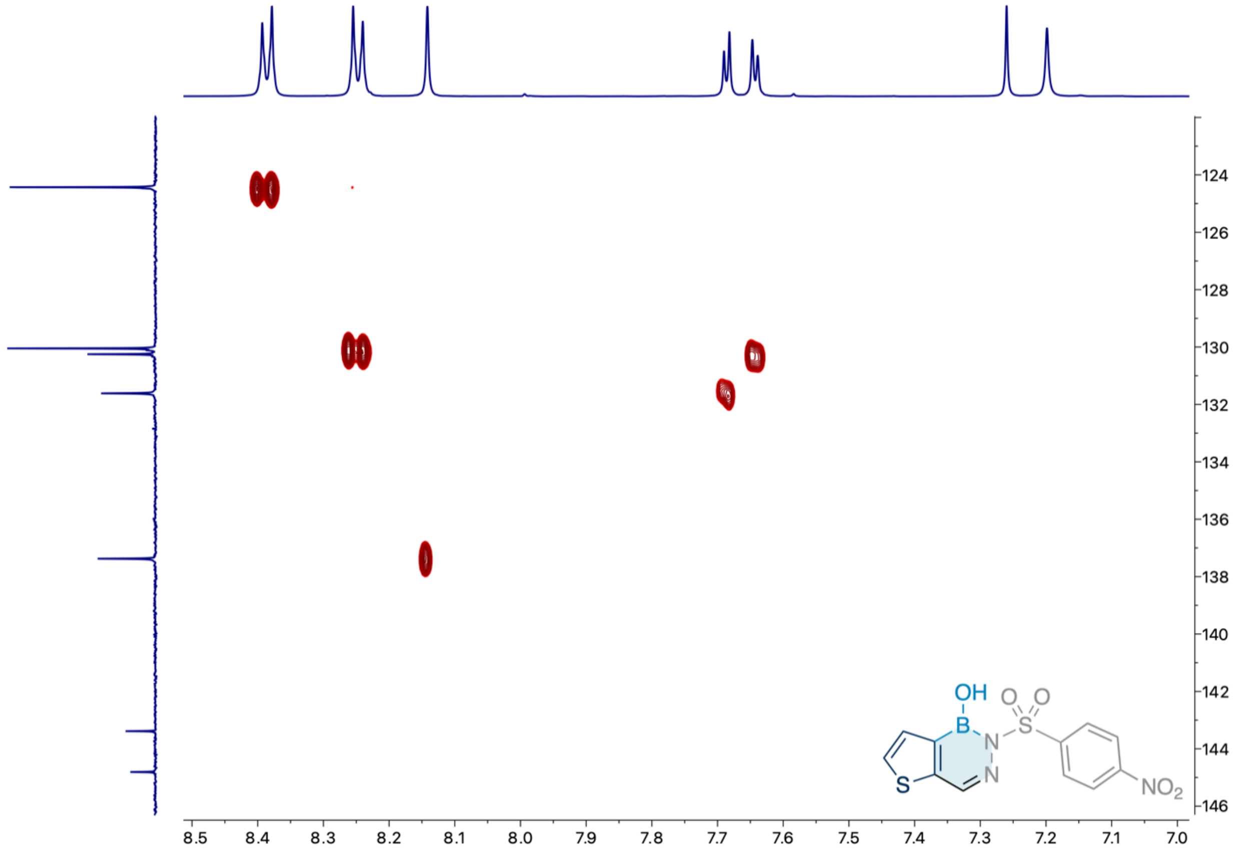

Figure S208. Diazaborine 39: ^{1}H-^{13}C gHSQC NMR ($CDCl_3$, 298 K)

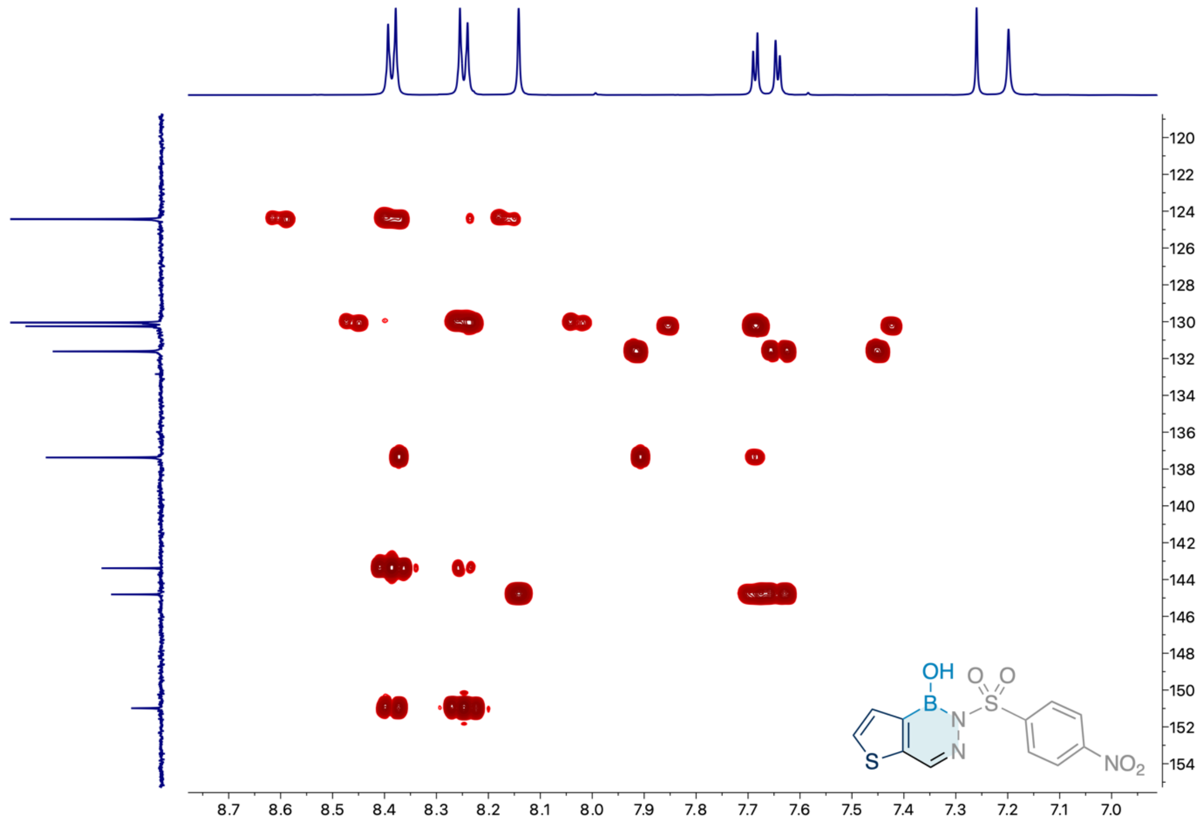

Figure S209. Diazaborine 39: ^{1}H-^{13}C gHMBC NMR ($CDCl_3$, 298 K)

Diazaborine 40

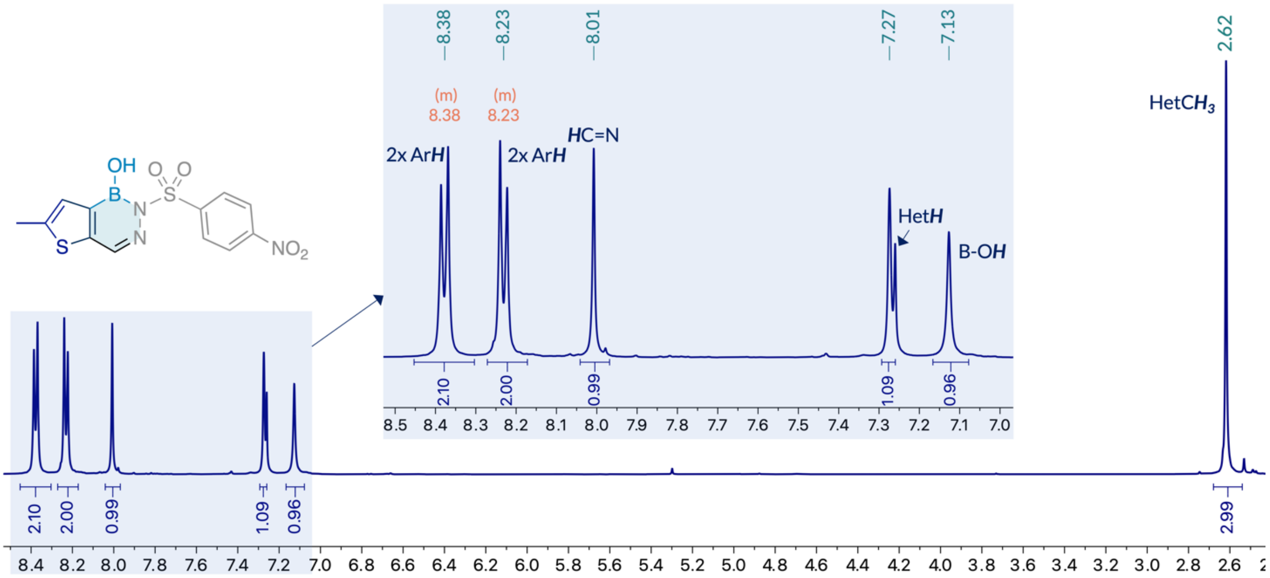

Figure S210. Diazaborine 40:^{1}H NMR (500 MHz, $CDCl_3$, 298 K)

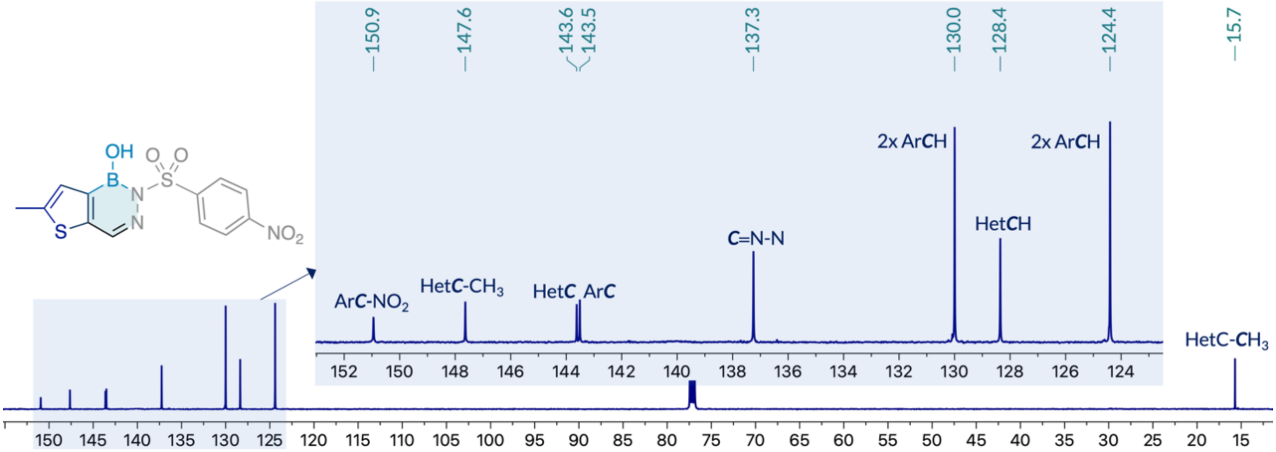

Figure S211. Diazaborine 40:^{13}C NMR (126 MHz, $CDCl_3$, 298 K)

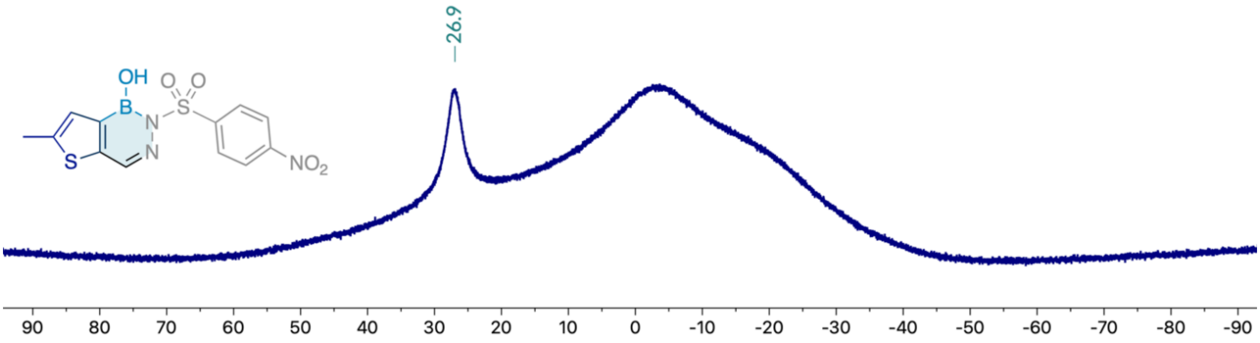

Figure S212. Diazaborine 40:^{11}B NMR (128 MHz, $CDCl_3$, 298 K)

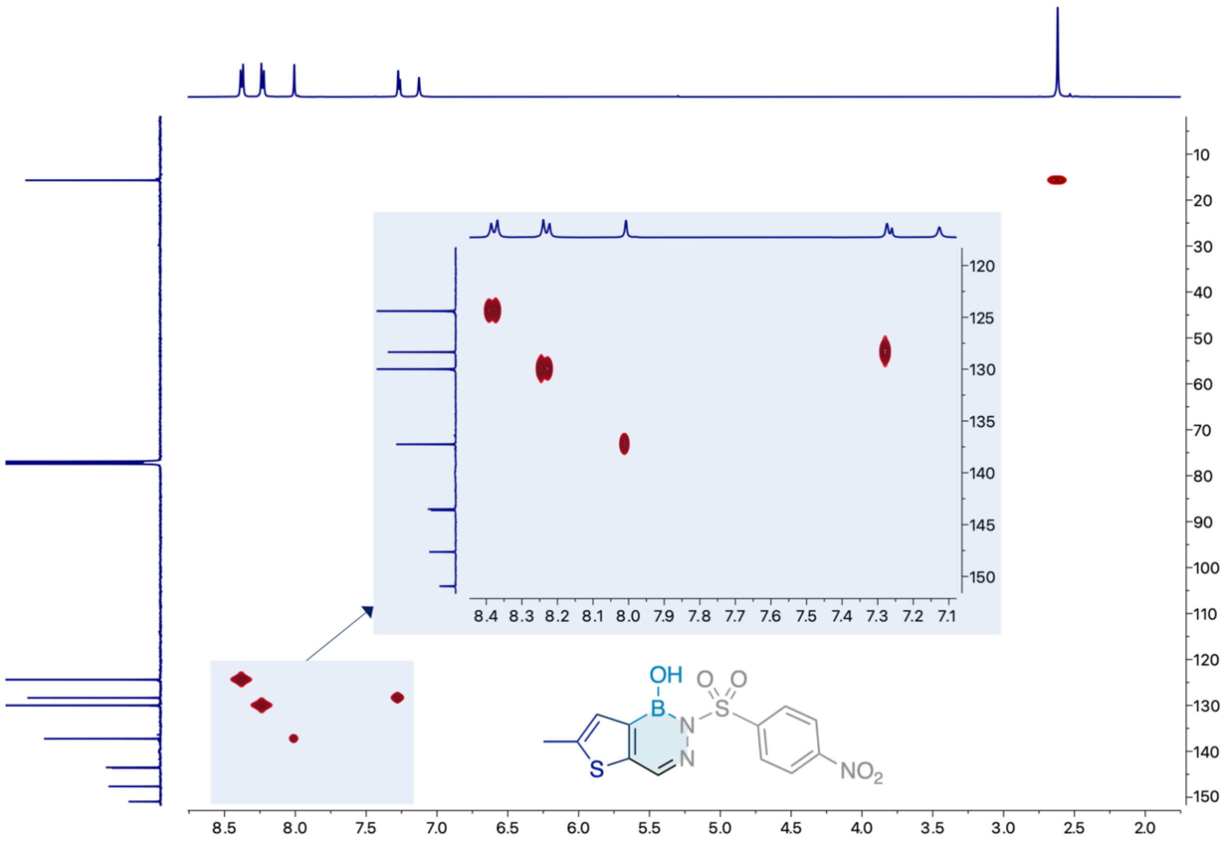

Figure S213. Diazaborine 40: ^{1}H-^{13}C gHSQC NMR ($CDCl_3$, 298 K)

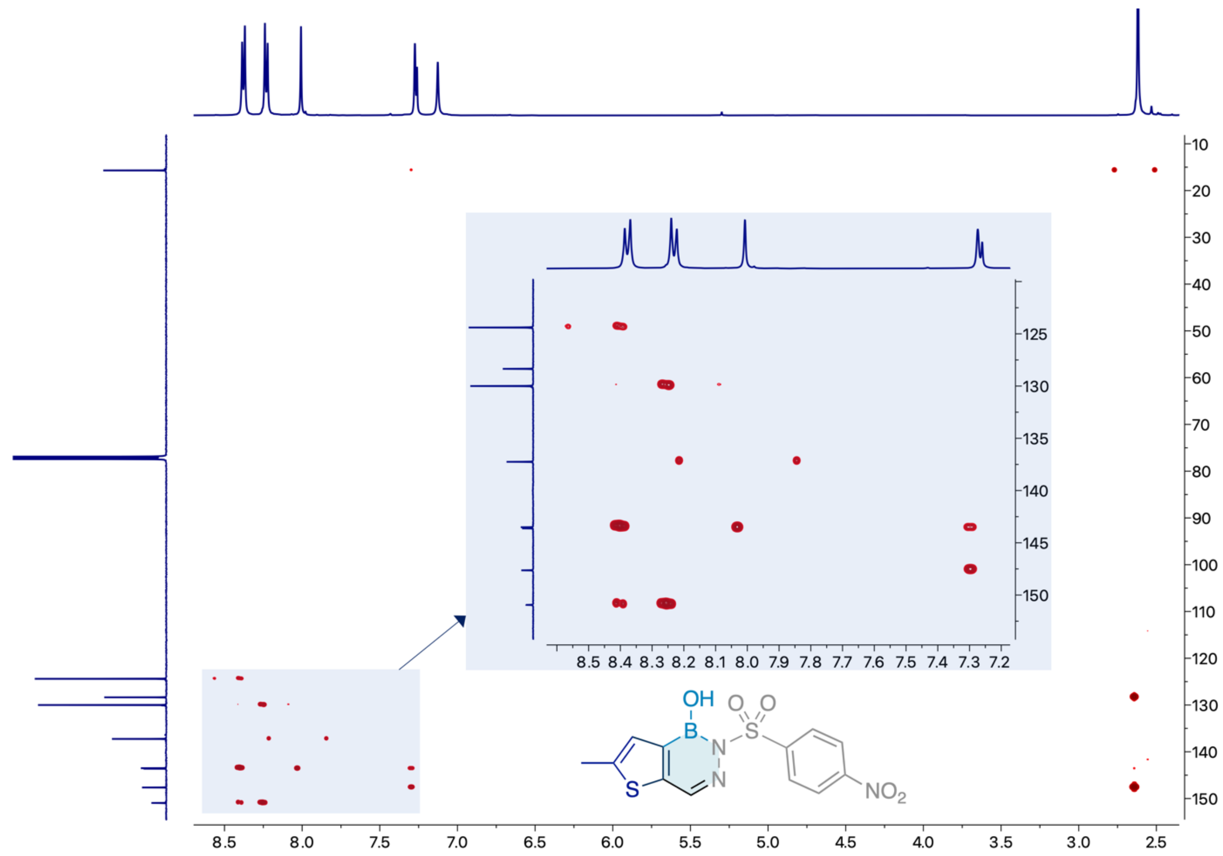

Figure S214. Diazaborine 40: ^{1}H-^{13}C gHMBC NMR ($CDCl_3$, 298 K)

Diazaborine 41

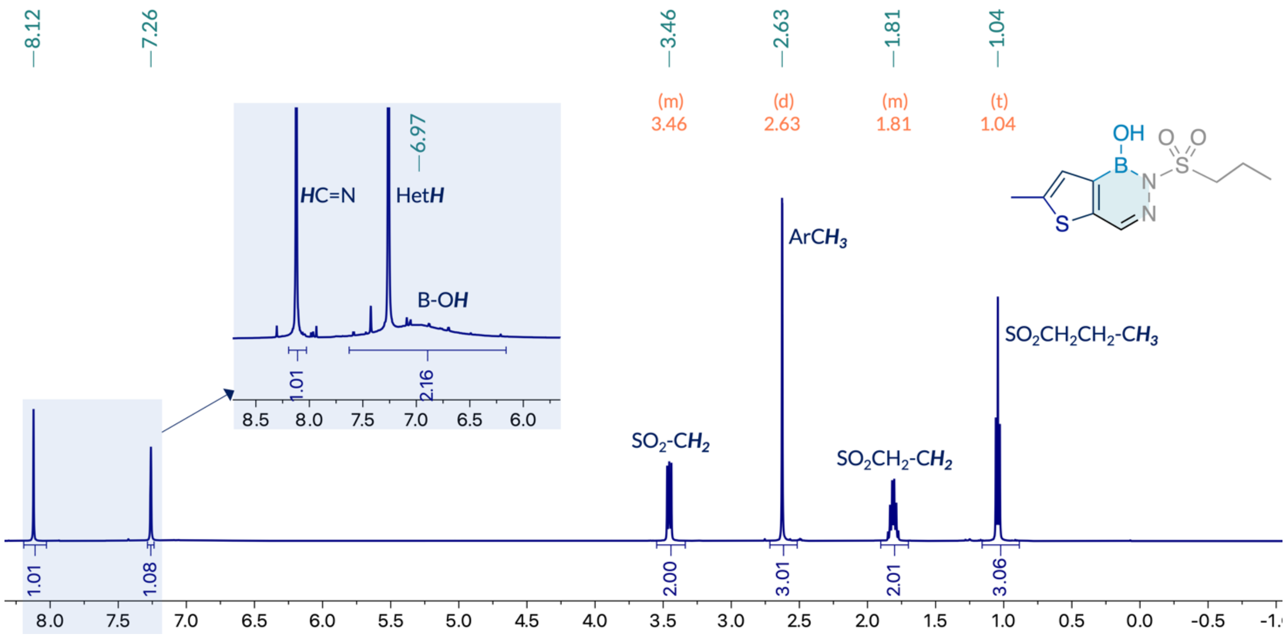

Figure S215. Diazaborine 41:^{1}H NMR (500 MHz, $CDCl_3$, 298 K)

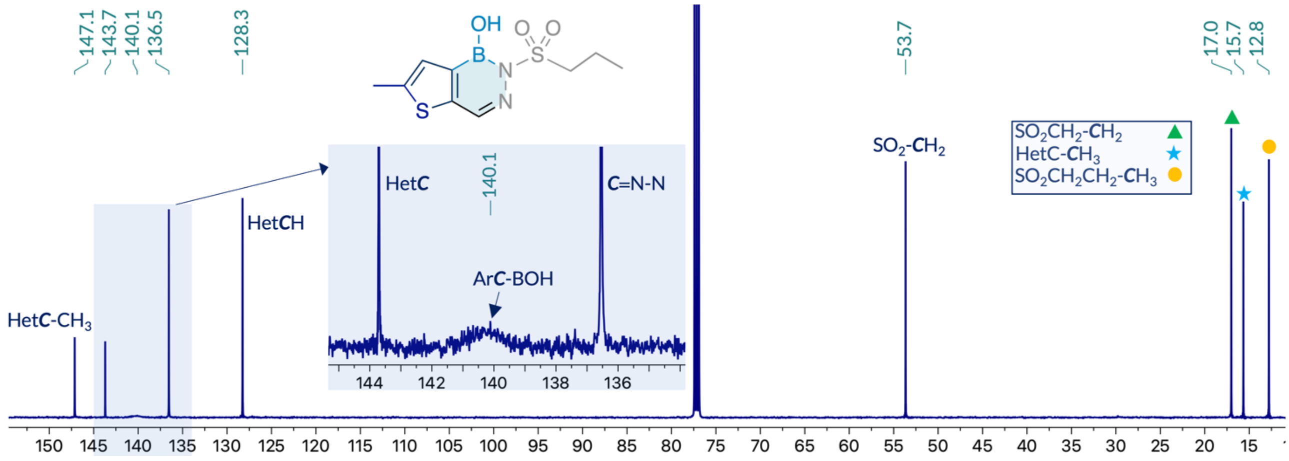

Figure S216. Diazaborine 41:^{13}C NMR (126 MHz, $CDCl_3$, 298 K)

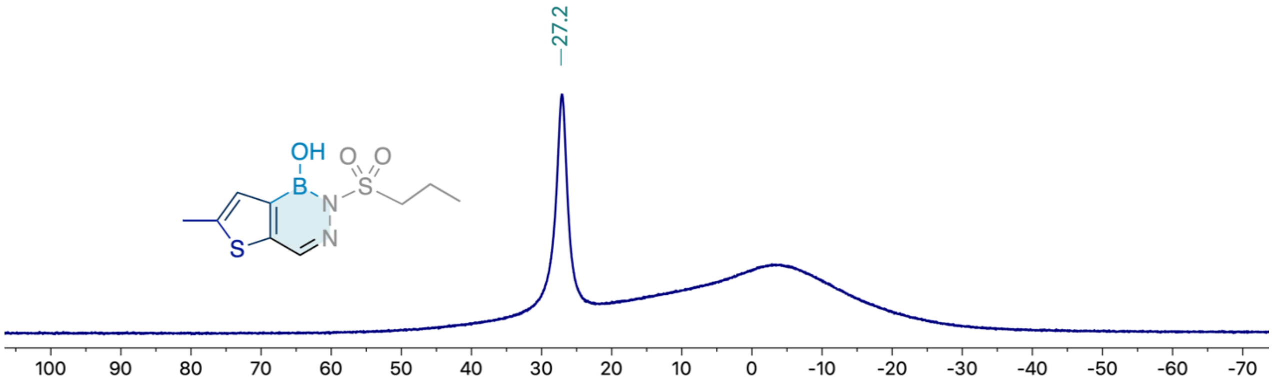

Figure S217. Diazaborine 41:^{11}B NMR (161 MHz, $CDCl_3$, 298 K)

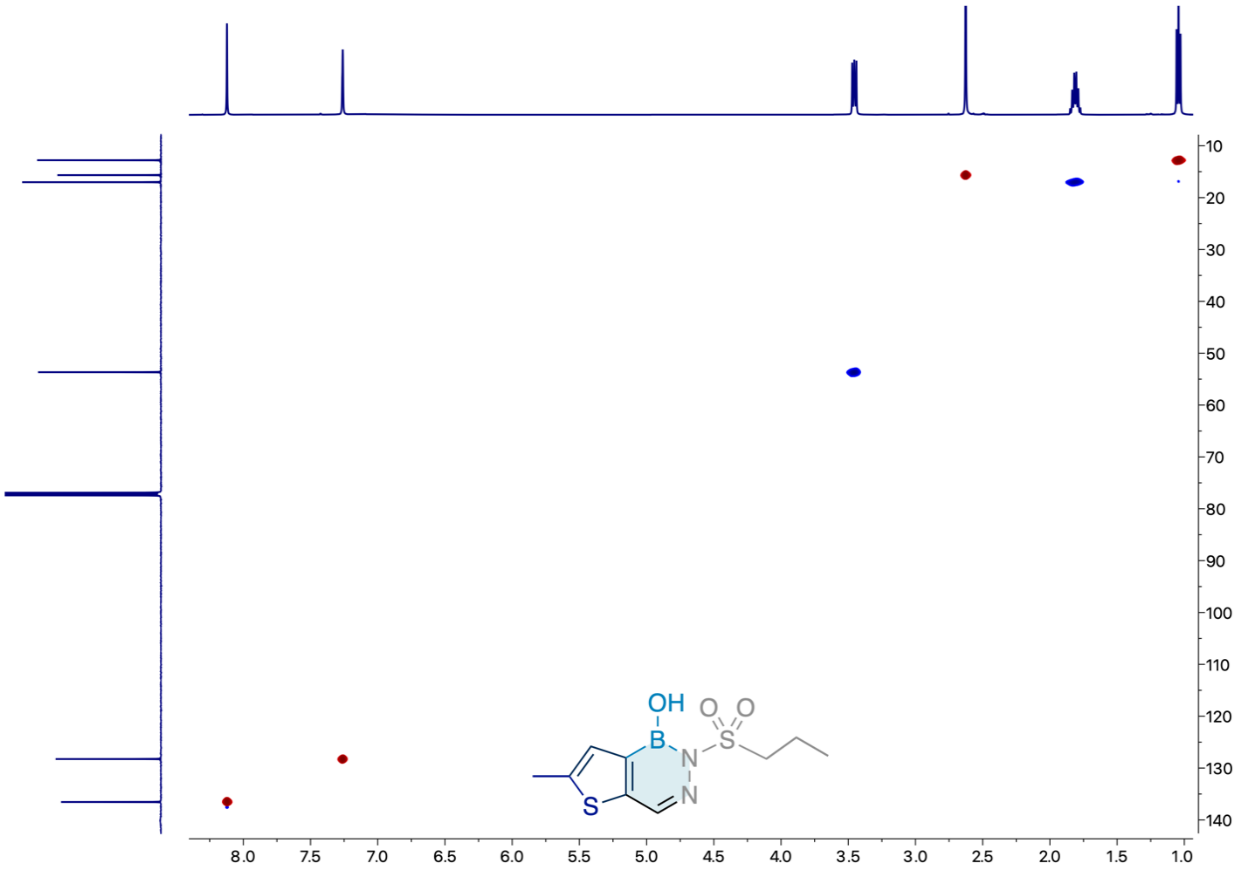

Figure S218. Diazaborine 41: ^{1}H-^{13}C gHSQC NMR ($CDCl_3$, 298 K)

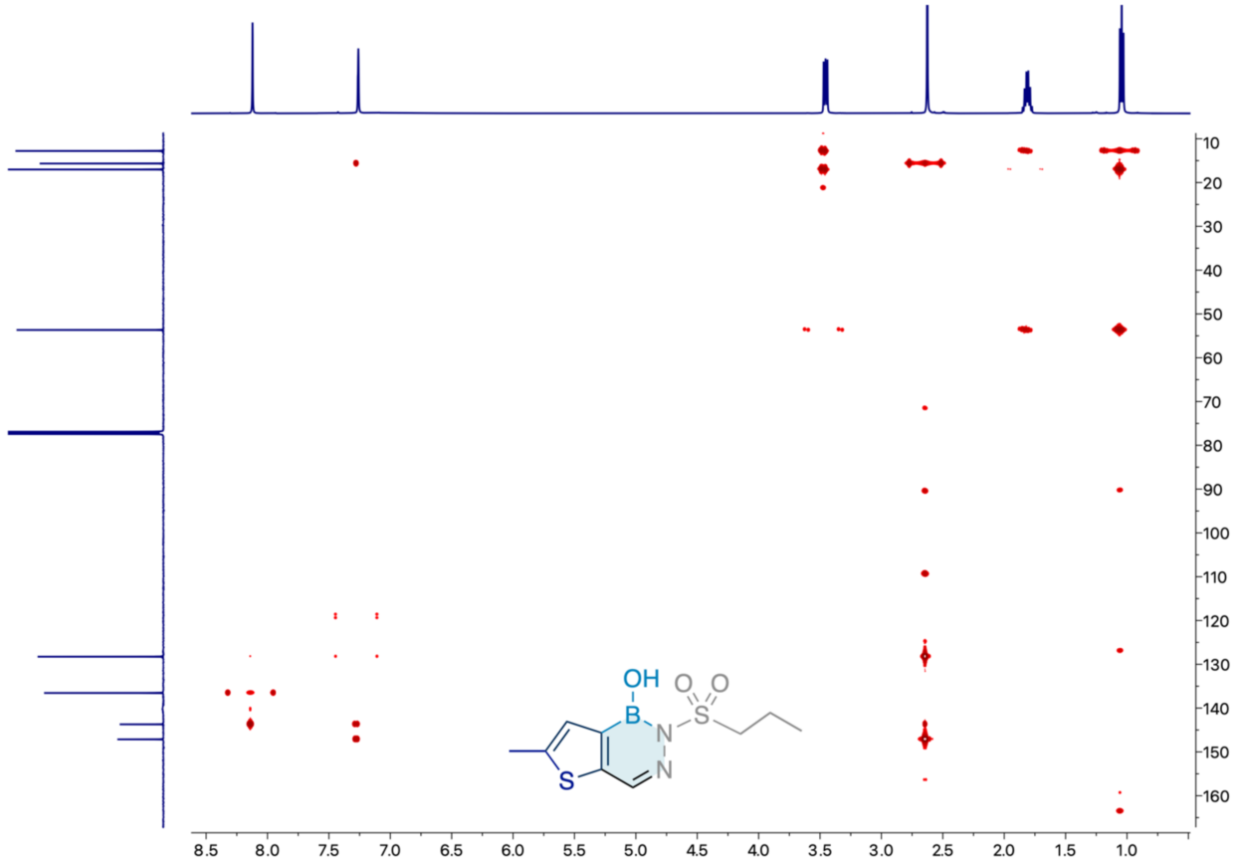

Figure S219. Diazaborine 41: ^{1}H-^{13}C gHMBC NMR ($CDCl_3$, 298 K)

Diazaborine 42

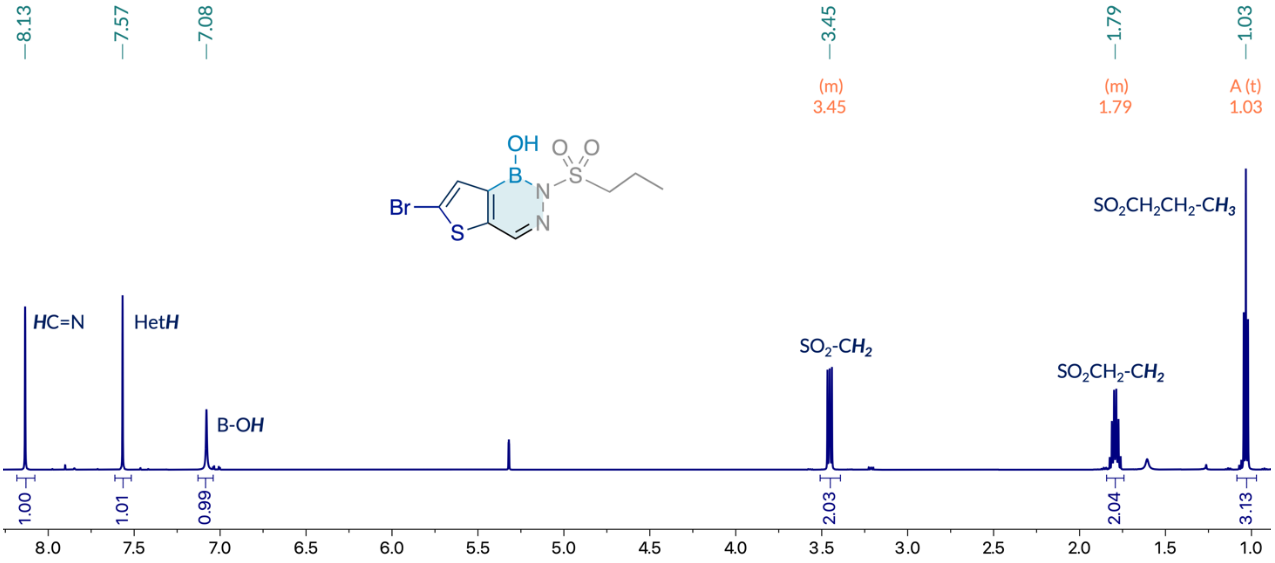

Figure S220. Diazaborine 42: ^{1}H NMR (600 MHz, CD_2Cl_2, 298 K)

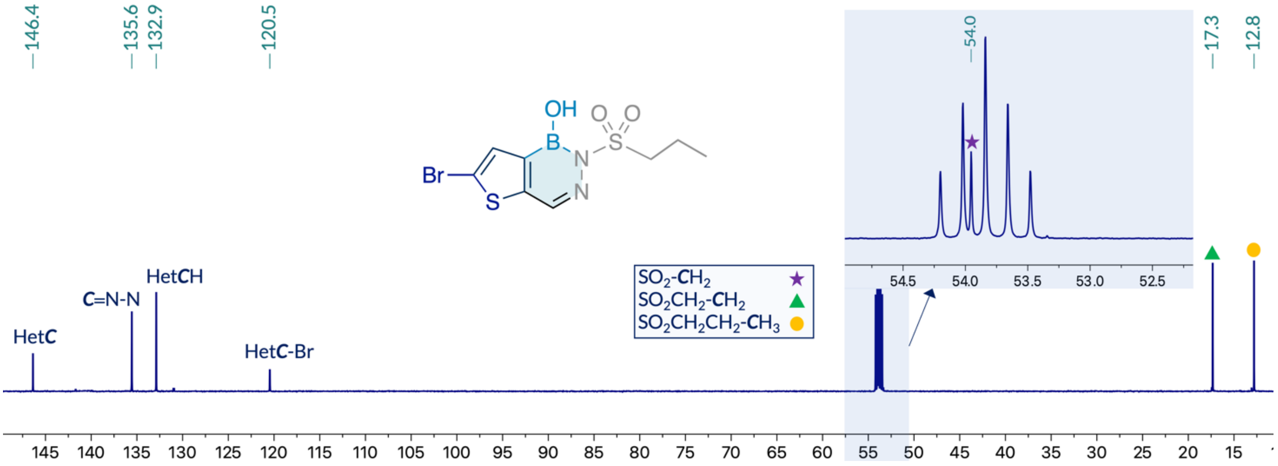

Figure S221. Diazaborine 42: ^{13}C NMR (151 MHz, CD_2Cl_2, 298 K)

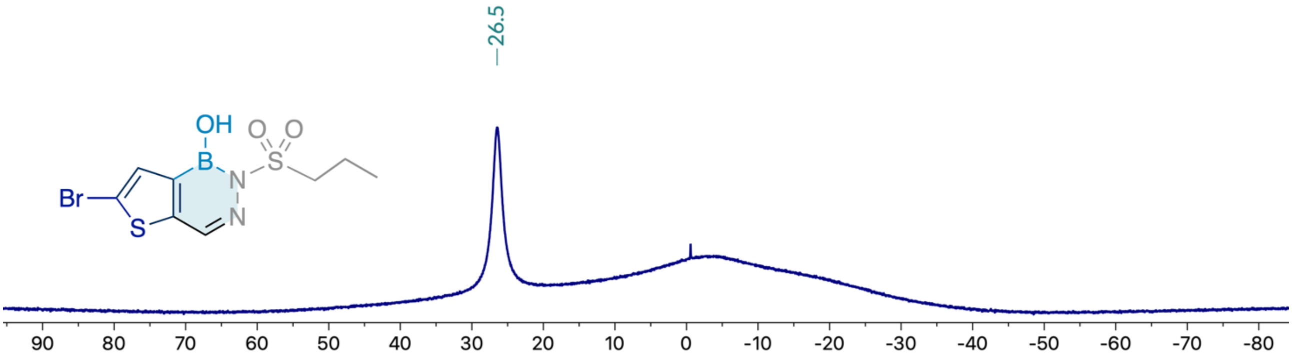

Figure S222. Diazaborine 42: ^{11}B NMR (128 MHz, CD_2Cl_2, 298 K)

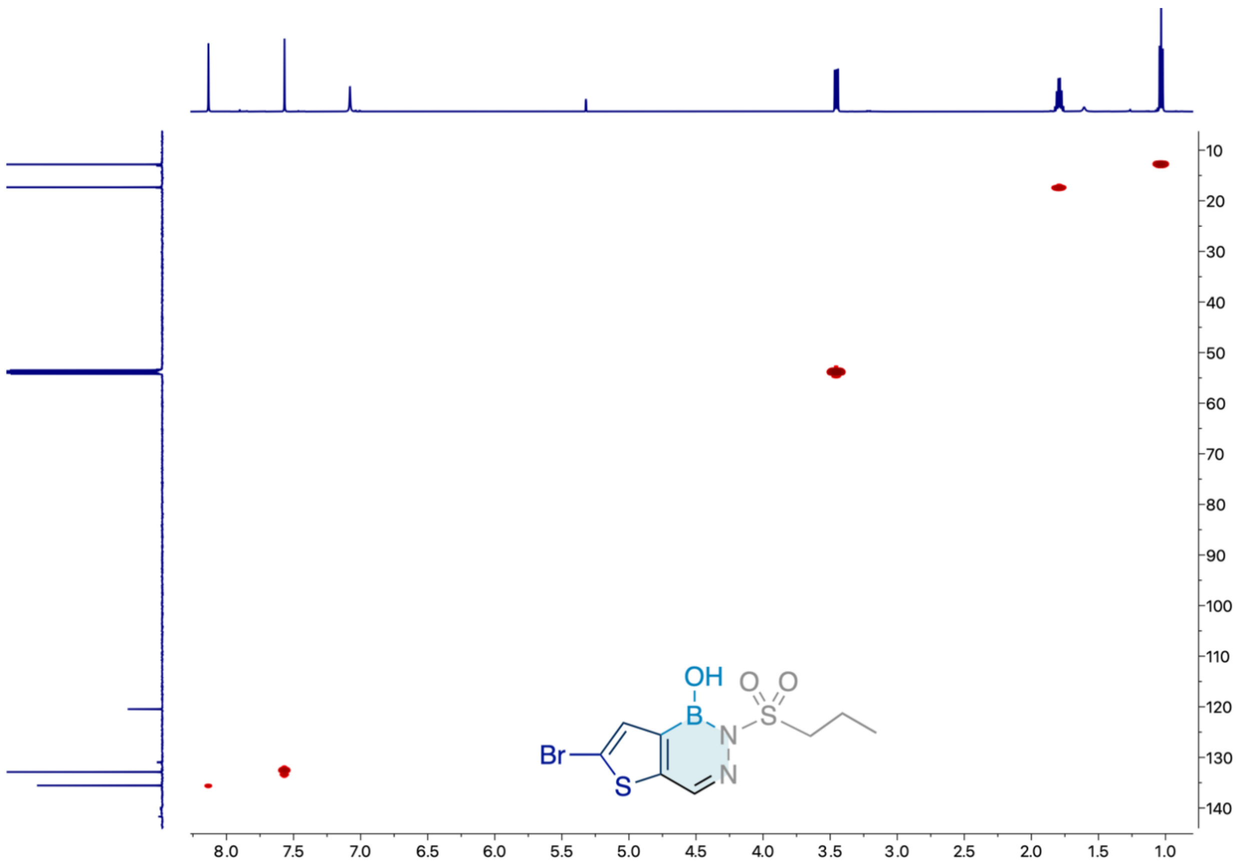

Figure S223. Diazaborine 42: ^{1}H-^{13}C gHSQC NMR (CD_2Cl_2, 298 K)

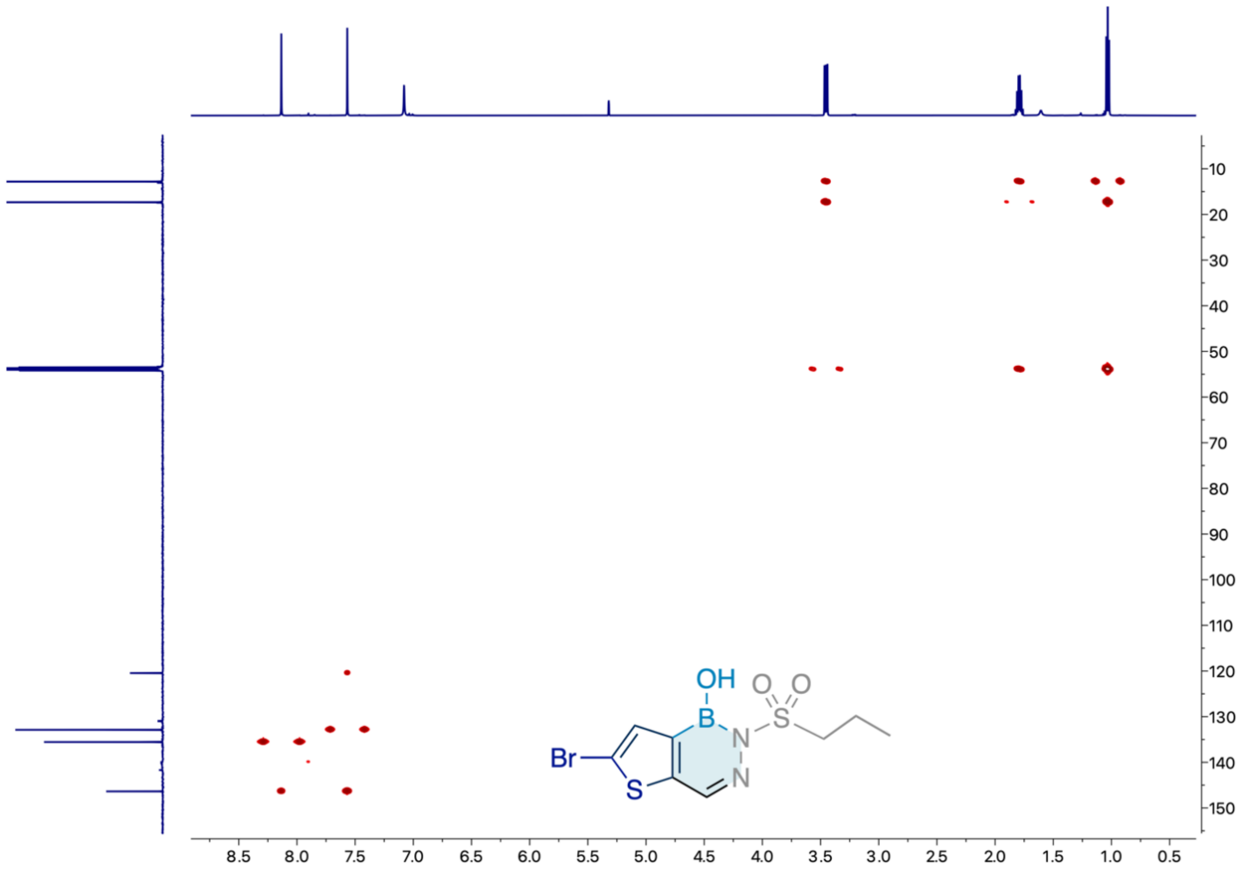

Figure S224. Diazaborine 42: ^{1}H-^{13}C gHMBC NMR (CD_2Cl_2, 298 K)

Diazaborine 43

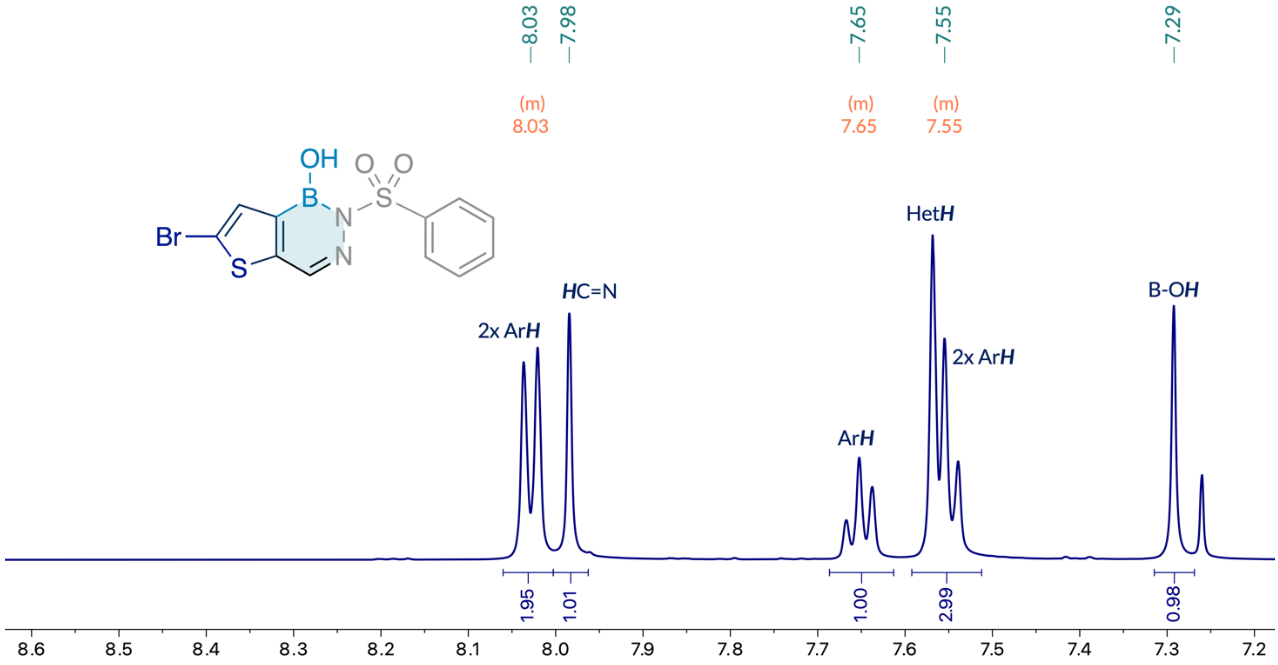

Figure S225. Diazaborine 43:^{1}H NMR (500 MHz, $CDCl_3$, 298 K)

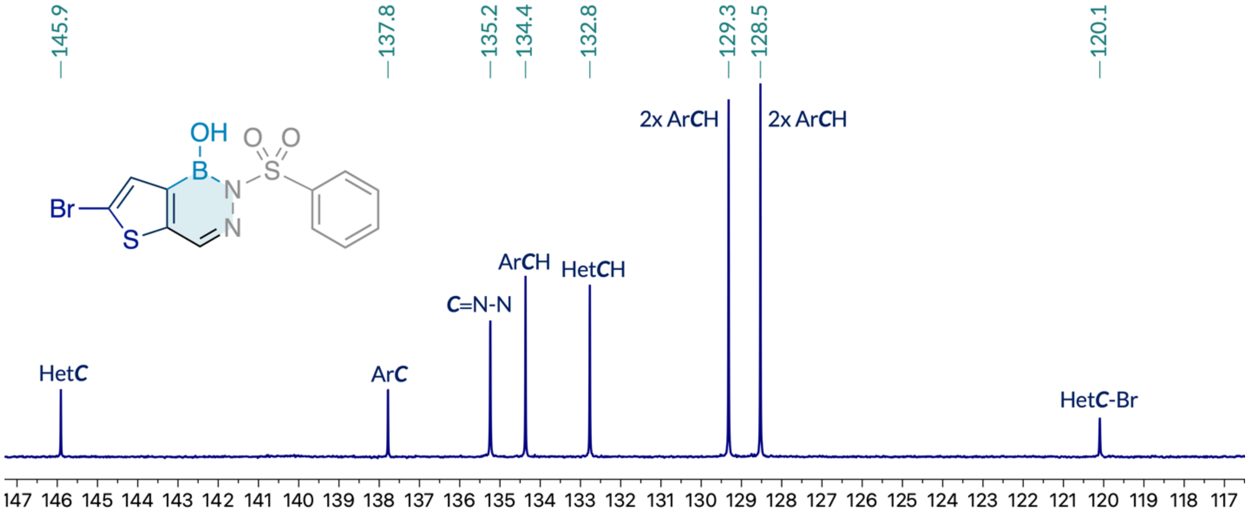

Figure S226. Diazaborine 43:^{13}C NMR (126 MHz, $CDCl_3$, 298 K)

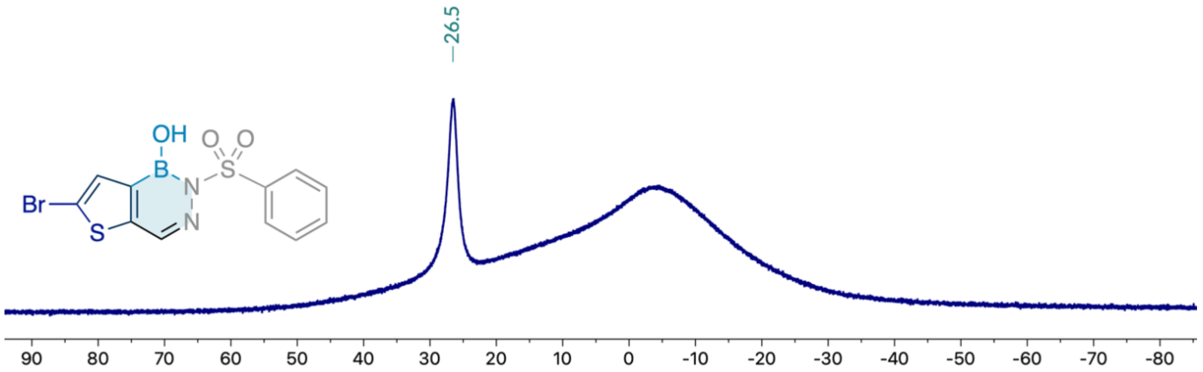

Figure S227. Diazaborine 43:^{11}B NMR (160 MHz, $CDCl_3$, 298 K)

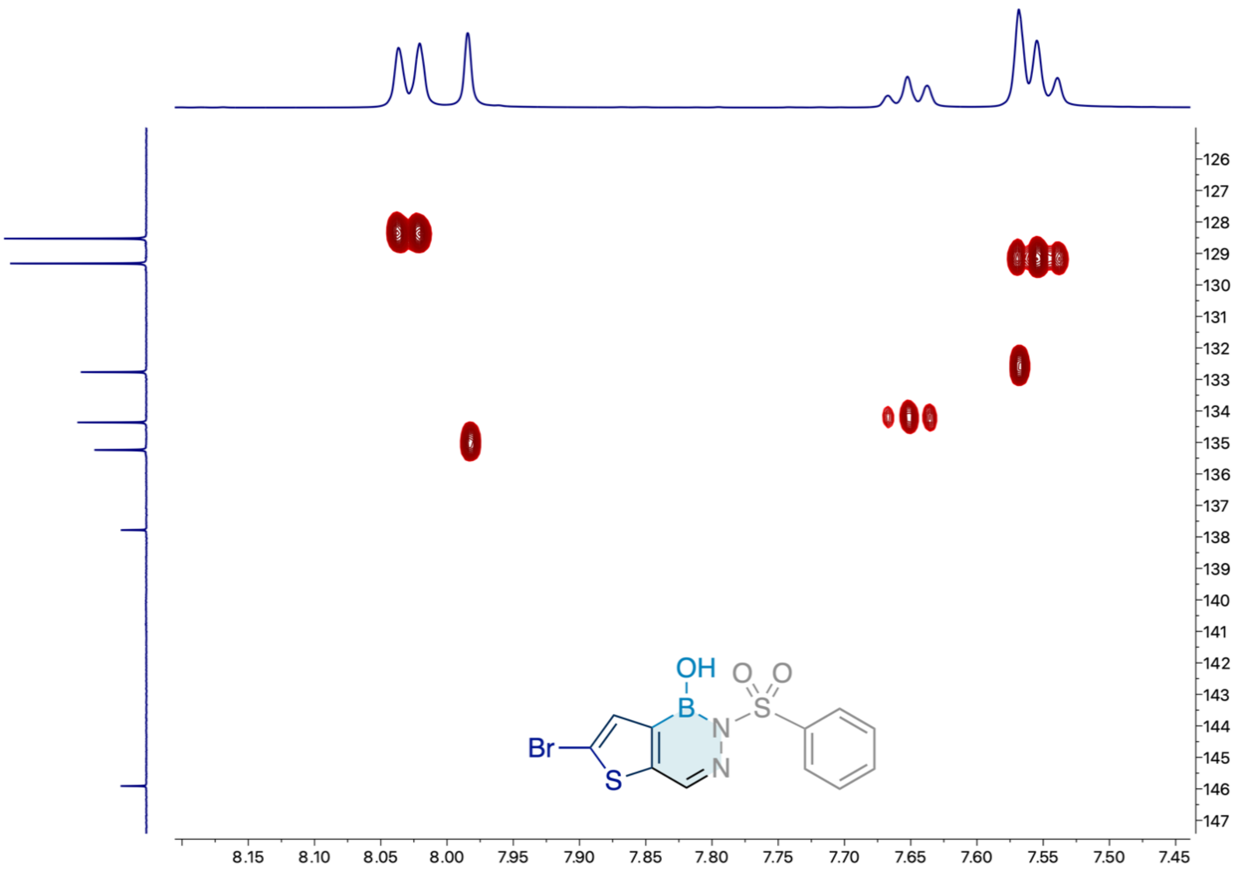

Figure S228. Diazaborine 43: ^{1}H-^{13}C gHSQC NMR ($CDCl_3$, 298 K)

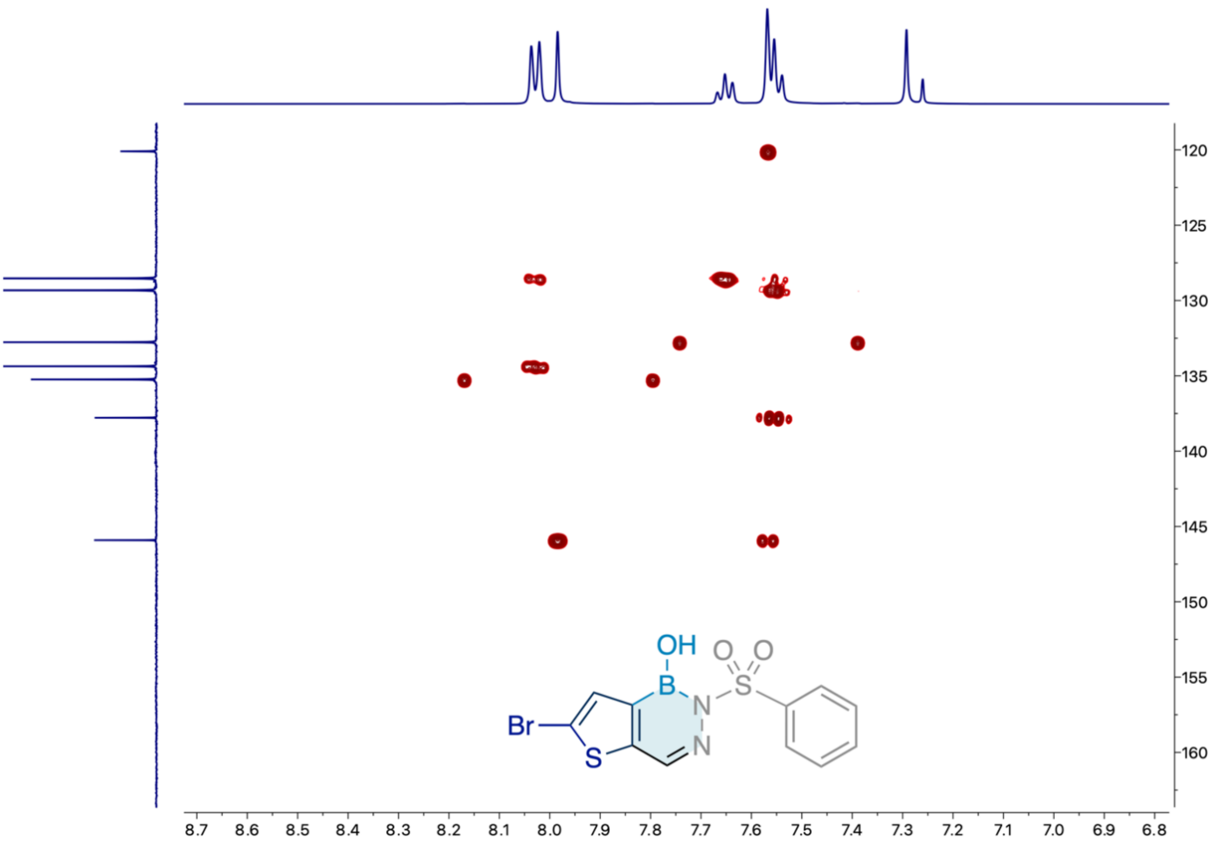

Figure S229. Diazaborine 43: ^{1}H-^{13}C gHMBC NMR ($CDCl_3$, 298 K)

Diazaborine 44

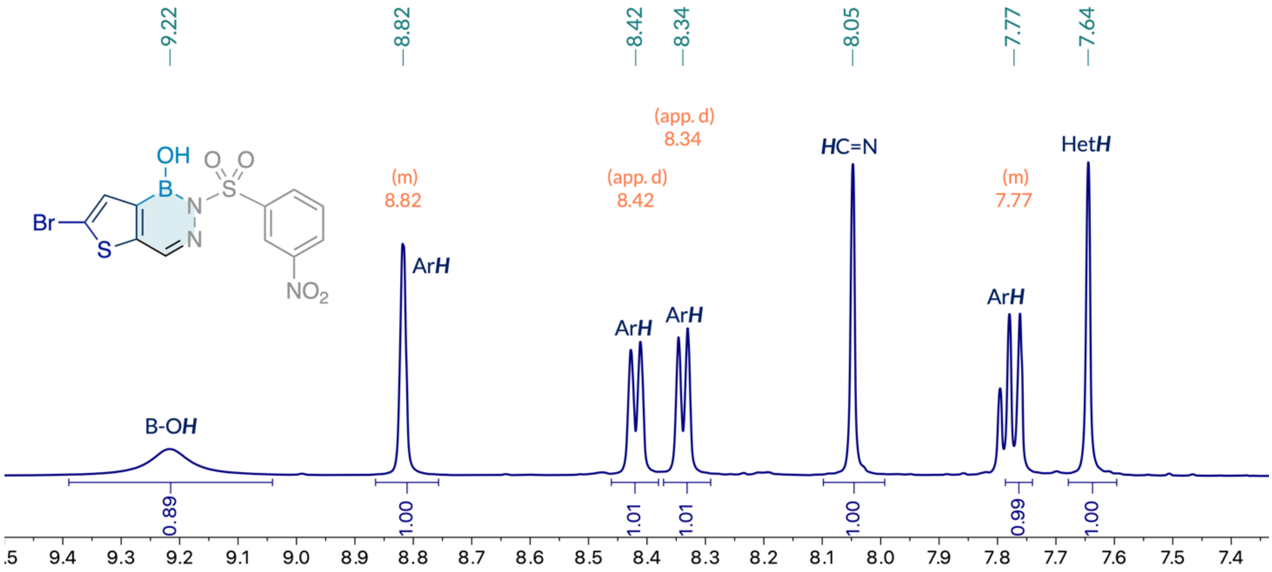

Figure S230. Diazaborine 44:^{1}H NMR (500 MHz, $CDCl_3$:DMSO-d_6 1.2:1, 298 K)

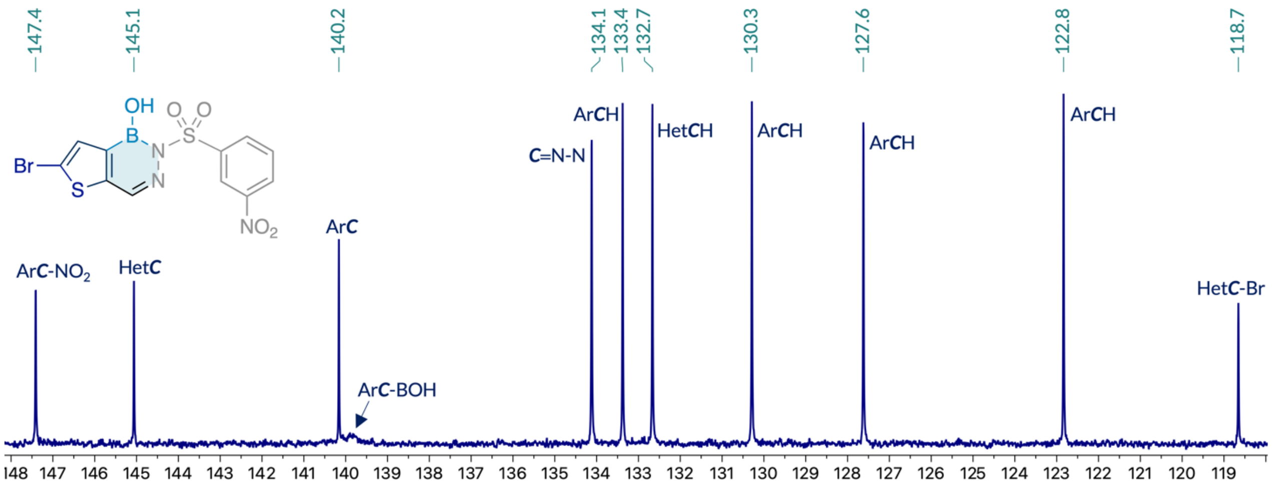

Figure S231. Diazaborine 44:^{13}C NMR (126 MHz, $CDCl_3$:DMSO-d_6 1.2:1, 298 K)

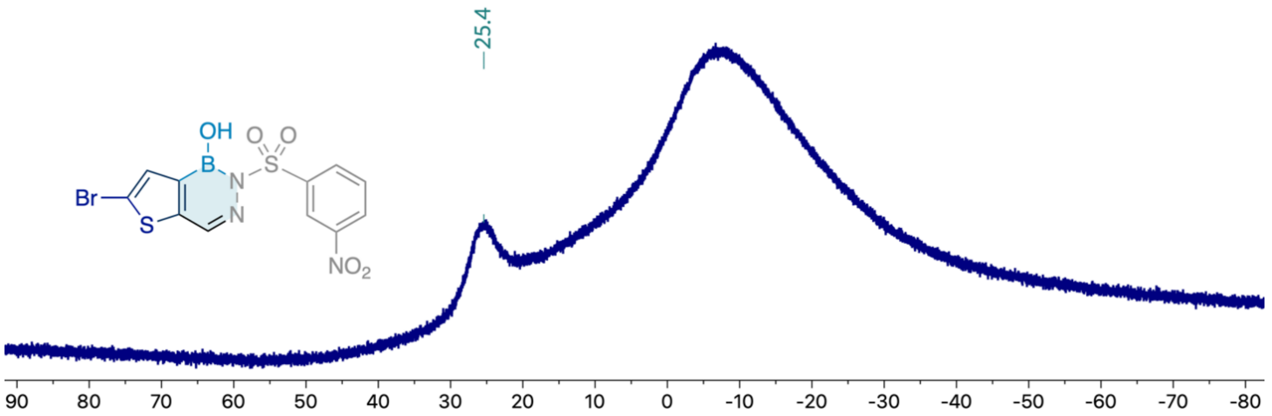

Figure S232. Diazaborine 44:^{11}B NMR (160 MHz, $CDCl_3$:DMSO-d_6 1.2:1, 298 K)

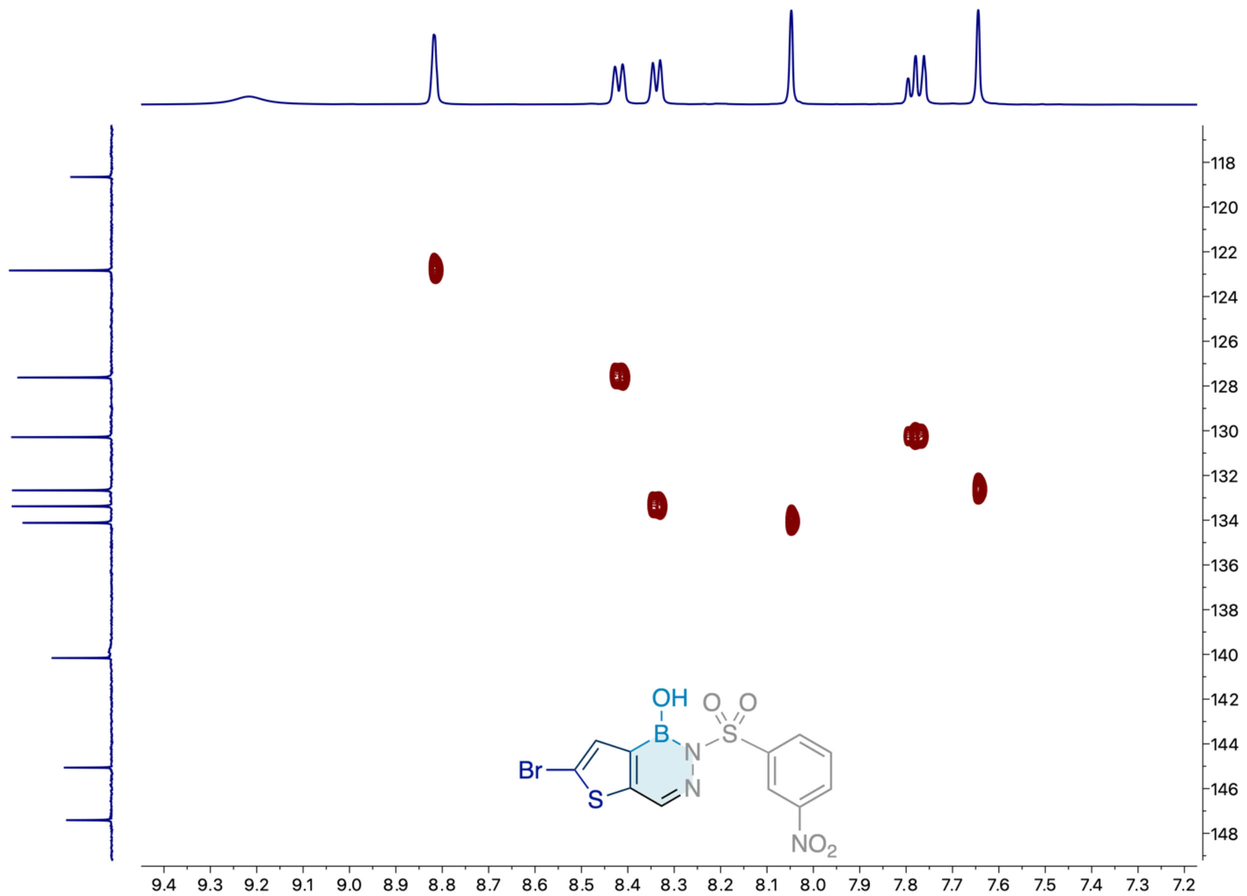

Figure S233. Diazaborine 44: ^{1}H-^{13}C gHSQC NMR ($CDCl_3$:DMSO-d_6 1.2:1, 298 K)

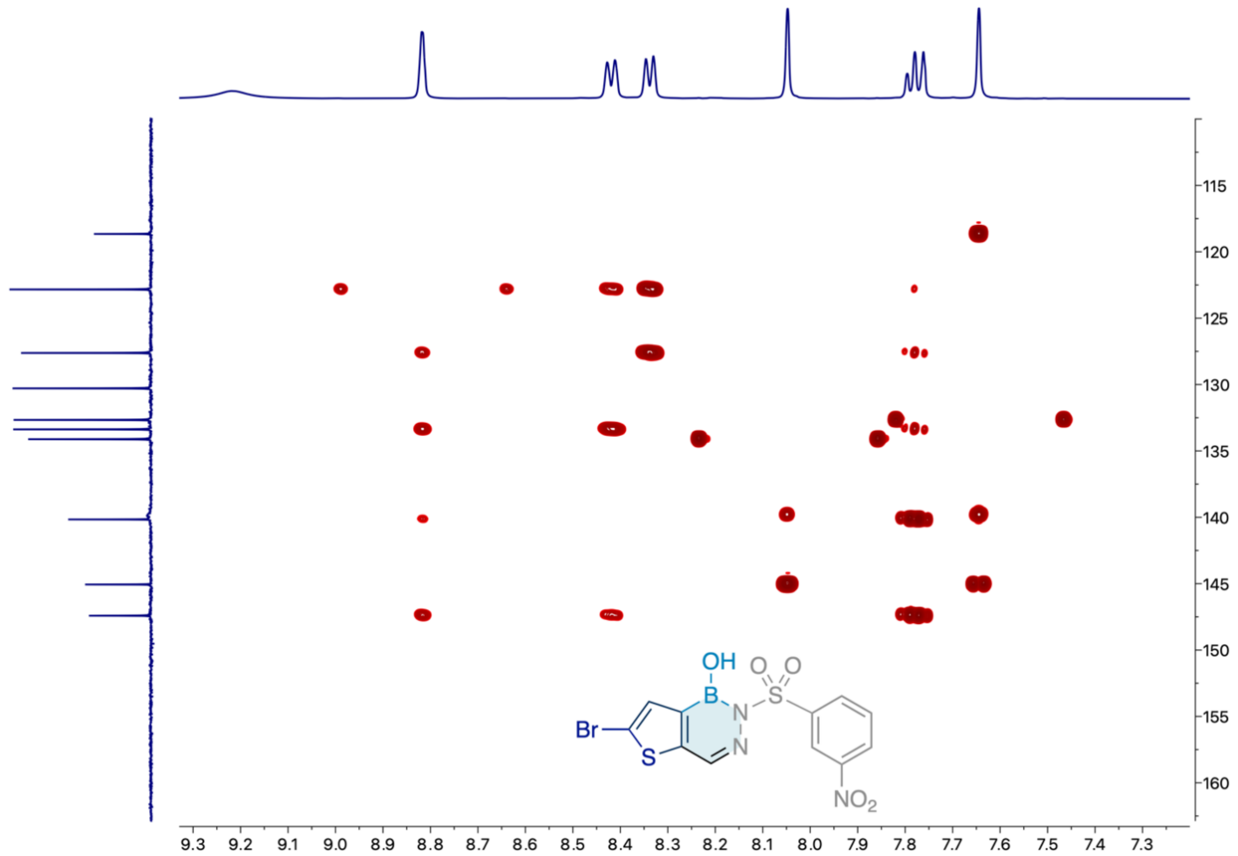

Figure S234. Diazaborine 44: ^{1}H-^{13}C gHMBC NMR ($CDCl_3$:DMSO-d_6 1.2:1, 298 K)

Diazaborine 45

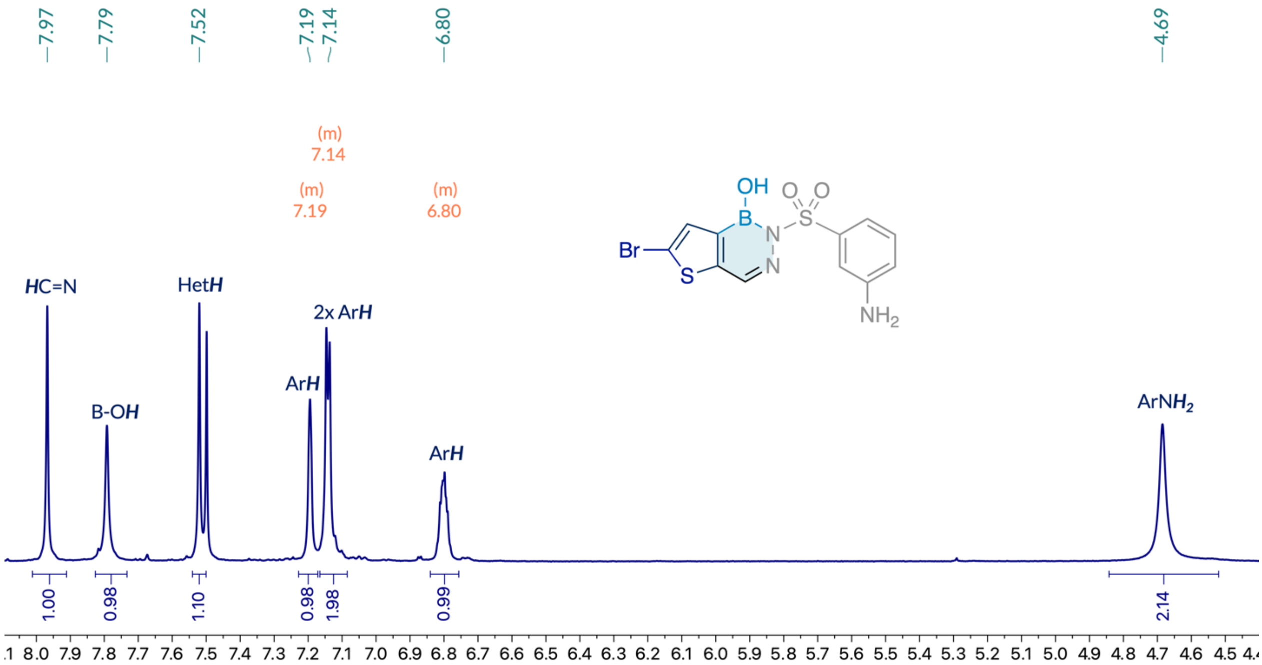

Figure S235. Diazaborine 45: ^{1}H NMR (500 MHz, $CDCl_3$:DMSO-d_6 2:1, 298 K)

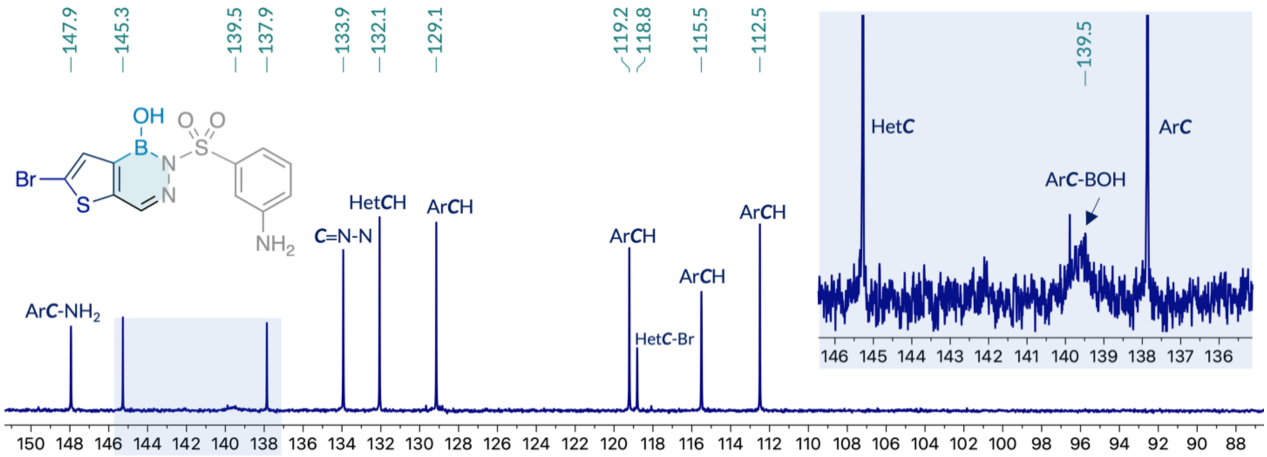

Figure S236. Diazaborine 45: ^{13}C NMR (126 MHz, $CDCl_3$:DMSO-d_6 2:1, 298 K)

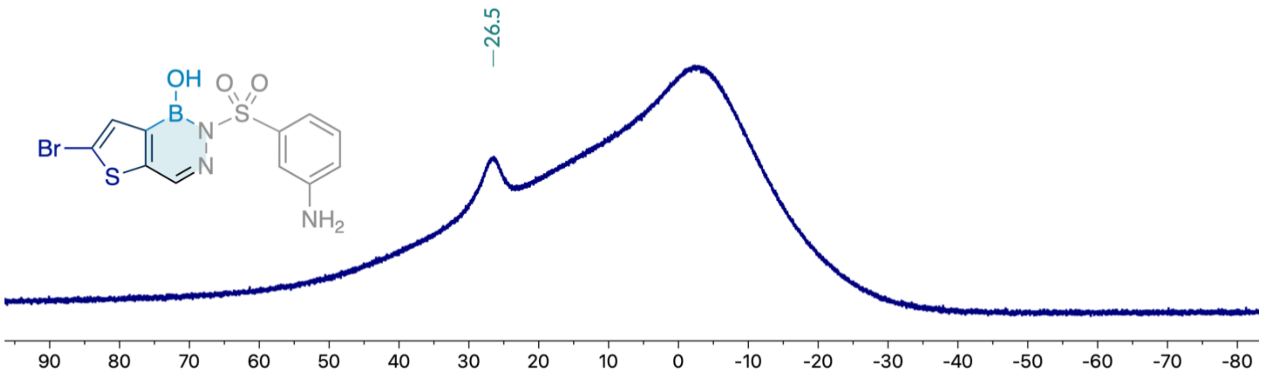

Figure S237. Diazaborine 45: ^{11}B NMR (160 MHz, $CDCl_3$:DMSO-d_6 2:1, 298 K)

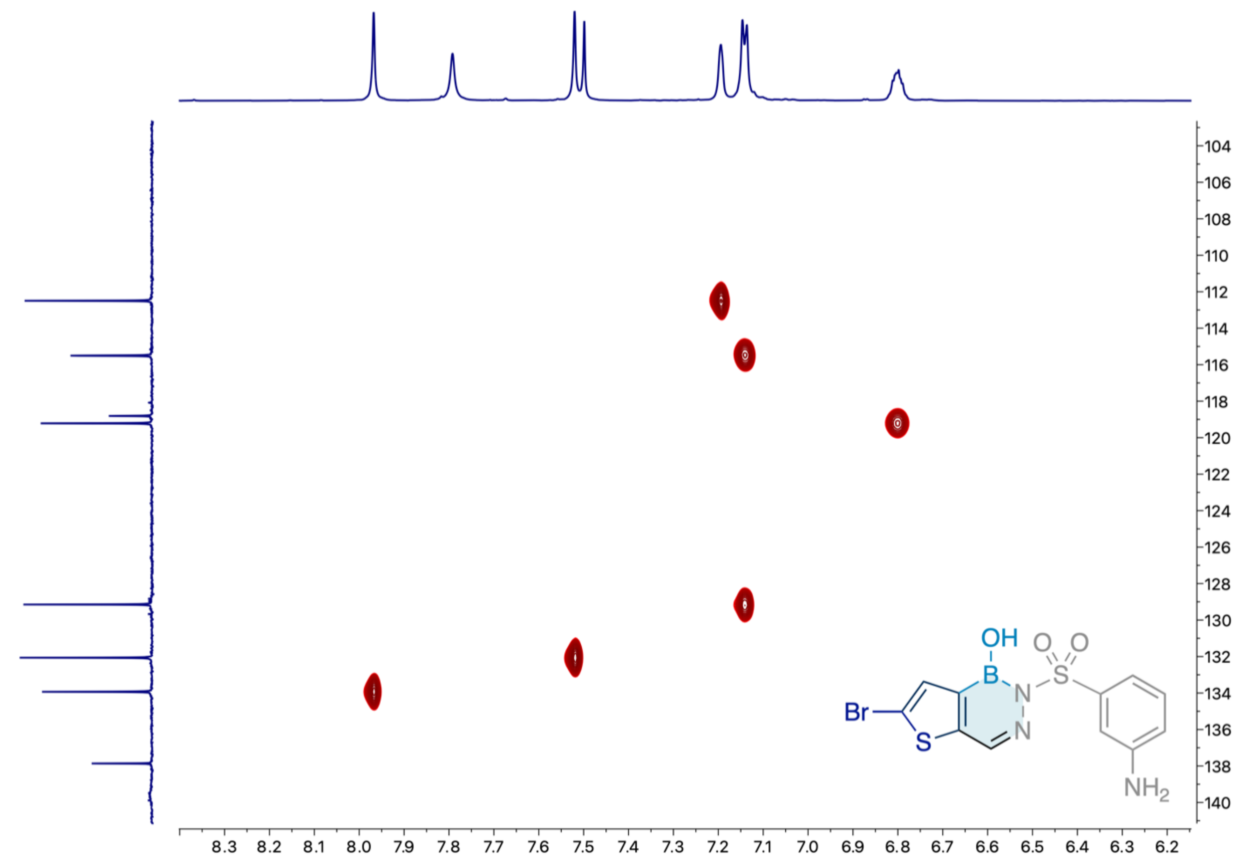

Figure S238. Diazaborine 45: ^{1}H-^{13}C gHSQC NMR (CDCl$_3$:DMSO-d_6 2:1, 298 K)

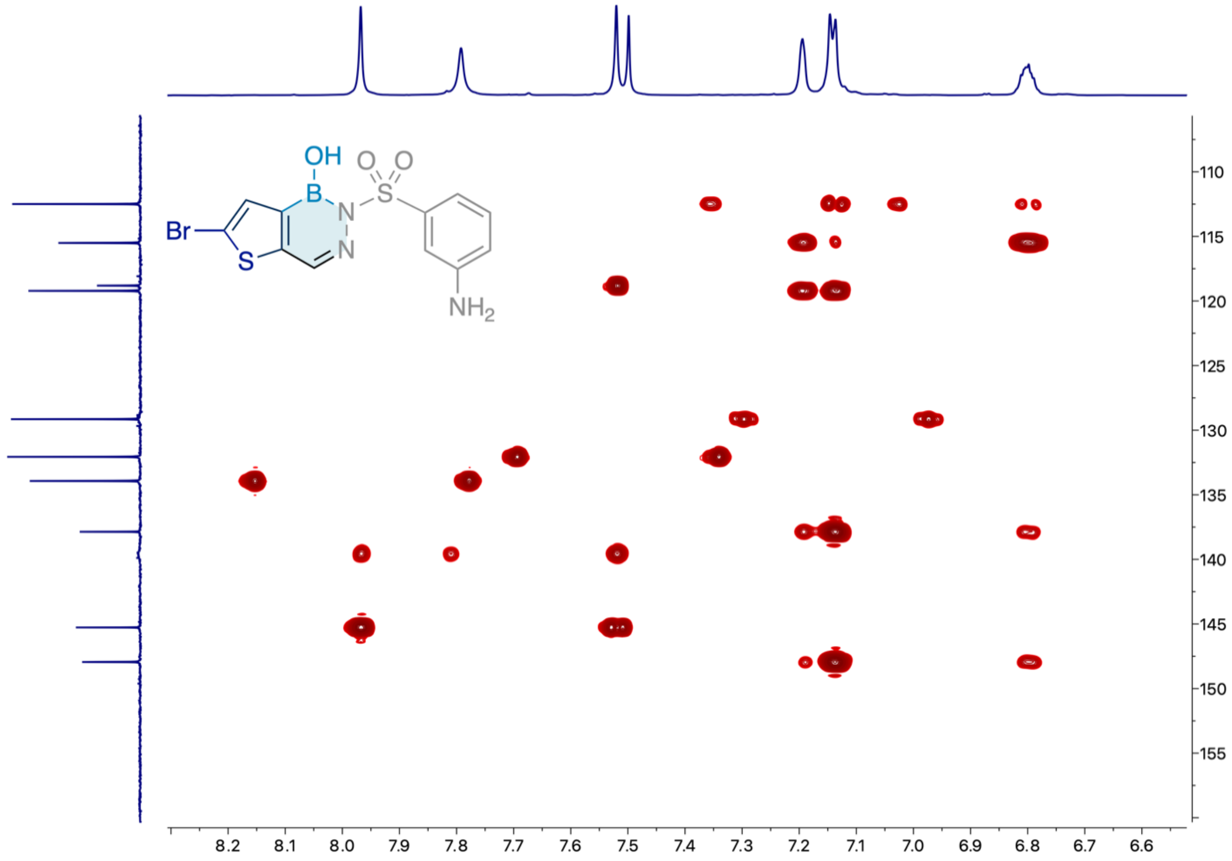

Figure S239. Diazaborine 45: ^{1}H-^{13}C gHMBC NMR (CDCl$_3$:DMSO-d_6 2:1, 298 K)

Diazaborine 46

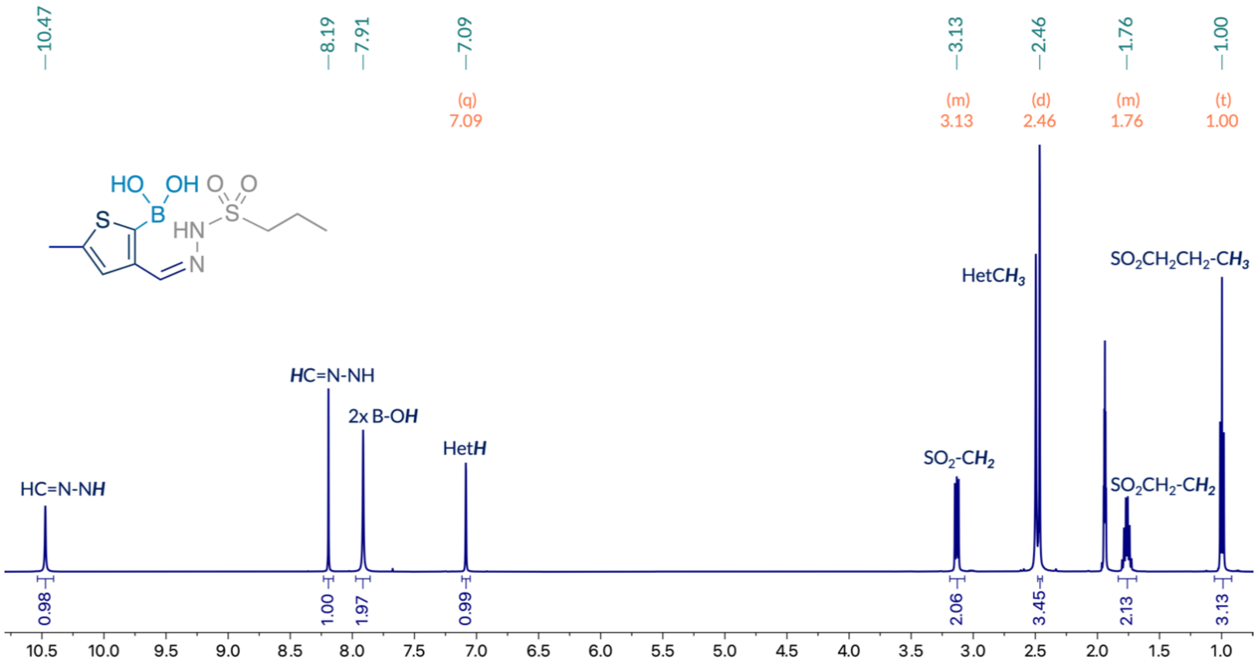

Figure S240. Diazaborine 46:^{1}H NMR (500 MHz, CD_3CN:DMSO-d_6 10:1, 298 K)

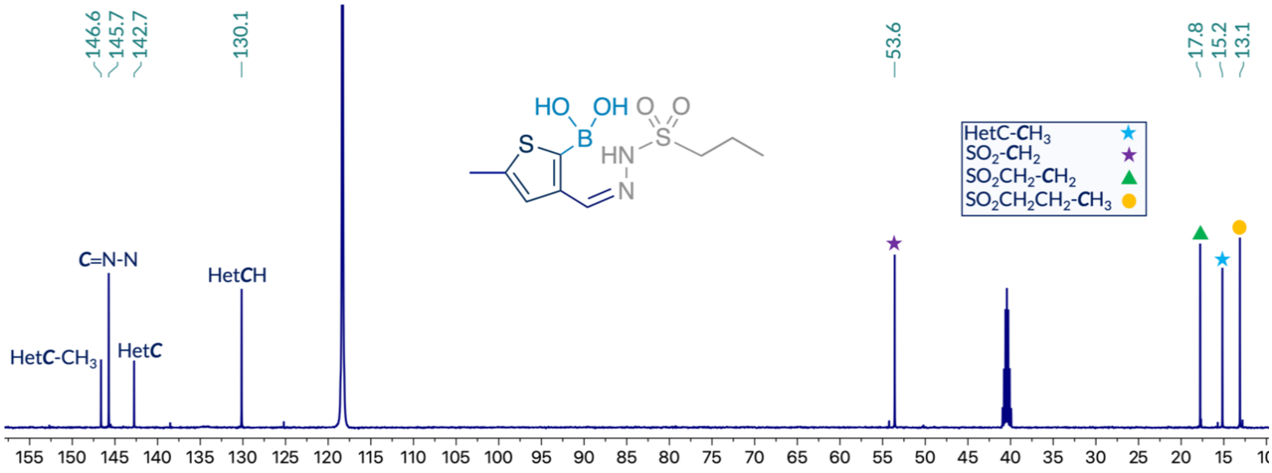

Figure S241. Diazaborine 46:^{13}C NMR (126 MHz, CD_3CN:DMSO-d_6 10:1, 298 K)

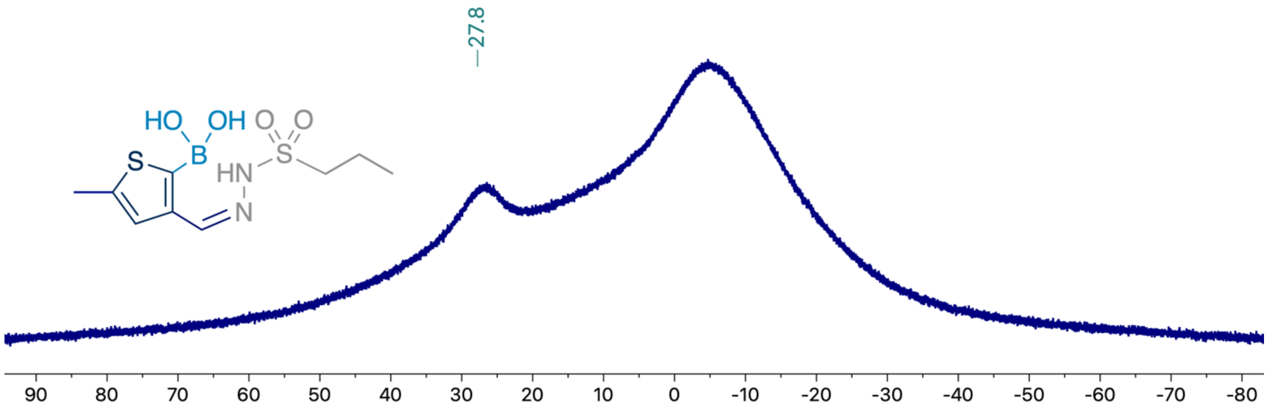

Figure S242. Diazaborine 46:^{11}B NMR (160 MHz, CD_3CN:DMSO-d_6 10:1, 298 K)

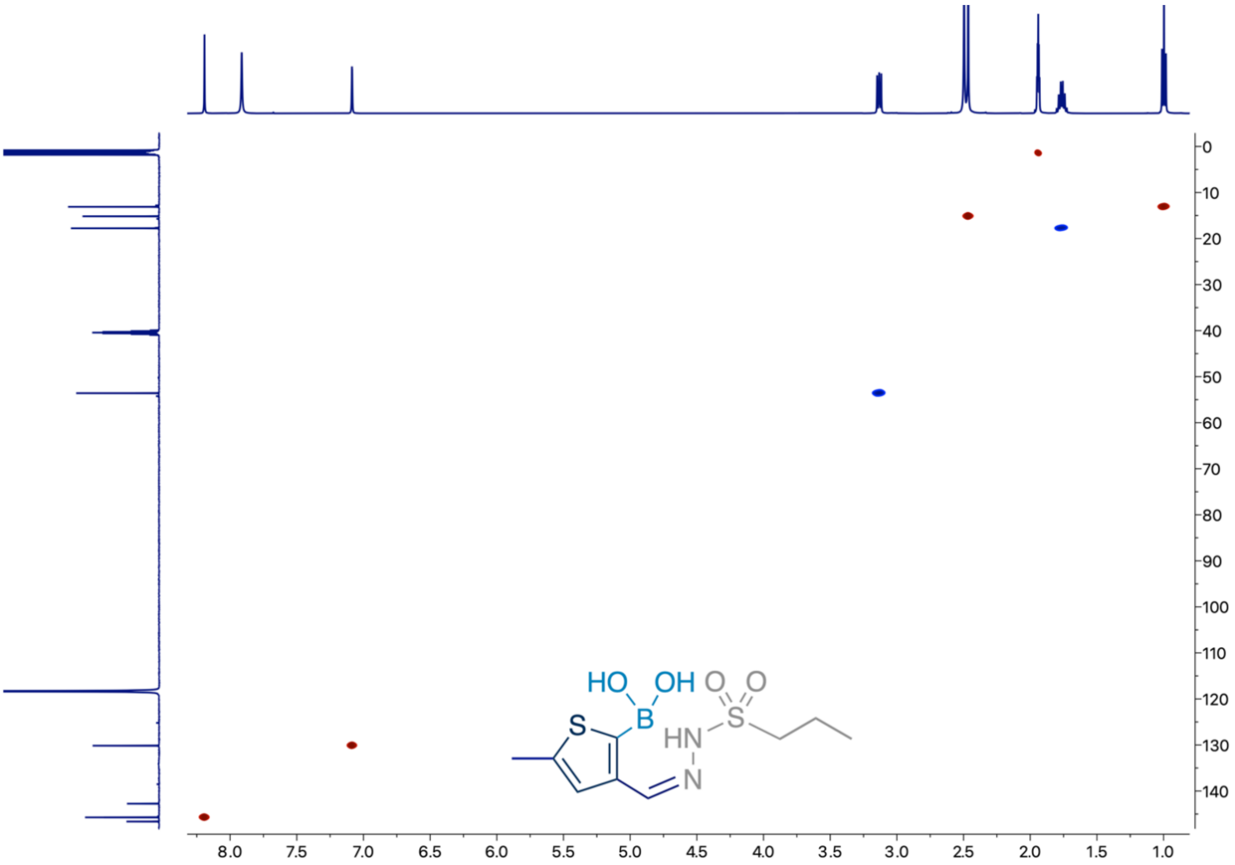

Figure S243. Diazaborine 46: ^{1}H-^{13}C gHSQC NMR (CD_3CN:DMSO-d_6 10:1, 298 K)

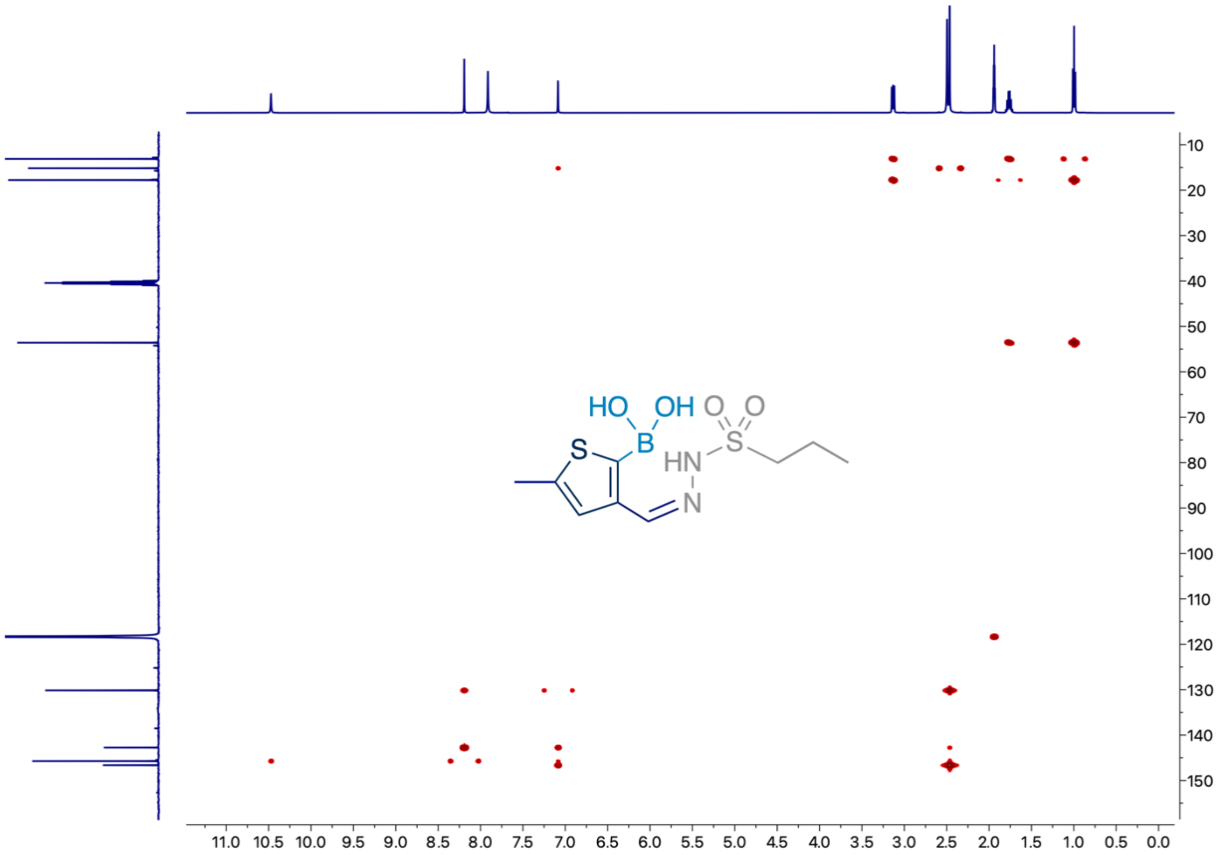

Figure S244. Diazaborine 46: ^{1}H-^{13}C gHMBC NMR (CD_3CN:DMSO-d_6 10:1, 298 K)

Diazaborine 47

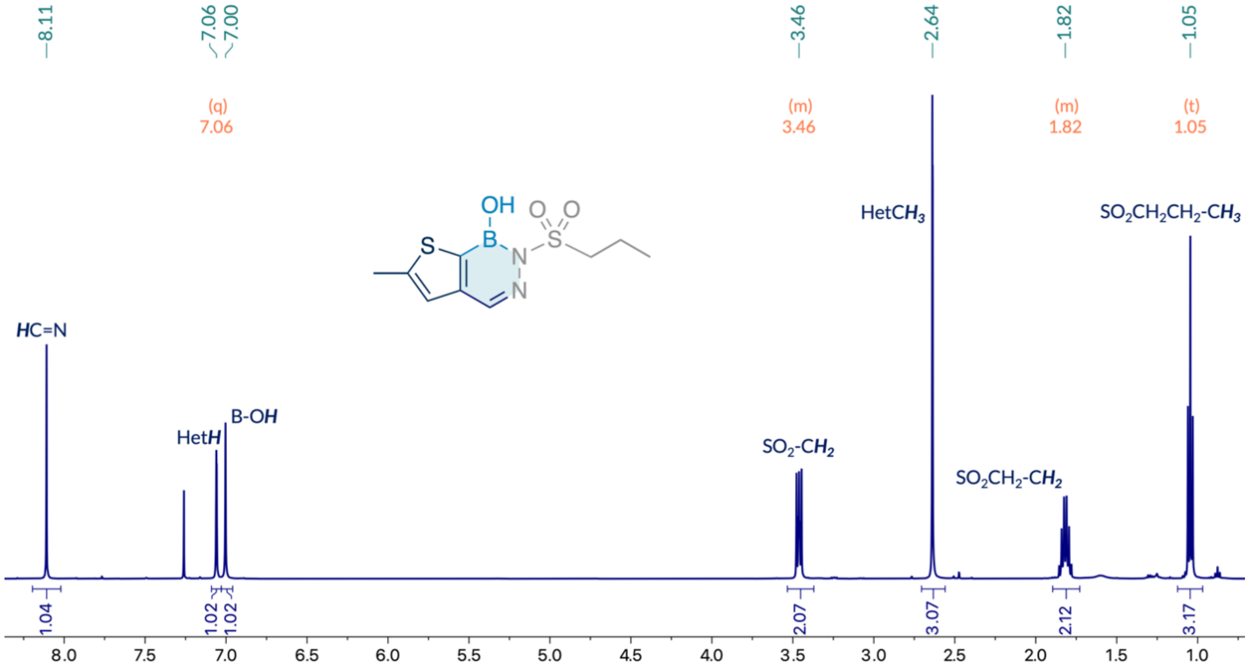

Figure S245. Diazaborine 47:^{1}H NMR (500 MHz, $CDCl_3$, 298 K)

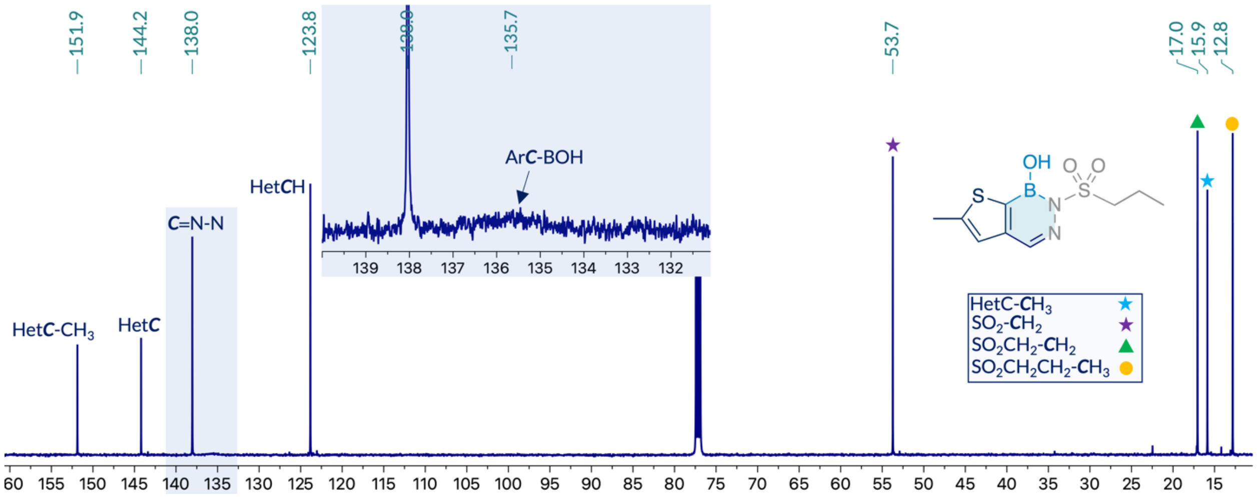

Figure S246. Diazaborine 47:^{13}C NMR (126 MHz, $CDCl_3$, 298 K)

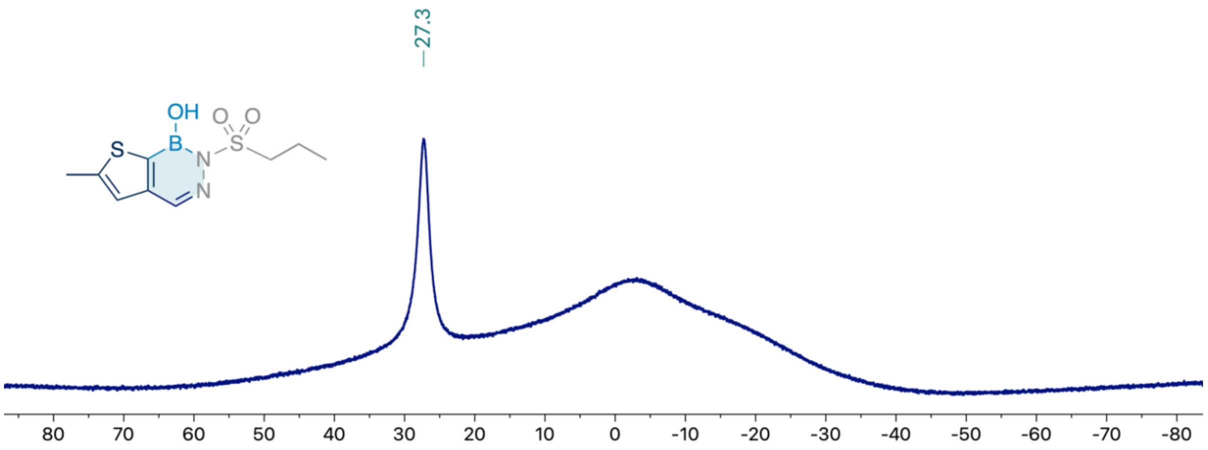

Figure S247. Diazaborine 47:^{11}B NMR (128 MHz, $CDCl_3$, 298 K)

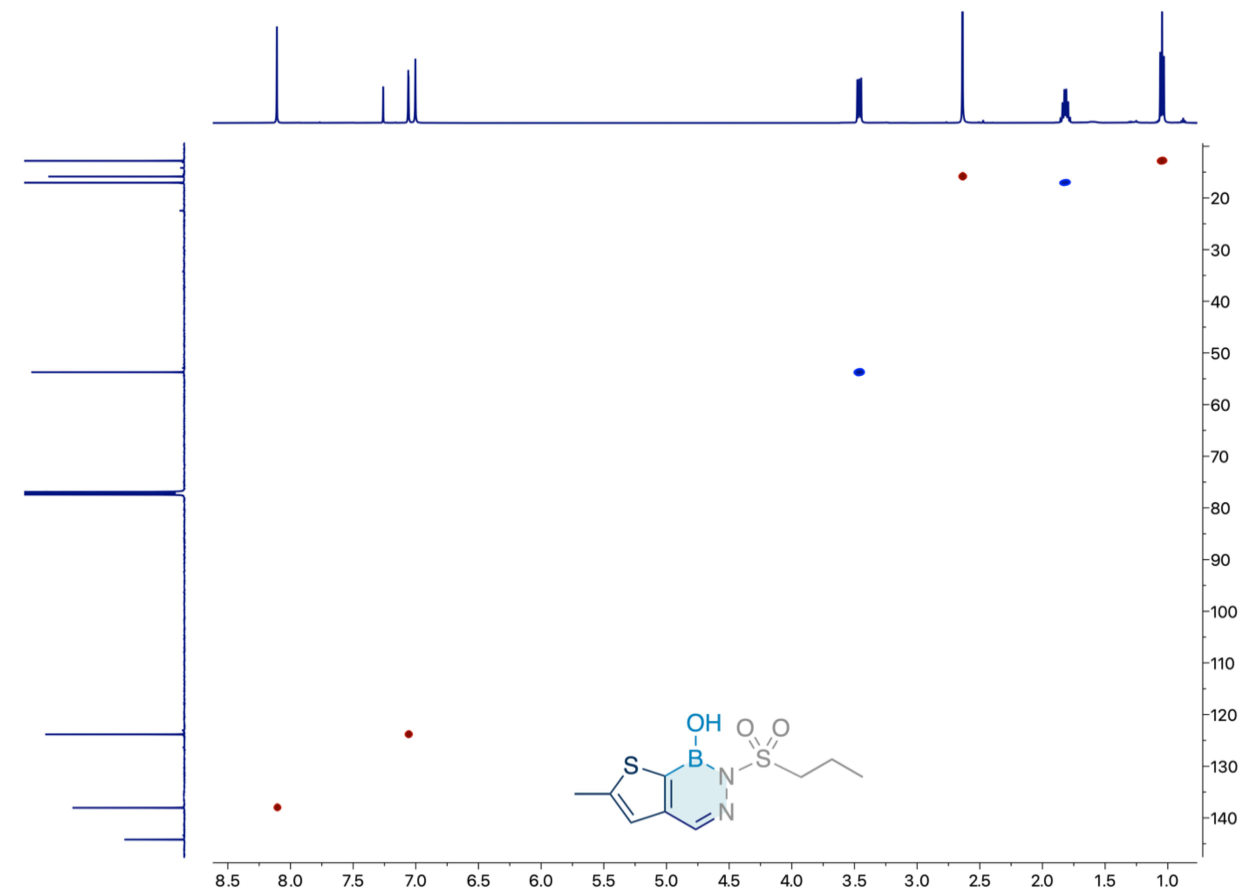

Figure S248. Diazaborine 47: ^{1}H-^{13}C gHSQC NMR ($CDCl_3$, 298 K)

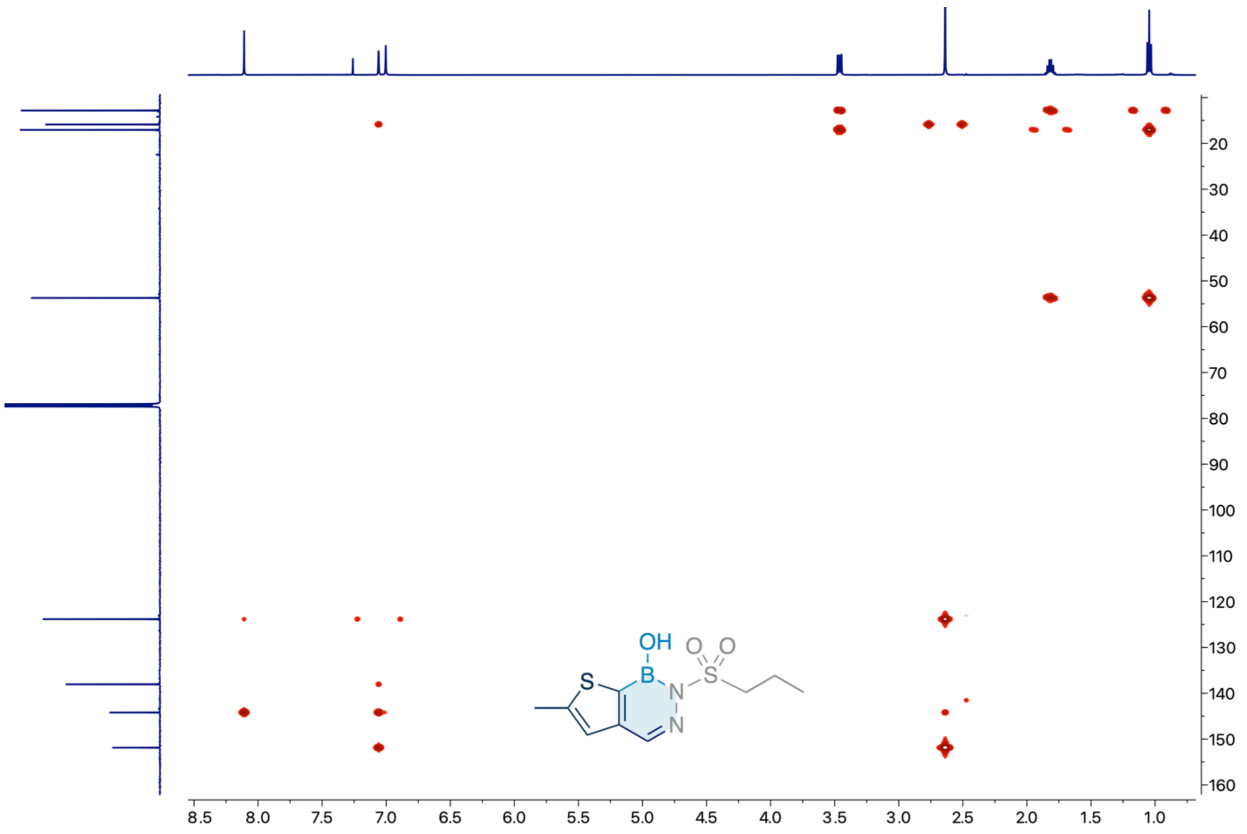

Figure S249. Diazaborine 47: ^{1}H-^{13}C gHMBC NMR ($CDCl_3$, 298 K)

II. Diazaborine derivatives

Diazaborine 48

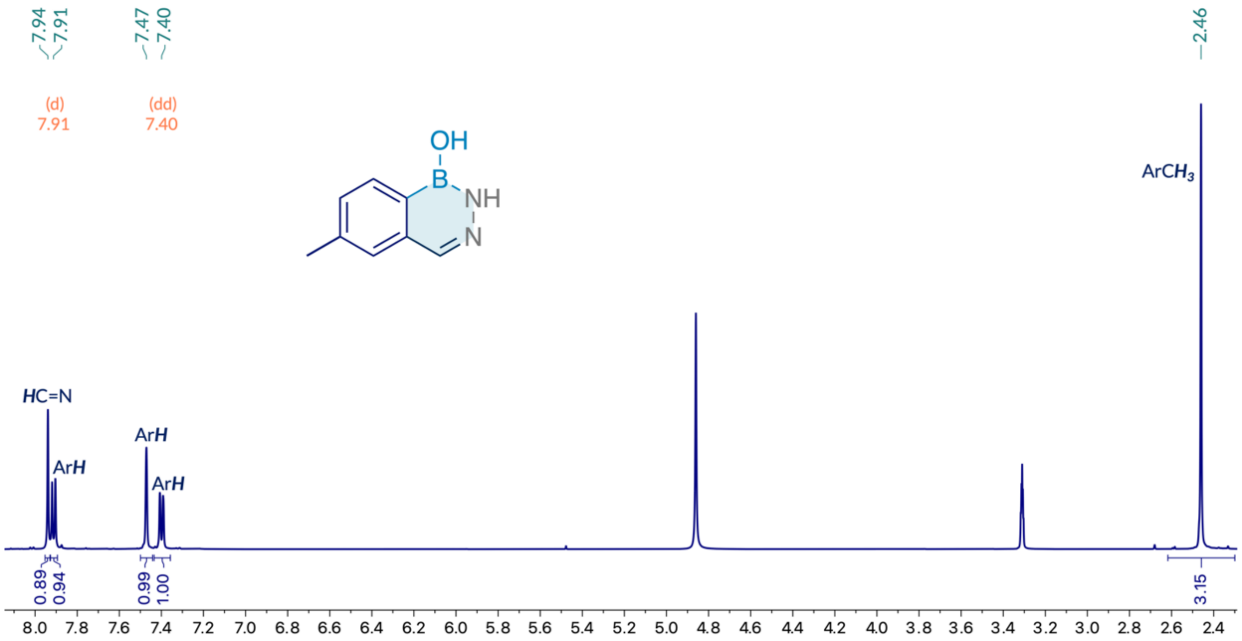

Figure S250. Diazaborine 48:^{1}H NMR (500 MHz, CD_3OD, 298 K)

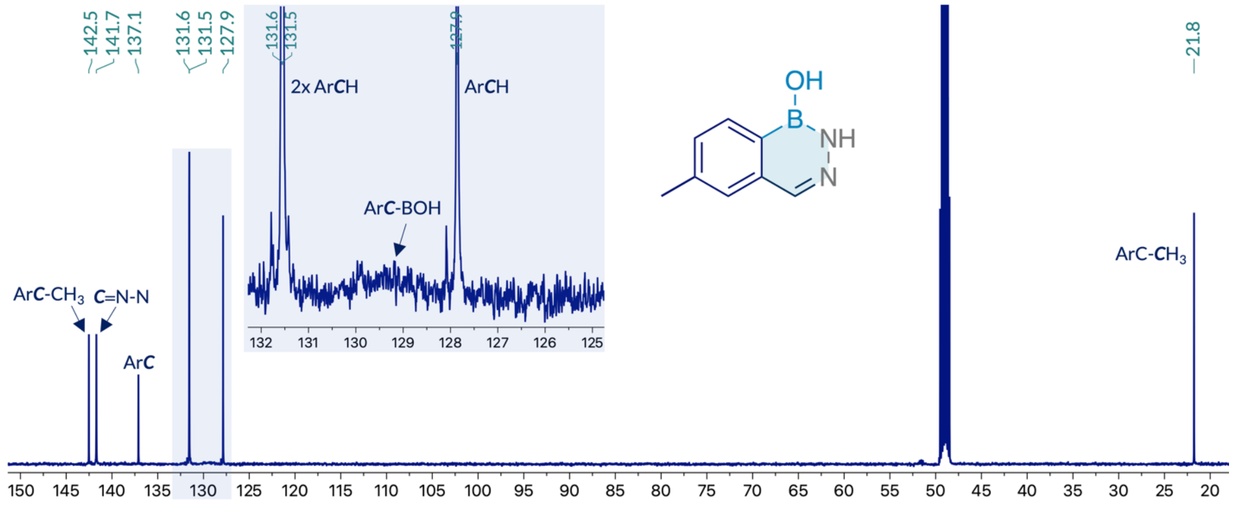

Figure S251. Diazaborine 48:^{13}C NMR (126 MHz, CD_3OD, 298 K)

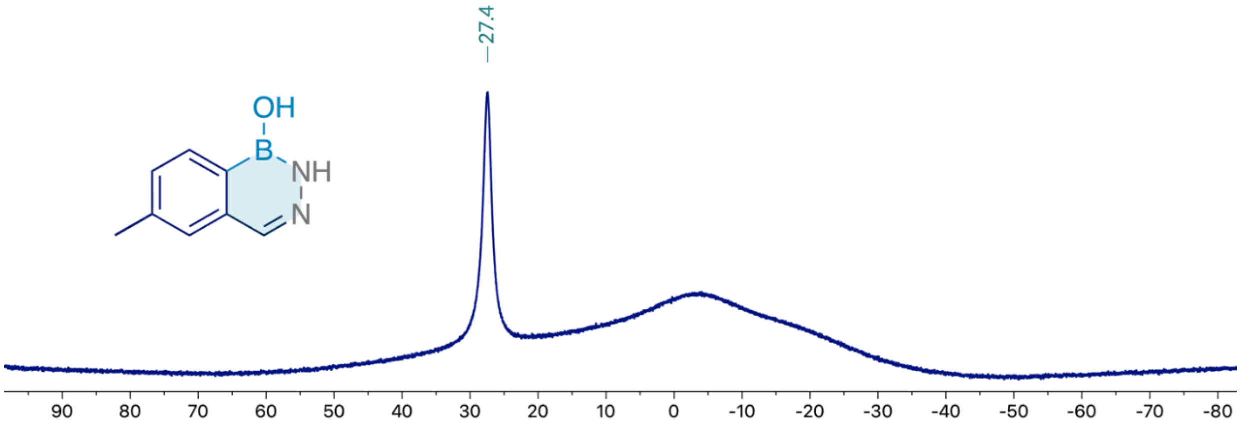

Figure S252. Diazaborine 48:^{11}B NMR (160 MHz, CD_3OD, 298 K)

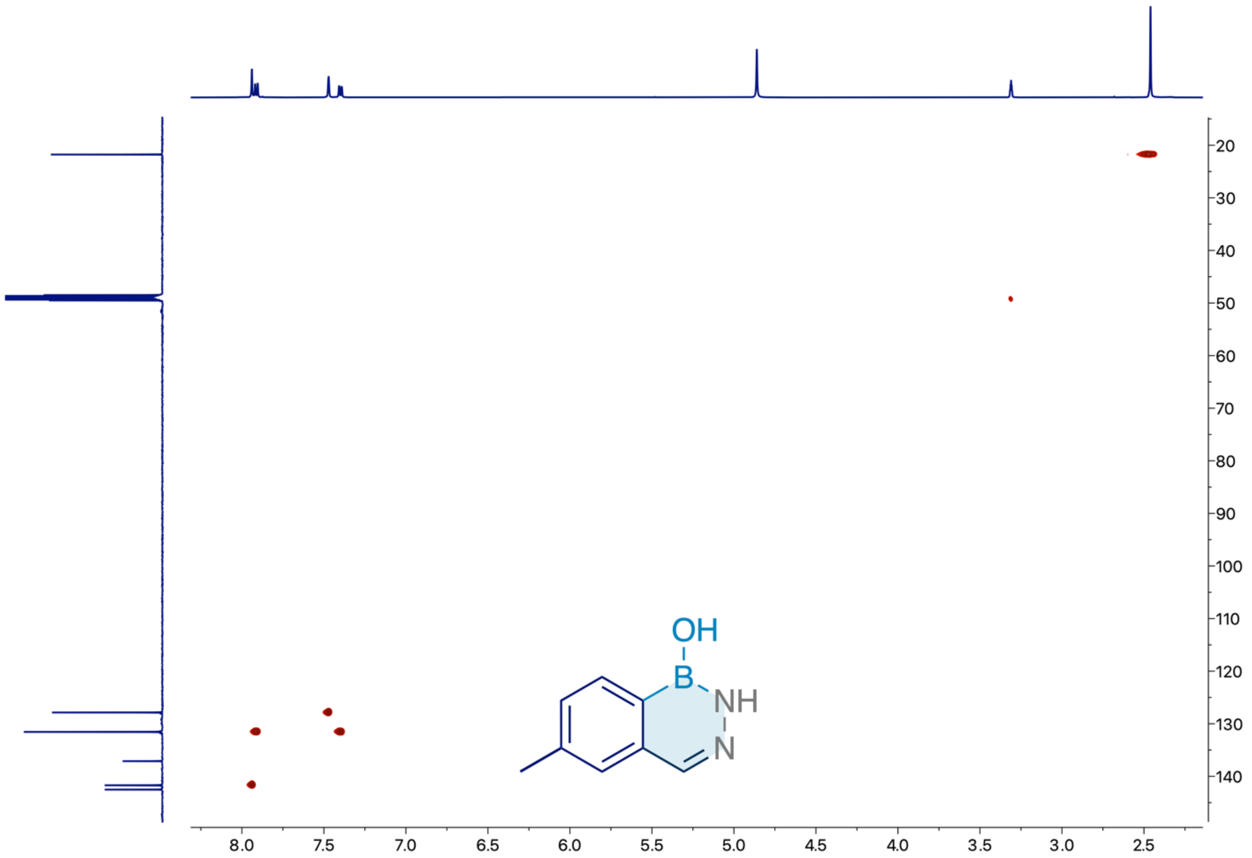

Figure S253. Diazaborine 48: ^{1}H-^{13}C gHSQC NMR (CD_3OD, 298 K)

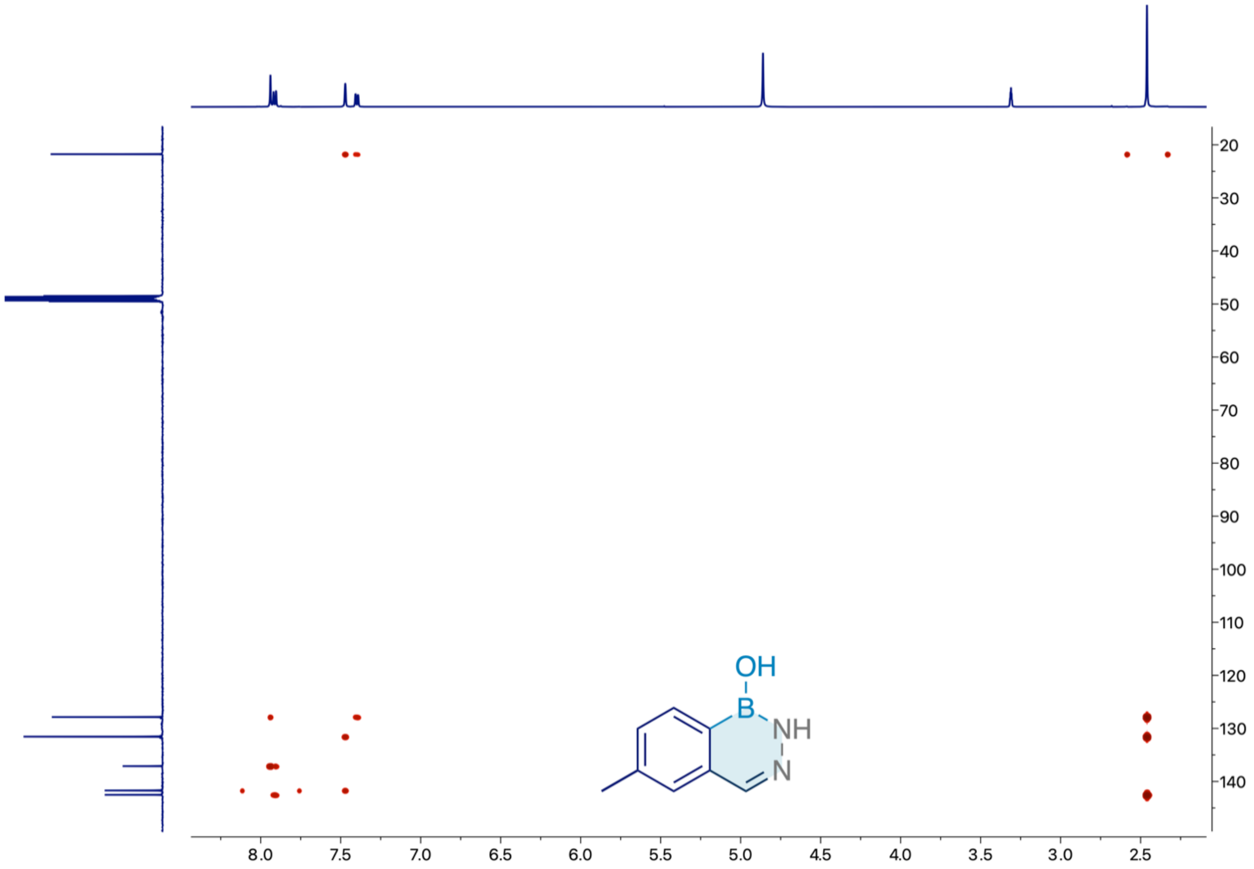

Figure S254. Diazaborine 48: ^{1}H-^{13}C gHMBC NMR (CD_3OD, 298 K)

Tetracyclic diazaborine 49

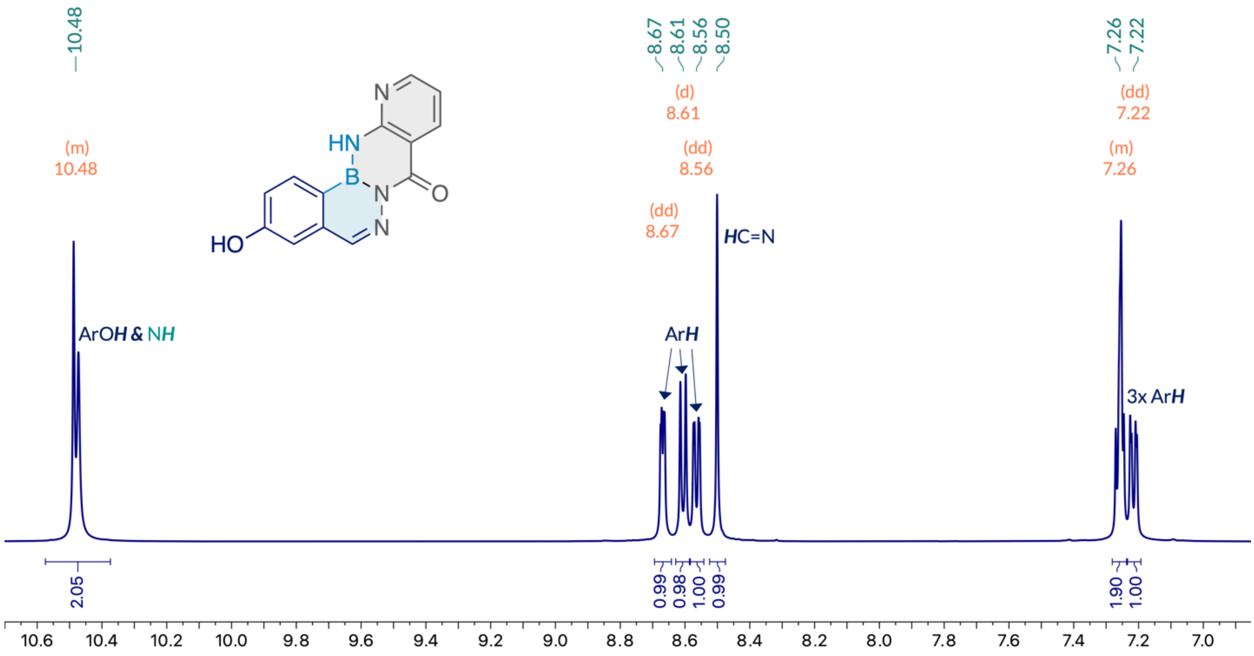

Figure S255. Tetracyclic diazaborine 49:^{1}H NMR (500 MHz, DMSO-d_6, 298 K)

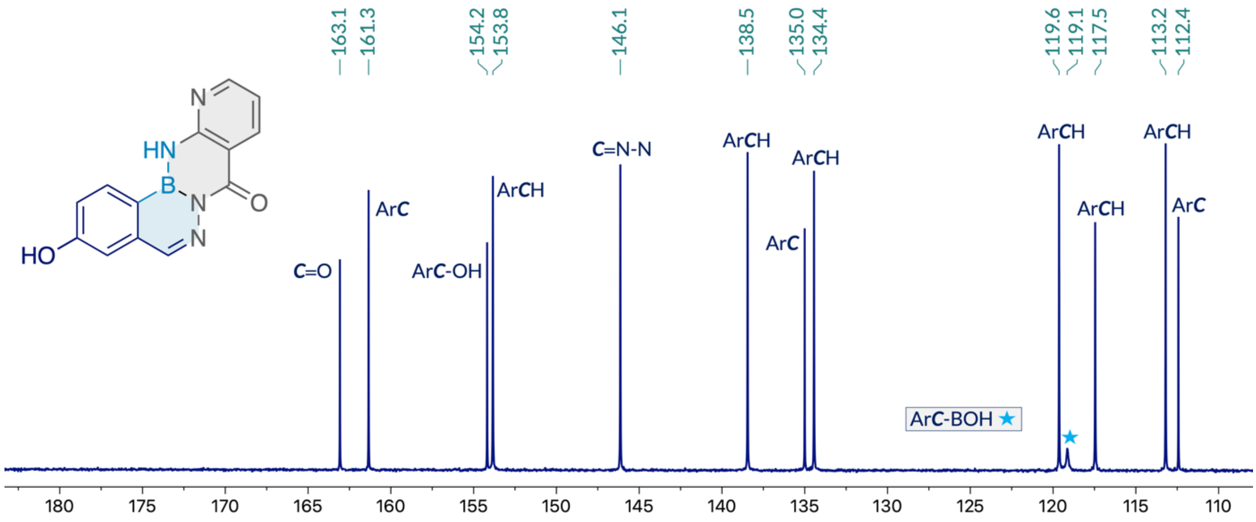

Figure S256. Tetracyclic diazaborine 49:^{13}C NMR (126 MHz, DMSO-d_6, 298 K)

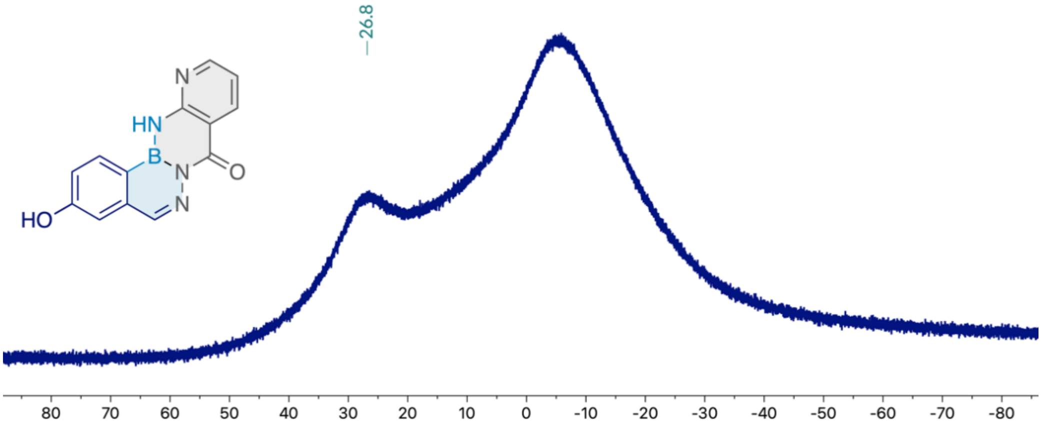

Figure S257. Tetracyclic diazaborine 49:^{11}B NMR (160 MHz, DMSO-d_6, 298 K)

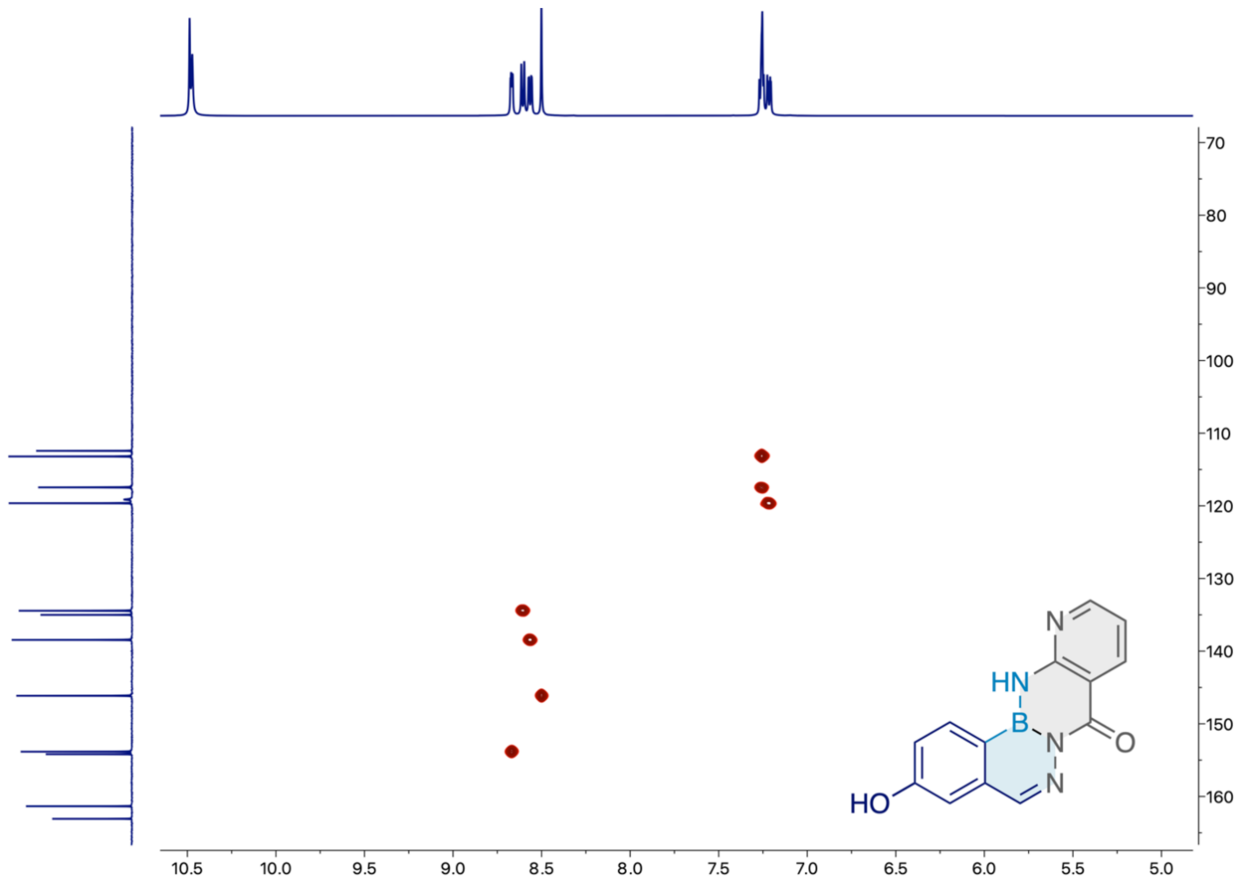

Figure S258. Tetracyclic diazaborine 49: ^{1}H-^{13}C gHSQC NMR (DMSO-d_6, 298 K)

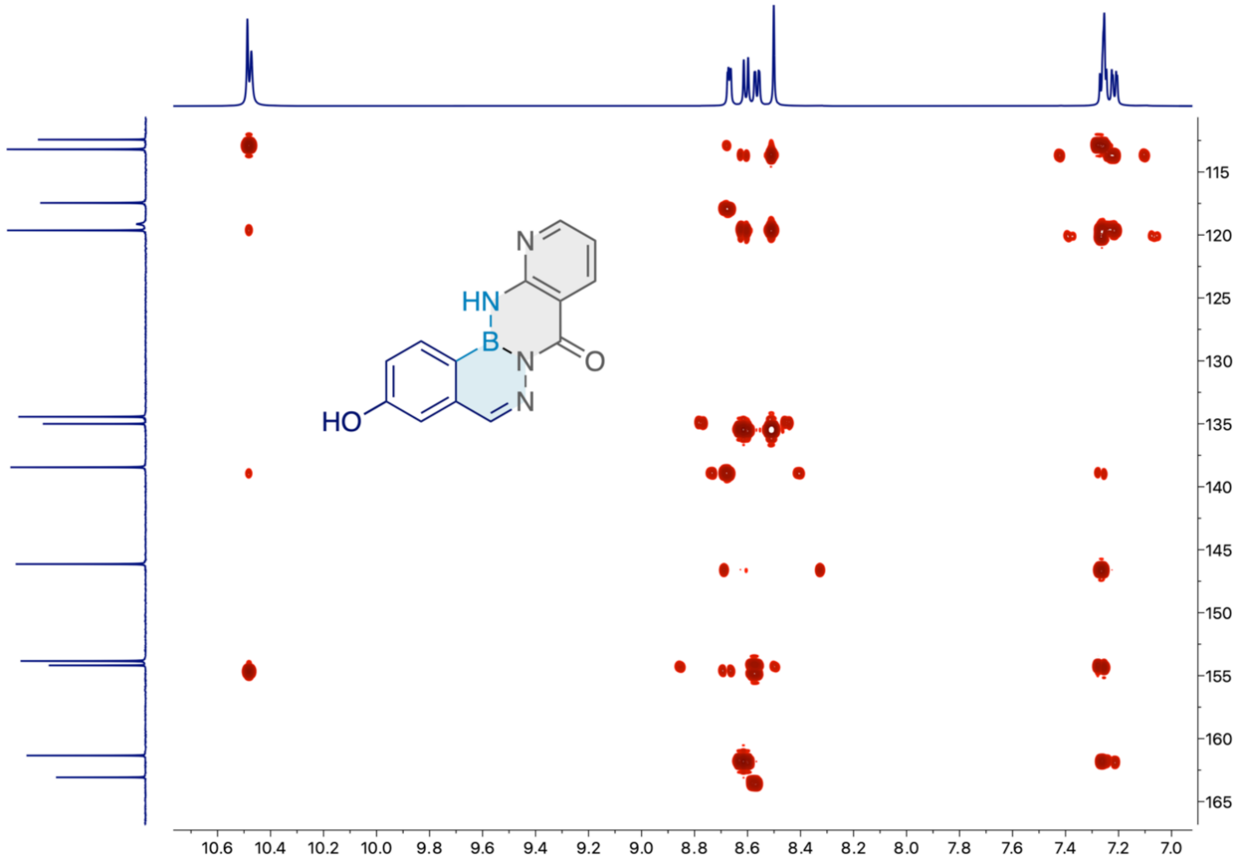

Figure S259. Tetracyclic diazaborine 49: ^{1}H-^{13}C gHMBC NMR (DMSO-d_6, 298 K)

Diazaborine 50

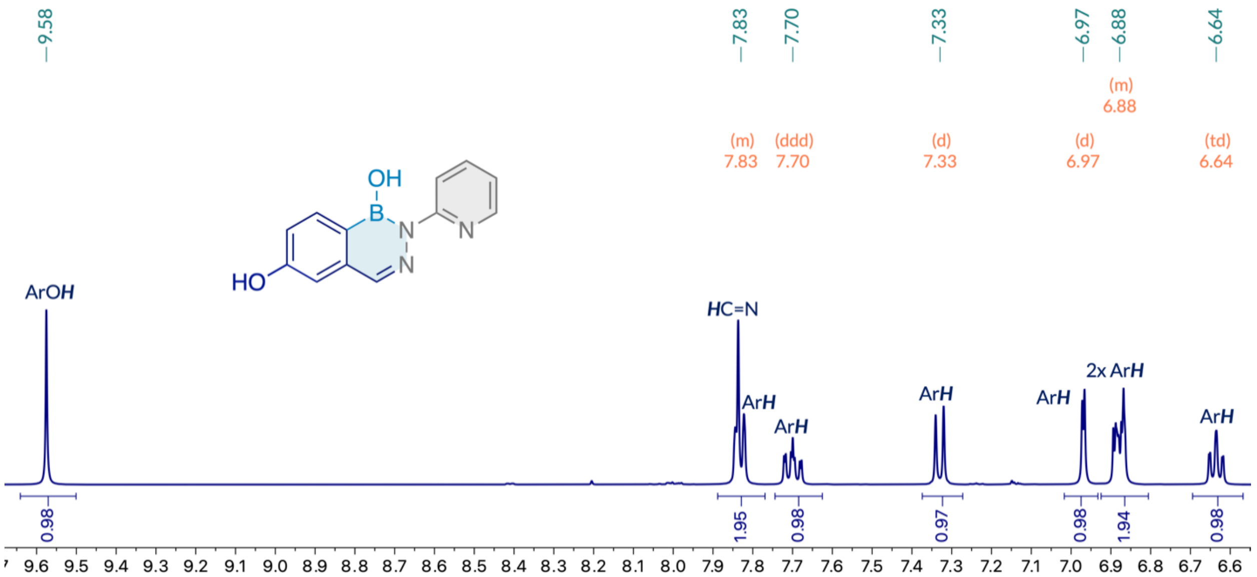

Figure S260. Diazaborine 50:^{1}H NMR (400 MHz, DMSO-d_6, 298 K)

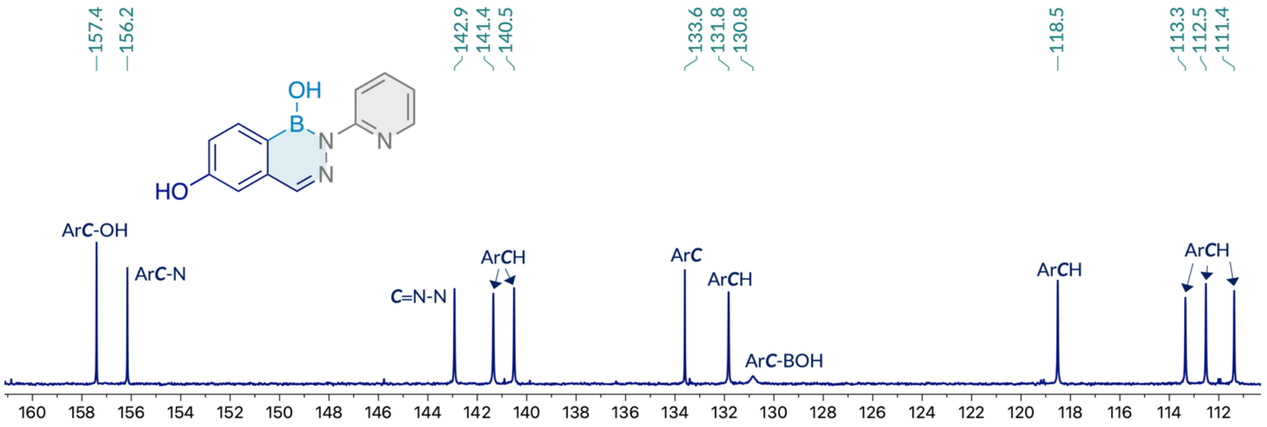

Figure S261. Diazaborine 50:^{13}C NMR (101 MHz, DMSO-d_6, 298 K)

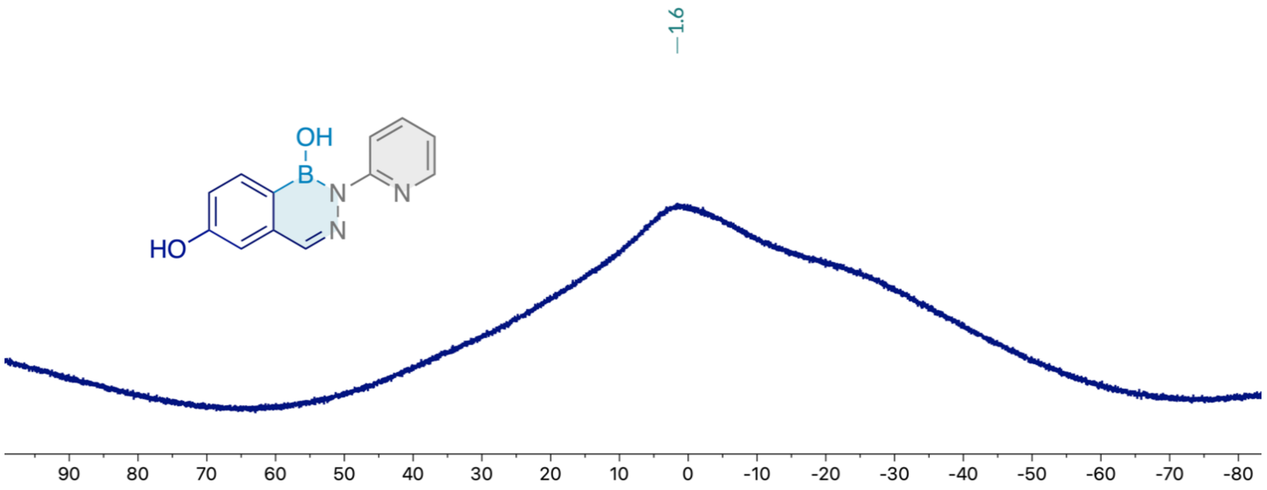

Figure S262. Diazaborine 50:^{11}B NMR (128 MHz, DMSO-d_6, 298 K)

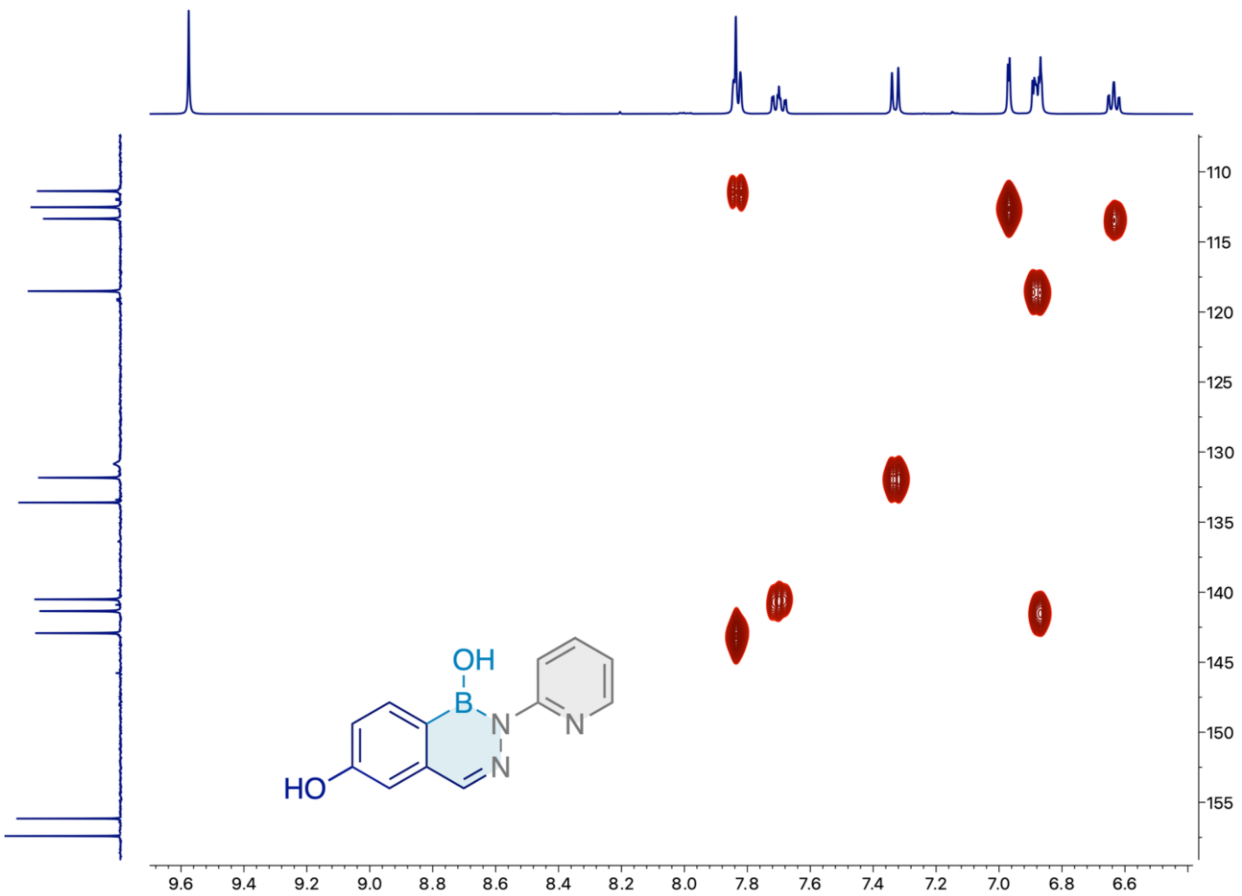

Figure S263. Diazaborine 50: ^{1}H-^{13}C gHSQC NMR (DMSO-d_6, 298 K)

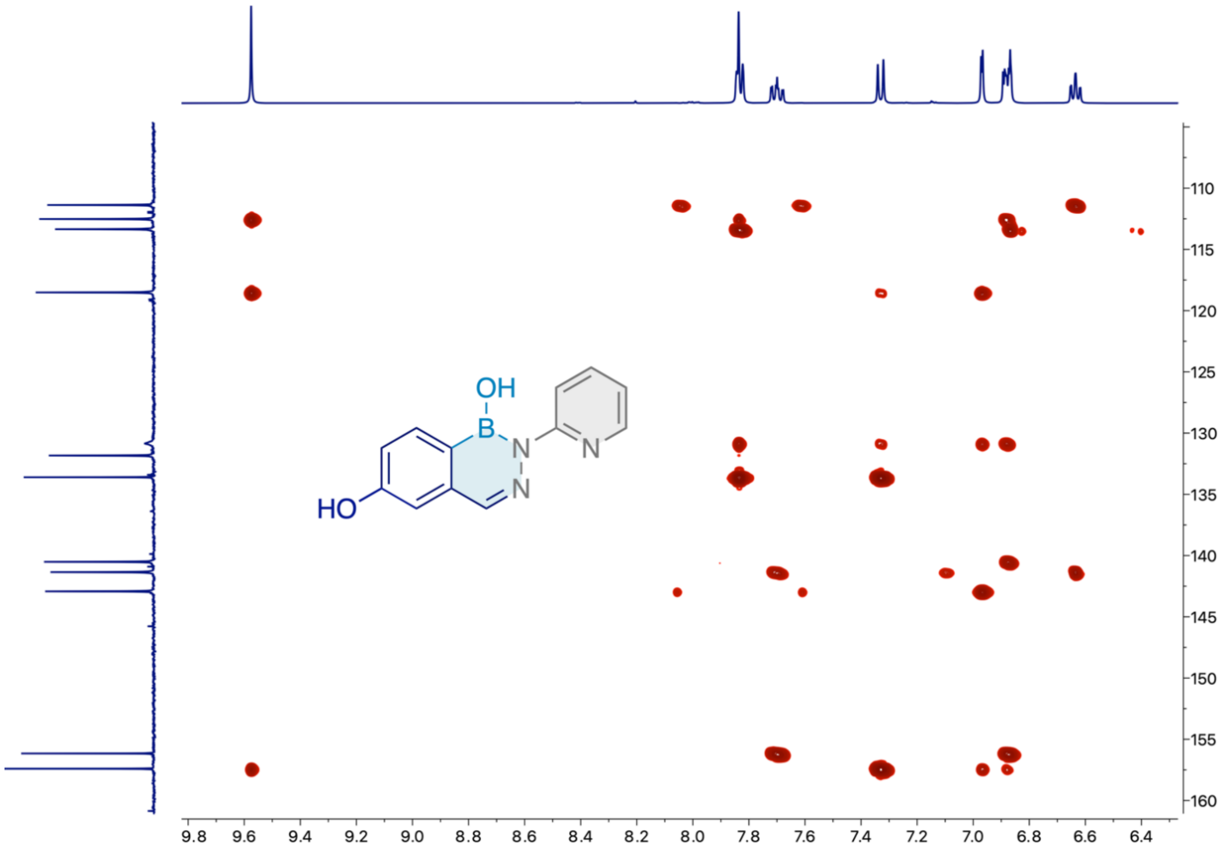

Figure S264. Diazaborine 50: ^{1}H-^{13}C gHMBC NMR (DMSO-d_6, 298 K)

Diazaborine 51

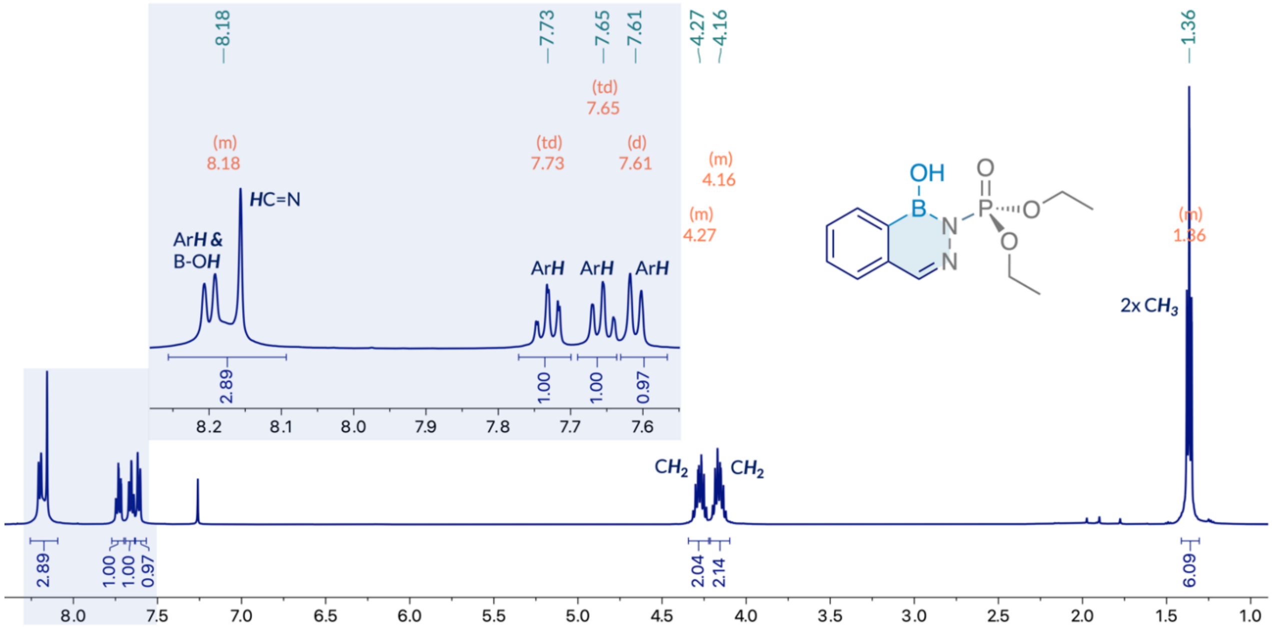

Figure S265. Diazaborine 51:^{1}H NMR (500 MHz, $CDCl_3$, 298 K)

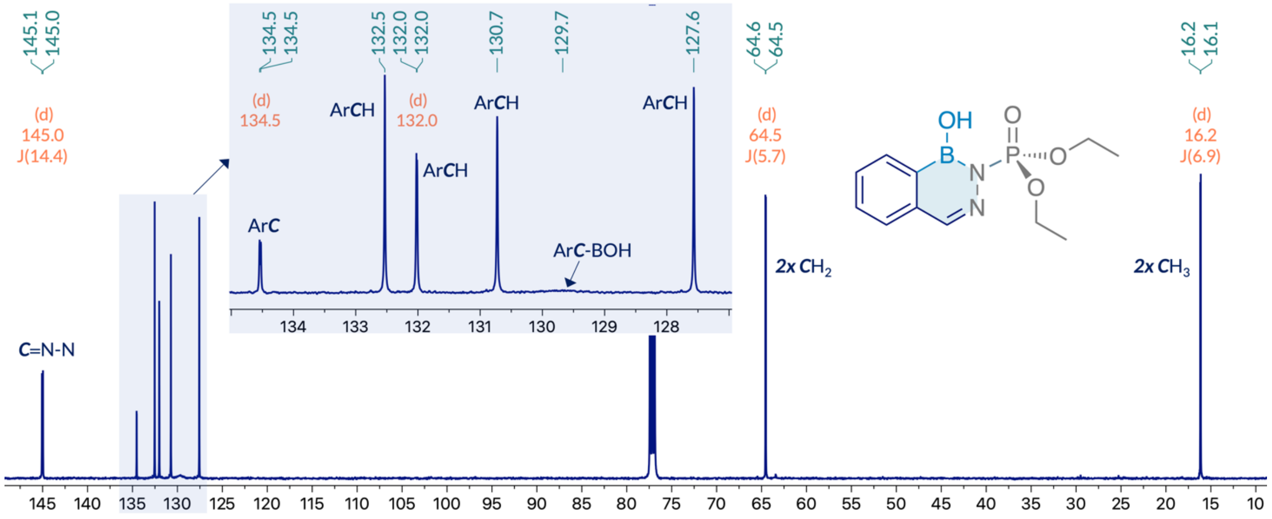

Figure S266. Diazaborine 51:^{13}C NMR (126 MHz, $CDCl_3$, 298 K)

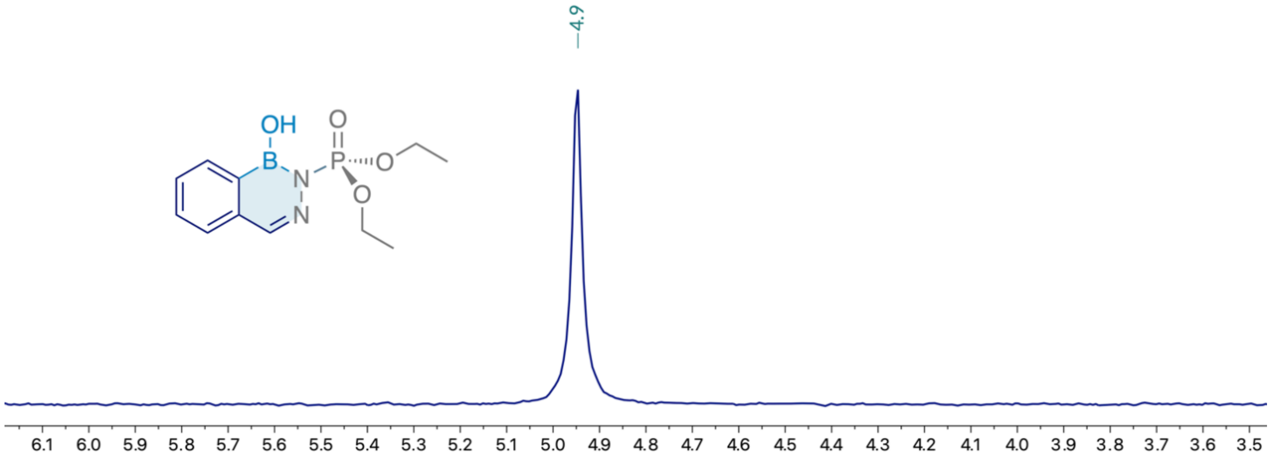

Figure S267. Diazaborine 51:^{31}P{^{1}H} NMR (162 MHz, $CDCl_3$, 298 K)

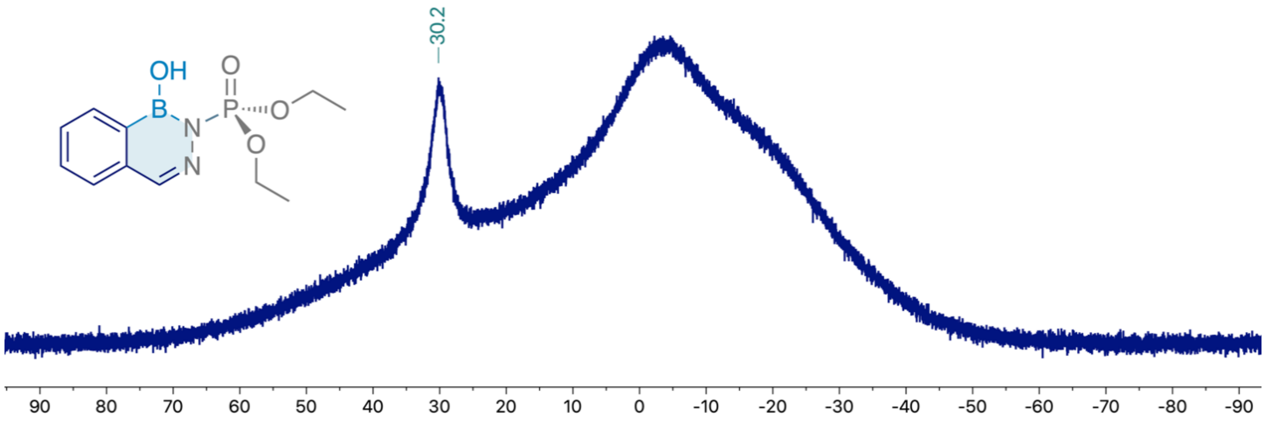

Figure S268. Diazaborine 51:^{11}B NMR (128 MHz, $CDCl_3$, 298 K)

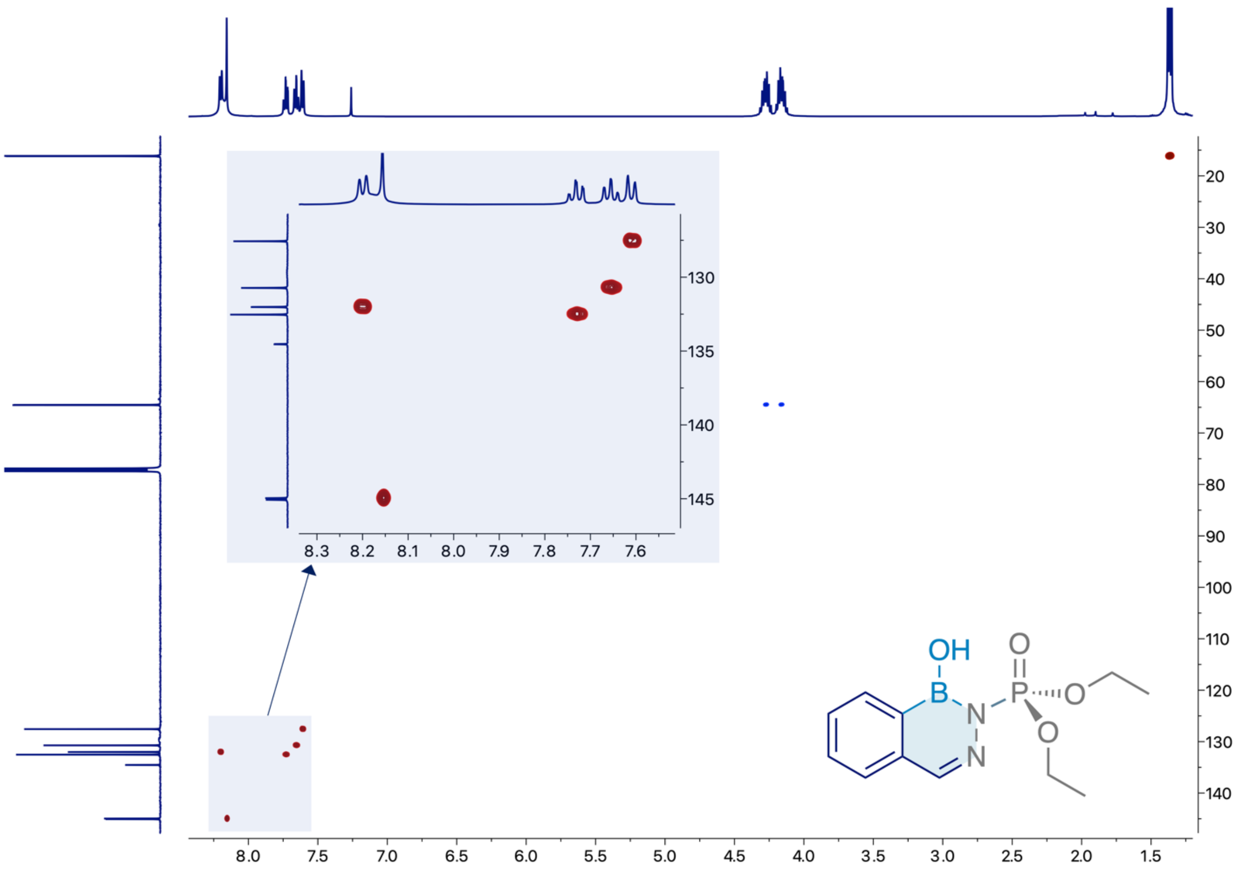

Figure S269. Diazaborine 51: ^{1}H-^{13}C gHSQC NMR ($CDCl_3$, 298 K)

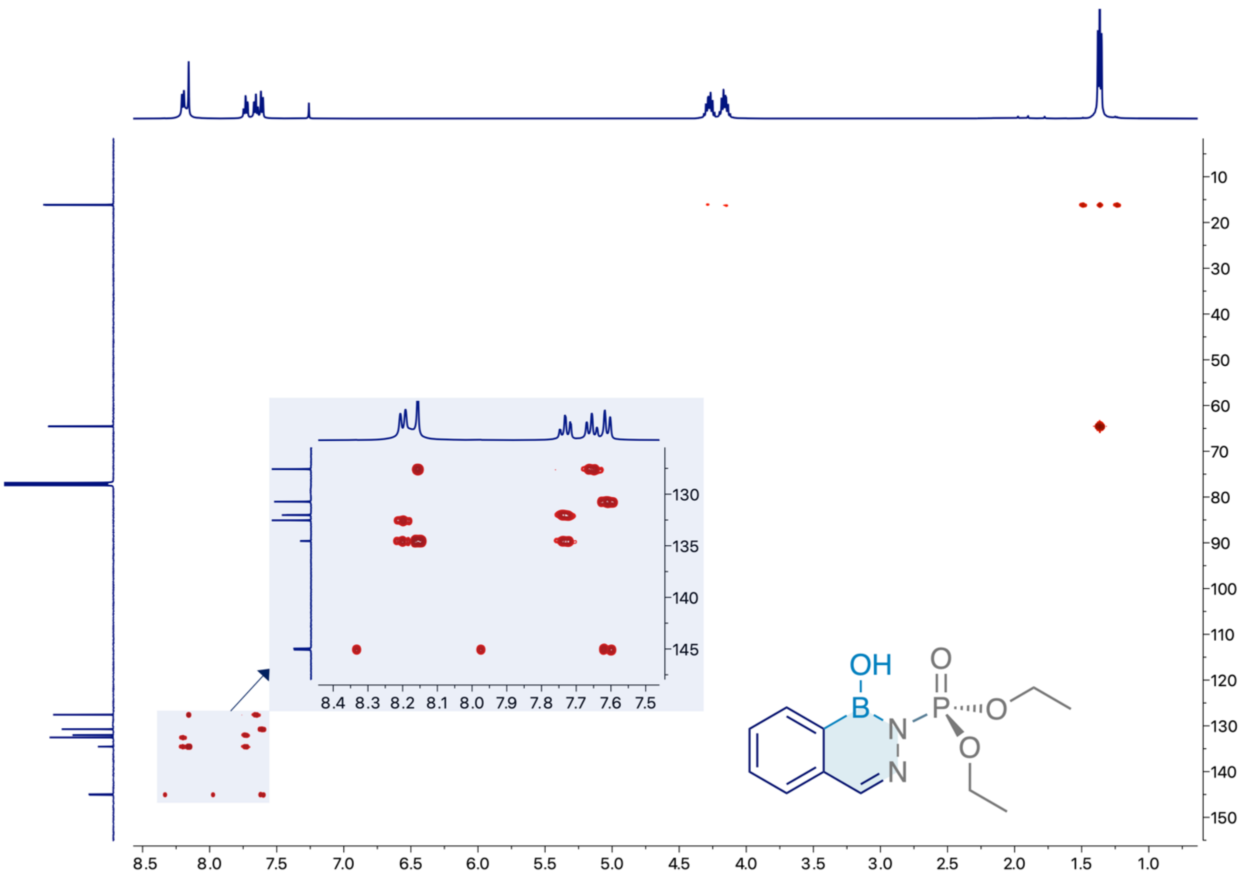

Figure S270. Diazaborine 51: ^{1}H-^{13}C gHMBC NMR ($CDCl_3$, 298 K)

Diazaborine 52

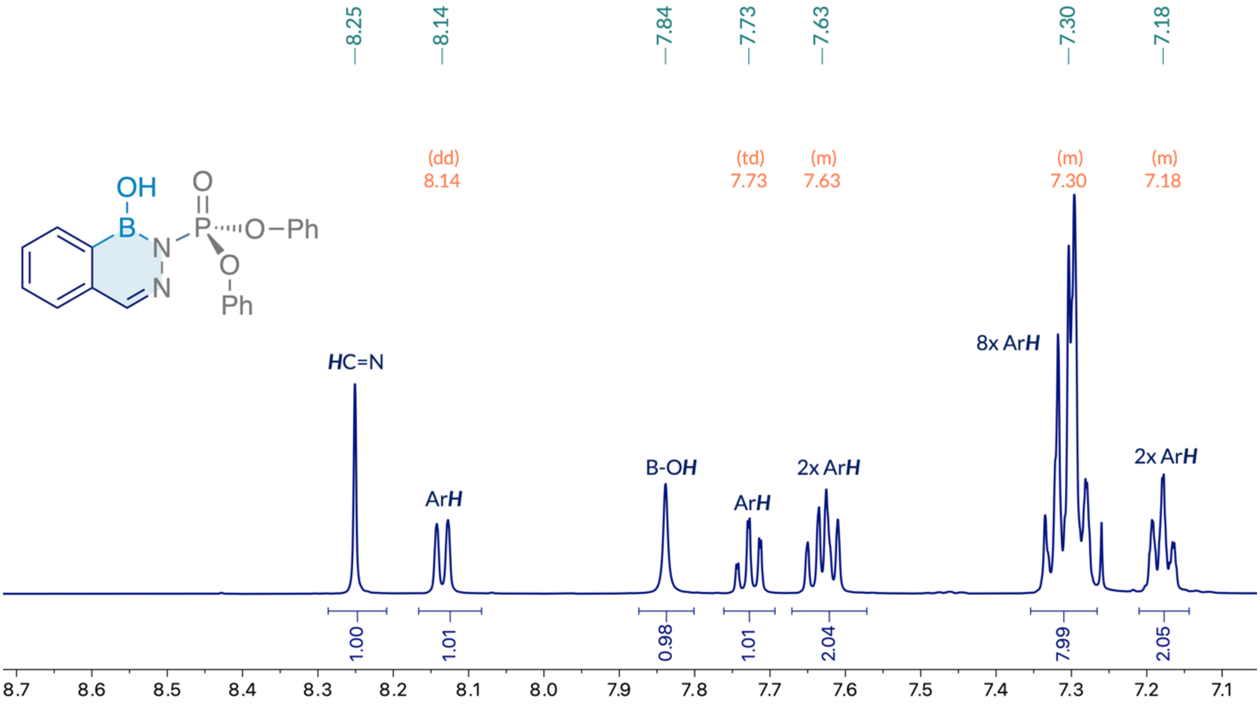

Figure S271. Diazaborine 52:^{1}H NMR (500 MHz, $CDCl_3$, 298 K)

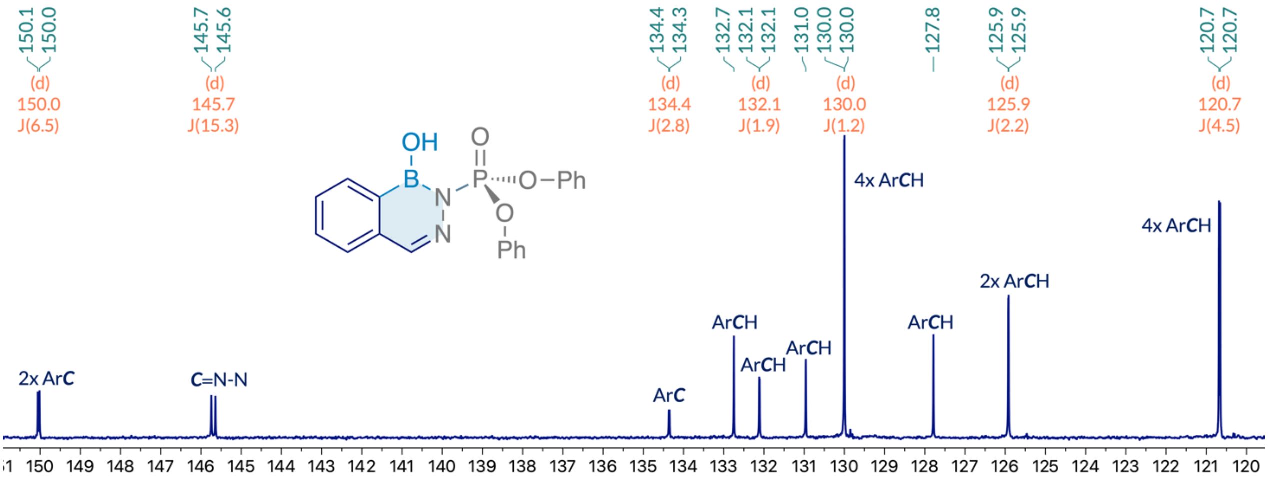

Figure S272. Diazaborine 52:^{13}C NMR (126 MHz, $CDCl_3$, 298 K)

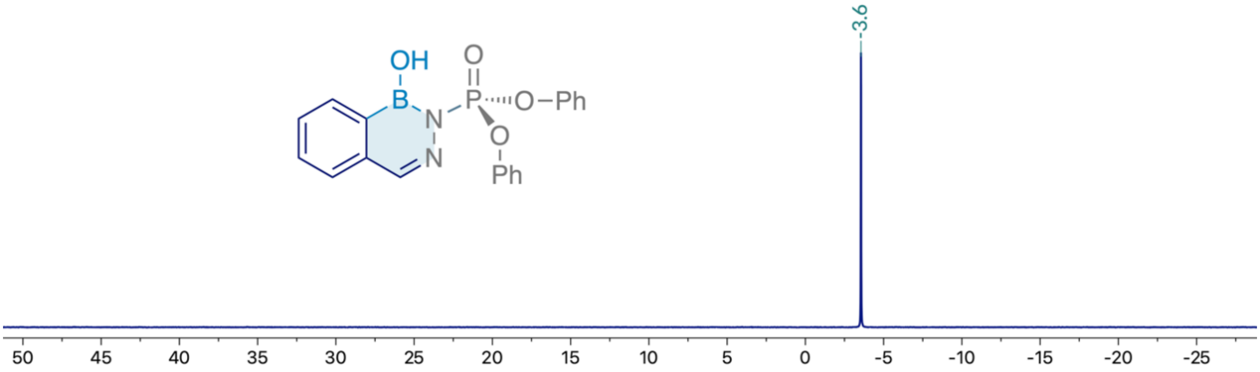

Figure S273. Diazaborine 52:^{31}P{^{1}H} NMR (202 MHz, $CDCl_3$, 298 K)

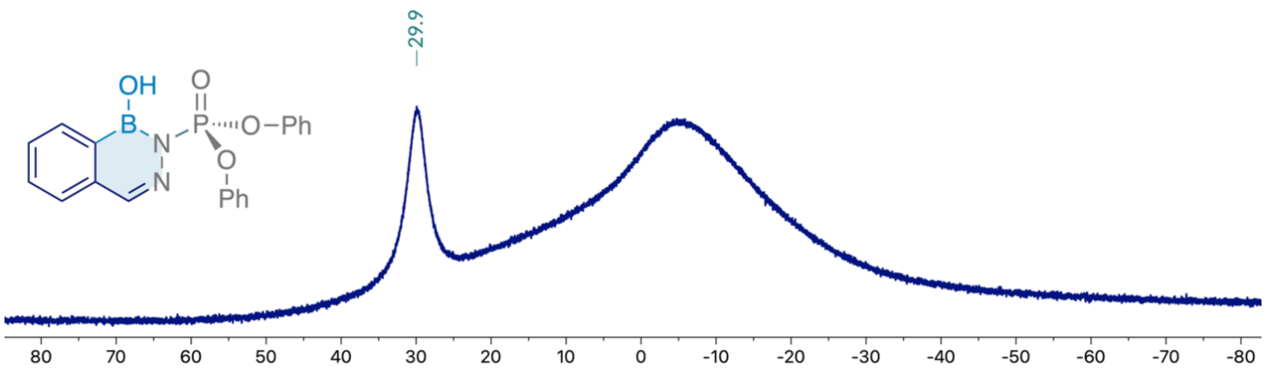

Figure S274. Diazaborine 52:^{11}B NMR (128 MHz, $CDCl_3$, 298 K)

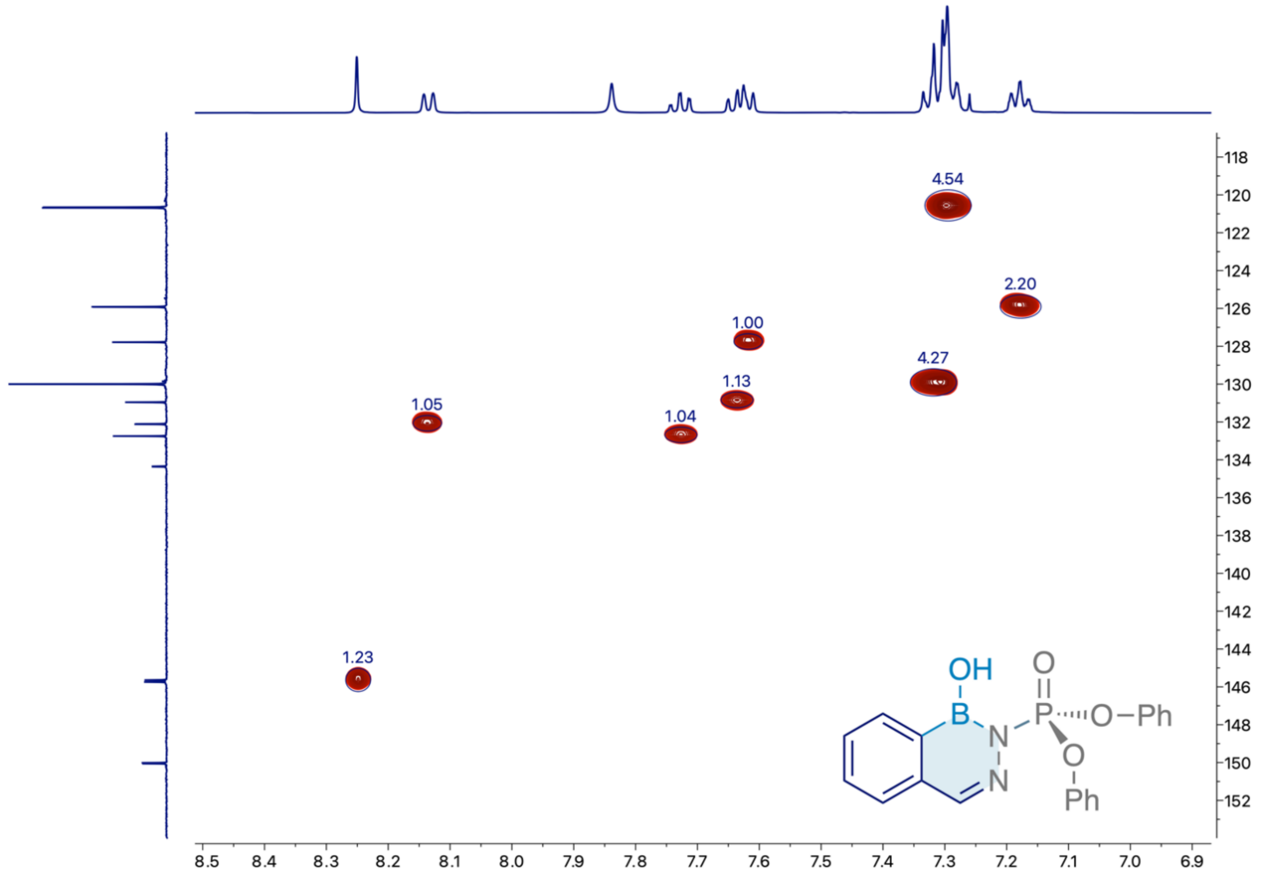

Figure S275. Diazaborine 52: ^{1}H-^{13}C gHSQC NMR ($CDCl_3$, 298 K)

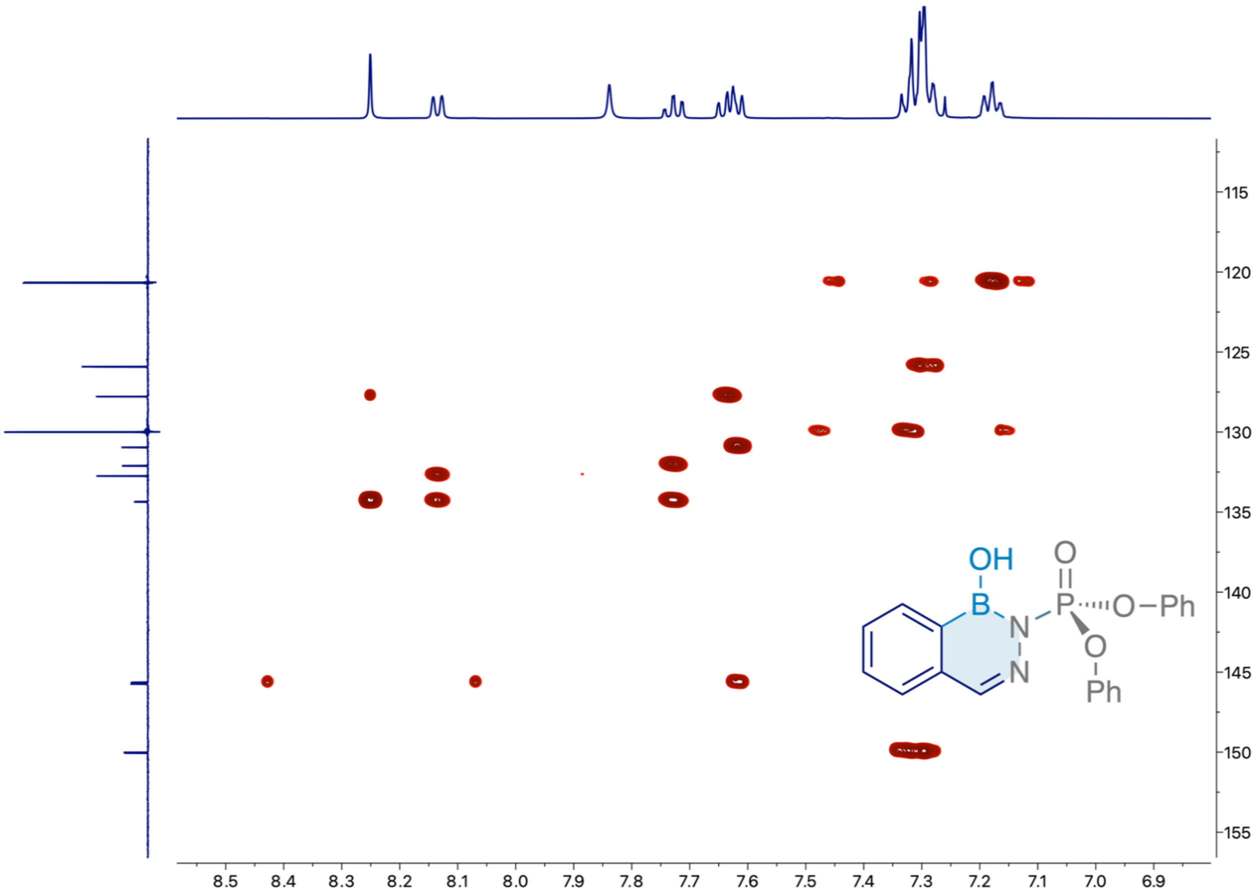

Figure S276. Diazaborine 52: ^{1}H-^{13}C gHMBC NMR ($CDCl_3$, 298 K)

Diazaborine 53

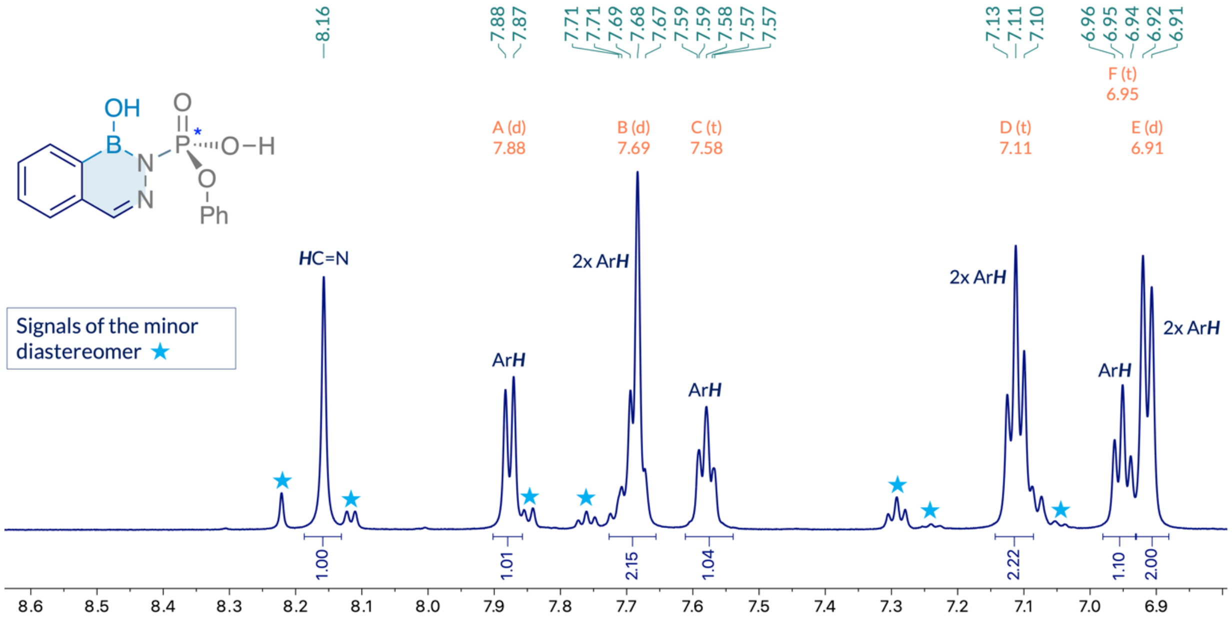

Figure S277. Diazaborine 53:^{1}H NMR (500 MHz, DMSO-d_6 :D_2O 1:1, 298 K)

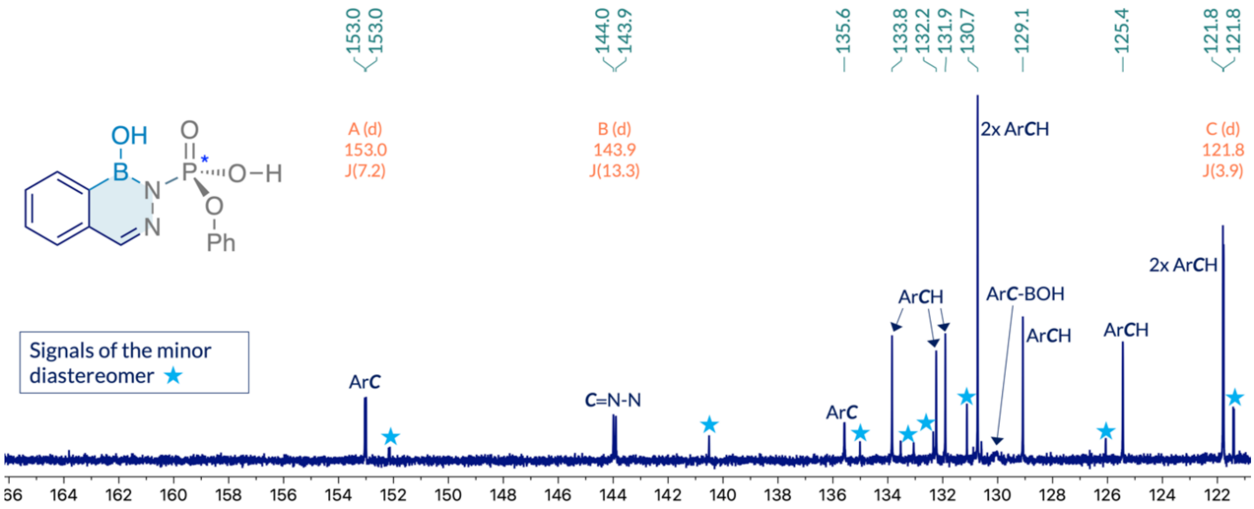

Figure S278. Diazaborine 53:^{13}C NMR (126 MHz, DMSO-d_6 :D_2O 1:1, 298 K)

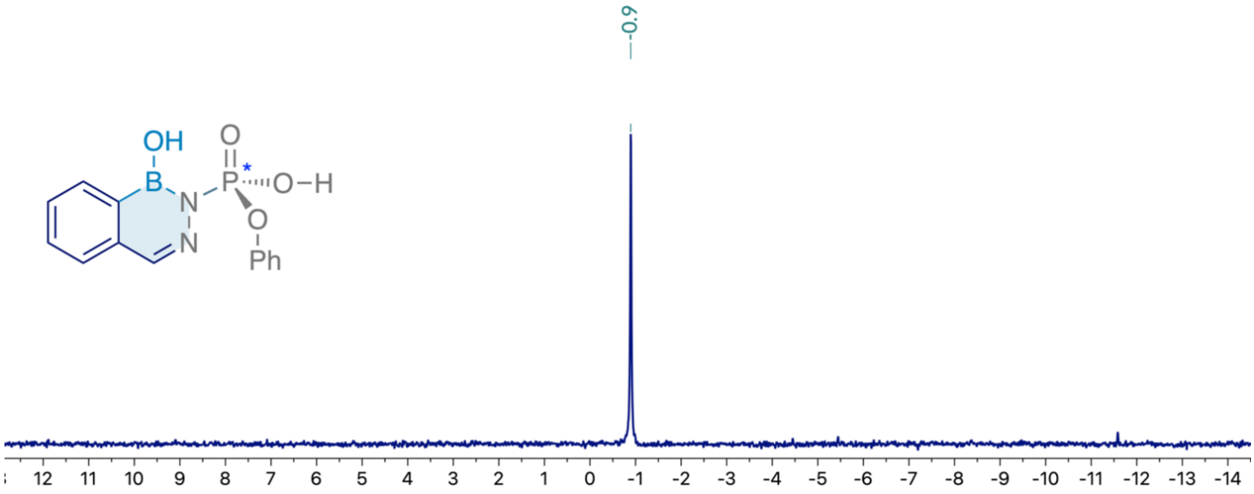

Figure S279. Diazaborine 53: ^{31}P{^{1}H} NMR (202 MHz, DMF-d_7, 298 K)

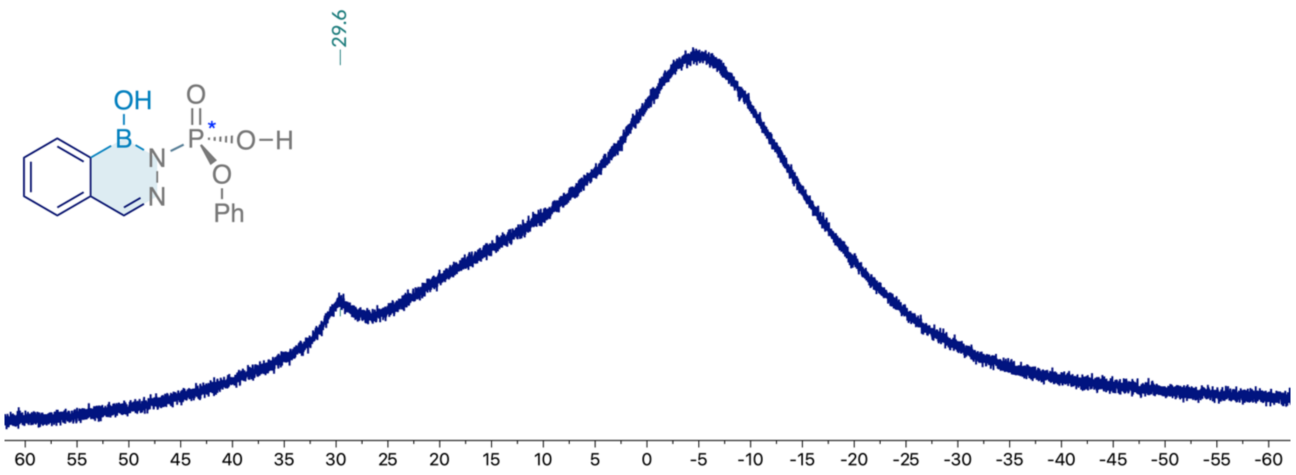

Figure S280. Diazaborine 53: ^{11}B NMR (160 MHz, DMF-d_7, 298 K)

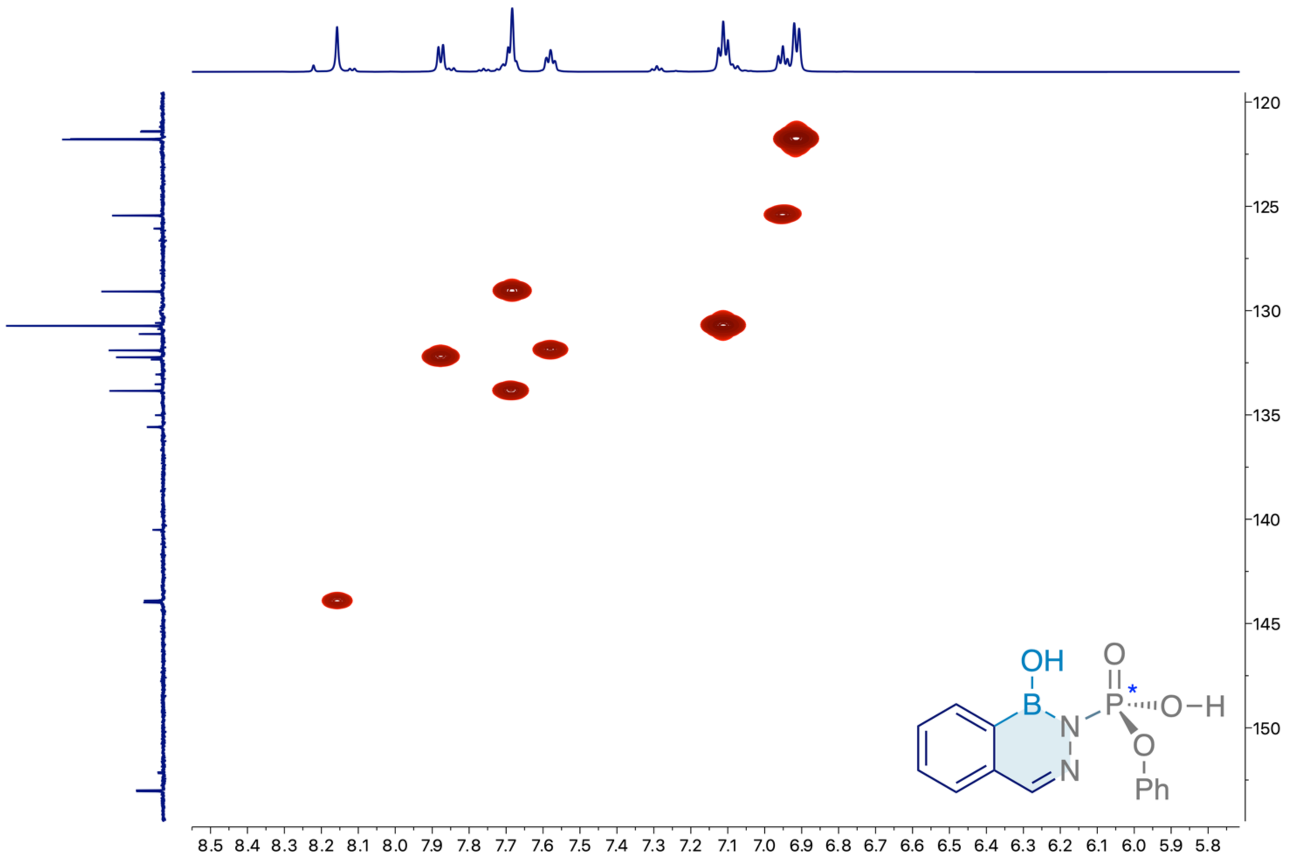

Figure S281. Diazaborine 53: ^{1}H-^{13}C gHSQC NMR (DMSO-d_6 :D_2O 1:1, 298 K)

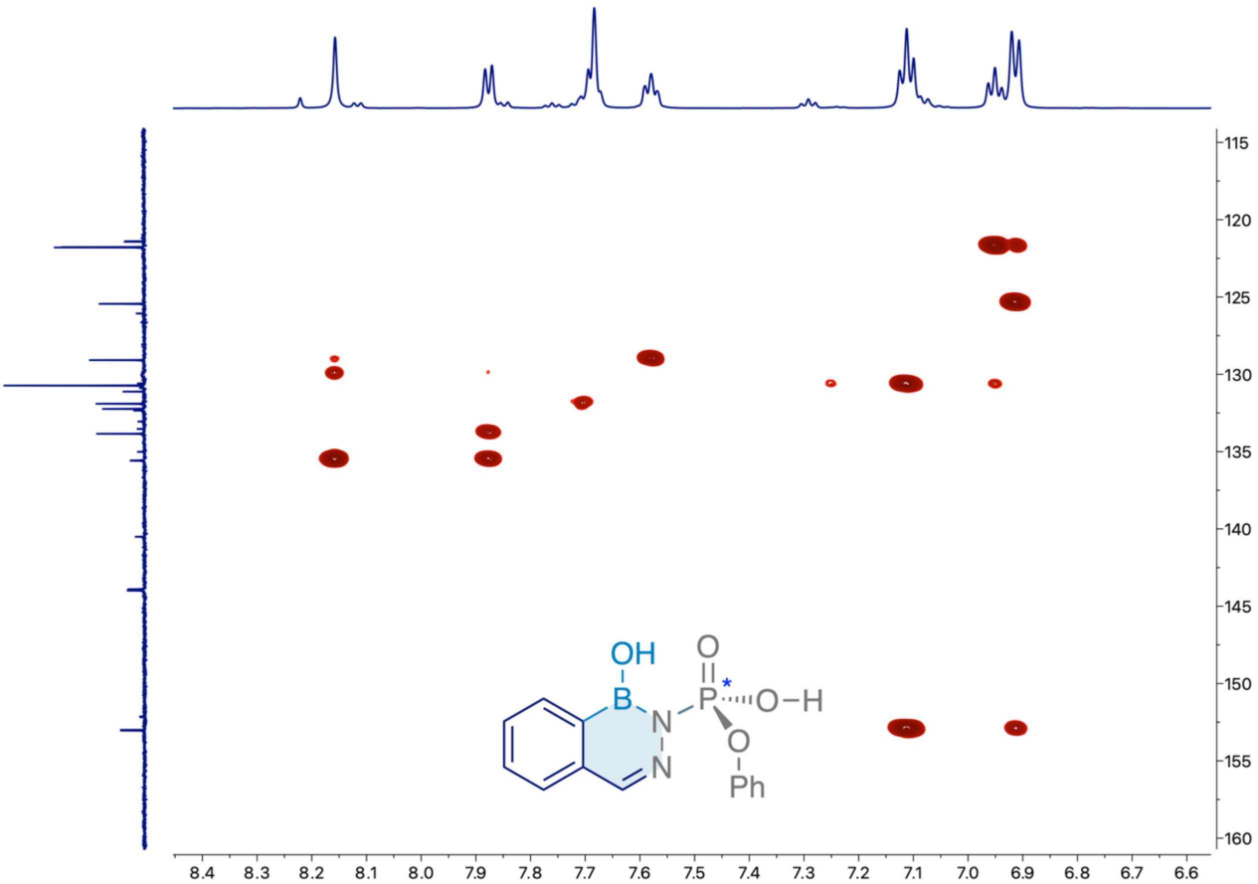

Figure S282. Diazaborine 53: ^{1}H-^{13}C gHMBC NMR (DMSO-d_6:D_2O 1:1, 298 K)

Diazaborine 54

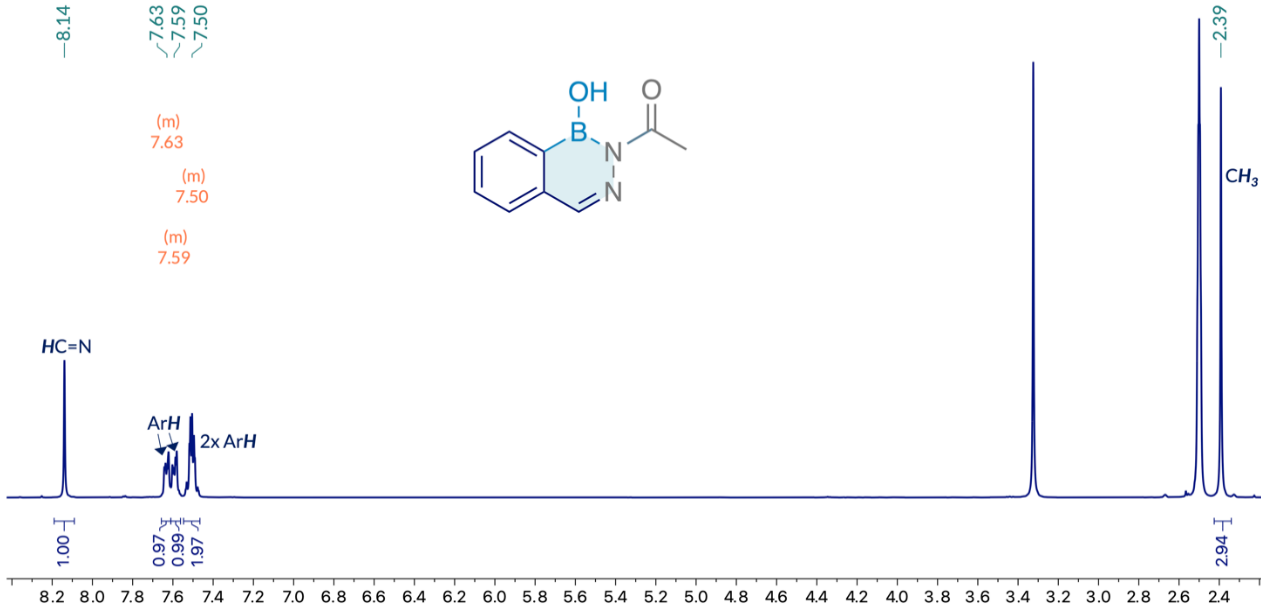

Figure S283. Diazaborine 54: ^{1}H NMR (400 MHz, DMSO-d_6, 298 K)

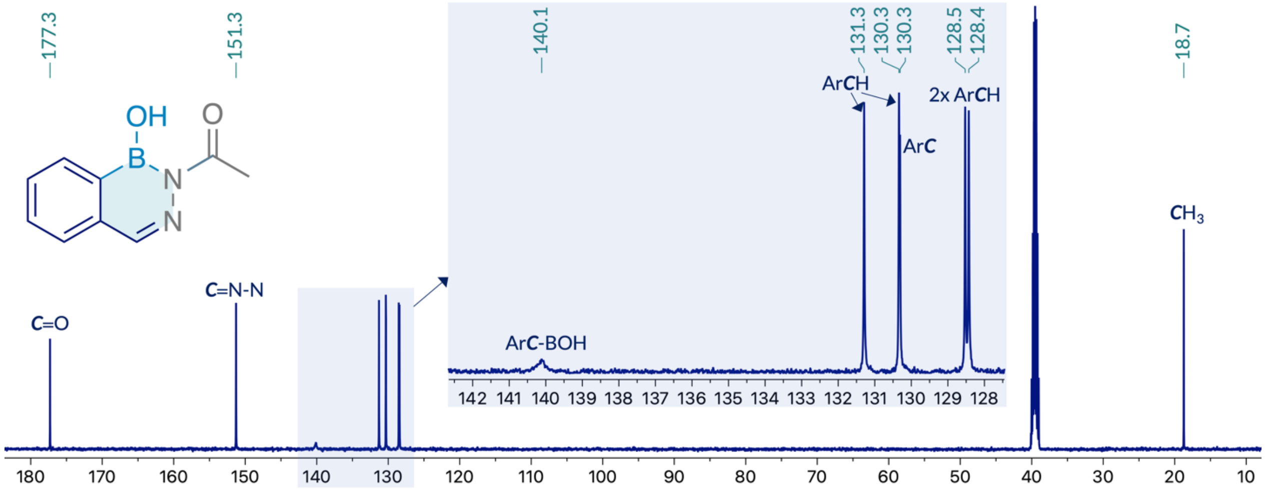

Figure S284. Diazaborine 54: ^{13}C NMR (126 MHz, DMSO-d_6, 298 K)

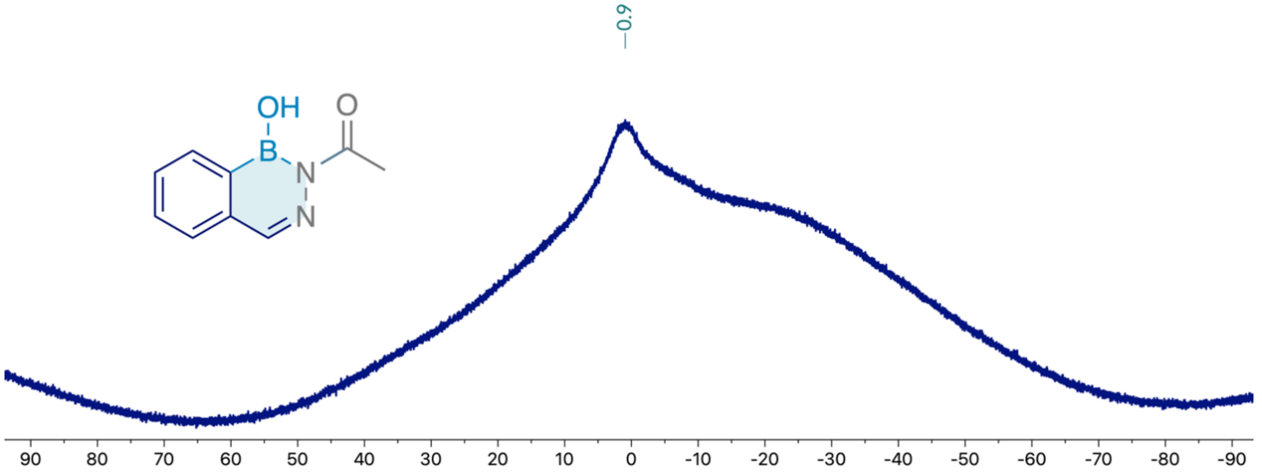

Figure S285. Diazaborine 54: ^{11}B NMR (128 MHz, DMSO-d_6, 298 K)

Diazaborine 55

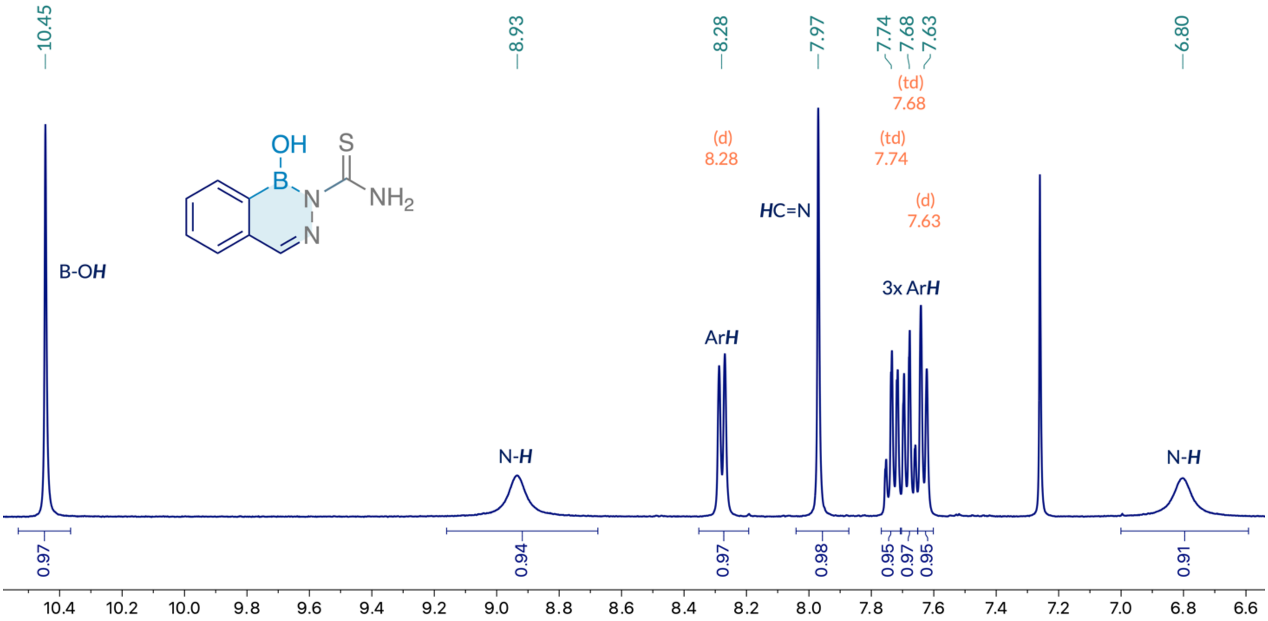

Figure S286. Diazaborine 55:^{1}H NMR (400 MHz, $CDCl_3$, 298 K)

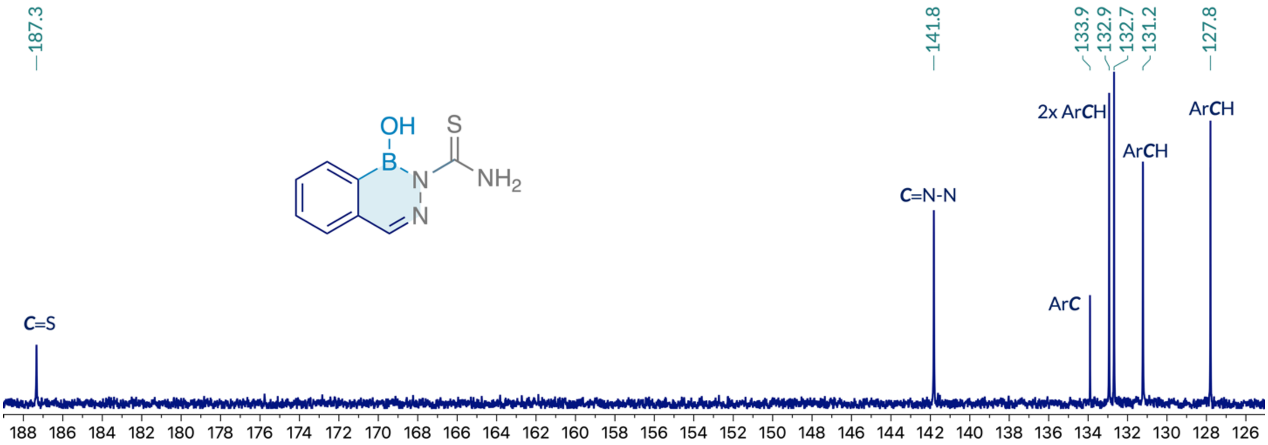

Figure S287. Diazaborine 55:^{13}C NMR (101 MHz, $CDCl_3$, 298 K)

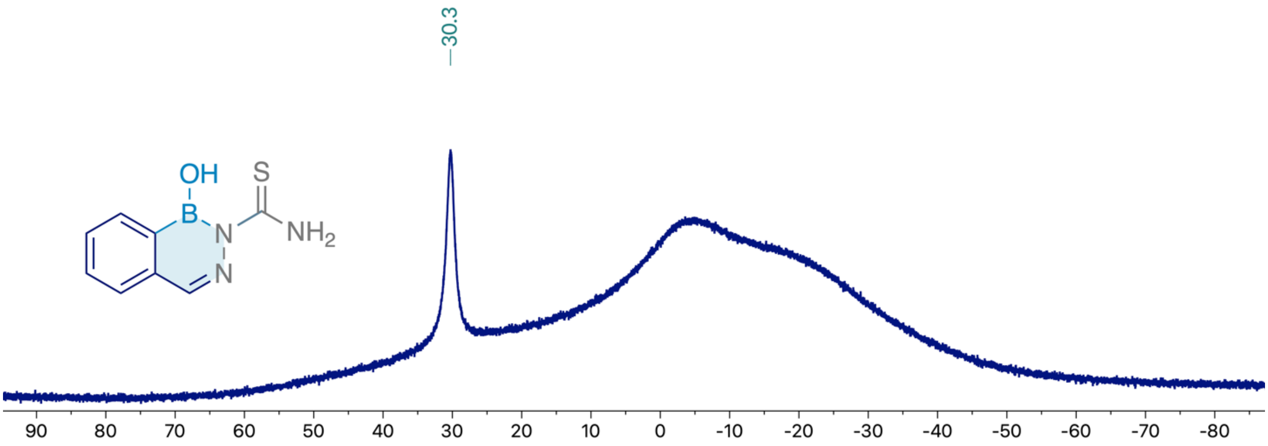

Figure S288. Diazaborine 55:^{11}B NMR (128 MHz, $CDCl_3$, 298 K)

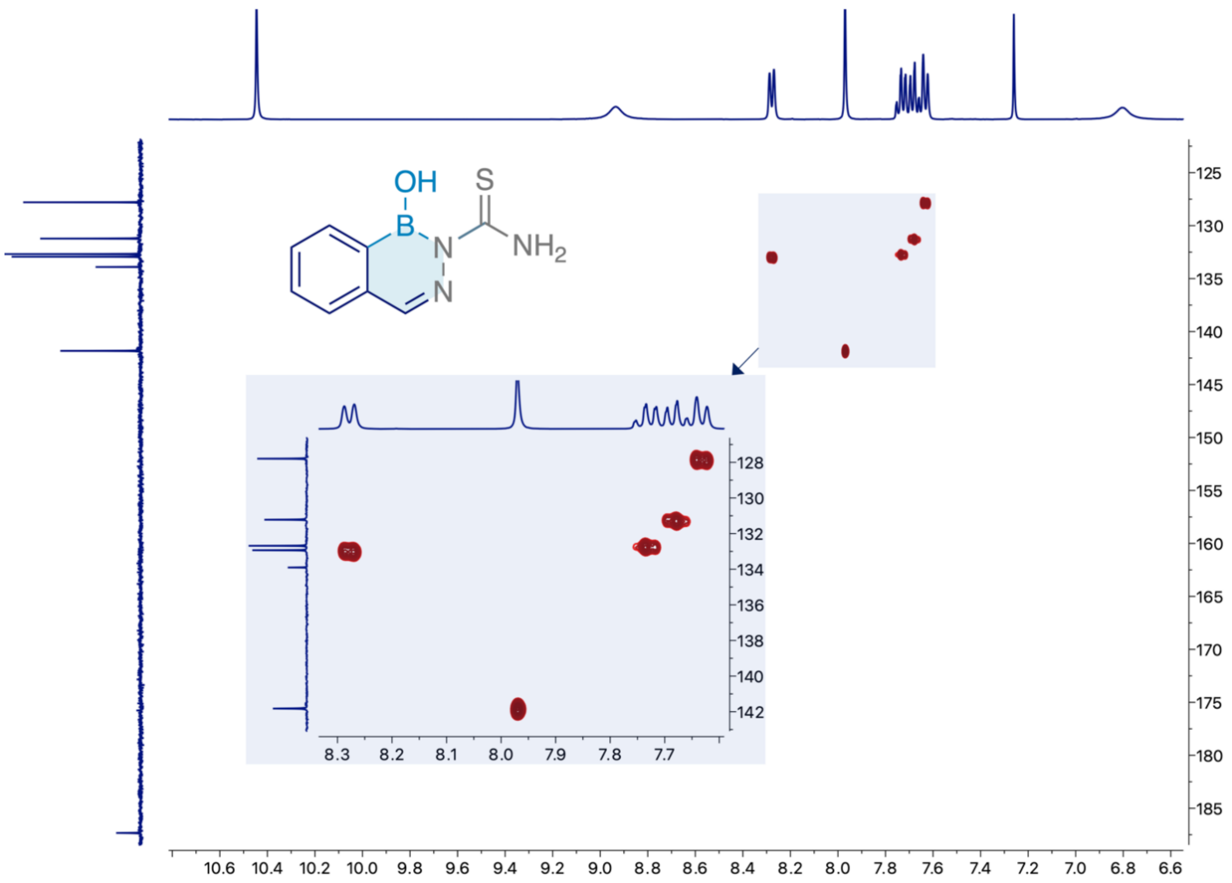

Figure S289. Diazaborine 55: ^{1}H-^{13}C gHSQC NMR ($CDCl_3$, 298 K)

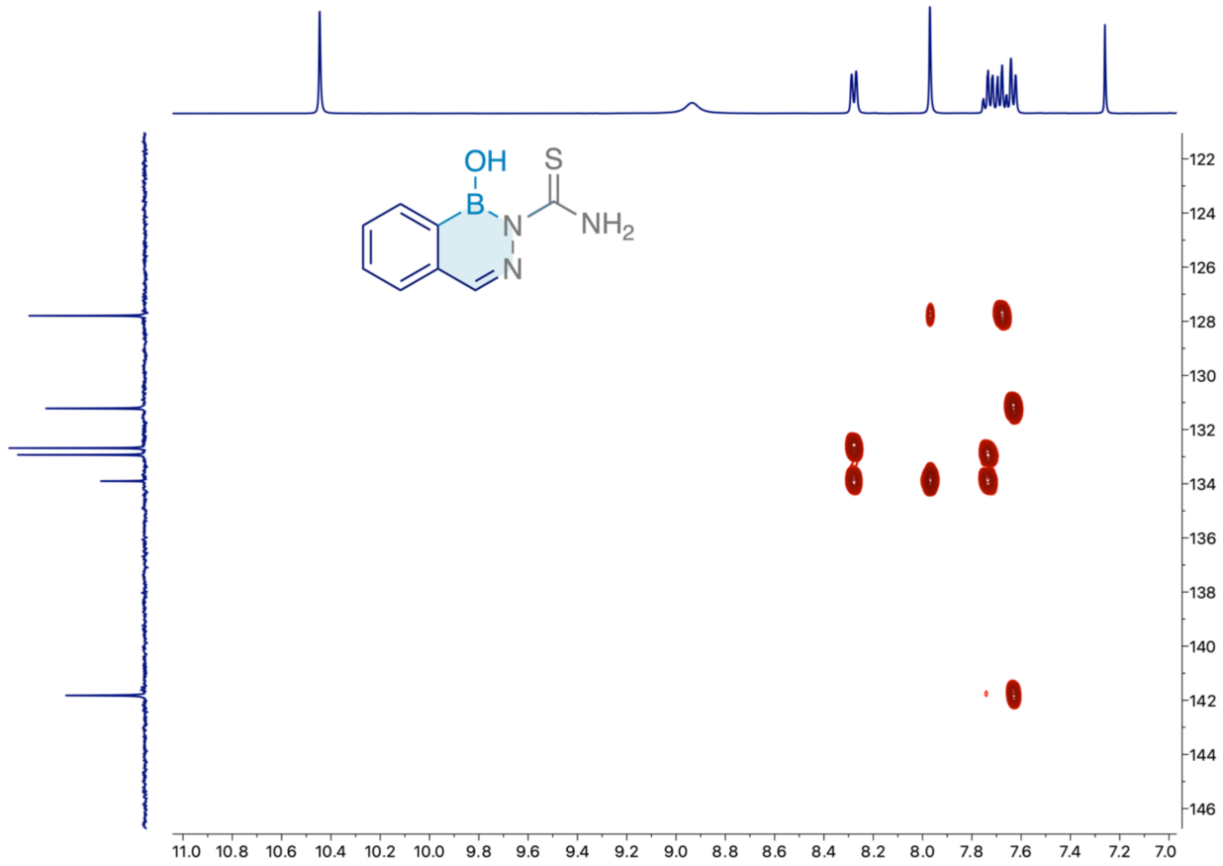

Figure S290. Diazaborine 55: ^{1}H-^{13}C gHMBC NMR ($CDCl_3$, 298 K)

Diazaborine 56

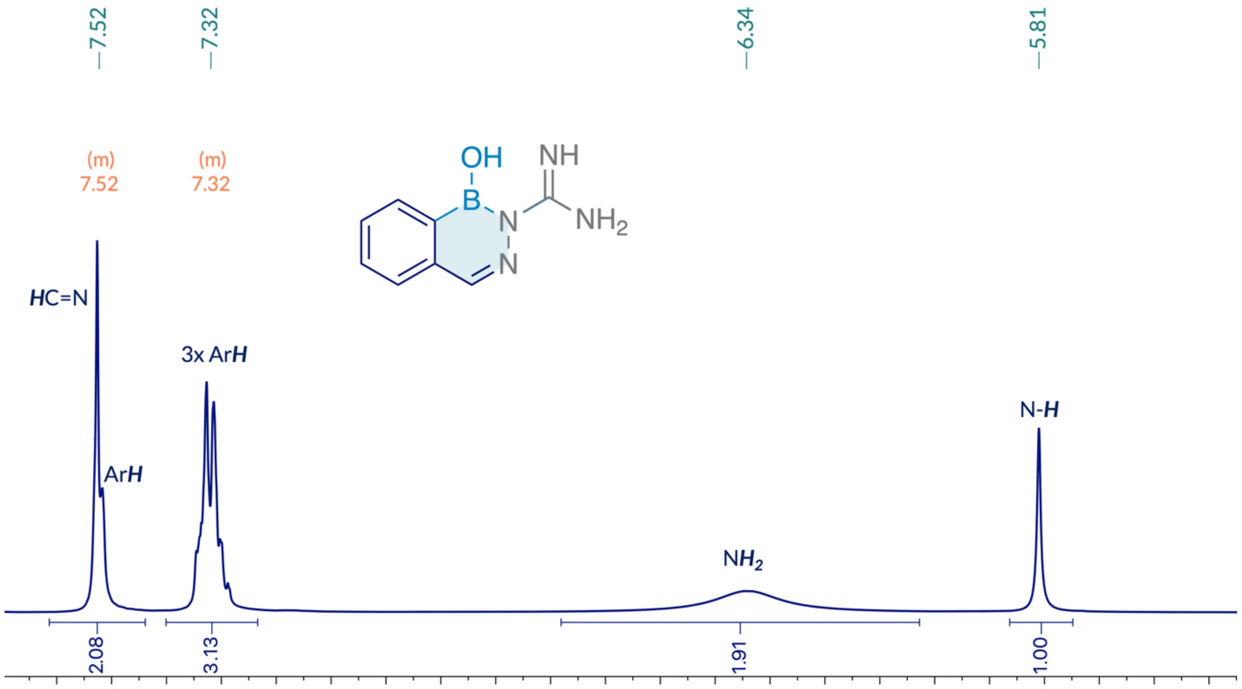

Figure S291. Diazaborine 56:^{1}H NMR (500 MHz, DMSO-d_6, 298 K)

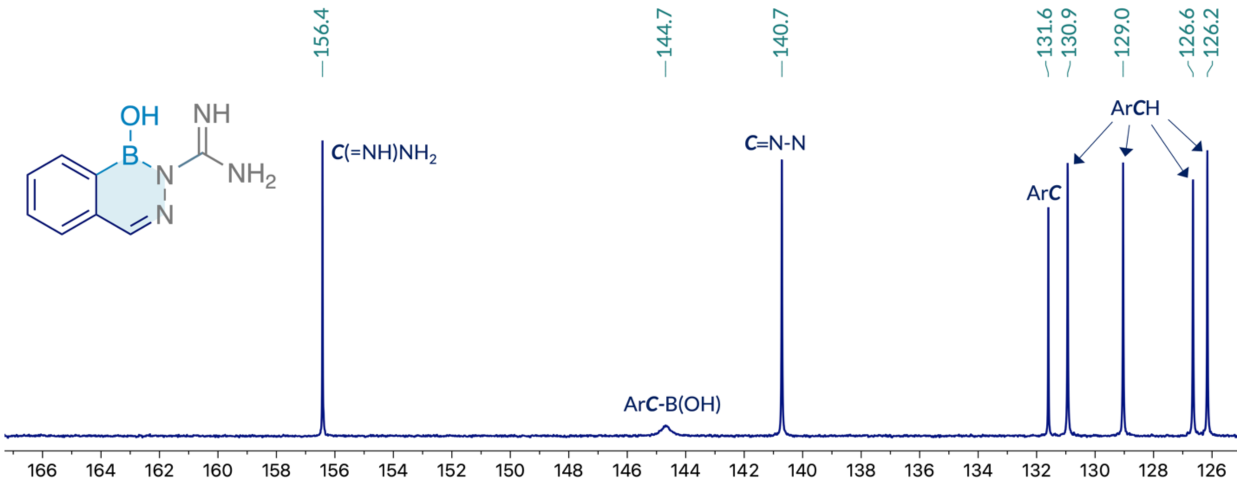

Figure S292. Diazaborine 56:^{13}C NMR (126 MHz, DMSO-d_6, 298 K)

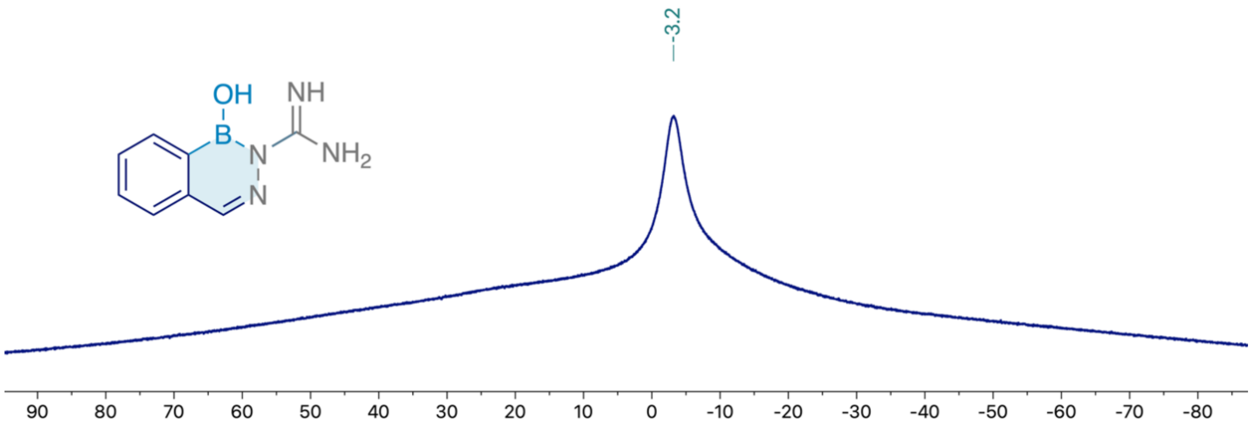

Figure S293. Diazaborine 56:^{11}B NMR (160 MHz, DMSO-d_6, 298 K)

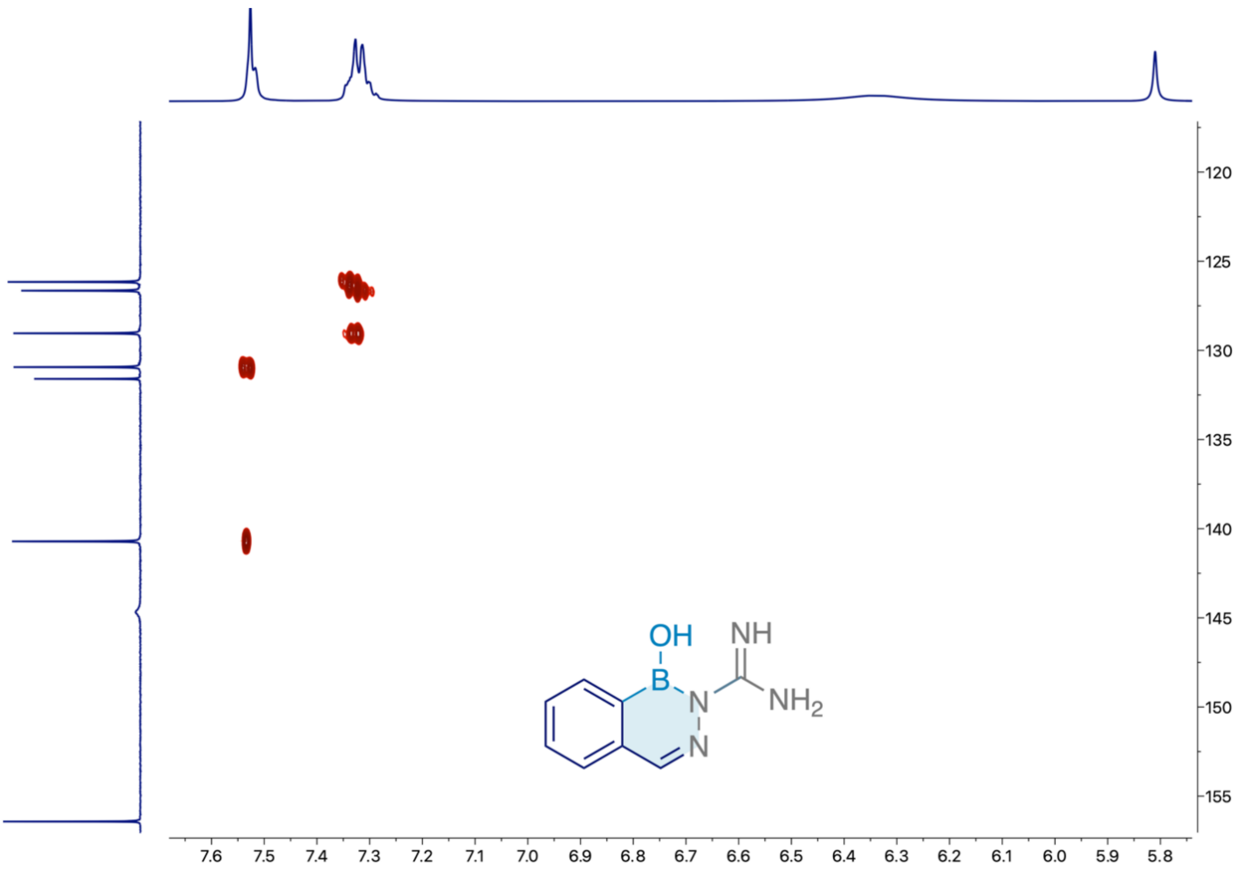

Figure S294. Diazaborine 56: ^{1}H-^{13}C gHSQC NMR (DMSO-d_6, 298 K)

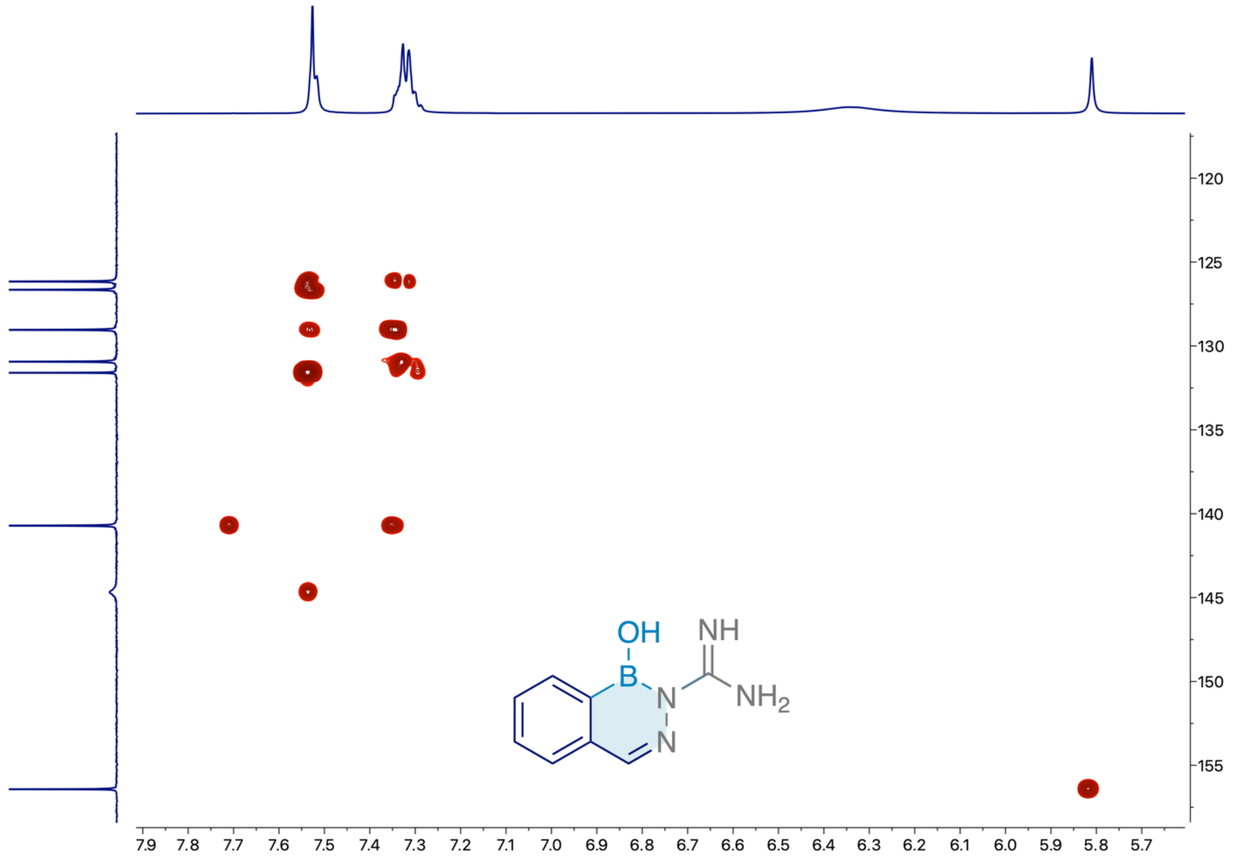

Figure S295. Diazaborine 56: ^{1}H-^{13}C gHMBC NMR (DMSO-d_6, 298 K)

Diazaborine 57

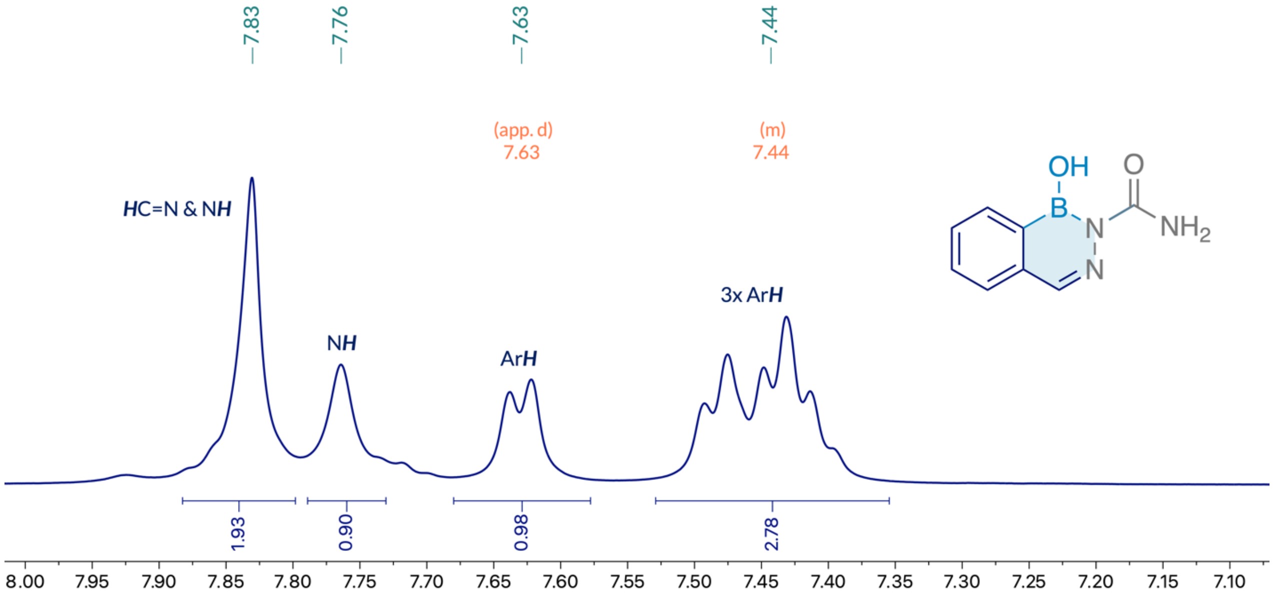

Figure S296. Diazaborine 57: ^{1}H NMR (400 MHz, DMSO-d_6, 298 K)

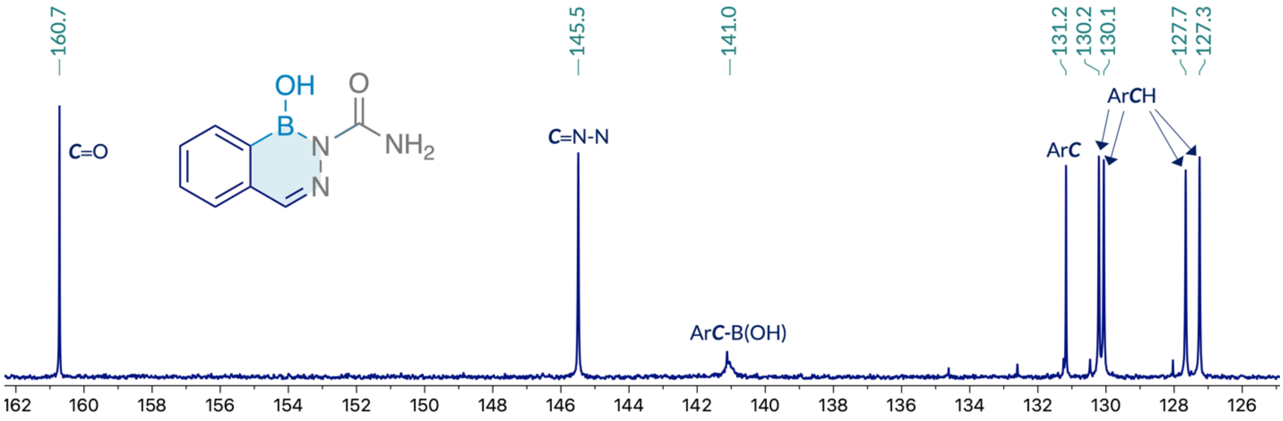

Figure S297. Diazaborine 57: ^{13}C NMR (126 MHz, DMSO-d_6, 298 K)

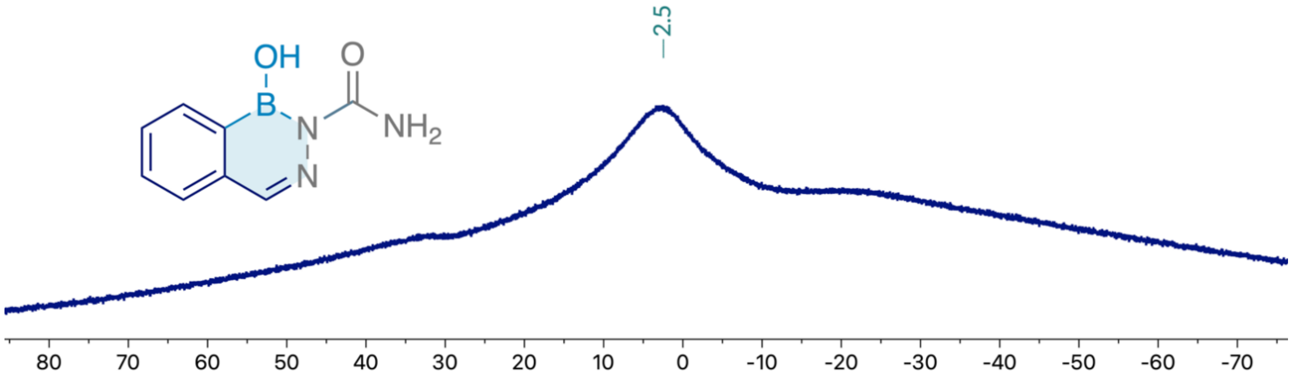

Figure S298. Diazaborine 57: ^{11}B NMR (160 MHz, DMSO-d_6, 298 K)

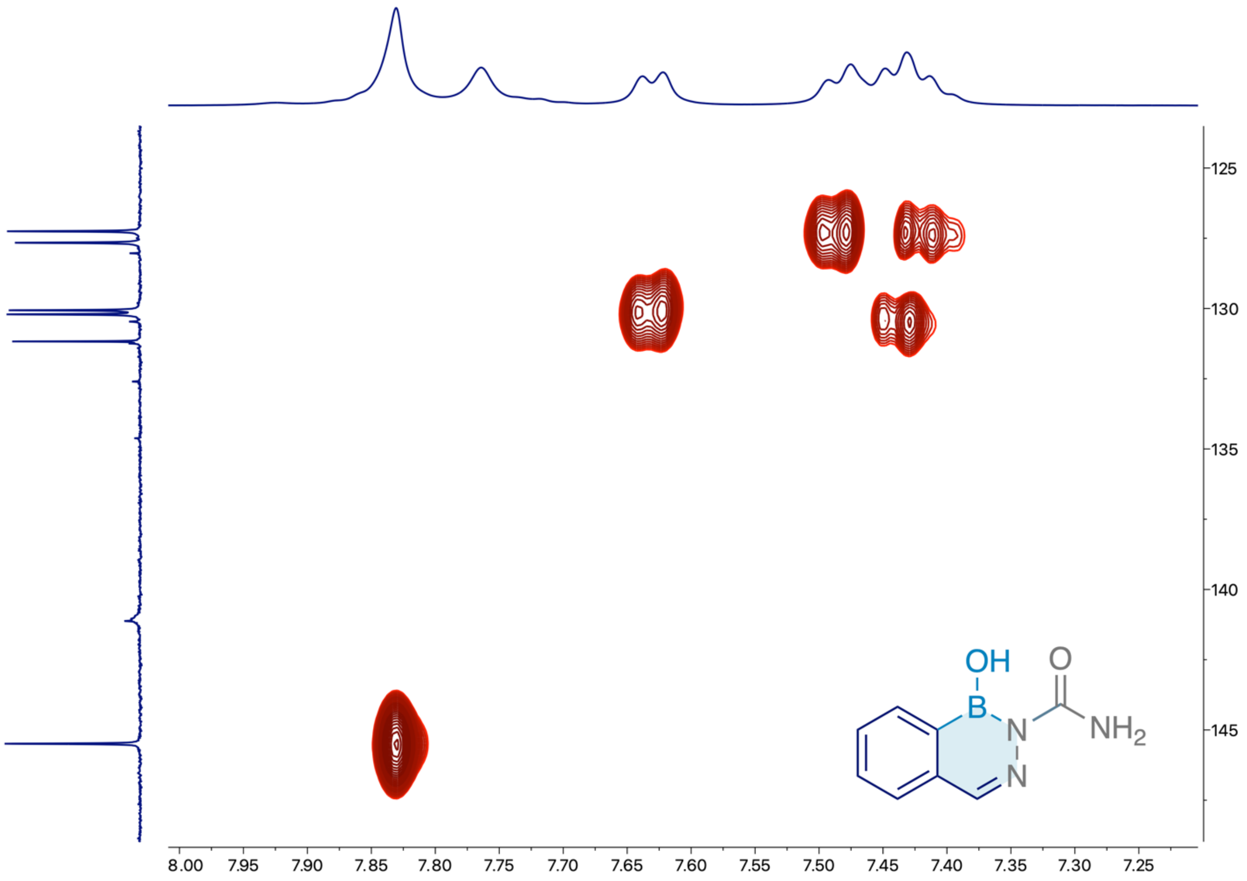

Figure S299. Diazaborine 57: ^{1}H-^{13}C gHSQC NMR (DMSO-d_6, 298 K)

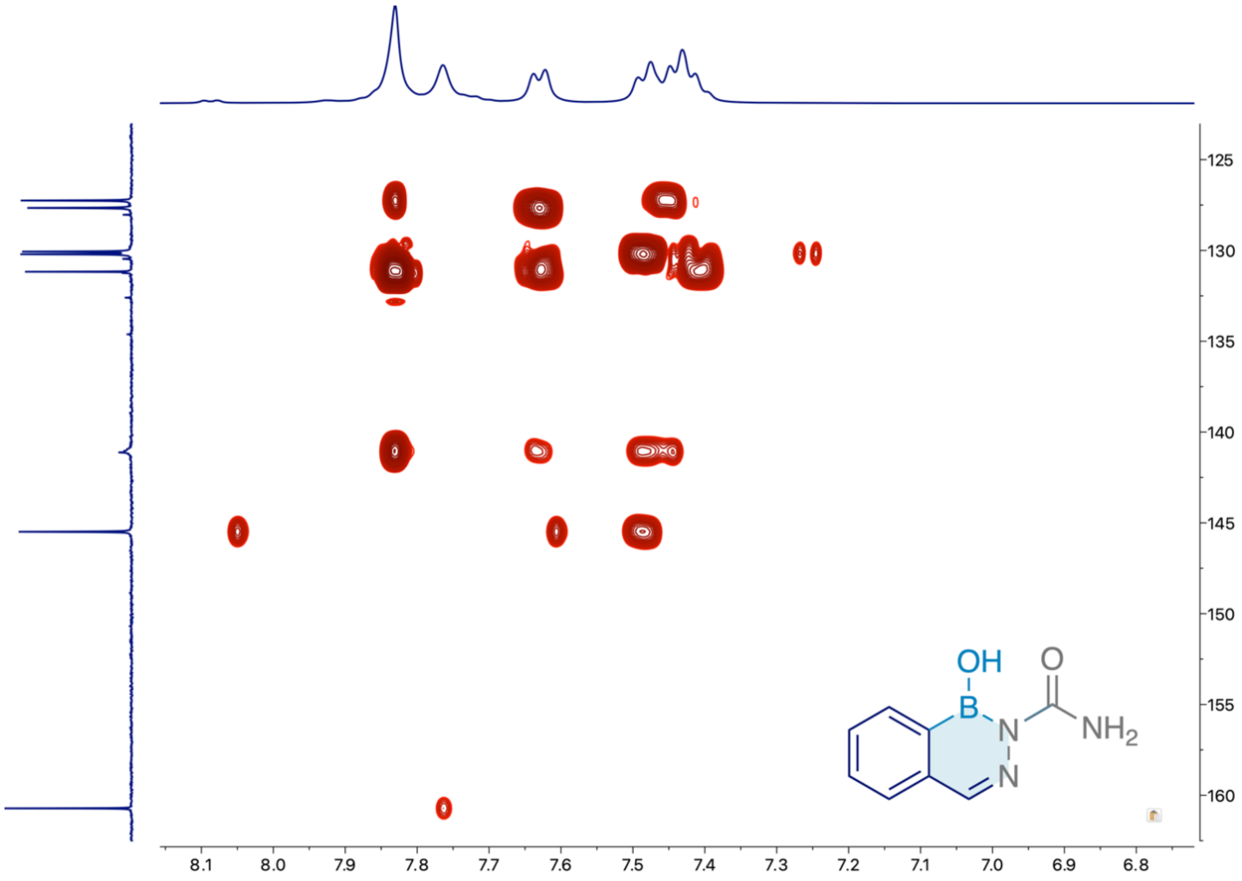

Figure S300. Diazaborine 57: ^{1}H-^{13}C gHMBC NMR (DMSO-d_6, 298 K)

N-Methyl derivative of diazaborine 11 (diazaborine 58)

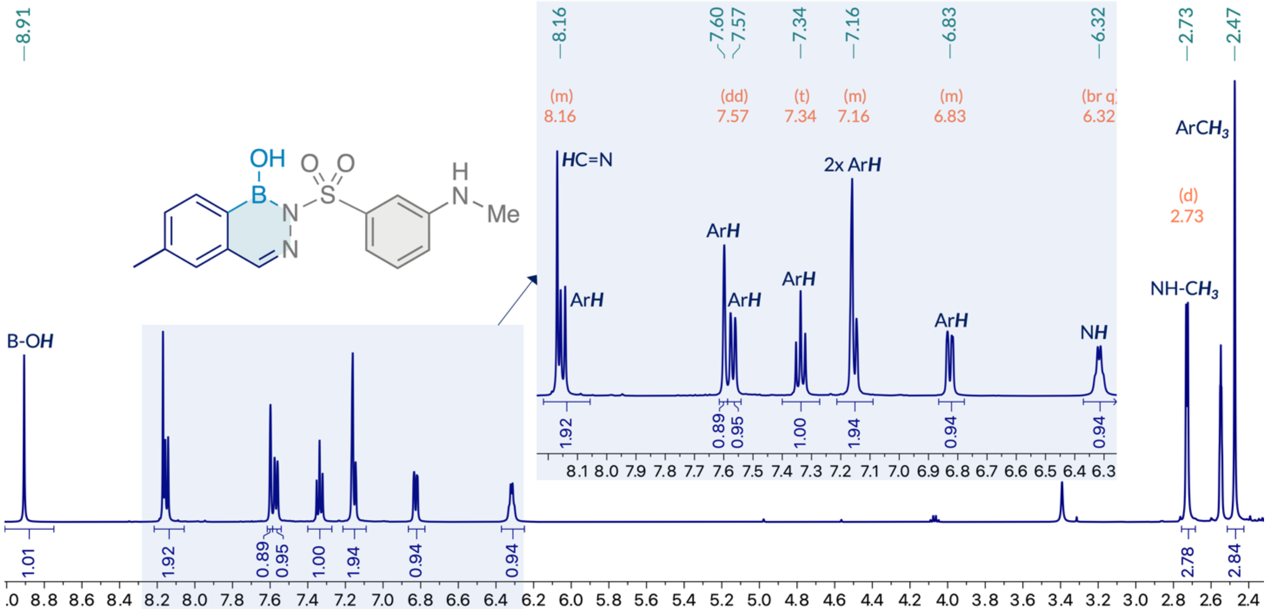

Figure S301. Diazaborine 58:^{1}H NMR (600 MHz, DMSO-d_6, 298 K)

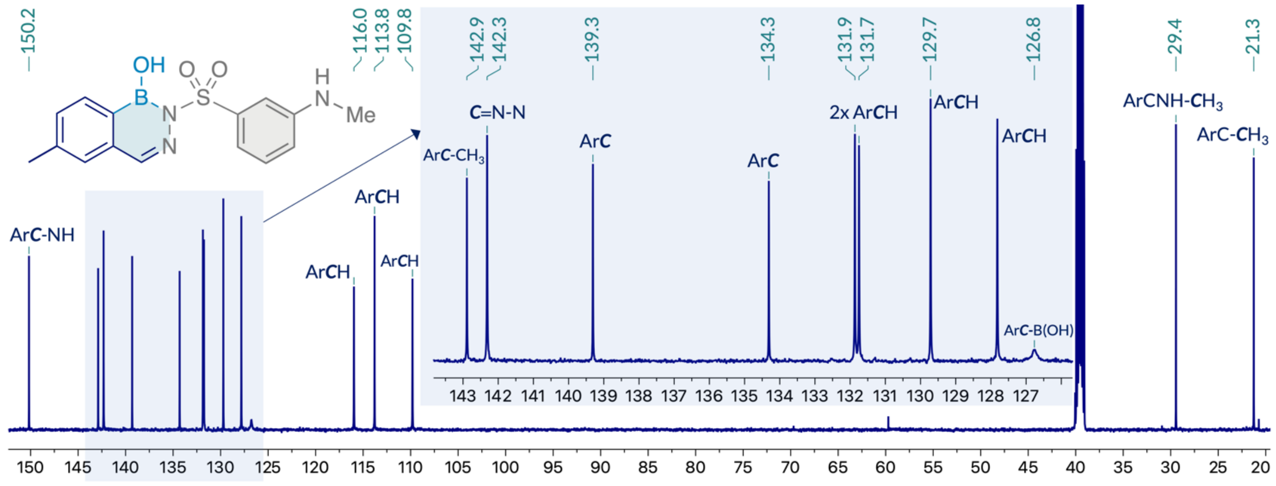

Figure S302. Diazaborine 58:^{13}C NMR (151 MHz, DMSO-d_6, 298 K)

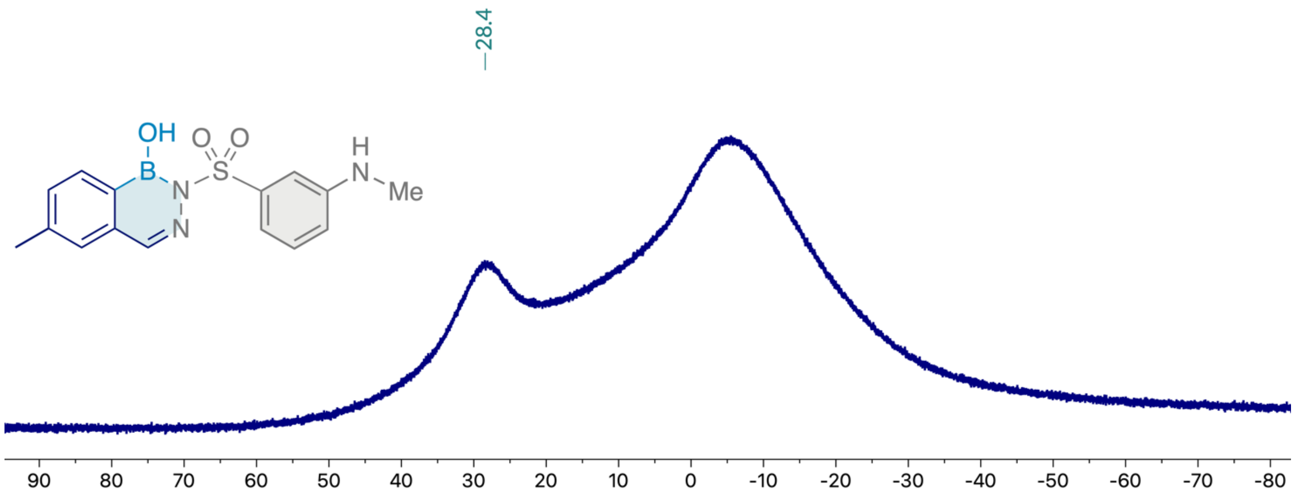

Figure S303. Diazaborine 58:^{11}B NMR (160 MHz, DMSO-d_6, 298 K)

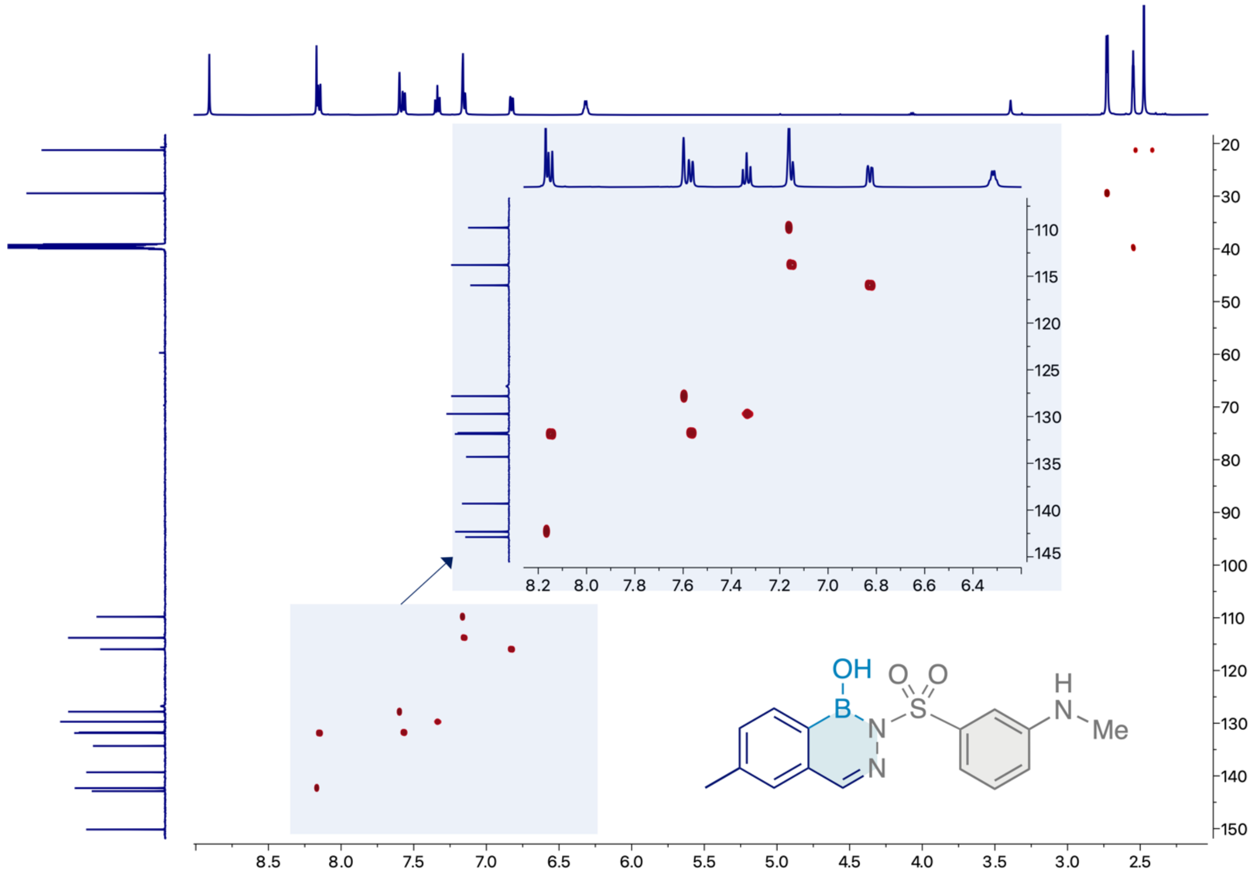

Figure S304. Diazaborine 58: ^{1}H-^{13}C gHSQC NMR (DMSO-d_6, 298 K)

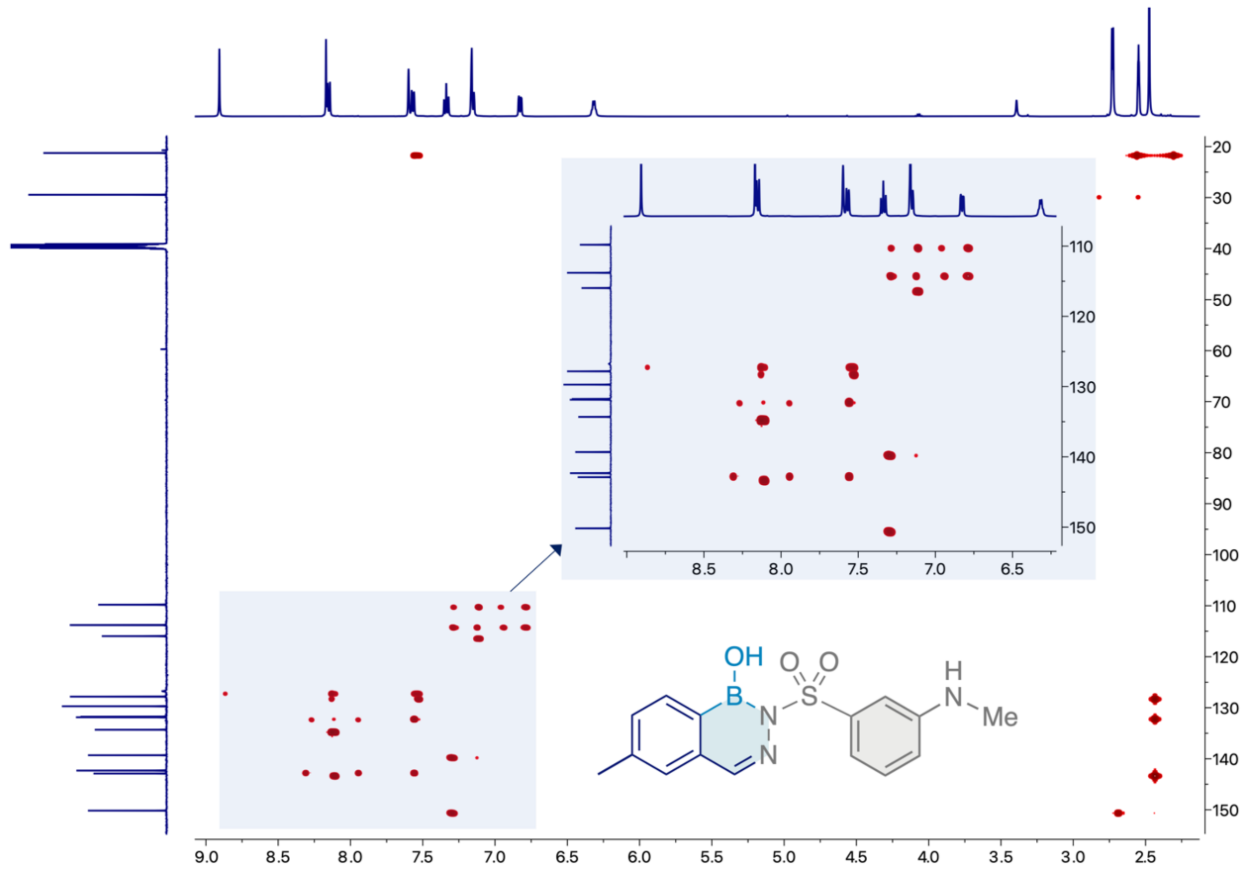

Figure S305. Diazaborine 58: ^{1}H-^{13}C gHMBC NMR (DMSO-d_6, 298 K)

Diazaborine derivatives

N-Acetyl derivative of diazaborine 11 (diazaborine 59)

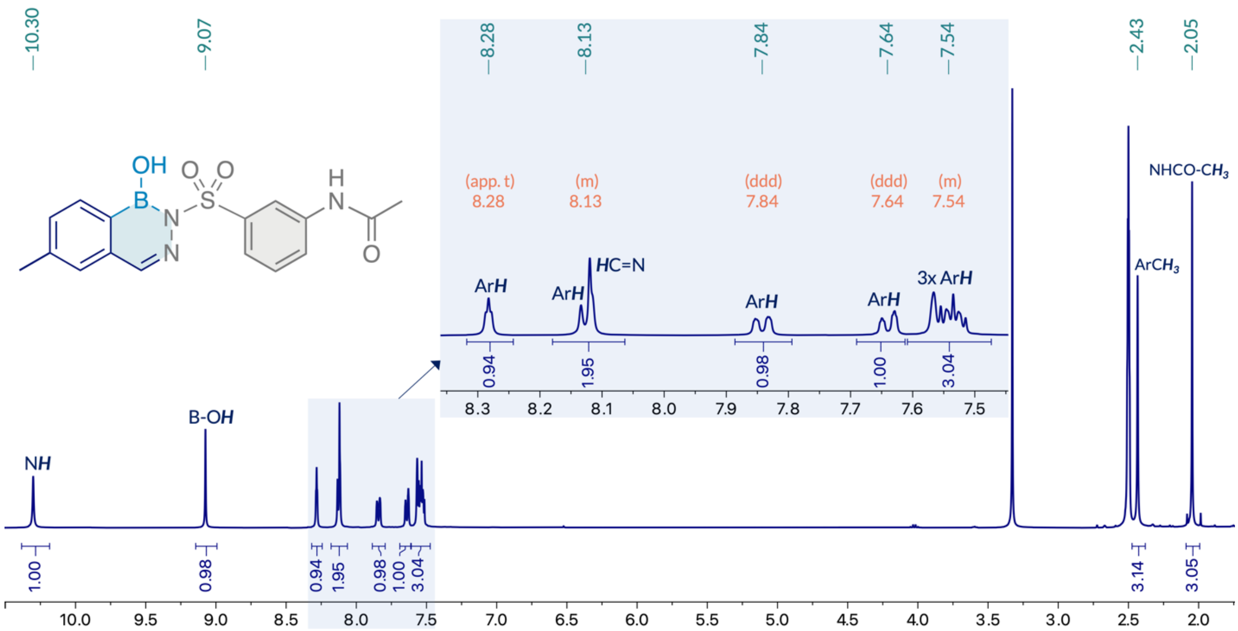

Figure S306. Diazaborine 59:^{1}H NMR (400 MHz, DMSO-d_6, 298 K)

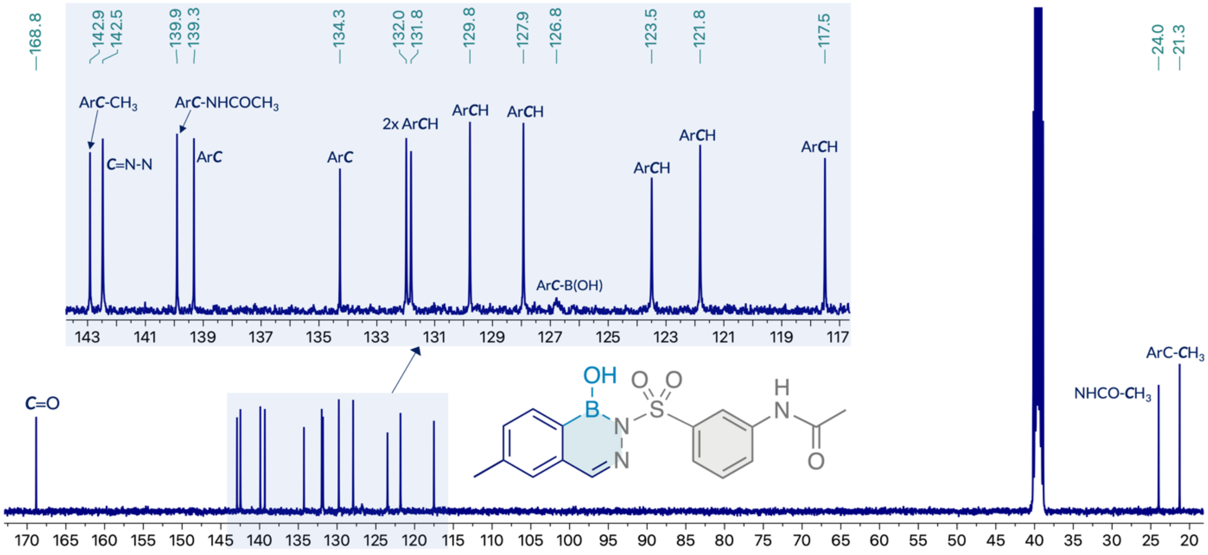

Figure S307. Diazaborine 59:^{13}C NMR (101 MHz, DMSO-d_6, 298 K)

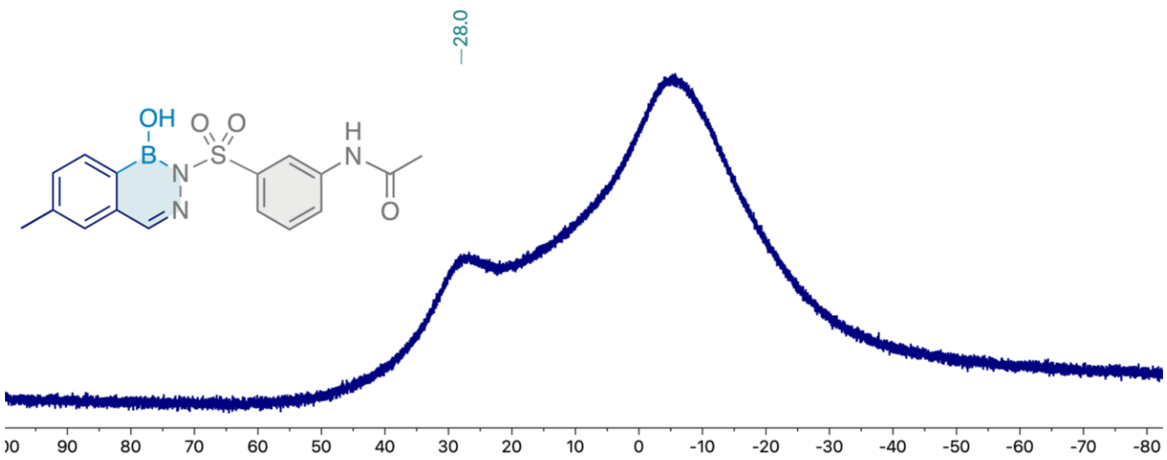

Figure S308. Diazaborine 59:^{11}B NMR (160 MHz, DMSO-d_6, 298 K)

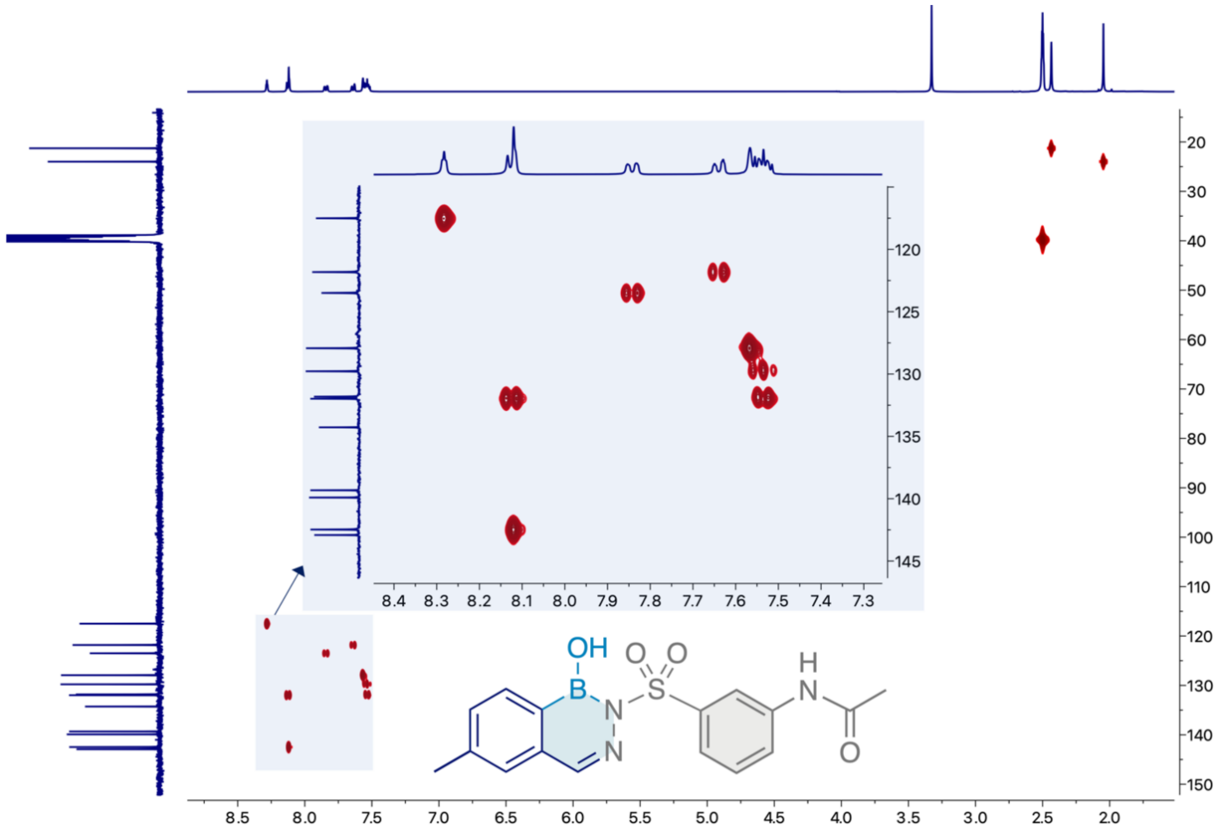

Figure S309. Diazaborine 59: ^{1}H-^{13}C gHSQC NMR (DMSO-d_6, 298 K)

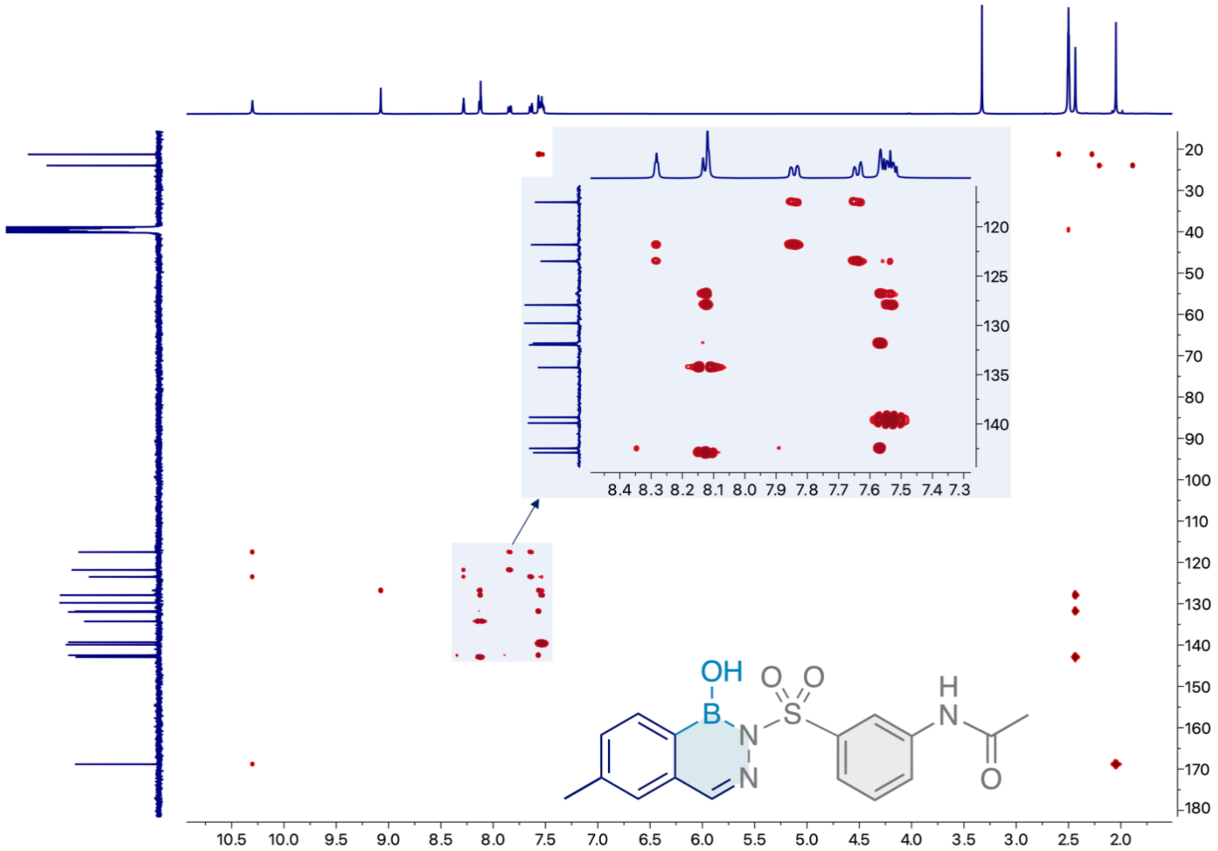

Figure S310. Diazaborine 59: ^{1}H-^{13}C gHMBC NMR (DMSO-d_6, 298 K)

Diazaborine salts

Potassium difluoroborate salt of diazaborine 1 (diazaborine 60)

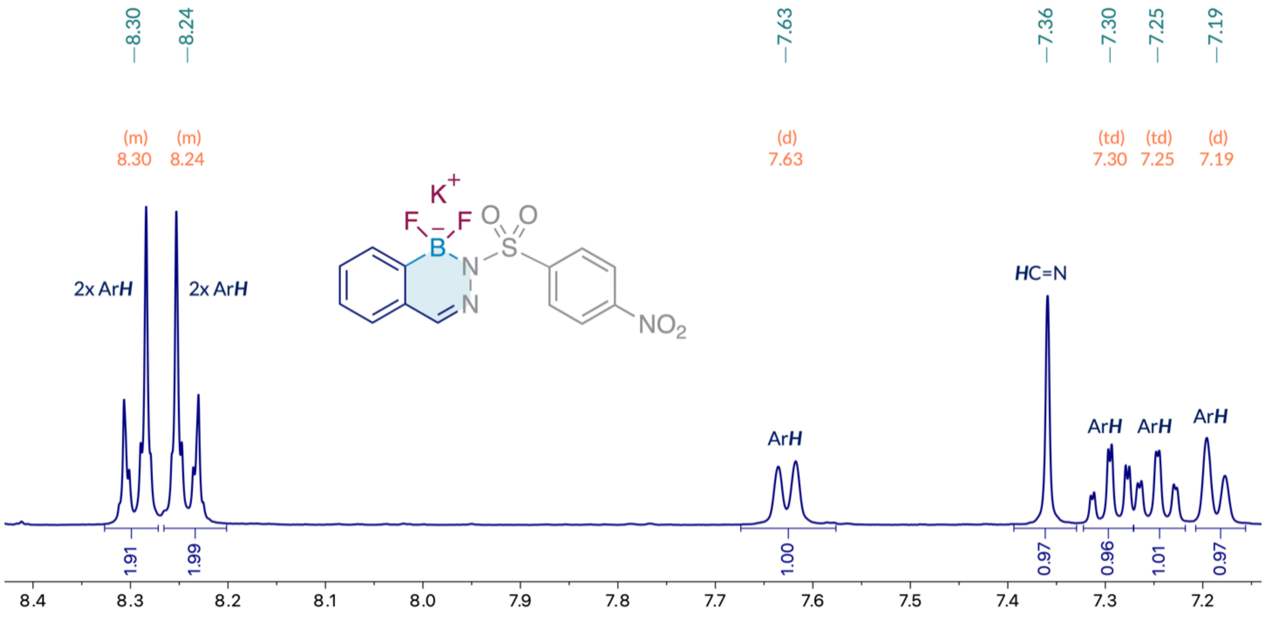

Figure S311. Diazaborine 60: ^{1}H NMR (400 MHz, Acetone-d_6, 298 K)

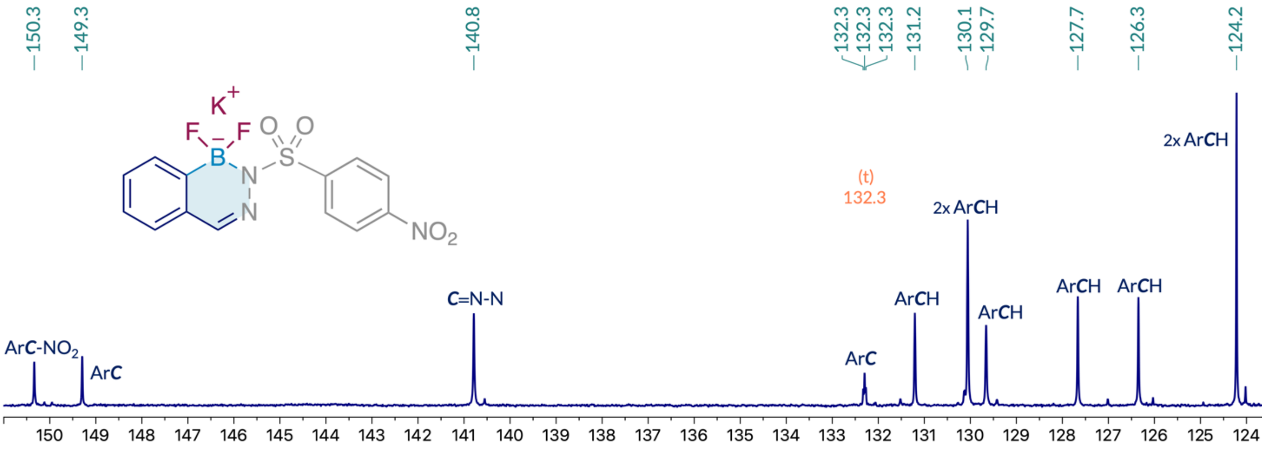

Figure S312. Diazaborine 60:^{13}C NMR (126 MHz, Acetone-d_6, 298 K)

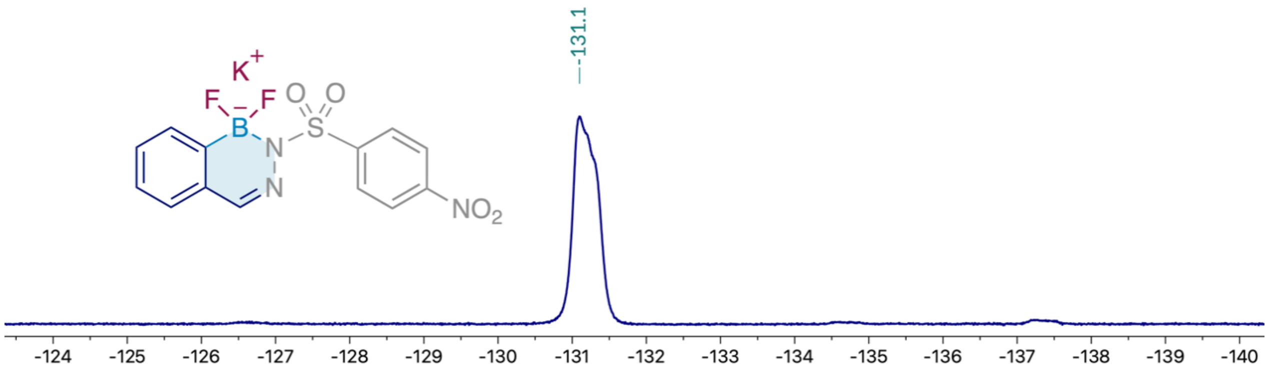

Figure S313. Diazaborine 60:^{19}F NMR (377 MHz, Acetone-d_6, 298 K)

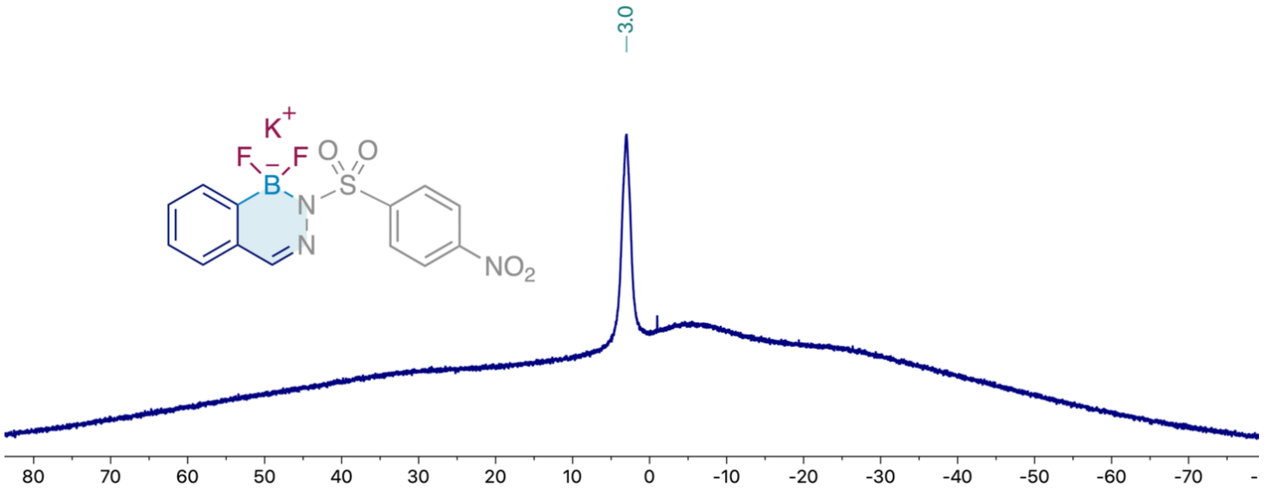

Figure S314. Diazaborine 60:^{11}B NMR (128 MHz, Acetone-d_6, 298 K)

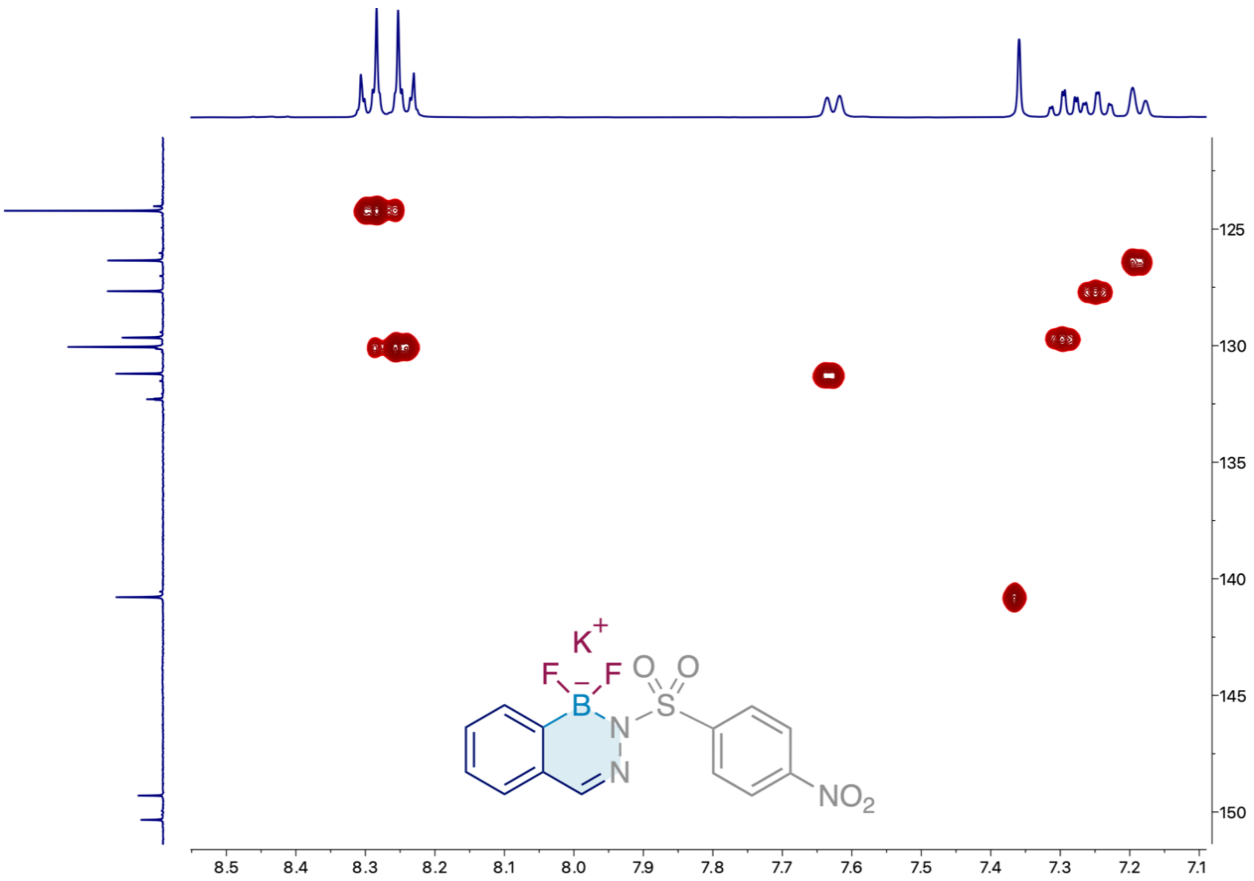

Figure S315. Diazaborine 60: ^{1}H-^{13}C gHSQC NMR (Acetone-d_6, 298 K)

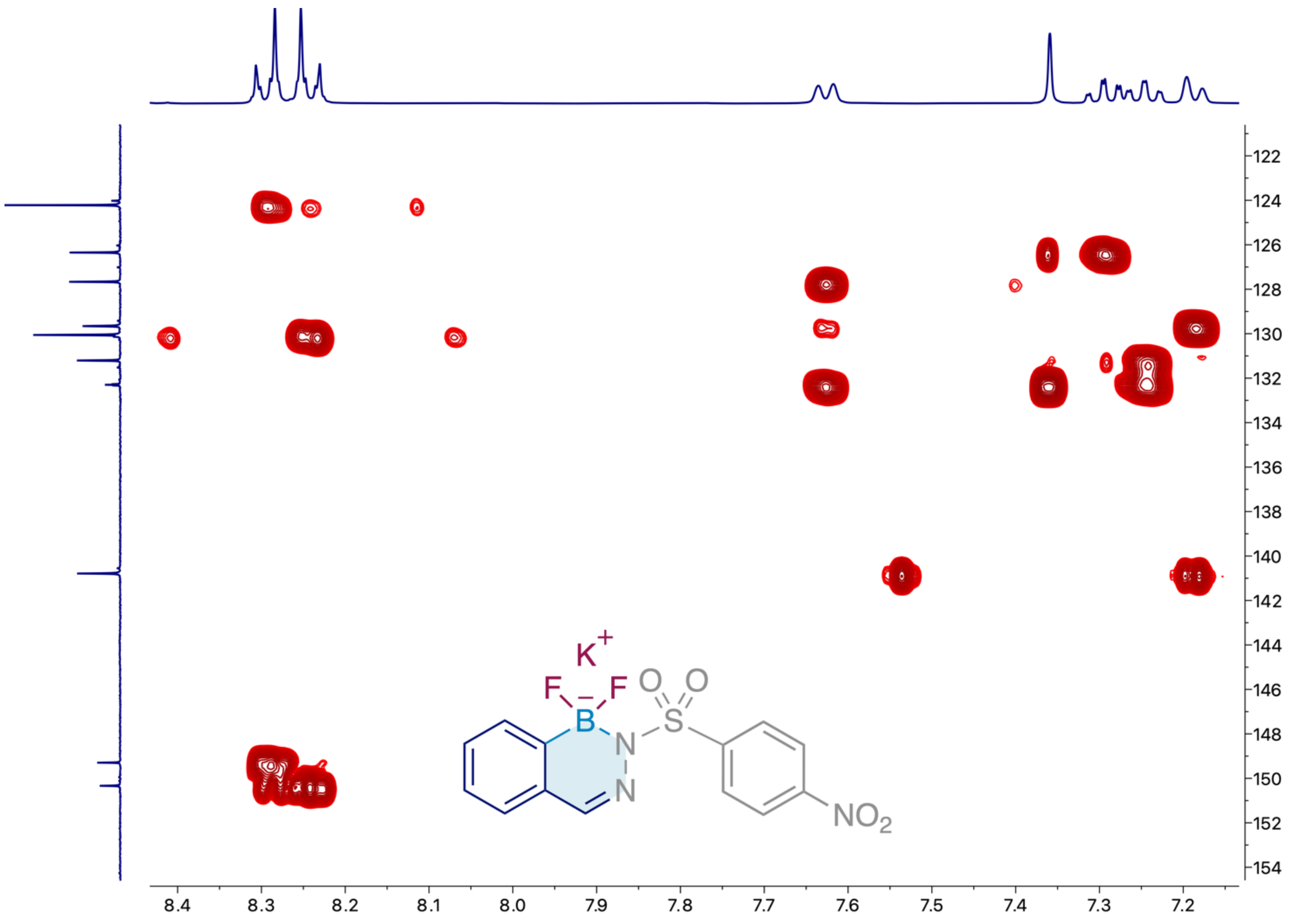

Figure S316. Diazaborine 60: ^{1}H-^{13}C gHMBC NMR (Acetone-d_6, 298 K)

Chloride salt of diazaborine 11 (diazaborine 61)

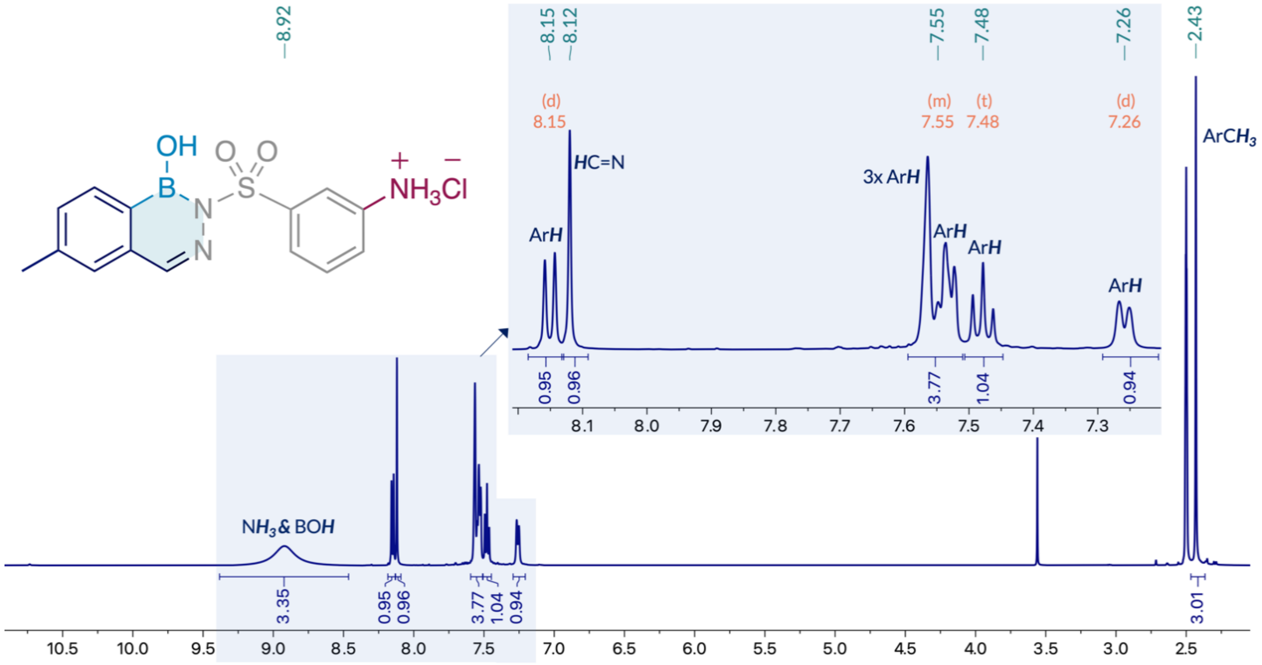

Figure S317. Diazaborine 61: ^{1}H NMR (500 MHz, DMSO-d_6, 298 K)

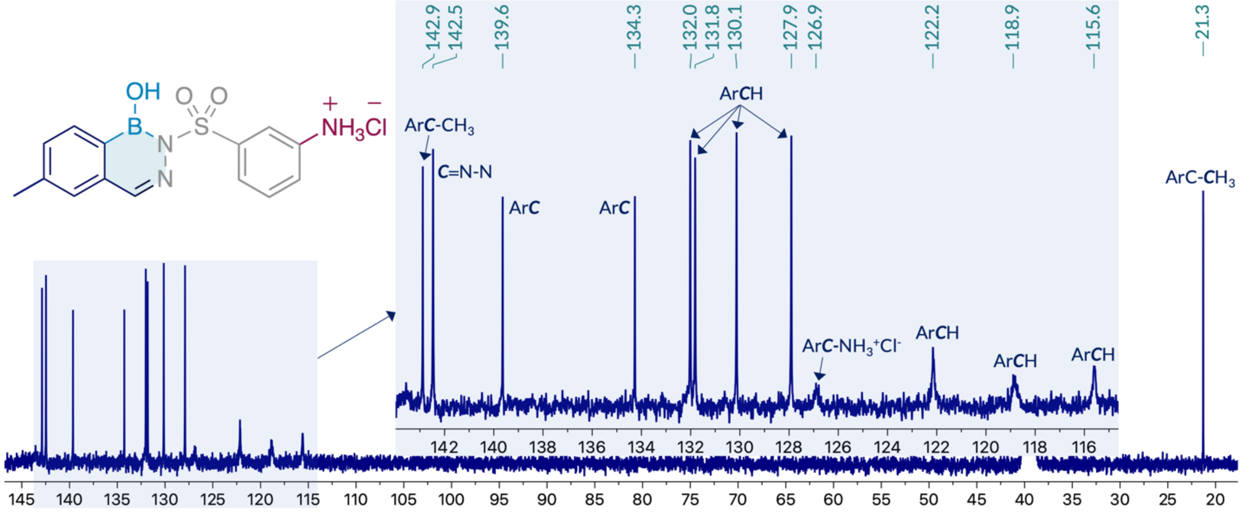

Figure S318. Diazaborine 61: ^{13}C NMR (101 MHz, DMSO-d_6, 298 K)

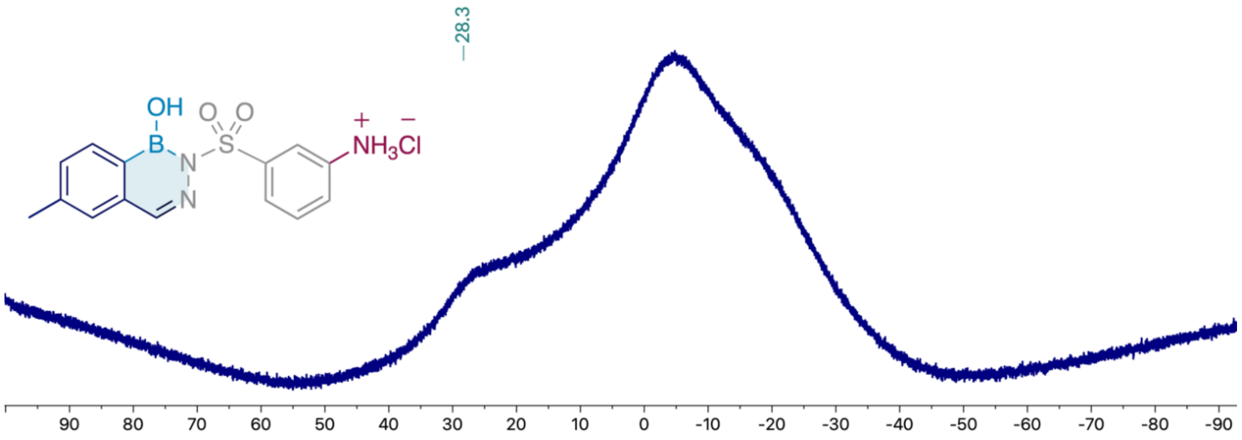

Figure S319. Diazaborine 61: ^{11}B NMR (160 MHz, DMSO-d_6, 298 K)

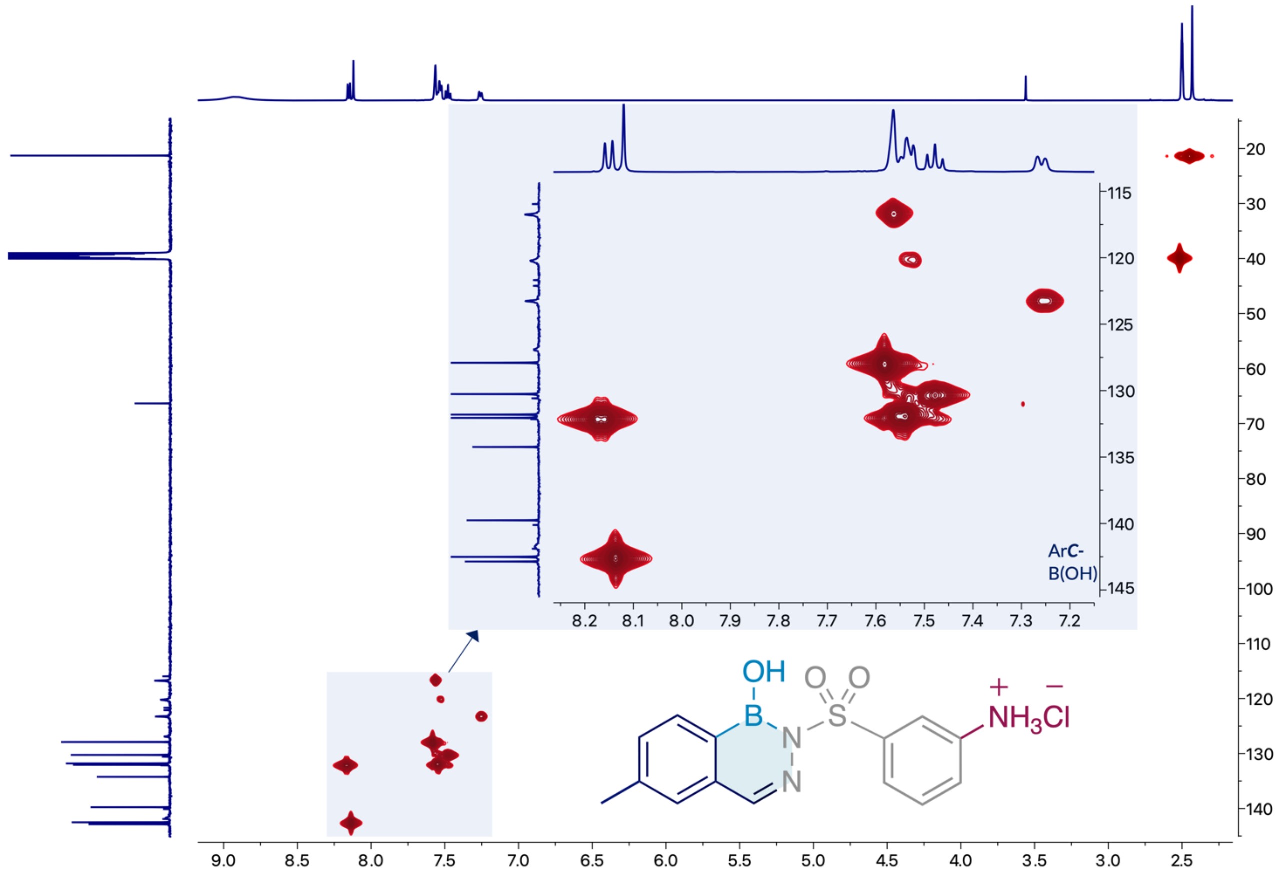

Figure S320. Diazaborine 61: ^{1}H-^{13}C gHSQC NMR (DMSO-d_6, 298 K)

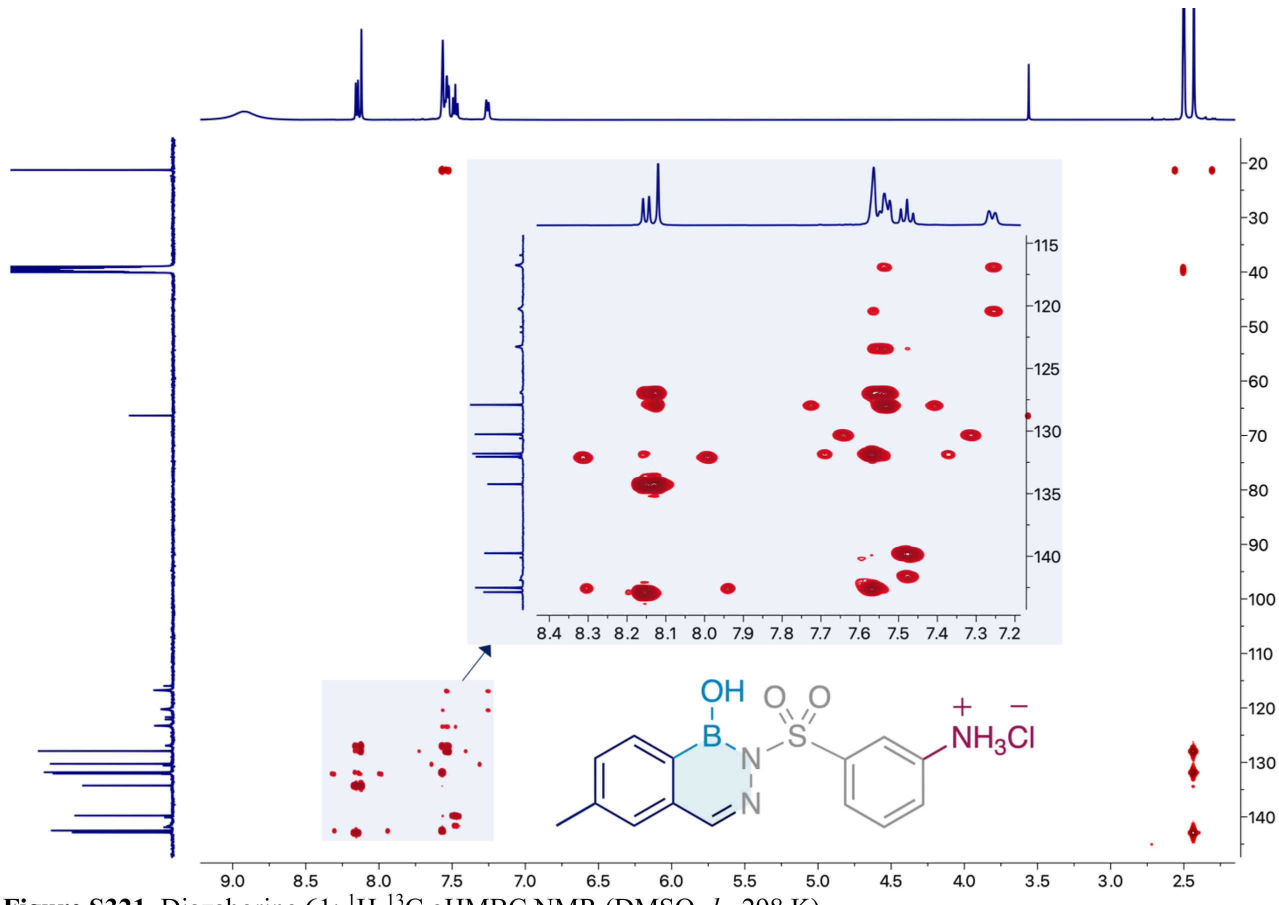

Figure S321. Diazaborine 61: ^{1}H-^{13}C gHMBC NMR (DMSO-d_6, 298 K)

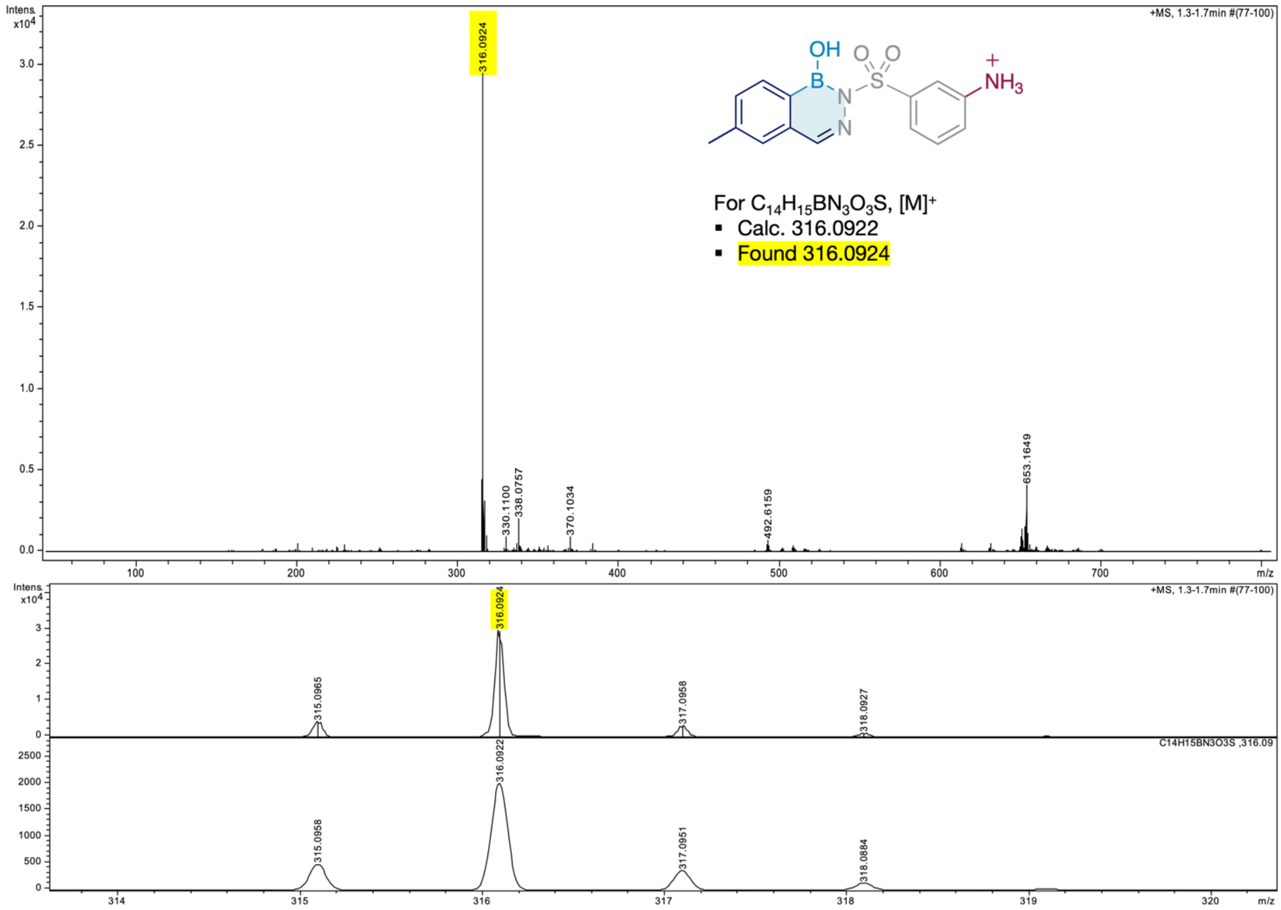

Figure S322. ESI-MS spectrum of Diazaborine 61 ($[M]^+$, ionized in MeOH-H_2O 5:1, positive mode).

Sodium salt of diazaborine 11 (diazaborine 62)

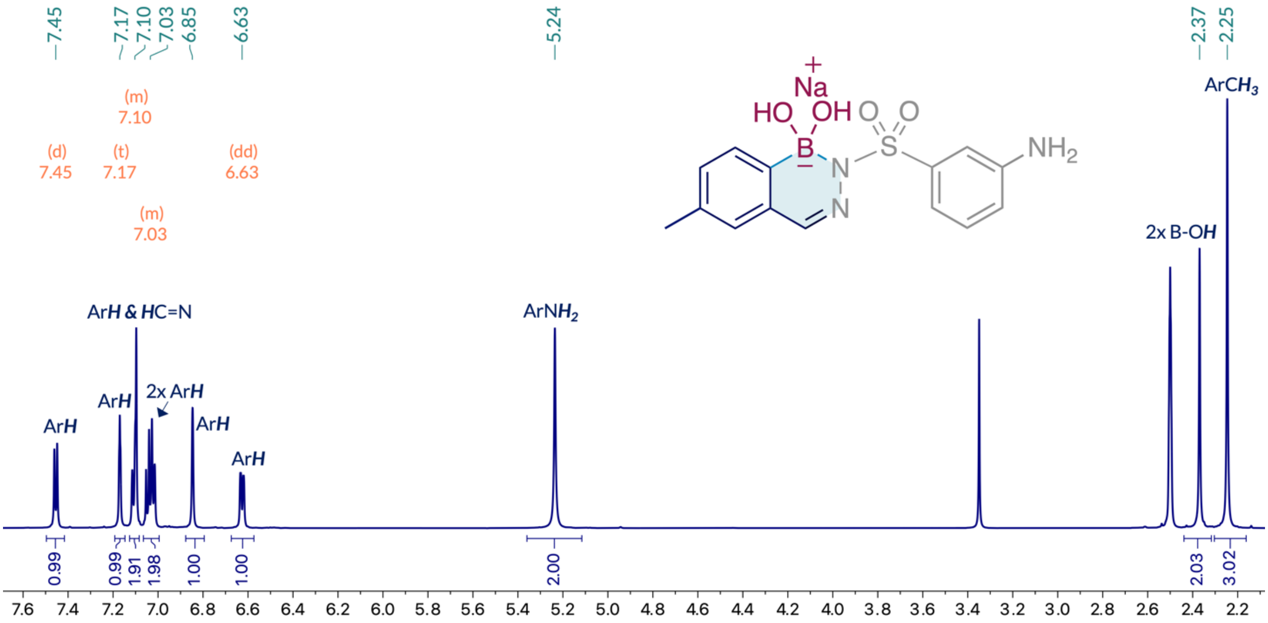

Figure S323. Diazaborine 62:^{1}H NMR (600 MHz, DMSO-d_6, 298 K)

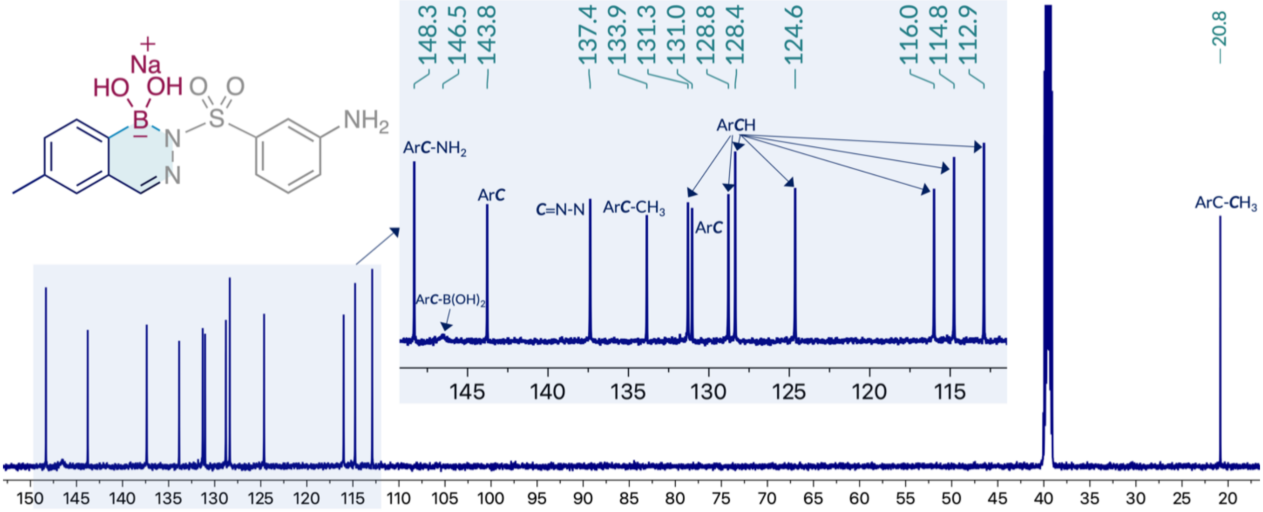

Figure S324. Diazaborine 62:^{13}C NMR (151 MHz, DMSO-d_6, 298 K)

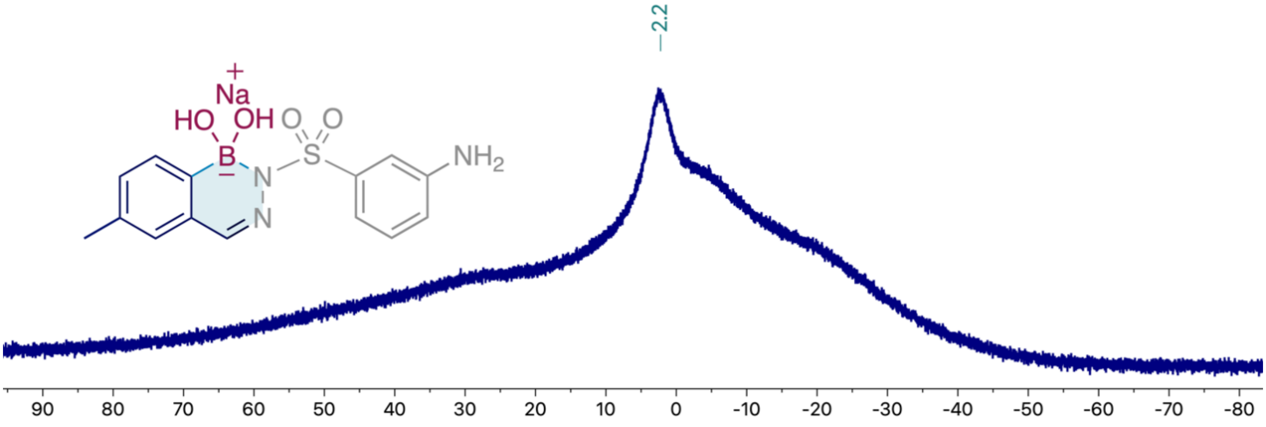

Figure S325. Diazaborine 62:^{11}B NMR (128 MHz, DMSO-d_6, 298 K)

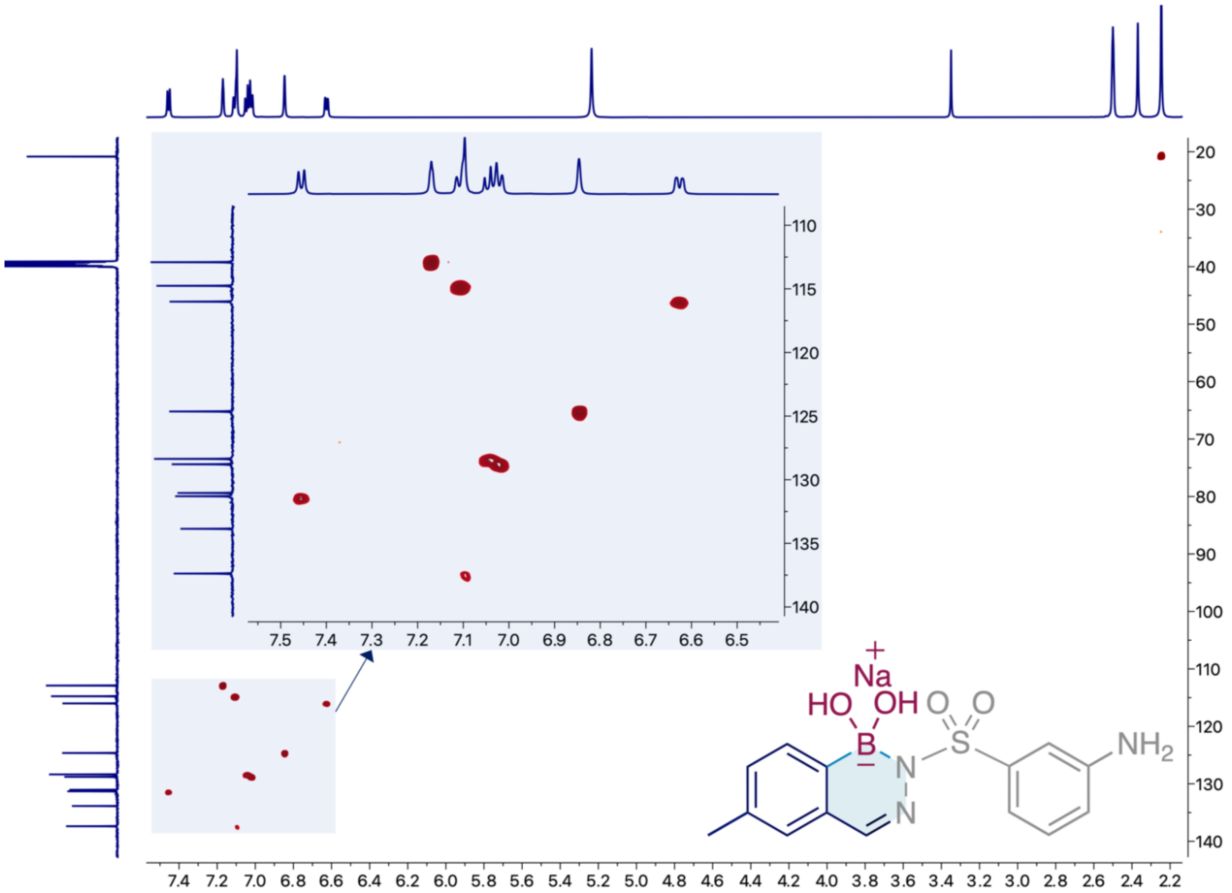

Figure S326. Diazaborine 62: ^{1}H-^{13}C gHSQC NMR (DMSO-d_6, 298 K)

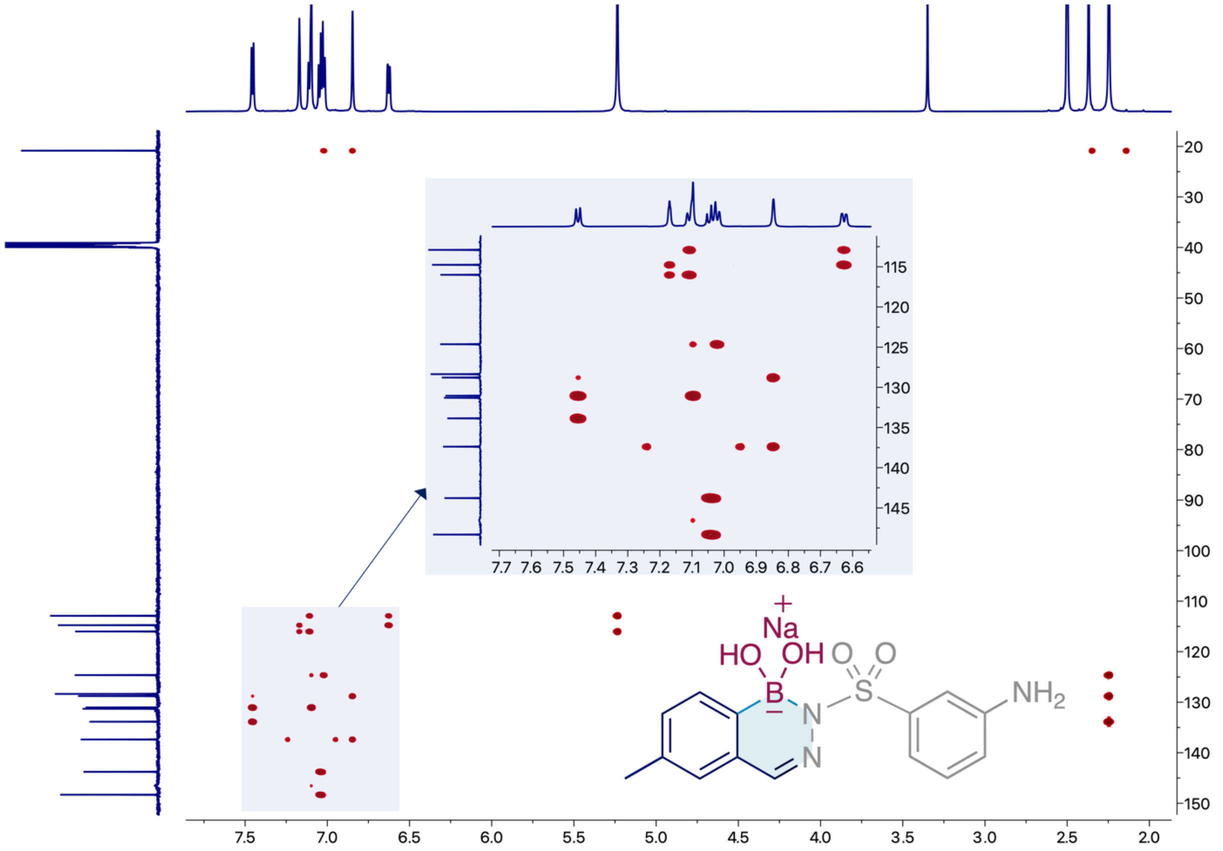

Figure S327. Diazaborine 62: ^{1}H-^{13}C gHMBC NMR (DMSO-d_6, 298 K)

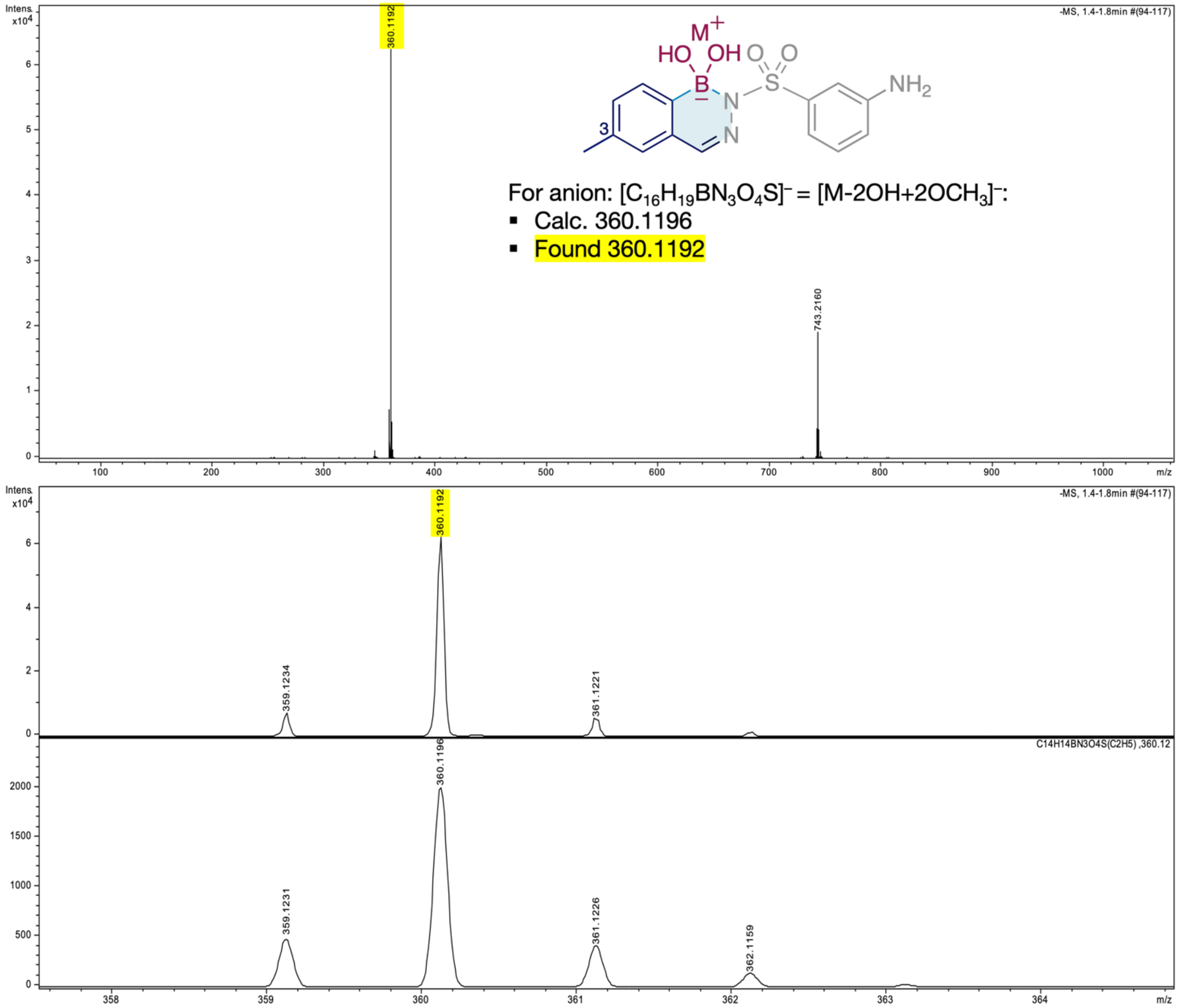

Figure S328. ESI-MS spectrum of Diazaborine 62 ([M–2OH+2OCH$_3$]$^–$, ionized in MeCN-H$_2$O 3:1, negative mode).

Potassium salt of diazaborine 11 (diazaborine 63)

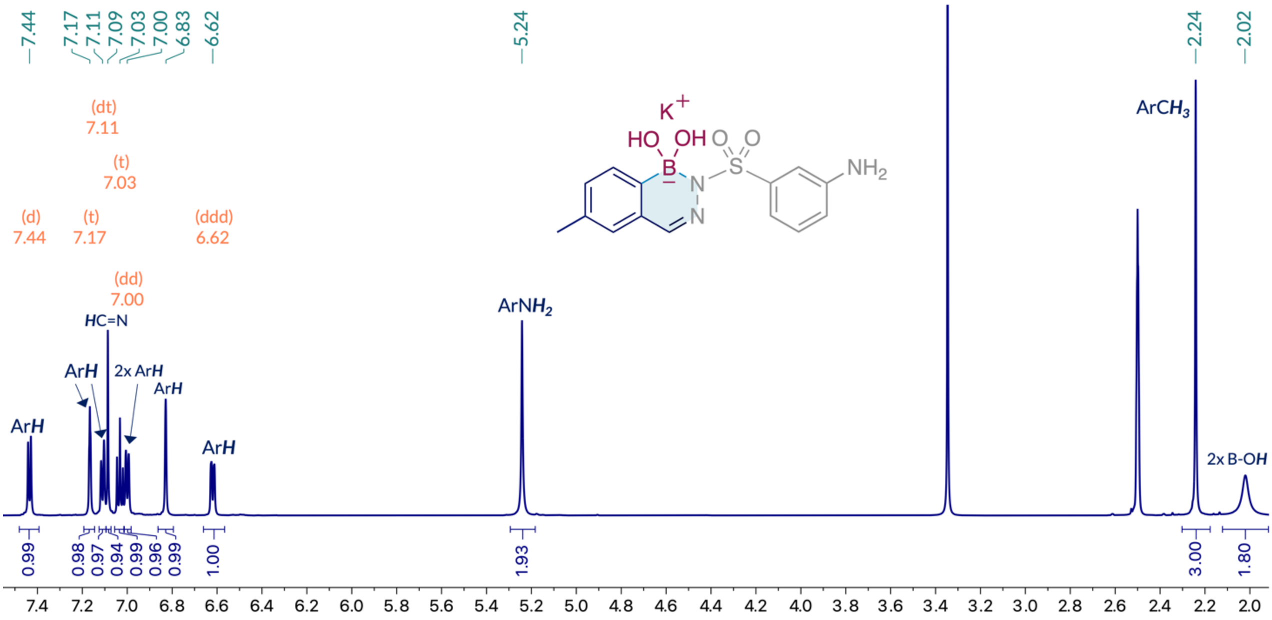

Figure S329. Diazaborine 63:^{1}H NMR (600 MHz, DMSO-d_6, 298 K)

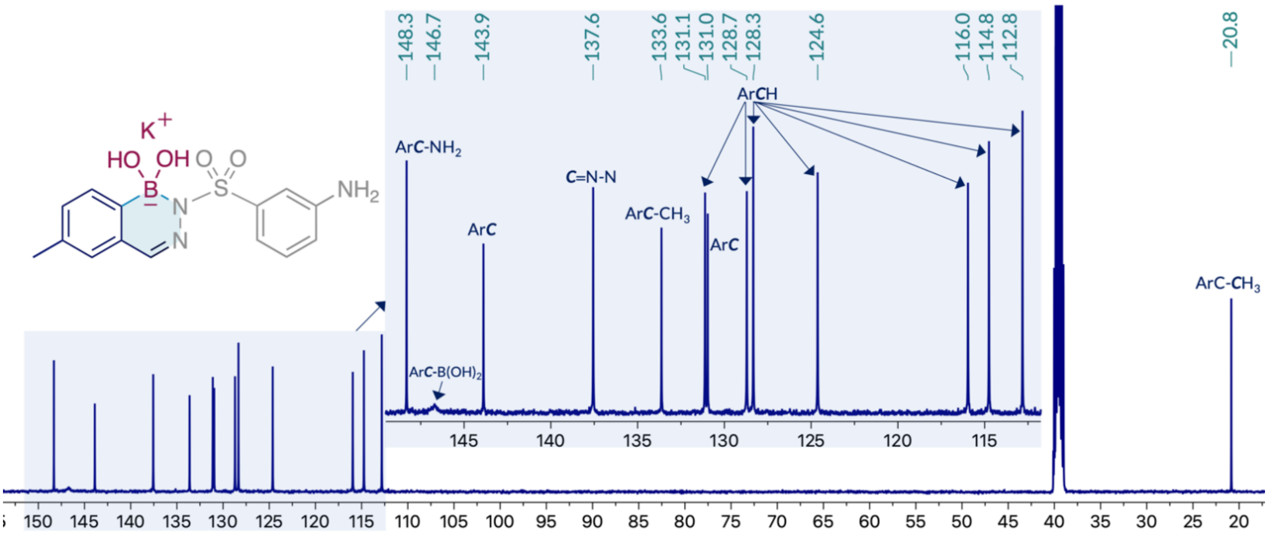

Figure S330. Diazaborine 63:^{13}C NMR (151 MHz, DMSO-d_6, 298 K)

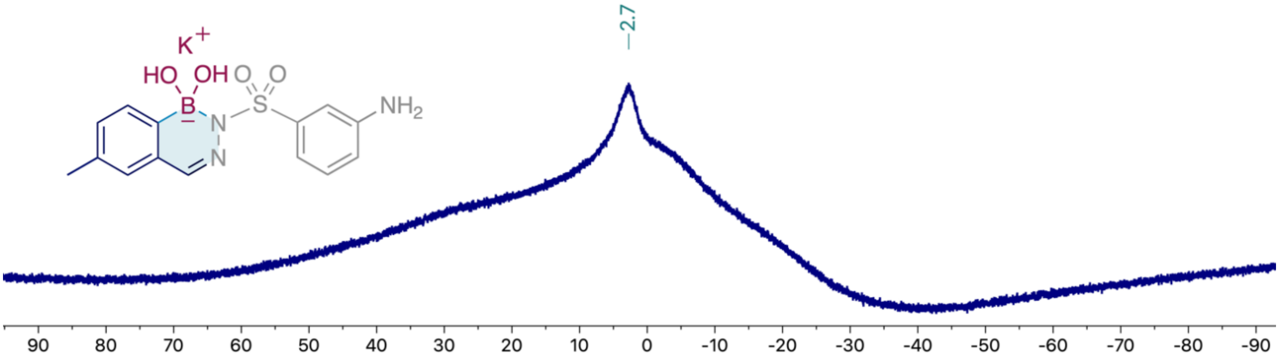

Figure S331. Diazaborine 63:^{11}B NMR (128 MHz, DMSO-d_6, 298 K)

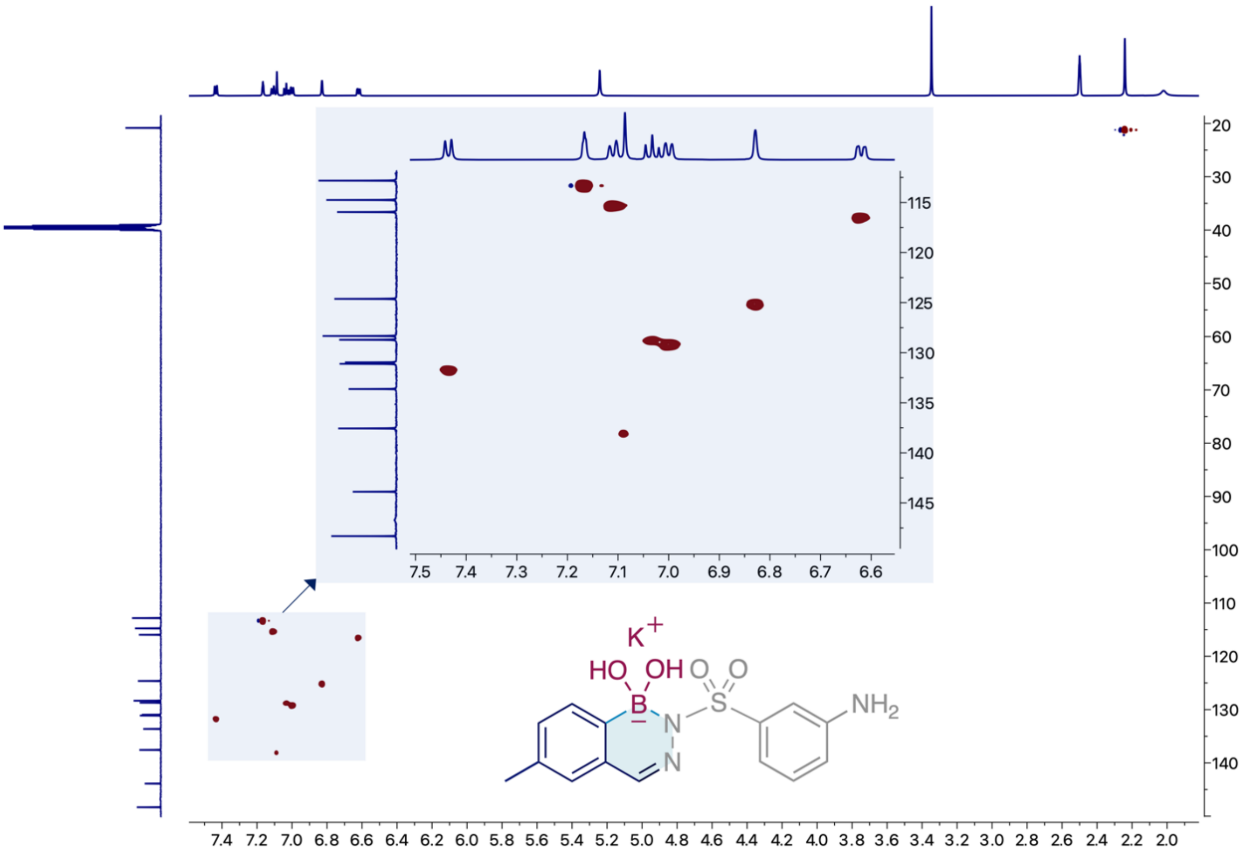

Figure S332. Diazaborine 63: ^{1}H-^{13}C gHSQC NMR (DMSO-d_6, 298 K)

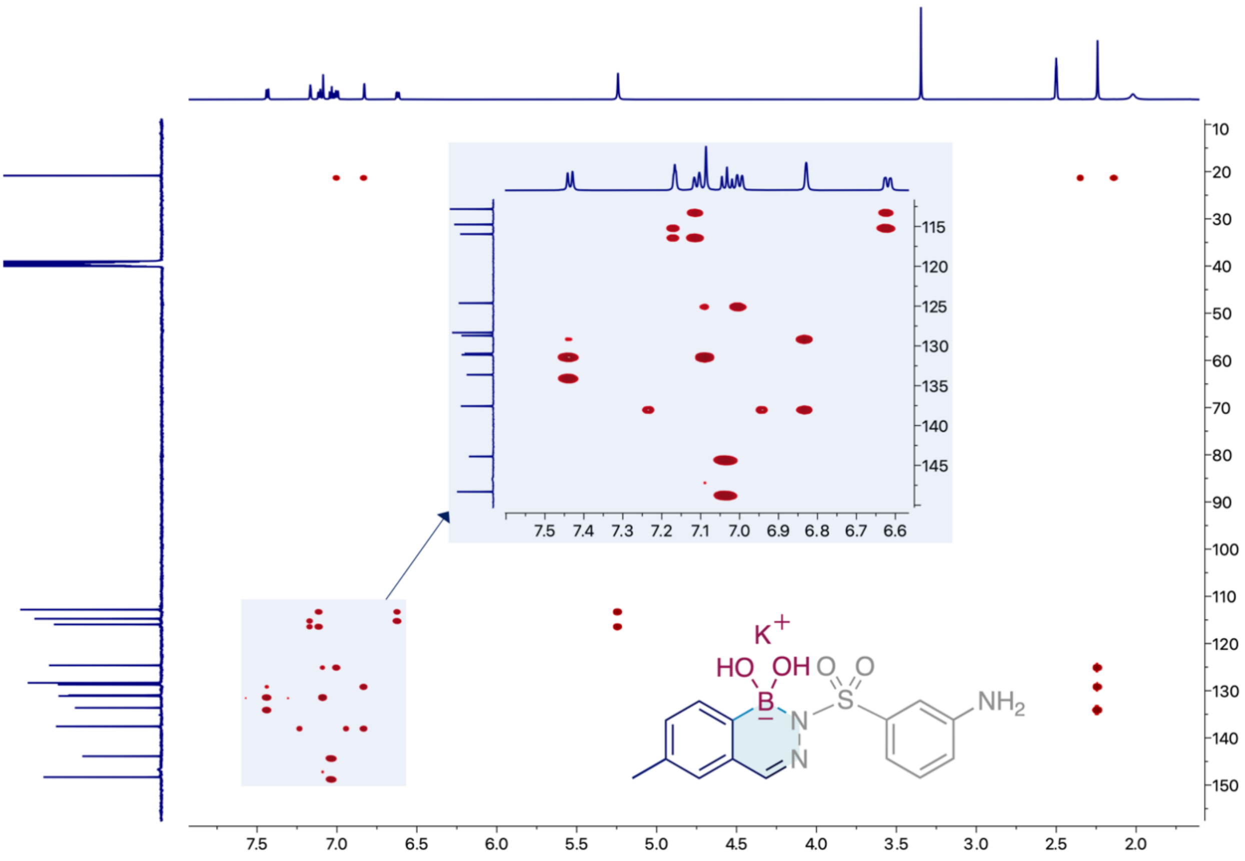

Figure S333. Diazaborine 63: ^{1}H-^{13}C gHMBC NMR (DMSO-d_6, 298 K)

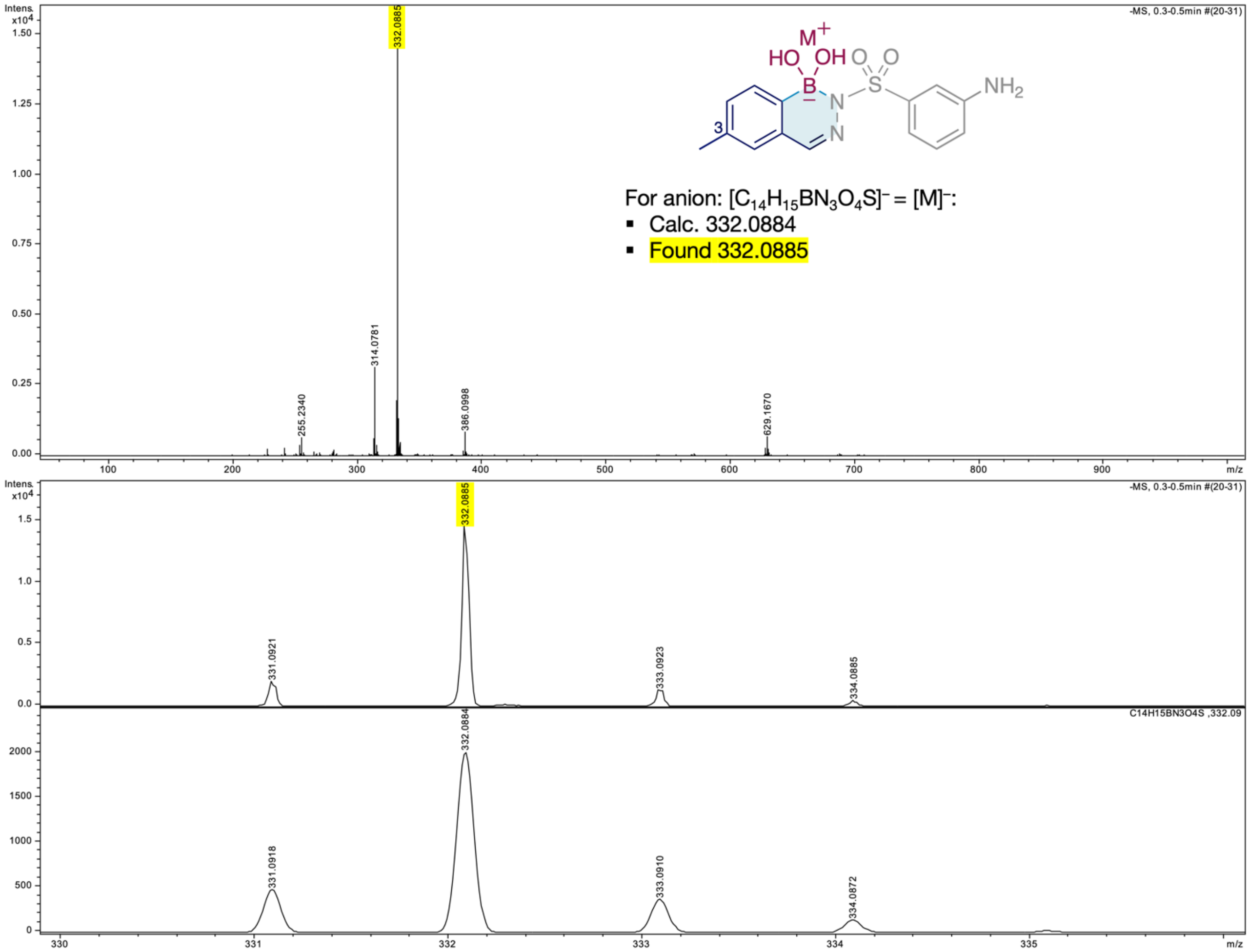

Figure S334. ESI-MS spectrum of Diazaborine 63 ([M]⁻, ionized in MeCN-H_2O 3:1, negative mode).

Sodium salt of diazaborine 13 (diazaborine 64)

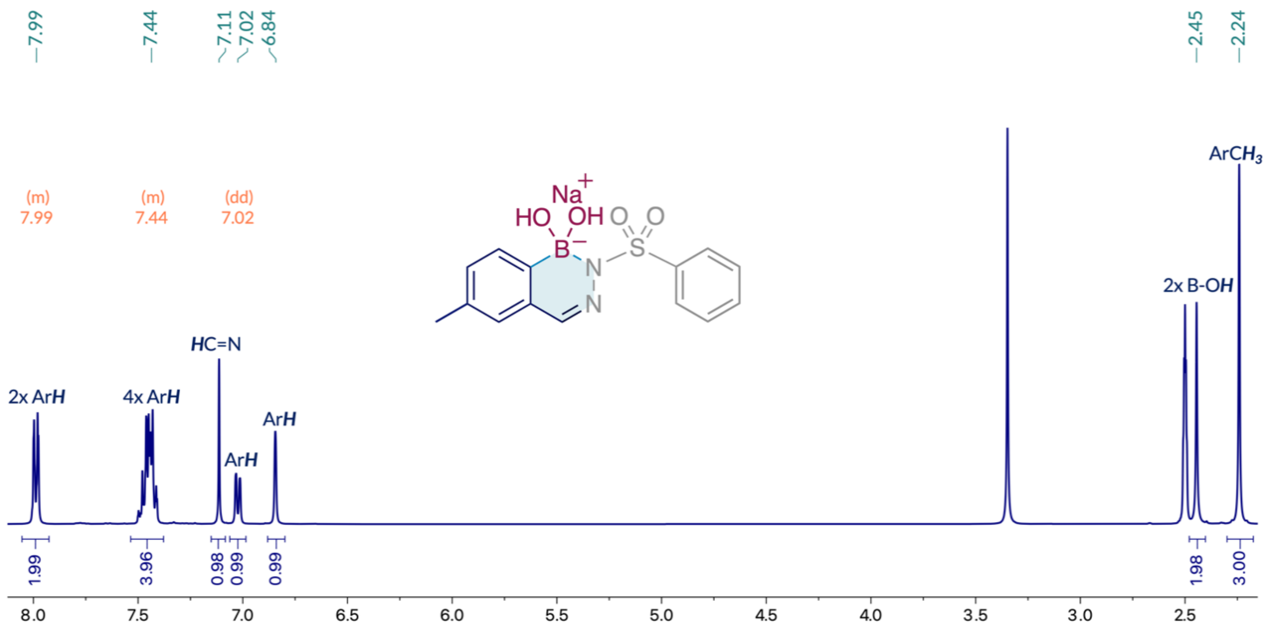

Figure S335. Diazaborine 64: ^{1}H NMR (400 MHz, D_2O, 298 K)

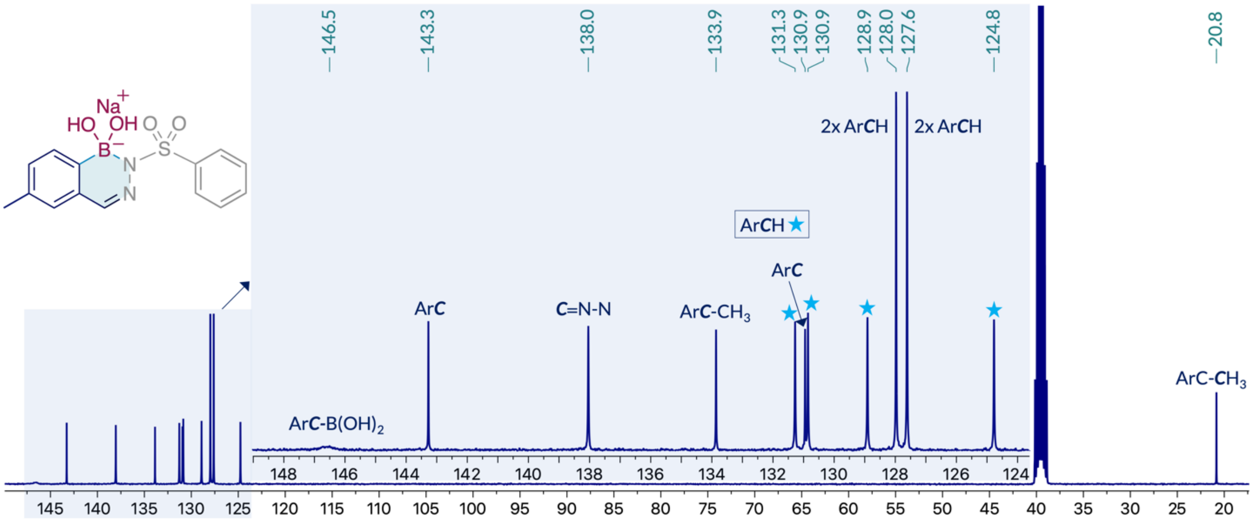

Figure S336. Diazaborine 64: ^{13}C NMR (101 MHz, D_2O, 298 K)

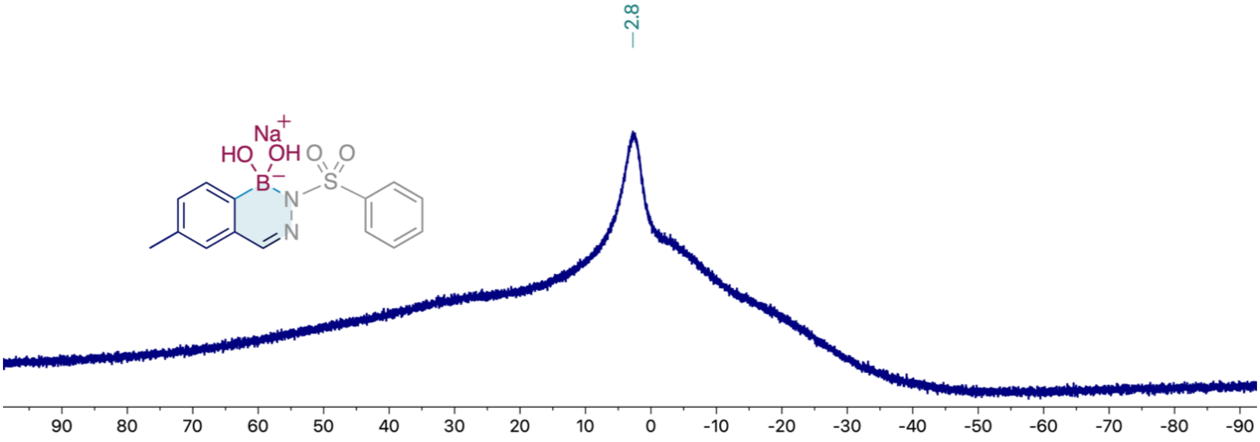

Figure S337. Diazaborine 64: ^{11}B NMR (128 MHz, D_2O, 298 K)

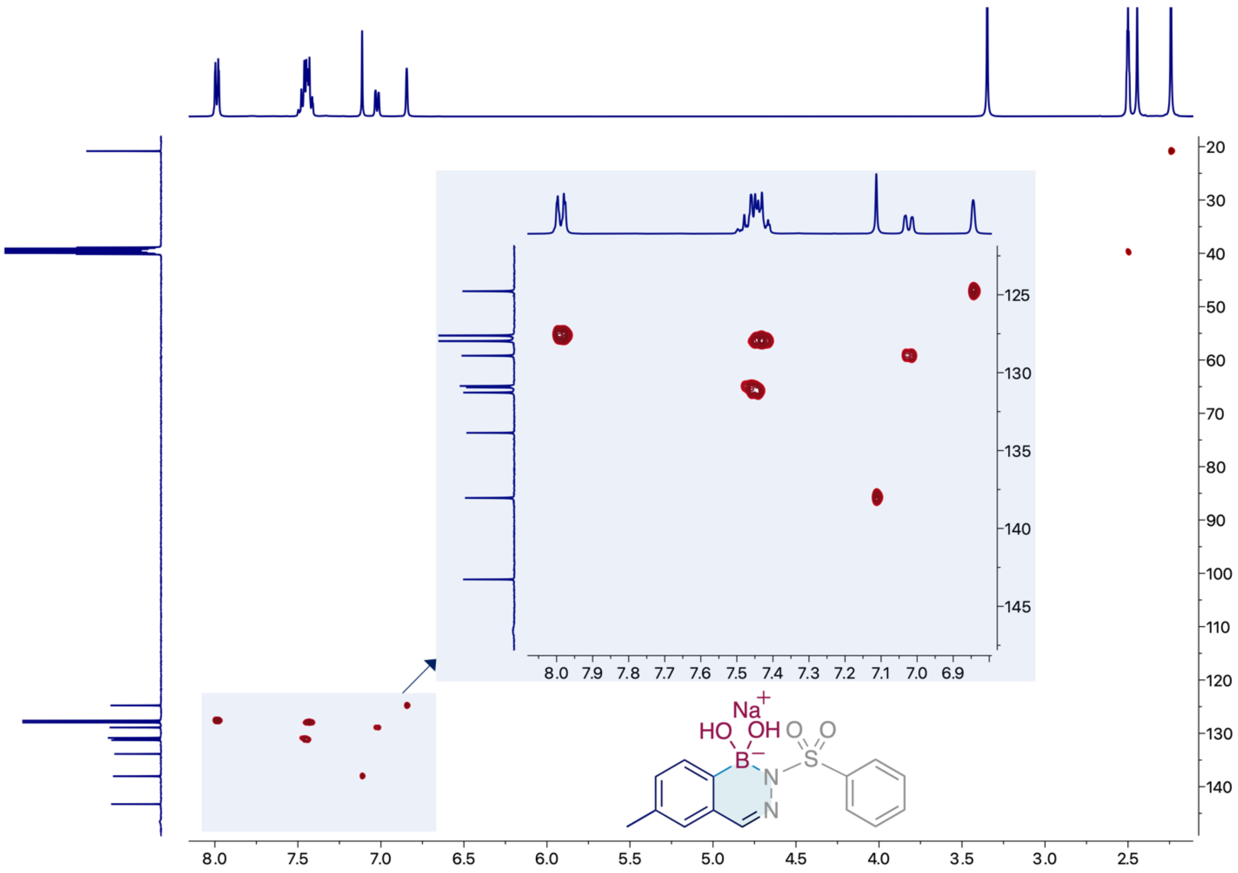

Figure S338. Diazaborine 64: ^{1}H-^{13}C gHSQC NMR (D_2O, 298 K)

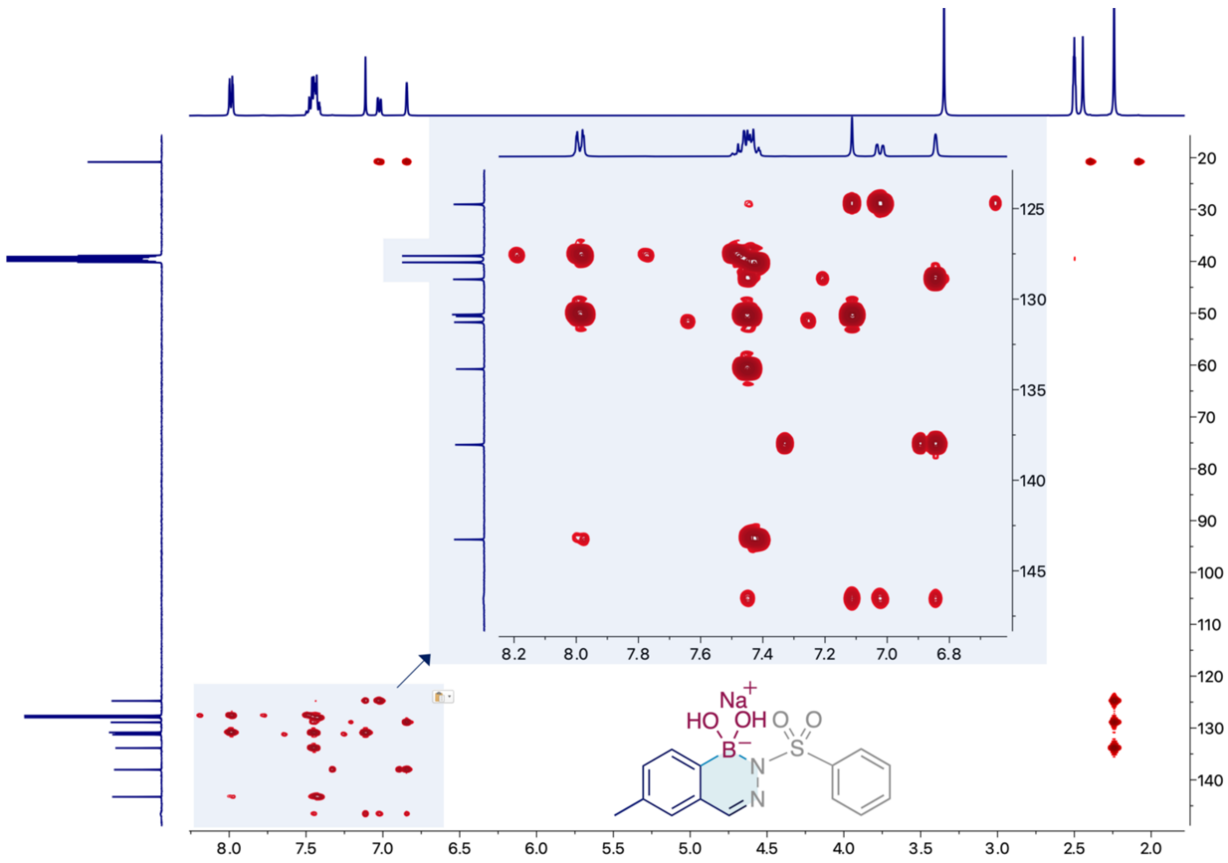

Figure S339. - Diazaborine 64: ^{1}H-^{13}C gHMBC NMR (D_2O, 298 K)

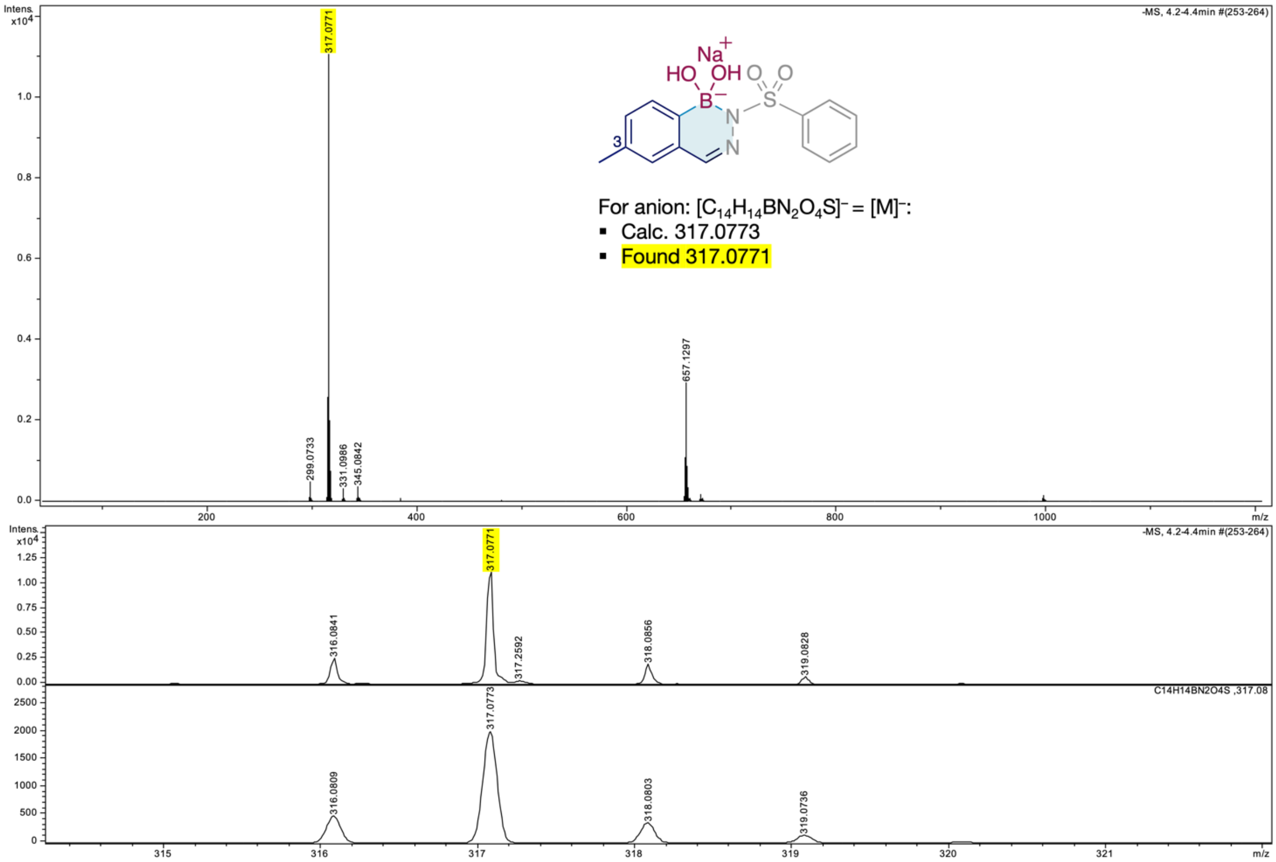

Figure S340. ESI-MS spectrum of Diazaborine 64 ($[M]^-$, ionized in H_2O, negative mode).

Sodium salt of diazaborine 41 (diazaborine 65)

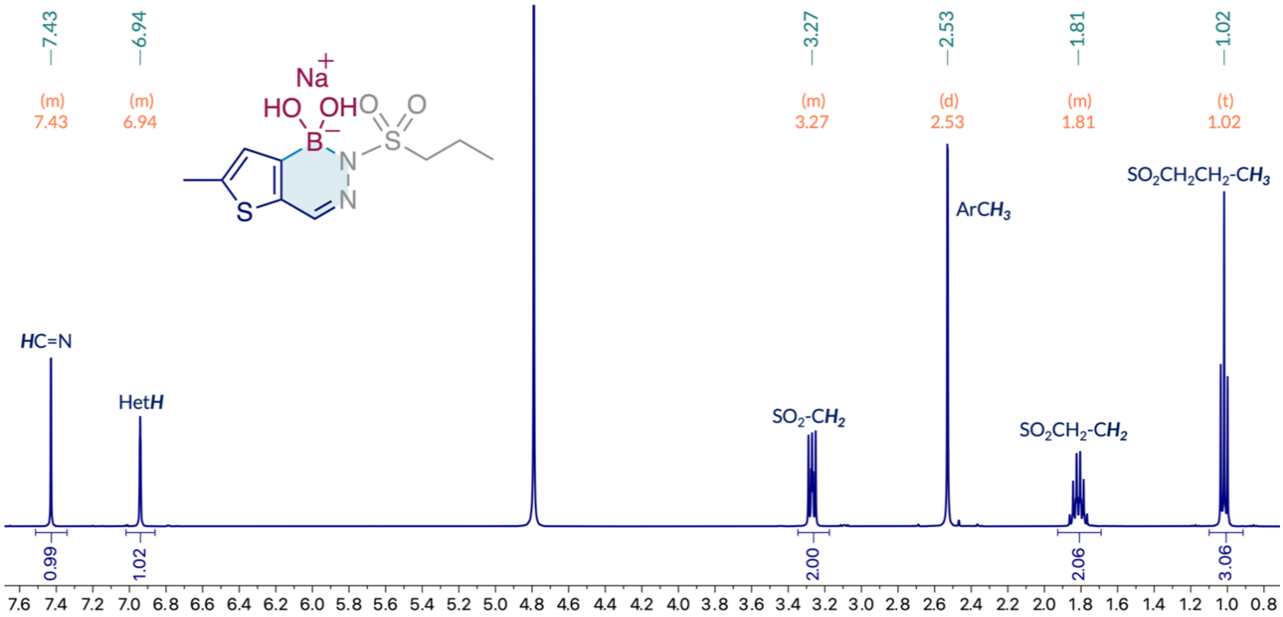

Figure S341. Diazaborine 65: ^{1}H NMR (400 MHz, D_2O, 298 K)

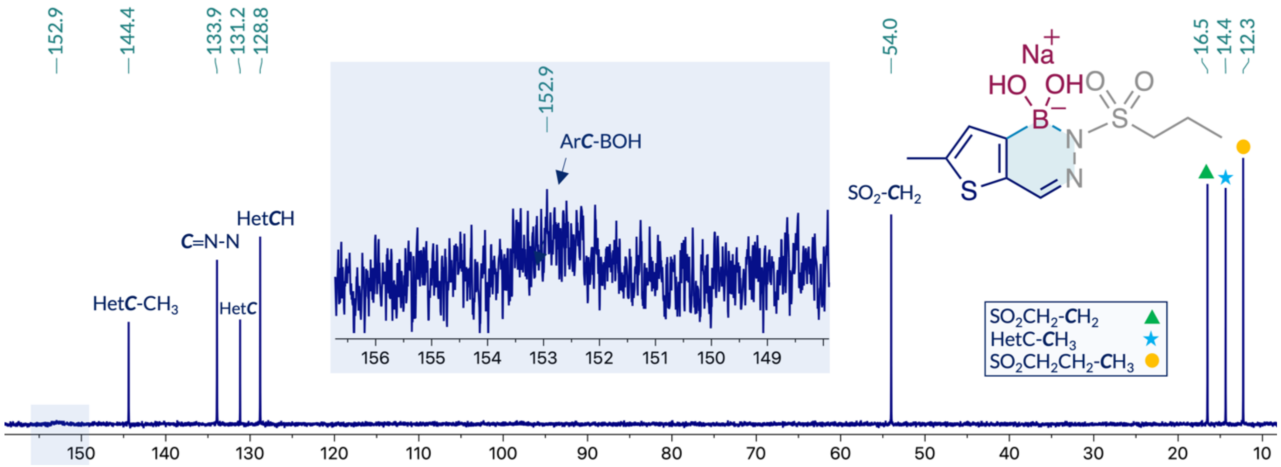

Figure S342. Diazaborine 65: ^{13}C NMR (101 MHz, D_2O, 298 K)

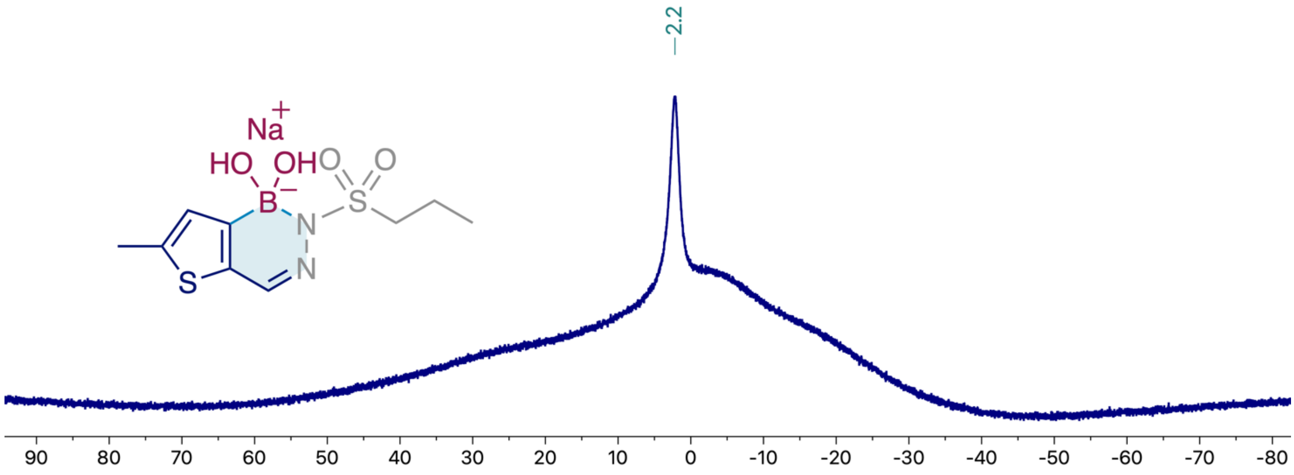

Figure S343. Diazaborine 65: ^{11}B NMR (128 MHz, D_2O, 298 K)

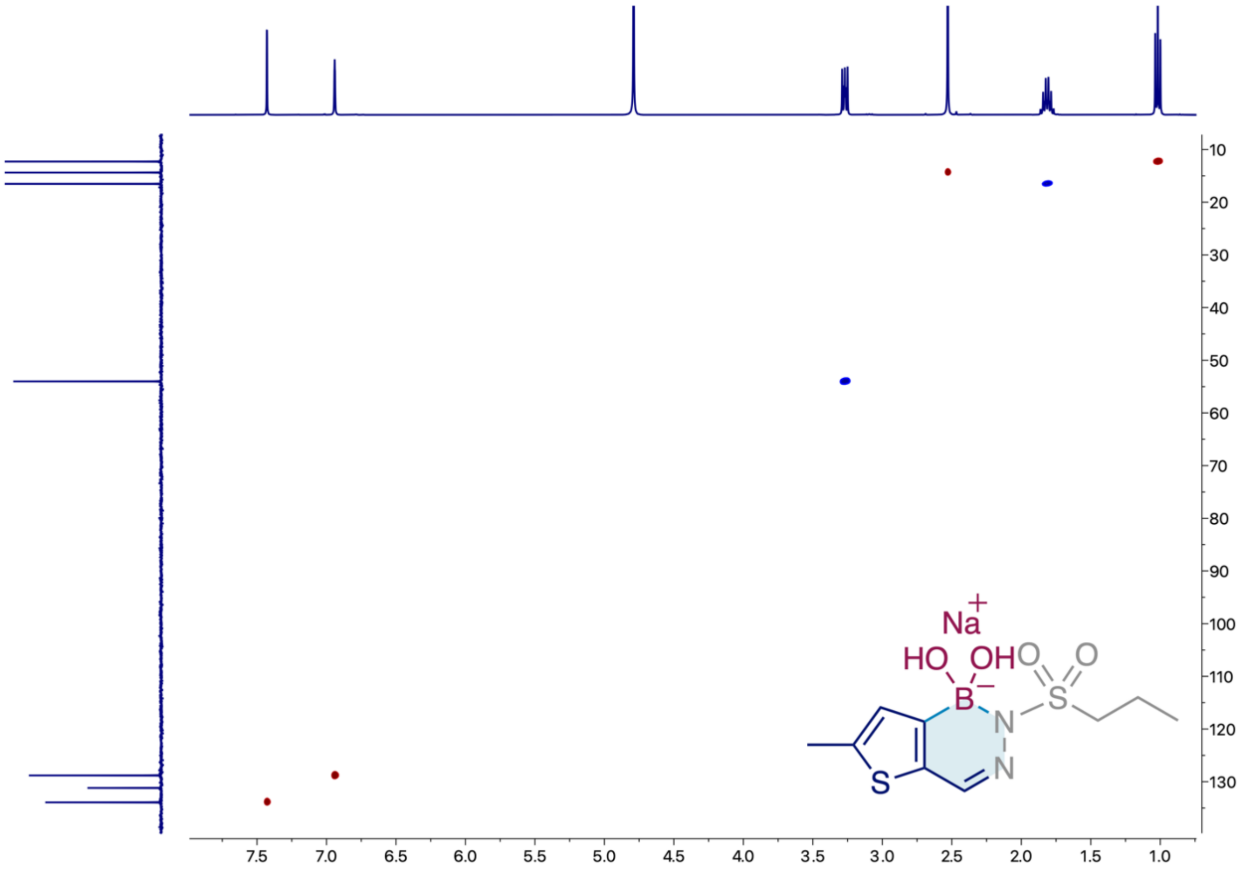

Figure S344. Diazaborine 65: ^{1}H-^{13}C gHSQC NMR (D_2O, 298 K)

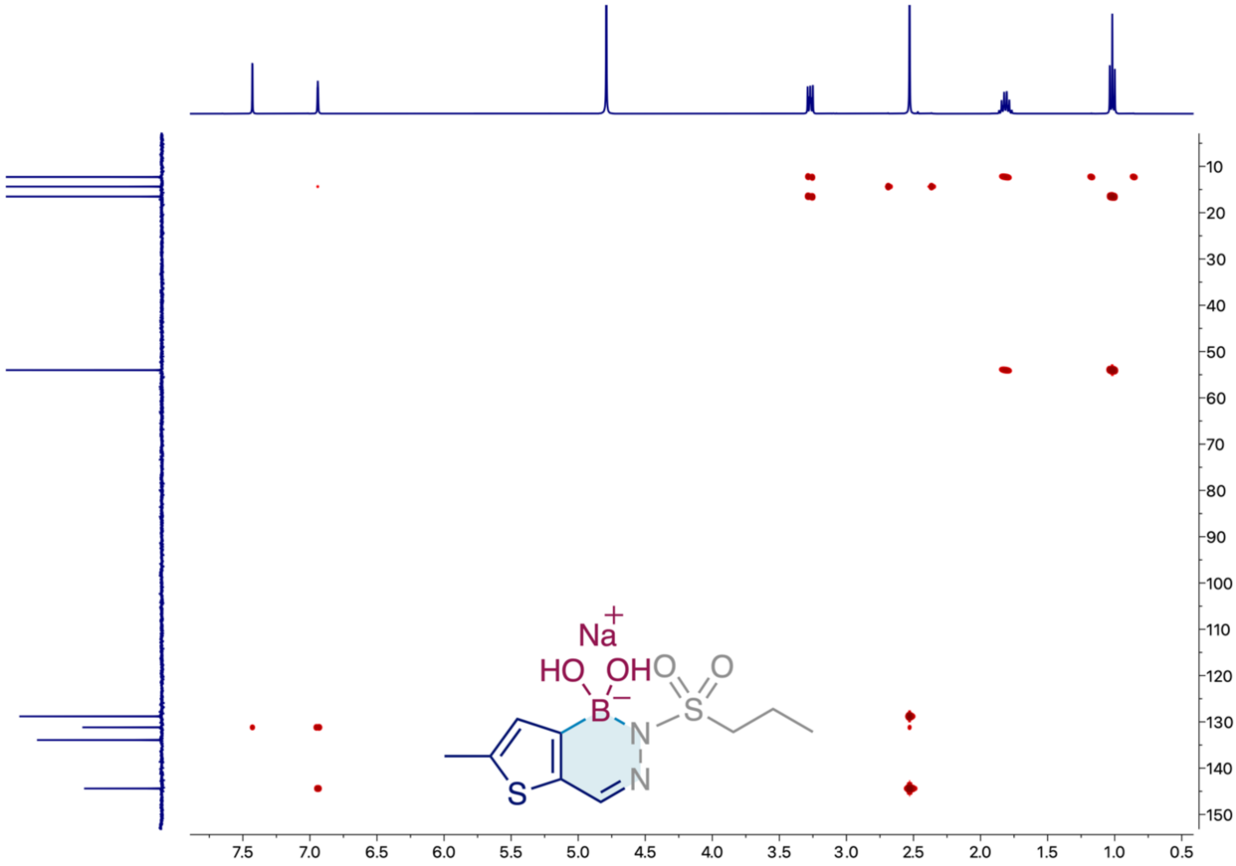

Figure S345. Diazaborine 65: ^{1}H-^{13}C gHMBC NMR (D_2O, 298 K)

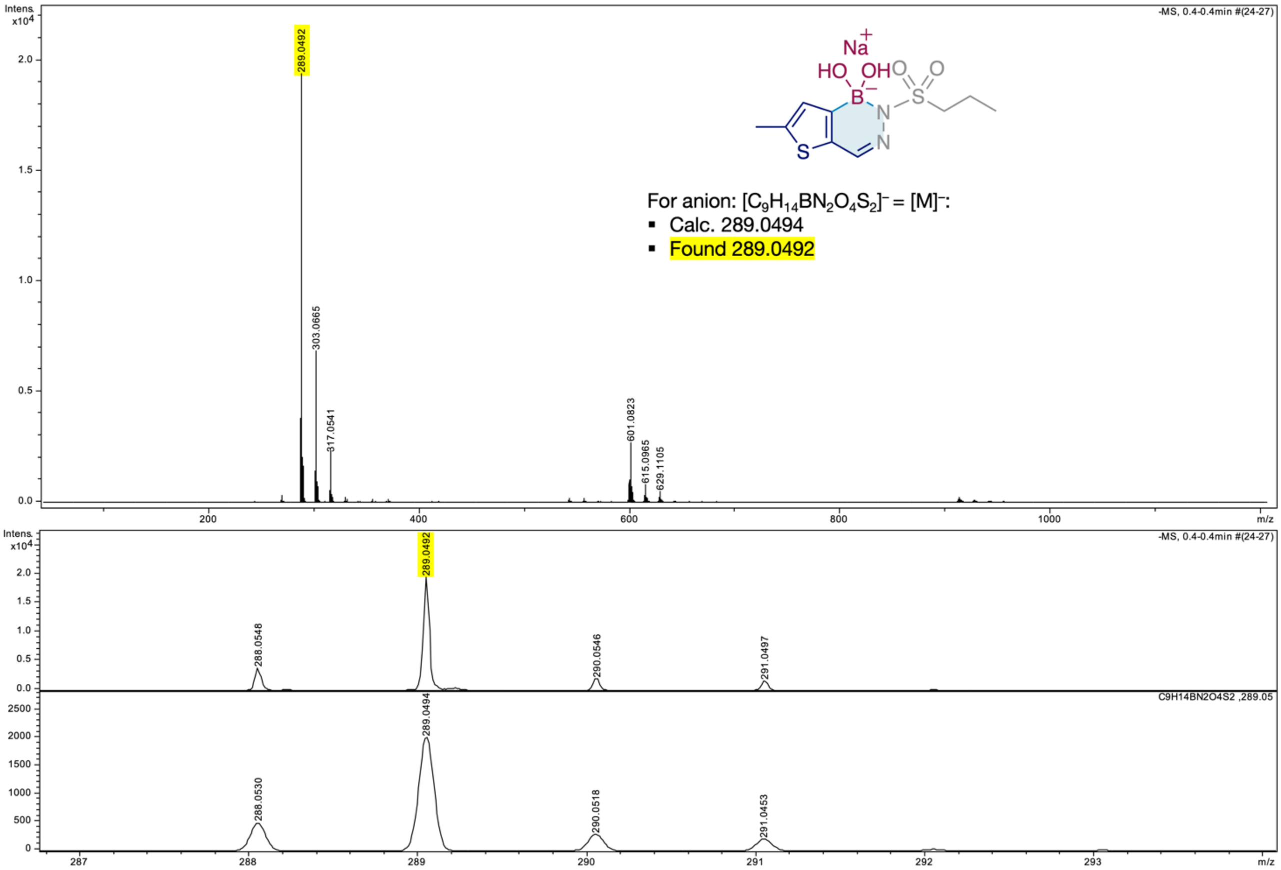

Figure S346. ESI-MS spectrum of Diazaborine 65 ($[M]^-$, ionized in MeOH-H_2O 1:2, negative mode)

Sodium salt of diazaborine 47 (diazaborine 66)

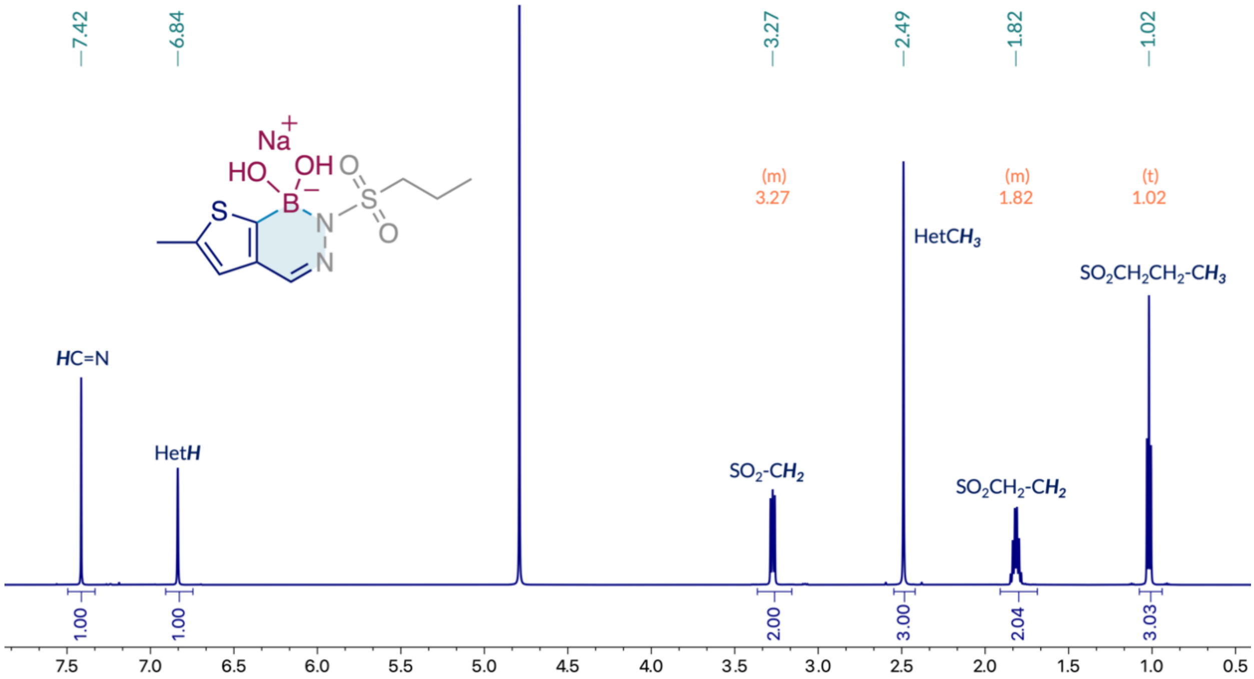

Figure S347. Diazaborine 66: ^{1}H NMR (600 MHz, D_2O, 298 K)

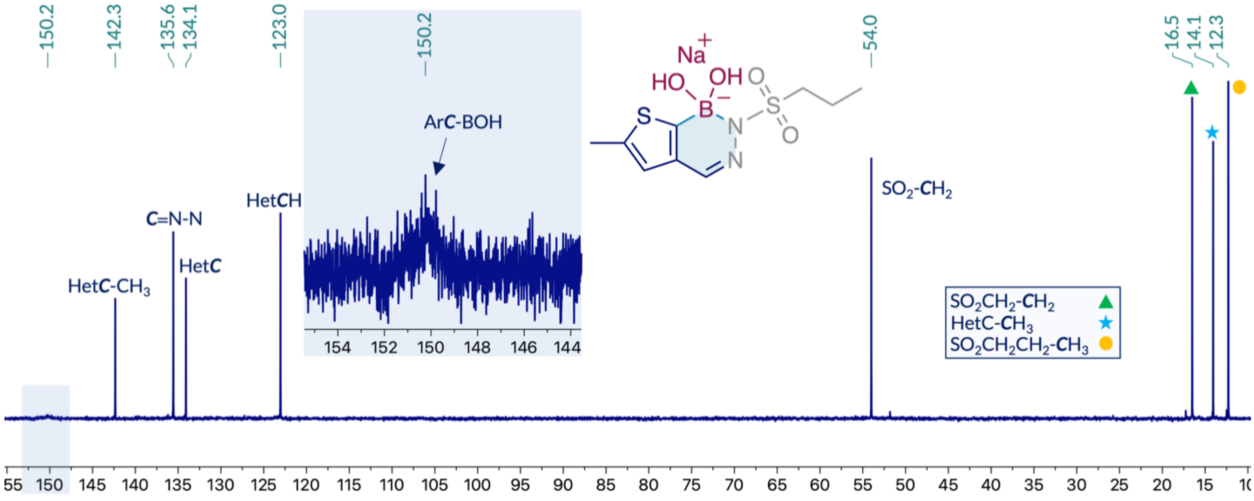

Figure S348. Diazaborine 66: ^{13}C NMR (101 MHz, D_2O, 298 K)

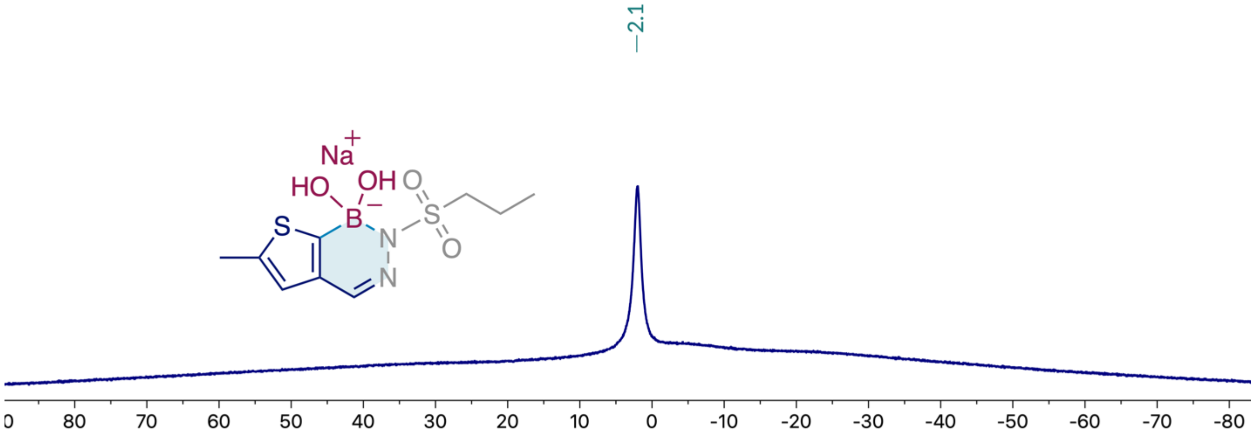

Figure S349. Diazaborine 66: ^{11}B NMR (128 MHz, D_2O, 298 K)

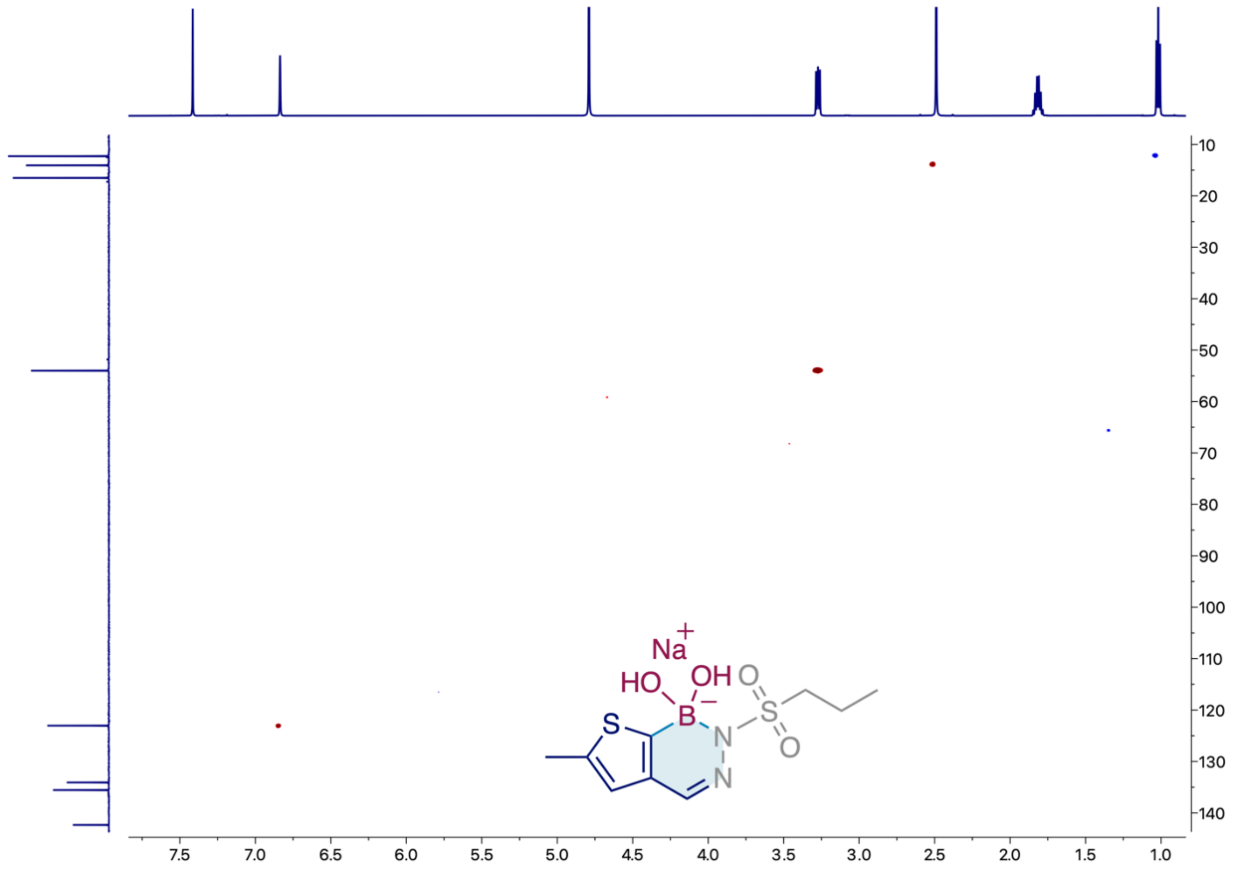

Figure S350. Diazaborine 66: ^1H-^{13}C gHSQC NMR (D_2O, 298 K)

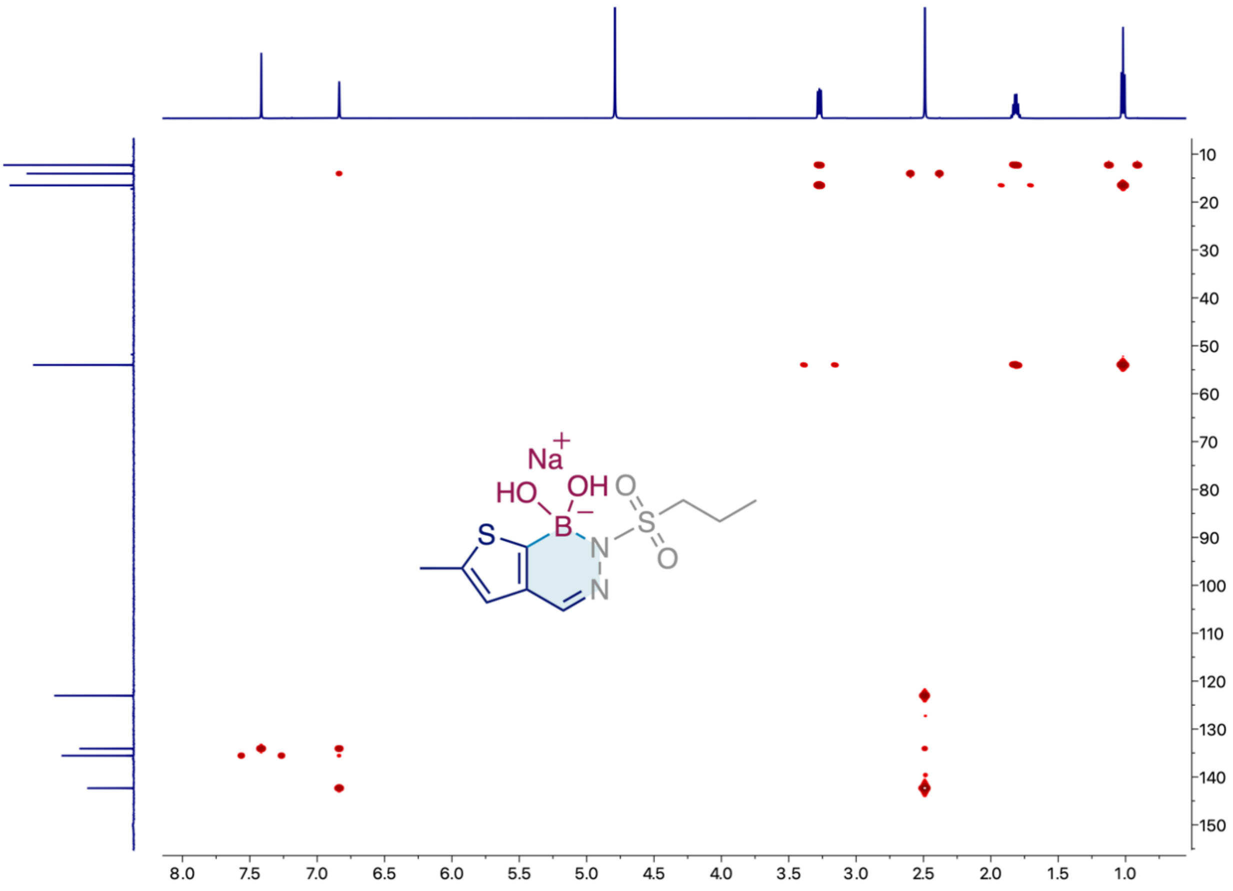

Figure S351. Diazaborine 66: ^1H-^{13}C gHMBC NMR (D_2O, 298 K)

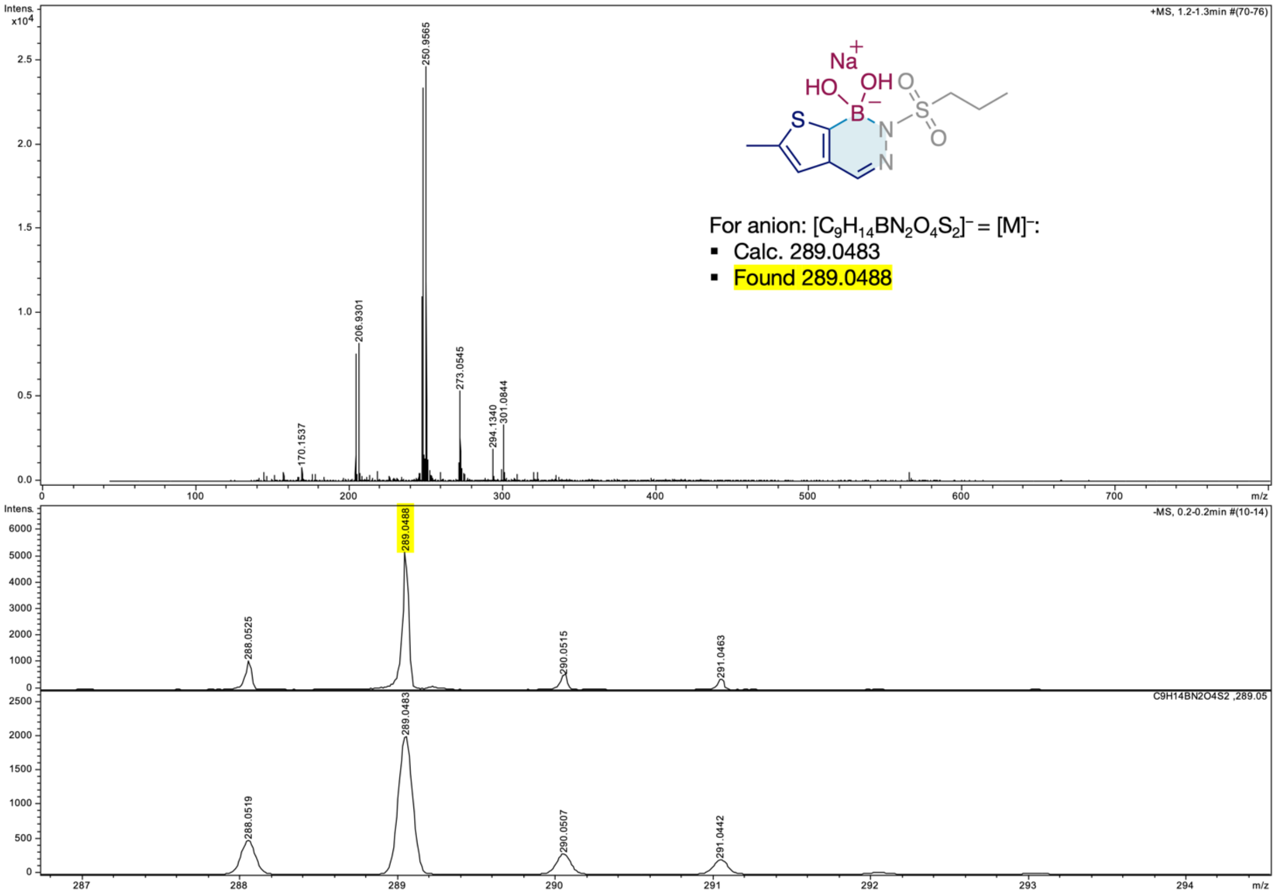

Figure S352. ESI-MS spectrum of Diazaborine 66 ([M]⁻, ionized in MeOH-H_2O 10:1, negative mode)

Conjugates

Conjugates Diazaborine-Phosphonium salt

Conjugate 67

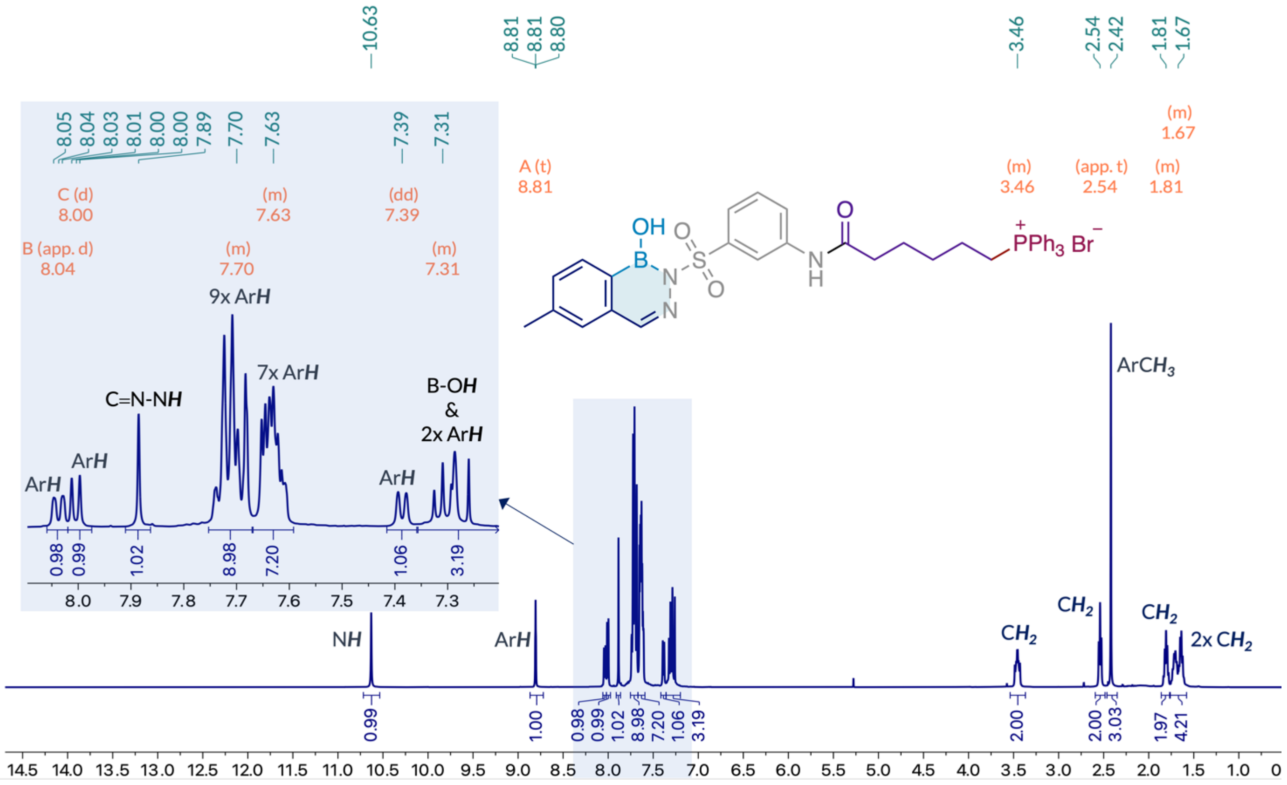

Figure S353. Conjugate 67: ^{1}H NMR (500 MHz, $CDCl_3$, 298 K)

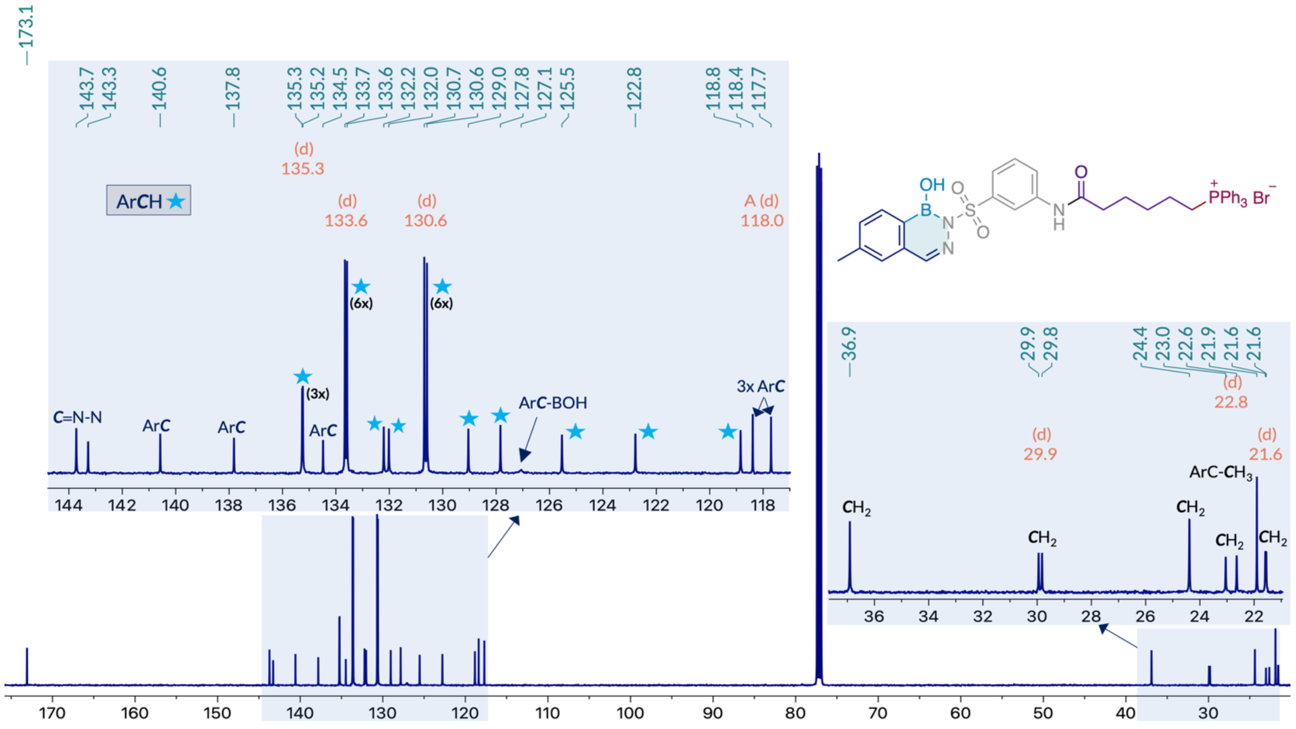

Figure S354. Conjugate 67: ^{13}C NMR (126 MHz, $CDCl_3$, 298 K)

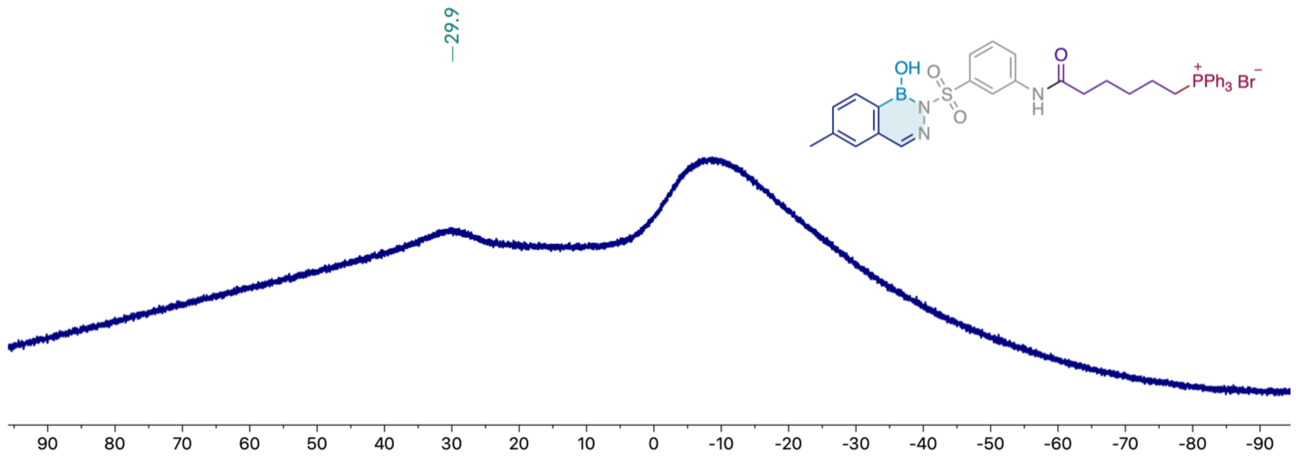

Figure S355. Conjugate 67:^{11}B NMR (160 MHz, $CDCl_3$, 298 K)

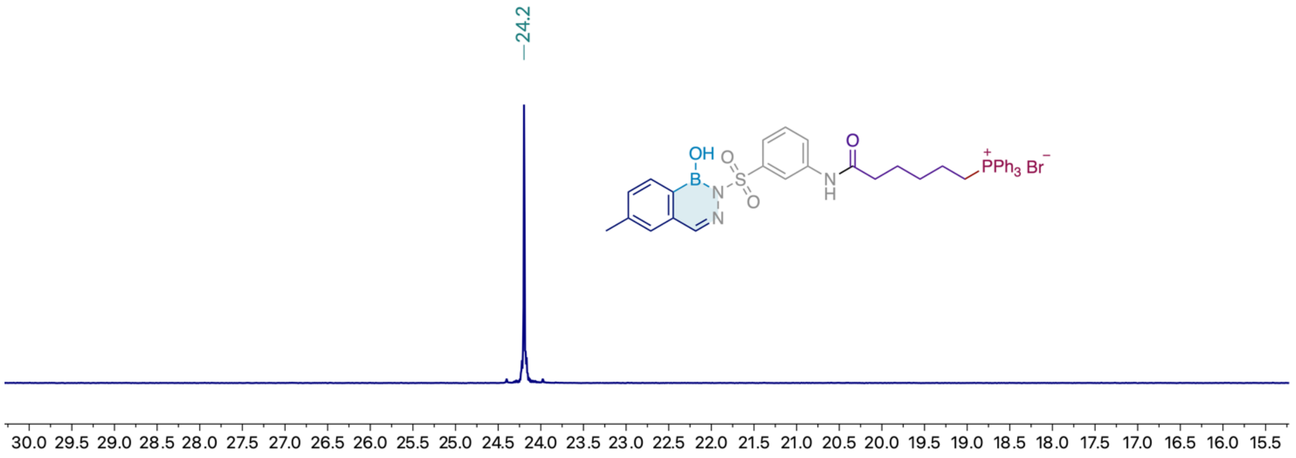

Figure S356. Conjugate 67: $^{31}P\{^1H\}$ NMR (202 MHz, $CDCl_3$, 298 K)

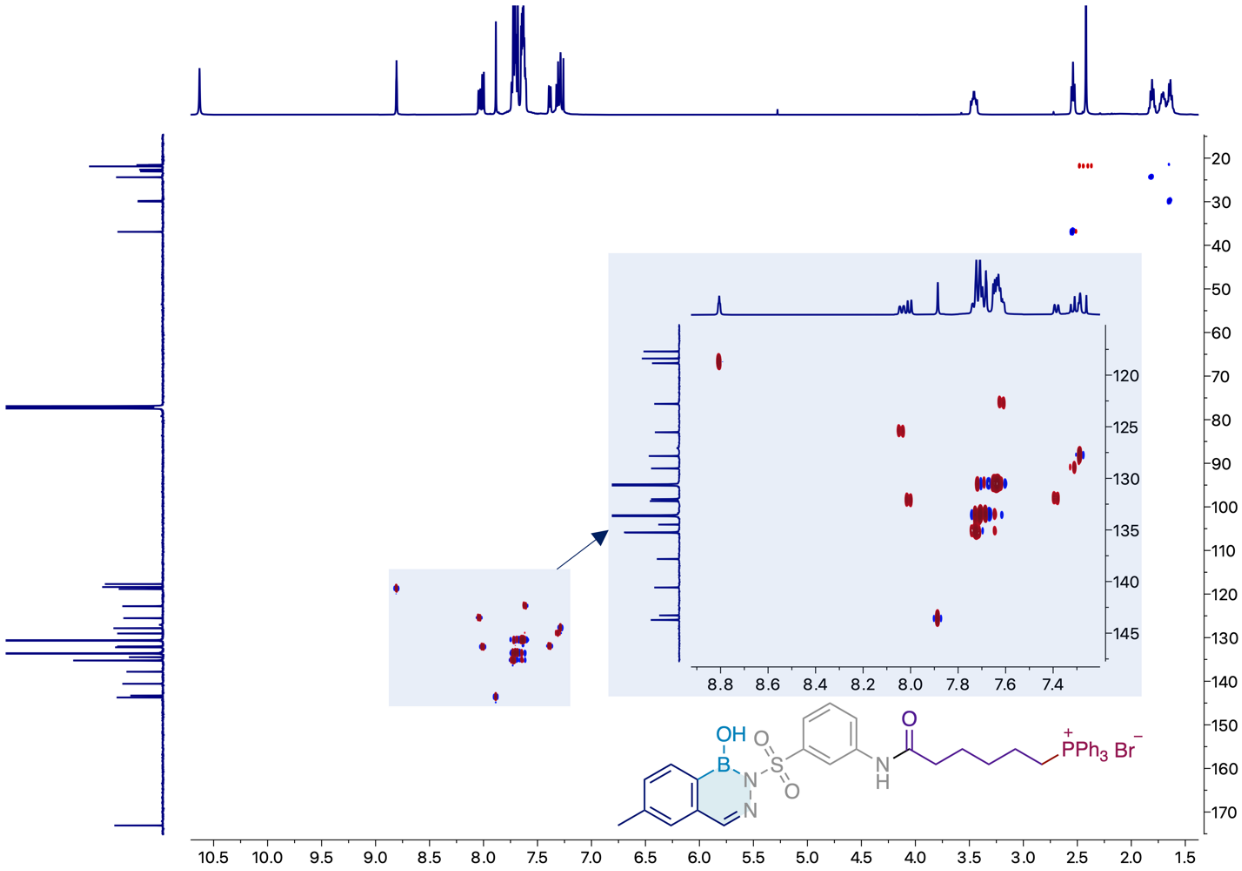

Figure S357. Conjugate 67: ^{1}H-^{13}C gHSQC NMR ($CDCl_3$, 298 K)

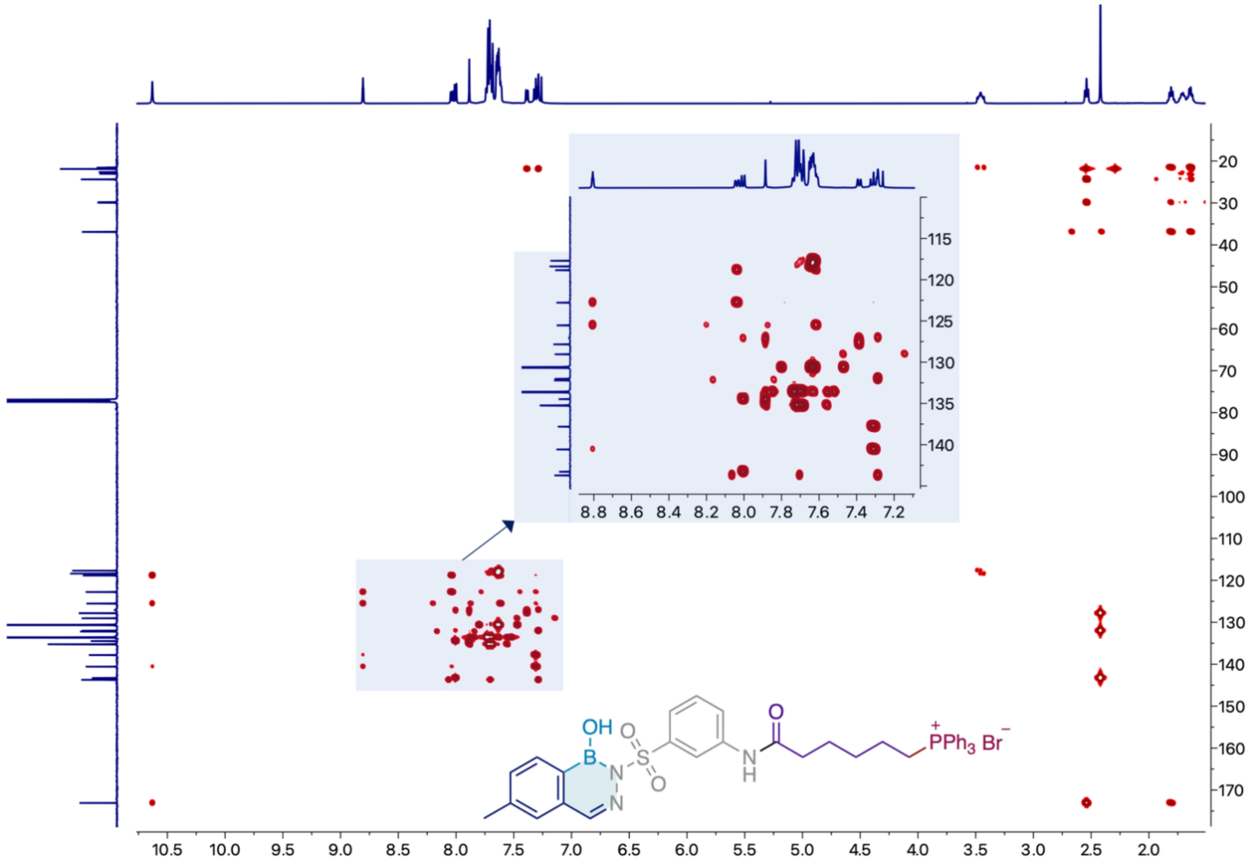

Figure S358. Conjugate 67: ^{1}H-^{13}C gHMBC NMR ($CDCl_3$, 298 K)

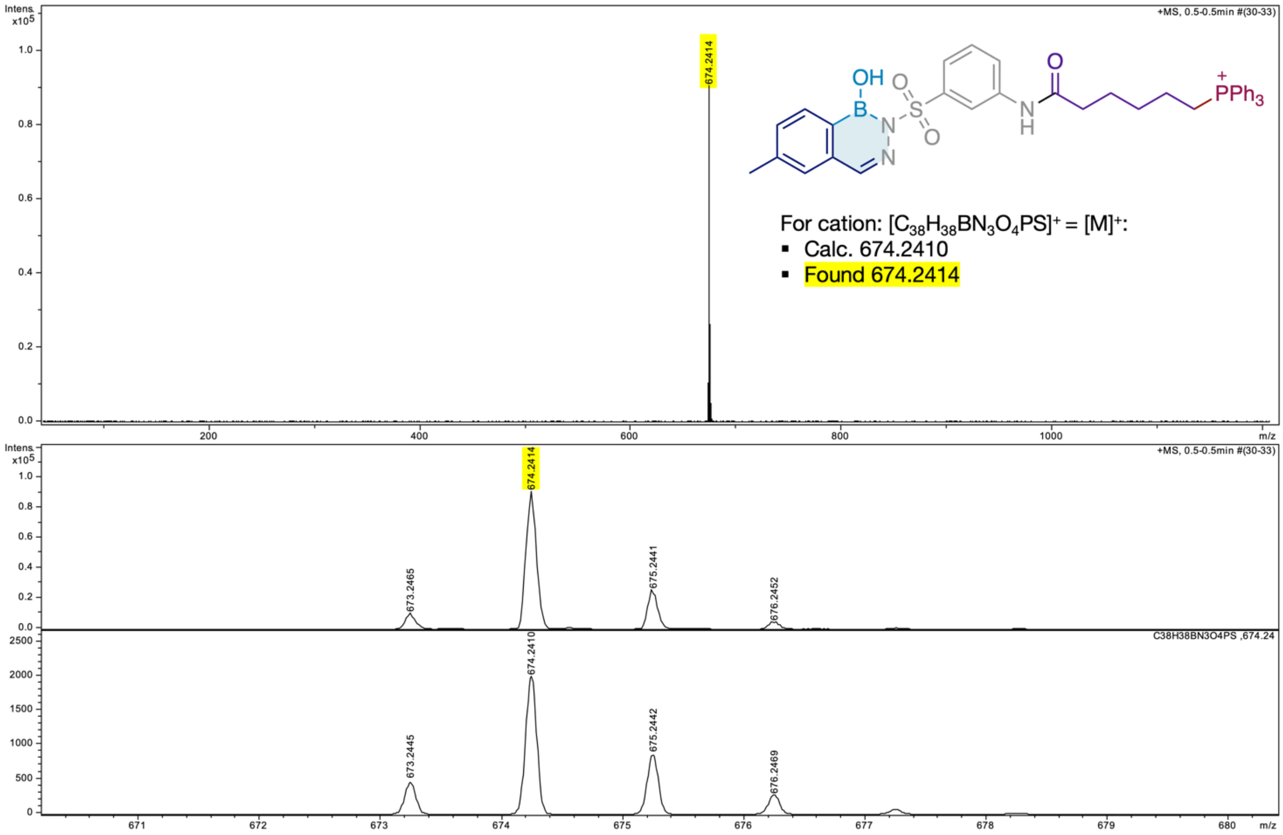

Figure S359. ESI-MS spectrum of Conjugate 67 ($[M]^+$, ionized in CH_3CN-H_2O 4:1, positive mode)

Conjugate 68

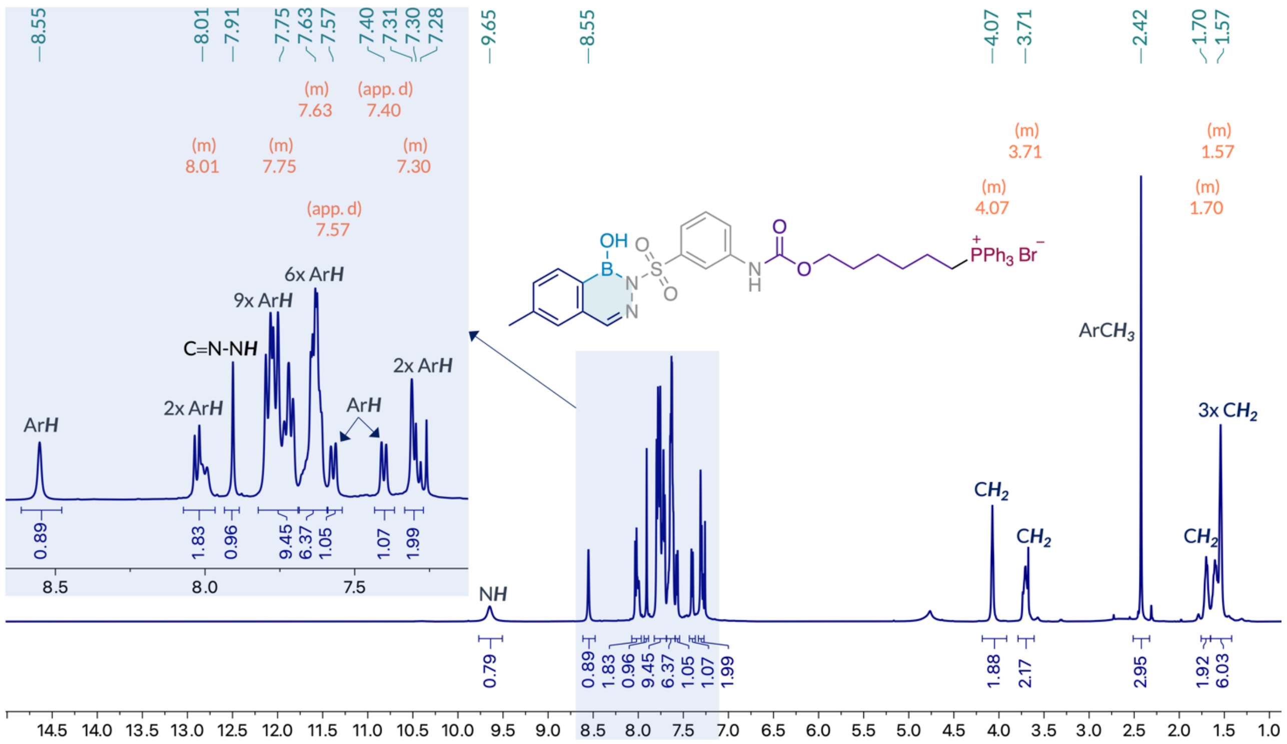

Figure S360. Conjugate 68:^{1}H NMR (500 MHz, $CDCl_3$, 298 K)

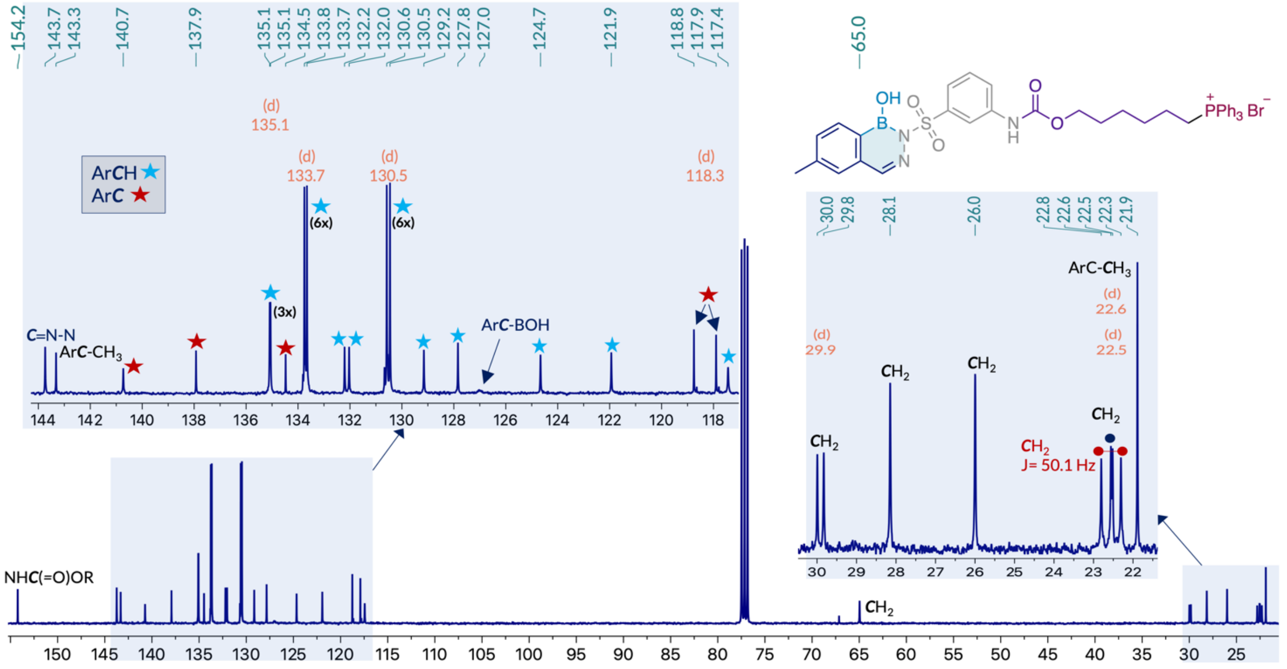

Figure S361. Conjugate 68:^{13}C NMR (101 MHz, $CDCl_3$, 298 K)

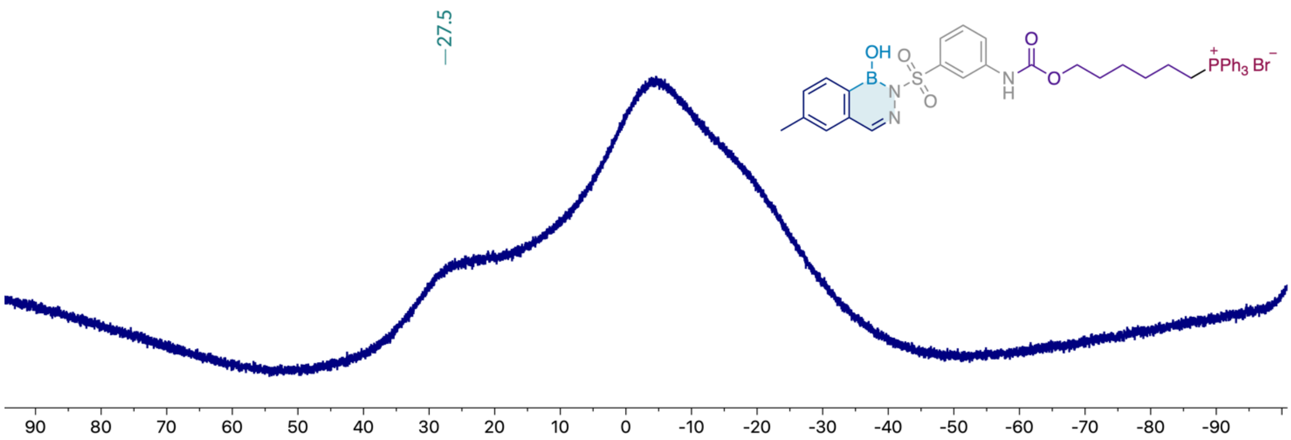

Figure S362. Conjugate 68:^{11}B NMR (128 MHz, $CDCl_3$, 298 K)

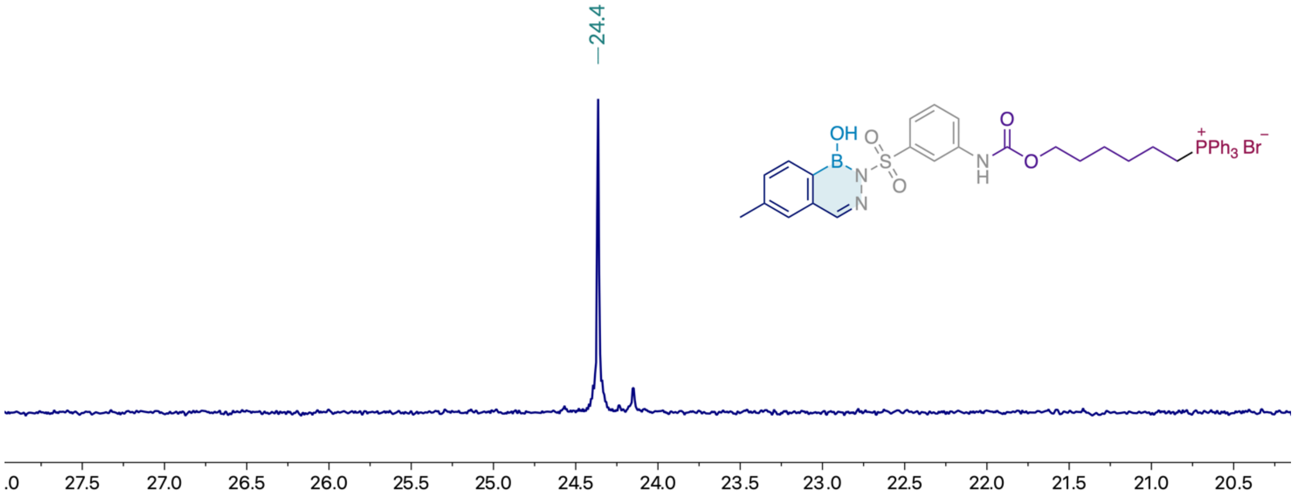

Figure S363. Conjugate 68: $^{31}P\{^{1}H\}$ NMR (202 MHz, $CDCl_3$, 298 K)

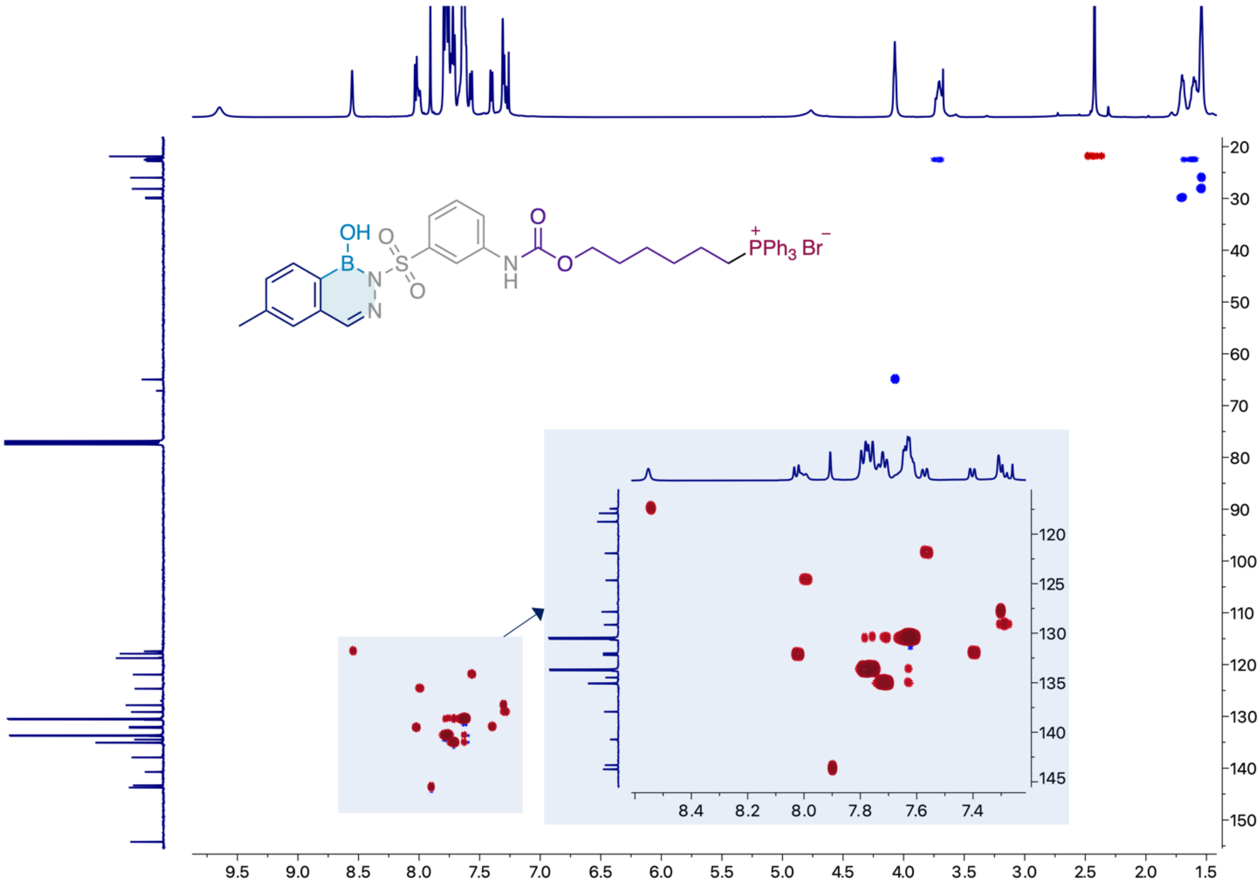

Figure S364. Conjugate 68: ^{1}H-^{13}C gHSQC NMR ($CDCl_3$, 298 K)

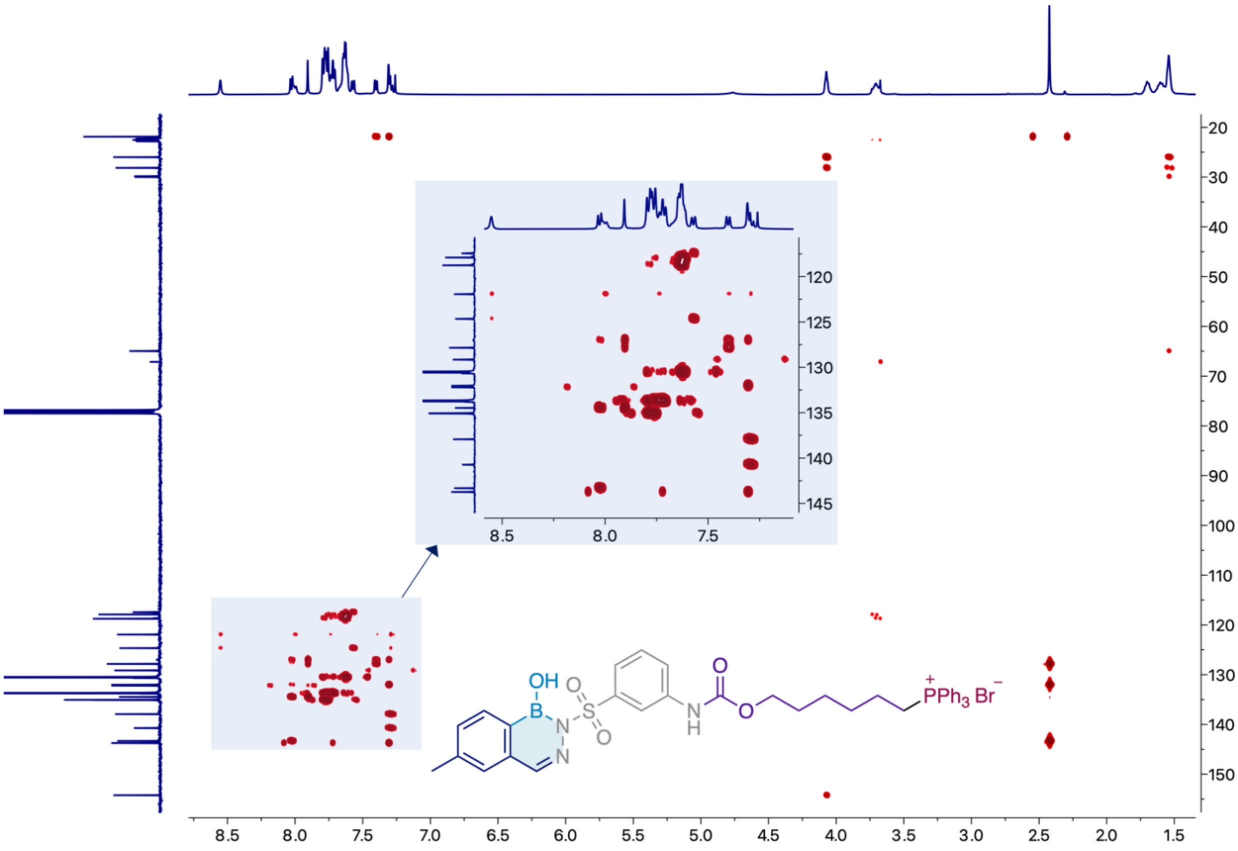

Figure S365. Conjugate 68: ^{1}H-^{13}C gHMBC NMR ($CDCl_3$, 298 K)

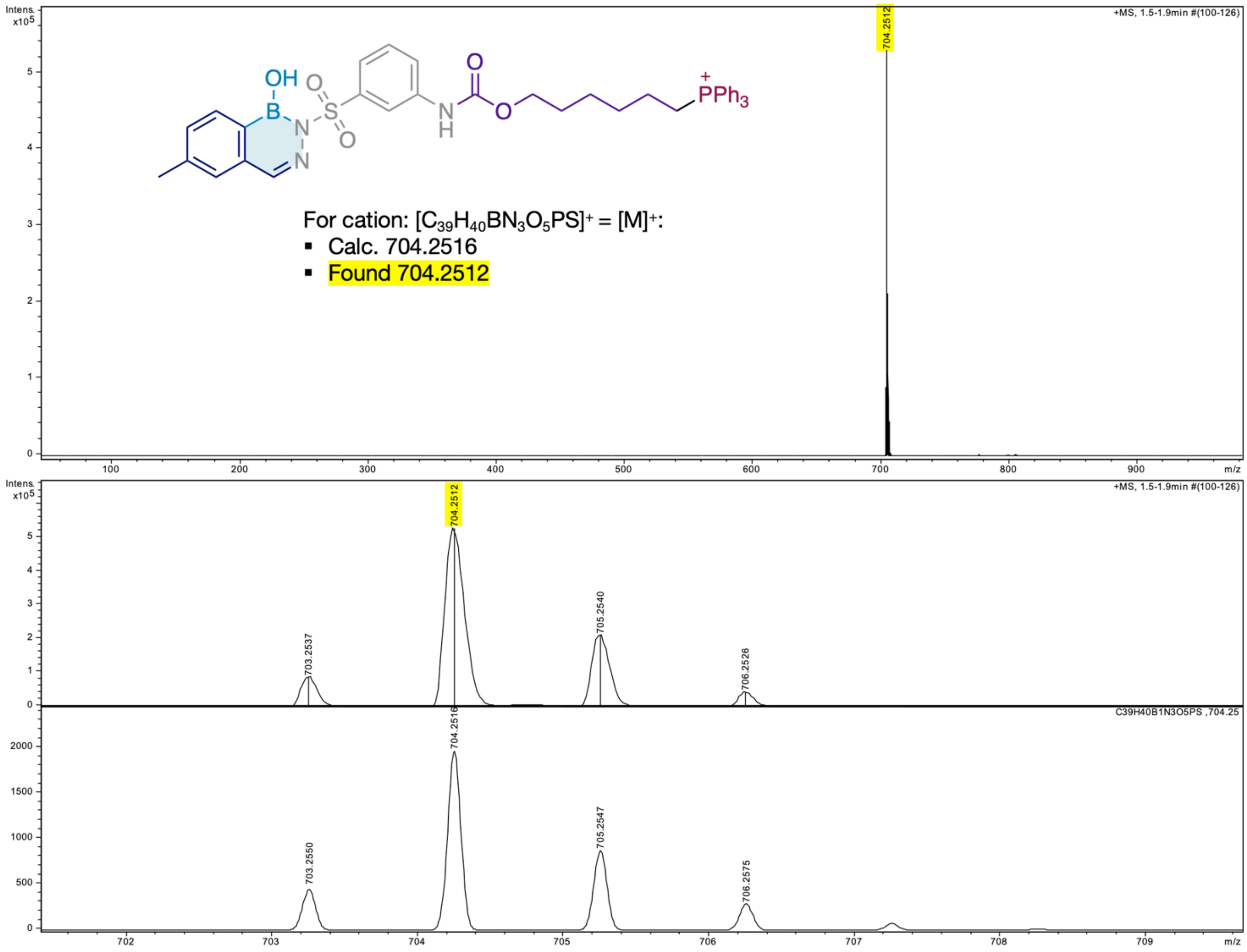

Figure S366. ESI-MS spectrum of Conjugate 68, ($[M]^+$, ionized in CH_3CN-H_2O 4:1, positive mode).

Conjugates Diazaborine-Aminoacid

Conjugate 69

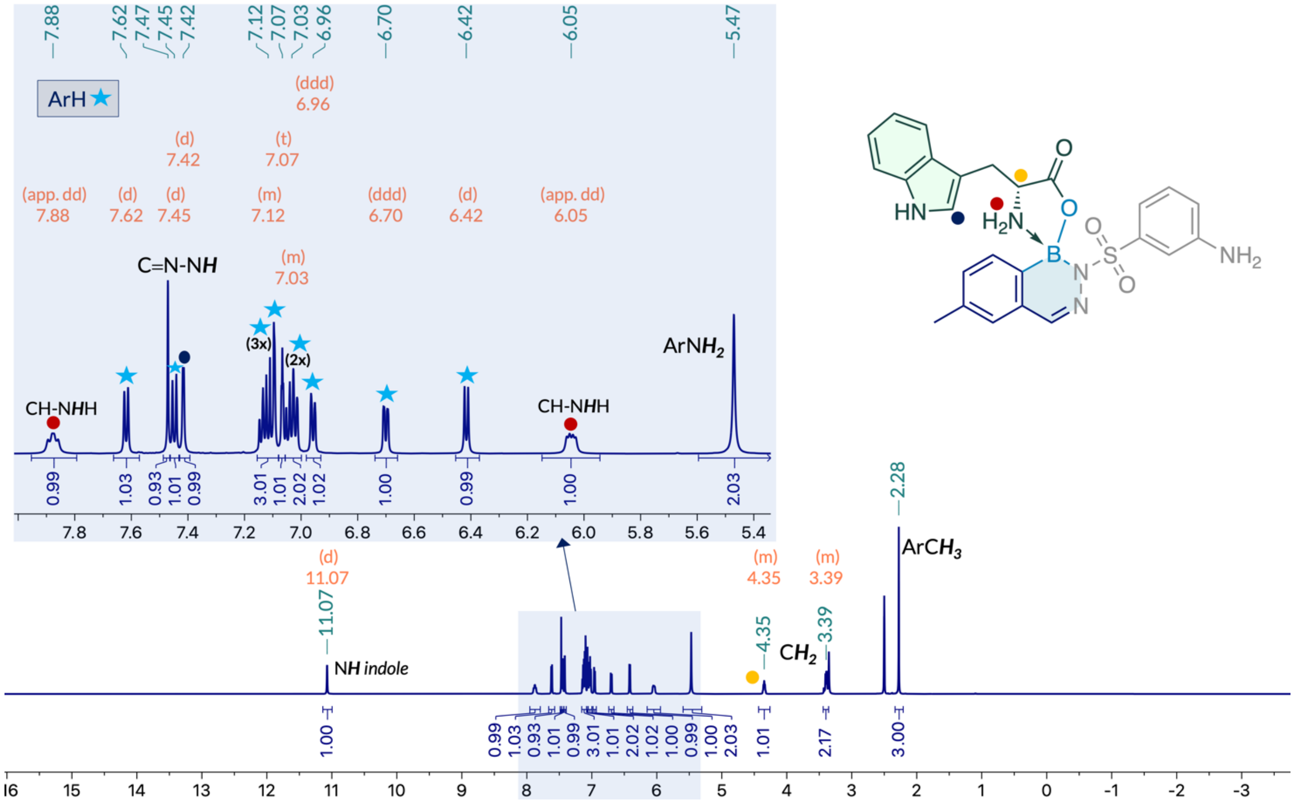

Figure S367. Conjugate 69:^{1}H NMR (600 MHz, DMSO-d_6, 298 K)

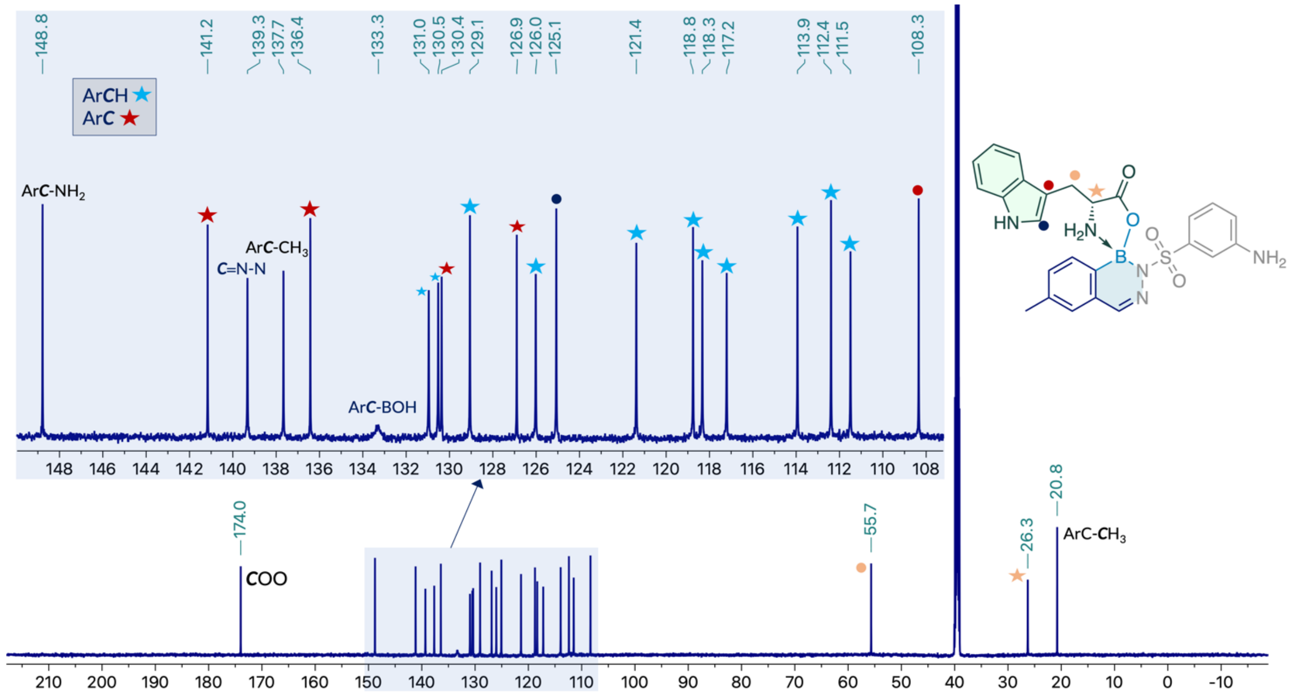

Figure S368. Conjugate 69:^{13}C NMR (151 MHz, DMSO-d_6, 298 K)

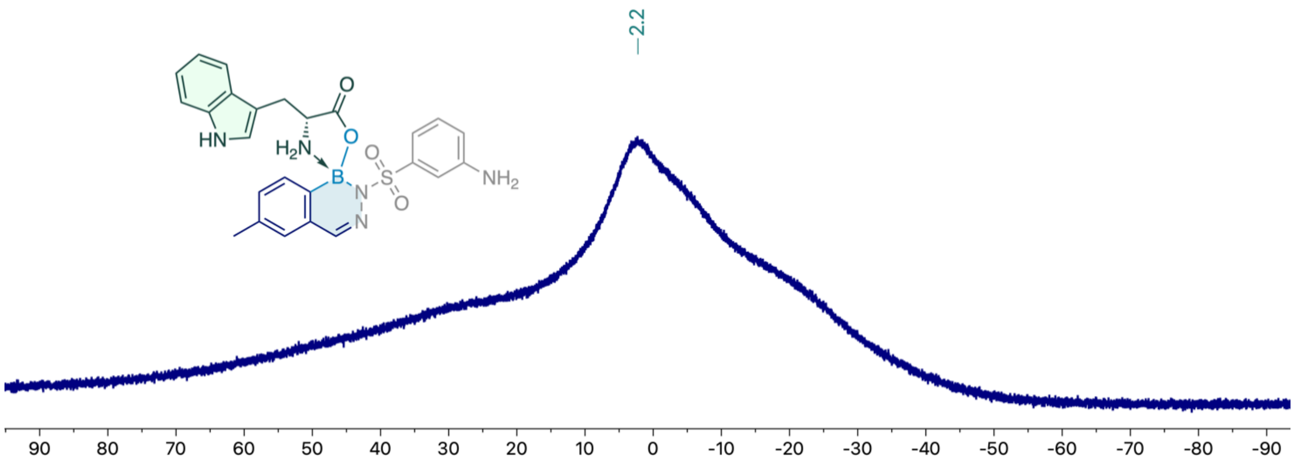

Figure S369. Conjugate 69:^{11}B NMR (128 MHz, DMSO-d_6, 298 K)

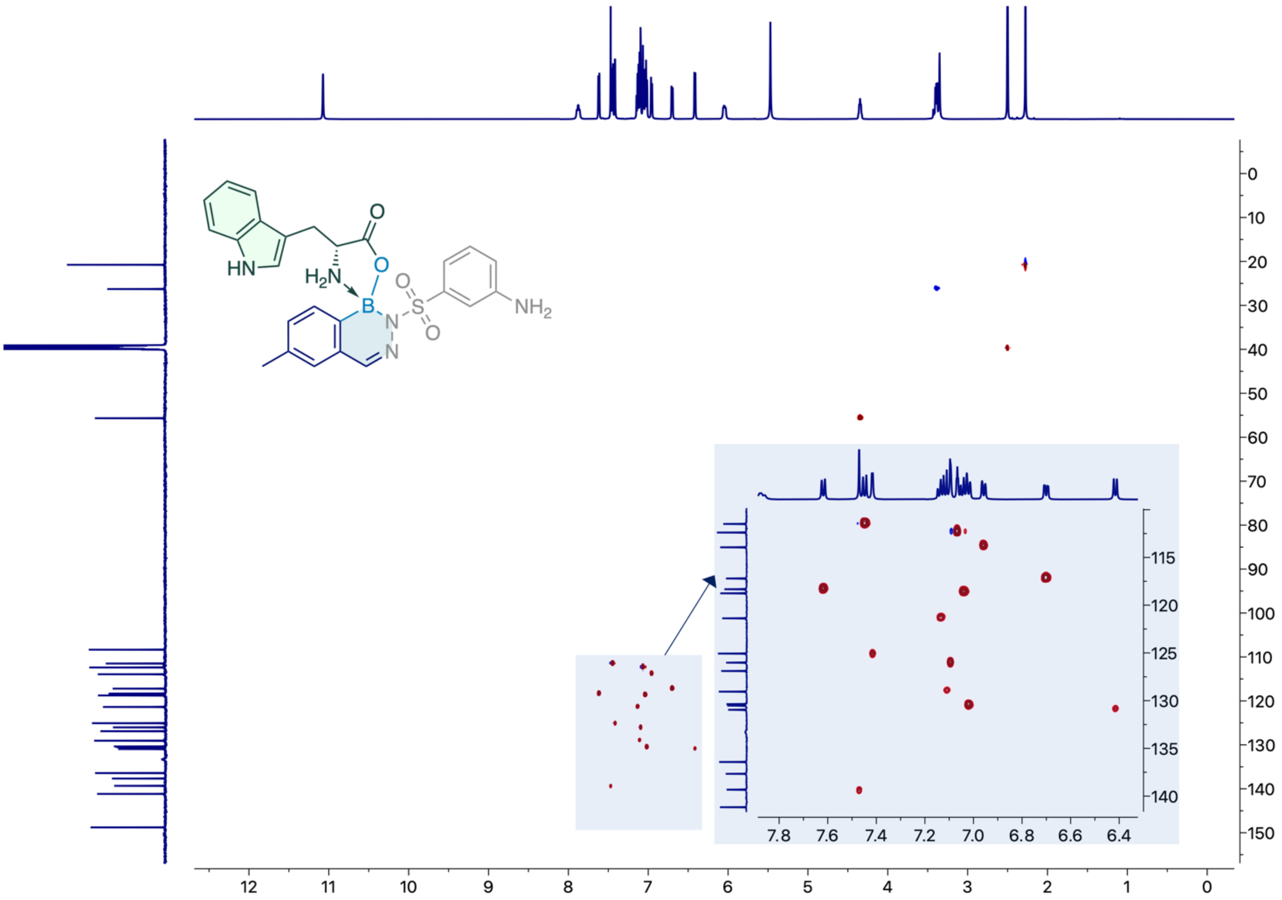

Figure S370. Conjugate 69: ^{1}H-^{13}C gHSQC NMR (DMSO-d_6, 298 K)

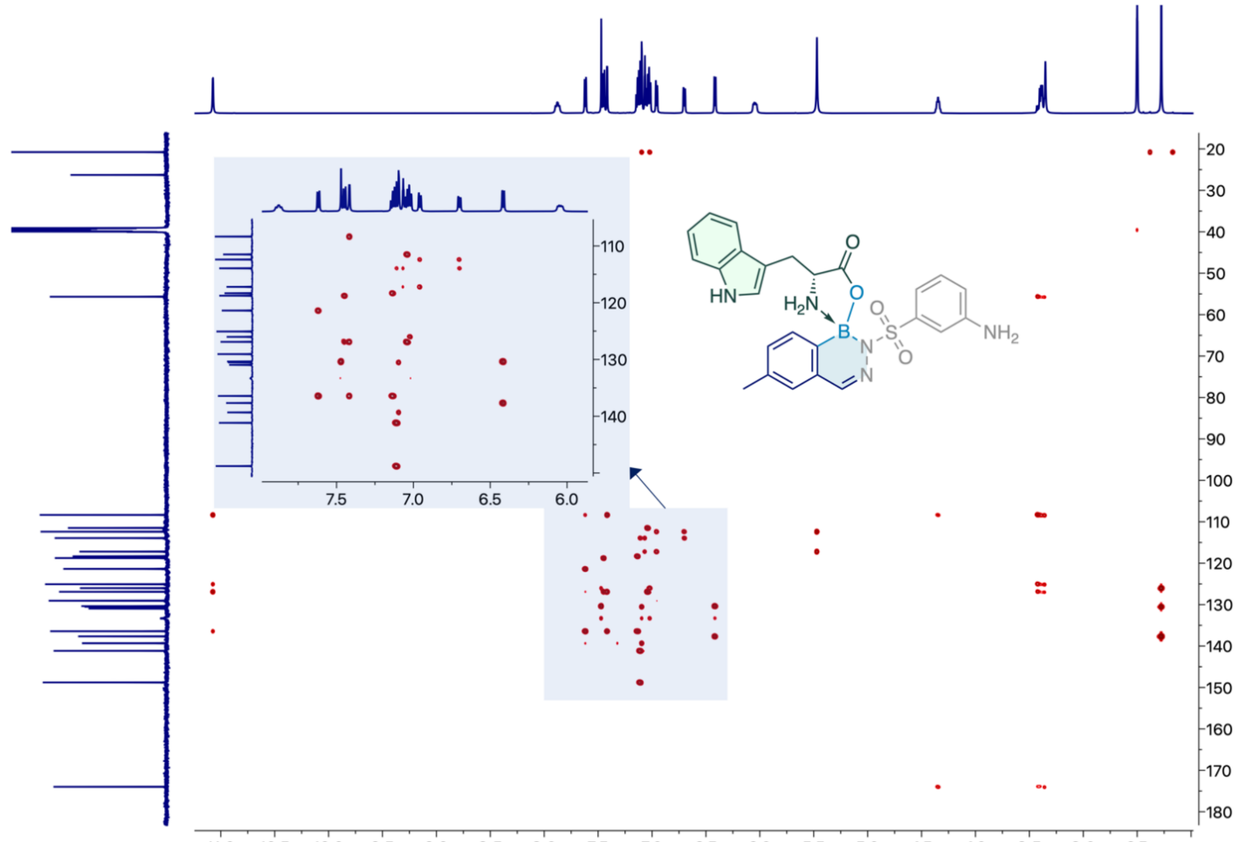

Figure S371. Conjugate 69: ^{1}H-^{13}C gHMBC NMR (DMSO-d_6, 298 K)

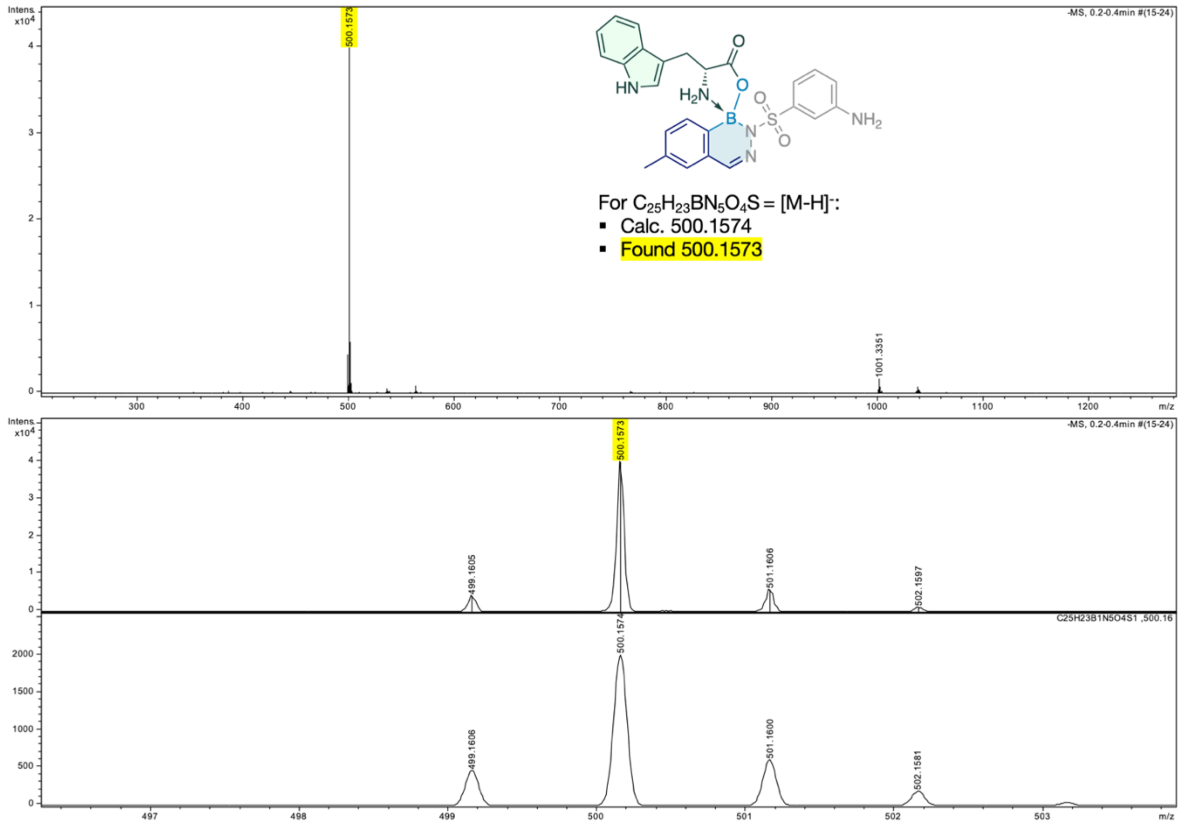

Figure S372. ESI-MS spectrum of Conjugate 69, ($[M-H]^{-}$, ionized in MeOH-H_2O 1:1, negative mode)

Conjugate 70

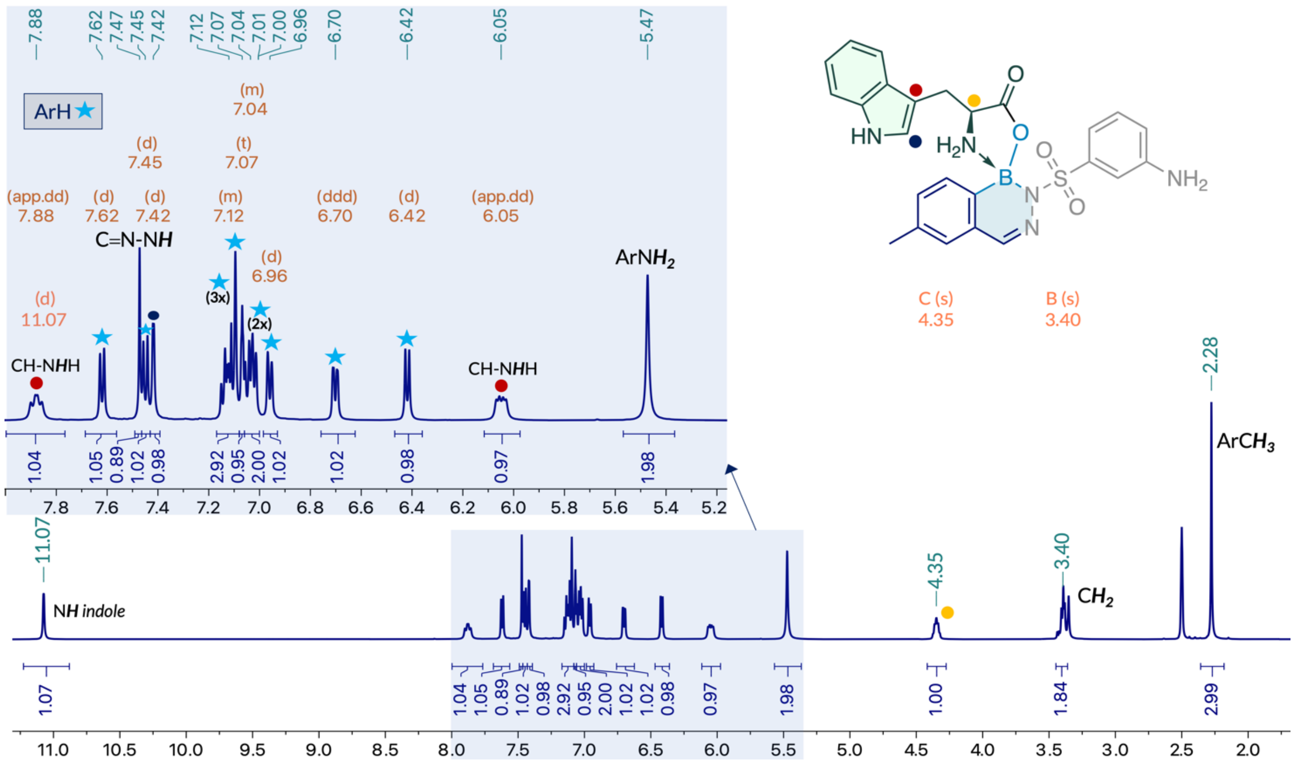

Figure S373. Conjugate 70: ^{1}H NMR (500 MHz, DMSO-d_6, 298 K)

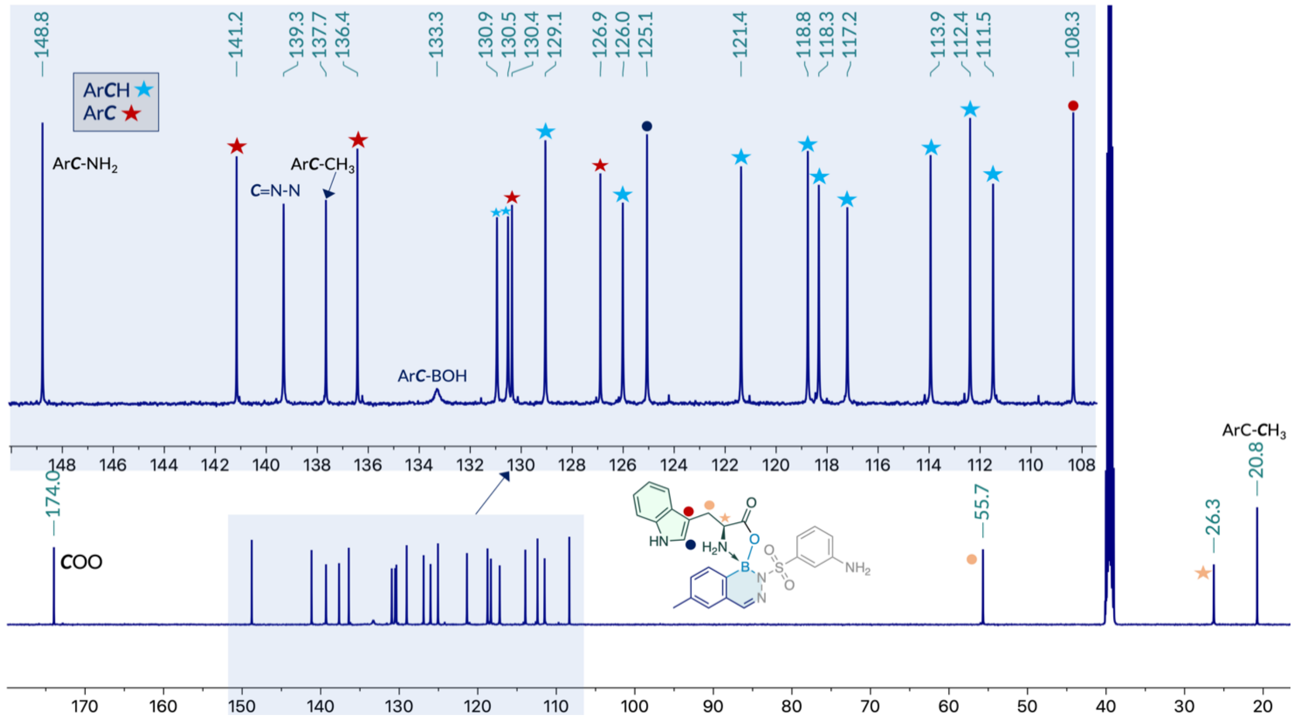

Figure S374. Conjugate 70: ^{13}C NMR (126 MHz, DMSO-d_6, 298 K)

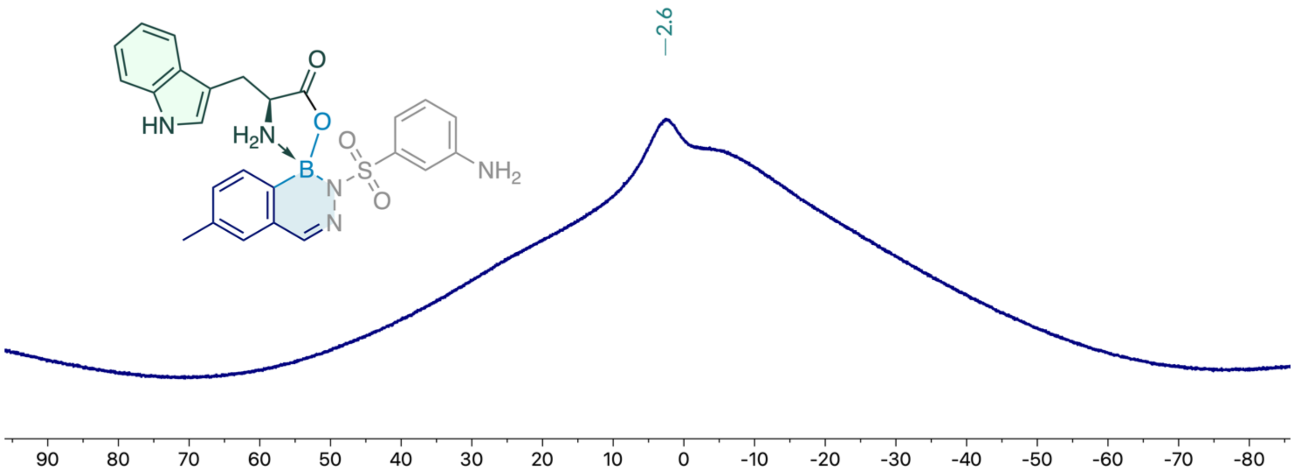

Figure S375. Conjugate 70:^{11}B NMR (160 MHz, DMSO-d_6, 298 K)

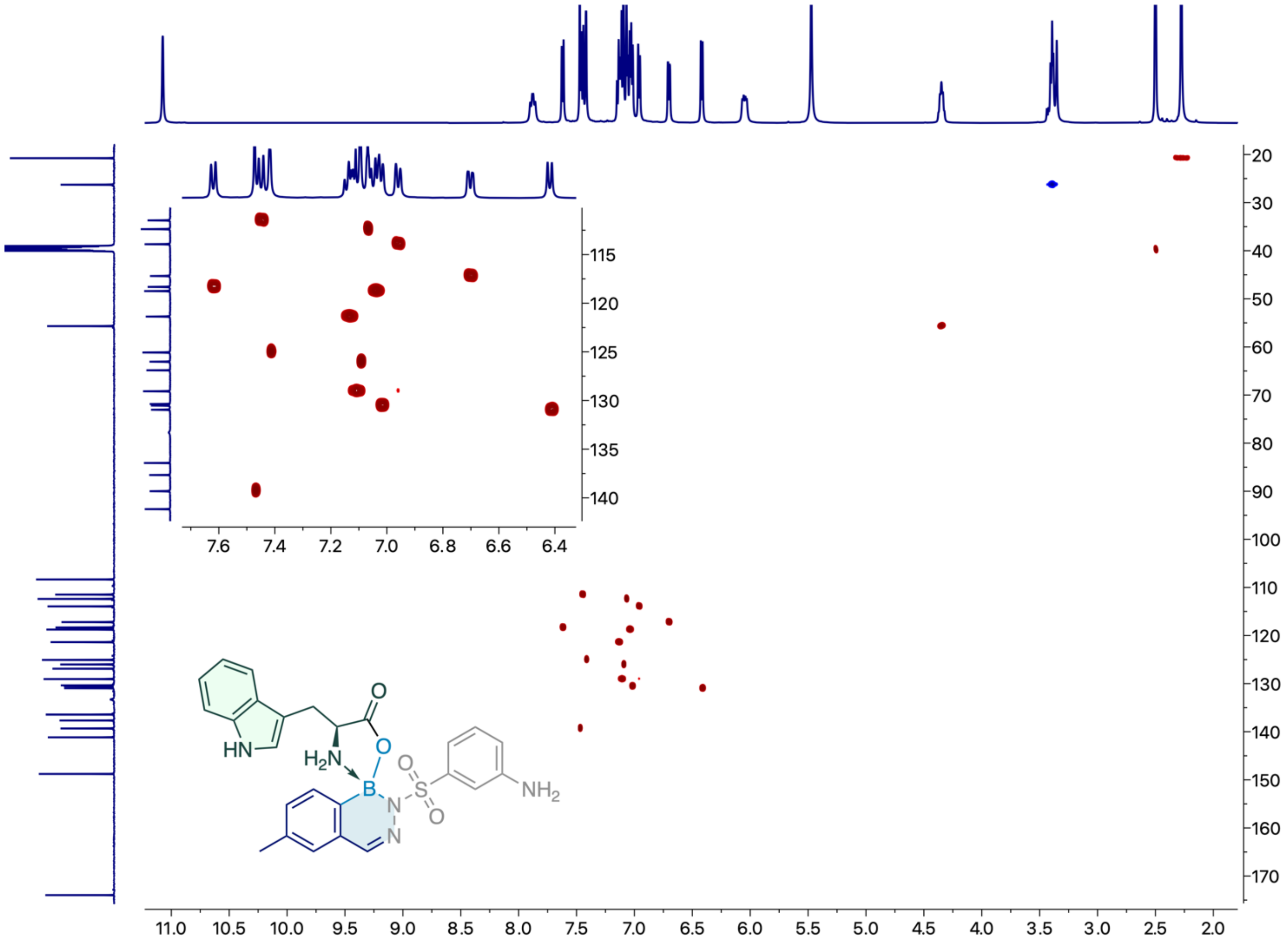

Figure S376. Conjugate 70: ^{1}H-^{13}C gHSQC NMR (DMSO-d_6, 298 K)

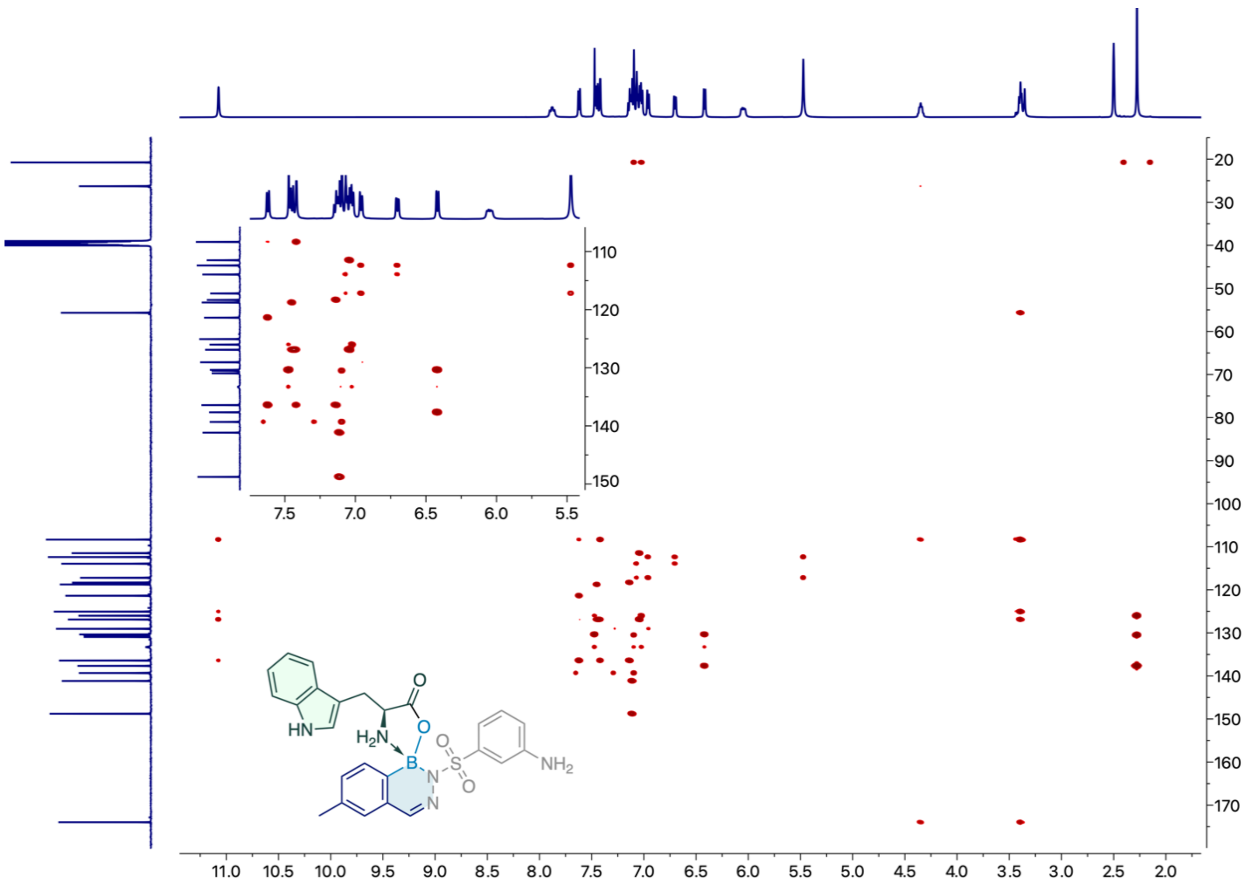

Figure S377. Conjugate 70: ^{1}H-^{13}C gHMBC NMR (DMSO-d_6, 298 K)

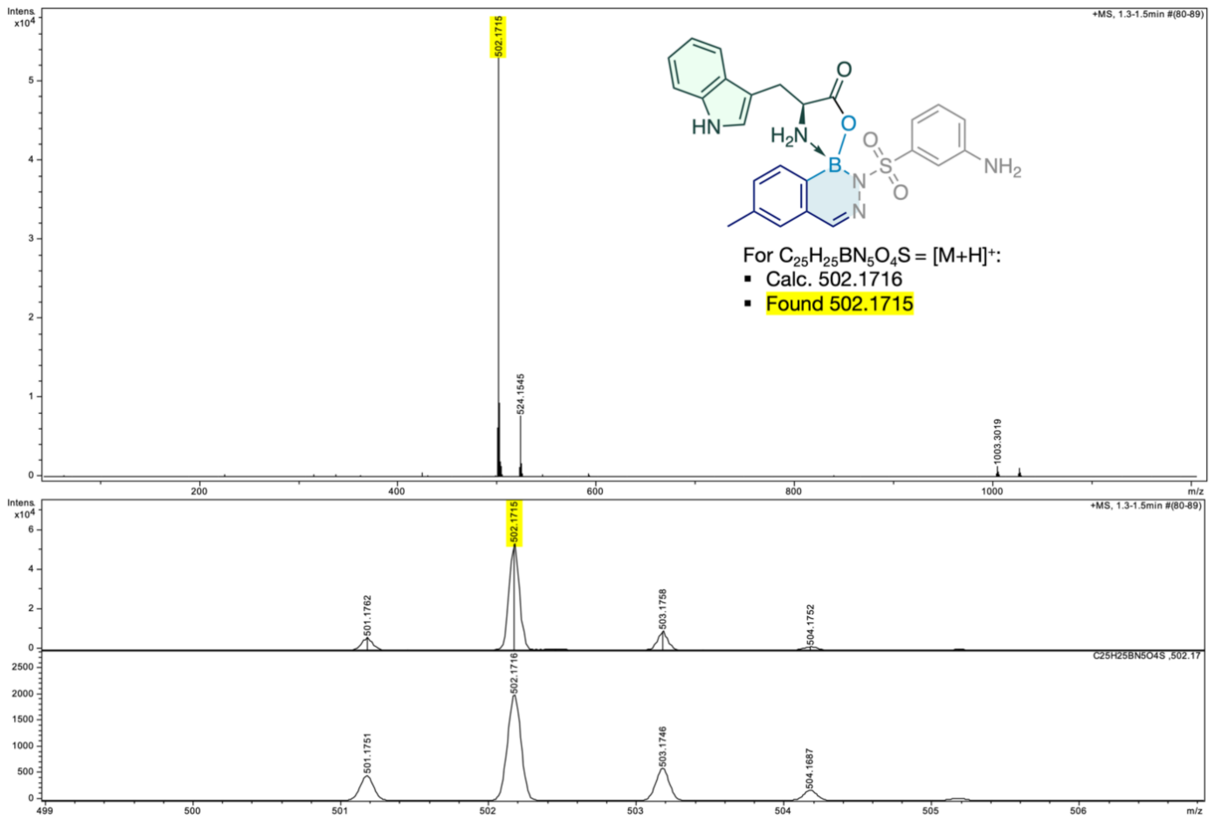

Figure S378. ESI-MS spectrum of Conjugate 70 ([M+H]$^+$, ionized in MeOH, positive mode).

Conjugates Diazaborine-Aminoacid-Phosphonium salt

The conjugates diazaborine-aminoacid-phosphonium (conjugates 71 to 82) were obtained as pure mixtures of two stereoisomers in ratios which depended on the solvent (in which they synthesis was conducted and also the solvent in which they are analyzed) due to the intramolecular interactions imposed by the presence of the triarylphosphonium moiety **(Figure S379)**.

However, the purity of such compounds was confirmed by the presence of one signal in their ^{31}P NMR spectra and ESI-MS spectra.

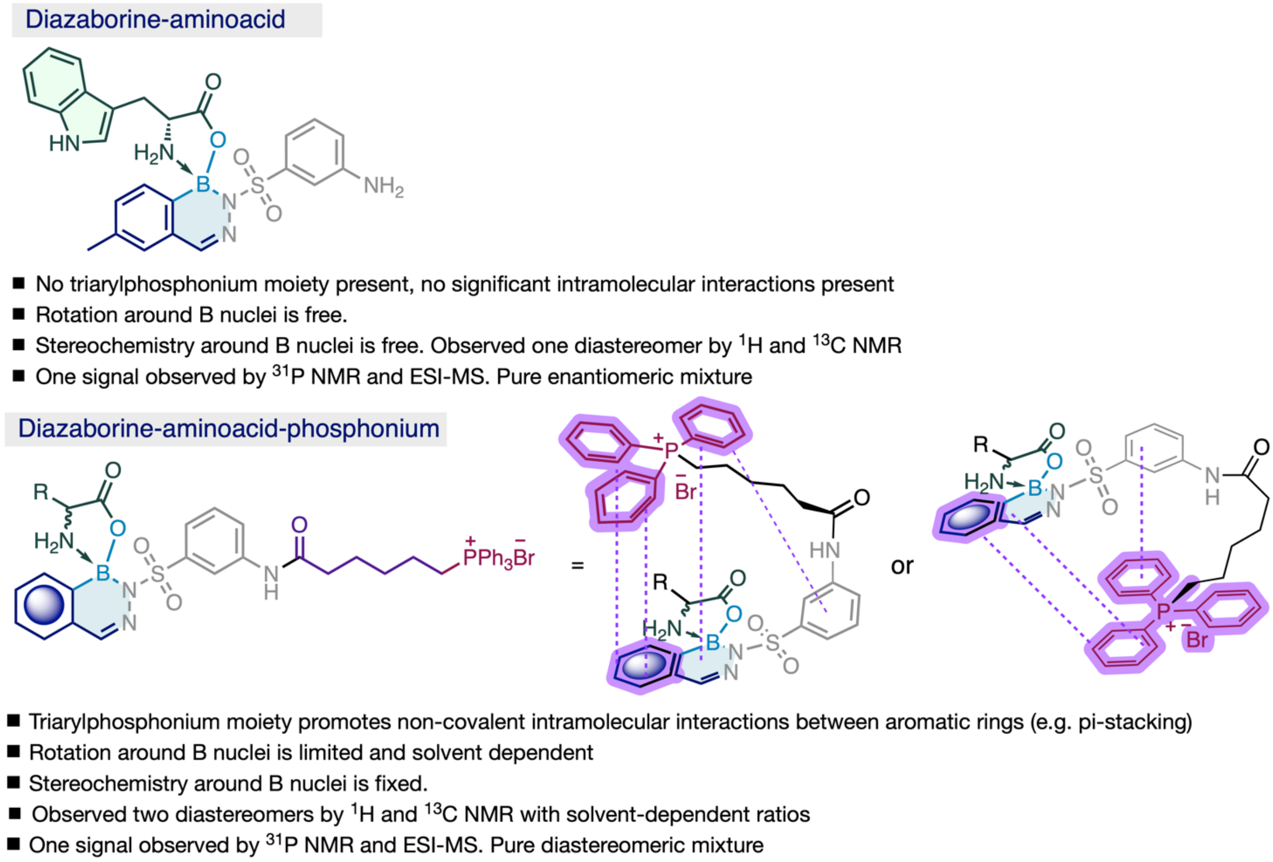

Figure S379. Rationale for the stereoisomeric mixtures observed for the Diazaborine-aminoacid-phosphonium salt conjugates in comparison to the enantiomeric mixtures obtained for the diazaborine-aminoacid conjugates

The ^{1}H, ^{13}C, ^{11}B NMR, NMR, 2D NMR are identical for every pair of conjugates **(Figure S380)** and only one spectra of each type is provided in this section for each couple.

^{31}P and ESI-MS is provided for every conjugate stereomeric mixture to corroborate its purity.

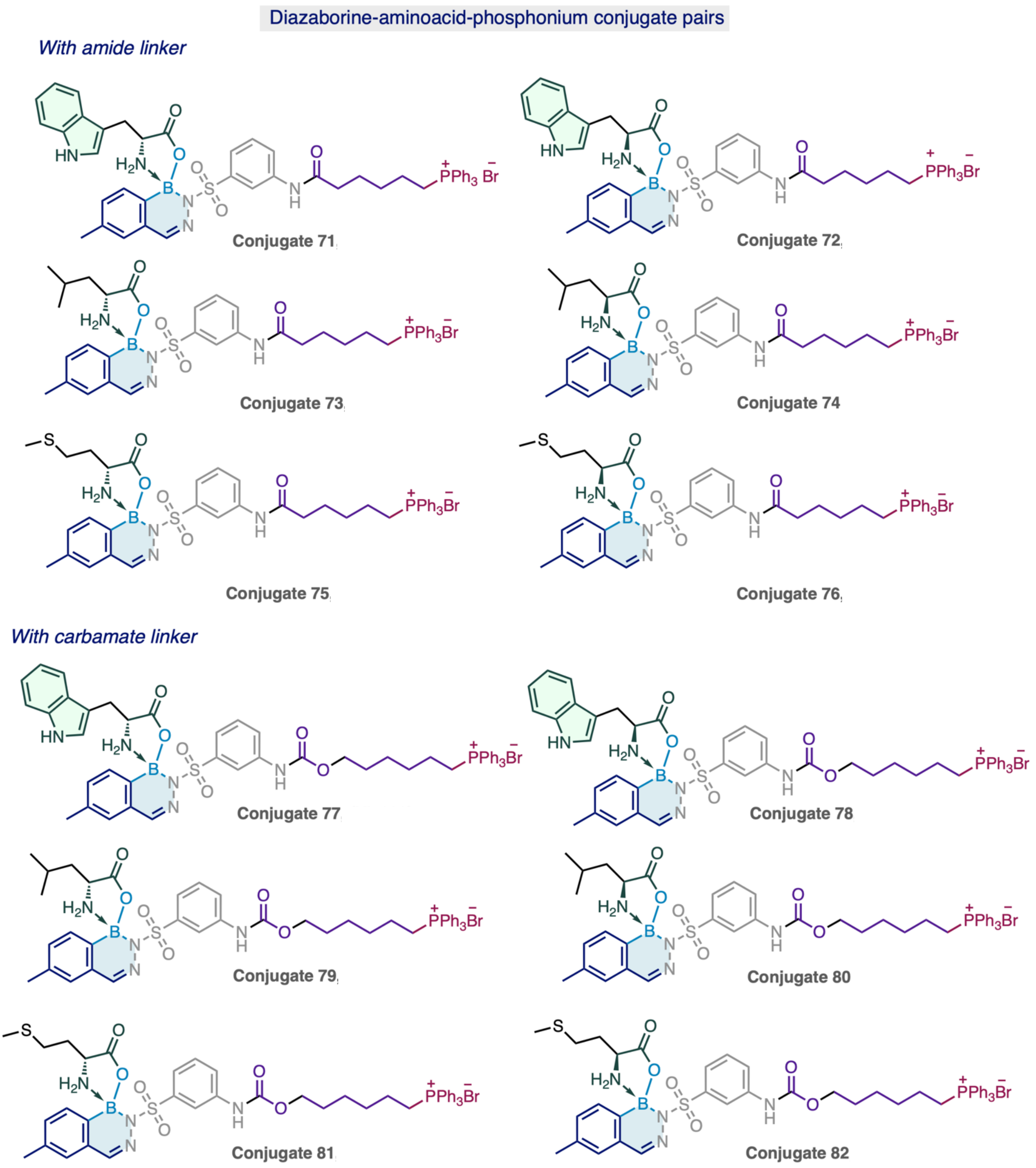

Figure S380. Conjugate pairs diazaborine-aminoacid-phosphonium salt comprised in this study

Conjugate diazaborines 71 and 72

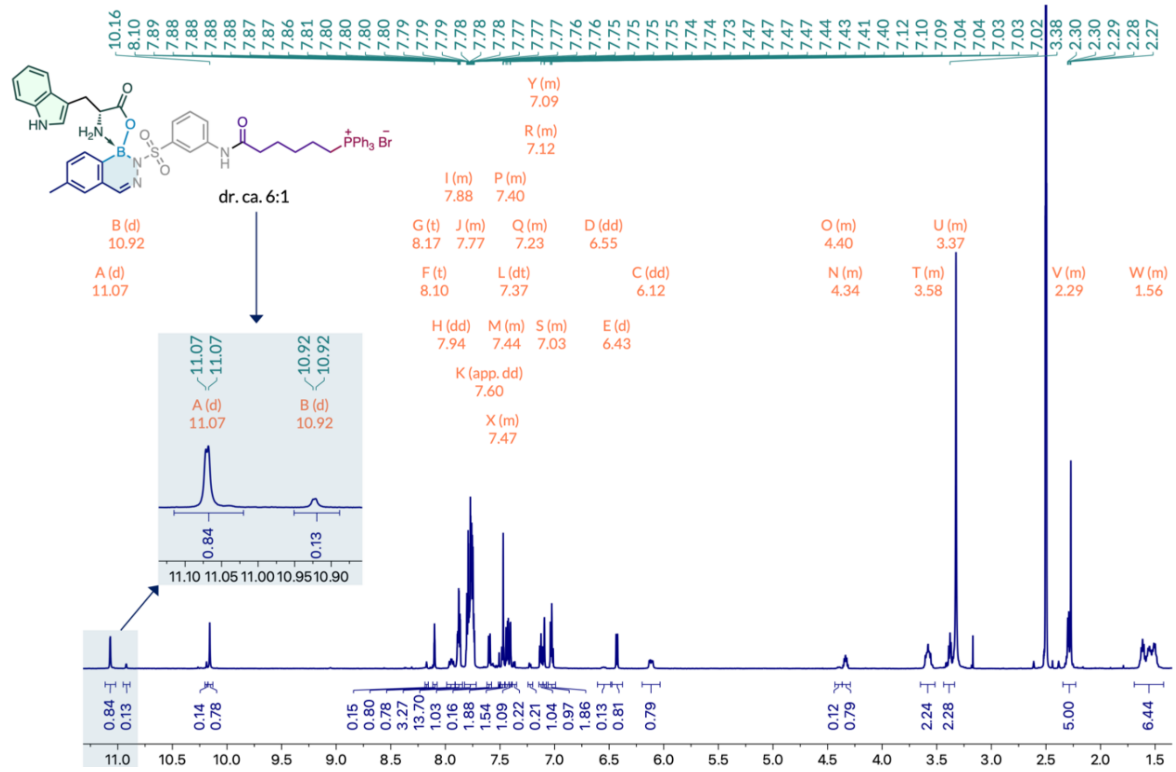

Figure S381. Conjugate 71 and 72 synthesized in EtOH:^{1}H NMR (600 MHz, DMSO-d_6, 298 K).

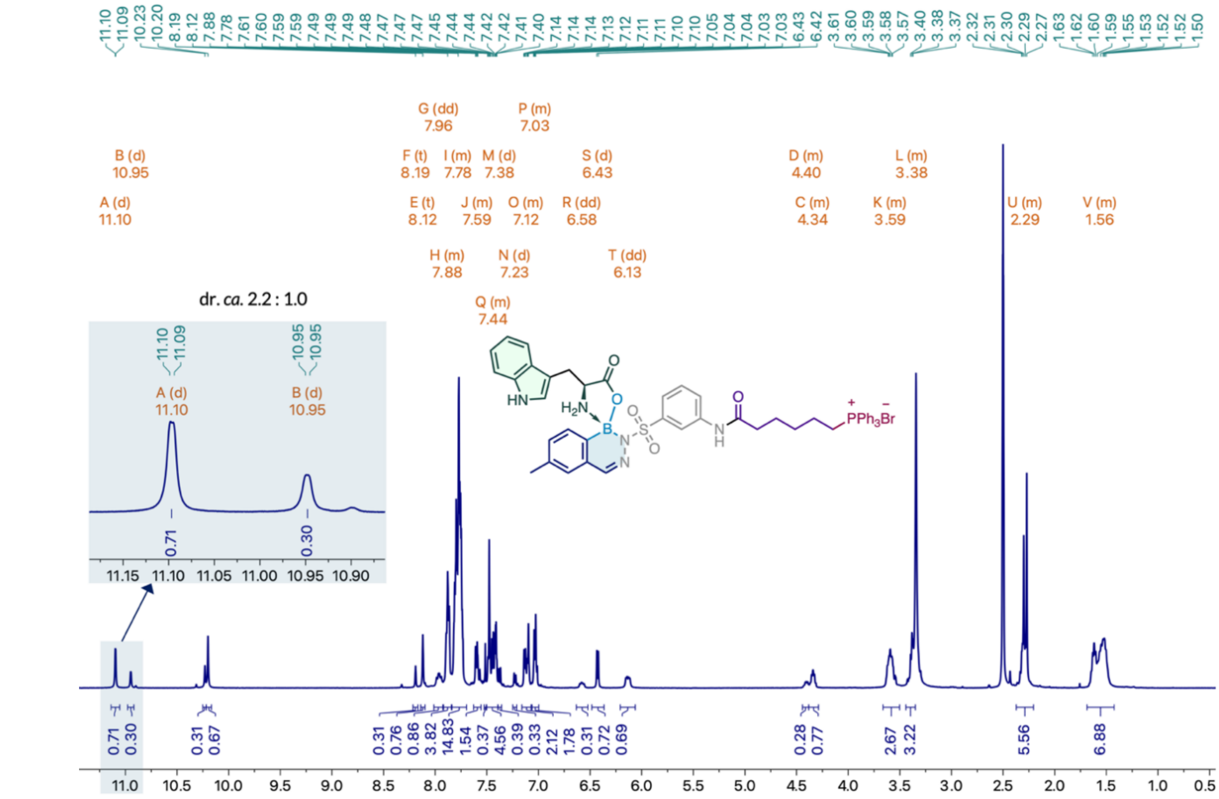

Figure S382. Conjugate 71 and 72 synthesized in MeCN:^{1}H NMR (600 MHz, DMSO-d_6, 298 K).

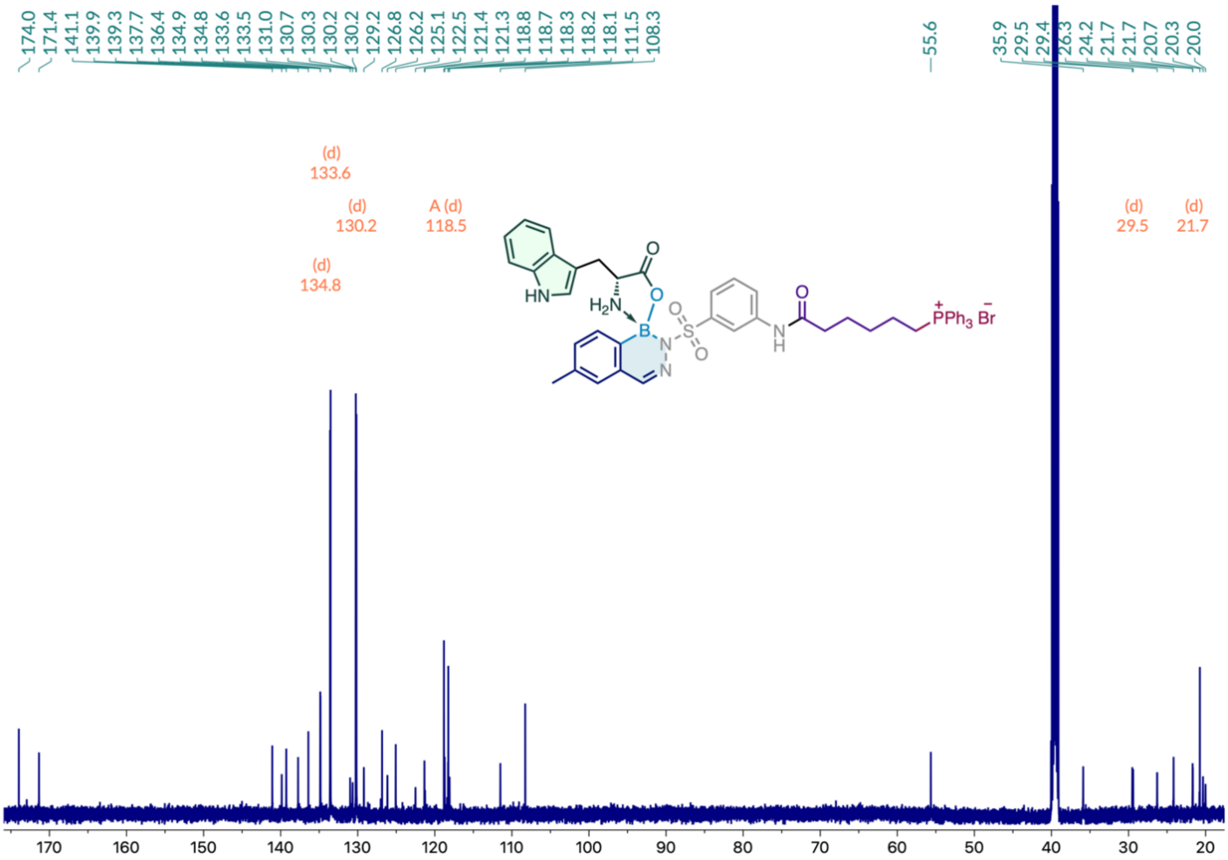

Figure S383. Conjugate 71 and 72:^{13}C NMR (151 MHz, DMSO-d_6, 298 K)

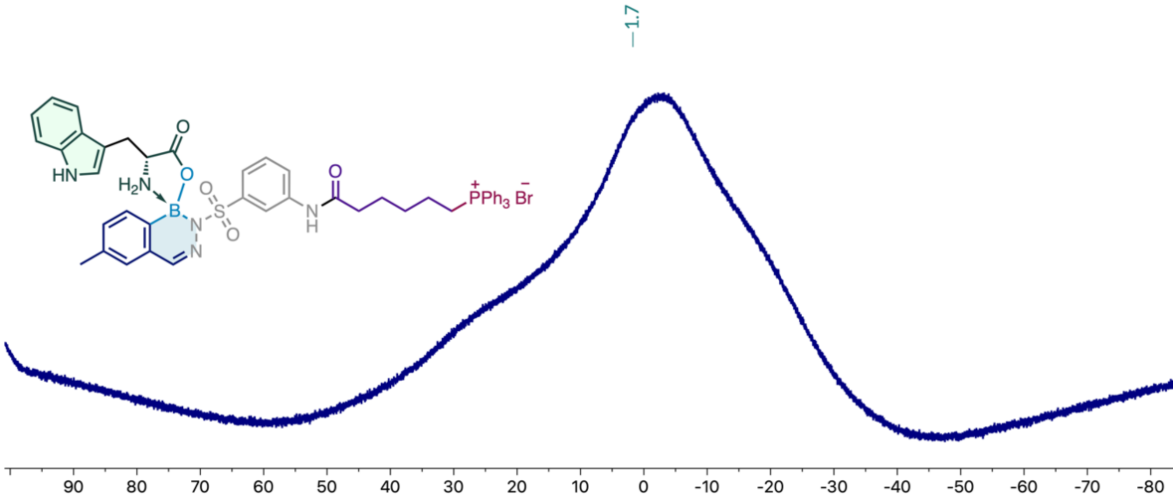

Figure S384. Conjugate 71 and 72:^{11}B NMR (128 MHz, DMSO-d_6, 298 K)

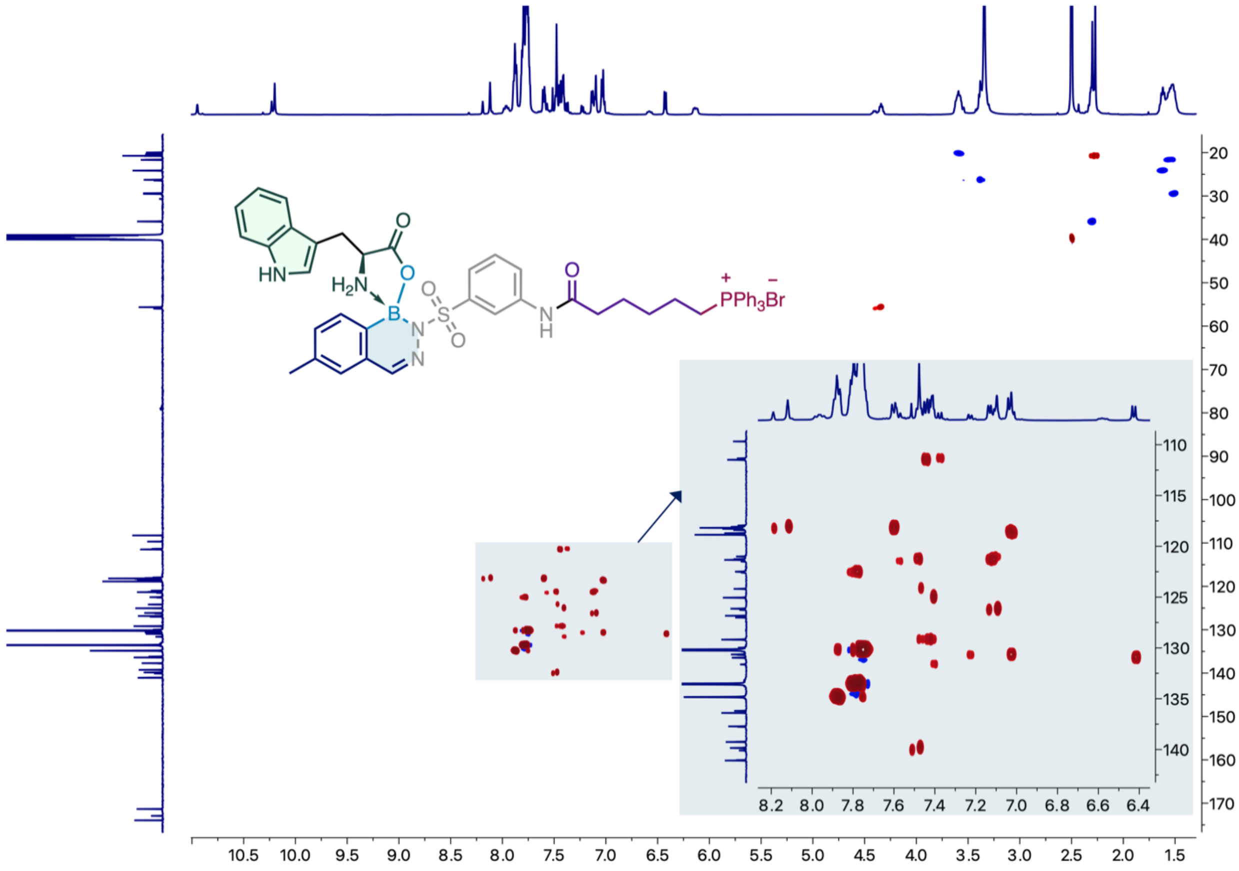

Figure S385. Conjugate 71 and 72: ^{1}H-^{13}C gHSQC NMR (DMSO-d_6, 298 K)

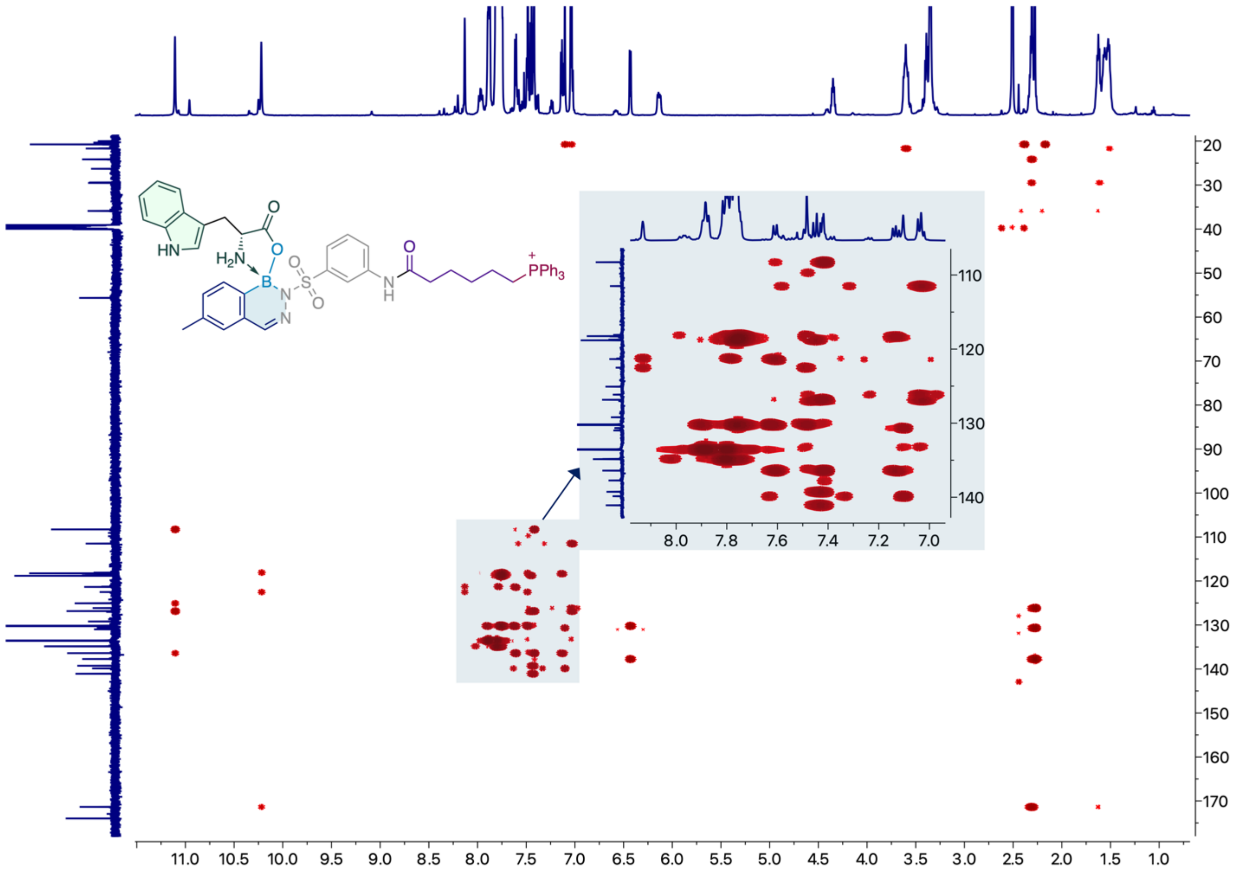

Figure S386. Conjugate 71 and 72: ^{1}H-^{13}C gHMBC NMR (DMSO-d_6, 298 K)

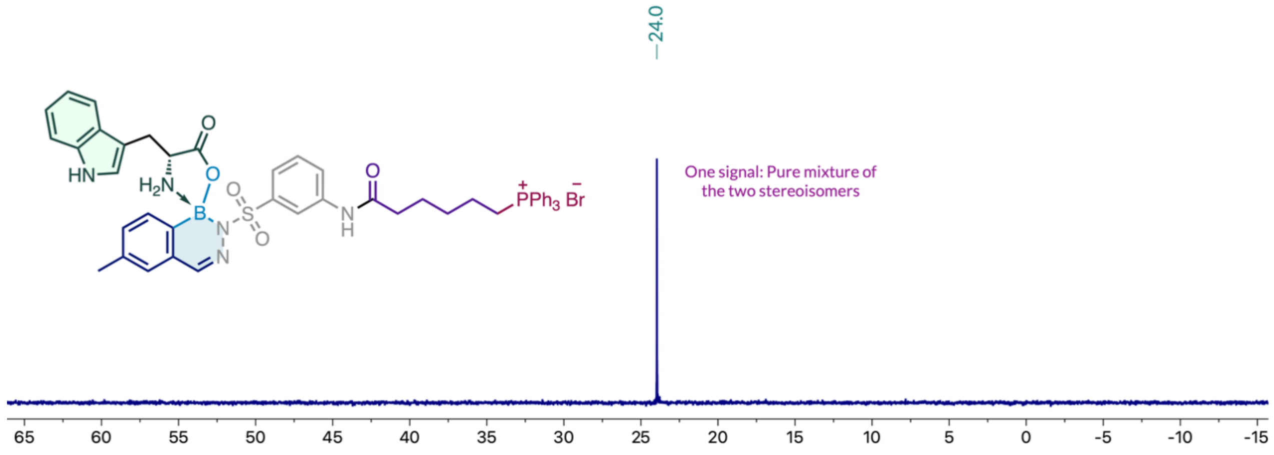

Figure S387. Conjugate 71: $^{31}P\{^{1}H\}$ NMR (243 MHz, DMSO-d_6, 298 K)

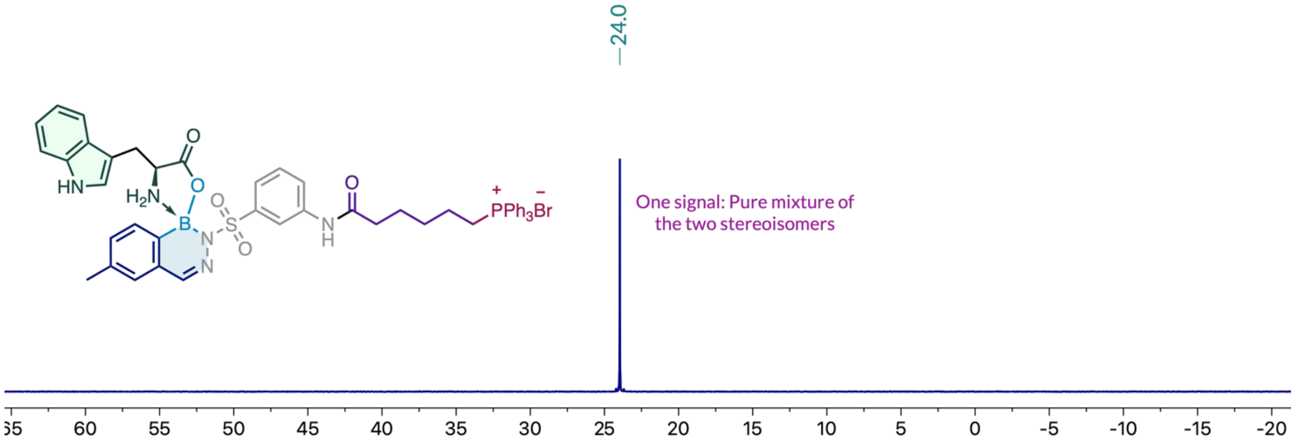

Figure S388. Conjugate 72: $^{31}P\{^{1}H\}$ NMR (243 MHz, DMSO-d_6, 298 K)

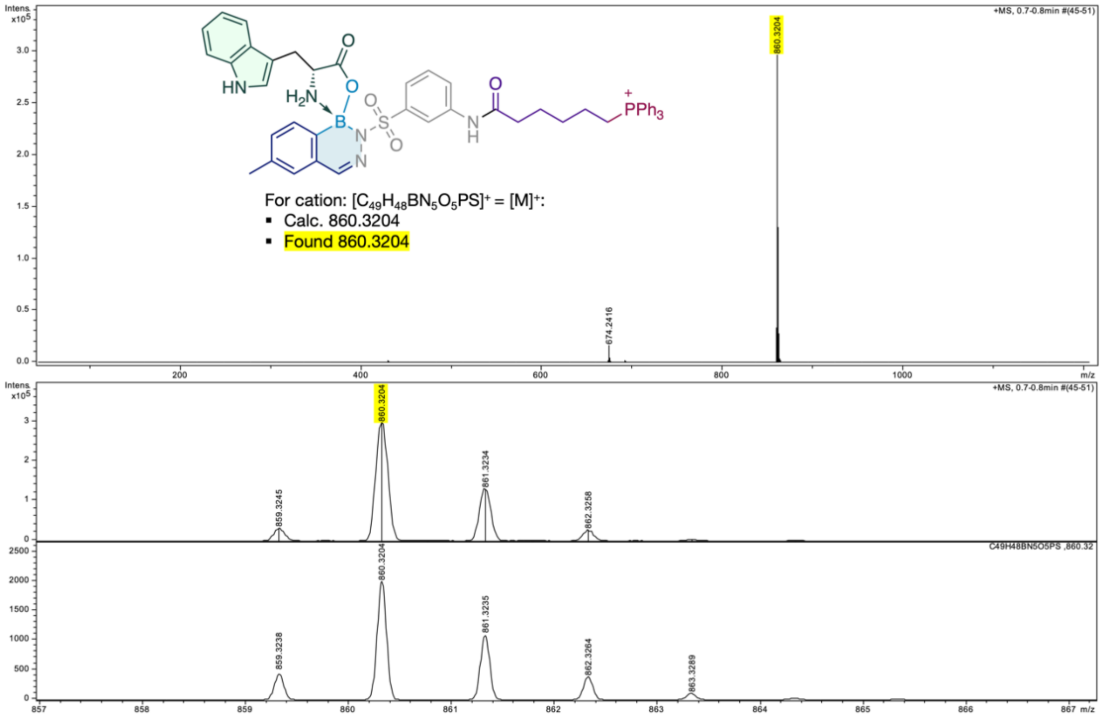

Figure S389. ESI-MS spectrum of Conjugate 71 ([M]$^+$, ionized in CH_3CN-H_2O 4:1, positive mode).

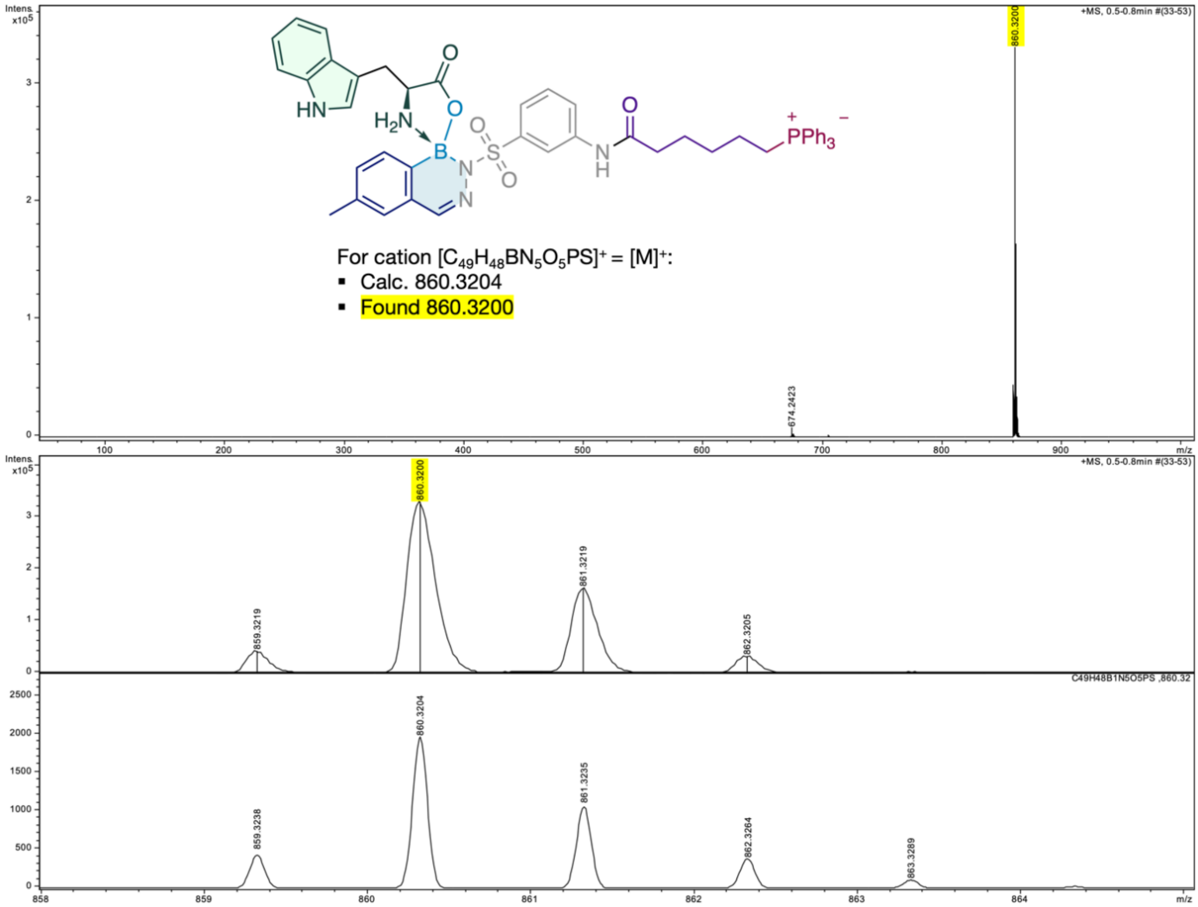

Figure S390. ESI-MS spectrum of Conjugate 72 ([M]$^+$, ionized in CH_3CN-H_2O 4:1, positive mode)

Conjugate diazaborines 73 and 74

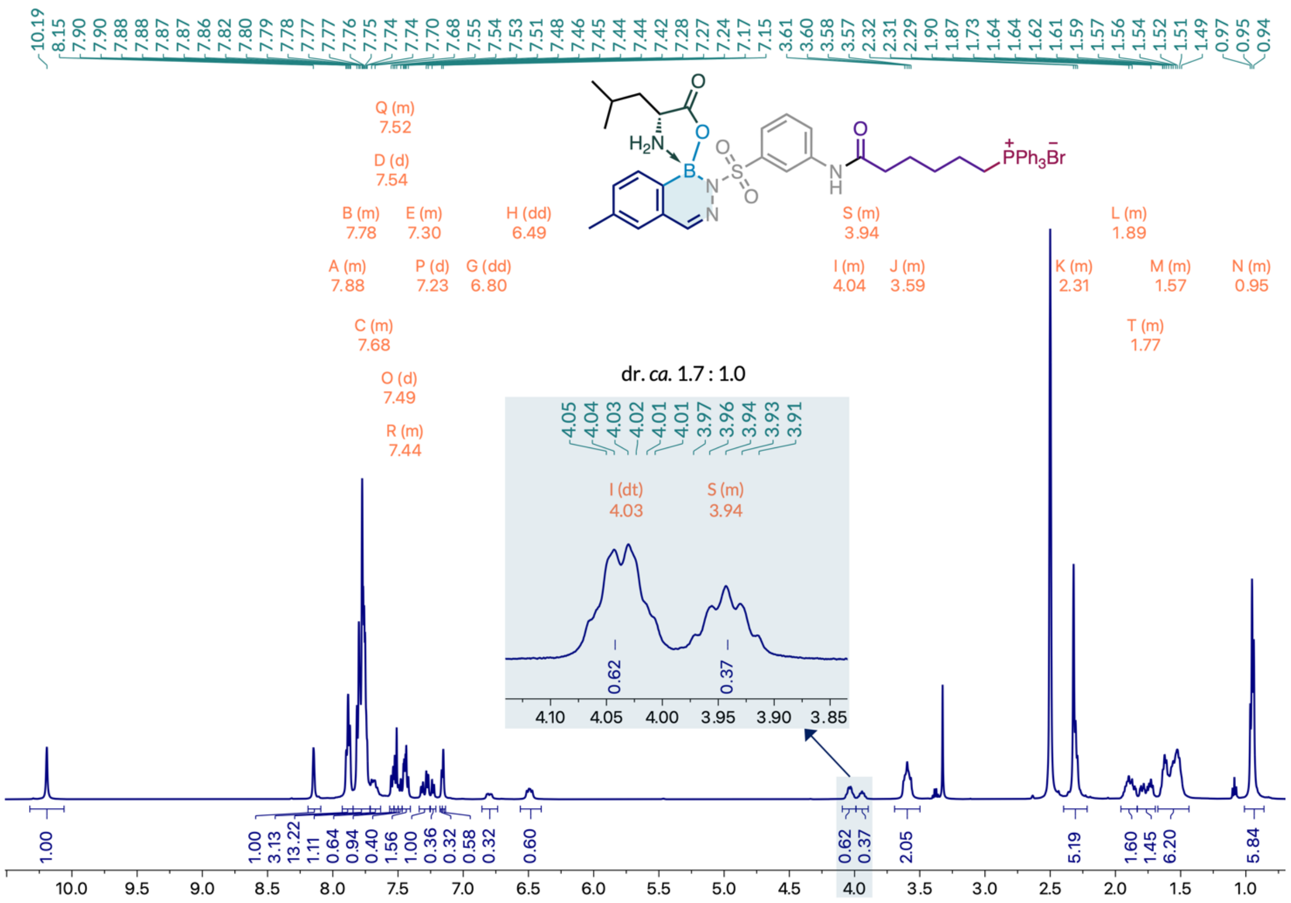

Figure S391. Conjugate 73 and 74: ^{1}H NMR (500 MHz, DMSO-d_6, 298 K)

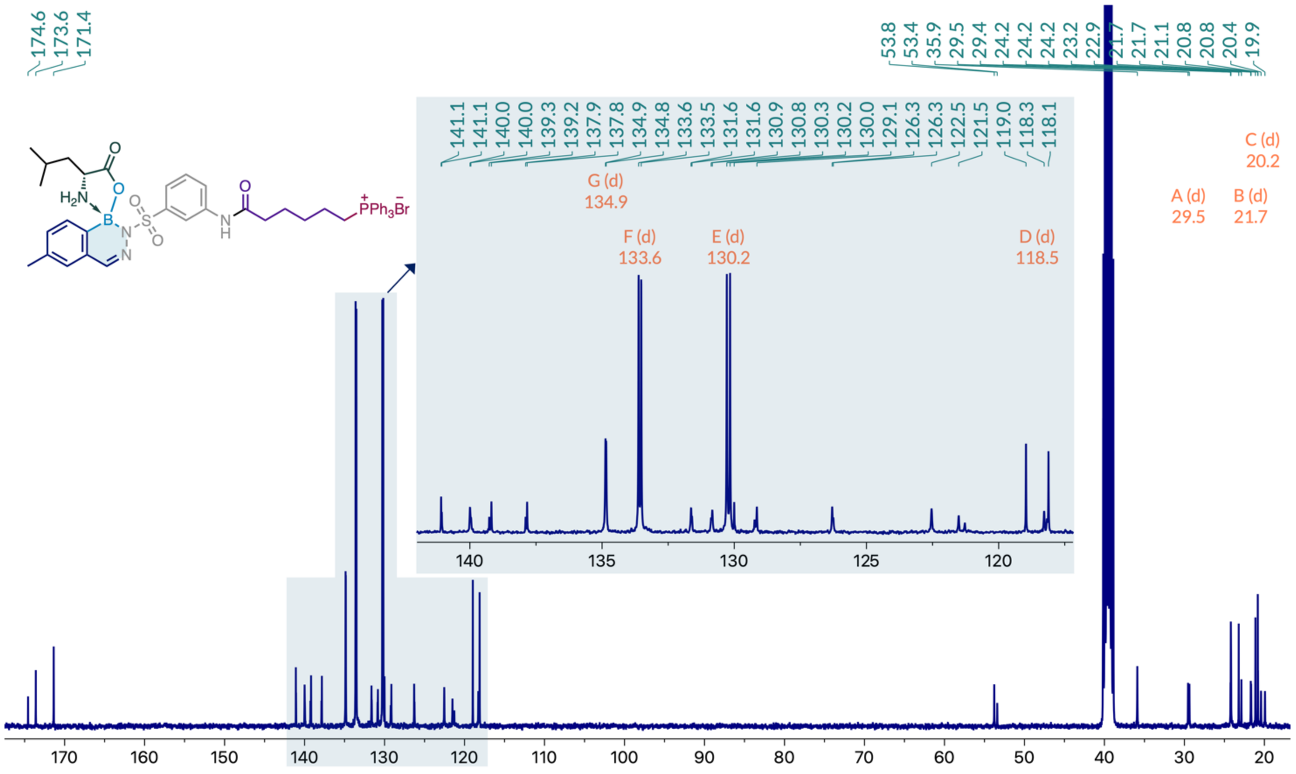

Figure S392. Conjugate 73 and 74: ^{13}C NMR (101 MHz, DMSO-d_6, 298 K)

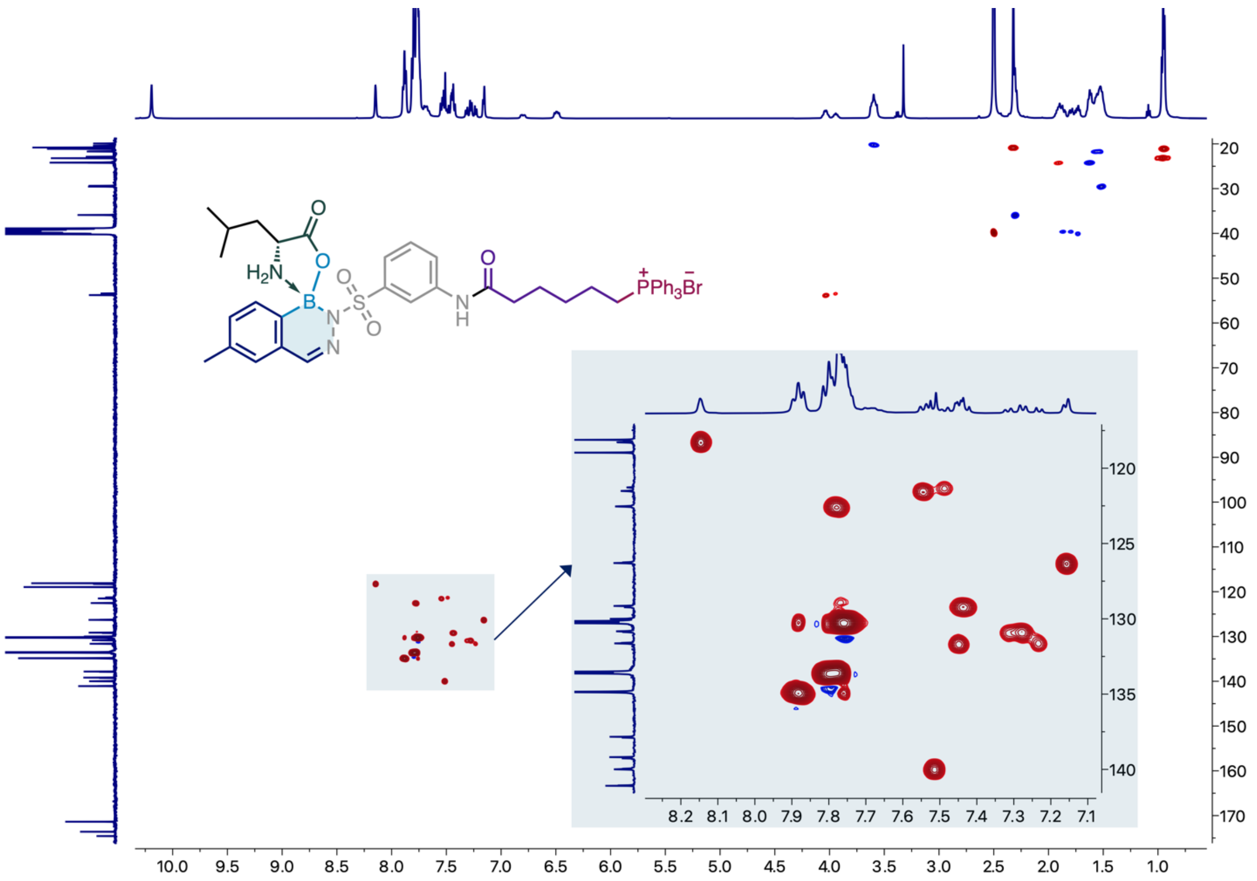

Figure S393. Conjugate 73 and 74: ^{1}H-^{13}C gHSQC NMR (DMSO-d_6, 298 K)

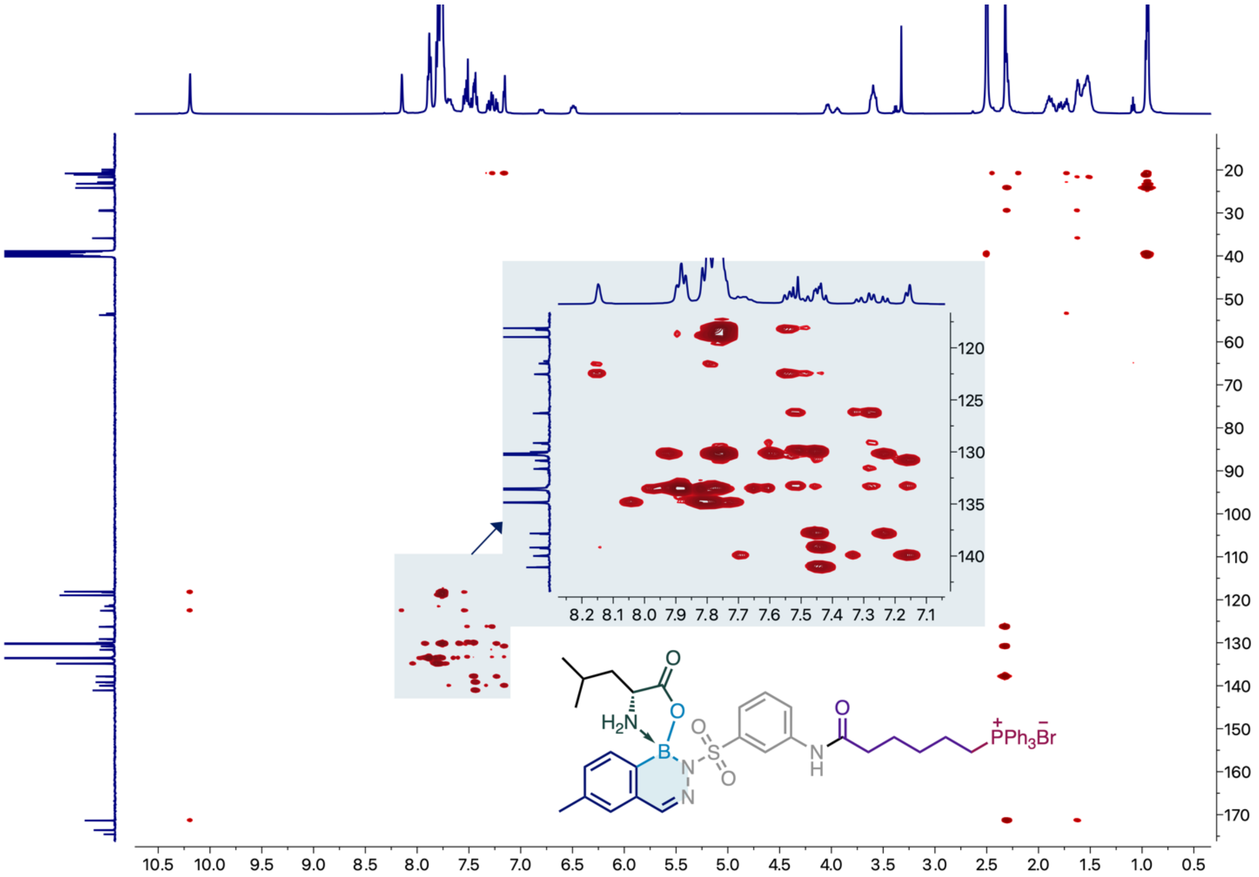

Figure S394. Conjugate 73 and 74: ^{1}H-^{13}C gHMBC NMR (DMSO-d_6, 298 K)

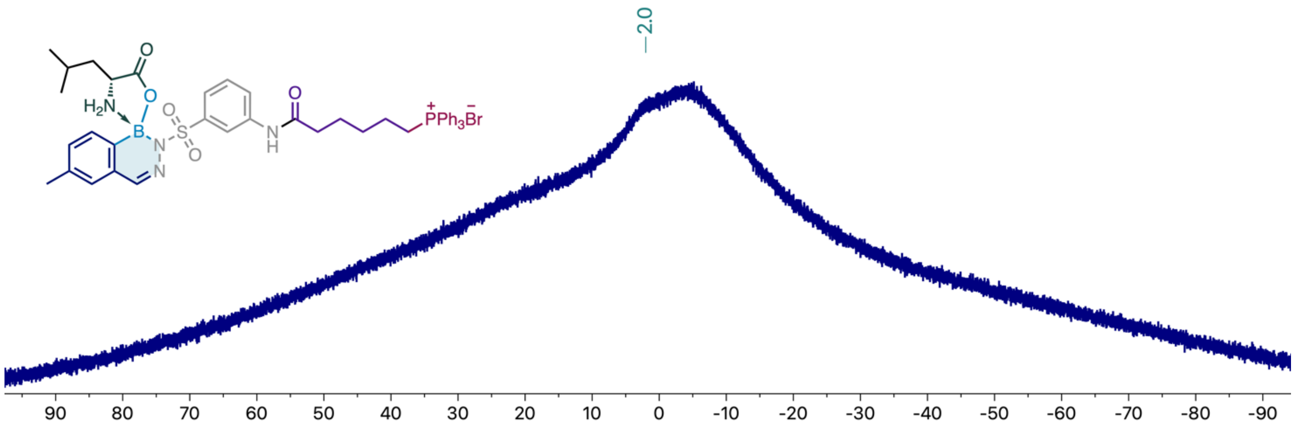

Figure S395. Conjugate 73 and 74:^{11}B NMR (160 MHz, DMSO-d_6, 298 K)

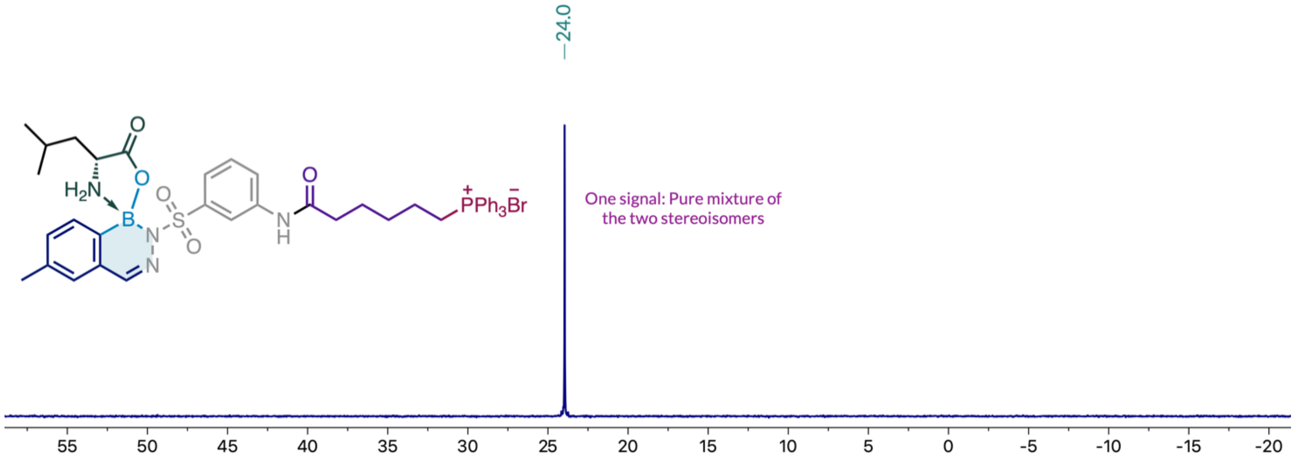

Figure S396. Conjugate 73:^{31}P{^{1}H} NMR (202 MHz, DMSO-d_6, 298 K)

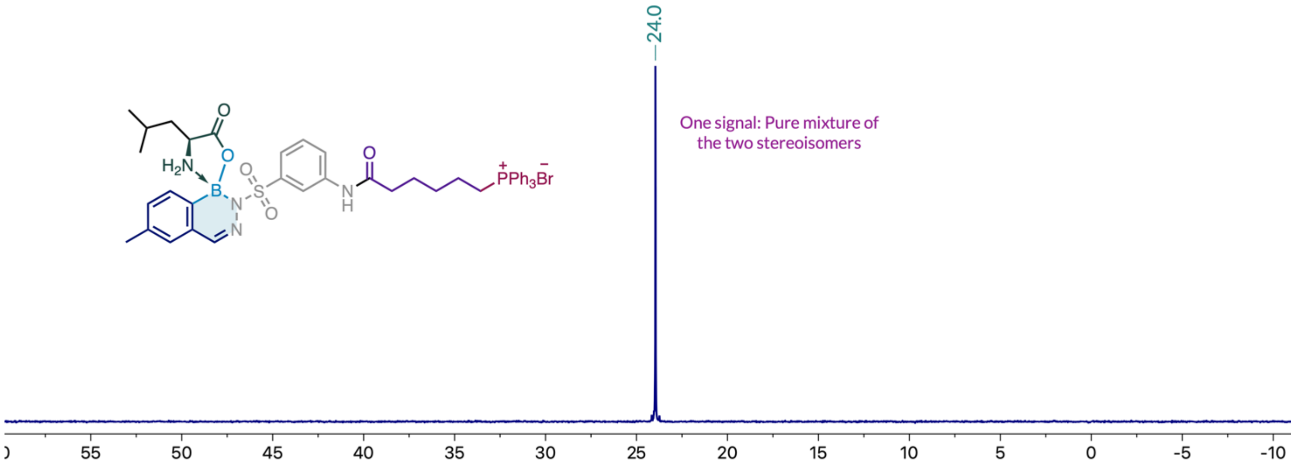

Figure S397. Conjugate 74:^{31}P{^{1}H} NMR (202 MHz, DMSO-d_6, 298 K)

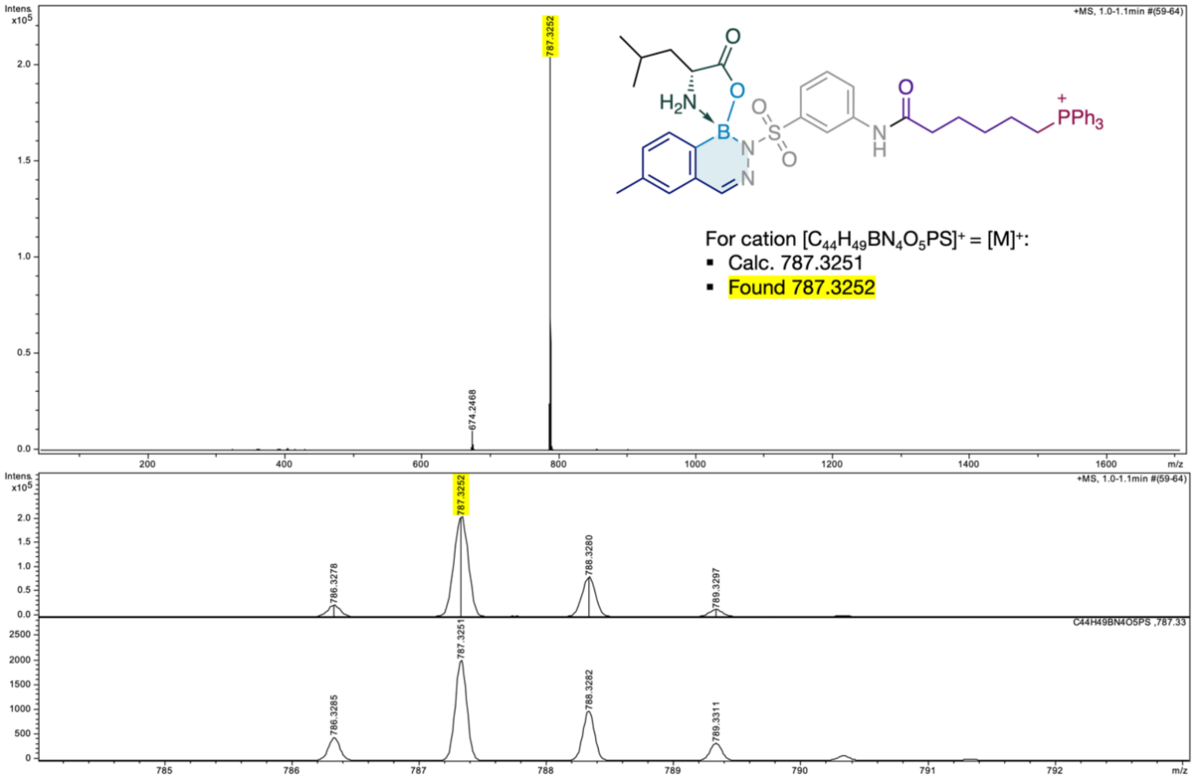

Figure S398. ESI-MS spectrum of Conjugate 73 ($[M]^+$, ionized in CH_3CN-H_2O 4:1, positive mode)

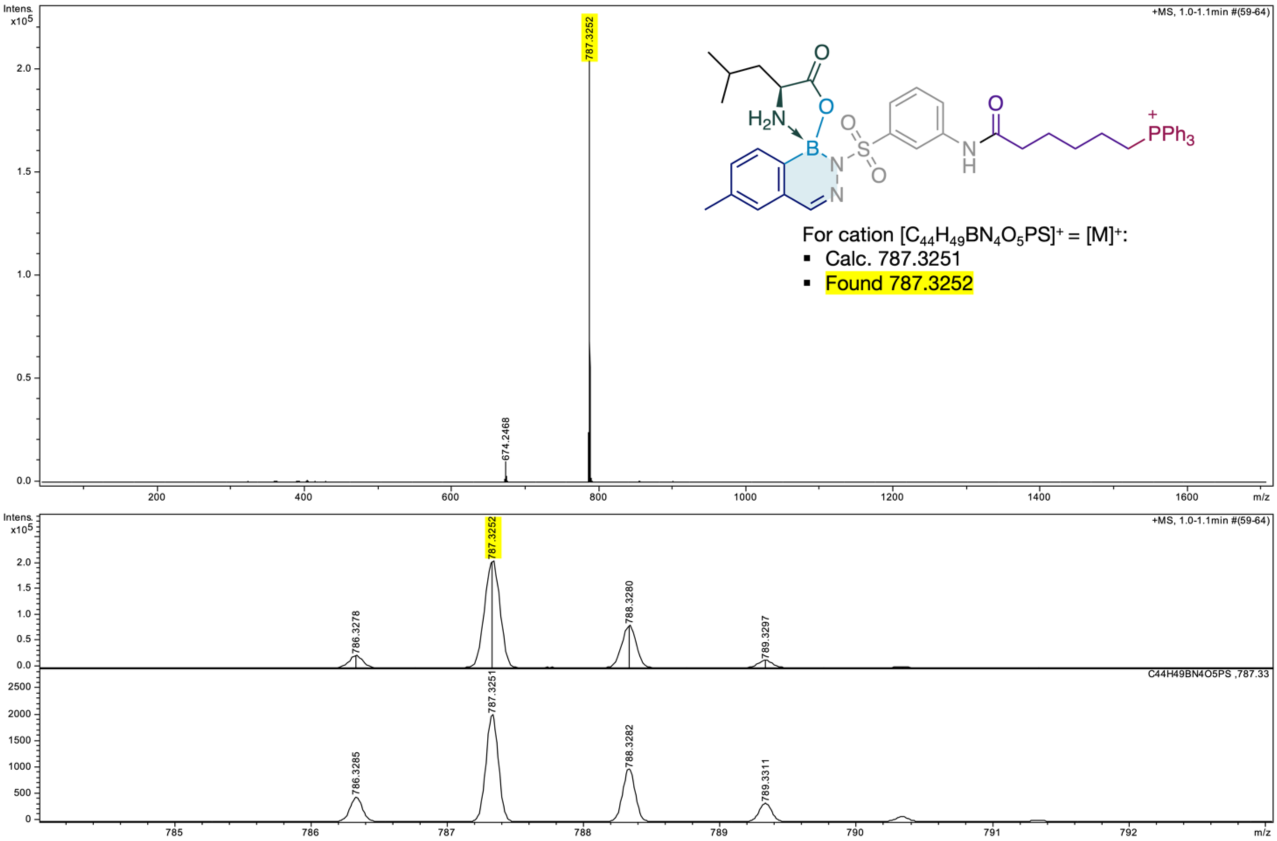

Figure S399. ESI-MS spectrum of Conjugate 74 ($[M]^+$, ionized in CH_3CN-H_2O 4:1, positive mode)

Conjugate diazaborines 75 and 76

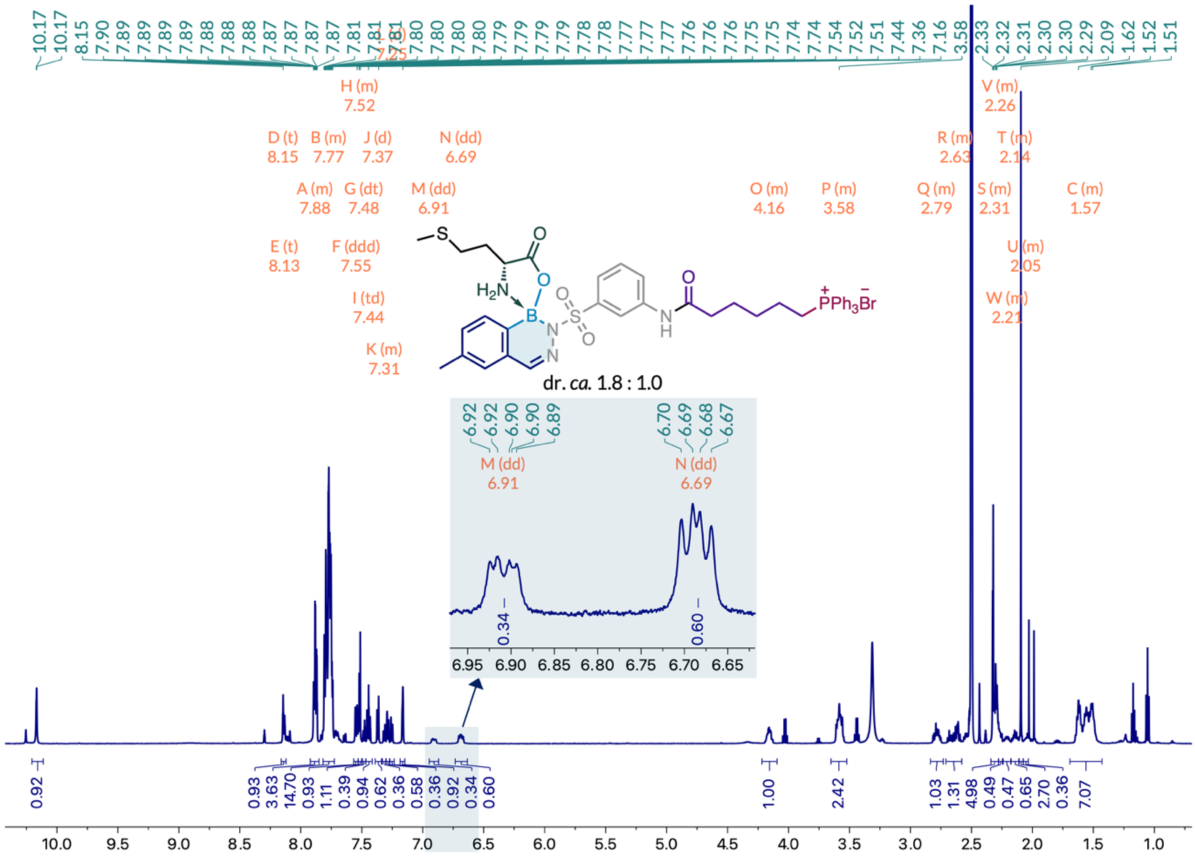

Figure S400. Conjugate 75 and 76:^{1}H NMR (600 MHz, DMSO-d_6, 298 K)

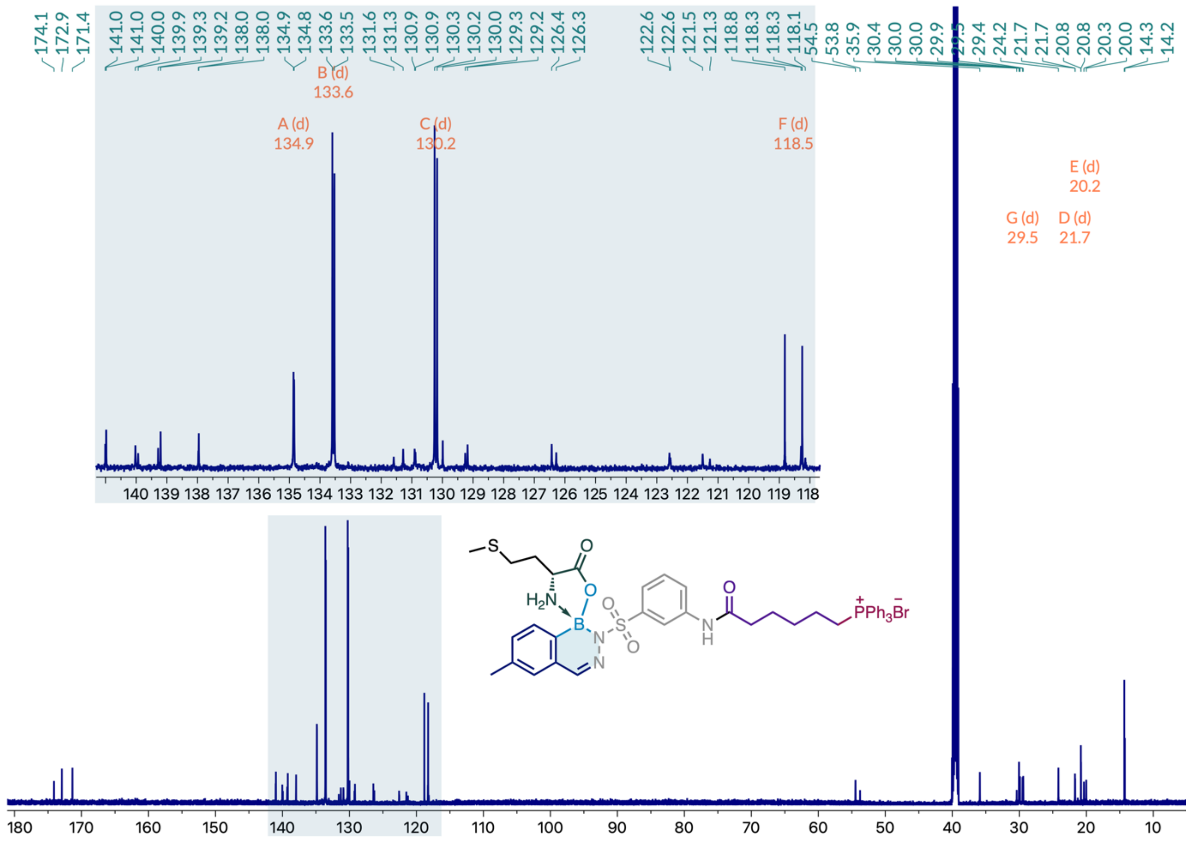

Figure S401. Conjugate 75 and 76:^{13}C NMR (151 MHz, DMSO-d_6, 298 K)

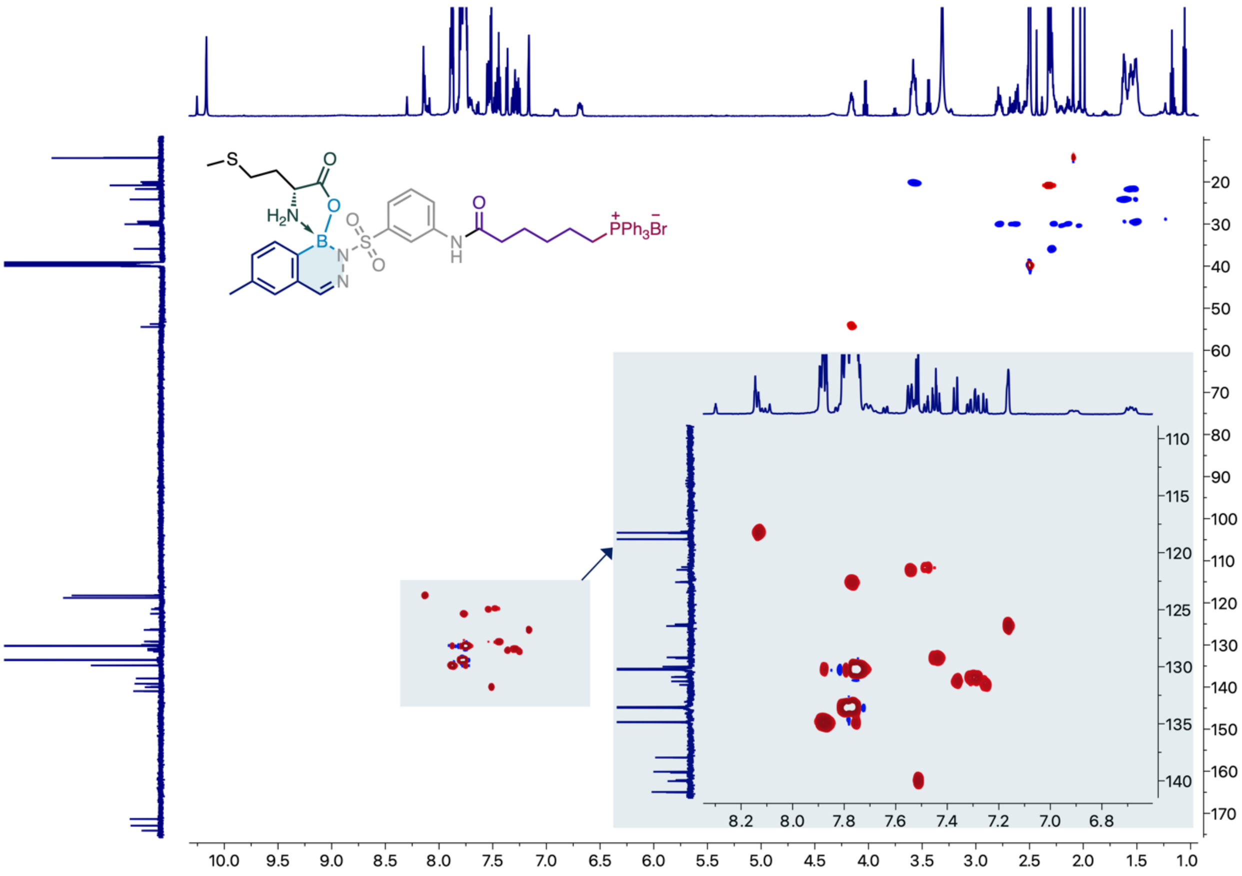

Figure S402. Conjugate 75 and 76: ^{1}H-^{13}C gHSQC NMR (DMSO-d_6, 298 K)

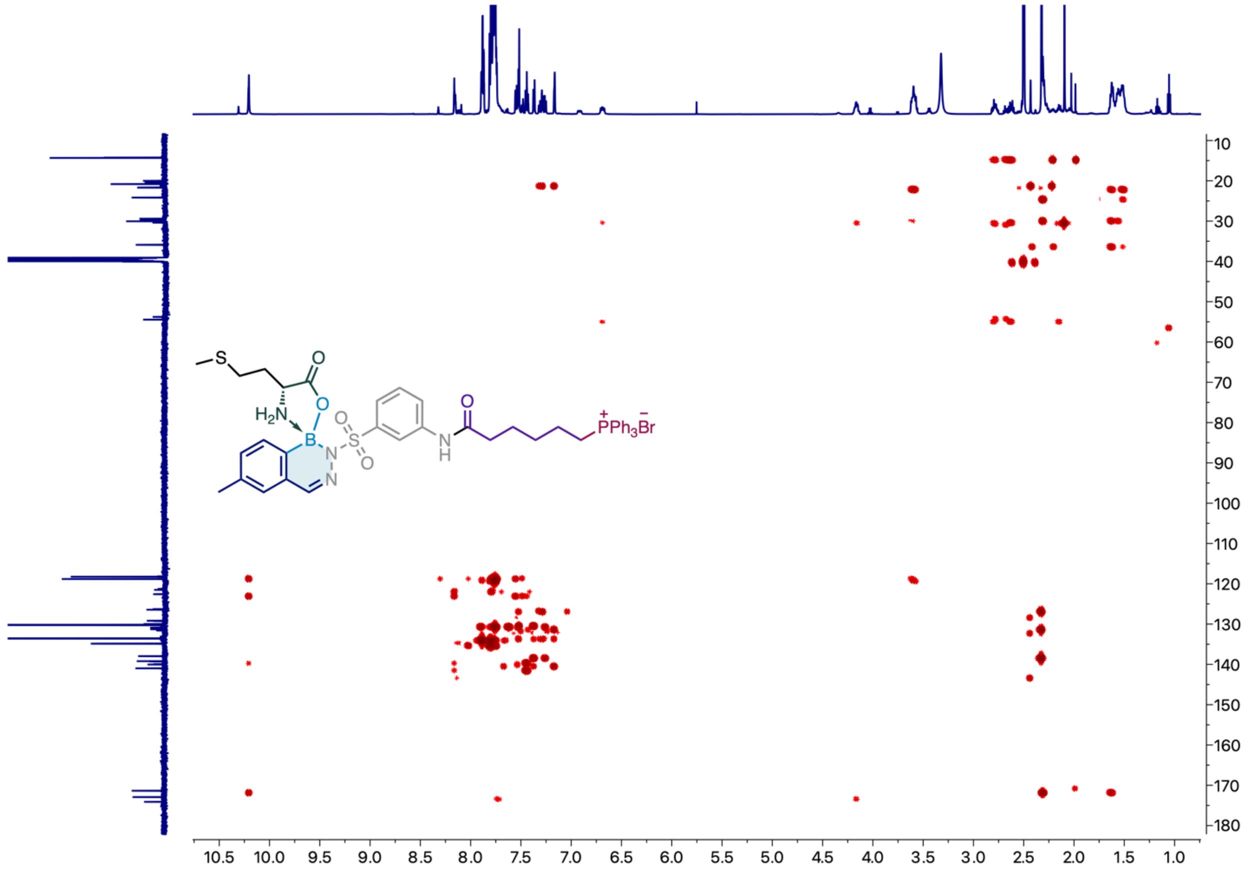

Figure S403. Conjugate 75 and 76: ^{1}H-^{13}C gHMBC NMR (DMSO-d_6, 298 K)

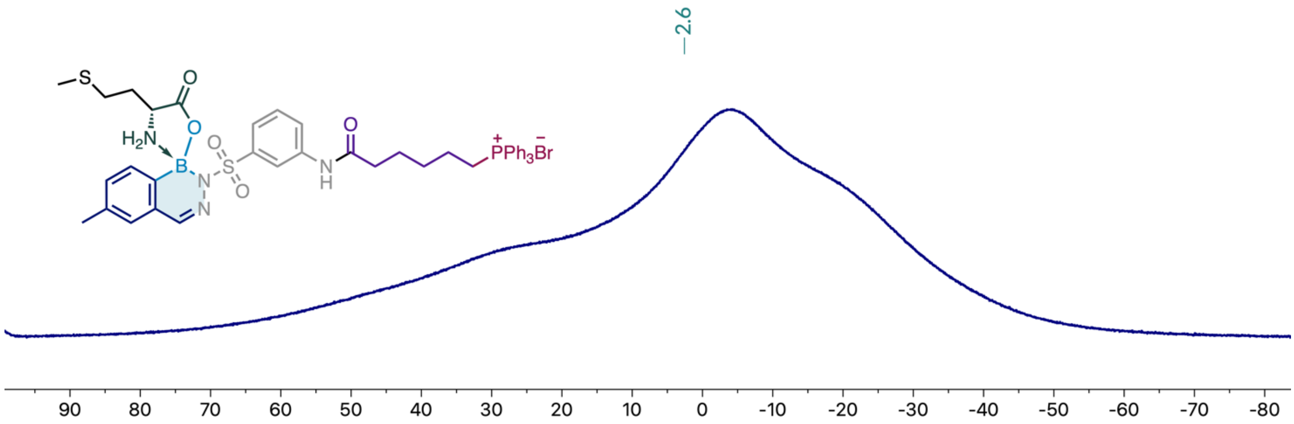

Figure S404. Conjugate 75 and 76: ^{11}B NMR (128 MHz, DMSO-d_6, 298 K)

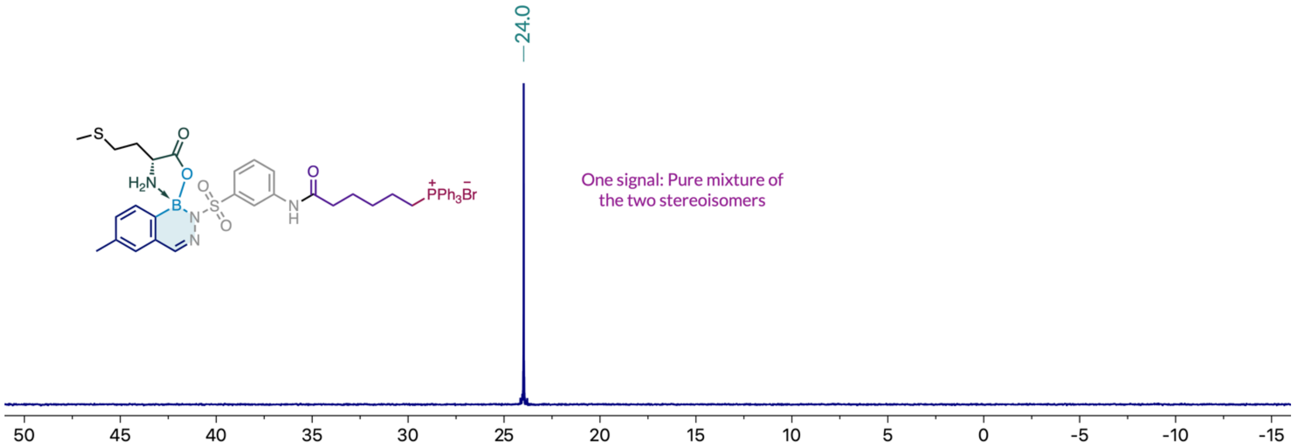

Figure S405. Conjugate 75: $^{31}P\{^1H\}$ NMR (243 MHz, DMSO-d_6, 298 K)

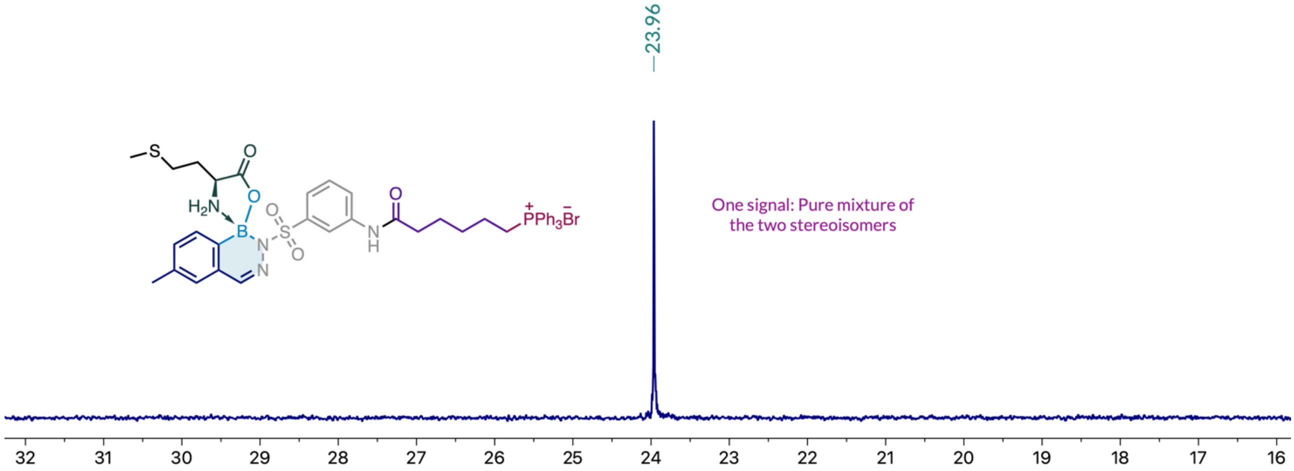

Figure S406. Conjugate 76: $^{31}P\{^1H\}$ NMR (243 MHz, DMSO-d_6, 298 K)

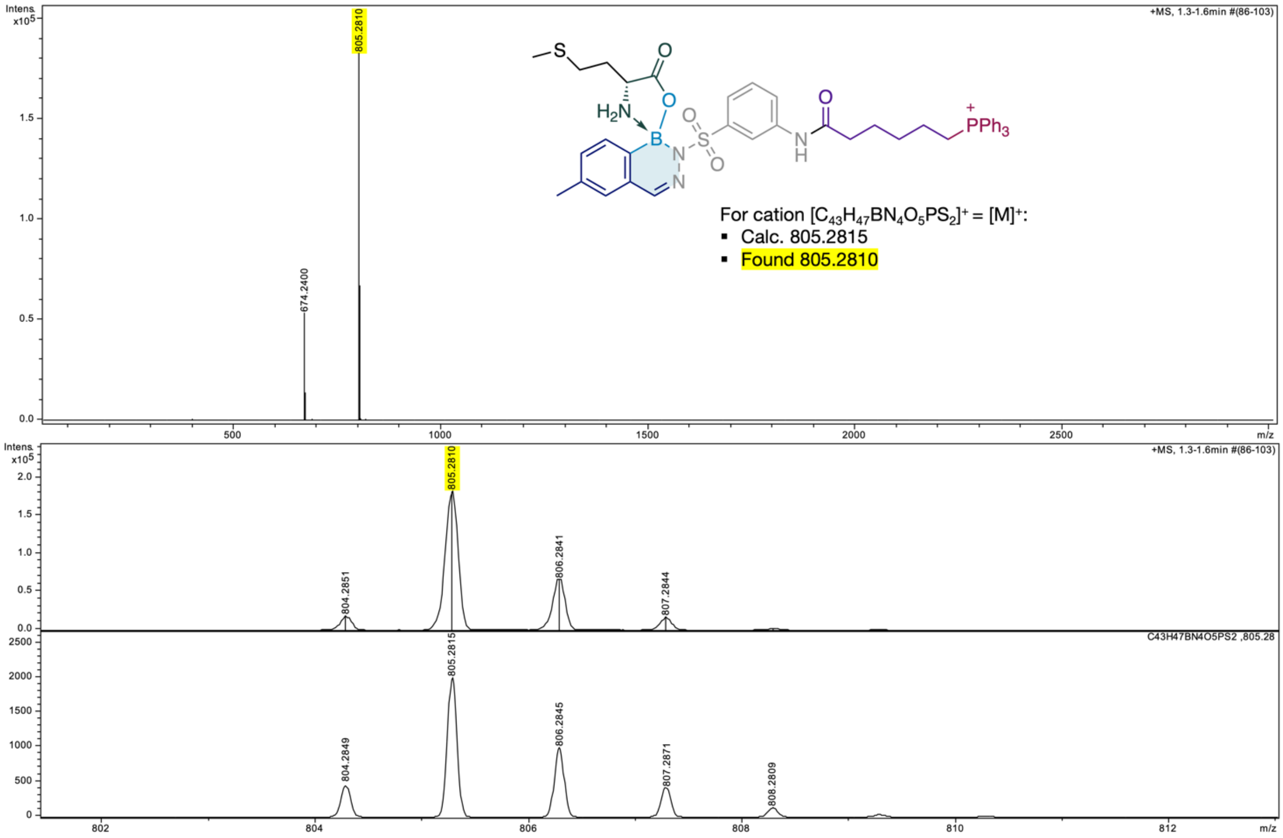

Figure S407. ESI-MS spectrum of Conjugate 75 ($[M]^+$, ionized in CH_3CN-H_2O 4:1, positive mode)

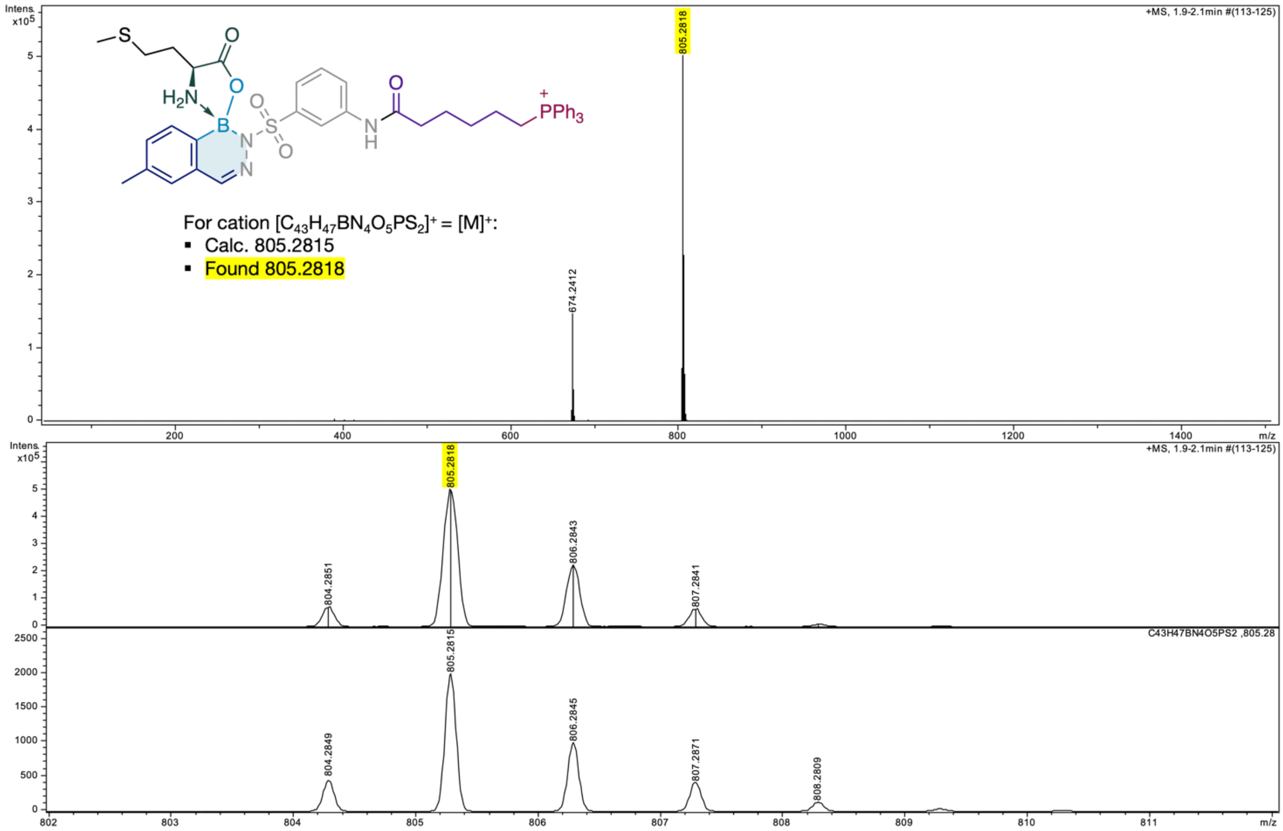

Figure S408. ESI-MS spectrum of Conjugate 76 ($[M]^+$, ionized in CH_3CN-H_2O 4:1, positive mode)

Conjugate diazaborines 77 and 78

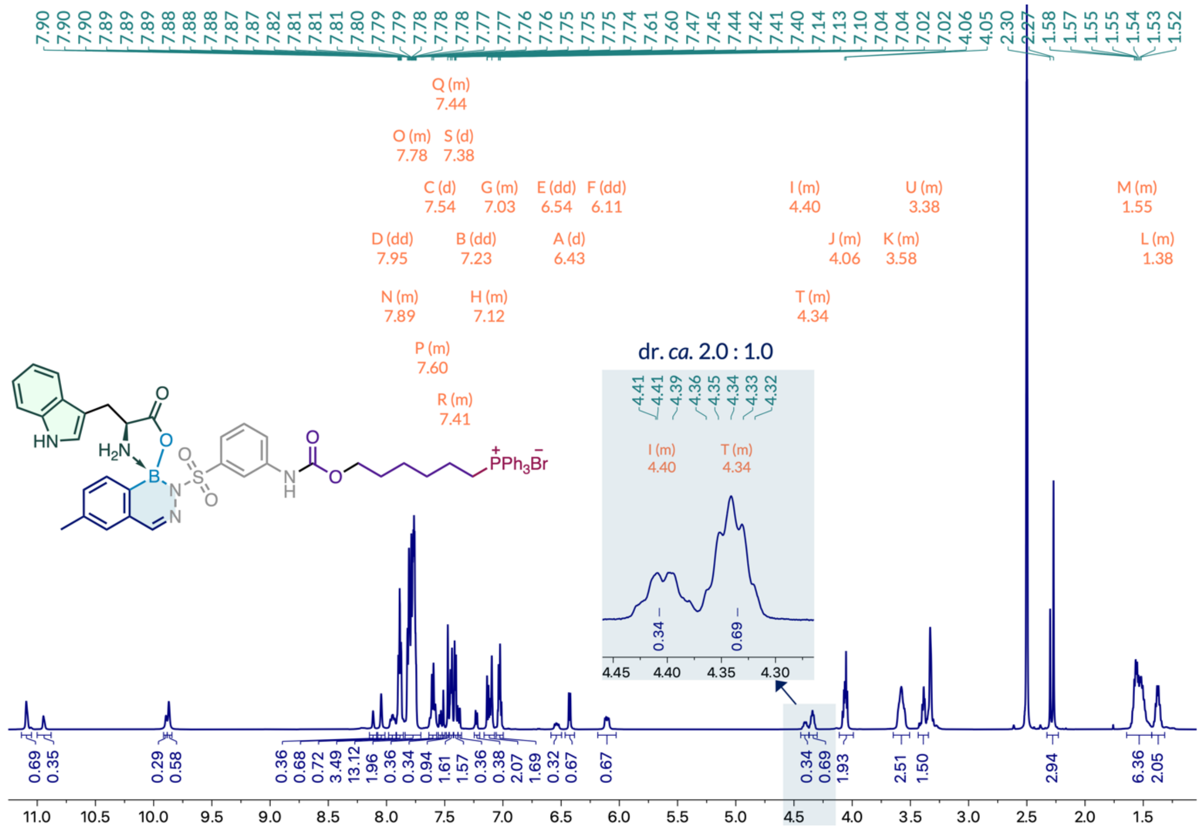

Figure S409. Conjugate 77 and 78:^{1}H NMR (600 MHz, DMSO-d_6, 298 K)

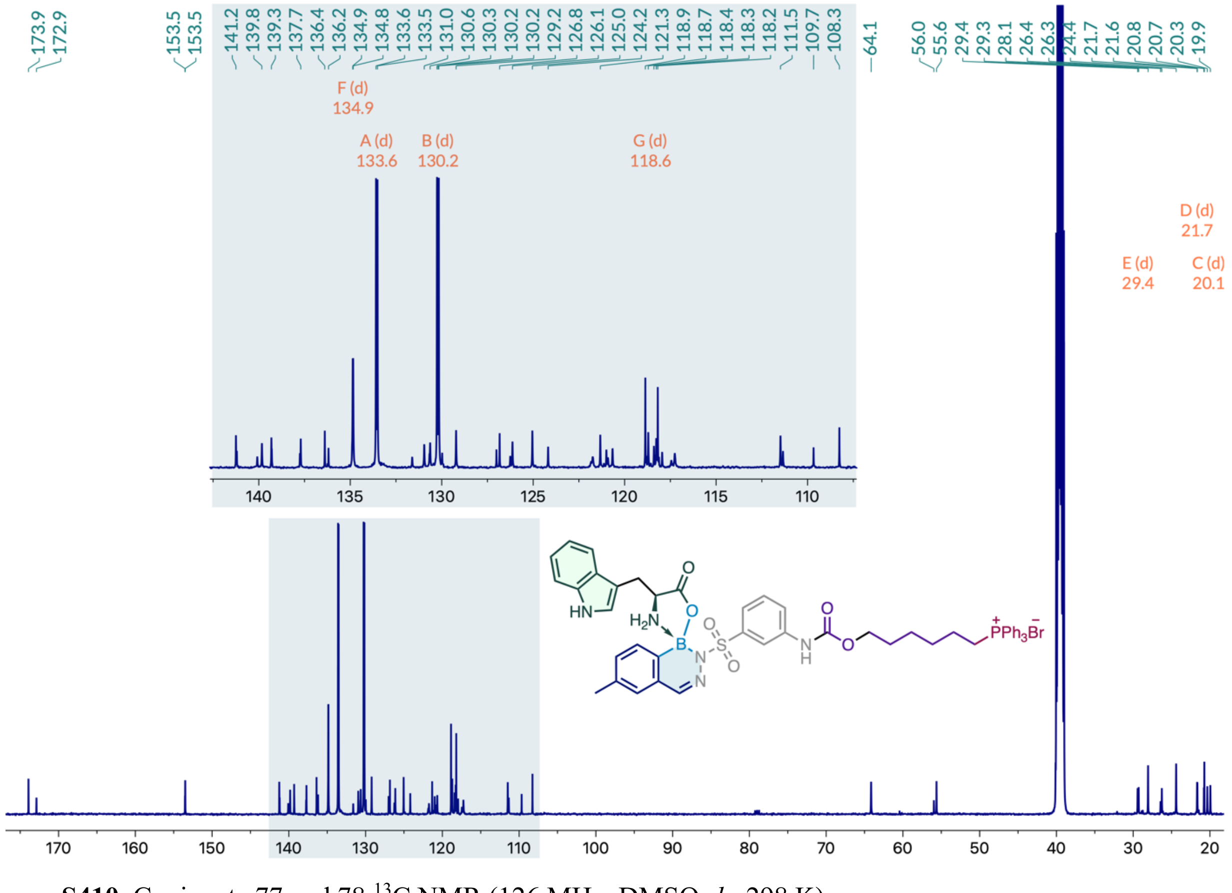

Figure S410. Conjugate 77 and 78:^{13}C NMR (126 MHz, DMSO-d_6, 298 K)

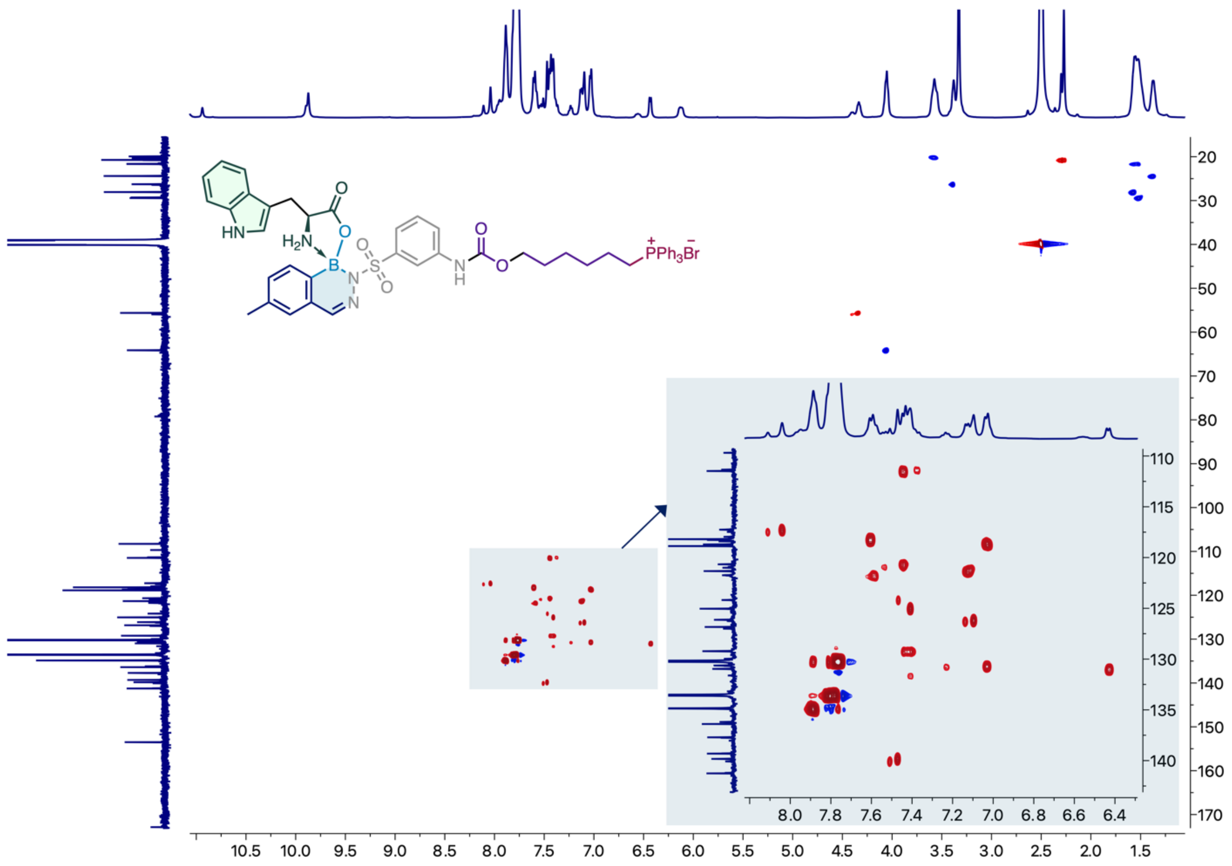

Figure S411. Conjugate 77 and 78: ^{1}H-^{13}C gHSQC NMR (DMSO-d_6, 298 K)

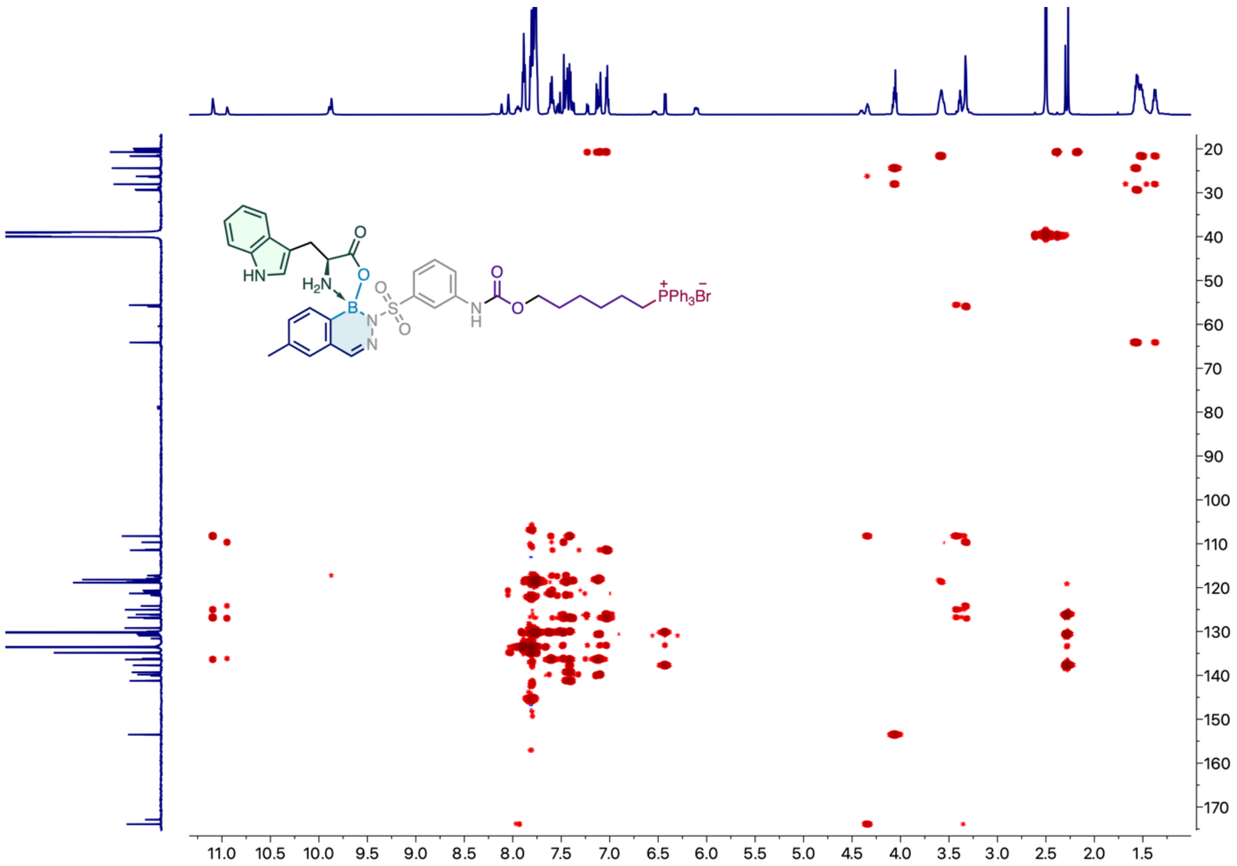

Figure S412. Conjugate 77 and 78: ^{1}H-^{13}C gHMBC NMR (DMSO-d_6, 298 K)

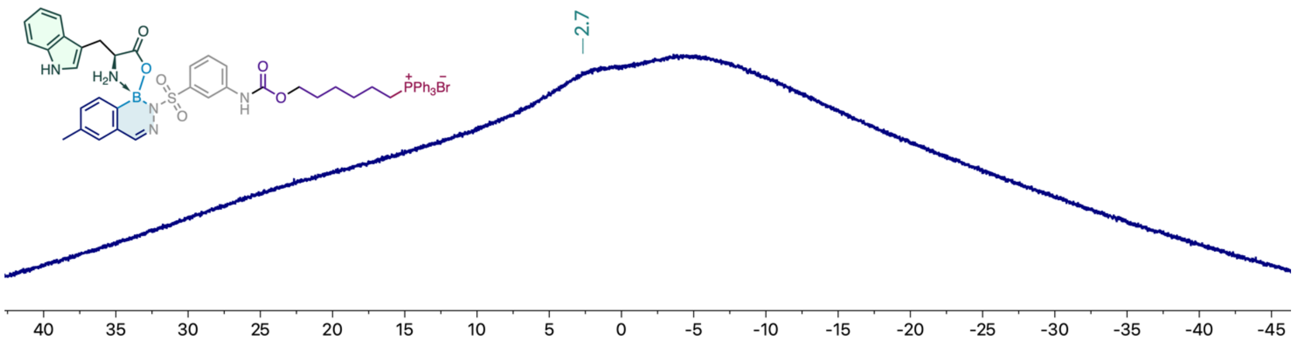

Figure S413. Conjugate 77 and 78: ^{11}B NMR (160 MHz, DMSO-d_6, 298 K)

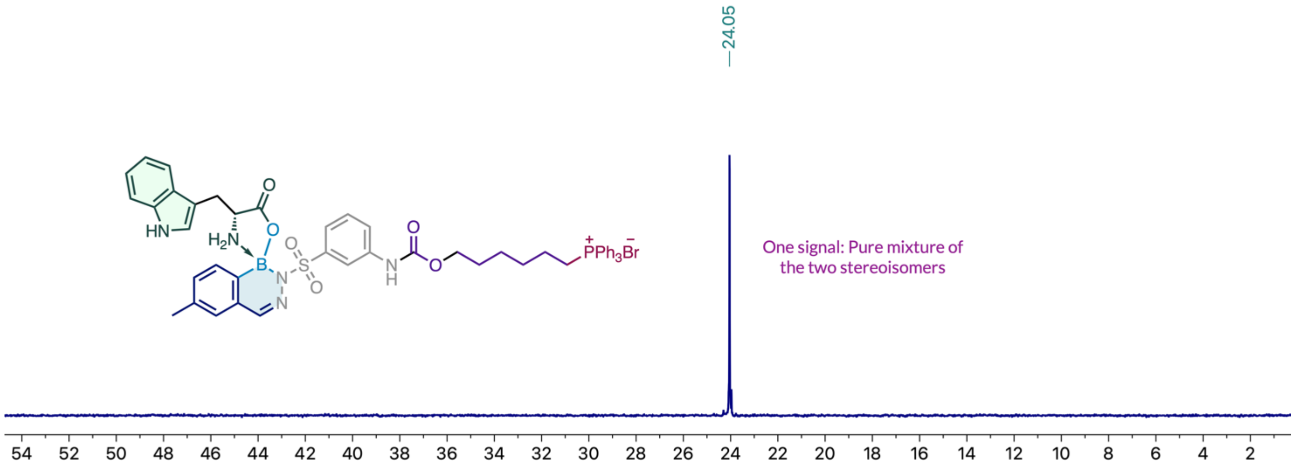

Figure S414. Conjugate 77: $^{31}P\{^1H\}$ NMR (162 MHz, DMSO-d_6, 298 K)

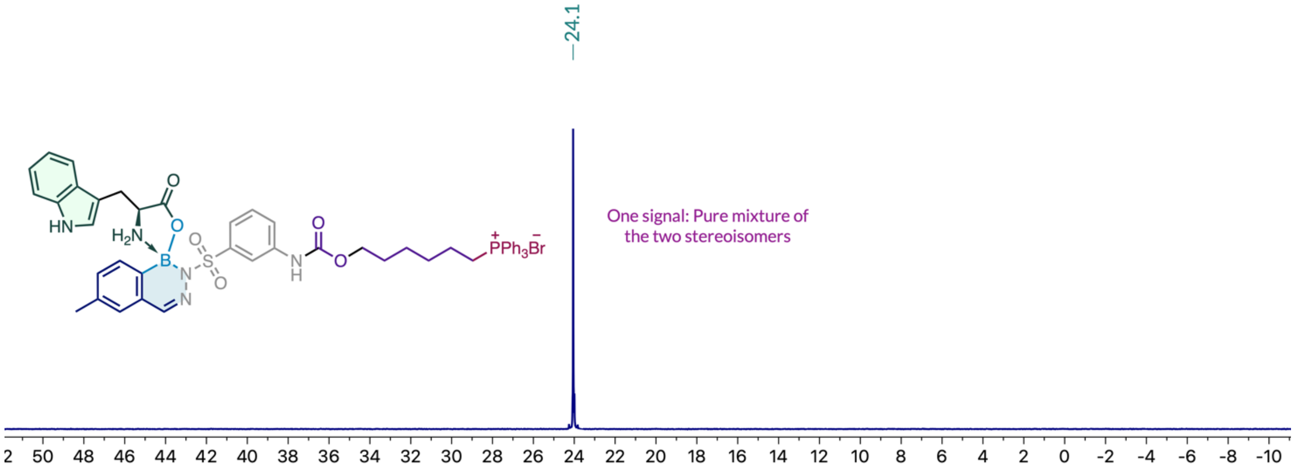

Figure S415. Conjugate 78: $^{31}P\{^1H\}$ NMR (202 MHz, DMSO-d_6, 298 K)

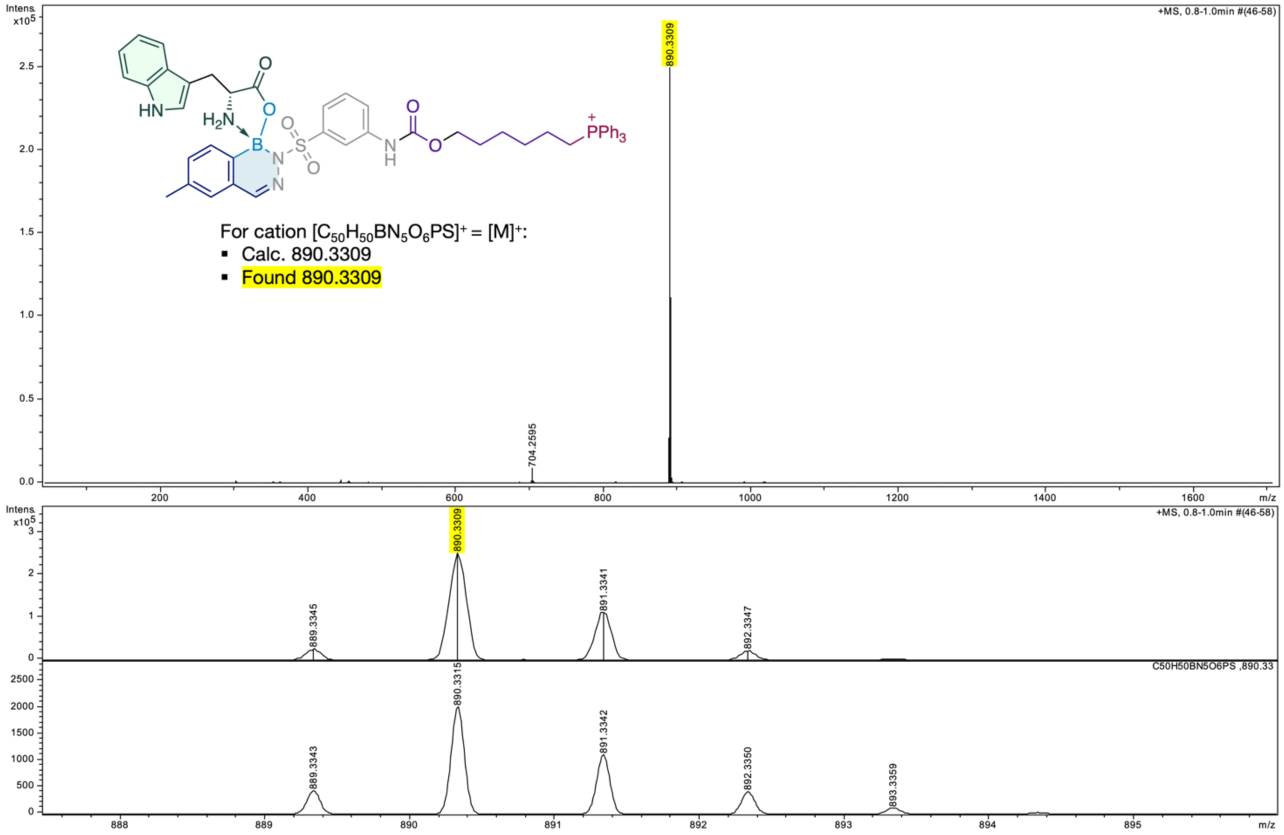

Figure S416. ESI-MS spectrum of Conjugate 77 ($[M]^+$, ionized in CH_3CN-H_2O 4:1, positive mode)

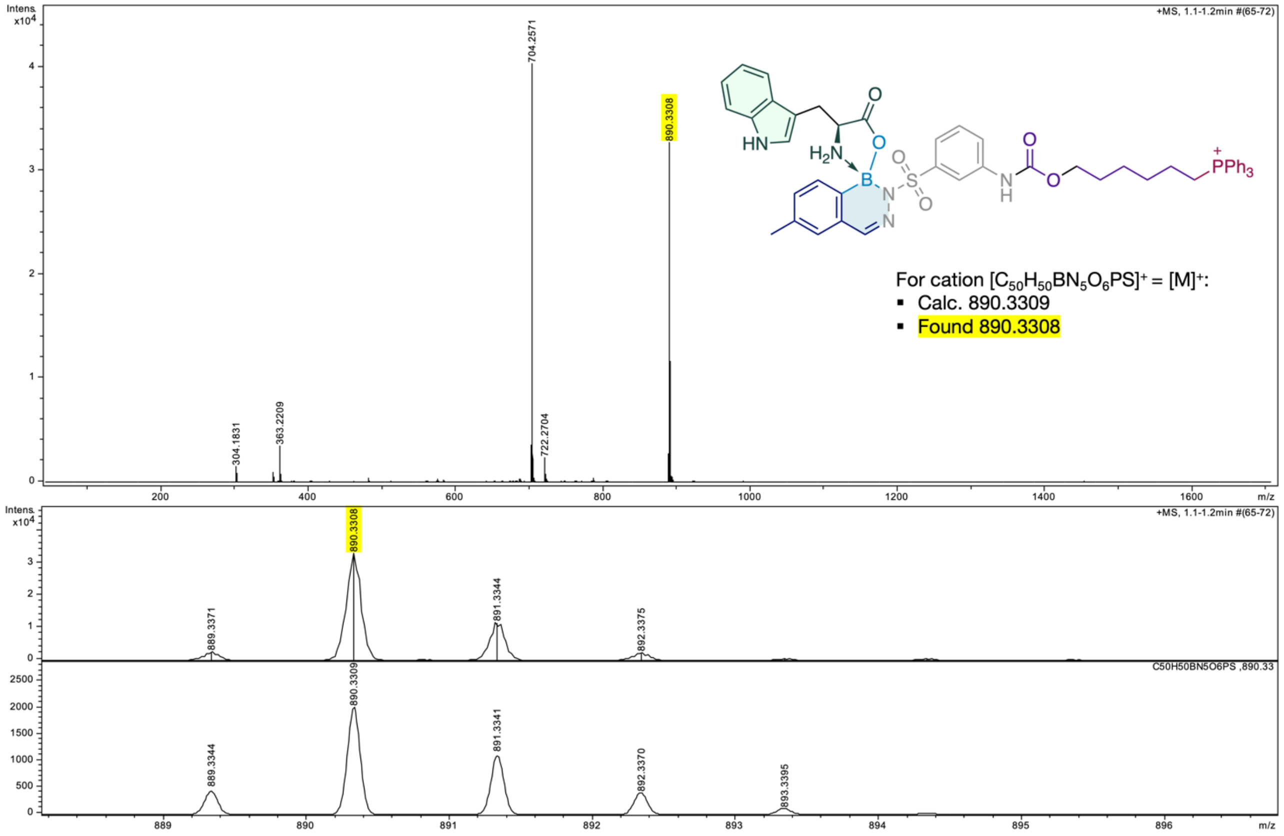

Figure S417. ESI-MS spectrum of Conjugate 78 ($[M]^+$, ionized in CH_3CN-H_2O 4:1, positive mode)

Conjugate diazaborines 79 and 80

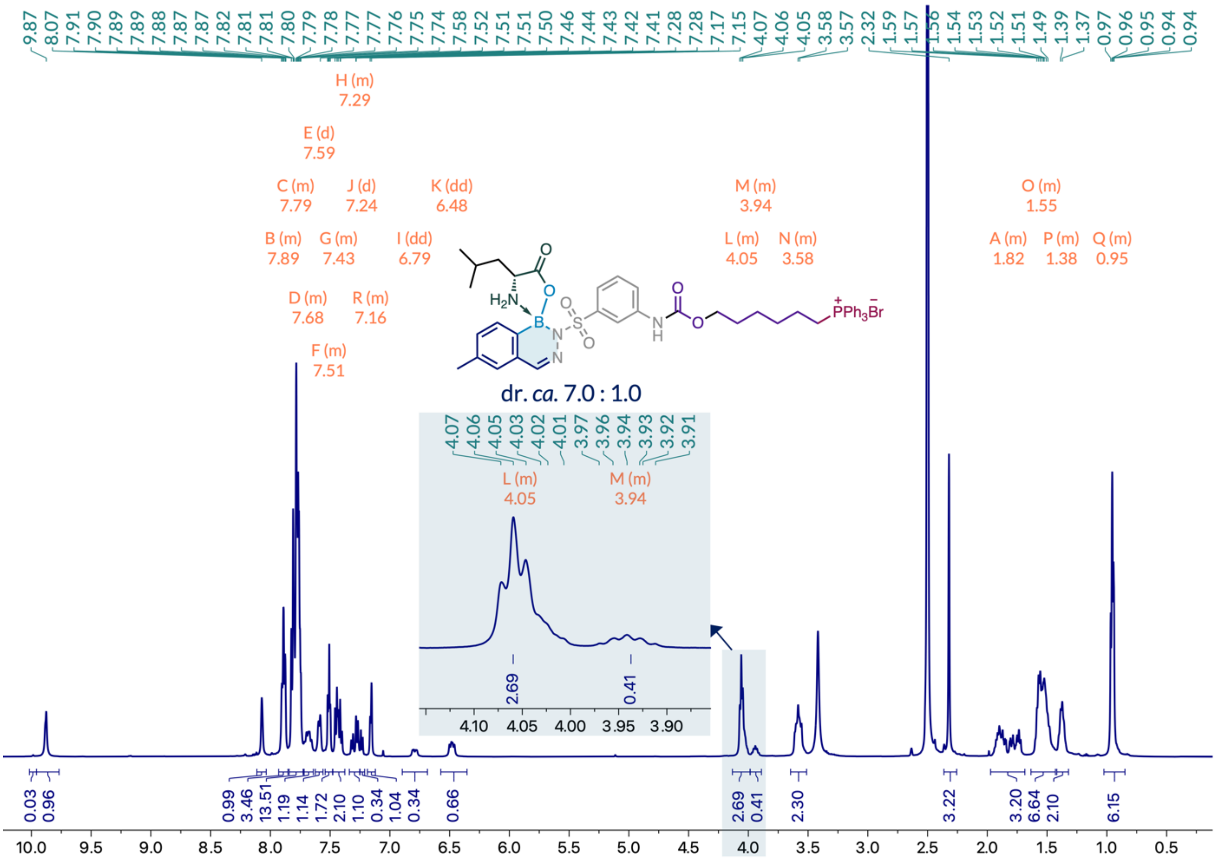

Figure S418. Conjugate 79 and 80: ^{1}H NMR (500 MHz, DMSO-d_6, 298 K)

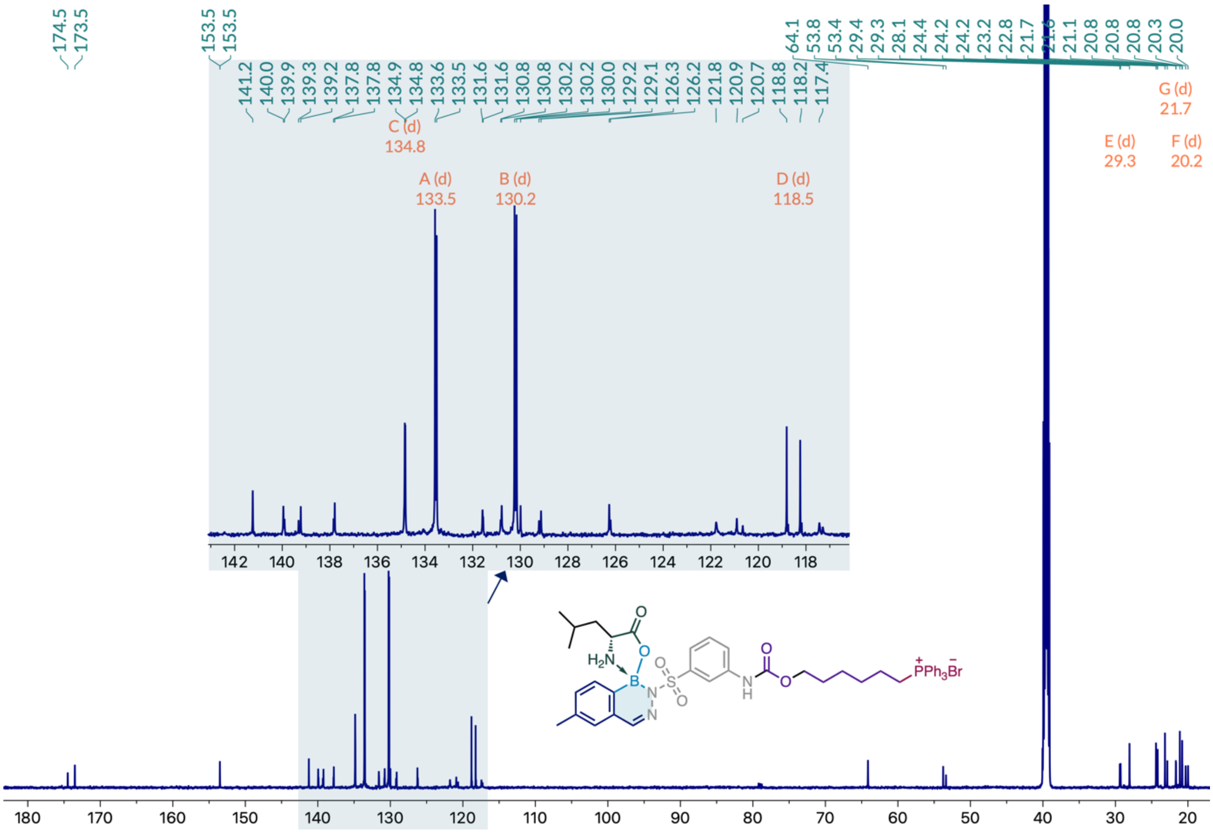

Figure S419. Conjugate 79 and 80: ^{13}C NMR (151 MHz, DMSO-d_6, 298 K)

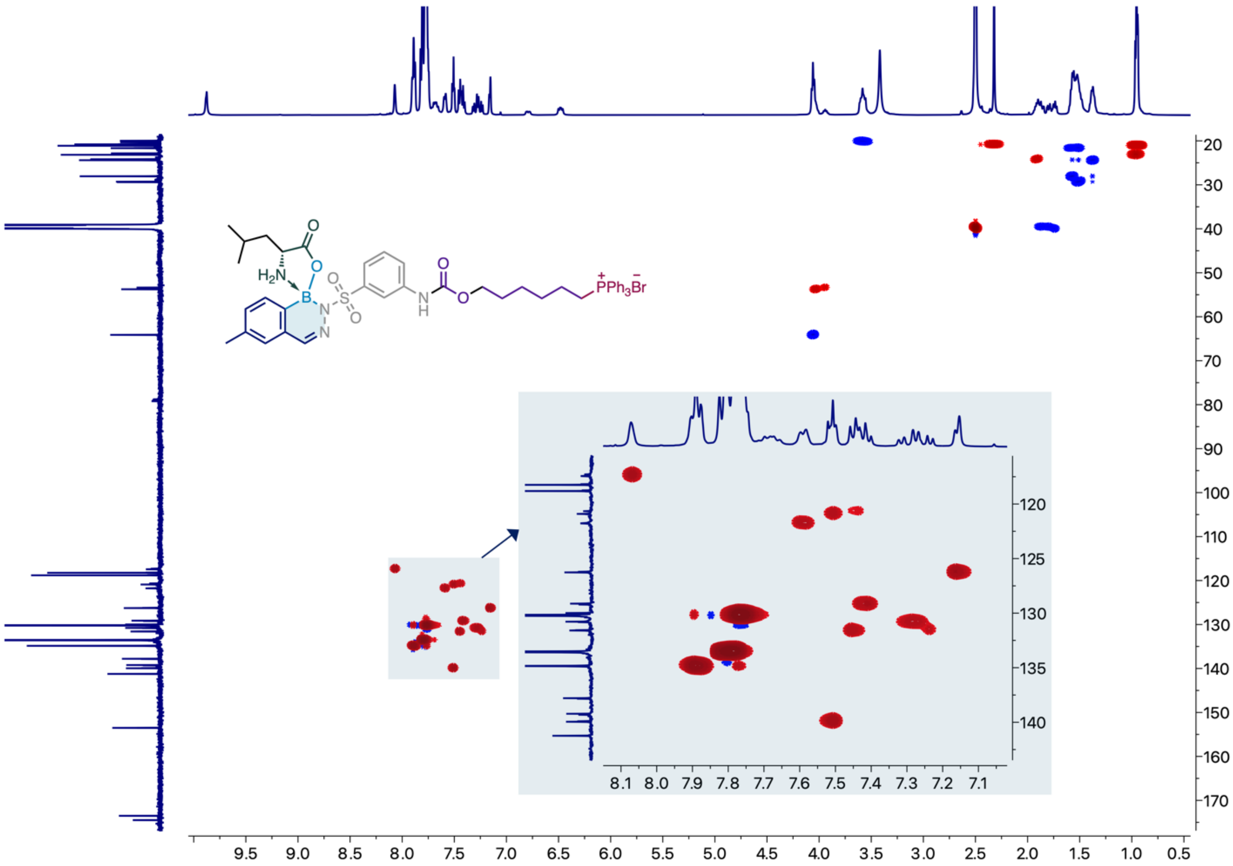

Figure S420. Conjugate 79 and 80: ^{1}H-^{13}C gHSQC NMR (DMSO-d_6, 298 K)

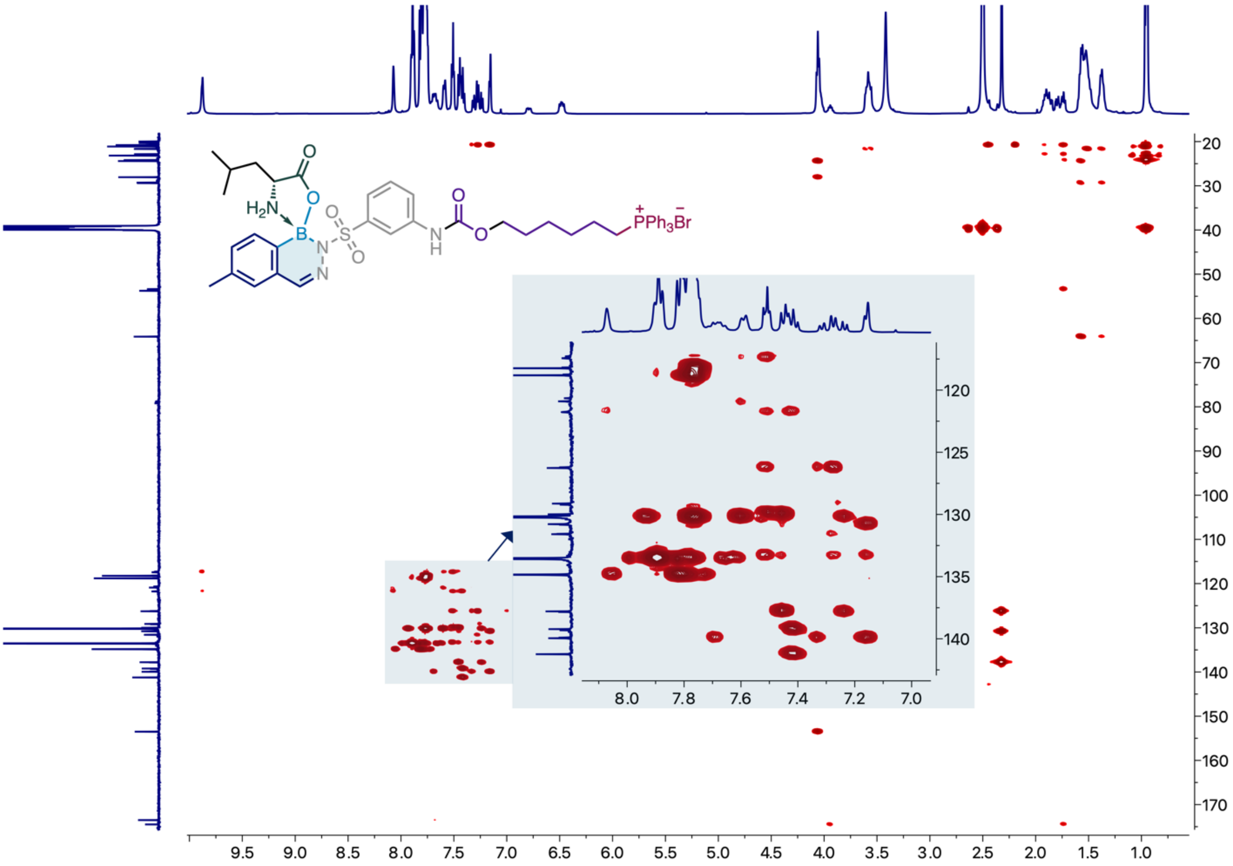

Figure S421. Conjugate 79 and 80: ^{1}H-^{13}C gHMBC NMR (DMSO-d_6, 298 K)

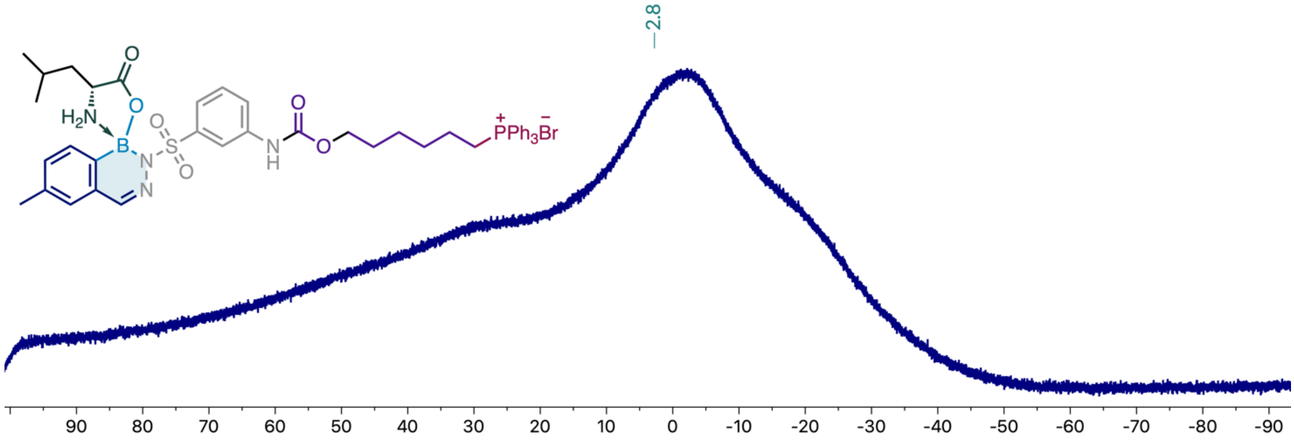

Figure S422. Conjugate 79 and 80: ^{11}B NMR (128 MHz, DMSO-d_6, 298 K)

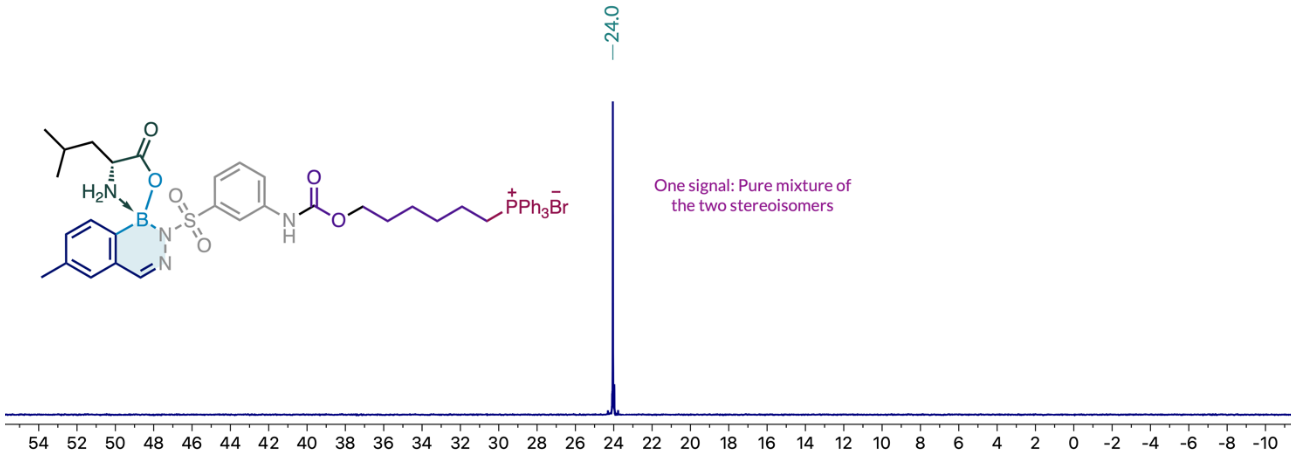

Figure S423. Conjugate 79: ^{31}P{^{1}H} NMR (162 MHz, DMSO-d_6, 298 K)

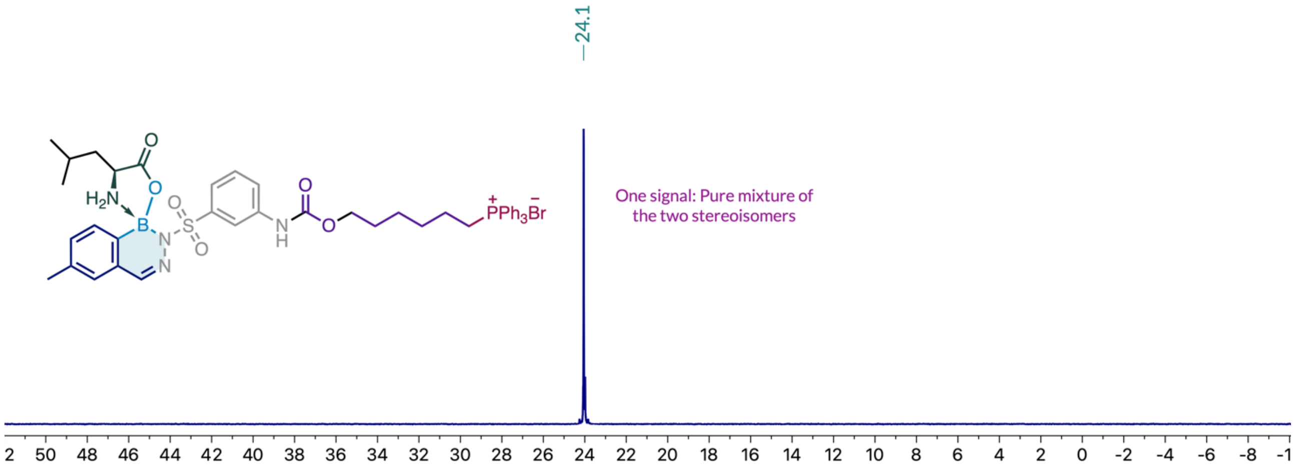

Figure S424. Conjugate 80: ^{31}P{^{1}H} NMR (202 MHz, DMSO-d_6, 298 K)

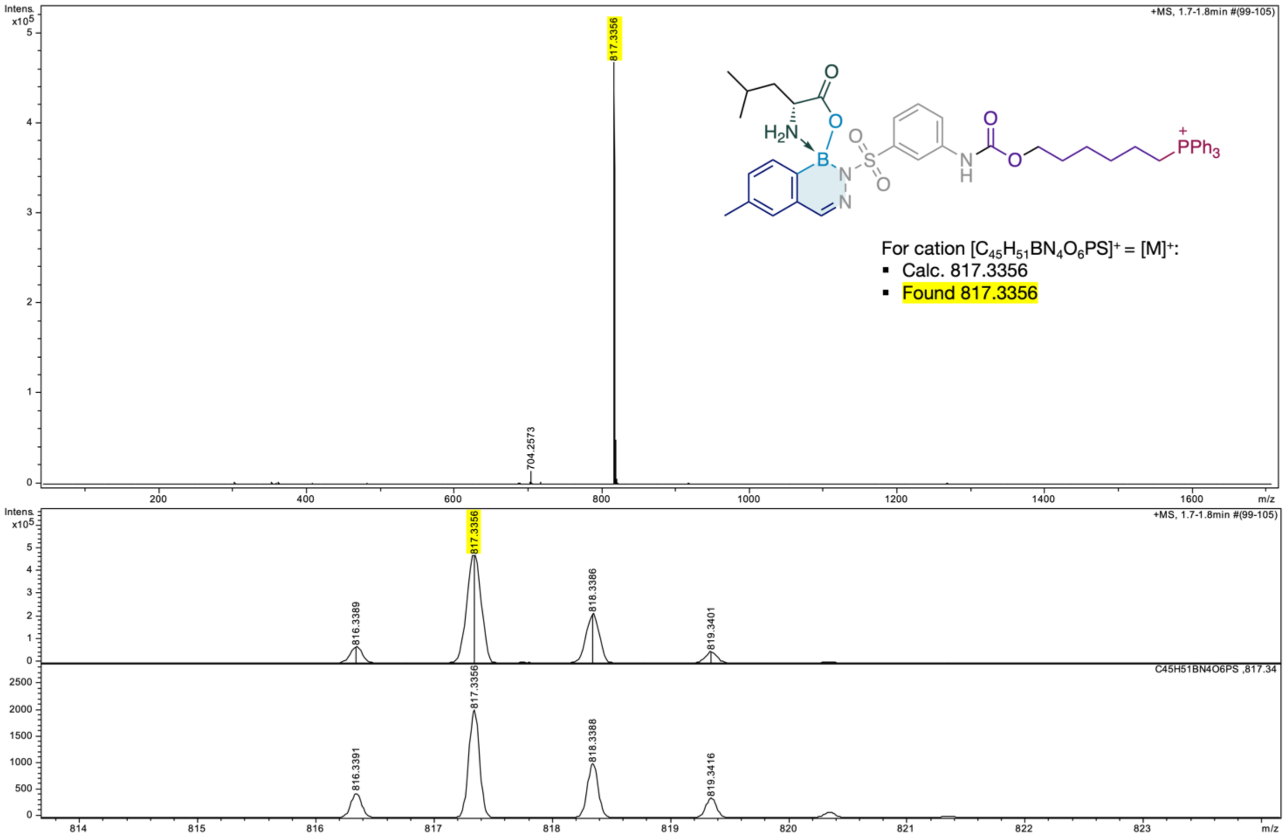

Figure S425. ESI-MS spectrum of Conjugate 79 ($[M]^+$, ionized in CH_3CN-H_2O 4:1, positive mode)

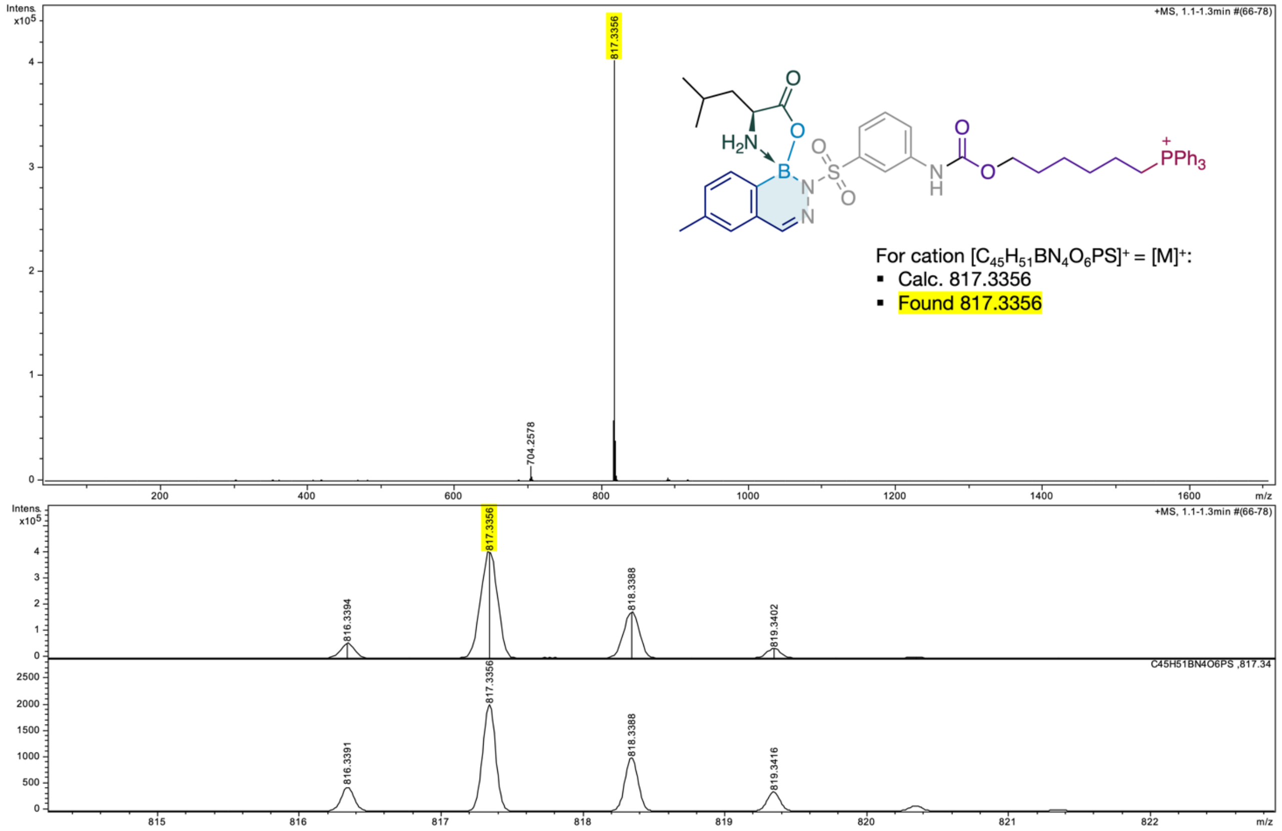

Figure S426. ESI-MS spectrum of Conjugate 80 ($[M]^+$, ionized in CH_3CN-H_2O 4:1, positive mode)

Conjugate diazaborines 81 and 82

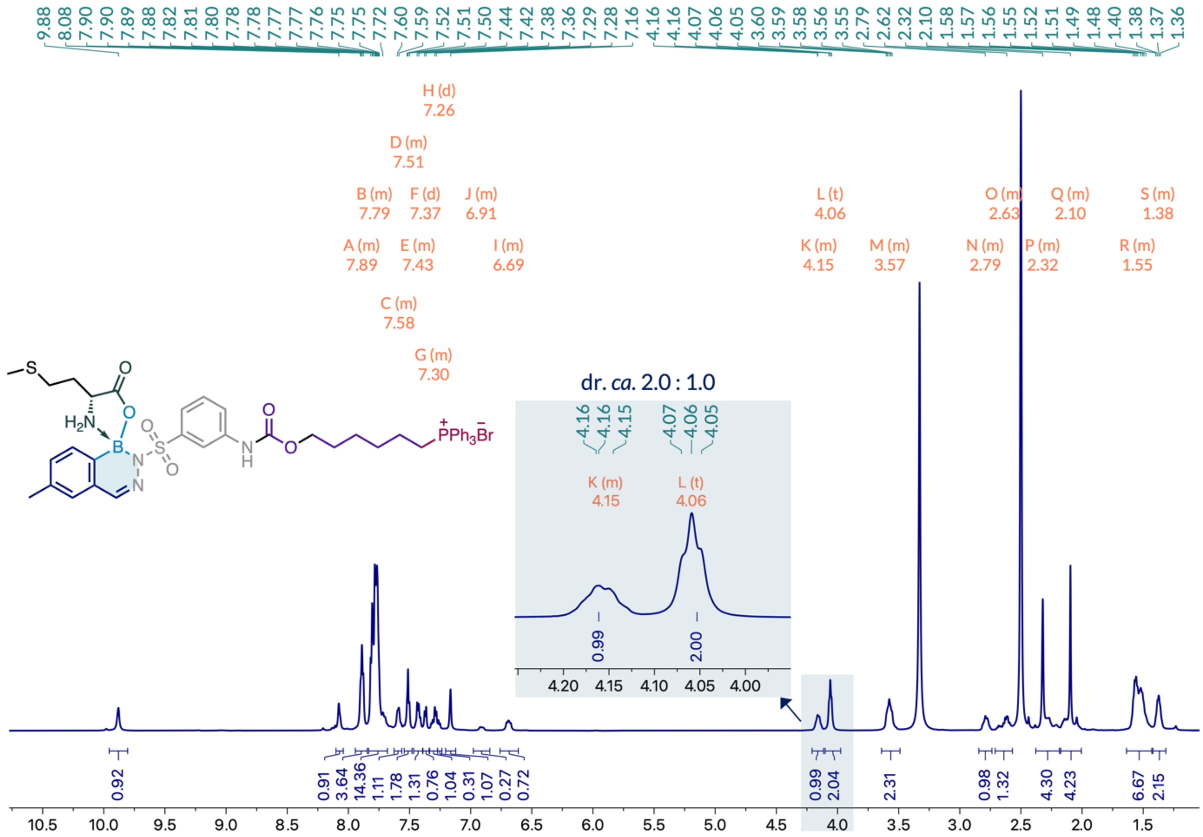

Figure S427. Conjugate 81 and 82:^{1}H NMR (600 MHz, DMSO-d_6, 298 K)

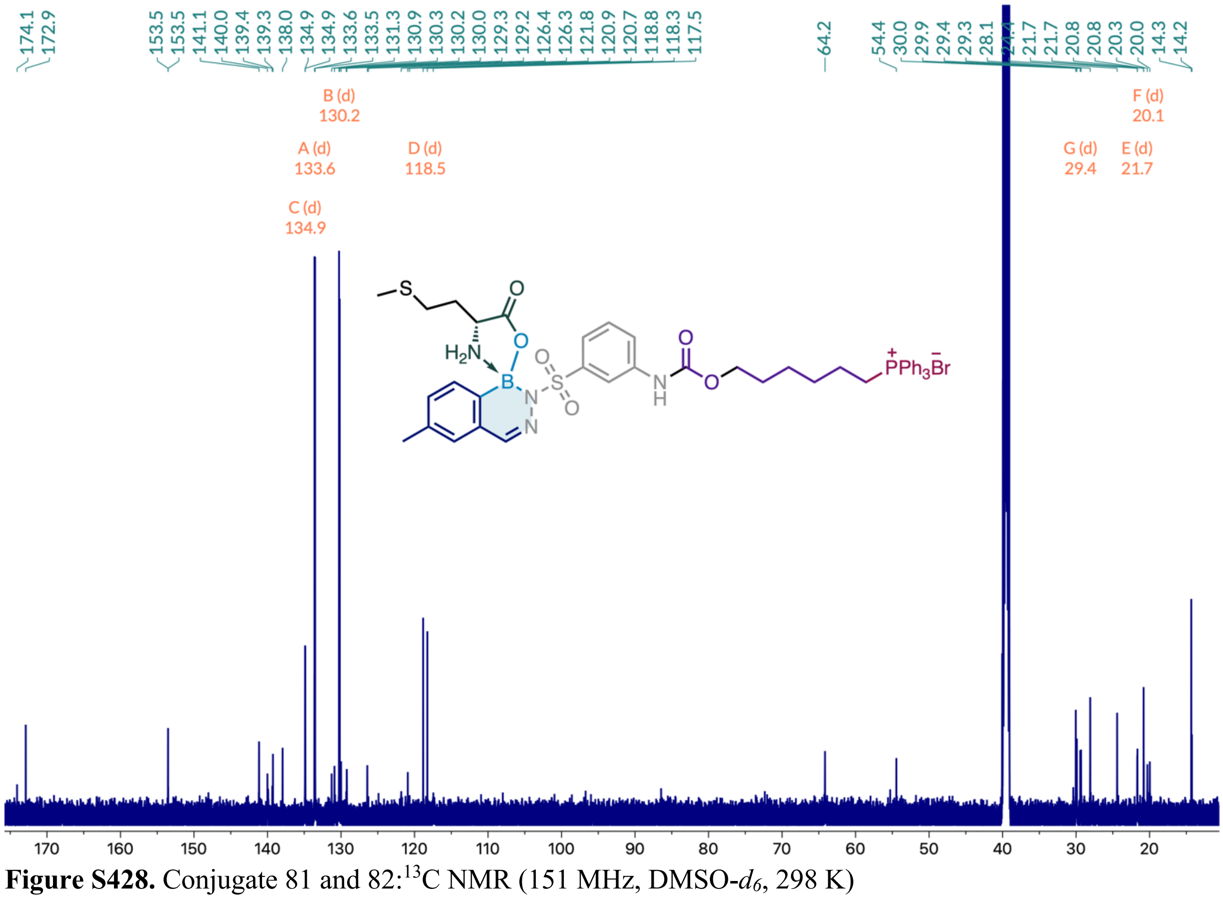

Figure S428. Conjugate 81 and 82:^{13}C NMR (151 MHz, DMSO-d_6, 298 K)

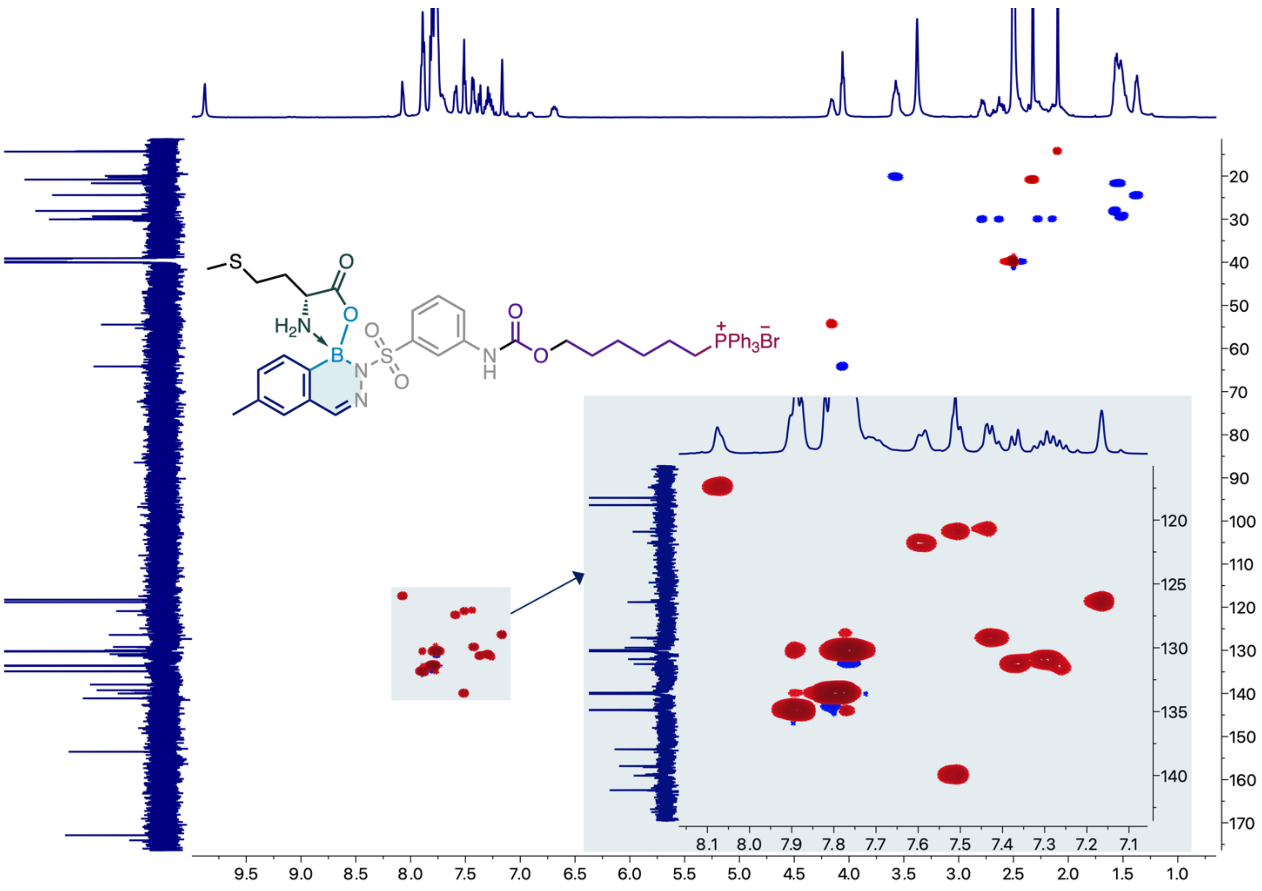

Figure S429. Conjugate 81 and 82: ^{1}H-^{13}C gHSQC NMR (DMSO-d_6, 298 K)

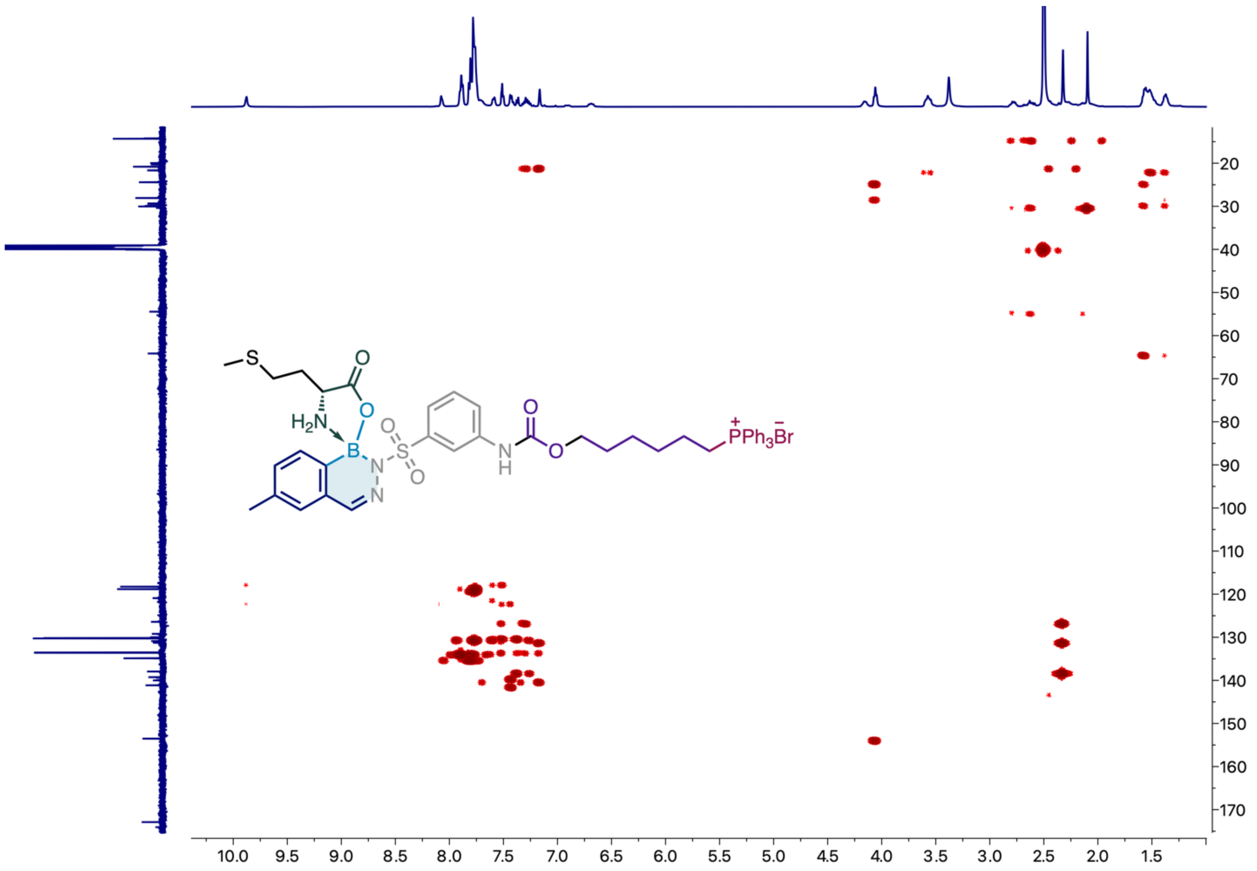

Figure S430. Conjugate 81 and 82: ^{1}H-^{13}C gHMBC NMR (DMSO-d_6, 298 K)

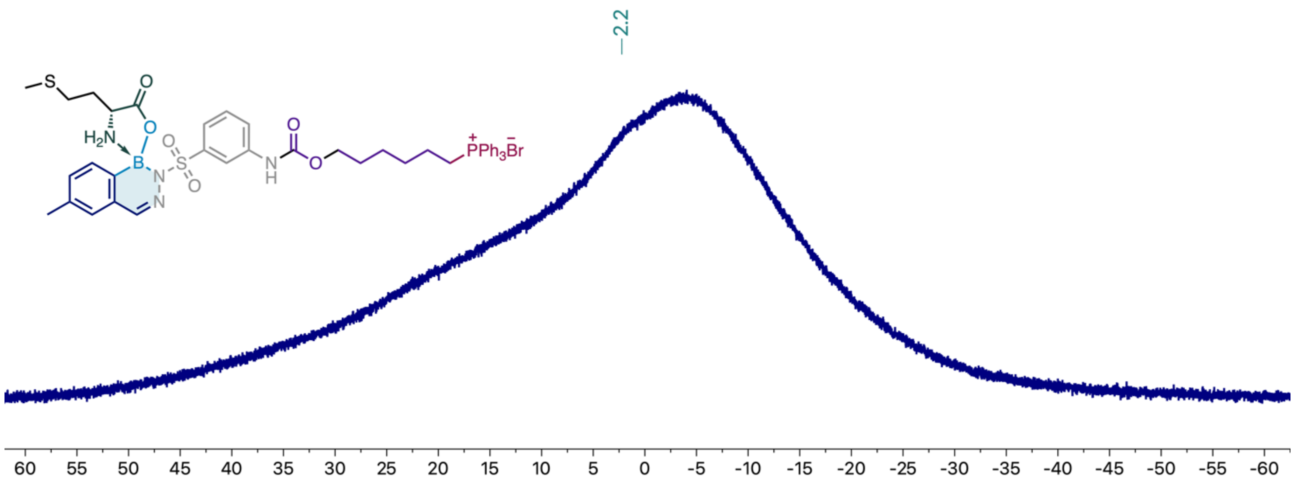

Figure S431. Conjugate 81 and 82: ^{11}B NMR (160 MHz, DMSO-d_6, 298 K)

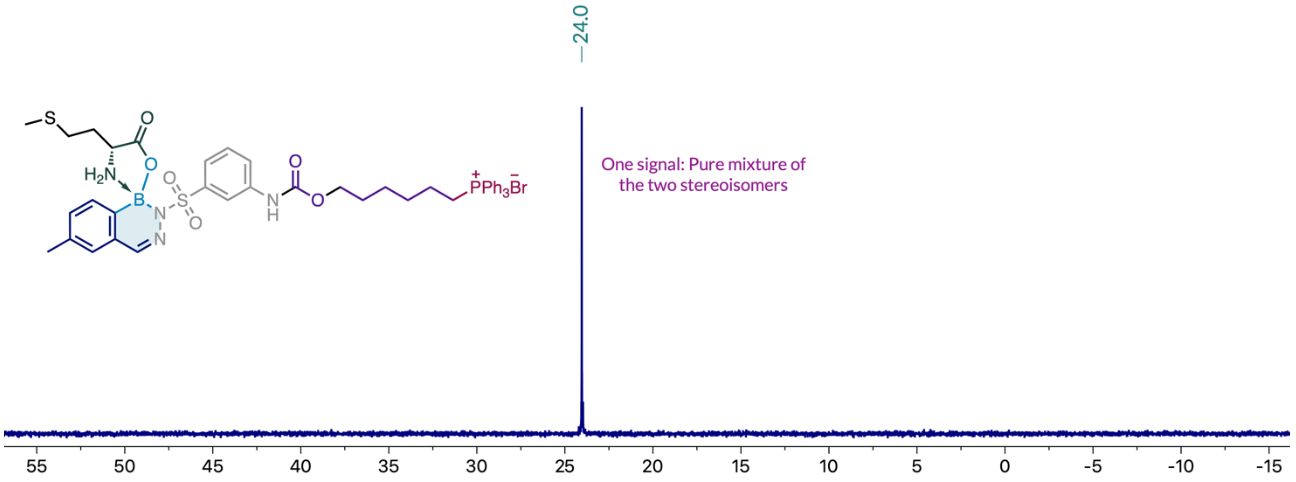

Figure S432. Conjugate 81: $^{31}P\{^1H\}$ NMR (243 MHz, DMSO-d_6, 298 K)

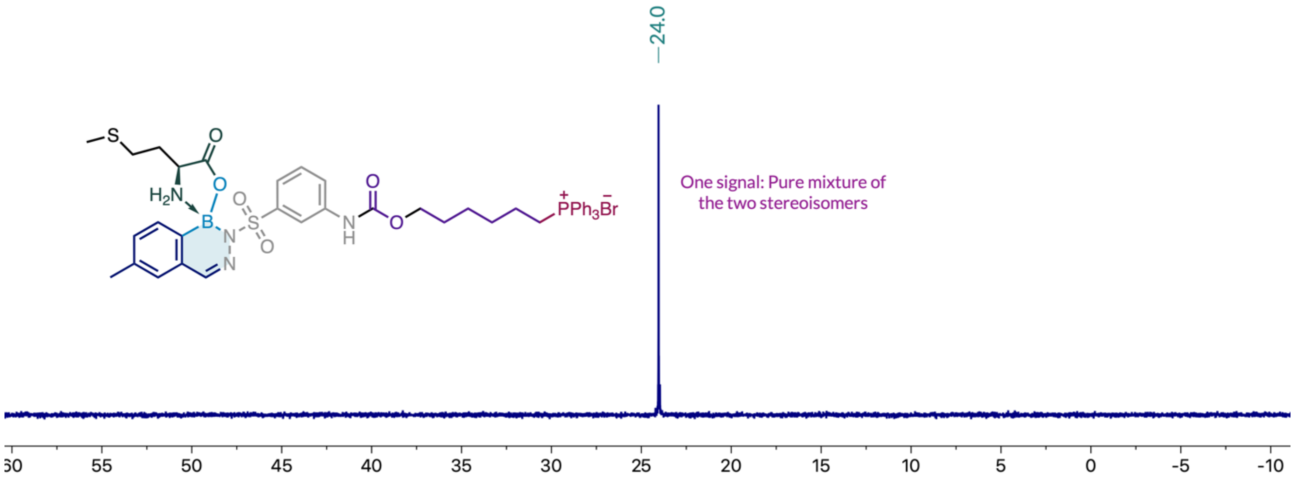

Figure S433. Conjugate 82: $^{31}P\{^1H\}$ NMR (243 MHz, DMSO-d_6, 298 K)

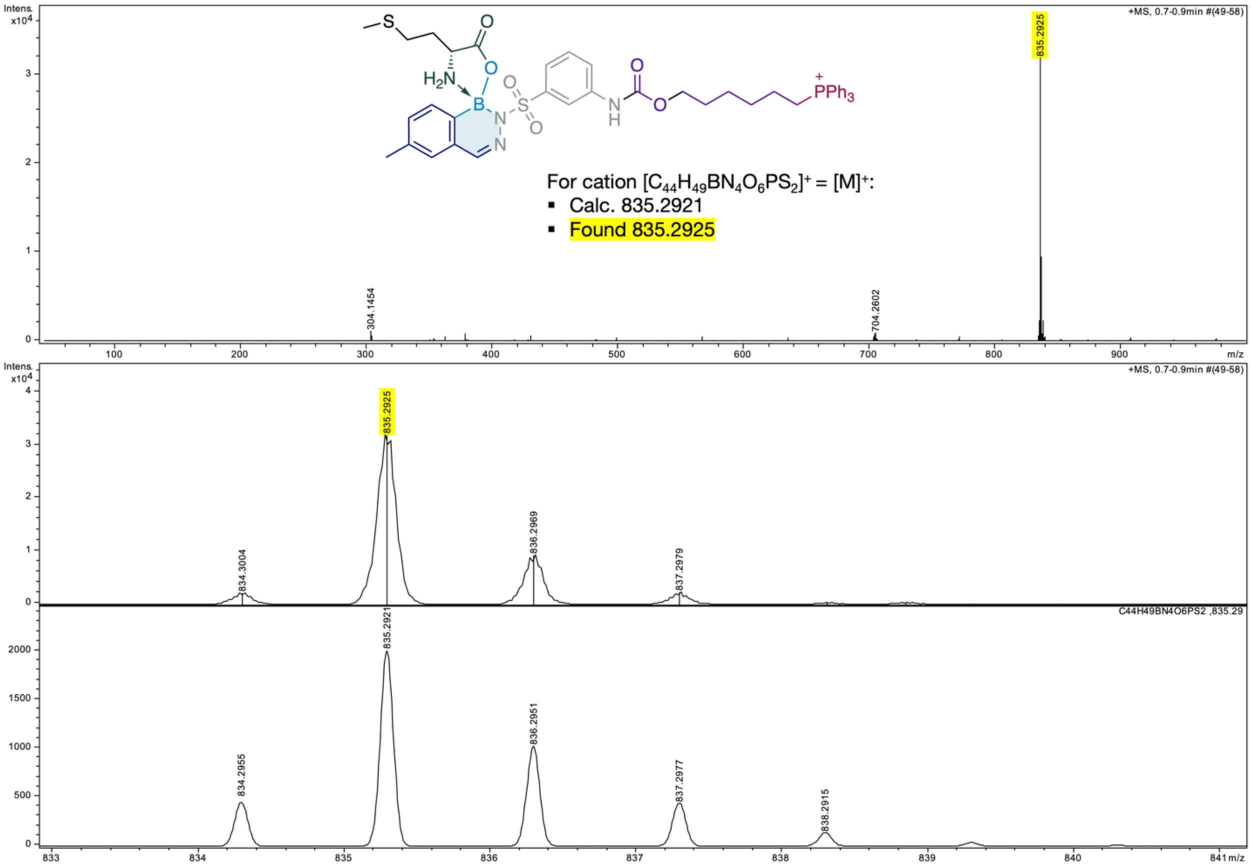

Figure S434. ESI-MS spectrum of Conjugate 81 ($[M]^+$, ionized in CH_3CN-H_2O 4:1, positive mode)

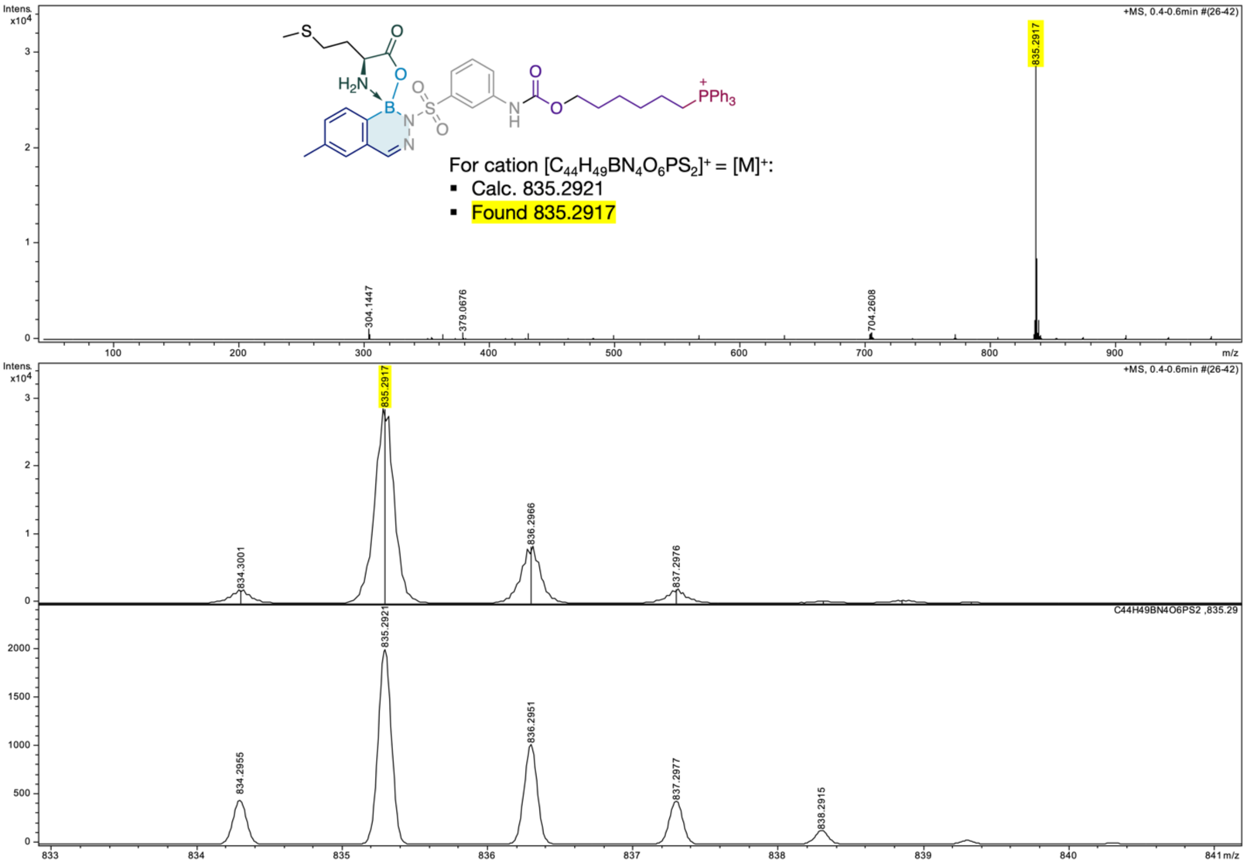

Figure S435. ESI-MS spectrum of Conjugate 82 ($[M]^+$, ionized in CH_3CN-H_2O 4:1, positive mode)

Precursors

Hydrazides

Benzenesulfonohydrazide (S1)

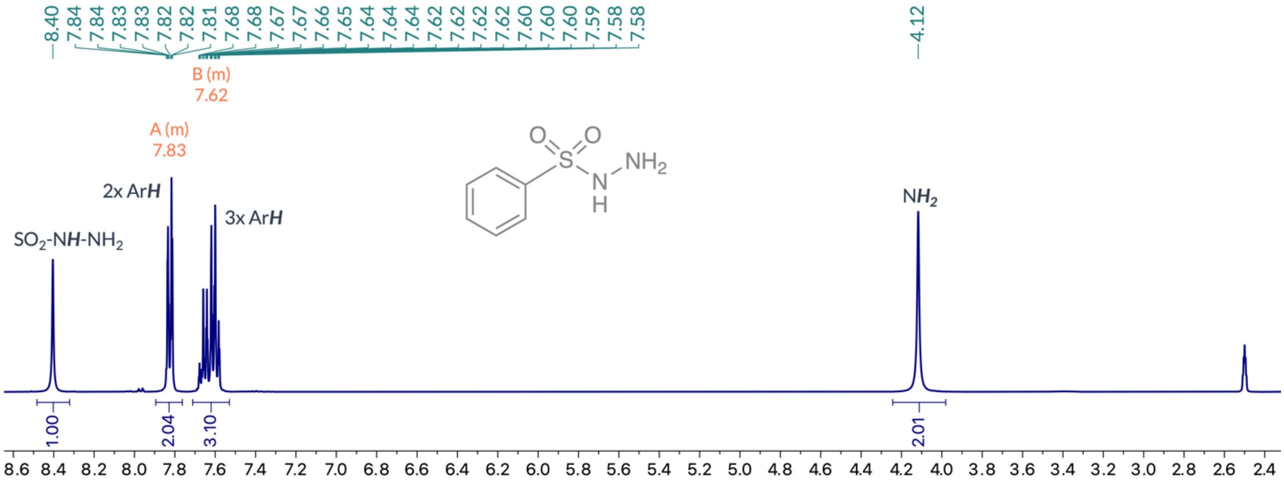

Figure S436. Hydrazide S1: ^{1}H NMR (400 MHz, DMSO-d_6, 298 K)

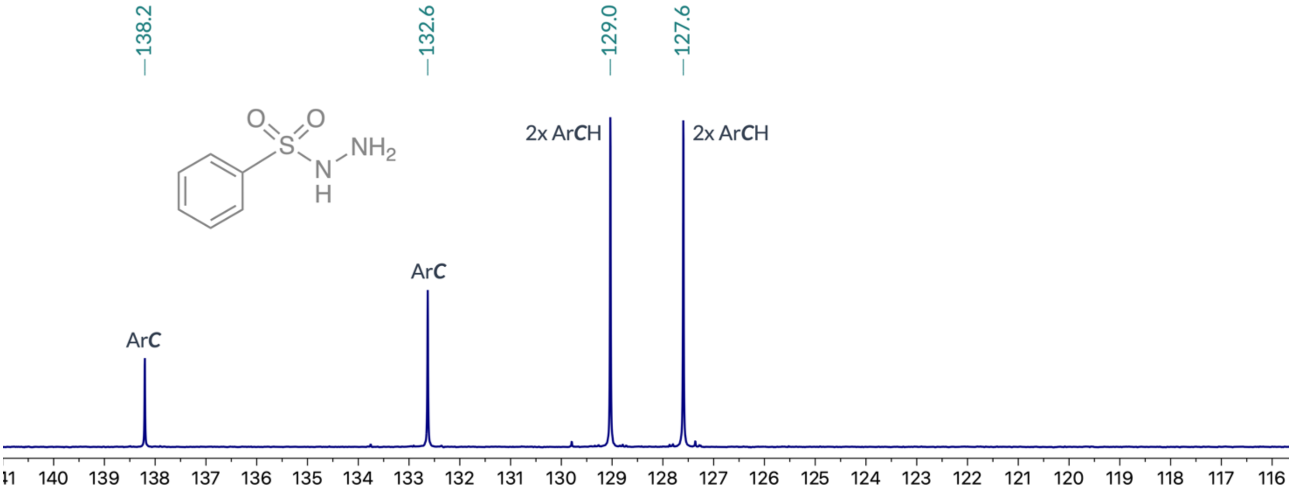

Figure S437. Hydrazide S1: ^{13}C NMR (101 MHz, DMSO-d_6, 298 K)

3-Nitrobenzenesulfonohydrazide (S3)

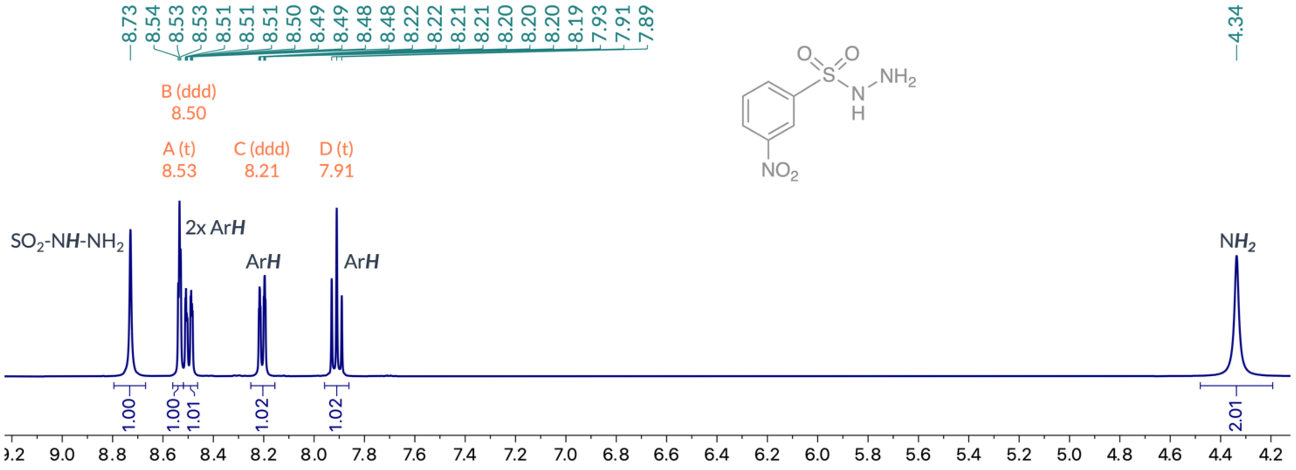

Figure S438. Hydrazide S3:^{1}H NMR (400 MHz, DMSO-d_6, 298 K)

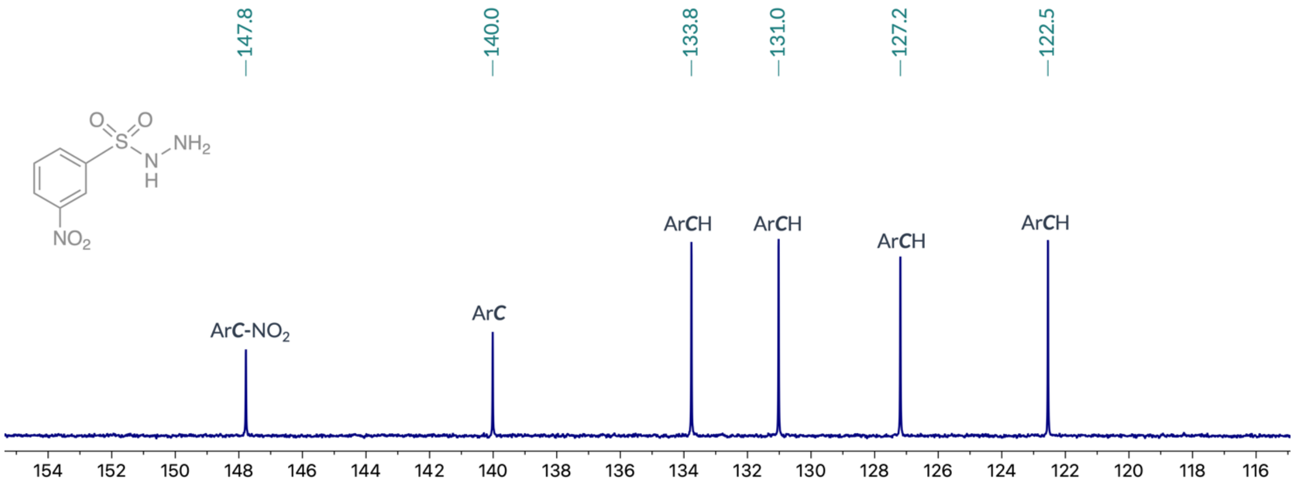

Figure S439. Hydrazide S3:^{13}C NMR (101 MHz, DMSO-d_6, 298 K)

4-Nitrobenzenesulfonohydrazide (S4)

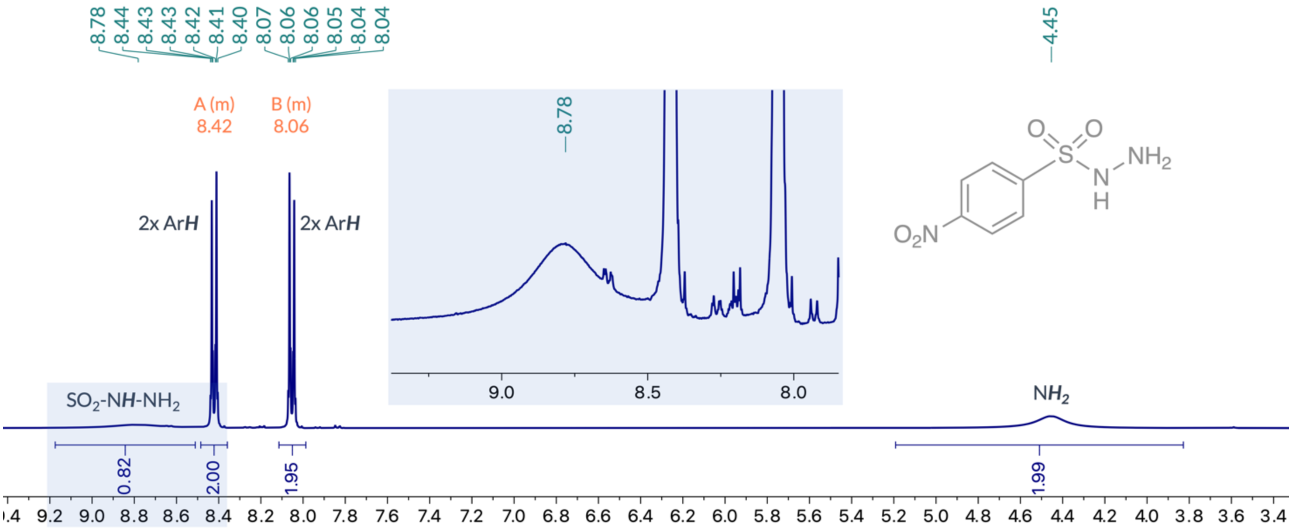

Figure S440. Hydrazide S4:^{1}H NMR (400 MHz, DMSO-d_6, 298 K)

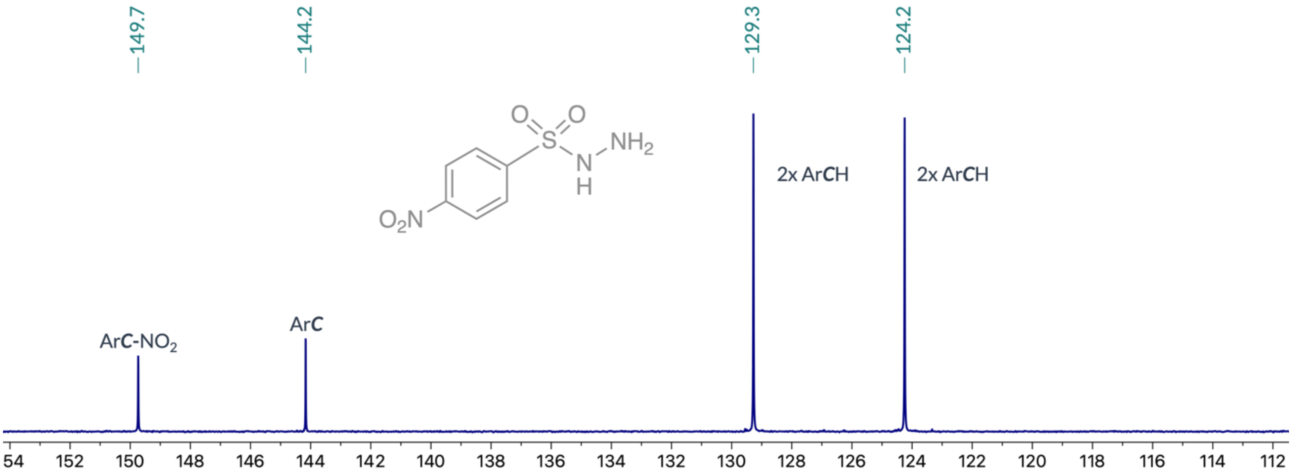

Figure S441. Hydrazide S4:^{13}C NMR (101 MHz, DMSO-d_6, 298 K)

4-Fluorobenzenesulfonohydrazide (S5)

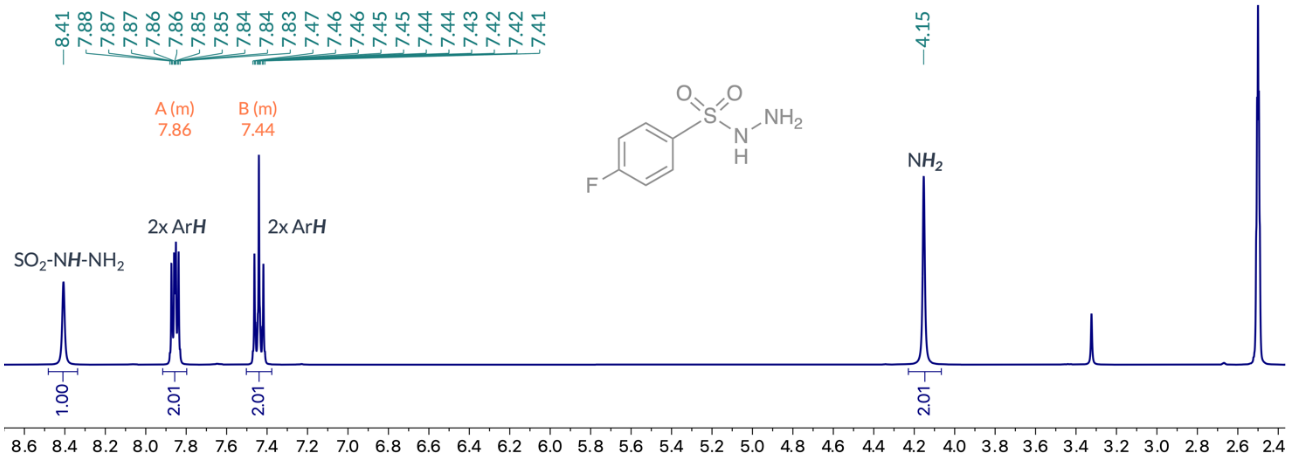

Figure S442. Hydrazide S5:^{1}H NMR (400 MHz, DMSO-d_6, 298 K)

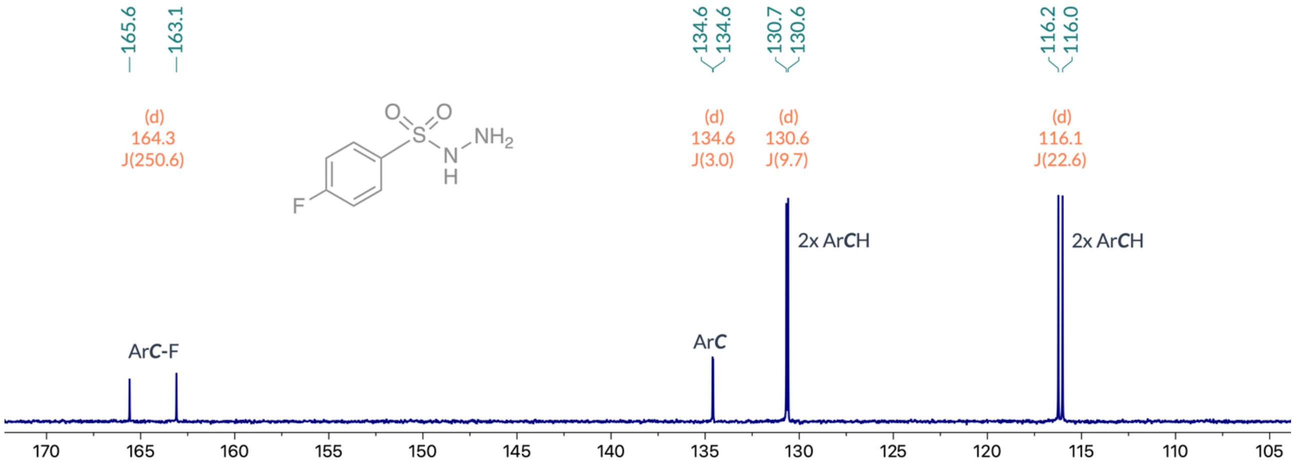

Figure S443. Hydrazide S5:^{13}C NMR (101 MHz, DMSO-d_6, 298 K)

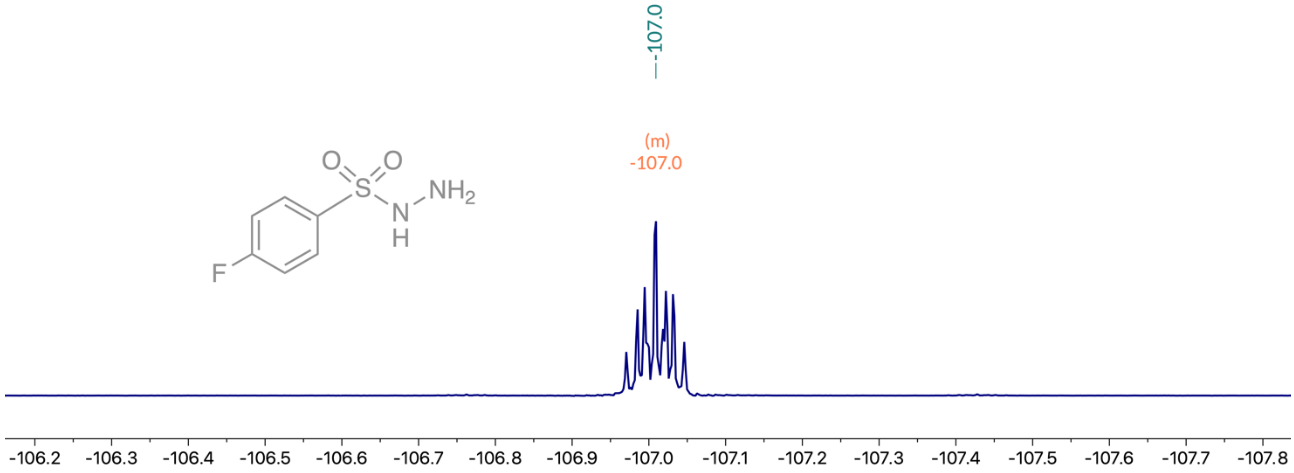

Figure S444. Hydrazide S5:^{19}F NMR (377 MHz, DMSO-d_6, 298 K)

Propane-1-sulfonohydrazide (S6)

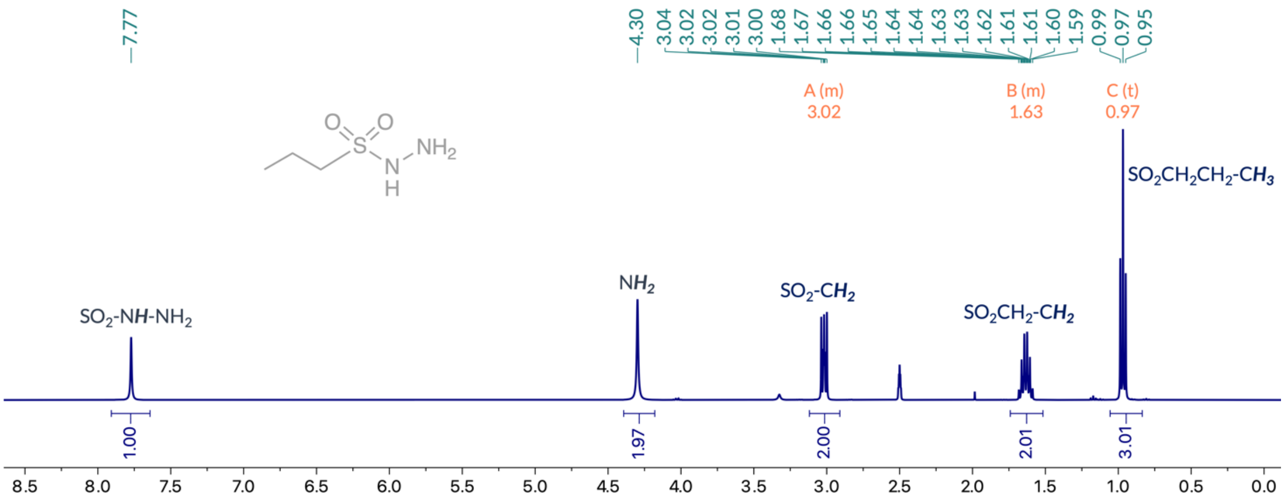

Figure S445. Hydrazide S6:^{1}H NMR (400 MHz, DMSO-d_6, 298 K)

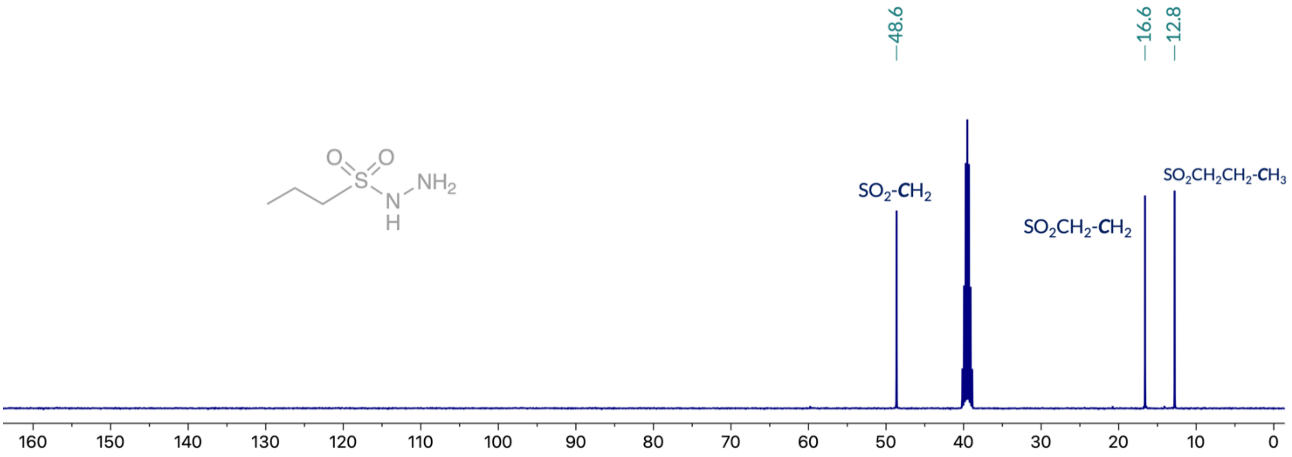

Figure S446. Hydrazide S6:^{13}C NMR (101 MHz, DMSO-d_6, 298 K)

3,3,3-trifluoropropane-1-sulfonohydrazide (S7)

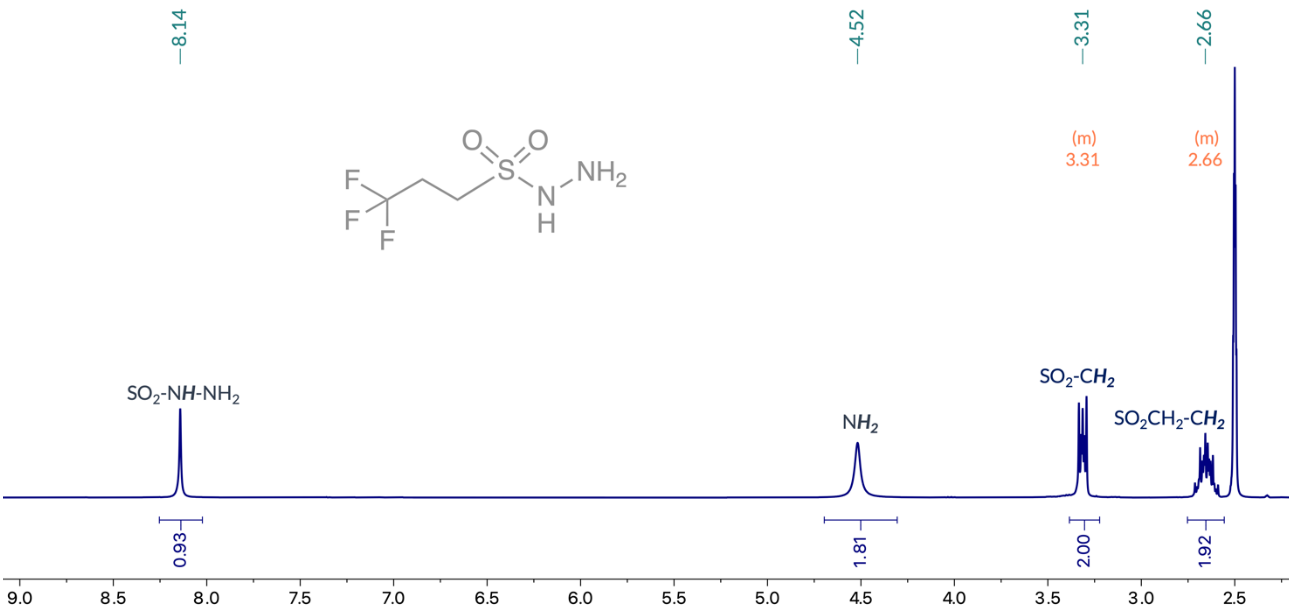

Figure S447. Hydrazide S7:^{1}H NMR (400 MHz, DMSO-d_6, 298 K)

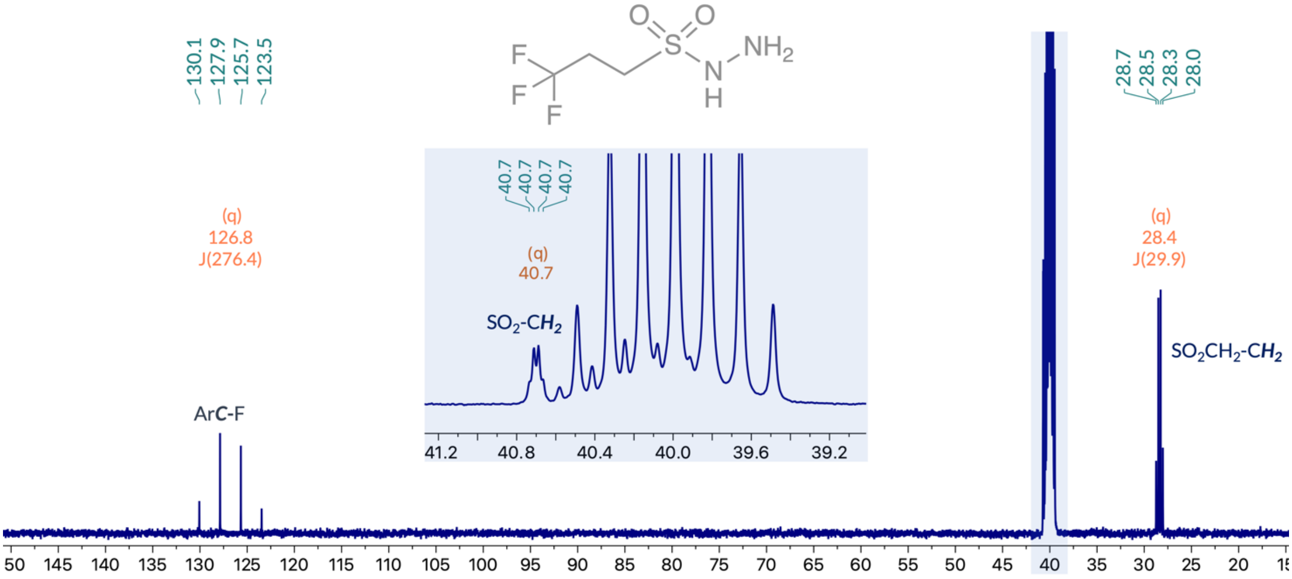

Figure S448. Hydrazide S7:^{13}C NMR (126 MHz, DMSO-d_6, 298 K)

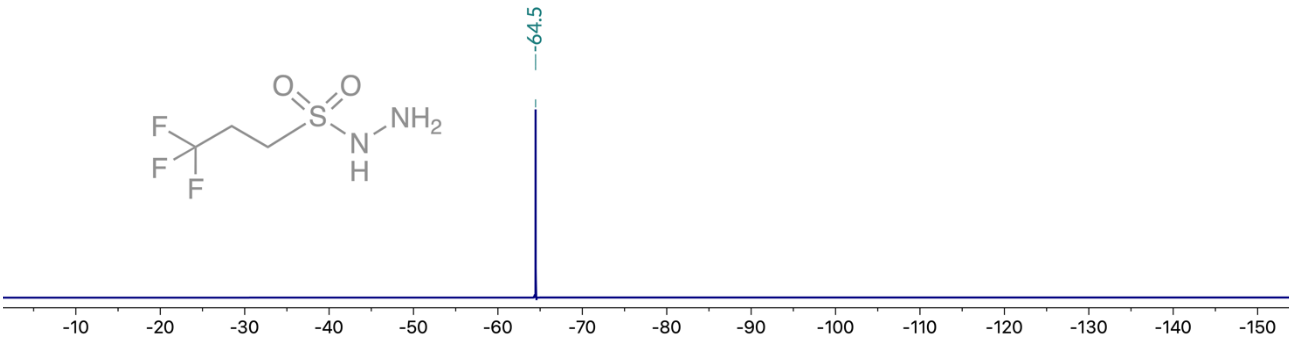

Figure S449. Hydrazide S7:^{19}F NMR (377 MHz, DMSO-d_6, 298 K)

5-Bromothiophene-2-sulfonohydrazide (S8)

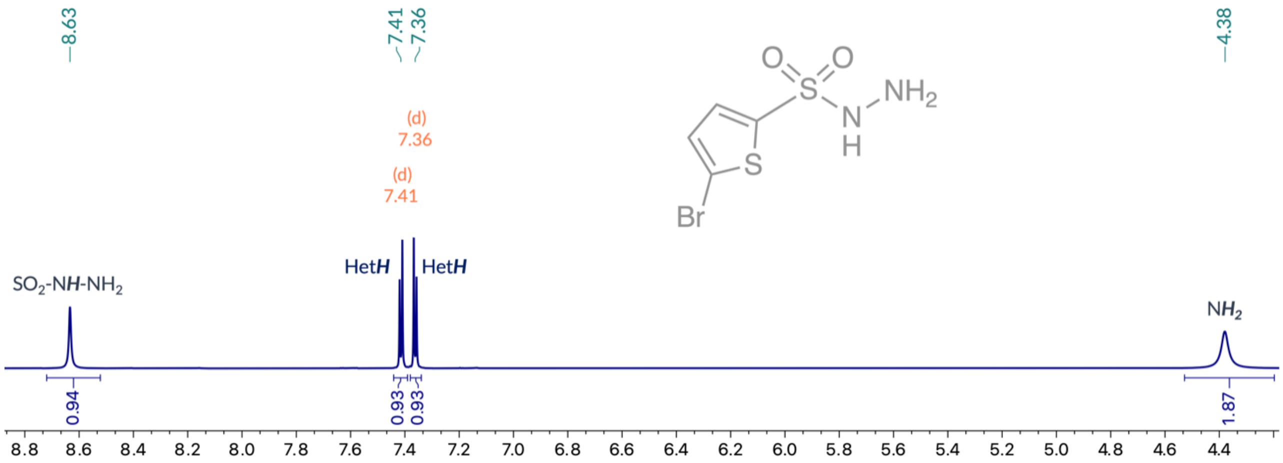

Figure S450. Hydrazide S8:^{1}H NMR (400 MHz, DMSO-d_6, 298 K)

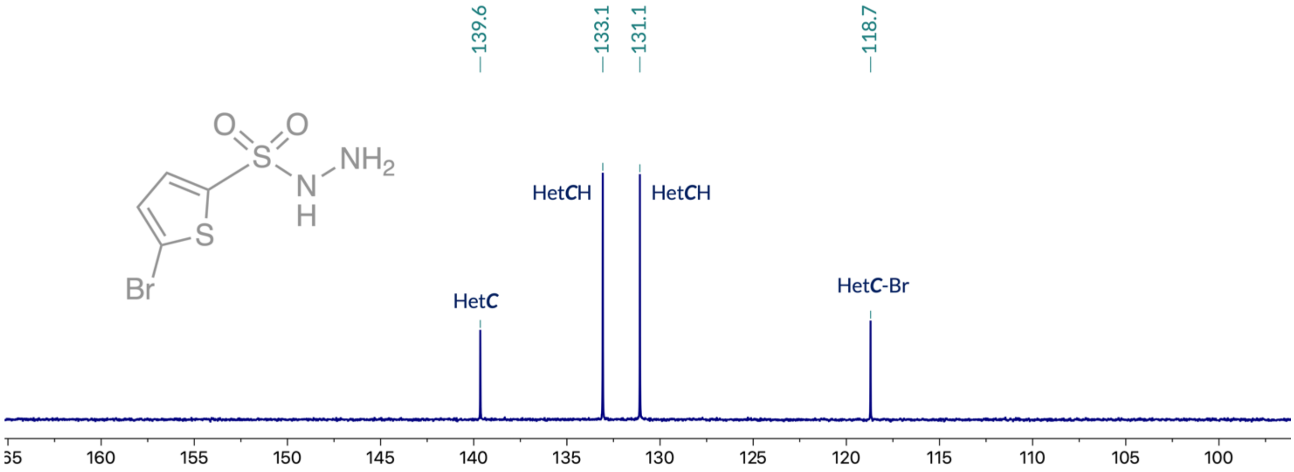

Figure S451. Hydrazide S8:^{13}C NMR (126 MHz, DMSO-d_6, 298 K)

5-Chlorothiophene-2-sulfonohydrazide (S9)

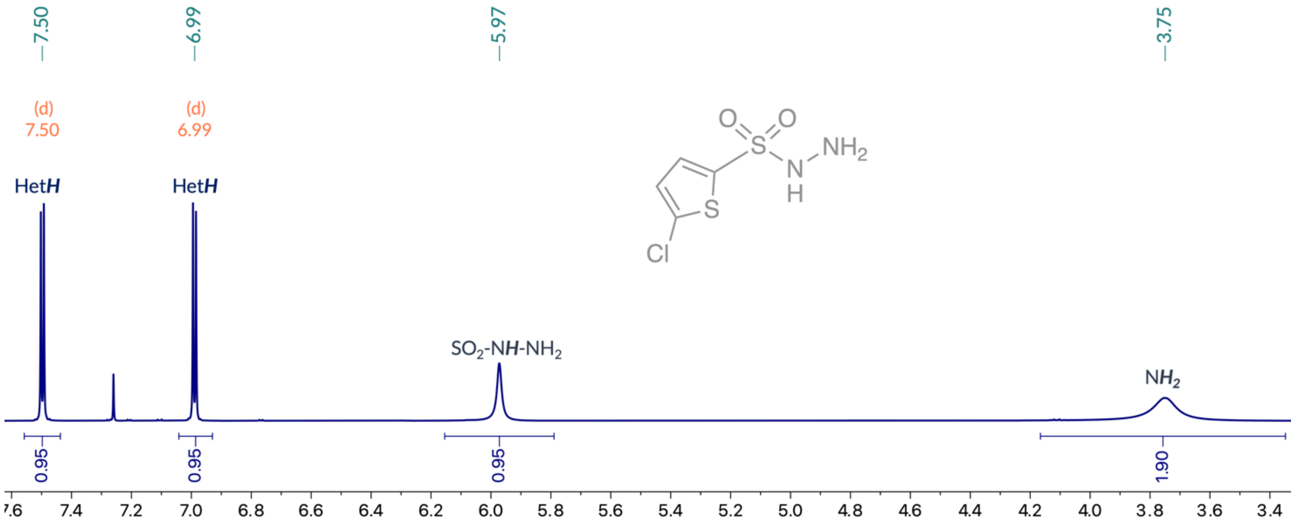

Figure S452. Hydrazide S9:^{1}H NMR (400 MHz, DMSO-d_6, 298 K)

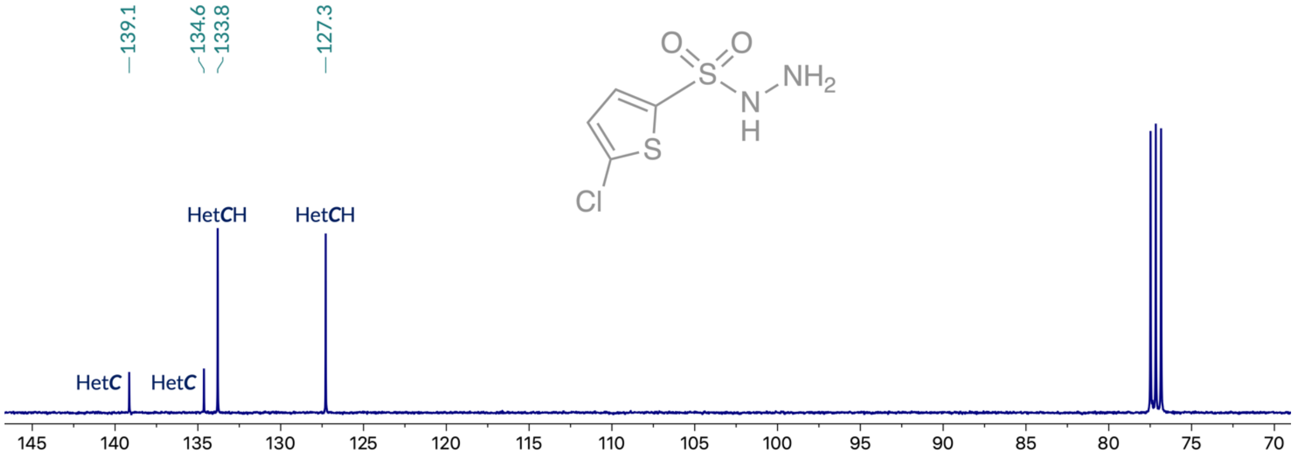

Figure S453. Hydrazide S9:^{13}C NMR (126 MHz, DMSO-d_6, 298 K)

Cyclopropane-1-sulfonyl hydrazide (S10)

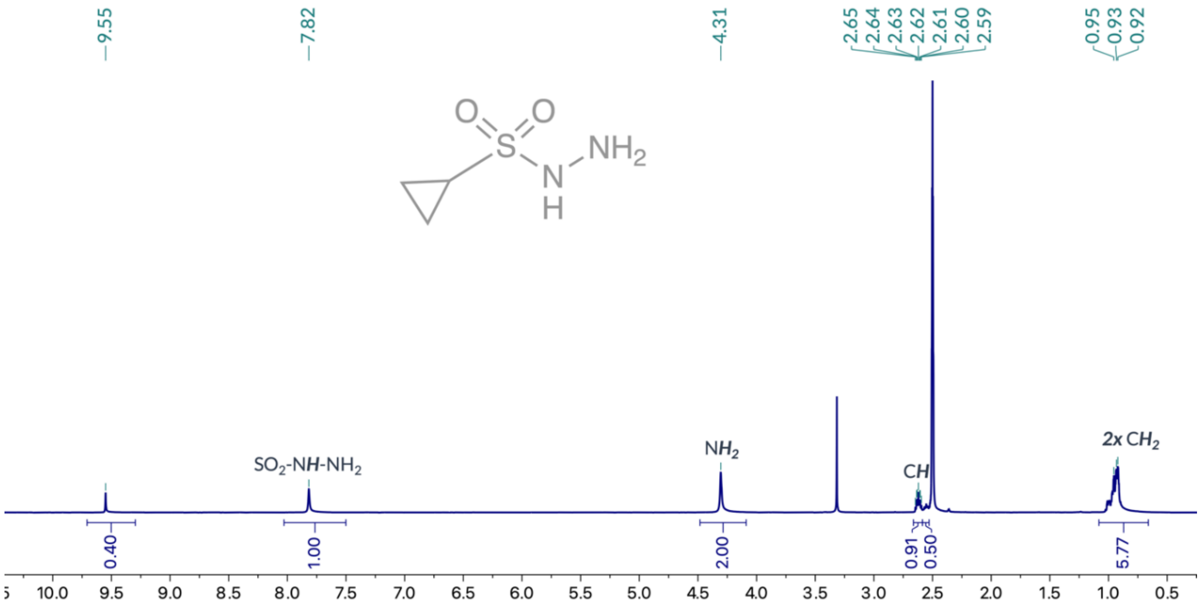

Figure S454. Hydrazine S10:^{1}H NMR (400 MHz, DMSO-d_6, 298 K)

Phosphohydrazidic acid diphenyl ester (S11)

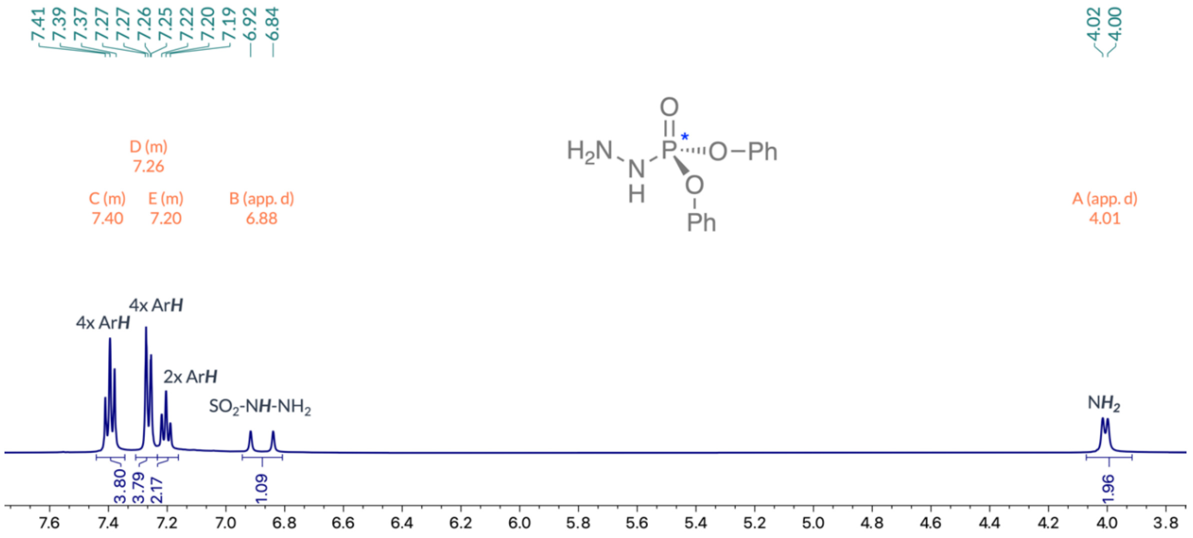

Figure S455. Phosphohydrazide S11:^{1}H NMR (400 MHz, DMSO-d_6, 298 K)

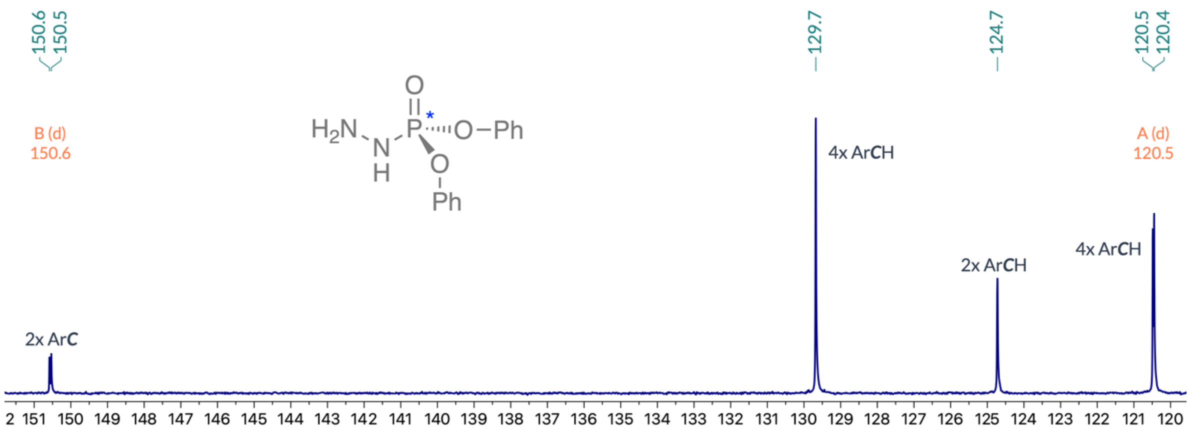

Figure S456. Phosphohydrazide S11:^{1}H NMR (126 MHz, DMSO-d_6, 298 K)

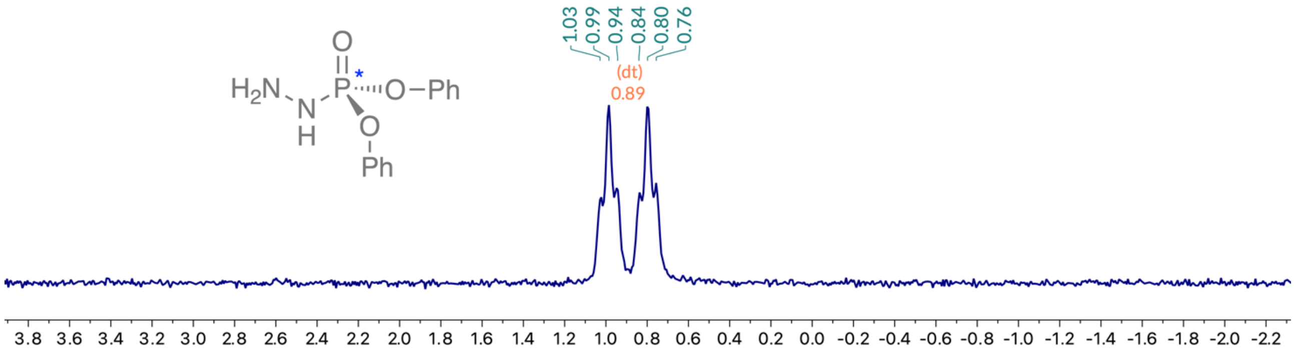

Figure S457. Phosphohydrazide S11: ^{31}P NMR (202 MHz, DMSO-d_6, 298 K)

Phosphohydrazidic acid diethyl ester (S12)

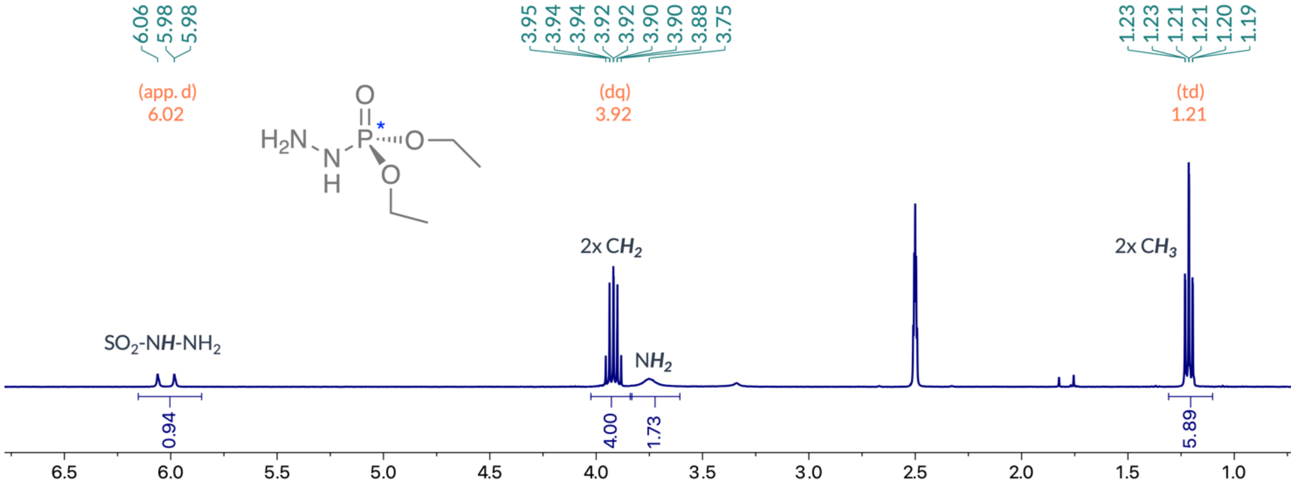

Figure S458. Phosphohydrazide S12: ^{1}H NMR (400 MHz, DMSO-d_6, 298 K)

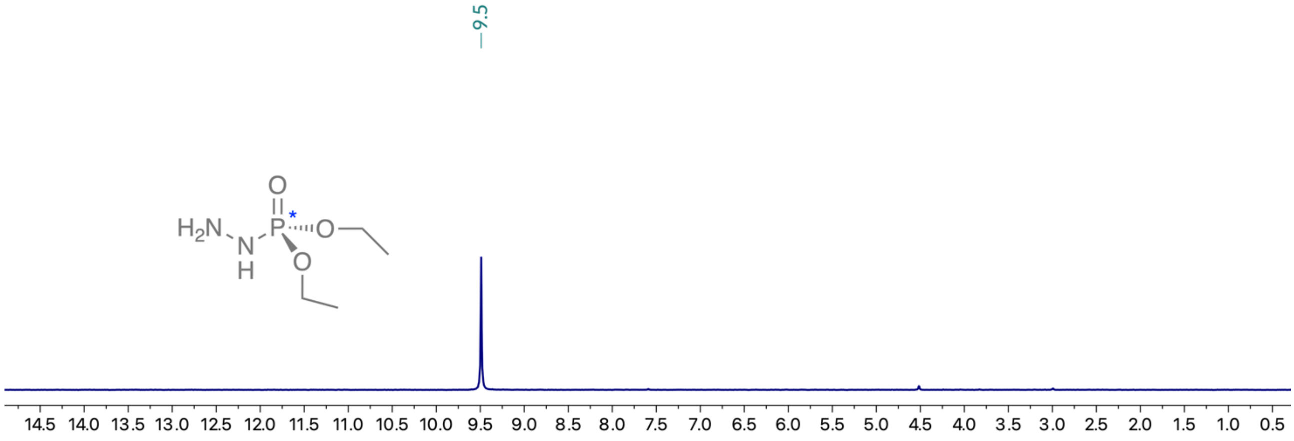

Figure S459. Phosphohydrazide S12: $^{31}P\{^{1}H\}$ NMR (162 MHz, DMSO-d_6, 298 K)

2-Aminonicotinohydrazide (S13)

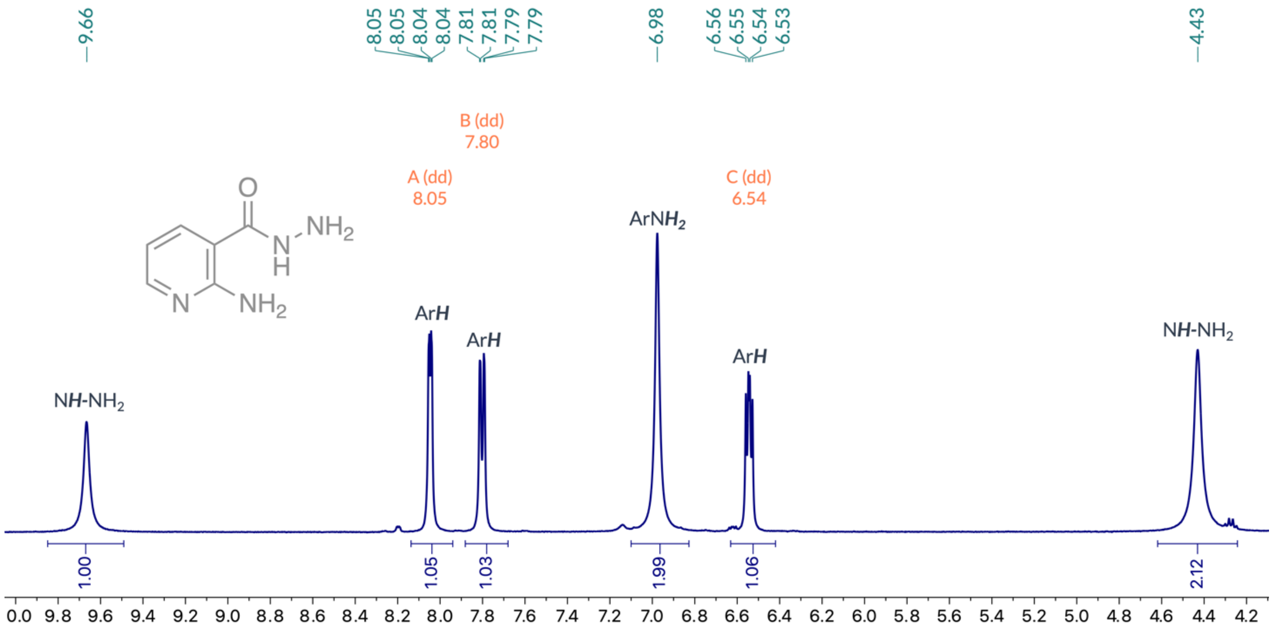

Figure S460. S13: ^{1}H NMR (400 MHz, DMSO-d_6, 298 K)

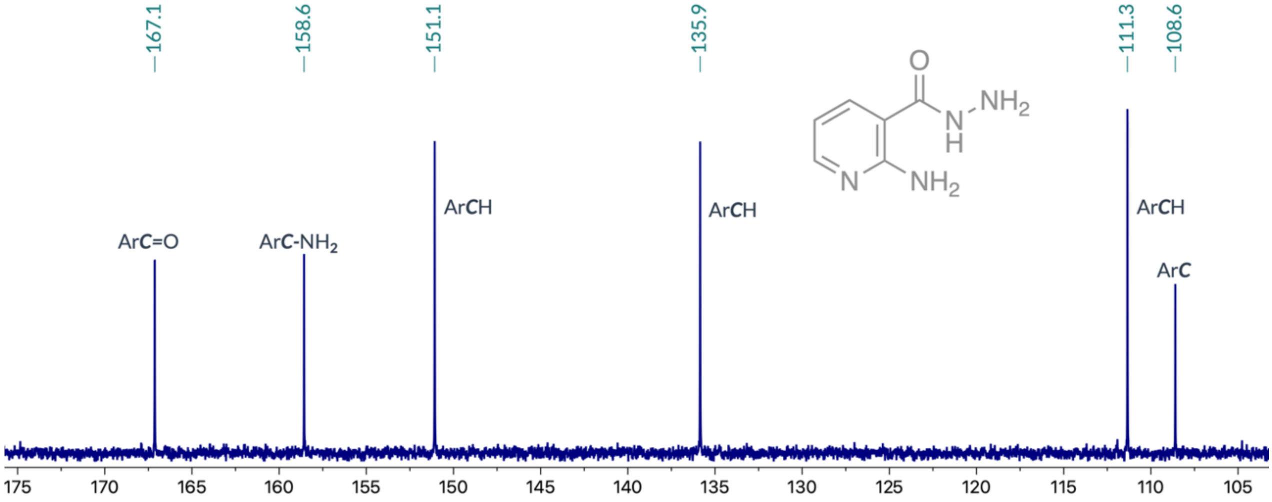

Figure S461. S13: ^{13}C NMR (101 MHz, DMSO-d_6, 298 K)

Hydrazones

Hydrazone S14

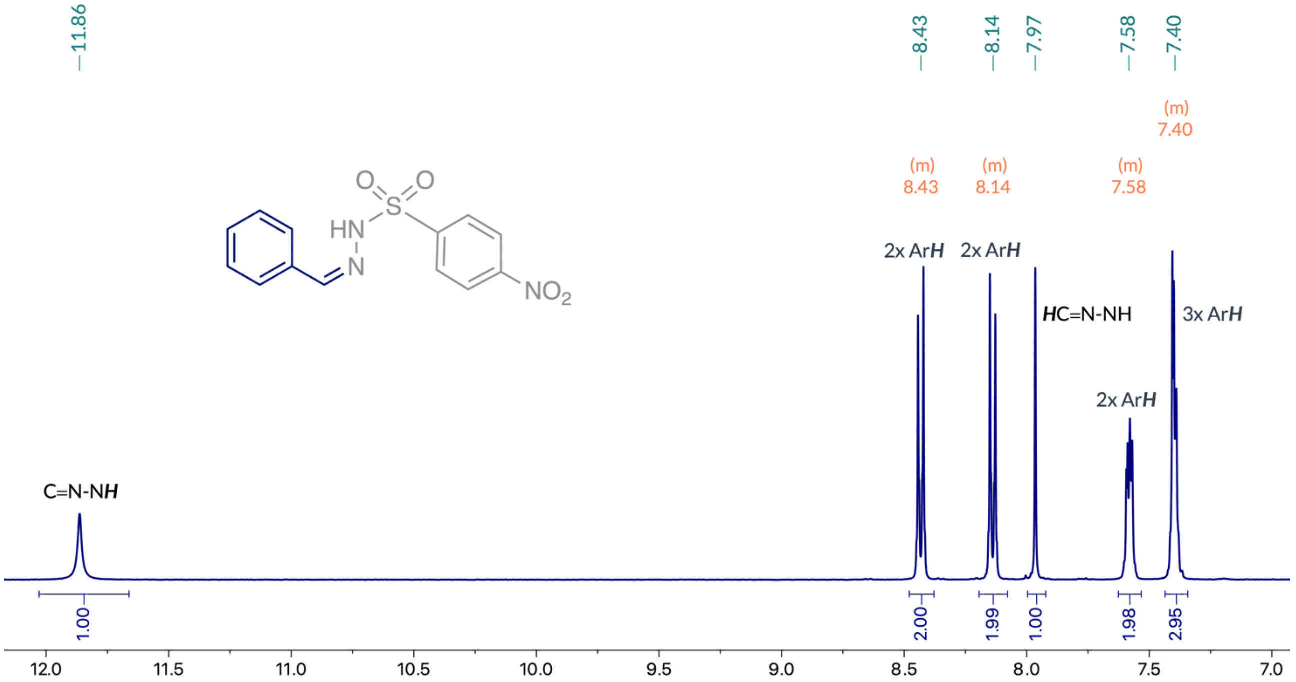

Figure S462. Hydrazone S14: ^{1}H NMR (400 MHz, DMSO-d_6, 298 K)

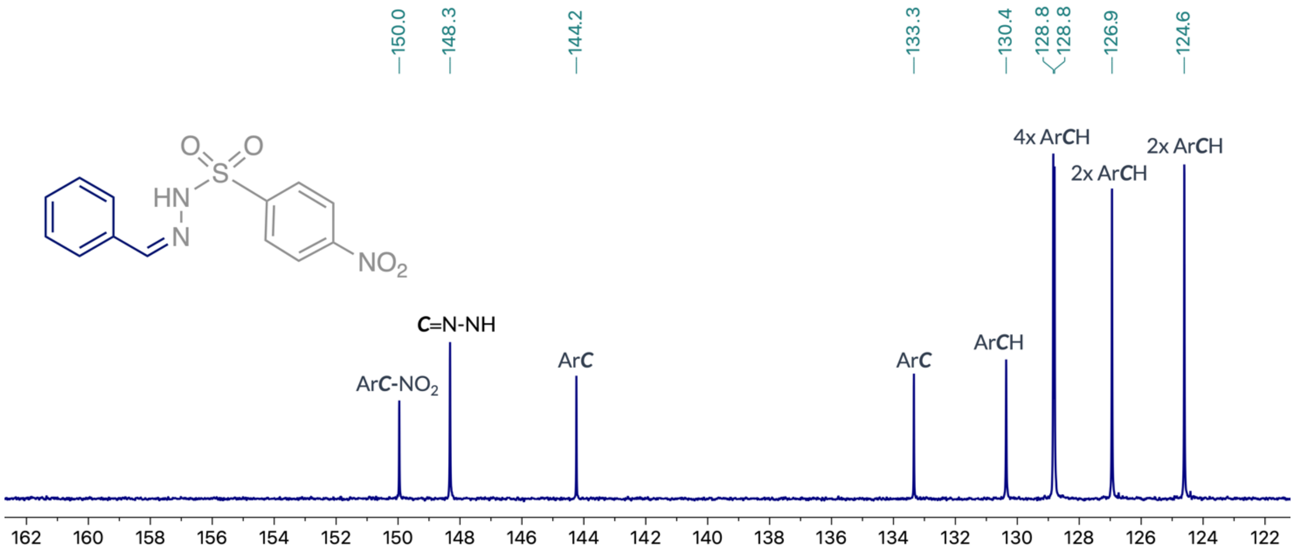

Figure S463. Hydrazone S14: ^{13}C NMR (101 MHz, DMSO-d_6, 298 K)

Hydrazone S15

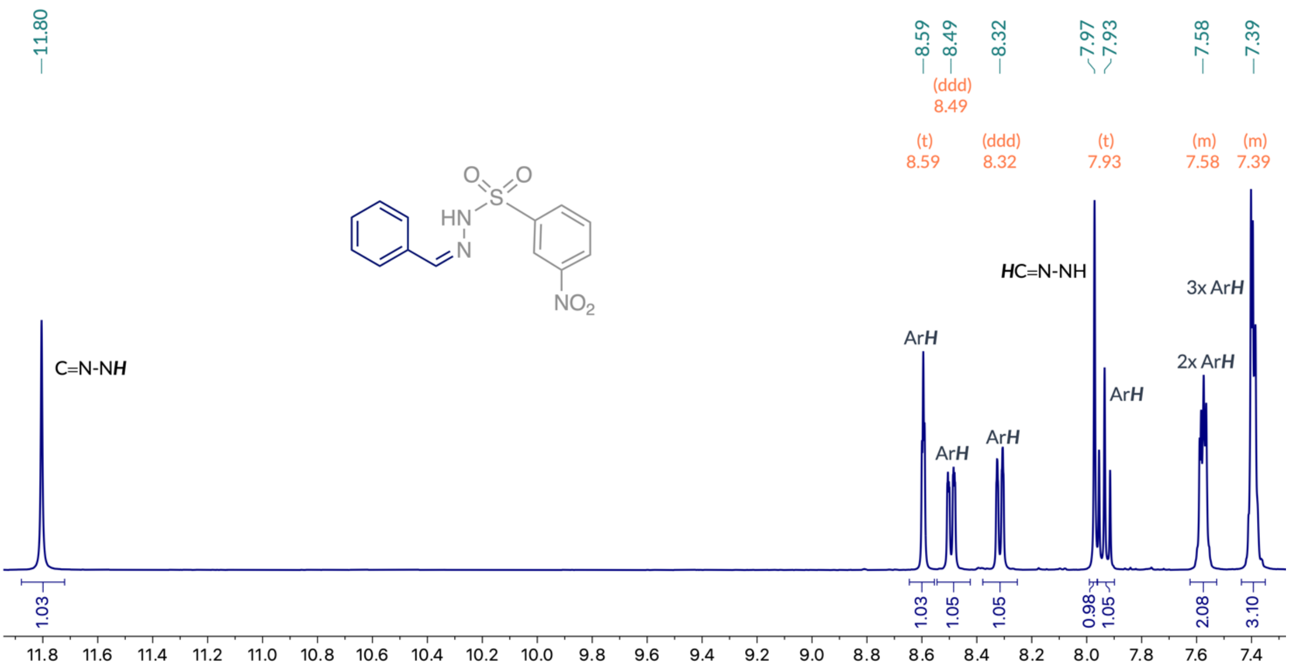

Figure S464. Hydrazone S15: ^{1}H NMR (400 MHz, DMSO-d_6, 298 K)

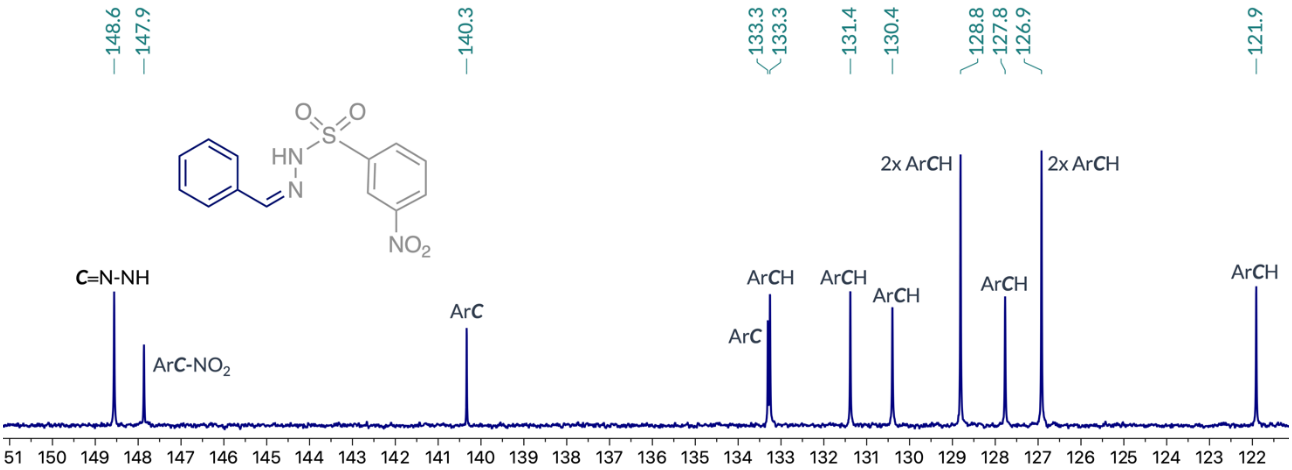

Figure S465. Hydrazone S15: ^{13}C NMR (101 MHz, DMSO-d_6, 298 K)

Hydrazone S16

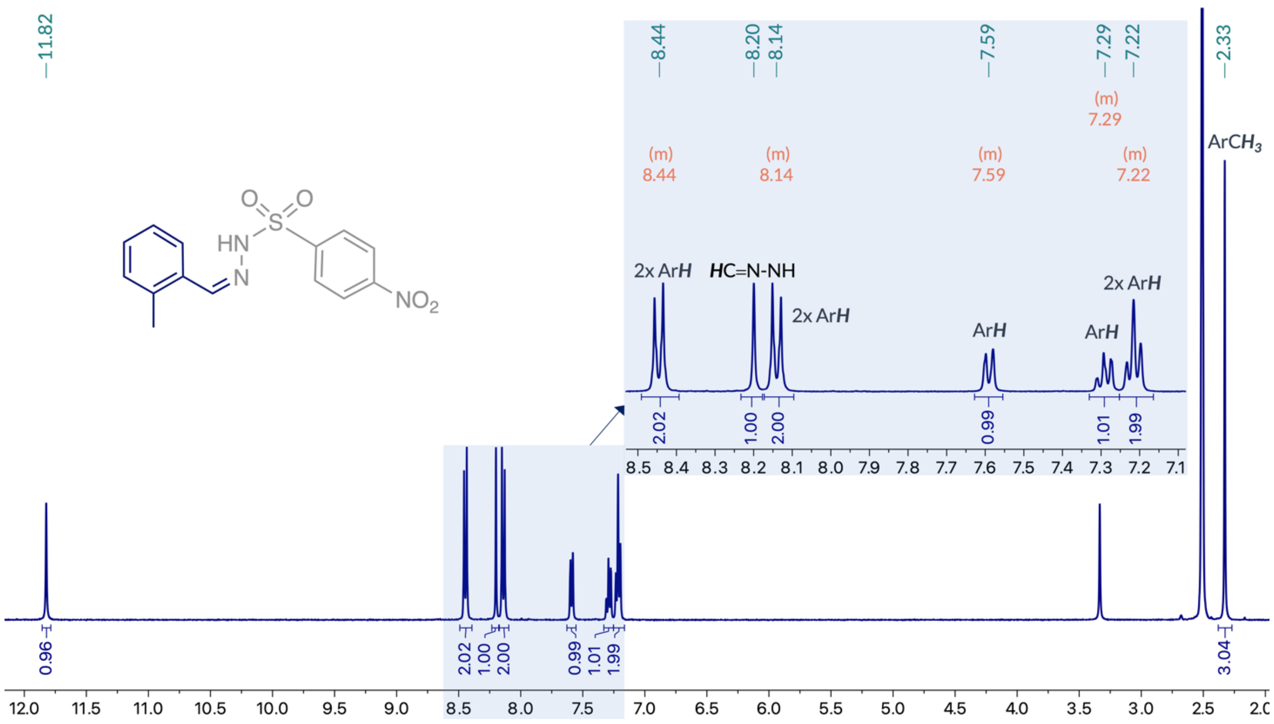

Figure S466. Hydrazone S16: ^{1}H NMR (400 MHz, DMSO-d_6, 298 K)

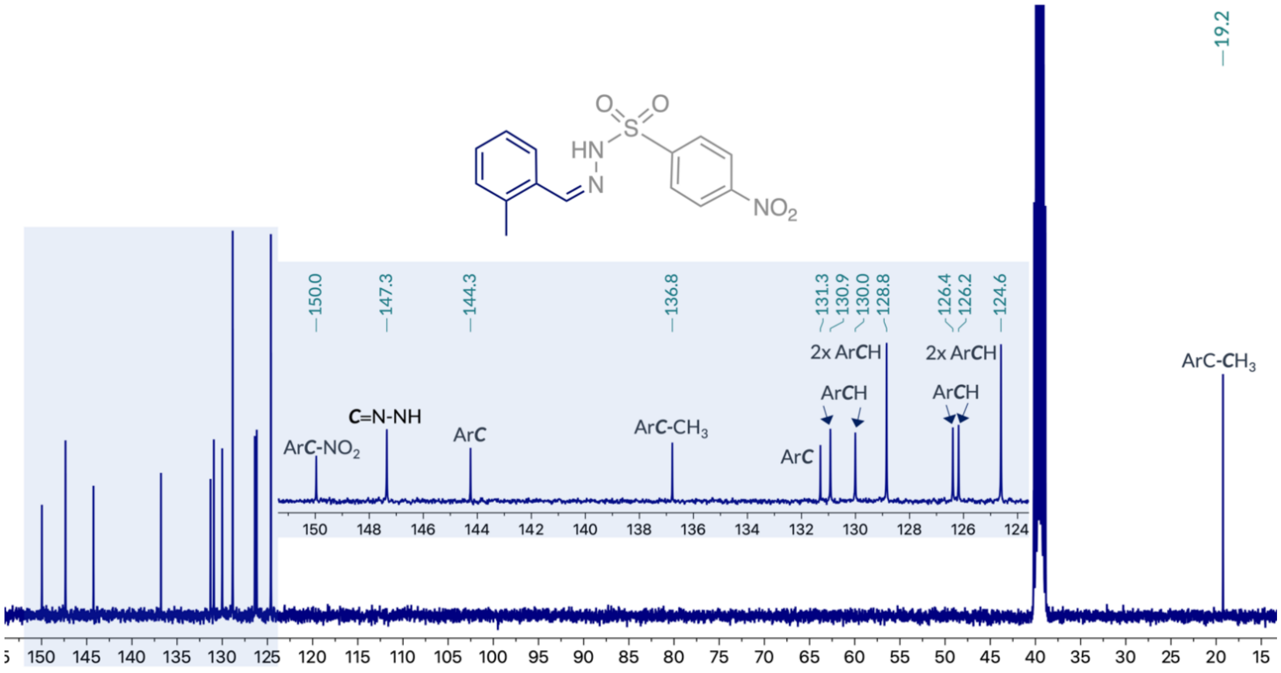

Figure S467. Hydrazone S16: ^{13}C NMR (101 MHz, DMSO-d_6, 298 K)

Hydrazone S17

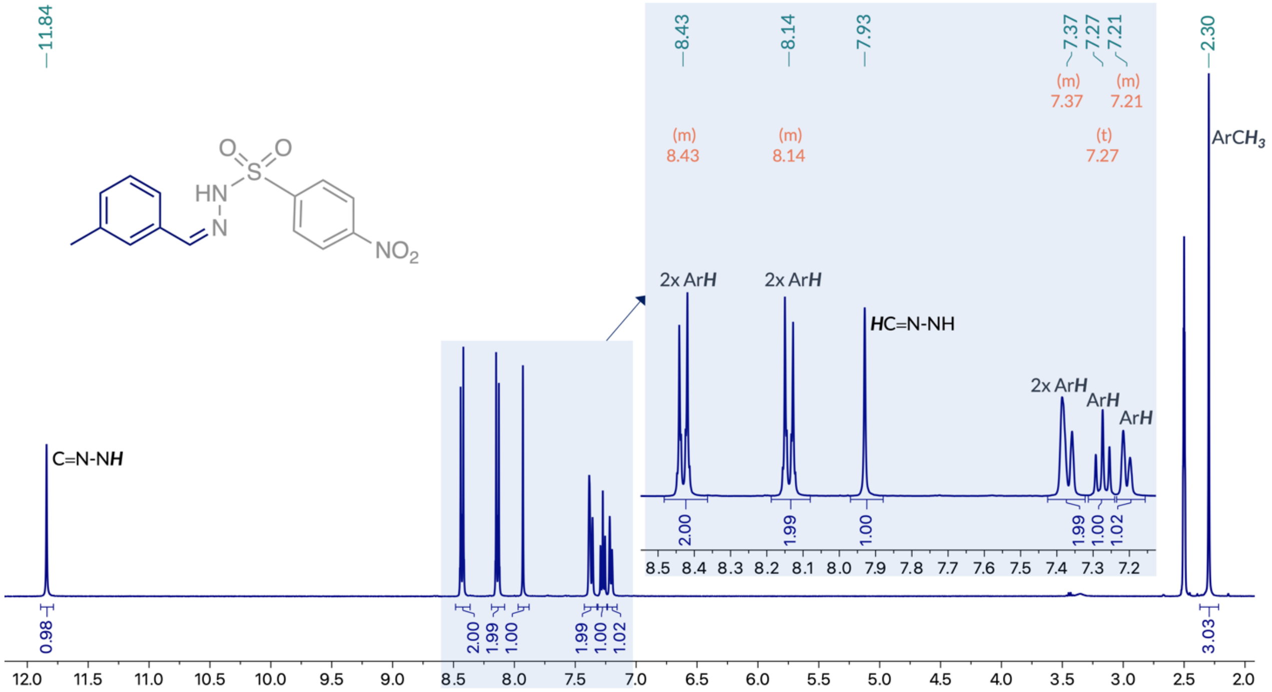

Figure S468. Hydrazone S17: ^{1}H NMR (400 MHz, DMSO-d_6, 298 K)

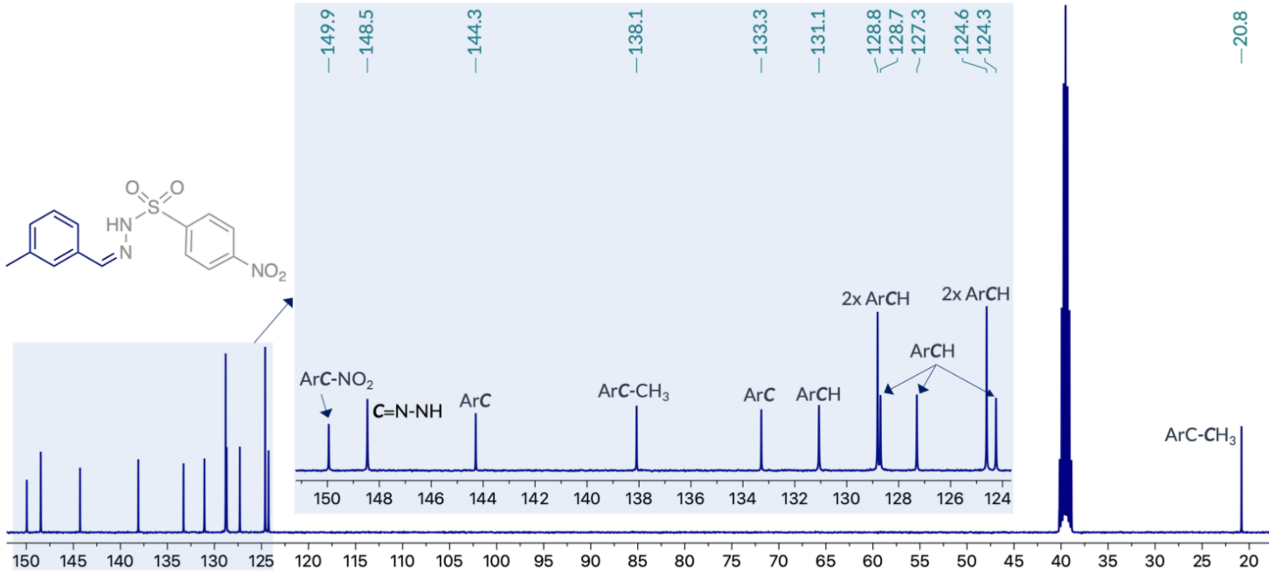

Figure S469. Hydrazone S17: ^{13}C NMR (101 MHz, DMSO-d_6, 298 K)

Hydrazone S18

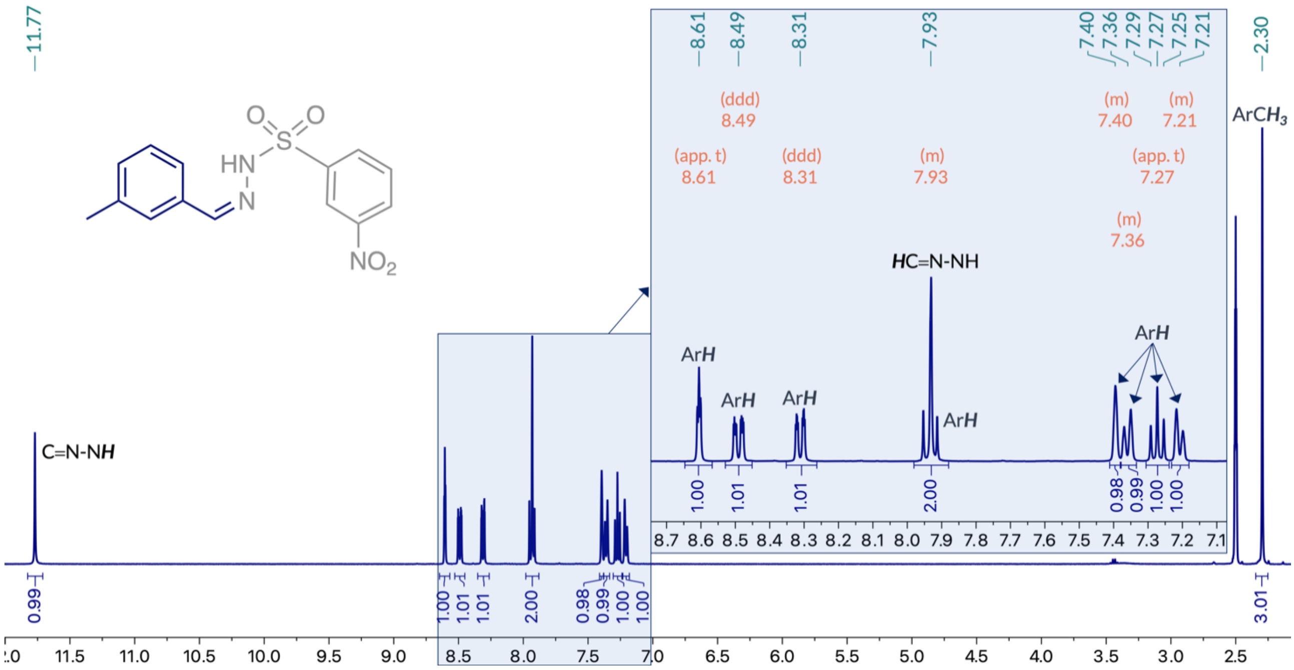

Figure S470. Hydrazone S18: ^{1}H NMR (400 MHz, DMSO-d_6, 298 K)

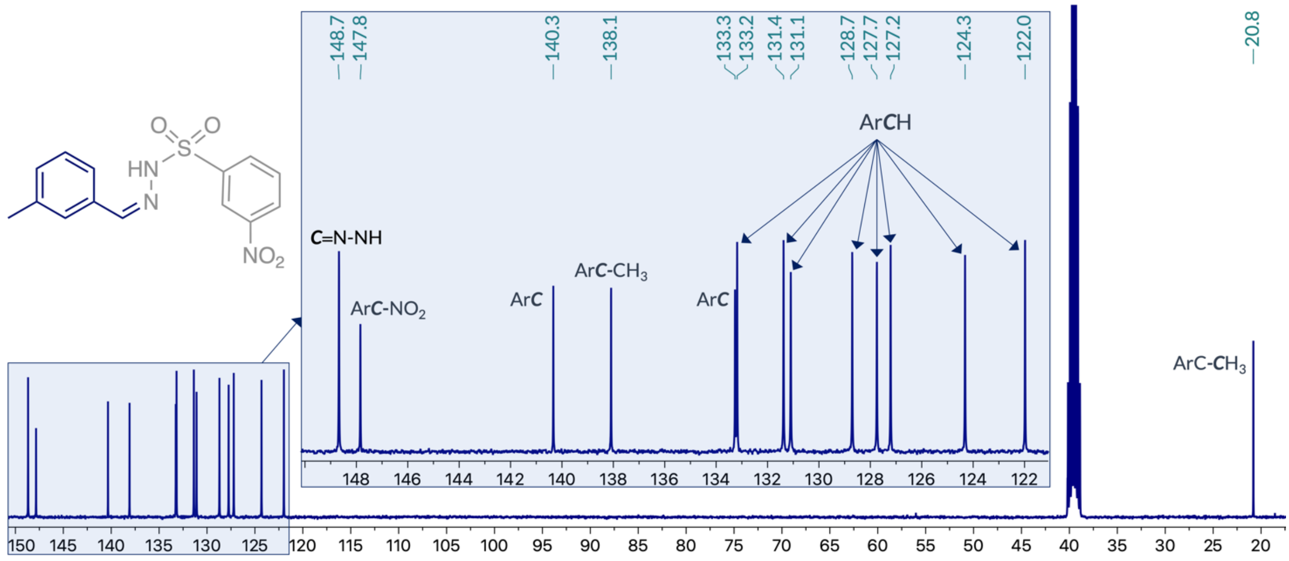

Figure S471. Hydrazone S18: ^{13}C NMR (101 MHz, DMSO-d_6, 298 K)

Hydrazone S19

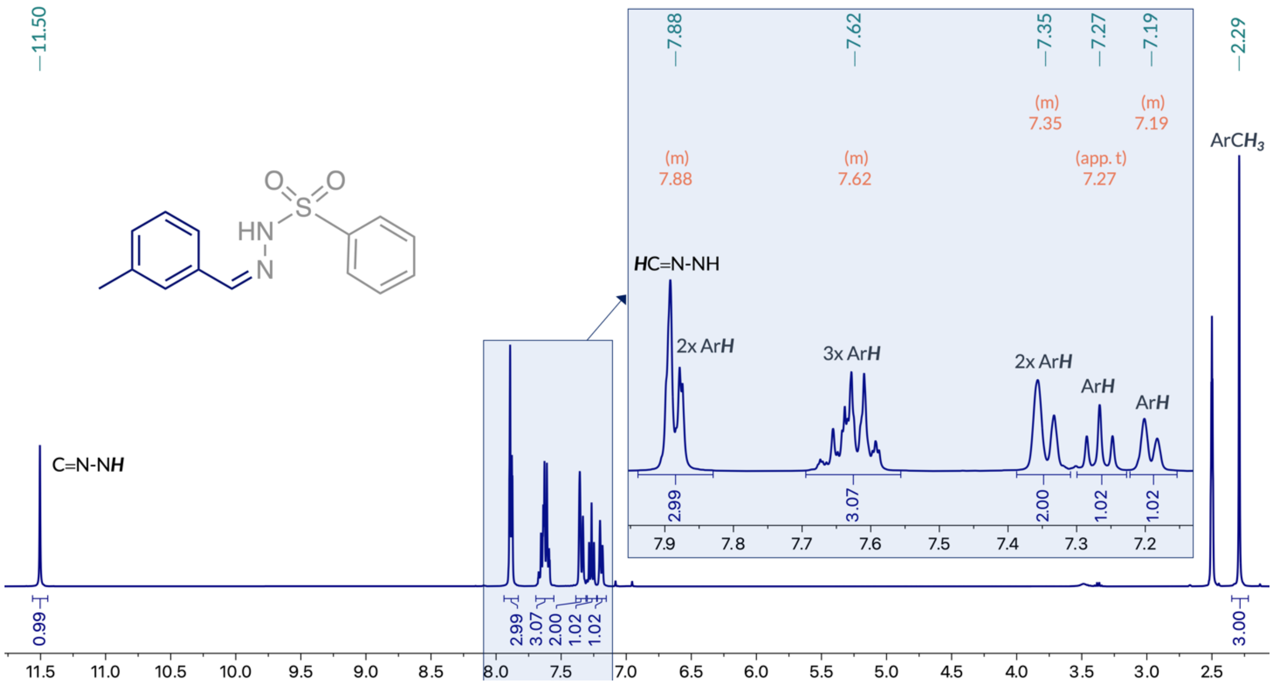

Figure S472. Hydrazone S19: ^{1}H NMR (400 MHz, DMSO-d_6, 298 K)

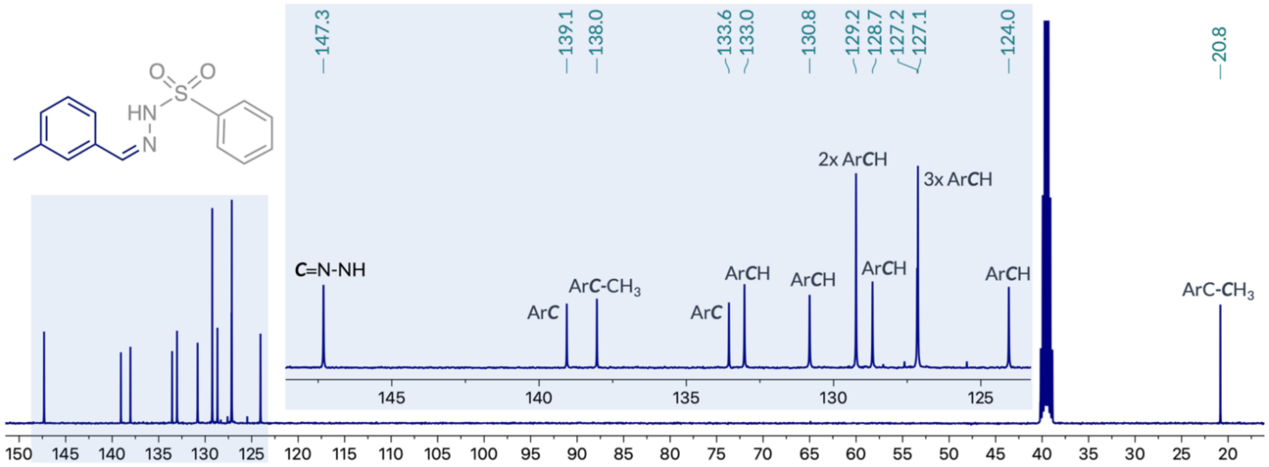

Figure S473. Hydrazone S19: ^{13}C NMR (101 MHz, DMSO-d_6, 298 K)

Hydrazone S20

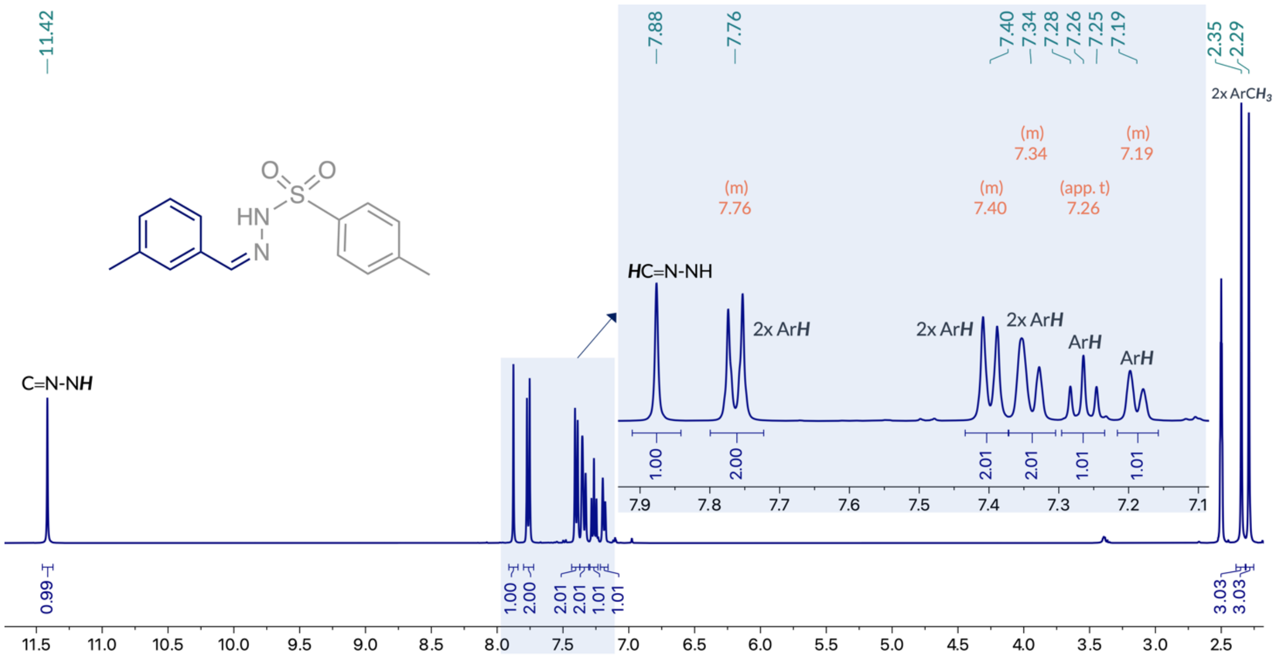

Figure S474. Hydrazone S20: ^{1}H NMR (400 MHz, DMSO-d_6, 298 K)

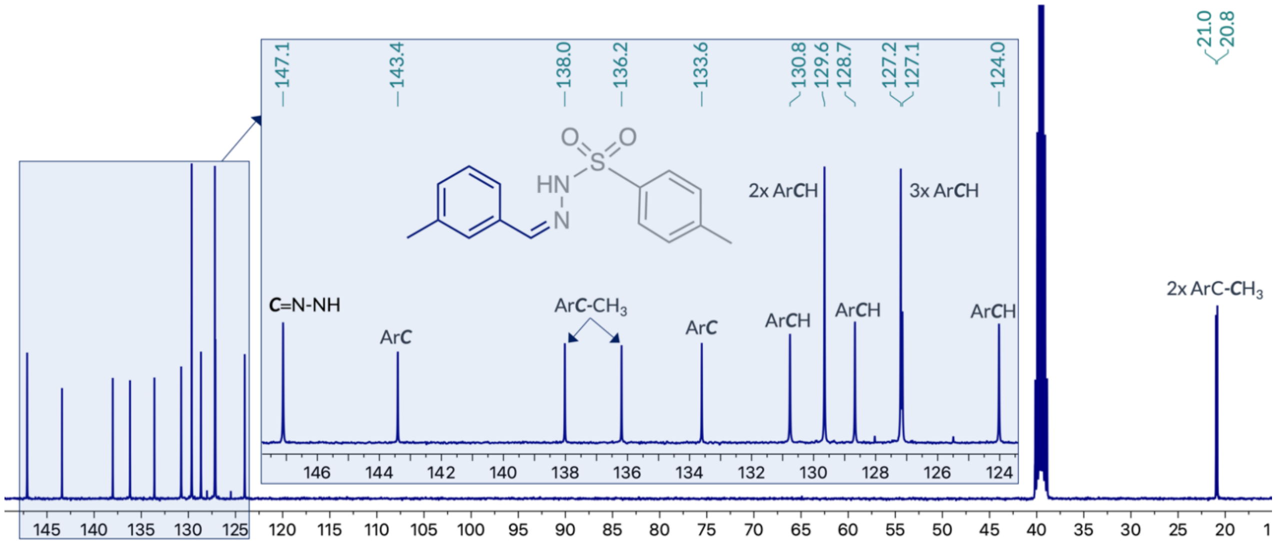

Figure S475. Hydrazone S20: ^{13}C NMR (101 MHz, DMSO-d_6, 298 K)

Hydrazone S21

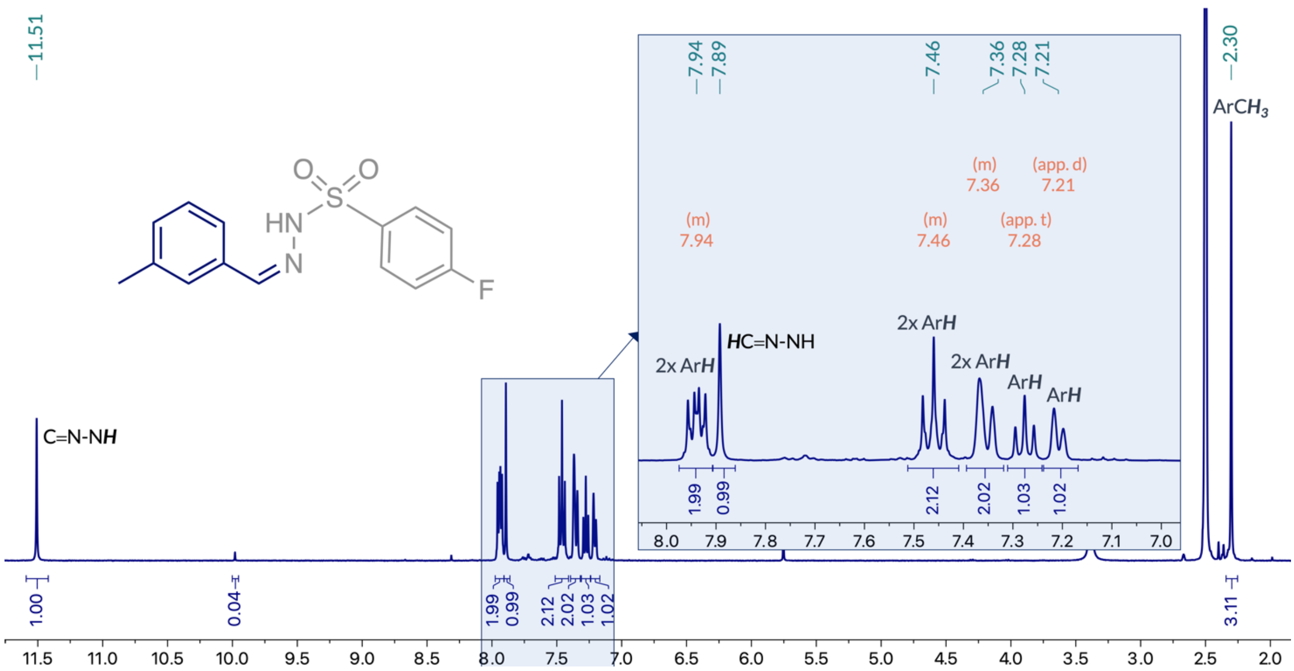

Figure S476. Hydrazone S21: ^{1}H NMR (400 MHz, DMSO-d_6, 298 K)

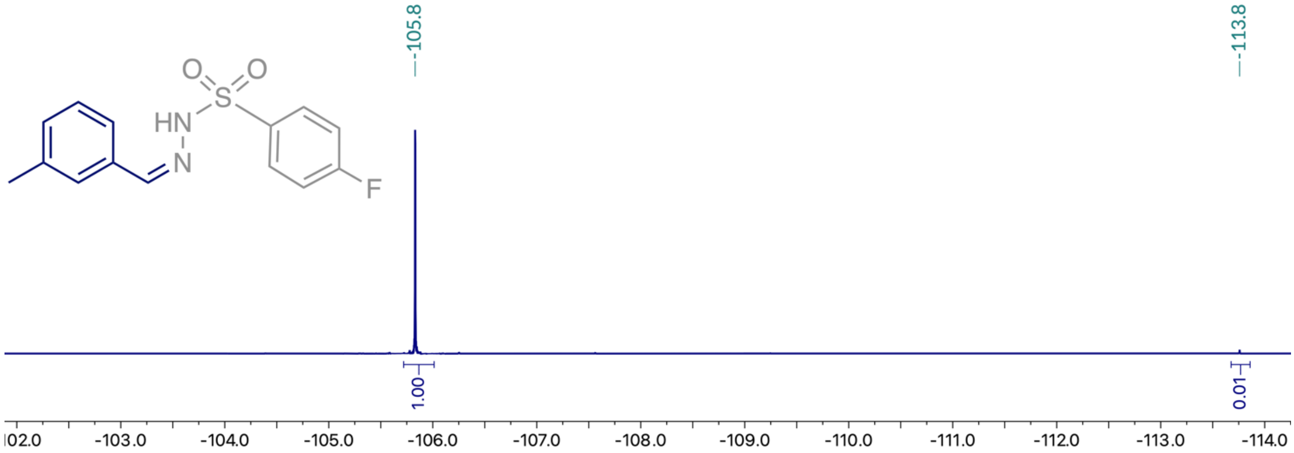

Figure S477. Hydrazone S21: ^{19}F{^{1}H} NMR (377 MHz, DMSO-d_6, 298 K)

Hydrazone S22

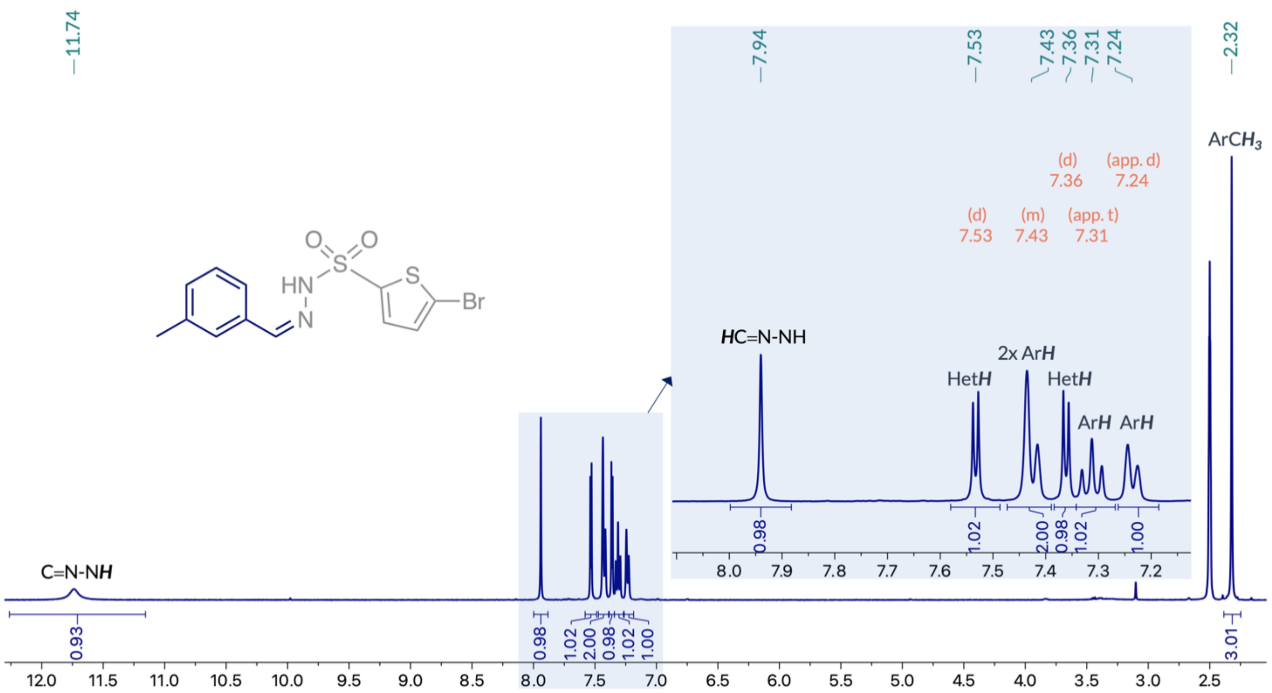

Figure S478. Hydrazone S22: ^{1}H NMR (400 MHz, DMSO-d_6, 298 K)

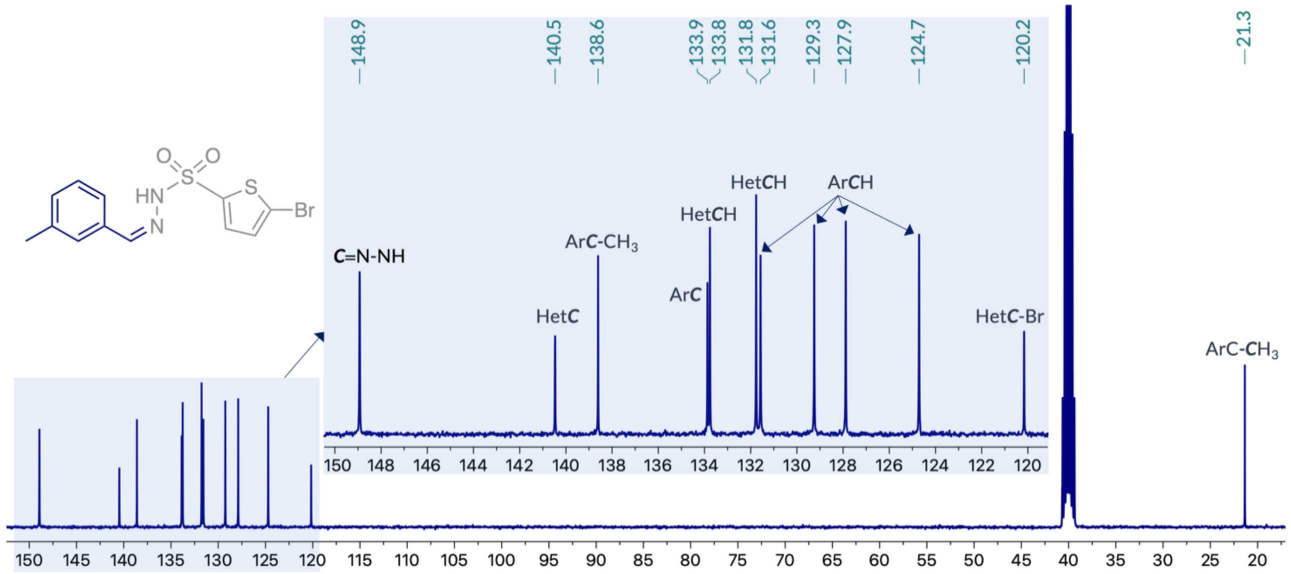

Figure S479. Hydrazone S22: ^{13}C NMR (101 MHz, DMSO-d_6, 298 K)

Hydrazone S23

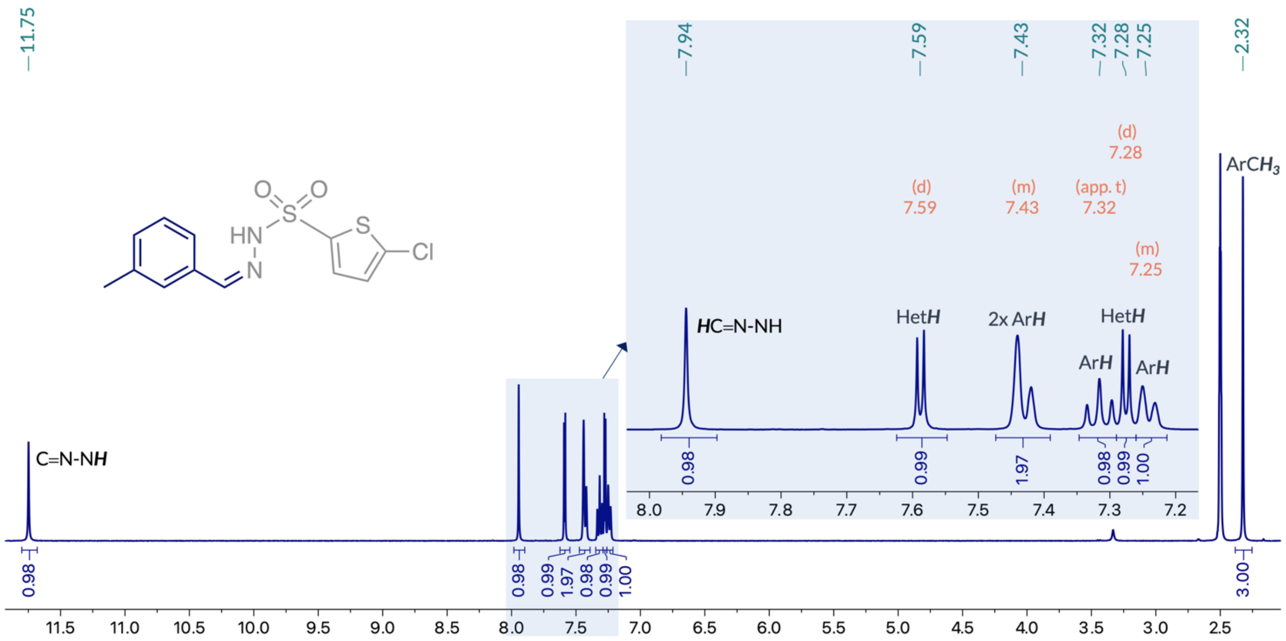

Figure S480. Hydrazone S23: ^{1}H NMR (400 MHz, DMSO-d_6, 298 K)

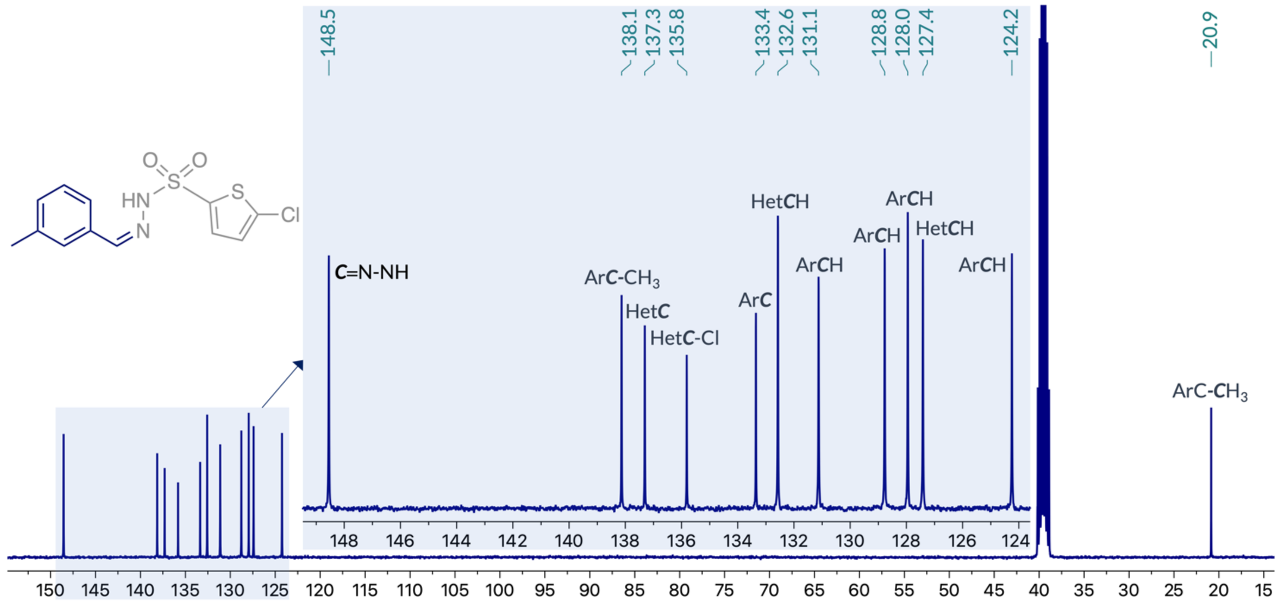

Figure S481. Hydrazone S23: ^{13}C NMR (101 MHz, DMSO-d_6, 298 K)

Hydrazone S24

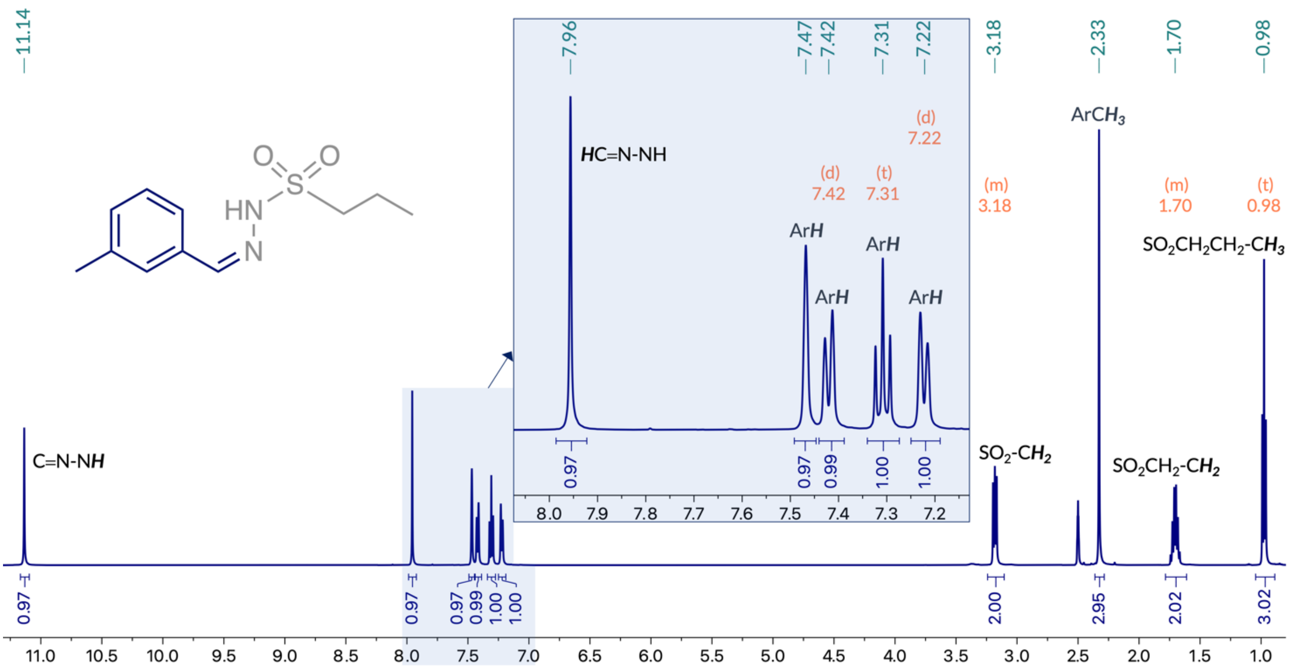

Figure S482. Hydrazone S24: ^{1}H NMR (500 MHz, DMSO-d_6, 298 K)

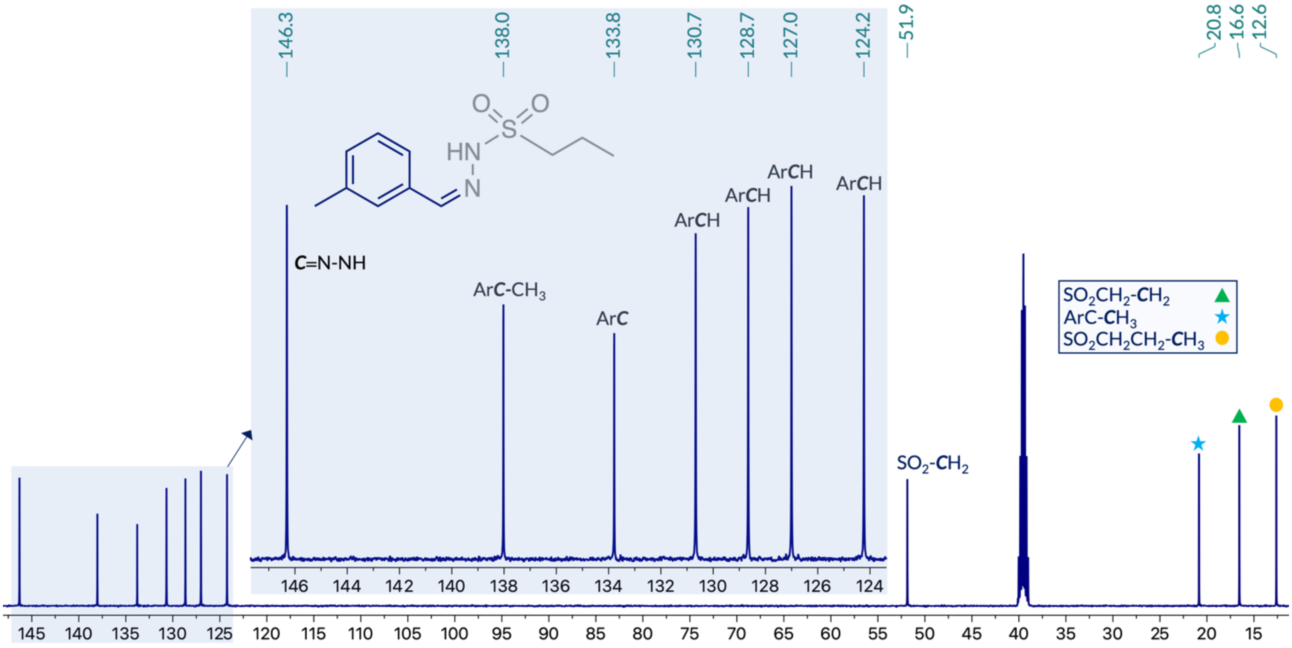

Figure S483. Hydrazone S24: ^{13}C NMR (126 MHz, DMSO-d_6, 298 K)

Hydrazone S25

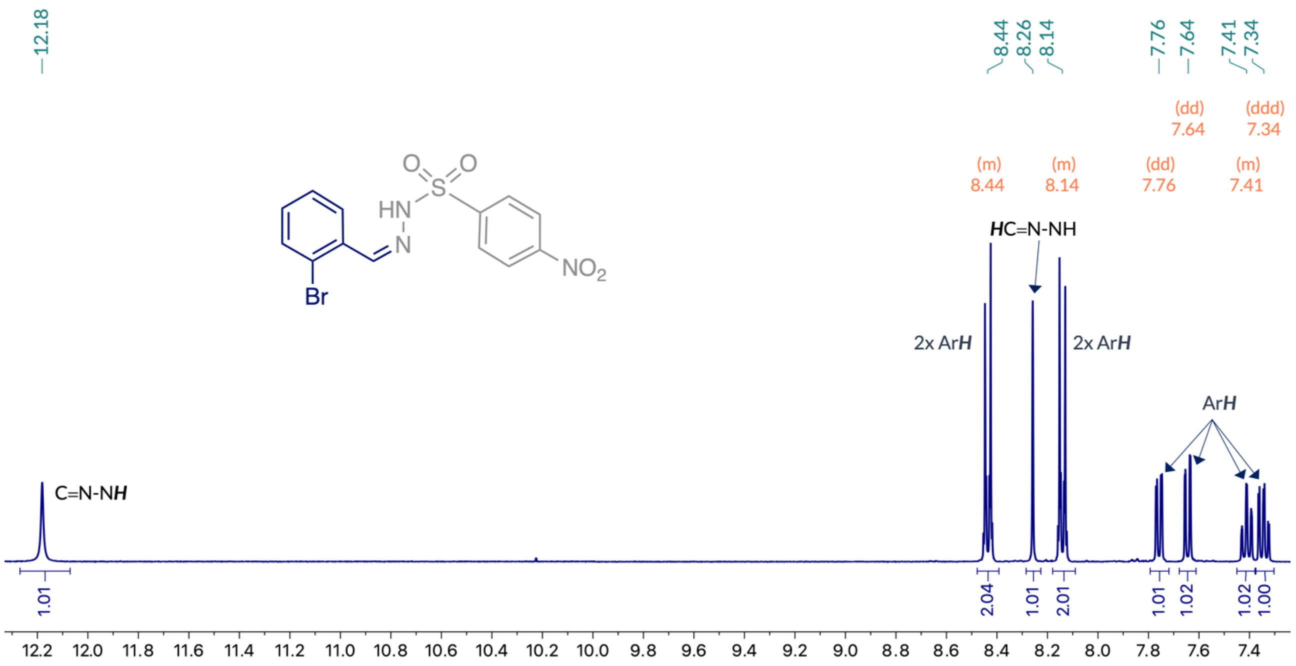

Figure S484. Hydrazone S25: ^{1}H NMR (400 MHz, DMSO-d_6, 298 K)

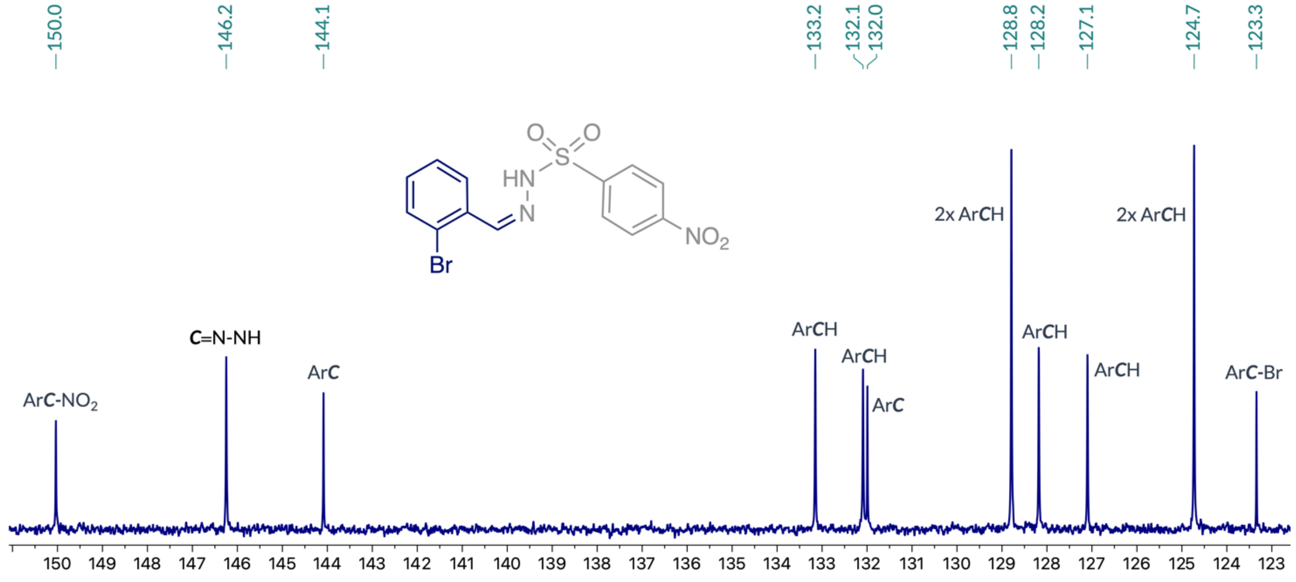

Figure S485. Hydrazone S25: ^{13}C NMR (101 MHz, DMSO-d_6, 298 K)

Hydrazone S26

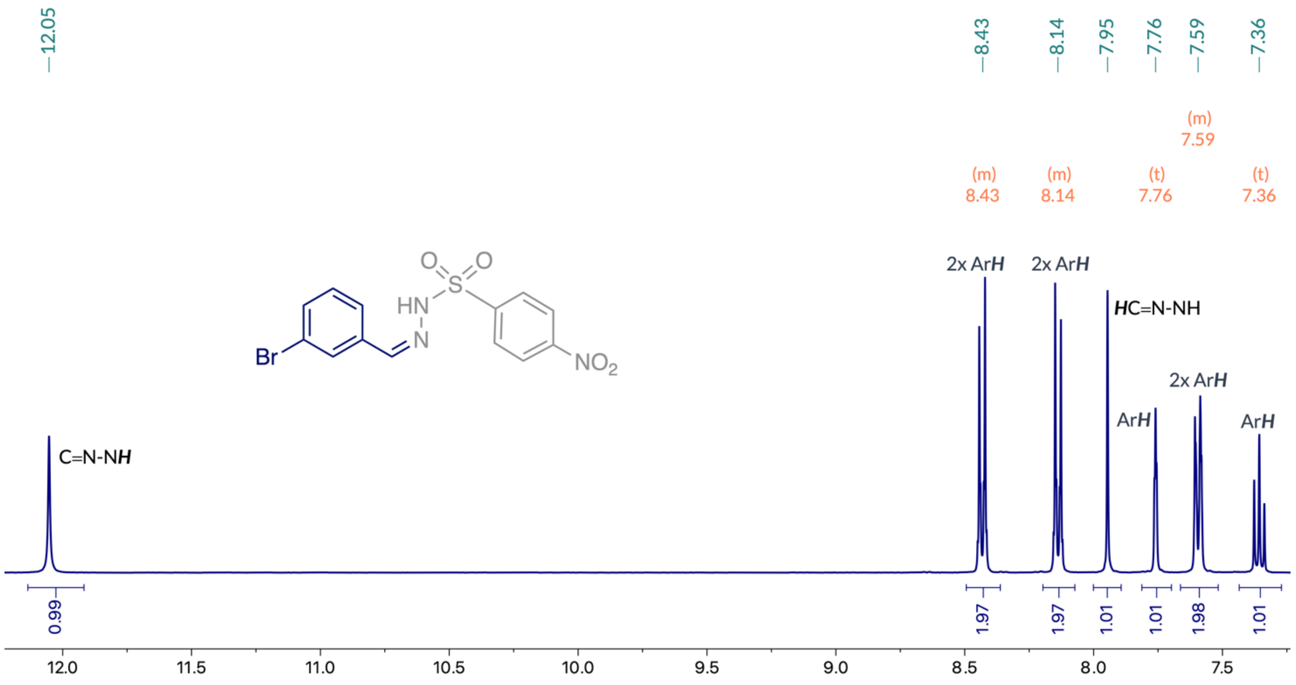

Figure S486. Hydrazone S26: ^{1}H NMR (400 MHz, DMSO-d_6, 298 K)

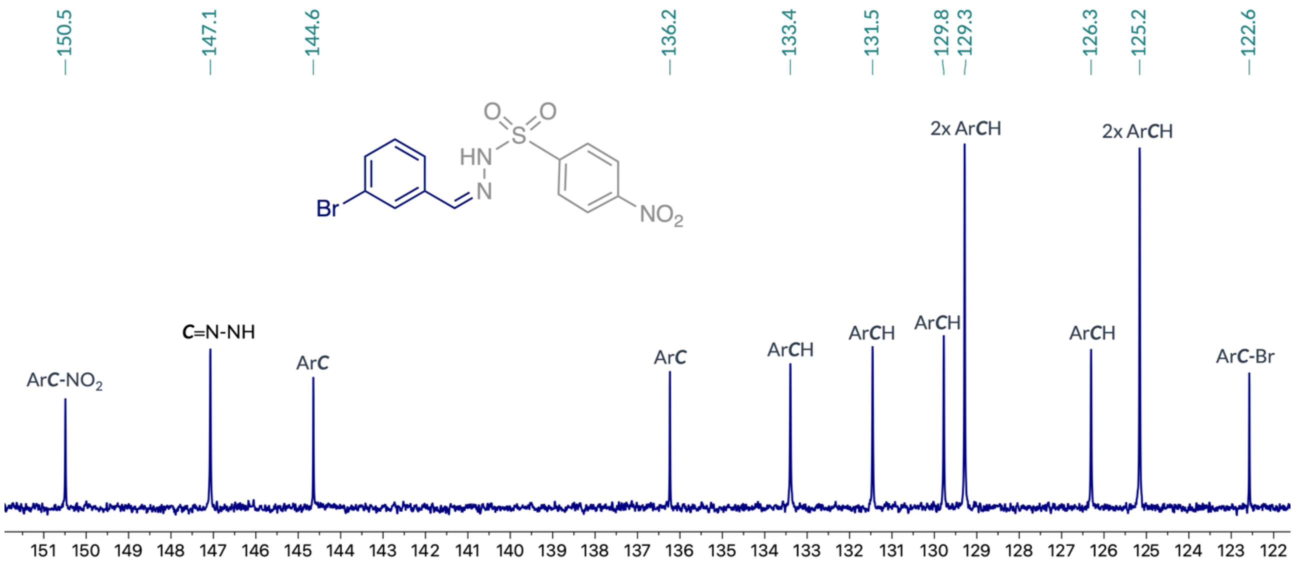

Figure S487. Hydrazone S26: ^{13}C NMR (101 MHz, DMSO-d_6, 298 K)

Hydrazone S27

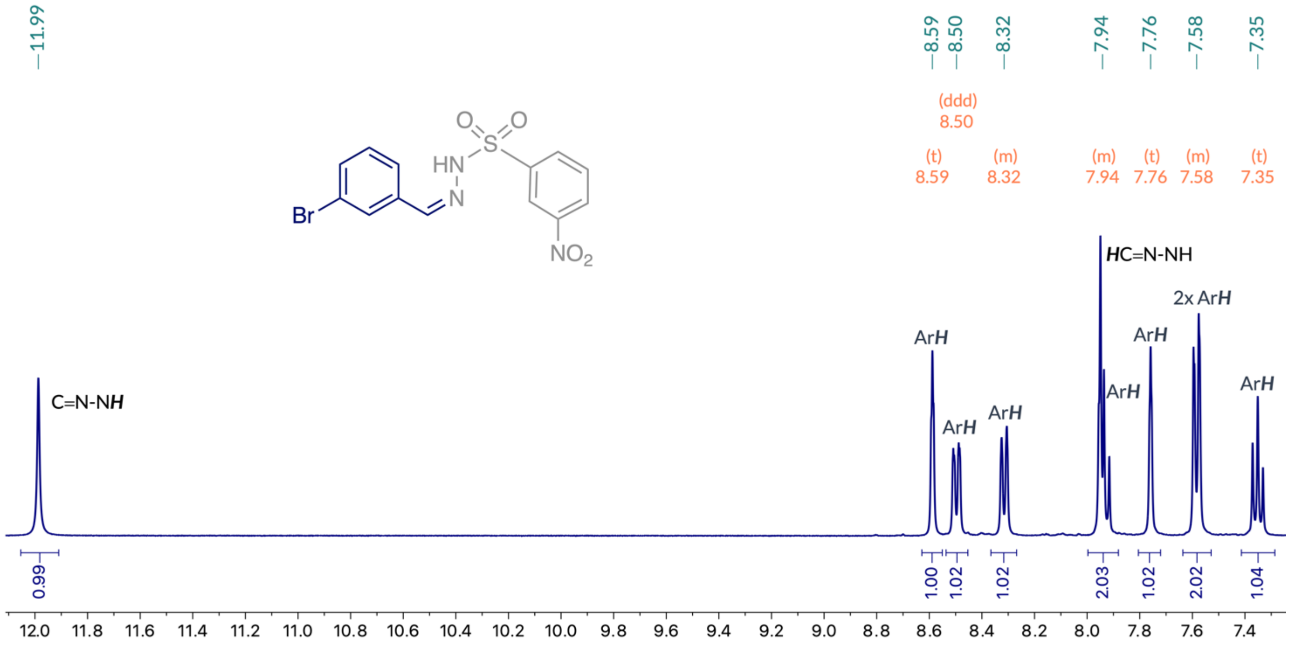

Figure S488. Hydrazone S27: ^{1}H NMR (400 MHz, DMSO-d_6, 298 K)

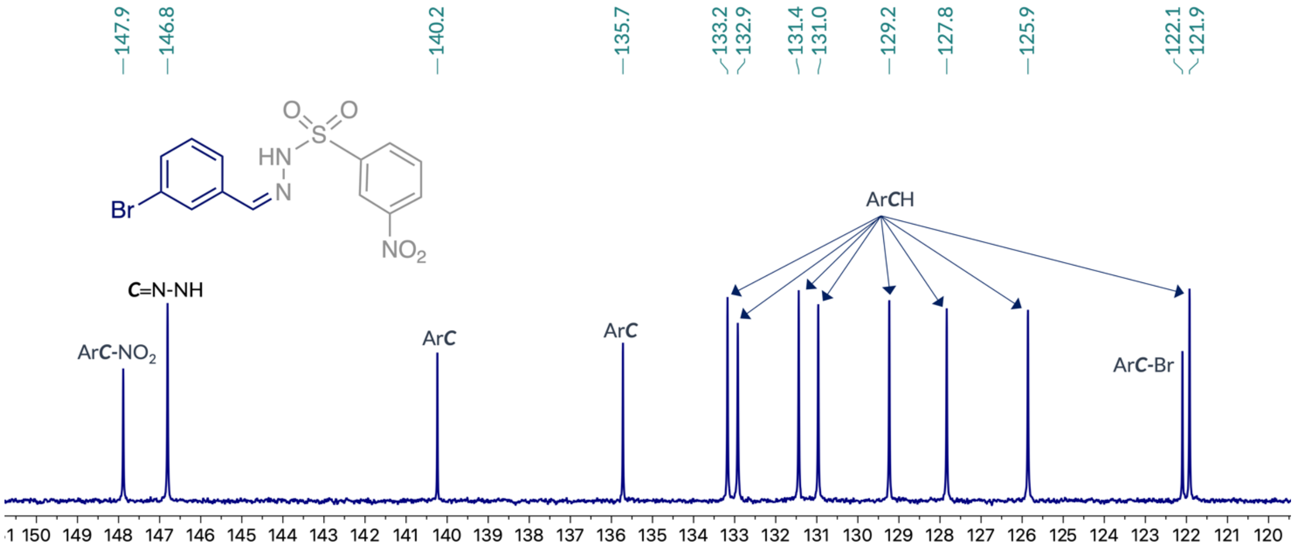

Figure S489. Hydrazone S27: ^{13}C NMR (101 MHz, DMSO-d_6, 298 K)

Hydrazone S28

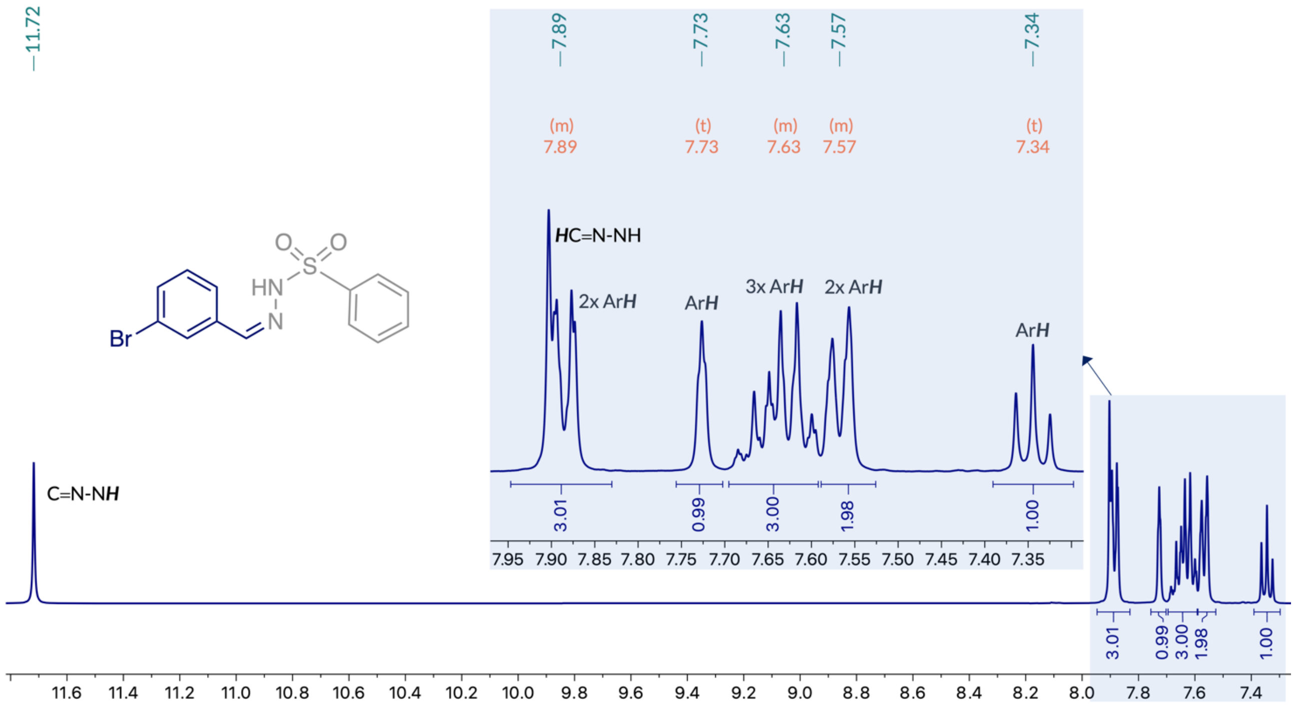

Figure S490. Hydrazone S28: ^{1}H NMR (400 MHz, DMSO-d_6, 298 K)

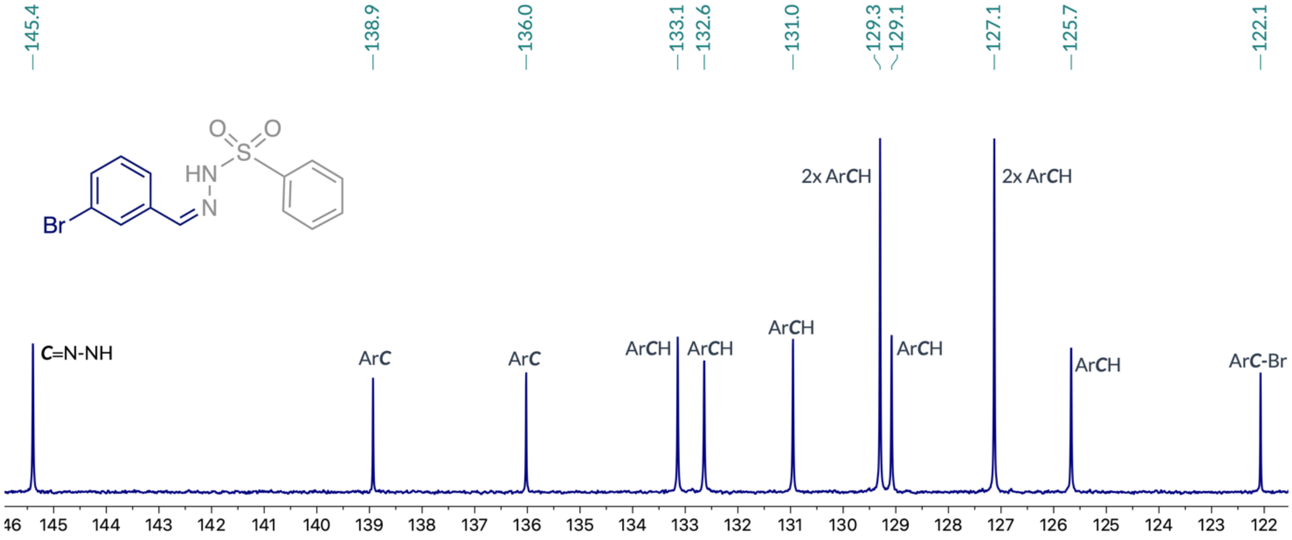

Figure S491. Hydrazone S28: ^{13}C NMR (101 MHz, DMSO-d_6, 298 K)

Hydrazone S29

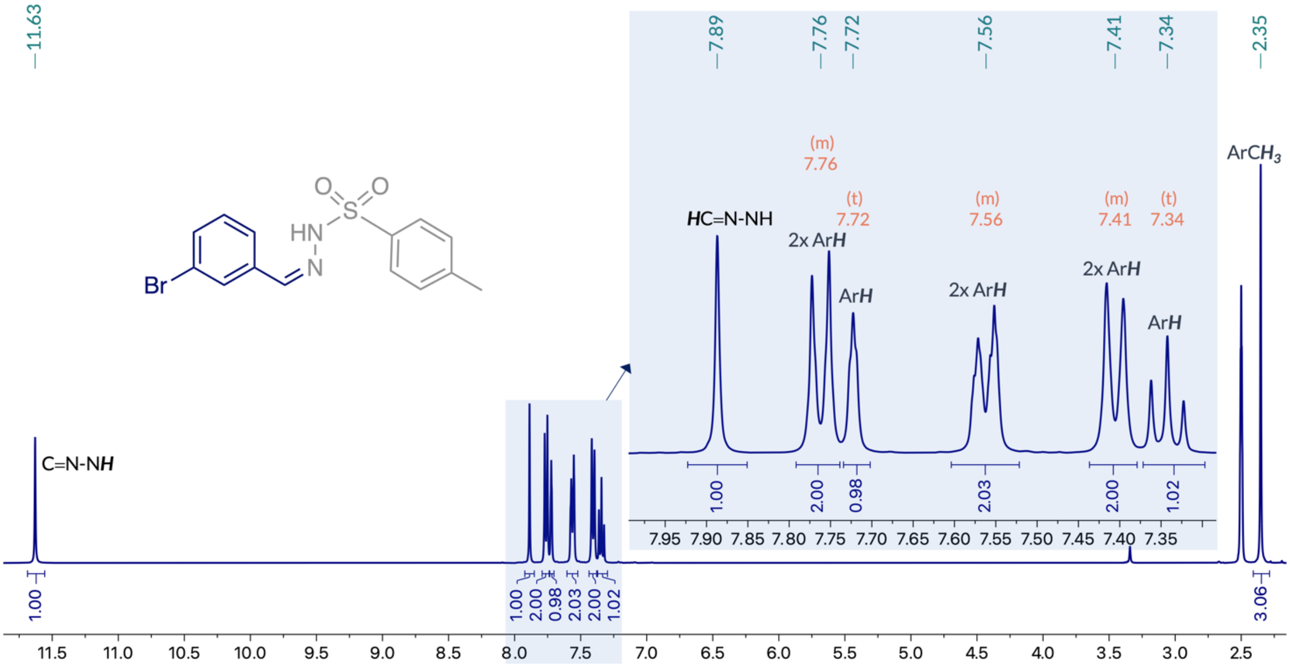

Figure S492. Hydrazone S29: ^{1}H NMR (400 MHz, DMSO-d_6, 298 K)

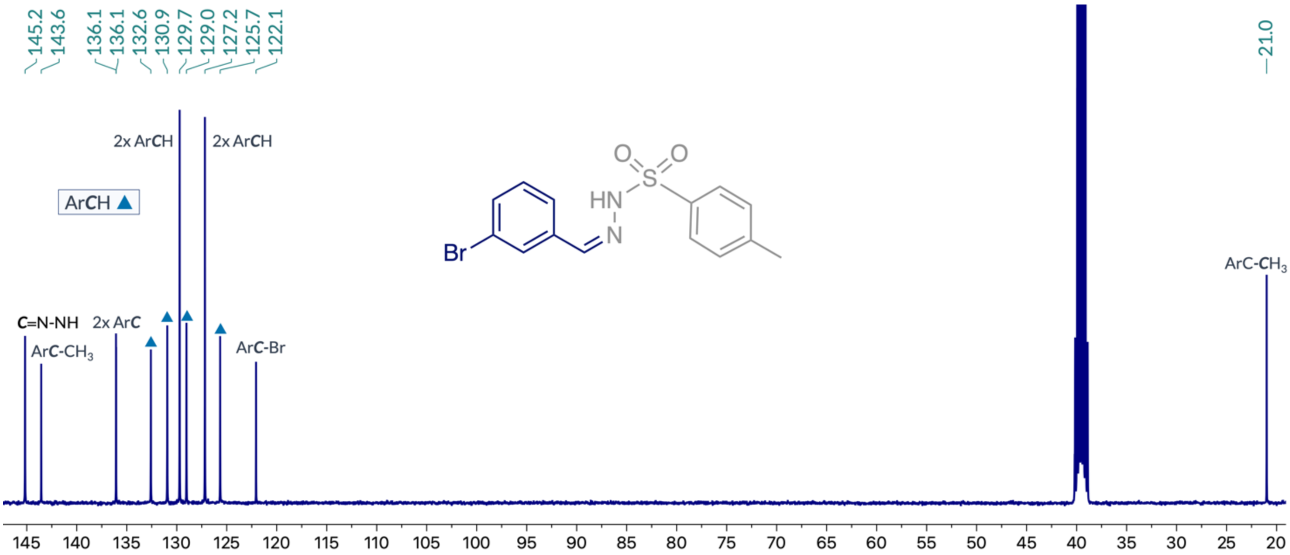

Figure S493. Hydrazone S29: ^{13}C NMR (101 MHz, DMSO-d_6, 298 K)

Hydrazone S30

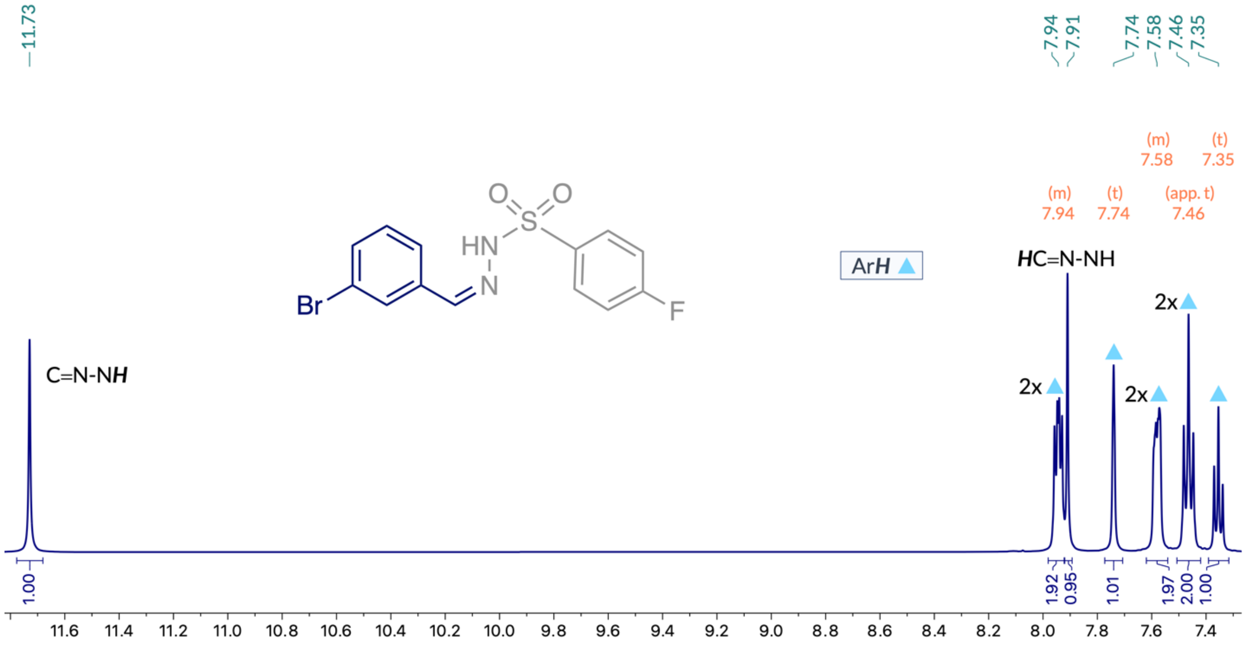

Figure S494. Hydrazone S30: ^{1}H NMR (500 MHz, DMSO-d_6, 298 K)

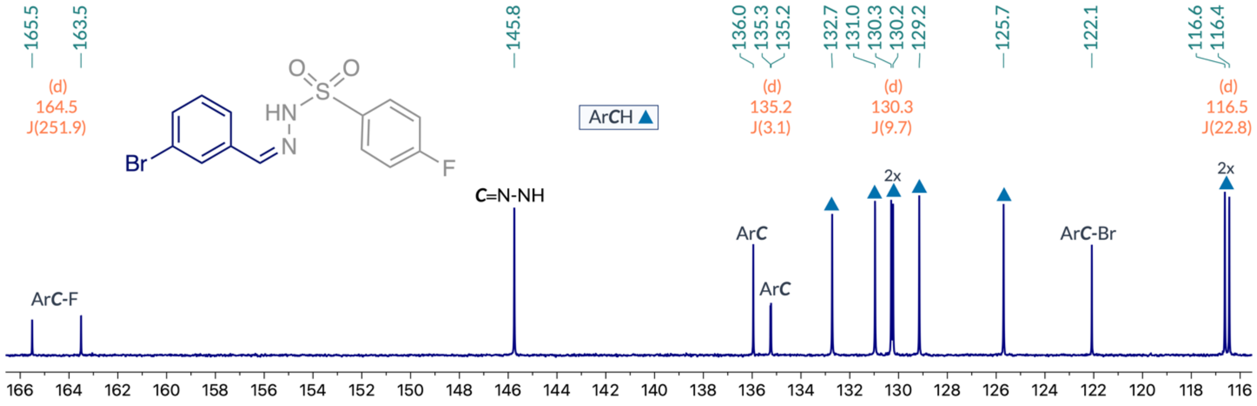

Figure S495. Hydrazone S30: ^{13}C NMR (126 MHz, DMSO-d_6, 298 K)

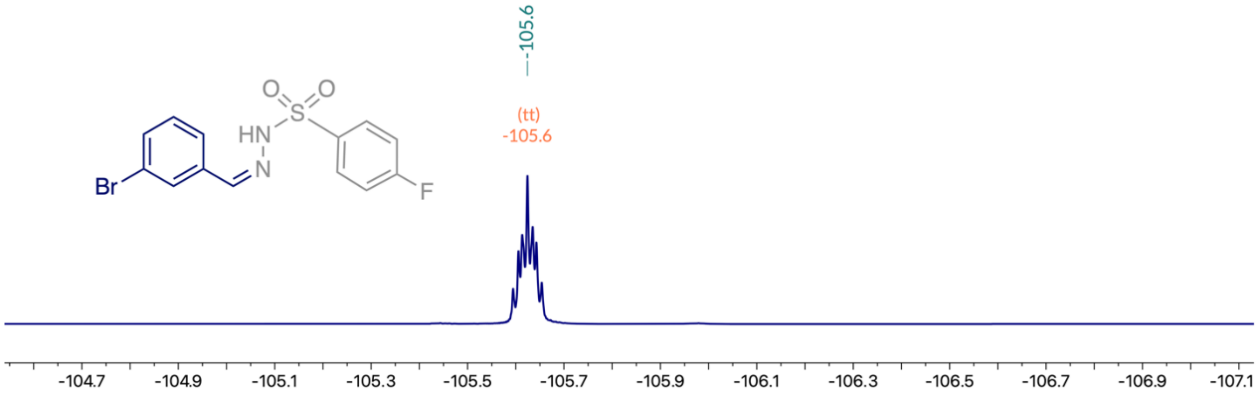

Figure S496. Hydrazone S30: ^{19}F NMR (470 MHz, DMSO-d_6, 298 K)

Hydrazone S31

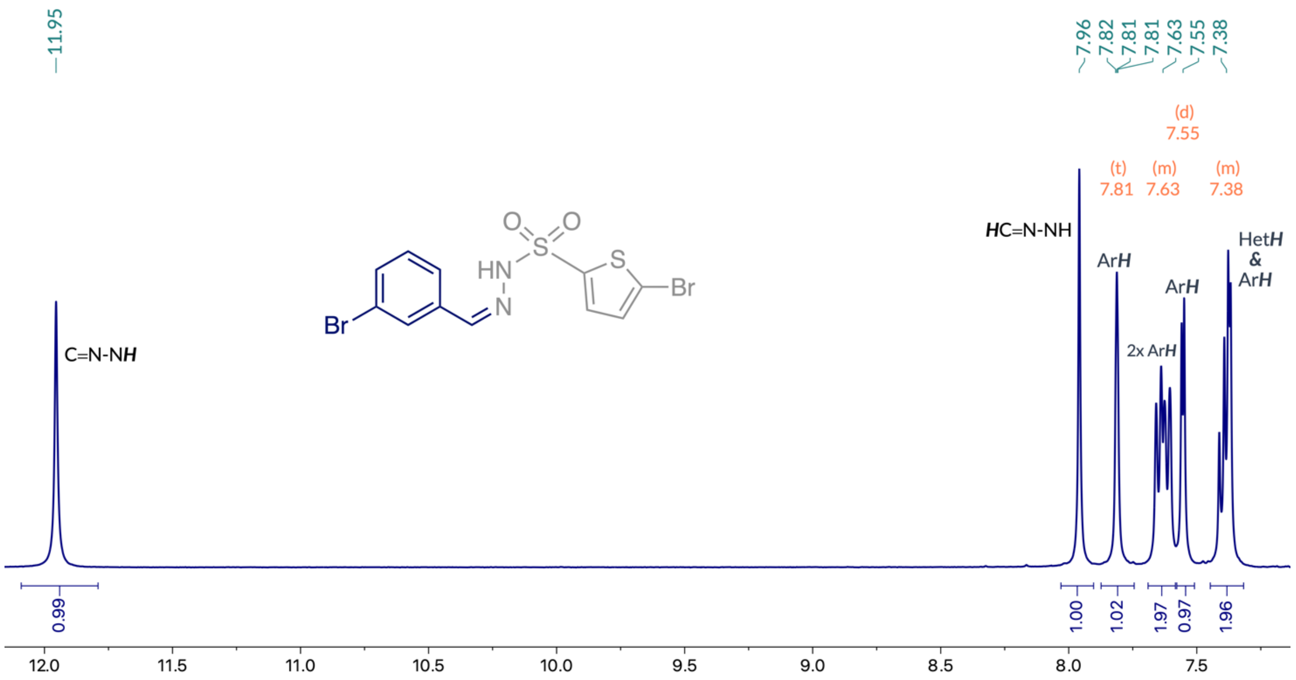

Figure S497. Hydrazone S31: ^{1}H NMR (400 MHz, DMSO-d_6, 298 K)

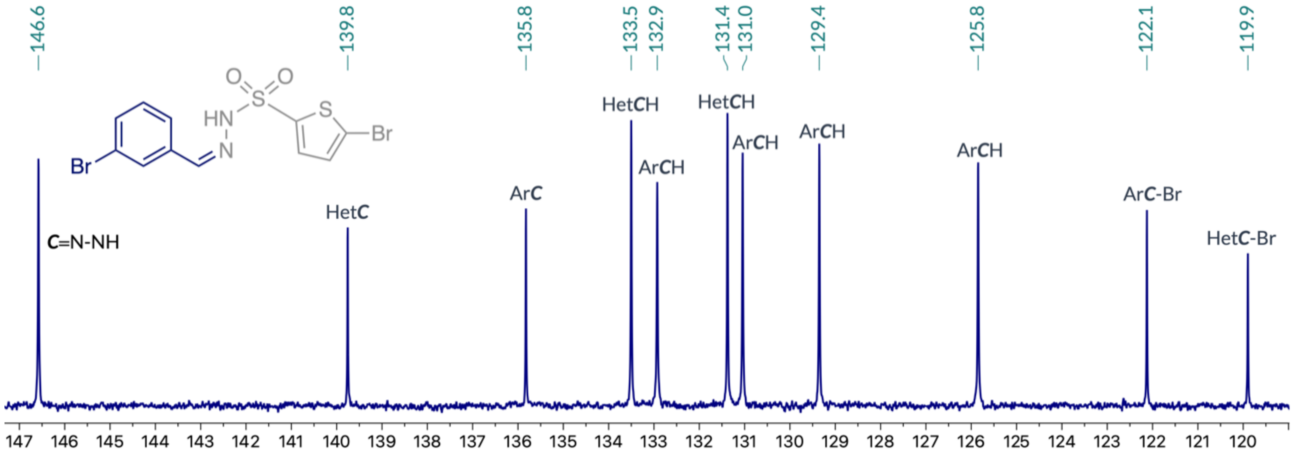

Figure S498. Hydrazone S31: ^{13}C NMR (101 MHz, DMSO-d_6, 298 K)

Hydrazone S32

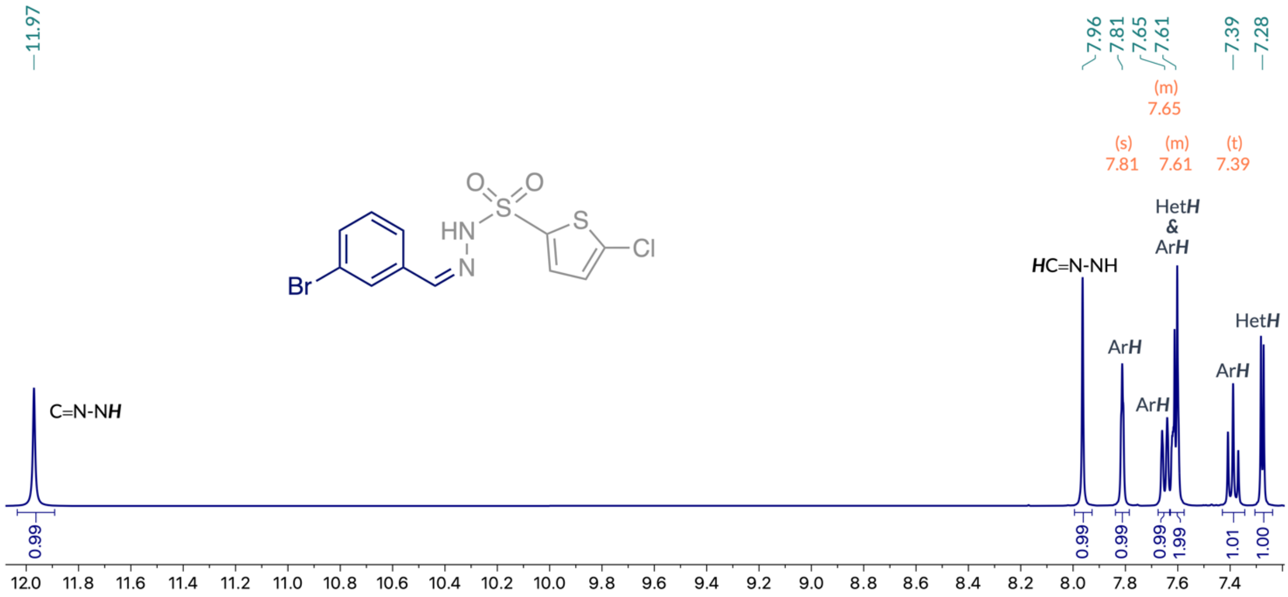

Figure S499. Hydrazone S32: ^{1}H NMR (400 MHz, DMSO-d_6, 298 K)

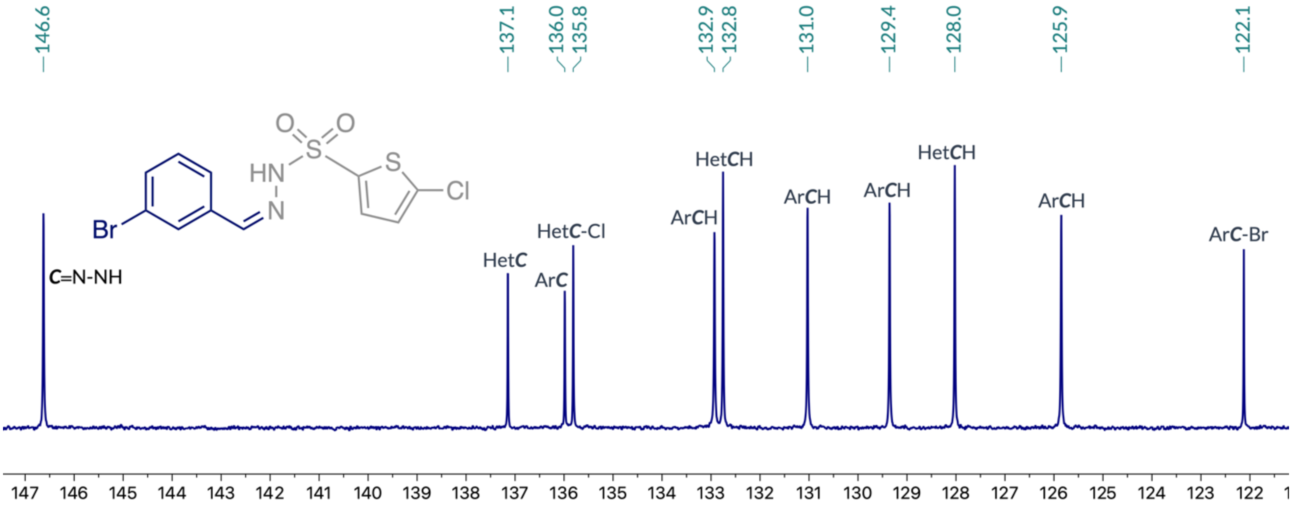

Figure S500. Hydrazone S32: ^{13}C NMR (101 MHz, DMSO-d_6, 298 K)

Hydrazone S33

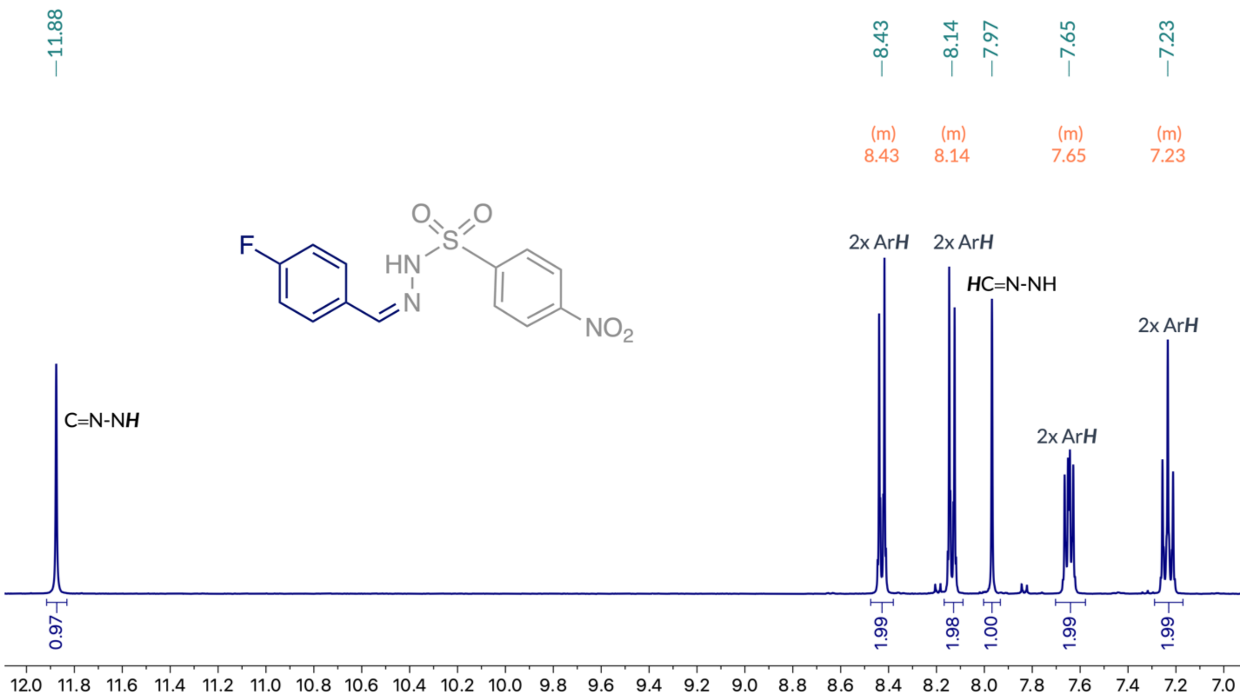

Figure S501. Hydrazone S33: ^{1}H NMR (400 MHz, DMSO-d_6, 298 K)

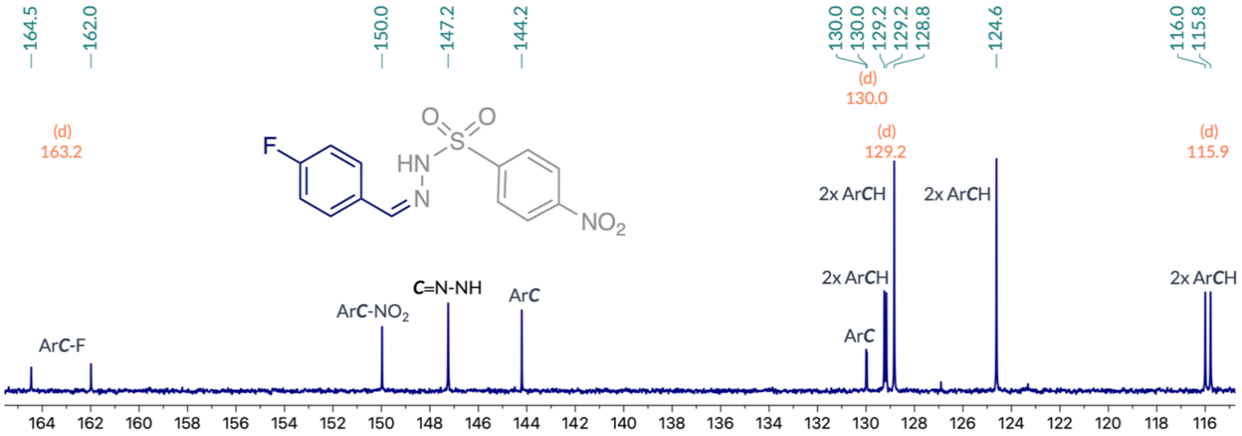

Figure S502. Hydrazone S33: ^{13}C NMR (101 MHz, DMSO-d_6, 298 K)

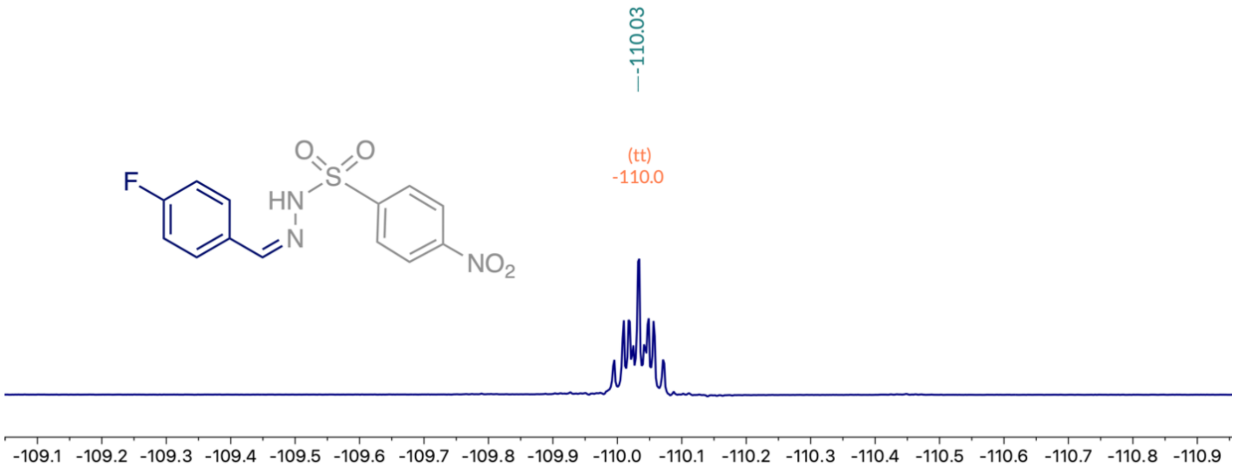

Figure S503. Hydrazone S33: ^{19}F NMR (377 MHz, DMSO-d_6, 298 K)

Hydrazone S34

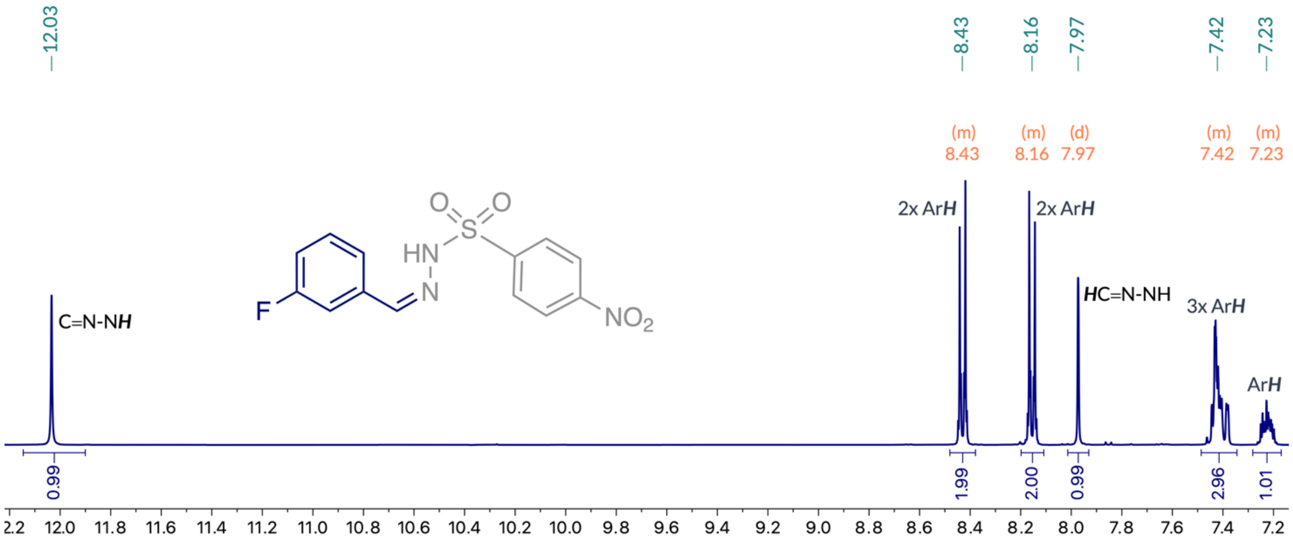

Figure S504. Hydrazone S34: ^{1}H NMR (400 MHz, DMSO-d_6, 298 K)

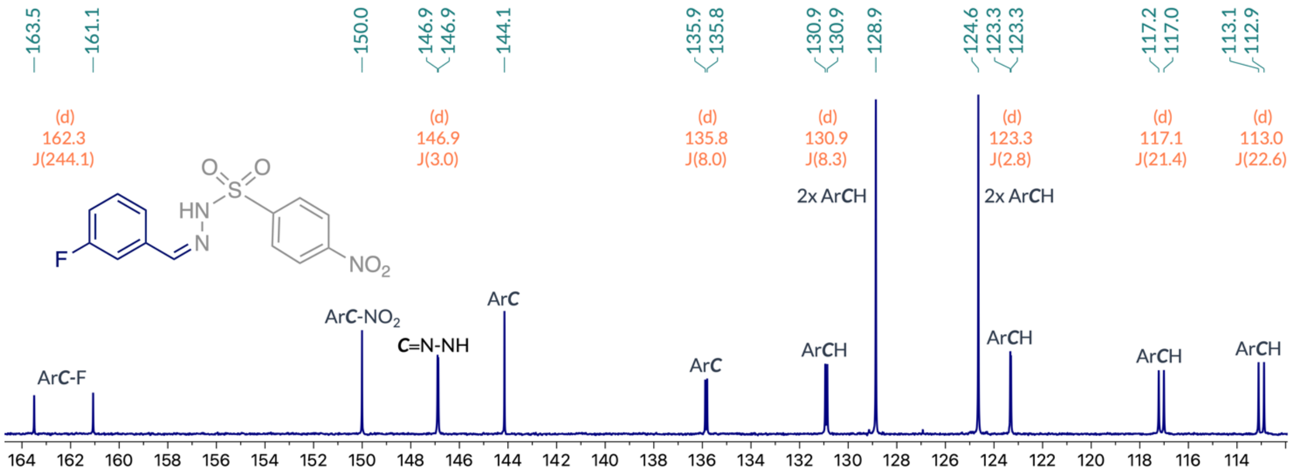

Figure S505. Hydrazone S34: ^{13}C NMR (101 MHz, DMSO-d_6, 298 K)

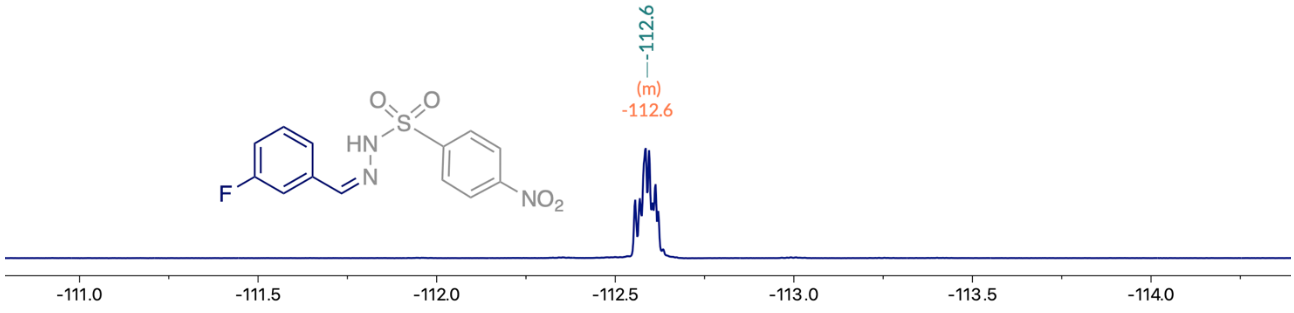

Figure S506. Hydrazone S34: ^{19}F NMR (377 MHz, DMSO-d_6, 298 K)

Hydrazone S35

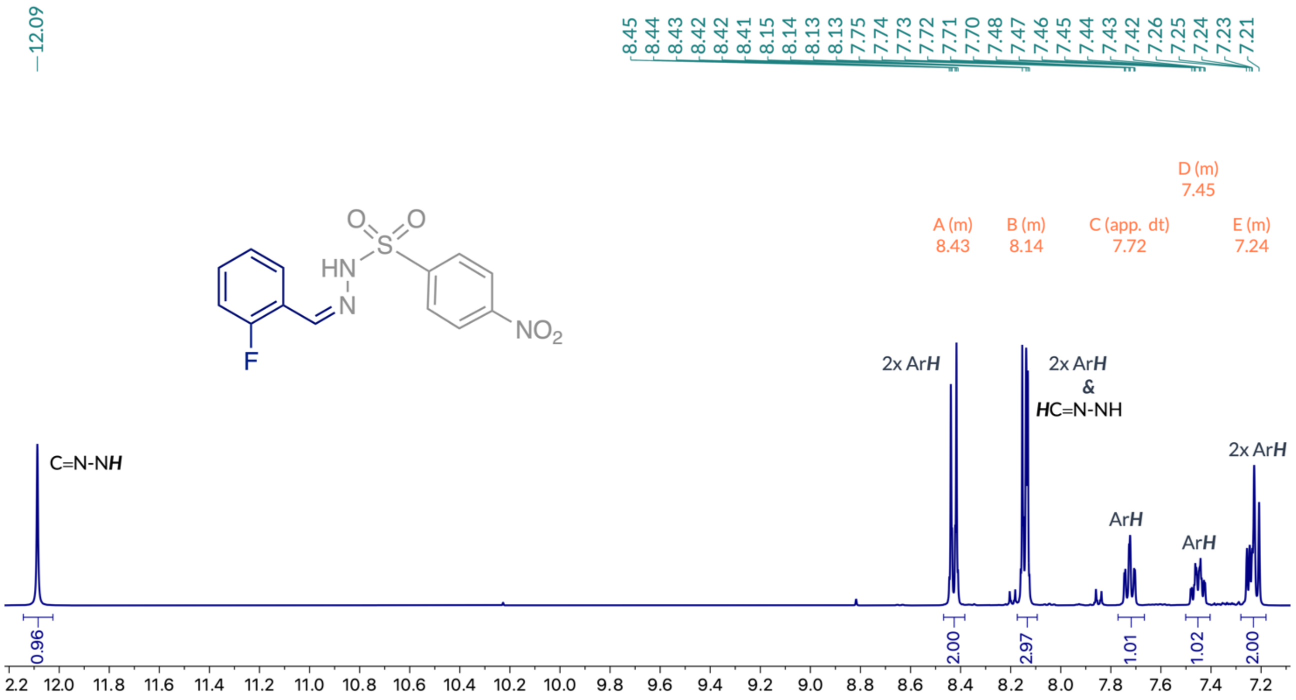

Figure S507. Hydrazone S35: ^{1}H NMR (400 MHz, DMSO-d_6, 298 K)

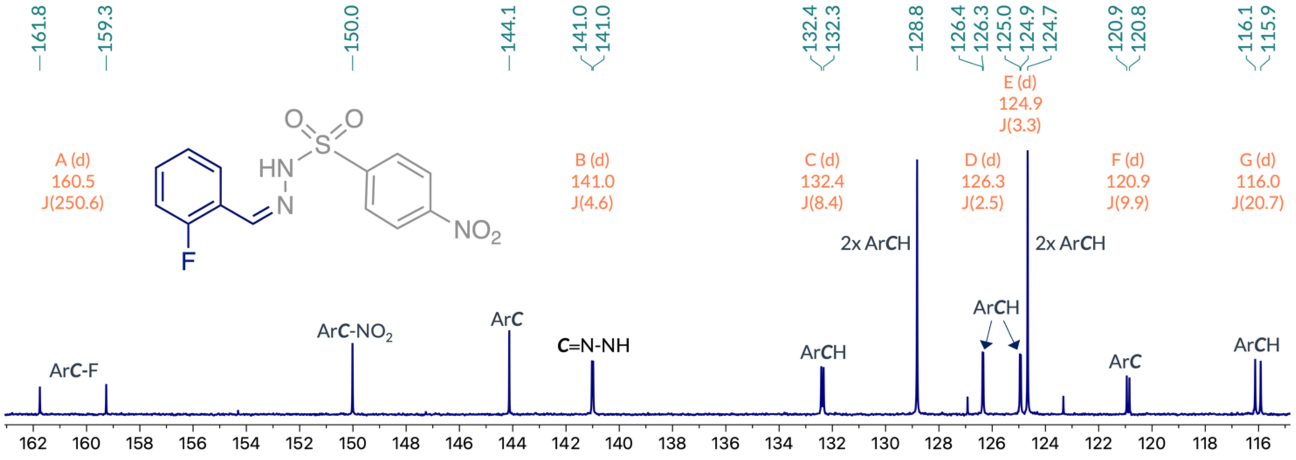

Figure S508. Hydrazone S35: ^{13}C NMR (101 MHz, DMSO-d_6, 298 K)

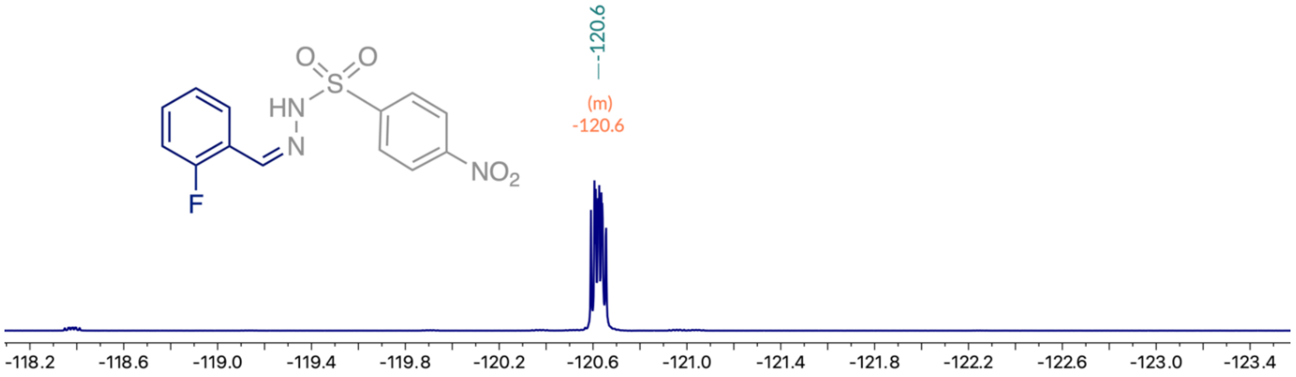

Figure S509. Hydrazone S35: ^{19}F NMR (377 MHz, DMSO-d_6, 298 K)

Hydrazone S36

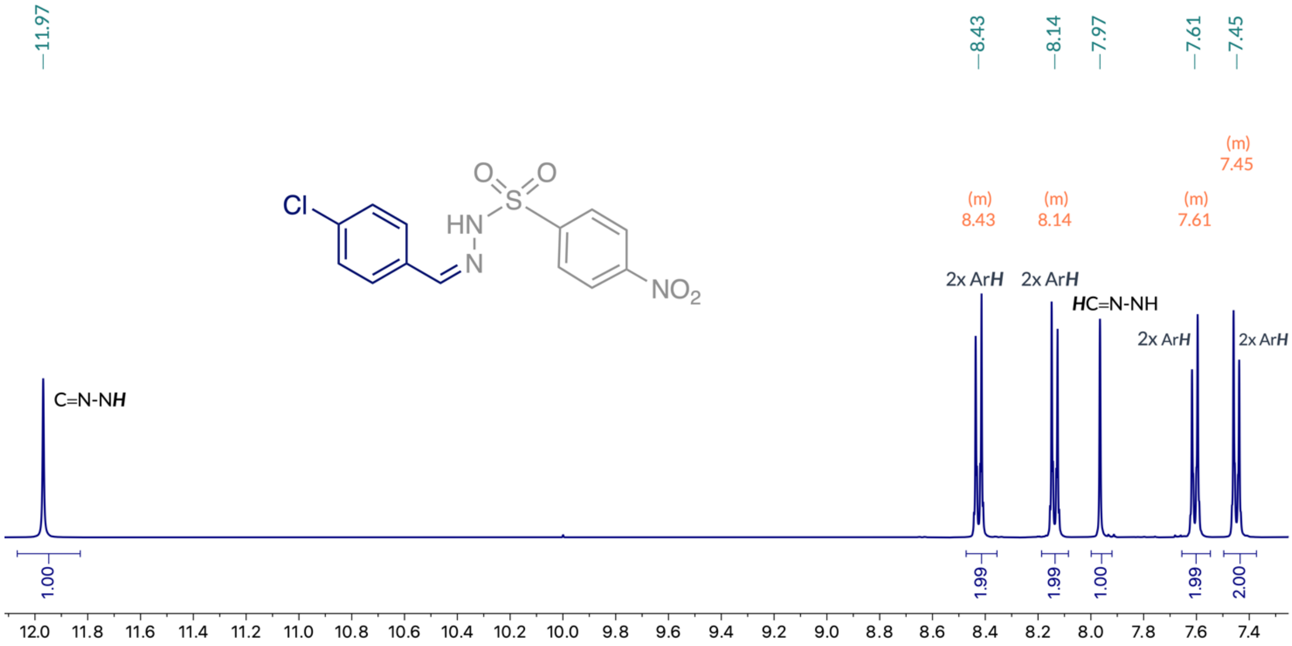

Figure S510. Hydrazone S36: ^{1}H NMR (400 MHz, DMSO-d_6, 298 K)

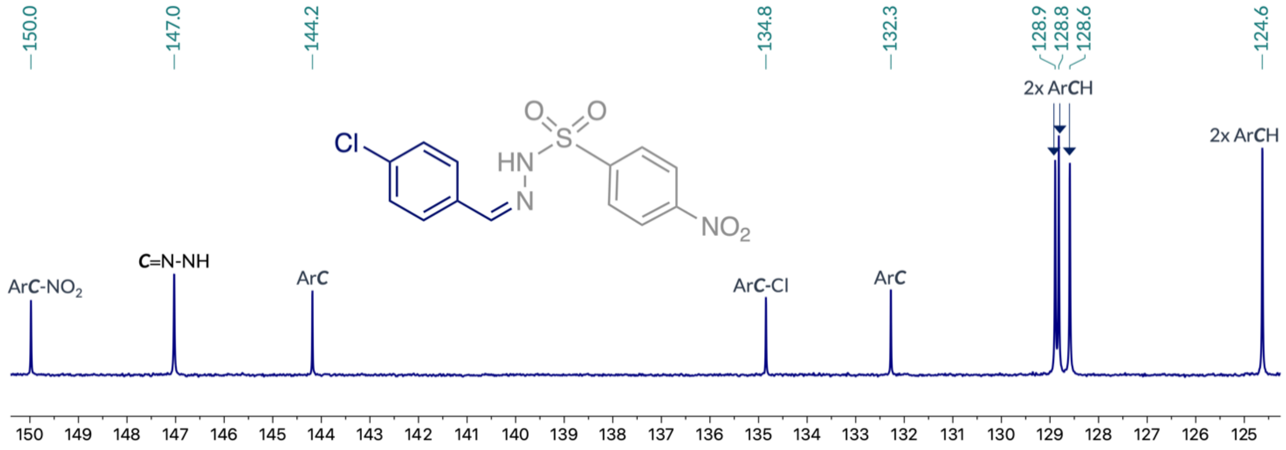

Figure S511. Hydrazone S36: ^{13}C NMR (101 MHz, DMSO-d_6, 298 K)

Hydrazone S37

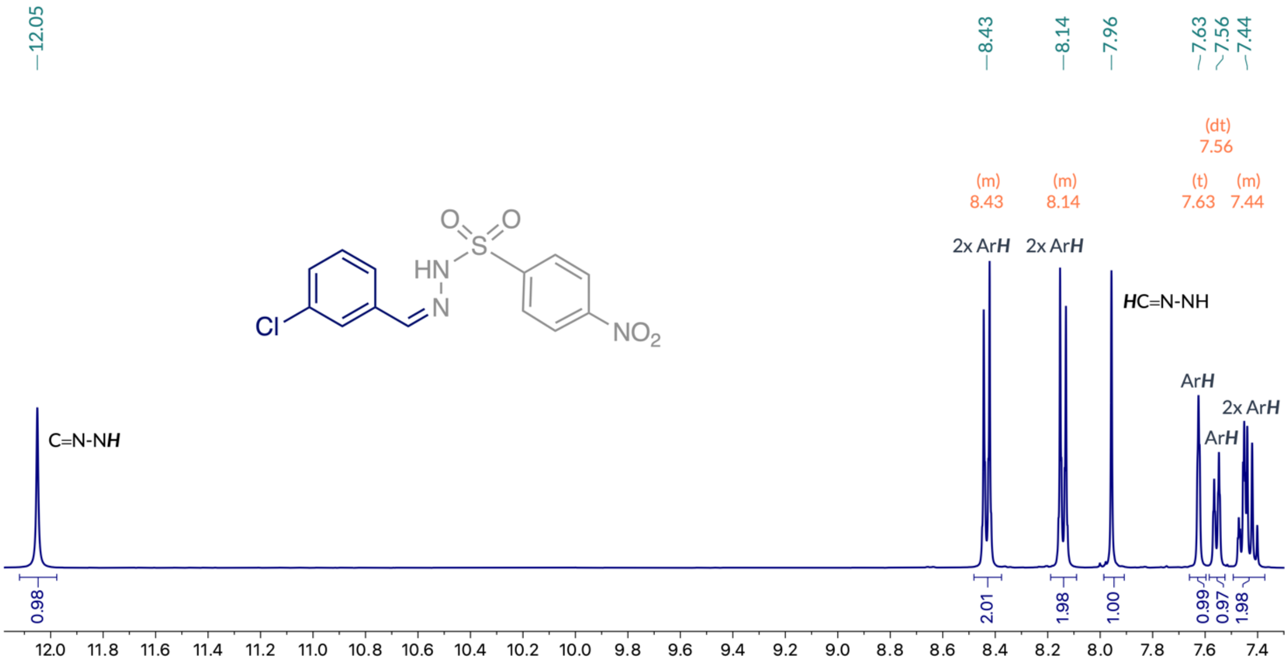

Figure S512. Hydrazone S37: ^{1}H NMR (400 MHz, DMSO-d_6, 298 K)

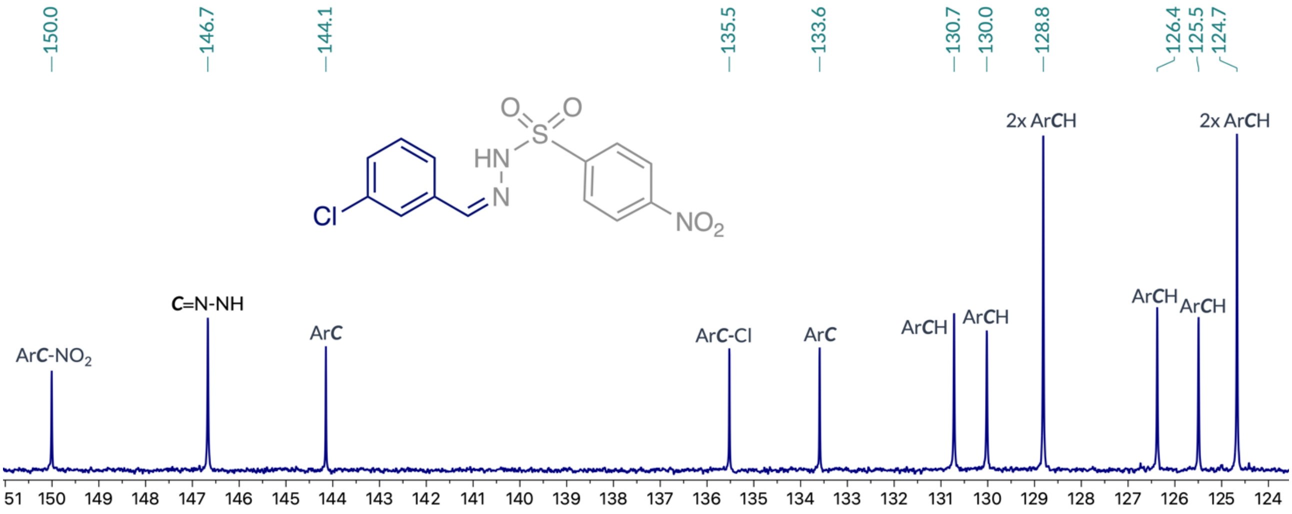

Figure S513. Hydrazone S37: ^{13}C NMR (101 MHz, DMSO-d_6, 298 K)

Hydrazone S38

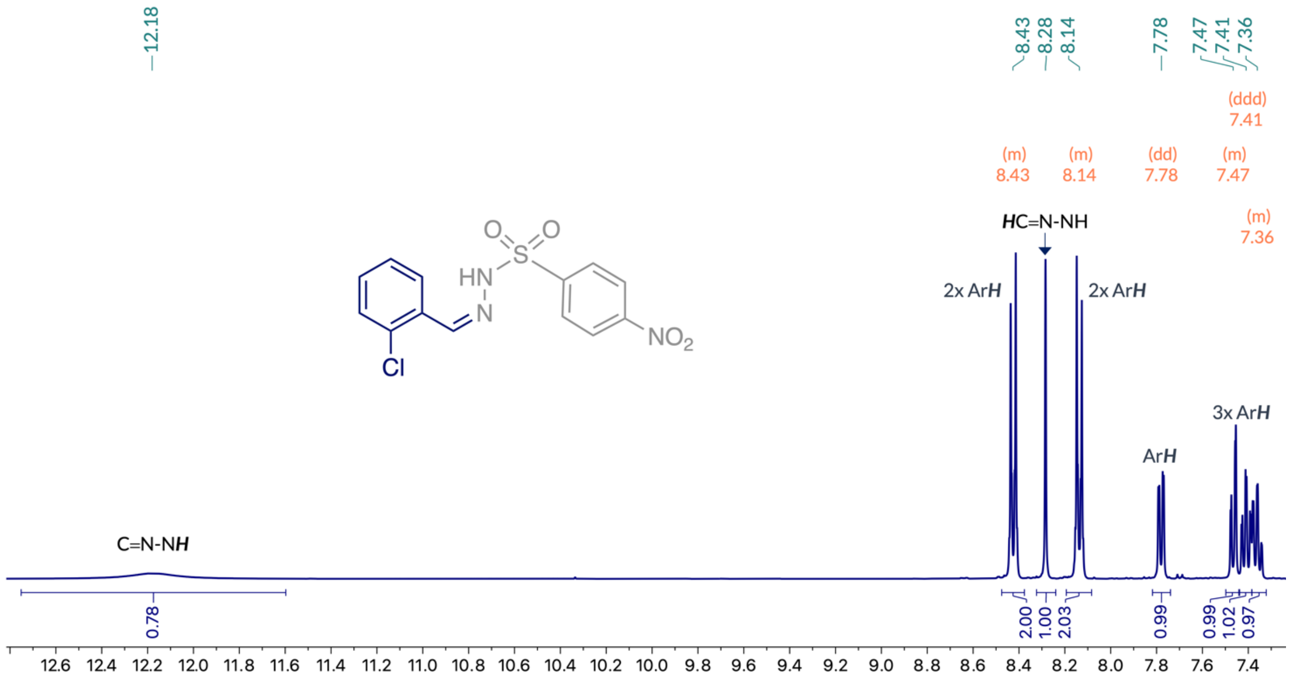

Figure S514. Hydrazone S38: ^{1}H NMR (400 MHz, DMSO-d_6, 298 K)

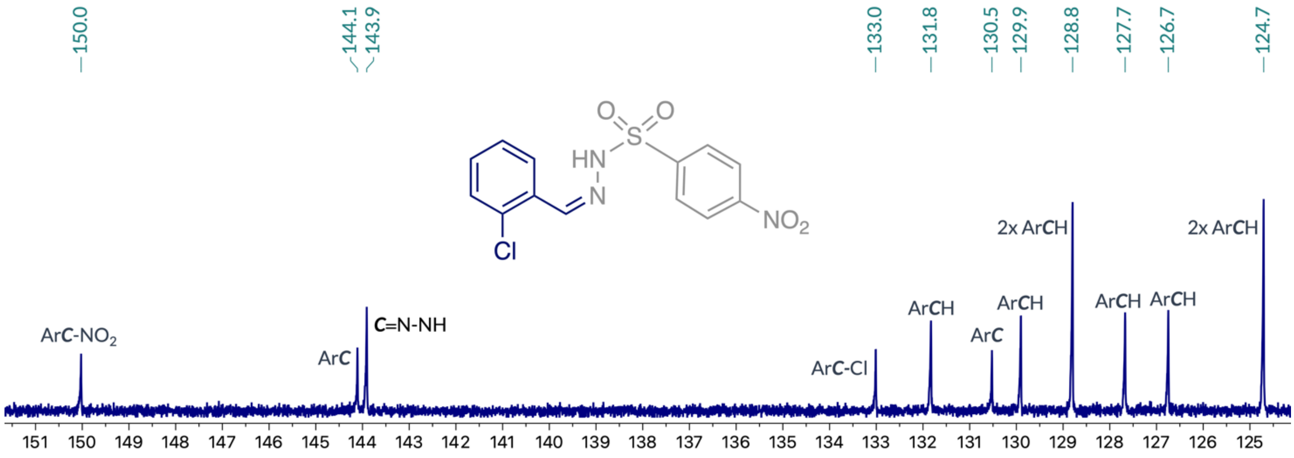

Figure S515. Hydrazone S38: ^{13}C NMR (101 MHz, DMSO-d_6, 298 K)

Hydrazone S39

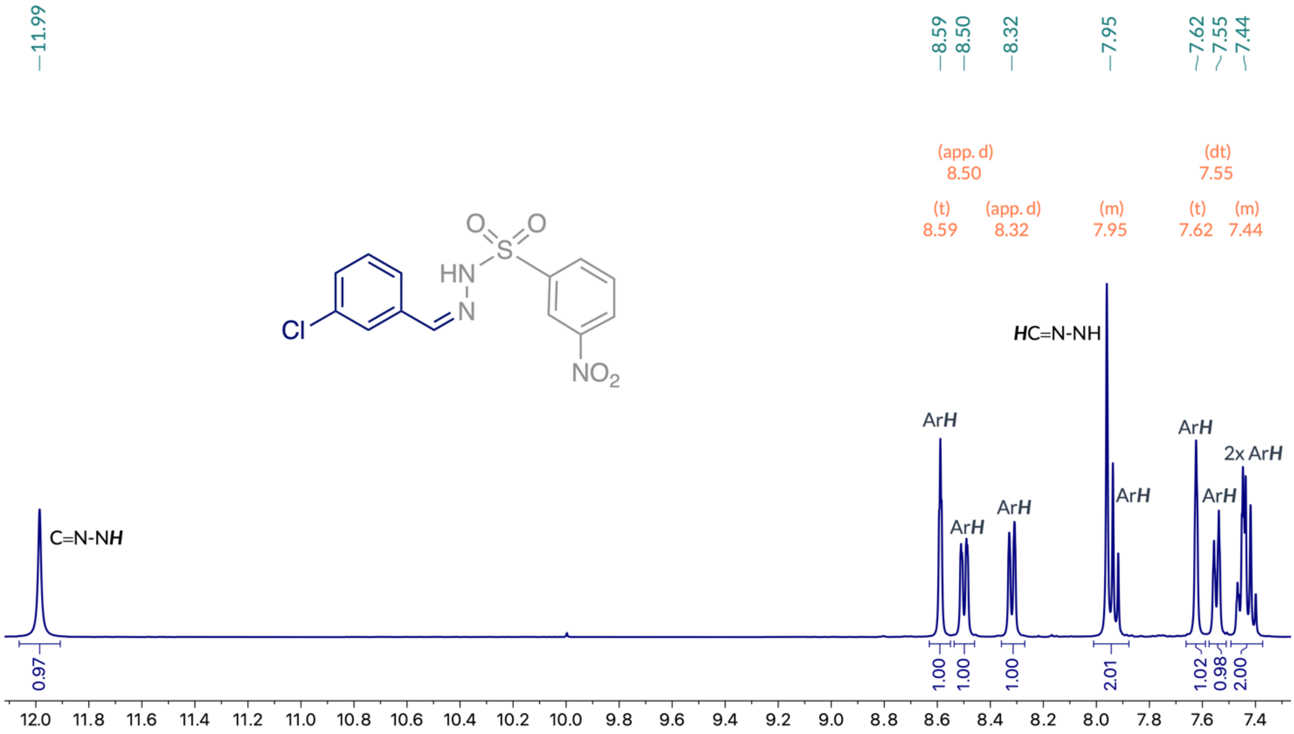

Figure S516. Hydrazone S39: ^{1}H NMR (400 MHz, DMSO-d_6, 298 K)

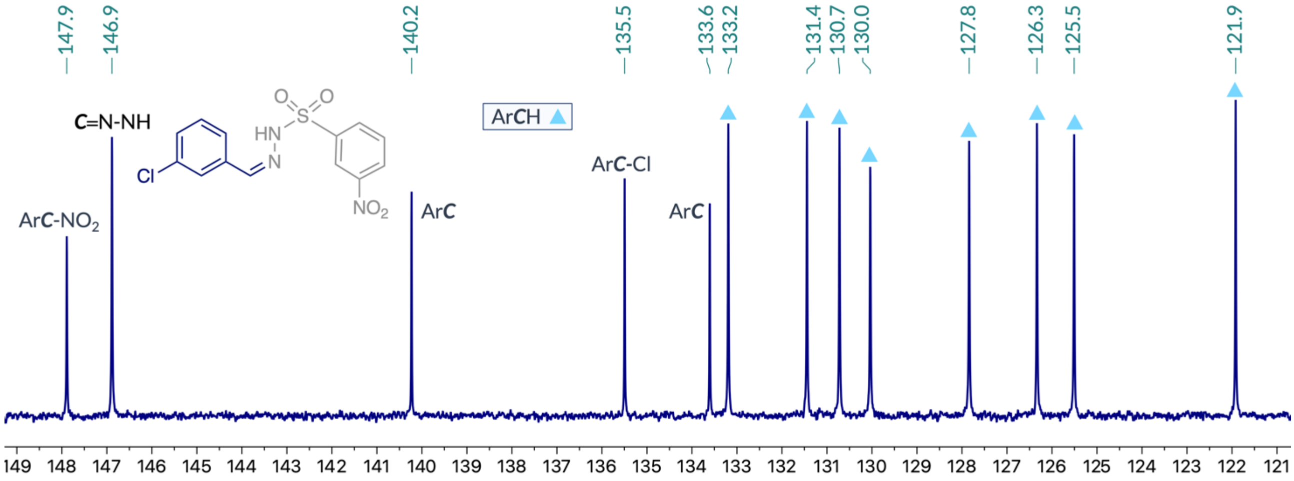

Figure S517. Hydrazone S39: ^{13}C NMR (101 MHz, DMSO-d_6, 298 K)

Hydrazone S40

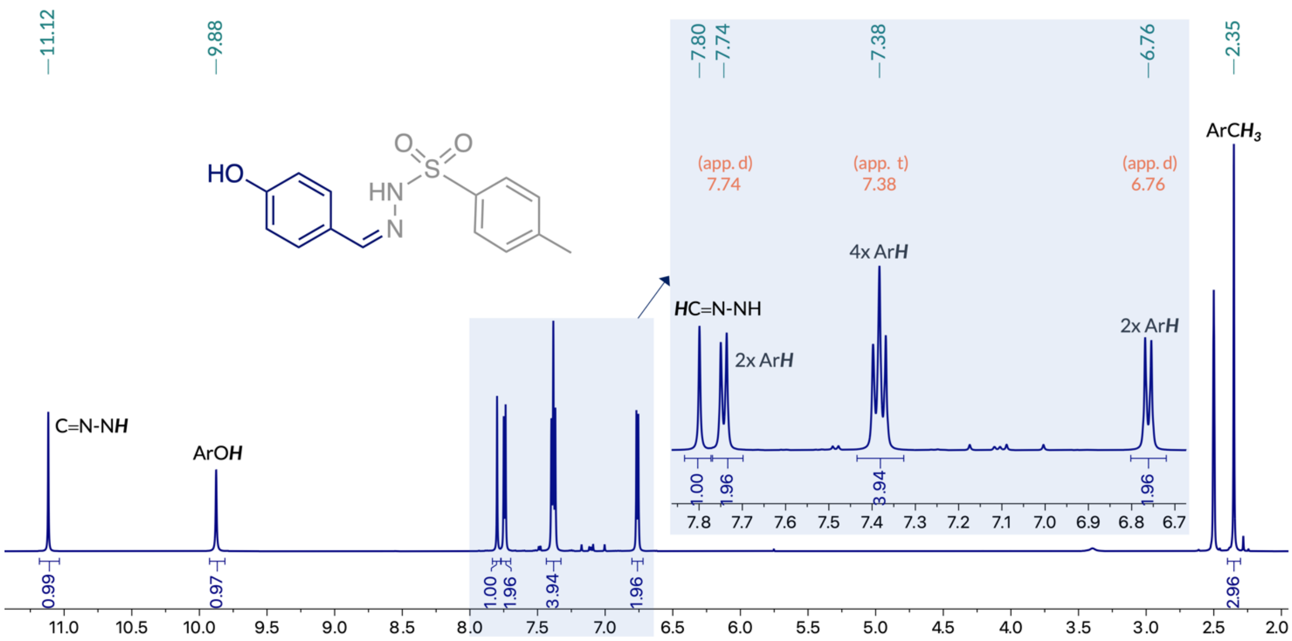

Figure S518. Hydrazone S40: ^{1}H NMR (600 MHz, DMSO-d_6, 298 K)

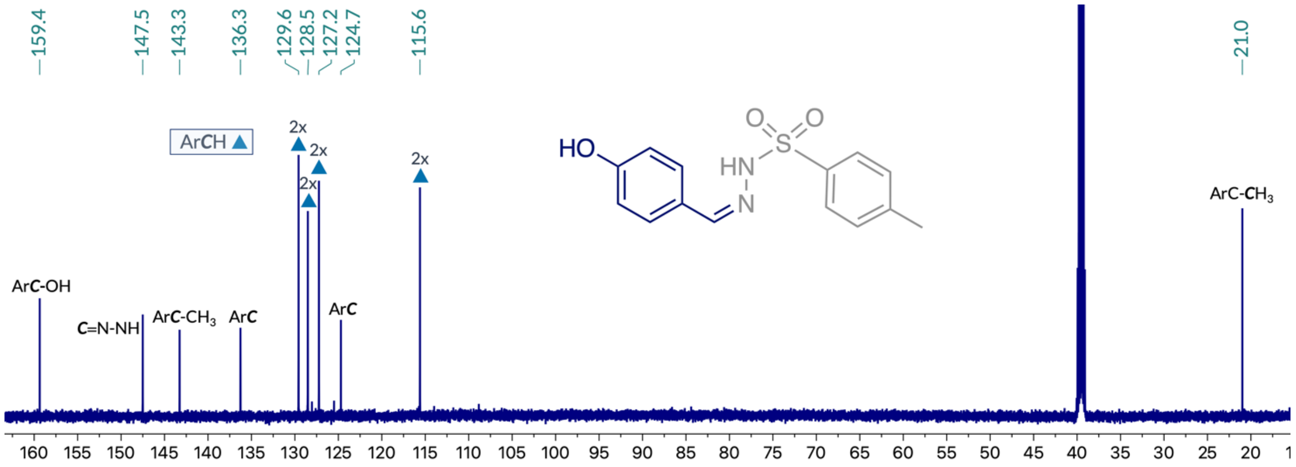

Figure S519. Hydrazone S40: ^{13}C NMR (151 MHz, DMSO-d_6, 298 K)

Hydrazone S41

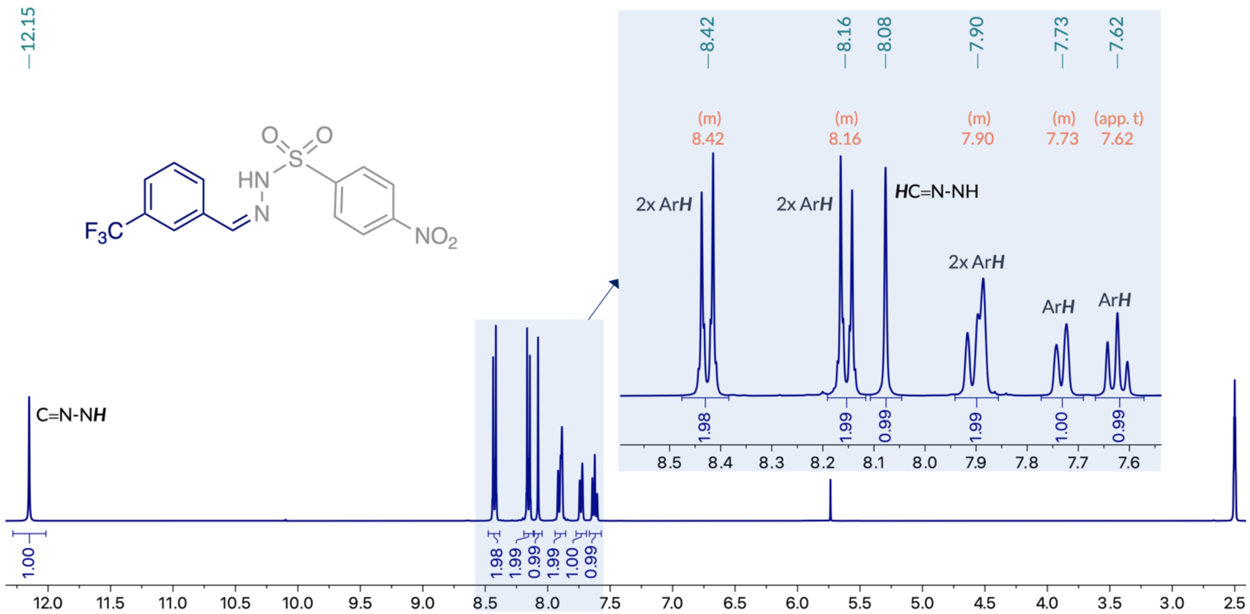

Figure S520. Hydrazone S41: ^{1}H NMR (400 MHz, DMSO-d_6, 298 K)

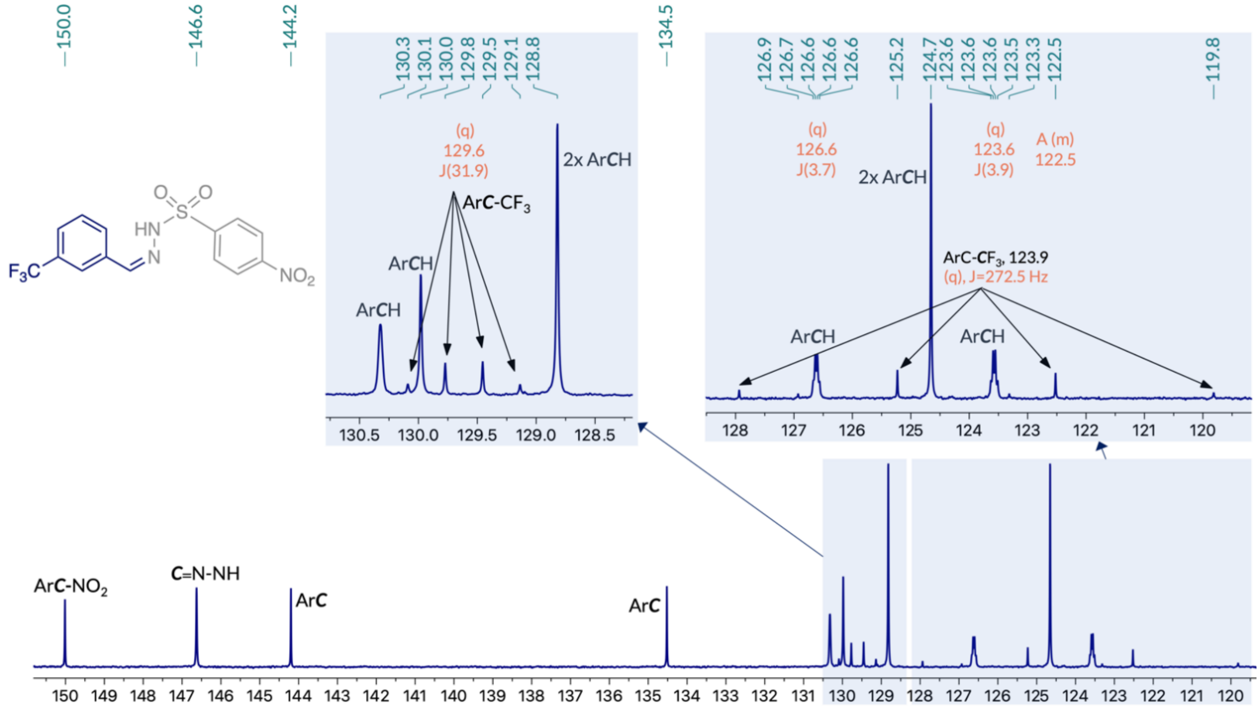

Figure S521. Hydrazone S41: ^{13}C NMR (101 MHz, DMSO-d_6, 298 K)

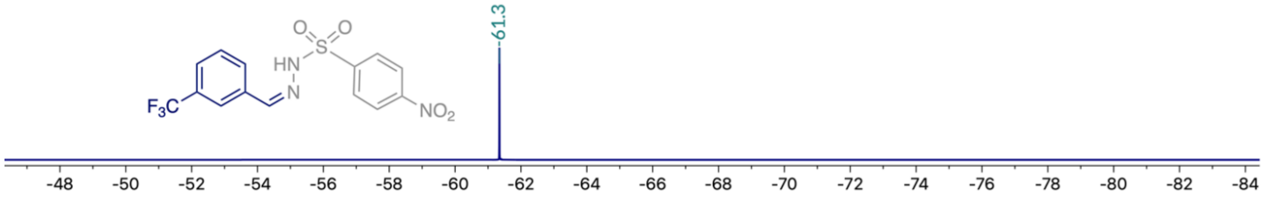

Figure S522. Hydrazone S41: ^{19}F{^{1}H} NMR (377 MHz, DMSO-d_6, 298 K)

Hydrazone S42

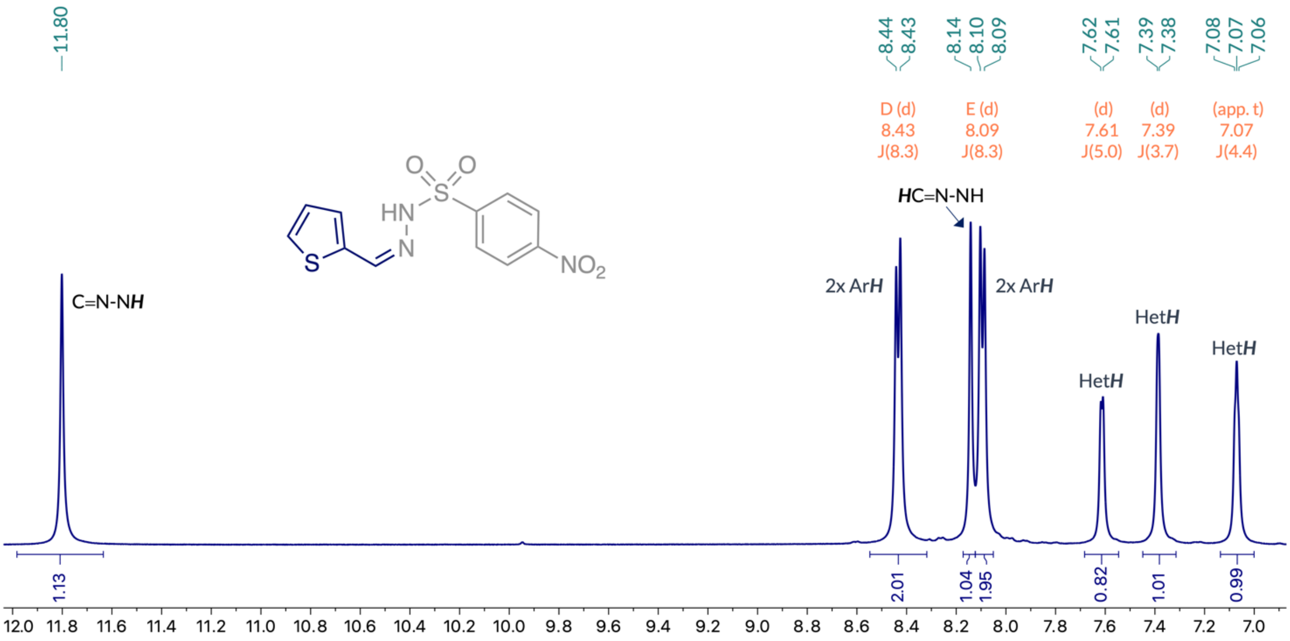

Figure S523. Hydrazone S42: ^{1}H NMR (400 MHz, DMSO-d_6, 298 K)

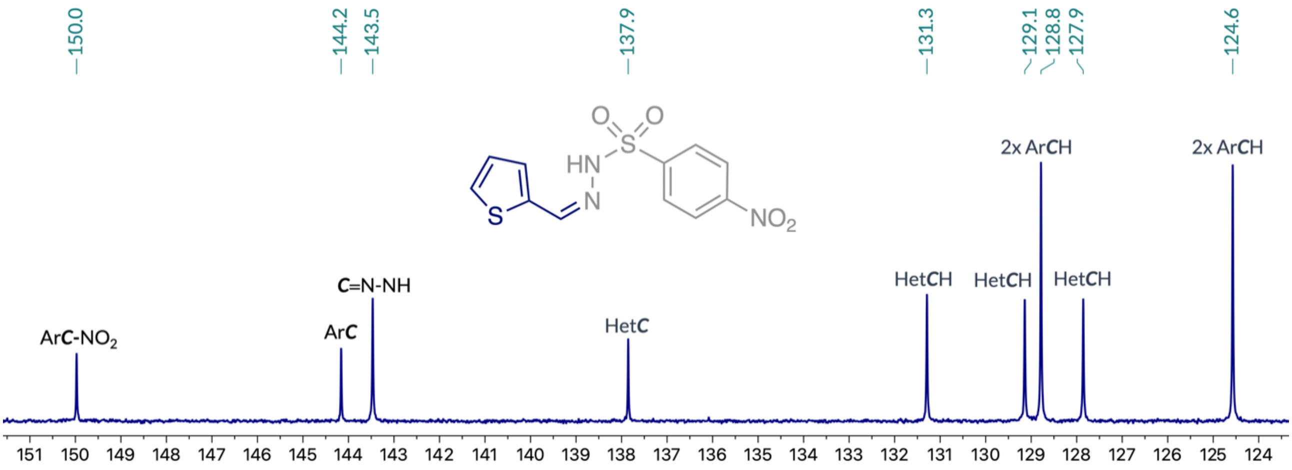

Figure S524. Hydrazone S42: ^{13}C NMR (101 MHz, DMSO-d_6, 298 K)

Hydrazone S43

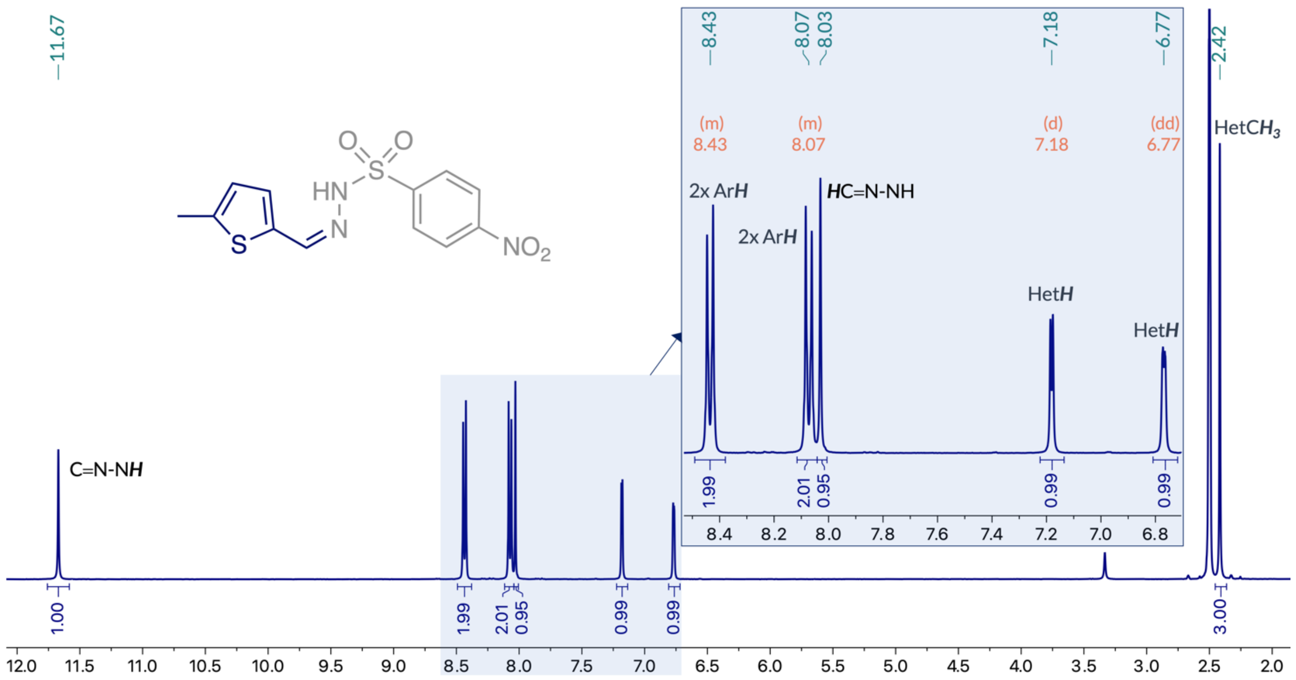

Figure S525. Hydrazone S43: ^{1}H NMR (400 MHz, DMSO-d_6, 298 K)

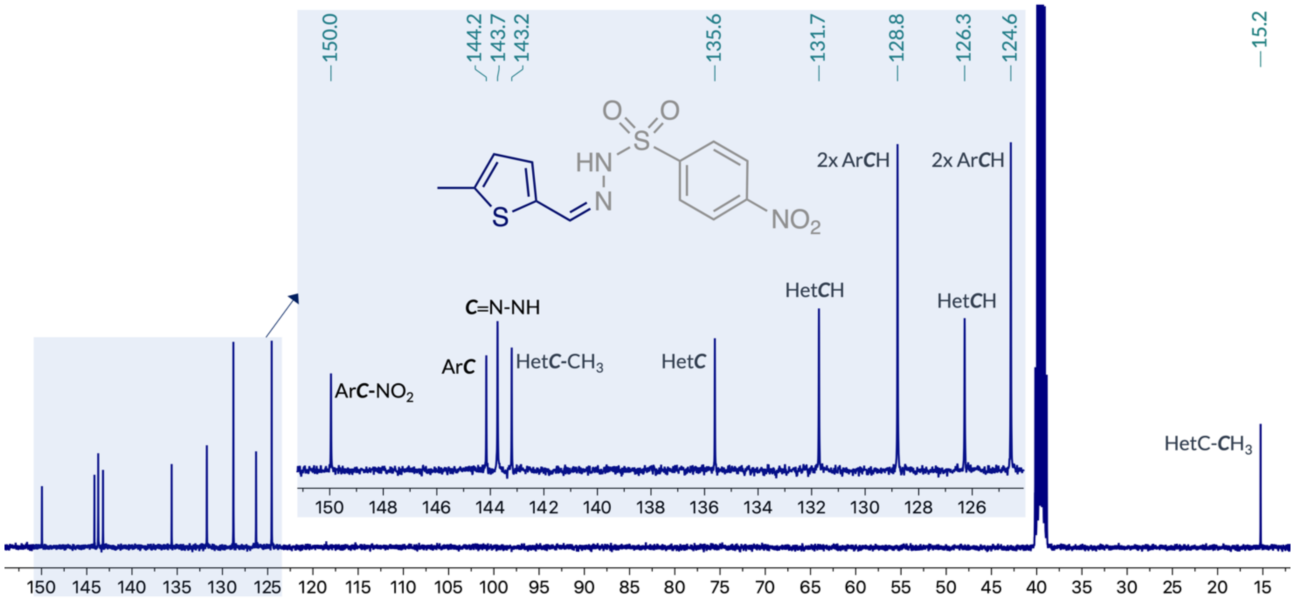

Figure S526. Hydrazone S43: ^{13}C NMR (101 MHz, DMSO-d_6, 298 K)

Hydrazone S44

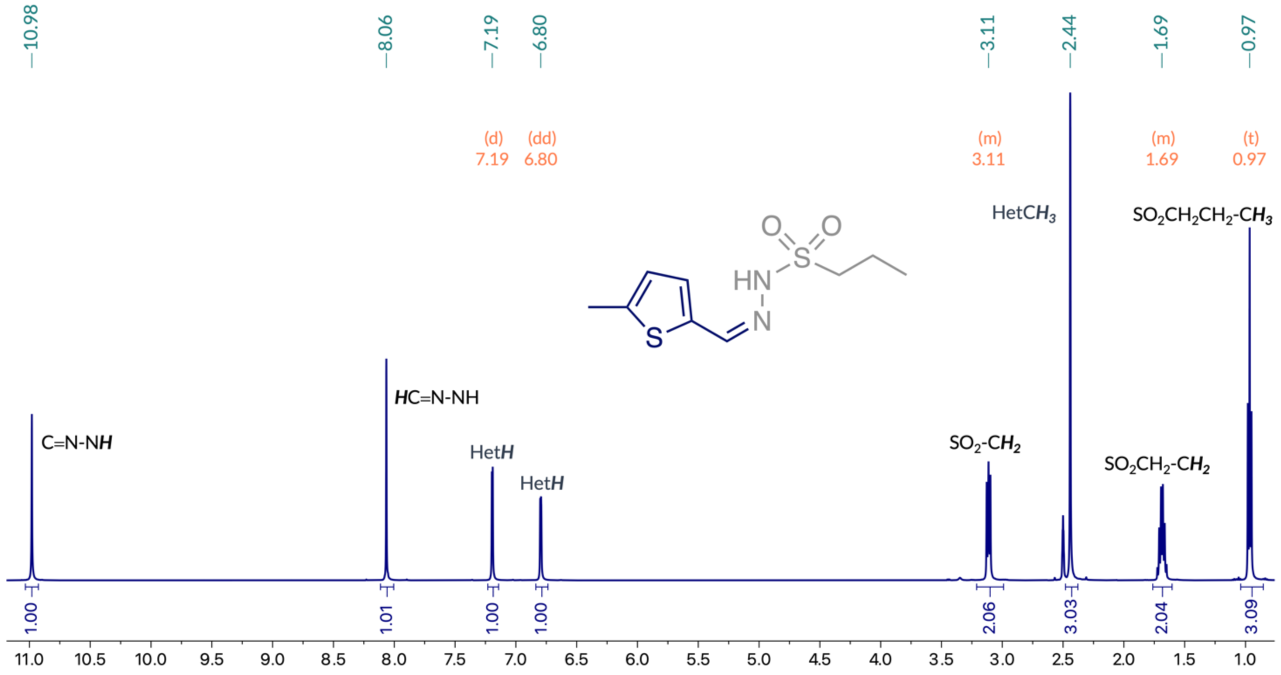

Figure S527. Hydrazone S44: ^{1}H NMR (500 MHz, DMSO-d_6, 298 K)

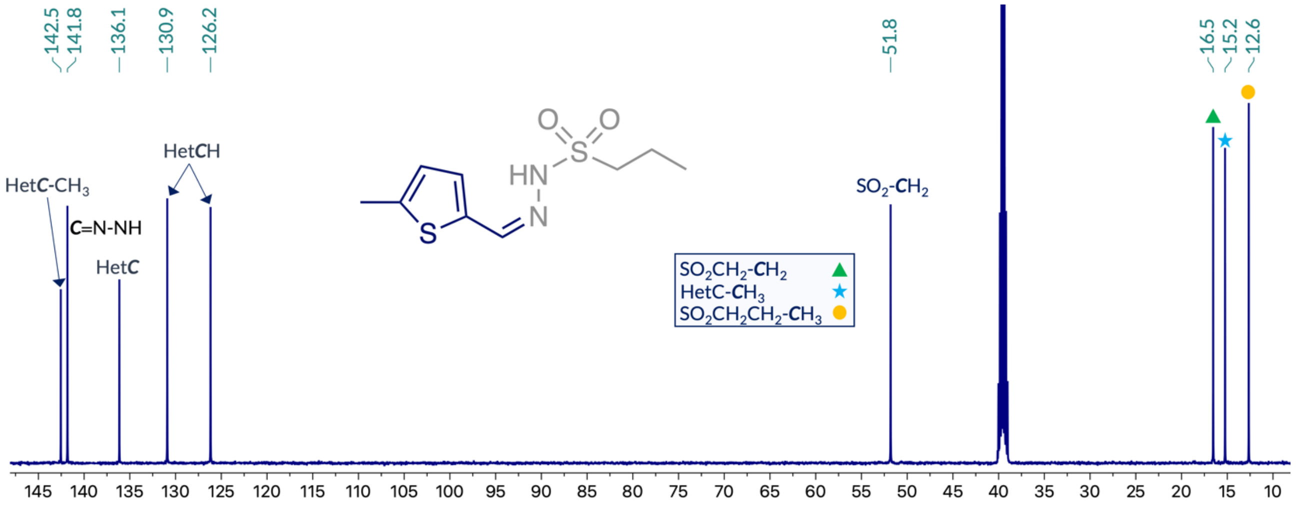

Figure S528. Hydrazone S44: ^{13}C NMR (126 MHz, DMSO-d_6, 298 K)

Hydrazone S45

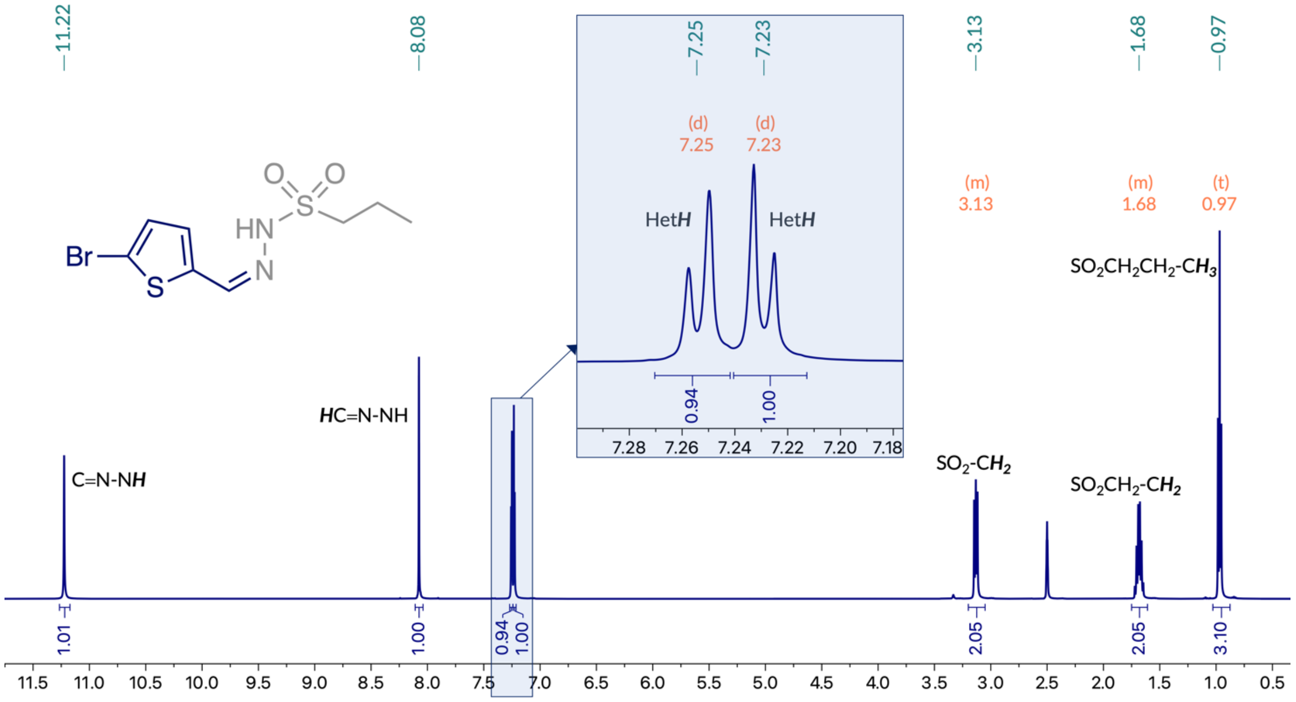

Figure S529. Hydrazone S45: ^{1}H NMR (500 MHz, DMSO-d_6, 298 K)

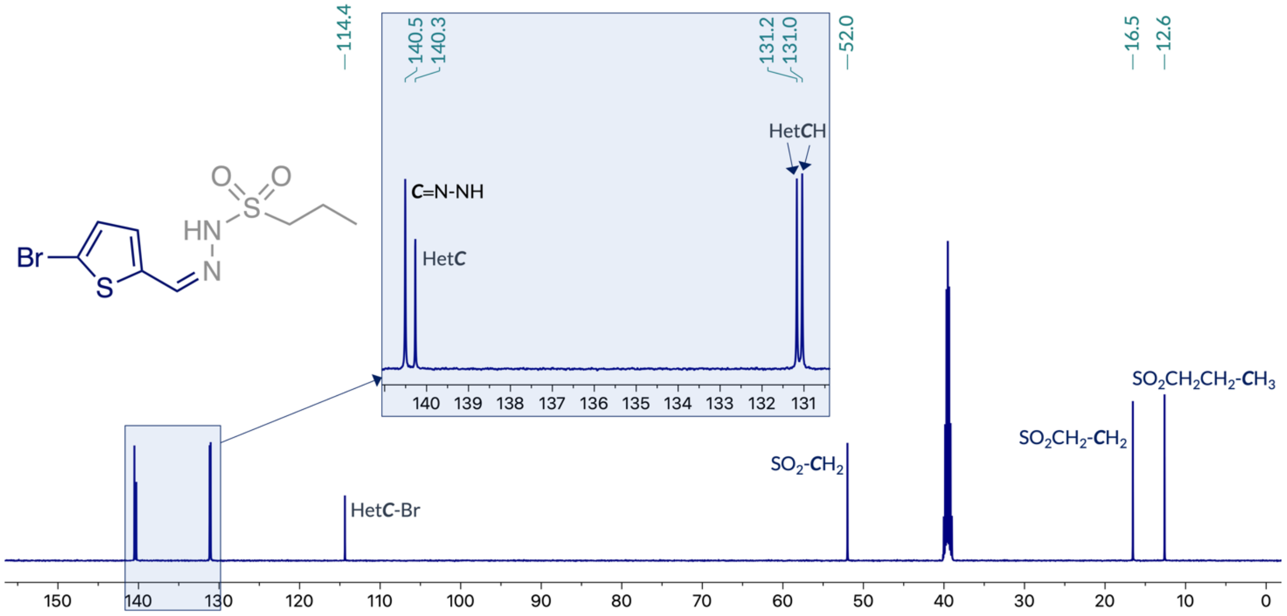

Figure S530. Hydrazone S45: ^{13}C NMR (126 MHz, DMSO-d_6, 298 K)

Hydrazone S46

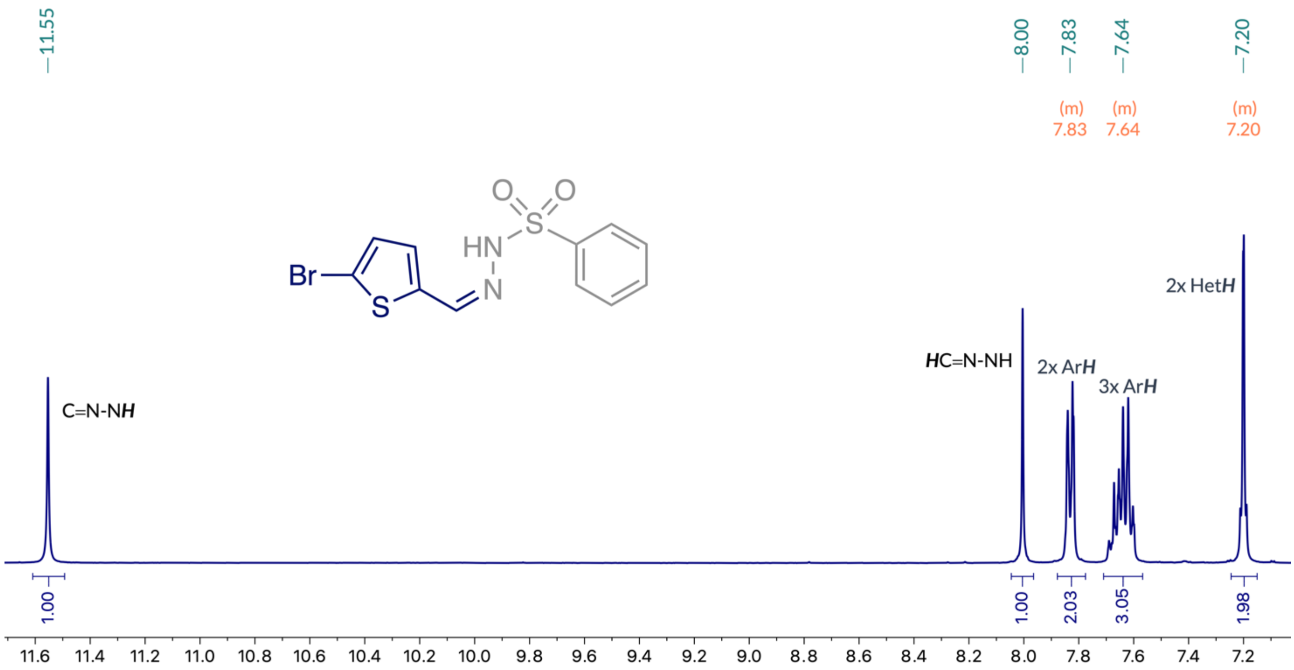

Figure S531. Hydrazone S46: ^{1}H NMR (400 MHz, DMSO-d_6, 298 K)

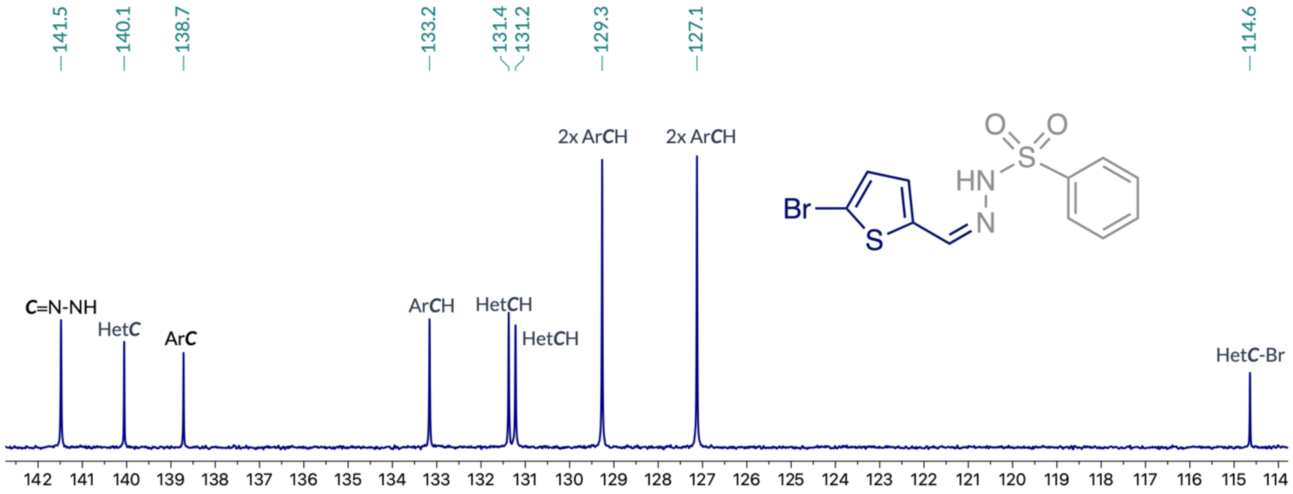

Figure S532. Hydrazone S46: ^{13}C NMR (101 MHz, DMSO-d_6, 298 K)

Hydrazone S47

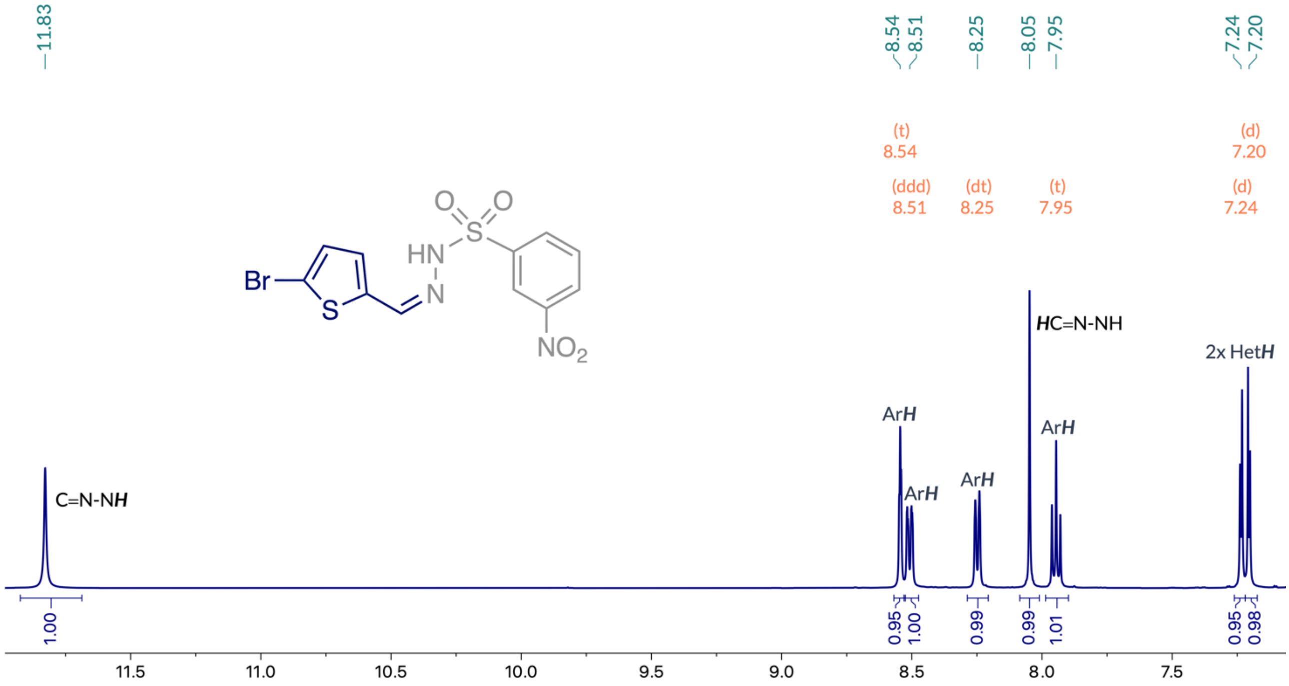

Figure S533. Hydrazone S47: ^{1}H NMR (500 MHz, DMSO-d_6, 298 K)

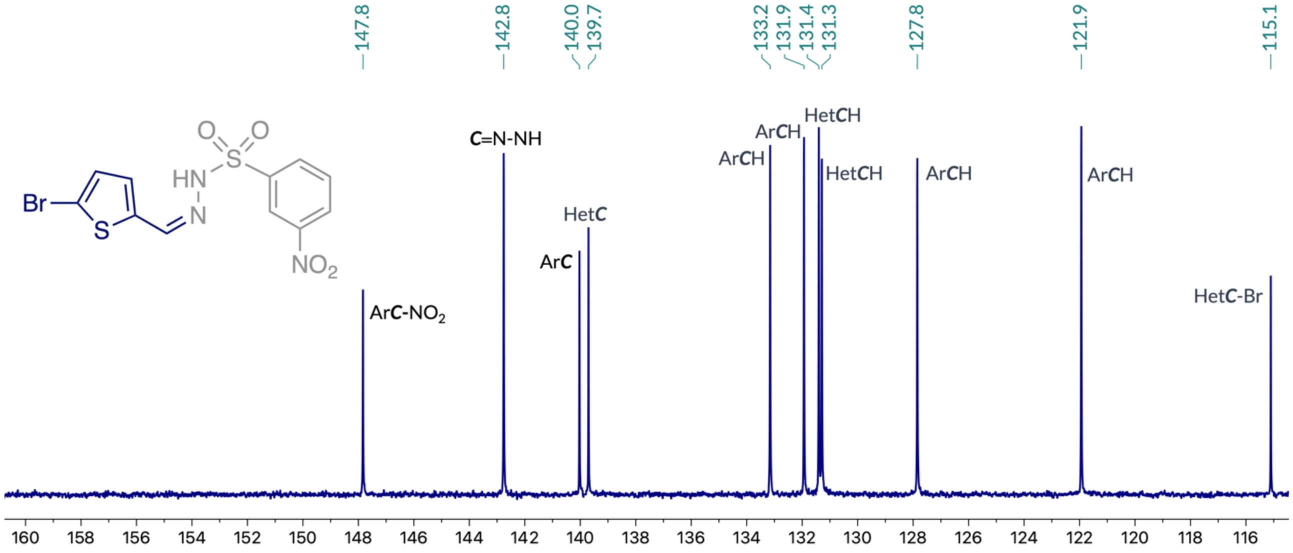

Figure S534. Hydrazone S47: ^{13}C NMR (126 MHz, DMSO-d_6, 298 K)

Other Precursors

Hydrazone S48

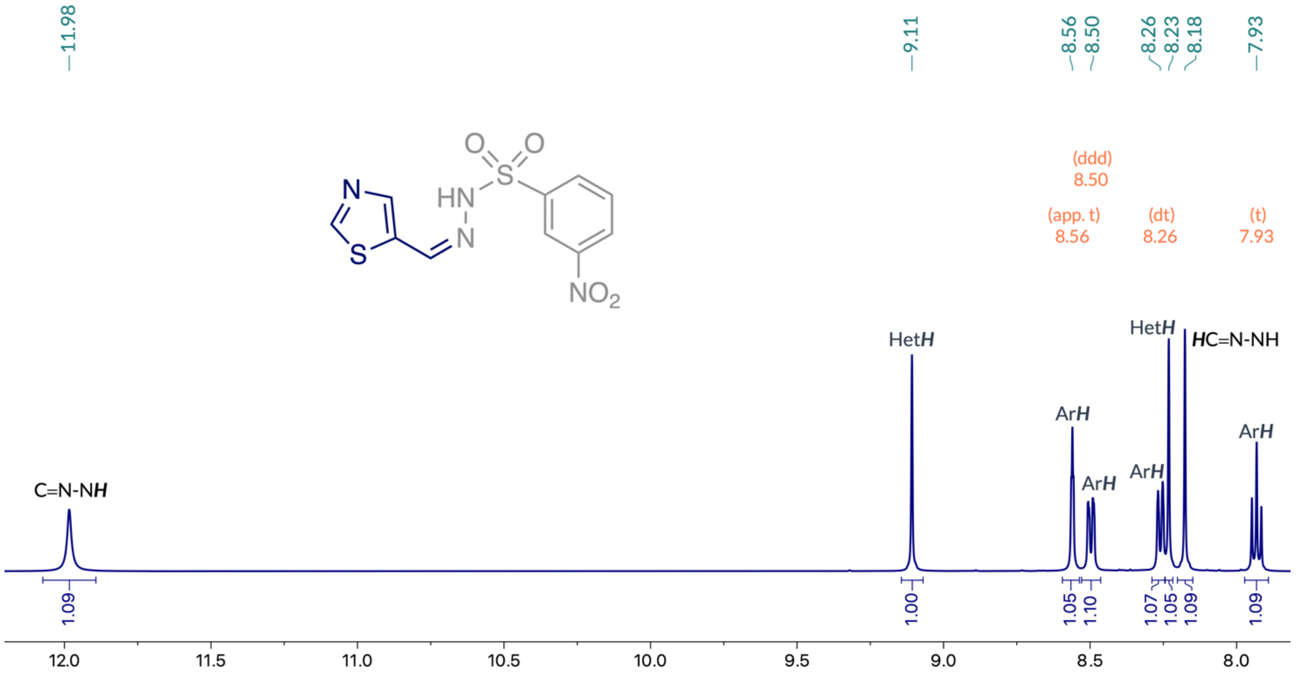

Figure S535. Hydrazone S48: ^{1}H NMR (500 MHz, DMSO-d_6, 298 K)

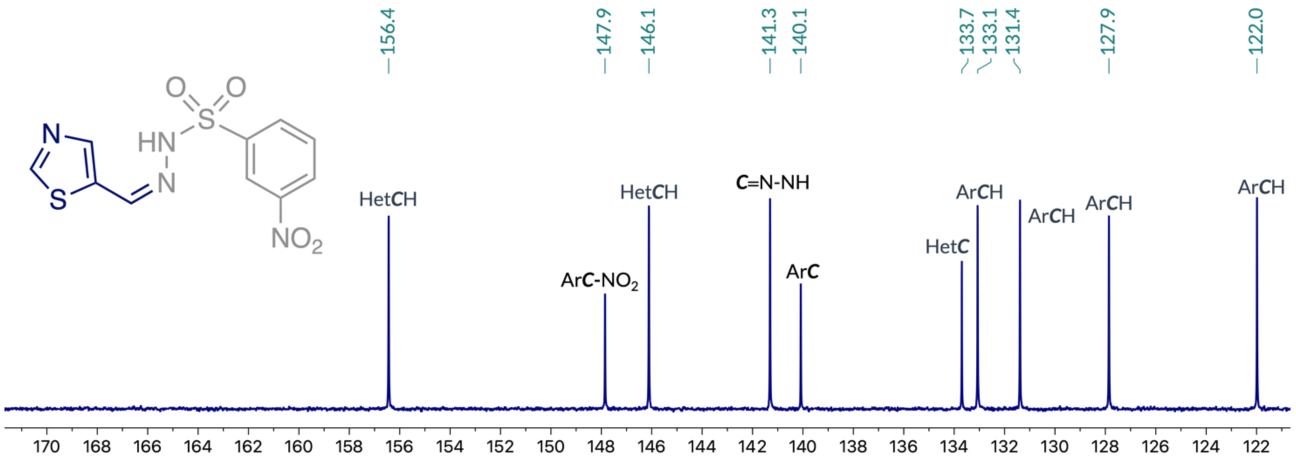

Figure S536. Hydrazone S48: ^{13}C NMR (126 MHz, DMSO-d_6, 298 K)

Hydrazone S49

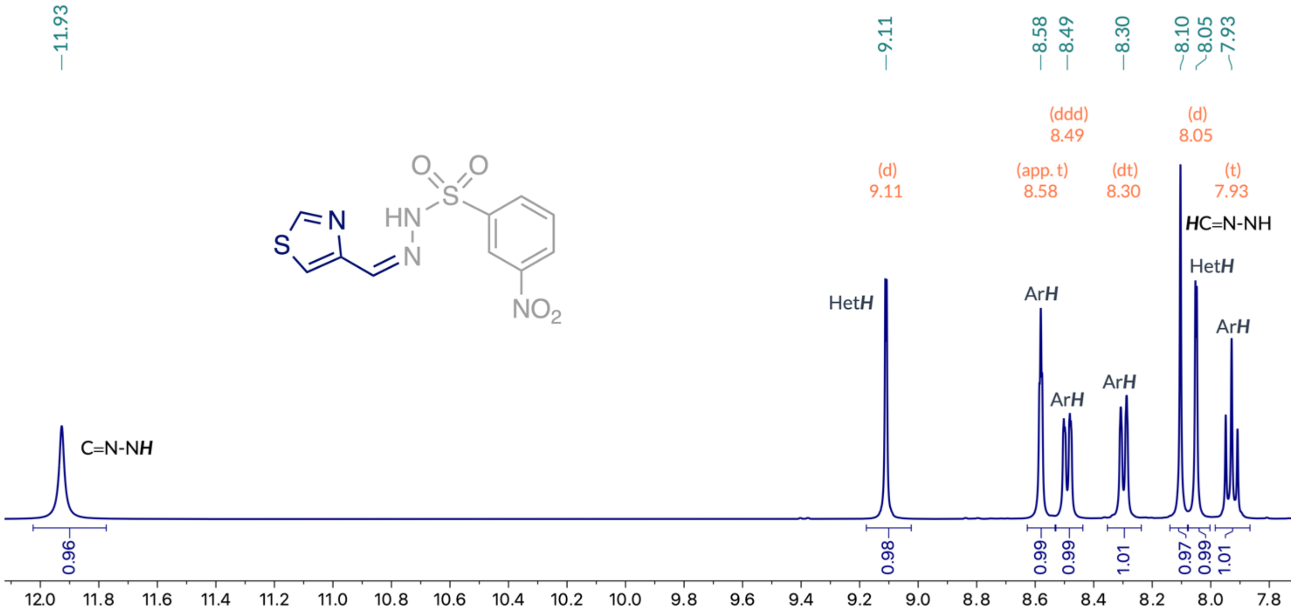

Figure S537. Hydrazone S49: ^{1}H NMR (400 MHz, DMSO-d_6, 298 K)

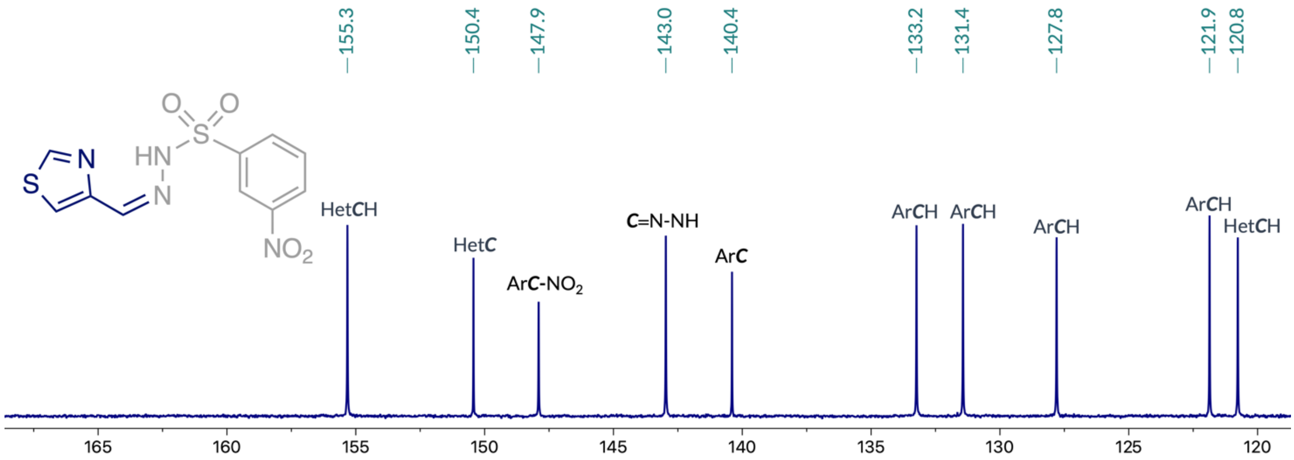

Figure S538. Hydrazone S49: ^{13}C NMR (101 MHz, DMSO-d_6, 298 K)

Hydrazone S50

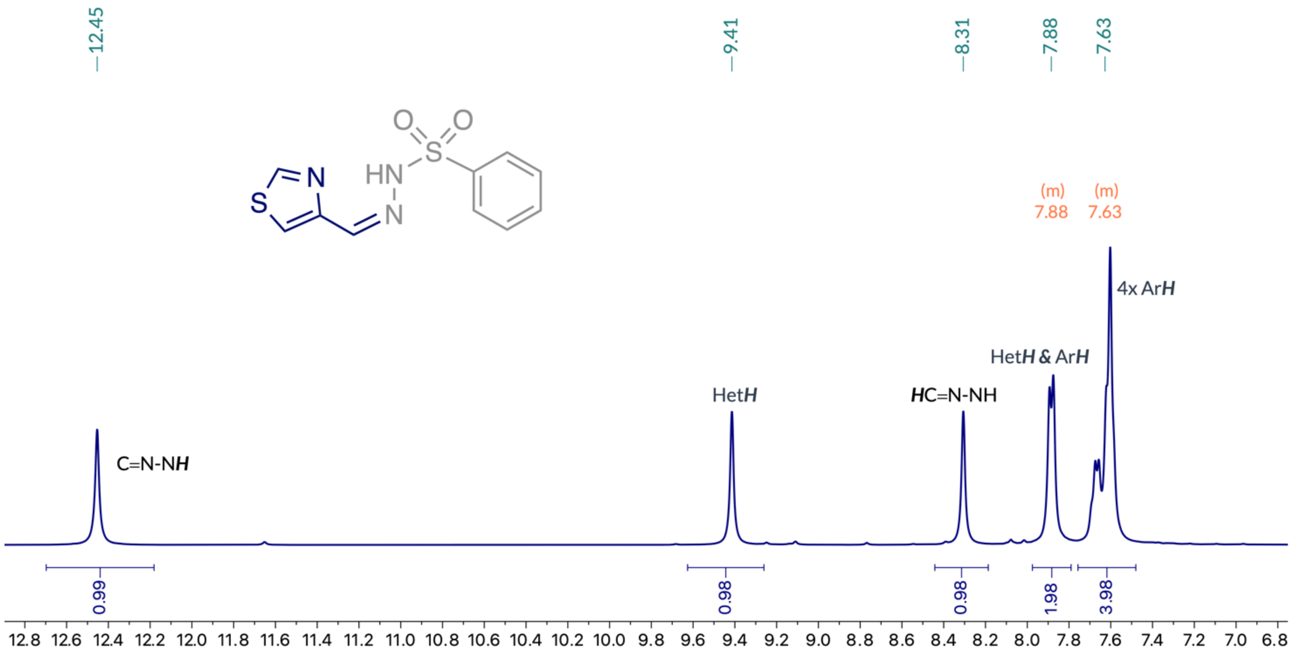

Figure S539. Hydrazone S50: ^{1}H NMR (400 MHz, DMSO-d_6, 298 K)

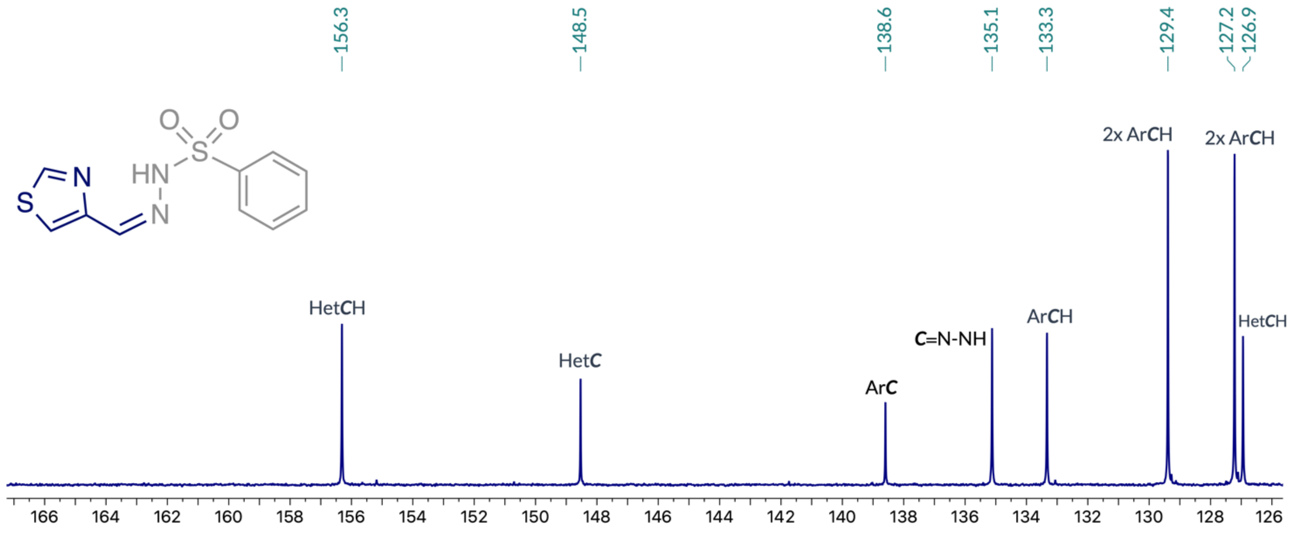

Figure S540. Hydrazone S50: ^{13}C NMR (101 MHz, DMSO-d_6, 298 K)

2-Bromo-5-hydroxybenzaldehyde S51

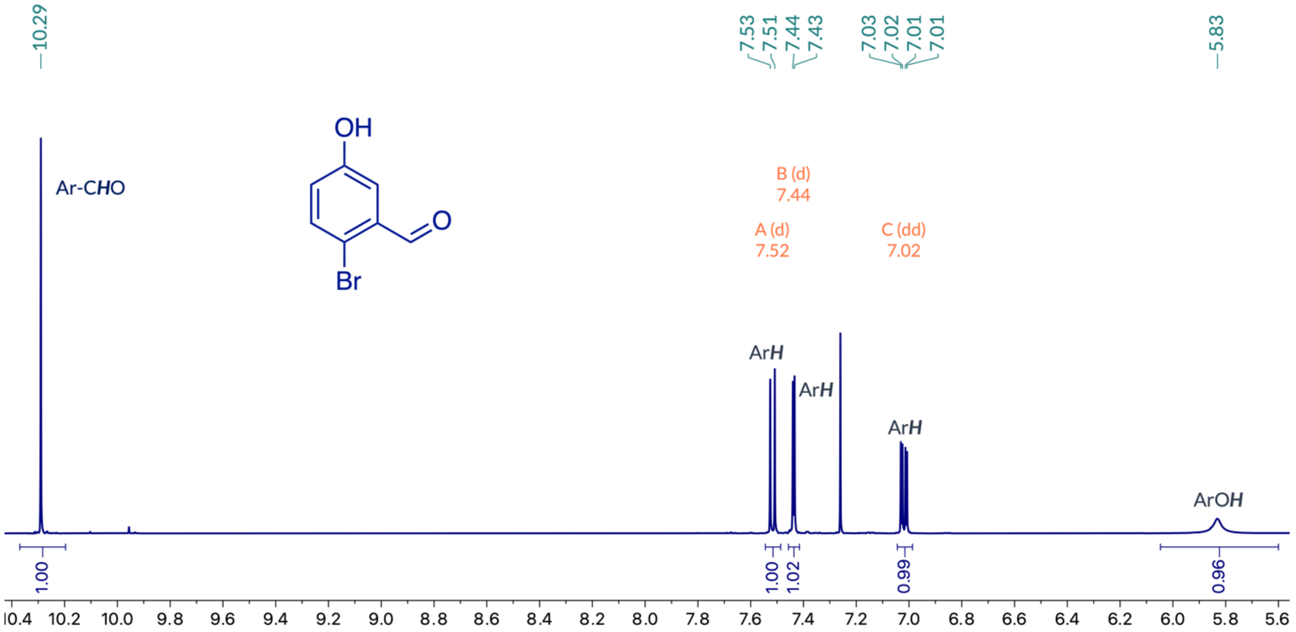

Figure S541. Intermediate S51: ^{1}H NMR (500 MHz, $CDCl_3$, 298 K)

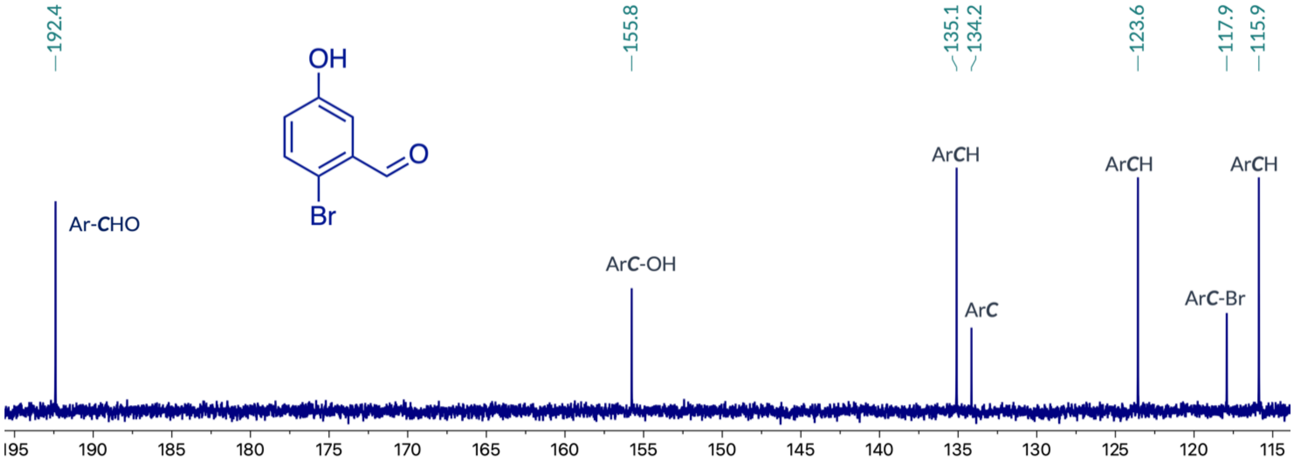

Figure S542. Intermediate S51: ^{13}C NMR (101 MHz, $CDCl_3$, 298 K)

5-Hydroxy-2-(4,4,5,5-tetramethyl-1,3,2-dioxaborolan-2-yl)benzaldehyde S52

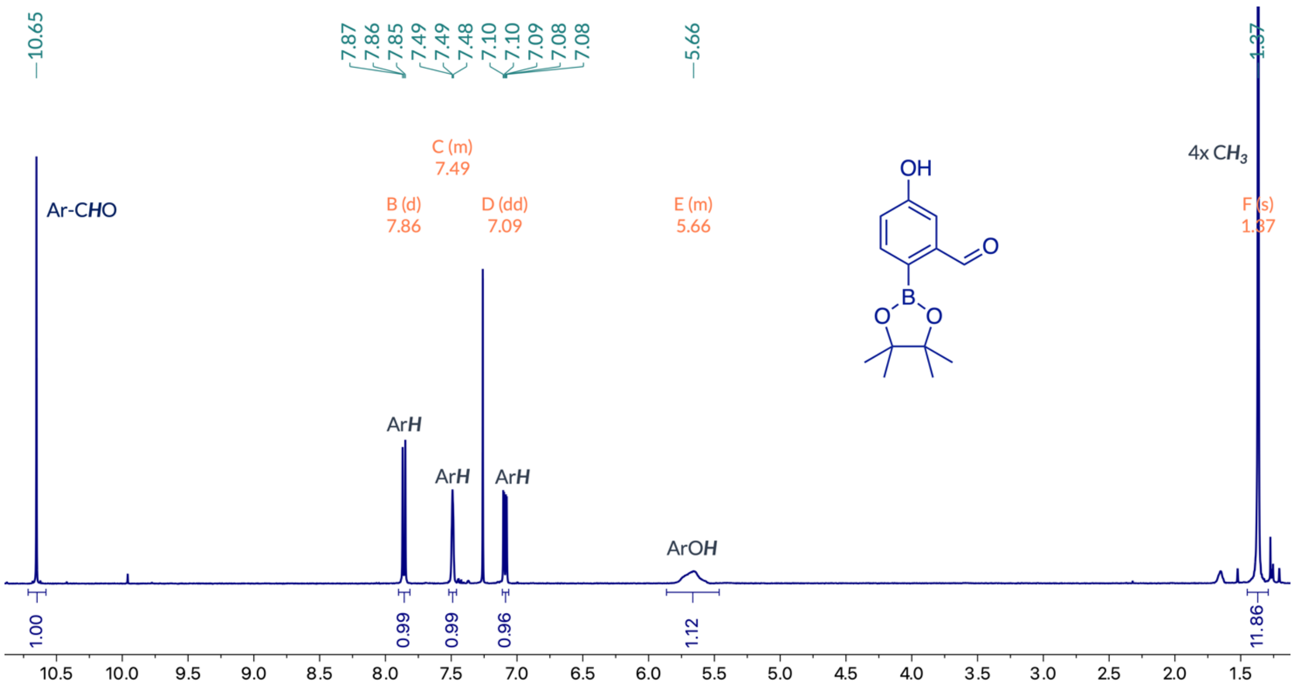

Figure S543. Intermediate S52: ^{1}H NMR (400 MHz, $CDCl_3$, 298 K)

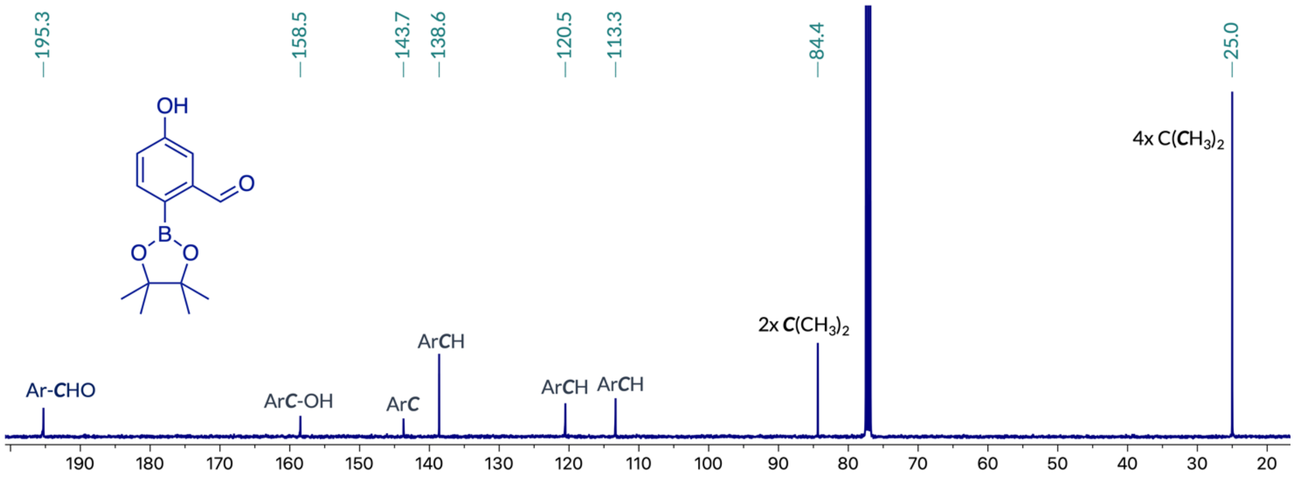

Figure S544. Intermediate S52: ^{13}C NMR (101 MHz, $CDCl_3$, 298 K)

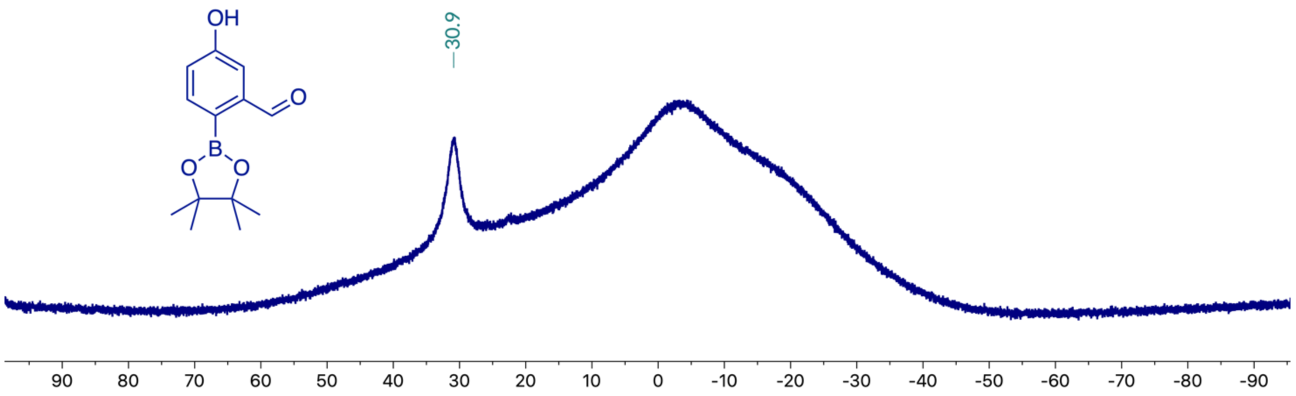

Figure S545. Intermediate S52: ^{11}B NMR (128 MHz, $CDCl_3$, 298 K)

Methyl 2-amino-5-methylthiophene-3-carboxylate S53

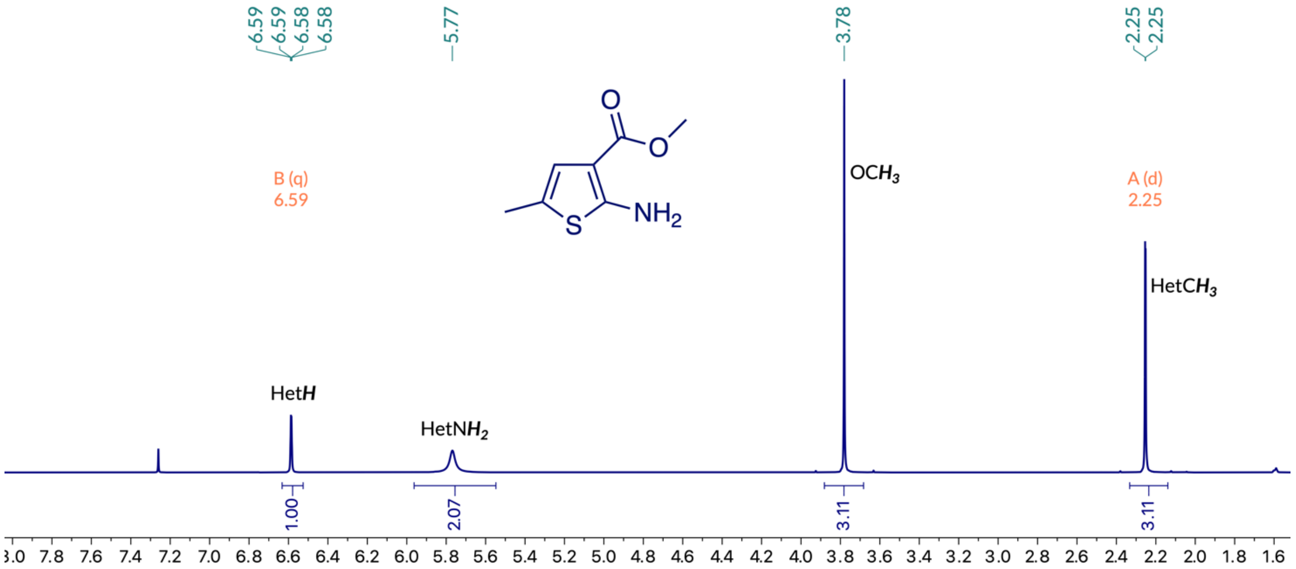

Figure S546. Intermediate S53: ^{1}H NMR (500 MHz, $CDCl_3$, 298 K)

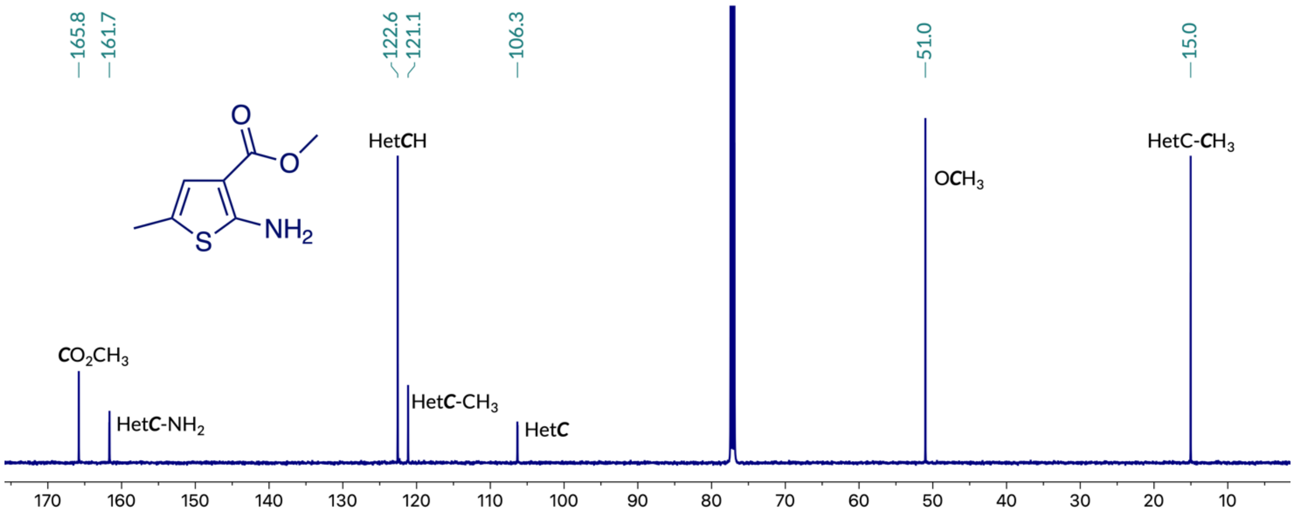

Figure S547. Intermediate S53: ^{13}C NMR (126 MHz, $CDCl_3$, 298 K)

Methyl 2-bromo-5-methylthiophene-3-carboxylate S54

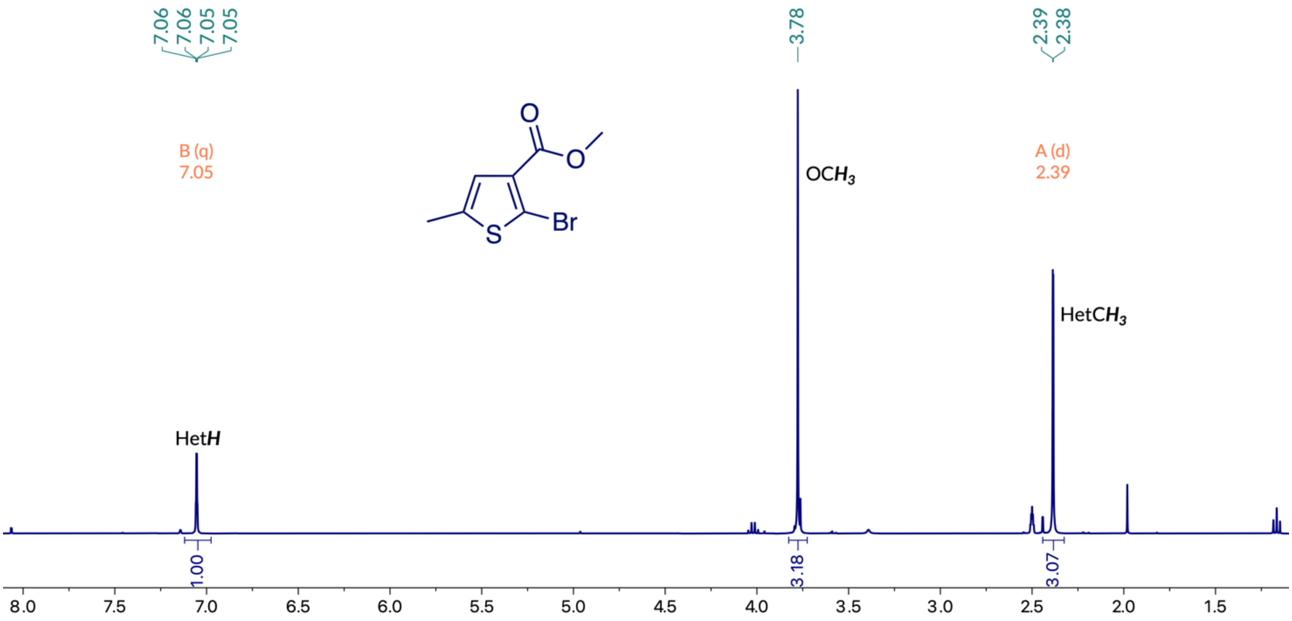

Figure S548. Intermediate S54: ^{1}H NMR (400 MHz, DMSO-d_6, 298 K)

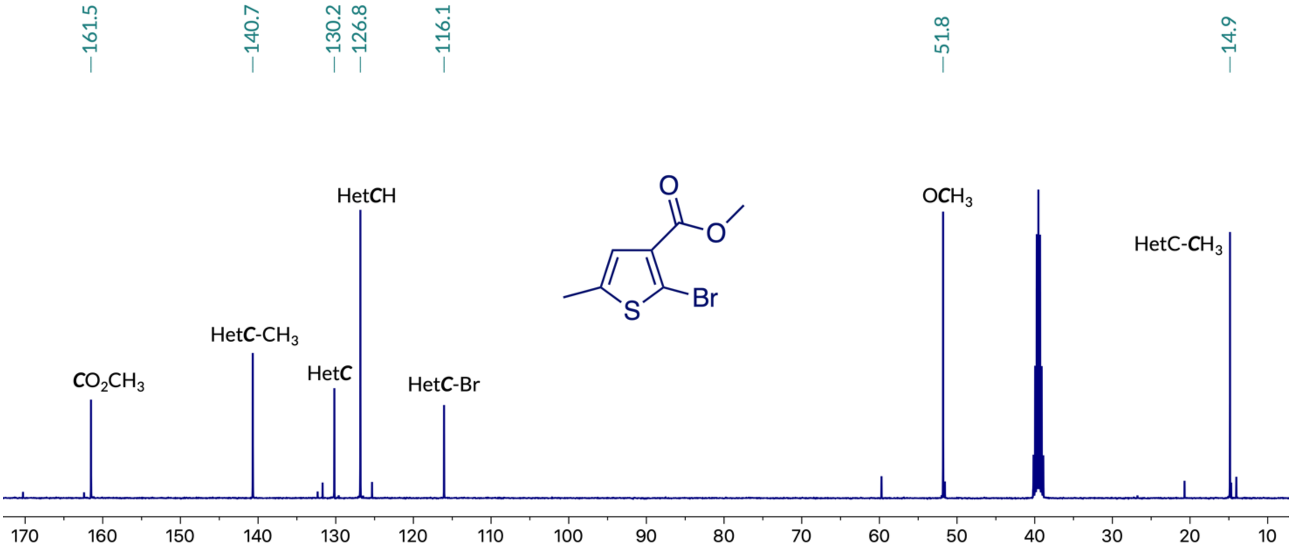

Figure S549. Intermediate S54: ^{13}C NMR (101 MHz, DMSO-d_6, 298 K)

2-Bromo-5-methylthiophene-3-carbaldehyde S55

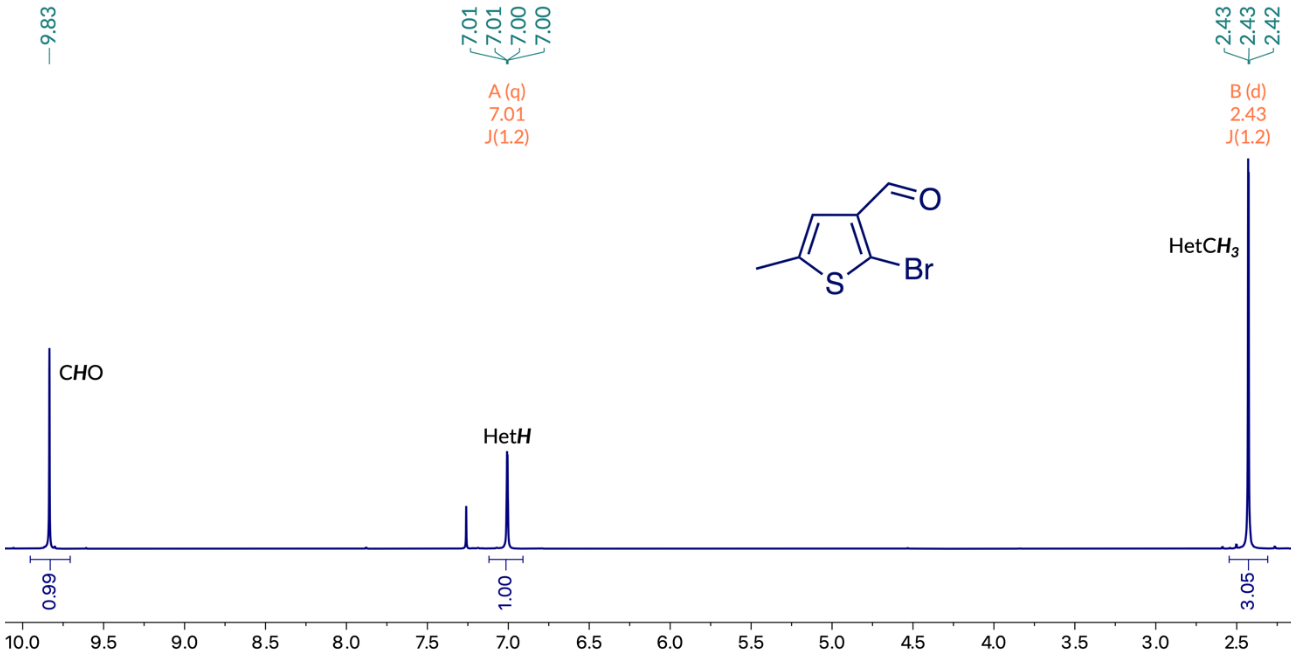

Figure S550. Intermediate S55: ^{1}H NMR (400 MHz, $CDCl_3$, 298 K)

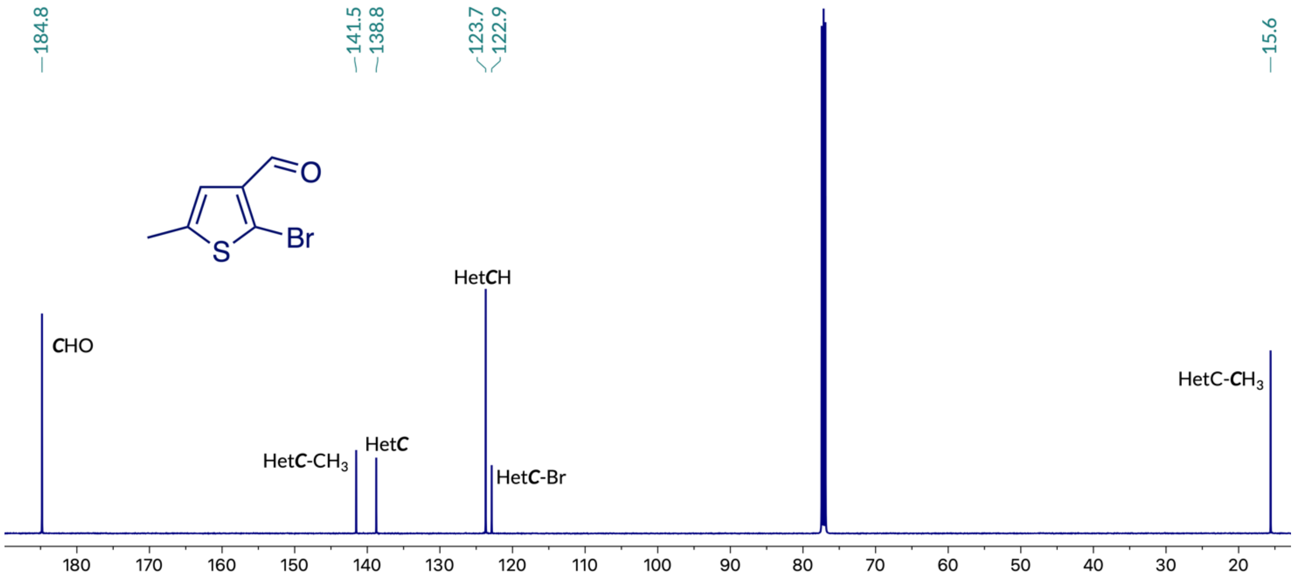

Figure S551. Intermediate S55: ^{13}C NMR (126 MHz, $CDCl_3$, 298 K)

2-(2-Bromo-5-methylthiophen-3-yl)-1,3-dioxolane S56

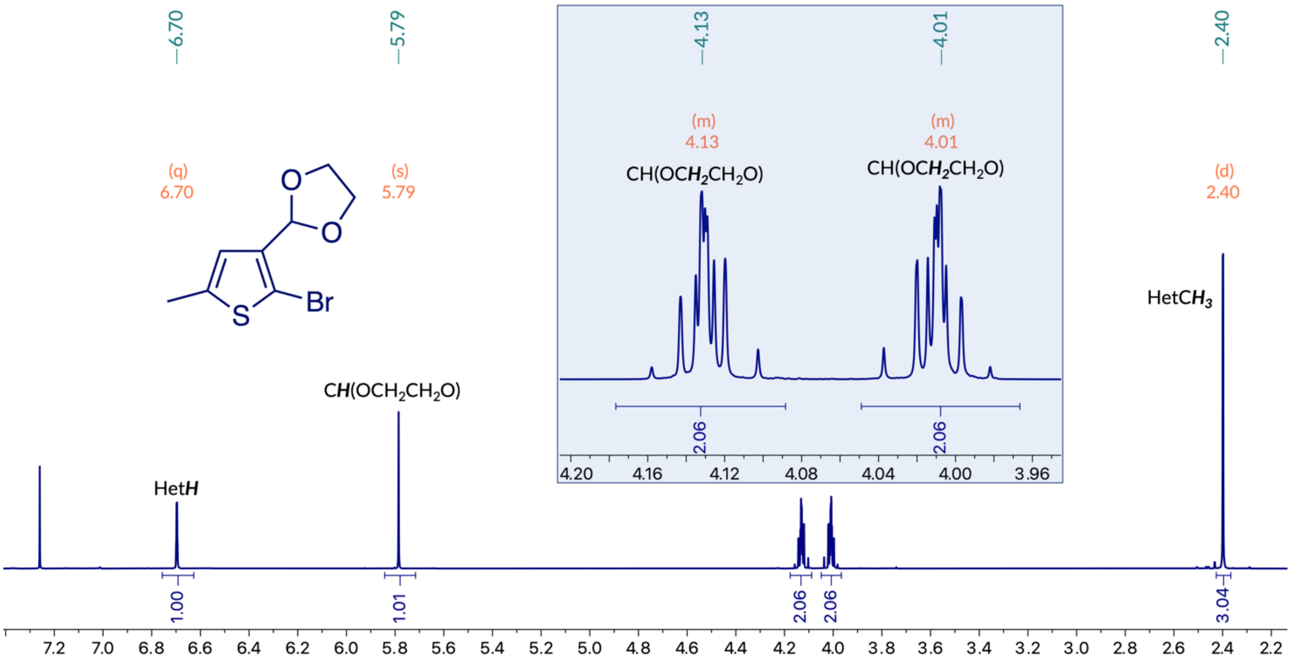

Figure S552. Intermediate S56: ^{1}H NMR (600 MHz, $CDCl_3$, 298 K)

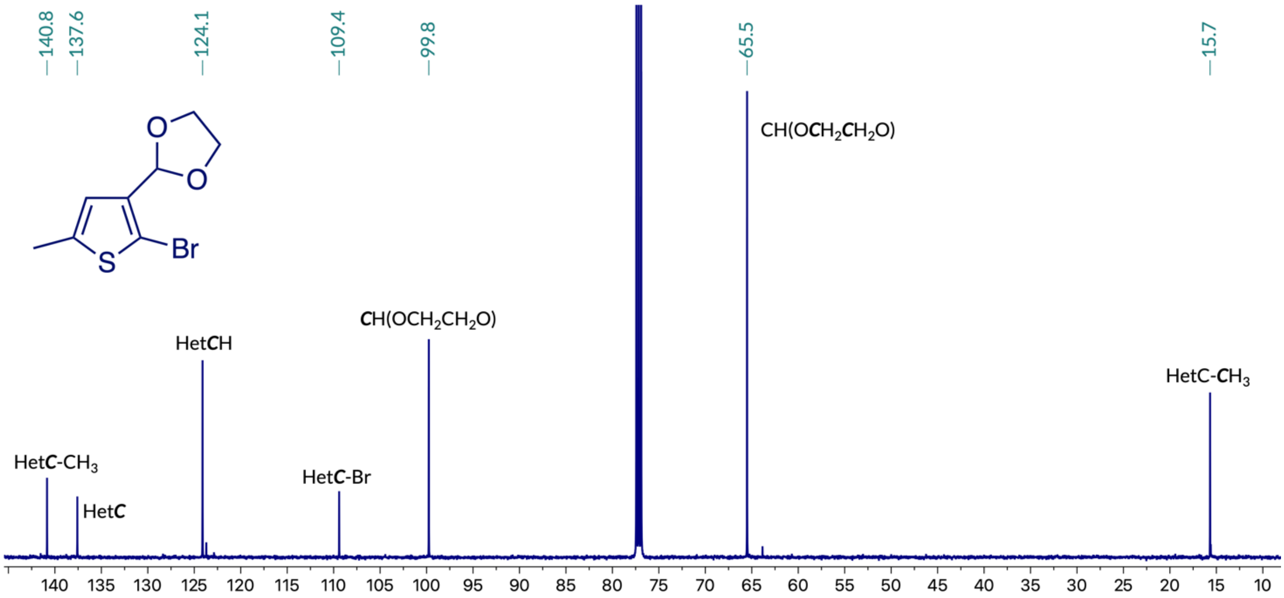

Figure S553. Intermediate S56: ^{13}C NMR (126 MHz, $CDCl_3$, 298 K)

(3-Formyl-5-methylthiophen-2-yl)boronic acid S57

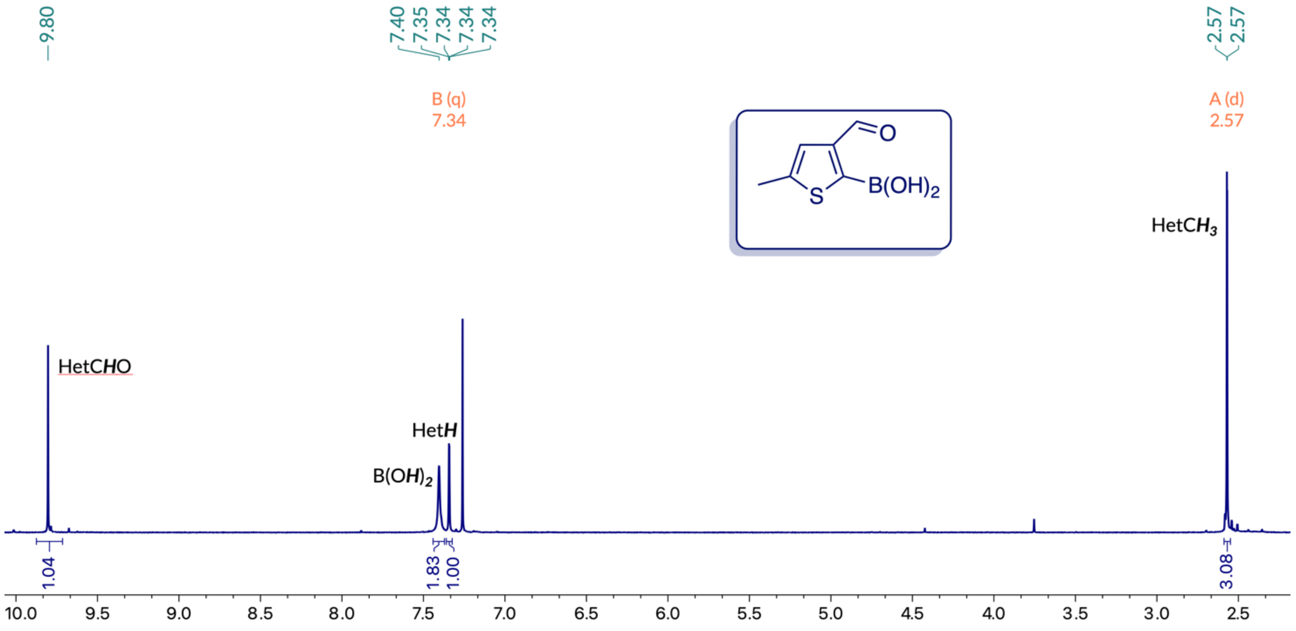

Figure S554. Intermediate S57: ^{1}H NMR (400 MHz, $CDCl_3$, 298 K)

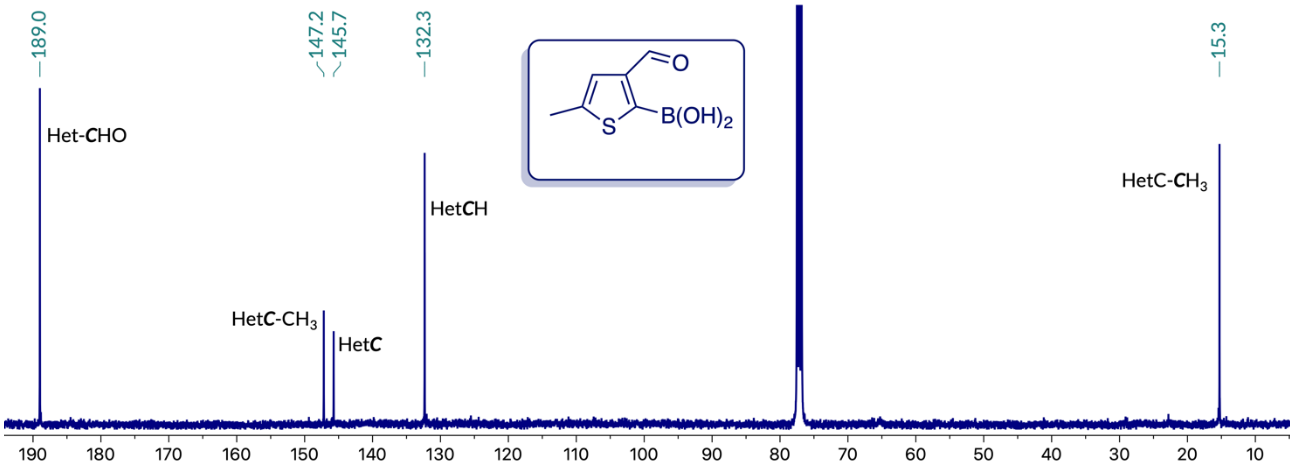

Figure S555. Intermediate S57: ^{13}C NMR (101 MHz, $CDCl_3$, 298 K)

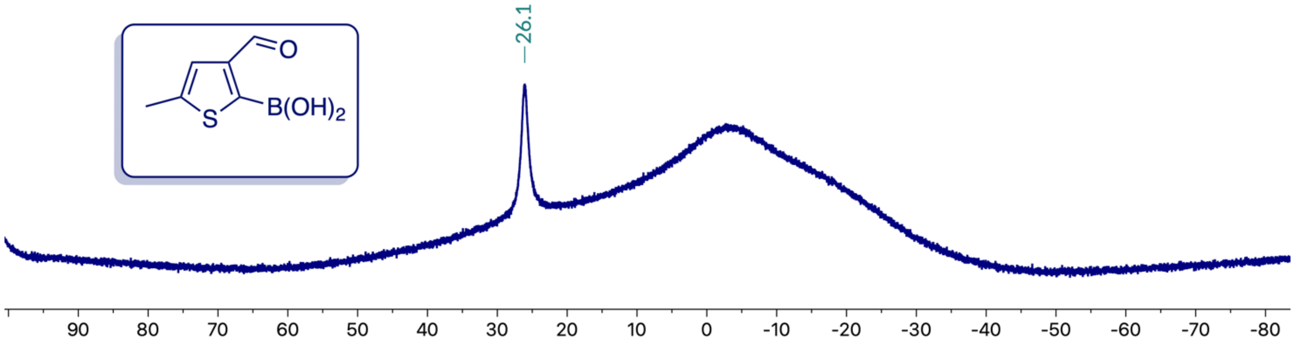

Figure S556. Intermediate S57: ^{11}B NMR (128 MHz, $CDCl_3$, 298 K)

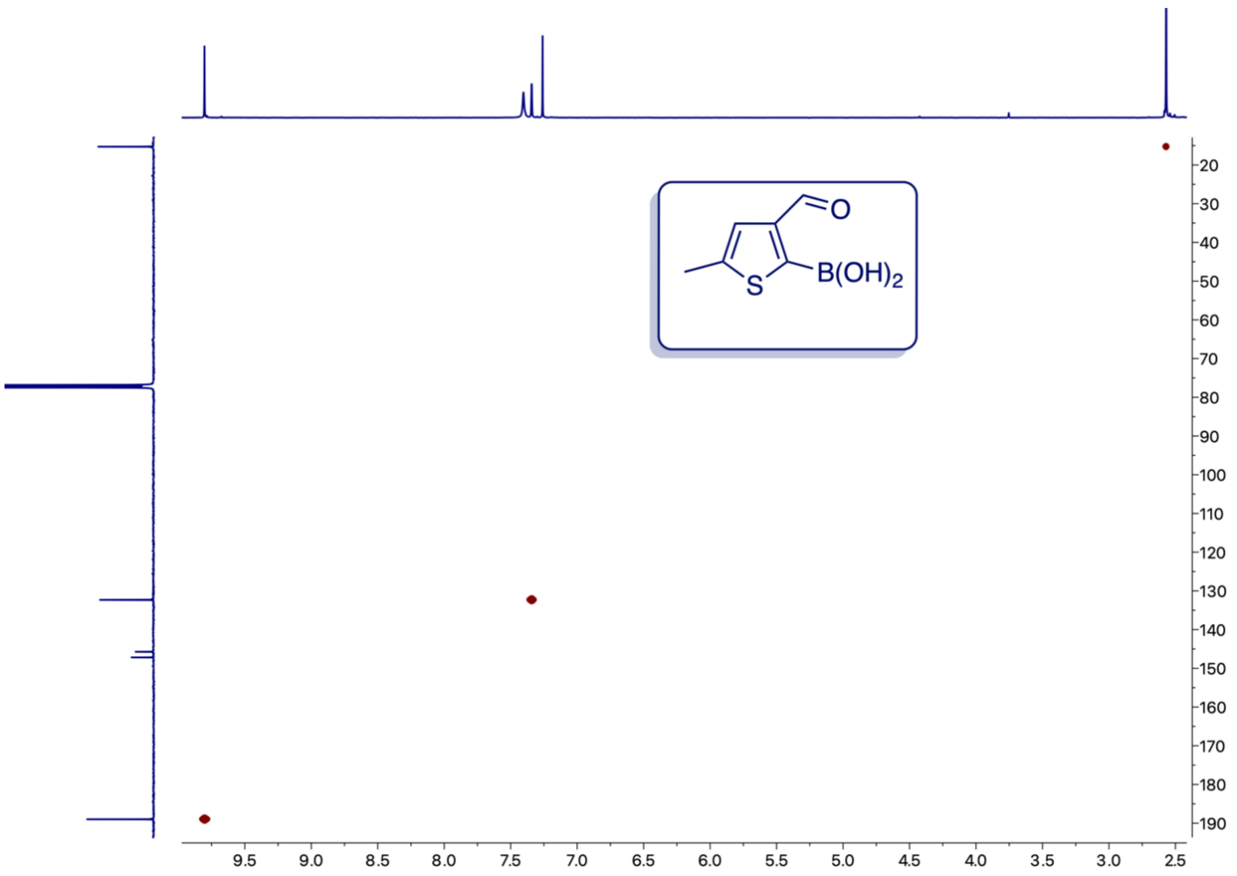

Figure S557. Intermediate S57:^{1}H-^{13}C gHSQC NMR ($CDCl_3$, 298 K)

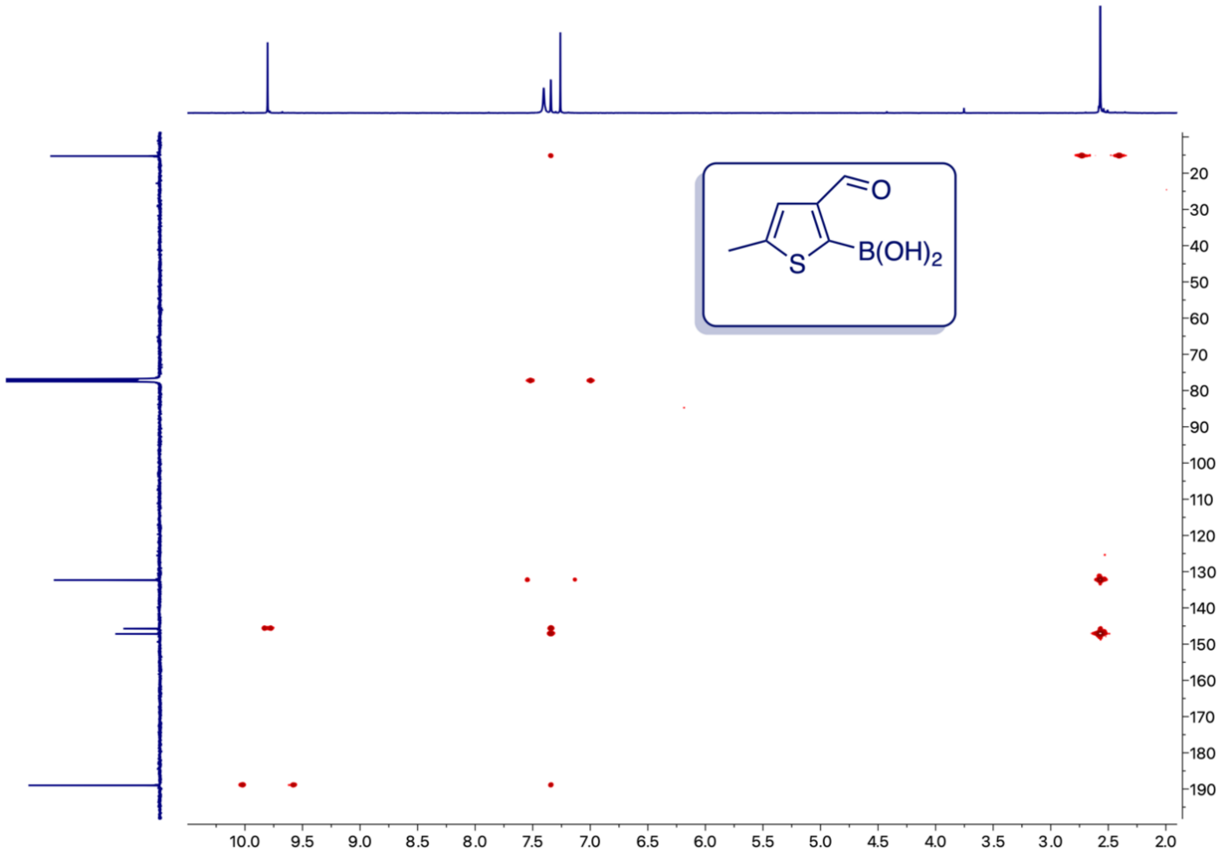

Figure S558. Intermediate S57:^{1}H-^{13}C gHMBC NMR ($CDCl_3$, 298 K)

(6-Carboxyhexyl)triphenylphosphonium bromide S59

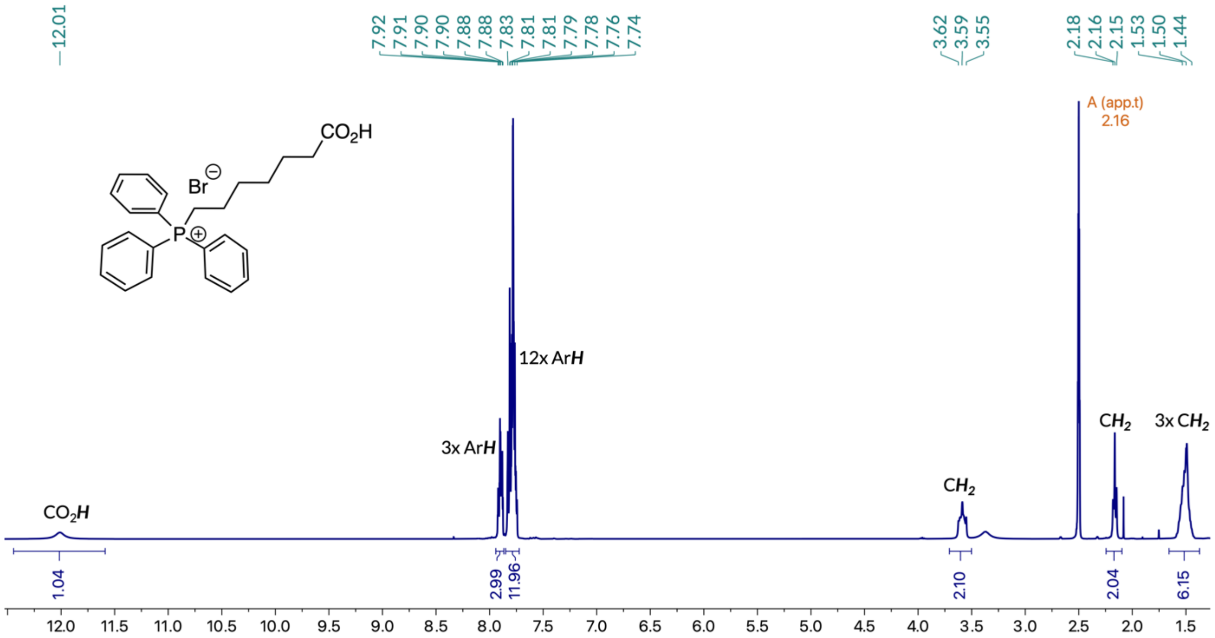

Figure S559. Intermediate S59: ^{1}H NMR (400 MHz, DMSO-d_6, 298 K)

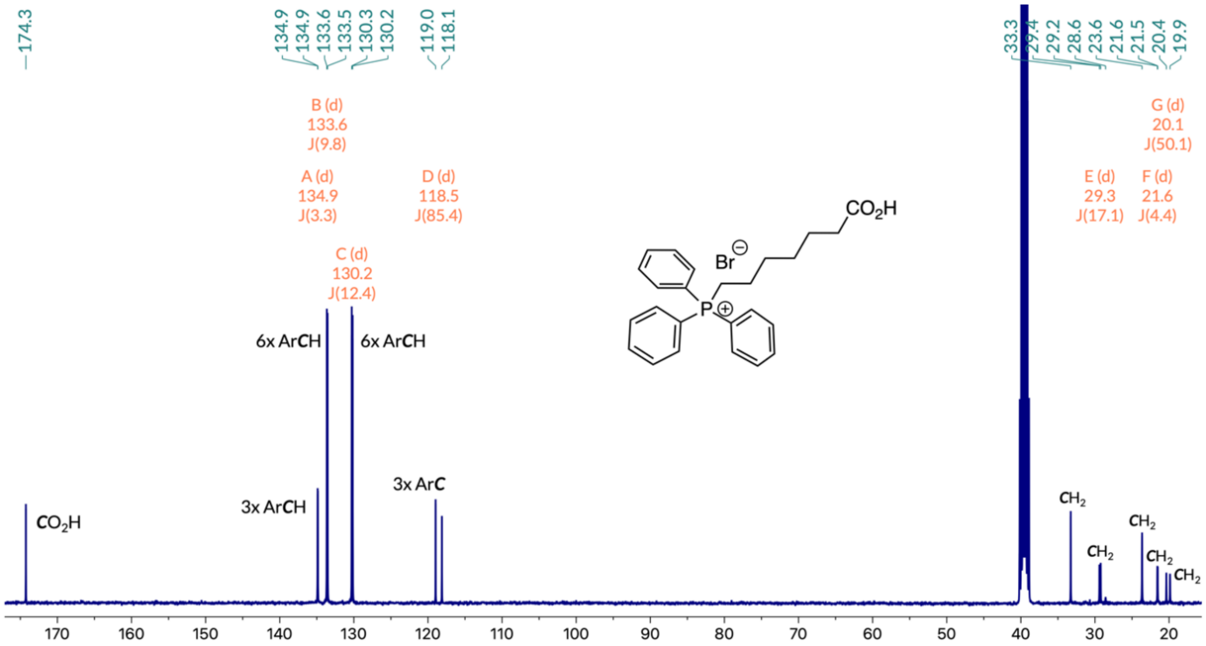

Figure S560. Intermediate S59: ^{13}C NMR (101 MHz, DMSO-d_6, 298 K)

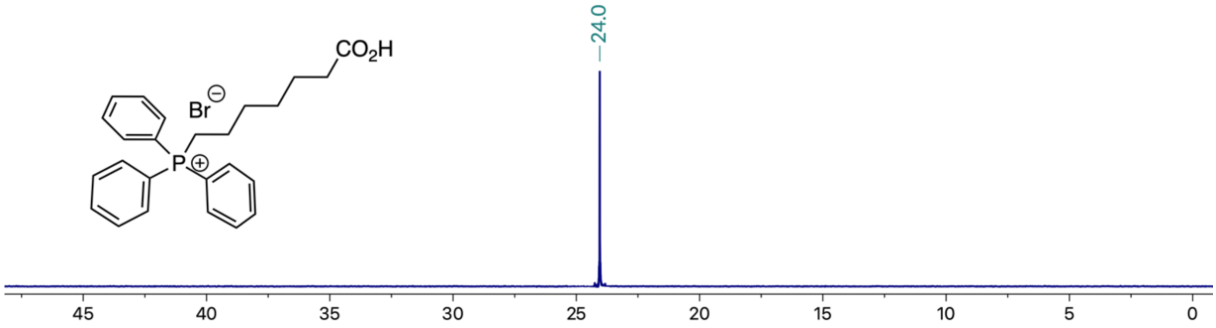

Figure S561. Intermediate S59: $^{31}P\{^{1}H\}$ NMR (162 MHz, DMSO-d_6, 298 K)

Stability studies

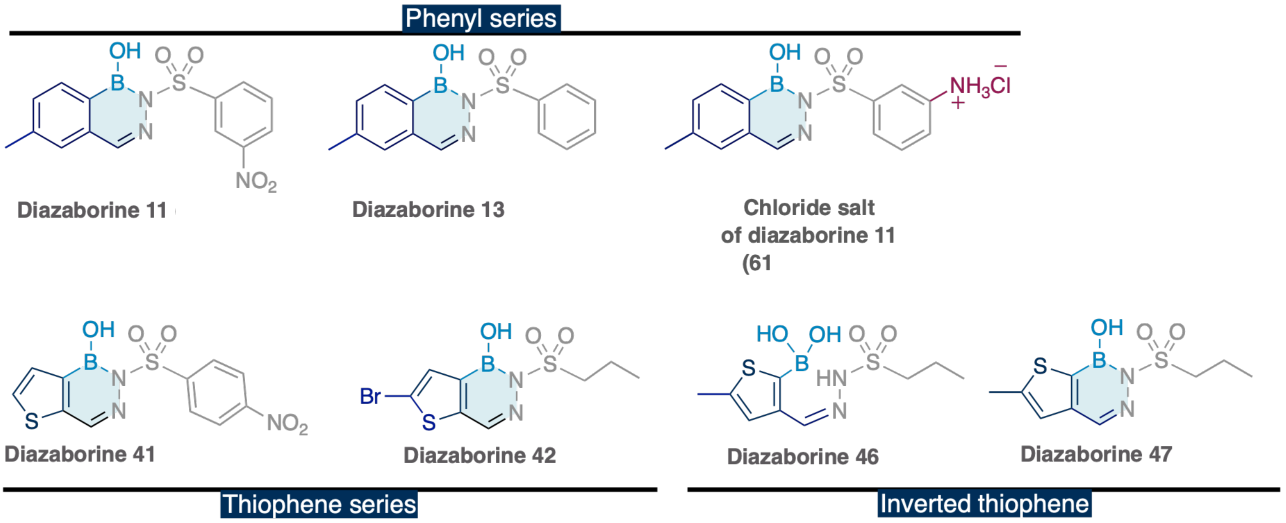

Figure S562. Selected compounds whose stability was evaluated in the freezing-defreezing studies

Freezing-defreezing studies

Diazaborine 11

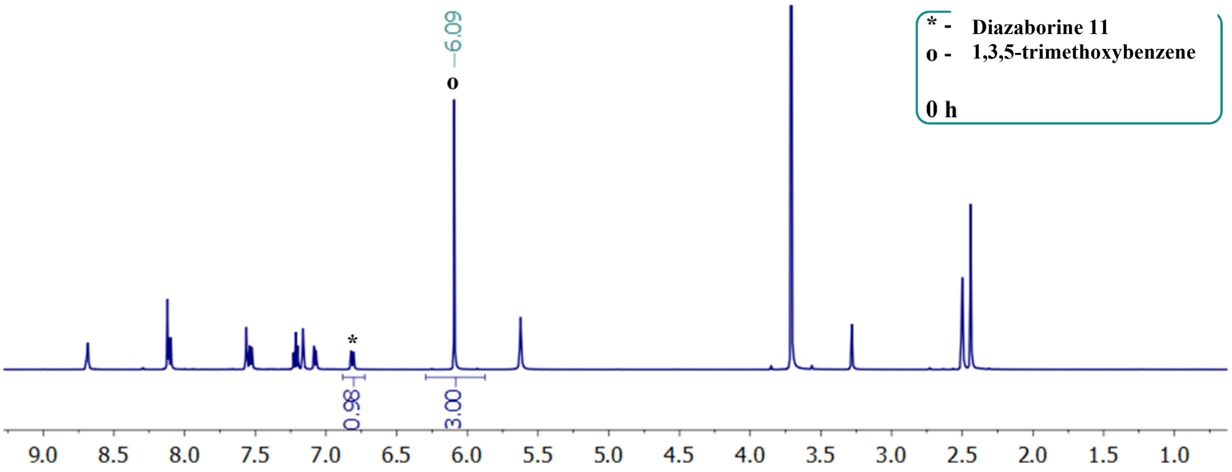

Figure S563. Initial ^{1}H NMR (600 MHz, DMSO-d_6, 310 K) spectrum of a 1:1 mixture of **Diazaborine 11** and 1,3,5-trimethoxybenzene as internal standard

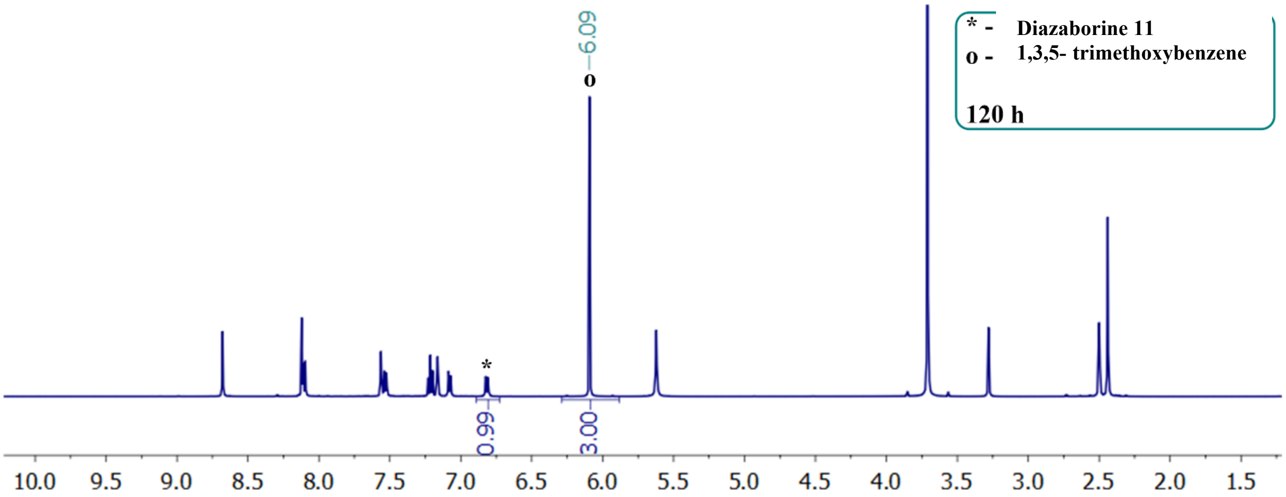

Figure S564. ^{1}H NMR (600 MHz, DMSO-d_6, 310 K) spectrum of a 1:1 mixture of **Diazaborine 11** and 1,3,5-trimethoxybenzene as internal standard after 120 h (5 cycles of freezing-defreezing)

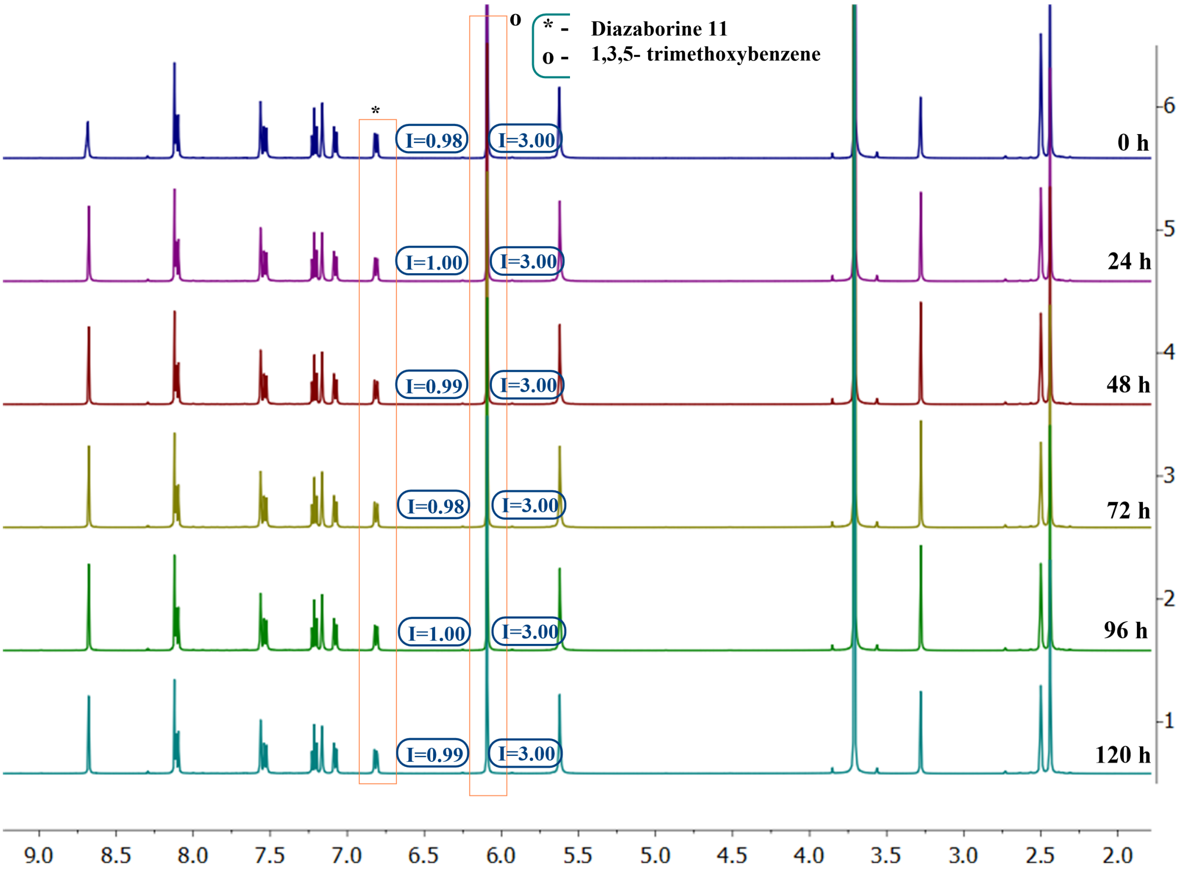

Figure S565. Stacked ^{1}H NMR spectra (600 MHz, DMSO-d_6, 310 K) of a 1:1 mixture of **Diazaborine 11** and 1,3,5-trimethoxybenzene as internal standard after 120 h (5 cycles of freezing-defreezing)

Diazaborine 13

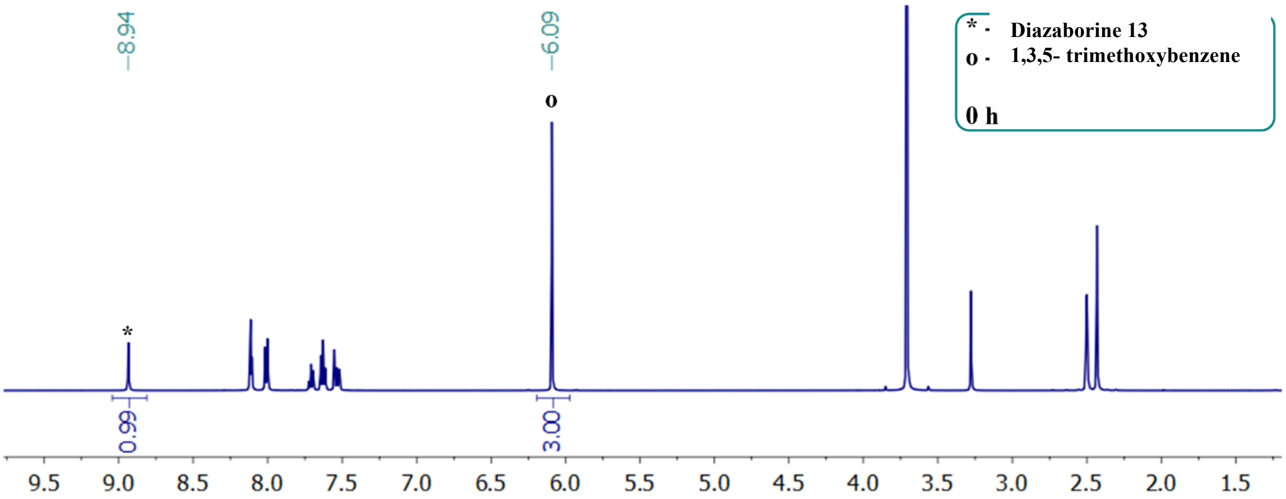

Figure S566. Initial ^{1}H NMR spectrum (600 MHz, DMSO-d_6, 310 K) of a 1:1 mixture of **Diazaborine 13** and 1,3,5-trimethoxybenzene as internal standard

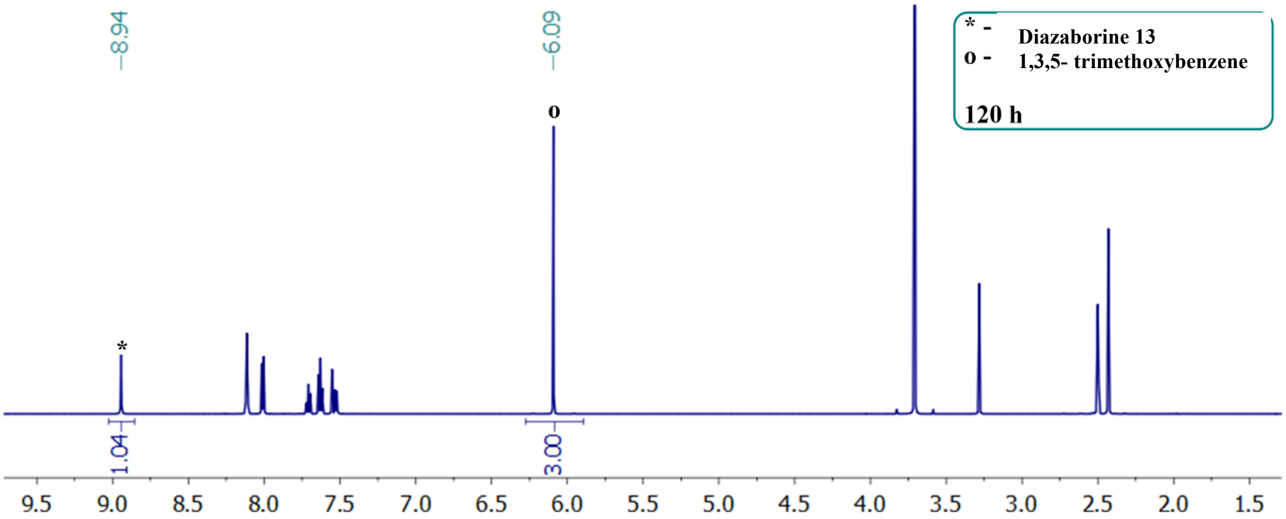

Figure S567. ^{1}H NMR spectrum (600 MHz, DMSO-d_6, 310 K) of a 1:1 mixture of **Diazaborine 13** and 1,3,5-trimethoxybenzene as internal standard after 120 h (5 cycles of freezing-defreezing)

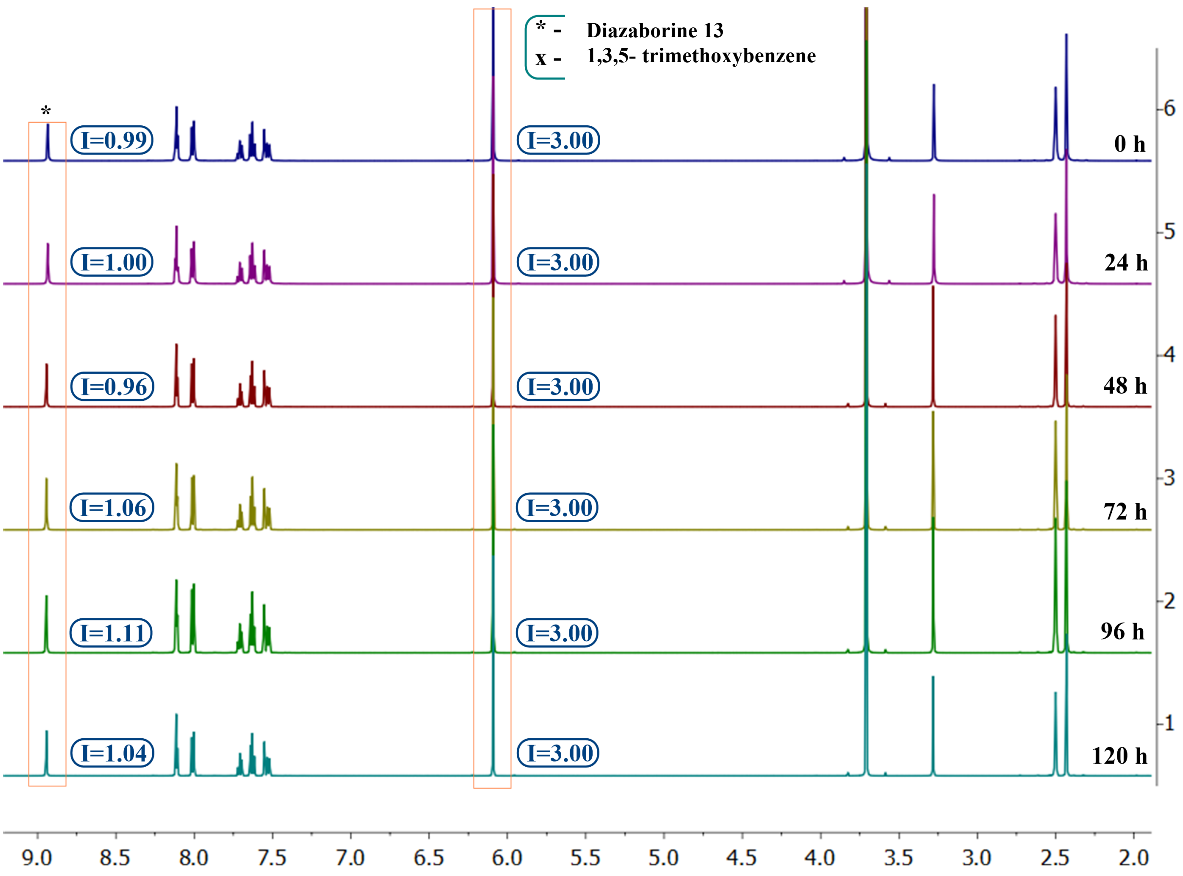

Figure S568. Stacked ^{1}H NMR spectra (600 MHz, DMSO-d_6, 310 K) of a 1:1 mixture of **Diazaborine 13** and 1,3,5-trimethoxybenzene as internal standard after 120 h (5 cycles of freezing-defreezing)

Diazaborine 11 derivative 61

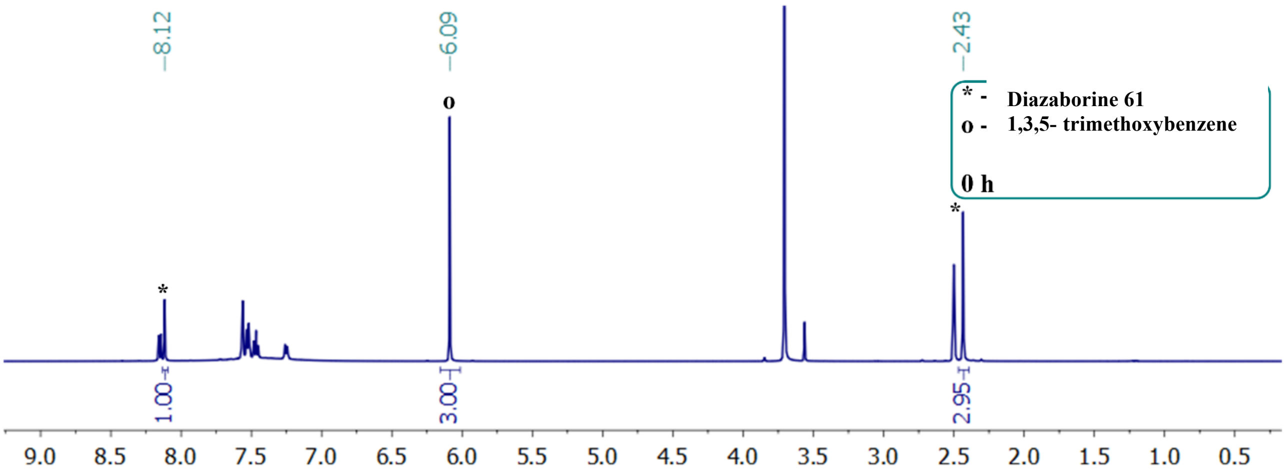

Figure S569. Initial ^{1}H NMR spectrum (600 MHz, DMSO-d_6, 310 K) of a 1:1 mixture of **Diazaborine 11 derivative 61** and 1,3,5-trimethoxybenzene as internal standard

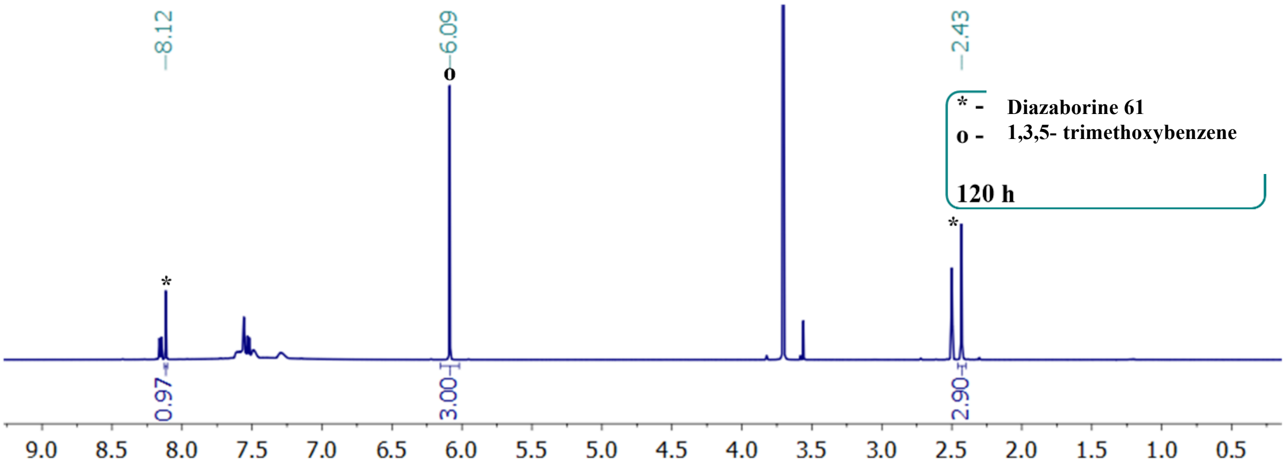

Figure S570. ^{1}H NMR spectrum (600 MHz, DMSO-d_6, 310 K) of a 1:1 mixture of **Diazaborine 11 derivative 61** and 1,3,5-trimethoxybenzene as internal standard after 120 h (5 cycles of freezing-defreezing)

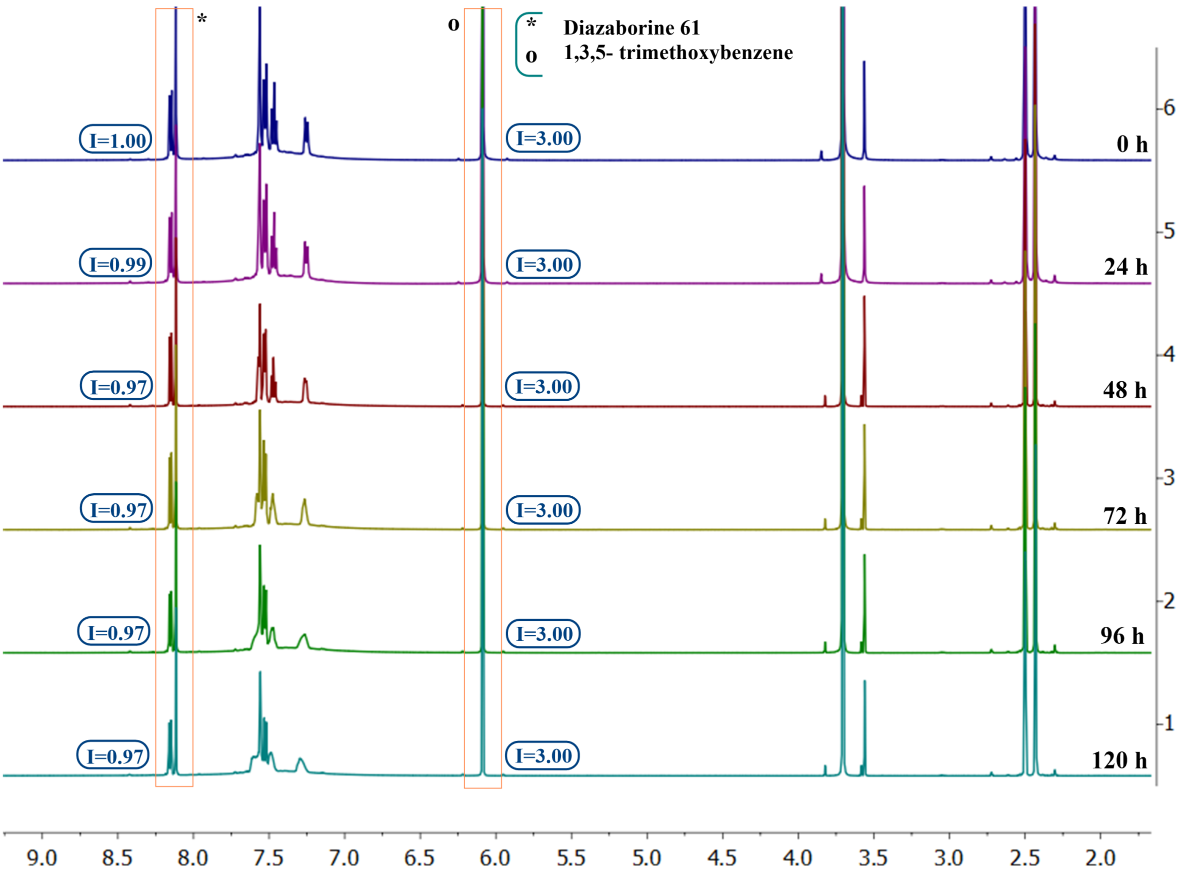

Figure S571. Stacked ^{1}H NMR spectra (600 MHz, DMSO-d_6, 310 K) of a 1:1 mixture of **Diazaborine 11 derivative 61** and 1,3,5-trimethoxybenzene as internal standard after 120 h (5 cycles of freezing-defreezing)

Diazaborine 41

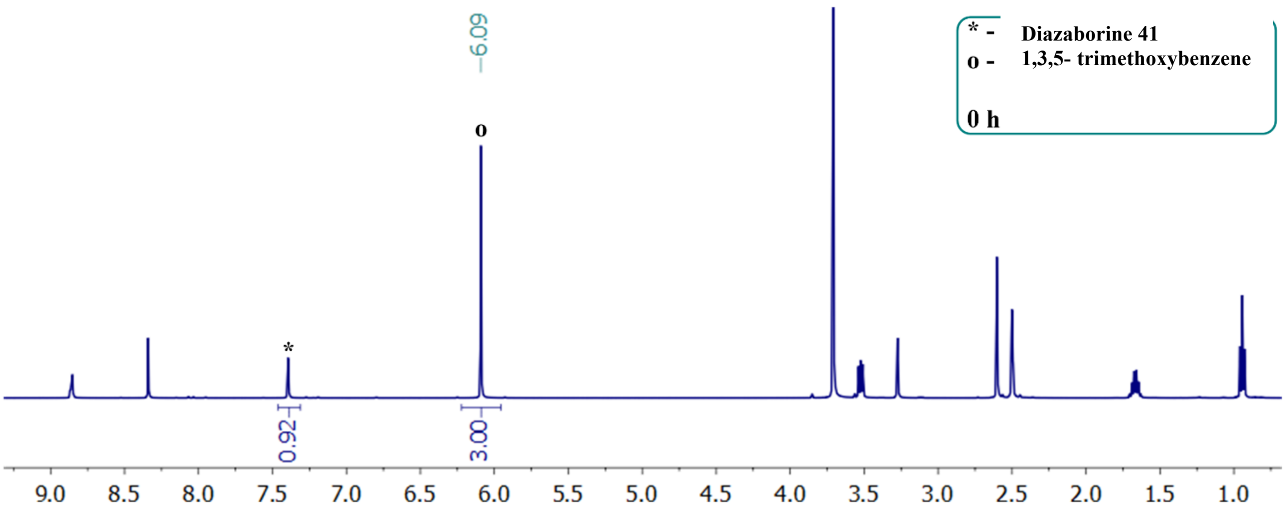

Figure S572. Initial ^{1}H NMR spectrum (600 MHz, DMSO-d_6, 310 K) of a 1:1 mixture of **Diazaborine 41** and 1,3,5-trimethoxybenzene as internal standard

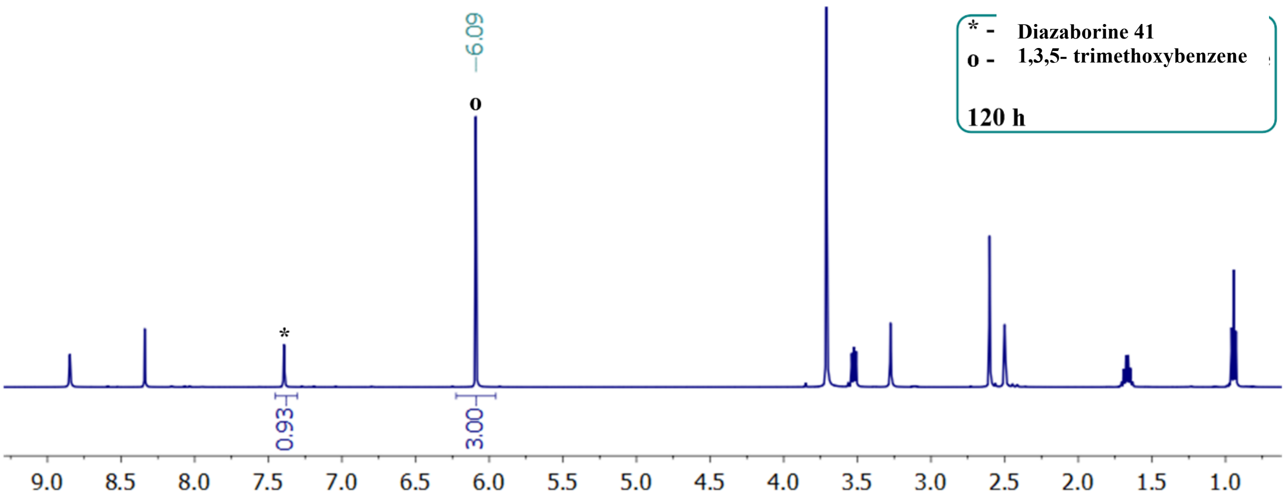

Figure S573. ^{1}H NMR spectrum (600 MHz, DMSO-d_6, 310 K) of a 1:1 mixture of **Diazaborine 41** and 1,3,5-trimethoxybenzene as internal standard after 120 h (5 cycles of freezing-defreezing)

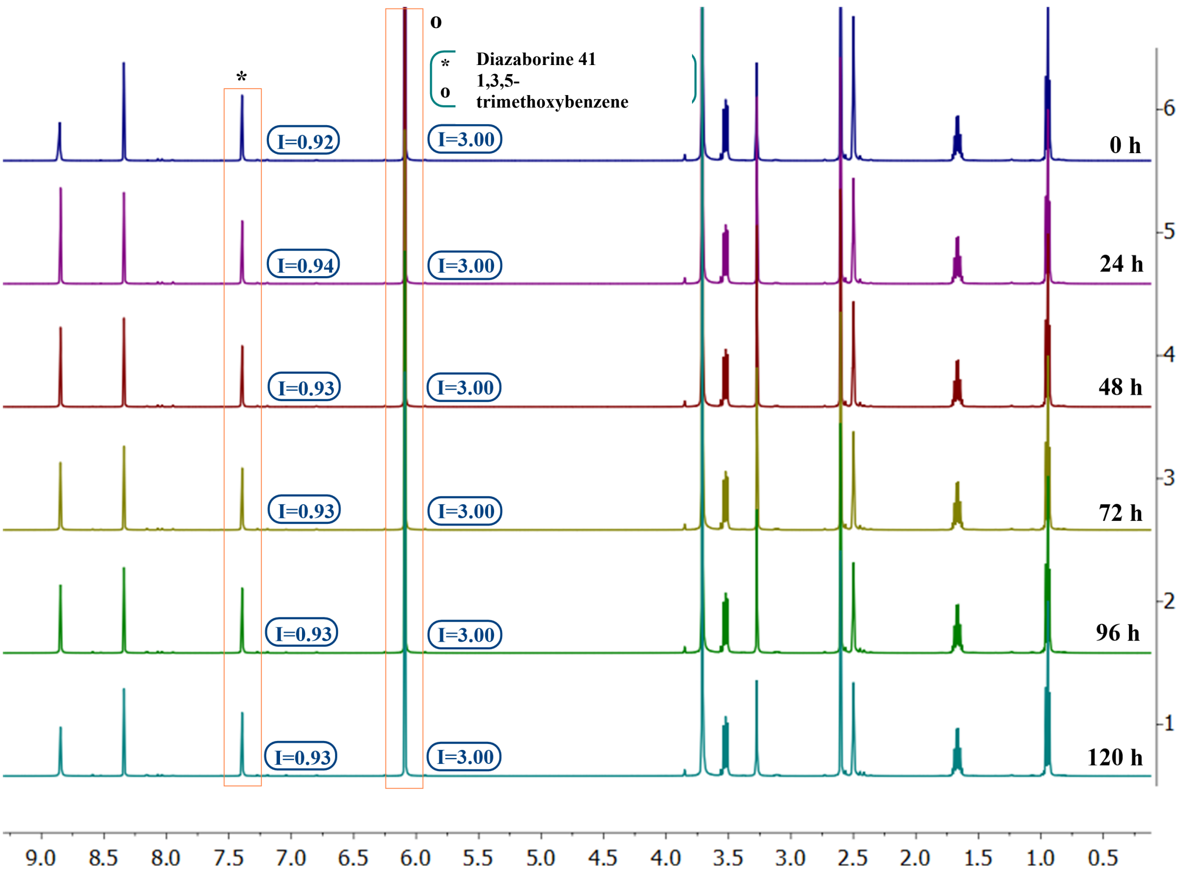

Figure S574. Stacked ^{1}H NMR spectra (600 MHz, DMSO-d_6, 310 K) of a 1:1 mixture of **Diazaborine 41** and 1,3,5-trimethoxybenzene as internal standard after 120 h (5 cycles of freezing-defreezing)

Diazaborine 42

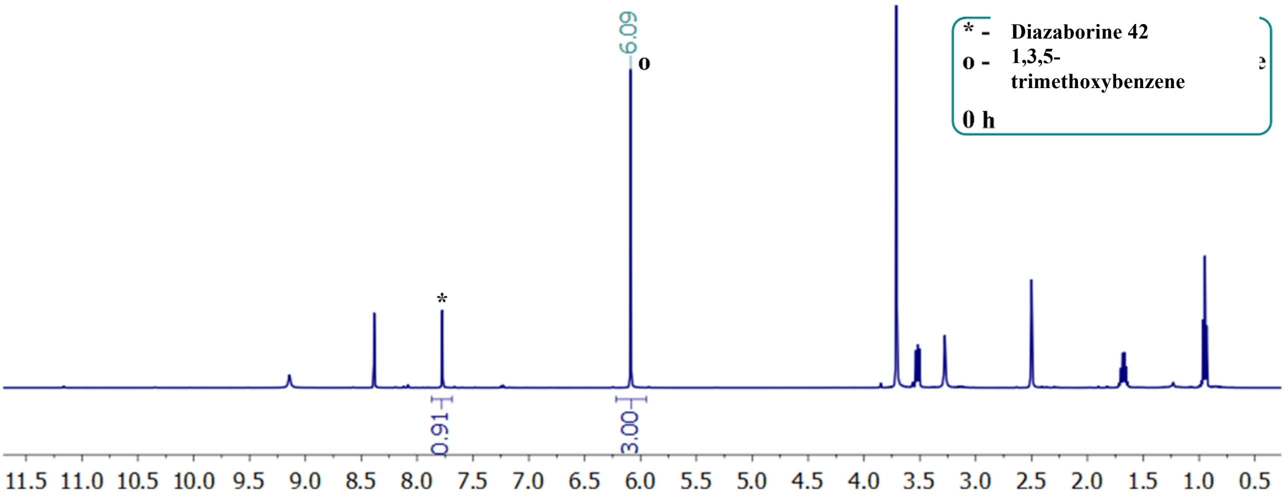

Figure S575. Initial ^{1}H NMR spectrum (500 MHz, DMSO-d_6, 310 K) of a 1:1 mixture of diazaborine **42** and 1,3,5-trimethoxybenzene as internal standard

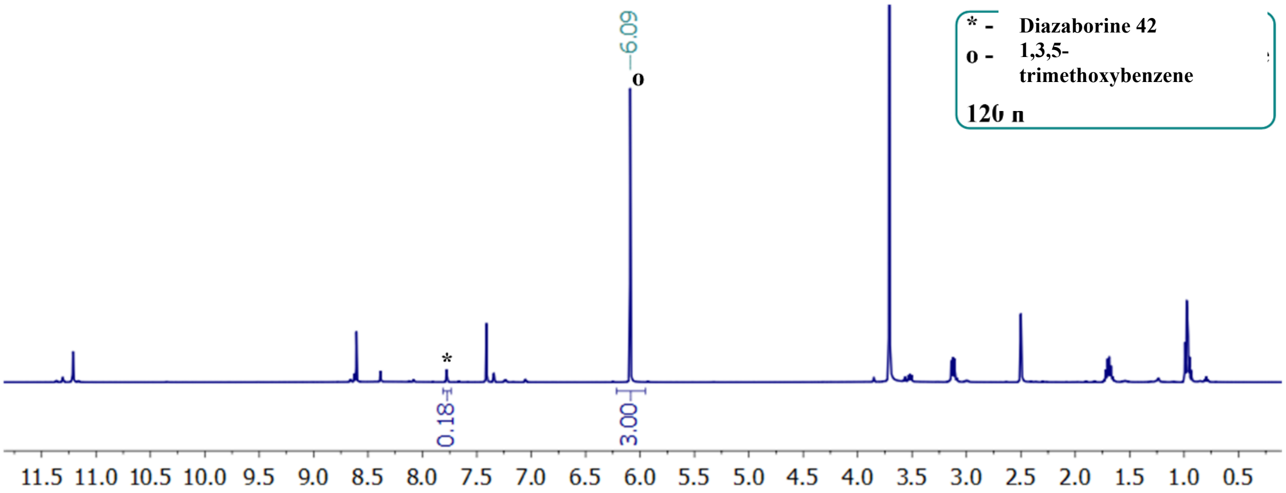

Figure S576. ^{1}H NMR spectrum (500 MHz, DMSO-d_6, 310 K) of a 1:1 mixture of diazaborine **42** and 1,3,5-trimethoxybenzene as internal standard after 120 h (5 cycles of freezing-defreezing)

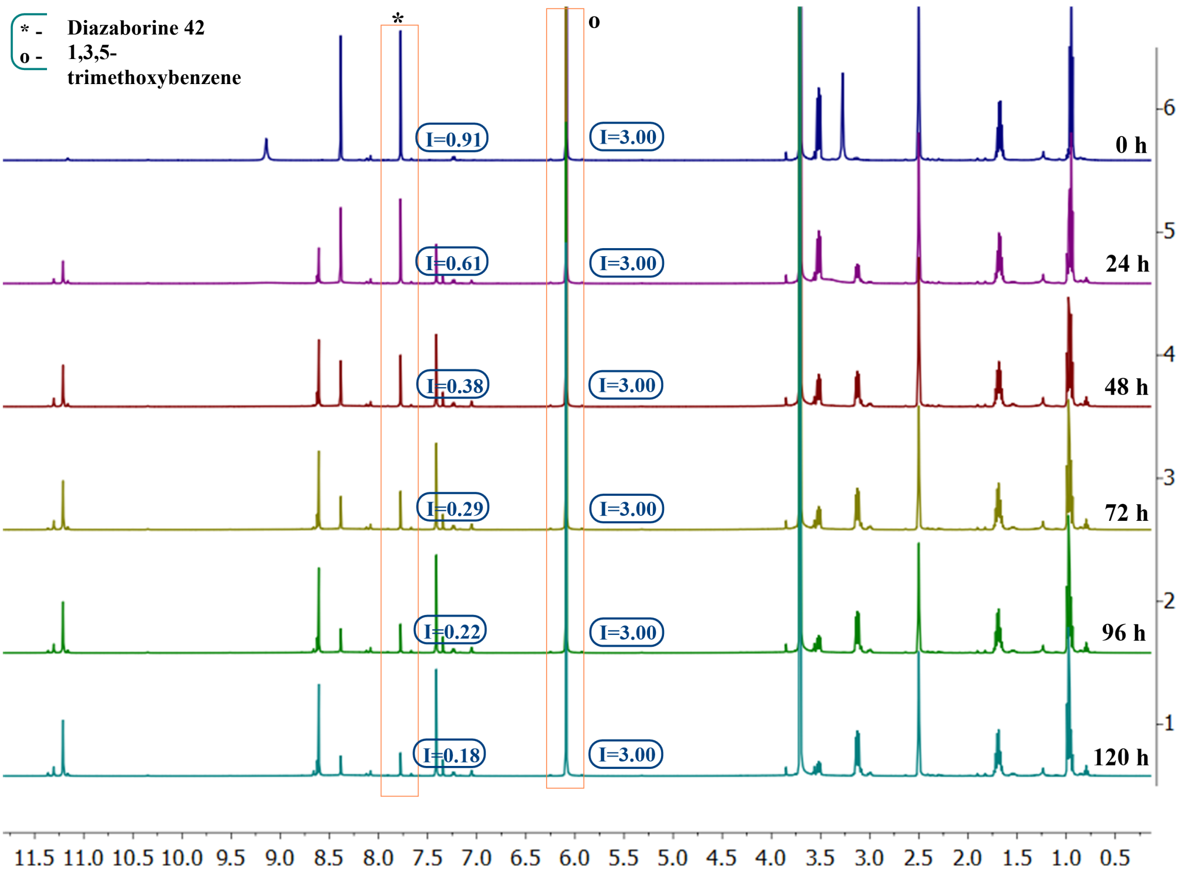

Figure S577. Stacked ^{1}H NMR spectra (500 MHz, DMSO-d_6, 310 K) of a 1:1 mixture of diazaborine **42** and 1,3,5-trimethoxybenzene as internal standard after 120 h (5 cycles of freezing-defreezing)

Diazaborine 41

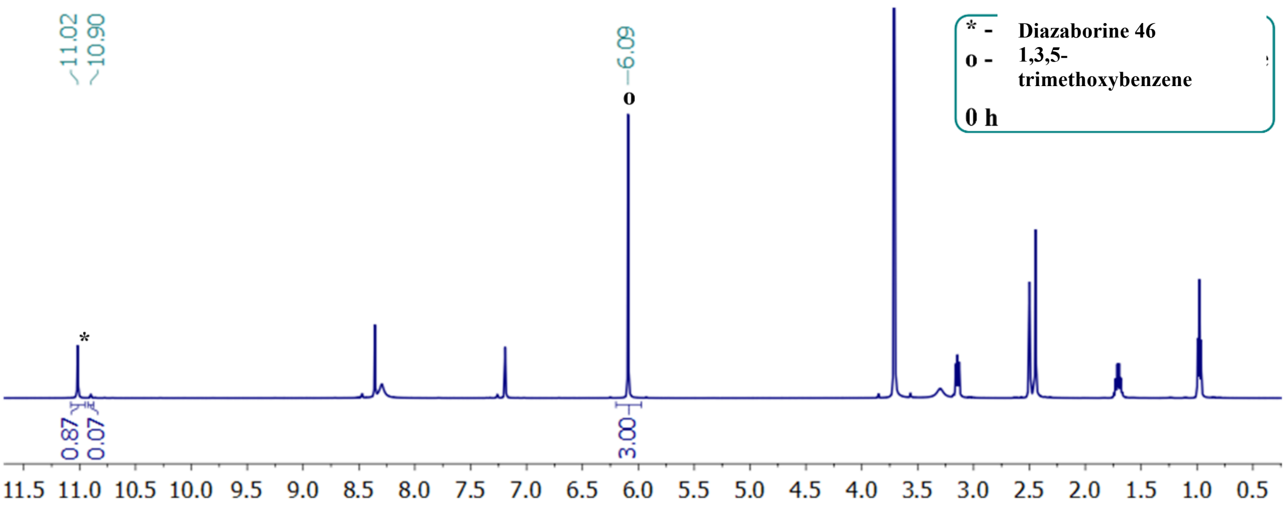

Figure S578. Initial ^{1}H NMR spectrum (600 MHz, DMSO-d_6, 310 K) of a 1:1 mixture of diazaborine **46** and 1,3,5-trimethoxybenzene as internal standard

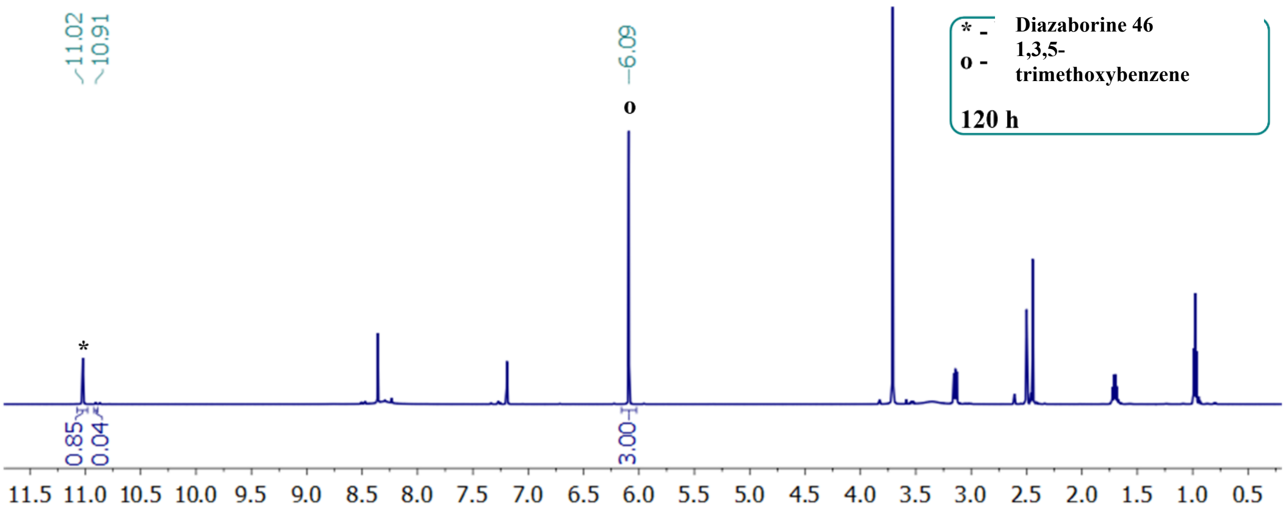

Figure S579. ^{1}H NMR spectrum (600 MHz, DMSO-d_6, 310 K) of a 1:1 mixture of diazaborine **46** and 1,3,5-trimethoxybenzene as internal standard after 120 h (5 cycles of freezing-defreezing)

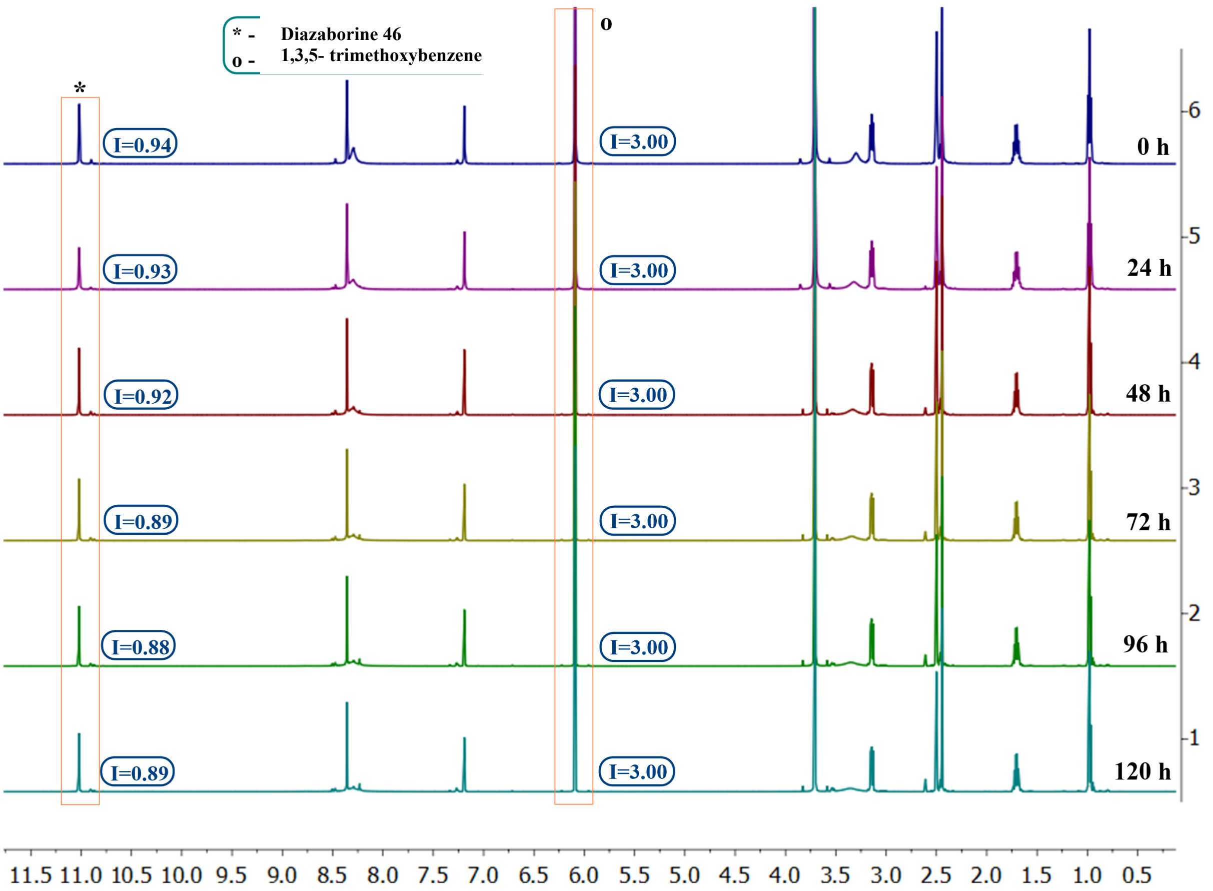

Figure S580. Stacked ^{1}H NMR spectra (600 MHz, DMSO-d_6, 310 K) of a 1:1 mixture of diazaborine **46** and 1,3,5-trimethoxybenzene as internal standard after 120 h (5 cycles of freezing-defreezing)

Diazaborine 47

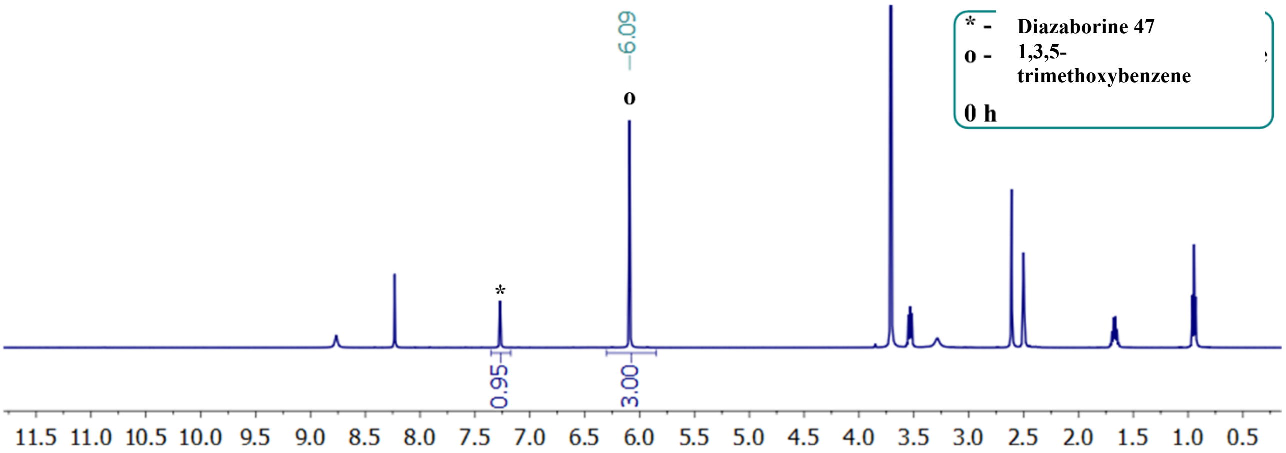

Figure S581. Initial ^{1}H NMR spectrum (500 MHz, DMSO-d_6, 310 K) of a 1:1 mixture of diazaborine **47** and 1,3,5-trimethoxybenzene as internal standard

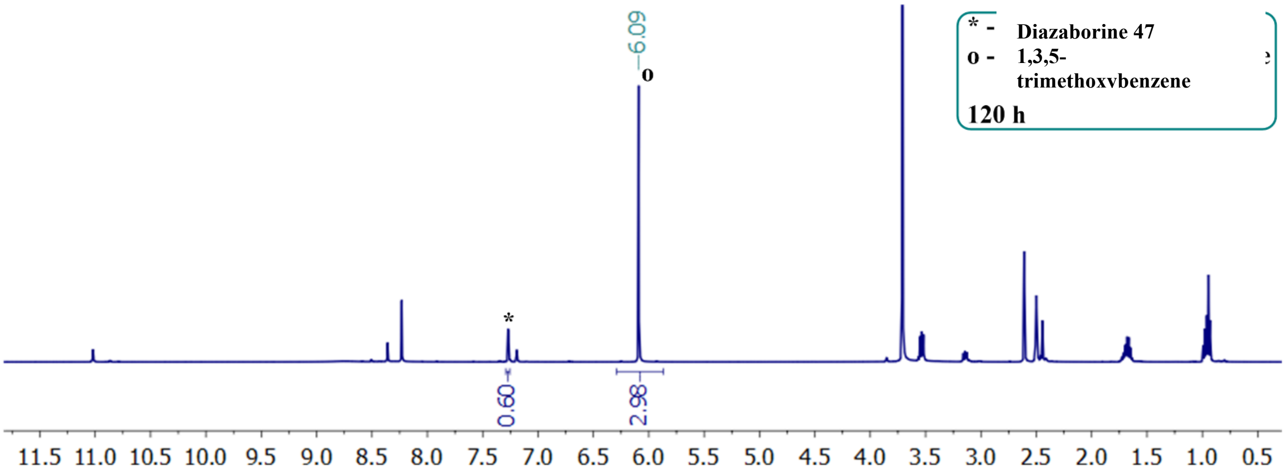

Figure S582. ^{1}H NMR spectrum (500 MHz, DMSO-d_6, 310 K) of a 1:1 mixture of diazaborine **47** and 1,3,5-trimethoxybenzene as internal standard after 120 h (5 cycles of freezing-defreezing)

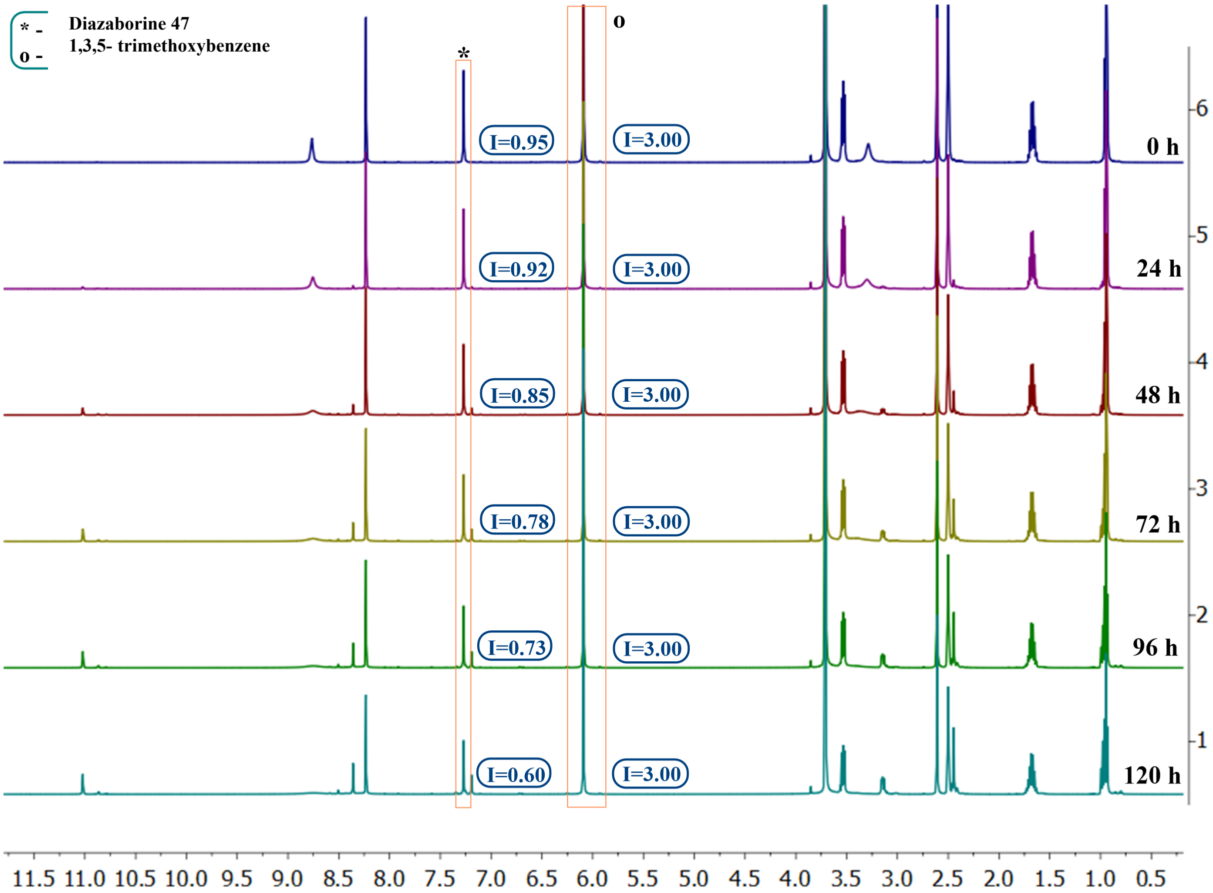

Figure S583. Stacked ^{1}H NMR spectra (500 MHz, DMSO-d_6, 310 K) of a 1:1 mixture of diazaborine **47** and 1,3,5-trimethoxybenzene as internal standard after 120 h (5 cycles of freezing-defreezing)

Calibration curves for freezing-defreezing studies

Diazaborine 11

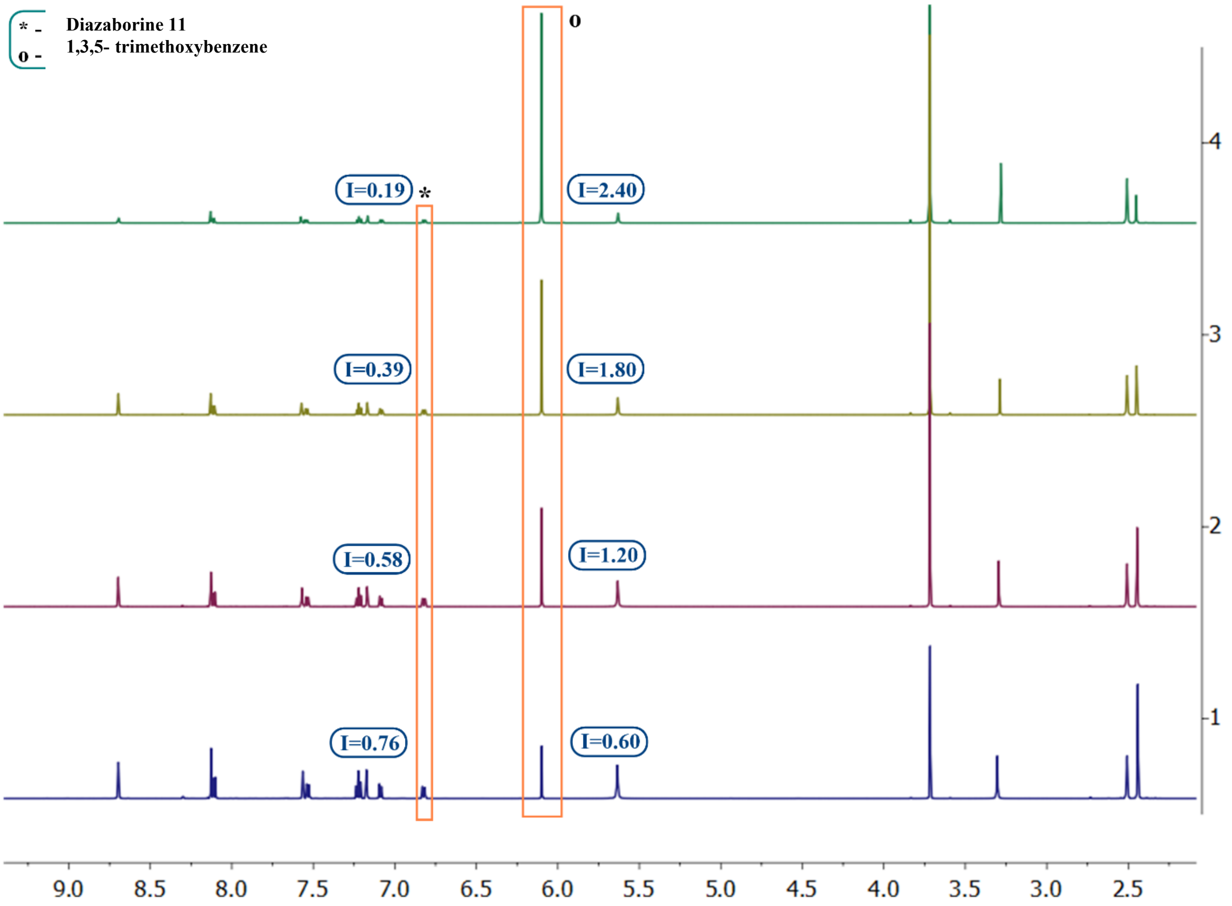

Figure S584. Stacked ^{1}H NMR spectra (600 MHz, DMSO-d_6, 310 K) of various ratios (**1**: 0.8:0.2, **2**: 0.6:0.4, **3**: 0.4:0.6, **4**: 0.2:0.8) of diazaborine **11** and 1,3,5-trimethoxybenzene.

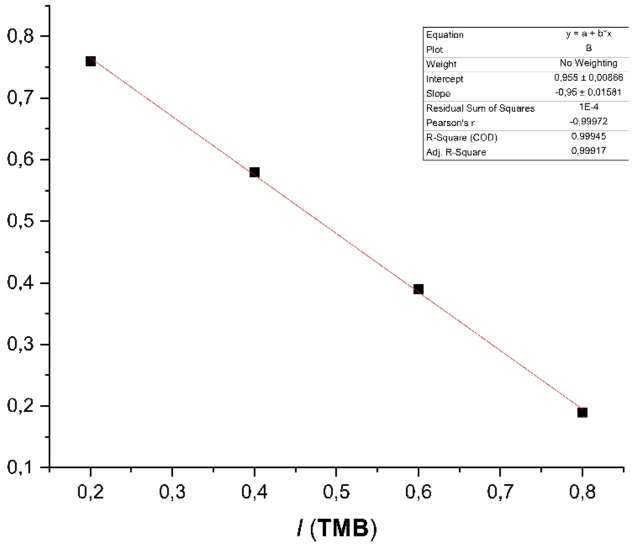

Figure S585. Calibration curve for ^{1}H NMR spectra (600 MHz, DMSO-d_6, 310 K) of different ratios (0.8:0.2, 0.6:0.4, 0.4:0.6, 0.2:0.8) of diazaborine **11** and 1,3,5-trimethoxybenzene.

Diazaborine 13

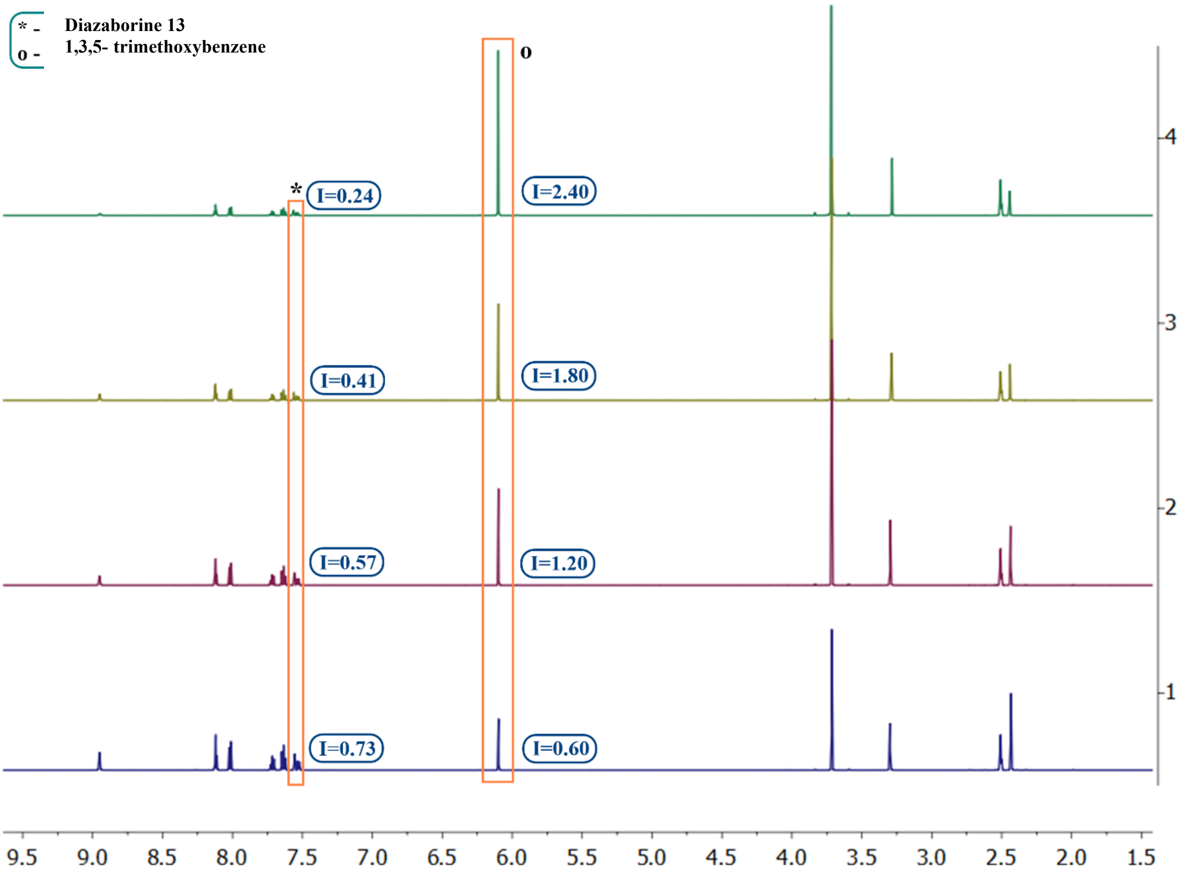

Figure S586. Stacked ^{1}H NMR spectra (600 MHz, DMSO-d_6, 310 K) of various ratios (**1**: 0.8:0.2, **2**: 0.6:0.4, **3**: 0.4:0.6, **4**: 0.2:0.8) of diazaborine **13** and 1,3,5-trimethoxybenzene.

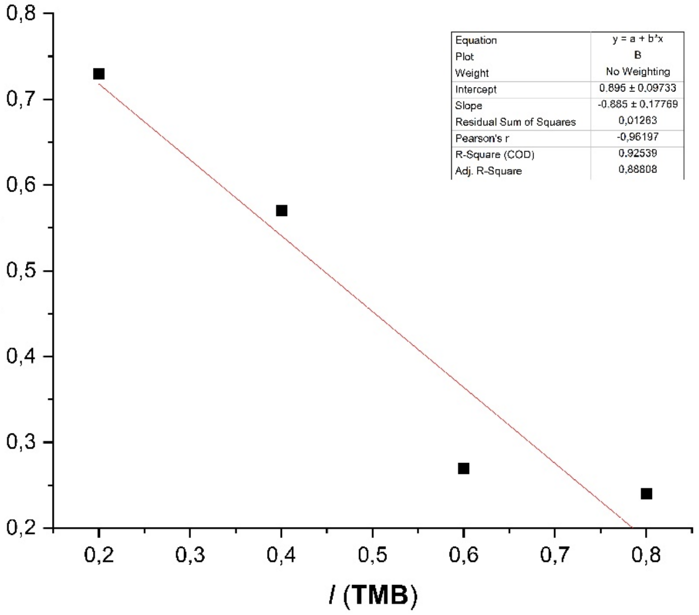

Figure S587. Calibration curve for the ^{1}H NMR spectra (600 MHz, DMSO-d_6, 310 K) of different ratios (0.8:0.2, 0.6:0.4, 0.4:0.6, 0.2:0.8) of diazaborine **13** and 1,3,5-trimethoxybenzene.

Diazaborine 61

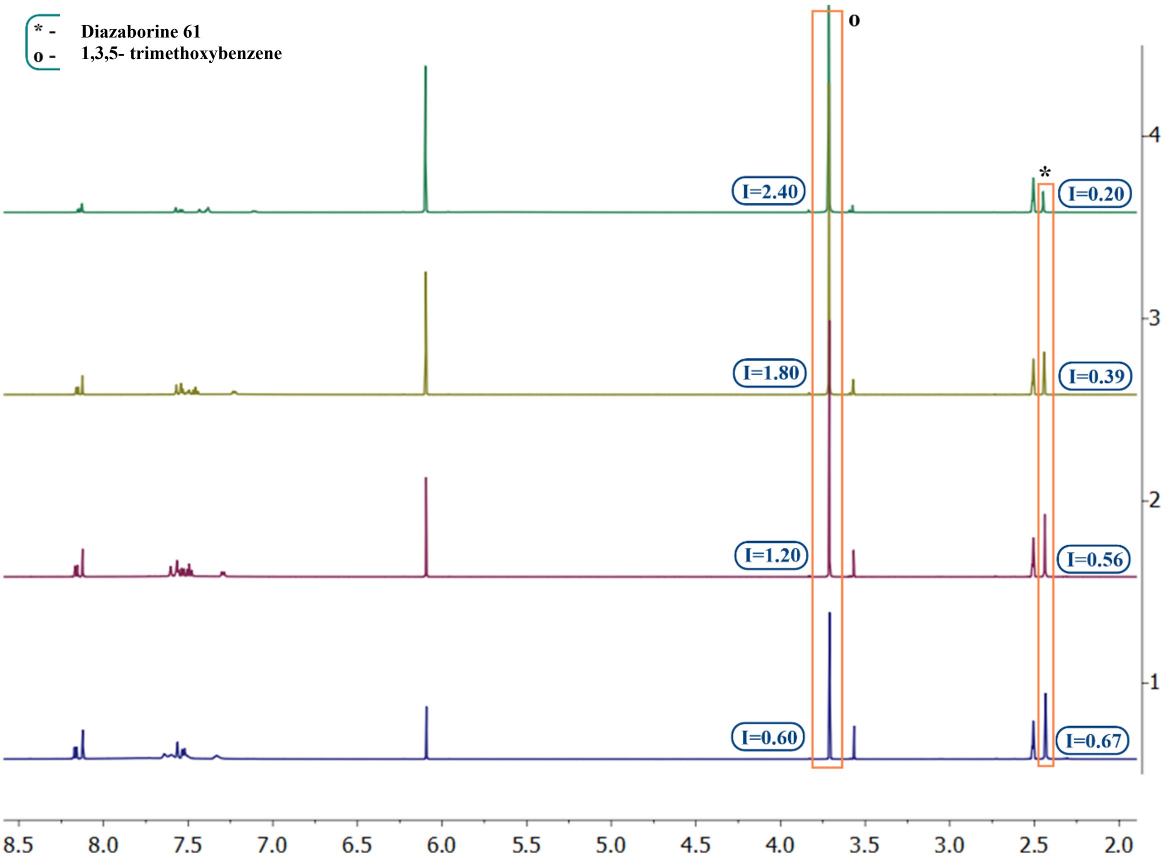

Figure S588. Stacked ^{1}H NMR spectra (600 MHz, DMSO-d_6, 310 K) of various ratios (**1**: 0.8:0.2, **2**: 0.6:0.4, **3**: 0.4:0.6, **4**: 0.2:0.8) of diazaborine **61** and 1,3,5-trimethoxybenzene.

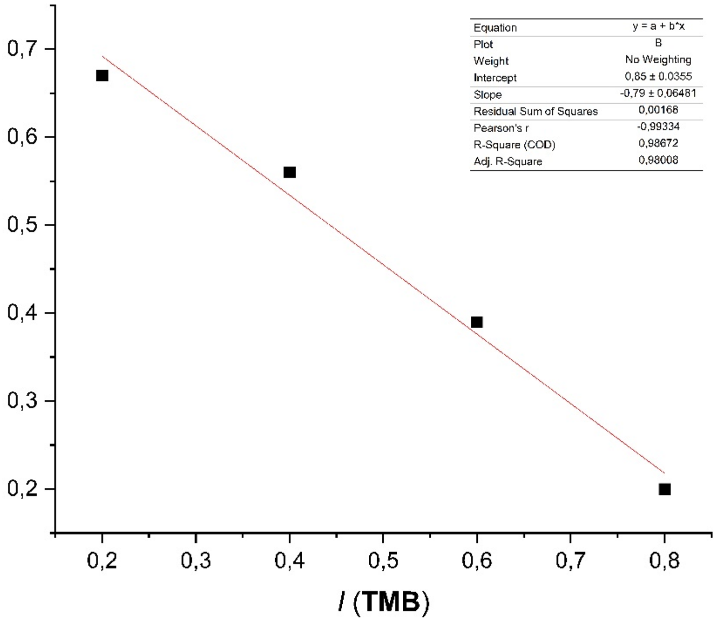

Figure S589. Calibration curve for the ^{1}H NMR spectra (600 MHz, DMSO-d_6, 310 K) of different ratios (0.8:0.2, 0.6:0.4, 0.4:0.6, 0.2:0.8) of diazaborine **61** and 1,3,5-trimethoxybenzene.

Diazaborine 41

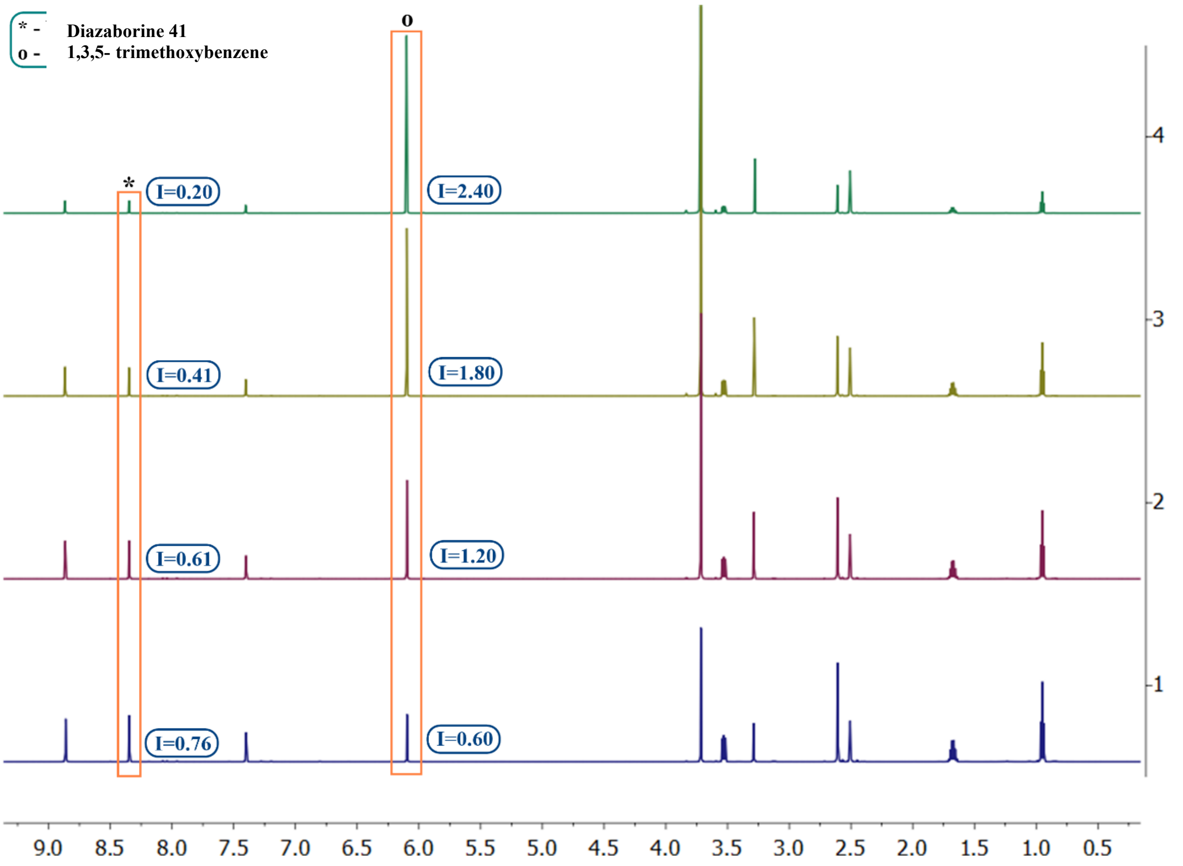

Figure S590. Stacked ^{1}H NMR spectra (600 MHz, DMSO-d_6, 310 K) of various ratios (**1**: 0.8:0.2, **2**: 0.6:0.4, **3**: 0.4:0.6, **4**: 0.2:0.8) of diazaborine **41** and 1,3,5-trimethoxybenzene.

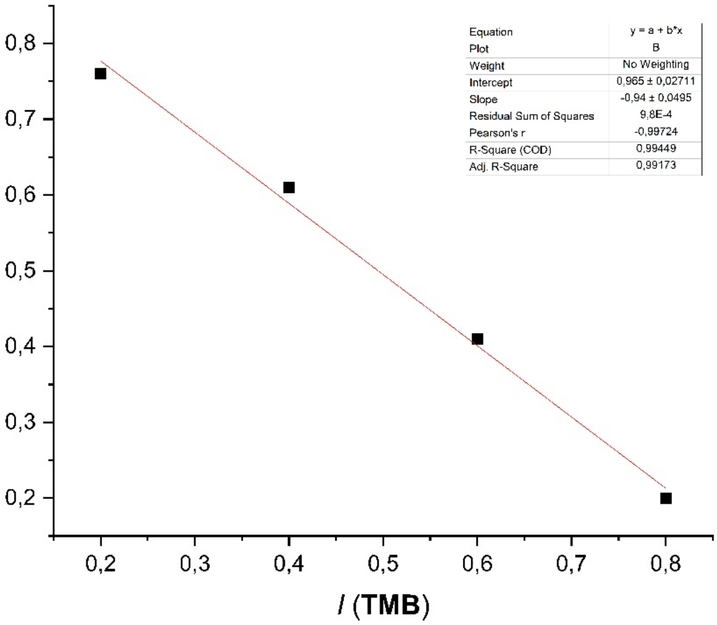

Figure S591. Calibration curve for the ^{1}H NMR spectra (600 MHz, DMSO-d_6, 310 K) of different ratios (0.8:0.2, 0.6:0.4, 0.4:0.6, 0.2:0.8) of diazaborine **41** and 1,3,5-trimethoxybenzene.

Diazaborine 42

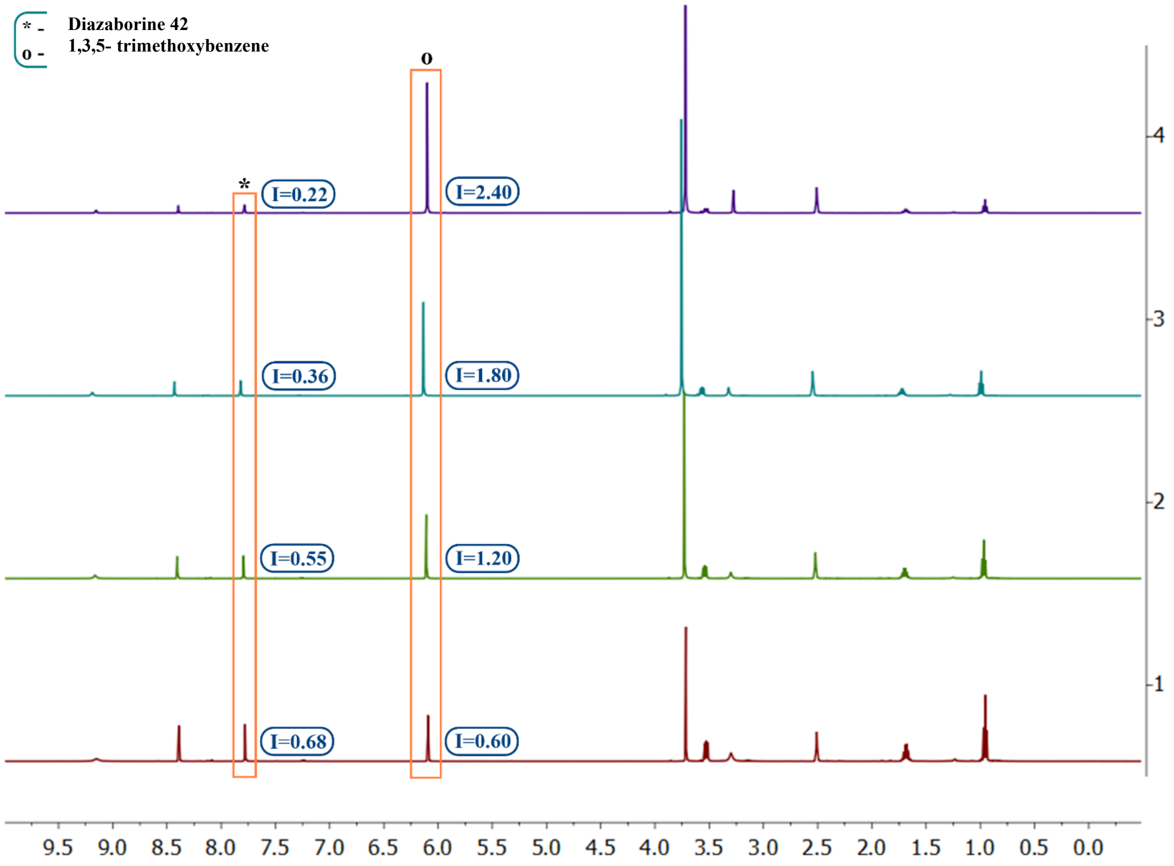

Figure S592. Stacked ^{1}H NMR spectra (500 MHz, DMSO-d_6, 310 K) of various ratios (**1**: 0.8:0.2, **2**: 0.6:0.4, **3**: 0.4:0.6, **4**: 0.2:0.8) of diazaborine **42** and 1,3,5-trimethoxybenzene.

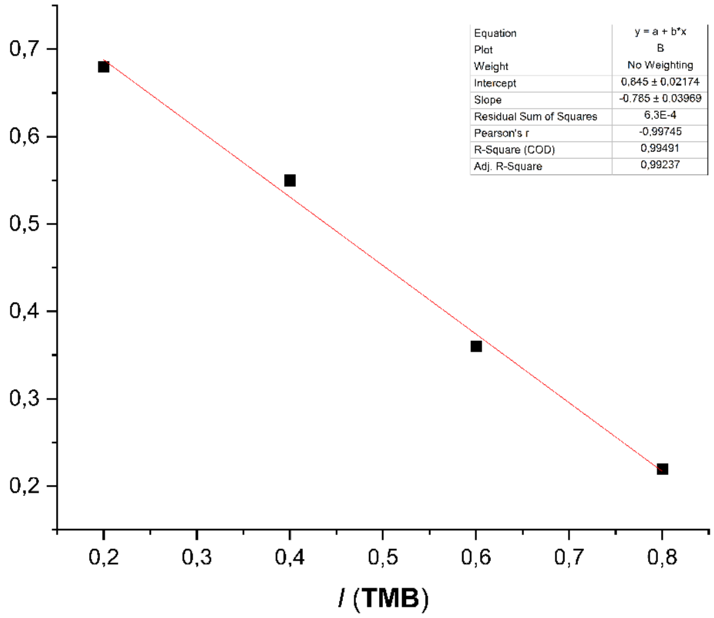

Figure S593. Calibration curve for the ^{1}H NMR spectra (500 MHz, DMSO-d_6, 310 K) of different ratios (0.8:0.2, 0.6:0.4, 0.4:0.6, 0.2:0.8) of diazaborine **42** and 1,3,5-trimethoxybenzene.

Diazaborine 46

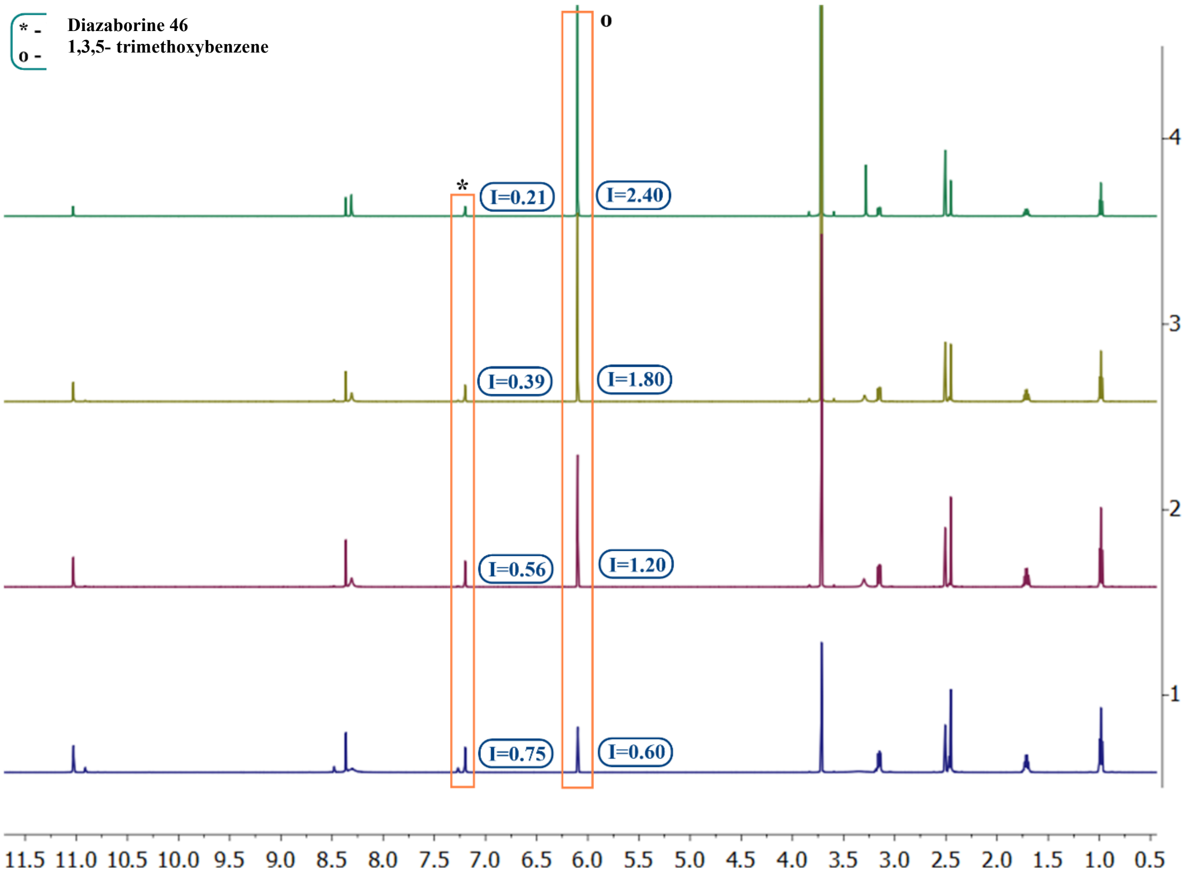

Figure S594. Stacking of the ^{1}H NMR spectra (600 MHz, DMSO-d_6, 310 K) of various ratios (**1**: 0.8:0.2, **2**: 0.6:0.4, **3**: 0.4:0.6, **4**: 0.2:0.8) of diazaborine **46** and 1,3,5-trimethoxybenzene.

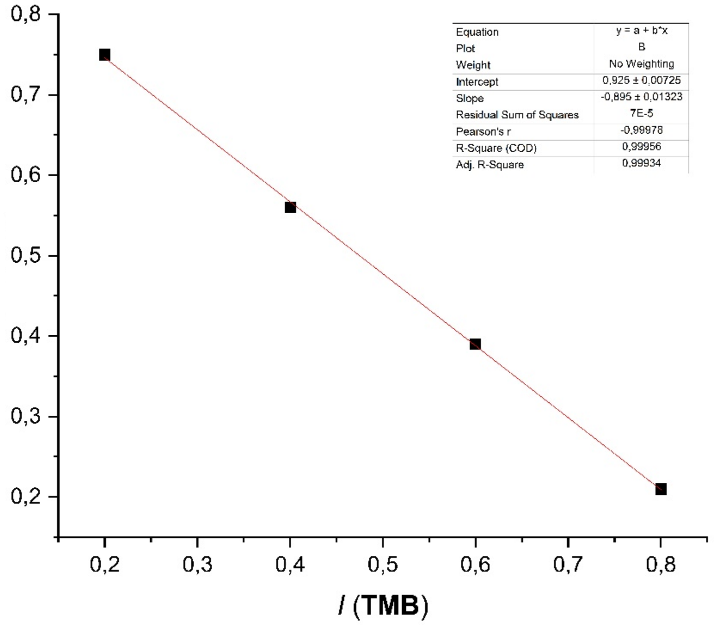

Figure S595. Calibration curve for the ^{1}H NMR spectra (600 MHz, DMSO-d_6, 310 K) of different ratios (0.8:0.2, 0.6:0.4, 0.4:0.6, 0.2:0.8) of diazaborine **46** and 1,3,5-trimethoxybenzene.

Diazaborine 47

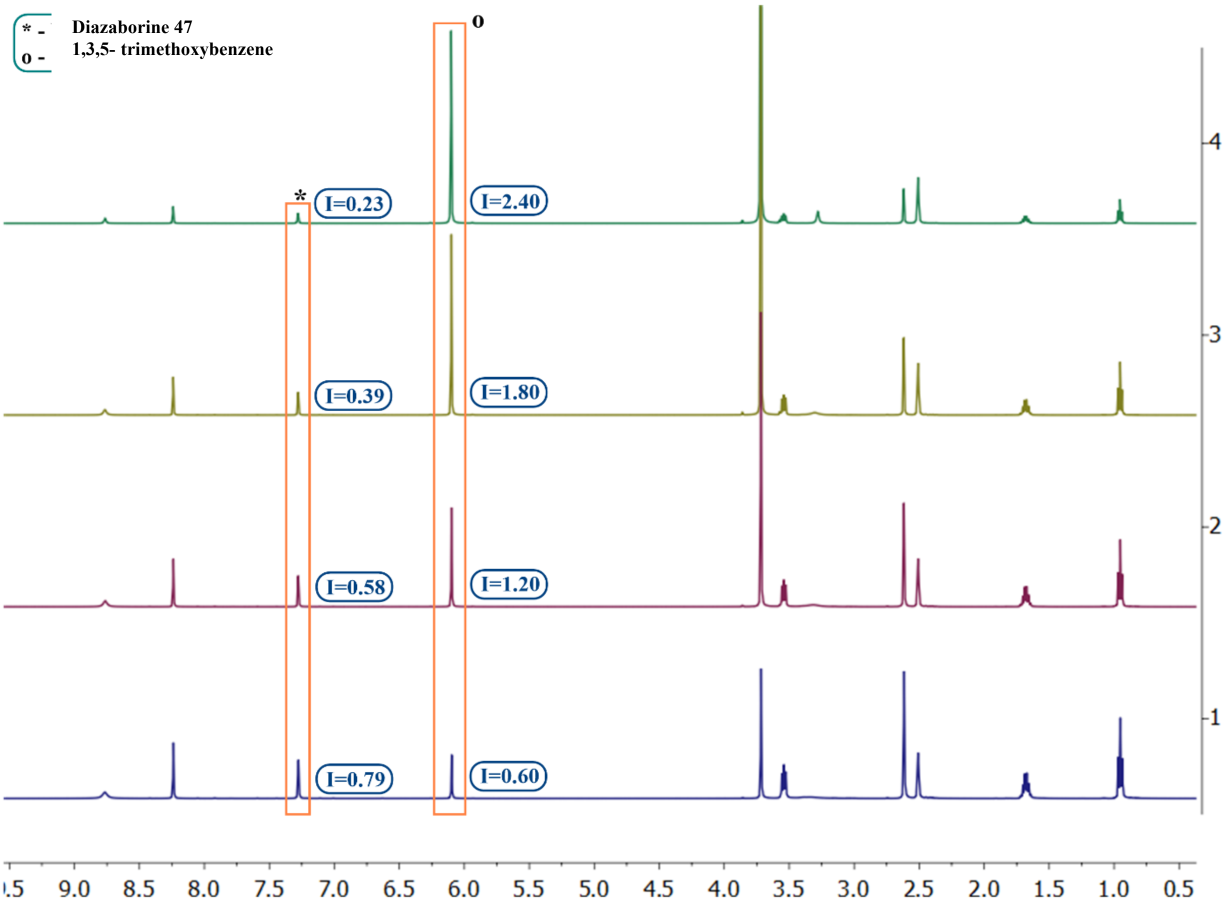

Figure S596. Stacked NMR spectra ^{1}H (500 MHz, DMSO-d_6, 310 K) of various ratios (**1**: 0.8:0.2, **2**: 0.6:0.4, **3**: 0.4:0.6, **4**: 0.2:0.8) of diazaborine **47** and 1,3,5-trimethoxybenzene.

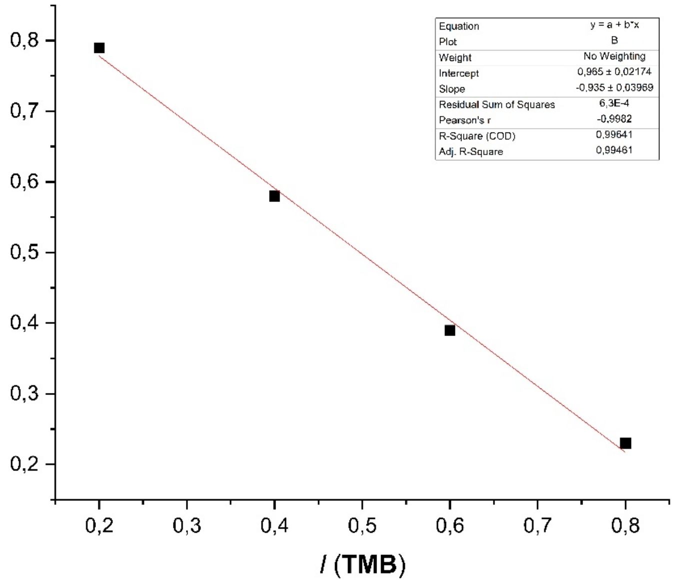

Figure S597. Calibration curve for the ^{1}H NMR spectra (500 MHz, DMSO-d_6, 310 K) of different ratios (0.8:0.2, 0.6:0.4, 0.4:0.6, 0.2:0.8) of diazaborine **47** and 1,3,5-trimethoxybenzene.

Stability studies in human plasma at 37 °C

Diazaborines are poorly soluble in aqueous media. Hence, we evaluated the stability of the most representative diazaborines of each series indirectly in plasma through its corresponding sodium salts which had shown similar antimicrobial profiles in previous assays, using acetamide as internal standards.

The plasma samples were allowed to stand at 310K and aliquots were taken at the indicated times. Then the aliquots were allowed to cool down at room temperature and their NMR spectrum was acquired after 5 min.

^{11}B NMR spectra of the plasma sample of each compound, at the beginning of the experiment (zero point) and at the end, are provided to confirm that the likely observed changes are not attributable to a mere switch on geometry at the boron nuclei from tetragonal to trigonal.

The precise experimenta l details are disclosed on the experimental section of this manuscript.

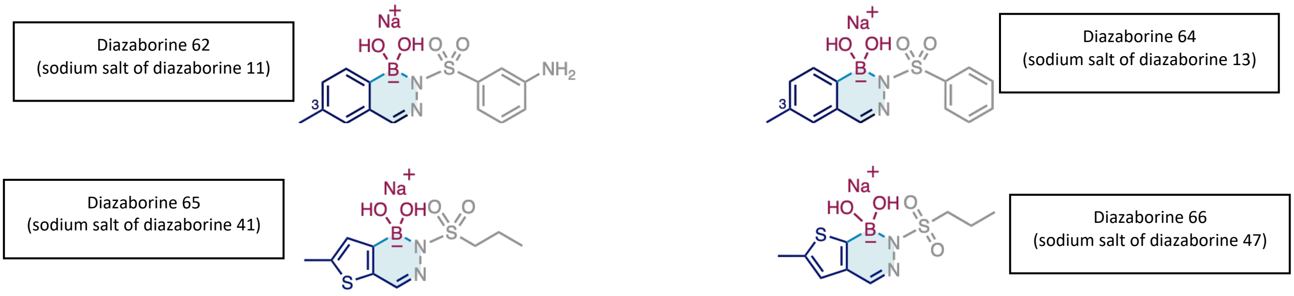

Figure S598. Selected sodium salts whose stability was evaluated in near-physiological aqueous conditions (plasma).

Diazaborine 62 (Sodium salt of diazaborine 11)

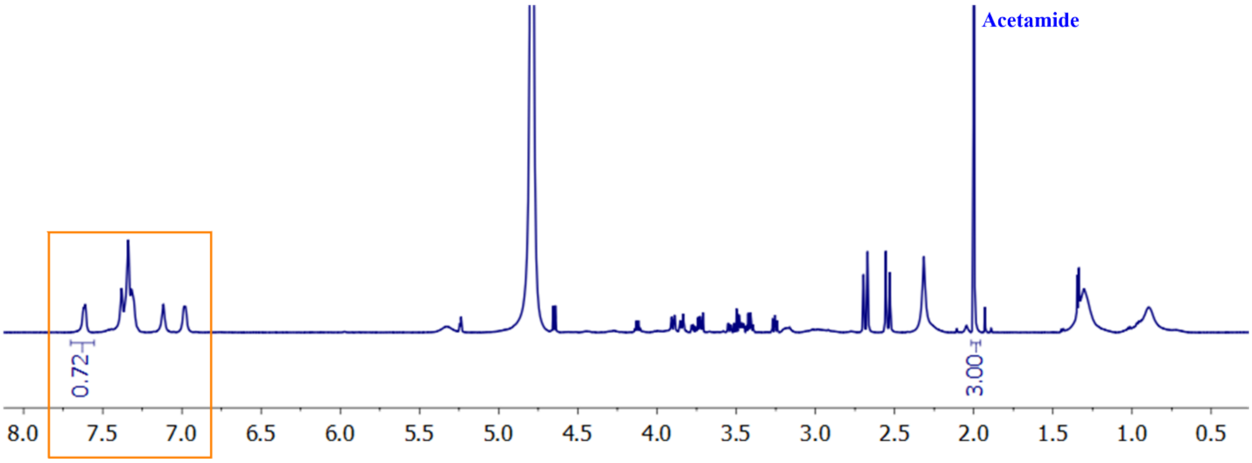

Figure S599. Initial ^{1}H NMR spectrum (600 MHz, D_2O, 298 K) of diazaborine **62** (orange frame), acetamide as internal standard and plasma (Aliquot maintained 0 h in plasma at 37 °C).

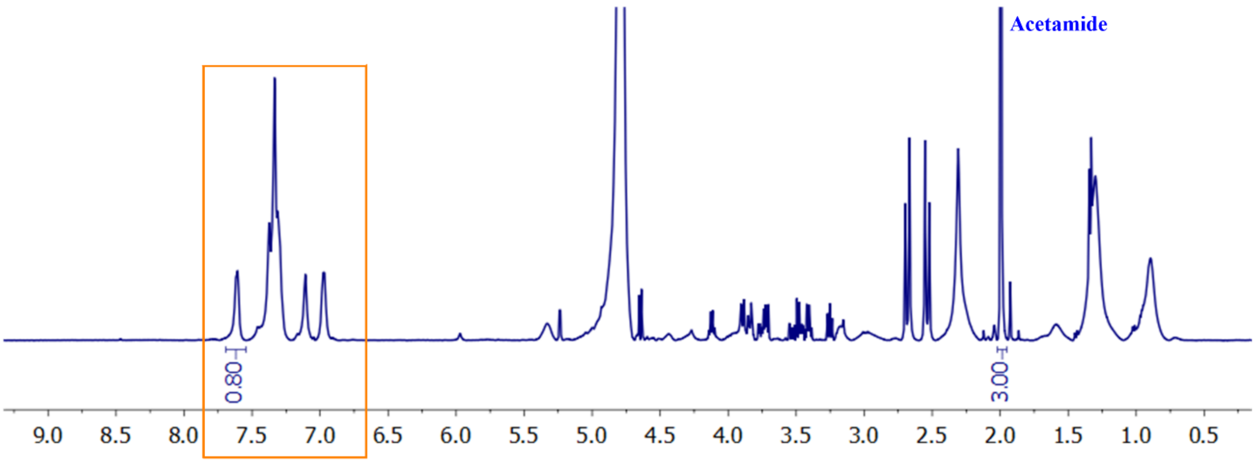

Figure S600. ^{1}H NMR spectrum (600 MHz, D_2O, 298 K) of diazaborine **62** (orange frame), acetamide as internal standard and plasma (Aliquot maintained 24 h in plasma at 37 °C).

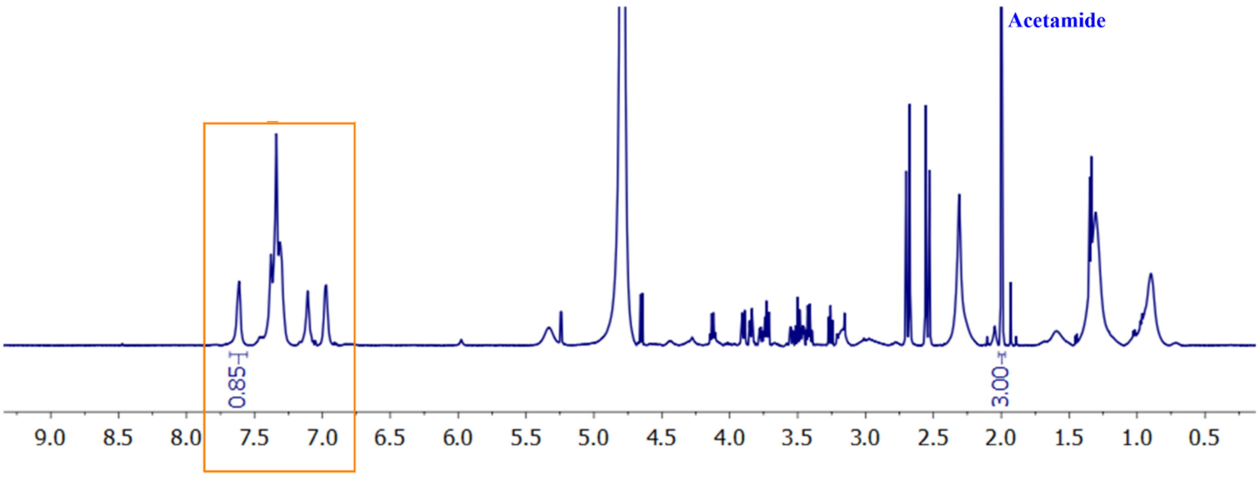

Figure S601. ^{1}H NMR spectrum (600 MHz, D_2O, 298 K) of diazaborine **62** (orange frame), acetamide as internal standard and plasma (Aliquot maintained 72 h in plasma at 37 °C).

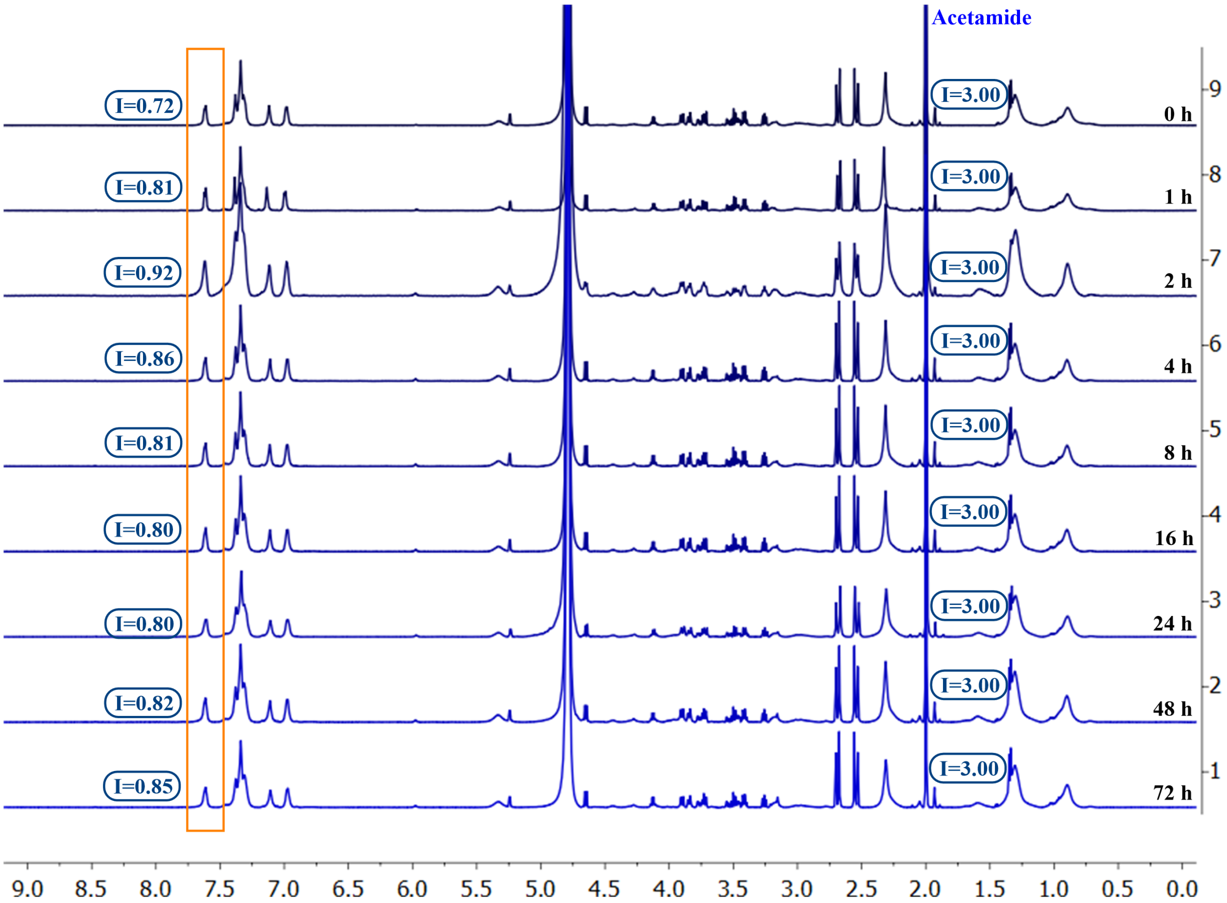

Figure S602. Stacked ^{1}H NMR spectra (600 MHz, D_2O, 298 K) of diazaborine **62** (orange frame), acetamide as internal standard and plasma (Aliquots maintained in plasma at 37 °C taken at different times).

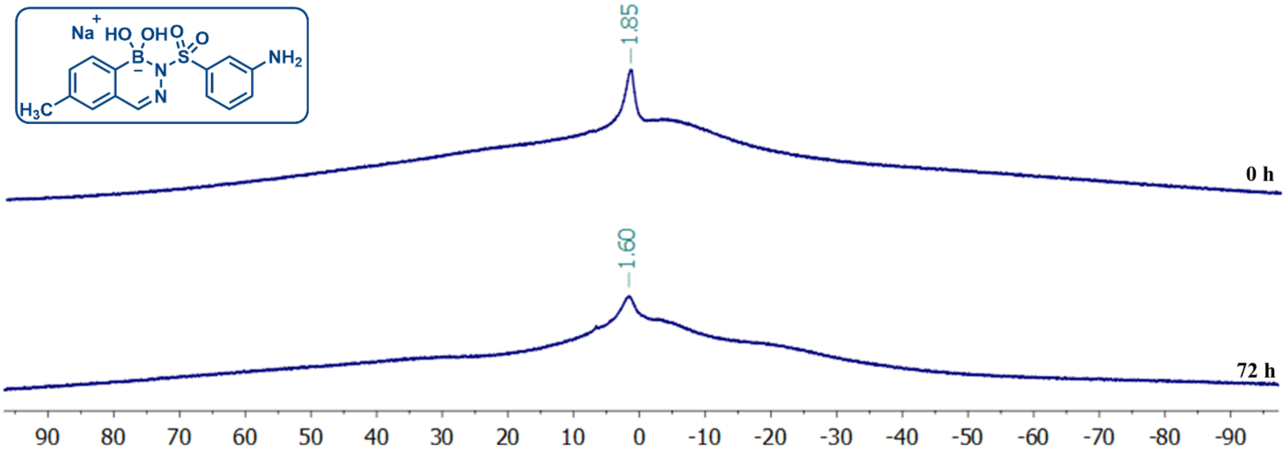

Figure S603. Up: Initial ^{11}B NMR spectrum (160 MHz, D_2O, 298 K) of a 1:1 mixture of diazaborine **62** and acetamide; Down: ^{11}B NMR spectrum (128 MHz, D_2O, 298 K) of a mixture of diazaborine **62**, acetamide as internal standard and plasma (Aliquot maintained 72 h in plasma at 37 °C).

Diazaborine 64 (Sodium salt of diazaborine 13)

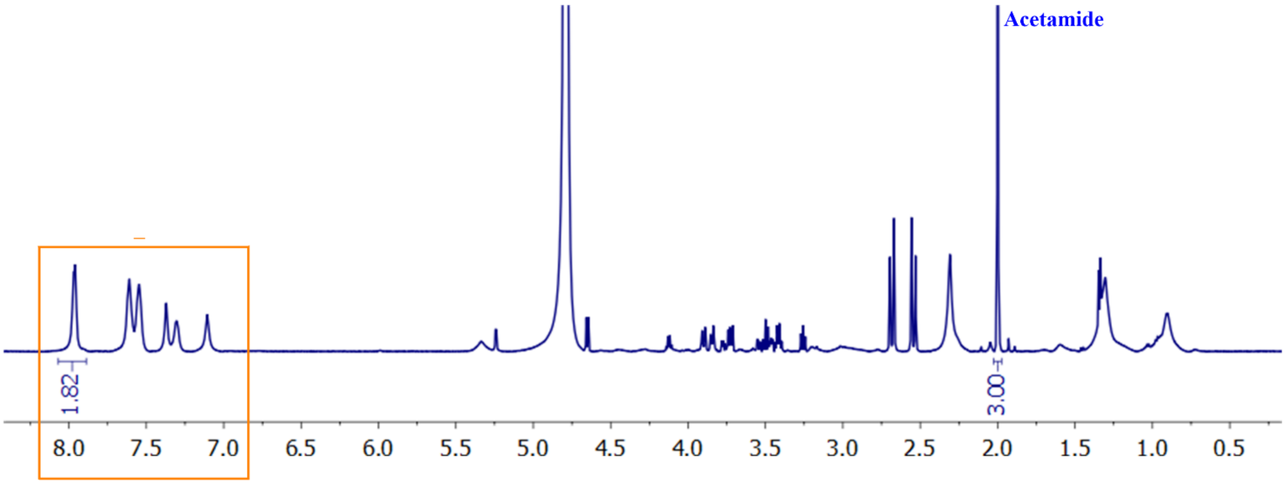

Figure S604. Initial ^{1}H NMR spectrum (600 MHz, D_2O, 298 K) of diazaborine **64** (orange frame), acetamide as internal standard and plasma (Aliquot maintained 0 h in plasma at 37 °C).

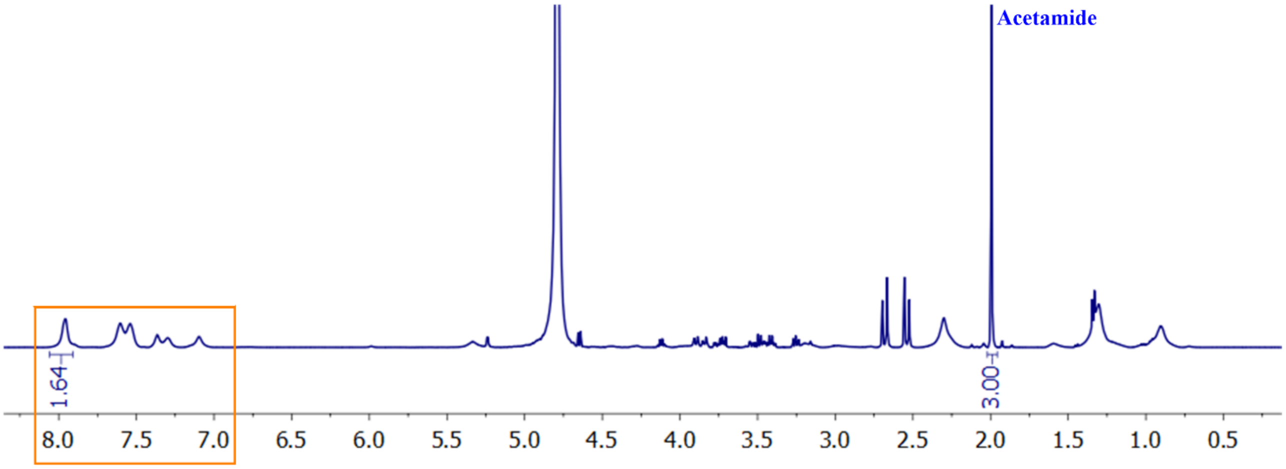

Figure S605. ^{1}H NMR spectrum (600 MHz, D_2O, 298 K) of diazaborine **64** (orange frame), acetamide as internal standard and plasma (Aliquot maintained 24 h in plasma at 37 °C).

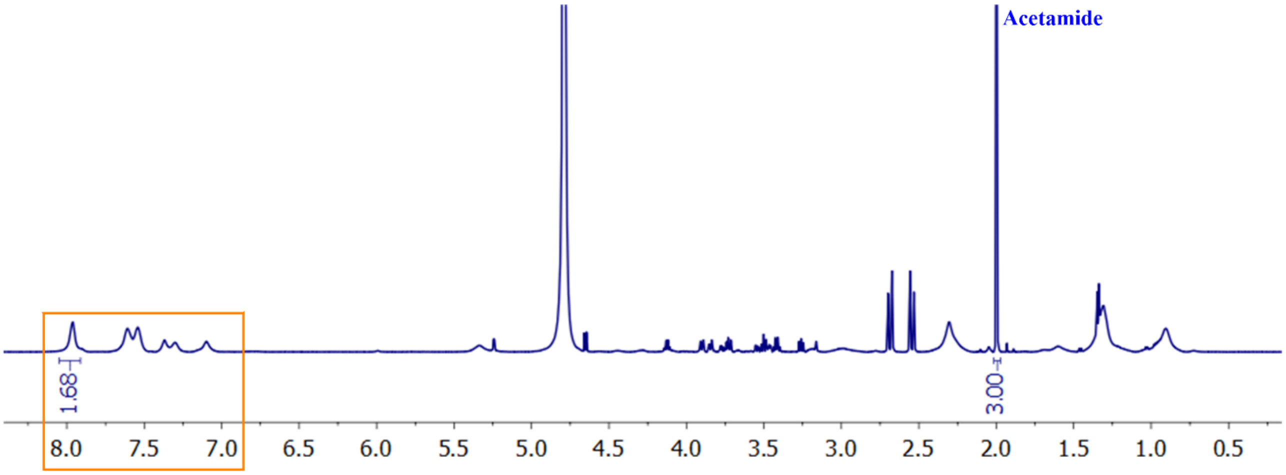

Figure S606. ^{1}H NMR spectrum (600 MHz, D_2O, 298 K) of diazaborine **64** (orange frame), acetamide as internal standard and plasma (Aliquot maintained 72 h in plasma at 37 °C).

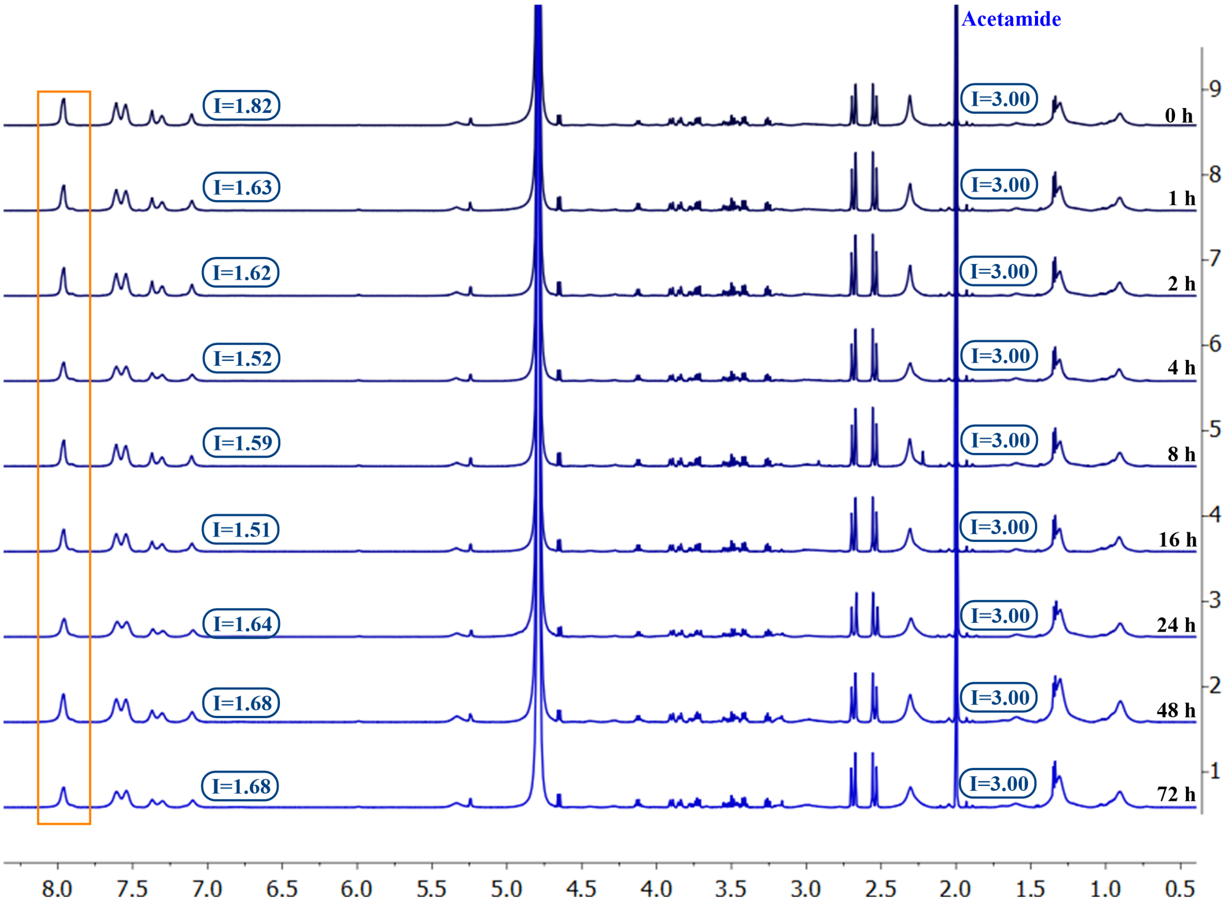

Figure S607. Stacked ^{1}H NMR spectra (600 MHz, D_2O, 298 K) of diazaborine **64** (orange frame), acetamide as internal standard and plasma (Aliquots maintained in plasma at 37 °C taken at different times).

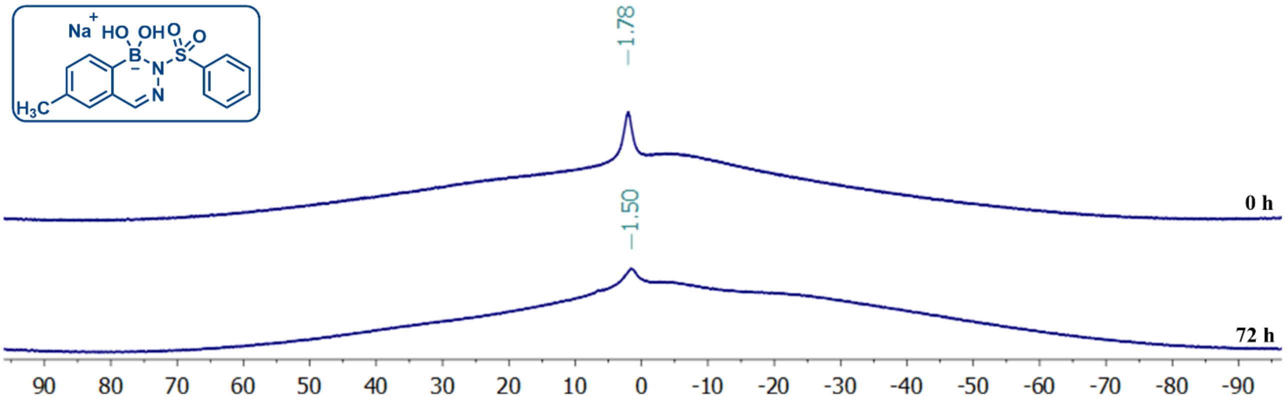

Figure S608. Up: Initial ^{11}B NMR spectrum (160 MHz, D_2O, 298 K) of a 1:1 mixture of diazaborine **64** and acetamide as internal standard; Down: ^{11}B NMR spectrum (128 MHz, D_2O, 298 K) of a mixture of diazaborine **64**, acetamide as internal standard and plasma (Aliquot maintained 72 h in plasma at 37 °C).

Diazaborine 65 (Sodium salt of diazaborine 41)

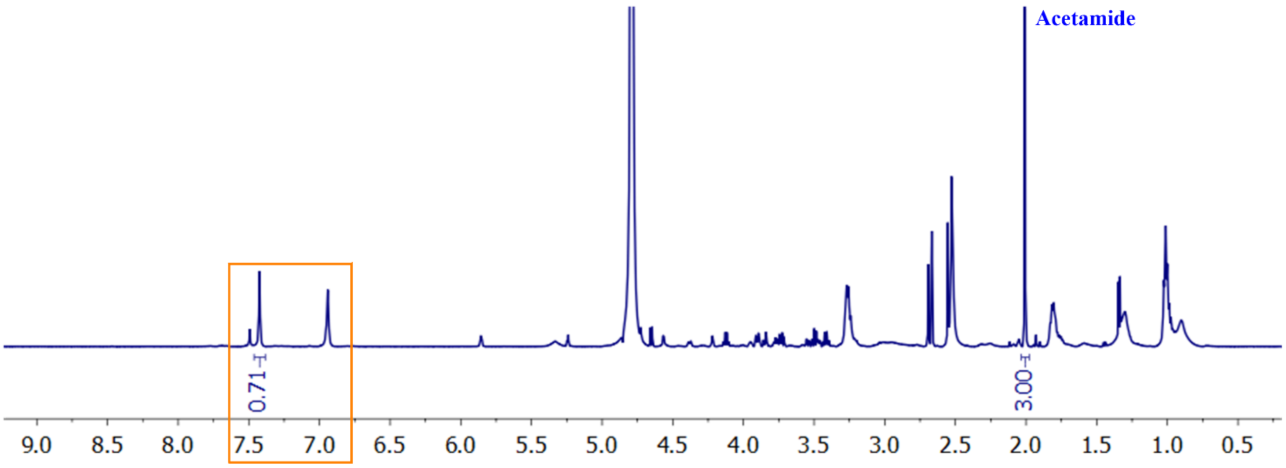

Figure S609. Initial ^{1}H NMR spectrum (600 MHz, D_2O, 298 K) of diazaborine **65** (orange frame), acetamide as internal standard and plasma (Aliquot maintained 0 h in plasma at 37 °C).

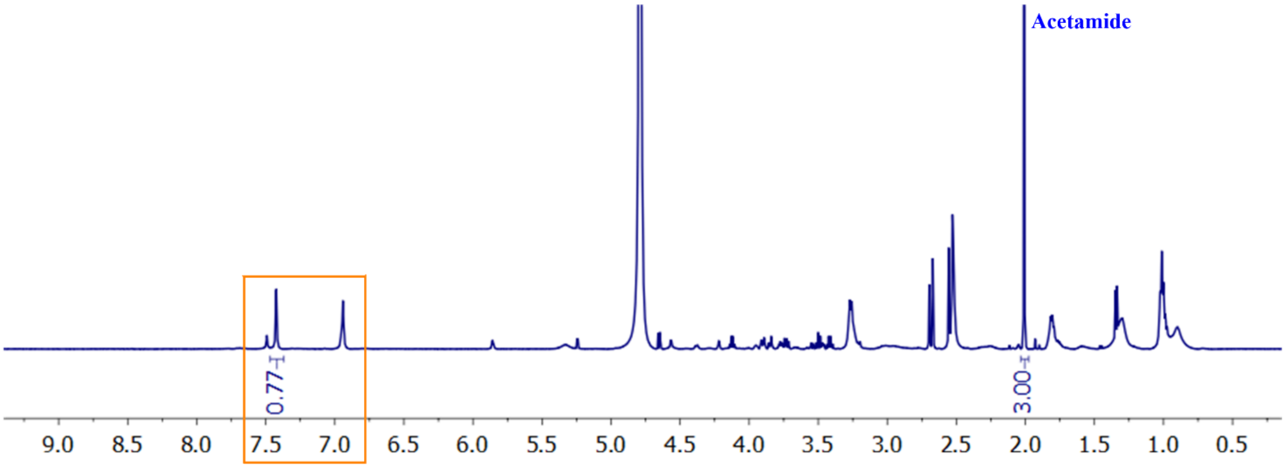

Figure S610. ^{1}H NMR spectrum (600 MHz, D_2O, 298 K) of diazaborine **65** (orange frame), acetamide as internal standard and plasma (Aliquot maintained 24 h in plasma at 37 °C).

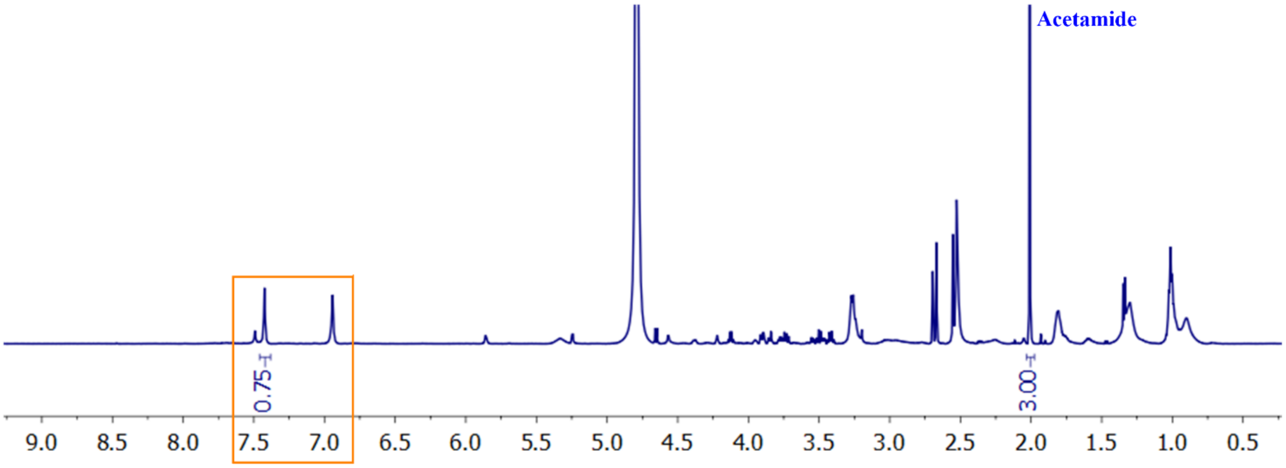

Figure S611. ^{1}H NMR spectrum (600 MHz, D_2O, 298 K) of diazaborine **65** (orange frame), acetamide as internal standard and plasma (Aliquot maintained 72 h in plasma at 37 °C).

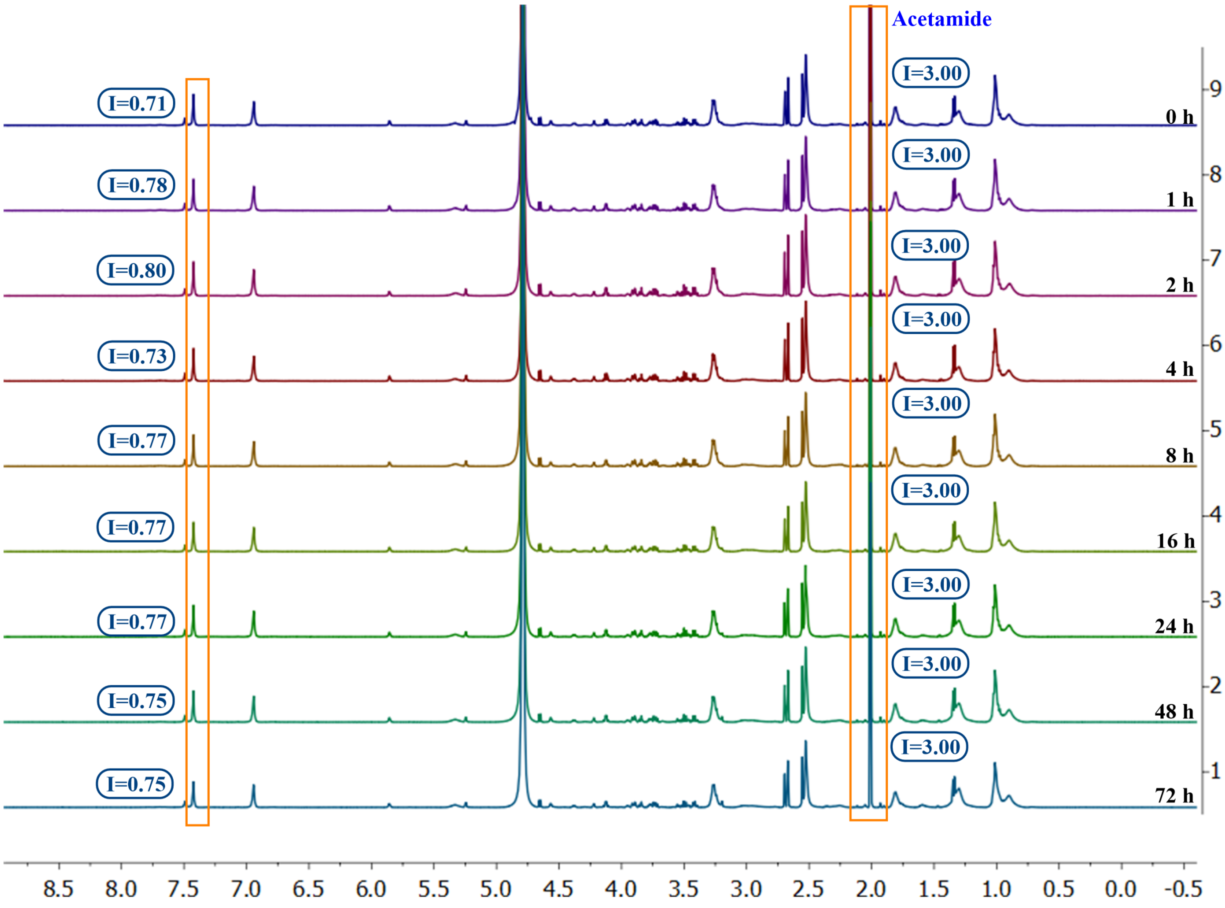

Figure S612. Stacked ^{1}H NMR spectra (600 MHz, D_2O, 298 K) of diazaborine **65** (orange frame), acetamide as internal standard and plasma (Aliquots maintained in plasma at 37 °C taken at different times).

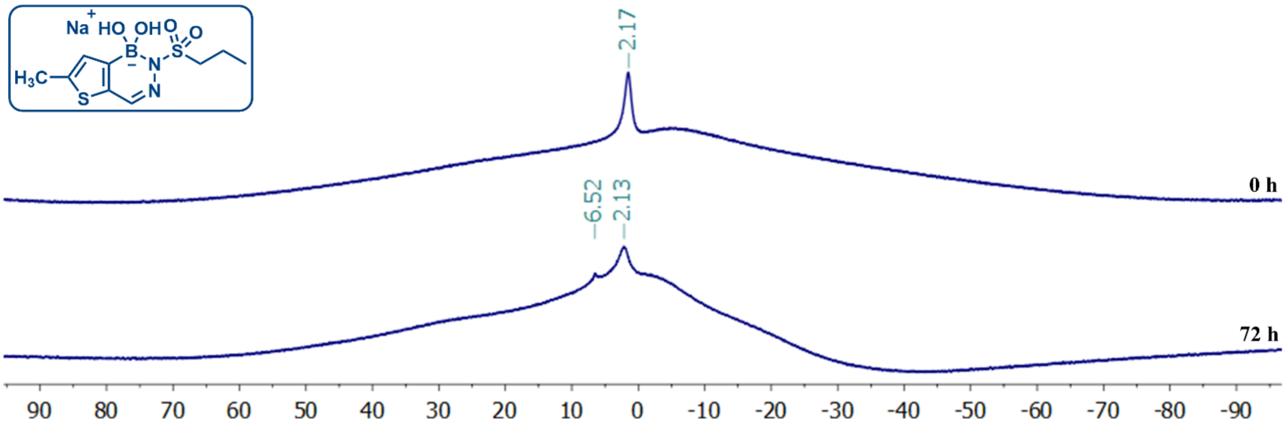

Figure S613. *Up*: Initial ^{11}B NMR spectrum (160 MHz, D_2O, 298 K) of a 1:1 mixture of diazaborine **65** and acetamide as internal standard; *Down*: ^{11}B NMR spectrum (128 MHz, D_2O, 298 K) of diazaborine **65**, acetamide as internal standard and plasma (Aliquot maintained 72 h in plasma at 37 °C).

Diazaborine 66 (Sodium salt of diazaborine 47)

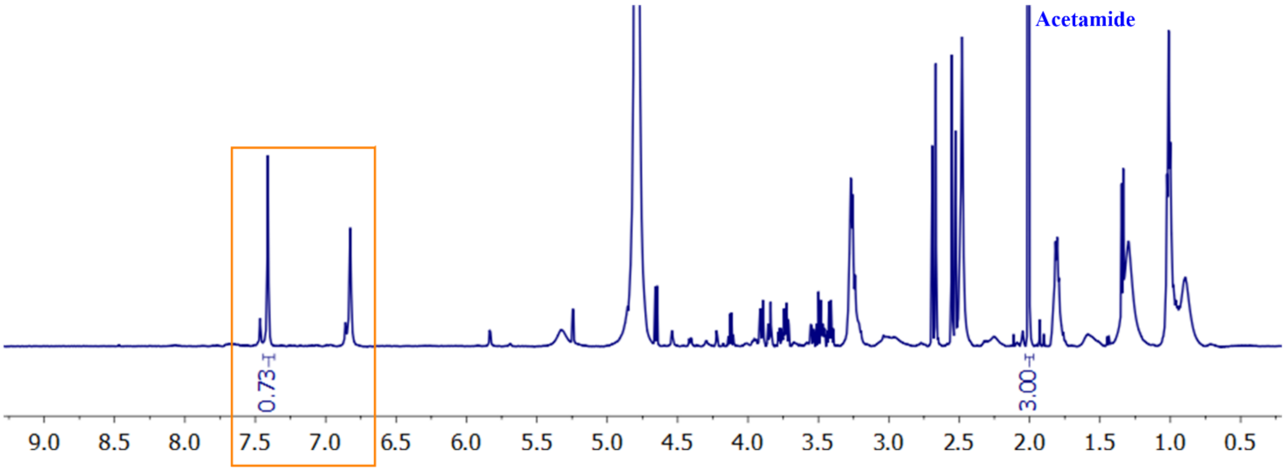

Figure S614. Initial ^{1}H NMR spectrum (600 MHz, D_2O, 298 K) of diazaborine **66** (orange frame), acetamide as internal standard and plasma (Aliquot maintained 0 h in plasma at 37 °C).

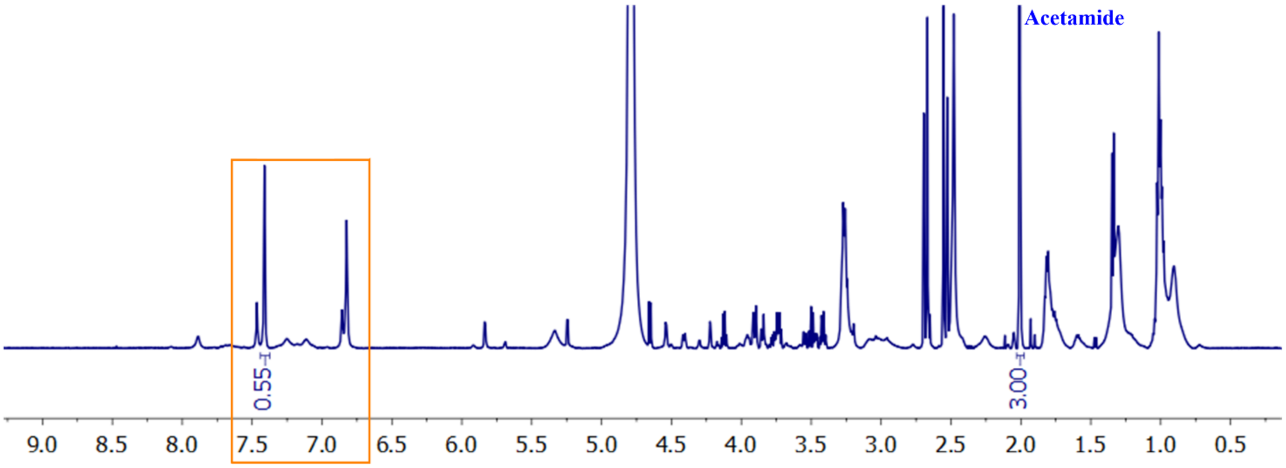

Figure S615. ^{1}H NMR spectrum (600 MHz, D_2O, 298 K) of diazaborine **66** (orange frame), acetamide as internal standard and plasma (Aliquot maintained 24 h in plasma at 37 °C).

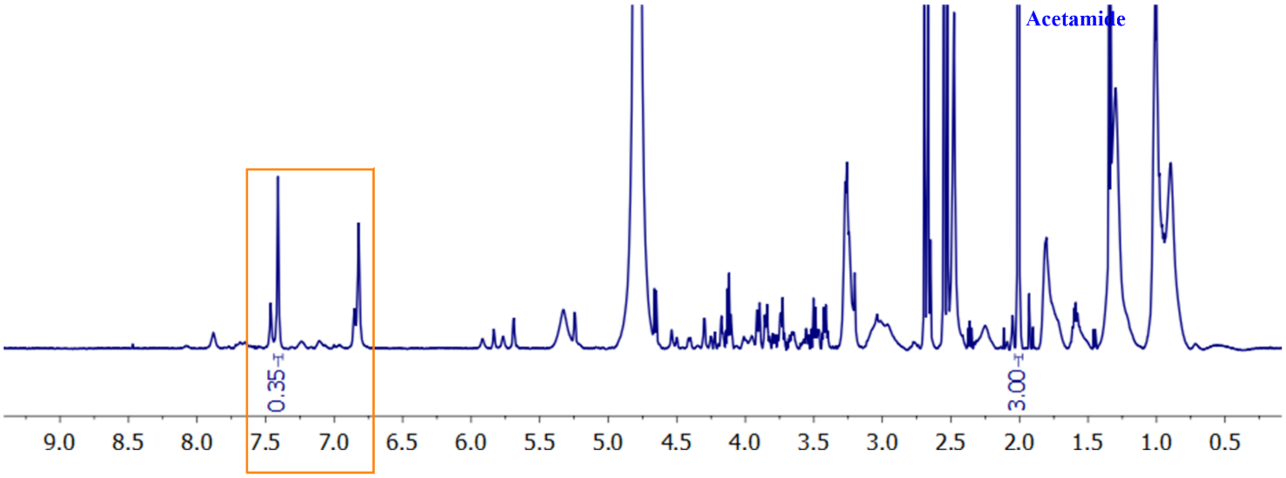

Figure S616. ^{1}H NMR spectrum (600 MHz, D_2O, 298 K) of diazaborine **66** (orange frame), acetamide as internal standard and plasma (Aliquot maintained 72 h in plasma at 37 °C).

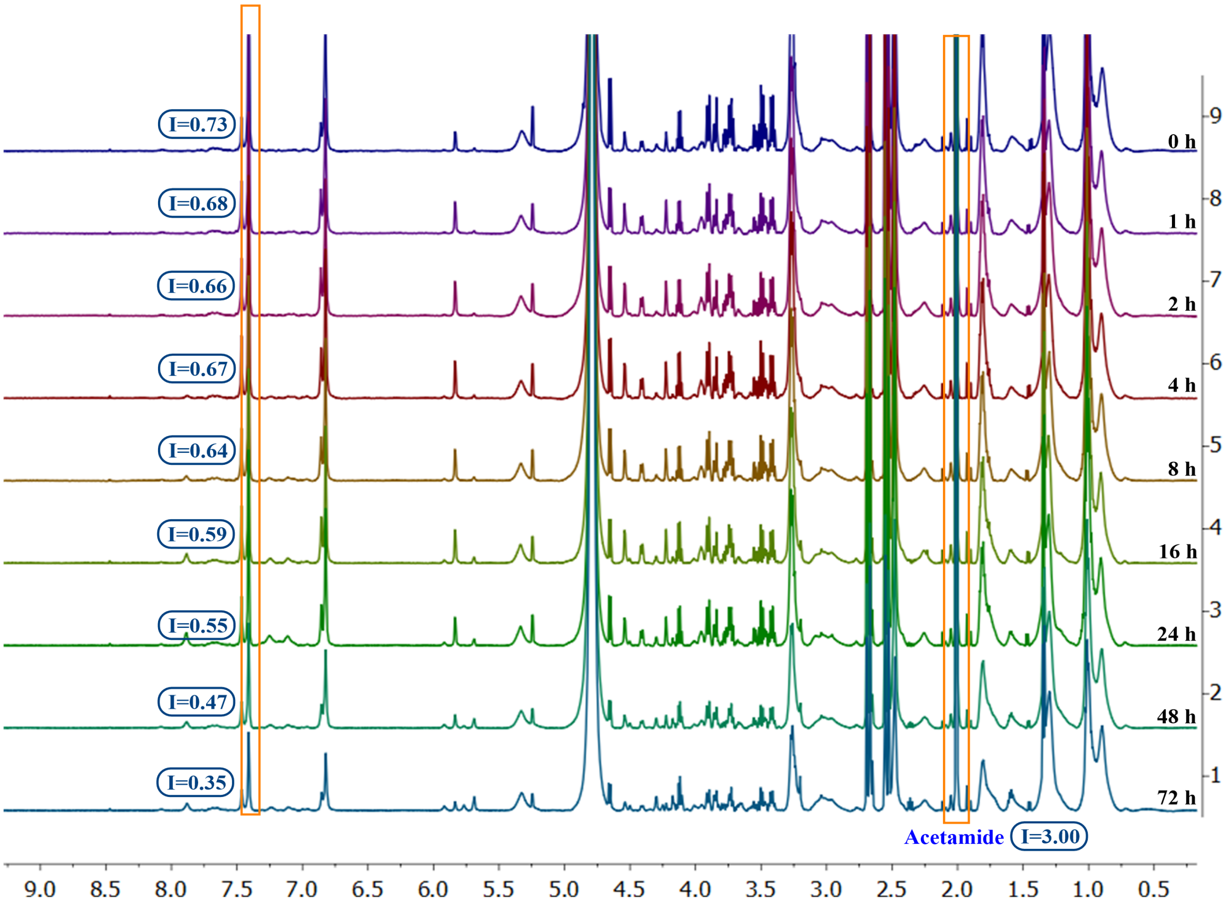

Figure S617. Stacked ^{1}H NMR spectra (600 MHz, D_2O, 298 K) of diazaborine **66** (orange frame), acetamide as internal standard and plasma (Aliquots maintained in plasma at 37 °C taken at different times).

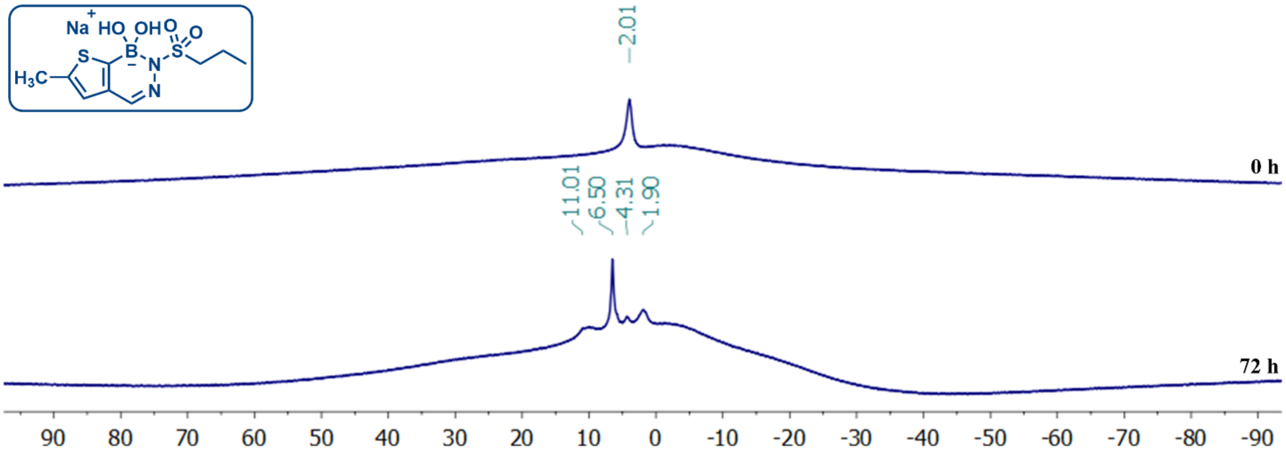

Figure S618. *Up*: Initial ^{11}B NMR spectrum (160 MHz, D_2O, 298 K) of a 1:1 mixture of diazaborine **66** and acetamide as internal standard; *Down*: ^{11}B NMR spectrum (128 MHz, D_2O, 298 K) of diazaborine **66**, acetamide as internal standard and plasma (Aliquot maintained 72 h in plasma at 37 °C).

Validation of the plasma studies on D_2O at 37 °C

To validate that the changes observed in the sodium salts of the diazaborines during their evaluation in plasma at 37 °C could be attributed to their intrinsic stability under those near-physiological conditions, and not to their intrinsic behavior on aqueous media at that temperature, we monitored the time-dependent behavior of those salts in D_2O at 37 °C.

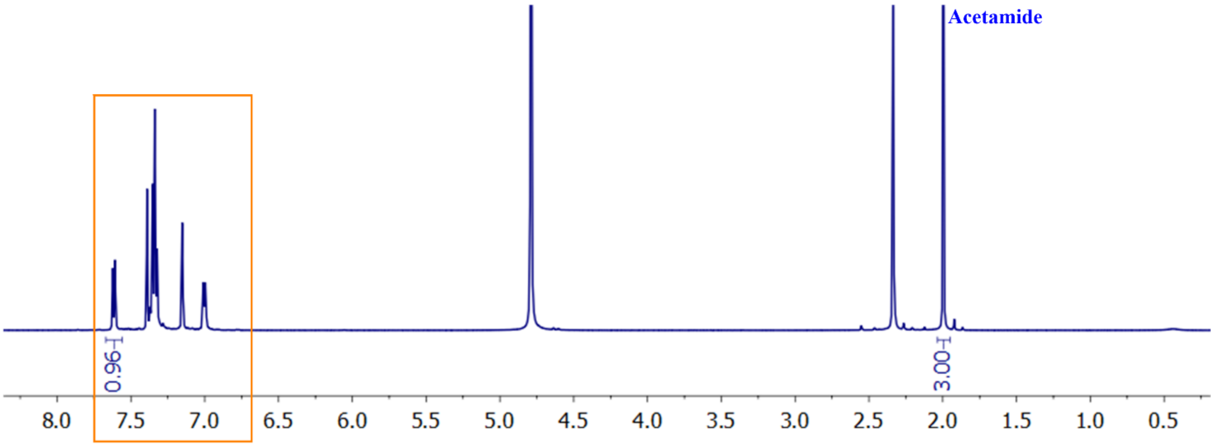

Figure S619. Initial ^{1}H NMR spectrum (500 MHz, D_2O, 298 K) of a 1:1 mixture of diazaborine **62** (orange frame) and acetamide as internal standard (Aliquot maintained 0 h in D_2O at 37 °C).

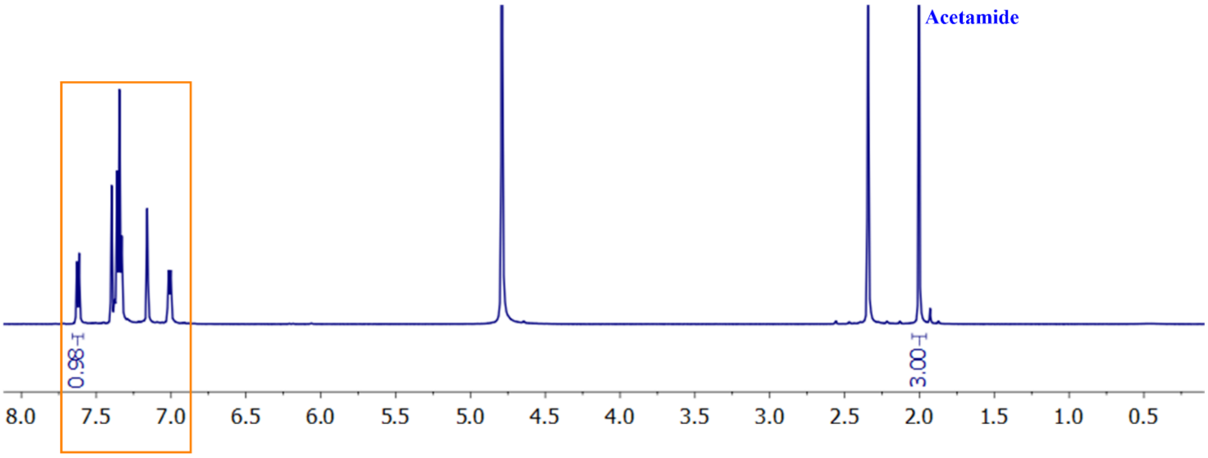

Figure S620. ^{1}H NMR spectrum (500 MHz, D_2O, 298 K) of a 1:1 mixture of diazaborine **62** (orange frame) and acetamide as internal standard (Aliquot maintained 24 h in D_2O at 37 °C).

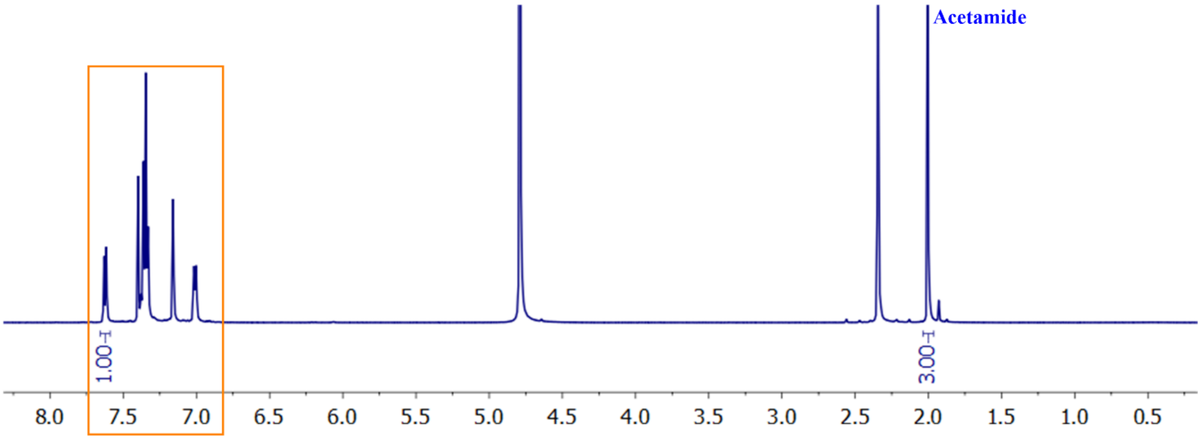

Figure S621. ^{1}H NMR spectrum (500 MHz, D_2O, 298 K) of a 1:1 mixture of diazaborine **62** (orange frame) and acetamide as internal standard (Aliquot maintained 72 h in D_2O at 37 °C).

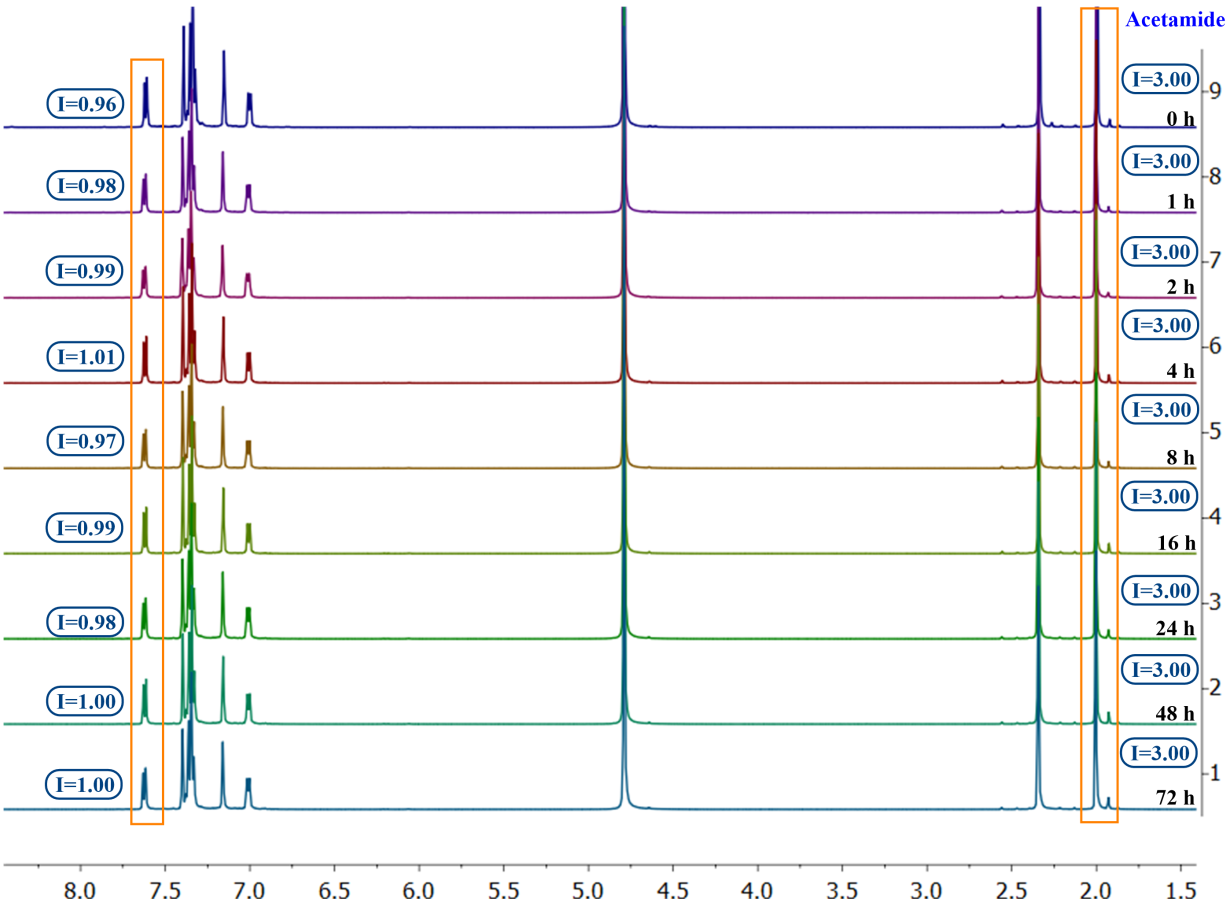

Figure S622. Stacked ^{1}H NMR spectra (500 MHz, D_2O, 298 K) of diazaborine **62** (orange frame) and acetamide as internal standard (Aliquots maintained in D_2O at 37 °C taken at different times).

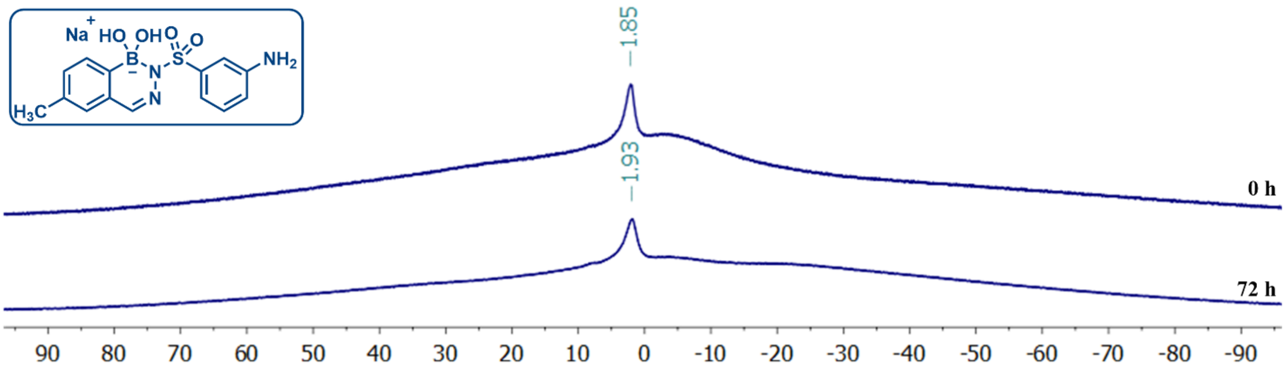

Figure S623. *Up*: Initial ^{11}B NMR spectrum (160 MHz, D_2O, 298 K) of a 1:1 mixture of diazaborine **62** and acetamide as internal standard; *Down*: ^{11}B NMR spectrum (128 MHz, D_2O, 298 K) of diazaborine **62** and acetamide as internal standard (Aliquot maintained 72 h in D_2O at 37 °C).

Diazaborine 64 (Sodium salt of diazaborine 13)

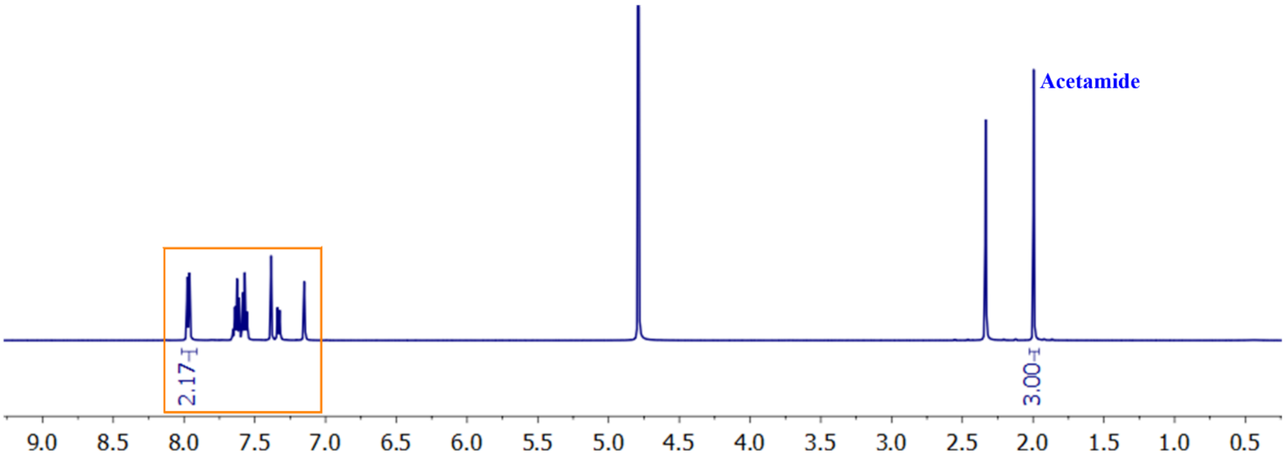

Figure S624. Initial ^{1}H NMR spectrum (500 MHz, D_2O, 298 K) of a 1:1 mixture of diazaborine **64** (orange frame) and acetamide as internal standard (Aliquot maintained 0 h in D_2O at 37 °C).

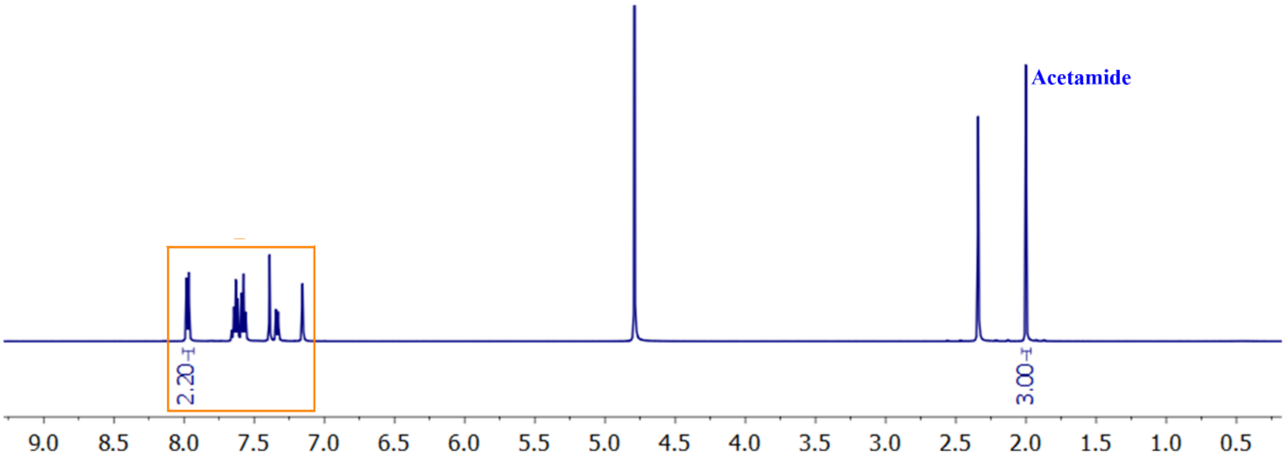

Figure S625. ^{1}H NMR spectrum (500 MHz, D_2O, 298 K) of a 1:1 mixture of diazaborine **64** (orange frame) and acetamide as internal standard (Aliquot maintained 24 h in D_2O at 37 °C).

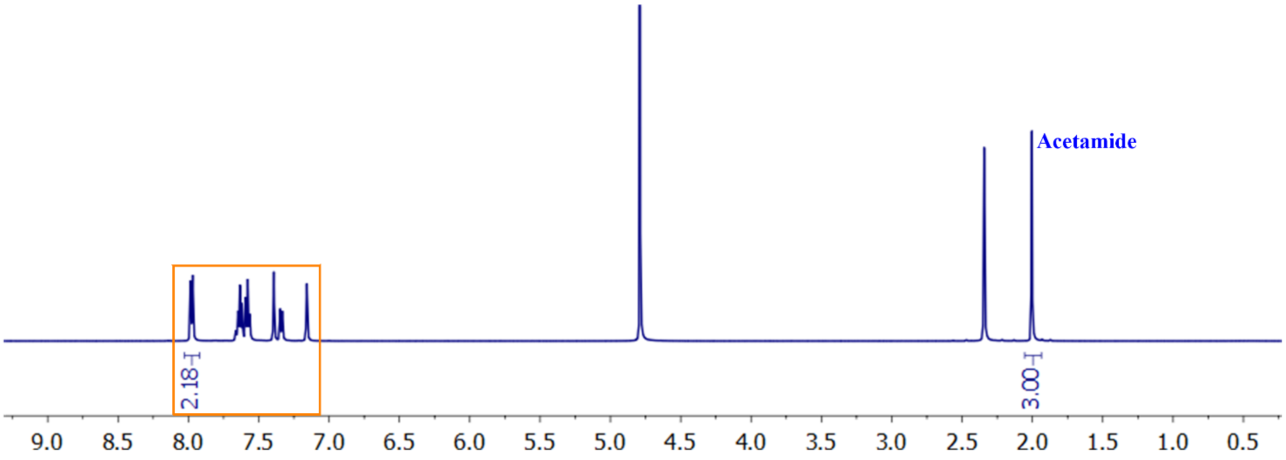

Figure S626. ^{1}H NMR spectrum (500 MHz, D_2O, 298 K) of a 1:1 mixture of diazaborine **64** (orange frame) and acetamide as internal standard (Aliquot maintained 72 h in D_2O at 37 °C).

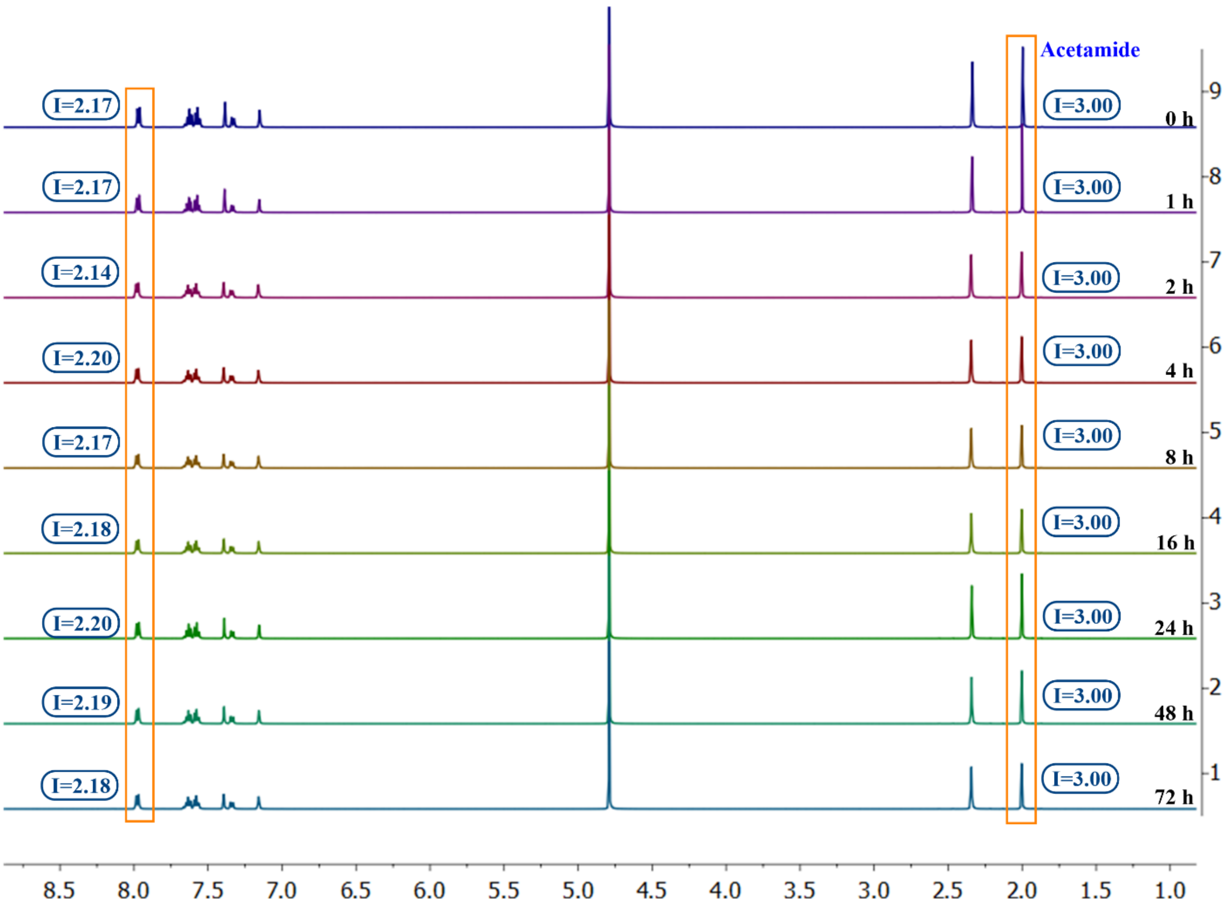

Figure S627. Stacked ^{1}H NMR spectra (500 MHz, D_2O, 298 K) of diazaborine **64** (orange frame) and acetamide as internal standard (Aliquots maintained in D_2O at 37 °C taken at different times).

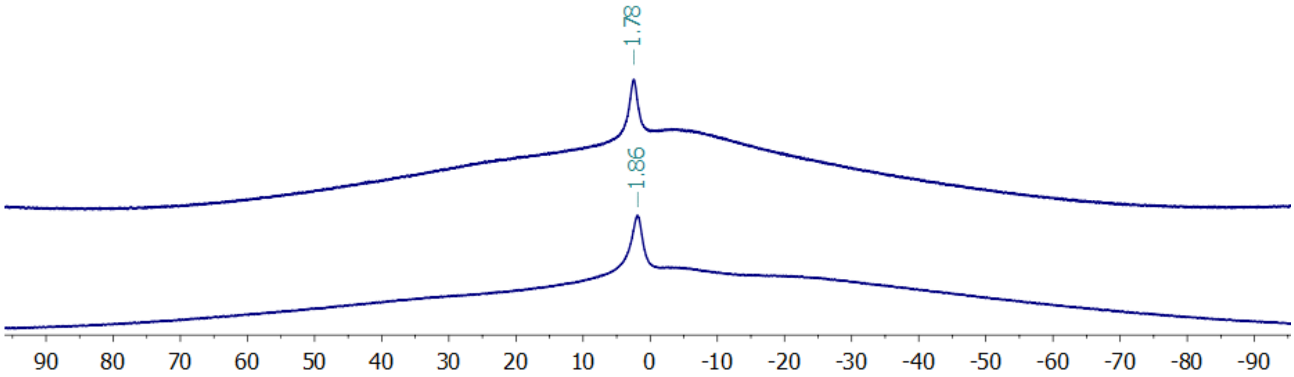

Figure S628. *Up*: Initial ^{11}B NMR spectrum (160 MHz, D_2O, 298 K) of a 1:1 mixture of diazaborine **64** and acetamide as internal standard; *Down*: ^{11}B NMR spectrum (128 MHz, D_2O, 298 K) of diazaborine **64** and acetamide as internal standard (Aliquot maintained 72 h in D_2O at 37 °C).

Diazaborine 65 (Sodium salt of diazaborine 41)

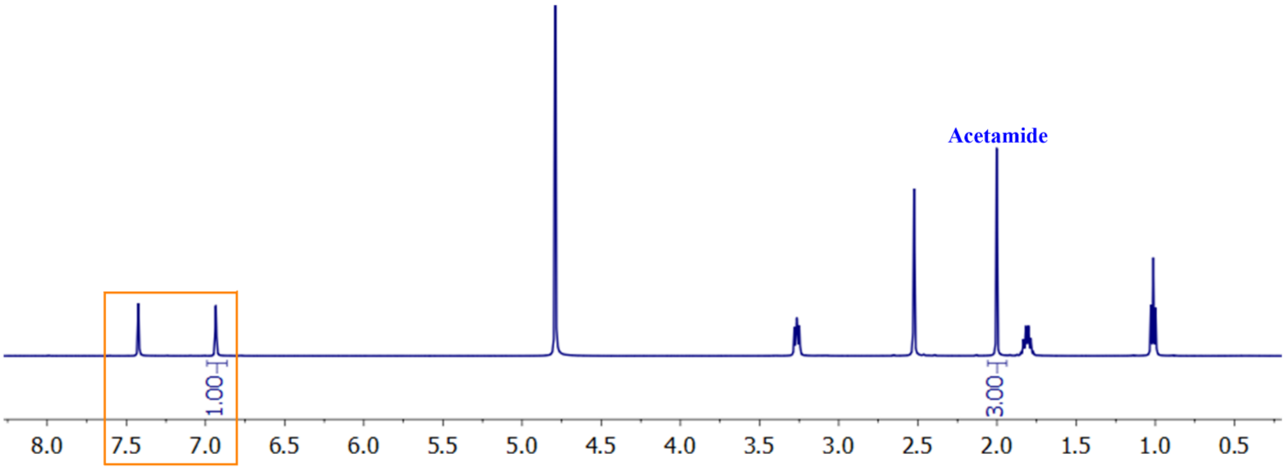

Figure S629. Initial ^{1}H NMR spectrum (500 MHz, D_2O, 298 K) of a 1:1 mixture of diazaborine **65** (orange frame) and acetamide as internal standard (Aliquot maintained 0 h in D_2O at 37 °C).

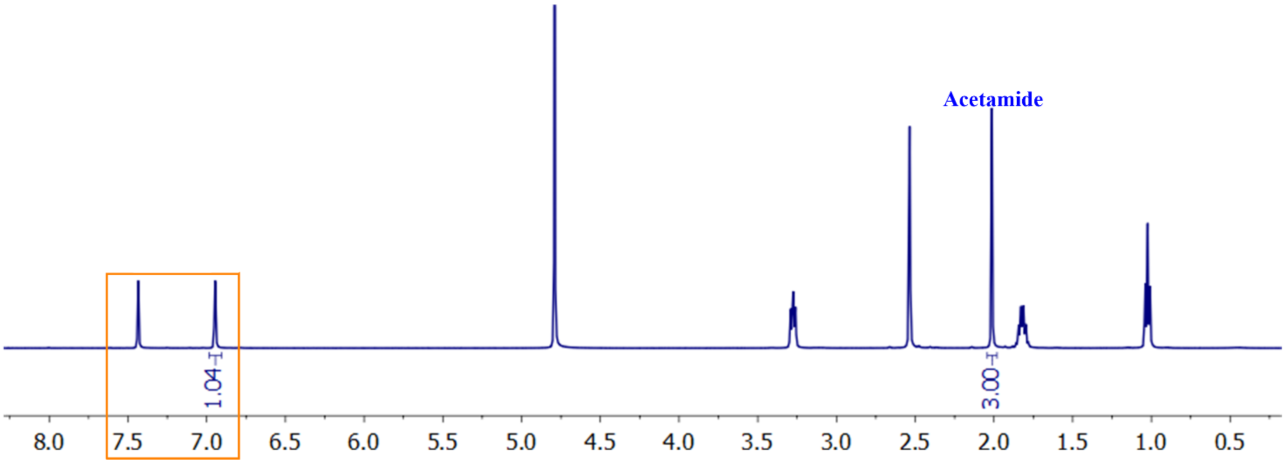

Figure S630. ^{1}H NMR spectrum (500 MHz, D_2O, 298 K) of a 1:1 mixture of diazaborine **65** (orange frame) and acetamide as internal standard (Aliquot maintained 24 h in D_2O at 37 °C).

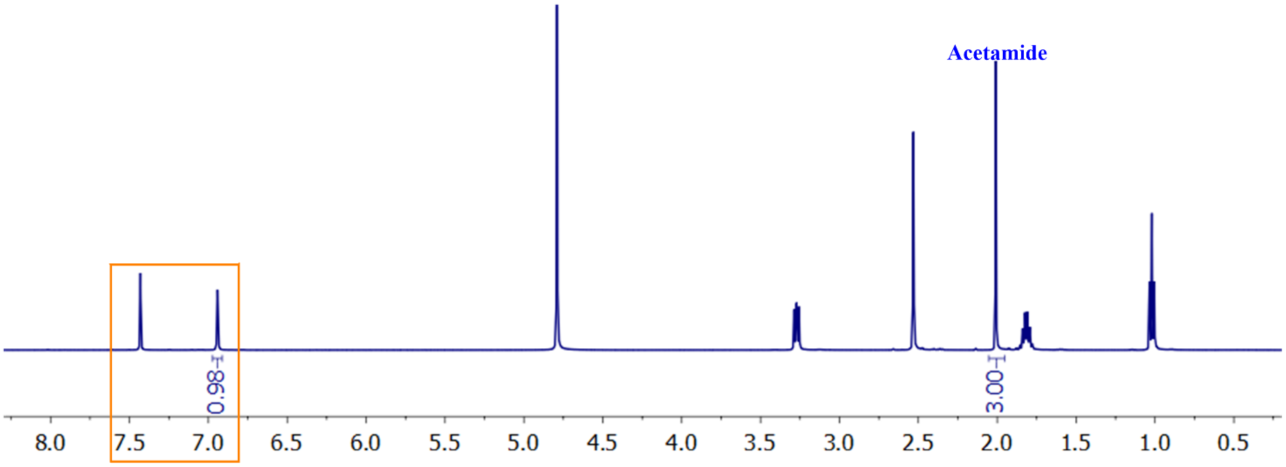

Figure S631. ^{1}H NMR spectrum (500 MHz, D_2O, 298 K) of a 1:1 mixture of diazaborine **65** (orange frame) and acetamide as internal standard (Aliquot maintained 72 h in D_2O at 37 °C).

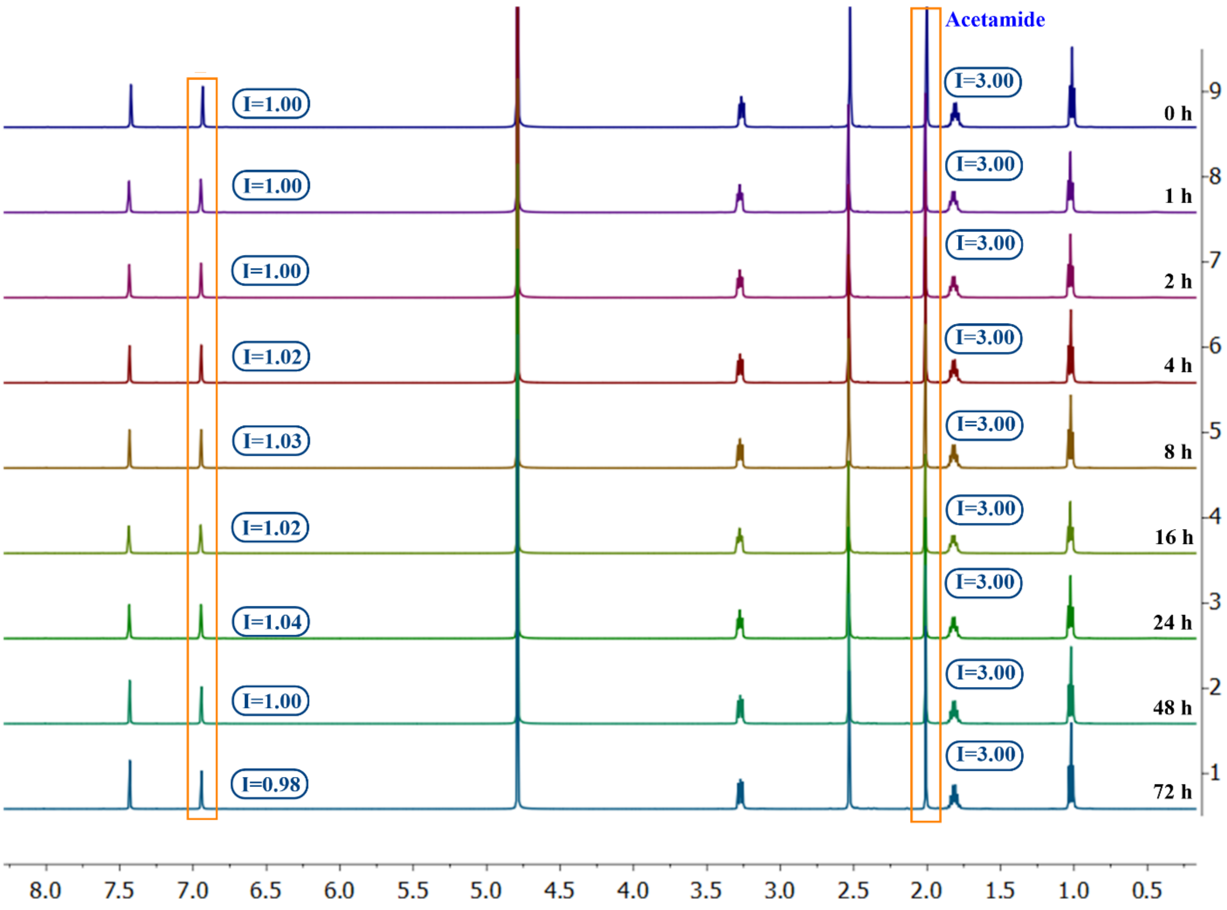

Figure S632. Stacked ^{1}H NMR spectra (500 MHz, D_2O, 298 K) of diazaborine **65** (orange frame) and acetamide as internal standard (Aliquots maintained in D_2O at 37 °C taken at different times).

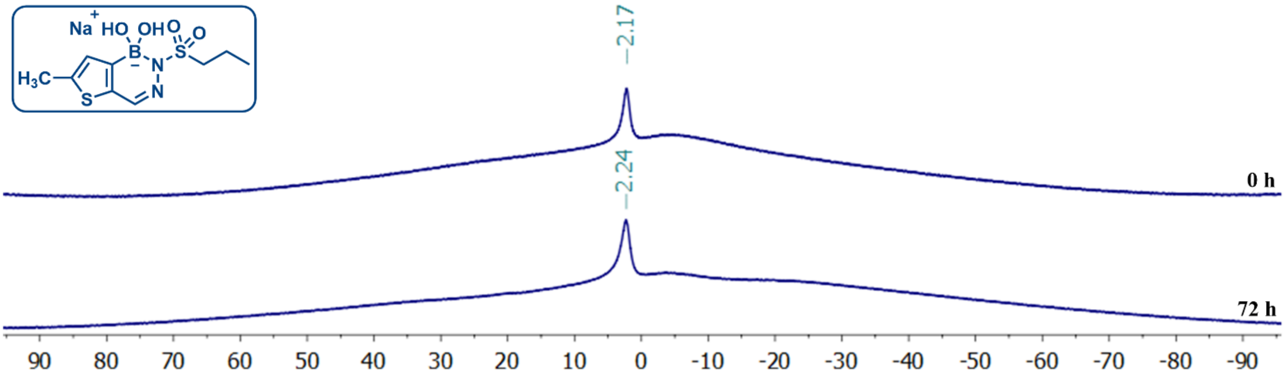

Figure S633. *Up*: Initial ^{11}B NMR spectrum (160 MHz, D_2O, 298 K) of a 1:1 mixture of diazaborine **65** and acetamide as internal standard; *Down*: ^{11}B NMR spectrum (128 MHz, D_2O, 298 K) of diazaborine **65** and acetamide as internal standard (Aliquot maintained 72 h in D_2O at 37 °C).

Diazaborine 66 (Sodium salt of diazaborine 47)

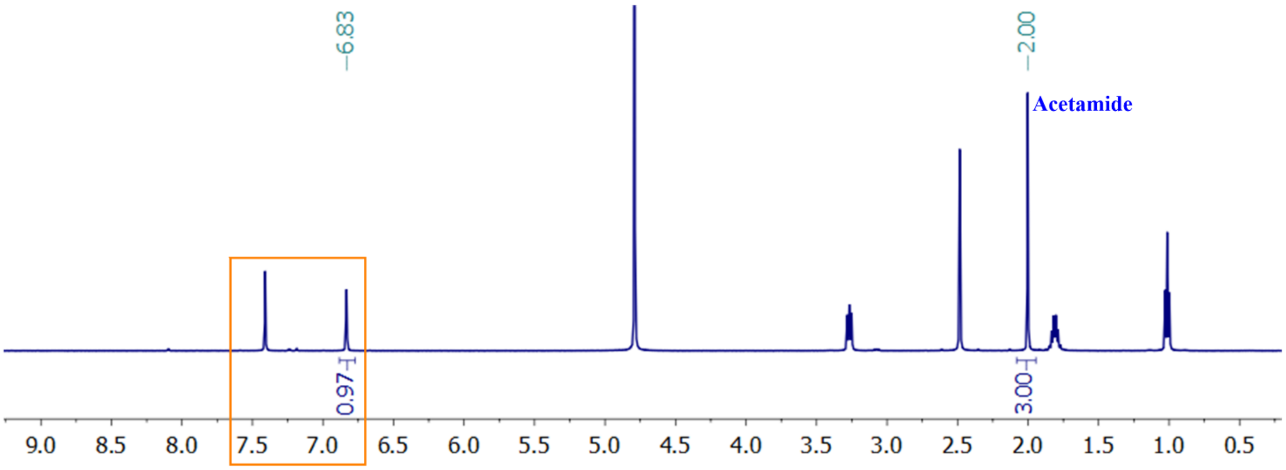

Figure S634. Initial ^{1}H NMR spectrum (500 MHz, D_2O, 298 K) of a 1:1 mixture of diazaborine **66** (orange frame) and acetamide as internal standard (Aliquot maintained 0 h in D_2O at 37 °C).

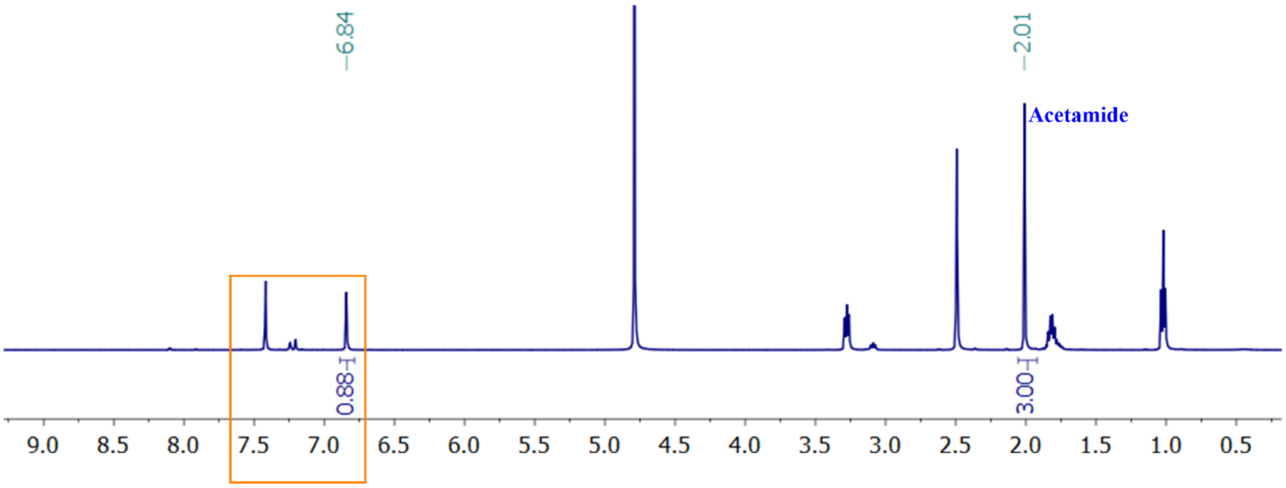

Figure S635. ^{1}H NMR spectrum (500 MHz, D_2O, 298 K) of a 1:1 mixture of diazaborine **66** (orange frame) and acetamide as internal standard (Aliquot maintained 24 h in D_2O at 37 °C).

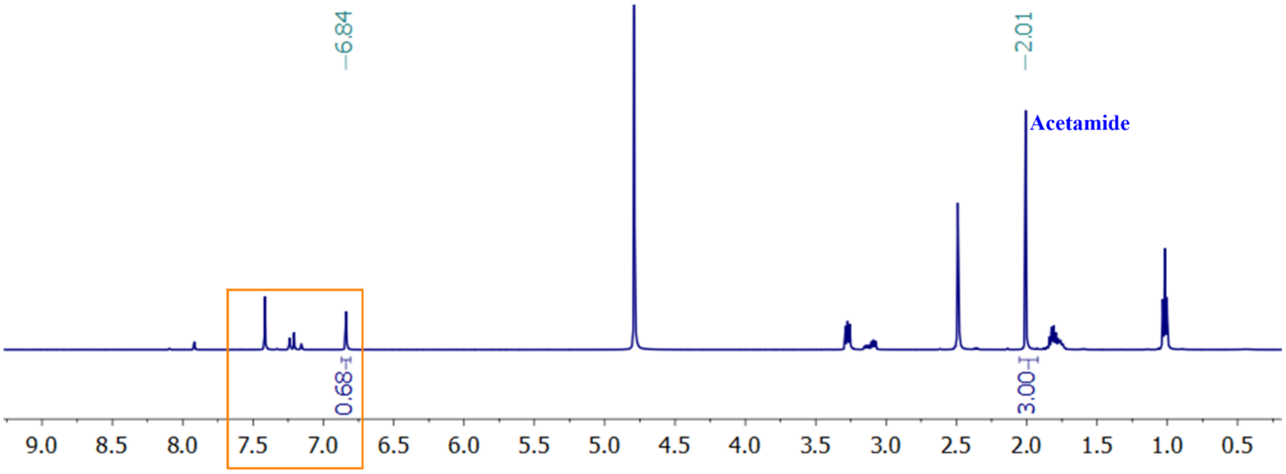

Figure S636. ^{1}H NMR spectrum (500 MHz, D_2O, 298 K) of a 1:1 mixture of diazaborine **66** (orange frame) and acetamide as internal standard (Aliquot maintained 72 h in D_2O at 37 °C).

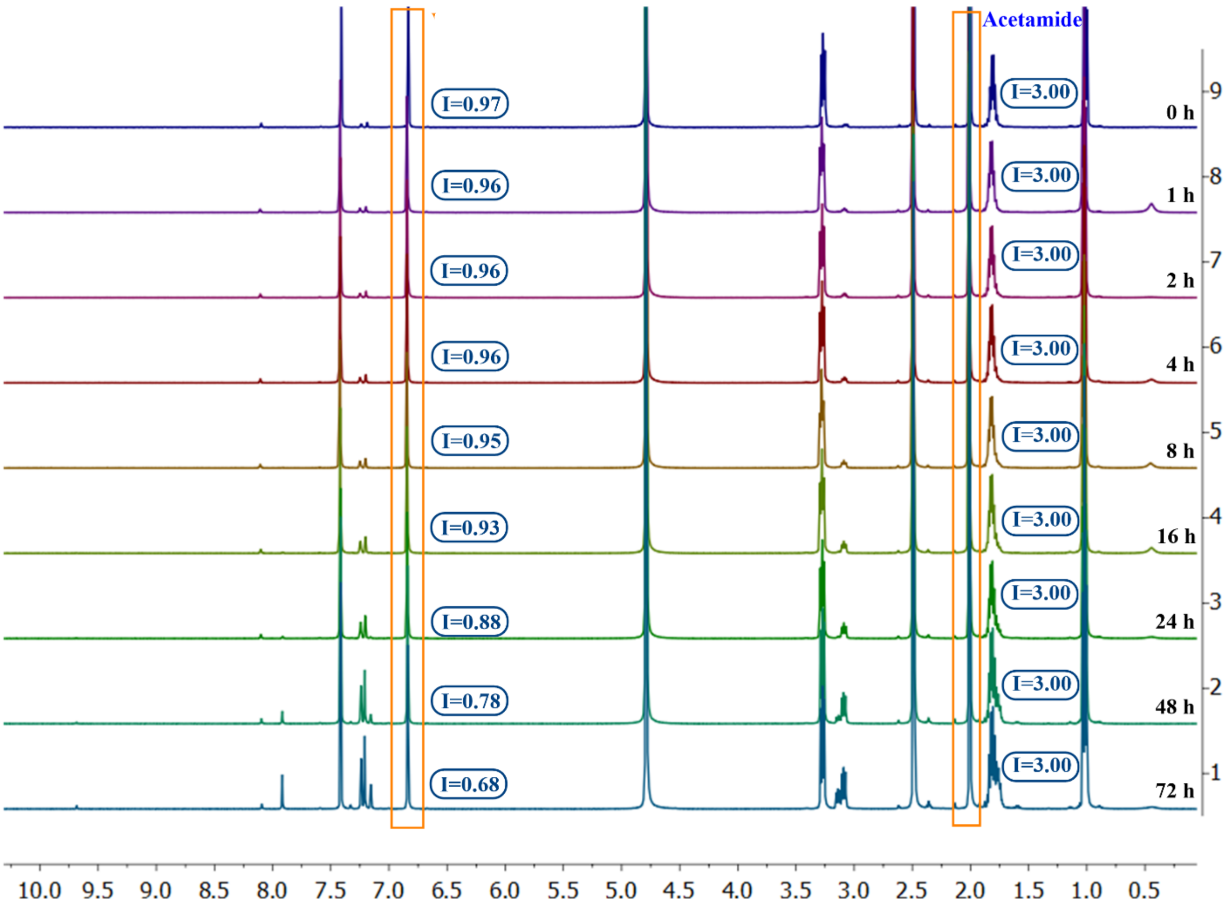

Figure S637. Stacked ^{1}H NMR spectra (500 MHz, D_2O, 298 K) of diazaborine **66** (orange frame) and acetamide as internal standard (Aliquots maintained in D_2O at 37 °C taken at different times).

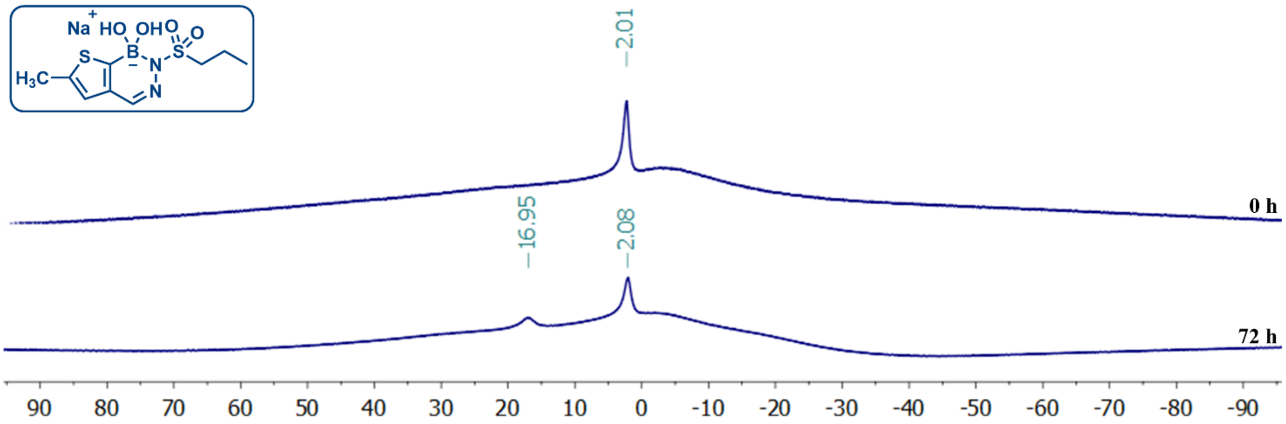

Figure S638. *Up*: Initial ^{11}B NMR spectrum (160 MHz, D_2O, 298 K) of a 1:1 mixture of diazaborine **66** and acetamide as internal standard; *Down*: ^{11}B NMR spectrum (128 MHz, D_2O, 298 K) of diazaborine **66** and acetamide as internal standard (Aliquot maintained 72 h in D_2O at 37 °C).

Validation of the plasma studies on DMSO-d_6 at 37 °C

Diazaborine 62 (Sodium salt of diazaborine 11)

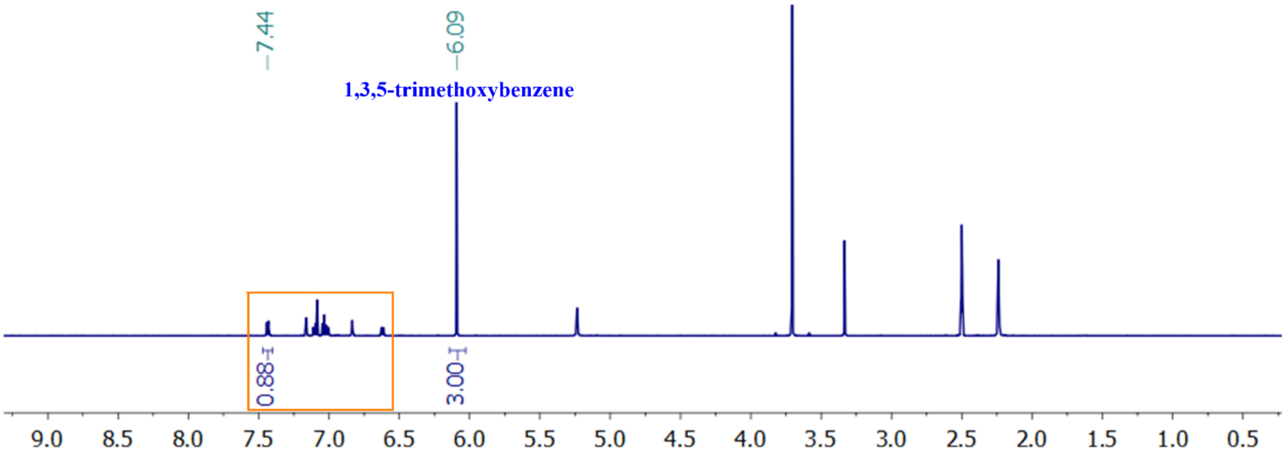

Figure S639. Initial ^{1}H NMR spectrum (600 MHz, DMSO-d_6, 298 K) of a 1:1 mixture of diazaborine **62** (orange frame) and 1,3,5-trimethoxybenzene as internal standard (Aliquot maintained 0 h in DMSO-d_6 at 37 °C).

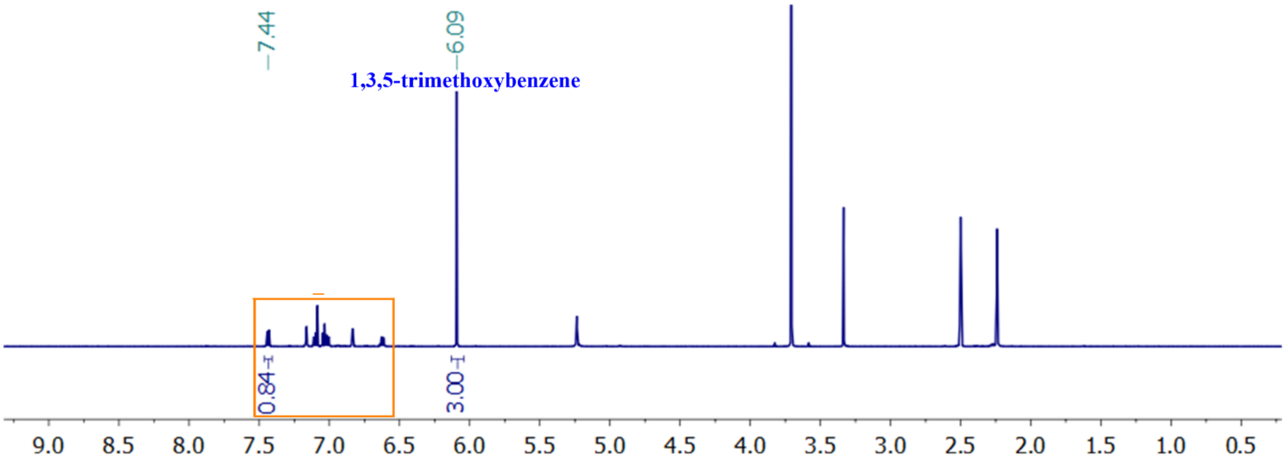

Figure S640. ^{1}H NMR spectrum (600 MHz, DMSO-d_6, 298 K) of a 1:1 mixture of diazaborine **62** (orange frame) and 1,3,5-trimethoxybenzene as internal standard (Aliquot maintained 24 h in DMSO-d_6 at 37 °C).

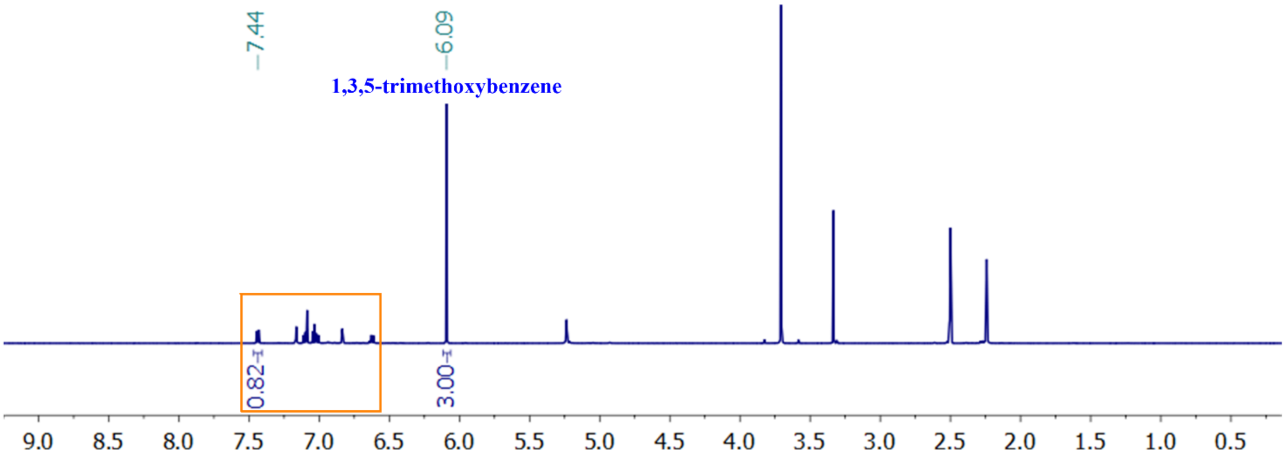

Figure S641. ^{1}H NMR spectrum (600 MHz, DMSO-d_6, 298 K) of a 1:1 mixture of diazaborine **62** (orange frame) and 1,3,5-trimethoxybenzene as internal standard (Aliquot maintained 72 h in DMSO-d_6 at 37 °C).

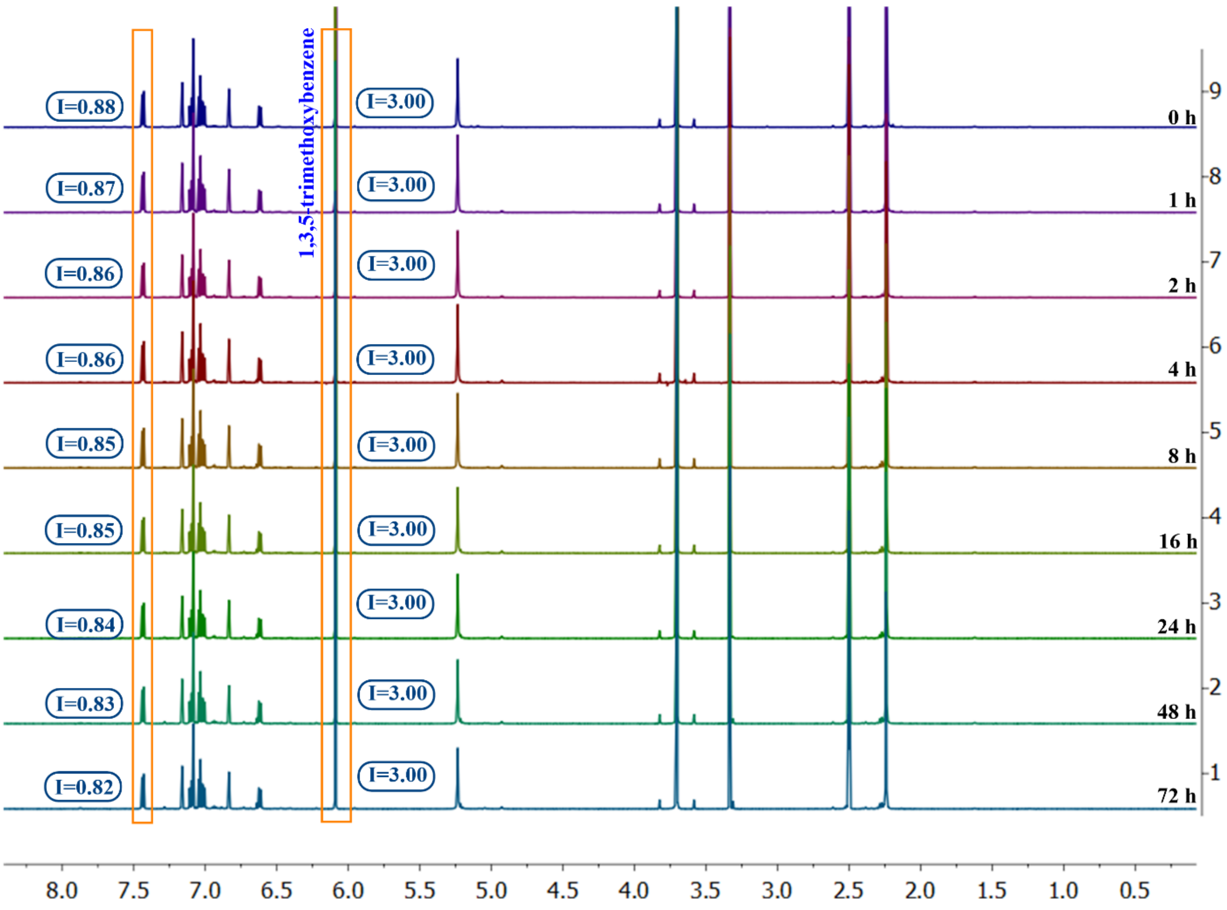

Figure S642. Stacked ^{1}H NMR spectra (600 MHz, DMSO-d_6, 298 K) of diazaborine **62** (orange frame)and 1,3,5-trimethoxybenzene as internal standard (Aliquots maintained in DMSO-d_6 at 37 °C taken at different times).

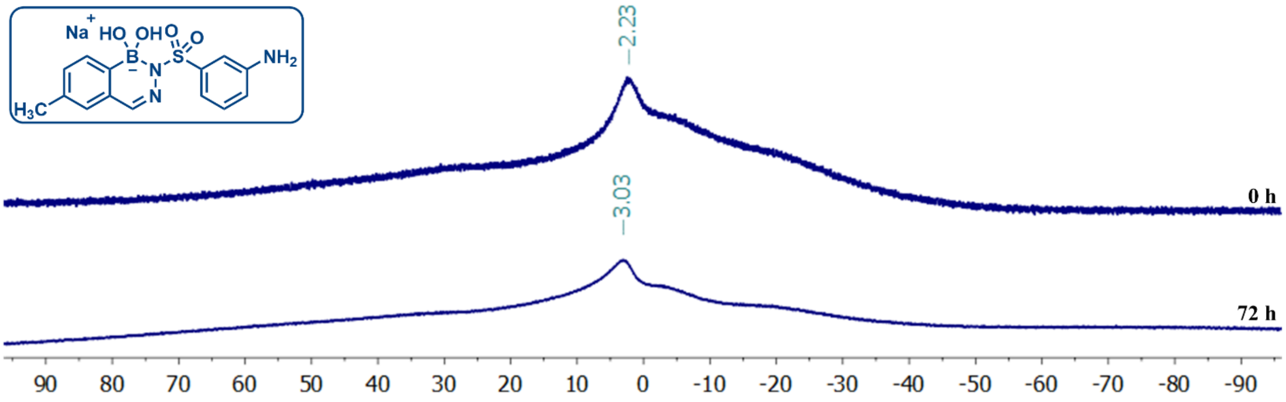

Figure S643. *Up*: Initial ^{11}B NMR spectrum (128 MHz, DMSO-d_6, 298 K) of a 1:1 mixture of diazaborine **62** and 1,3,5-trimethoxybenzene as internal standard; *Down*: ^{11}B NMR spectrum (128 MHz, DMSO-d_6, 298 K) of diazaborine **62** and 1,3,5-trimethoxybenzene as internal standard (Aliquot maintained 72 h in DMSO-d_6 at 37 °C).

Diazaborine 64 (Sodium salt of diazaborine 13)

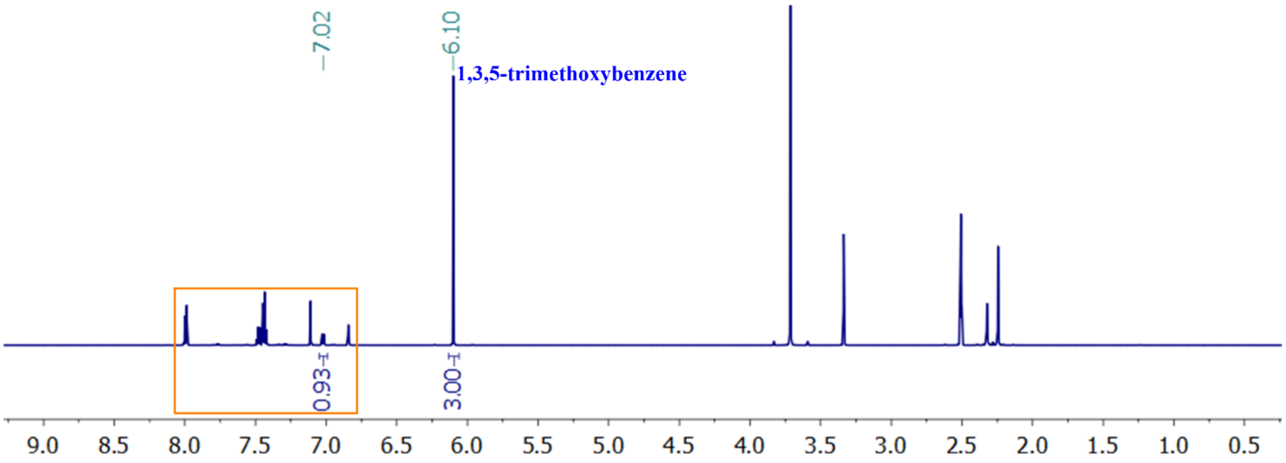

Figure S644. Initial ^{1}H NMR spectrum (600 MHz, DMSO-d_6, 298 K) of a 1:1 mixture of diazaborine **64** (orange frame) and 1,3,5-trimethoxybenzene as internal standard (Aliquot maintained 0 h in DMSO-d_6 at 37 °C).

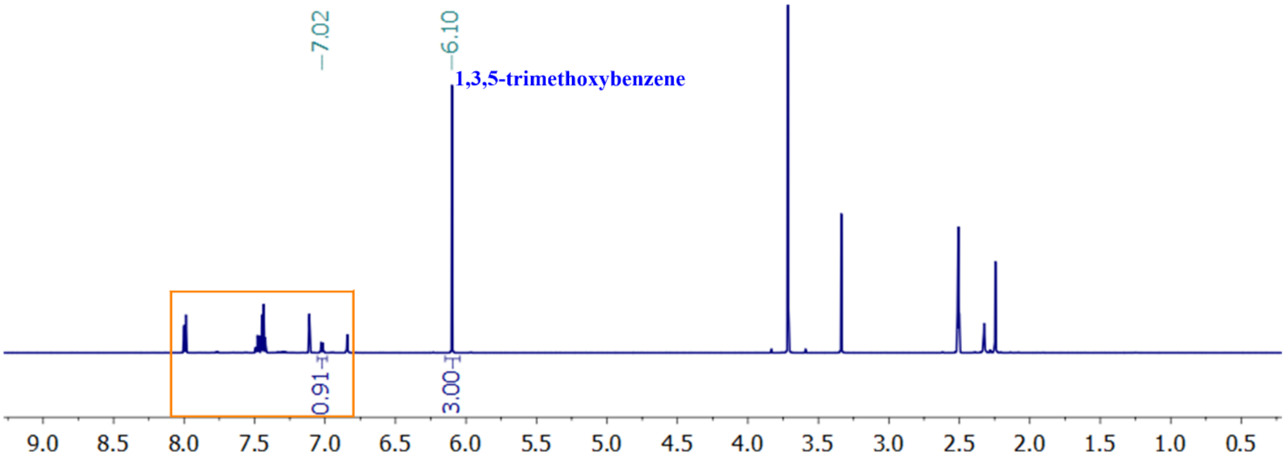

Figure S645. ^{1}H NMR spectrum (600 MHz, DMSO-d_6, 298 K) of a 1:1 mixture of diazaborine **64** (orange frame) and 1,3,5-trimethoxybenzene as internal standard (Aliquot maintained 24 h in DMSO-d_6 at 37 °C).

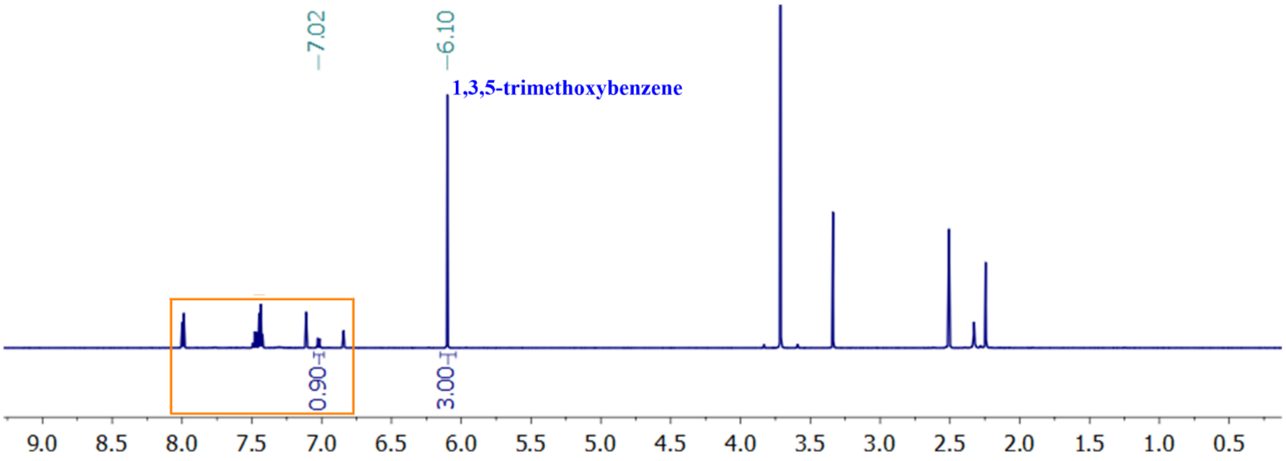

Figure S646. ^{1}H NMR spectrum (600 MHz, DMSO-d_6, 298 K) of a 1:1 mixture of diazaborine **64** (orange frame) and 1,3,5-trimethoxybenzene as internal standard (Aliquot maintained 72 h in DMSO-d_6 at 37 °C).

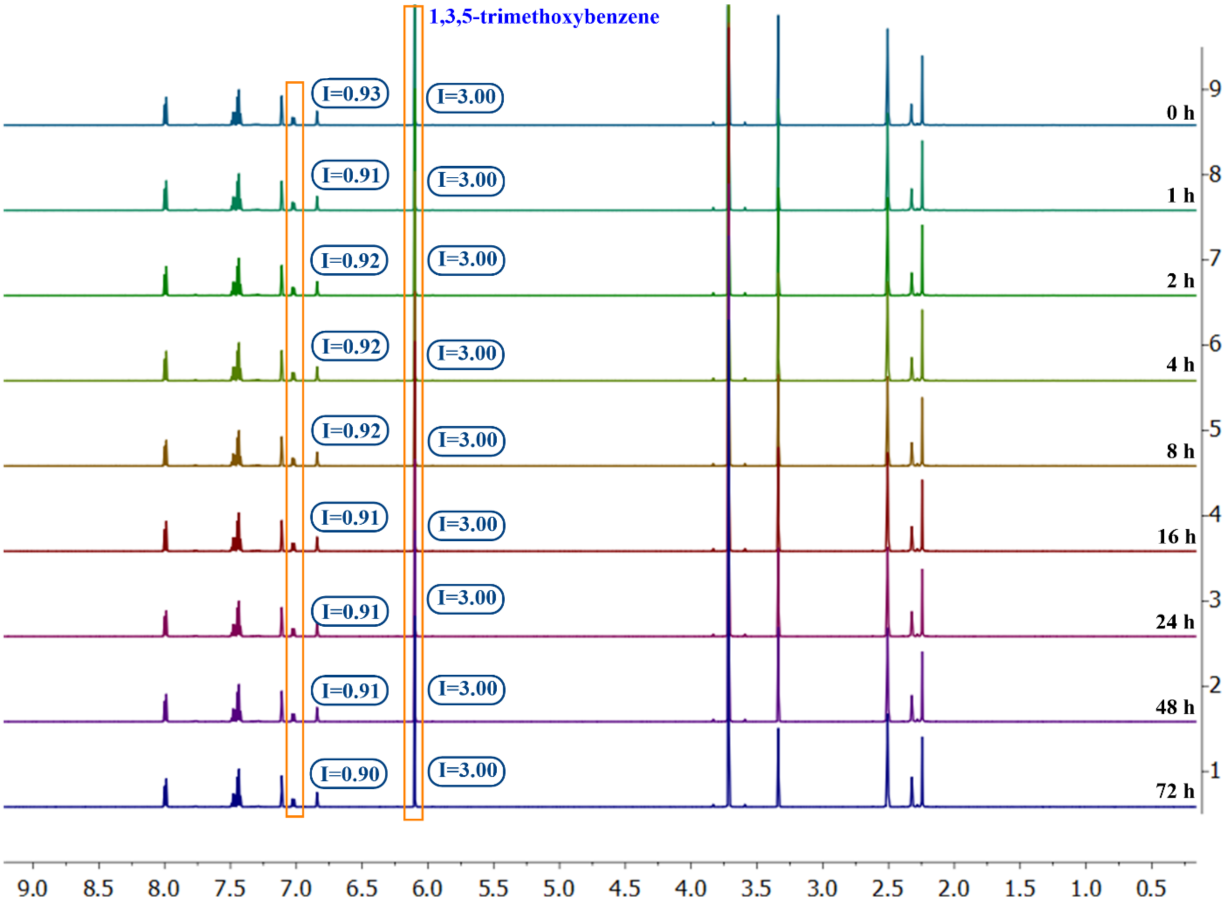

Figure S647. Stacked ^{1}H NMR spectra (600 MHz, DMSO-d_6, 298 K) of diazaborine **64** (orange frame) and 1,3,5-trimethoxybenzene as internal standard (Aliquots maintained in DMSO-d_6 at 37 °C taken at different times).

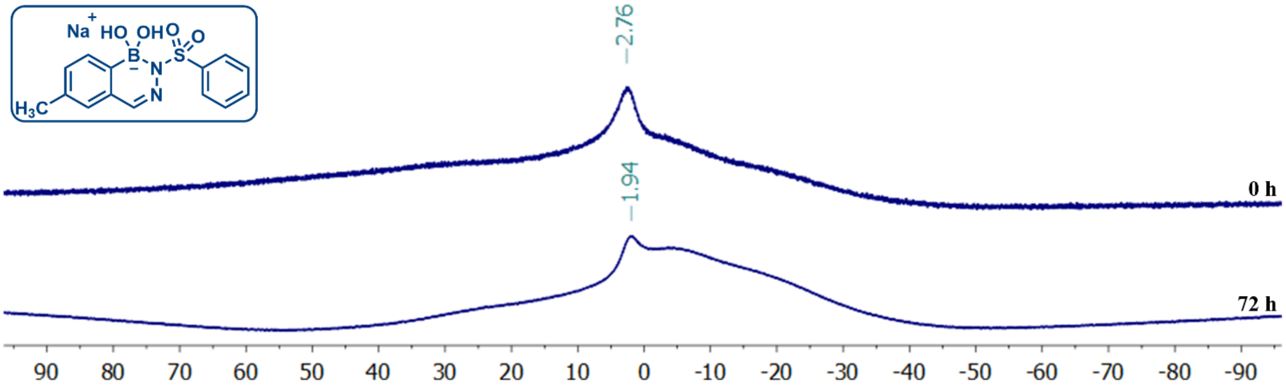

Figure S648. *Up*: Initial ^{11}B NMR spectrum (128 MHz, DMSO-d_6, 298 K) of a 1:1 mixture of diazaborine **64** and 1,3,5-trimethoxybenzene as internal standard; *Down*: ^{11}B NMR spectrum (128 MHz, DMSO-d_6, 298 K) of diazaborine **64** and 1,3,5-trimethoxybenzene as internal standard (Aliquot maintained 72 h in DMSO-d_6 at 37 °C).

HPLC analysis of lead compounds (Figures S649-S651)

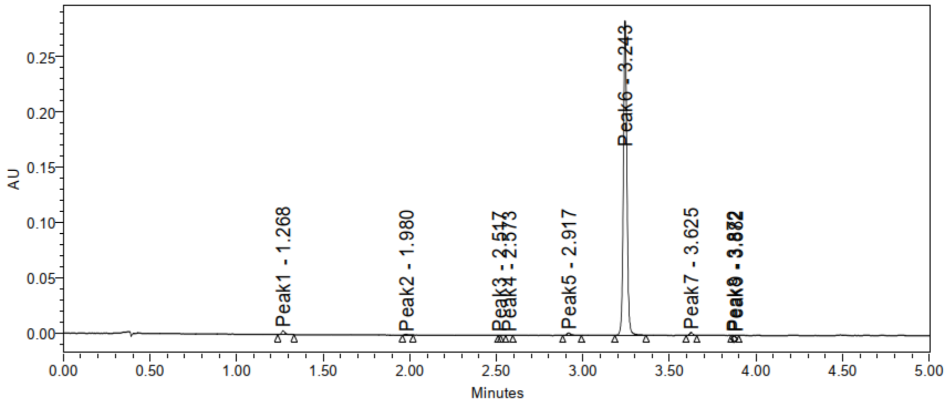

	Peak Name	RT	Area	% Area	Height
1	Peak1	1.268	4676	1.14	3378
2	Peak2	1.980	996	0.24	657
3	Peak3	2.517	34	0.01	69
4	Peak4	2.573	276	0.07	214
5	Peak5	2.917	3594	0.88	2056
6	Peak6	3.243	395187	96.75	284343
7	Peak7	3.625	3314	0.81	2607
8	Peak8	3.872	104	0.03	196
9	Peak9	3.882	282	0.07	284

Figure S649. UHPLC chromatogram for diazaborine **11**. Experimental details described in Materials and Methods.

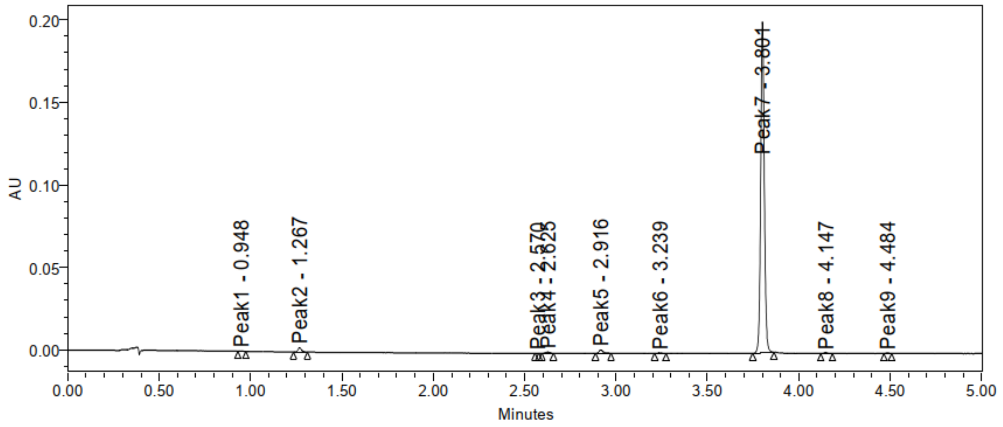

	Peak Name	RT	Area	% Area	Height
1	Peak1	0.948	294	0.11	264
2	Peak2	1.267	3483	1.25	2648
3	Peak3	2.570	41	0.01	68
4	Peak4	2.625	1182	0.42	737
5	Peak5	2.916	3239	1.16	1976
6	Peak6	3.239	533	0.19	357
7	Peak7	3.801	268582	96.40	200615
8	Peak8	4.147	803	0.29	588
9	Peak9	4.484	456	0.16	380

Figure S650. UHPLC chromatogram for diazaborine **13.** Experimental details described in Materials and Methods.

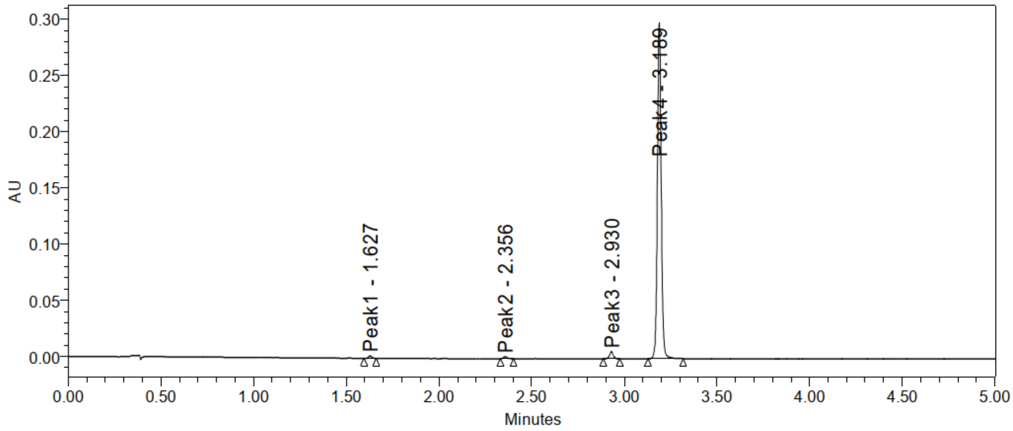

	Peak Name	RT	Area	% Area	Height
1	Peak1	1.627	2510	0.57	2156
2	Peak2	2.356	2538	0.58	1884
3	Peak3	2.930	9044	2.05	6416
4	Peak4	3.189	426929	96.80	299286

Figure S651. UHPLC chromatogram for diazaborine **41.** Experimental details described in Materials and Methods.
